# C–H magnesiation of unactivated arenes by a bulky β-diketiminato magnesium *n*-butyl complex

**DOI:** 10.1039/d6sc04717c

**Published:** 2026-09-24

**Authors:** Louis J. Morris, Agustín Morales, Andrew S. S. Wilson, Michael S. Hill, Claire L. McMullin

**Affiliations:** a Department of Chemistry, University of Bath Claverton Down Bath BA2 7AY UK msh27@bath.ac.uk cm2025@bath.ac.uk

## Abstract

The bulky monomeric *n*-butyl magnesium complex [(^Ad^BDI)Mg^*n*^Bu] (1^Ad^; ^Ad^BDI = HC(C(Ad)Ndipp)_2_, dipp = 2,6-diisopropylphenyl; Ad = 1-adamantyl) deprotonates benzene to provide direct access to the corresponding phenylmagnesium derivative [(^Ad^BDI)MgPh] (3^Ad^) which, in turn, can be utilised in palladium-catalysed Kumada biaryl cross-coupling. By contrast, when the less sterically demanding methyl-substituted ligand, ^Me^BDI (HC(C(Me)Ndipp)_2_) is used, the deprotonation reaction is slower and competitive with β-hydride elimination of 1-butene. The enhanced steric demand of 1^Ad^ results in the deprotonation of benzene, even in the presence of more acidic 1,2,3,4-tetramethylcyclopentadiene (Cp^4M^H). In contrast, Cp^4M^H is deprotonated by [(^Me^BDI)Mg(µ-^*n*^Bu)]_2_ ([1^Me^]_2_) in benzene to yield [(^Me^BDI)Mg(η^5^-Cp^4M^)] (4). Compound 1^Ad^ also mediates the kinetically undiscriminating magnesiation of the C(sp^3^)–H and *meta*- and *para*-C(sp^2^)–H bonds of toluene, *ortho*-xylene, and *meta*-xylene at 120 °C, albeit *para*-xylene is deprotonated exclusively in the C(sp^3^)–H position while 1,3-di-*tert*butylbenzene is selectively deprotonated at the 5-position of the aromatic ring. Both 1^Ad^ and [1^Me^]_2_ mediate the directed *ortho*-magnesiation of anisole. The mechanism of benzene- and toluene deprotonation and a possible origin for the contrasting reactivity of 1^Ad^ and [1^Me^]_2_ has been investigated computationally.

## Abstract 2

A bulky β-diketiminate ligand enables the synthesis of aryl magnesium complexes *via* direct C–H deprotonation of arenes by the corresponding *n*-butyl magnesium reagent.

## Graphical abstract

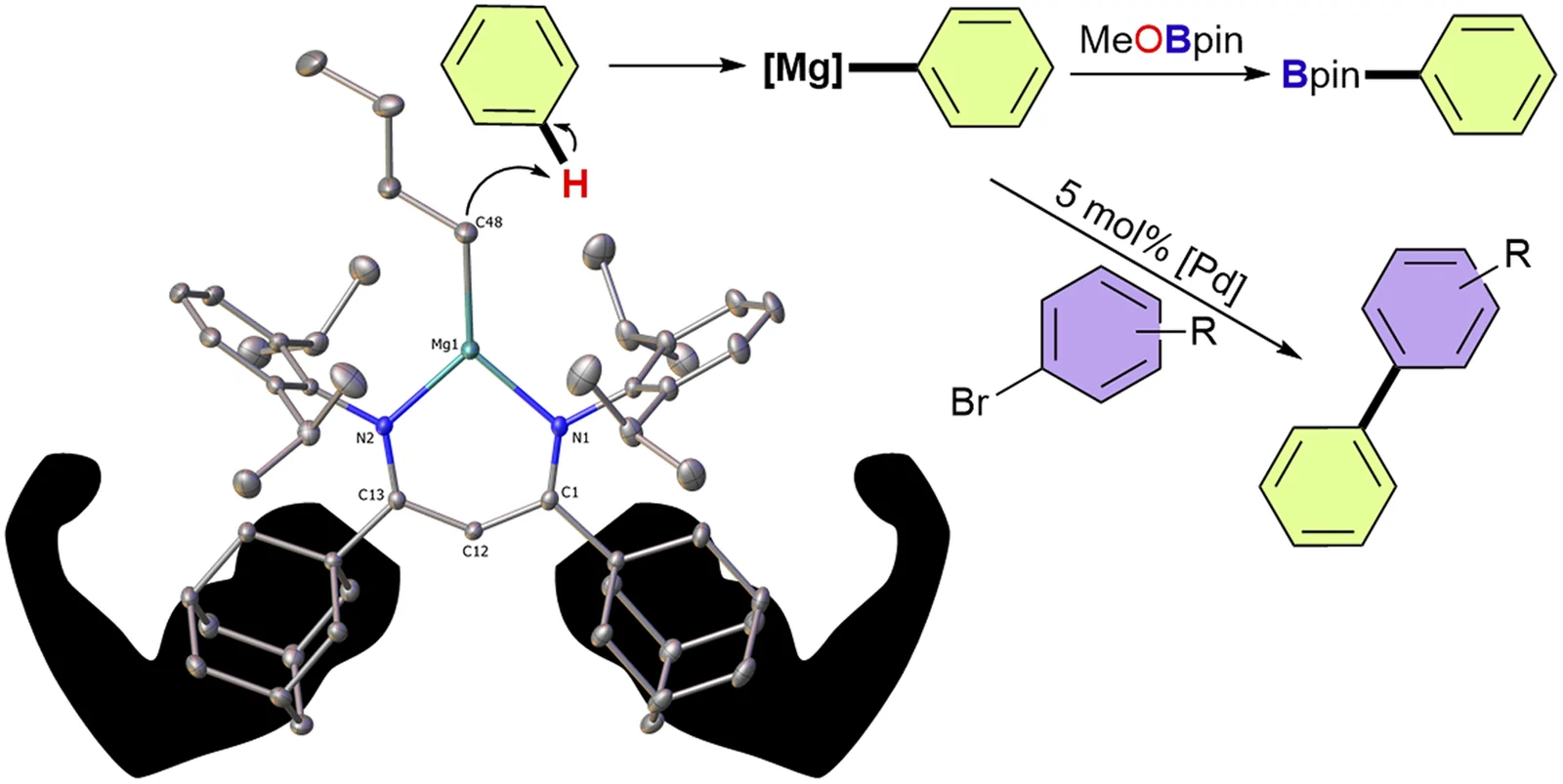

## Introduction

Grignard's ubiquitous aryl magnesium derivatives play a leading role as nucleophilic reagents in synthetic chemistry. In addition to the direct delivery of an aryl nucleophile to electrophilic substrates, the transition metal-catalysed Kumada cross-coupling of arylmagnesium and organohalide provides an especially powerful protocol for the formation of C–C bonds. Almost invariably, however, the organomagnesium reagents themselves must be prepared through the heterogeneous Grignard reaction of metallic magnesium with an aryl halide. Magnesiation of C–H bonds provides an alternative synthetic approach to accessing Mg–Ar reagents. Following early examples of the *ortho*-magnesiation of activated arenes by the Hauser base, (TMP)MgBr, and diamide Mg(TMP)_2_ (TMP = 2,2,6,6-tetramethylpiperidyl),^1^ the related heteroleptic β-diketiminato amide-, *n*-butyl-, and hydride-derivatives [(^Me^BDI)Mg(TMP)] (^Me^BDI = HC(C(Me)Ndipp)_2_, dipp = 2,6-diisopropylphenyl), [(^Me^BDI)Mg^*n*^Bu·thf] (1^Me^·thf), [(^Me^BDI)Mg(µ-^*n*^Bu)]_2_ ([1^Me^]_2_), and [(^Me^BDI)Mg(µ-H)]_2_ ([2^Mg,Me^]_2_) have been more recently utilised to magnesiate comparatively acidic heterocycles such as benzoxazole (Scheme 1a), benzothiozole, *N*-methyl benzimidazole, *N*-methylimidazole, pyrazine, triazoles and substituted pyridines.^2–4^ Notably, however, magnesiation of the less acidified C–H bonds (*i.e.* p*K*_a_ > 40) of hydrocarbon arenes has only been accomplished in heterometallic systems, where the combination of magnesium with a group 1 metal alkyl, amide, or halide generates alkali metal magnesiate species with substantially increased basicity compared to the monometallic magnesium precursor.^5,6^ For example, ‘Turbo’ Hauser bases (TMP)_2_Mg·2LiCl^7,8^ and (TMP)MgCl·LiCl^9,10^ have been utilised in C–H metalation–functionalisation protocols (Scheme 1b), whilst advances in the chemistry of amido/alkyl alkali metal magnesiates derived from Na/K–Mg ^*n*^Bu/TMP have led to the synthesis and isolation of discrete molecular heterometallic aryl complexes *via* a variety of selective C(sp^2^)–H metalation reactions with benzene, toluene and naphthalene, as well as various heteroatom-substituted benzene derivatives (Scheme 1c).^5,11–17^

**Scheme 1:**
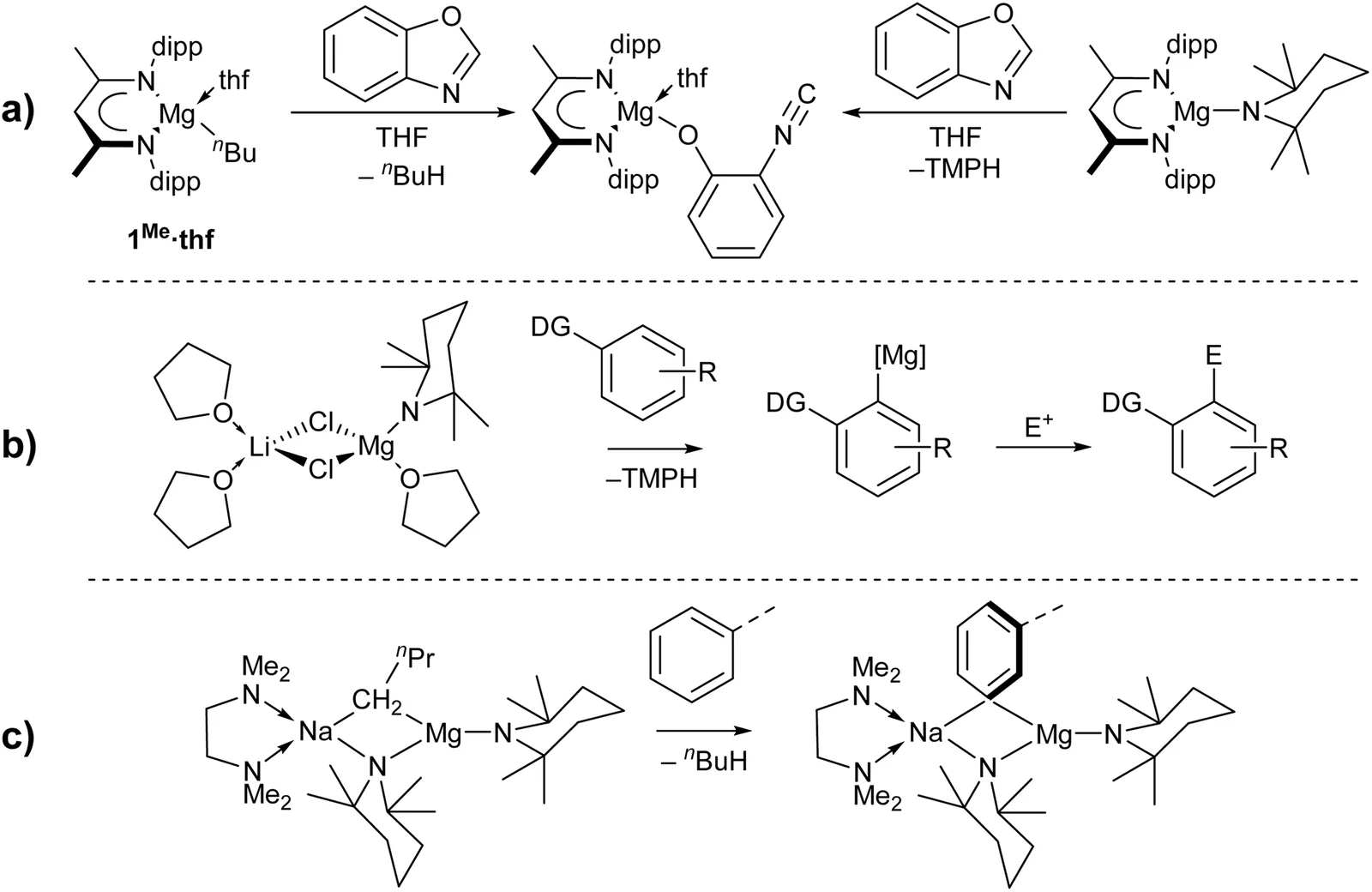
(a) Monometallic C–H magnesiation using β-diketiminato magnesium bases;^2^ (b) arene functionalisation *via* directed metalation with a ‘Turbo’ Hauser base (DG = directing group; E^+^ = electrophile);^9,10^ (c) metallation of benzene and toluene by a sodium magnesiate.^12^ dipp = 2,6-di-isopropylphenyl.

The strictly monometallic magnesiation of aromatic hydrocarbons is restricted to photo-activated Mg(i) dimers [LMg–MgL] or *in situ*-generated Mg(i) radicals. Jones and co-workers reported that UV irradiation of the Mg(i) dimer [(^Me^BDI)Mg]_2_ in benzene provided access to [(^Me^BDI)MgPh] (3^Me^) and hydride complex [2^Mg,Me^]_2_*via* a dimeric cyclohexadienyl intermediate [((^Me^BDI)Mg)_2_(µ^2^,µ^4^-C_6_H_6_)] (Scheme 2a).^18^ The same reaction in toluene, *ortho*-xylene, *meta*-xylene, or *para*-xylene resulted in the respective isolation of C(sp^2^)–Mg products [(^Me^BDI)Mg(C_6_H_4_-3-Me)], [(^Me^BDI)isoMg(C_6_H_4_-2,3-Me_2_)], [(^Me^BDI)Mg(C_6_H_4_-3,5-Me_2_)], and [(^Me^BDI)Mg(C_6_H_4_-2,5-Me_2_)], alongside [2^Mg,Me^]_2_.^18^ Photolysis of the related guanidinate derivative [(^i^Pr_2_NC(Ndipp)_2_)Mg]_2_ in toluene, *ortho*-xylene, *meta*-xylene, or mesitylene provided dimeric aryl-hydride complexes [(^i^Pr_2_NC(Ndipp)_2_)_2_Mg_2_(µ-Ar)(µ-H)] *via* selective C(sp^2^)–H activation, providing a mixture of magnesiated isomers in the case of toluene and xylenes, in preference to the more kinetically accessible and weaker C(sp^3^)–H bonds (Scheme 2a).^19^ Additionally, Crimmin and co-workers have reported the palladium-catalysed magnesiation of benzene by [(^Me^BDI^Mes^)Mg]_2_ (^Me^BDI^Mes^ = HC(C(Me)NMes)_2_; Mes = 2,4,6-trimethylphenyl) to provide [((^Me^BDI^Mes^)Mg)_2_(µ-Ph)(µ-H)].^20^ Palladium-^21^ and calcium^22^ catalysts have also been utilised to mediate the C–H alumination of benzene, toluene and xylenes through heterometallic cooperativity.

**Scheme 2:**
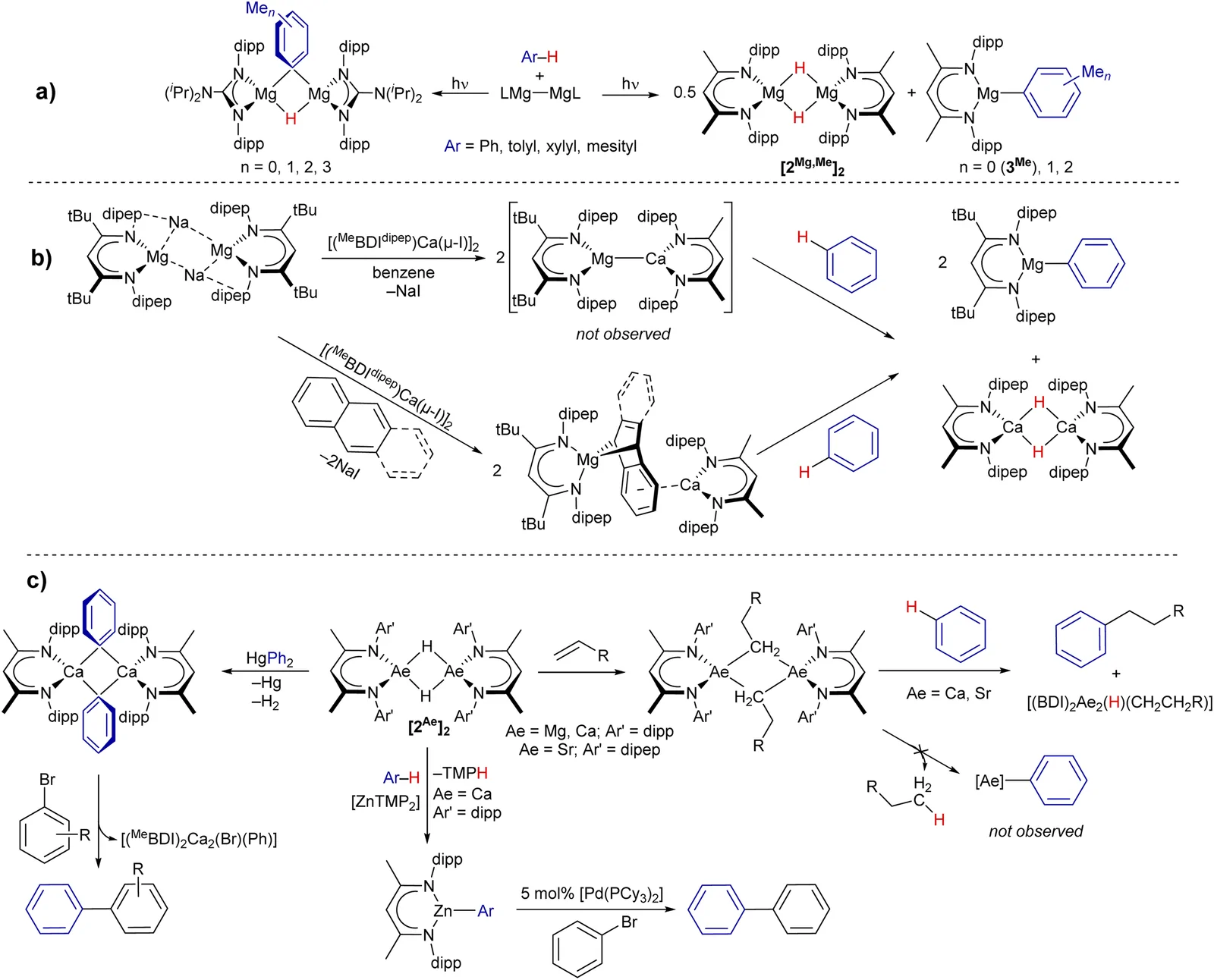
(a) Photolytic magnesiation of arenes by Mg(i) dimers;^18,19^ (b) magnesiation of benzene by transient or ‘masked’ Mg–Ca compounds;^27^ (c) arene functionalisation strategies involving β-diketiminato alkaline-earth hydride complexes ([2^Ae^]_2_): nucleophilic alkylation of benzene by calcium and strontium *n*-alkyl complexes,^28,29^ biaryl cross-coupling *via* calcium phenyl complex^32^ and zincation of hydrocarbon arenes by [2^Ca^]_2_ and [Zn(TMP)_2_].^35^ Note: not all byproducts and reaction stoichiometries are shown.

Harder and co-workers reported the isolation of [(^Me^BDI^dipep^)MgPh·(κ^1^-TMEDA)] (^Me^BDI^dipep^ = HC(C(Me)Ndipep)_2_, dipep = 2,6-di-isopentylphenyl; TMEDA = *N*,*N*,*N*′,*N*′-tetramethylethylenediamine) alongside [((^Me^BDI^dipep^)Mg)_2_(µ^2^,µ^4^-C_6_H_6_)] when [(^Me^BDI^dipep^)MgI(OEt_2_)] was reduced by sodium in benzene/TMEDA solution.^23^ Related to this work, the same group reported that attempts to synthesise compounds with Mg–Ca bonds by salt metathesis reactions of the unique Mg^0^ complex^24^ [(^tBu^BDI^dipep^)MgNa]_2_ with Ca^II^ reagents led to intramolecular C(sp^3^)–H activation^25,26^ or benzene C–H activation to yield [(^*t*Bu^BDI^dipep^)MgPh] and [(^Me^BDI^dipep^)Ca(µ-H)]_2_*via* a presumed heterometallic cyclohexadienyl intermediate [((^*t*Bu^BDI^dipep^)Mg)(µ^2^,µ^4^-C_6_H_6_)(Ca(^Me^BDI^dipep^))] (Scheme 2b).^27^ Isolable heterometallic naphthalene and anthracene derivatives, [((^*t*Bu^BDI^dipep^)Mg)(µ^2^,µ^4^-napth/anth)(Ca(^Me^BDI^dipep^))], also produce [(^*t*Bu^BDI^dipep^)MgPh] and [(^Me^BDI^dipep^)Ca(µ-H)]_2_ upon reaction with benzene (Scheme 2b).^27^ Notably, each of these examples (Scheme 2a and b) generates a stoichiometric equivalent of magnesium or calcium hydride in addition to the magnesium aryl moiety. Thus, a selective monometallic C–H deprotonation–magnesiation protocol remains to be achieved for wholly hydrocarbon arenes.

A major focus of our own research has been the reactivity of a base-free β-diketiminato calcium hydride complex, [(^Me^BDI)Ca(µ-H)]_2_ ([2^Ca^]_2_). Insertion of *n*-alkenes into the Ca–H bonds provides heteroleptic calcium *n*-alkyls, [(^Me^BDI)Ca(µ-R)]_2_, which react with benzene-*d*_6_*via* nucleophilic substitution to provide alkylated benzene RC_6_D_5_ derivatives under elimination of deuteride as a leaving group (Scheme 2c).^28^ Harder and co-workers reported that the strontium analogue [(^Me^BDI^dipep^)Sr(µ-H)]_2_ ([2^Sr^]_2_) and its *n*-alkyl derivatives display an even higher degree of nucleophilic reactivity towards benzene.^29^ Whilst unactivated *n*-alkenes also insert into the Mg–H bonds of β-diketiminato magnesium hydride complex [2^Mg,Me^]_2_ at elevated temperatures,^30^ nucleophilic arene alkylation akin to that observed for the heavier congeners was not observed. Arene deprotonation by [2^Ae^]_2_ or the corresponding alkyl derivatives has not been previously observed under the conditions studied. Heteroleptic calcium aryl complexes such as [(^Me^BDI)Ca(µ-Ph)]_2_ could, however, be accessed *via* reductive transmetallation of HgAr_2_,^31,32^ and employed in the uncatalysed biphenyl cross-coupling with aryl bromides (Scheme 2c).^32^ Extending this transmetallation approach to diorganozinc reagents provides access to alkyl calcium complexes^33^ including the previously inaccessible methyl derivative [(^Me^BDI)Ca(µ-Me)]_2_*via* organo-hydride calcium zincate intermediates.^34^ In a further recent advance,^35^ we found that reacting the diamidozinc reagent [Zn(TMP)_2_] with [2^Ca^]_2_ in aromatic solvents benzene, toluene or mesitylene results in C(sp^2^)–H metalation and transmetallation of the ^Me^BDI ligand to provide access to aryl zinc complexes [(^Me^BDI)ZnAr], the phenyl variant of which was utilised in the palladium-catalysed Negishi biaryl cross-coupling *via* a telescoped one-pot procedure (Scheme 2c). Whilst the same reaction in methylcyclohexane provided the highly basic calcium amide, [(^Me^BDI)Ca(TMP)], C(sp^2^)–H metalation chemistry in this system was ultimately deduced to be dependent on the synergistic action of both calcium and zinc *via* a hitherto unknown mechanism.

Here, we show that an *n*-butyl magnesium complex, [(^Ad^BDI)Mg^*n*^Bu] (1^Ad^), supported by a very bulky β-diketiminate ligand, ^Ad^BDI (HC(C(Ad)Ndipp)_2_; Ad = 1-adamantyl) deprotonates benzene, toluene, xylenes, and anisole to yield the corresponding aryl- and benzyl-magnesium products at 100–120 °C without requisite addition of any further heterometallic component. ^Ad^BDI counterintuitively promotes both faster reaction rate and higher selectivity compared to the significantly less bulky ^Me^BDI, such that, whilst [1^Me^]_2_ also readily mediates the directed *ortho*-metallation of anisole at 100 °C, magnesiation of benzene is extremely sluggish and competitive with β-hydride elimination. The use of [(^Ad^BDI)MgPh] (3^Ad^) obtained *via* benzene deprotonation as a source of nucleophilic phenyl in Kumada biaryl cross-coupling is also described.

## Results and discussion

### Magnesiation of benzene and 1,2,3,4-tetramethylcyclopentadiene

Our serendipitous entry-point to C–H magnesiation chemistry was provided by attempts to prepare 1,2,3,4-tetramethylcylopentadienyl (Cp^4M^) magnesium derivatives *via* protonolysis of *n*-butyl magnesium β-diketiminates [1^Me^]_2_ and 1^Ad^ with 1,2,3,4-tetramethylcylopentadiene (Cp^4M^H). Compound 1^Ad^ was straightforwardly synthesised by protonolysis of Mg^*n*^Bu_2_ with β-diimine, ^Ad^BDIH, at 60 °C (see SI for synthesis and characterisation of ^Ad^BDIH and 1^Ad^) and is structurally similar to other bulky monomeric BDI-magnesium *n*-butyl complexes [(^R^BDI^Ar^)Mg^*n*^Bu] (1^Ar,R^; Ar, R = dipp, ^*t*^Bu;^36^ dipep, ^*t*^Bu;^37^ dipp, ^i^Pr^38^). One molar equivalent (relative to magnesium) of Cp^4M^H was added to a benzene-*d*_6_ solution of 1^Ad^ or [1^Me^]_2_ and the sealed J. Young's NMR tube was heated to 100 °C (Scheme 3). In the case of [1^Me^]_2_ (Scheme 3a), periodic analysis by *in situ*^1^H NMR spectroscopy revealed the gradual disappearance of both the methylene resonance of Cp^4M^H (*δ* 2.57 ppm) and backbone methine resonance of [1^Me^]_2_ (*δ* 4.92 ppm) over the course of 6 days and appearance of a new compound (4), characterised by two singlets of equal relative intensity at *δ* 5.81, 4.70 ppm. Gradual evaporation of the reaction solvent at room temperature provided colourless needle-shaped single crystals suitable for X-ray diffraction analysis, the results of which confirmed the formation of the cyclopentadienyl magnesium derivative [(^Me^BDI)MgCp^4M^] (4), which was isolated in 78% yield and fully characterised.

**Scheme 3:**
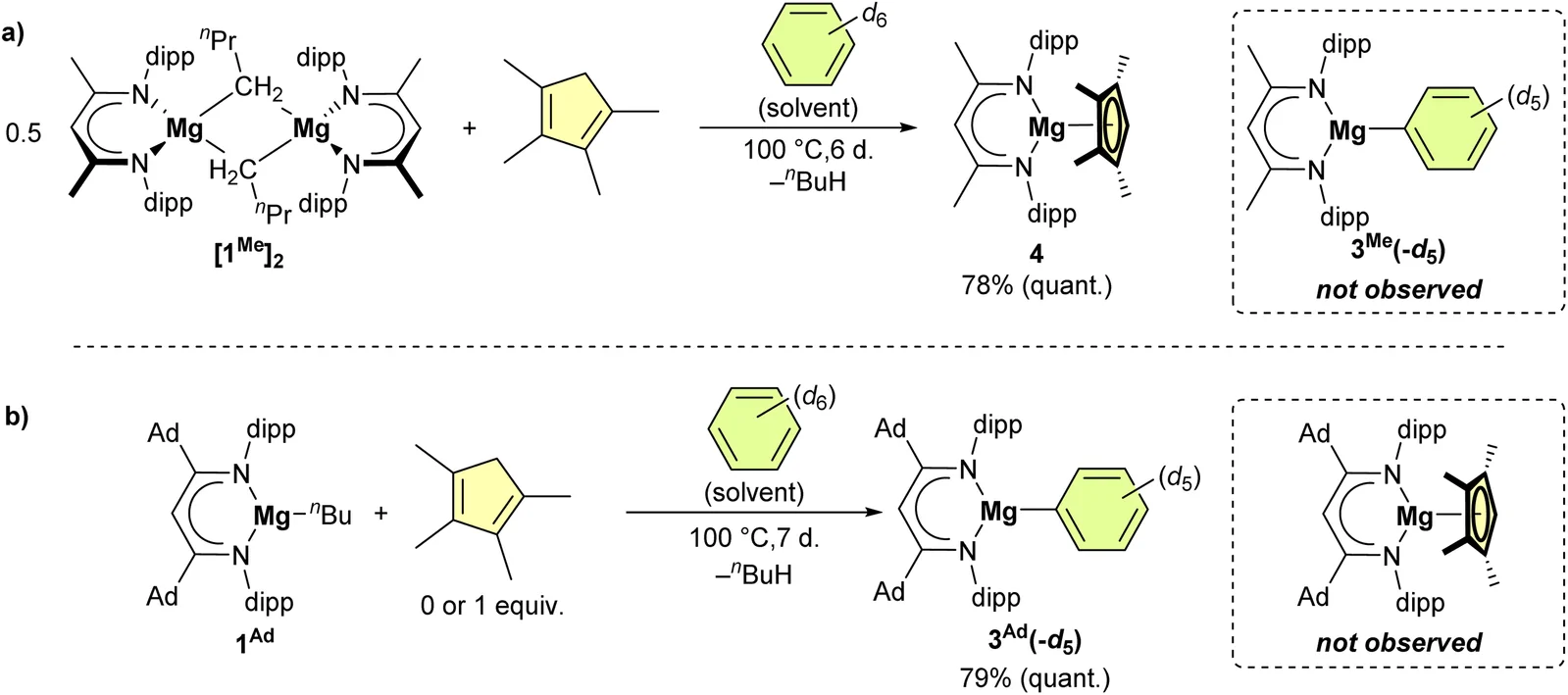
(a) reaction of [1^Me^]_2_ with Cp^4M^H to give 4 and (b) the reaction of 1^Ad^ with benzene(-*d*_6_) in the presence or absence of Cp^4M^H to give 3^Ad^(-*d*_5_). Isolated yields are shown with ^1^H NMR spectroscopic yields in parentheses.

The same reaction procedure starting from 1^Ad^ (Scheme 3b) resulted in a similar disappearance of the BDI-backbone methine signal (*δ* 5.67 ppm) and the magnesium-bound methylene multiplet (*δ* −0.62 ppm) in the ^1^H NMR spectrum and near-quantitative formation of a single new BDI-containing species (3^Ad^-*d*_5_) with a methine resonance at *δ* 5.74 ppm after 7 days at 100 °C. Whilst further scrutiny of the ^1^H NMR spectrum confirmed the evolution of *n*-butane (*δ*(CH_3_) 0.86 ppm), no evidence for the consumption of Cp^4M^H was observed and no resonance attributable to deprotonated Cp^4M^ was detected. Recrystallisation of the crude product from a saturated *n*-hexane/toluene solution at room temperature provided colourless block-shaped single crystals, which were isolated in 42% yield. Subsequent X-ray diffraction analysis revealed the structure of 3^Ad^-*d*_5_ as the magnesium phenyl-*d*_5_ complex, [(^Ad^BDI)Mg(C_6_D_5_)], formed by deprotonation of the reaction solvent. The same product was obtained in quantitative ^1^H NMR spectroscopic yield when the reaction was repeated in the absence of Cp^4M^H. When 1^Ad^ was heated in *proteo*-benzene for 7 days in either the presence or absence of Cp^4M^H, the non-deuterated complex [(^Ad^BDI)MgPh] (3^Ad^) was isolated as a pale-yellow solid in 79% yield after removal of volatiles (quantitative by NMR). The *ortho*-CH protons of the magnesium-bound phenyl moiety in compound 3^Ad^ display a characteristic doublet at *δ* 6.17 ppm in the ^1^H NMR spectrum, which integrates 2H relative to the BDI-backbone methine (*δ* 5.74 ppm), whilst the *meta*- and *para*-CH protons give rise to a multiplet (3H) at *δ* 7.05 ppm. Resonances at the same chemical shifts were observed in the corresponding ^2^H NMR spectrum of 3^Ad^-*d*_5_. Notably, benzene-*d*_6_ solutions of 3^Ad^ gradually transform into the isotopically labelled compound 3^Ad^-*d*_5_ in quantitative ^1^H NMR spectroscopic yields after 3 days at 100 °C, due to exchange with the NMR solvent.

The crystal structure of compound 4 contains a *pseudo*-3-coordinate magnesium centre in a distorted trigonal pyramidal geometry with the magnesium essentially co-planar to the BDI ligand backbone (Fig. 1a). Although disorder with respect to rotation about the Cp(centroid)–Mg axis prevents detailed discussion of distances and angles, the bulky Cp^4M^ ligand clearly adopts an η^5^-coordination mode, with identical Mg1–Cp(plane) and Mg1–Cp(centroid) distances within error (2.055(3), 2.056(3) Å, respectively; calculated for the major component of the disordered Cp^4M^ ligand). The Mg1–Cp(centroid) distance is expectedly greater than those of [(^Me^BDI)MgCp] (2.0463(9) Å)^39^ and [(^Me^BDI^Mes^)MgCp] (2.0391(8), 2.0377(9) Å), but shorter than that in the even more sterically congested derivative [(^Me^BDI)MgCp*] (2.0781(6) Å).^40^

**Fig. 1:**
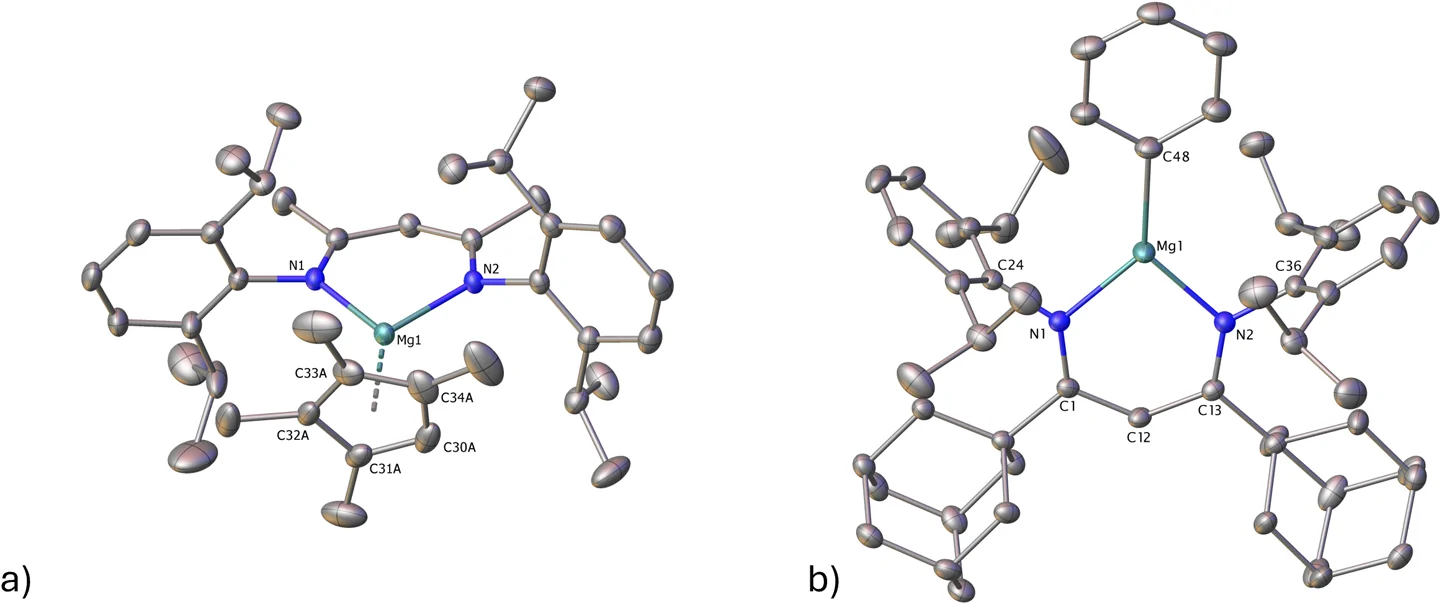
Displacement ellipsoid plots (30% level) of (a) 4 and (b) 3^Ad^. Minor component of the disordered Cp^4M^ in 4, and of the disordered isopropyl and adamantyl moieties in 3^Ad^ are omitted. Hydrogen atoms are also omitted for clarity. Distances (Å) and angles (°) for 4:* Mg1–N1 2.0670(12), Mg1–N2 2.0708(11), Mg1–C30A 2.331(4), Mg1–C31A 2.364(4), Mg1–C32A 2.396(5), Mg1–C33A 2.412(10), Mg1–C34A 2.400(6), Mg1–Cp(centroid) 2.056(3), N1–Mg1–N2 92.55(4), N1–Mg1-(centroid) 135.27(10), N2–Mg1-(centroid) 132.13(10). *Distances and angles involving the Cp^4M^ moiety refer to major component only. Distances (Å) and angles (°) for 3^Ad^: Mg1–N1 2.0162(10), Mg1–N2 2.0091(10), Mg1–C48 2.1052(13), C48–C49 1.402(2), C48–C53 1.404(2), C49–C50 1.3926(19), C50–C51 1.376(2), C51–C52 1.375(2), C52–C53 1.390(3), N1–Mg1–C48 133.39(5), N2–Mg1–N1 94.74(4), N2–Mg1–C48 131.72(5).

The crystal structure of 3^Ad^ (Fig. 1b) also presents a 3-coordinate distorted trigonal planar coordination geometry at magnesium (*Σ*_angles_(Mg) = 359.84°), with a Mg1–C48 distance (2.1052(13) Å) that is essentially the same as that in the less sterically hindered derivative 3^Me^ (2.095(3) Å).^41^ Additionally, the phenyl ligand is almost co-planar with the BDI ligand (C53–C48–Mg1–N2 = 5.30(16)°; C49–C48–Mg1–N1 5.25(16)°), such that the *ortho*-CH atoms point towards the centroids of the flanking dipp phenyl rings. Due to the effect of the bulky backbone adamantyl groups, the dipp groups are pushed forwards, resulting in larger C^ipso^–N–C angles (129.02(9), 127.57(10)°) compared to 3^Me^ (120.39(11)°) and significantly smaller fold angles between the mean planes of the dipp rings (3^Ad^ = 104.7(5)°, 3^Me^ = 126.6(1)°). The bending angles between the N–C^ipso^ bonds and the dipp centroids are highly asymmetric (centroid-C24–N1 = 170.45(10), centroid-C36–N2 = 174.59(10)°), but smaller on-average (172.52(10)°) compared to 3^Me^ (174.30(14)°). The C^ipso^–N–C angles in 3^Ad^ are also larger than those in the similarly 3-coordinate [(^*t*Bu^BDI^dipep^)MgPh] (3*^t^*^Bu,dipep^; average C^ipso^–N–C = 126.52°),^24^ whilst the fold angle between the dipp rings and the centroid-C^ipso^–N angles are both more acute than those observed in 3*^t^*^Bu,dipep^ (average fold angle = 114.44°; average centroid-C^ipso^–N = 174.44°), probably due to the bulkier dipep groups in the latter compound.

In contrast to 1^Ad^, when a benzene-*d*_6_ solution of [1^Me^]_2_ was heated to 100 °C in the absence of Cp^4M^H, negligible change to the ^1^H NMR spectrum was observed after 1 week, and phenyl complex 3^Me^-*d*_5_ was not observed. Heating the same solution at an increased temperature of 120 °C for 2 weeks, however, resulted in conversion of most of the starting complex into two new BDI-containing species, which were identified as the known phenyl 3^Me^-*d*_5_ (39% ^1^H NMR spectroscopic yield) and hydride [2^Mg,Me^]_2_ (48%) complexes by comparison to the reported NMR data (Scheme 4).^41,42^ The presence of 1-butene in the same ^1^H NMR spectrum suggests that [2^Mg,Me^]_2_ is formed through β-hydride elimination of [1^Me^]_2_. To identify whether the magnesium hydride [2^Mg,Me^]_2_ reacts with the solvent, a separately synthesised sample of [2^Mg,Me^]_2_ was also heated at 120 °C in benzene-*d*_6_ for 2 weeks. Subsequent analysis of the reaction mixture by ^1^H NMR spectroscopy confirmed the selective formation of 3^Me^-*d*_5_ in 38% spectroscopic yield.

**Scheme 4:**
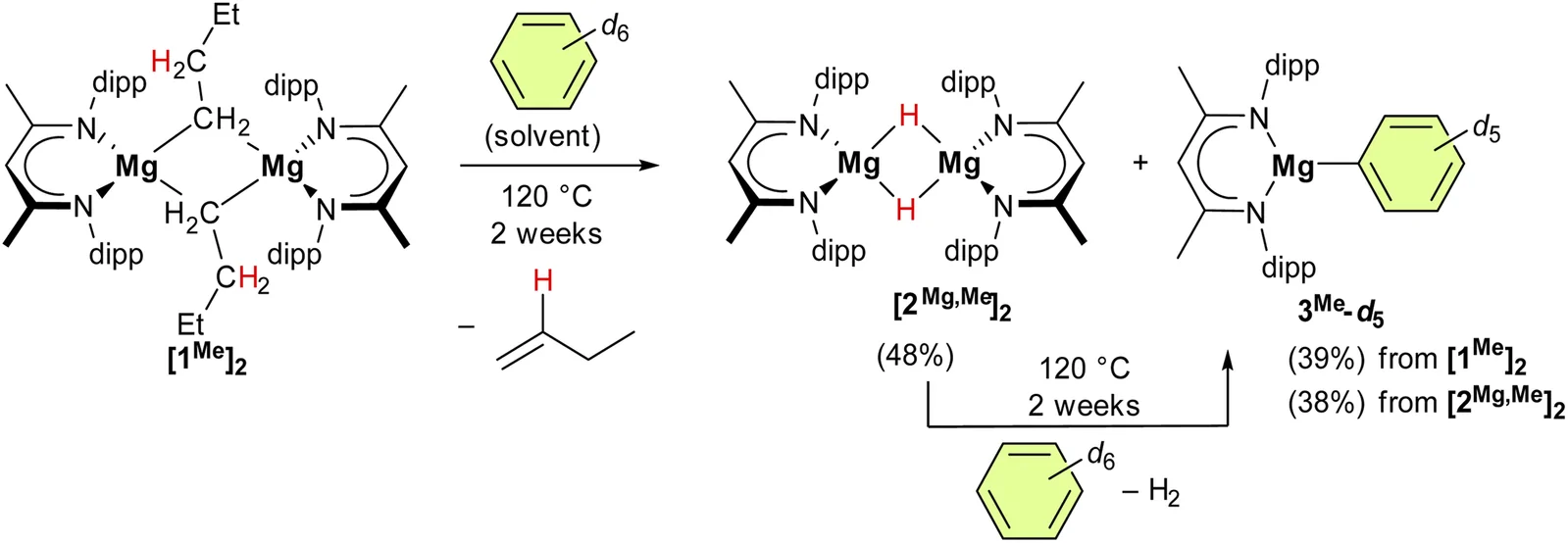
Reaction of [1^Me^]_2_ and [2^Mg,Me^]_2_ with benzene-*d*_6_. ^1^H NMR spectroscopic yields shown in parentheses.

The divergence in reactivity of 1^Ad^ compared to [1^Me^]_2_ and [2^Mg,Me^]_2_, which further contrasts to their heavier congeners, [(^Me^BDI)Ca(µ-R)]_2_ (R = *n*-alkyl) and [2^Ca^]_2_, is striking. Whilst the bulky, monomeric magnesium *n*-butyl derivative 1^Ad^ reacts as a base, deprotonating benzene under forcing conditions *via* C–H activation, the analogous reaction for [1^Me^]_2_ is apparently less kinetically facile and competitive with β-hydride elimination to generate [2^Mg,Me^]_2_, which also slowly deprotonates benzene to provide 3^Me^. By contrast, [2^Ca^]_2_ readily undergoes H/D exchange at 60 °C in benzene-*d*_6_*via* nucleophilic aromatic substitution.^43^ Comparable nucleophilic behaviour is observed for [(^Me^BDI)Ca(µ-R)]_2_, yielding [(^Me^BDI)Ca(µ-D)]_2_ and C_6_D_5_R at 60 °C rather than solvent deprotonation.^28^

### Steric profile of backbone-substituted β-diketiminate ligands

To begin to rationalise the faster, and more selective, reaction of 1^Ad^ with benzene compared to the less sterically hindered, yet dimeric [1^Me^]_2_, the steric bulk of 1^Ad^ was compared to other crystallographically characterised monomeric 1^Ar,R^ derivatives (Ar, R = dipp, ^*t*^Bu;^36^ dipep, ^*t*^Bu;^37^ dipp, ^i^Pr^38^), and to the computationally optimised structure of the monomerised form of [(^Me^BDI)Mg^*n*^Bu] (1^Me^) (see SI for computational details). Comparing the crystal structure of 1^Ad^ (Fig. 2a) with the calculated structure of 1^Me^, it is apparent that the bulky adamantyl groups force the flanking dipp groups forwards, constraining the binding pocket around the magnesium centre (Fig. 2c). This can be quantified by comparing the C^ipso^–N–C angles (1^Ad^ = 126.31(8), 127.81(8)°), which are similar to those of 1^dipp,^*^t^*^Bu^ (126.4(4), 127.1(4)°)^36^ and 1^dipep,^*^t^*^Bu^ (126.13(9), 126.09(10)°),^37^ but significantly greater than that of 1^dipp,iPr^ (120.72(12), 120.78(12)°),^38^ and 1^Me^ (120.02, 120.05°). Additionally, the fold-angle between the mean planes of the dipp phenyl rings is significantly smaller for 1^Ad^ (112.53(4)°) compared to 1^Me^ (131.51°) and 1^dipp,iPr^ (125.89(6)°), but slightly greater than that of 1^dipp,^*^t^*^Bu^ (110.4(2)°). It should, however, be noted that the fold angle is also influenced by bending of the C^ipso^–N bond out of the mean plane of the dipp ring. The dipp centroid-C^ipso^–N angles for 1^Ad^ are smaller on average (centroid-C24–N1 = 176.93(9)°, centroid-C36–N2 = 171.66(8)°, average = 174.30°) compared to 1^Me^ (176.25, 176.32°), but highly asymmetric, possibly due to attractive dispersion forces between the dipp group and the *n*-butyl chain. Visual inspection of the crystal structure of 1^Ad^ in a space filling representation (Fig. 2b) indicates that the magnesium centre remains relatively accessible, especially if its coordination environment should distort to a more pyramidal geometry. A more extensive comparison of the buried volume (%*V*_bur_) and steric mapping using the SambVca web tool^44^ across the series of *n*-butyl complexes 1^Ad^ and 1^Ar,R^ shows that steric crowding of the metal centre in ^Ad^BDI^dipp^ (1^Ad^, %*V*_bur_ = 65.7%, XRD) is comparable to ^tBu^BDI^dipp^ (1^dipp,^*^t^*^Bu^, %*V*_bur_ = 67.4%, XRD)^36^ and ^*t*Bu^BDI^dipep^ (1^dipep,^*^t^*^Bu^, %*V*_bur_ = 66.0%, XRD),^37^ but significantly greater than ^Me^BDI^dipp^ (1^Me^, %*V*_bur_ = 60.3%, DFT), with ^iPr^BDI^dipp^ (1^dipp,iPr^, %*V*_bur_ = 62.6%, XRD)^38^ intermediate in the series. The distribution of steric bulk around the metal centre (*i.e.* the shape of the coordination ‘pocket’) is similar across the series, but the bulky backbone groups clearly cause the the *N*-aryl substituents to project further forwards (Fig. 2d; SI Section 3).

**Fig. 2:**
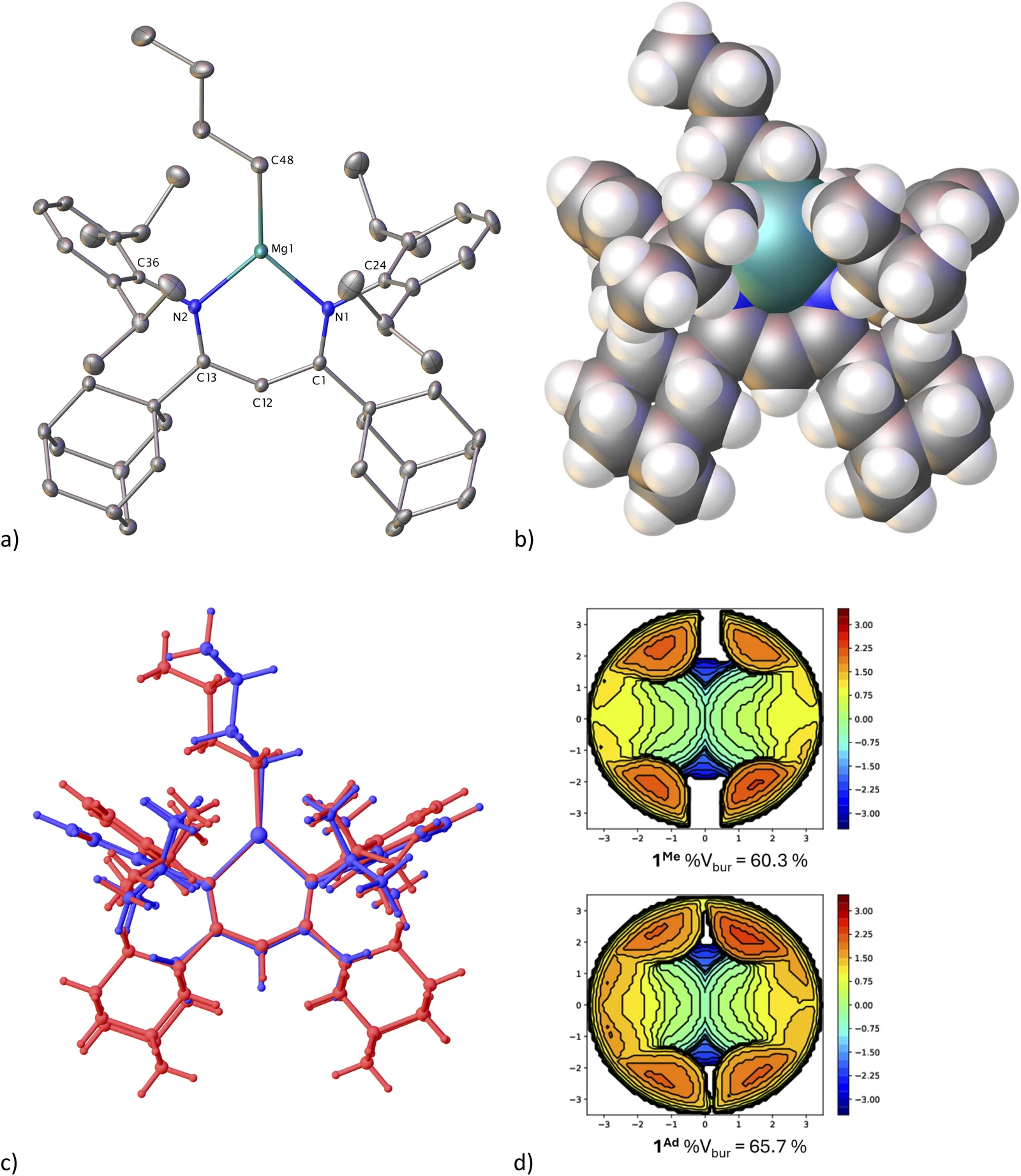
(a) Displacement ellipsoid plot (30%) of the crystal structure of 1^Ad^; (b) space filling representation of the crystal structure of 1^Ad^; (c) overlay of the crystal structure of 1^Ad^ (red) with the calculated structure of 1^Me^ (blue); (d) steric contour maps generated by the SambVca web tool^44^ from the crystal structure of 1^Ad^ and the calculated structure of 1^Me^ including hydrogen atoms and using the magnesium centre to define the centre of a sphere of radius 3.5 Å. Hydrogen atoms are omitted from (a) for clarity. Disordered solvent (methylcyclohexane) in the crystal structure of 1^Ad^ is omitted. Selected distances (Å) and angles (°) for 1^Ad^: Mg1–N1 2.0257(9), Mg1–N2 2.0266(9), Mg1–C48 2.1074(12), N1–Mg1–N2 95.29(4), N1–Mg1–C48 132.81(4), N2–Mg1–C48 131.76(4), C24–N1–C1 126.31(8), C36–N2–C13 127.81(8). See Table S13 in the SI for further comparison of distances and angles for magnesium *n*-butyl complexes 1^Ar,R^.

### Calculation of reaction pathway by DFT

To gain further mechanistic insight, reaction pathways for the reaction of 1^Ad^ and [1^Me^]_2_ with benzene (Fig. 3) and the competing β-hydride elimination (Fig. 4) were calculated using DFT methods at the BP86-D3^BJ^(SMD = C_6_H_6_)/def2-TZVPP//BP86/def2-SVP level. Starting from monomeric *n*-butyl complexes 1^R^ (R = Ad, Me), deprotonation of benzene was calculated to proceed *via* an encounter complex, A^R^ (A^Ad^ = 3.0 kcal mol^−1^, A^Me^ = 2.7 kcal mol^−1^), the transition state TS(A^R^–B^R^) (R = Ad, 38.9 kcal mol^−1^; R = Me, 32.9 kcal mol^−1^), and dissociation of *n*-butane from intermediate B^R^ to provide 3^Ad^ (−5.7 kcal mol^−1^) or 3^Me^ (−7.6 kcal mol^−1^). Whilst the transition state TS(A^R^–B^R^) is 6 kcal mol^−1^ lower for the methyl-substituted system, the dimeric *n*-butyl complex [1^Me^]_2_ was calculated to reside 2.3 kcal mol^−1^ lower on the potential energy surface relative to monomeric 1^Me^. Thus, when the prerequisite dissociation of dimeric [1^Me^]_2_ is accounted for, the total calculated barrier to benzene deprotonation is comparable for both the adamantyl and methyl cases.

**Fig. 3:**
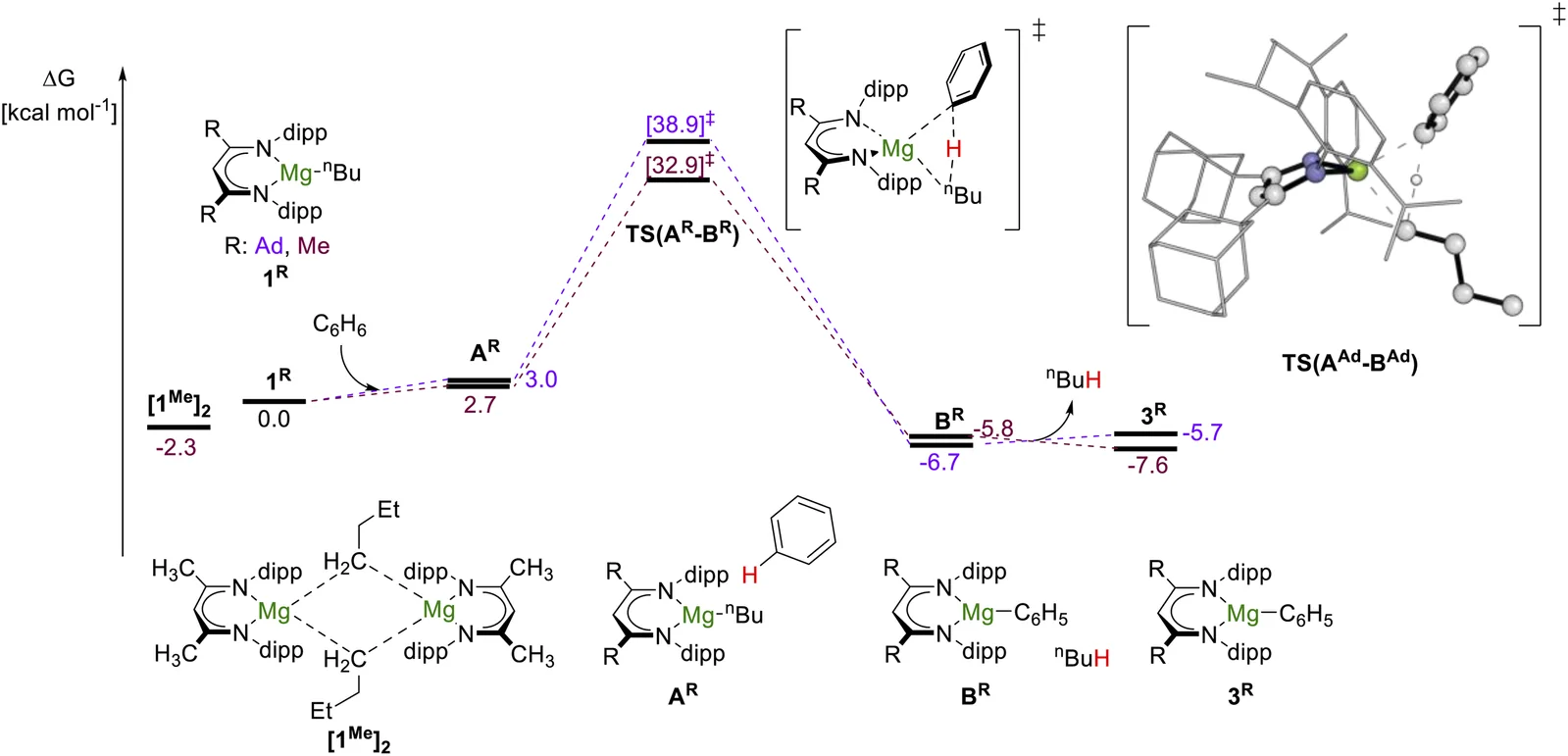
DFT-calculated potential energy surface (BP86-D3^BJ^(SMD = C_6_H_6_)/def2-TZVPP//BP86/def2-SVP) for the deprotonation and mangesiation of benzene by [1^Me^]_2_ or 1^Ad^ to give respective phenyl complexes 3^Me^ and 3^Ad^, with a depiction of the calculated transition state TS(A^Ad^–B^Ad^).

**Fig. 4:**
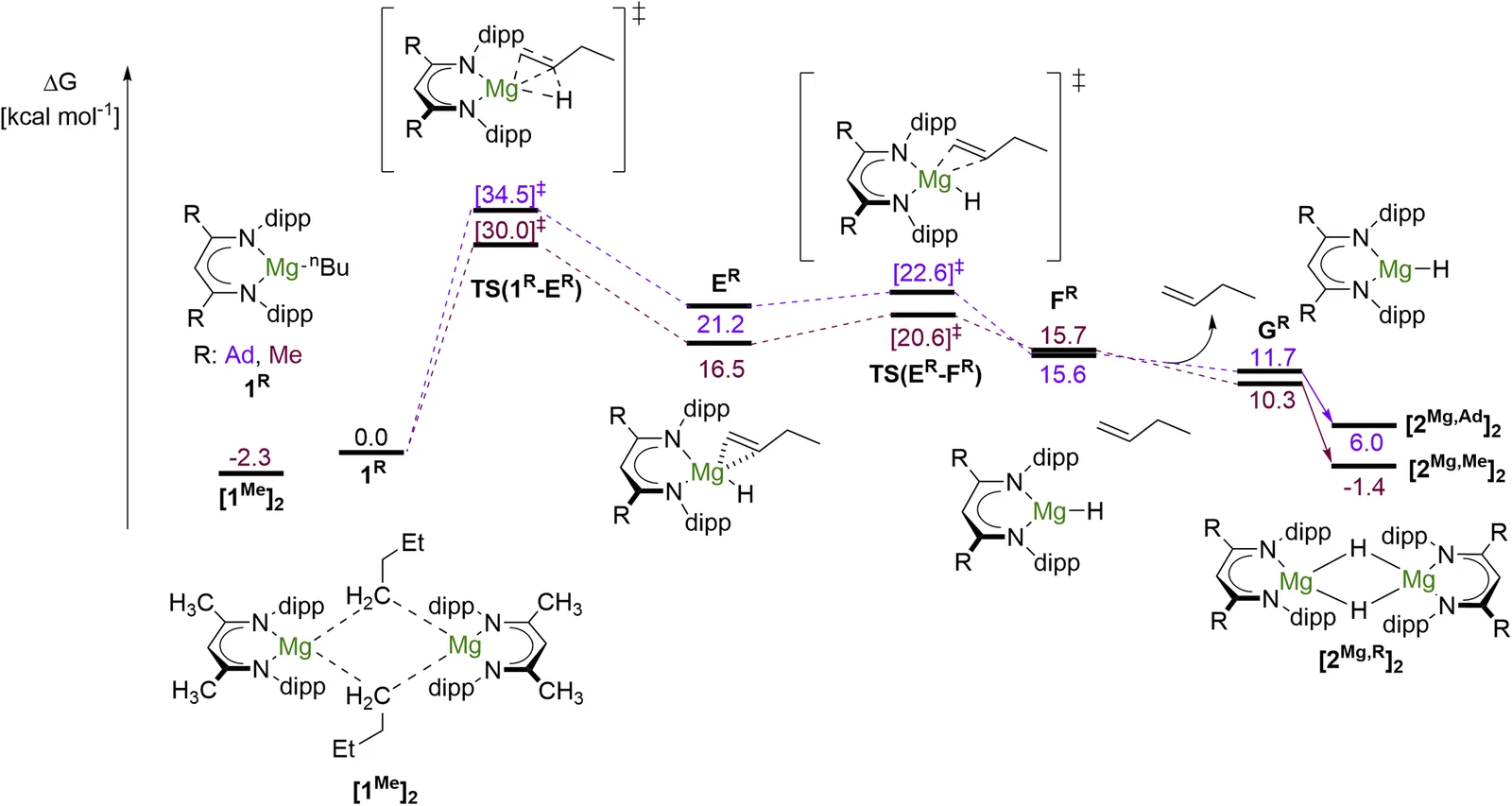
DFT-calculated potential energy surface (BP86-D3^BJ^(SMD = C_6_H_6_)/def2-TZVPP//BP86/def2-SVP) for the β-hydride elimination of 1-butene from [1^Me^]_2_ or 1^Ad^.

Closer inspection of the calculated intrinsic reaction coordinate for R = Ad revealed an inflection point at 20.1 kcal mol^−1^, prior to TS(A^Ad^–B^Ad^) (38.9 kcal mol^−1^). Although the structure at this point (A′^Ad^) is not a local minimum, it is informative to note that the BDI ligand backbone is twisted (NCCN torsion = 16.7°) and the magnesium centre projected some 1.219 Å from the mean plane of the ligand backbone, whilst the incoming benzene molecule remains separated from the metal by more than 7 Å (see Table S14 in the SI). The substantial structural distortion accounts for more than half of the total energy barrier, and results in far greater accessibility of the *n*-butyl base and magnesium centre to the substrate. In TS(A^Ad^–B^Ad^) (Fig. 3), the magnesium centre is displaced from the mean plane of the β-diketiminate ligand backbone by 0.714 Å, whilst the ligand backbone itself is somewhat twisted, as indicated by the NCCN torsion angle of 11.9°. For R = Me, significantly less structural reorganisation is required; the magnesium centre is relatively less crowded for TS(A^Me^–B^Me^) (Fig. S291), such that it is only projected from the BDI mean plane by 0.490 Å, and the NCCN torsion angle is essentially nil. In the transition state TS(A^R^–B^R^), the Mg–C^Bu^ bond is elongated (R = Ad, 2.334 Å; Me, 2.323 Å) compared to the preceding intermediate A^R^ (R = Ad, 2.139 Å; Me, 2.135 Å), such that the proton is directly transferred from benzene to the carbanionic carbon of the *n*-butyl chain with a near-linear C^Ar^–H–C^Bu^ arrangement (Fig. 3 and S291). The geometry of TS(A^R^–B^R^) closely resembles that calculated for the deprotonation of benzene by [(^Me^BDI)Ca(TMP)].^35^

The calculated reaction coordinate was not found to involve a Mg⋯π interaction, and TS(A^R^–B^R^) also does not involve any interaction of the benzene π system with the metal centre. The lack of such an interaction is in marked contrast to the nucleophilic alkylation of benzene by congeneric BDI–calcium, strontium, and ytterbium(ii) alkyl complexes. In these cases, poly-hapto π-coordination was calculated to play a crucial role in activating the arene substrate towards nucleophilic attack and stabilising the resulting Meisenheimer intermediate.^28,29,45^ The contrasting reaction outcomes and calculated mechanisms are consistent with the smaller size of magnesium, which causes the metal centre to be less sterically acessible to the π-face of benzene, besides leading to a more charge dense and less polarisable (harder) metal centre that is less able to interact strongly with the (soft) π-electron density of aromatic rings. Further, the significantly less polarised metal–carbon bond compared to the heavier congeners is expected to result in relatively attenuated nucleophilicity.

The occurrence of proton transfer during transition state TS(A^R^–B^R^) would be expected to yield a large primary KIE. In contradiction to this assumption, however, periodic ^1^H NMR analysis of the *pseudo* first-order reaction of 1^Ad^ with benzene-*d*_6_ and with *proteo*-benzene at 102 °C revealed a very small secondary kinetic isotope effect (KIE) of 
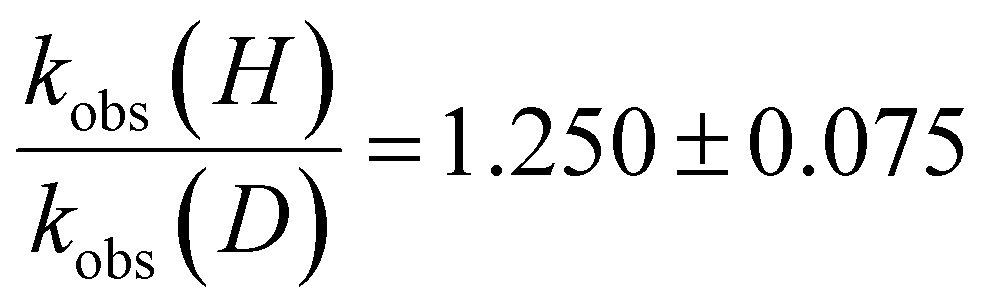
 (Fig. S264 and S265 in the SI). The significant contribution that structural reorganisation makes to the total energy barrier of TS(A^R^–B^R^)*via* distorted structure A′^Ad^ (Table S14) would be expected to provide a relatively small KIE value. Given the very small magnitude of the experimental KIE and considering the dimeric mechanisms previously calculated for related strontium-^29^ and ytterbium-mediated^45^ nucleophilic alkylation reactions, therefore, an alternative mechanism was considered (see Tables S15 and S16 in the SI), where endergonic dimerization prior to deprotonation would be consistent with a secondary KIE. Attempts to computationally optimise a dimeric structure for 1^Ad^ (*i.e.* [(^Ad^BDI)Mg(µ-^*n*^Bu)]_2_) were, however, unsuccessful. Whilst transition states involving two magnesium centres in a benzene deprotonation step were found, they are not connected to dimeric reactants or products in the IRC, and the energy barriers associated with them are prohibitively high (R = Me, 49.2 kcal mol^−1^; R = Ad, 52.0 kcal mol^−1^; see SI, Tables S15 and S16). Noting previous examples of very bulky BDI ligands adopting a ring-opened κ^1^-amidoimine binding mode,^37^ an alternative energy pathway *via* ring-opened two-coordinate intermediate [(κ^1-Ad^BDI)Mg^*n*^Bu] was also calculated (see Fig. S307 and S308 in the SI) but found to be kinetically unviable.

Starting from monomeric *n*-butyl complex 1^R^, β-hydride elimination (Fig. 4) was calculated to traverse transition state TS(1^R^–E^R^) (R = Ad, 34.5 kcal mol^−1^; R = Me, 30.0 kcal mol^−1^), before dissociation of 1-butene from magnesium hydride-butene intermediate E^R^ to provide a monomeric terminal hydride, G^R^ (R = Ad, 11.7 kcal mol^−1^; R = Me, 10.3 kcal mol^−1^). Subsequent dimerization of G^Me^ to form the experimentally observed magnesium hydride dimer [2^Mg,Me^]_2_ leads to a near-thermoneutral process (−1.4 kcal mol^−1^), whilst dimerization of G^Ad^ is much less advantageous, and the corresponding hypothetical dimeric hydride [2^Mg,Ad^]_2_ was calculated to reside at 6.0 kcal mol^−1^. Taking into account the endergonic (2.3 kcal mol^−1^) dissociation of dimeric [1^Me^]_2_, the total barrier to formation of [2^Mg,Me^]_2_ and 1-butene *via* β-hydride elimination is competitive with that for benzene deprotonation and formation of 3^Me^ and *n*-butane. For 1^Ad^, the β-hydride elimination pathway is calculated to be endergonic by 6.0 kcal mol^−1^. Formation of 3^R^*via* benzene deprotonation by monomeric hydride G^R^ was calculated to be endergonic (R = Ad, 4.0 kcal mol^−1^; R = Me, 3.5 kcal mol^−1^; relative to G^R^), traversing a transition state of 30.9 kcal mol^−1^ (R = Ad) or 30.1 kcal mol^−1^ (R = Me; see Fig. S309 in the SI).

Overall, the computational study suggests that whilst benzene deprotonation and β-hydride elimination pathways are energetically competitive for both 1^Ad^ and 1^Me^, the latter process is thermodynamically disfavoured for the bulkier derivative due to the lower stability of the hypothetical µ-hydride dimer ([2^Mg,Ad^]_2_). The preference for monomeric structures exerted by the bulkier ^Ad^BDI ligand causes the β-hydride elimination pathway to be thermodynamically disfavoured. Although these results do not account for the lower reaction temperature and significantly faster reaction rate for deprotonation of benzene by 1^Ad^ compared to [1^Me^]_2_, or the unusually low KIE, the combined experimental and computational data emphasise that even subtle differences in kinetics afforded by variations in the ligand scaffold can result in highly divergent reaction outcomes.

### Deprotonation and magnesiation of toluene

The remarkable kinetic discrimination of 1^Ad^ for benzene over the hindered, yet more acidic, Cp^4M^H prompted an investigation of the broader scope of arene deprotonation. Heating a toluene-*d*_8_ solution of 1^Ad^ and Cp^4M^H to 100 °C for 8 days in a sealed J. Young's NMR tube resulted in 89% conversion of the starting complex to a mixture of several BDI-containing products, according to *in situ*^1^H NMR spectroscopy, and the deposition of colourless block-shaped single crystals at the meniscus. A subsequent X-ray diffraction experiment provided a structure solution which, albeit hampered by significant disorder, was fully consistent with *para*- and *meta*-magnesiation of the reaction solvent to provide the magnesium tolyl complexes [(^Ad^BDI)Mg(C_6_D_4_-3-CD_3_)] (5-*m-d*_7_) and [(^Ad^BDI)Mg(C_6_D_4_-4-CD_3_)] (5-*p-d*_7_) as a disordered co-crystalline mixture (Fig. 5a. Occupancy 0.76 : 0.24, respectively, see SI for details). Further unassigned electron density in the difference map, however, hinted at the possible presence of a third minor co-crystalline component and analysis of the isolated crystals by ^1^H NMR spectroscopy in benzene-*d*_6_ was consistent with this. The reaction was, thus, repeated in *proteo*-toluene at the higher temperature of 120 °C in the absence of Cp^4M^H. After 13 days, volatiles were removed under vacuum. Analysis of the crude product mixture by ^1^H and ^13^C NMR spectroscopy was consistent with the quantitative consumption of 1^Ad^ to form a 1 : 2 : 2 mixture of the tolyl derivatives 5-*p*, 5-*m*, and the benzyl complex [(^Ad^BDI)Mg(CH_2_C_6_H_5_)] (5′; Fig. 6, Scheme 5). Compound 5′ was also separately synthesised from [(^Ad^BDI)MgBr] (13, *vide infra*) and benzyl potassium. Surprisingly, the only previous example of a crystallographically characterised three-coordinate benzyl magnesium complex is the potassium magnesiate [(tmeda)K-η^6^-C_6_H_5_CH_2_-Mg(N(SiMe_3_)_2_)].^46^ In the ^1^H NMR spectrum (Fig. 6), compound 5-*m* displays two characteristic doublets at *δ* 5.83 (*J* = 2.0 Hz, 1H) and 6.04 (*J* = 6.9 Hz, 1H) ppm, which correspond to the protons in the 2- and 6-positions of the tolyl rings, respectively. The analogous *ortho*-CH protons in 5-*p* appear as a single doublet at *δ* 6.08 (*J* = 7.6 Hz, 2H) ppm. Each of these species also displays a respective singlet at *δ* 2.16 and 2.08 ppm for the tolyl-methyl environment. The respective *ortho*-, *para*- and *meta*-benzyl-resonances of compound 5′ can be identified in the ^1^H NMR spectrum by a set of mutually coupled doublet of doublets, triplet of triplets, and apparent triplet signals at *δ* 6.21 (*J* = 7.9, 1.0 Hz, 2H), 6.75 (*J* = 7.3, 1.3 Hz, 1H), 6.95 (*J* = 7.6 Hz, 2H) ppm. The magnesium-bound methylene group gives a singlet (2H) in the ^1^H NMR spectrum at *δ* 1.07 ppm. Accordingly, 1^Ad^ deprotonates toluene at the *meta*- and *para*-C(sp^2^)–H positions to provide the statistical mixture of tolyl isomers, as well as the benzylic C(sp^3^)–H position to form the benzyl derivative. The hindered *ortho*-C(sp^2^)–H remains inert under these conditions.

**Fig. 5:**
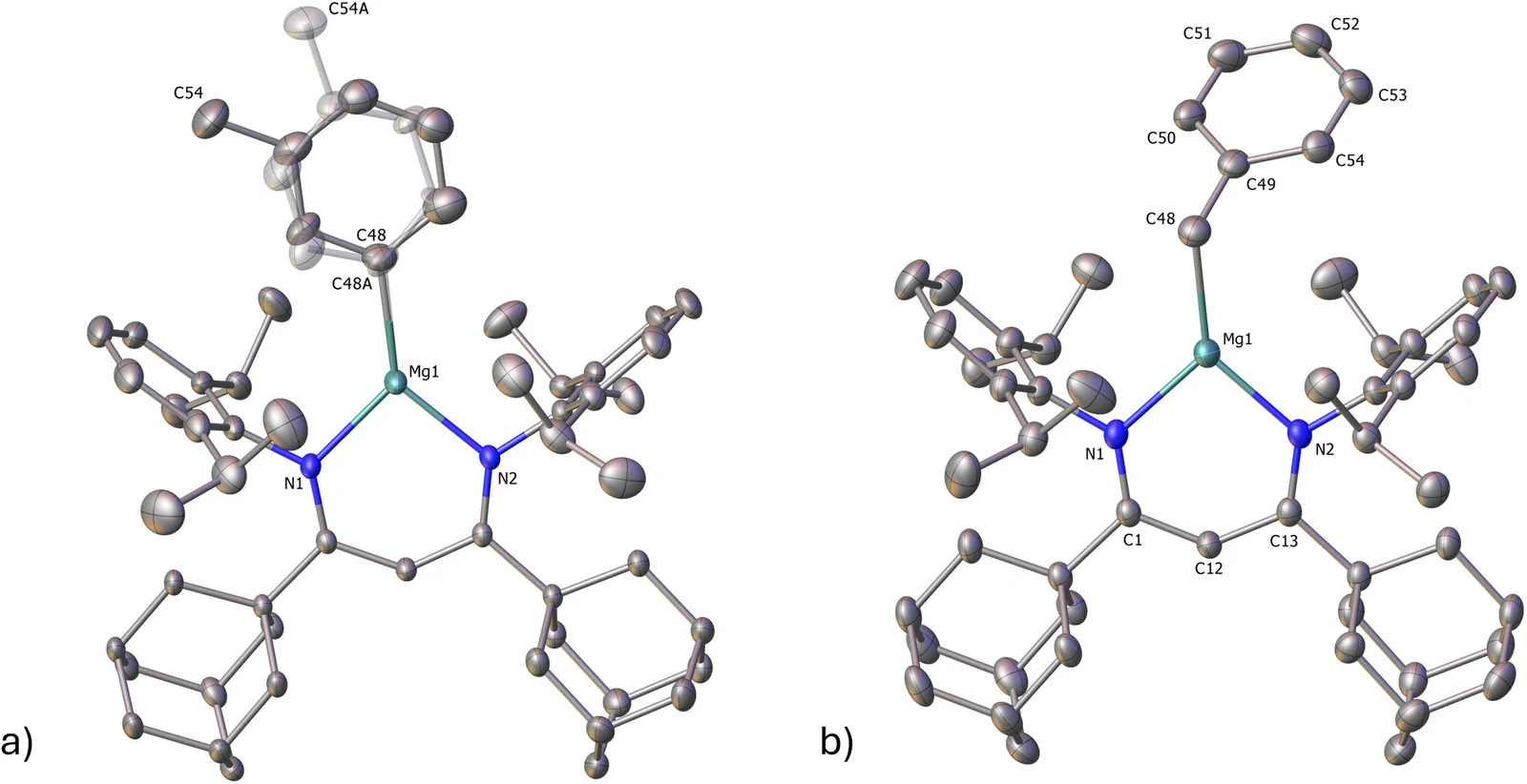
Displacement ellipsoid plots (30%) of (a) the Mg1-containing molecule in the X-ray structure solution of the disordered co-crystal of 5-*m*/5-*p*, with the minor component (5-*p*) shown semi-transparent; (b) compound 5′. Hydrogen atoms, disordered solvent molecules, and the Mg2-containing molecule of 5-*m*/5-*p* are omitted for clarity. Distances and angles for 5′: Mg1–N1 2.0182(13), Mg1–N2 2.0131(14), Mg1–C48 2.1160(19), C48–C49 1.477(3), N1–Mg1–C48 127.46(7), N2–Mg1–N1 95.41(5), N2–Mg1–C48 136.53(7), C49–C48–Mg1 118.40(13). Distances and angles for 5-*m*/5-*p* are not discussed due to disorder of the tolyl moiety.

**Fig. 6:**
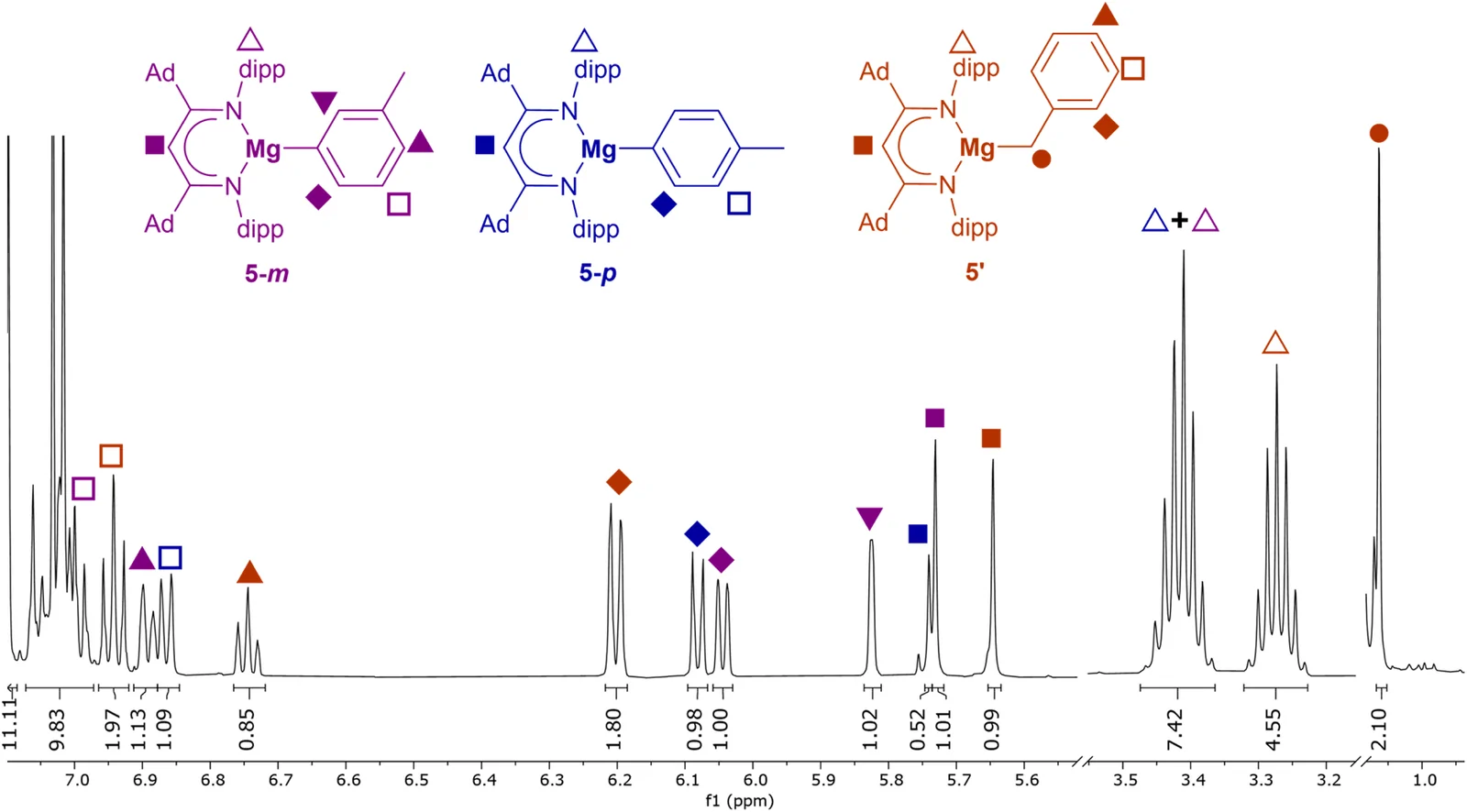
Selected regions of the ^1^H NMR spectrum (500 MHz, benzene-*d*_6_) showing the product mixture of 5-*m*, 5-*p*, and 5′ obtained from the reaction of 1^Ad^ with toluene after removal of volatiles and dissolution in benzene-*d*_6_.

**Scheme 5:**
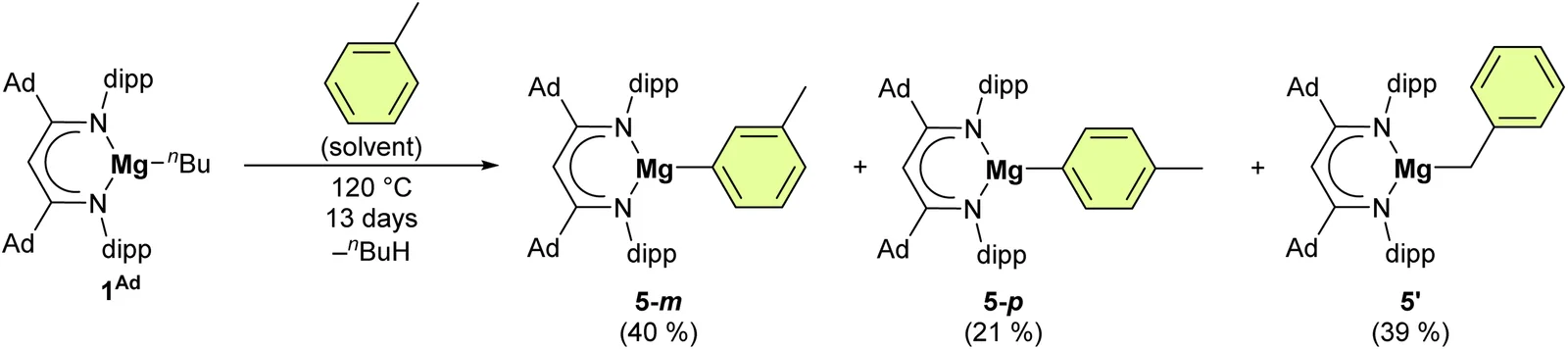
Reaction of 1^Ad^ with toluene. ^1^H NMR spectroscopic conversions are shown in parentheses. Compounds 5-*p*/5-*m*/5′ can be isolated in a combined 45% crystalline yield as a mixture of isomers.

The disordered co-crystalline mixture of isomers in the crystal structure of 5-*m*/5-*p* precludes detailed structural discussion (Fig. 5a), besides noting the planar three-coordinate magnesium centre, near-coplanarity of the tolyl ring and BDI ligand backbone, and unremarkable Mg–C distances (respectively 2.130(3), 2.1353(19) Å for 5-*m* and 5-*p*) compared to previously reported examples of three-coordinate BDI–magnesium aryl derivatives.^18,41^ The Mg–C distance in 5′ (Fig. 5b; 2.1160(19) Å) is significantly but expectedly shorter than that in [(tmeda)K-η^6^-C_6_H_5_CH_2_-Mg(N(SiMe_3_)_2_)] (2.187(2) Å).^46^ The Mg–C bond in 5′ is also contracted relative to the related 4-coordinate BDI–magnesium benzyl complex [(^Me^BDI^dipp^)Mg(CH_2_Ph)(thf)] (2.1325(18) Å).^47^

The DFT-calculated reaction pathway for toluene deprotonation by 1^Ad^ is consistent with the experimentally observed regioselectivity (Table 1). The Gibbs energies for the respective *ortho*-, *meta*-, *para*-, and benzyl C(sp^3^)-magnesiated products plus *n*-butane were calculated to be −4.2, −6.6, −7.0, and −7.3 kcal mol^−1^. In the encounter complex, C^Me^, which precedes C(sp^3^)–H deprotonation, the magnesium is already significantly displaced from the BDI ligand plane and, thus, significantly higher in energy (15.1 kcal mol^−1^) compared to C (4.0 kcal mol^−1^), which precedes C(sp^2^)–H deprotonation with the magnesium centre coplanar with the ligand. The geometries of both subsequent transition states, however, are similar, with magnesium centres projecting out of the planes of the BDI ligands whilst a proton is directly transferred from toluene to the *n*-butyl groups *via* a linear C–H–C arrangement. The transition states for C(sp^2^)–H deprotonation were located at 42.8 kcal mol^−1^ for the *ortho* position, and at 40.0, 39.9 kcal mol^−1^ for the *meta*- and *para*-positions, respectively. Relative to intermediate C^Me^, the transition state for C(sp^3^)–H deprotonation (TS(C^Me^-D^Me^)) is lower (24.8 kcal mol^−1^) than those for C(sp^2^)–H deprotonation. Due to the already distorted geometry of C^Me^ (15.1 kcal mol^−1^), however, the overall energy barrier to C(sp^3^)–H deprotonation *via*TS(C^Me^-D^Me^) of 39.9 kcal mol^−1^, is essentially identical to that for *meta*- or *para*-C(sp^2^)–H deprotonation. Thus, whilst *ortho*-magnesiation is kinetically disfavoured, there is no kinetic discrimination between deprotonation of the remaining C–H bonds, reflecting the essentially statistical mixture of isomers observed experimentally. The significantly higher energy barrier compared to benzene deprotonation is consistent with the slower reaction and higher temperature required.

**Table 1:** DFT-calculated Gibbs energies (BP86-D3^BJ^(SMD = toluene)/def2-TZVPP//BP86/def2-SVP level, in kcal mol^−1^) for the formation of 5-*o*/*m*/*p* or 5′ and ^*n*^BuH through the C(sp^2^)–H deprotonation of toluene by 1^Ad^; depictions of the calculated transition states for deprotonation of the methyl, *meta*- or *para*-C–H bonds of toluene

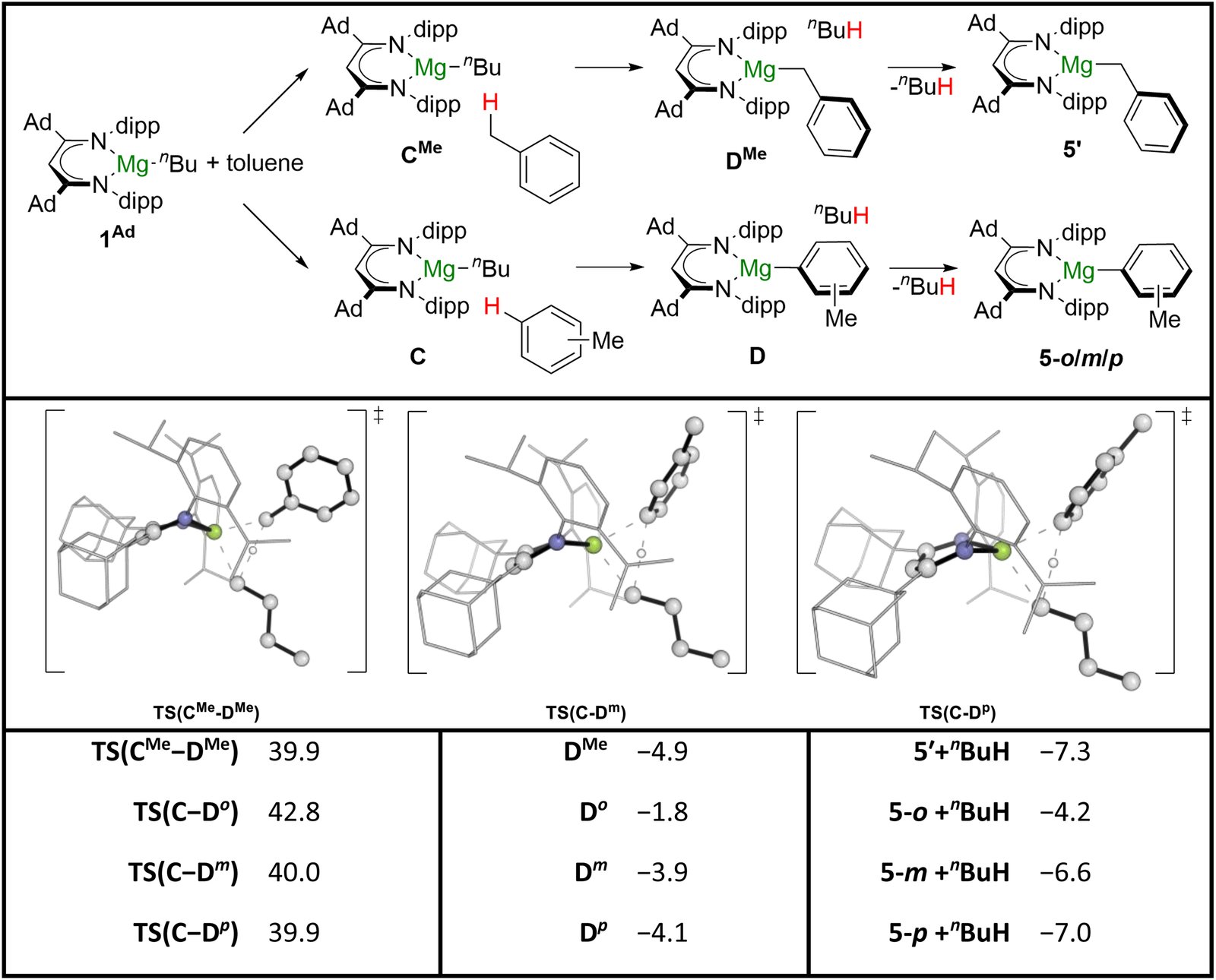

### Deprotonation and magnesiation of xylenes and 1,3-di-*tert*butylbenzene

Consistent with the regioselectivity observed for the reaction of 1^Ad^ with toluene, *ortho*-xylene is slowly deprotonated at 120 °C to provide a mixture of the *meta*-magnesiated xylyl derivative [(^Ad^BDI)Mg(C_6_H_3_-3,4-Me_2_)] (6) and *ortho*-methyl benzyl derivative [(^Ad^BDI)Mg(CH_2_C_6_H_4_-2-Me)] (6′). After 10 days at 120 °C, 6 and 6′ were determined to form in respective ^1^H NMR spectroscopic conversions of 62% and 38% according to analysis of the crude product mixture, which was isolated free of 1^Ad^ in 94% yield (Scheme 6). Single crystals suitable for X-ray diffraction analysis were obtained from the NMR tube of the reaction mixture at 100 °C and isolated in 14% yield. The resulting structure solution was refined as a disordered co-crystalline mixture of 6 and 6′ with the two respective components in a 0.8 : 0.2 occupancy ratio (Fig. 7a).

**Scheme 6:**
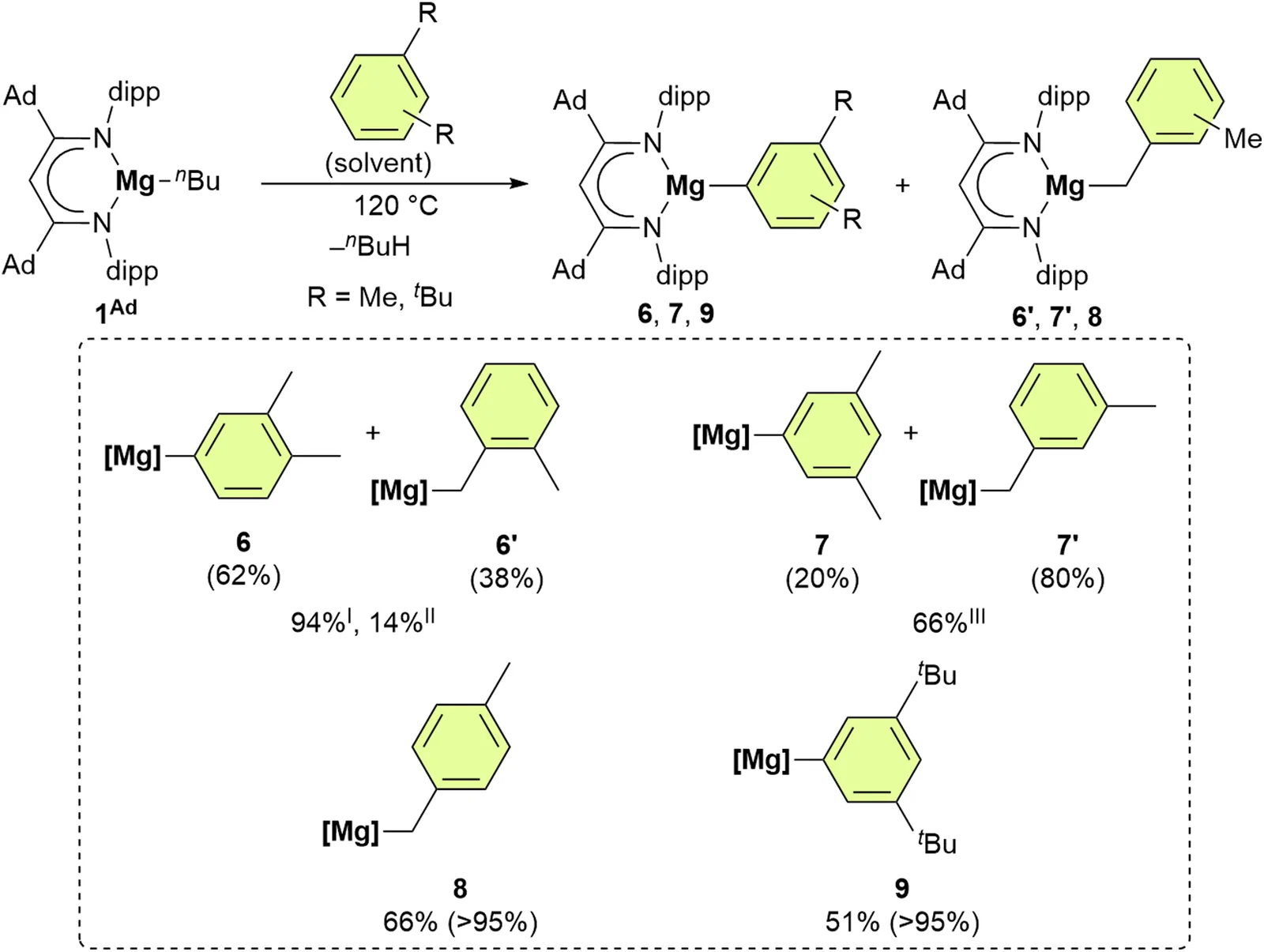
Reaction of 1^Ad^ with xylenes or 1,3-di-*tert*butylbenzene. Reaction duration was 10 days, except the reaction with 1,3-di-*tert*butylbenzene, which was stopped after 8 days. Yields refer to isolated product after crystallisation unless otherwise specified, with ^1^H NMR spectroscopic yields in parentheses. ^I^Isolated yield of non-crystalline product mixture. ^II^Isolated yield of crystalline product as 80 : 20 co-crystalline mixture of 6/6′ after reaction at 100 °C for 11 days. ^III^Isolated yield of crystalline product as 90 : 10 co-crystalline mixture of 7/7′.

**Fig. 7:**
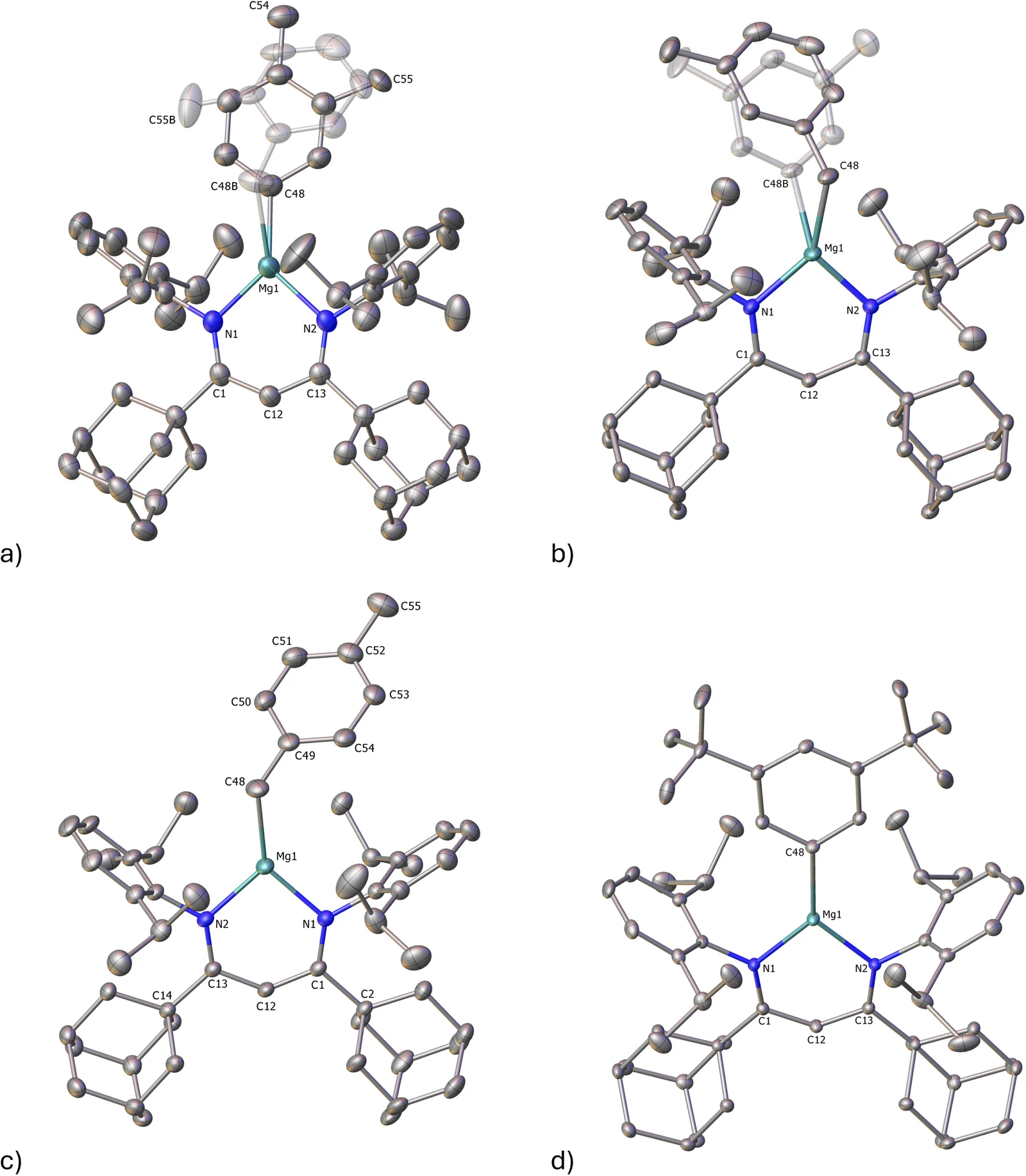
Displacement ellipsoid plots (30% level) of (a) the co-crystal of 6 (occupancy 0.8) and 6′ (occupancy 0.2, semi-transparent); (b) 7 (occupancy 0.1, semi-transparent) and 7′ (occupancy 0.9); (c) 8; (d) 9. For clarity, hydrogen atoms are omitted. The minor components of disordered magnesium-bound xylyl or benzyl moieties are shown semi-transparent, whilst for other disordered residues, the minor component is omitted. Note that in the major component (6) of the disordered co-crystal of 6/6′, C55 is also split across two positions with respect to rotation of the xylyl moiety about the Mg1–C48 bond; only the major component of this disordered feature is shown. Crystal structure of pure 7 (without component of 7′) is shown in the SI (Fig. S277). Distances (Å) and angles (°) for 8: Mg1–N1 2.0155(11), Mg1–N2 2.0126(12), Mg1–C48 2.1174(15), C48–C49 1.482(2), C52–C55 1.516(3), N1–Mg1–C48 137.01(7), N2–Mg1–N1 94.68(4), N2–Mg1–C48 127.70(6), C49–C48–Mg1 120.10(11). Distances (Å) and angles (°) for 9: Mg1–N1 2.0184(14), Mg1–N2 2.0121(14), Mg1–C48 2.1027(16), N1–Mg1–C48 133.26(6), N2–Mg1–N1 94.86(6), N2–Mg1–C48 131.80(7), C49–C48–Mg1 119.19(12), C49–C48–C53 115.76(14), C53–C48–Mg1 124.95. Distances and angles for 6/6′ and 7/7′ are not reported here due to disorder.

For *meta*-xylene, the benzylic C(sp^3^)–H bonds are less hindered, and formation of the *meta*-methyl benzyl derivative [(^Ad^BDI)Mg(CH_2_-C_6_H_4_-3-CH_3_)] (7′) is relatively favoured compared to the *meta*-magnesiated xylyl product [(^Ad^BDI)Mg(C_6_H_3_-3,5-Me_2_)] (7). After 10 days at 120 °C and removal of volatiles under vacuum, analysis of the crude reaction mixture by ^1^H NMR spectroscopy indicated >95% conversion of 1^Ad^ to 7 and 7′ in respective spectroscopic yields of 20% and 80% (Scheme 6). Pale-yellow block-shaped single crystals were obtained after recrystallisation from *n*-hexane and isolated in 66% yield. The results of a subsequent X-ray diffraction experiment provided a structure solution that was refined as a disordered co-crystal of 7′ and 7 in two components of relative occupancy 0.9 : 0.1 (Fig. 7b), whilst a few colourless plate-shaped single crystals of pure 7 suitable for X-ray diffraction analysis were obtained from the walls of the J. Young's NMR tube above the meniscus of the reaction mixture at 100 °C (See SI, Fig. S277).

The C(sp^2^)–H bonds of *para*-xylene are kinetically inaccessible under similar conditions, and heating 1^Ad^ at 120 °C in this solvent for 10 days exclusively provided the *para*-methyl benzyl derivative [(^Ad^BDI)Mg(CH_2_-C_6_H_4_-4-CH_3_)] (8) in >95% spectroscopic conversion according to ^1^H NMR spectroscopy (Scheme 6). Pale-yellow rod-shaped single crystals of 8 suitable for X-ray diffraction analysis were obtained in 66% yield after recrystallisation from *n*-hexane/toluene (Fig. 7c).

Finally, 1,3-di-*tert*butylbenzene – which lacks acidic C(sp^3^)–H bonds – yields the 3,5-di-*tert*-butyl phenyl complex [(^Ad^BDI)Mg(C_6_H_3_-3,5-^*t*^Bu_2_)] (9) as the sole product after 8 days at 120 °C (Scheme 6). Compound 9 was isolated in 51% yield as colourless block-shaped crystals suitable for X-ray diffraction analysis (Fig. 7d), which deposited directly from the reaction mixture.

The X-ray crystal structures of compounds 6–9 are unremarkable besides providing structural verification of the reaction products (Fig. 7). Similarly to compounds 3^R^ and 5-*m*/5-*p*, however, in each of the C(sp^2^)–H activated products, the benzene ring is approximately co-planar with the BDI ligand backbone. Although detailed discussion of distances and angles are limited by disorder in most cases, the Mg–C distances of the aryl derivatives (6 = 2.049(4) Å; 7 = 2.1016(18) Å; 9 = 2.1027(16) Å) fall within a relatively narrow range and are similar to previously reported examples of three-coordinate BDI-magnesium aryl derivatives,^18,41^ albeit significantly shorter than 5-*m*/5-*p* (2.130(3), 2.1353(19) Å). The Mg–C distances in the benzyl derivatives are also mutually similar (5′ = 2.1160(19); 7′ = 2.1223(16); 8 = 2.1174(15) Å).

### Deprotonation and magnesiation of anisole

In a further utilisation of the magnesium *n*-butyl moiety as a strong base, anisole solutions of compounds 1^Ad^ and [1^Me^]_2_ were heated at 100 °C. The methoxy group exerts a directing and kinetically activating effect, such that the *ortho*-magnesiated anisyl derivatives 10^Ad^ and 10^Me^ were selectively obtained after 7 days or 16 hours, respectively (Scheme 7). Recrystallisation from *n*-hexane/benzene or *n*-hexane/THF provided the respective anisole- and THF-adducted derivatives [(^Ad^BDI)Mg(κ^1^*C*-C_6_H_4_-2-OCH_3_)(κ^1^*O*-O(CH_3_)C_6_H_5_)] (10^Ad^·anisole) and [(^Me^BDI)Mg(κ^2^*C*,*O*-C_6_H_4_-2-OMe)(thf)] (10^Me^·thf). This mode of reactivity contrasts with that of hydridocalcium derivative [2^Ca^]_2_, which reacts with anisole *via* nucleophilic substitution of the CH_3_ group, eliminating methane and forming a calcium phenoxide complex.^48^ The related cationic aza-macrocycle-supported calcium hydride [(Me_4_TACD)Ca(µ-H)_2_Ca(thf)(Me_4_TACD)]^2+^ (Me_4_TACD = *N*,*N*′,*N*″,*N*‴-1,4,7,10-tetraazacyclododecane) does, however, mediate the directed *ortho*-metallation of anisole and 1,3-di(methoxy)benzene.^49^ The X-ray crystal structure of 10^Ad^·anisole (Fig. 8b) contains a distorted tetrahedral four-coordinate (*τ*_4_ = 0.826)^50^ magnesium centre coordinated by the chelating BDI, κ^1^-*C*-bound anisyl, and neutral *O*-bound anisole ligands. By contrast, the anisyl ligand in the less bulky derivative 10^Me^·thf chelates magnesium in a κ^2^-*C*,*O* fashion (Fig. 8a), such that the magnesium centre is five-coordinate, adopting a square-pyramidal geometry (*τ*_5_ = 0.156; value calculated for the major component of the disordered anisyl moiety)^51^ with THF bound in the apical site and a shorter Mg–C distance (2.137(3) Å for the major component of 10^Me^·thf; 2.178(5) Å for 10^Ad^·anisole).

**Scheme 7:**
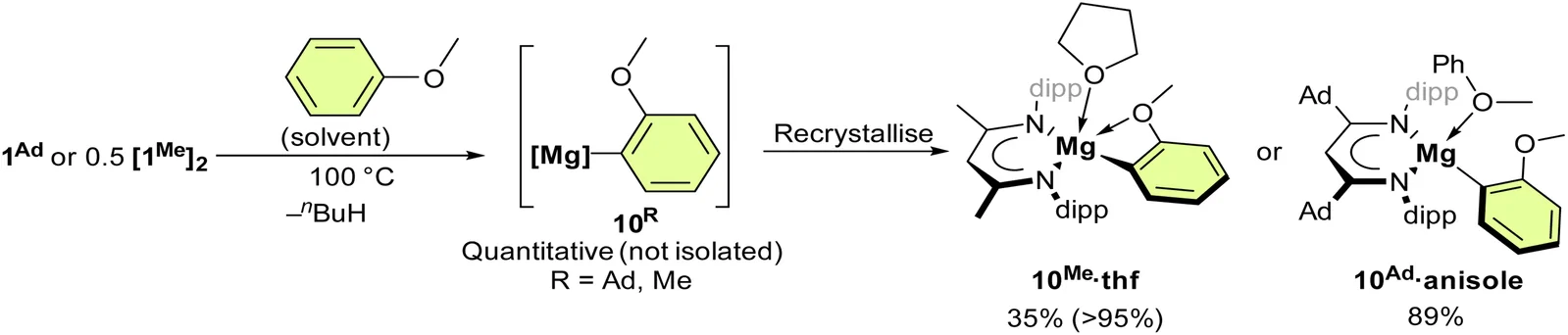
Reaction of compounds 1^Ad^ and [1^Me^]_2_ with anisole. Isolated yields are shown with ^1^H NMR spectroscopic yields in parentheses.

**Fig. 8:**
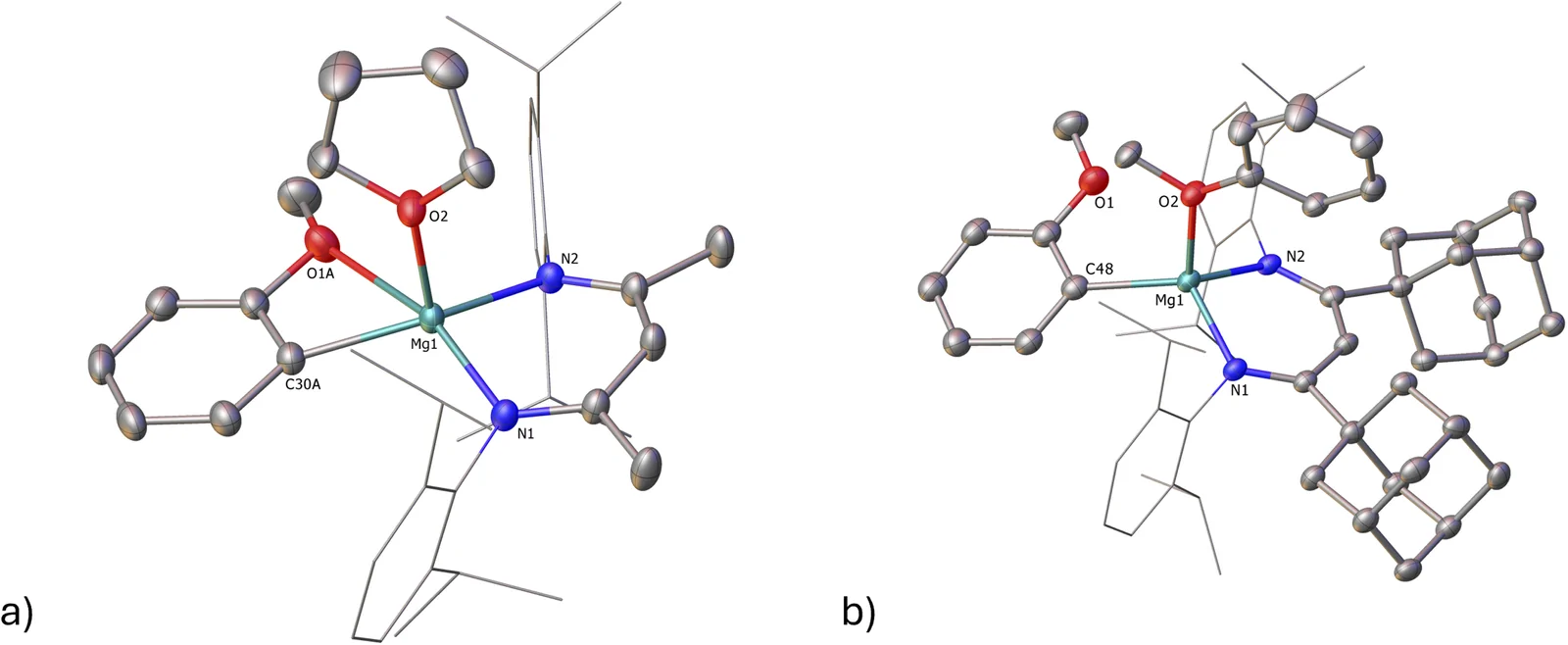
Displacement ellipsoid plots (30% level) of the crystal structures of 10^Me^·thf (left) and 10^Ad^·anisole (right). Only the major component of the disordered anisyl moiety in 10^Me^·thf is shown, dipp groups are shown as wireframes, and hydrogen atoms are omitted for clarity. Distances (Å) and angles (°) for 10^Me^·thf: Mg1–O2 2.0542(14), Mg1–N1 2.0740(15), Mg1–N2 2.0740(14), Mg1–O1A 2.729, Mg1–C30A 2.137(3), Mg1–O1B 2.461(10), Mg1–C30B 2.179(13), O2–Mg1–N1 104.03(6), O2–Mg1–N2 102.66(6), O2–Mg1–O1A 94.34(7), O2–Mg1–C30A 101.28(8), O2–Mg1–O1B 94.9(2), O2–Mg1–C30B 98.7(3), N1–Mg1–O1A 160.38(7), N1–Mg1–C30A 113.36(12), N1–Mg1–O1B 95.1(2), N1–Mg1–C30B 146.3(4), N2–Mg1–N1 92.48(6), N2–Mg1–O1A 90.13(7), N2–Mg1–C30A 138.89(10), N2–Mg1–O1B 158.5(2), N2–Mg1–C30B 106.5(4), C30A–Mg1–O1A 55.07(12), C30B–Mg1–O1B 58.1(5), C31A–O1A–Mg1 83.10(19), C31A–O1A–C36A 117.5(3), C36A–O1A–Mg1 158.16(19), C31A–C30A–Mg1 109.9(2), C31B–C30B–Mg1 101.5(10), C35B–C30B–Mg1 142.3(13). Distances (Å) and angles (°) for 10^Ad^·anisole: Mg1–O2 2.208(3), Mg1–N1 2.070(3), Mg1–N2 2.106(3), Mg1–C48 2.178(5), N1–Mg1–O2 107.85(13), N1–Mg1–N2 95.58(13), N1–Mg1–C48 115.89(16), N2–Mg1–O2 108.37(13), N2–Mg1–C48 127.29(15), C48–Mg1–O2 100.99(14).

### Magnesium-mediated dehydroborylation of benzene

Compound 3^Ad^ was utilised as a nucleophilic phenyl-transfer reagent in a one-pot procedure, starting from 1^Ad^ and benzene (Scheme 8). After heating at 100 °C for 1 week, removal of volatiles under vacuum, and redissolution in benzene-*d*_6_, the as-obtained solution of 3^Ad^ was added to MeOBpin and a bright yellow solution was immediately obtained. Analysis by ^1^H and ^11^B NMR spectroscopies confirmed successful phenyl transfer to generate the commonly employed Suzuki cross-coupling precursor, PhBpin, by comparison to the NMR data of a commercial sample. The fate of the magnesium depends on the number of equivalents of MeOBpin employed. A strict 1 : 1 molar ratio of 3^Ad^ and MeOBpin generates the dimeric methoxide [(κ^2-Ad^BDI)Mg(µ-OMe)_2_Mg(κ^1-Ad^BDI)] (11), which was isolated as colourless plate-shaped crystals after recrystallisation from *n*-hexane/benzene and separately synthesised and characterised after a similar reaction of 1^Ad^ with MeOBpin (Fig. 9a). Under conditions of excess MeOBpin, the bright yellow boronate derivative [(^Ad^BDI)Mg(µ-OMe)_2_(Bpin)] (12) was preferentially formed. Compound 12 was also separately synthesised from 1^Ad^ and MeOBpin and crystallographically characterised after recrystallisation from *n*-hexane/toluene (Fig. 9b).

**Scheme 8:**
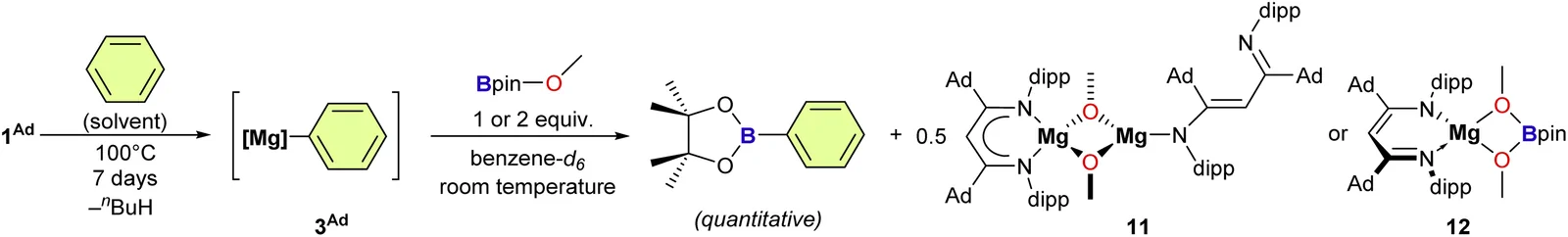
One-pot, two-step dehydroborylation of benzene to yield PhBpin (quantitative spectroscopic yield by ^1^H NMR) and 11 and/or 12. Compounds 11 and 12 can be isolated in respective 55% and 54% crystalline yield from the direct reaction of 1 with 1 or >2 equiv. MeOBpin.

**Fig. 9:**
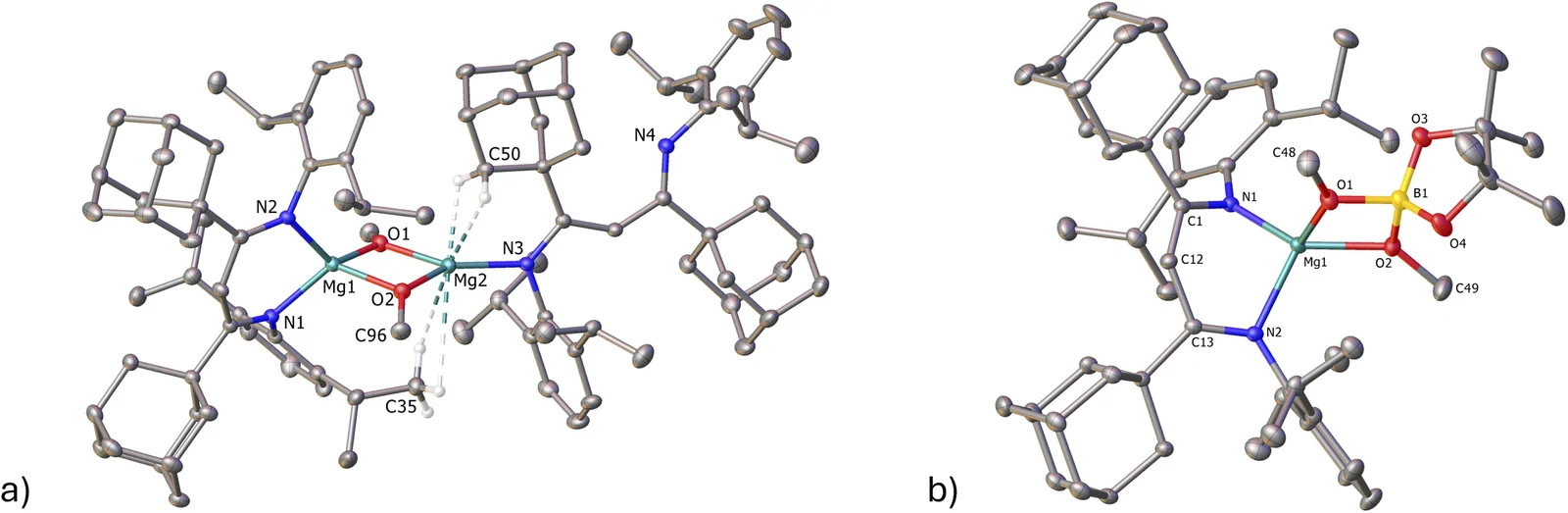
Displacement ellipsoid plots (30%) of the crystal structures of (a) 11 and (b) 12. Hydrogen atoms are omitted for clarity, except for those bound to C35 and C50 in compound 11. Solvent molecules present in the crystal structure of 11 are also omitted for clarity. Distances (Å) and angles (°) for 11: Mg1–O1 1.9571(14), Mg1–O2 1.9762(15), Mg1–N1 2.0253(16), Mg1–N2 2.0602(15), Mg2–O1 1.9430(15), Mg2–O2 1.9243(14), Mg2–N3 2.0069(16), Mg2–H50B 2.2195(8), Mg2–H50A 2.0937(8), Mg2–H35C 2.4624(8), N1–C1 1.343(2), N2–C13 1.330(2), N3–C48 1.380(2), N4–C60 1.293(2), C1–C12 1.405(2), C12–C13 1.427(2), C59–C60 1.451(3), C59–C48 1.386(2), O1–Mg1–O2 83.03(6), O1–Mg1–N1 113.11(6), O1–Mg1–N2 131.94(6), O2–Mg1–N1 121.05(6) O2–Mg1–N2 112.88(6), N1–Mg1–N2 96.36(6), O1–Mg2–N3 132.07(6), O2–Mg2–O1 84.77(6), O2–Mg2–N3 142.95(7), Mg2–O1–Mg1 95.96(6), Mg2–O2–Mg1 945.94(6). Distances (Å) and angles (°) for 12: Mg1–O1 1.9723(9), Mg1–O2 1.9590(9), Mg1–N1 2.0100(10), Mg1–N2 2.0680(10), O1–B1 1.523(16), O2–B1 1.5318(16), O3–B1 1.4304(17), O4–B1 1.4220(17), N1–C1 1.3720(15), N2–C13 1.3060(15), C1–C12 1.3856(16), C12–C13 1.4576(15), O1–Mg1–N1 119.16(4), O1–Mg1–N2 120.53(4), O2–Mg1–O1 69.05(4), O2–Mg1–N1 137.50(4), O2–Mg1–N2 115.06(4), N1–Mg1–N2 96.61(40), O1–B1–O2 94.10(9), O3–B1–O1 110.71(11), O3–B1–O2 115.28(11), O4–B1–O1 116.68(11), O4–B1–O2 112.07(11), O4–B1–O3 107.80(10).

The crystal structures of compounds 11 and 12 merit brief discussion, as they illustrate the steric limits of the extremely bulky ^Ad^BDI ligand (Fig. 9). In each case, the strongly oxophilic magnesium centres bind to two µ-OMe groups. In compound 11, this results in a dimeric structure which is unable to accommodate two chelating ^Ad^BDI ligands. This causes one ^Ad^BDIMg chelate to ring-open, such that Mg2 is three-coordinate trigonal planar (*Σ*_angles_(Mg) = 359.79°) and bound *via* N3 (Mg2–N3 = 2.0069(16) Å) to an ^Ad^BDI unit as an open-chain enamido-imine with localised C59–C48 (1.386(2) Å) and C60–N4 (1.293(2) Å) double bonds (*cf.* C59–C60, C48–N3 single bonds = 1.541(3), 1.380(2) Å), whilst Mg1 is four-coordinate and chelated by a typical κ^2^-β-diketiminate with delocalised π bonding (N1–C1, N2–C13, C1–C12, C12–C13 = 1.343(2), 1.330(2), 1.405(2), 1.427(2) Å). The coordination sphere of the planar three-coordinate Mg2 centre appears to be further augmented by close contacts with the C50-bound hydrogen atoms of the nearby adamantyl group (Mg2–H50B, 2.2195(8); Mg2–H50A, 2.0937(8) Å), and the C35-bound hydrogen atoms belonging to an isopropyl group of the Mg1-bound ^Ad^BDI ligand (Mg2–H35C = 2.4624(8) Å). This structure is retained in benzene-*d*_6_ solution, such that the backbone methine resonances of the two dissimilar ^Ad^BDI ligands are observed in the ^1^H NMR spectrum as singlets of equal (1H) relative intensity at *δ* 5.79 (κ^2-Ad^BDI), 4.41 (κ^1-Ad^BDI) ppm. Further, the κ^1^-bound ^Ad^BDI ligand displays two inequivalent dipp and adamantyl environments in the ^1^H and ^13^C NMR spectra, whereas individual chemically equivalent dipp and adamantyl environments are observed for the κ^2^-bound ^Ad^BDI ligand. A similar closed-open combination of two BDI ligands has been observed for the magnesium(i) dimer supported by an extremely bulky ^tBu^BDI^dipep^ ligand.^37^

Although the ^Ad^BDI ligand of compound 12 appears to be conventionally coordinated to the magnesium centre in a κ^2^-fashion, closer inspection reveals a significant twisting of the normally planar BDI ligand backbone, as shown by the N1–C1–C13–N2 torsion angle of 25.68(9)°. Further, the N2–C13 (1.3060(15) Å) and C1–C12 (1.3856(16) Å) distances are significantly shorter than the N1–C1 (1.3720(15) Å) and C12–C13 distances 1.4576(15) Å), indicating localised N=C and C=C double bonds and a β-enamidoimine form rather than a delocalised β-diketiminate (Fig. 9b). Additionally, the magnesium centre is significantly projected above the mean N1–C12–N2 plane by 1.1785(11) Å to accommodate the tetra-alkoxyboronate moiety. Hence, the extremely congested coordination environment enforces a loss of π-delocalisation, and a twisted ligand conformation. This situation can be viewed as an intermediate *en route* towards the chain-opened form observed in 11.

### Utilisation of magnesium phenyl and anisyl complexes in palladium-catalysed Kumada coupling

We have previously reported that the dimeric calcium phenyl complex [(^Me^BDI)Ca(µ,η^1^:η^1^-Ph)]_2_ reacts directly with bromobenzene to provide biphenyl and [((^Me^BDI)Ca)_2_(µ,η^1^:η^6^-Ph)(µ-Br)] (Scheme 2c).^32^ By contrast, 1^Ad^ does not react directly with bromobenzene, and application of elevated temperature solely results in phenyl-exchange with the benzene-*d*_6_ solvent. We^35^ and Crimmin and co-workers^52^ have previously demonstrated that a telescoped palladium-catalysed aryl–aryl coupling may be achieved *via* BDI–zinc aryl complexes. Compound 3^Ad^ was, thus, similarly generated *in situ* by reaction of 1^Ad^ with benzene, followed by removal of volatiles, redissolution in benzene-*d*_6_ and addition of bromobenzene (1 equiv.) and catalytic [Pd(PCy_3_)_2_] (4–5 mol%). After 3 days at 60 °C, 93% spectroscopic yield of biphenyl was confirmed by *in situ*^1^H NMR spectroscopy and the product additionally identified by GC-MS (Scheme 9). The magnesium bromide by-product, [(^Ad^BDI)MgBr] (13), crystallised directly from the reaction mixture and was fully characterised (see SI). Although similar monomeric 3-coordinate iodide species have been reported,^37,42,53^ compound 13 represents the first example of a monomeric three-coordinate BDI magnesium bromide complex. The one-pot telescoped protocol was extended towards the cross-coupling of 3^Ad^ with 2-ethyl bromobenzene, as well as 4-methoxy bromobenzene, which respectively provided the cross-coupled biphenyl derivatives, 2-ethyl-1-biphenyl and 4-methoxy-1-biphenyl, in quantitative ^1^H NMR spectroscopic yields after 2 days at 60 °C (Scheme 9). Further, 3^Ad^-*d*_5_ was employed to generate the deuterated biphenyl derivate 4-MeO-C_6_H_4_-1-C_6_D_5_ in quantitative yield *via* a telescoped one-pot procedure starting from 1^Ad^ and benzene-*d*_6_. Reacting [1^Me^]_2_ with anisole followed by removal of volatiles and palladium-catalysed cross-coupling of the as-formed 10^Me^ with bromobenzene yielded 2-methoxy-1-biphenyl in quantitative ^1^H NMR spectroscopic yield after 24 hours at 60 °C, with elimination of the known dimeric magnesium bromide complex [(^Me^BDI)Mg(µ-Br)]_2_ (Scheme 9).^41^ Whilst palladium-catalysed Kumda coupling is well established using Grignard reagents, without needing a ‘specialised’ ligand such as ^Ad^BDI, the ability to access magnesium aryl reagents *via* direct C–H activation may enable the development of new synthetic approaches.

**Scheme 9:**
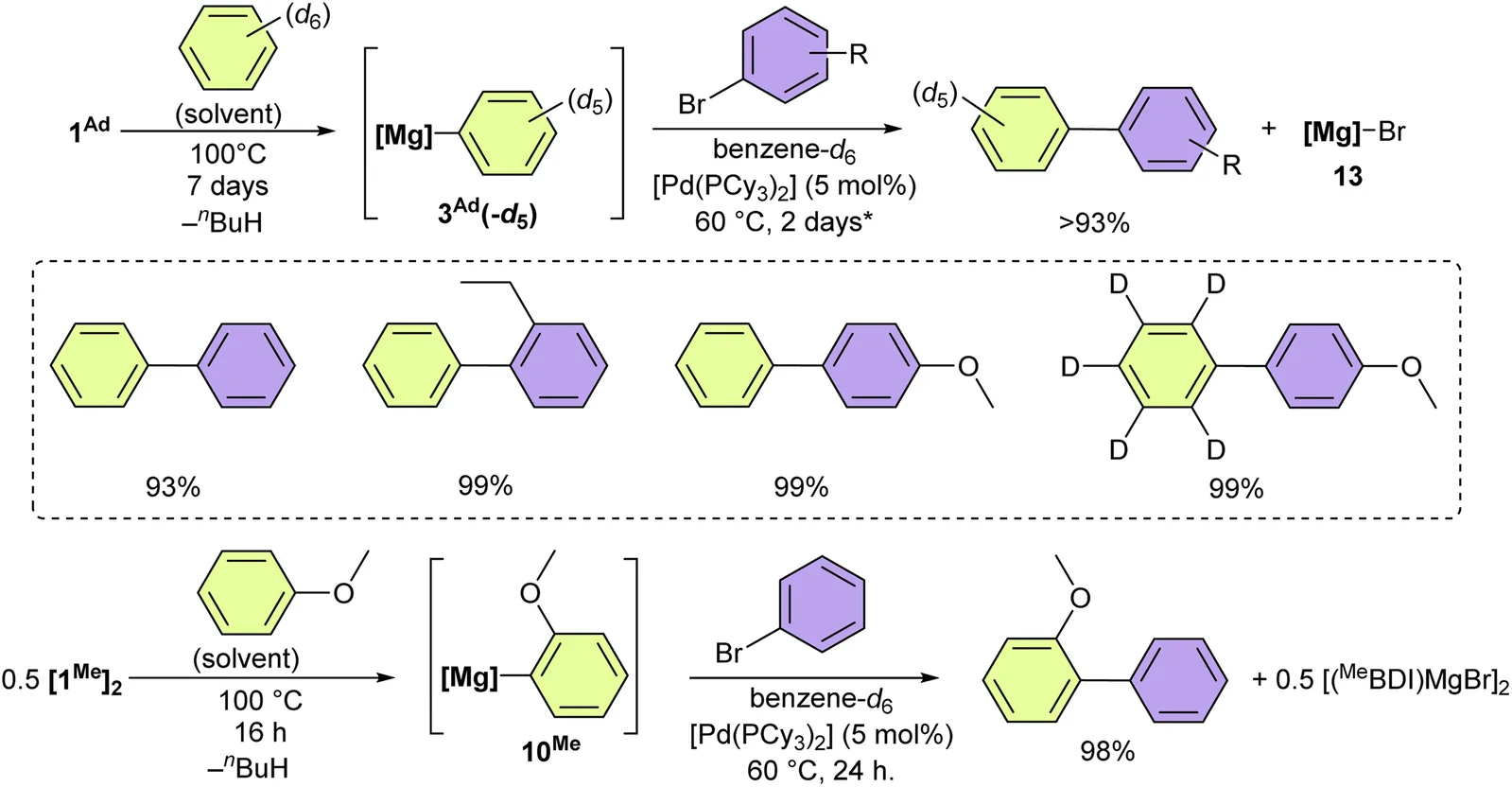
One-pot, two-step palladium-catalysed Kumada cross-coupling of benzene with aryl bromides *via*3^Ad^ (top) to give 13 and biphenyl products (middle); one-pot, two-step palladium-catalysed Kumada cross-coupling of anisole with bromobenzene *via*10^Me^ to give 2-methoxybiphenyl and [(^Me^BDI)Mg(µ-Br)]_2_ (bottom).*Three-days reaction time for the reaction with bromobenzene. Yields given were determined by integration of the *in situ*^1^H NMR spectrum against hexamethylbenzene as an internal standard.

## Conclusions

Although thermodynamically consistent with the p*K*_a_s of the relevant C–H bonds, the C(sp^2^) and C(sp^3^) deprotonation of unactivated arenes by alkyl magnesium reagents is kinetically challenging. The magnesium *n*-butyl complex 1^Ad^ with the bulky ^Ad^BDI ligand acts as a robust and sterically hindered base, enabling the deprotonation of benzene, toluene, xylenes, and 1,3-di-*tert*butylbenzene under forcing conditions (100–120 °C) without competing side reactions such as ligand degradation or β-hydride elimination. The reaction of dimeric *n*-butyl complex [1^Me^]_2_ – which is supported by the less sterically demanding ^Me^BDI ligand – with benzene is not only much slower than that of 1^Ad^ but also suffers from β-hydride elimination of 1-butene as a side reaction. Although the reason for the greater kinetic facility of 1^Ad^ for arene deprotonation compared to [1^Me^]_2_ remains to be resolved, its resistance to competing β-hydride elimination may be attributed to lower stability of the hypothetical µ-hydride dimer, [(^Ad^BDI)Mg(µ-H)]_2_ ([2^Mg,Ad^]_2_). Whilst the steric demand of the ^Ad^BDI ligand enables selective deprotonation of benzene in the presence of the substantially more acidic, yet hindered, 1,2,3,4-tetramethylcyclopentadiene, and leaves *ortho*-C(sp^2^)–H bonds in the studied alkyl benzenes intact, little kinetic discrimination is observed between the various sterically accessible C(sp^2^)–H and C(sp^3^)–H bonds of toluene, *ortho*- and *meta*-xylene. By contrast, both 1^Ad^ and [1^Me^]_2_ readily mediate the directed *ortho*-magnesiation of anisole. The anisyl and phenyl complexes were harnessed in a telescoped “one-pot” Kumada biaryl cross-coupling procedure, utilising the reaction solvent as the aryl source.

Compared to the facile alkylation of benzene by dimeric β-diketiminato calcium-, ytterbium-, and strontium *n*-alkyl complexes, the C–H deprotonation chemistry of 1^Ad^ is striking. These contrasting observations are, however, consistent with lower nucleophilicity of the Mg–C bond compared to the heavier congeners. Being smaller, harder (less polarisable), and less electropositive, magnesium is additionally less able to interact strongly with the π-electron density of aromatic rings. Such interactions are a regular feature of heavier alkaline earth organometallics and have been suggested to be crucial in the nucleophilic alkylation chemistry of these elements. The ability to directly derive aryl magnesium reagents from the parent hydrocarbon in cross-coupling chemistry potentially opens new possibilities in C–H functionalisation protocols, and future efforts to develop soluble and kinetically discriminating alkaline-earth reagents of exceptional basicity may be fruitful in this regard.

## Author contributions

L. J. M.: conceptualisation, investigation, formal analysis, methodology, data curation, visualisation, writing – original draft, writing – review and editing. A. M.: investigation, formal analysis, methodology, data curation, visualisation, writing – review and editing. A. S. S. W.: conceptualisation, investigation. M. S. H.: funding acquisition, project administration, resources, writing – review and editing. C. L. M.: funding acquisition, project administration, resources, writing – review and editing.

## Conflicts of interest

There are no conflicts to declare.

## Supplementary Material

SC-OLF-D6SC04717C-s001

SC-OLF-D6SC04717C-s002

## Data Availability

CCDC 2557915–2557930 contain the supplementary crystallographic data for this paper.^54*a*–*p*^ Other data supporting this manuscript can be found in the supplementary information (SI). Supplementary information: experimental details, NMR spectra, chromatograms, mass spectra, details of steric mapping analysis, crystallographic details, and computational details, and Cartesian coordinates of the DFT optimised structures. See DOI: https://doi.org/10.1039/d6sc04717c.
