# Supplementary material for: C–H magnesiation of unactivated arenes by a bulky β-diketiminato magnesium n-butyl complex
Source: Chem Sci. 2026 Sep 24. Online ahead of print. doi: 10.1039/d6sc04717c (PMC13635820; doi:10.1039/d6sc04717c)
Supplement: Supplementary file 1 [file SC-OLF-D6SC04717C-s001.pdf]

Supplementary Information (SI) for Chemical Science.
This journal is © The Royal Society of Chemistry 2026

C–H Magnesiation of Unactivated Arenes by a Bulky β-Diketiminato Magnesium *n*-Butyl Complex

Louis J. Morris,*[,a†] Agustín Morales,[a] Andrew S. S. Wilson,[a] Michael S. Hill,*[,a] Claire L. McMullin*[,a]

[a]Department of Chemistry, University of Bath, Claverton Down, Bath, BA2 7AY, UK; msh27@bath.ac.uk, cm2025@bath.ac.uk

[†]Current address: Department of Chemistry, Durham University, Stockton Road, Durham, DH1 3LE, UK; louis.morris@durham.ac.uk

Supporting information

Contents

1 General experimental considerations 3

2 Experimental procedures and characterisation 3

2.1 Synthesis and characterisation of ^{Ad}BDIH 3

2.2 Synthesis of [(^{Ad}BDI)Mg^{n}Bu] (**1^{Ad}**) 6

2.3 Synthesis of [(^{Me}BDI)Mg(Cp^{4M})] (**4**) 9

2.4 Attempted reaction of [(^{Ad}BDI)Mg^{n}Bu] (**1^{Ad}**) with Cp^{4M}H in benzene-*d*$_6$, synthesis and characterisation of [(^{Ad}BDI)Mg(C_6D_5)] (**3^{Ad}-*d*$_5$**) 12

2.5 Attempted reaction of [(^{Ad}BDI)Mg^{n}Bu] (**1^{Ad}**) with Cp^{4M}H in benzene, synthesis and characterisation of [(^{Ad}BDI)MgPh] (**3^{Ad}**) 14

2.6 Reaction of [(^{Ad}BDI)Mg^{n}Bu] (**1^{Ad}**) with a 1:1 mixture of C_6D_6 and C_6H_6 19

2.7 Exchange reaction of [(^{Ad}BDI)MgPh] (**3^{Ad}**) with benzene-*d*$_6$ to form [(^{Ad}BDI)Mg(C_6D_5)] (**3^{Ad}-*d*$_5$**) 20

2.8 Reaction of [(^{Ad}BDI)Mg^{n}Bu] (**1^{Ad}**) with $PhSiH_3$ 23

2.9 Reaction of [(^{Ad}BDI)Mg^{n}Bu] (**1^{Ad}**) with H_2 28

2.10 Reaction of [(^{Me}BDI)Mg^{n}Bu]$_2$ (**[1^{Me}]$_2$**) and [(^{Me}BDI)Mg(μ-H)]$_2$ (**[2^{Mg,Me}]$_2$**) with benzene-*d*$_6$ 30

2.11 Reaction of [(^{Ad}BDI)Mg^{n}Bu] (**1^{Ad}**) with toluene, synthesis and characterisation of [(^{Ad}BDI)Mg(C_6H_4Me)] (**5-*m*/5-*p***) 35

2.12 Synthesis of [(^{Ad}BDI)Mg($CH_2C_6H_5$)] (**5'**) 54

2.13 Reaction of [(^{Ad}BDI)Mg^{n}Bu] (1^{Ad}) with *ortho*-xylene, synthesis and characterisation of [(^{Ad}BDI)Mg(C_6H_3-3,4-Me$_2$)] (**6**) and [(^{Ad}BDI)Mg($CH_2C_6H_4$-2-Me)] (**6'**) 61

2.14 Reaction of [(^{Ad}BDI)Mg^{n}Bu] (1^{Ad}) with *meta*-xylene, synthesis and characterisation of [(^{Ad}BDI)Mg(C_6H_3-3,5-Me$_2$)] (**7**) and [(^{Ad}BDI)Mg($CH_2C_6H_4$-3-Me)] (**7'**) 71

2.15 Reaction of [(^{Ad}BDI)Mg^{n}Bu] (**1^{Ad}**) with *para*-xylene, synthesis and characterisation of [(^{Ad}BDI)Mg($CH_2C_6H_4$-4-Me)] (**8**) 80

2.16 Reaction of [(^{Ad}BDI)Mg^{n}Bu] (**1^{Ad}**) with 1,3-di(tert-butyl)benzene, synthesis and characterisation of [(^{Ad}BDI)Mg(C_6H_3-3,5-^{t}Bu$_2$)] (**9**) 83

2.17 Reaction of [(^{Me}BDI)Mg^{n}Bu]$_2$ **[1^{Me}]$_2$** with anisole, synthesis and characterisation of [(^{Me}BDI)Mg(κ^2*C,O*-C_6H_4-2-OMe)(thf)] **10^{Me}·thf** 90

2.18 Reaction of [(^{Ad}BDI)Mg^{n}Bu] (**1^{Ad}**) with anisole, synthesis and characterisation of [(^{Ad}BDI)Mg(C_6H_4-2-OMe)($MeOC_6H_5$)] (**10^{Ad}·anisole**) 94

2.19 Reaction of [(^{Ad}BDI)MgPh] (**3^{Ad}**) with 1.2 equiv. MeOBpin to form [(^{Ad}BDI)Mg(μ-OMe)$_2$Mg(κ^1-^{Ad}BDI)] (**11**) and [(^{Ad}BDI)Mg(μ-OMe)$_2$(Bpin)] (**12**) and crystallisation of **11** 98

2.20 Reaction of [(^{Ad}BDI)MgPh] (**3^{Ad}**) with 2 equiv. MeOBpin to form [(^{Ad}BDI)Mg(μ-OMe)$_2$(Bpin)] (**12**) 104

2.21 Synthesis and characterisation of [(^{Ad}BDI)Mg(μ-OMe)$_2$Mg(κ^1-^{Ad}BDI)] (**11**)................ 108

2.22 Synthesis and characterisation of [(^{Ad}BDI)Mg(μ-OMe)$_2$Bpin] (**12**) 114

2.23 Attempted uncatalyzed reaction of [(^{Ad}BDI)MgPh] (**3^{Ad}**) with bromobenzene 118

2.24 Procedure for telescoped Kumada coupling 120

2.24.1 General procedure for cross-coupling via [(^{Ad}BDI)MgPh] (**3^{Ad}**) 120

2.24.2 Cross-coupling of benzene and bromobenzene to form biphenyl.................. 120

2.24.3 Cross-coupling of benzene and 4-bromoanisole to form 4-methoxybiphenyl. 124

2.24.4 Cross-coupling of benzene-d$_6$ and 4-bromoanisole to form 4-methoxybiphenyl-d$_5$ 130

2.24.5 Cross-coupling of benzene and 2-ethylbromobenzene to form 2-ethylbiphenyl... 136

2.24.6 Cross-coupling of anisole and bromobenzene to form 2-methoxybiphenyl.... 140

2.25 Synthesis and characterisation of [(^{Ad}BDI)MgBr] (**13**) 146

3 Steric mapping 149

4 Kinetic isotope effect experiments.. 162

5 X-ray diffraction 164

5.1 Crystal structure determination of ^{Ad}BDIH 169

5.2 Crystal structure determination of [(^{Ad}BDI)Mg^{n}Bu] (**1^{Ad}**).. 169

5.3 Crystal structure determination of [(^{Ad}BDI)MgPh] (**3^{Ad}**) 170

5.4 Crystal structure determination of [(^{Me}BDI)MgCp^{4M}] (**4**) 171

5.5 Crystal structure determination of [(^{Ad}BDI)Mg(C$_6$H$_4$Me)] (**5-*m*, 5-*p***) 172

5.6 Crystal structure determination of [(^{Ad}BDI)Mg(CH$_2$Ph)] (**5'**).................................. 175

5.7 Crystal structure determination of [(^{Ad}BDI)Mg(C$_6$H$_3$-3,4-Me$_2$)]/ [(^{Ad}BDI)Mg(CH$_2$C$_6$H$_4$-2-Me)] (**6/6'**) 176

5.8 Crystal structure determination of [(^{Ad}BDI)Mg(C$_6$H$_3$-3,5-Me$_2$)] (**7**) 178

5.9 Crystal structure determination of [(^{Ad}BDI)Mg(CH$_2$C$_6$H$_4$-3-Me)] (**7'**)........................ 179

5.10 Crystal structure determination of [(^{Ad}BDI)Mg(CH$_2$C$_6$H$_4$-4-Me)] (**8**) 180

5.11 Crystal structure determination of [(^{Ad}BDI)Mg(C$_6$H$_3$-3,5-^{t}Bu$_2$)] (**9**) 181

5.12 Crystal structure determination of [(^{Me}BDI)Mg(κ^2*C,O*-C$_6$H$_4$-2-OMe)(thf)] (**10^{Me}·thf**) . 182

5.13 Crystal structure determination of [(^{Me}BDI)Mg(κ^1,*C*-C$_6$H$_4$-2-OMe)(κ^1,*O*-C$_6$H$_5$OMe)] (**10^{Ad}·anisole**)........ 184

5.14 Crystal structure determination of [(κ^2-^{Ad}BDI)Mg(μ-OMe)$_2$Mg(κ^1-^{Ad}BDI)] (**11**)........... 185

5.15 Crystal structure determination of [(^{Ad}BDI)Mg(μ-OMe)$_2$(Bpin)] (**12**) 186

5.16 Crystal structure determination of [(^{Ad}BDI)MgBr] (**13**) 187

6 Computational details 189

6.1 Computational details 189
6.2 Breakdown of Energy Contribution 189
6.3 Optimised structures and transition states 190
6.4 Calculated potential energy surfaces 201
6.5 Energy contributions for calculated structures and transition states 207
6.6 Cartesian Coordinates and Raw Electronic Energies (in Hartrees) 211
7 References 286

1 General experimental considerations

All manipulations were carried out using standard Schlenk line and glovebox techniques under an inert atmosphere of argon. NMR experiments were conducted in J. Young's NMR tubes and prepared in a glovebox. NMR spectra were recorded on a Bruker BioSpin GmbH spectrometer operating at 400.13 MHz (1H) and 100.62 MHz (13C), or an Agilent ProPulse spectrometer operating at 500 MHz (^{1}H) or 126 MHz (^{13}C). Elemental analyses were performed by the Elemental Analysis Services Team at London Metropolitan University. GC-MS analysis were performed with an Agilent/Markes 8890 gas chromatography system coupled with TDX100R MSD, used for the analysis. Split injections of 0.8 µL were performed, with a split ratio of 40:1 (split flow of 40 mL/min), using a split inlet liner, with tapered focus and deactivated glass wool (Agilent 5190-2293). The inlet was heated to 250°C with 3 mL/min septum purge flow. An Agilent HP-5MS (30 m, 0.25mm, 0.25µm) column was used with He (BOC, N5.5) as the carrier gas, at a constant flow of 1.0 mL/min. The column oven gradient was started at 35°C, held for 0.5 min, then ramped at 40°C/min to 320°C, with a 1 min hold. The MSD transfer line was set at 250°C, MSD source at 230°C, and the MSD quad temperature was set to 150°C. After an initial solvent delay of 2.8 min the MSD detection was performed using full scan mode, over the range of 42 – 300 m/z, with a gain factor of 15. Data analysis was performed in the Agilent Qualitative Analysis v.10.0 and used the NIST 17 library to identify and confirm compounds through spectral matching. Diethylether, toluene, and hexane were dried by passage through a commercially available solvent purification system and stored under argon in ampoules over 4 Å molecular sieves. C_6D_6, C_7D_8, methylcyclohexane, xylenes, 1,3-*tert*-butylbenzene, and THF were purchased from Merck, dried over potassium, distilled and stored over molecular sieves. [Pd(PCy$_3$)$_2$] (Cy = (CH$_2$)$_5$CH), was purchased from Fluorochem and used without purification. TMEDA, anisole, and aryl bromide reagents were dried over CaH_2, distilled, and stored over 4 Å molecular sieves. Other commercially available reagents were used as received.

Benzyl potassium was prepared from ^{n}BuLi, KO^{t}Bu, and toluene according to standard procedures. DippNC(Cl)Ad was synthesised according to literature procedures.[1] DippNC(CH$_3$)Ad was prepared from DippNC(Cl)Ad according to the analogous literature procedure for the synthesis of DippNC(CH$_3$)^{t}Bu.[2] Mg^{n}Bu$_2$ was synthesised according to literature procedures,[3] or purchased as 1.0 M solution in *n*-heptane and used as received. [(^{Me}BDI)Mg(µ-^{n}Bu)]$_2$ (**[1^{Me}]$_2$**) and [(^{Me}BDI)Mg(µ-H)]$_2$ (**[2^{Mg}]$_2$**) were prepared according to literature procedures.[4, 5]

2 Experimental procedures and characterisation

2.1 Synthesis and characterisation of ^{Ad}BDIH

DippNC(CH$_3$)Ad (12.19 g, 36.11 mmol), TMEDA (5.4 ml, 36.02 mmol), and Et_2O (60 ml) were combined in a Schlenk flask. The yellow solution was cooled to -40°C and ^{n}BuLi (2.5 M in hexane, 14.4 ml, 36 mmol) was added dropwise. The reaction mixture was stirred overnight and allowed to slowly warm to room temperature. The resulting solution of DippNC(Ad)CH$_2$Li was cooled to -

40°C and a Et_2O solution (60 ml) of DippNC(Cl)Ad (12.93 g, 36.12 mmol) added dropwise to provide an orange suspension. The reaction mixture was allowed to slowly warm to room temperature overnight then quenched with water (50 ml) and ice. The organic phases were combined and volatiles removed under vacuum. The crude product was extracted into hot chloroform (400 ml) and filtered, then recrystallised from chloroform/methanol mixture at -20°C. The product was collected by filtration, washed with methanol, then dried under vacuum at 60°C to provide 15.15 g ^{Ad}BDIH in 64% yield.

Large colourless block-like single crystals suitable for X-ray diffraction analysis were obtained by slowly concentrating a saturated ethanol/methylene chloride solution at room temperature.

^{1}H NMR (500 MHz, $CDCl_3$) δ 7.04 (d, *J* = 7.6 Hz, 4H, *meta*-C_6H_3-*i*Pr_2), 6.95 (q, *J* = 7.7, 6.5 Hz, 2H, *para*-C_6H_3-*i*Pr_2), 3.27 (s, 2H, NC(Ad)C*H*$_2$C(Ad)N), 2.62 (br, 4H, *H*C(CH_3)$_2$), 1.88 (br, 6H, ^{Ad}C*H*), 1.84 – 1.46 (m, 24H, ^{Ad}C*H*$_2$), 1.19 (d, *J* = 6.8 Hz, 12H, HC(C*H*$_3$)$_2$), 1.10 (d, *J* = 6.7 Hz, 12H, HC(C*H*$_3$)$_2$).

^{13}C NMR (126 MHz, $CDCl_3$) δ 145.91 (*ipso*-*C*$_6$H$_3$-*i*Pr$_2$), 134.99 (br, *ortho*-*C*$_6$H$_3$-*i*Pr$_2$), 122.48 (*meta*-*C*$_6$H$_3$-*i*Pr$_2$), 122.40 (*para*-*C*$_6$H$_3$-*i*Pr$_2$), 44.82 (br, NC^{Ad}*C*CH$_2$), 40.34 (^{Ad}*C*H$_2$), 36.75 (^{Ad}*C*H$_2$), 34.07 (br, NC(Ad)*C*H$_2$C(Ad)N), 28.66 (^{Ad}*C*H), 28.49 (H*C*(CH$_3$)$_2$), 23.62 (HC(*C*H$_3$)$_2$), 22.50 (br, HC(*C*H$_3$)$_2$). Iminic *C*N carbon resonance not detected.

Analysis calculated for $C_{47}H_{66}N_2$: C, 85.66; H, 10.06; N, 4.24%. Calculated: C, 85.42; H, 10.01; N, 4.19%.

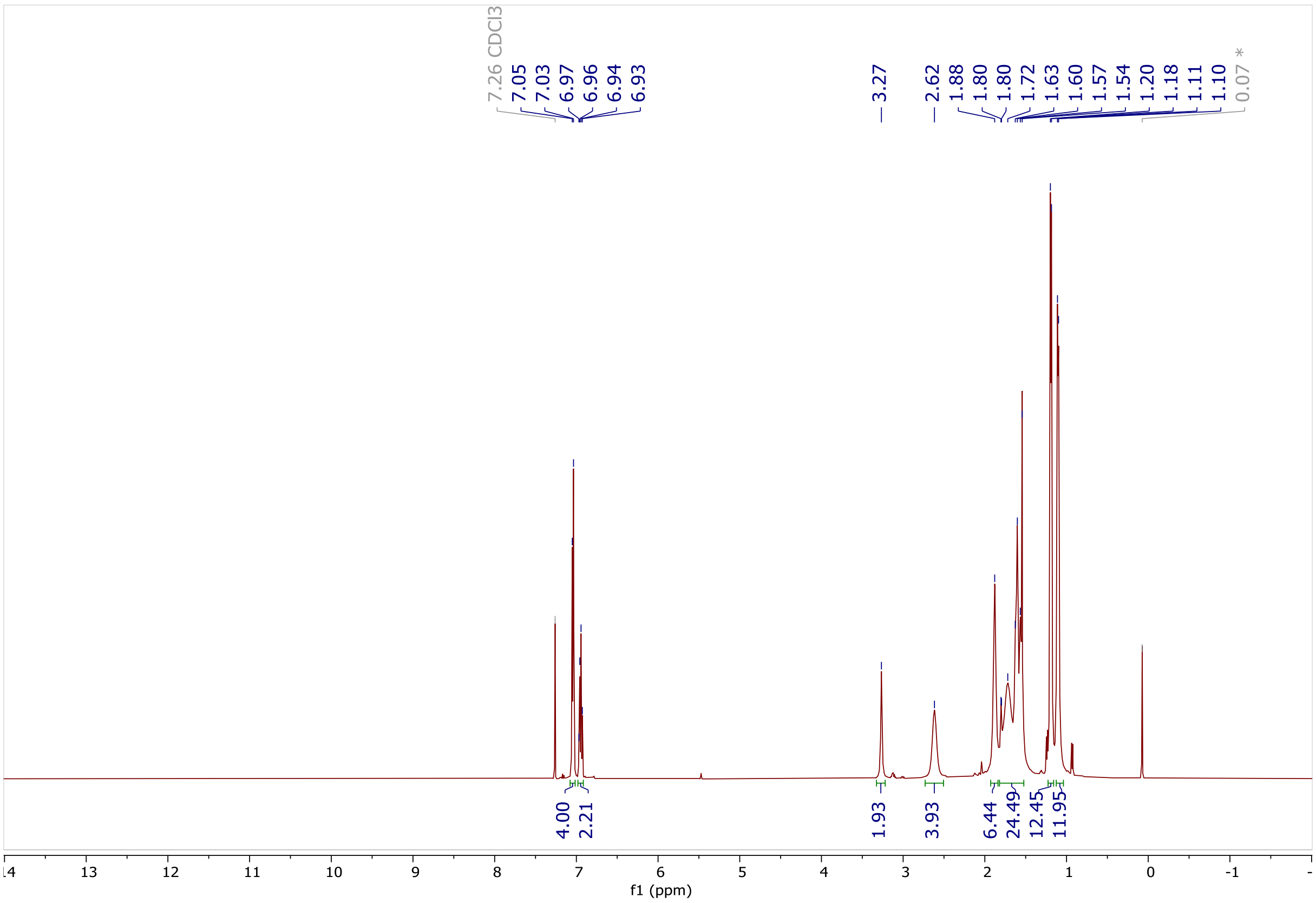

Figure S1: ^{1}H NMR (500 MHz, $CDCl_3$). *Grease.

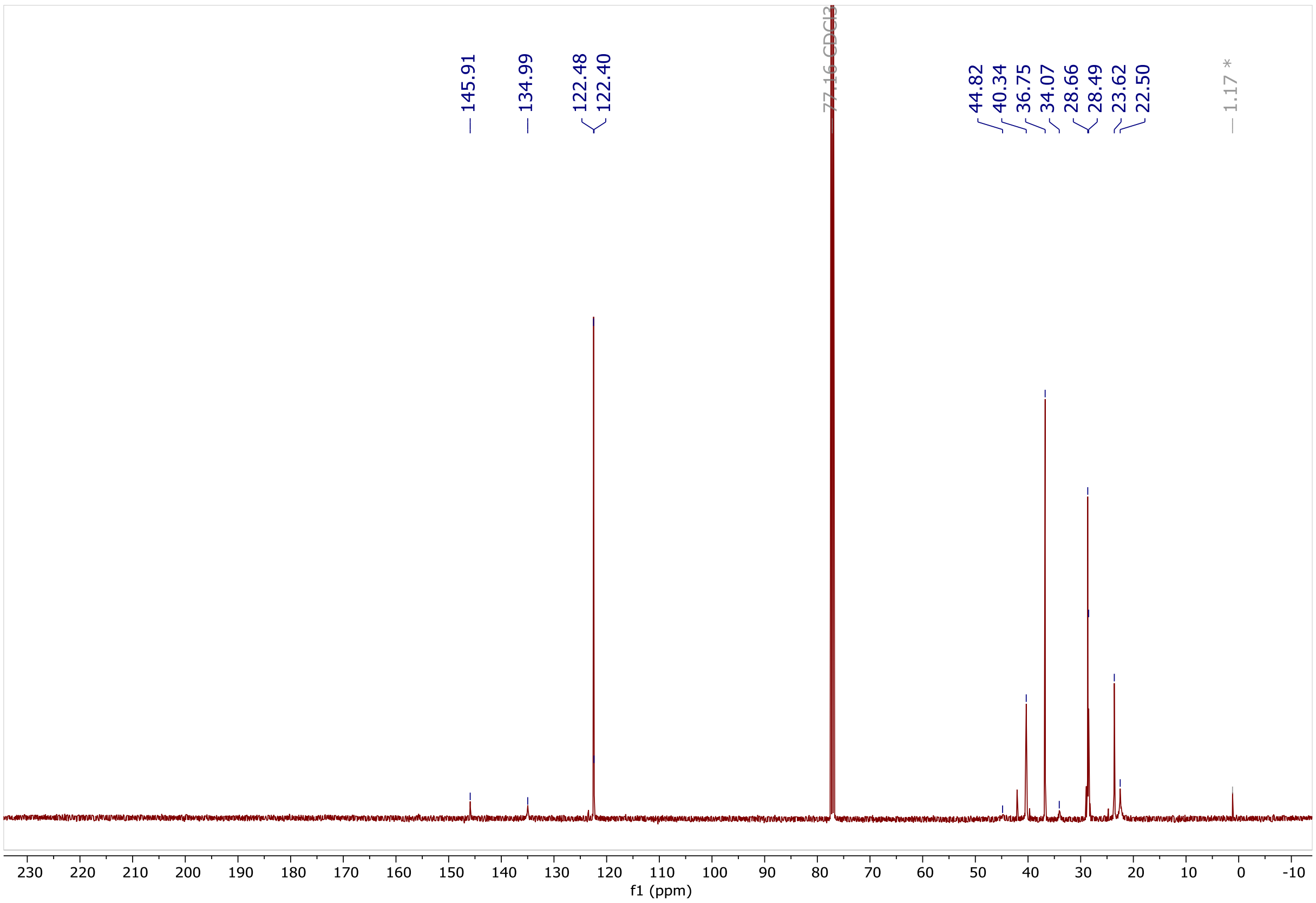

Figure S2: $^{13}C\{^{1}H\}$ NMR (126 MHz, $CDCl_3$). *Grease.

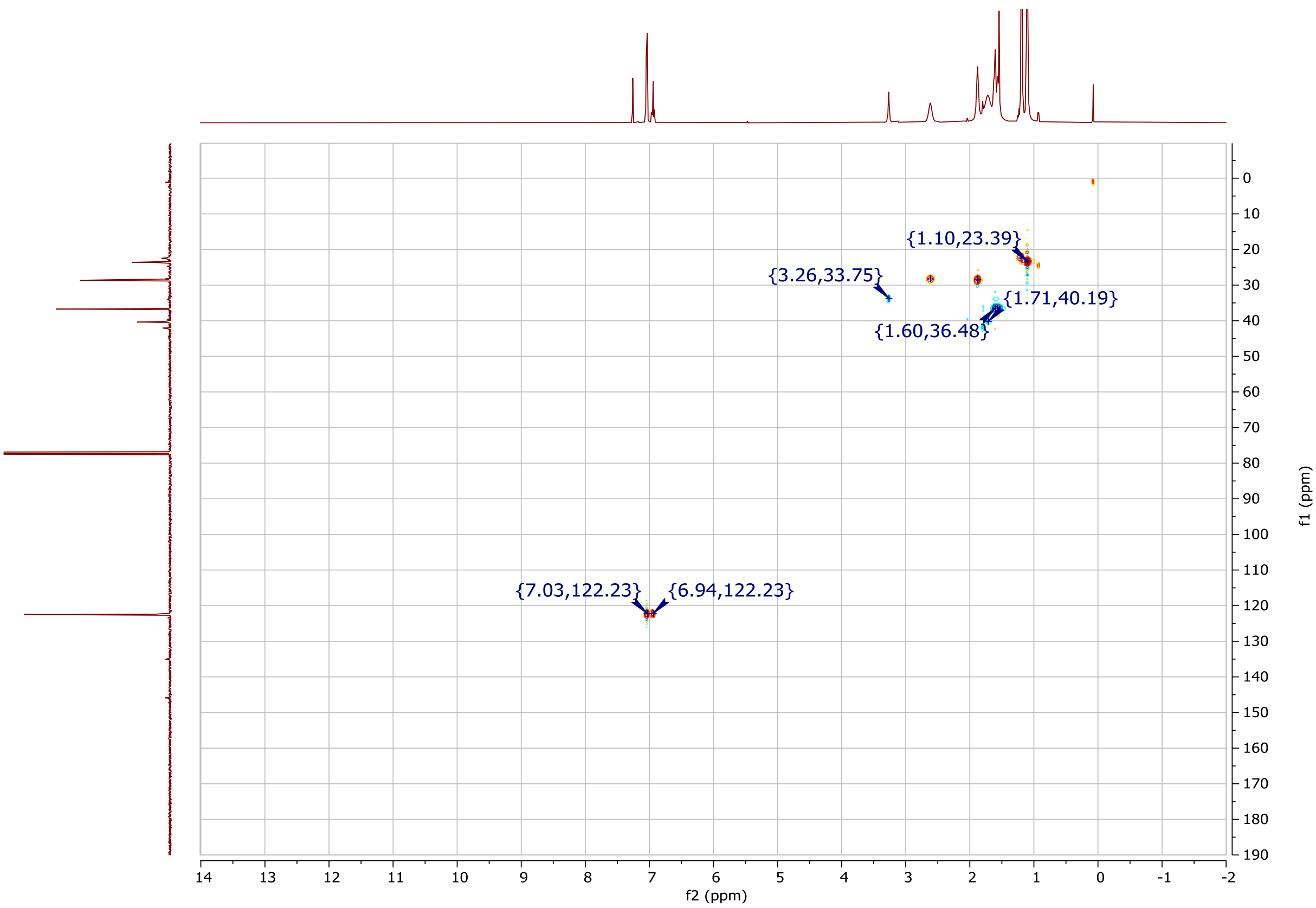

Figure S3: ^{13}C-^{1}H HSQC spectrum ($CDCl_3$)

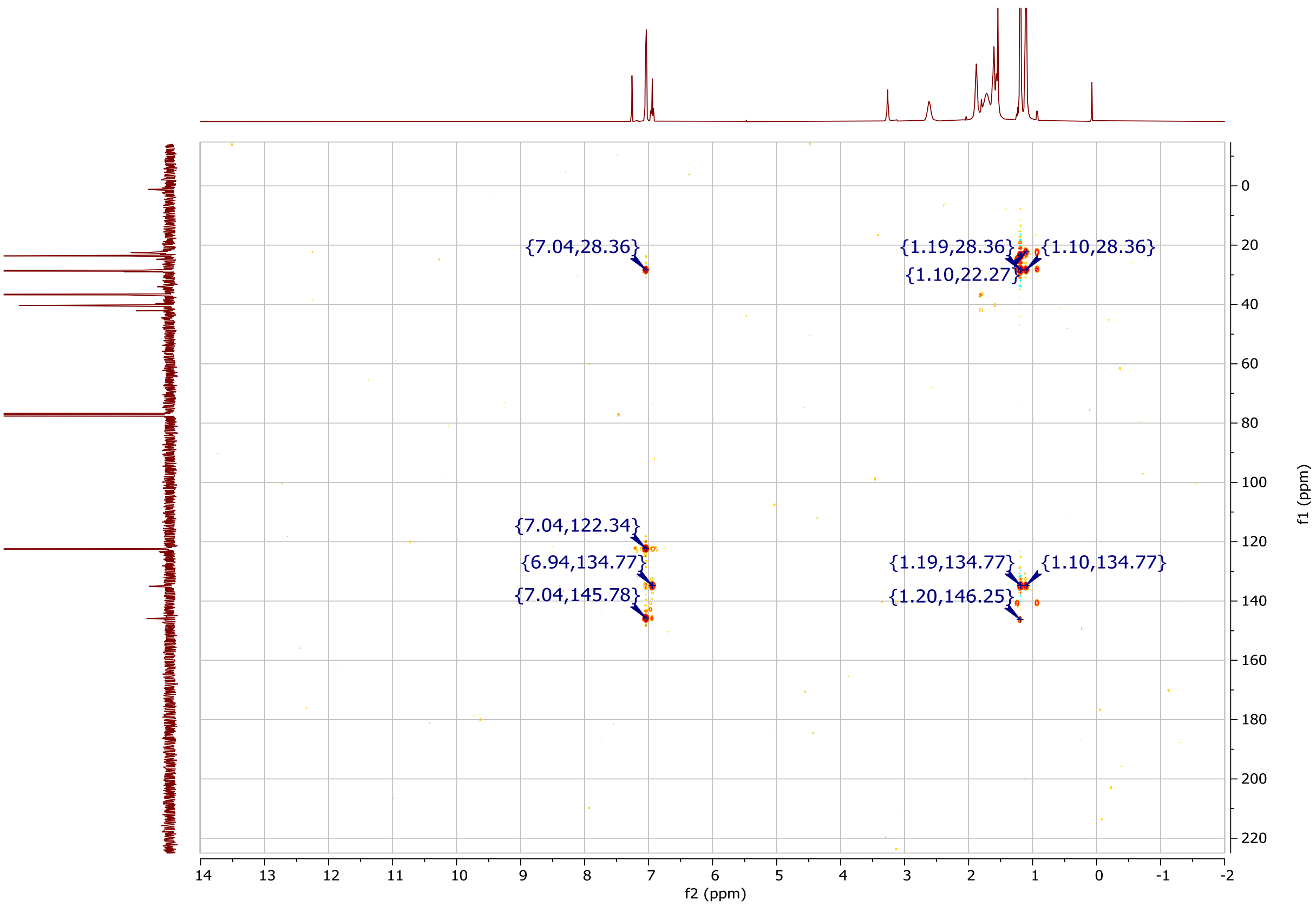

Figure S4: ^{13}C-^{1}H HMBC spectrum ($CDCl_3$)

2.2 Synthesis of [(^{Ad}BDI)Mg^{n}Bu] (1^{Ad})

In a Schlenk flask, 1.98 g ^{Ad}BDIH (3.00 mmol) was dissolved in 40 ml toluene to provide a yellow solution. Mg^{n}Bu$_2$ (3 ml, 1.0 M in *n*-heptane, 3.00 mmol) was added at the reaction mixture stirred at 60°C for 16 hours. Volatiles were removed under vacuum at 80°C to provide a pale-yellow solid, which was redissolved in hot *n*-heptane (approx. 40 ml). Storing the resulting yellow solution at room temperature overnight provided 0.95 g **1^{Ad}** as a light yellow crystalline solid, which was isolated by cannula filtration. The filtrate was concentrated to incipient crystallisation, then transferred to a freezer at -20°C for 1 week, resulting in a second crop of crystals (0.38 g), which were isolated by cannula filtration. Total yield 1.33 g, 60%. Single crystals suitable for X-ray diffraction analysis were obtained from a saturated methylcyclohexane solution at -35°C.

Analysis calculated for $C_{51}H_{74}MgN_2$: C, 82.84; H, 10.09; N, 3.79%. Found: C, 82.41; H, 10.48; N, 3.66%.

^{1}H NMR (500 MHz, C_6D_6) δ 7.07 (dd, *J* = 8.6, 6.5 Hz, 2H, *p*-NC$_6$$\underline{H_3}$-*i*Pr$_2$), 7.05 – 6.99 (m, 4H, *m*-NC$_6$$\underline{H_3}$-*i*Pr$_2$), 5.67 (s, 1H, $\underline{H}$CC(Ndipp)), 3.36 (p, *J* = 6.9 Hz, 4H, $\underline{H}$C(CH$_3$)$_2$), 2.13 (d, *J* = 3.0 Hz, 12H, ^{Ad}CC$\underline{H_2}$CH), 1.88 – 1.82 (m, 6H, ^{Ad}C$\underline{H}$), 1.65 – 1.56 (m, 6H, ^{Ad}CHC$\underline{H_2}$CH), 1.56 – 1.49 (m, 6H, ^{Ad}CHC$\underline{H_2}$CH), 1.35 (d, *J* = 5.0 Hz, 12H, HC(C$\underline{H_3}$)$_2$), 1.33 (d, *J* = 5.4 Hz, 12H, HC(C$\underline{H_3}$)$_2$), 0.97 (tq, *J* = 6.3, 3.0 Hz, 4H, MgCH$_2$(C$\underline{H_2}$)$_2$CH$_3$), 0.83 (td, *J* = 8.1, 7.3, 3.0 Hz, 3H, Mg(CH$_2$)$_3$C$\underline{H_3}$), -0.62 (m, 2H, MgC$\underline{H_2}$).

^{13}C NMR (126 MHz, C_6D_6) δ 176.05 (N=$\underline{C}$), 145.47 (*ipso*-N$\underline{C_6}$H$_3$-*i*Pr$_2$), 141.06 (*ortho*-N$\underline{C_6}$H$_3$-*i*Pr$_2$), 125.23 (*para*-N$\underline{C_6}$H$_3$-*i*Pr$_2$), 123.76 (*meta*-N$\underline{C_6}$H$_3$-*i*Pr$_2$), 93.66 (H$\underline{C}$C(Ndipp)), 47.81 (NC^{Ad}$\underline{C}$), 43.71 (^{Ad}C$\underline{C}$H$_2$CH), 37.01 (^{Ad}CH$\underline{C}$H$_2$CH), 30.79 (MgCH$_2$($\underline{C}$H$_2$)$_2$CH$_3$), 30.60 (MgCH$_2$($\underline{C}$H$_2$)$_2$CH$_3$), 29.53 (^{Ad}$\underline{C}$H), 28.44 (H$\underline{C}$(CH$_3$)$_2$), 25.35 (HC($\underline{C}$H$_3$)$_2$), 23.23 (HC($\underline{C}$H$_3$)$_2$), 14.33 (Mg(CH$_2$)$_3$$\underline{C}H_3$), 6.96 (Mg$\underline{C}H_2$).

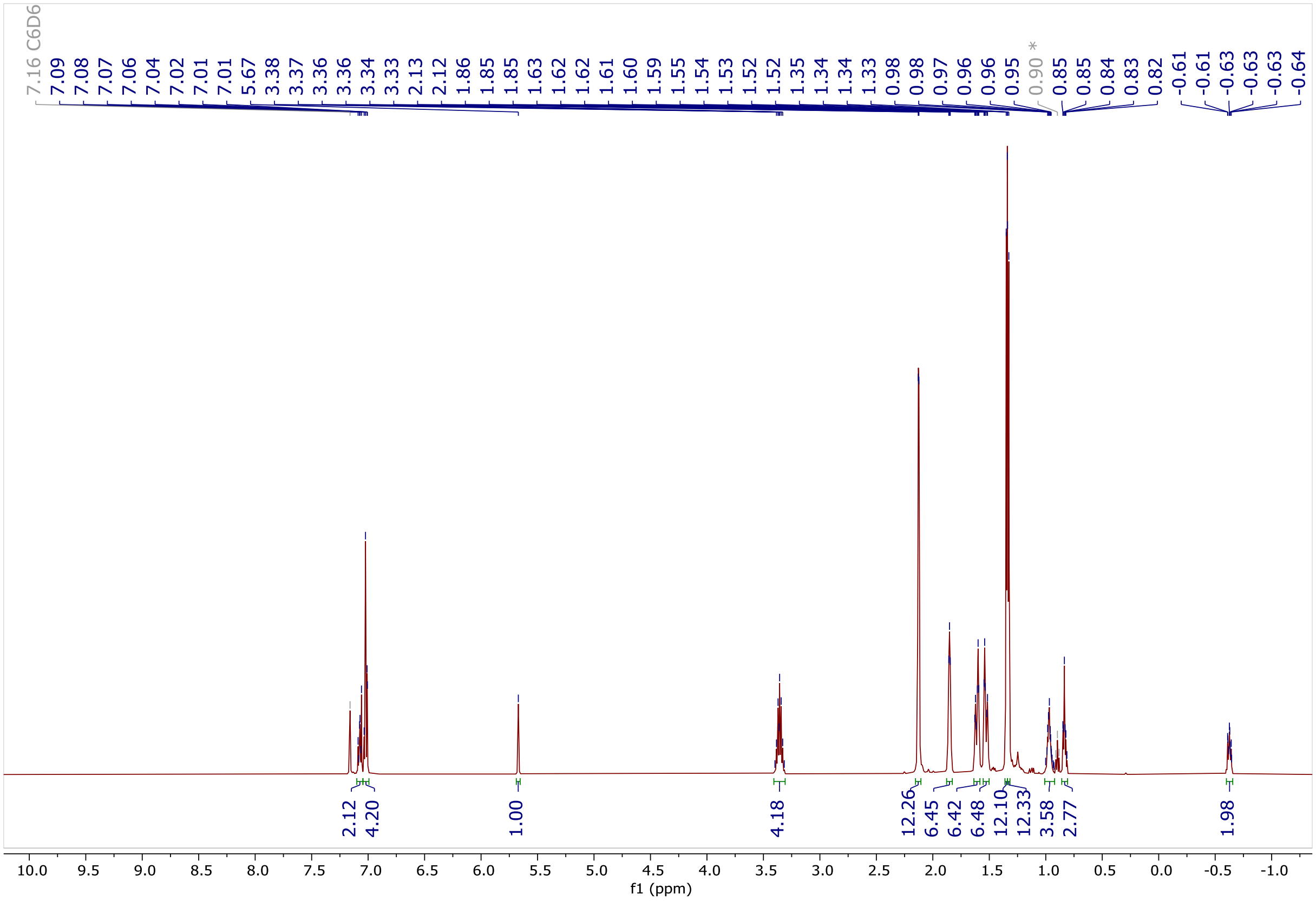

Figure S5: ^{1}H NMR (500 MHz, C_6D_6). *n-heptane.

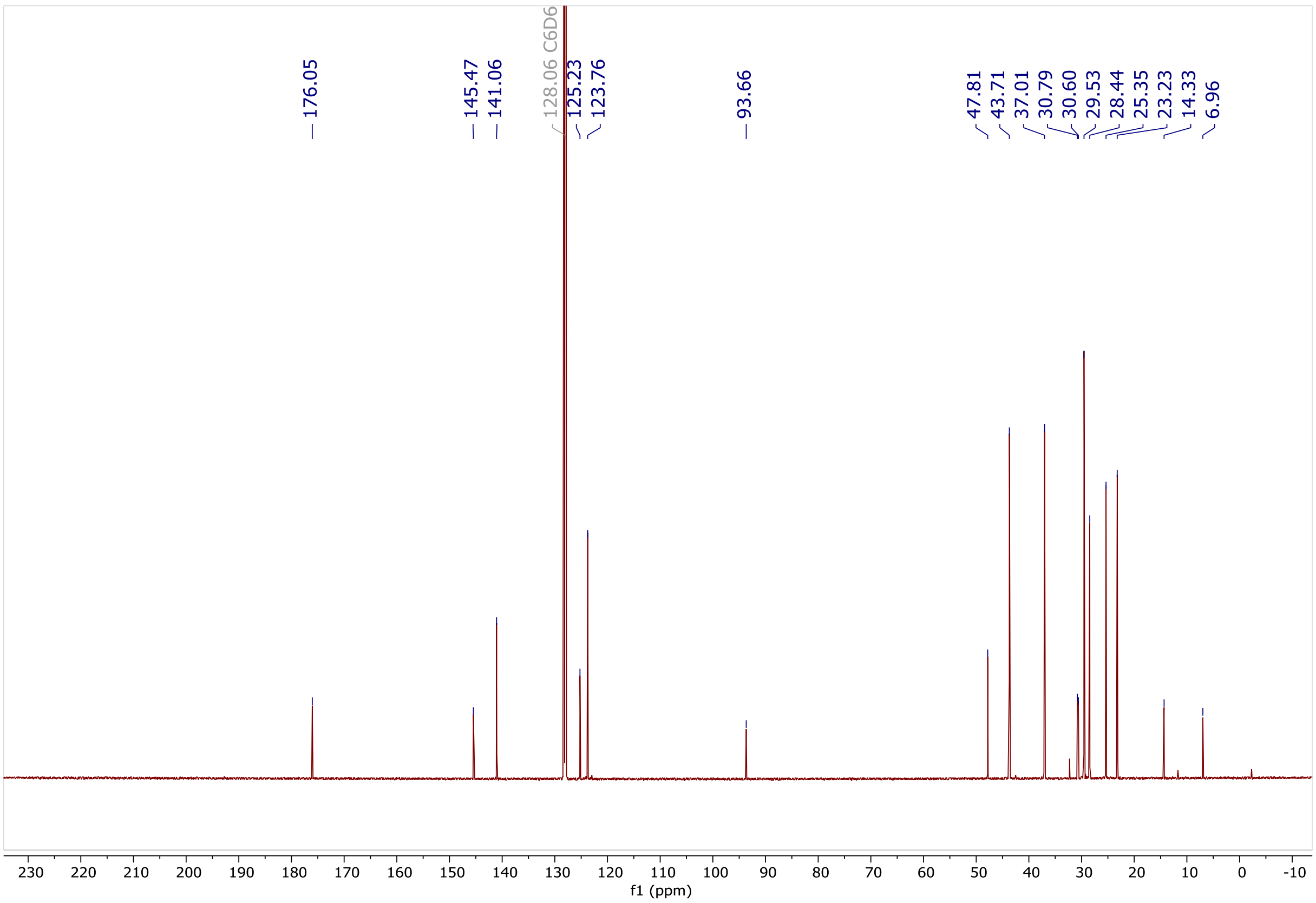

Figure S6: $^{13}C\{^{1}H\}$ NMR (126 MHz, C_6D_6).

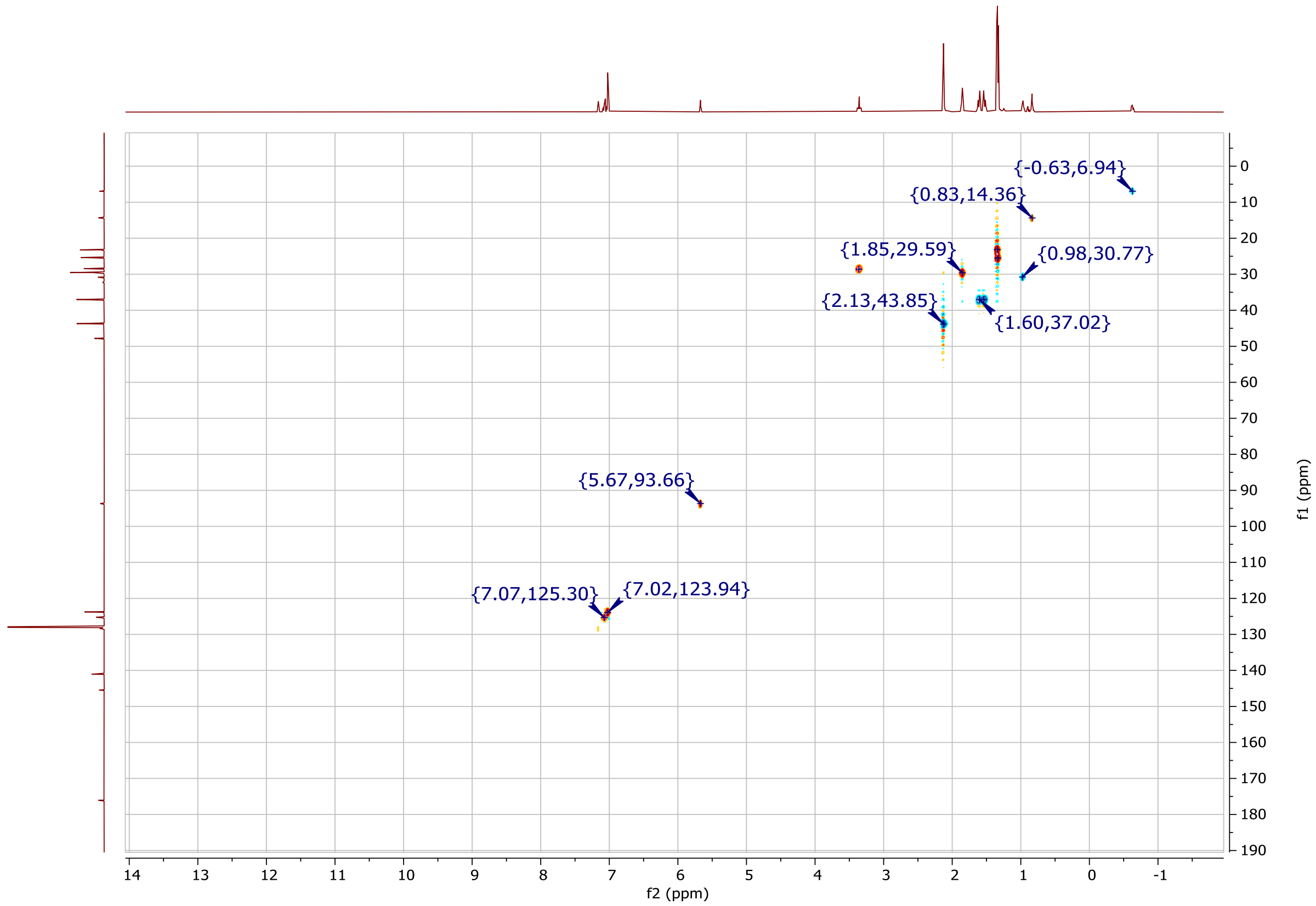

Figure S7: ^{13}C-^{1}H HSQC NMR (126 MHz, C_6D_6).

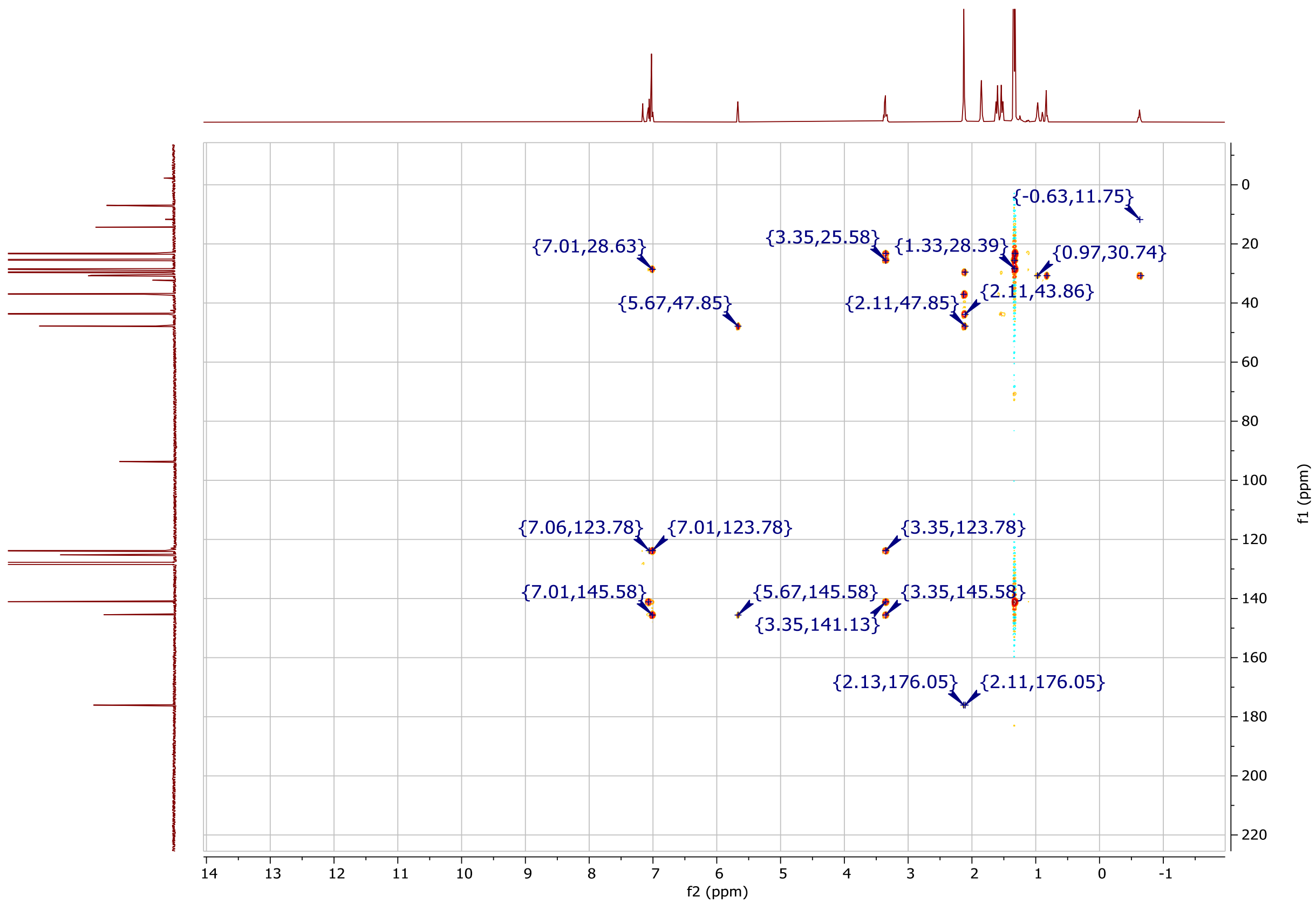

Figure S8: ^{13}C-^{1}H HMBC NMR (126 MHz, C_6D_6).

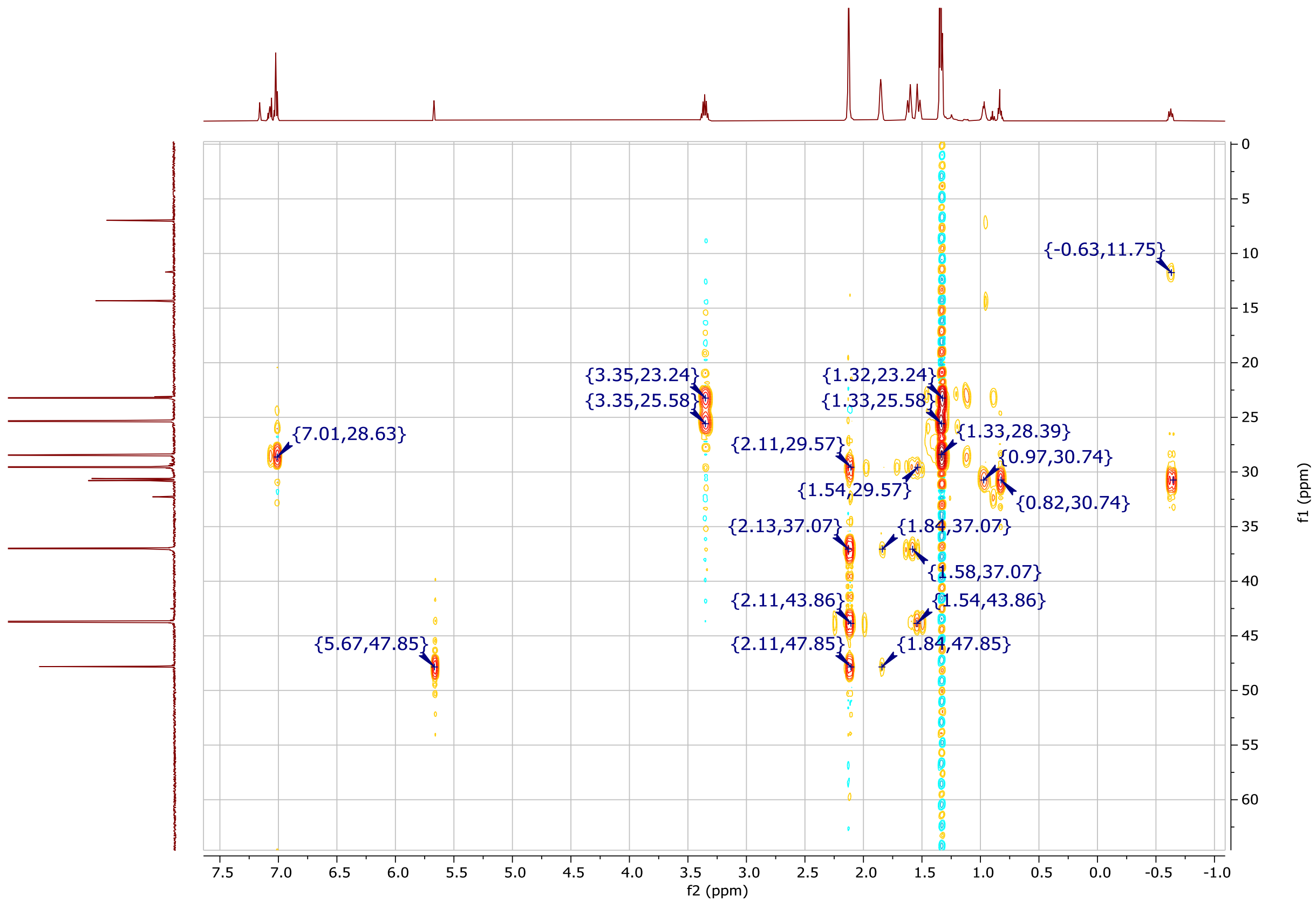

Figure S9: Expanded ^{13}C-^{1}H HMBC NMR (126 MHz, C_6D_6).

2.3 Synthesis of [(^{Me}BDI)Mg(Cp^{4M})] (4)

To a J. Young's NMR containing 50 mg [(^{Me}BDI)Mg^{n}Bu]$_2$ (**[1^{Me}]$_2$**, 0.05 mmol) in 0.6 ml C_6D_6 was added 15 µl $C_5Me_4H_2$ (0.1 mmol). The reaction tube was sealed and heated to 100°C for 6 days. Slow evaporation of the reaction mixture provided colourless needle-shaped crystals of **4** suitable for single crystal X-ray diffraction analysis, which were isolated in 44 mg yield, 78% after washing with *n*-pentane.

Analysis calculated for $C_{38}H_{54}MgN_2$: C, 81.04; H, 9.67; N, 4.97%. Found: C, 81.61; H, 9.52; N, 4.81%.

^{1}H NMR (500 MHz, C_6D_6) δ 7.18 (s, 6H, Dipp-C<u>*H*</u>), 5.81 (s, 1H, C_5Me_4<u>*H*</u>), 4.70 (s, 1H, <u>*H*</u>CC(Me)N), 3.05 (hept, *J* = 6.8 Hz, 4H, <u>*H*</u>C(CH_3)$_2$), 1.81 (s, 6H, C_5(C<u>H_3</u>)$_4$H), 1.53 (s, 6H, C_5(C<u>H_3</u>)$_4$H), 1.49 (s, 6H, NCC<u>H_3</u>), 1.43 (d, *J* = 6.8 Hz, 12H, HC(C<u>H_3</u>)$_2$), 1.10 (d, *J* = 6.8 Hz, 12H, HC(C<u>H_3</u>)$_2$).

^{13}C NMR (126 MHz, C_6D_6) δ 170.43 (<u>*C*</u>=N), 147.43 (1-<u>C_6</u>$H_3{}^{i}Pr_2$), 142.10 (2,6-<u>C_6</u>$H_3{}^{i}Pr_2$), 125.76 (4-<u>C_6</u>$H_3{}^{i}Pr_2$), 124.41 (3,5-<u>C_6</u>$H_3{}^{i}Pr_2$), 114.49 (<u>C_4</u>Me_4CH), 112.76 (<u>C_4</u>Me_4CH), 104.75 (H<u>*C*</u>C_4Me_4), 94.90 (H<u>*C*</u>C(Me)N), 29.20 (H<u>*C*</u>(CH_3)$_2$), 25.25 (NC<u>*C*</u>H_3), 24.53 (HC(<u>*C*</u>H_3)$_2$), 24.45 (HC(<u>*C*</u>H_3)$_2$), 12.67 (C_5(<u>*C*</u>H_3)$_4$H), 10.98 (C_5(<u>*C*</u>H_3)$_4$H).

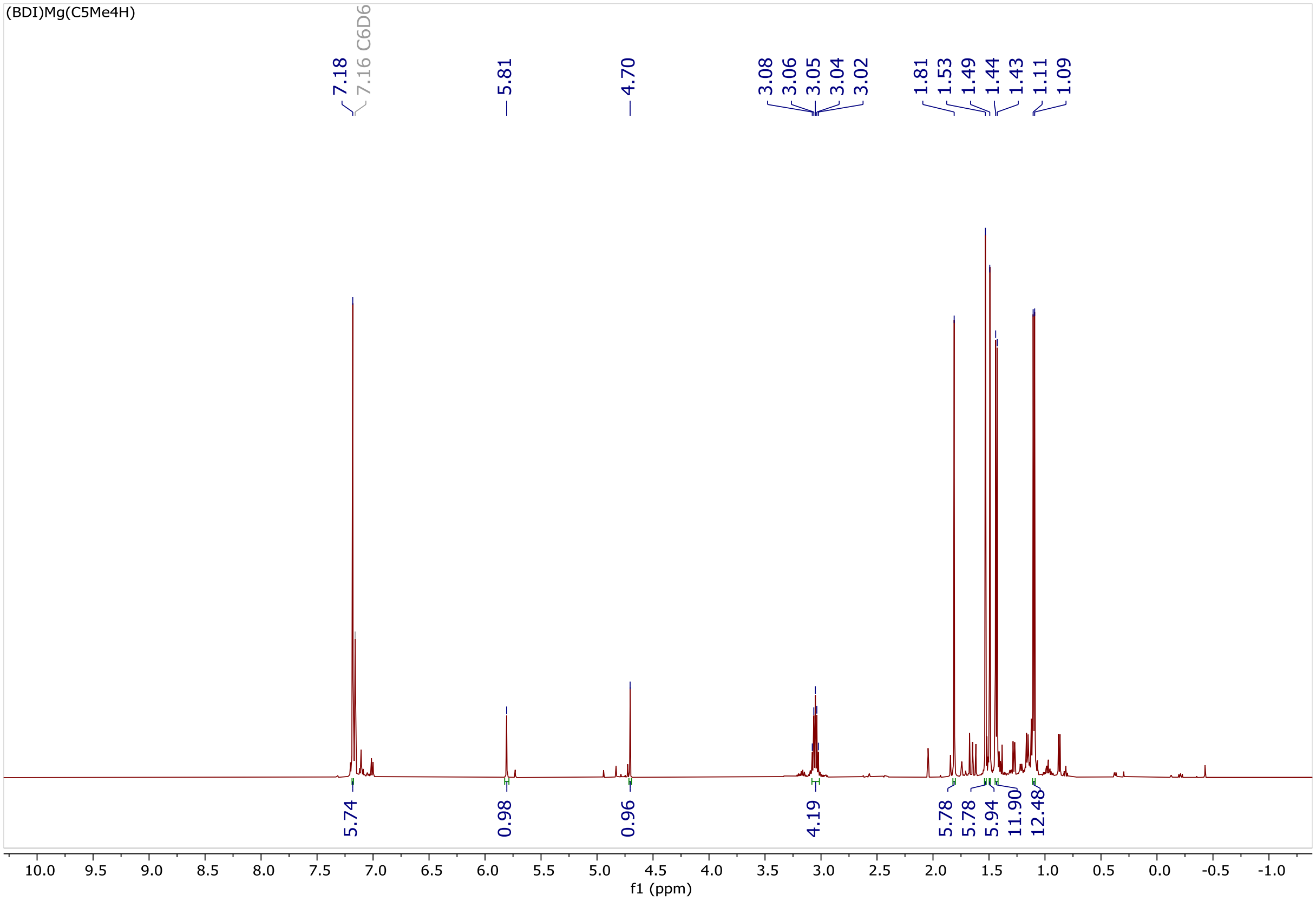

Figure S10: ^{1}H NMR (500 MHz, C_6D_6)

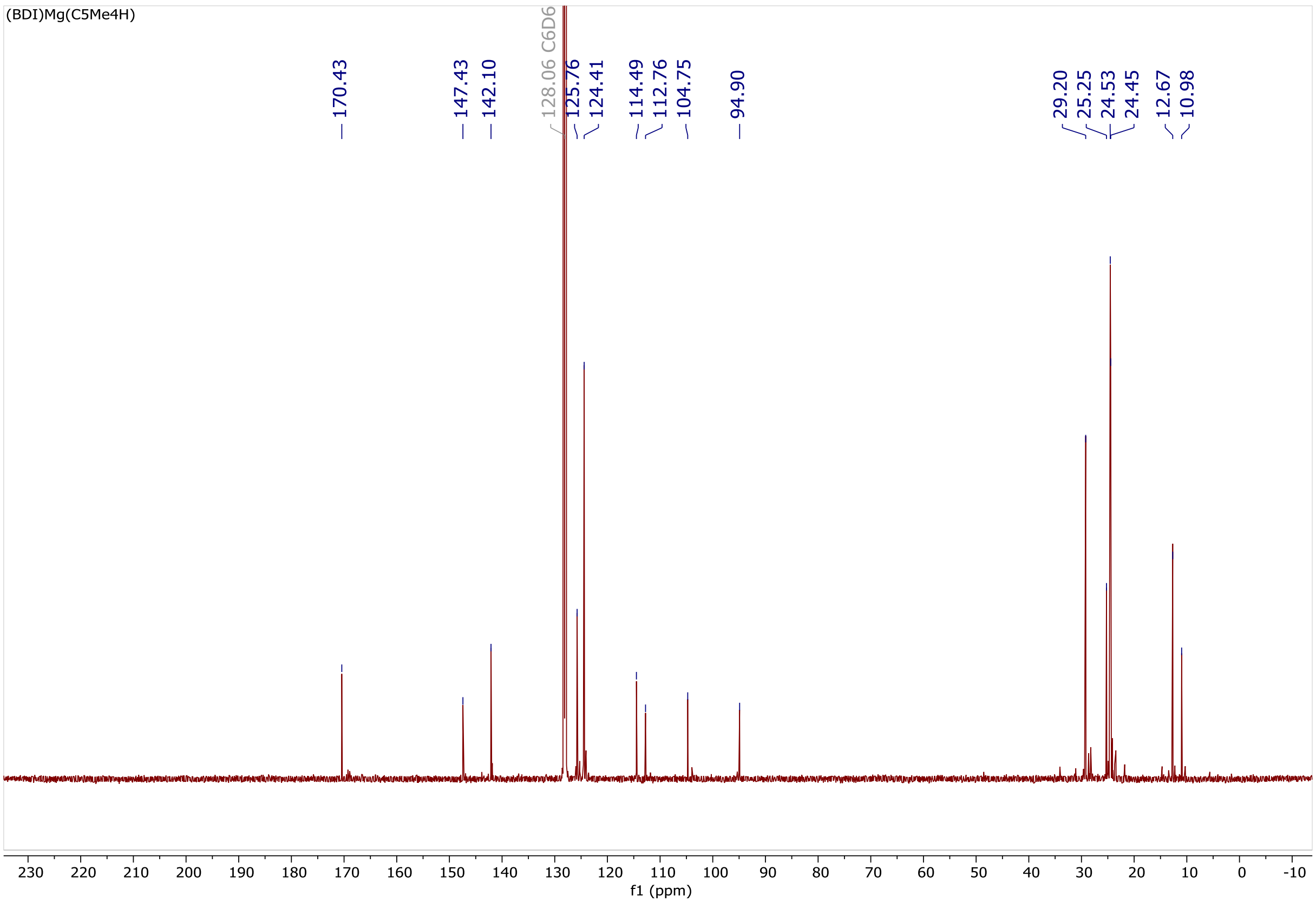

Figure S11: $^{13}C\{^{1}H\}$ NMR (126 MHz, C_6D_6)

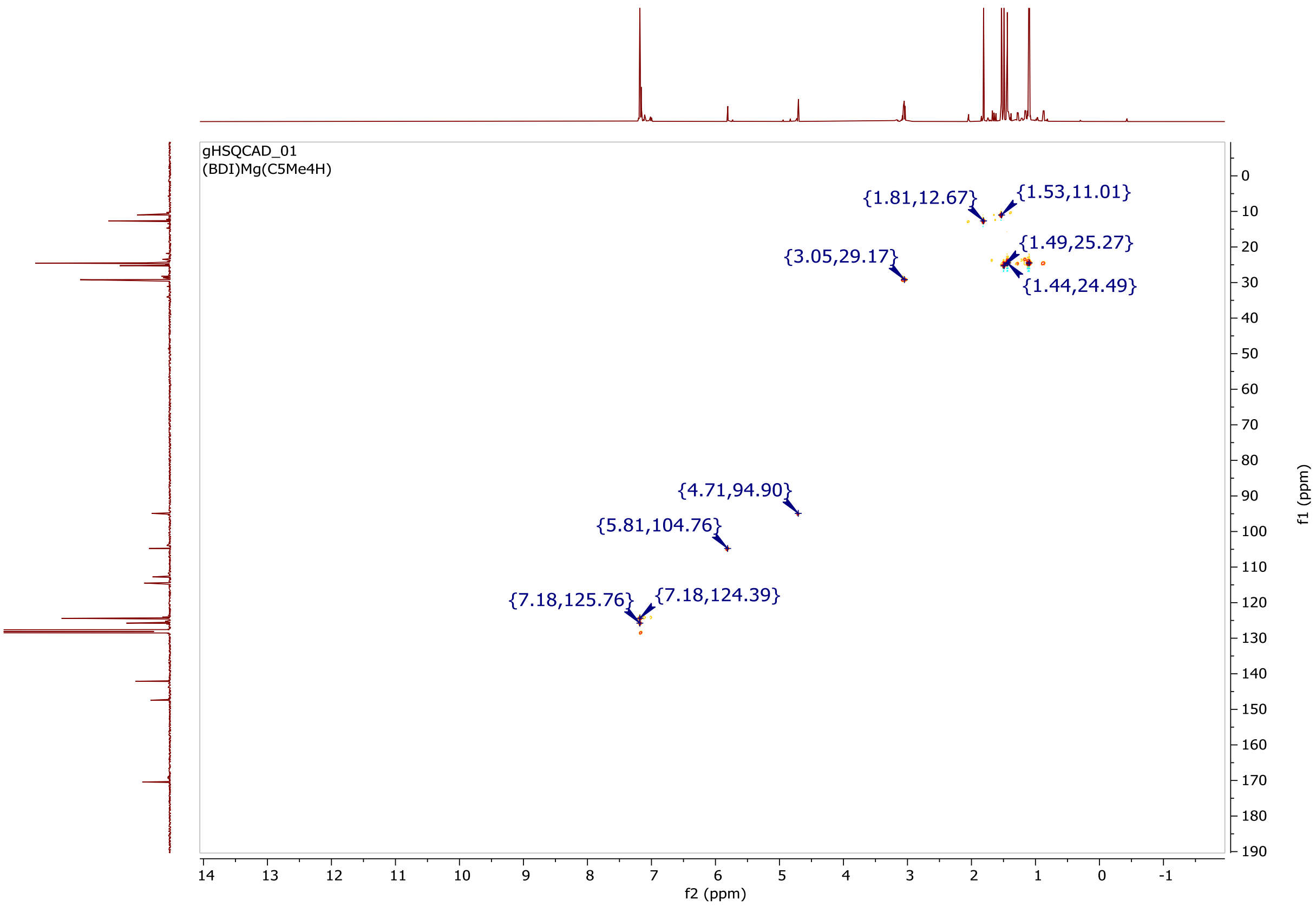

Figure S12: ^{13}C-^{1}H HSQC NMR (126 MHz, C_6D_6).

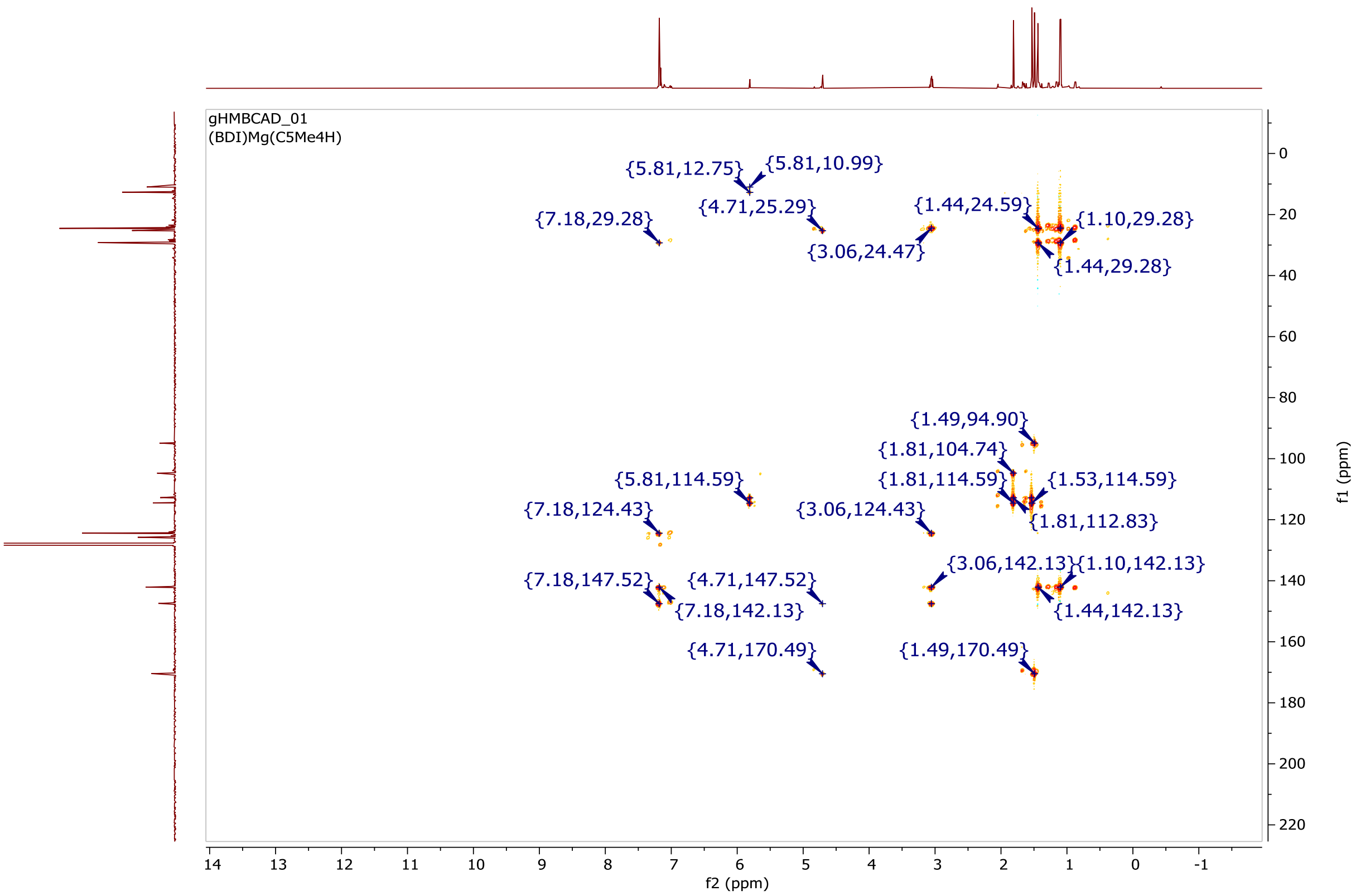

Figure S13: ^{13}C-^{1}H HMBC NMR (126 MHz, C_6D_6).

2.4 Attempted reaction of [(^{Ad}BDI)Mg^{n}Bu] (1^{Ad}) with Cp^{4M}H in benzene-d_6, synthesis and characterisation of [(^{Ad}BDI)Mg(C_6D_5)] (3^{Ad}-d_5)

A J. Young's NMR tube was charged with 15 mg **1^{Ad}** (0.02 mmol) and 0.6 ml benzene-d_6, then 3 µl Cp^{4M}H (0.02 mmol) was added. No reaction was observed after 24 hours at 60°C. After heating at 100°C for 7 days, near-quantitative conversion of the starting complex to a single new BDI-containing species was observed but Cp^{4M}H was not consumed. Volatiles were removed under vacuum to yield a yellow residue which was extracted into a *n*-hexane-toluene mixture. Slow evaporation of the solvent provided colourless block-like single crystals of **3^{Ad}-*d*$_5$** suitable for X-ray diffraction analysis. Yield after washing with *n*-hexane: 6 mg, 42%.

3^{Ad}-*d*$_5$ was also obtained by the same procedure without addition of Cp^{4}H. Isolated yield after crystallisation: 5 mg, 31%.

^{1}H NMR (400 MHz, C_6D_6) δ 7.23 (dd, *J* = 8.2, 7.1 Hz, 2H, *p*-NC_6H_3-*i*Pr$_2$), 7.11 (d, *J* = 7.7 Hz, 4H, *m*-NC_6H_3-*i*Pr$_2$), 5.74 (s, 1H, *H*CC(Ndipp)), 3.42 (p, *J* = 6.7 Hz, 4H, *H*C(CH$_3$)$_2$), 2.15 (d, *J* = 3.0 Hz, 12H, ^{Ad}CC*H*$_2$CH), 1.86 (dd, *J* = 6.9, 3.8 Hz, 12H, ^{Ad}C*H*), 1.61 (d, *J* = 12.4 Hz, 6H, ^{Ad}CHC*H*$_2$CH), 1.53 (d, *J* = 12.3 Hz, 6H, ^{Ad}CHC*H*$_2$CH), 1.33 (d, *J* = 6.9 Hz, 12H, HC(C*H*$_3$)$_2$), 1.22 (d, *J* = 6.9 Hz, 12H, HC(C*H*$_3$)$_2$).

^{2}H NMR (77 MHz, C_6H_6) δ 7.01 (*m*,*p*-MgC_6D_5), 6.17 (*o*-MgC_6D_5), 1.19*. *Tentatively assigned to partial deuteration of *iso*-propyl methyl positions.

^{13}C NMR (101 MHz, C_6D_6) δ 176.57 (N=*C*), 158.45 (Mg*C*)*, 145.27 (*ipso*-NC_6H_3-*i*Pr$_2$), 141.53 (*ortho*-NC_6H_3-*i*Pr$_2$), 125.58 (*para*-NC_6H_3-*i*Pr$_2$), 124.19 (*meta*-NC_6H_3-*i*Pr$_2$), 93.78 (H*C*C(Ndipp)), 47.91 (NC^{Ad}*C*), 43.72 (^{Ad}*C*CH$_2$CH), 36.98 (^{Ad}CH*C*H$_2$CH), 29.50 (^{Ad}*C*H), 28.53 (H*C*(CH$_3$)$_2$), 25.79 (HC(*C*H$_3$)$_2$), 23.13 (HC(*C*H$_3$)$_2$). Resonances for deuterium-bound carbon atoms not observed due to signal loss arising from ^{13}C-^{2}D coupling. **ipso*-Mg*C* resonance qualitatively reduced in intensity compared to *proteo* compound due to effect of ^{2,3}J-coupling to deuterium.

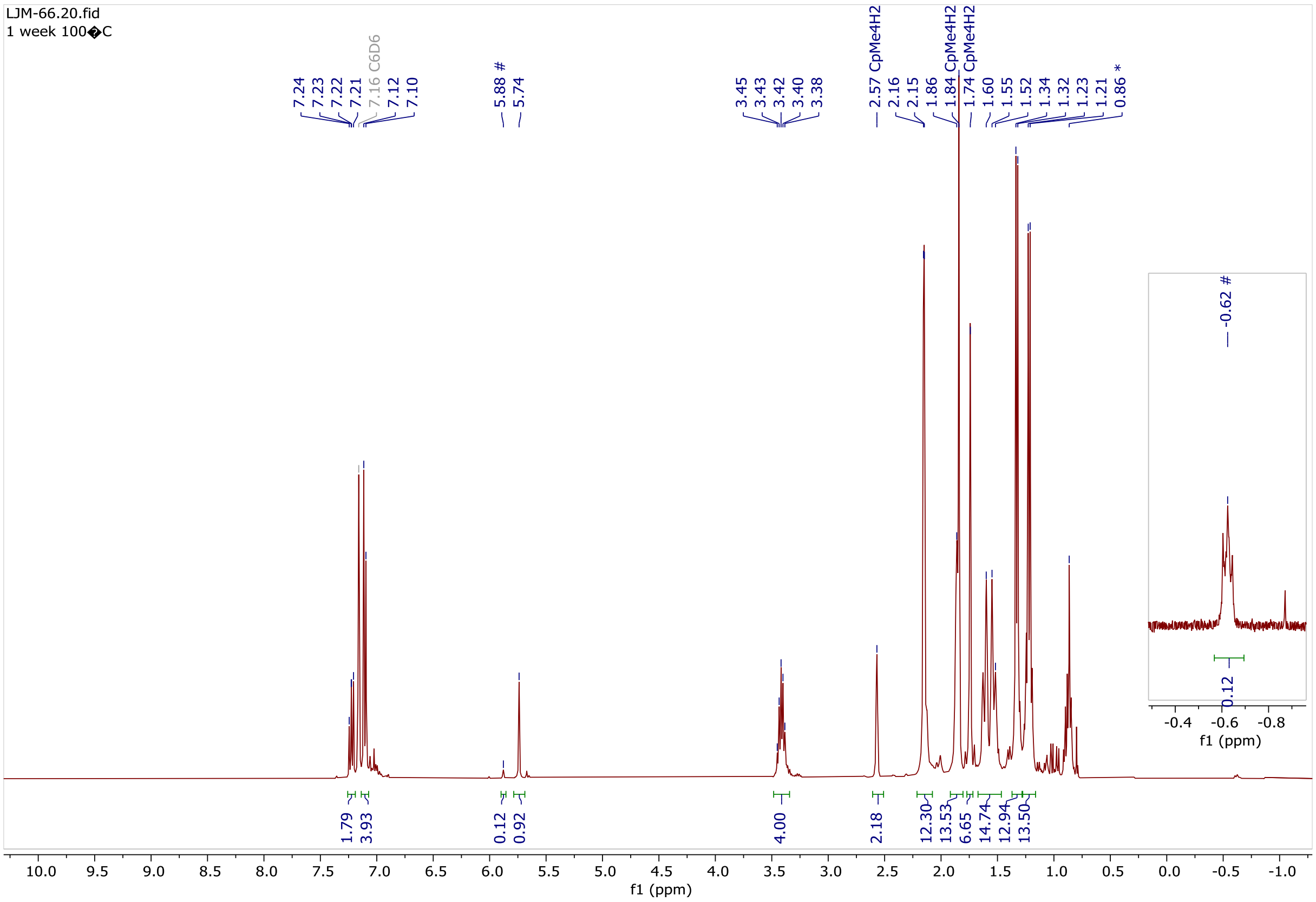

Figure S14: *In situ* ^{1}H NMR spectrum (400 MHz, C_6D_6) after 1 week at 100°C. **n*-butane. #Expanded region showing residual **1^{Ad}**. Resonances corresponding to unreacted Cp^{4M}H are annotated.

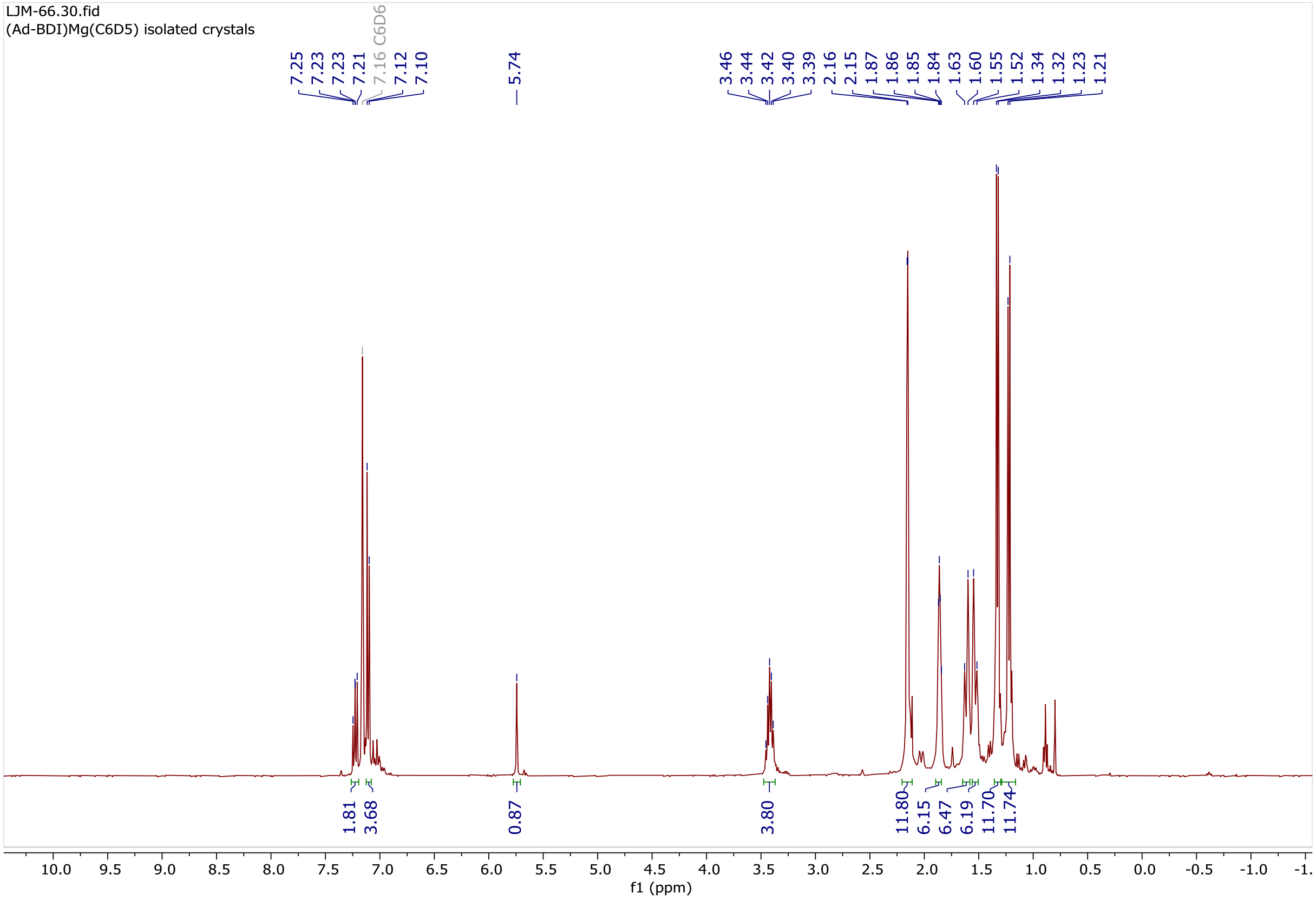

Figure S15: ^{1}H NMR of $\mathbf{3^{Ad}}$-$\boldsymbol{d_5}$ (400 MHz, C_6D_6).

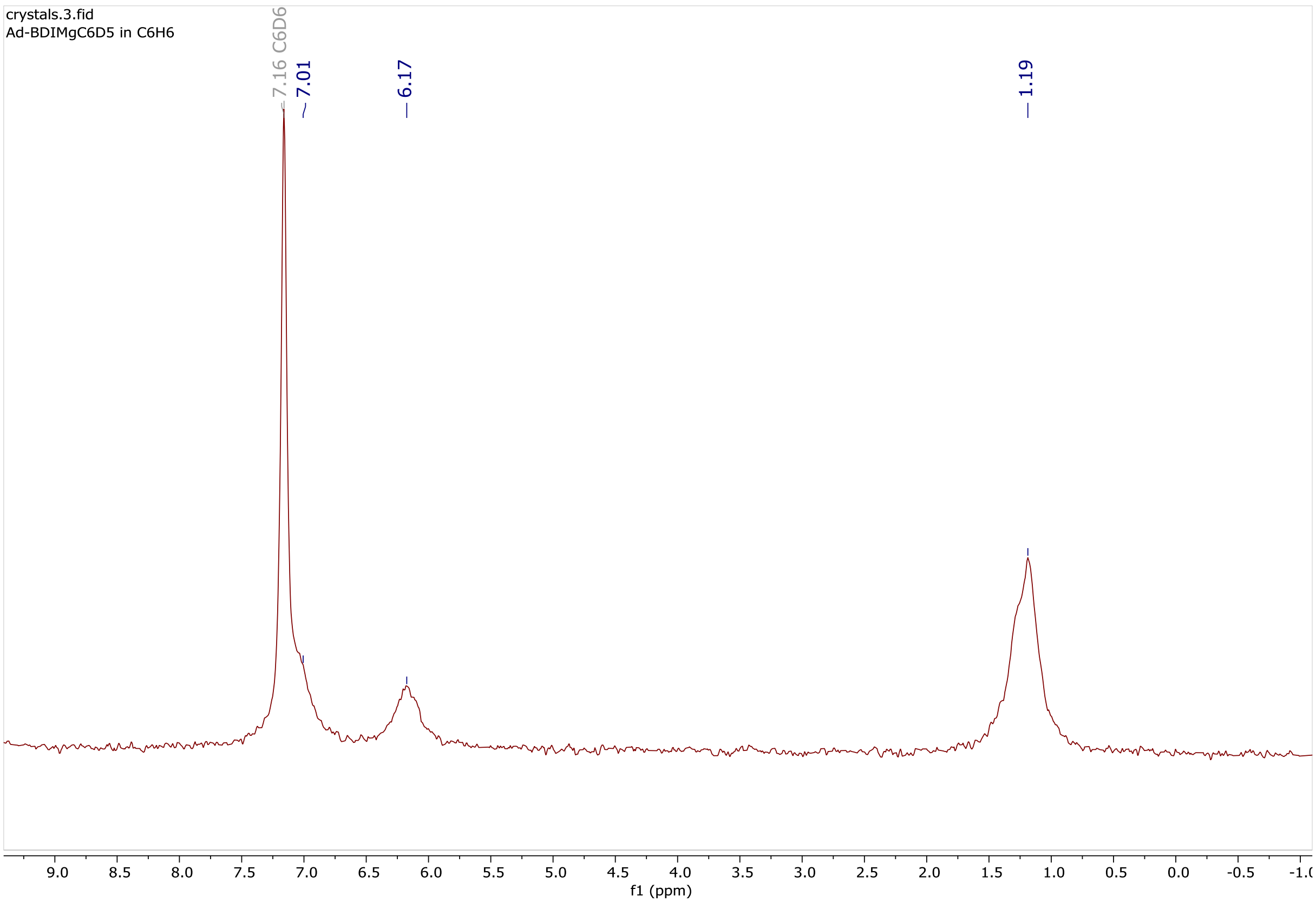

Figure S16: ^{2}H NMR of $\mathbf{3^{Ad}}$-$\boldsymbol{d_5}$ (77 MHz, C_6H_6). Residual C_6D_6 observed despite prolonged evacuation prior to dissolution in C_6H_6. Resonance at 1.19 ppm tentatively attributed to deuteration of *iso*propyl groups.

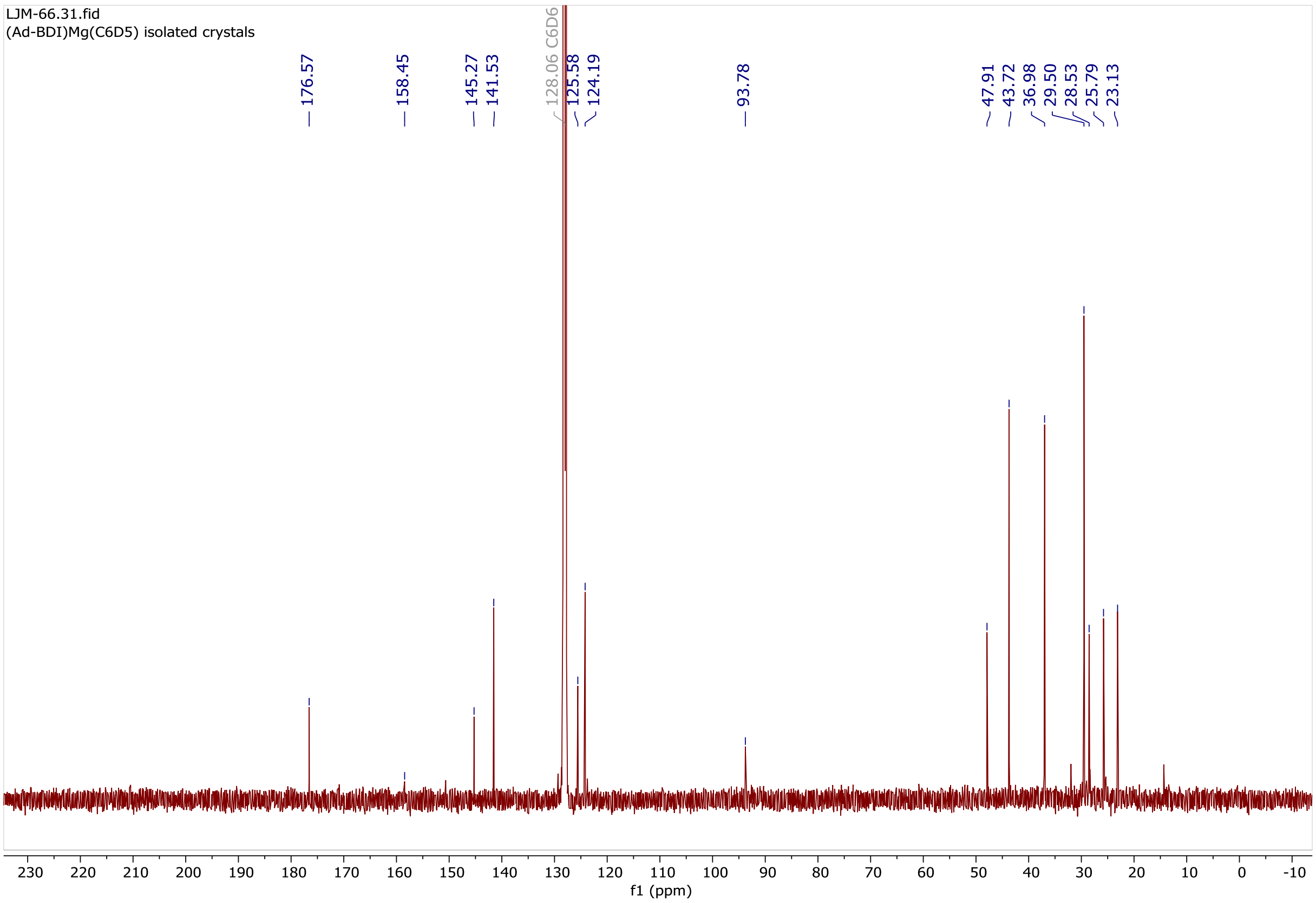

Figure S17: $^{13}C\{^{1}H\}$ NMR of $\mathbf{3^{Ad}}$-$\boldsymbol{d_5}$ (101 MHz, C_6D_6)

2.5 Attempted reaction of [(^{Ad}BDI)Mg^{n}Bu] ($\mathbf{1^{Ad}}$) with Cp^{4M}H in benzene, synthesis and characterisation of [(^{Ad}BDI)MgPh] ($\mathbf{3^{Ad}}$)

A J. Young's NMR tube was charged with 25 mg $\mathbf{1^{Ad}}$ (0.034 mmol) and 0.6 ml benzene, then 4 µl Cp^{4}H (0.034 mmol) was added. The reaction was heated to 100°C for 8 days and volatiles were removed under vacuum. Dissolution of the crude product in C_6D_6 showed quantitative formation of $\mathbf{3^{Ad}}$, which was isolated as a pale-yellow powder after freeze-drying the C_6D_6 solution. 20 mg yield, 79%.

Quantitative formation of $\mathbf{3^{Ad}}$ was also observed when the same experiment was repeated in the absence of Cp^{4M}H. After 8 days at 100°C, volatiles were removed under vacuum. Analysis of the crude product by ^{1}H NMR spectroscopy after redissolution in benzene-d_6 showed quantitative formation of $\mathbf{3^{Ad}}$ as the only product.

^{1}H NMR (400 MHz, C_6D_6) δ 7.27 – 7.19 (m, 2H, *p*-NC$_6$*H*$_3$-*i*Pr$_2$), 7.11 (d, *J* = 7.7 Hz, 4H, *m*-NC$_6$*H*$_3$-*i*Pr$_2$), 7.05 (m, 3H, *m*,*p*-MgC$_6$*H*$_5$), 6.17 (m, 2H, *o*-MgC$_6$*H*$_5$), 5.74 (s, 1H, *H*CC(Ndipp)), 3.42 (hept, *J* = 7.2 Hz, 4H, *H*C(CH$_3$)$_2$), 2.16 (d, *J* = 3.0 Hz, 12H, ^{Ad}CC*H*$_2$CH), 1.86 (br, 6H, ^{Ad}C*H*), 1.62 (dm, unresolved, *J* = 12.5 Hz, 6H, ^{Ad}CHC*H*$_2$CH), 1.54 (dm, unresolved, *J* = 12.4 Hz, 6H, ^{Ad}CHC*H*$_2$CH), 1.33 (d, *J* = 6.8 Hz, 12H, HC(C*H*$_3$)$_2$), 1.22 (d, *J* = 6.8 Hz, 12H, HC(C*H*$_3$)$_2$).

^{13}C NMR (101 MHz, C_6D_6) δ 176.57 (N=*C*), 158.86 (Mg*C*), 145.27 (*ipso*-NC$_6$H$_3$-*i*Pr$_2$), 141.53 (*ortho*-N*C*$_6$H$_3$-*i*Pr$_2$), 140.88 (*ortho*-Mg*C*$_6$H$_5$), 126.51 (*meta*-Mg*C*$_6$H$_5$), 126.11 (*para*-Mg*C*$_6$H$_5$), 125.58 (*para*-N*C*$_6$H$_3$-*i*Pr$_2$), 124.19 (*meta*-N*C*$_6$H$_3$-*i*Pr$_2$), 93.79 (H*C*C(Ndipp)), 47.91 (NC^{Ad}*C*), 43.72 (^{Ad}C*C*H$_2$CH), 36.98 (^{Ad}CH*C*H$_2$CH), 29.51 (^{Ad}C*H*), 28.53 (H*C*(CH$_3$)$_2$), 25.79 (HC(*C*H$_3$)$_2$), 23.13 (HC(*C*H$_3$)$_2$).

Analysis calculated for $C_{53}H_{70}MgN_2$: C, 83.82; H, 9.29; N, 3.69%. Found: C, 83.10; H, 10.02; N, 3.42%.

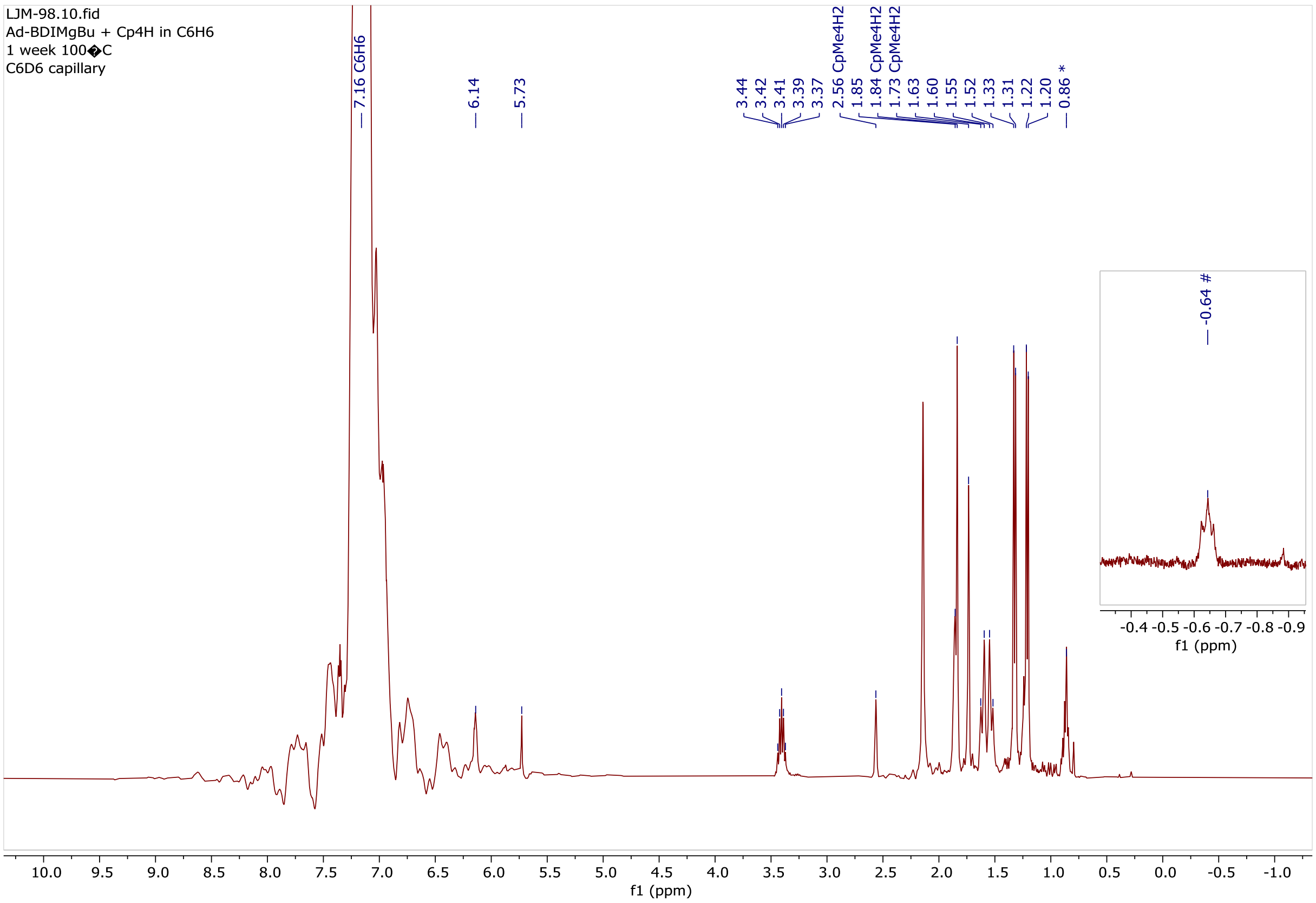

Figure S18: *In situ* ^{1}H NMR spectrum (400 MHz, C_6H_6, locked to C_6D_6 capillary) of the attempted reaction of **1**$^{\mathbf{Ad}}$ with Cp^{4M}H in benzene after 1 week at 100°C, indicating near-quantitative formation of **3**$^{\mathbf{Ad}}$. **n*-butane. #Expanded region showing residual **1**$^{\mathbf{Ad}}$. Resonances corresponding to unreacted Cp^{4M}H are annotated.

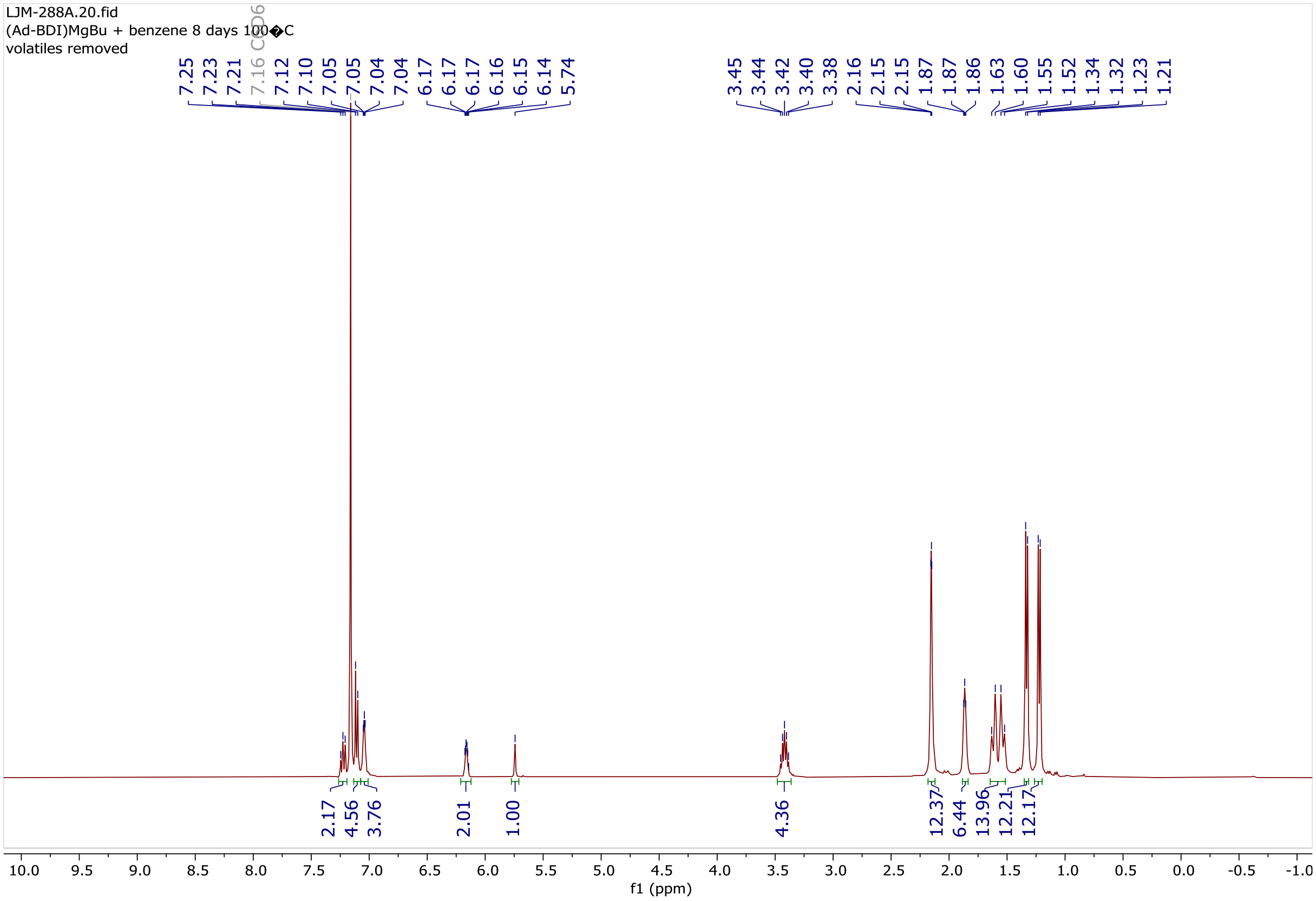

Figure S19: ^{1}H NMR spectrum (400 MHz, C_6D_6) of the crude product from the reaction of **1^{Ad}** with benzene (in the absence of Cp^{4M}) after 8 days at 100°C and removal of volatiles under vacuum.

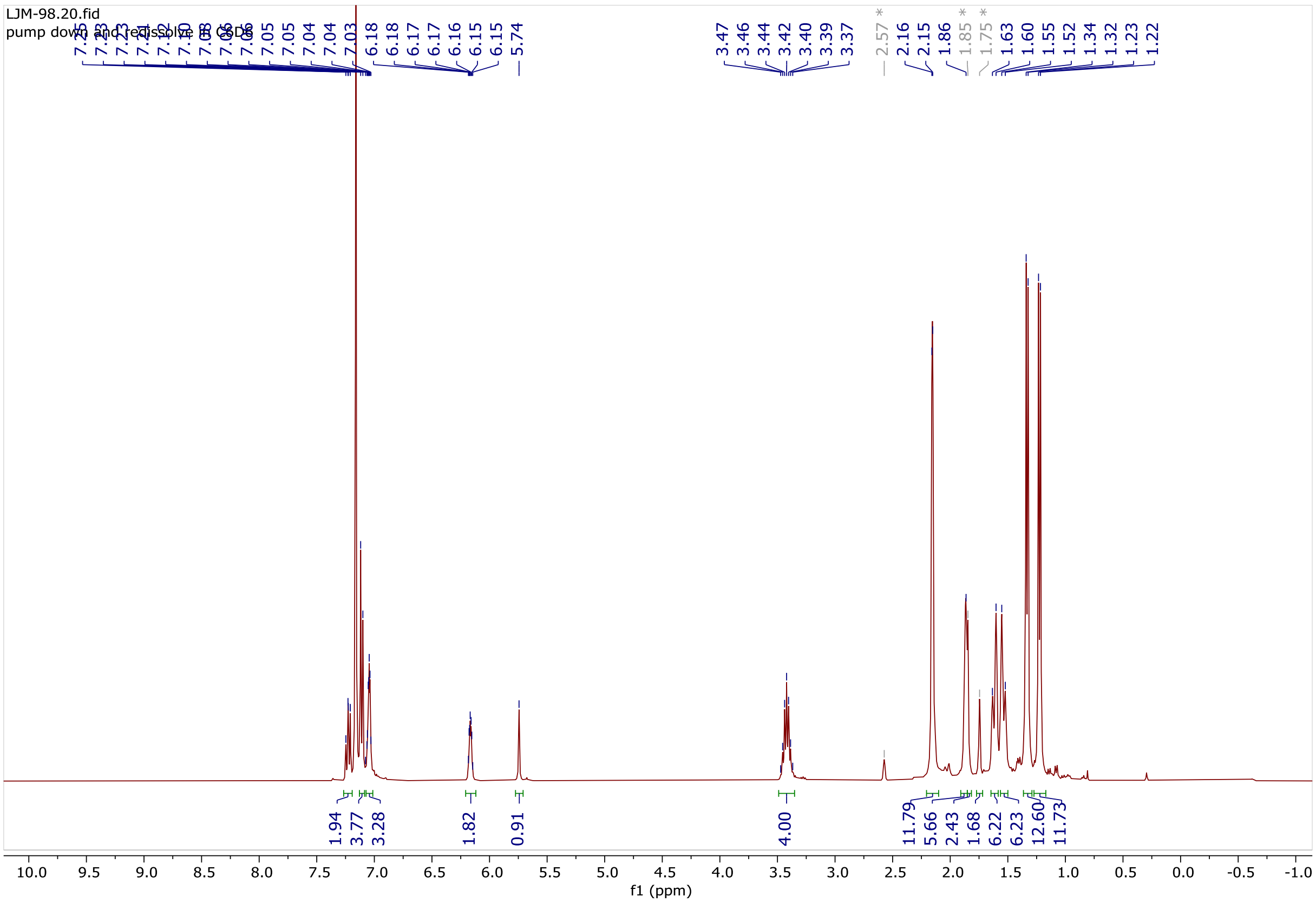

Figure S20: ^{1}H NMR (400 MHz, C_6D_6) of **3^{Ad}**. *residual $Cp^{4}H$.

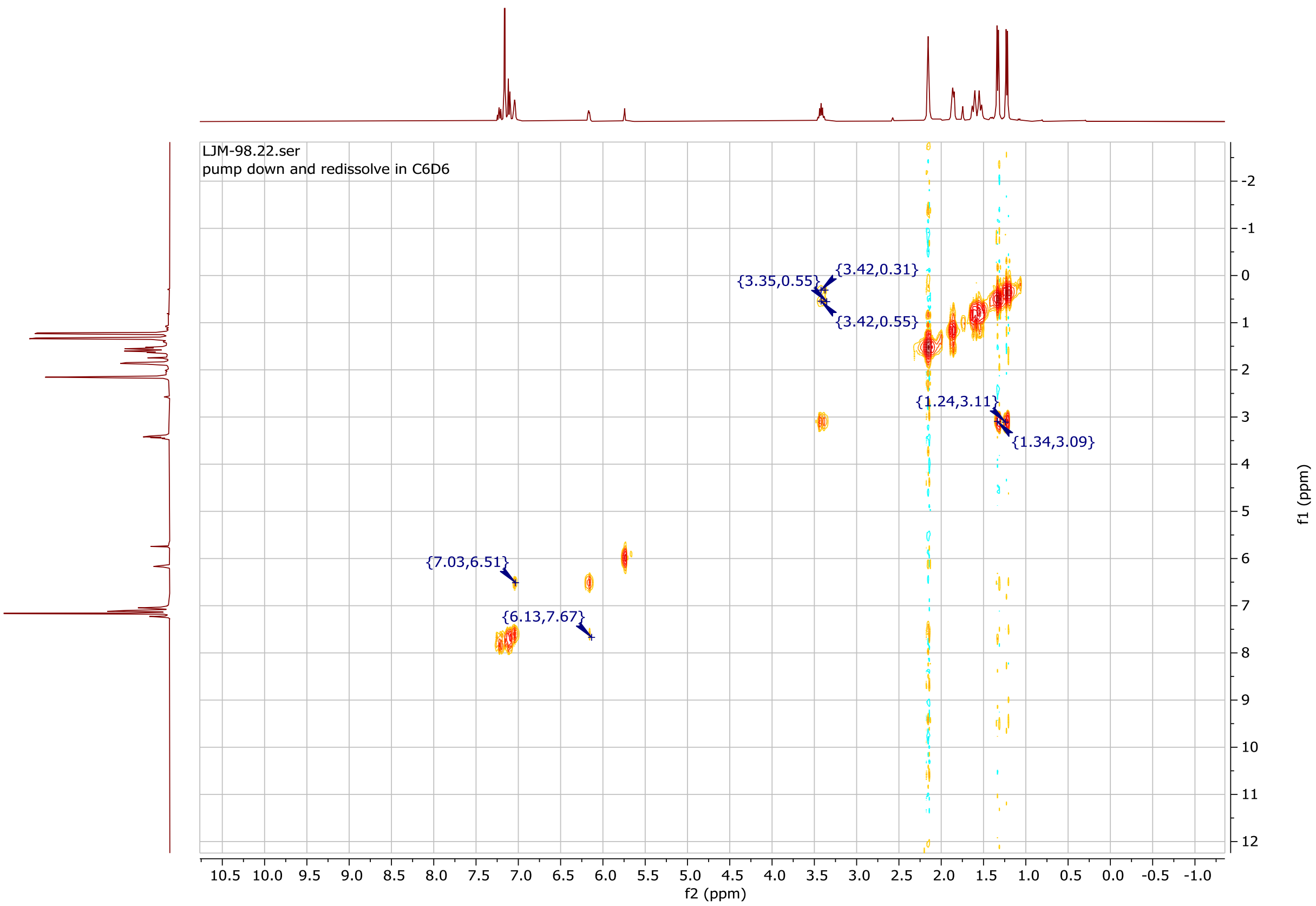

Figure S21: ^{1}H-^{1}H COSY NMR (400 MHz, C_6D_6) of **3**^{Ad}.

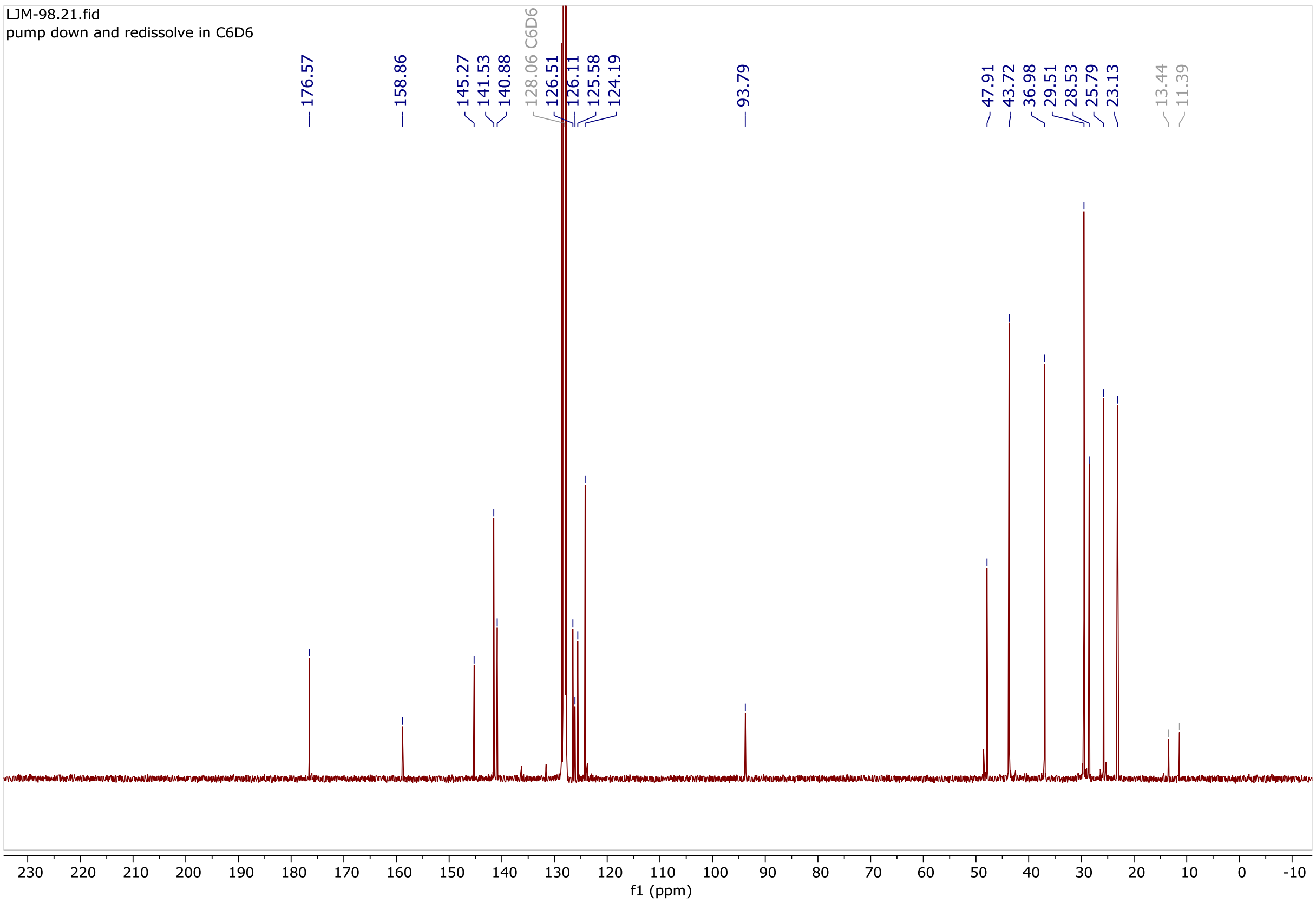

Figure S22: $^{13}C\{^{1}H\}$ NMR (101 MHz, C_6D_6) of **3**^{Ad}.

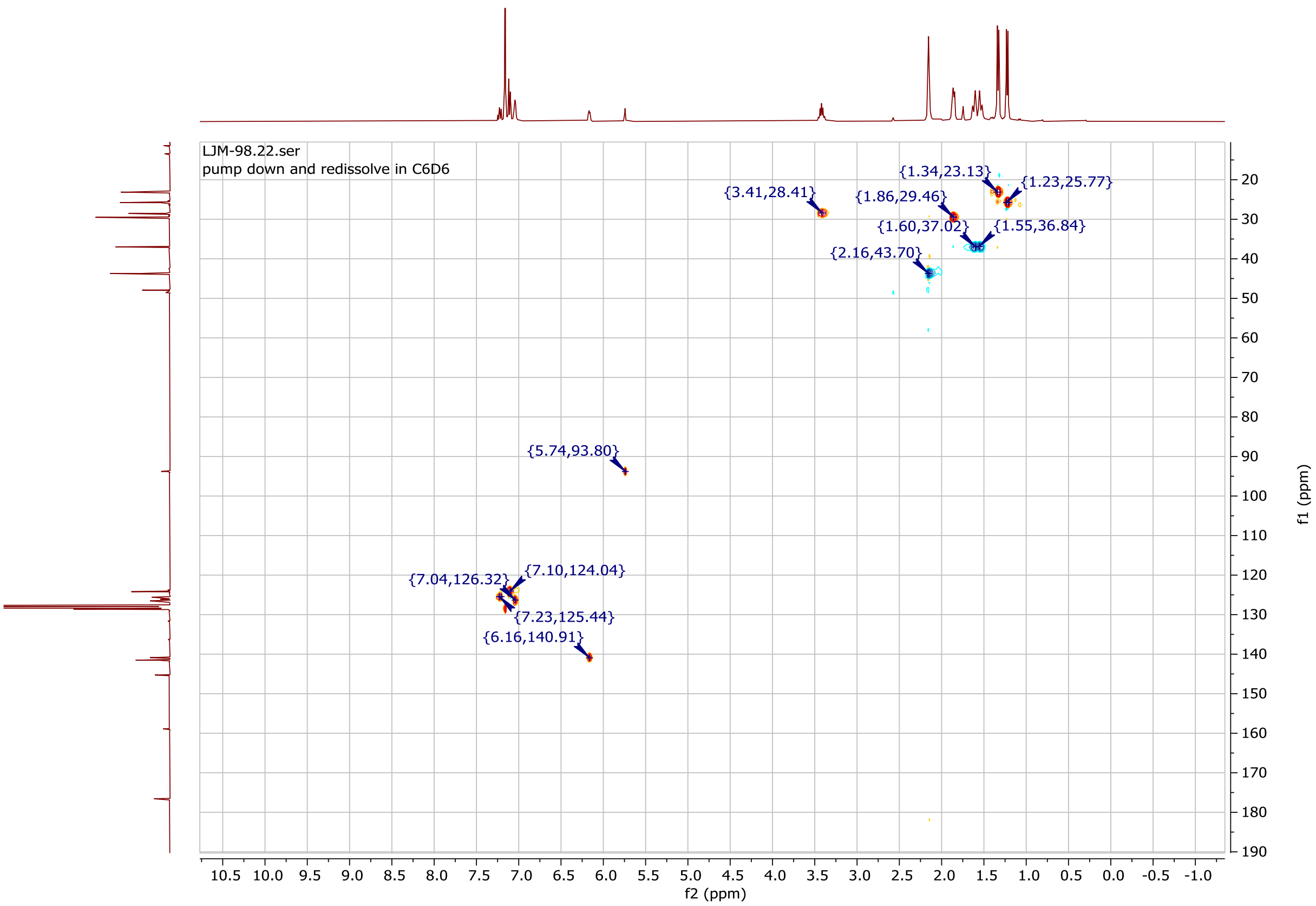

Figure S23: ^{13}C-^{1}H HSQC NMR (101 MHz, C_6D_6) of **3^{Ad}**.

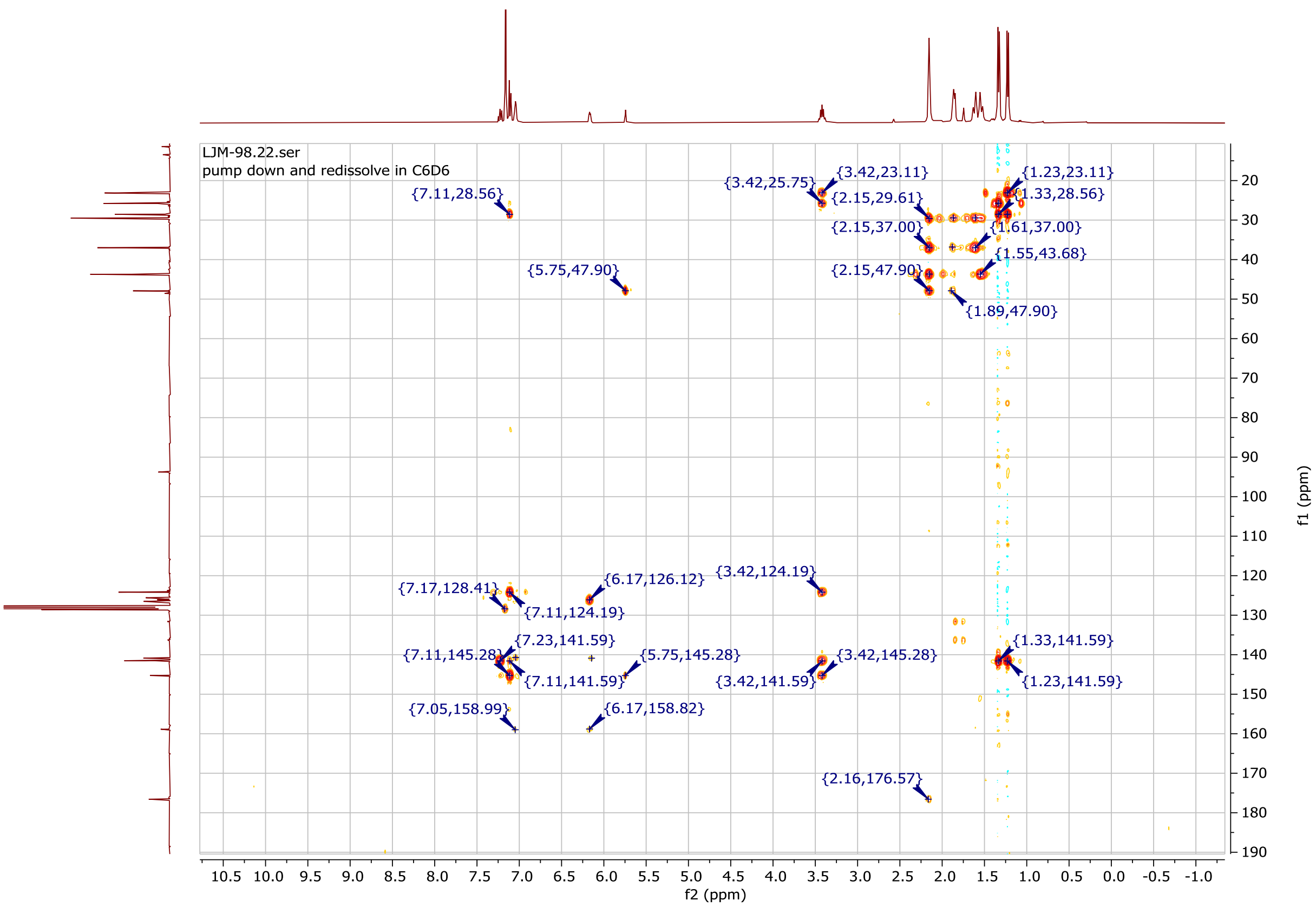

Figure S24: ^{13}C-^{1}H HMBC NMR (101 MHz, C_6D_6) of **3^{Ad}**.

2.6 Reaction of [(^{Ad}BDI)Mg^{n}Bu] (**1^{Ad}**) with a 1:1 mixture of C_6D_6 and C_6H_6

A J. Young's NMR tube was charged with 18 mg **1^{Ad}** (0.024 mmol), which was dissolved in 0.6 ml 1:1 v/v C_6D_6/C_6H_6. The reaction was heated at 100°C for 6 days, then all volatiles were removed under vacuum. Analysis of the crude product mixture as a solution in C_6D_6 indicated the formation of a 1:1 mixture of **1^{Ad}** and **1^{Ad}-*d*$_5$** according to relative integrals of the γ-C<u>*H*</u> and *ortho*-C<u>*H*</u> resonances of the BDI- and phenyl ligands respectively. Subsequent experiments demonstrating the exchange of **3^{Ad}** with C_6D_6 to give **3^{Ad}-*d*$_5$** indicated that the isotopomer distribution obtained from the reaction in C_6D_6/C_6H_6 mixtures bears no indication on any kinetic isotope effect.

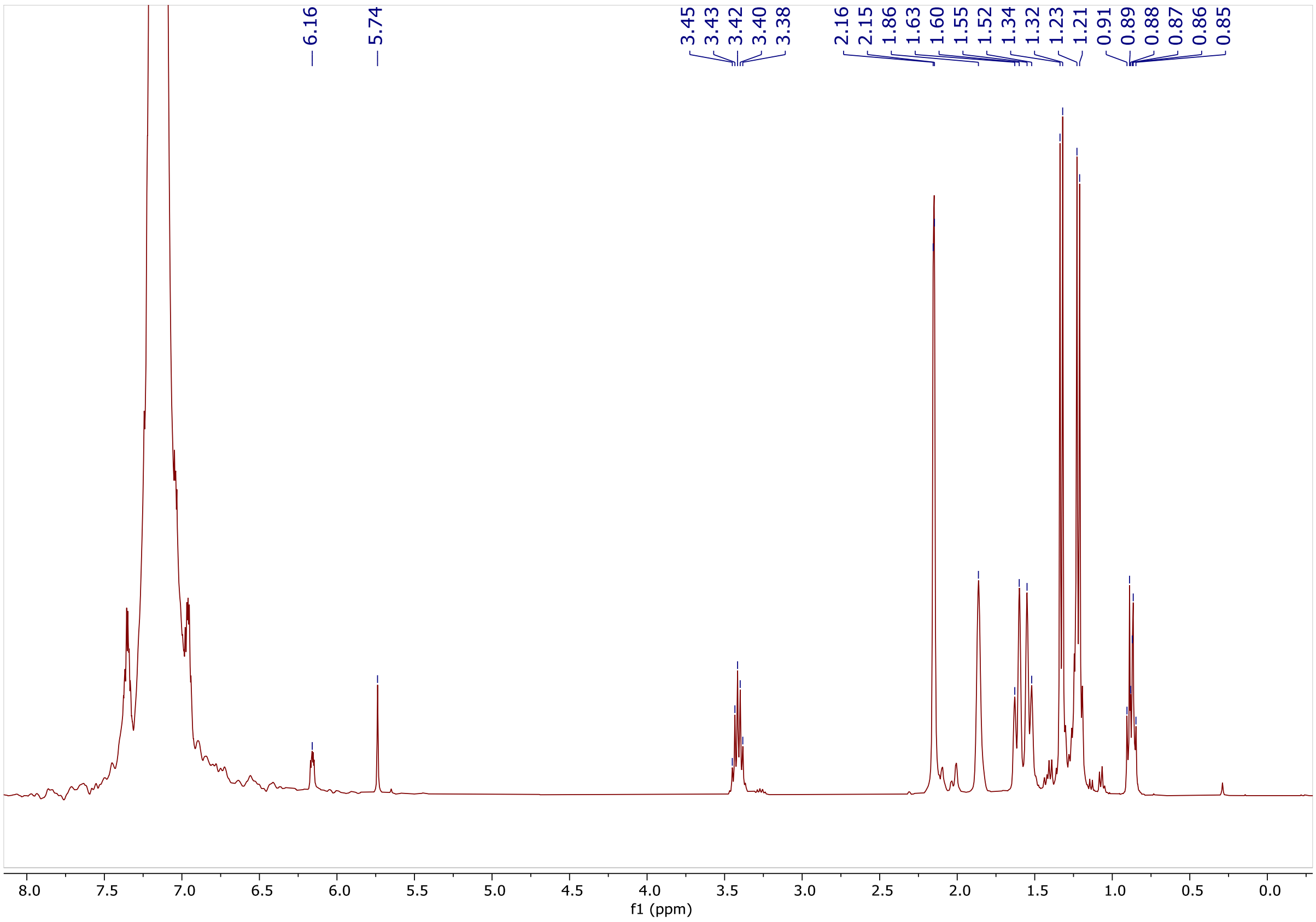

Figure S25: ^{1}H NMR spectrum (400 MHz, C_6H_6) of the reaction mixture after heating at 100°C for 6 days.

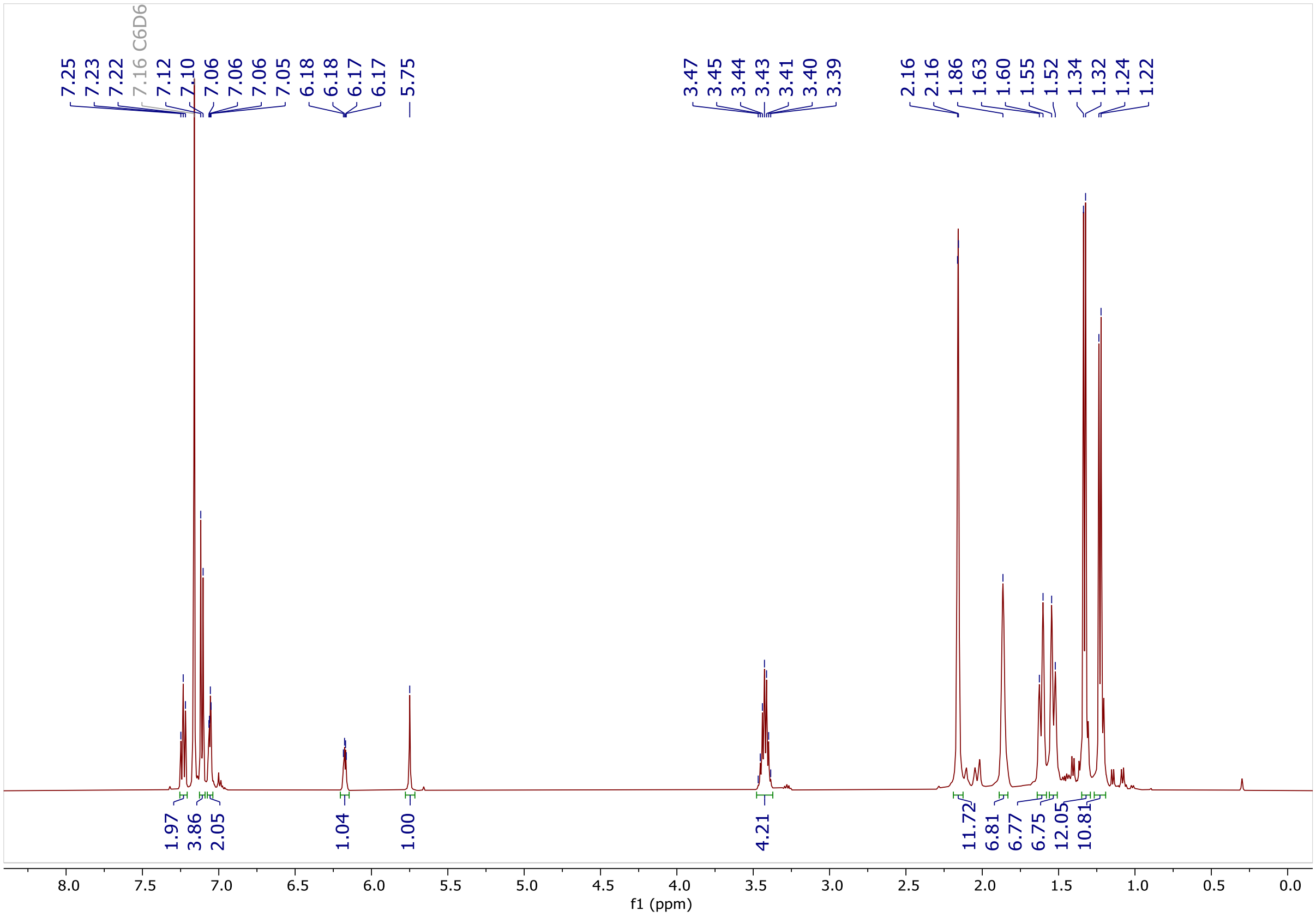

Figure S26: ^1H NMR spectrum (400 MHz, C_6D_6) of the product after removing volatiles under vacuum.

2.7 Exchange reaction of [(^{Ad}BDI)MgPh] (3^{Ad}) with benzene-d_6 to form [(^{Ad}BDI)Mg(C_6D_5)] (3^{Ad}-d_5)

A J. Young's NMR tube was charged with 13 mg [(^{Ad}BDI)Mg^{n}Bu] (**1^{Ad}**, 0.018 mmol) and 0.6 ml benzene, then heated to 100°C for 6 days. Analysis of the reaction mixture by *in situ* ^1H NMR spectroscopy indicated quantitative consumption of the starting complex. Volatiles were removed under vacuum to provide **3^{Ad}**, which was then dissolved in 0.6 ml benzene-d_6 and heated at 100°C for 3 days. **3^{Ad}-d_5** was formed in >95% yield, as determined by *in situ* NMR analysis.

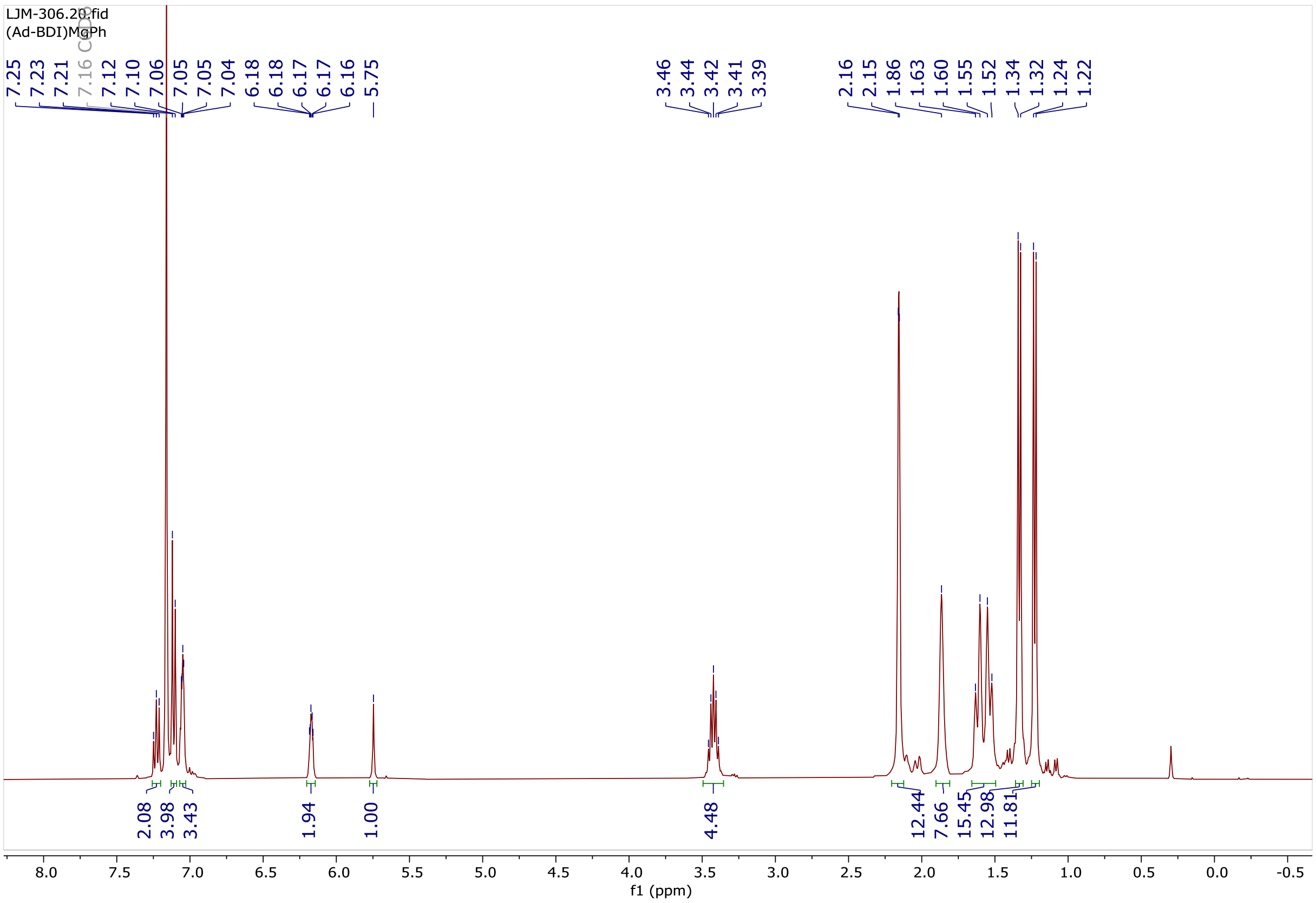

Figure S27: ^{1}H NMR spectrum (400 MHz, C_6D_6) of **3^{Ad}** after dissolution in benzene-*d*$_6$.

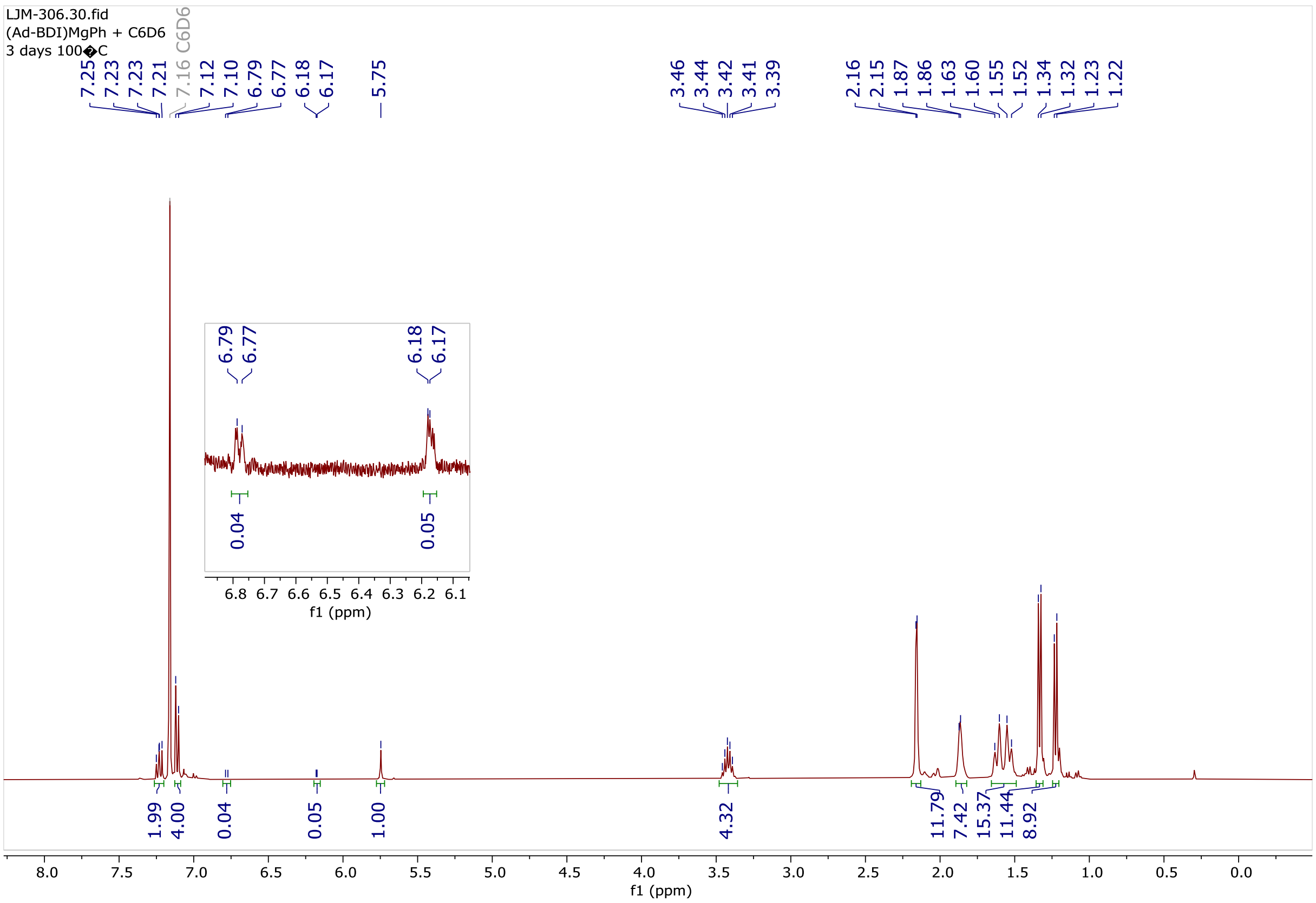

Figure S28: ^{1}H NMR spectrum (400 MHz, C_6D_6) after heating **3^{Ad}** in benzene-*d*$_6$ at 100°C for 3 days, showing formation of **3^{Ad}-*d*$_5$**. Expanded region showing residual **3^{Ad}**.

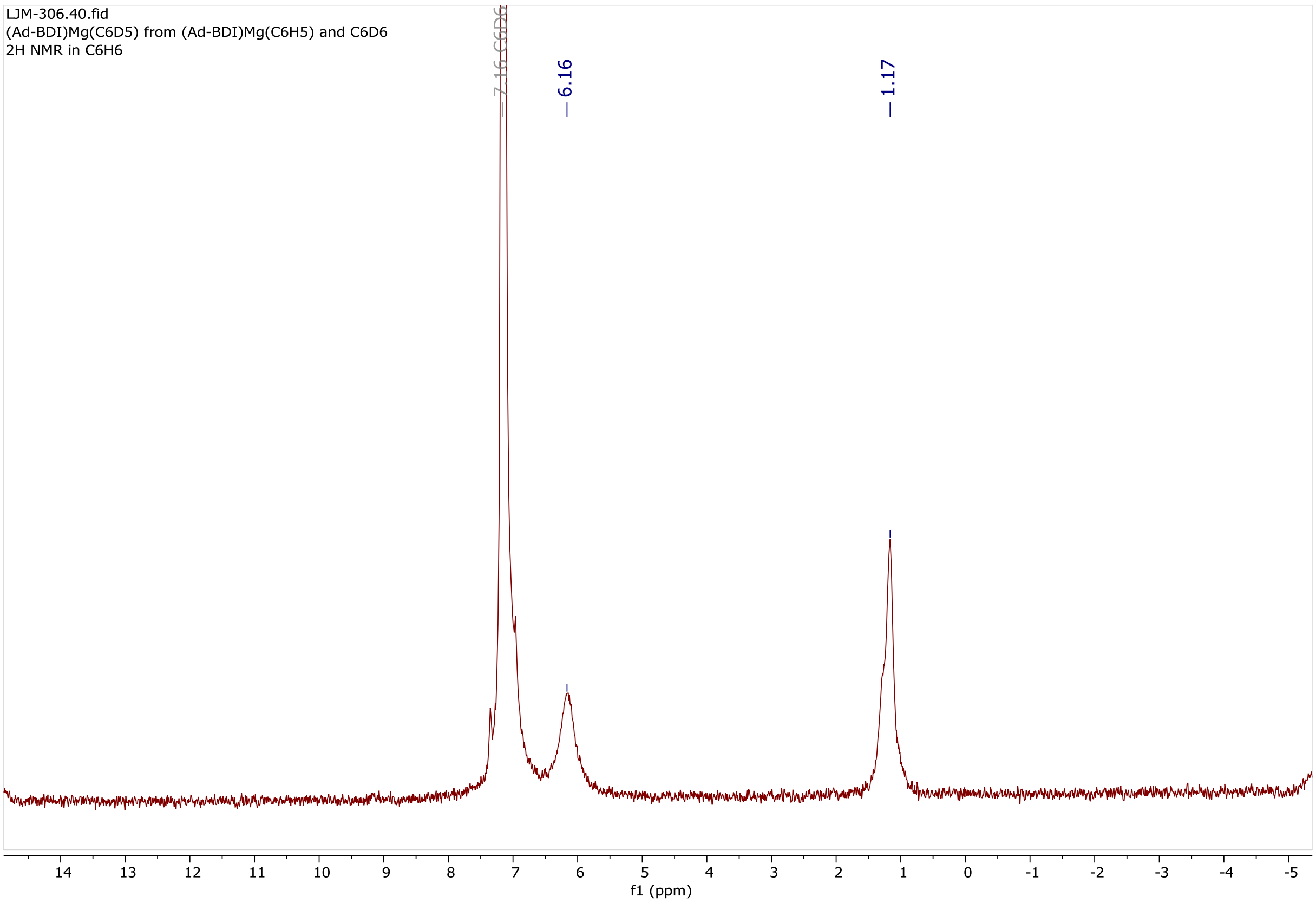

Figure S29: ^{2}H NMR spectrum (61 MHz, C_6H_6) after heating **3^{Ad}** in benzene-d_6 at 100°C for 3 days, removal of volatiles under vacuum, and dissolution in benzene, showing formation of **3^{Ad}-*d*$_5$**. Partial deuteration of the *iso*-propyl methyl groups is also apparent, this is consistent with slightly low integral of the corresponding resonances in the ^{1}H NMR spectrum.

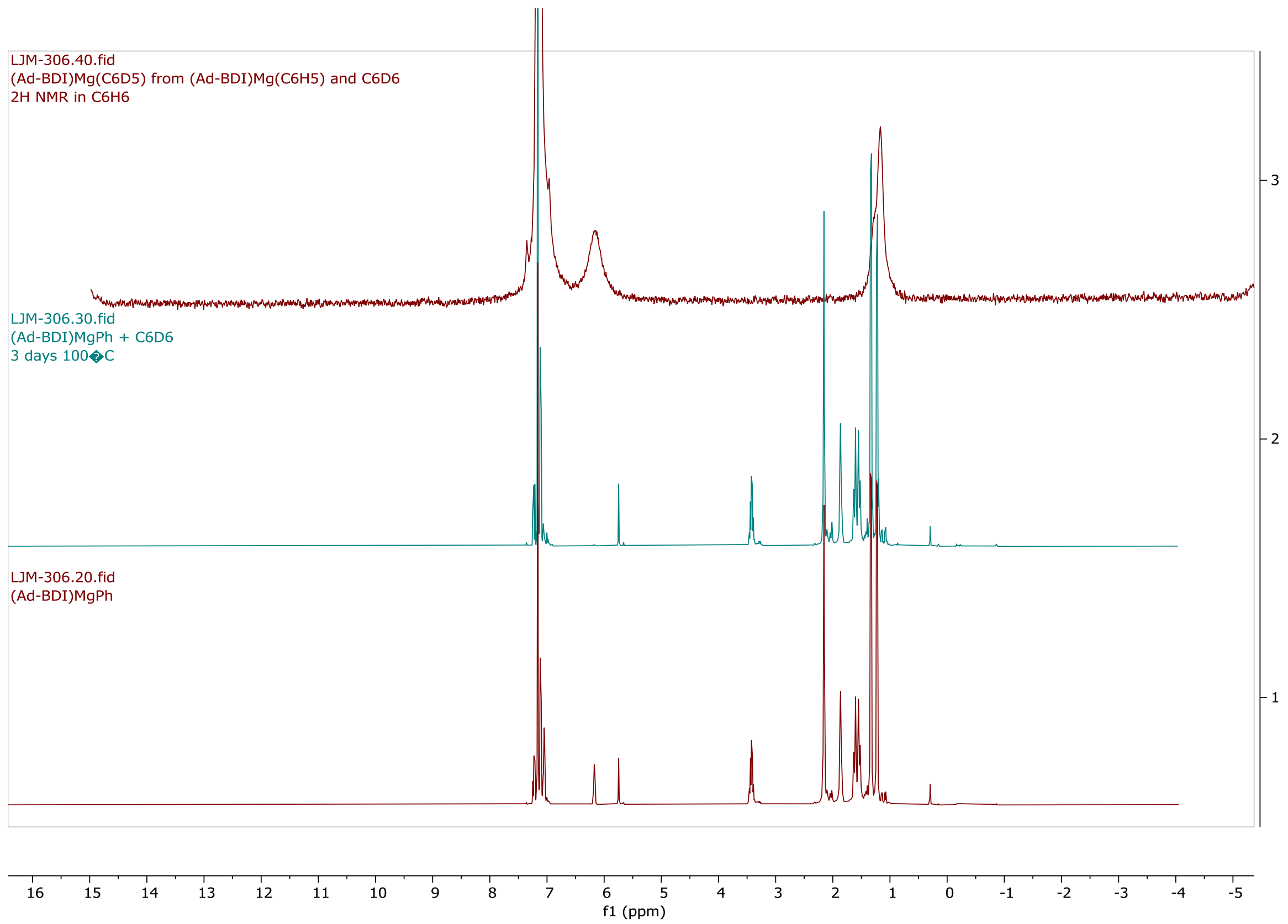

Figure S30: Stacked ^{2}H (top, 61 MHz, C_6H_6) and ^{1}H (400 MHz, C_6D_6) NMR spectra before (bottom) and after (top, middle) heating **3^{Ad}** in benzene-d_6 at 100°C for 3 days.

2.8 Reaction of [(^{Ad}BDI)Mg^{n}Bu] (1^{Ad}) with $PhSiH_3$

Phenylsilane (3.5 µl, 0.028 mmol) was added to a J. Young's NMR tube containing at 0.6 ml methylcyclohexane solution of **1^{Ad}** (24 mg, 0.032 mmol). The sealed tube was heated at 80°C for 16 hours, but little reaction was observed. Subsequent heating at 100°C for 3 days resulted in consumption of the starting complex and formation of mixture of species. Volatiles were removed under vacuum at room temperature, then the product mixture was dissolved in benzene-d_6. The most well-defined and only identifiable BDI-containing species was compound **3^{Ad}**. *n*-Butyl(phenyl)silane was readily identified in the ^{1}H and ^{29}Si-^{1}H HMBC NMR spectra, as well as a further Si*H* resonance at δ_H = 3.66 ppm, which correlates to a ^{29}Si resonance at δ_{Si} = –72 ppm in the ^{29}Si-^{1}H HMBC NMR spectra. Although this species remains unidentified, it might plausibly be a magnesium silanide complex [(^{Ad}BDI)Mg(SiH_3)] or [(^{Ad}BDI)Mg($SiPhH_2$)].

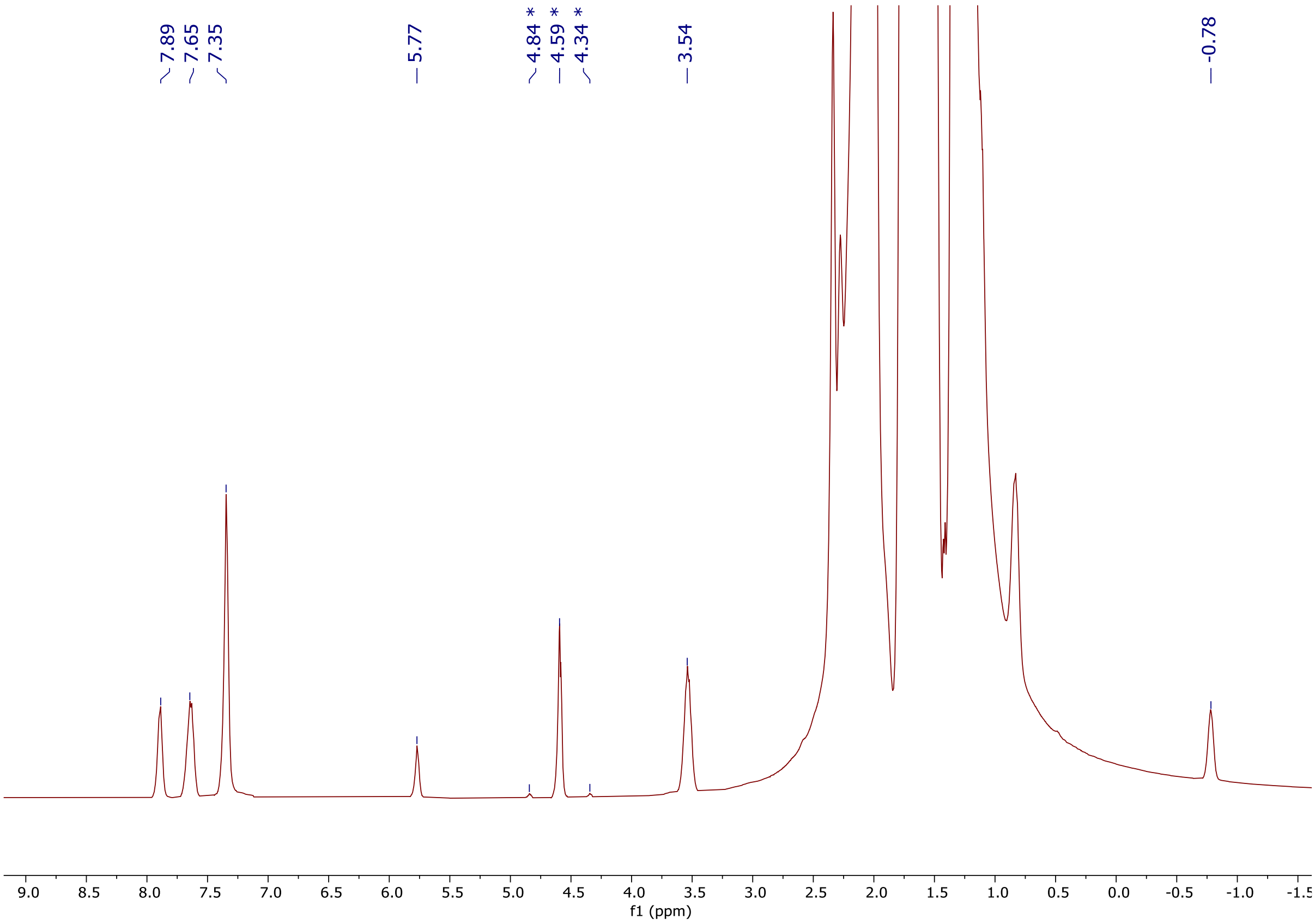

Figure S31: ^{1}H NMR spectrum (400 MHz, methylcyclohexane) of **1^{Ad}** and phenylsilane(*).

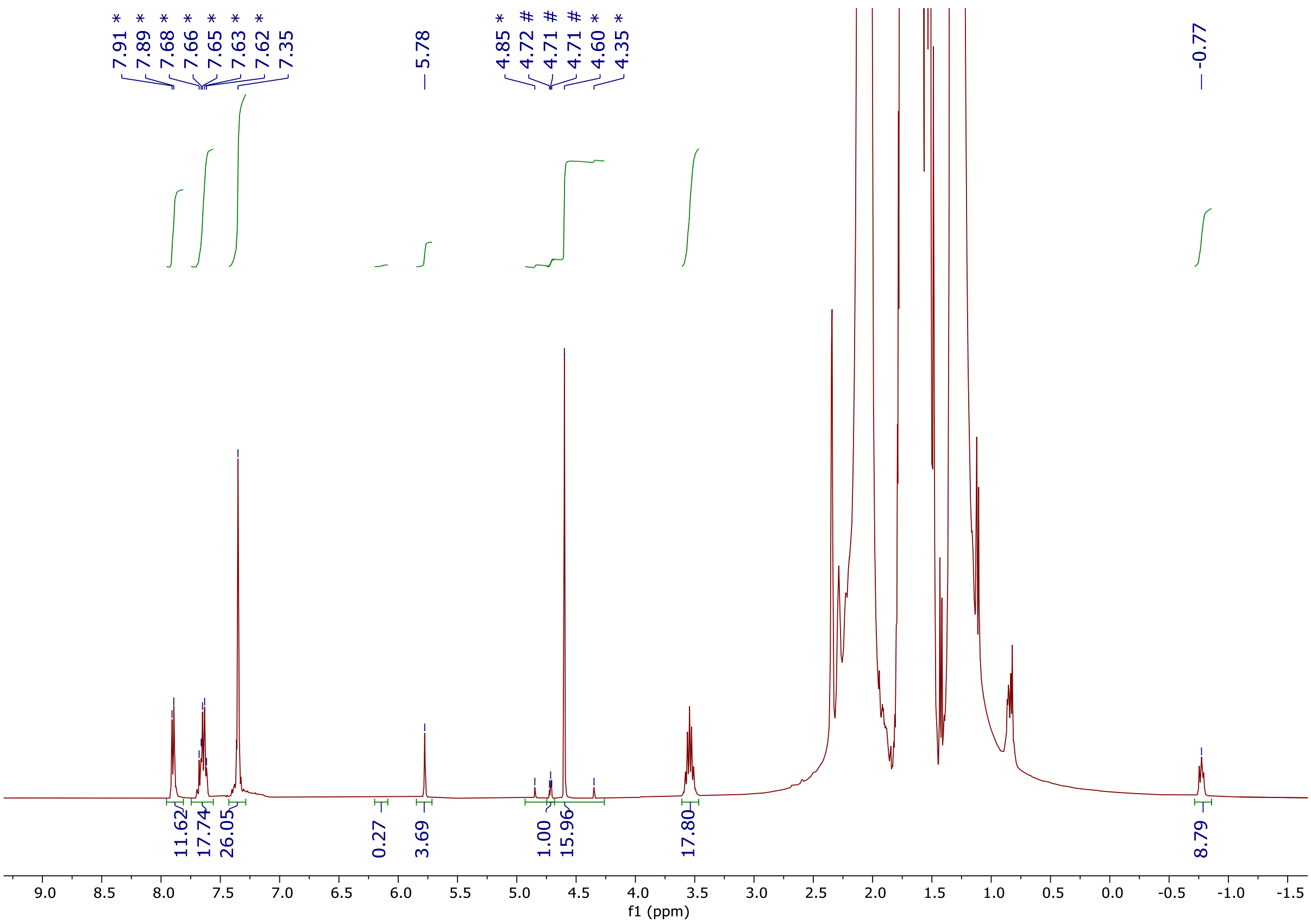

Figure S32: ^{1}H NMR spectrum (400 MHz, methylcyclohexane) after 20 hours at 80°C. (*) phenylsilane. (#) *n*-butyl(phenyl)silane.

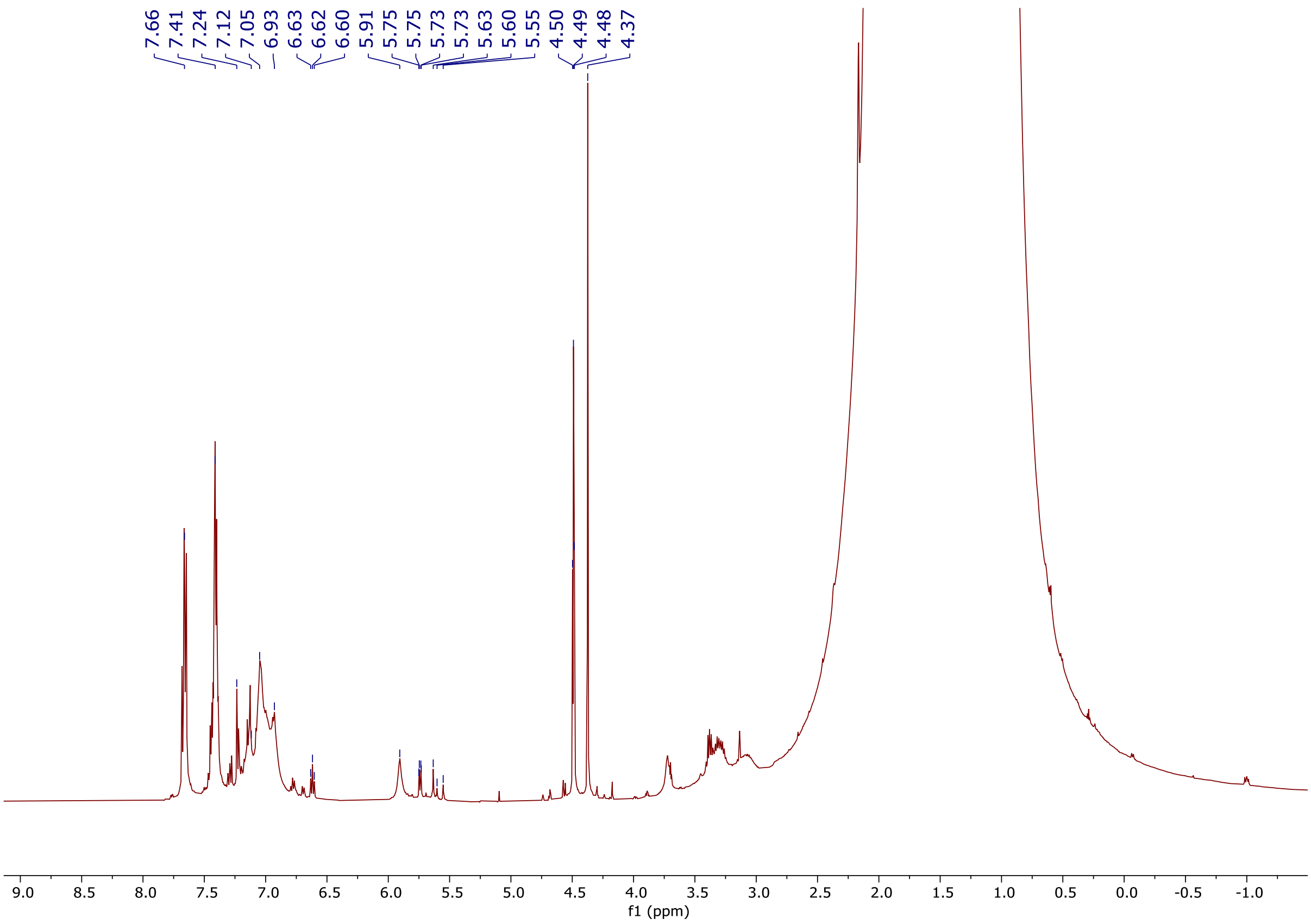

Figure S33: ^{1}H NMR spectrum (500 MHz, methylcyclohexane) after 3 days at 100°C. (*) phenylsilane. (#) *n*-butyl(phenyl)silane.

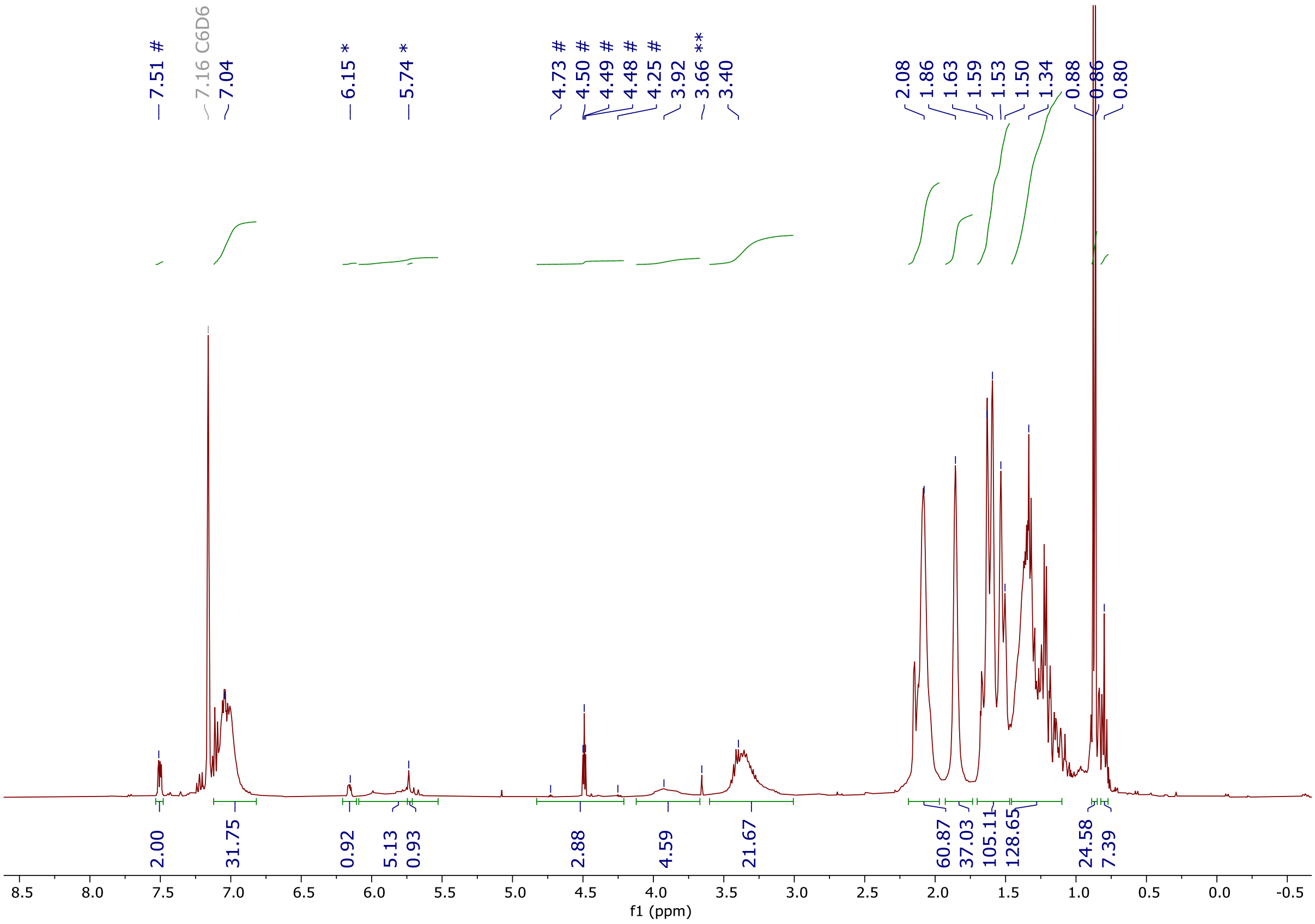

Figure S34: ^{1}H NMR spectrum (400 MHz, C_6D_6) after removal of volatiles, showing formation of (*) **3[Ad]**, (#) *n*-butyl(phenyl)silane, (**) possible magnesium silanide.

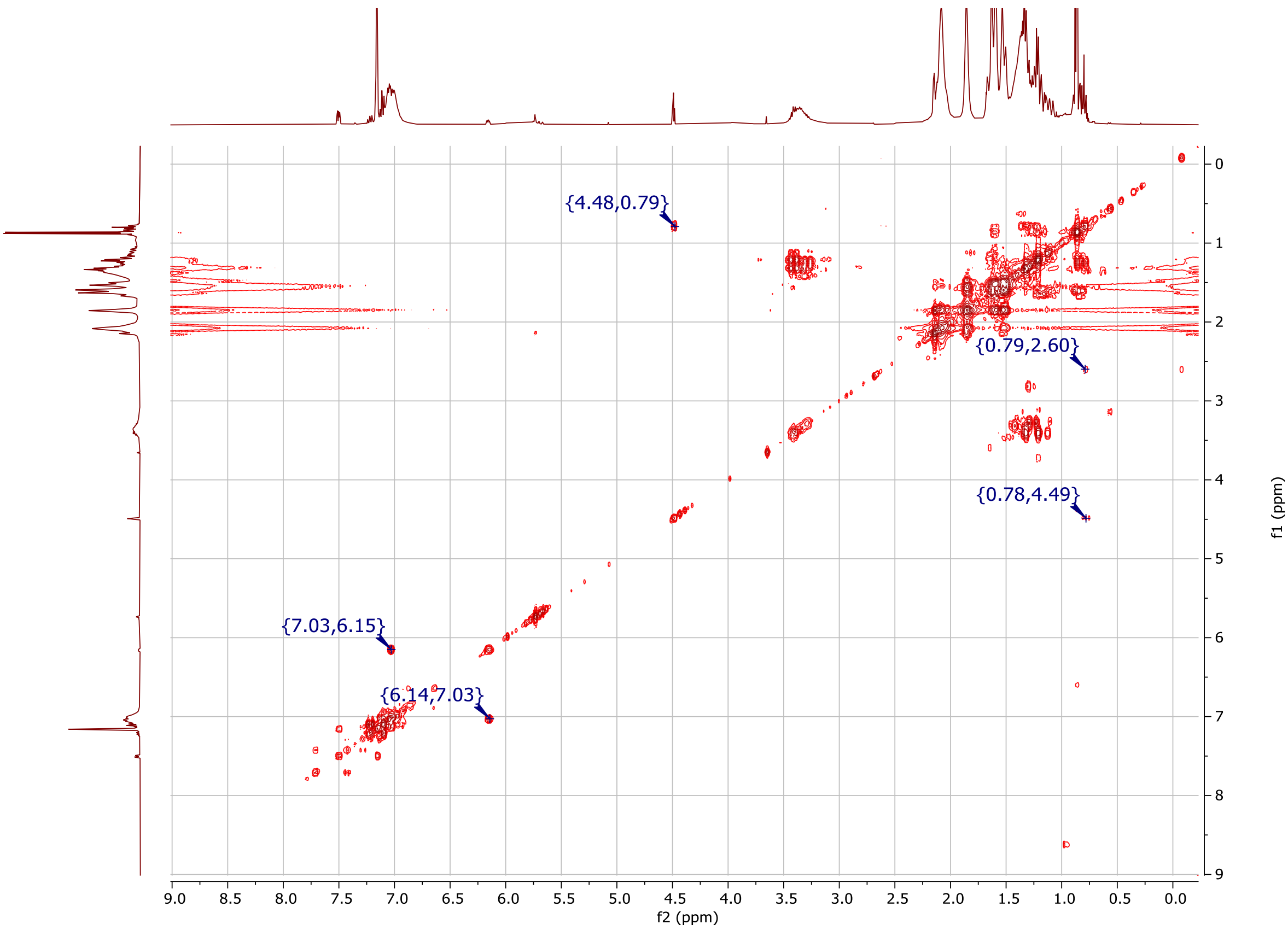

Figure S35: ^{1}H-^{1}H COSY spectrum (C_6D_6).

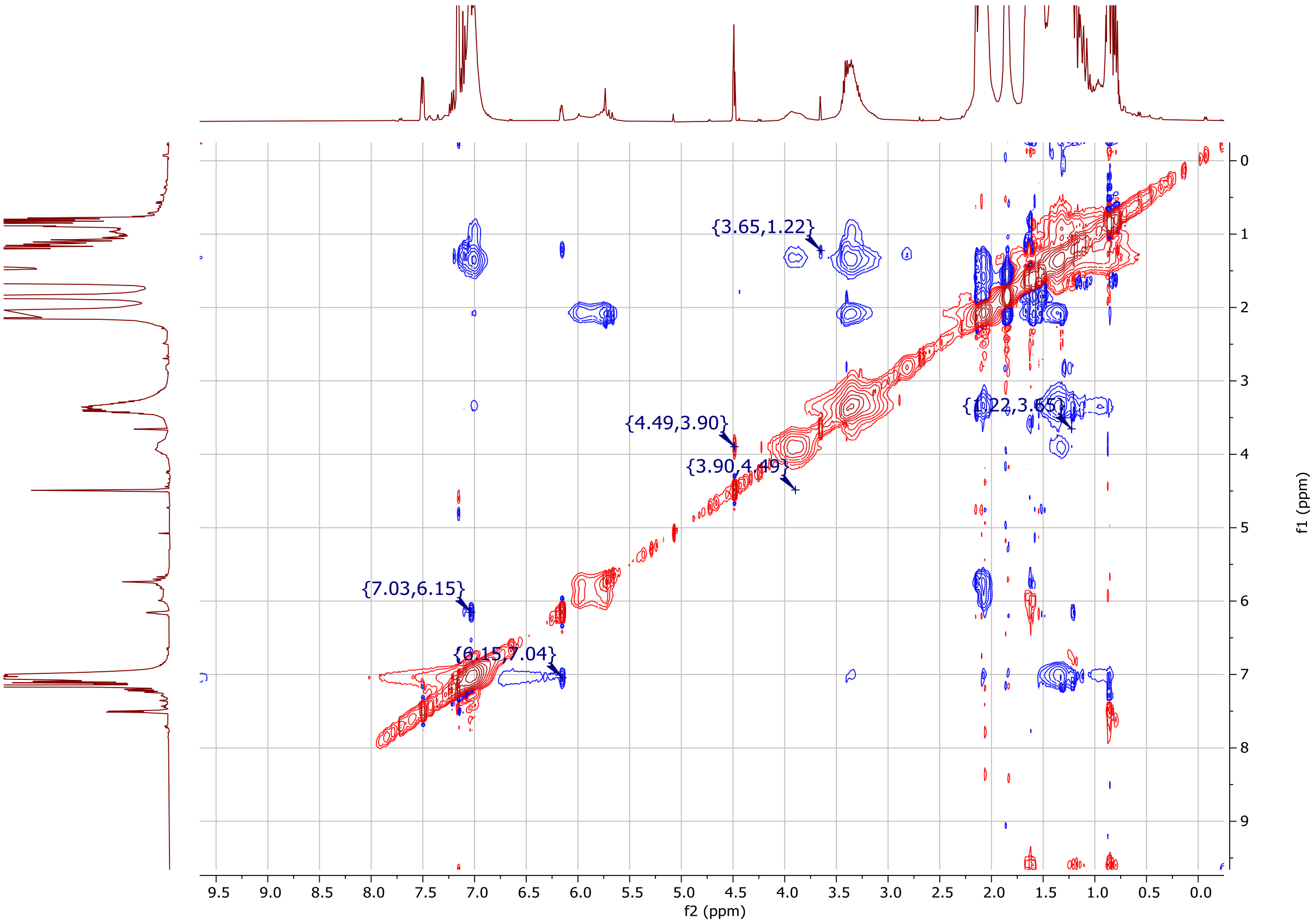

Figure S36: ^{1}H-^{1}H NOESY spectrum (C_6D_6).

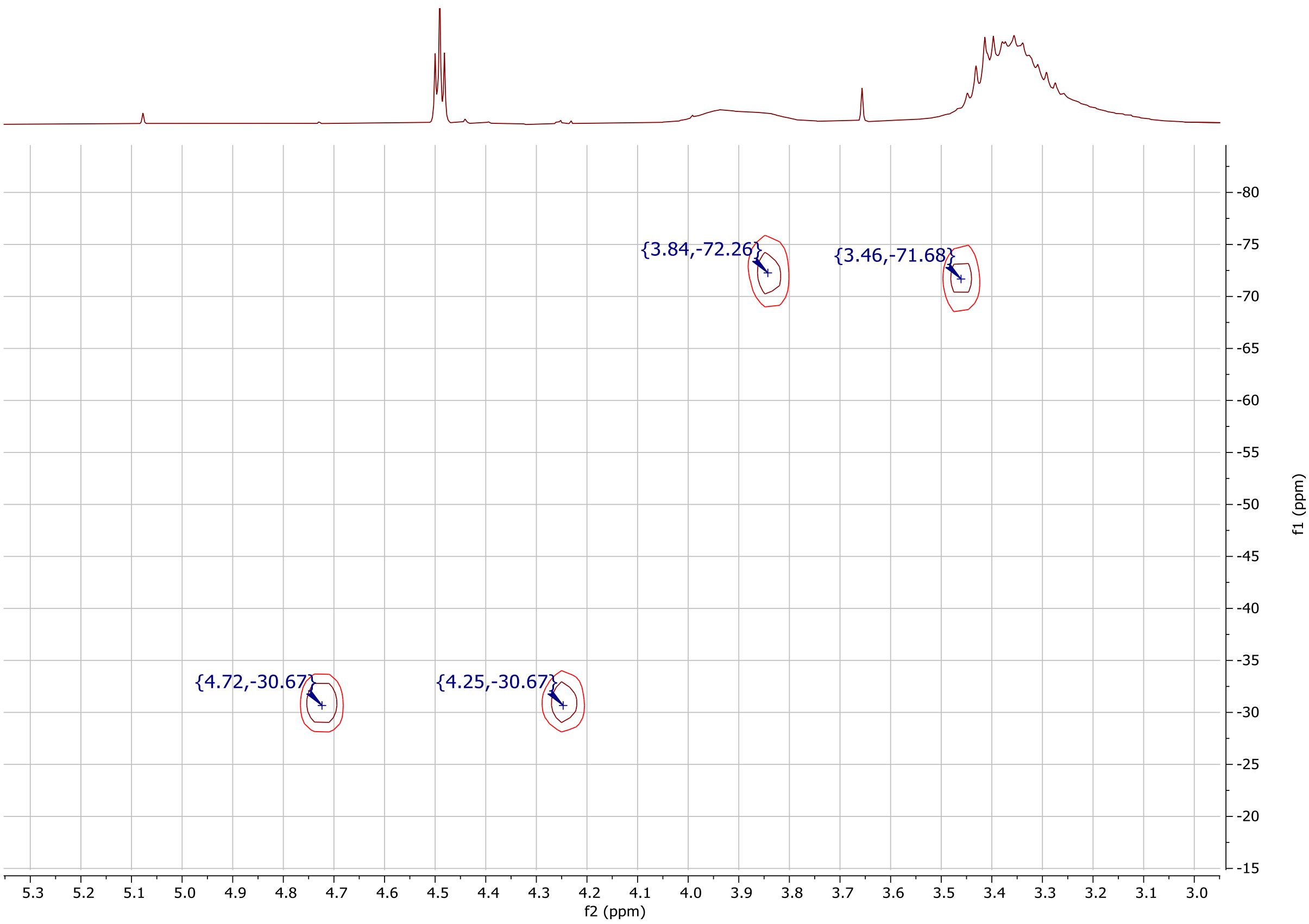

Figure S37: ^{29}Si-^{1}H HMBC spectrum (C_6D_6) set to 190 Hz coupling.

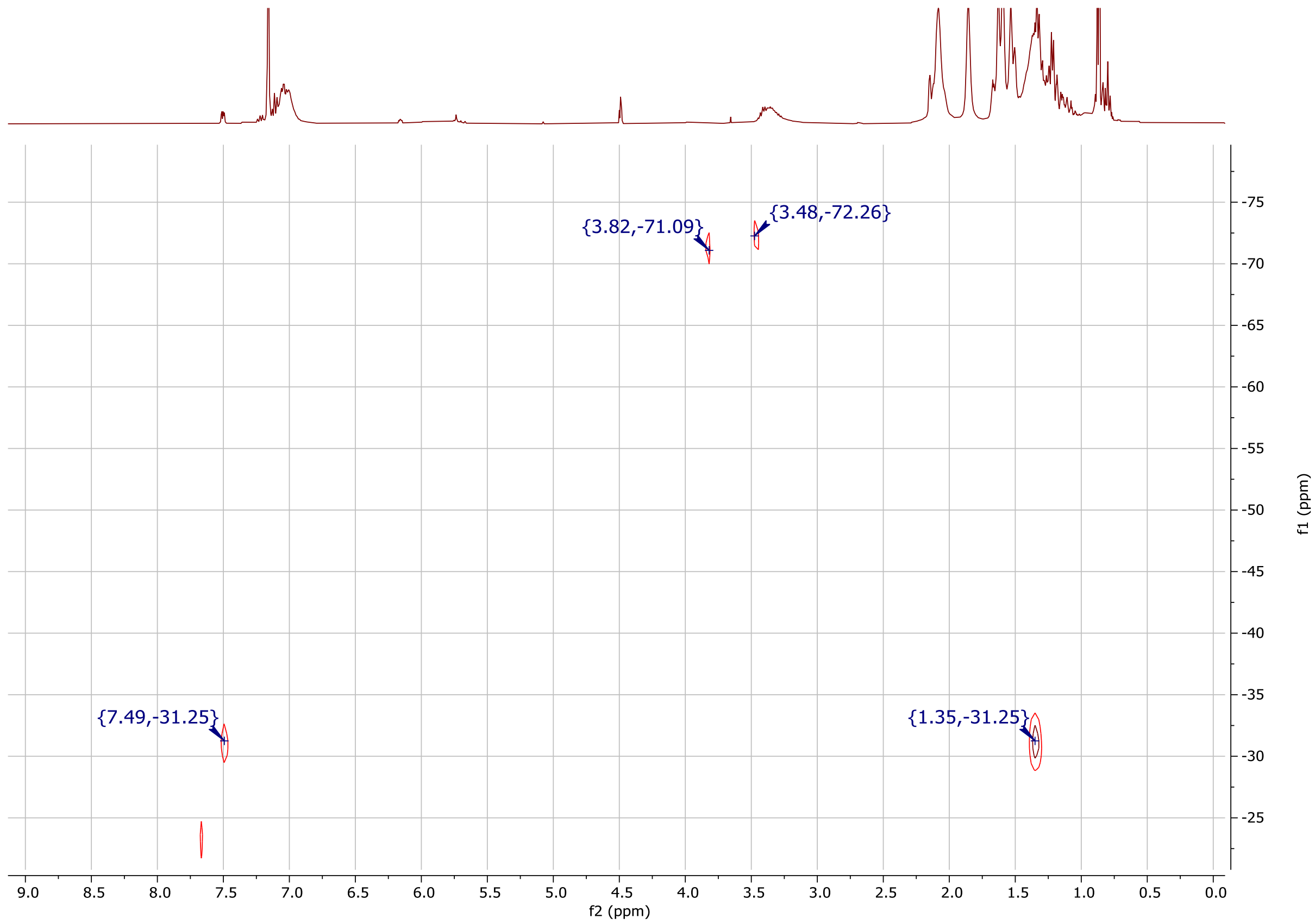

Figure S38: ^{29}Si-^{1}H HMBC spectrum (C_6D_6) set to 7 Hz coupling.

2.9 Reaction of [(^{Ad}BDI)Mg^{n}Bu] (**1^{Ad}**) with H_2

A J. Young's NMR tube was charged with 15 mg **1^{Ad}** (0.02 mmol) and 0.6 ml methylcyclohexane. The pale-yellow solution was freeze-pump-thaw degassed, then filled with 1 bar H_2. After heating for 18 h. at 60°C, no reaction was observed. Further heating at 100°C for 1 week showed unreacted **1^{Ad}** alongside an unidentified BDI-containing species in an approximate integral ratio of 0.55:0.45 based on the singlet γ-methine signal of the BDI ligand backbone. A further singlet at δ 5.15 ppm integrated with the same relative intensity as the γ-methine signal of the unidentified product and might be consistent with formation of magnesium hydride [(^{Ad}BDI)MgH] (**2^{Mg,Ad}**, or its dimer). Efforts to isolate and characterise the product were unsuccessful.

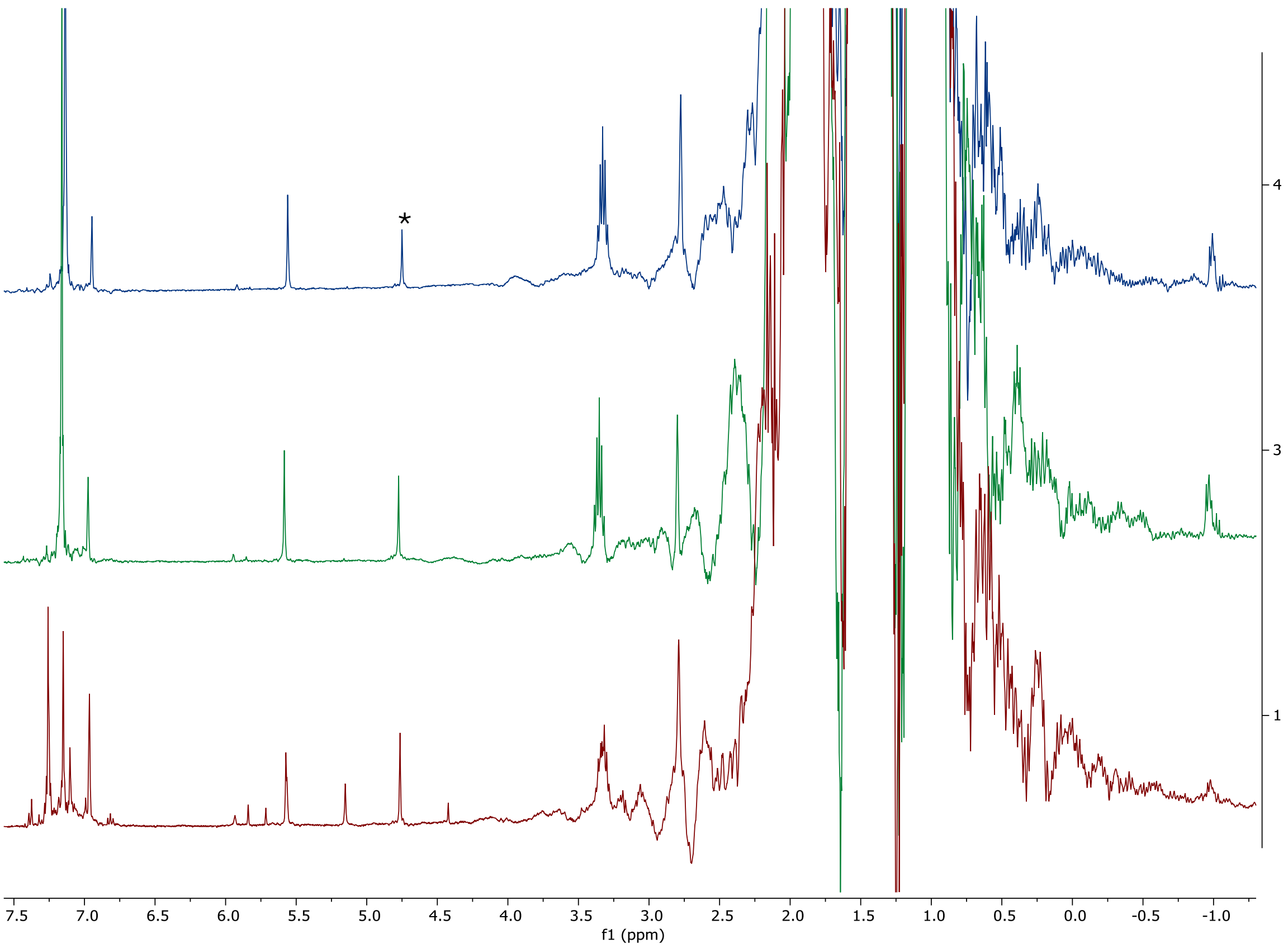

Figure S39: ^{1}H NMR spectra (400 MHz, methylcyclohexane) of the attempted reaction of **1^{Ad}** with H_2 (*). Top: initial reaction mixture; centre: after 18 h. at 60°C; bottom: after 1 week at 100°C.

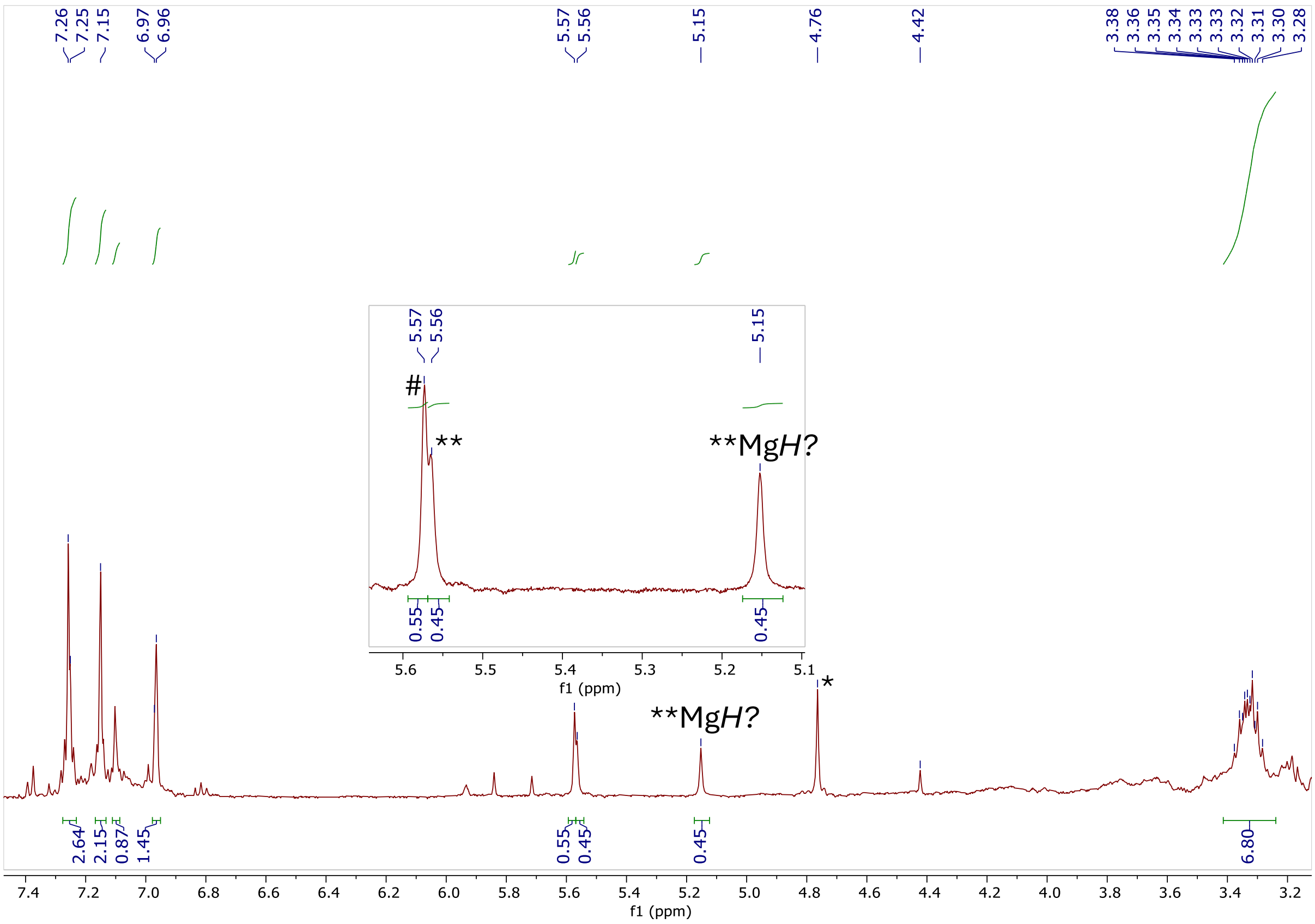

Figure S40: Expanded ^{1}H NMR spectrum (400 MHz, methylcyclohexane) of the reaction of **1^{Ad}** (#) with H_2 (*) after 1 week at 100°C highlighting the formation of a new BDI-containing species (**), which may be [(^{Ad}BDI)MgH] or its dimer.

2.10 Reaction of $[(^{Me}BDI)Mg^{n}Bu]_2$ ($[\mathbf{1^{Me}}]_2$) and $[(^{Me}BDI)Mg(\mu\text{-}H)]_2$ ($[\mathbf{2^{Mg,Me}}]_2$) with benzene-d_6

Reaction of $[\mathbf{1^{Me}}]_2$ with benzene-d_6

A J. Young's tube was charged with $[\mathbf{1^{Me}}]_2$ (10 mg, 0.010 mmol) and benzene-d_6 (0.6 ml). The tube was closed and heated to 120°C in an oil bath for 14 days. Analysis by ^{1}H NMR spectroscopy indicated conversion of most of the starting complex to a mixture which contained $[(^{Me}BDI)MgPh]$ ($\mathbf{3^{Me}}$)[6] as the major product and $[(^{Me}BDI)Mg(\mu\text{-}H)]_2$ [5] as a minor product, besides traces of several other species. 1-butene was also identified in the reaction mixture by ^{1}H NMR spectroscopy. Removal of volatiles and recrystallisation from Et_2O/THF mixture provided colourless block-shaped single crystals which were identified as the previously reported magnesium hydride complex $[(^{Me}BDI)Mg(\mu\text{-}H)\cdot thf]_2$ by comparison to the published unit cell.[5]

Reaction of $[\mathbf{2^{Mg,Me}}]_2$ with benzene-d_6

A J. Young's tube was charged with $[\mathbf{2^{Mg,Me}}]_2$ (12 mg, 0.014 mmol) and benzene-d_6 (0.6 ml). The tube was closed and heated to 120°C in an oil bath for 12 days. Analysis by ^{1}H NMR spectroscopy indicated 38% yield of $\mathbf{3^{Me}}$, which was identified by comparison to the published NMR data,[6] and 10% yield of a second, unidentified BDI-containing species. According to integration of the hydride resonance, some H/D exchange to give the deuteride $[(^{Me}BDI)Mg(\mu\text{-}D)]_2$ may also occur.

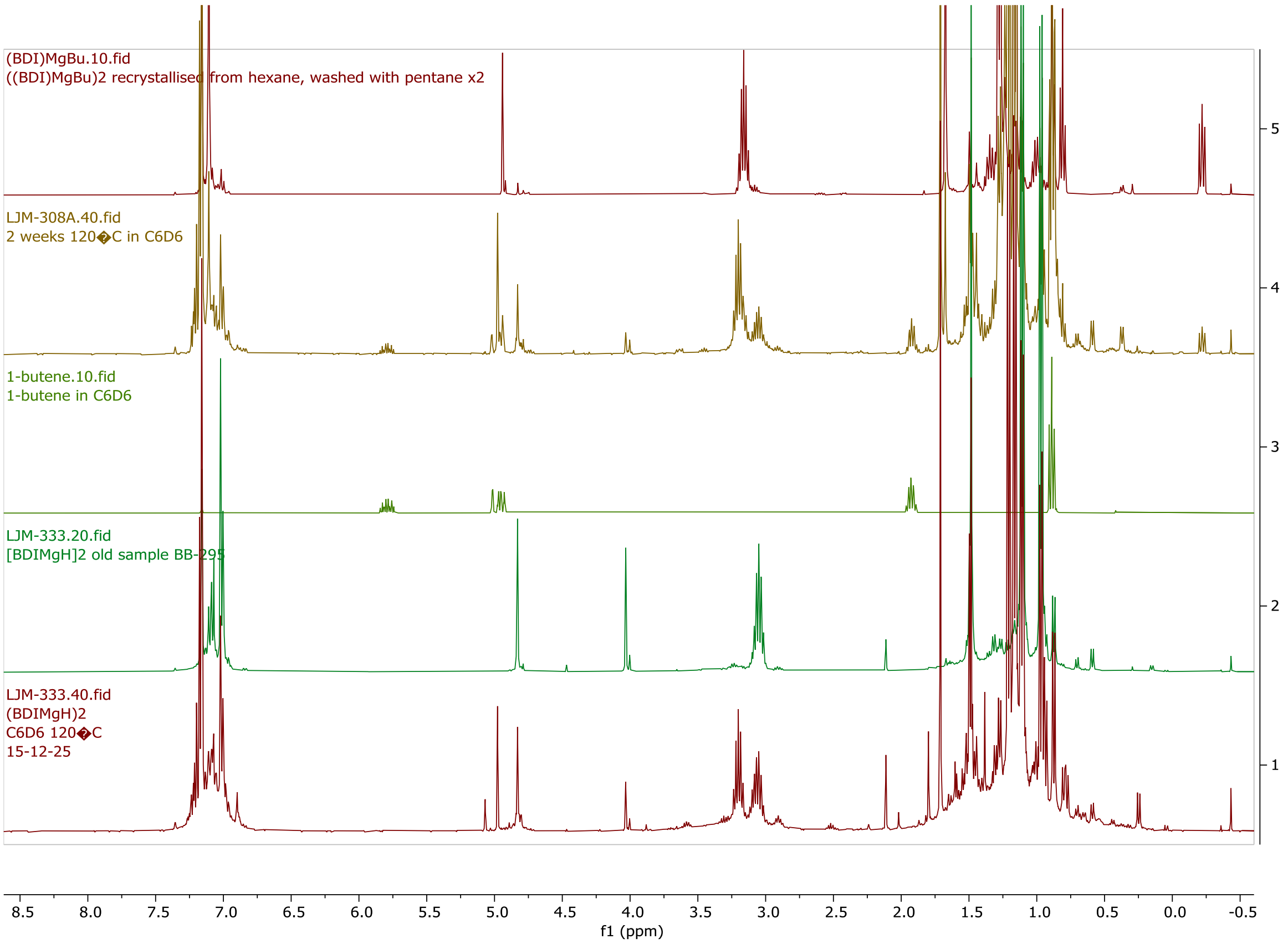

Figure S41: Stacked ^{1}H NMR spectra (400 MHz, C_6D_6), from top to bottom: $[\mathbf{1^{Me}}]_2$; after heating $[\mathbf{1^{Me}}]_2$ at 120°C for 2 weeks; 1-butene; $[\mathbf{2^{Mg}}]_2$; after heating $[\mathbf{2^{Mg}}]_2$ at 120°C for 2 weeks.

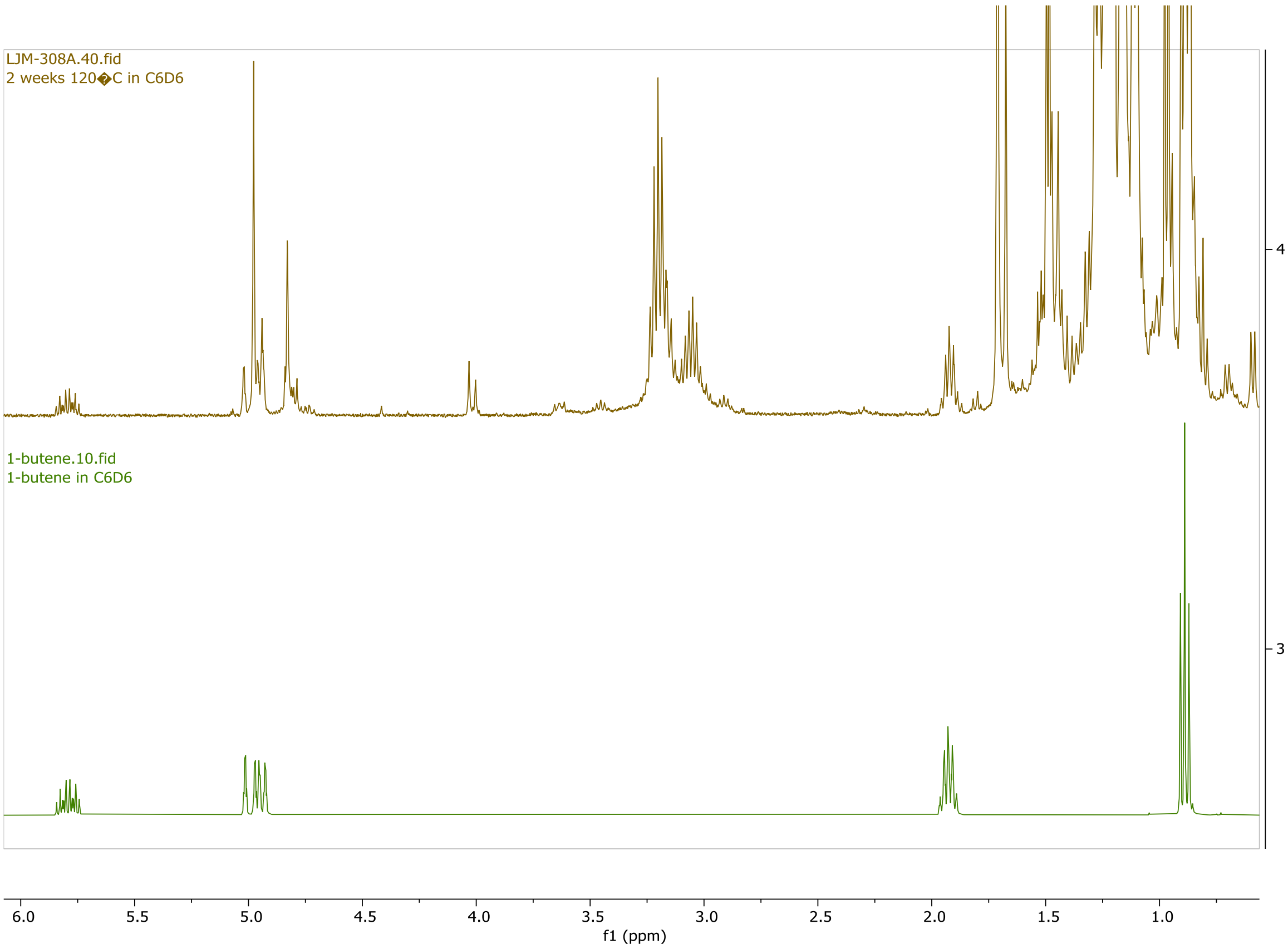

Figure S42: Stacked ^{1}H NMR spectra (400 MHz, C_6D_6) highlighting formation of 1-butene after heating **[1^{Me}]$_2$** at 120°C for 2 weeks (top) compared to authentic sample of 1-butene (bottom).

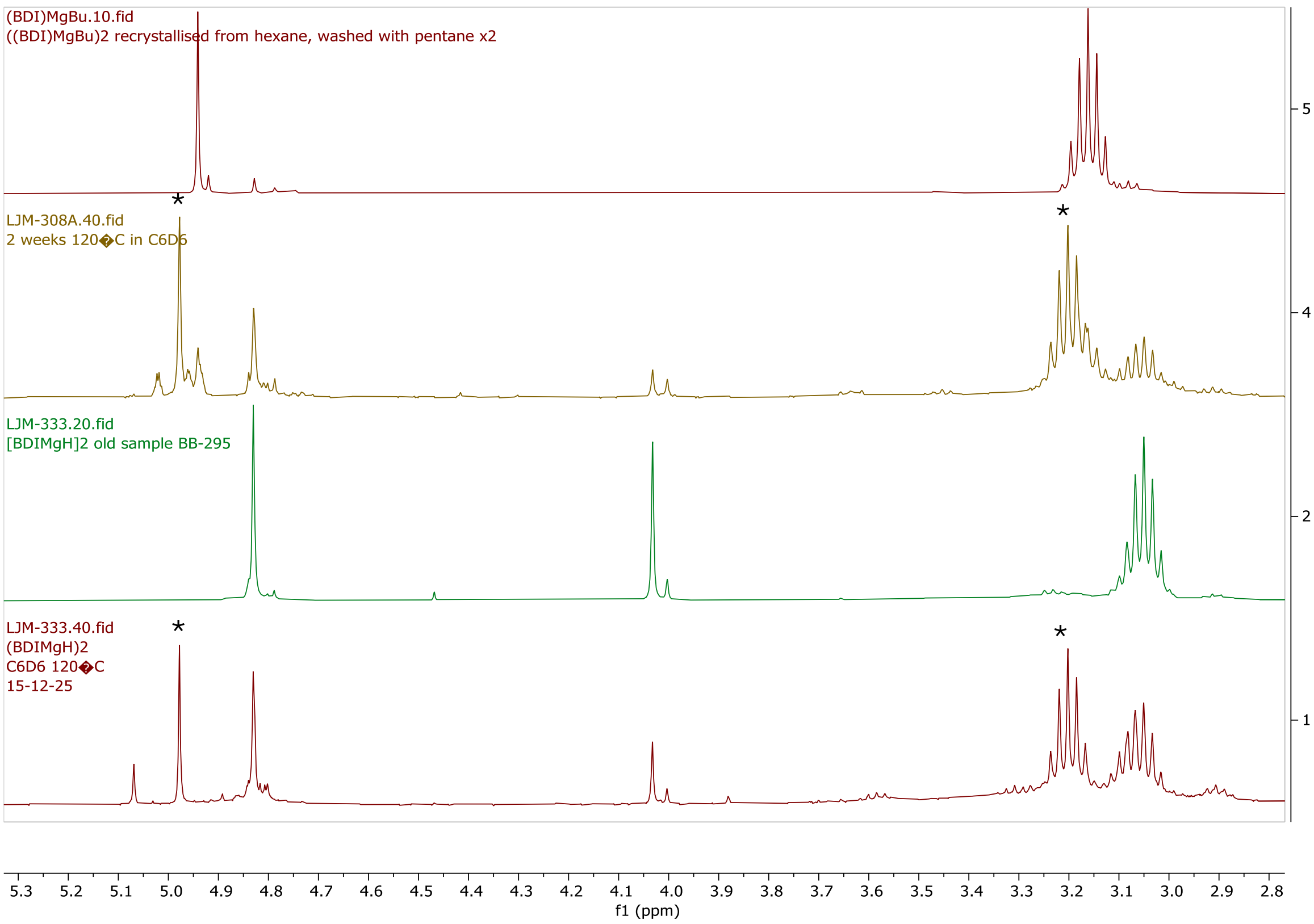

Figure S43: Expanded stacked ^{1}H NMR spectra (400 MHz, C_6D_6) highlighting formation of **[2^{Mg}]$_2$** and **3^{Me}**(*) after heating **[1^{Me}]$_2$** in benzene-d$_6$ at 120°C for 2 weeks, and highlighting formation of **3^{Me}** (*) after heating **[2^{Mg}]$_2$** in benzene-d$_6$ at 120°C for 2 weeks; from top to bottom: **[1^{Me}]$_2$**; after heating **[1^{Me}]$_2$** at 120°C for 2 weeks; **[2^{Mg}]$_2$**; after heating **[2^{Mg}]$_2$** at 120°C for 2 weeks.

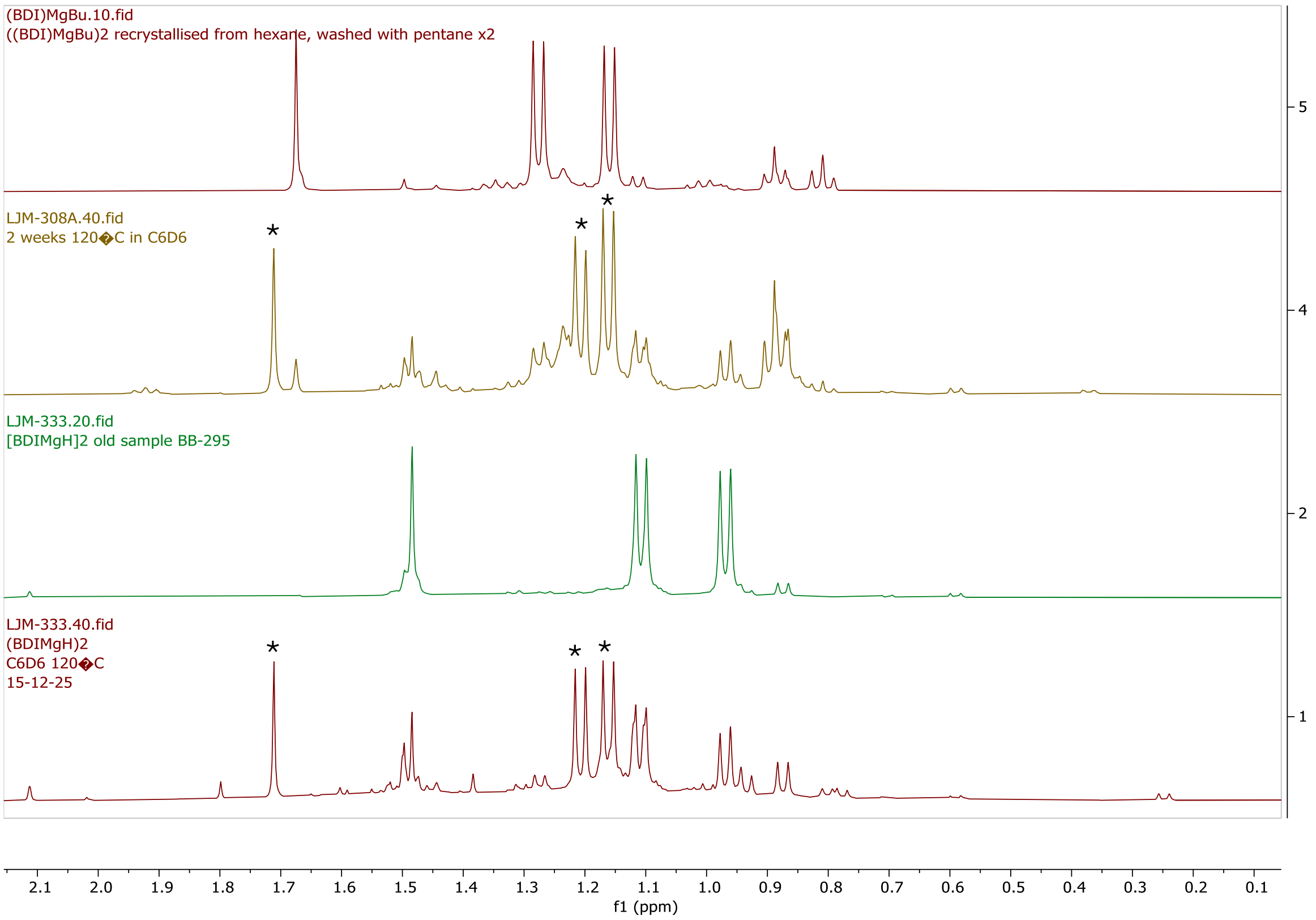

Figure S44: Expanded stacked ^{1}H NMR spectra (400 MHz, C_6D_6) highlighting formation of **[2^{Mg}]$_2$** and **3^{Me}**(*) after heating **[1^{Me}]$_2$** in benzene-d$_6$ at 120°C for 2 weeks, and highlighting formation of **3^{Me}**(*) after heating **[2^{Mg}]$_2$** in benzene-d$_6$ at 120°C for 2 weeks; from top to bottom: **[1^{Me}]$_2$**; after heating **[1^{Me}]$_2$** at 120°C for 2 weeks; **[2^{Mg}]$_2$**; after heating **[2^{Mg}]$_2$** at 120°C for 2 weeks.

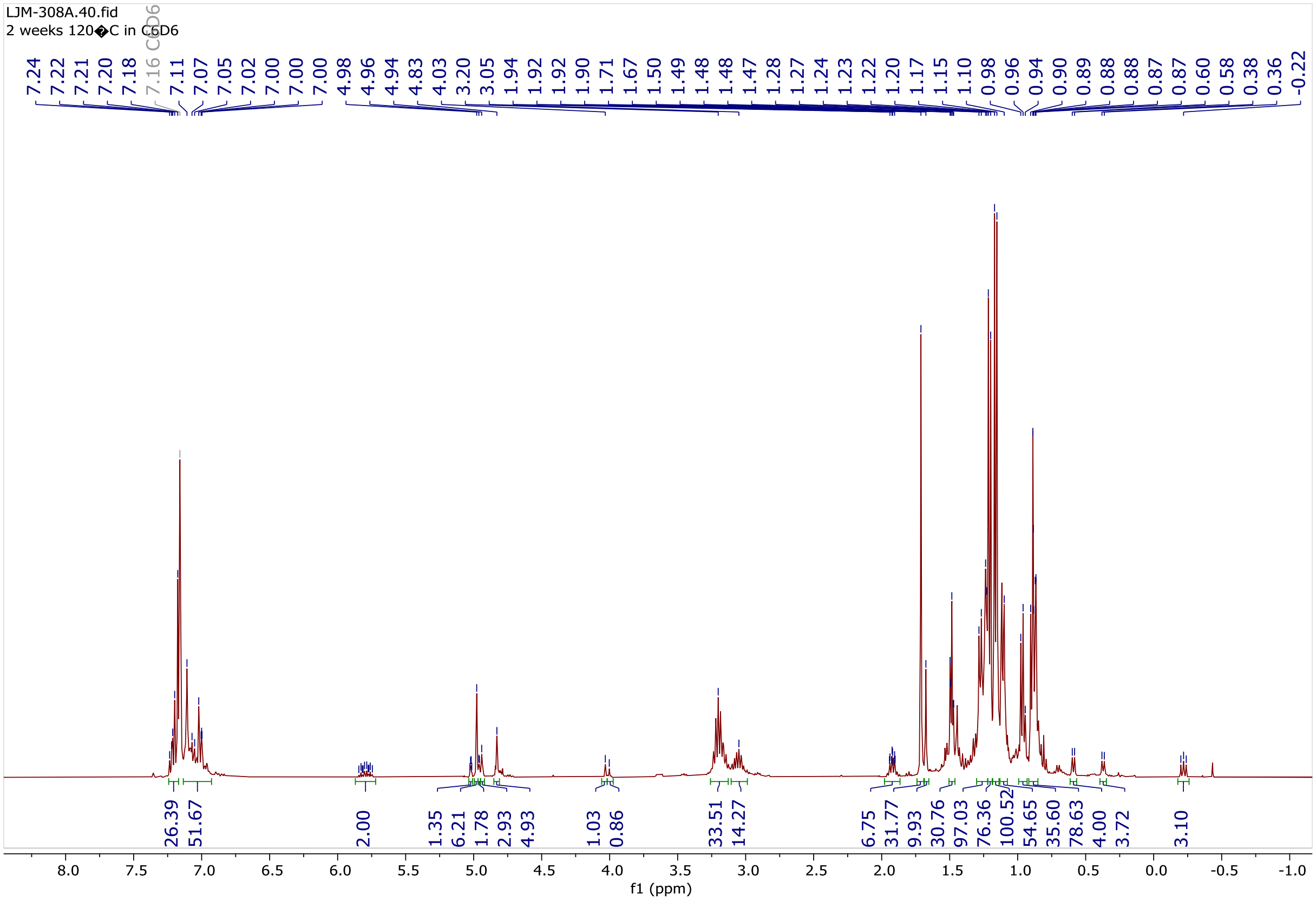

Figure S45: ^1H NMR spectrum (400 MHz, C_6D_6) after heating **[1^{Me}]$_2$** in benzene-d_6 at 120°C for 2 weeks to form **[2^{Mg}]$_2$** and **3^{Me}**.

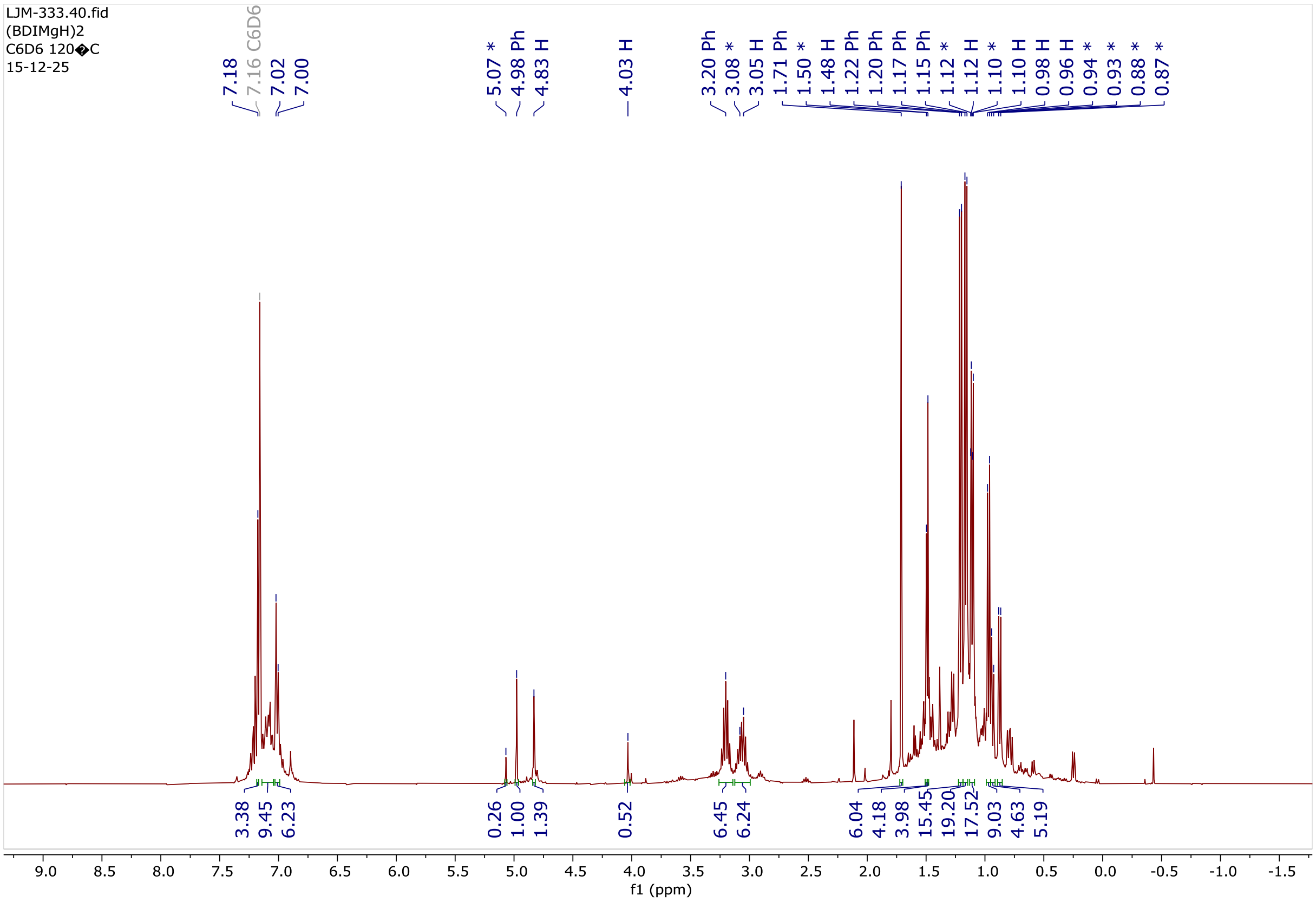

Figure S46: ^1H NMR spectrum (400 MHz, benzene-d_6) after heating a benzene-d_6 solution of **[2^{Mg}]$_2$** for 12 days at 120°C. Resonances are annotated to indicate **3^{Me}** (Ph), **[2^{Mg}]$_2$** (H), and unidentified product (*).

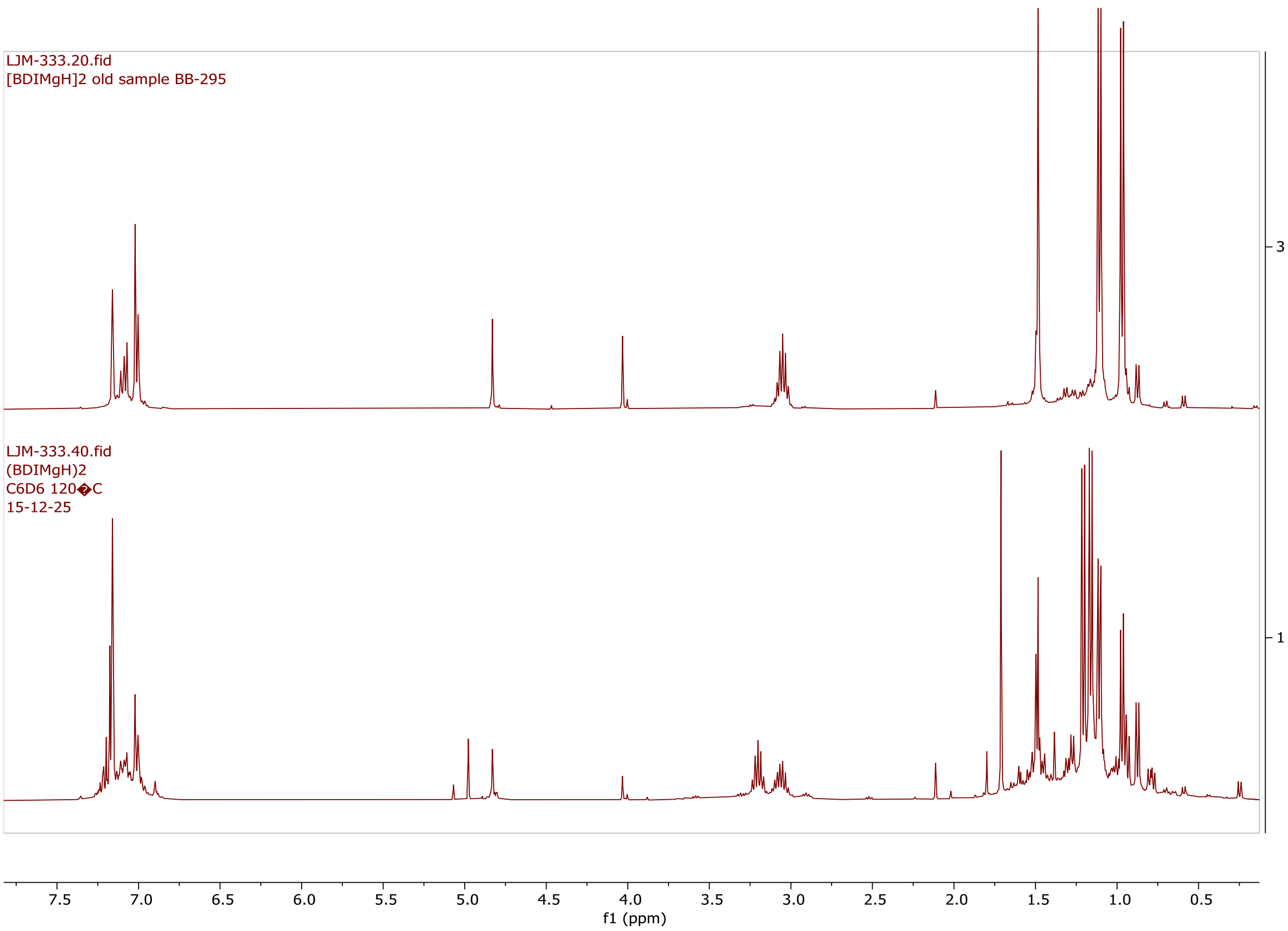

Figure S47: Stacked ^{1}H NMR spectra (400 MHz, C_6D_6) of **[2^{Mg}]** before (top) and after (bottom) heating at 120°C for 12 days.

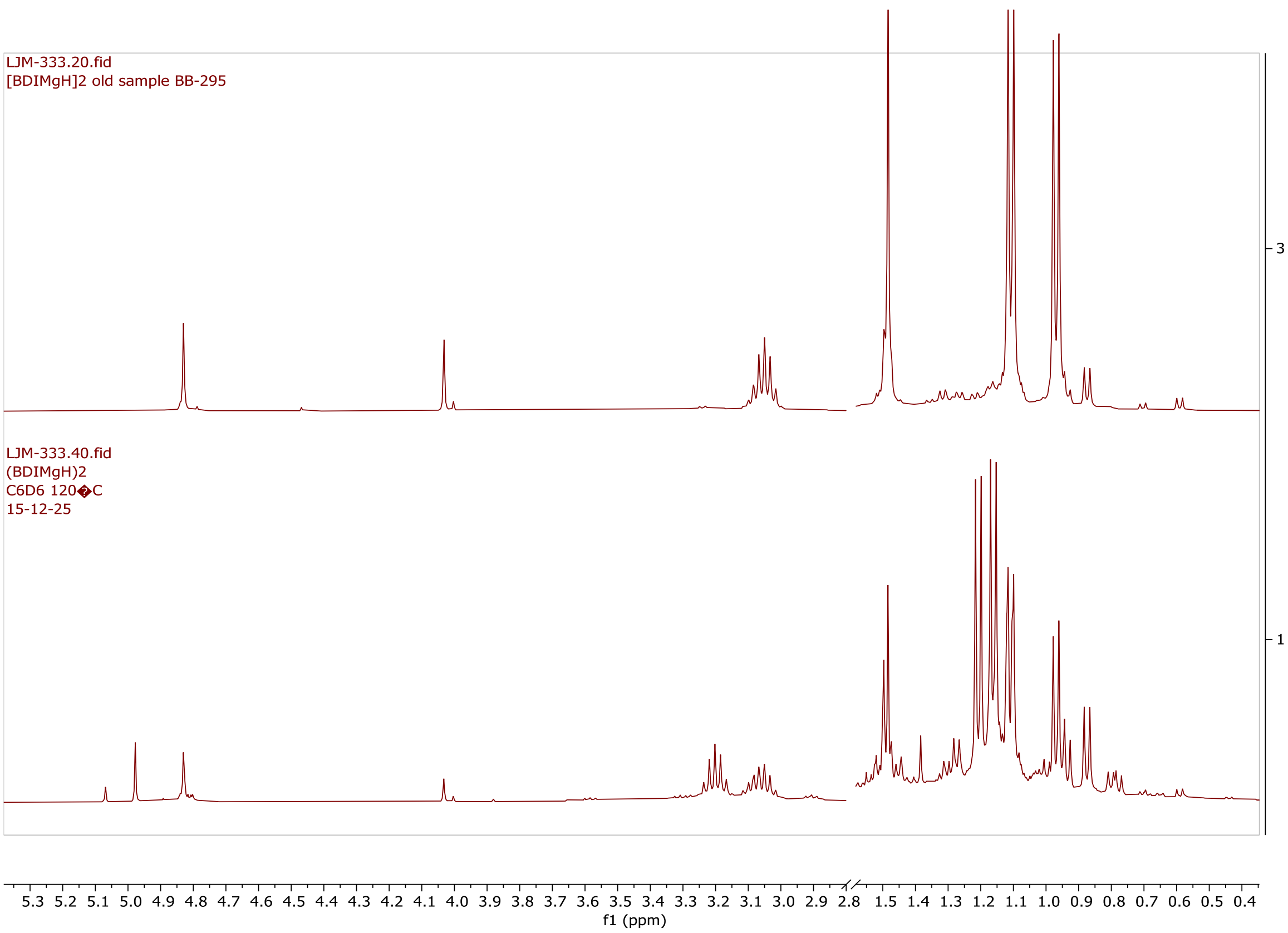

Figure S48: Expanded stacked ^{1}H NMR spectra (400 MHz, C_6D_6) of **[2^{Mg}]** before (top) and after (bottom) heating at 120°C for 12 days.

2.11 Reaction of [(^{Ad}BDI)Mg^{n}Bu] (**1^{Ad}**) with toluene, synthesis and characterisation of [(^{Ad}BDI)Mg(C_6H_4Me)] (5-*m*/5-*p*)

A J. Young's NMR tube was charged with 15 mg **1^{Ad}** (0.02 mmol) and 0.6 ml toluene-*d$_8$*, then 3 µl Cp^{4M}H (0.02 mmol) was added. The pale-yellow solution was heated at 100°C for 8 days. Analysis of the reaction solution by ^{1}H NMR indicated 89% conversion of the starting complex to form several BDI-containing products, but no consumption of Cp^{4M}H. Colourless block like crystals of [(^{Ad}BDI)Mg(C_6D_4-CD_3)] (co-crystalline mixture of [(^{Ad}BDI)Mg(C_6D_4-3-CD_3)] ***5-m-d$_7$*** and [(^{Ad}BDI)Mg(C_6D_4-4-CD_3) ***5-p-d$_7$***] in a 0.76:0.24 ratio) suiTable S for X-ray diffraction analysis formed above the meniscus in the NMR tube. The crystal structure was solved as a disordered co-crystal of ***5-m-d$_7$*** and ***5-p-d$_7$*** in a 0.76:0.24 ratio, but further residual electron density suggests the presence of [(^{Ad}BDI)Mg($CD_2C_6D_5$)] (**5'-*d$_7$***) as a further, minor component. Analysis of the isolated crystals by ^{1}H NMR spectroscopy is consistent with this. The reaction mixture was decanted into a vial and stored at -30°C to provide 7 mg ***5-m-d$_7$*/*5-p-d$_7$*** as colourless crystals in 44% yield.

The reaction was repeated in *proteo*-toluene in the absence of Cp^{4M}H. The pale-yellow solution was heated at 100°C for 7 days, then decanted into a vial. Slow evaporation of the solvent provided colourless crystals in 45% yield (7 mg) after washing with *n*-hexane. Analysis of the crystals by ^{1}H NMR spectroscopy indicated a mixture of **5-*m*** (51%), **5-*p*** (18%), **1^{Ad}** (16%) and **5'** (15%).

The reaction was repeated at a temperature of 120°C. After 13 days, ^{1}H NMR of the reaction solution indicated quantitative consumption of **1^{Ad}**. Volatiles were removed under vacuum, and the crude products redissolved in benzene-*d$_6$*. Subsequent analysis by NMR spectroscopy showed the **5-*p***, **5-*m***, and **5'** in respective spectroscopic yields of 21%, 40%, and 39%, according to NMR integration.

Analysis calculated for $C_{54}H_{72}MgN_2$ (mixture of isomers): C, 83.85; H, 9.38; N, 3.62%. Found: C, 84.89; H, 9.98; N, 3.02%.

NMR characterisation of ***5-p***

^{1}H NMR (500 MHz, C_6D_6) δ 7.25 (t, *J* = 7.7 Hz, 2H, *para*-$C_6\underline{H_3}$-*i*Pr_2),* 7.15 – 7.09 (m, *meta*-$C_6\underline{H_3}$-*i*Pr_2),* 6.86 (d, *J* = 7.3 Hz, 2H, 1-Mg-*3,5*-C_6H_4-4-Me), 6.08 (d, *J* = 7.6 Hz, 2H, 1-Mg-*2,6*-C_6H_4-4-Me), 5.74 (s, 1H, , 3.48 – 3.34 (m, *J* = 7.0 Hz, *H*C(CH_3)$_2$),* 2.16 (d, ^{Ad}CCH_2CH),* 2.08 (s, 3H, MgC_6H_3C*H$_3$*), 1.86 (m, ^{Ad}C*H*),* 1.65 – 1.48 (m, ^{Ad}CHC$\underline{H_2}$CH),* 1.34 (d, *J* = 6.9 Hz, 12H, HC(C*H$_3$*)$_2$), 1.24 (d, *J* = 6.9 Hz, 12H, HC(C*H$_3$*)$_2$). *Signal overlaps with another isomer, accurate integral not determined.

^{13}C NMR (126 MHz, C_6D_6) δ 176.53 ($\underline{C}$=N), 158.48 (Mg$\underline{C}$), 145.30 (*ipso*-$\underline{C_6}H_3$-*i*Pr_2),141.57 (*ortho*-$\underline{C_6}H_3$-*i*Pr_2),* 140.95 (1-Mg-*2,6*-$\underline{C_6}H_4$-4-Me), 134.51 (Mg-4-$\underline{C_6}H_3$-4-CH_3), 127.50 (1-Mg-*3,5*-$\underline{C_6}H_4$-4-Me), 125.53 (*para*-$\underline{C_6}H_3$-*i*Pr_2),* 124.17 (*meta*-$\underline{C_6}H_3$-*i*Pr_2),* 93.75 (, 47.91 (NC$^{Ad}\underline{C}$),* 43.73 (^{Ad}C$\underline{C}H_2$CH),* 36.99 (^{Ad}CH$\underline{C}H_2$CH),* 28.54 (H$\underline{C}$(CH_3)$_2$), 25.83 (HC($\underline{C}H_3$)$_2$), 23.17 (HC($\underline{C}H_3$)$_2$), 21.64 (MgC_6H_4*C*H_3). *Resonance assumed to overlap with the equivalent resonance in **5-*m***.

NMR characterisation of ***5-m***

^{1}H NMR (500 MHz, C_6D_6) δ 7.22 (t, *J* = 7.7 Hz, *para*-$C_6\underline{H_3}$-*i*Pr_2),* 7.15 – 7.09 (m, *meta*-$C_6\underline{H_3}$-*i*Pr_2),* 7.08 – 6.97 (m, 1-Mg-*5*-$C_6\underline{H_4}$-3-Me),* 6.89 (d, *J* = 7.4 Hz, 1H, 1-Mg-*4*-$C_6\underline{H_4}$-3-Me), 6.04 (d, *J* = 6.9 Hz, 1H, 1-Mg-*6*-C_6H_4-3-Me), 5.83 (d, *J* = 2.0 Hz, 1H, 1-Mg-*2*-$C_6\underline{H_4}$-3-Me), 5.73 (s, 1H,), 3.48 – 3.34 (m, *J* = 7.0 Hz, *H*C(CH_3)$_2$),* 2.16 (d, *J* = 3.3 Hz, 21H, MgC_6H_3C*H$_3$* + ^{Ad}CC*H$_2$*CH),* 1.86 (m, ^{Ad}C*H*),* 1.65 – 1.48 (m, ^{Ad}CHC$\underline{H_2}$CH),* 1.33 (d, *J* = 6.9 Hz, 12H, HC(C*H$_3$*)$_2$), 1.22 (d, *J* = 6.9 Hz, 12H, HC(C*H$_3$*)$_2$). *Signal overlaps with another isomer, accurate integral not determined.

^{13}C NMR (126 MHz, C_6D_6) δ 176.51 ($\underline{C}$=N), 154.35 (Mg$\underline{C_6}H_4$), 145.34 (*ipso*-$\underline{C_6}H_3$-*i*Pr_2), 141.93 (1-Mg-*2*-$\underline{C_6}H_4$-3-Me), 141.57 (*ortho*-$\underline{C_6}H_3$-*i*Pr_2),* 134.17 (Mg-3-$\underline{C_6}H_3$-3-CH_3), 126.80 (Mg-4-$\underline{C_6}H_3$-3-CH_3),

126.35 (1-Mg-5-$\underline{C_6}$H$_4$-3-Me), 125.53 (*para*-$\underline{C_6}$H$_3$-*i*Pr$_2$),* 124.17 (*meta*-$\underline{C_6}$H$_3$-*i*Pr$_2$),* 93.70 (, 47.91 (NC$^{Ad}\underline{C}$),* 43.73 (^{Ad}C$\underline{C}$H$_2$CH^{A}),* 36.99 (^{Ad}CH$\underline{C}$H$_2$CH),* 28.53 (H$\underline{C}$(CH$_3$)$_2$), 25.81 (HC($\underline{C}$H$_3$)$_2$), 23.15 (HC($\underline{C}$H$_3$)$_2$), 21.74 (MgC$_6$H$_4$CH$_3$). *Resonance assumed to overlap with the equivalent resonance in **5-*p***.

*For NMR characterisation of **5'**, see separate synthesis, section 2.12*

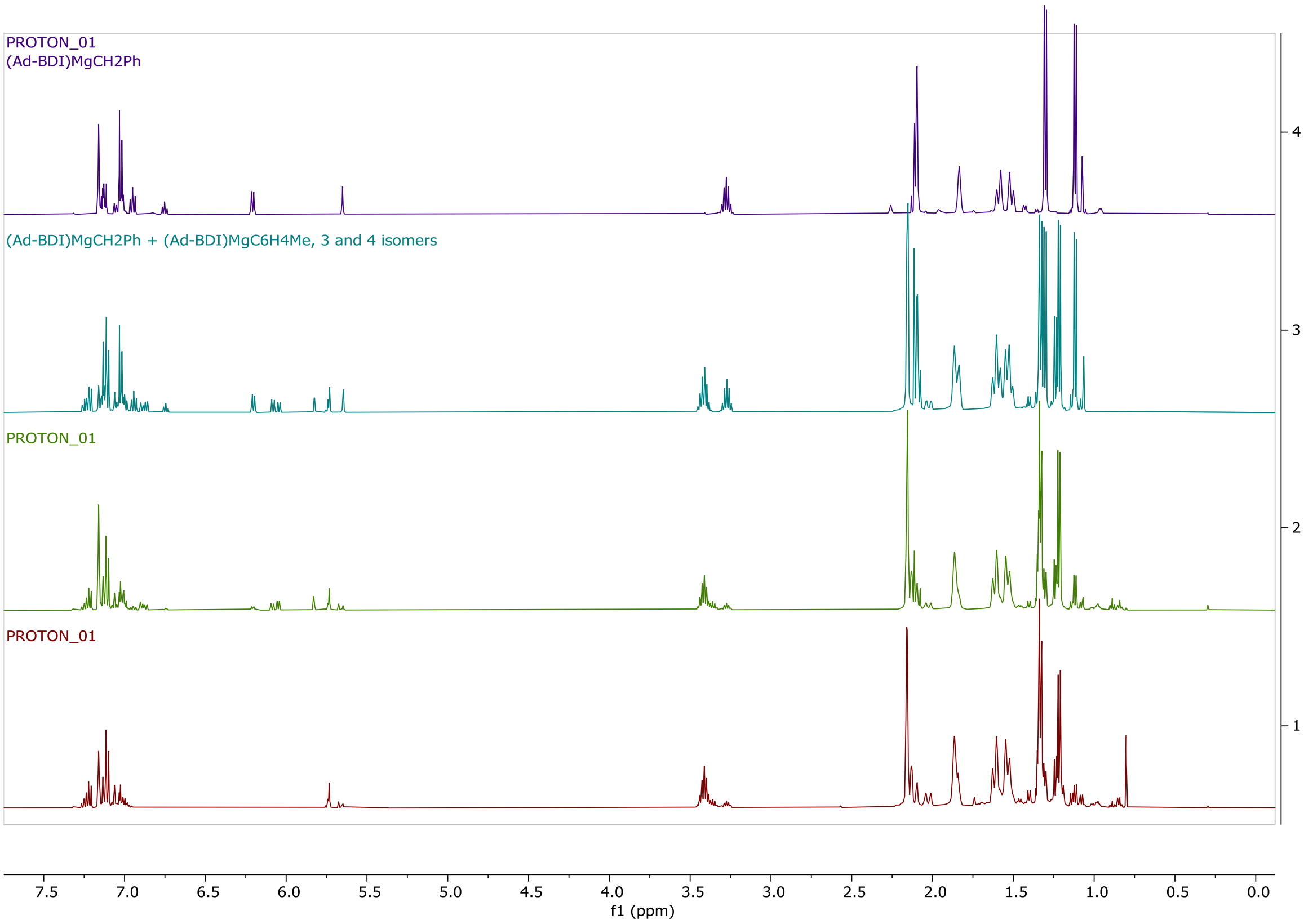

Figure S49: Stacked ^{1}H **5-*m***, **5-*p***, and **5'** obtained from reaction of **1^{Ad}** with toluene at 120°C; isolated crystals of mixture of isomers **5-*m***, **5-*p***, and **5'** contaminated with **1^{Ad}** from reaction of **1^{Ad}** with toluene at 100°C; isolated crystals of mixture of isomers **5-*m*-*d$_7$***, **5-*p*-*d$_7$***, and **5'** contaminated with **1^{Ad}** from reaction of **1^{Ad}** with toluene-*d$_8$* at 100°C.

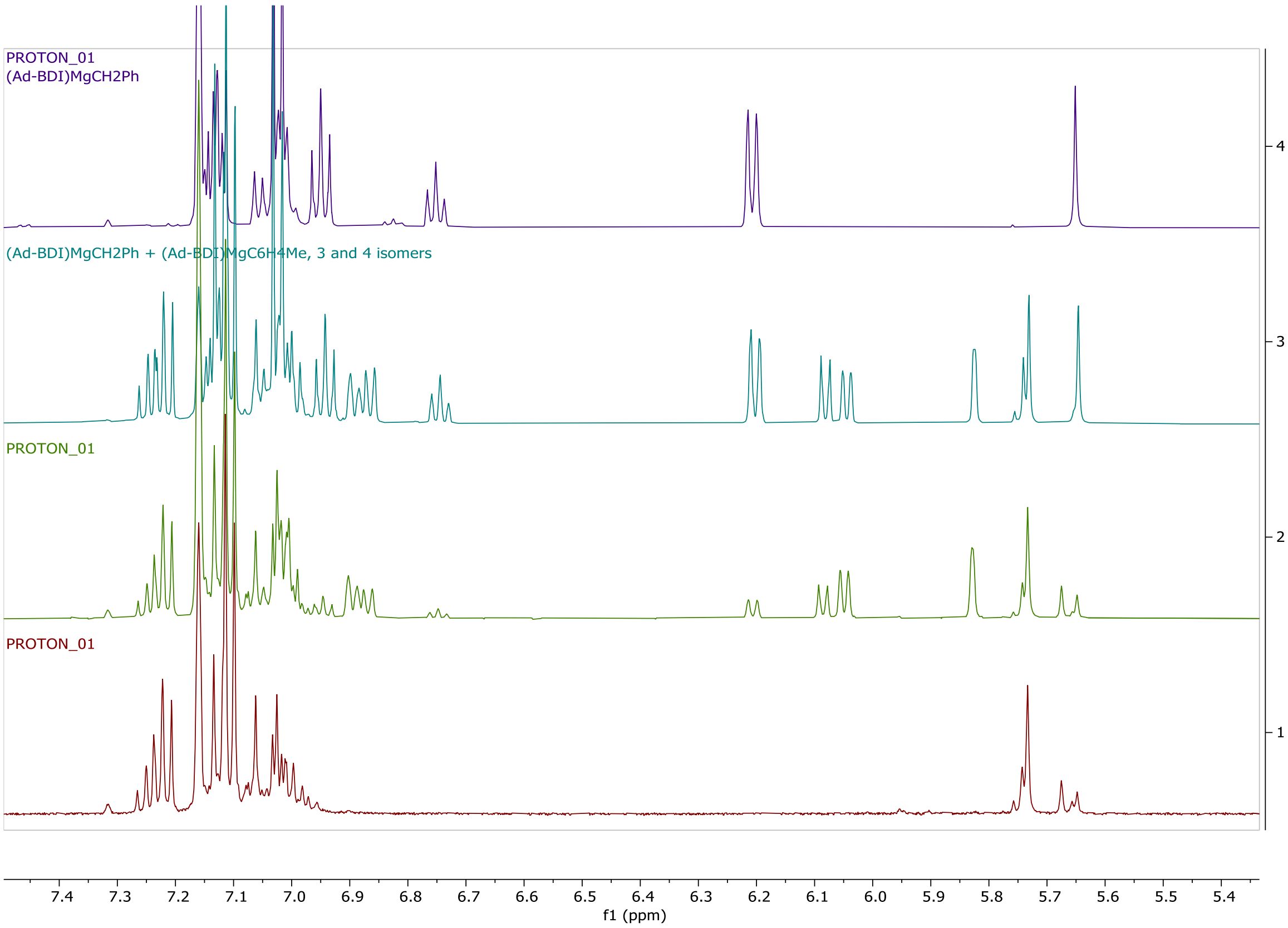

Figure S50: Stacked ^{1}H **5-*m***, **5-*p***, and **5'** obtained from reaction of $\mathbf{1}^{\mathbf{Ad}}$ with toluene at 120°C; isolated crystals of mixture of isomers **5-*m***, **5-*p***, and **5'** contaminated with $\mathbf{1}^{\mathbf{Ad}}$ from reaction of $\mathbf{1}^{\mathbf{Ad}}$ with toluene at 100°C; isolated crystals of mixture of isomers **5-*m*-*d*₇**, **5-*p*-*d*₇**, and **5'** contaminated with $\mathbf{1}^{\mathbf{Ad}}$ from reaction of $\mathbf{1}^{\mathbf{Ad}}$ with toluene-d_8 at 100°C.

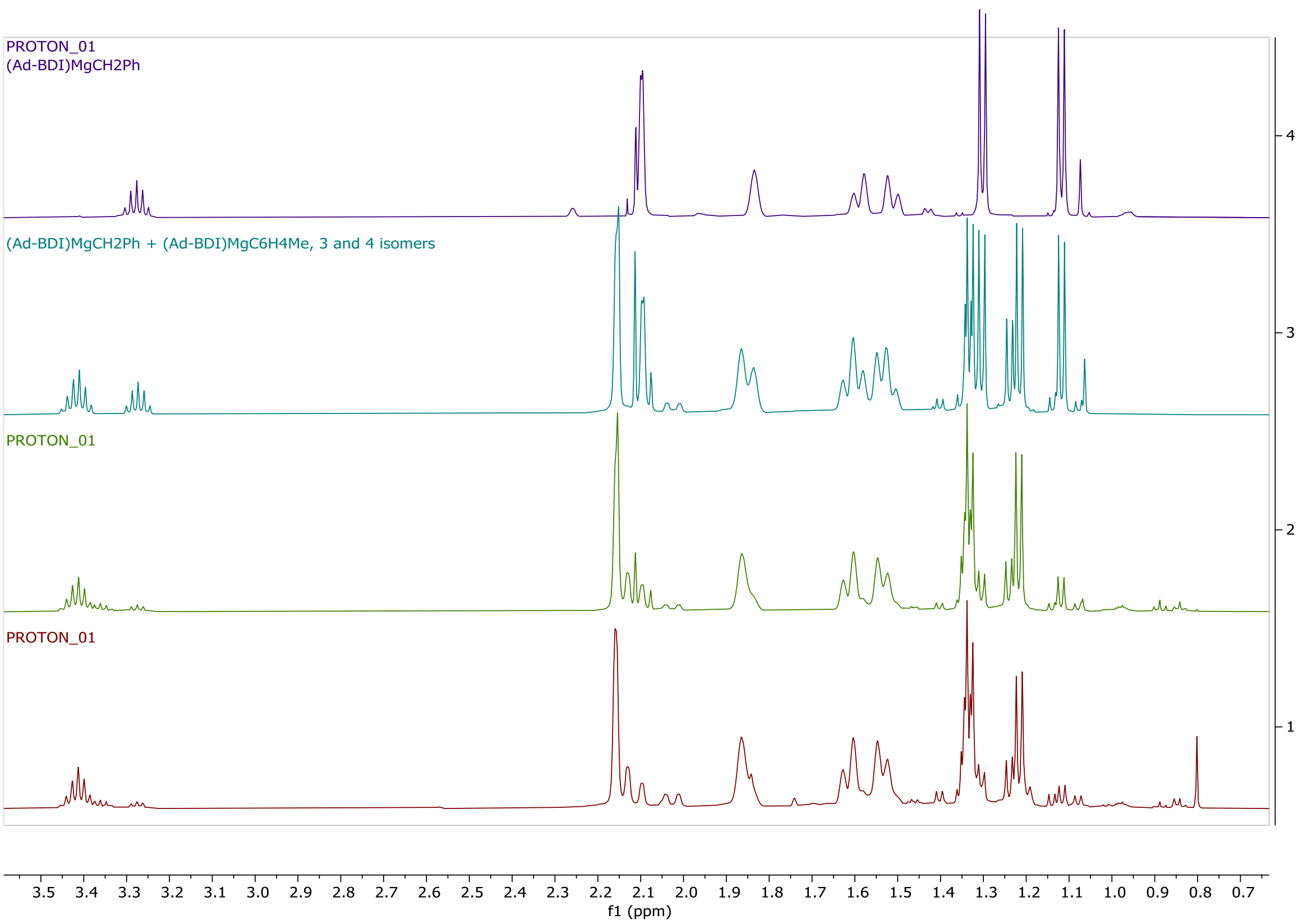

Figure S51: Stacked ^{1}H **5-*m***, **5-*p***, and **5'** obtained from reaction of $\mathbf{1}^{\mathbf{Ad}}$ with toluene at 120°C; isolated crystals of mixture

of isomers **5-*m***, **5-*p***, and **5'** contaminated with $\mathbf{1^{Ad}}$ from reaction of $\mathbf{1^{Ad}}$ with toluene at 100°C; isolated crystals of mixture of isomers **5-*m*-*d*₇**, **5-*p*-*d*₇**, and **5'** contaminated with $\mathbf{1^{Ad}}$ from reaction of $\mathbf{1^{Ad}}$ with toluene-d_8 at 100°C.

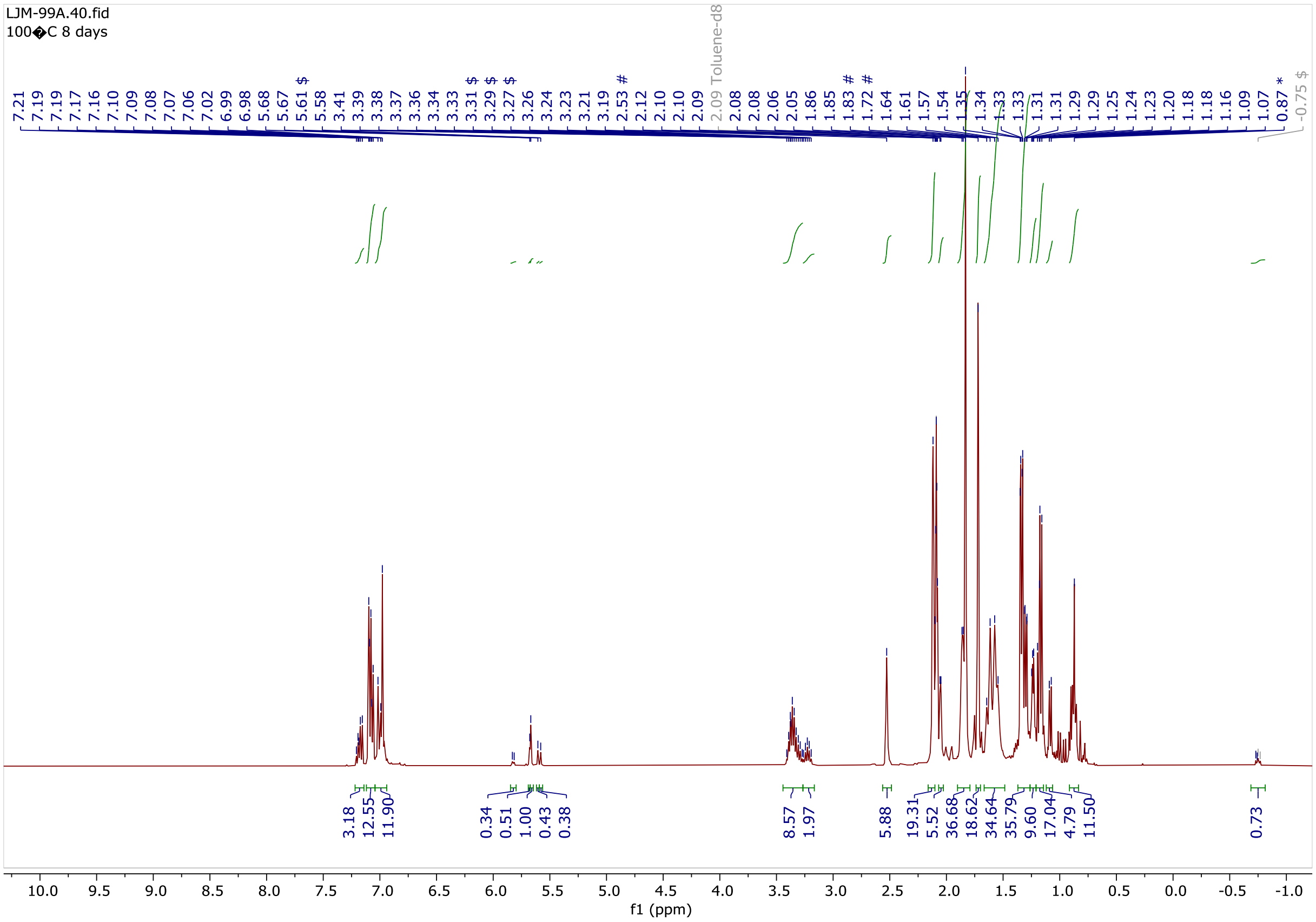

Figure S52: ^{1}H NMR spectrum (400 MHz, toluene-d_8) of reaction of $\mathbf{1^{Ad}}$ with toluene-d_8 in the presence of Cp^{4M}H after 8 days at 100°C. #Cp^{4M}H. *n-butane. $^{\$}\mathbf{1^{Ad}}$.

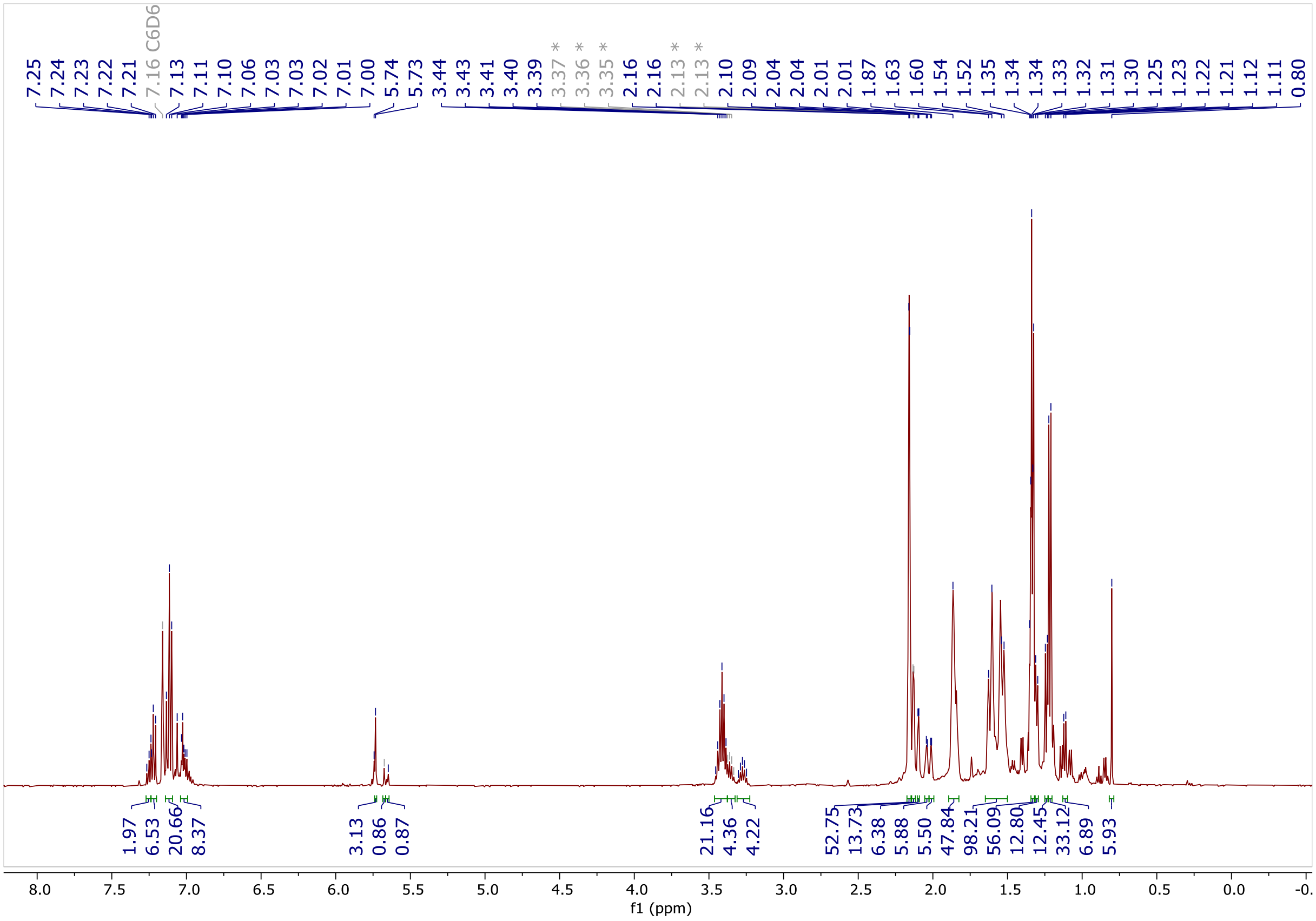

Figure S53: ^{1}H NMR (500 MHz, C_6D_6) of isolated co-crystals of **5-*m*-*d*$_7$/5-*p*-*d*$_7$/5'**. *$\mathbf{1^{Ad}}$.

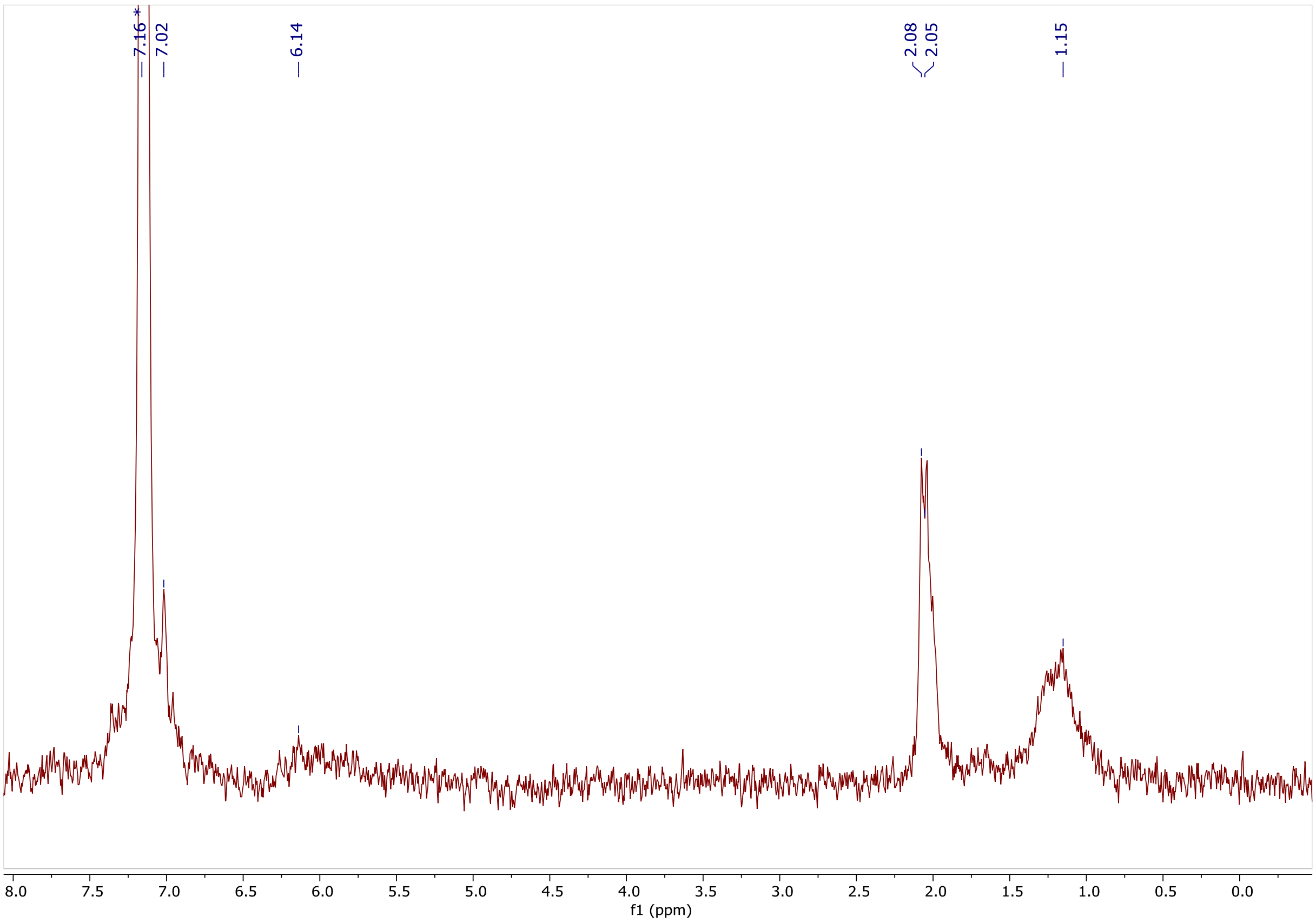

Figure S54: ^{2}H NMR spectrum (61 MHz, C_6H_6) of isolated co-crystals of **5-*m*-*d*$_7$/5-*p*-*d*$_7$/5'**. *Residual C_6D_6.

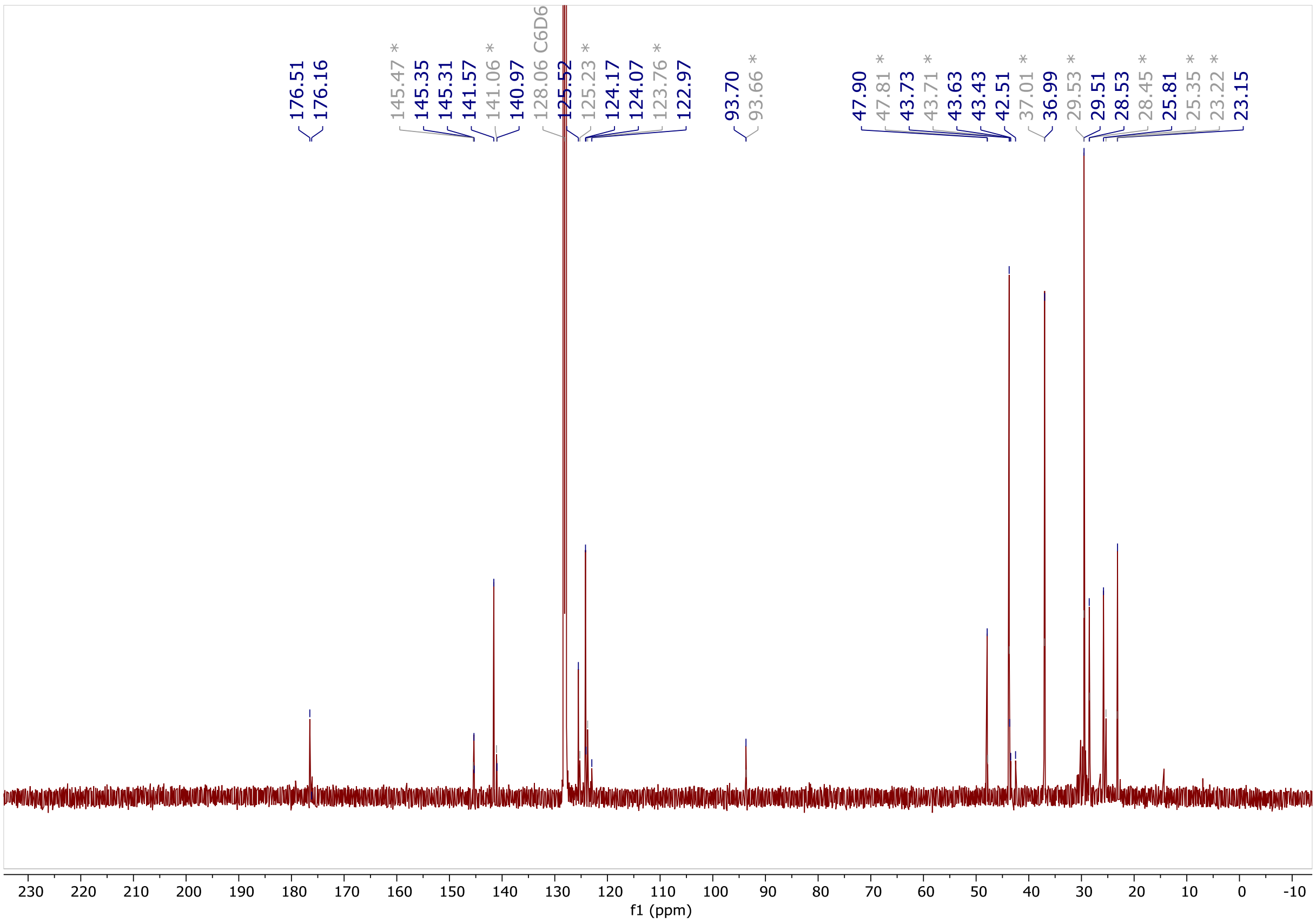

Figure S55: $^{13}C\{^1H\}$ NMR (126 MHz, C_6D_6) of isolated co-crystals of **5-*m*-*d*$_7$/5-*p*-*d*$_7$/5'**. *$\mathbf{1^{Ad}}$.

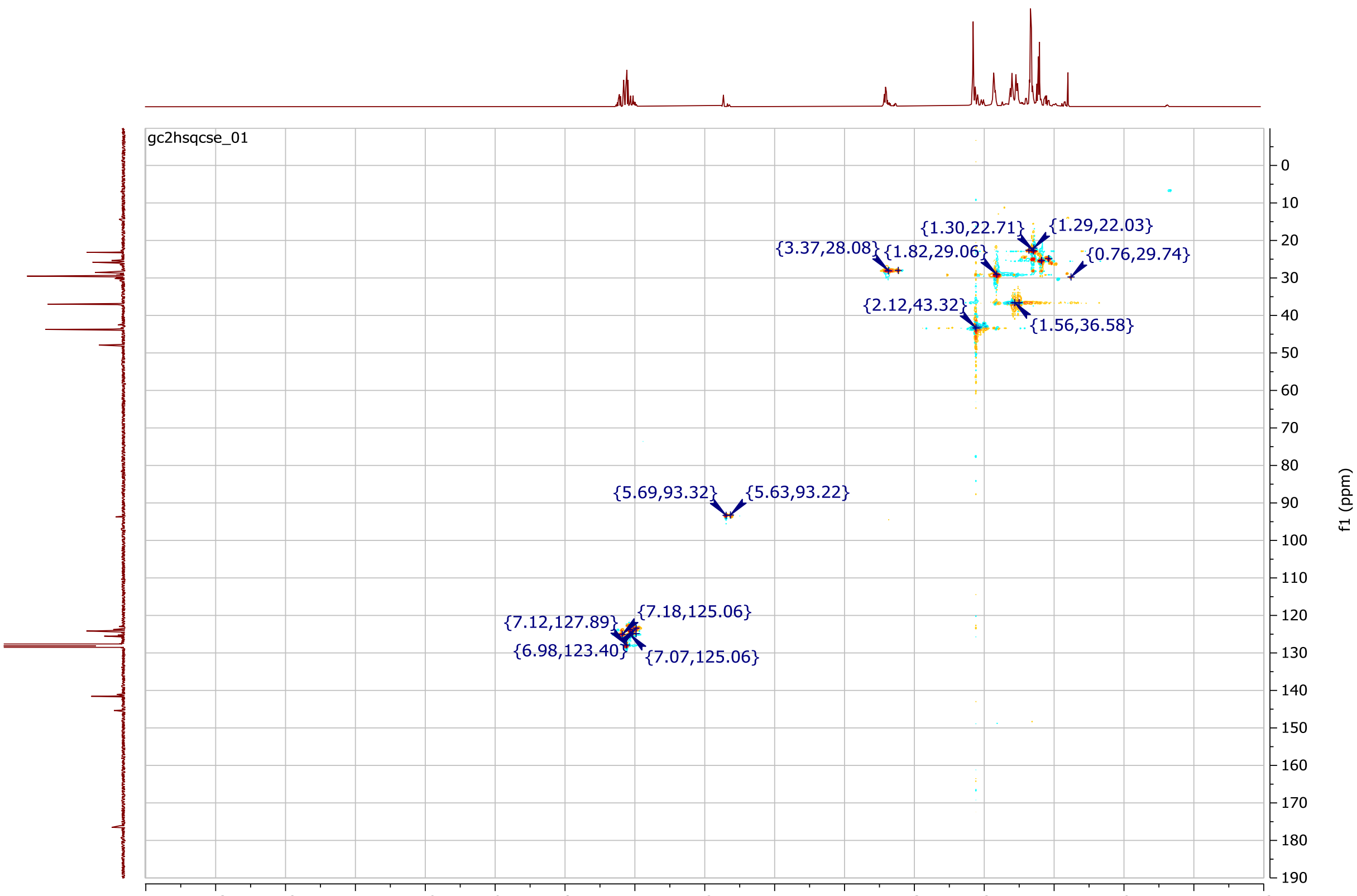

Figure S56: $^{13}C\{^1H\}$ HSQC NMR (126 MHz, C_6D_6) of isolated co-crystals of **5-*m*-*d*$_7$/5-*p*-*d*$_7$/5'**. *$\mathbf{1^{Ad}}$.

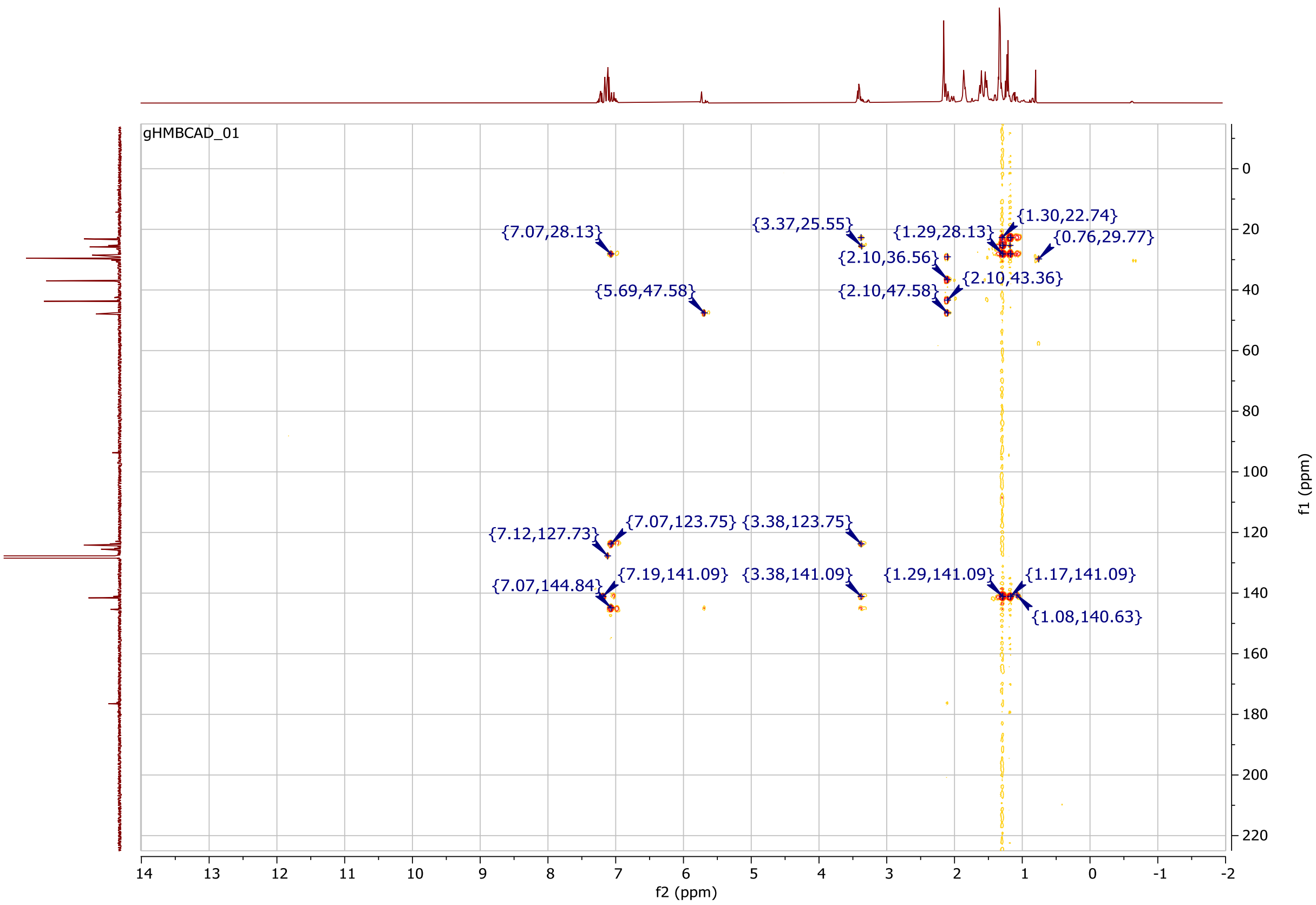

Figure S57: $^{13}C\{^{1}H\}$ HMBC NMR (126 MHz, C_6D_6) of isolated co-crystals of **5-*m*-*d*$_7$/5-*p*-*d*$_7$/5'**. *$\mathbf{1^{Ad}}$.

<u>*NMR spectra of isolated crystals from 100°C reaction.*</u> Contains **5'** and $\mathbf{1^{Ad}}$ as minor impurities.

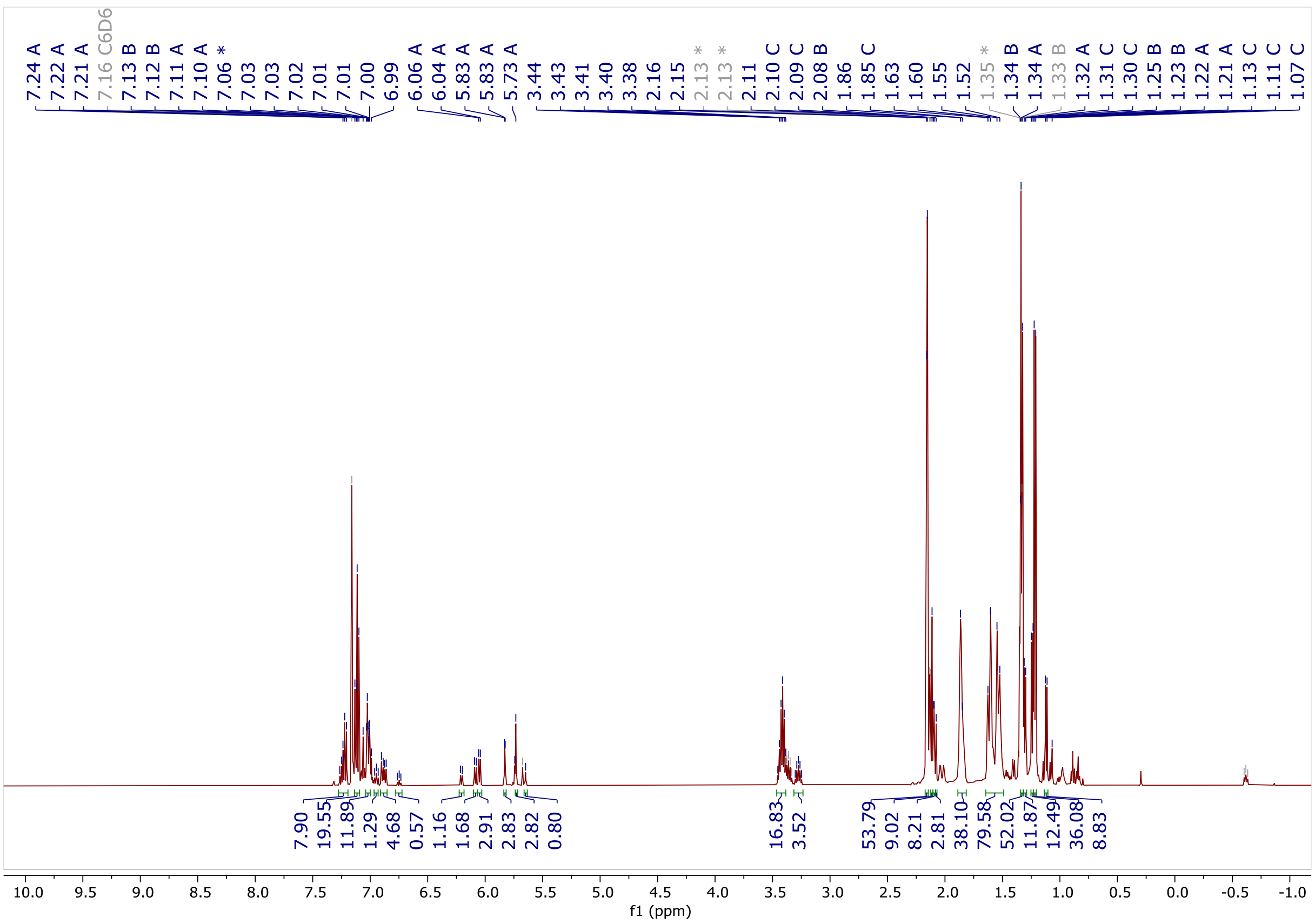

Figure S58: ^{1}H NMR (500 MHz, C_6D_6). Resonances annotated to denote isomers: A, **5-*m***; B, **5-*p***; C, **5'**. *$\mathbf{1^{Ad}}$

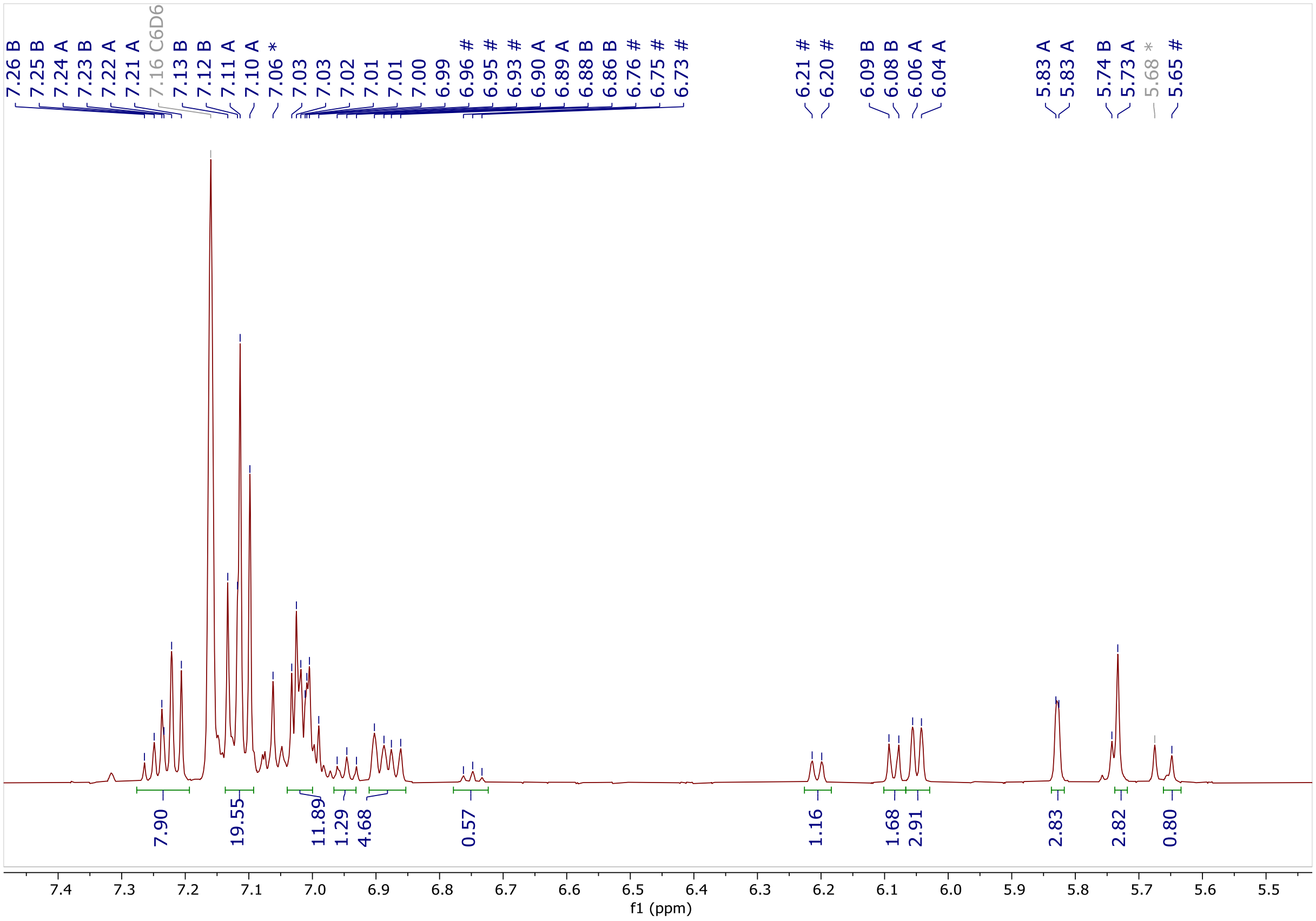

Figure S59: Expanded ^{1}H NMR (500 MHz, C_6D_6). Resonances annotated to denote isomers: A, **5-*m***; B, **5-*p***; C, **5'**. ***1**^{Ad}**.

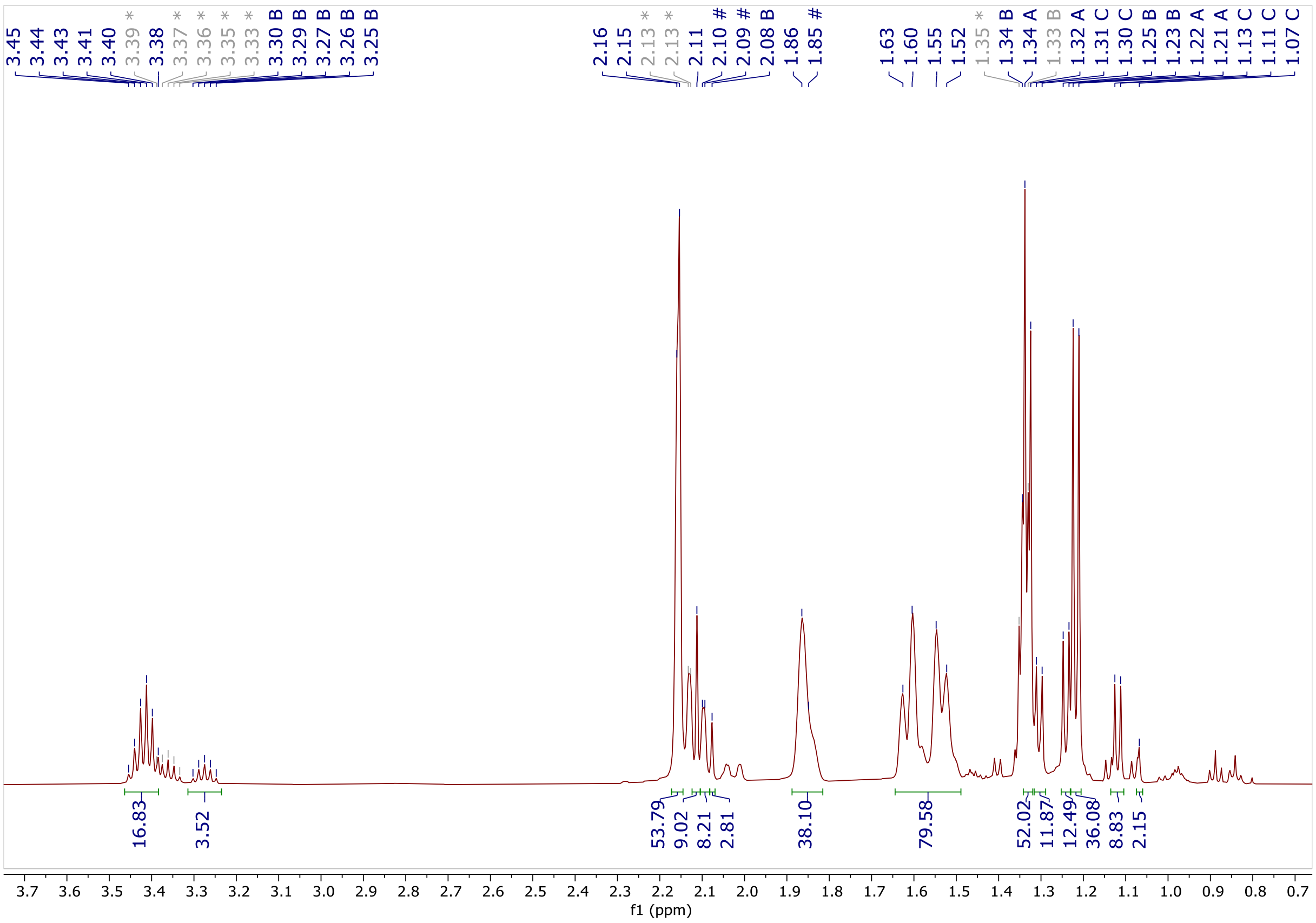

Figure S60: Expanded ^{1}H NMR (500 MHz, C_6D_6). Resonances annotated to denote isomers: A, **5-*m***; B, **5-*p***; C, **5'**. ***1**^{Ad}**.

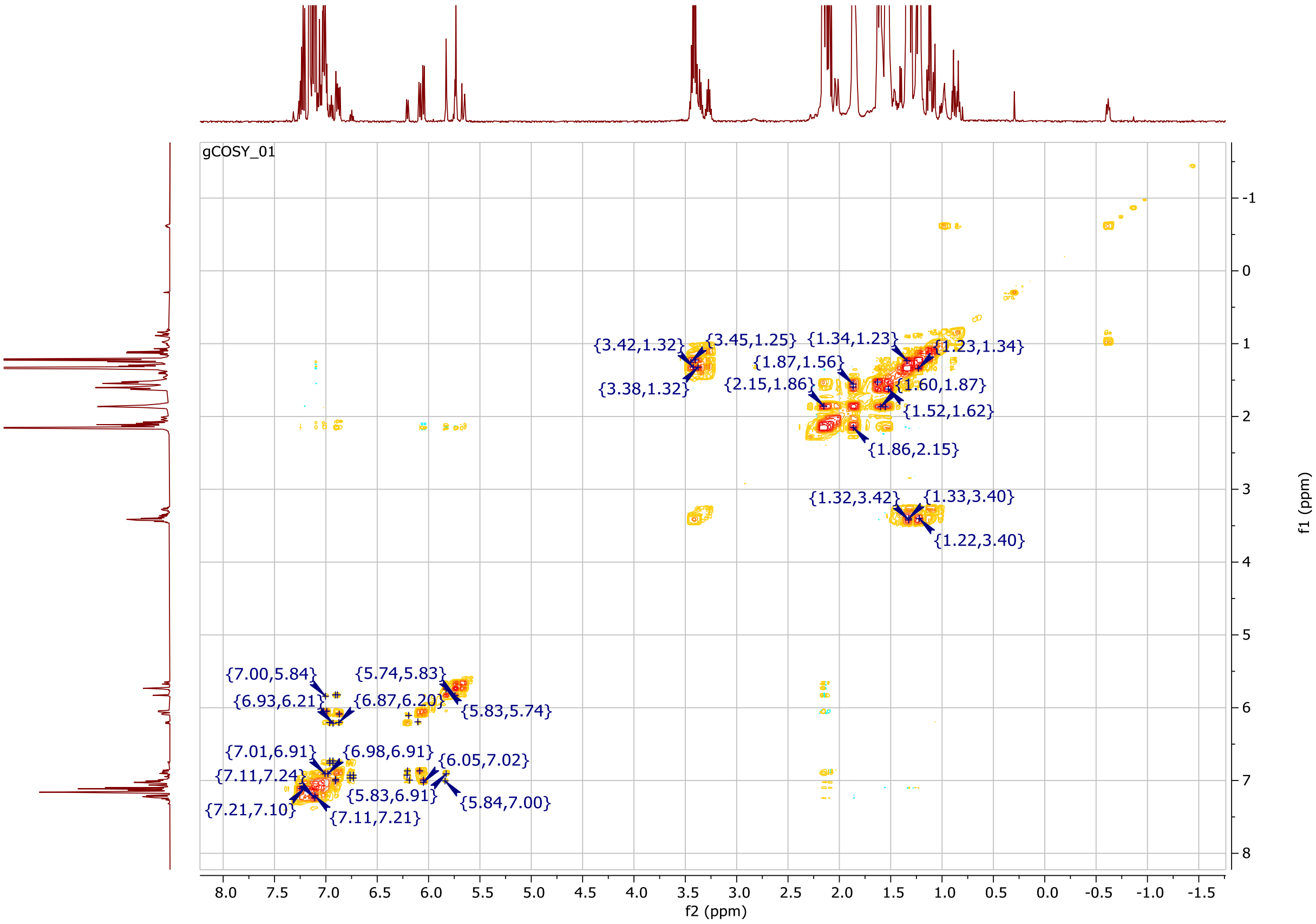

Figure S61: ^{1}H-^{1}H COSY spectrum (500 MHz, C_6D_6).

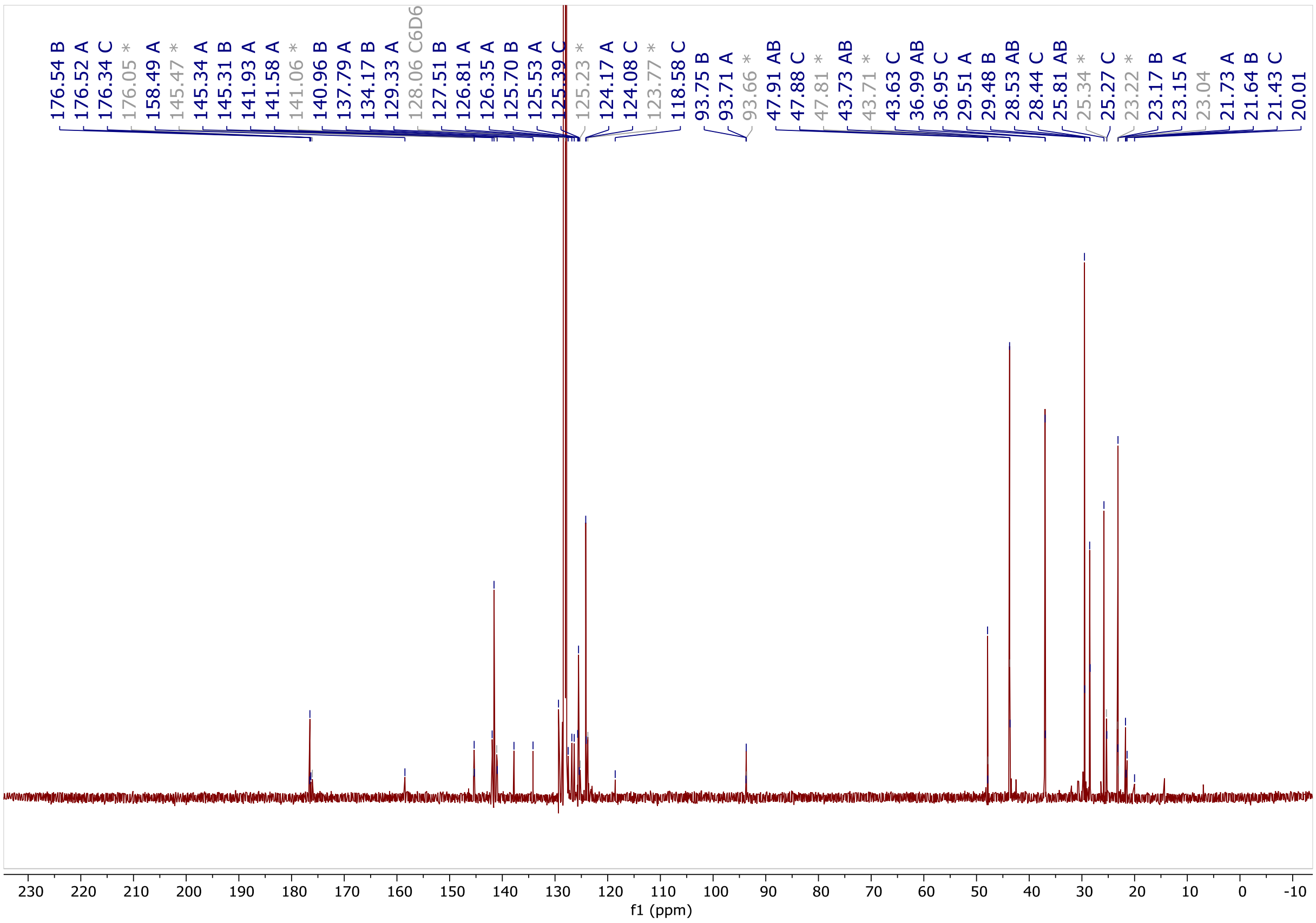

Figure S62: $^{13}C\{^{1}H\}$ NMR (126 MHz, C_6D_6). Resonances annotated to denote isomers A, **5-*m***; B, **5-*p***; C, **5'**. ***1**[Ad]*.

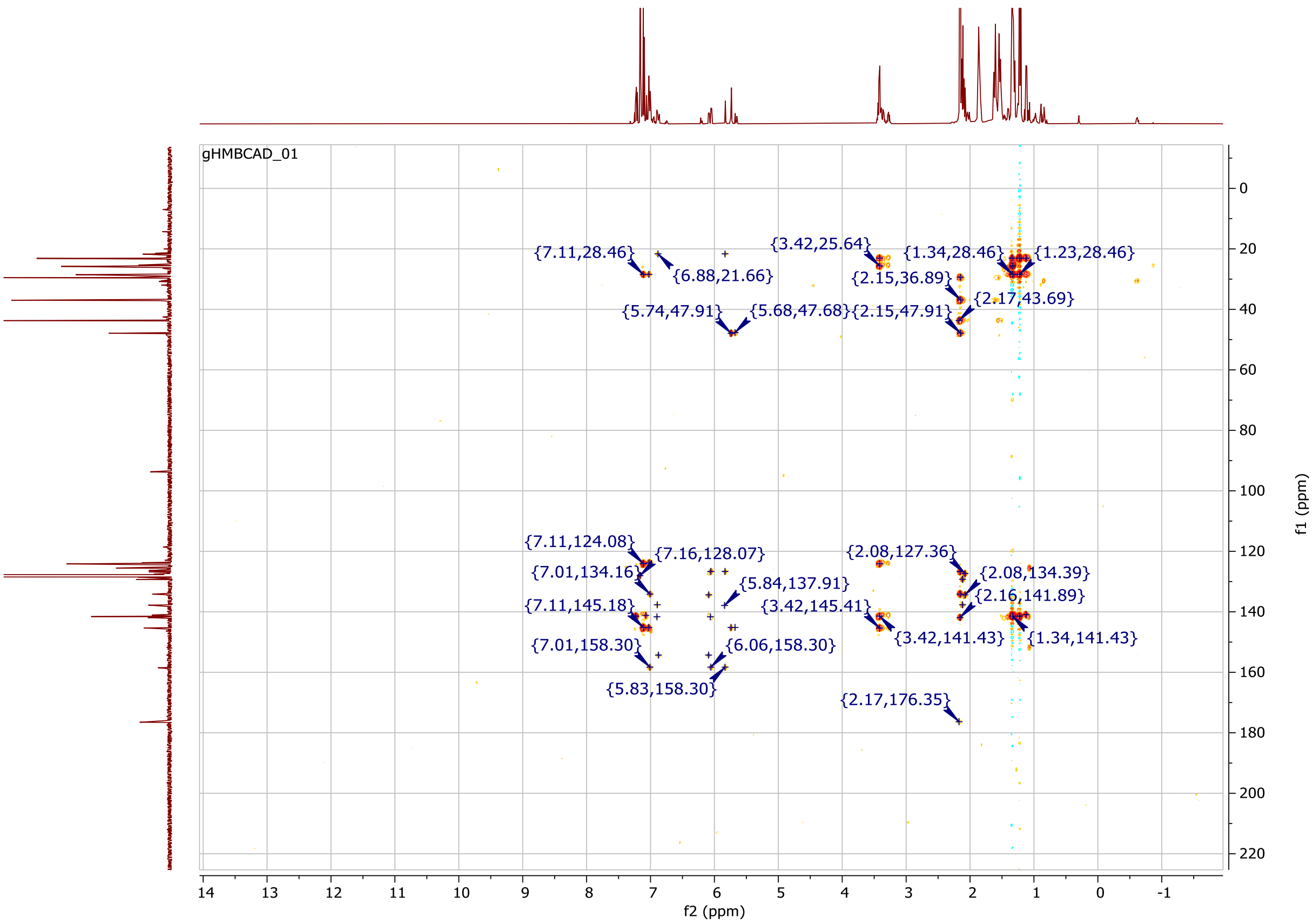

Figure S63: ^{13}C-^{1}H HMBC NMR (126 MHz, C_6D_6)

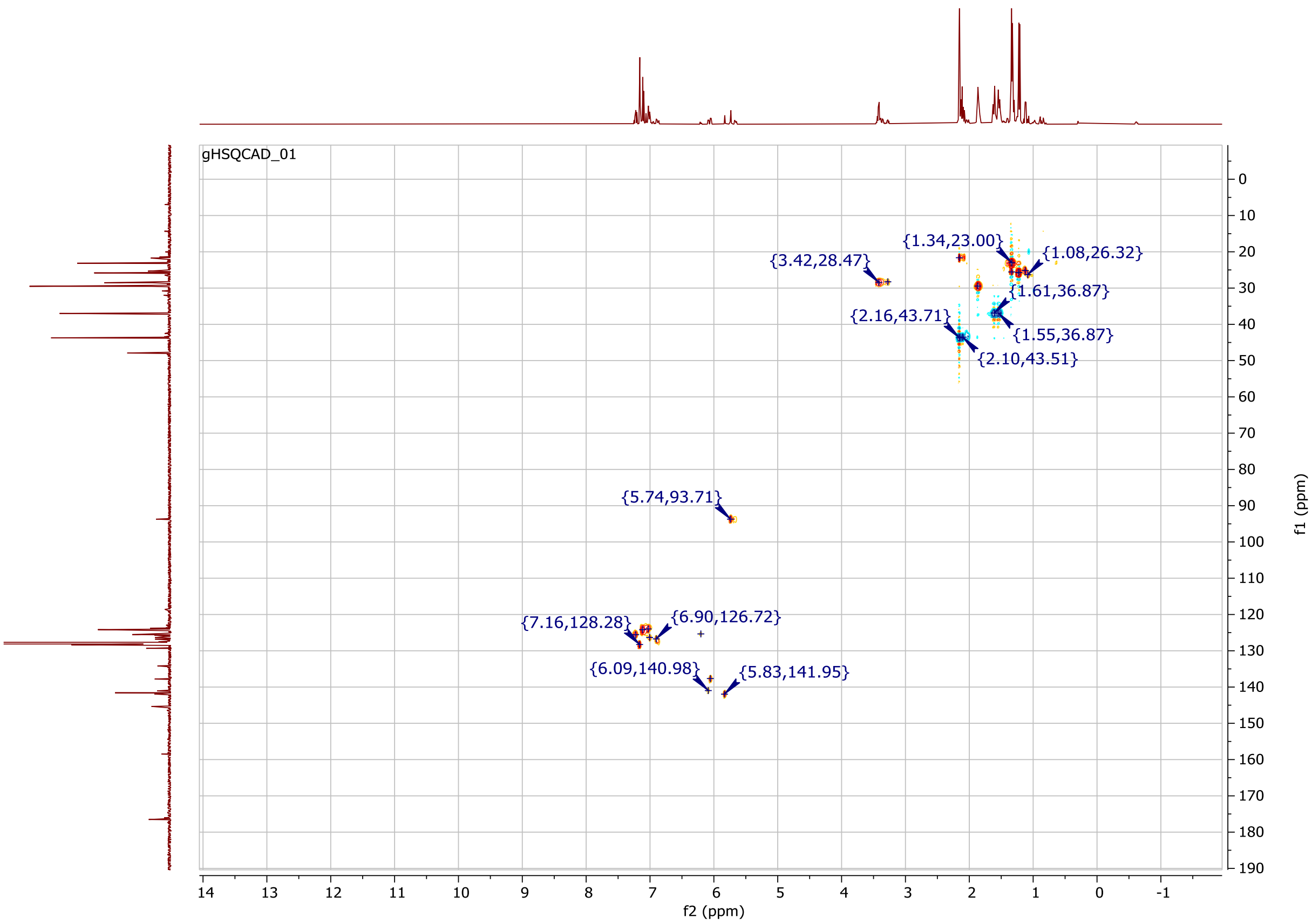

Figure S64: ^{13}C-^{1}H HSQC NMR (126 MHz, C_6D_6).

NMR spectra from 120°C reaction.

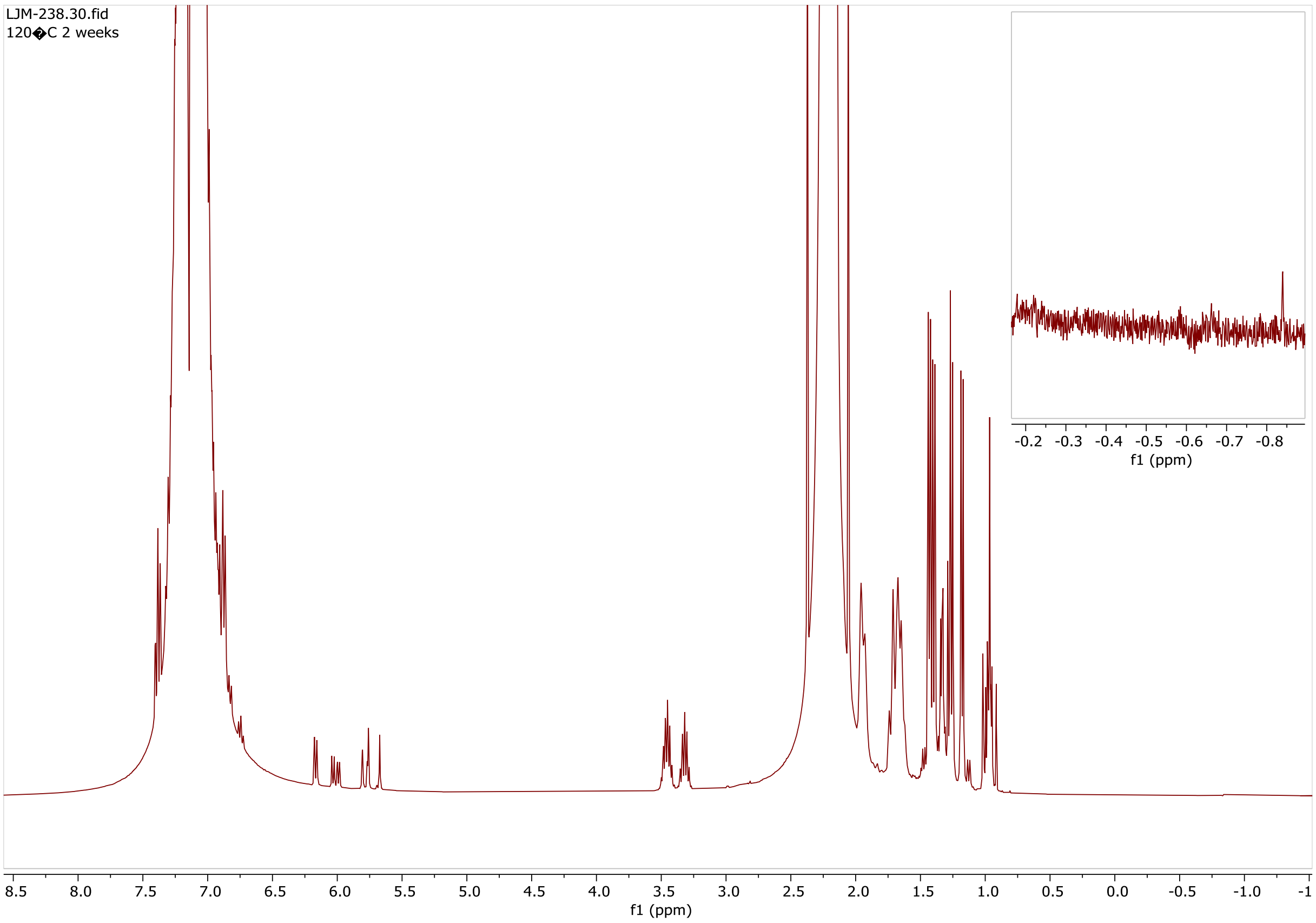

Figure S65: Crude ^{1}H NMR spectrum (400 MHz, toluene) after 13 days, indicating quantitative consumption of $\mathbf{1^{Ad}}$.

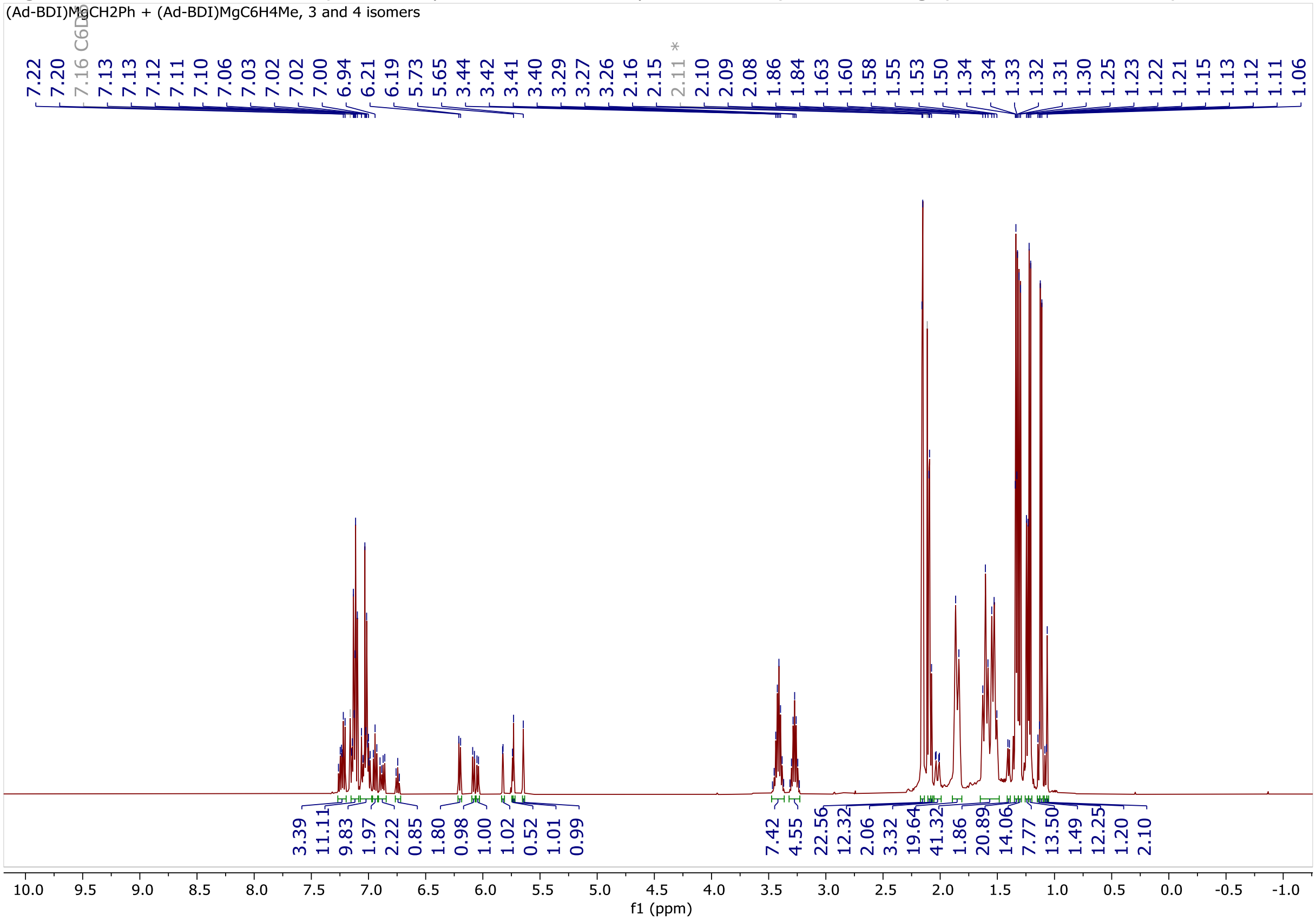

Figure S66: ^{1}H NMR (500 MHz, C_6D_6) of the product mixture after removal of volatiles. *Toluene.

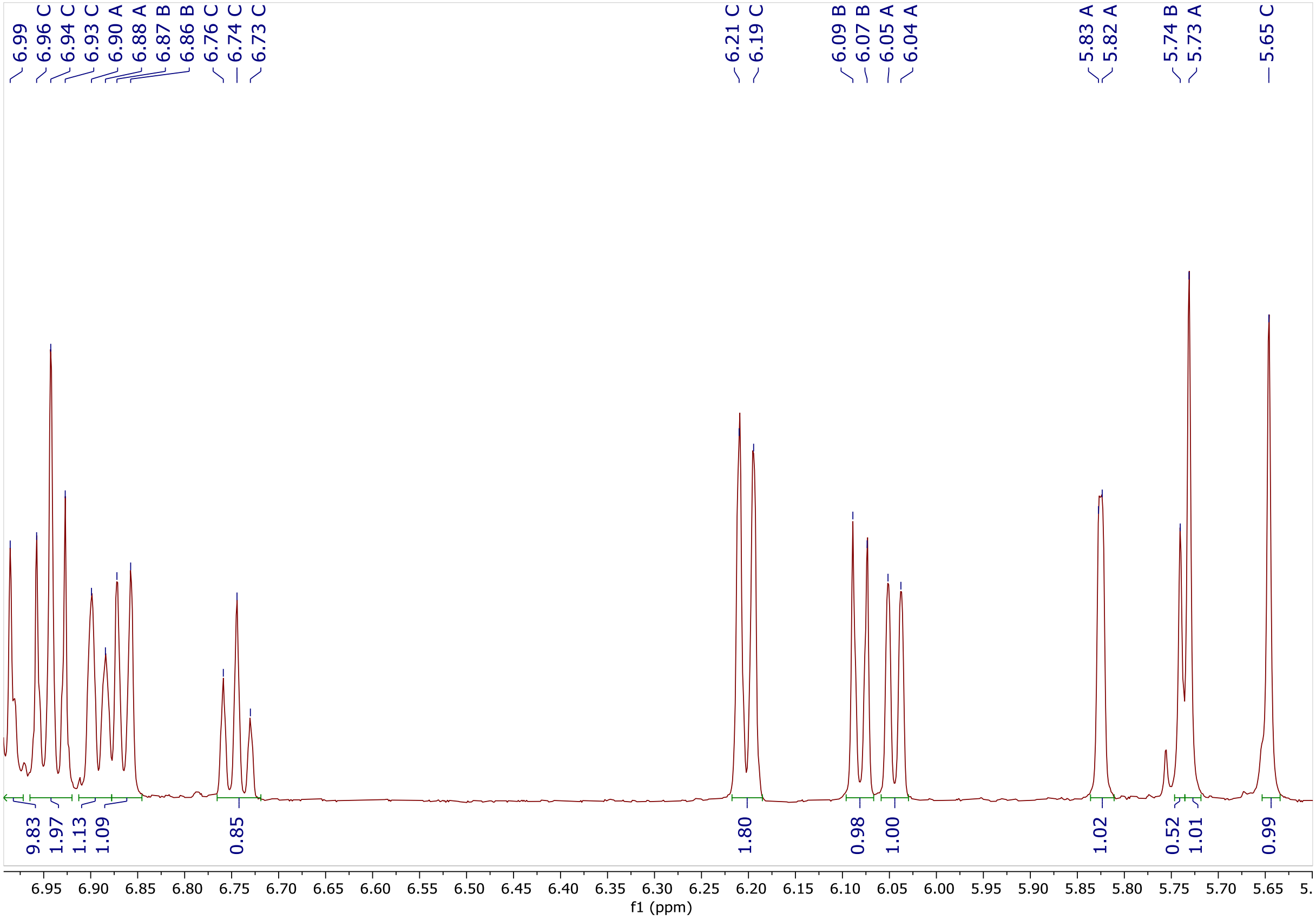

Figure S67: Expanded ^{1}H NMR (500 MHz, C_6D_6) of the product mixture after removal of volatiles. *Toluene.

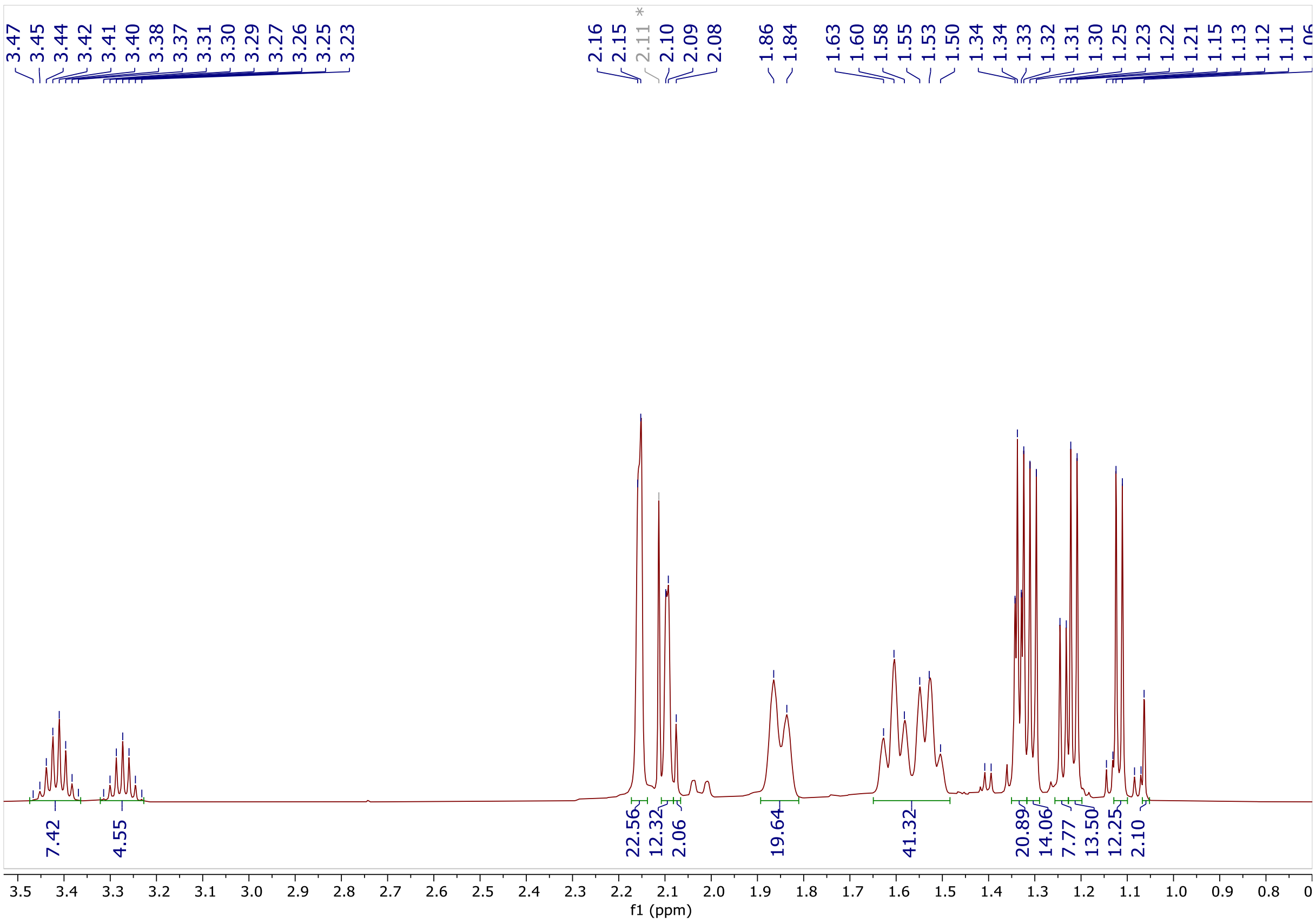

Figure S68: Expanded ^{1}H NMR (500 MHz, C_6D_6) of the product mixture after removal of volatiles. *Toluene.

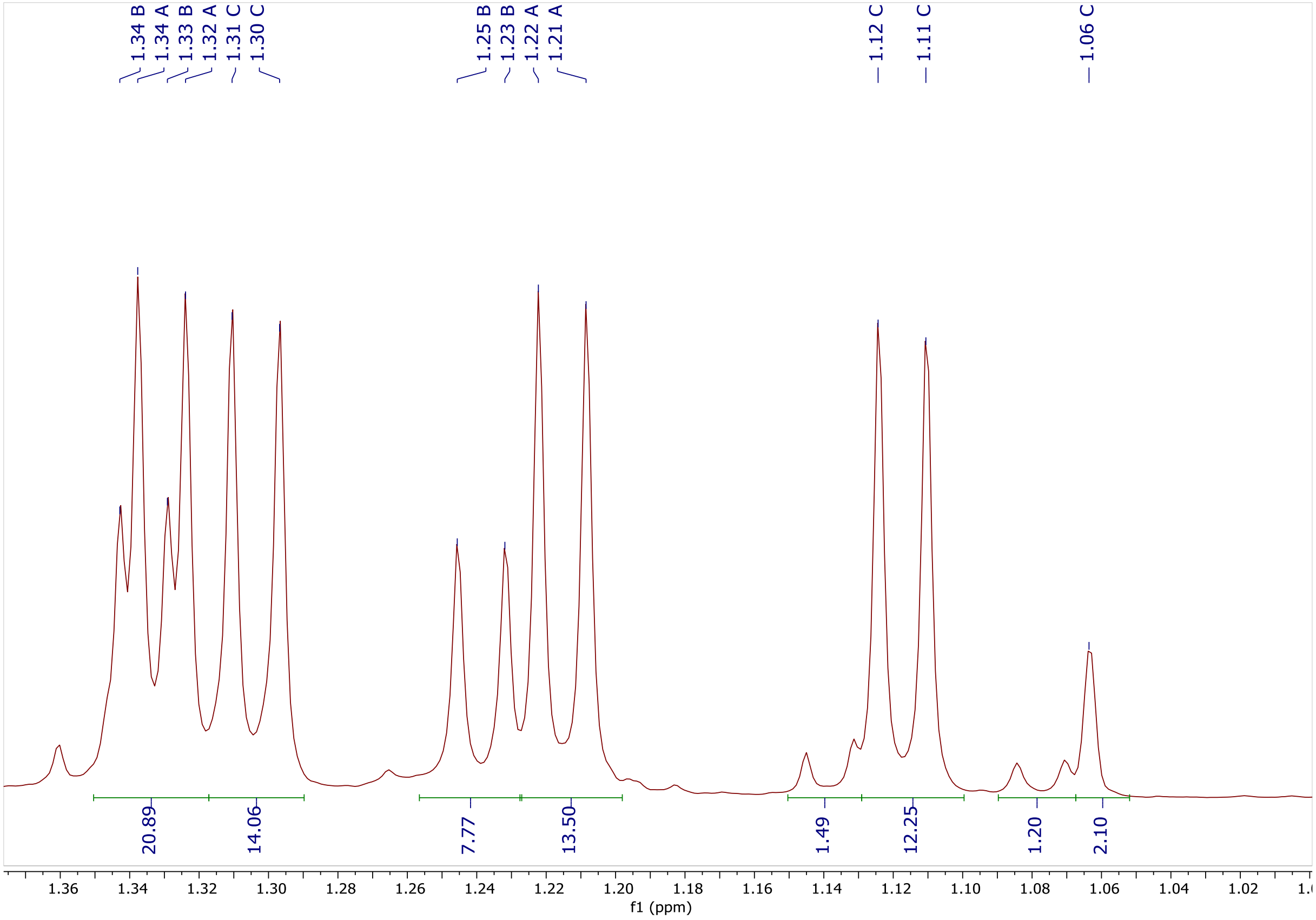

Figure S69: Expanded ^1H NMR (500 MHz, C_6D_6) of the product mixture after removal of volatiles.

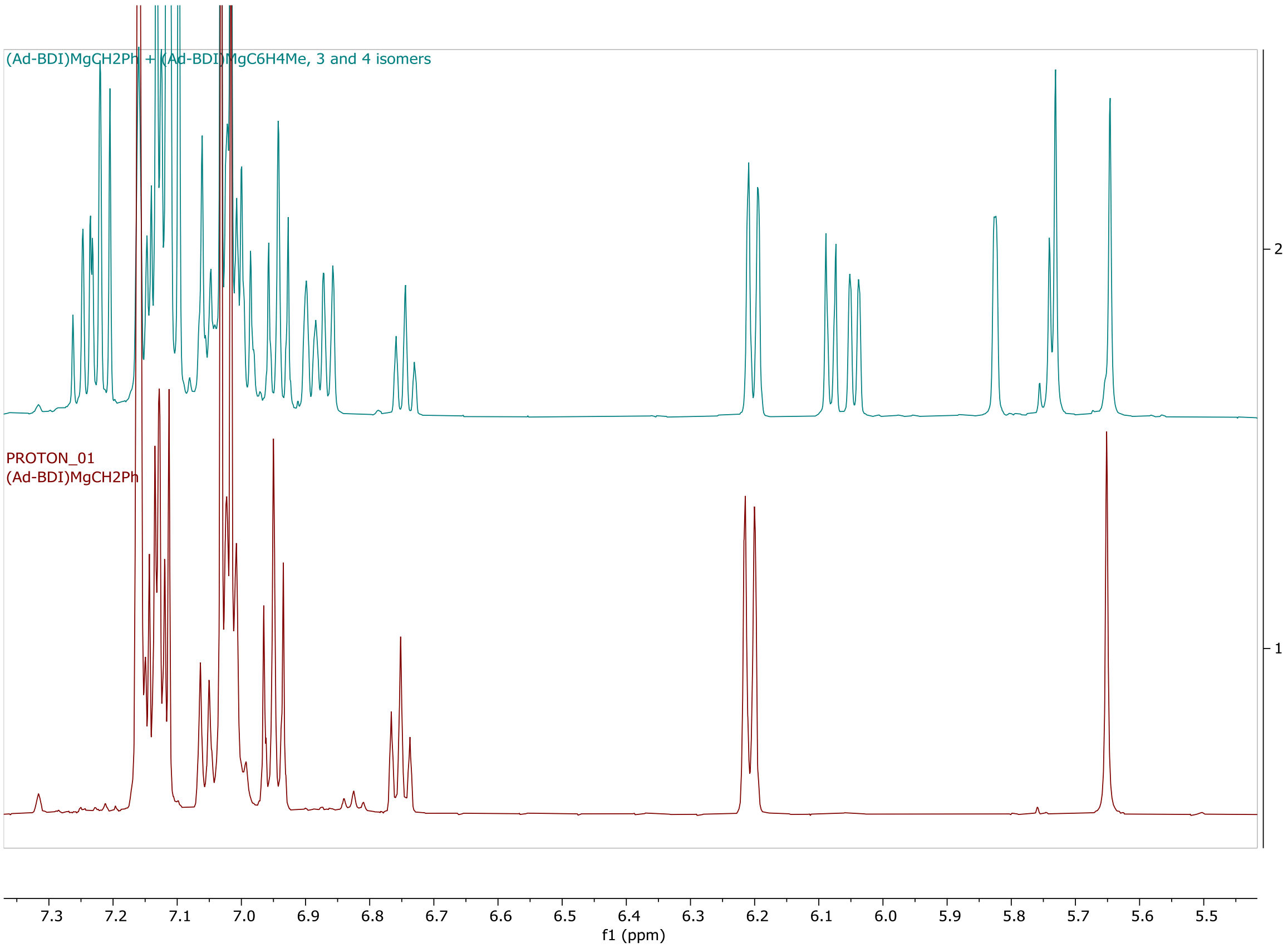

Figure S70: Stacked expanded ^1H NMR spectra (500 MHz, C_6D_6) of mixture of isomers obtained from toluene deprotonation (top) and of **5'** synthesised separately from [(^{Ad}BDI)MgBr] (**13**) and KCH_2Ph (bottom).

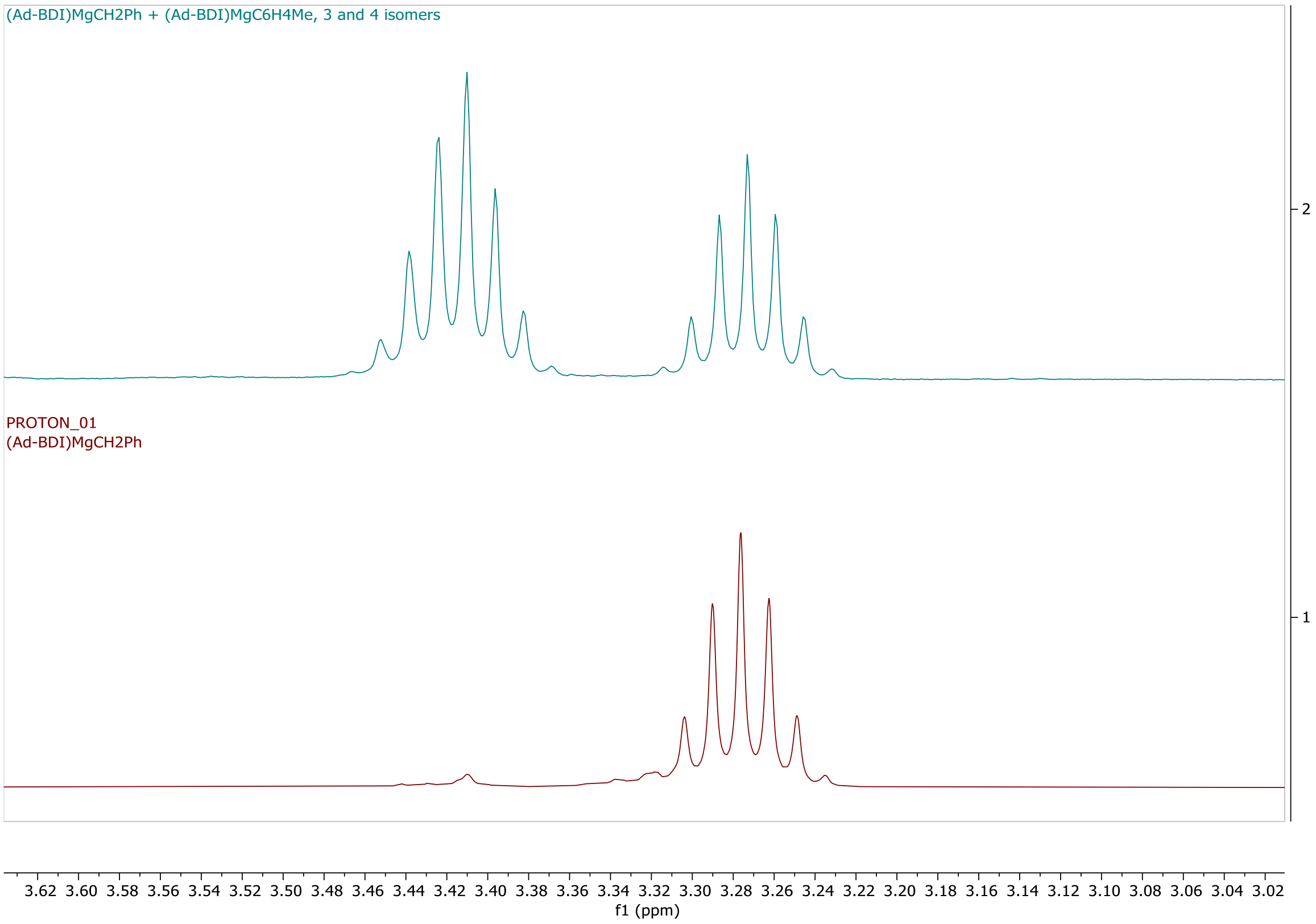

Figure S71: Stacked expanded ^{1}H NMR spectra (500 MHz, C_6D_6) of mixture of isomers obtained from toluene deprotonation (top) and of **5'** synthesised separately from [(^{Ad}BDI)MgBr] (**13**) and KCH_2Ph (bottom).

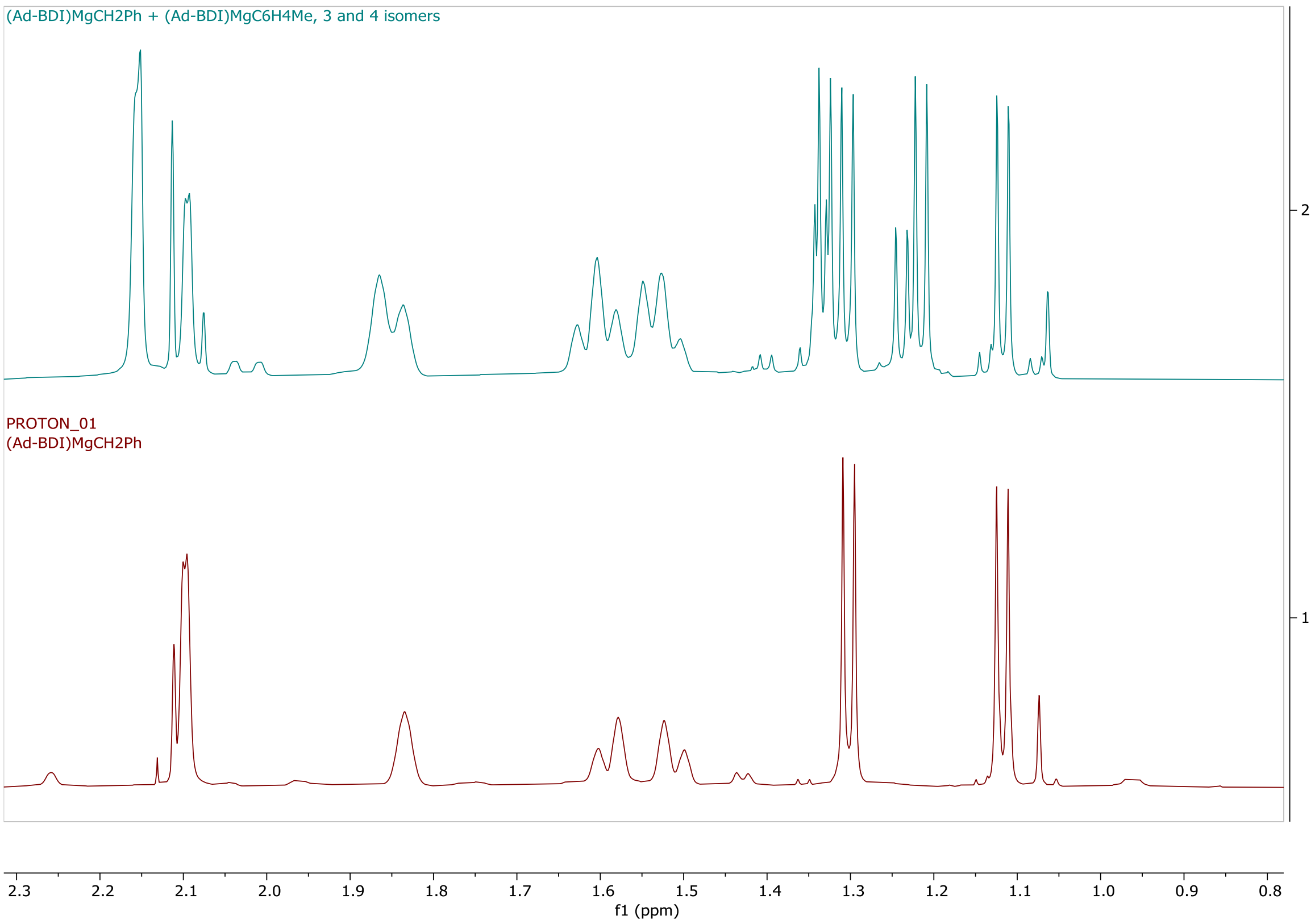

Figure S72: Stacked expanded ^{1}H NMR spectra (500 MHz, C_6D_6) of mixture of isomers obtained from toluene deprotonation (top) and of **5'** synthesised separately from [(^{Ad}BDI)MgBr] (**13**) and KCH_2Ph (bottom).

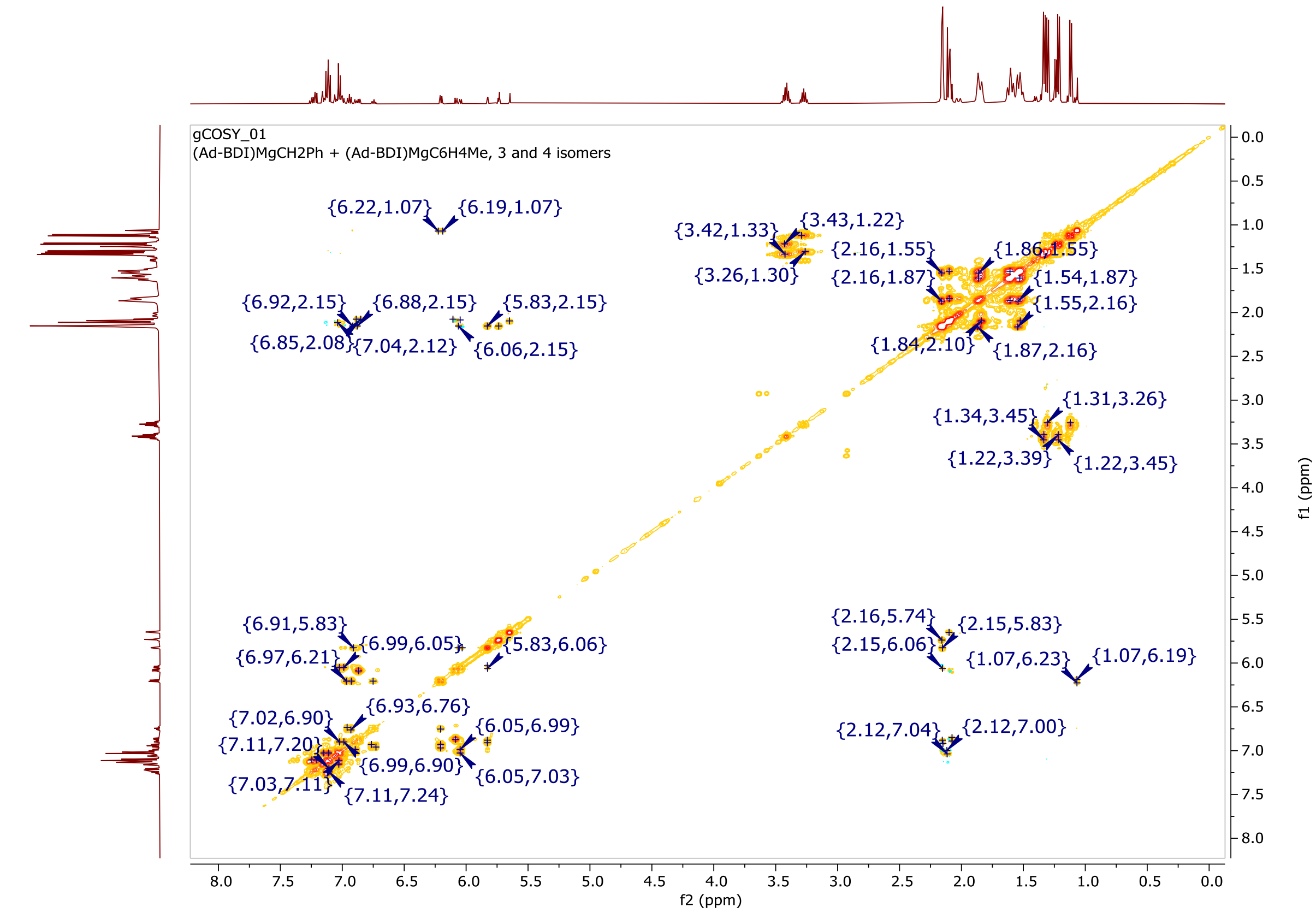

Figure S73: ^{1}H-^{1}H COSY NMR (500 MHz, C_6D_6).

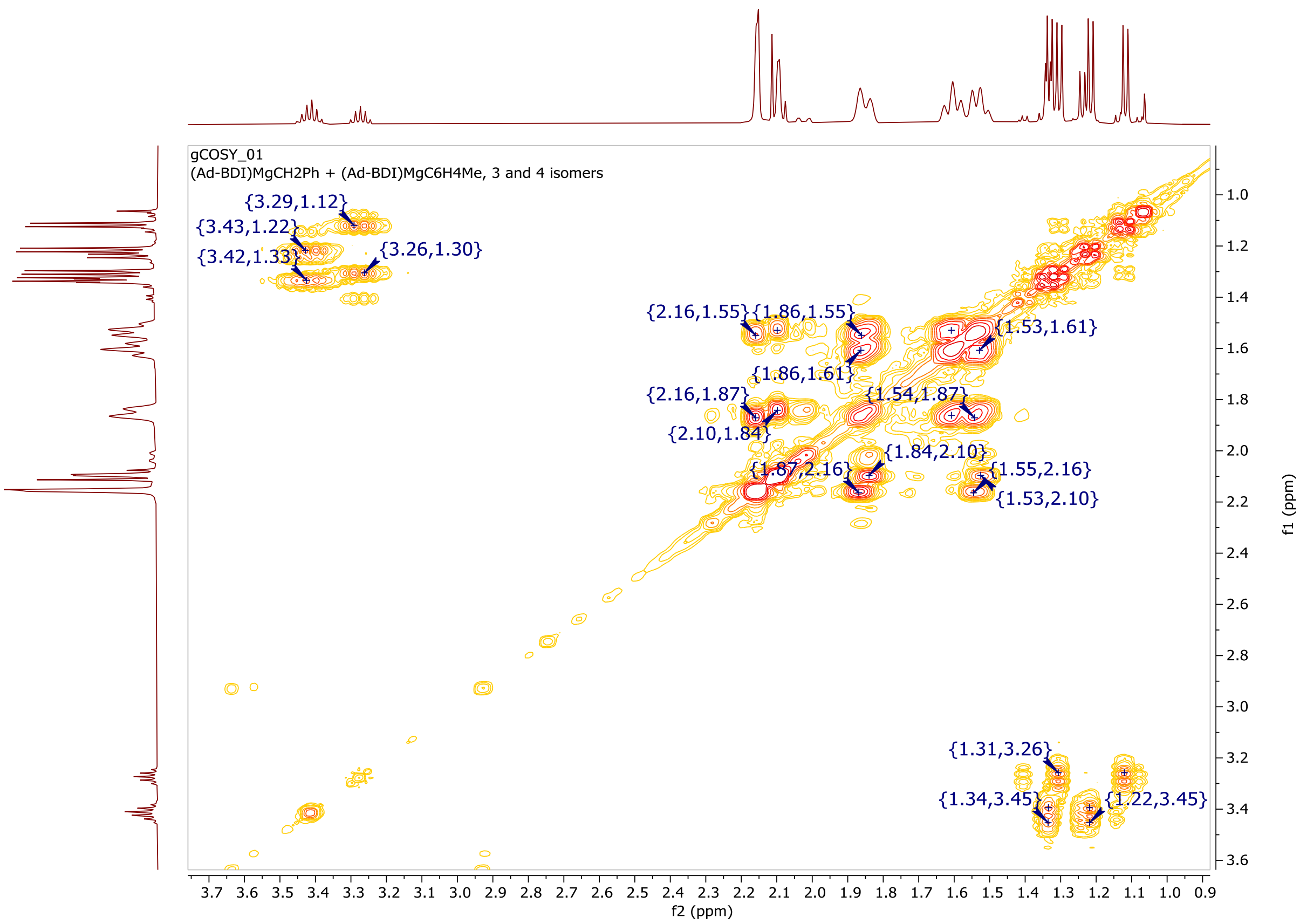

Figure S74: Expanded ^{1}H-^{1}H COSY NMR (500 MHz, C_6D_6).

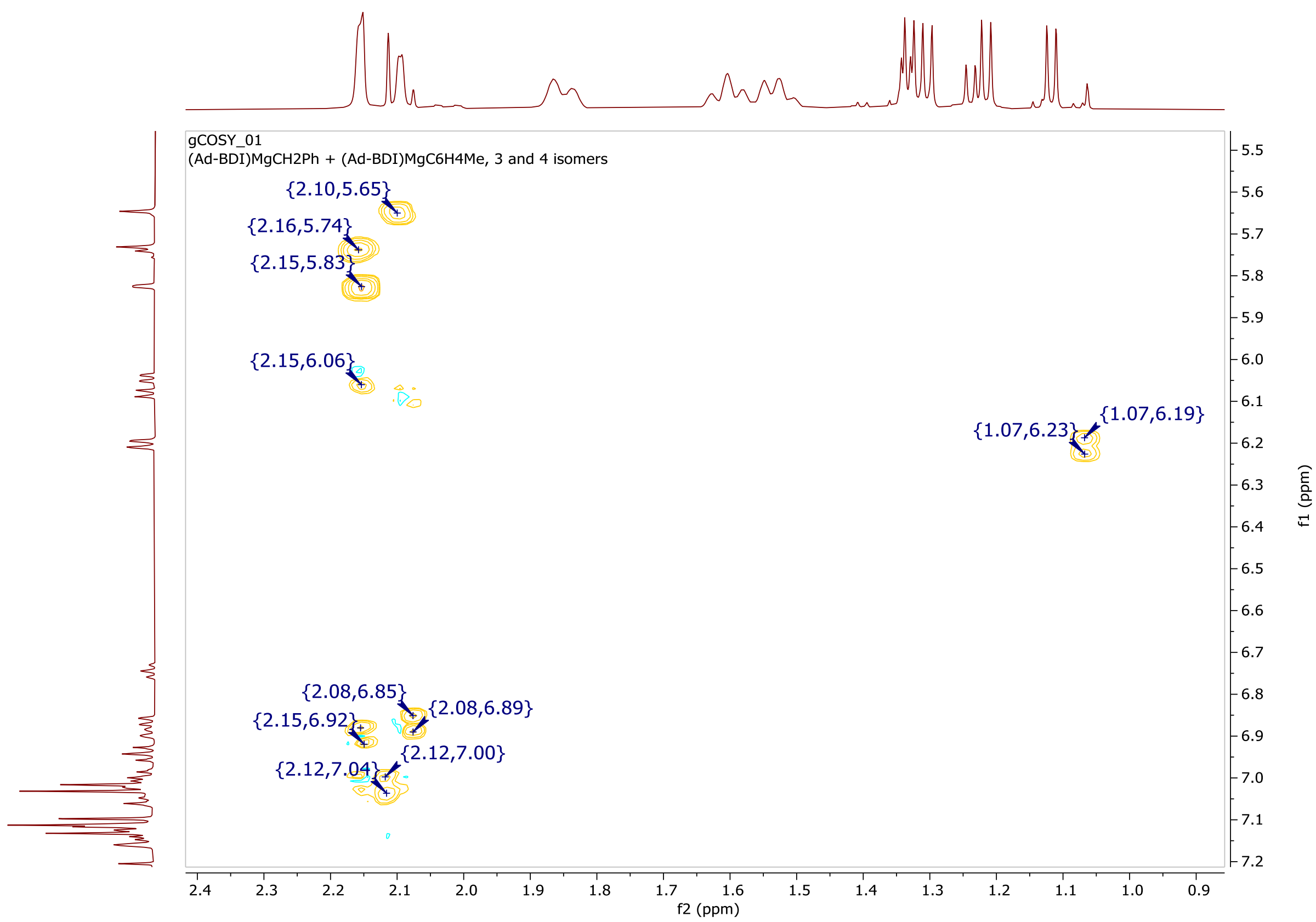

Figure S75: Expanded ^{1}H-^{1}H COSY NMR (500 MHz, C_6D_6).

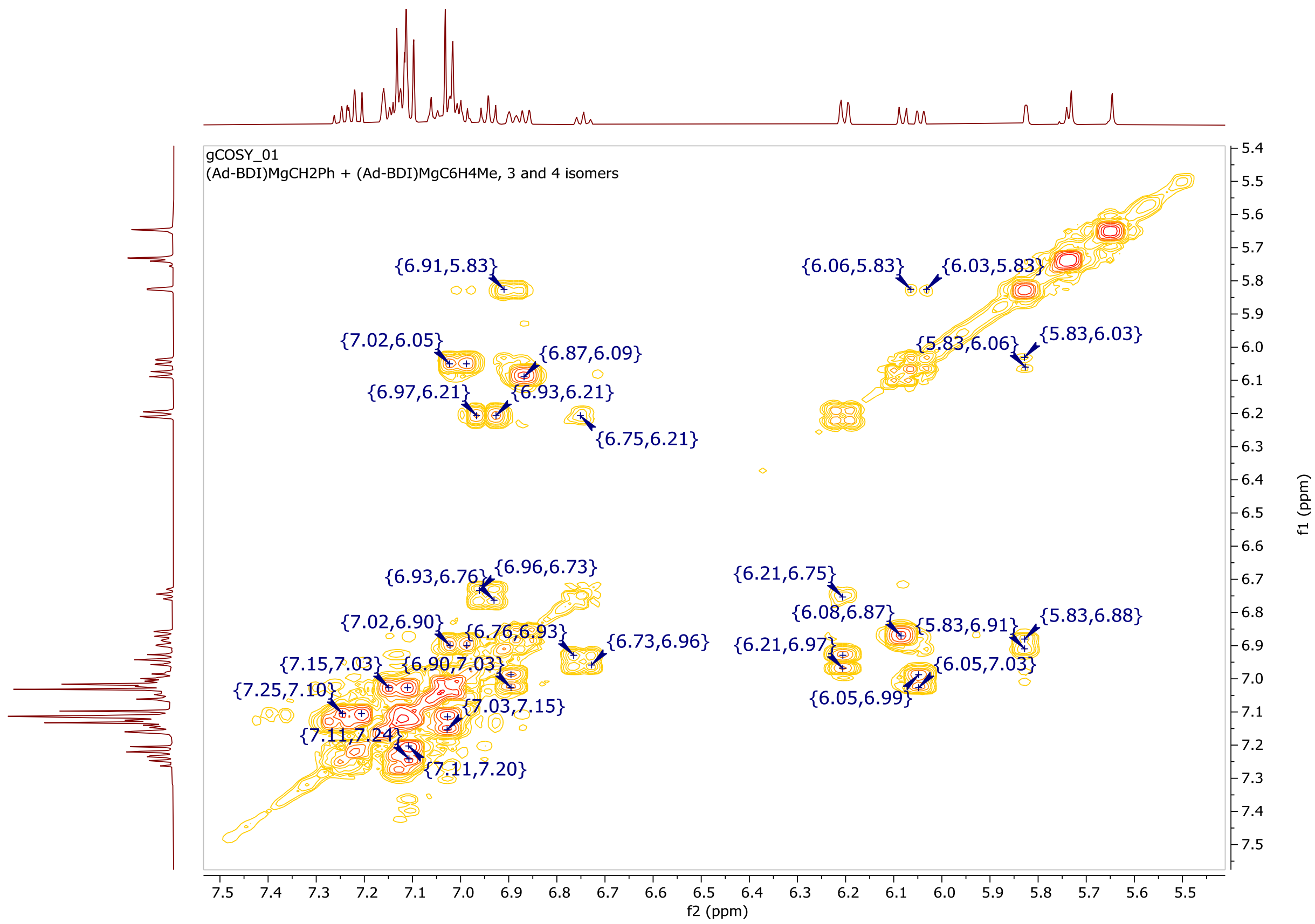

Figure S76: Expanded ^{1}H-^{1}H COSY NMR (500 MHz, C_6D_6).

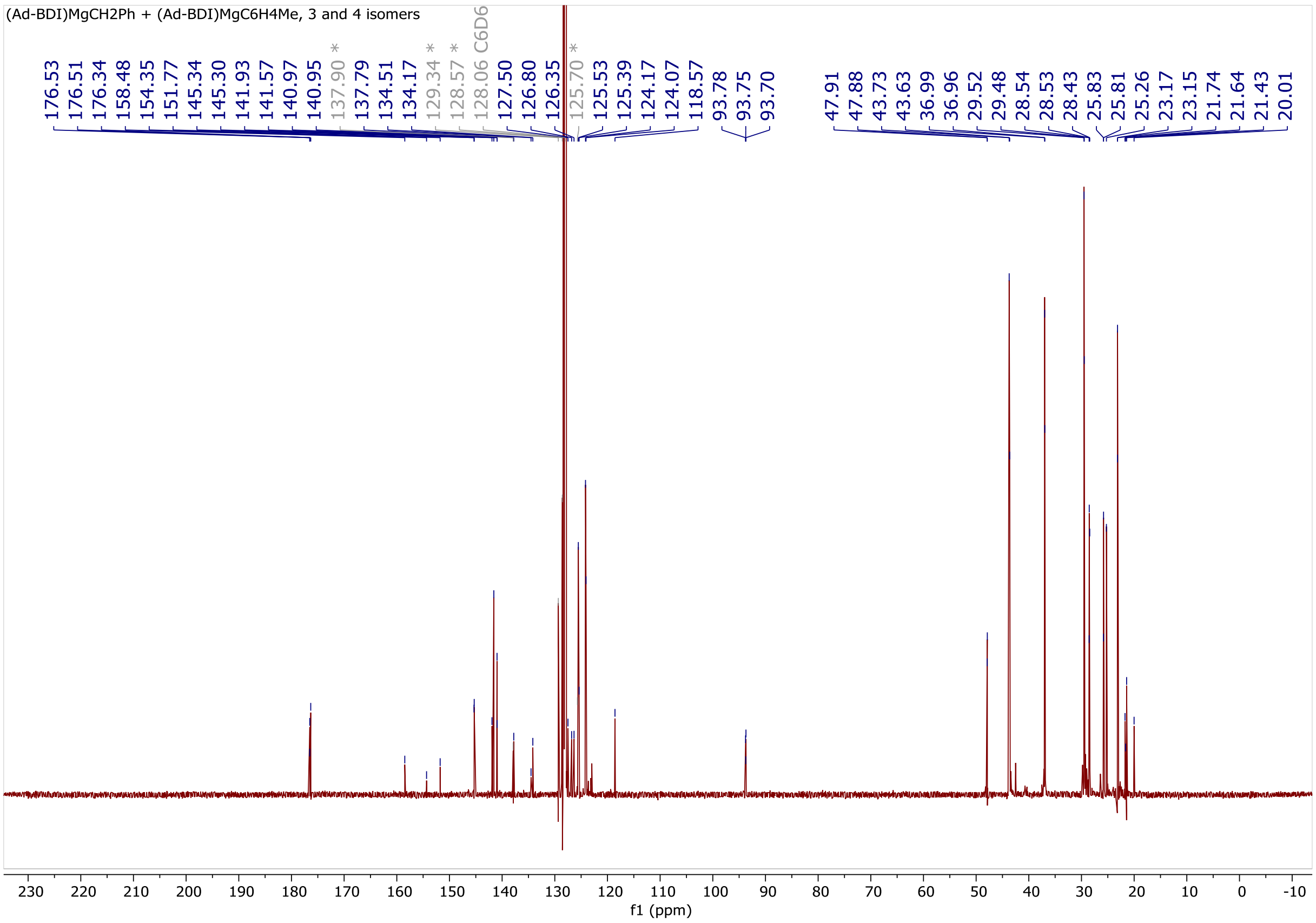

Figure S77: $^{13}C\{^1H\}$ NMR (126 MHz, C_6D_6).

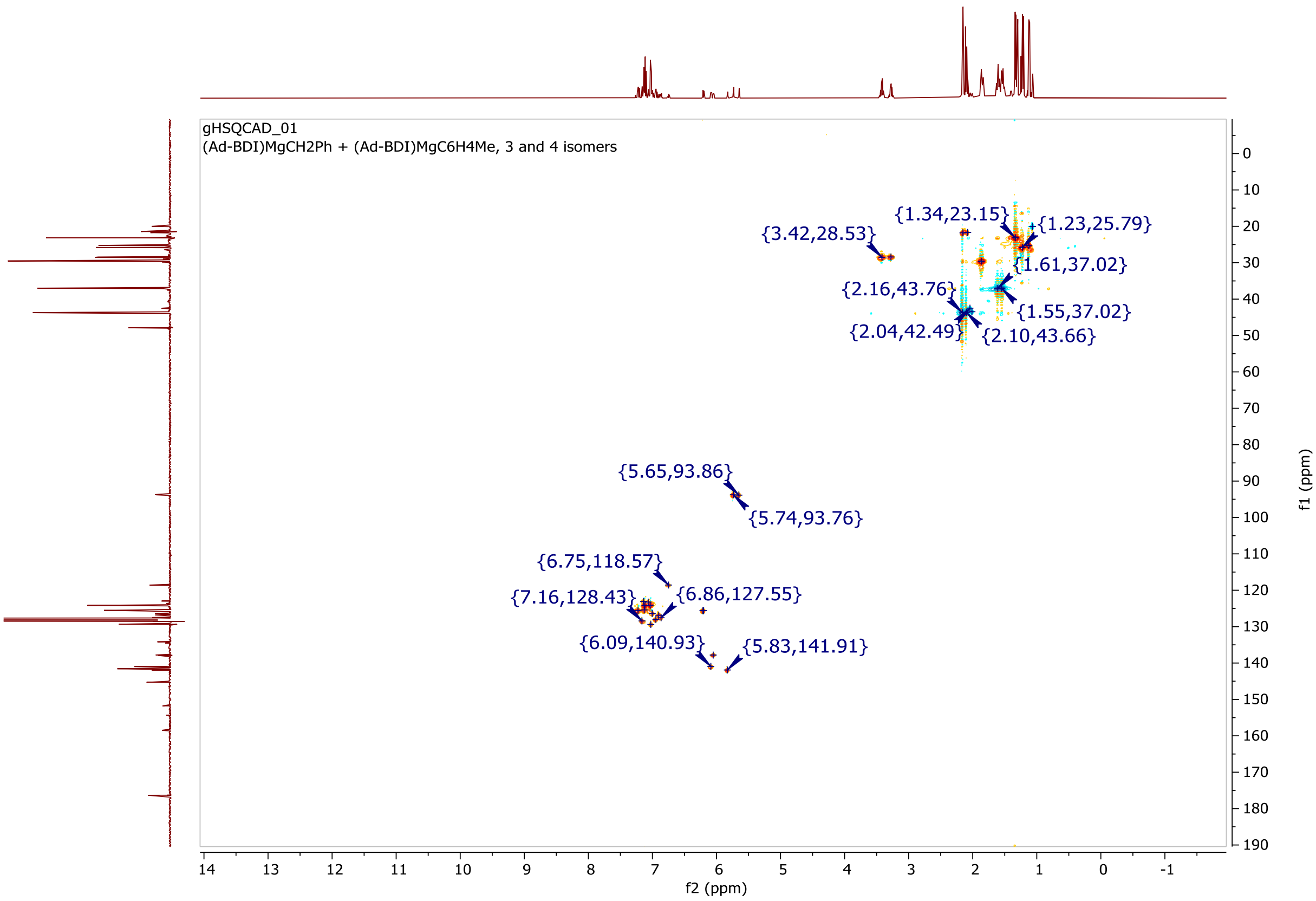

Figure S78: ^{13}C-^1H HSQC (126 MHz, C_6D_6).

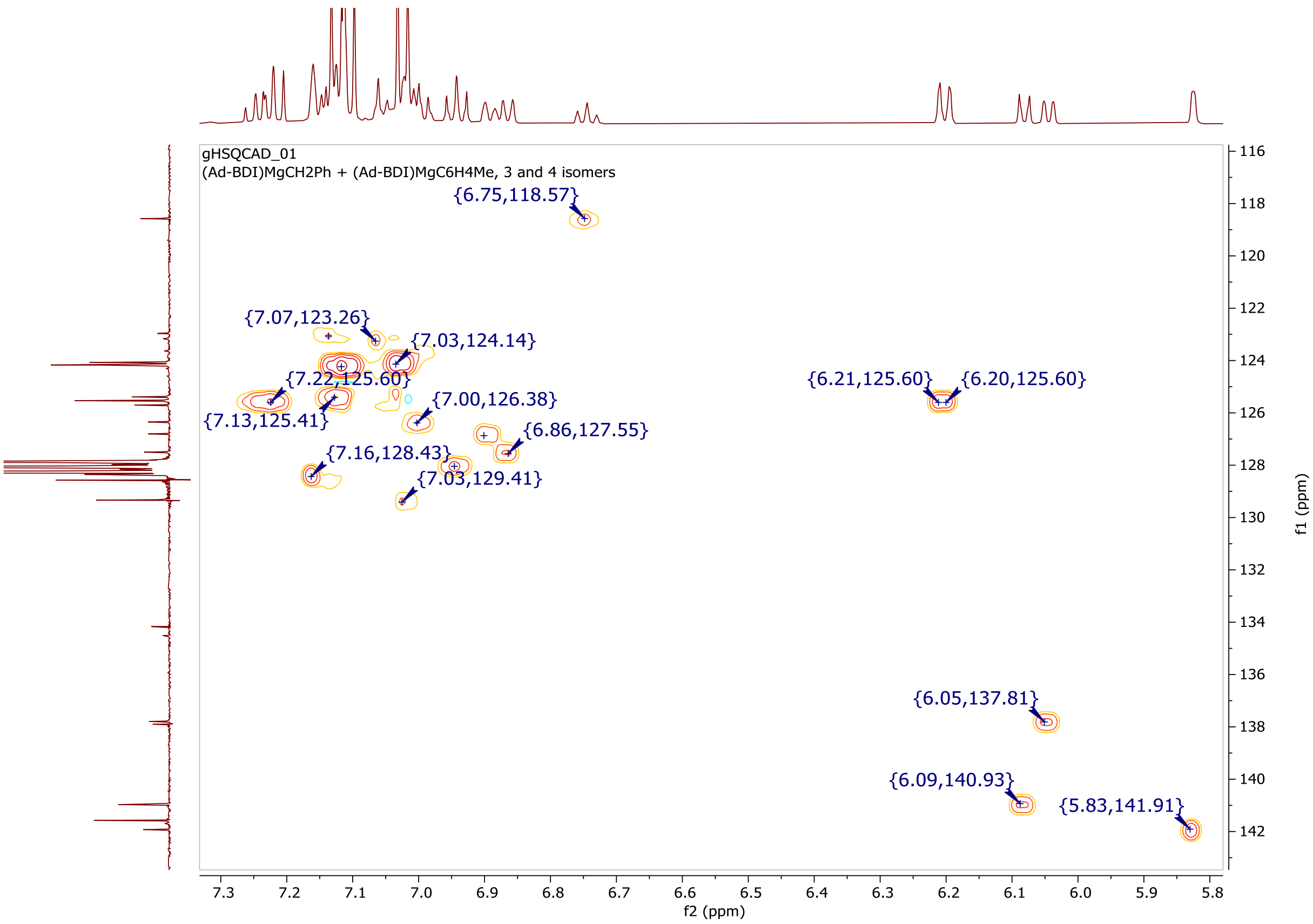

Figure S79: Expanded ^{13}C-^{1}H HSQC (126 MHz, C_6D_6).

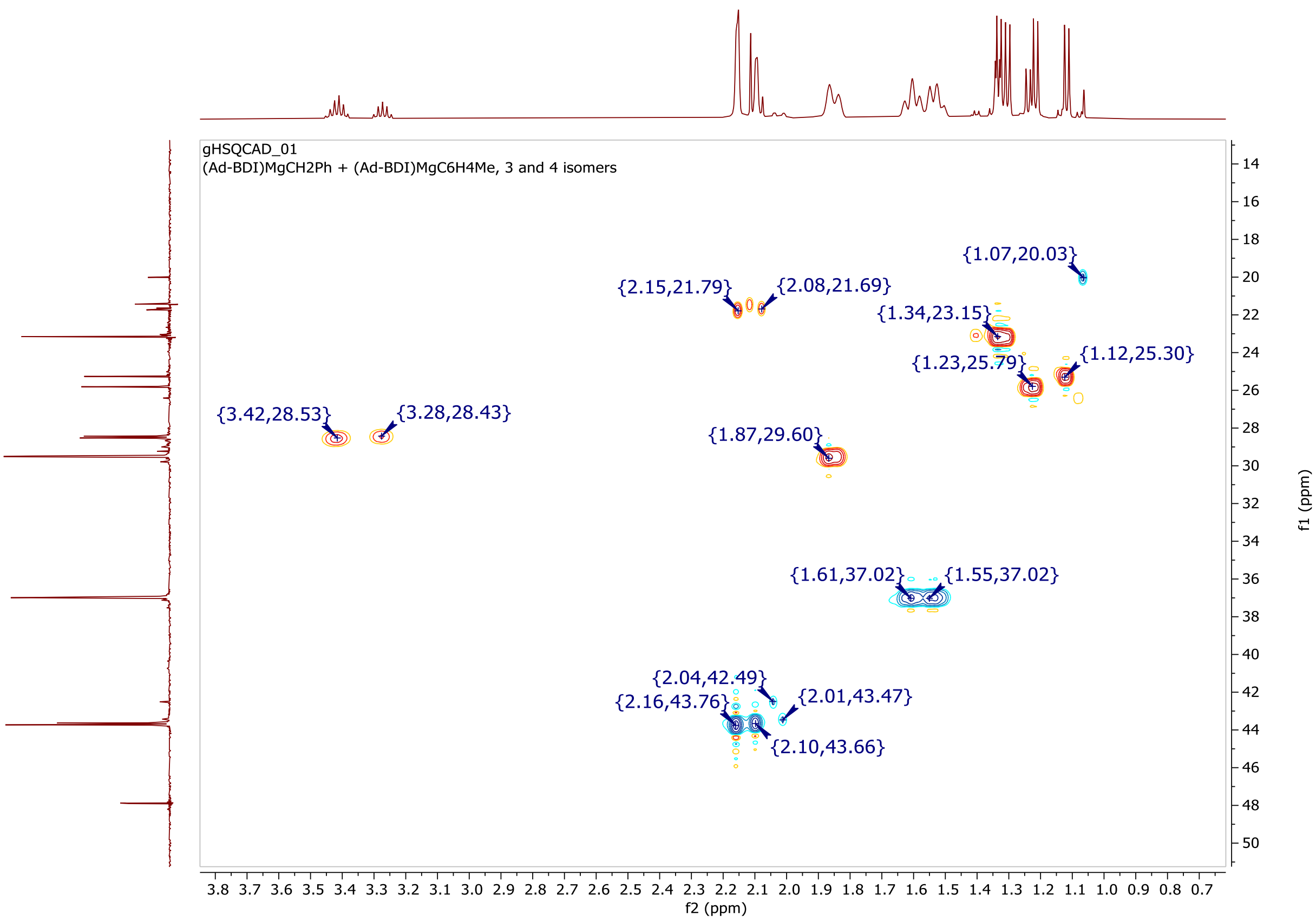

Figure S80: Expanded ^{13}C-^{1}H HSQC (126 MHz, C_6D_6).

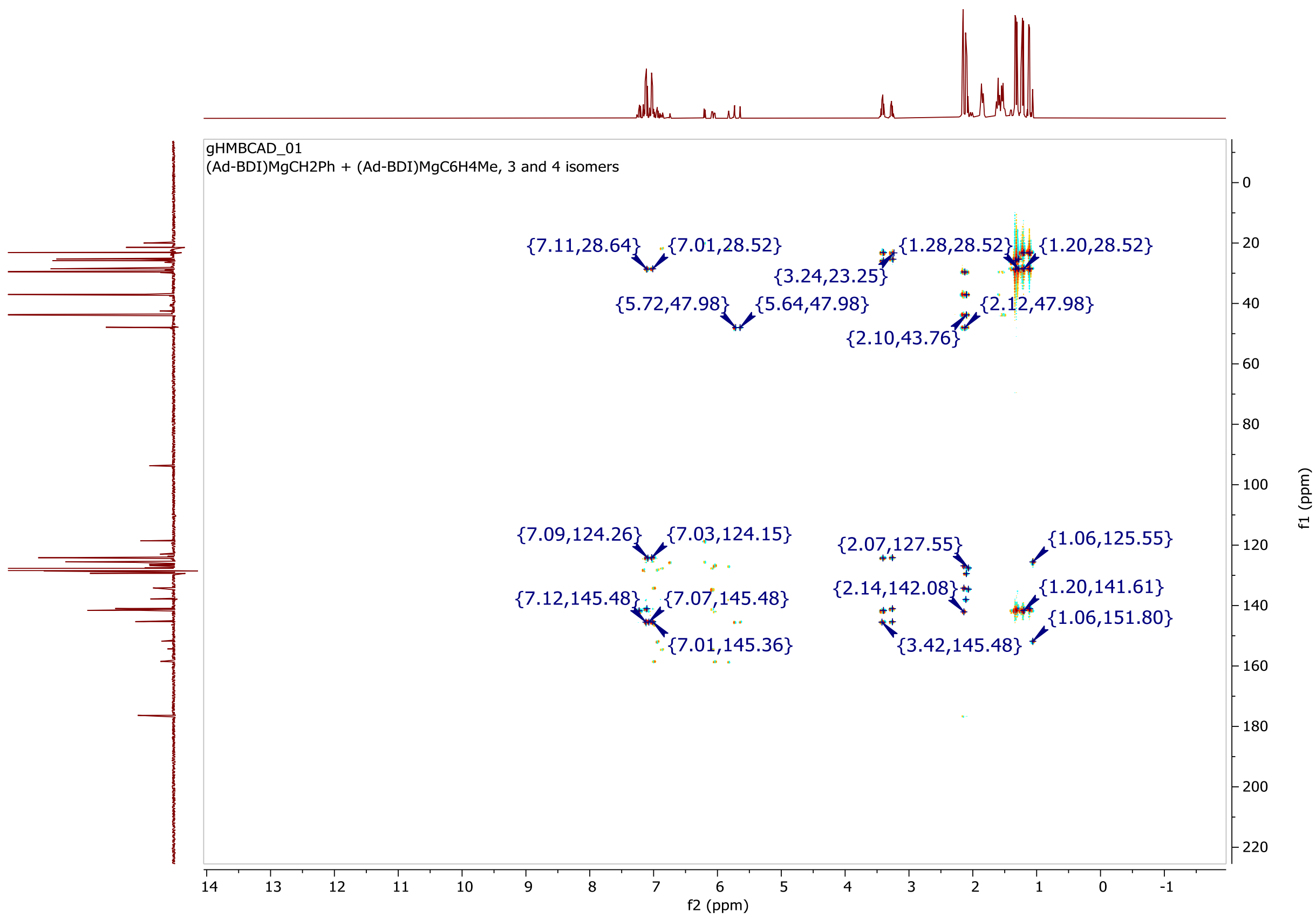

Figure S81: ^{13}C-^{1}H HMBC (126 MHz, C_6D_6).

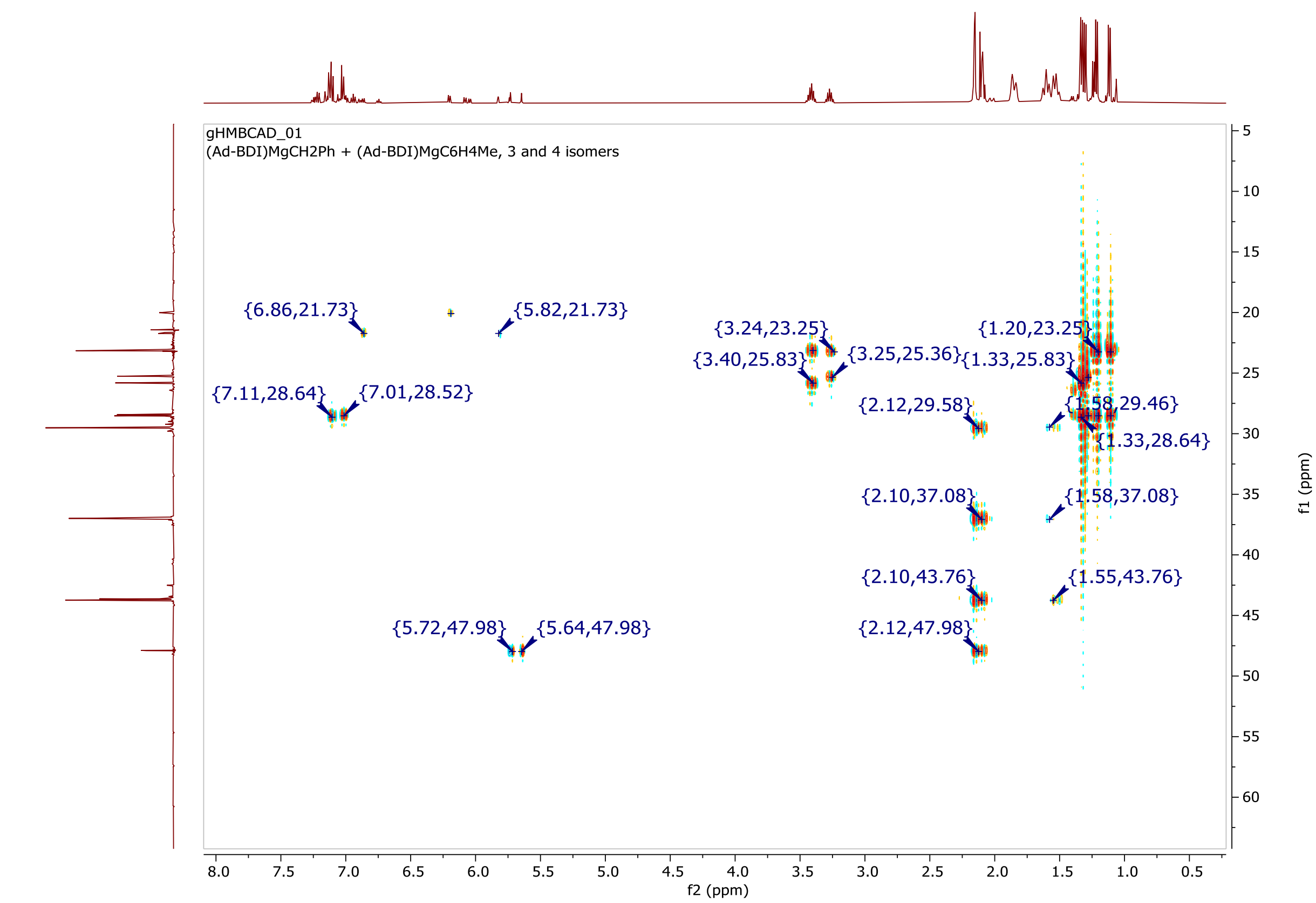

Figure S82: Expanded ^{13}C-^{1}H HMBC (126 MHz, C_6D_6).

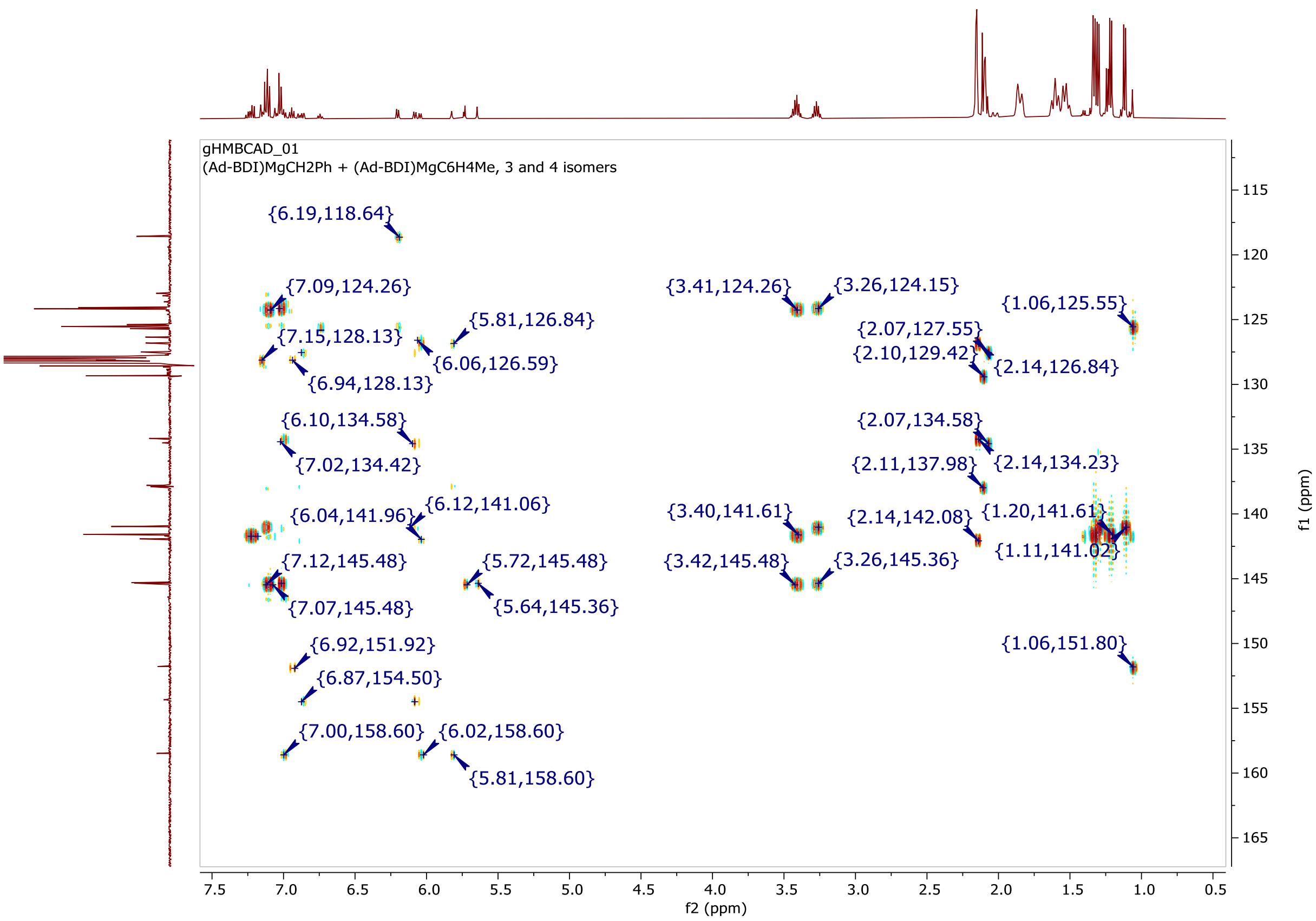

Figure S83: Expanded ^{13}C-^{1}H HMBC (126 MHz, C_6D_6).

2.12 Synthesis of [(^{Ad}BDI)Mg($CH_2C_6H_5$)] (5')

A J. Young's NMR tube was charged with 9.2 mg [(^{Ad}BDI)MgBr] (**13**, 0.012 mmol) and 2 mg KCH_2Ph (0.014 mmol). Toluene (0.6 ml) was added, then the reaction mixture was heated at 60°C for 3 h. After removing volatiles under vacuum, the crude product was extracted into benzene-d_6 and filtered. Analysis by ^{1}H NMR spectroscopy indicated the selective formation of **5'**. Slow evaporation of the benzene solvent provided an oily yellow residue, to which n-pentane was added. The concentrated yellow solution was stored at -35°C, to provide pale-yellow block-shaped single crystals of **5'** suitable for X-ray diffraction analysis. 9 mg yield, 96%.

Satisfactory elemental analysis for **5'** was not obtained due to high sensitivity of the compound, and difficulty in obtaining sufficient quantities of the (highly soluble) pure crystalline compound in the absence of a synthetic route towards synthetically useful quantities of **13.**

^{1}H NMR (500 MHz, C_6D_6) δ 7.16 – 7.09 (m, 2H, *para*-$C_6\underline{H_3}$-*i*Pr_2),* 7.02 (d, *J* = 7.5 Hz, 4H, *meta*-$C_6\underline{H_3}$-*i*Pr_2),* 6.95 (t, *J* = 7.6 Hz, 2H, *meta*-$CH_2C_6\underline{H_5}$), 6.75 (tt, *J* = 7.3, 1.3 Hz, 1H *para*-$CH_2C_6\underline{H_5}$), 6.21 (dd, *J* = 7.9, 1.0 Hz, 2H, *ortho*-$CH_2C_6\underline{H_5}$), 5.65 (s, 1H,), 3.28 (hept, *J* = 7.1 Hz, 4H, *H*C(CH_3)$_2$), 2.10 (d, *J* = 2.9 Hz, 12H, ^{Ad}CC*H*$_2$CH), 1.86 – 1.81 (m, 6H, ^{Ad}C*H*), 1.59 (d, *J* = 12.4 Hz, 6H, ^{Ad}CHC$\underline{H_2}$CH), 1.51 (dt, *J* = 12.3, 2.9 Hz, 6H ^{Ad}CHC$\underline{H_2}$CH), 1.30 (d, *J* = 6.9 Hz, 12H, HC(C*H*$_3$)$_2$), 1.12 (d, *J* = 6.9 Hz, 12H, HC(C*H*$_3$)$_2$), 1.07 (s, 2H, MgC$\underline{H_2}$). *inaccurate integral due to overlap with residual toluene.

^{13}C NMR (126 MHz, C_6D_6) δ 176.32 ($\underline{C}$=N), 151.77 (*ipso*-$CH_2\underline{C_6}H_5$), 145.28 (*ipso*-$\underline{C_6}H_3$-*i*Pr_2), 140.95 (*ortho*-$\underline{C_6}H_3$-*i*Pr_2), 128.57, 128.02 (*meta*-$CH_2\underline{C_6}H_5$), 125.52 (*ortho*-$CH_2\underline{C_6}H_5$), 125.38 (*para*-$\underline{C_6}H_3$-*i*Pr_2), 124.07 (*meta*-$\underline{C_6}H_3$-*i*Pr_2), 118.57 (*para*-$CH_2\underline{C_6}H_5$), 93.78 (, 47.86 (NC$^{Ad}\underline{C}$), 43.62 (^{Ad}C$\underline{C}$H$_2$CH), 36.94 (^{Ad}CH$\underline{C}$H$_2$CH), 29.45 ($^{Ad}\underline{C}$H), 28.42 (H$\underline{C}$(CH_3)$_2$), 25.27 (HC($\underline{C}H_3$)$_2$), 23.16 (HC($\underline{C}H_3$)$_2$), 20.02 (Mg$\underline{C}H_2$).

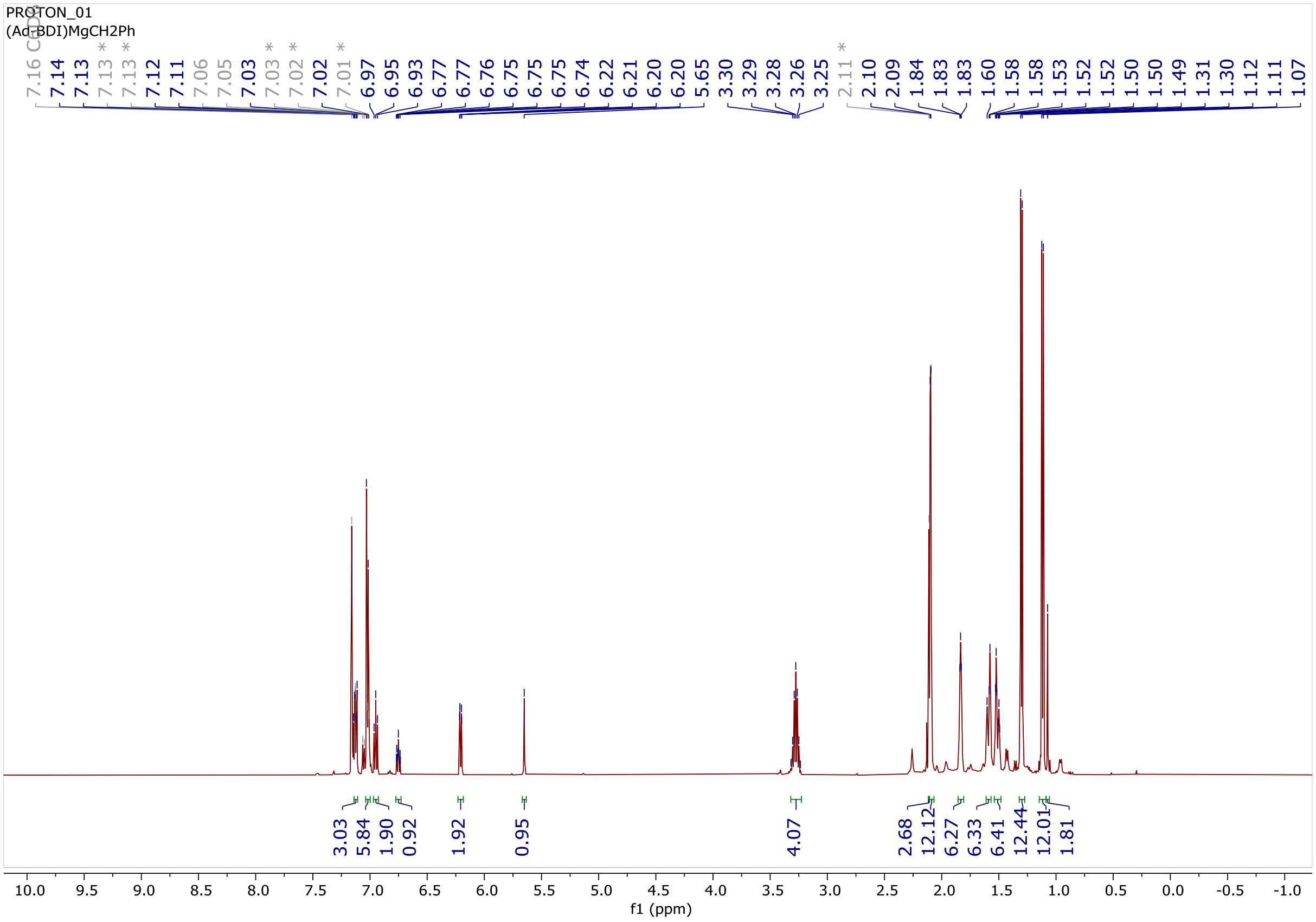

Figure S84: ^{1}H NMR (500 MHz, C_6D_6). *Toluene.

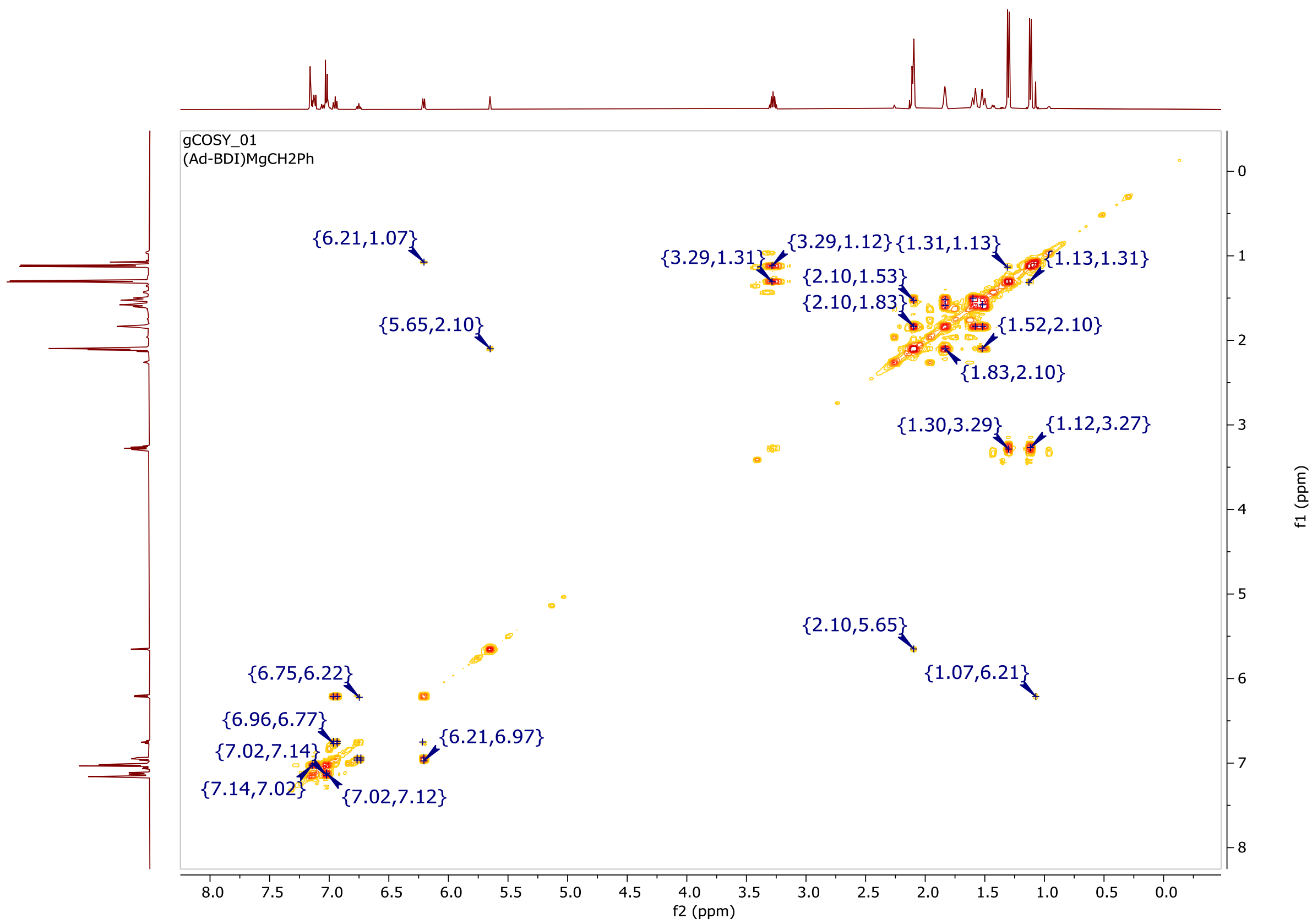

Figure S85: ^{1}H-^{1}H COSY NMR (500 MHz, C_6D_6).

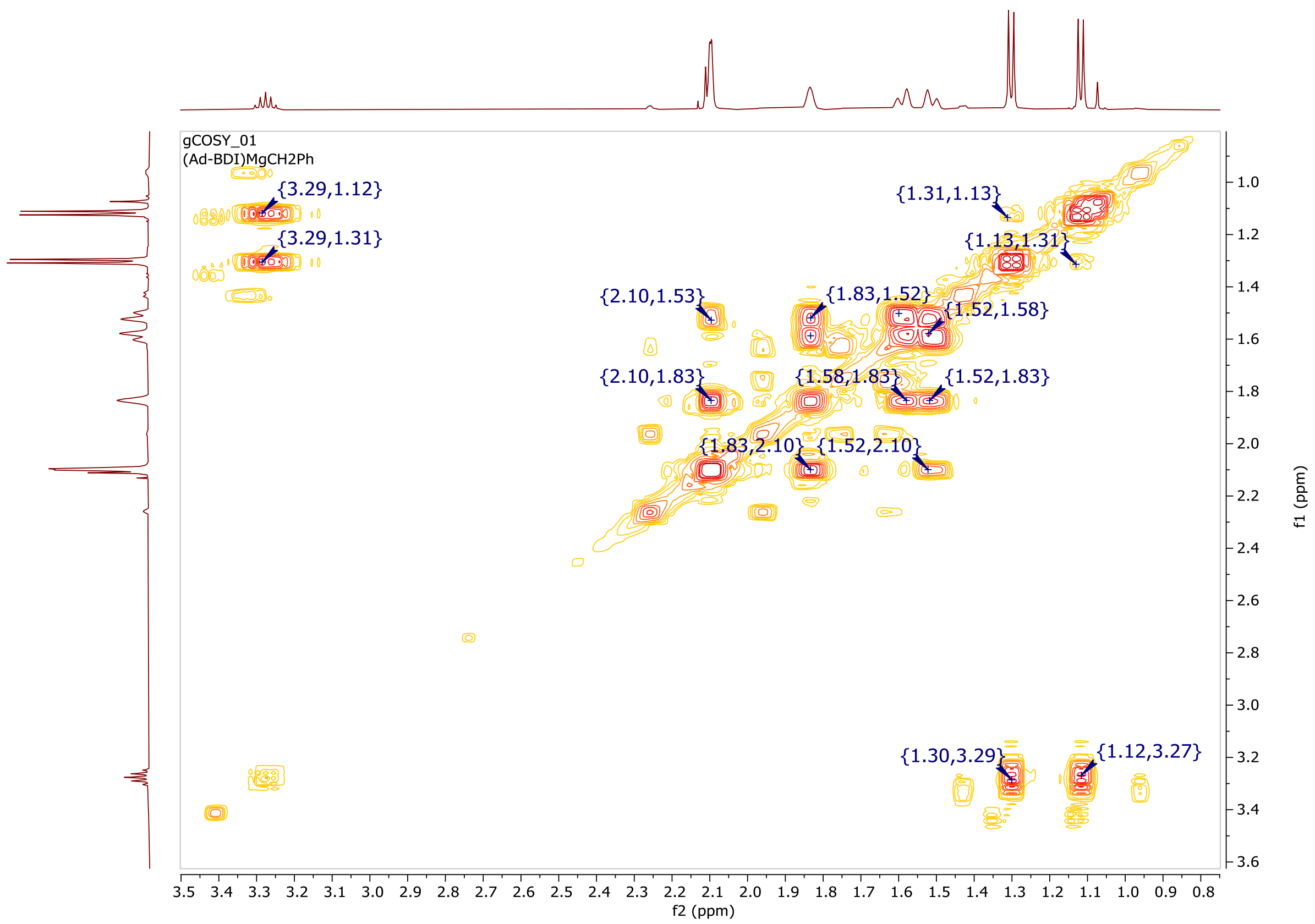

Figure S86: Expanded ^{1}H-^{1}H COSY NMR (500 MHz, C_6D_6).

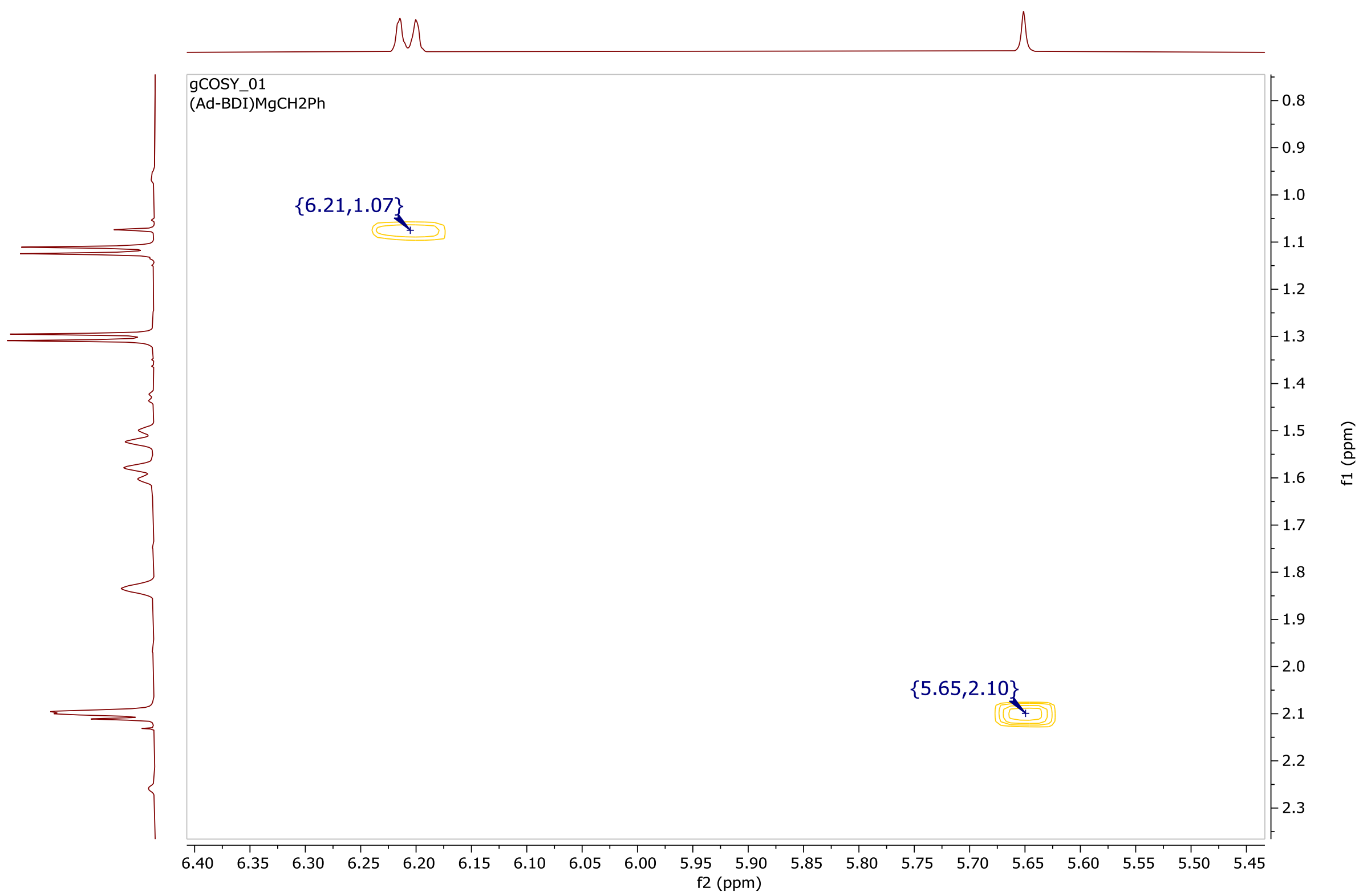

Figure S87: Expanded ^{1}H-^{1}H COSY NMR (500 MHz, C_6D_6).

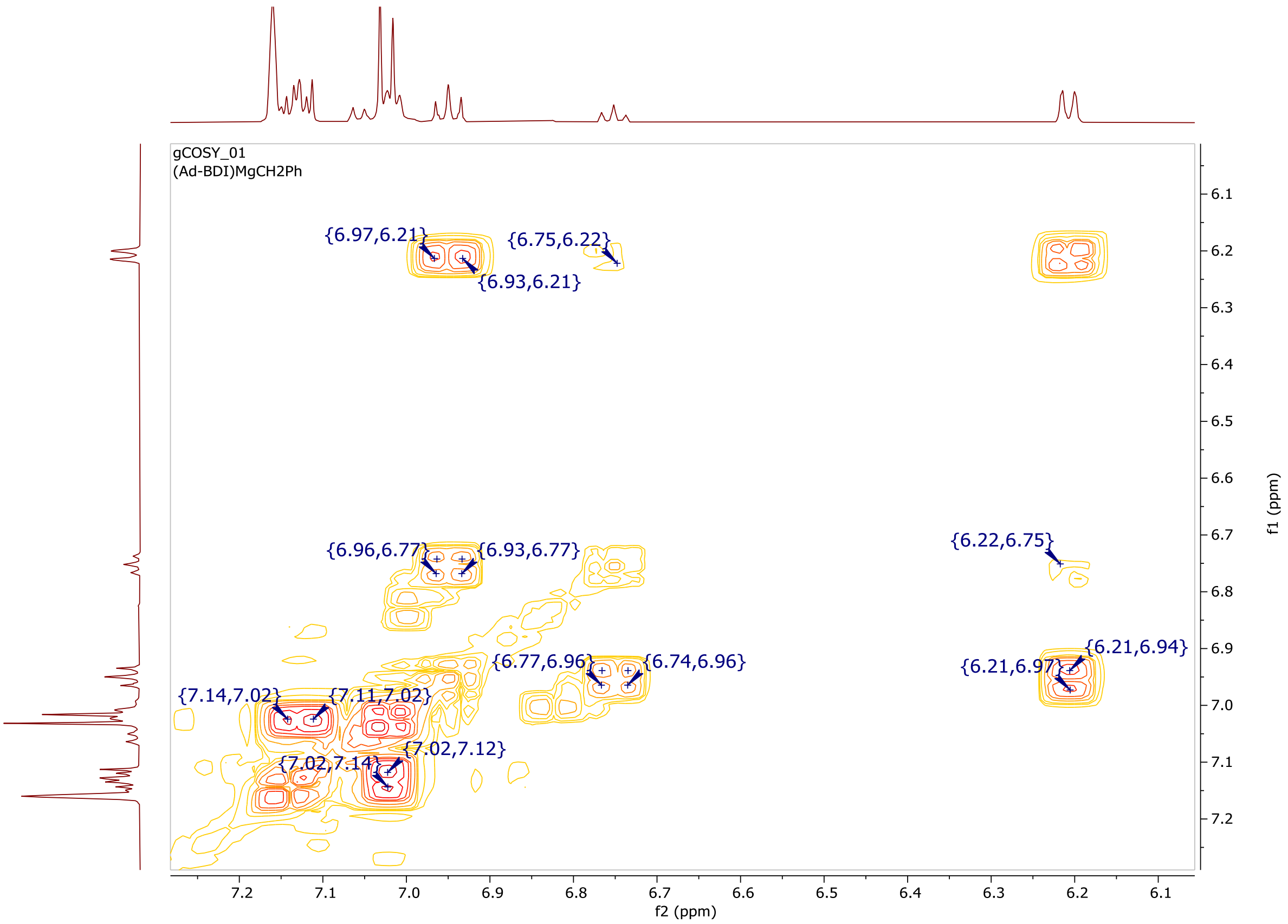

Figure S88: Expanded ^{1}H-^{1}H COSY NMR (500 MHz, C_6D_6).

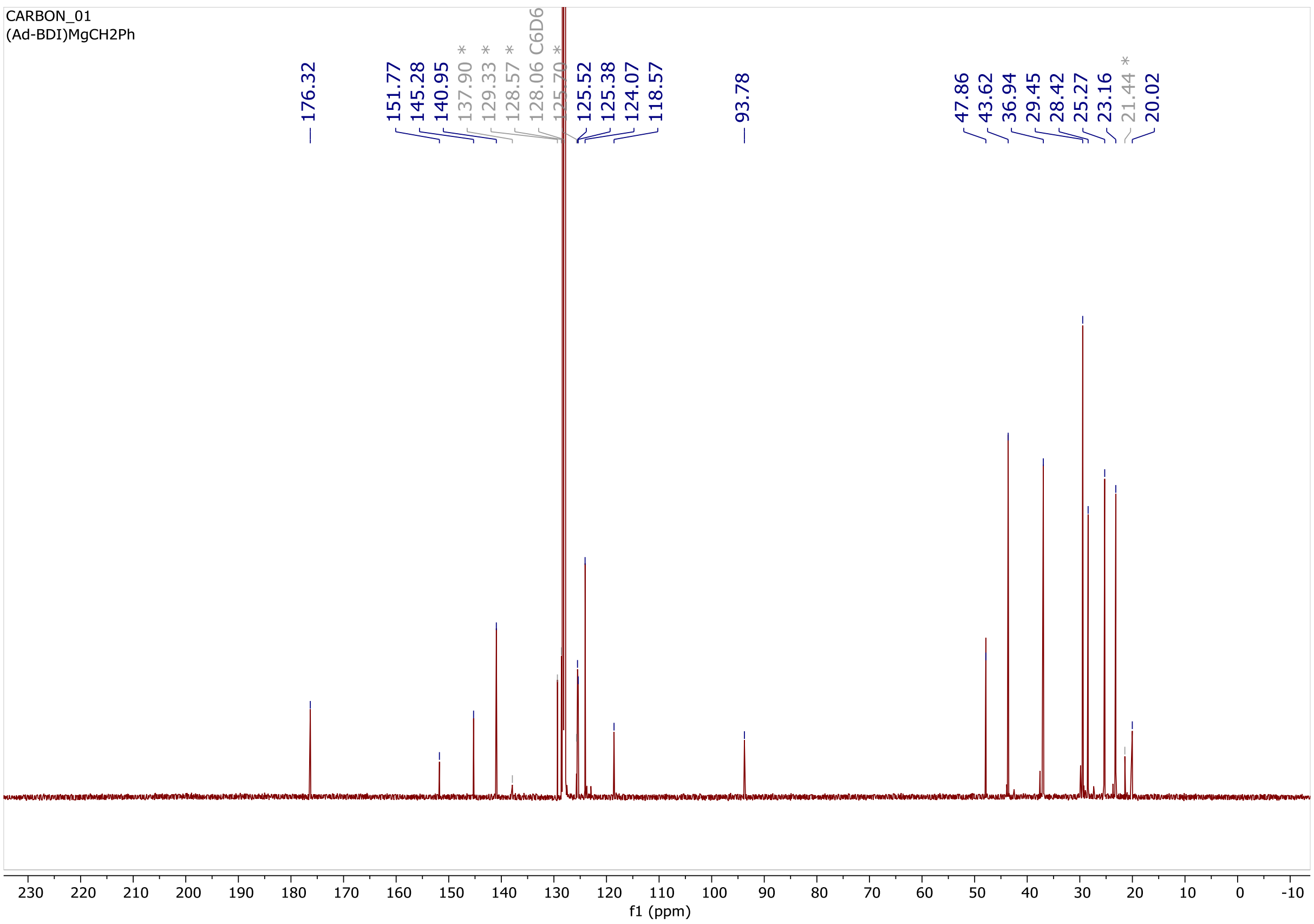

Figure S89: $^{13}C\{^{1}H\}$ NMR (126 MHz, C_6D_6).

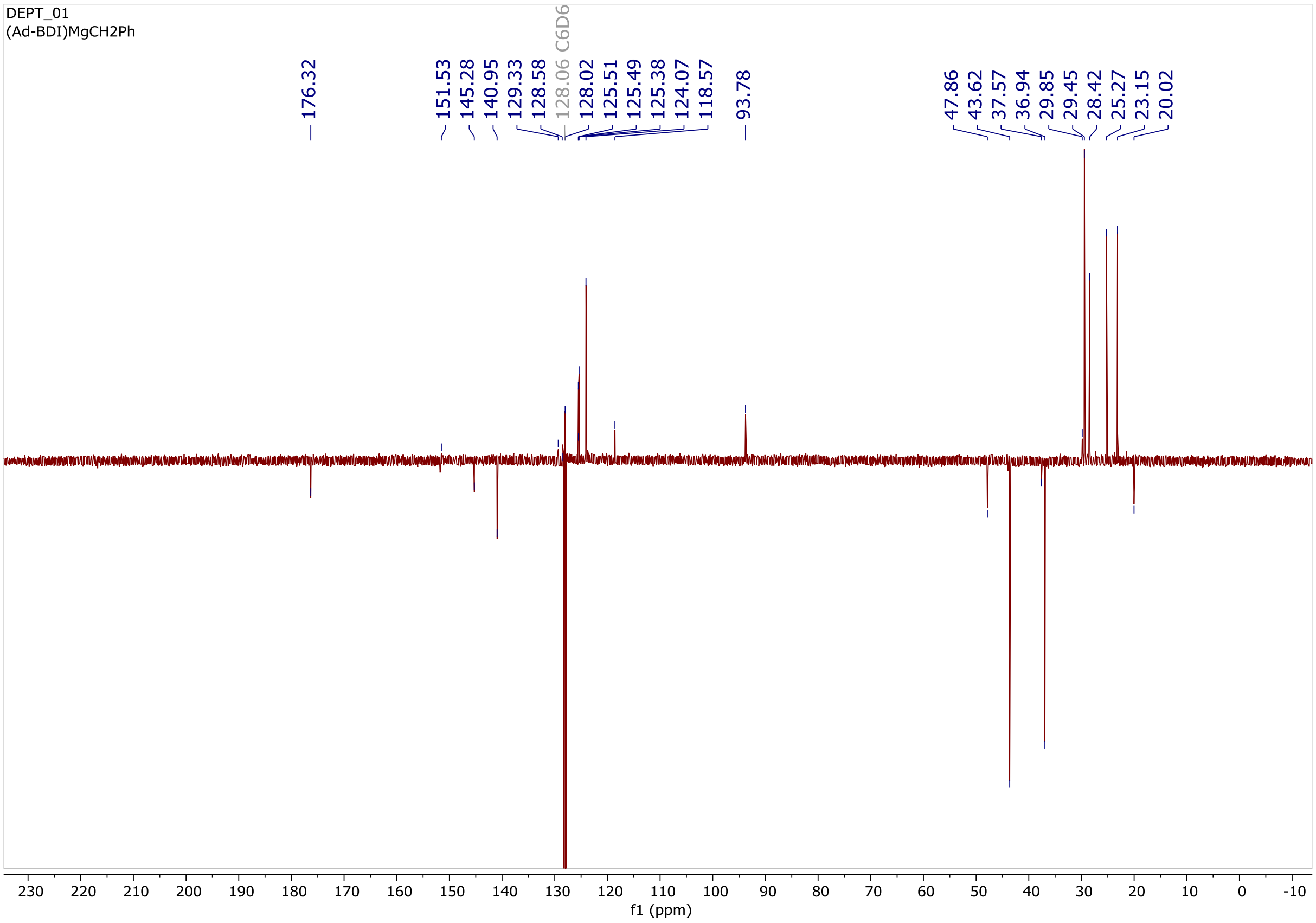

Figure S90: $^{13}C\{^{1}H\}$ DEPT NMR (126 MHz, C_6D_6).

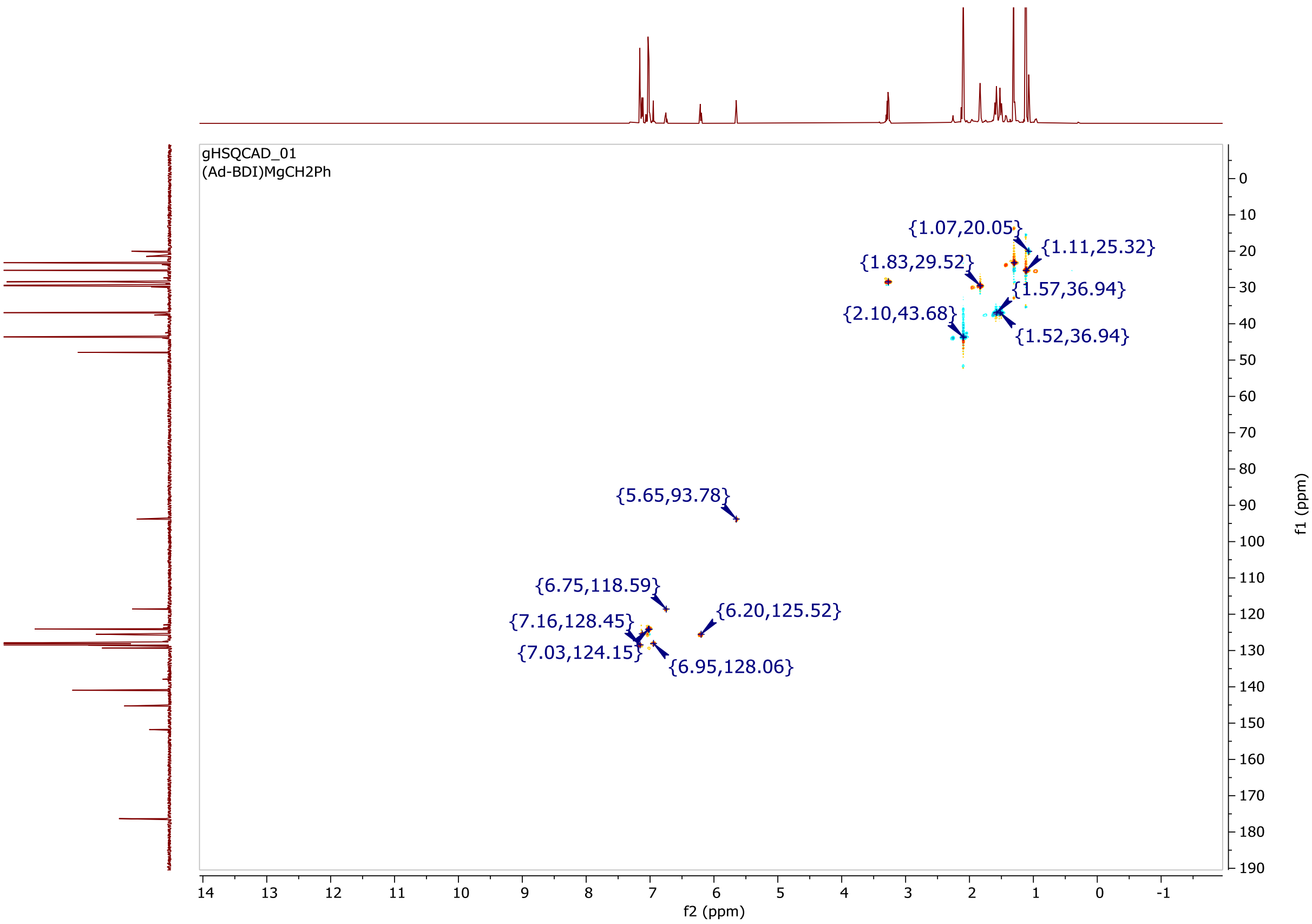

Figure S91: ^{13}C-^{1}H HSQC NMR (126 MHz, C_6D_6).

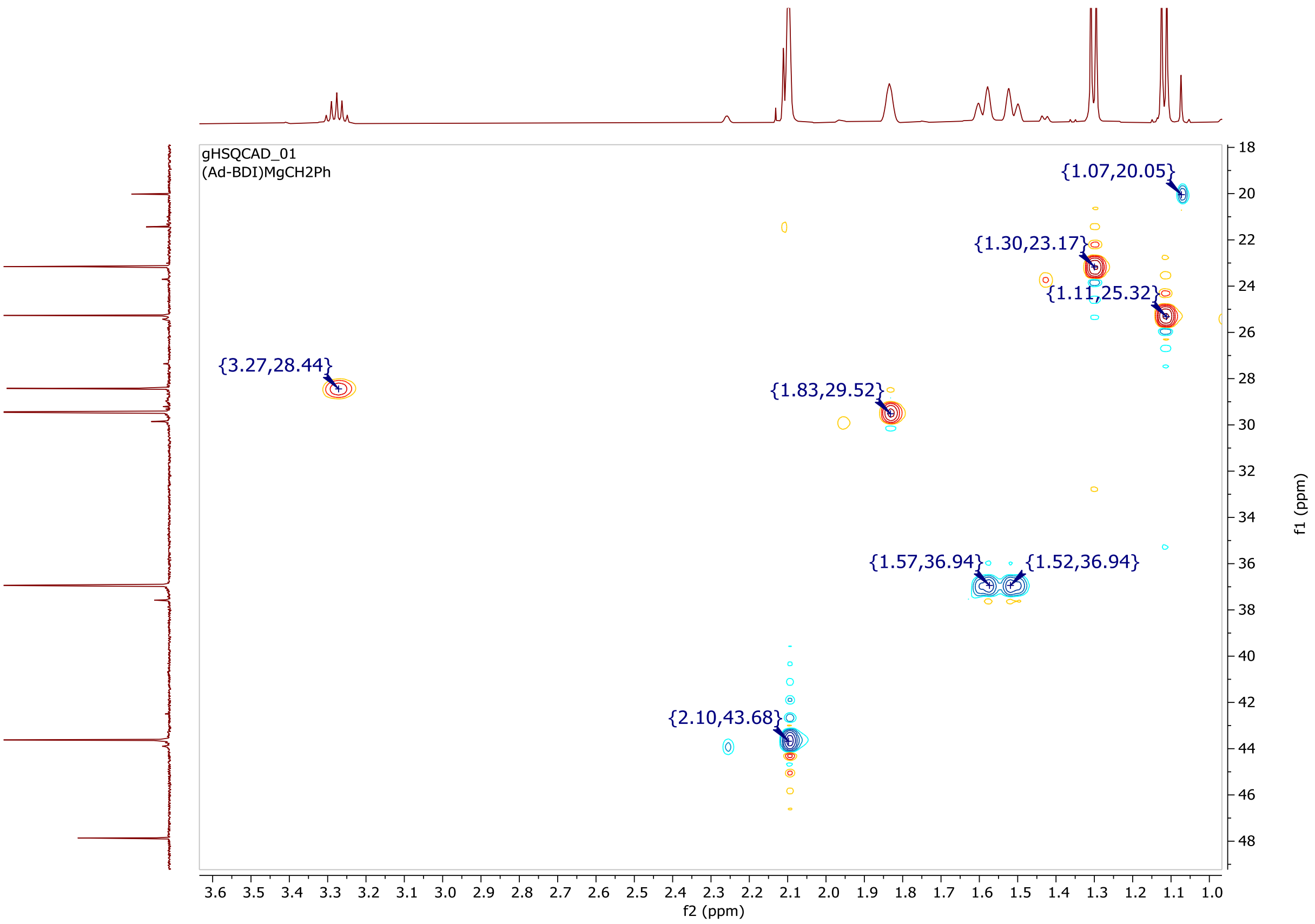

Figure S92: Expanded ^{13}C-^{1}H HSQC NMR (126 MHz, C_6D_6).

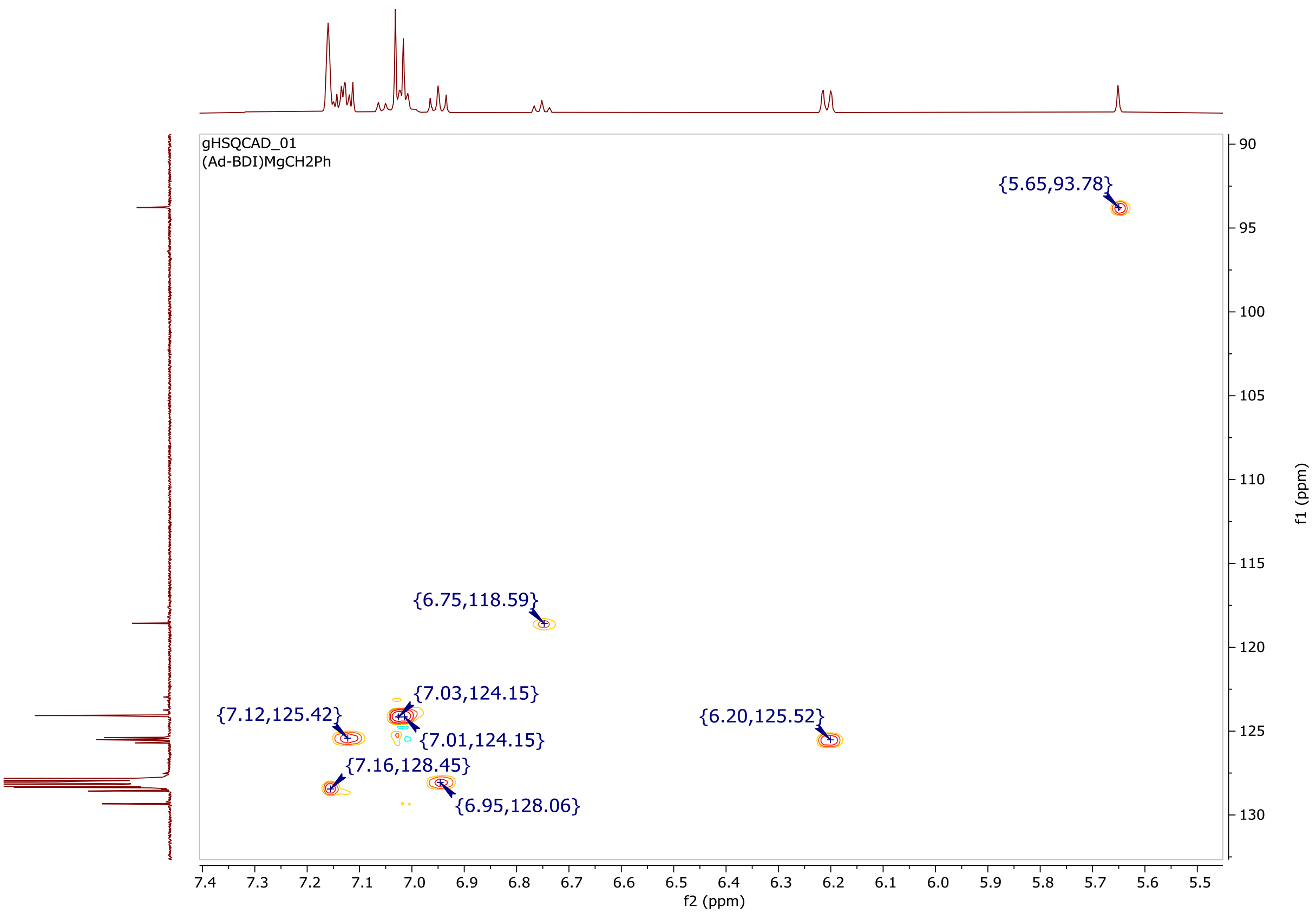

Figure S93: Expanded ^{13}C-^{1}H HSQC NMR (126 MHz, C_6D_6).

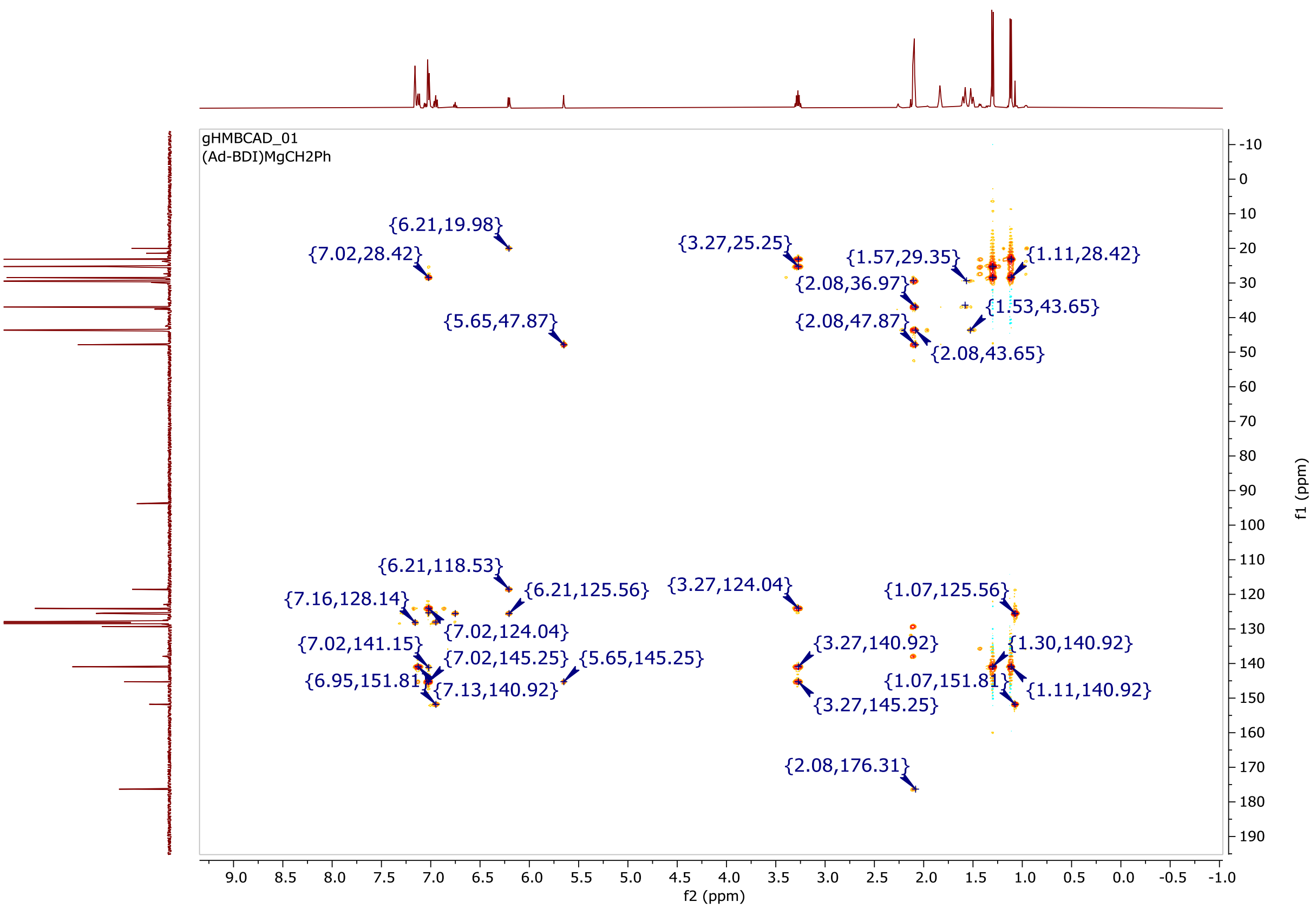

Figure S94: ^{13}C-^{1}H HMBC NMR (126 MHz, C_6D_6).

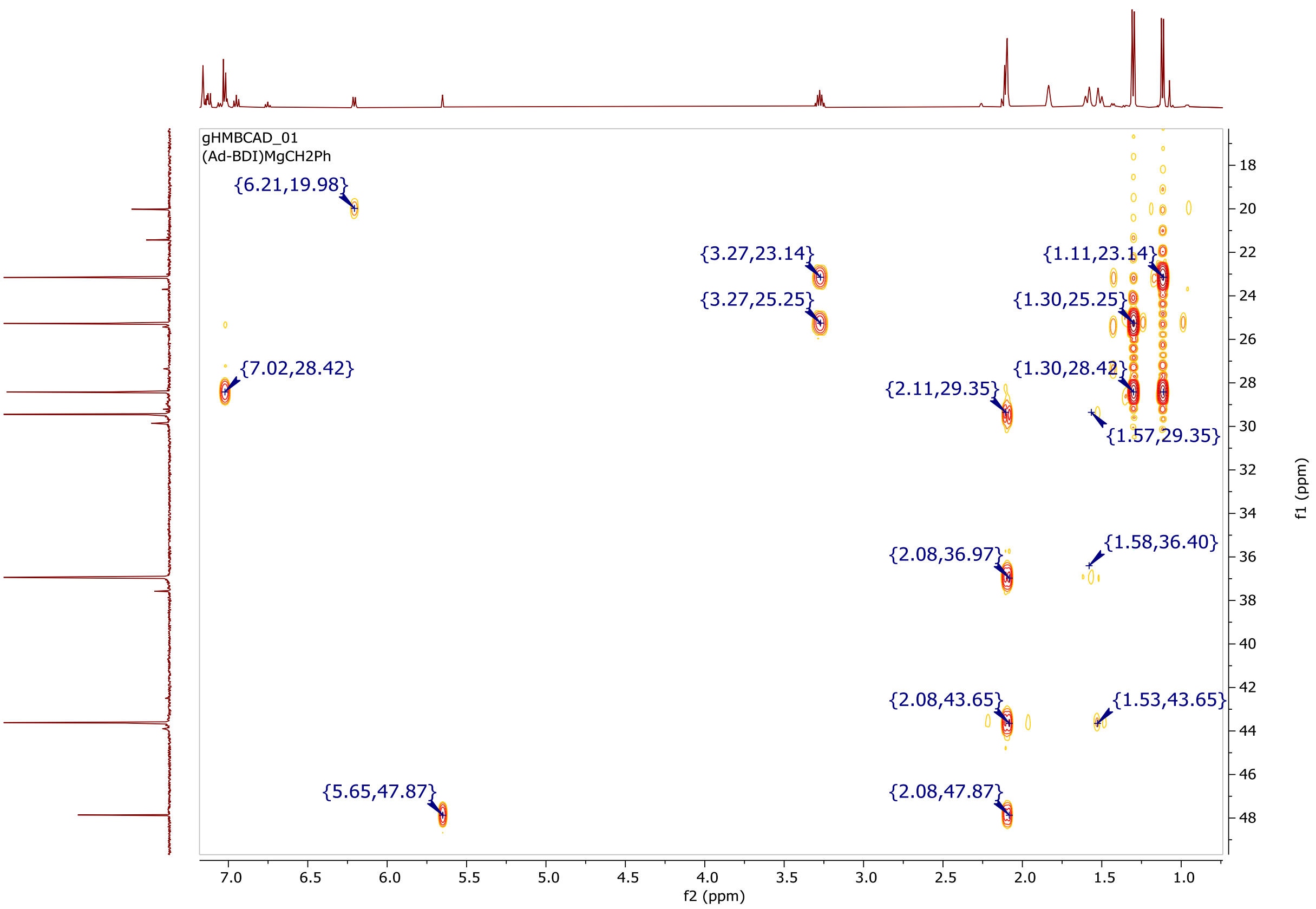

Figure S95: Expanded ^{13}C-^{1}H HMBC NMR (126 MHz, C_6D_6).

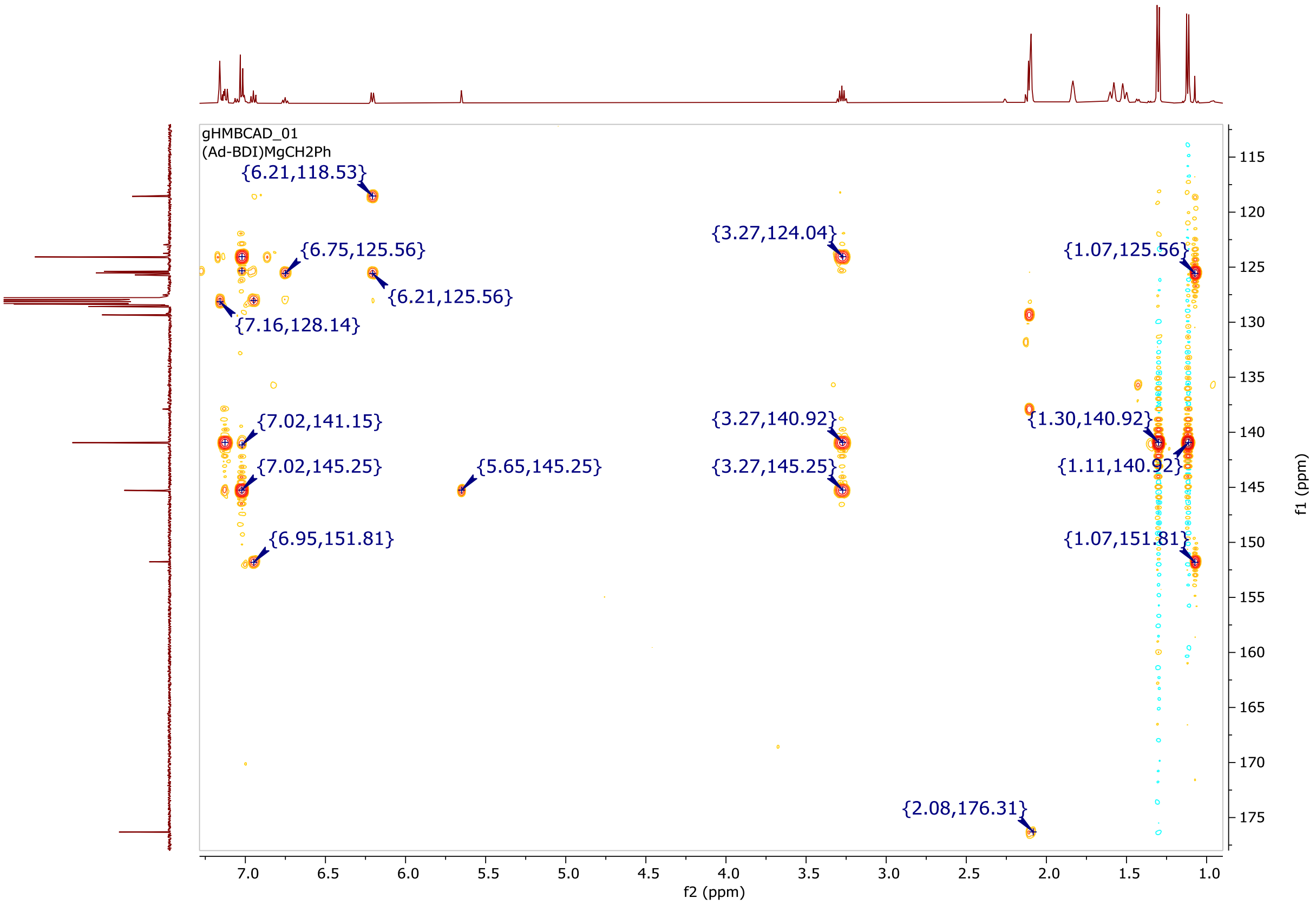

Figure S96: Expanded ^{13}C-^{1}H HMBC NMR (126 MHz, C_6D_6).

2.13 Reaction of [(^{Ad}BDI)Mg^{n}Bu] (1^{Ad}) with *ortho*-xylene, synthesis and characterisation of [(^{Ad}BDI)Mg(C_6H_3-3,4-Me_2)] (6) and [(^{Ad}BDI)Mg($CH_2C_6H_4$-2-Me)] (6')

A J. Young's NMR tube was charged with 13 mg **1^{Ad}** (0.018 mmol) and 0.6 ml *ortho*-xylene. The pale-yellow solution was heated at 100°C for 11 days. Analysis of the reaction solution by ^{1}H NMR indicated partial conversion of the starting complex and the apparent formation of several BDI-containing products. Volatiles were removed under vacuum and *n*-hexane (0.5 ml) was added. The NMR tube was sealed and heated at 80°C for 20 hours, resulting in the formation of colourless block-like crystals suitable for X-ray diffraction analysis on the walls of the NMR tube above the meniscus. The crystal structure was solved and refined as an 80:20 co-crystalline mixture of the respective isomers **6** and **6'**. The crystals were isolated in 2 mg yield, 14%.

The procedure was repeated at a higher temperature of 120°C. After 10 days, volatiles were removed under vacuum and the crude product mixture was redissolved in benzene-*d*$_6$. Analysis by ^{1}H NMR spectroscopy showed quantitative consumption of **1^{Ad}** and the formation of **6** and **6'** as the only products in respective spectroscopic yields of 62% and 38%. The products were isolated as a pale-yellow powder (14 mg, 94%) after freeze-drying the benzene-*d*$_6$ solution under vacuum. The two isomers were not separated.

Analysis calculated for $C_{55}H_{73}MgN_2$: C, 83.88; H, 9.47; N, 3.56%. Found (mixture of isomers): C, 83.69; H, 9.96; N, 4.06%.

*NMR data for **6***

^{1}H NMR (500 MHz, C_6D_6) δ 7.24 (t, *J* = 7.7 Hz, 2H, *p*-NC_6H_3-*i*Pr_2), 7.12 (d, *J* = 7.8 Hz, 4H, *m*-NC_6H_3-*i*Pr_2), 6.84 (d, *J* = 7.0 Hz, 1H, 1-Mg-*5*-C_6H_3-3,4-CH_3), 5.99 (d, *J* = 7.0 Hz, 1H, 1-Mg-*6*-C_6H_3-3,4-CH_3), 5.80 (s, 1H, 1-Mg-*2*-C_6H_3-3,4-CH_3), 5.73 (s, 1H, *H*CC(Ndipp)), 3.42 (hept, *J* = 7.0 Hz, 4H, *H*C(CH_3)$_2$), 2.16 (s, 12H, ^{Ad}CC*H*$_2$CH), 2.07 (s, 3H, 1-Mg-C_6H_3-*3*,4-C*H*$_3$), 2.01 (s, 3H, 1-Mg-C_6H_3-3,*4*-

C*H*$_3$), 1.87 (br, 7H, ^{Ad}C*H*),* 1.64 – 1.58 (m, 8H, ^{Ad}CHC*H*$_2$CH),* 1.56 – 1.50 (m, 9H, ^{Ad}CHC*H*$_2$CH),* 1.33 (d, *J* = 6.8 Hz, 8H, HC(C*H*$_3$)$_2$), 1.24 (d, *J* = 7.0 Hz, 12H, HC(C*H*$_3$)$_2$).* Integral not accurately determined due to overlap with other isomer.

^{13}C NMR (126 MHz, C_6D_6) δ 176.47 (N=*C*), 155.16 (1-Mg-*1*-*C*$_6$H$_3$-3,4-CH$_3$), 145.36 (*ipso*-NC$_6$H$_3$-*i*Pr$_2$), 142.63 (1-Mg-*2*-*C*$_6$H$_3$-3,4-CH$_3$), 141.61 (*o*-N*C*$_6$H$_3$-*i*Pr$_2$), 138.73 (1-Mg-*6*-*C*$_6$H$_3$-3,4-CH$_3$), 133.19 (1-Mg-*4*-*C*$_6$H$_3$-3,4-CH$_3$), 133.07 (1-Mg-*3*-*C*$_6$H$_3$-3,4-CH$_3$), 128.57 (1-Mg-*5*-*C*$_6$H$_3$-3,4-CH$_3$), 125.52 (*p*-N*C*$_6$H$_3$-*i*Pr$_2$), 124.16 (*m*-N*C*$_6$H$_3$-*i*Pr$_2$), 93.67 (H*C*C(Ndipp)), 47.90 (NC^{Ad}*C*), 43.73 (^{Ad}*C*CH$_2$CH), 37.00 (^{Ad}CH<u>C</u>H$_2$CH), 29.52 (^{Ad}*C*H), 28.53 (H*C*(CH$_3$)$_2$), 25.86 (HC(*C*H$_3$)$_2$), 23.17 (HC(*C*H$_3$)$_2$), 19.82 (1-Mg-C$_6$H$_3$-3,*4*-*C*H$_3$), 19.66 (1-Mg-C$_6$H$_3$-<u>3</u>,4-*C*H$_3$).

*NMR data for **6'***

^{1}H NMR (400 MHz, C_6D_6) δ 7.12 (m, NC$_6$*H*$_3$-*i*Pr$_2$),* 7.04 (m, NC$_6$*H*$_3$-*i*Pr$_2$),* 6.98 – 6.91 (m, 2H, MgCH$_2$ -*3,4*-C$_6$*H*$_4$-2-Me), 6.79 (t, *J* = 7.2 Hz, 1H, MgCH$_2$ -<u>5</u>-C$_6$*H*$_4$-2-Me), 6.19 (d, *J* = 7.5 Hz, 1H, MgCH$_2$ -<u>6</u>-C$_6$*H*$_4$-2-Me), 5.67 (s, 1H, *H*CC(Ndipp)), 3.28 (h, *J* = 6.9 Hz, 4H, *H*C(CH$_3$)$_2$), 2.09 (d, *J* = 2.9 Hz, 12H, ^{Ad}CC*H*$_2$CH), 1.84 (br, ^{Ad}C*H*),* 1.57 (m, ^{Ad}CHC*H*$_2$CH), 1.43 (s, 3H, MgCH$_2$-C$_6$H$_4$-C*H*$_3$), 1.30 (d, *J* = 6.9 Hz, 12H, HC(C*H*$_3$)$_2$), 1.09 (d, *J* = 6.8 Hz, 12H, HC(C*H*$_3$)$_2$), 0.96 (s, 2H, MgC*H*$_2$-C$_6$H$_4$-CH$_3$).*Integral not accurately determined due to overlap with other isomer.

^{13}C NMR (126 MHz, C_6D_6) δ 176.40 (N=<u>C</u>), 149.59 (MgCH$_2$ -<u>1</u>-<u>C</u>$_6$H$_4$-2-Me), 145.56 (*ipso*-NC$_6$H$_3$-*i*Pr$_2$), 141.15 (*o*-N*C*$_6$H$_3$-*i*Pr$_2$), 130.82 (MgCH$_2$ -<u>2</u>-<u>C</u>$_6$H$_4$-2-Me), 129.22 (MgCH$_2$ -<u>4</u>-C$_6$*H*$_4$-2-Me), 126.59 (MgCH$_2$ -<u>6</u>-<u>C</u>$_6$H$_4$-2-Me), 125.70 (MgCH$_2$ -<u>3</u>-C$_6$*H*$_4$-2-Me), 125.55 (*m*-N*C*$_6$H$_3$-*i*Pr$_2$), 124.13 (*m*-NC$_6$H$_3$-*i*Pr$_2$), 119.14 (MgCH$_2$ -<u>5</u>-<u>C</u>$_6$H$_4$-2-Me), 93.98 (H<u>C</u>C(Ndipp)), 47.96 (NC^{Ad}<u>C</u>), 43.63 (^{Ad}C<u>C</u>H$_2$CH), 36.96 (^{Ad}CH<u>C</u>H$_2$CH), 29.50 (^{Ad}<u>C</u>H), 28.48 (H<u>C</u>(CH$_3$)$_2$), 25.25 (HC(<u>C</u>H$_3$)$_2$), 23.15 (HC(<u>C</u>H$_3$)$_2$), 20.54 (MgCH$_2$-C$_6$H$_4$-<u>C</u>H$_3$), 18.37 (MgC<u>H</u>$_2$-C$_6$H$_4$-CH$_3$).

NMR spectra from 100°C reaction

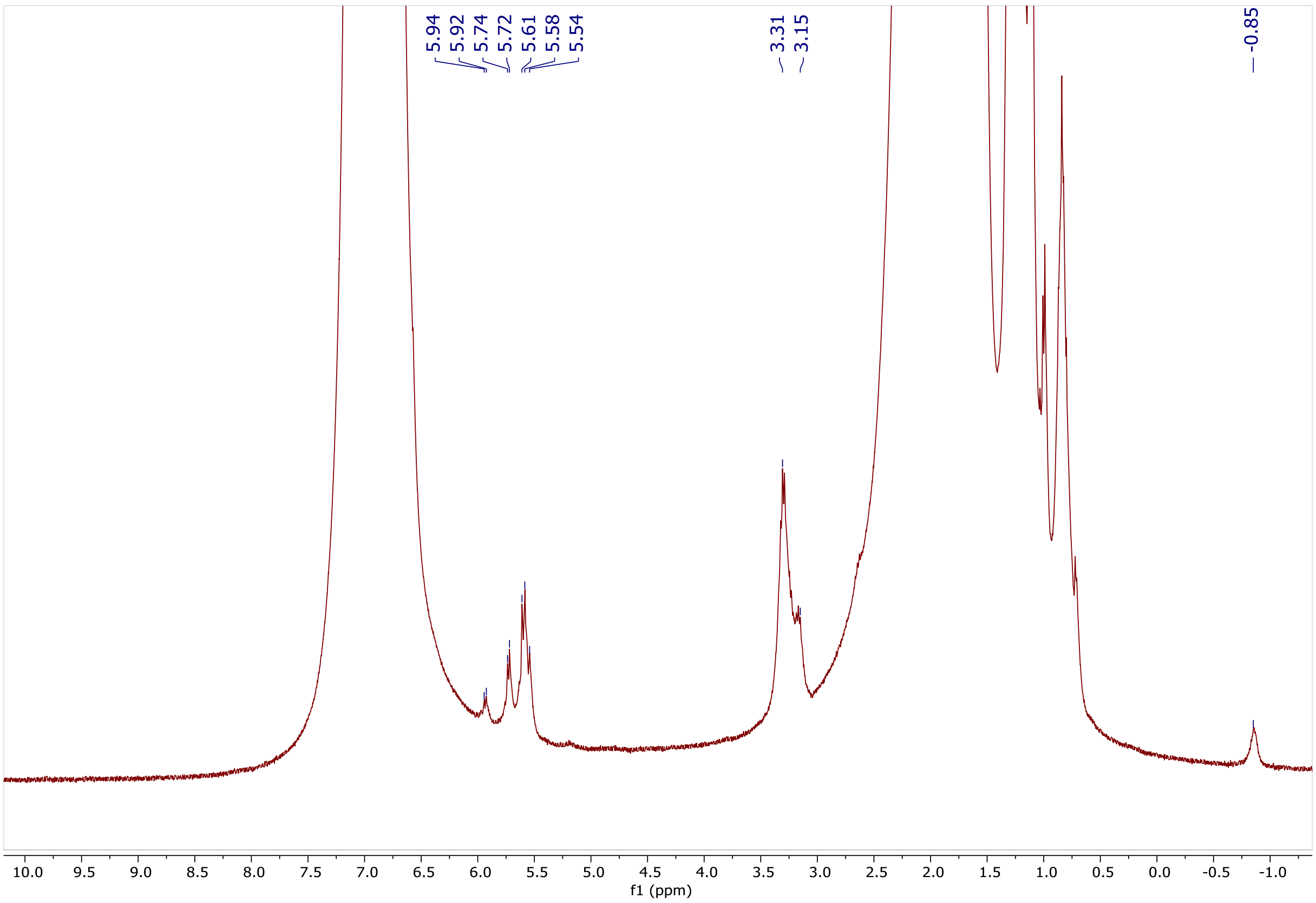

Figure S97: In situ ^{1}H NMR spectrum (400 MHz, ortho-xylene, unlocked) after 11 days at 100°C.

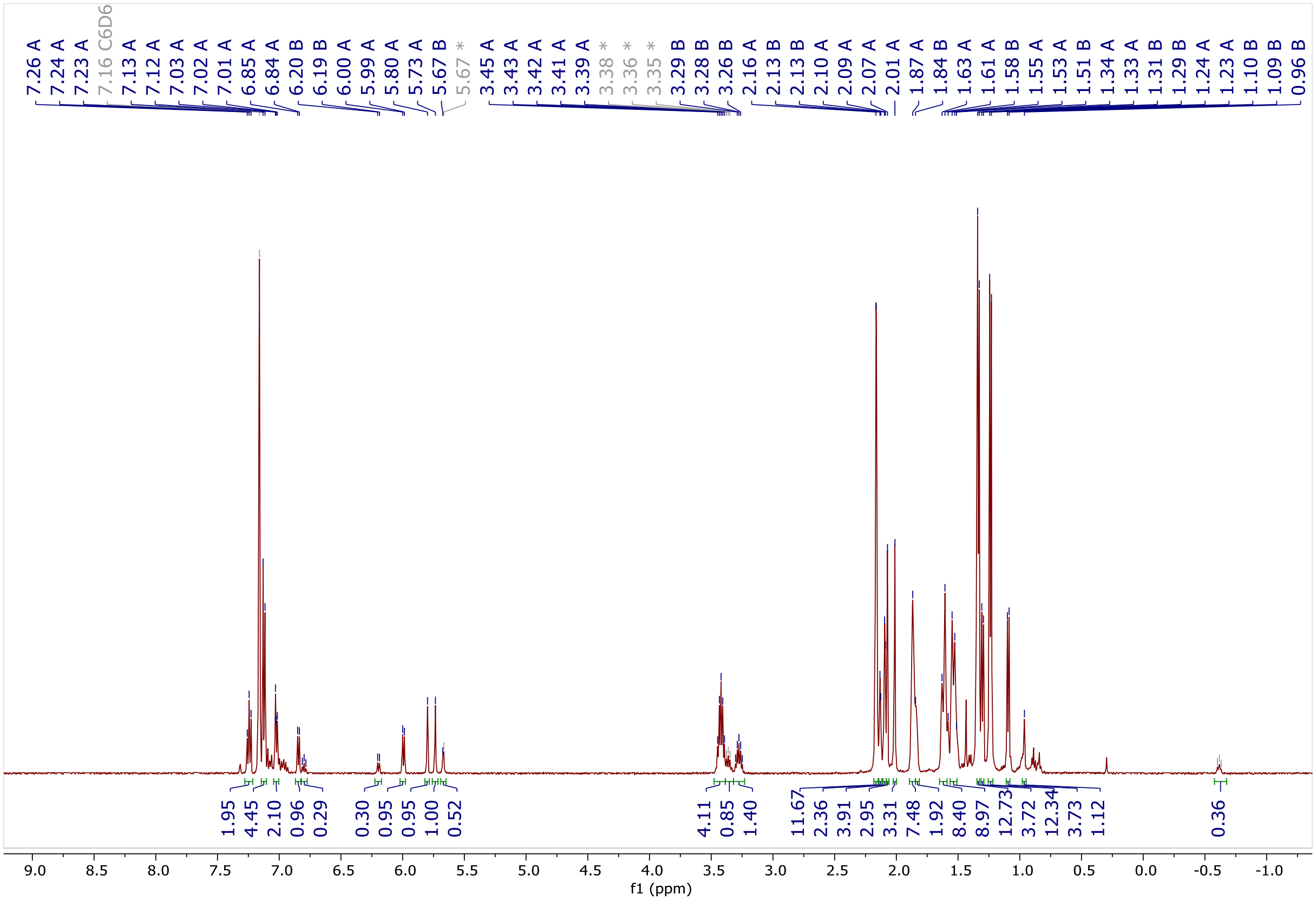

Figure S98: ^{1}H NMR (500 MHz, C_6D_6) of isolated crystals. A, **6**; B, **6'**. *Residual $\mathbf{1^{Ad}}$.

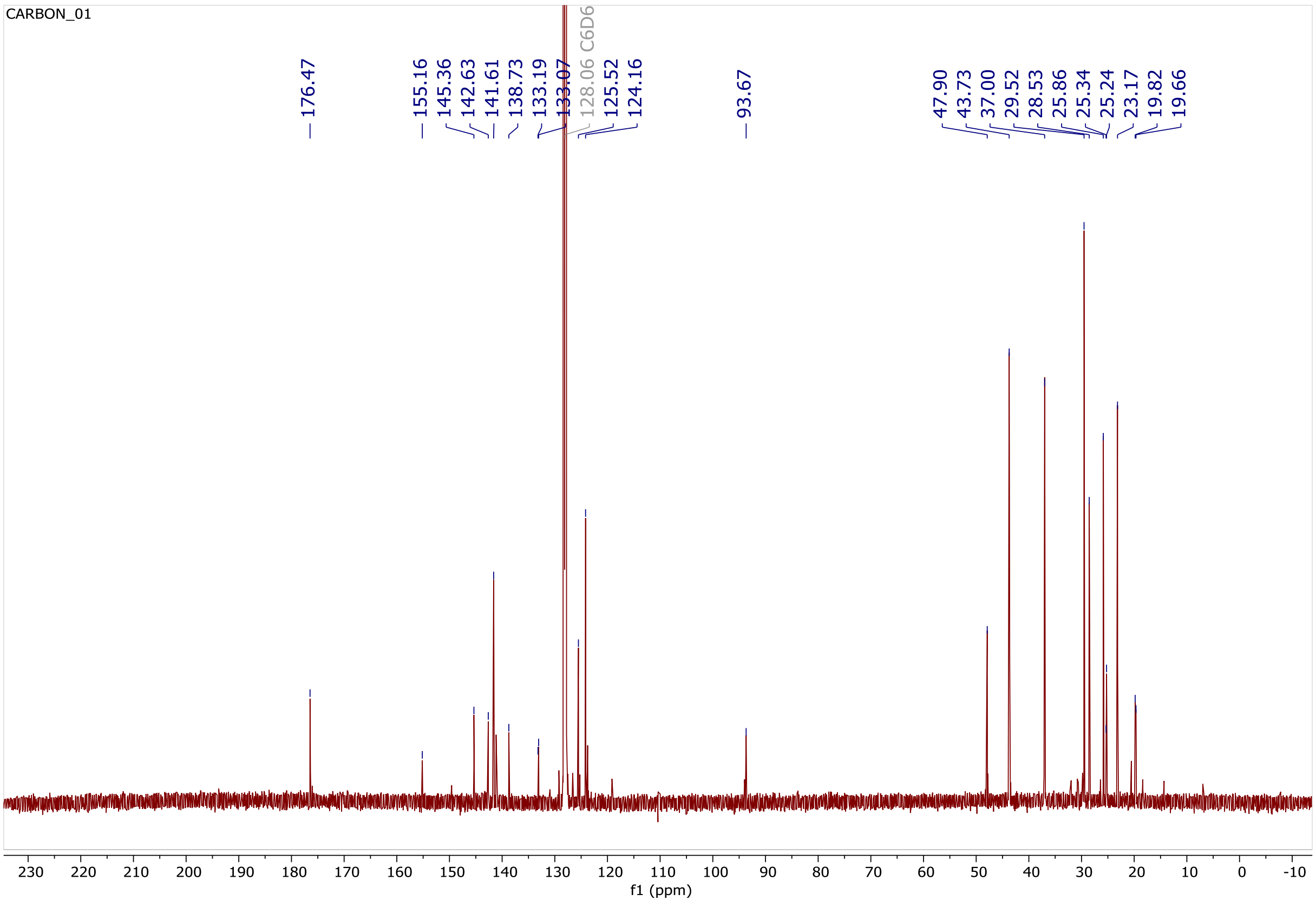

Figure S99: $^{13}C\{^{1}H\}$ NMR (126 MHz, C_6D_6) of isolated crystals.

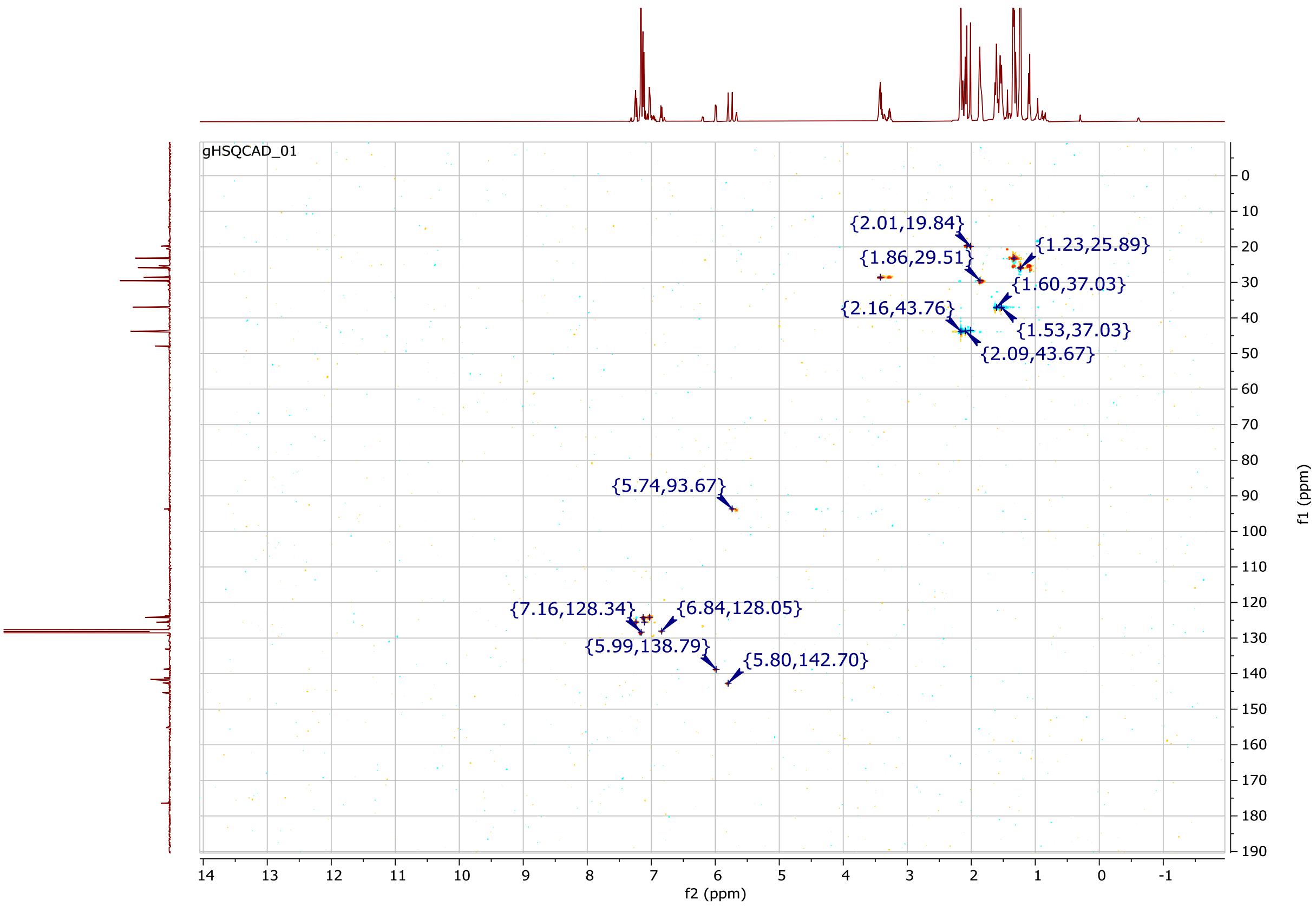

Figure S100: ^{13}C-^{1}H HSQC NMR (126 MHz, C_6D_6) of isolated crystals.

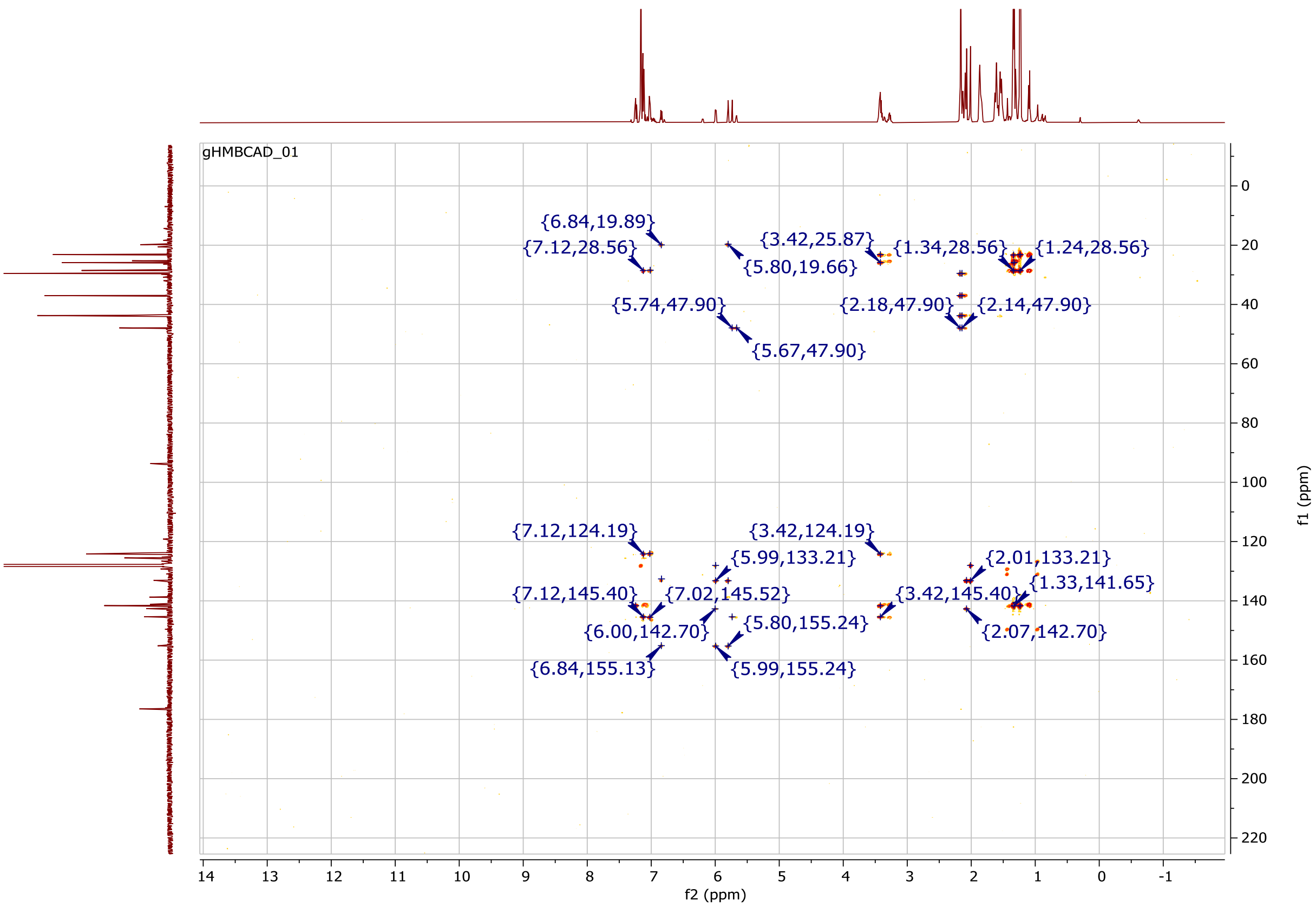

Figure S101 ^{13}C-^{1}H HMBC NMR (126 MHz, C_6D_6) of isolated crystals.

<u>NMR spectra from 120°C reaction</u>

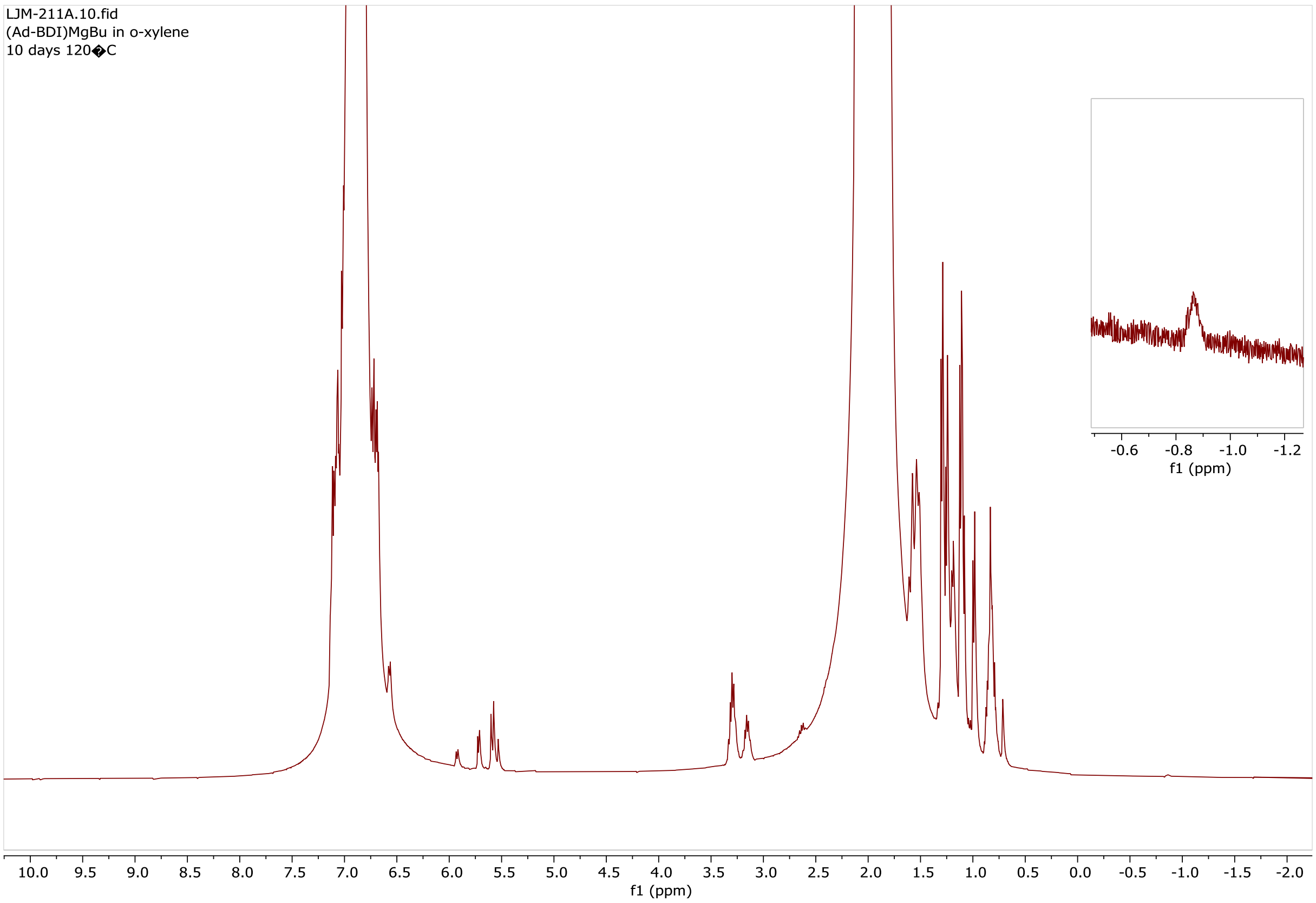

Figure S102: ¹H NMR (400 MHz, ortho-xylene, unlocked) after 10 days at 120°C, highlighting near-quantitative consumption of $\mathbf{1}^{\mathbf{Ad}}$.

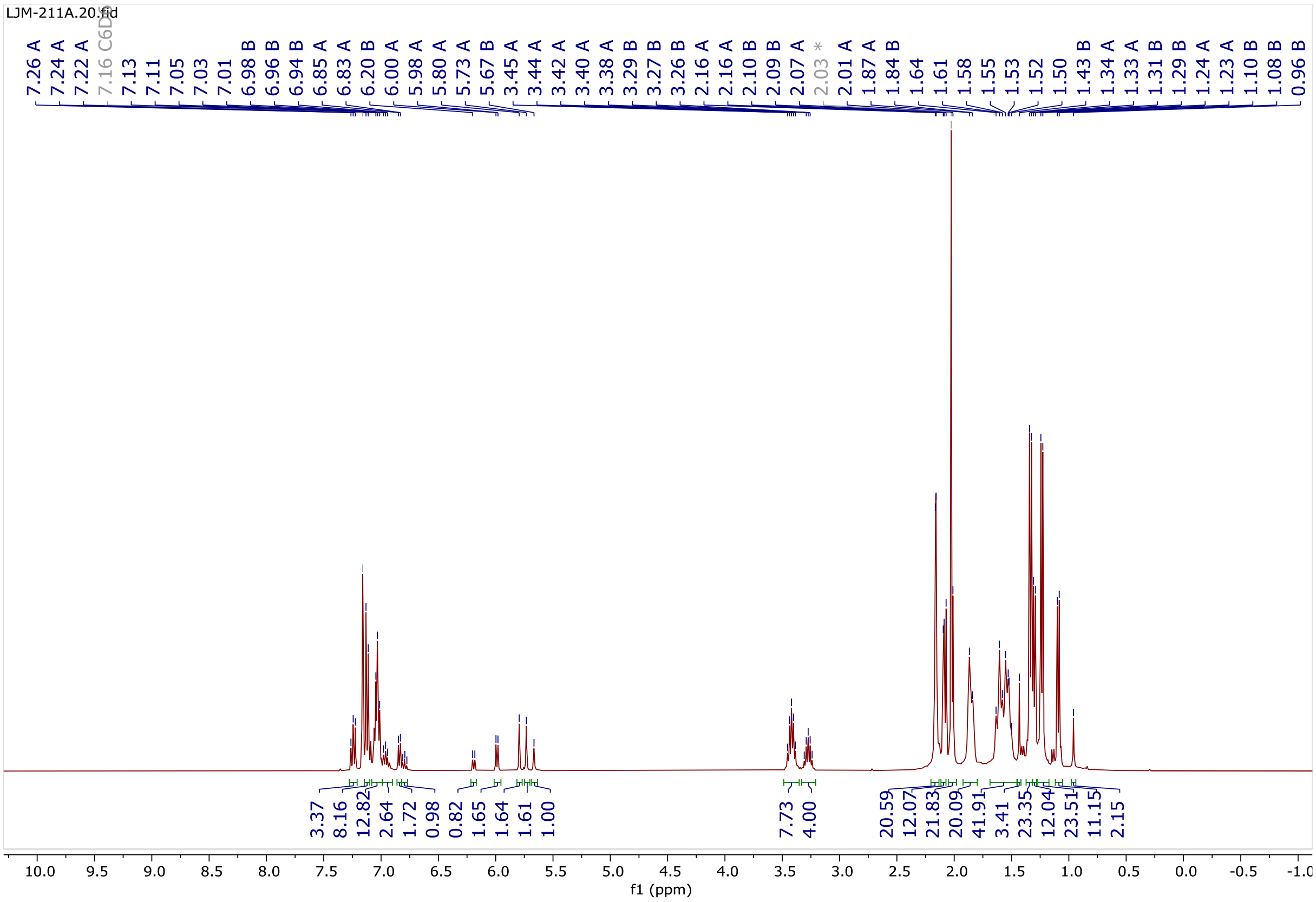

Figure S103: ¹H NMR spectrum (400 MHz, C_6D_6) after removal of volatiles. Resonances are annotated to denote **6** (A) and **6'** (B). Un-annotated resonances are overlapping isomers. *Residual ortho-xylene.

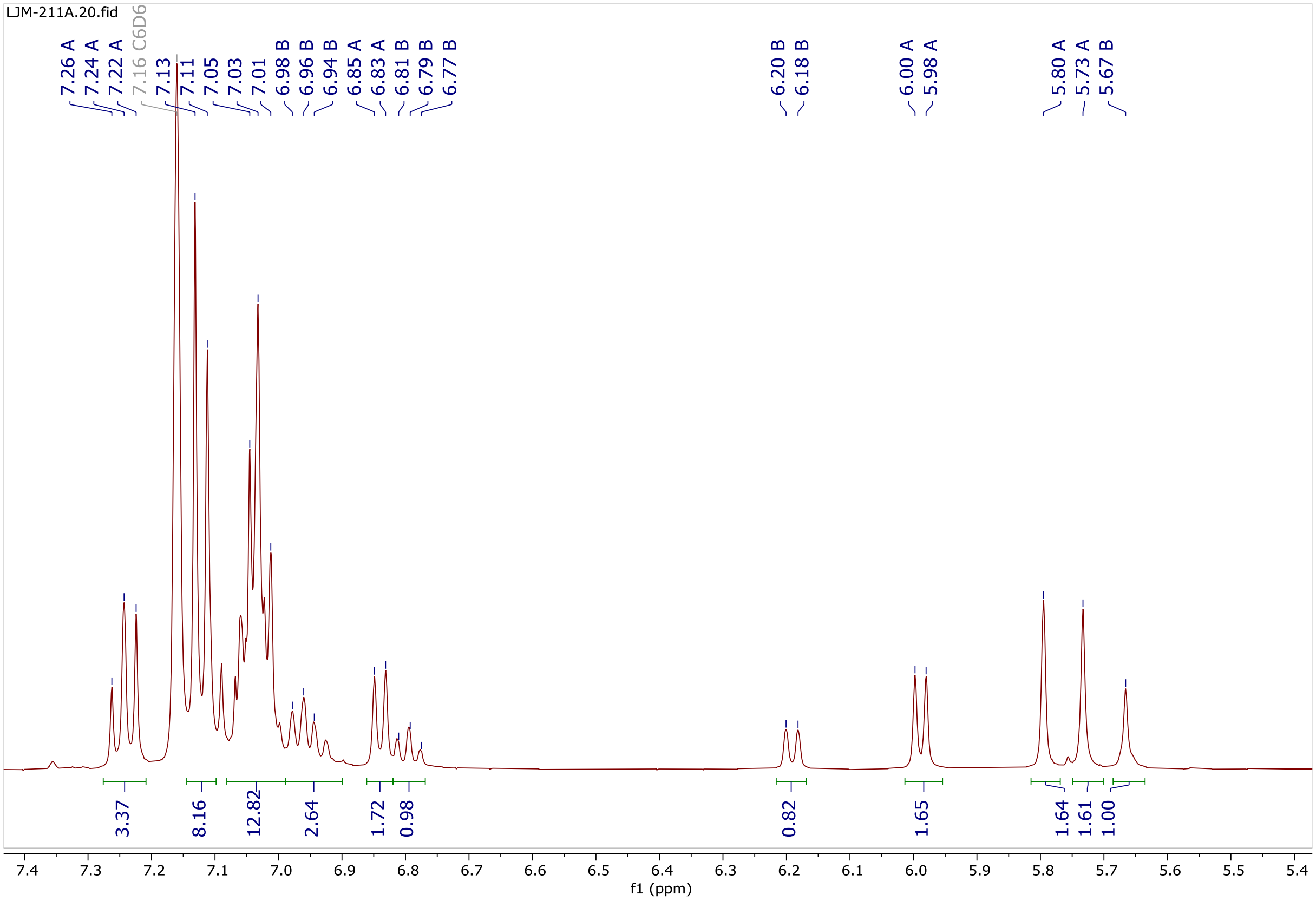

Figure S104:Expanded ^{1}H NMR spectrum (400 MHz, C_6D_6) after removal of volatiles. Resonances are annotated to denote **6** (A) and **6'** (B). Un-annotated resonances are overlapping isomers. *Multiplet at 7.08 – 6.99 is also overlapped with residual ortho-xylene.

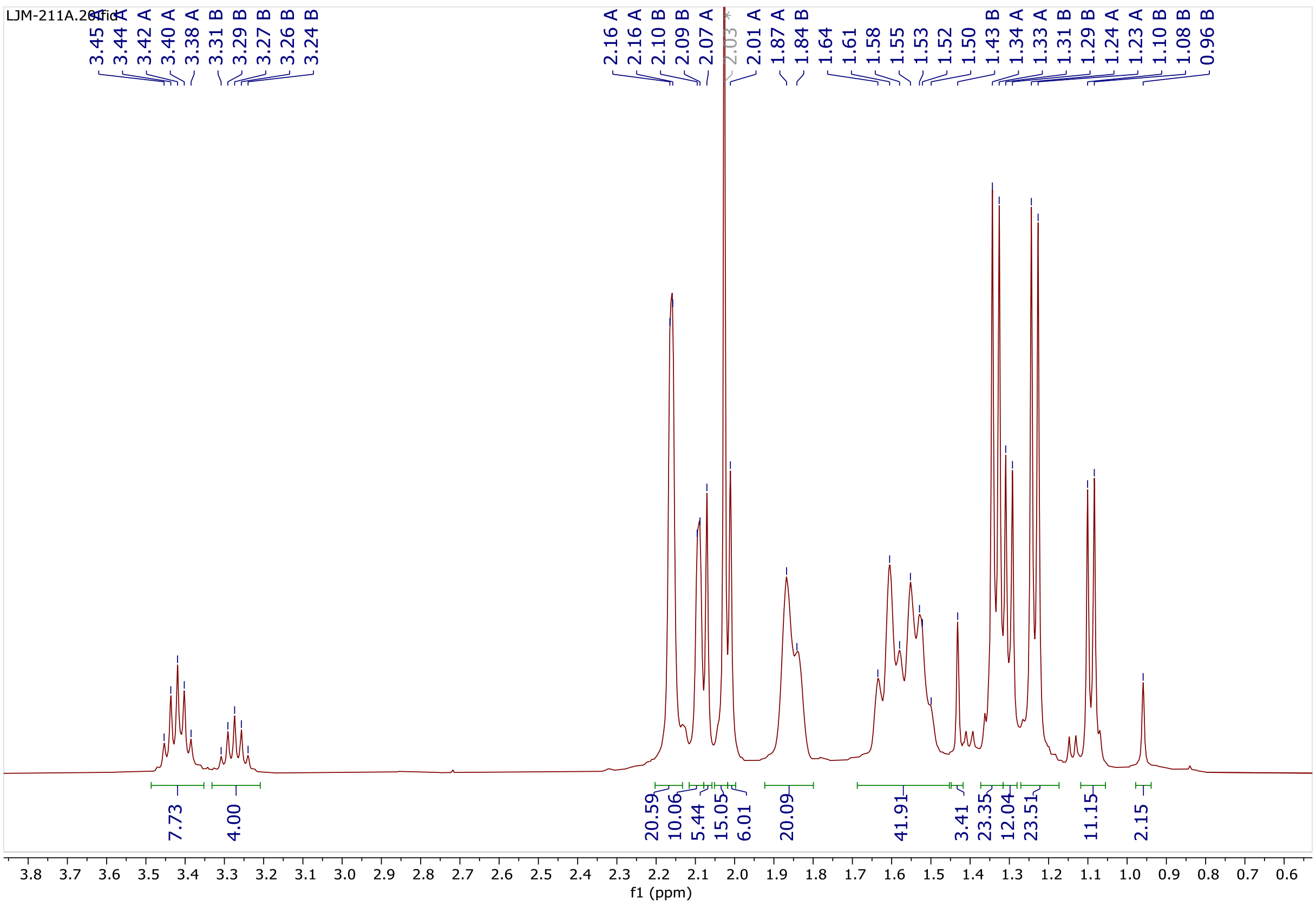

Figure S105: Expanded ^{1}H NMR spectrum (400 MHz, C_6D_6) after removal of volatiles. Resonances are annotated to denote **6** (A) and **6'** (B). Un-annotated resonances are overlapping isomers. *Residual ortho-xylene.

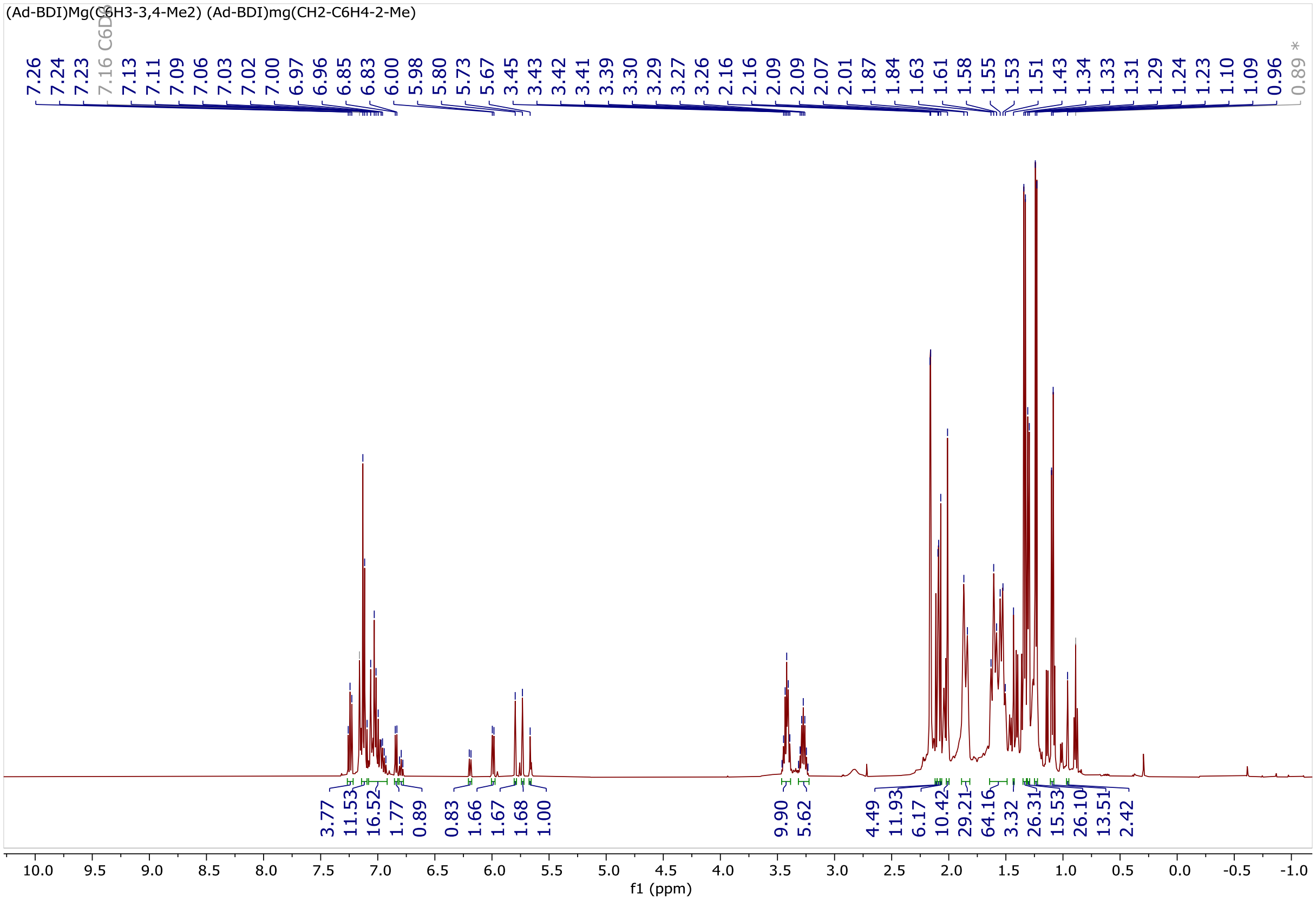

Figure S106: ^{1}H NMR spectrum (500 MHz, C_6D_6) of freeze-dried product. *hexane.

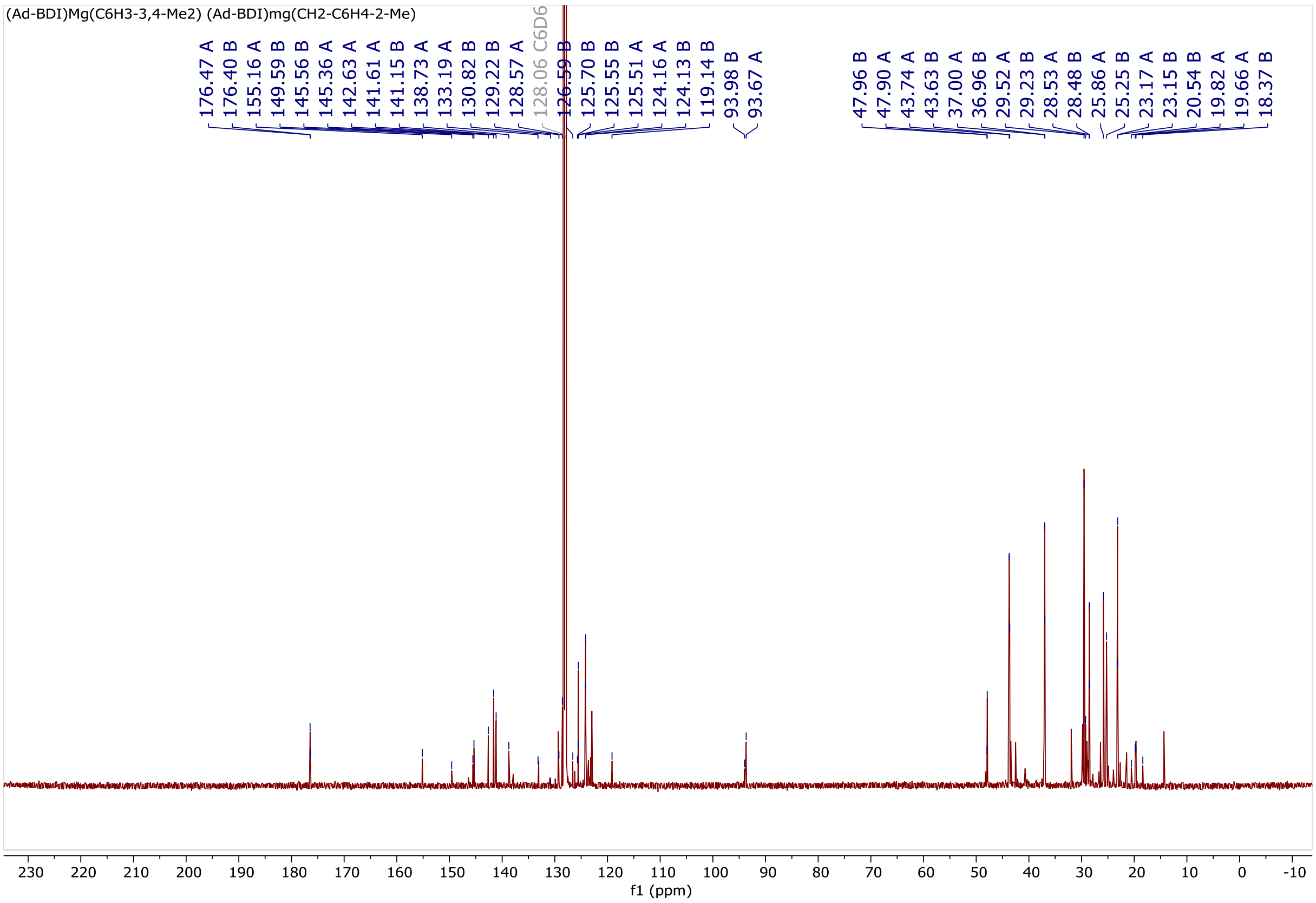

Figure S107: $^{13}C\{^{1}H\}$ NMR (500 MHz, C_6D_6). Resonances are annotated to denote **6** (A) and **6'** (B).

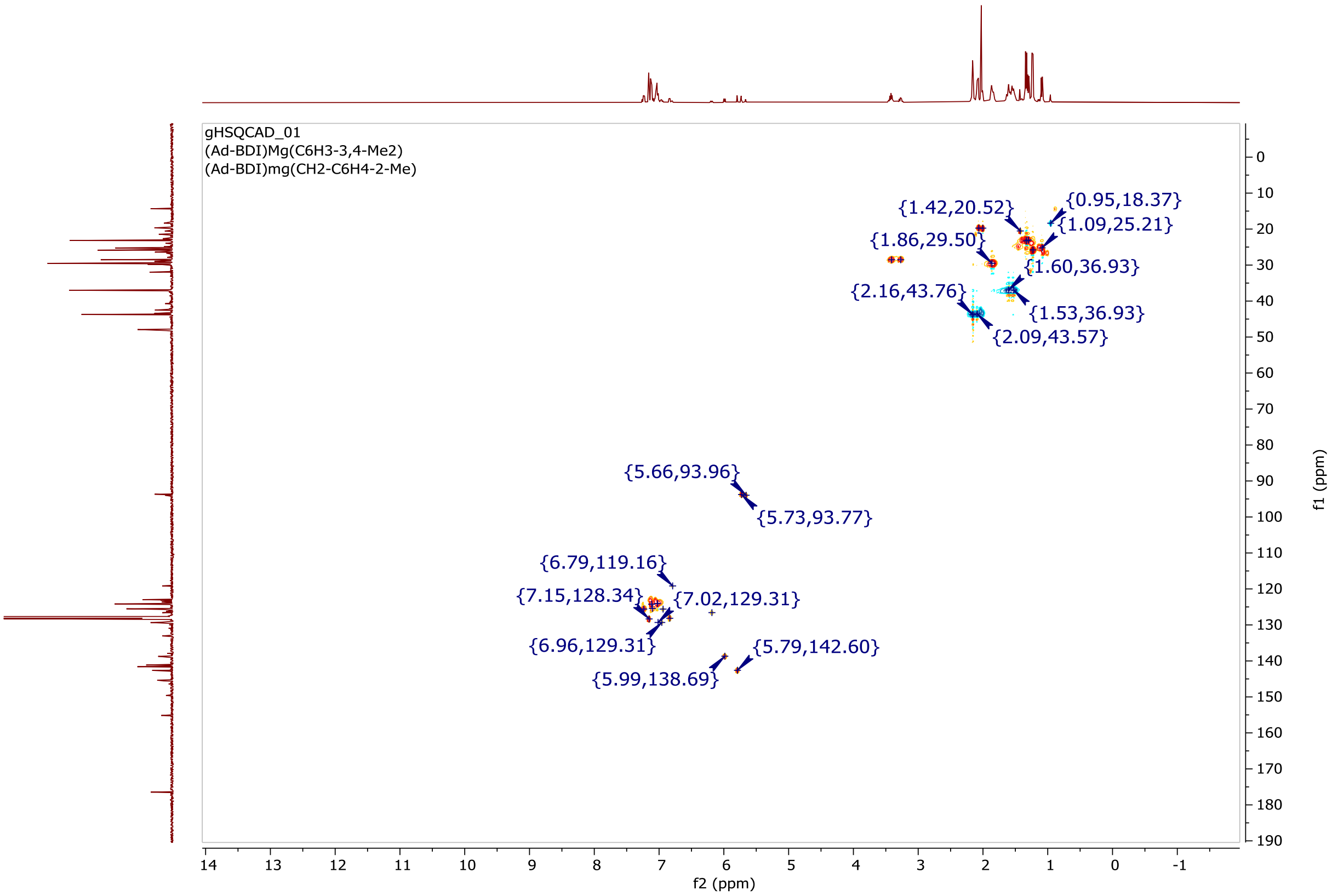

Figure S108: ^{13}C-^{1}H HSQC NMR (500 MHz, C_6D_6).

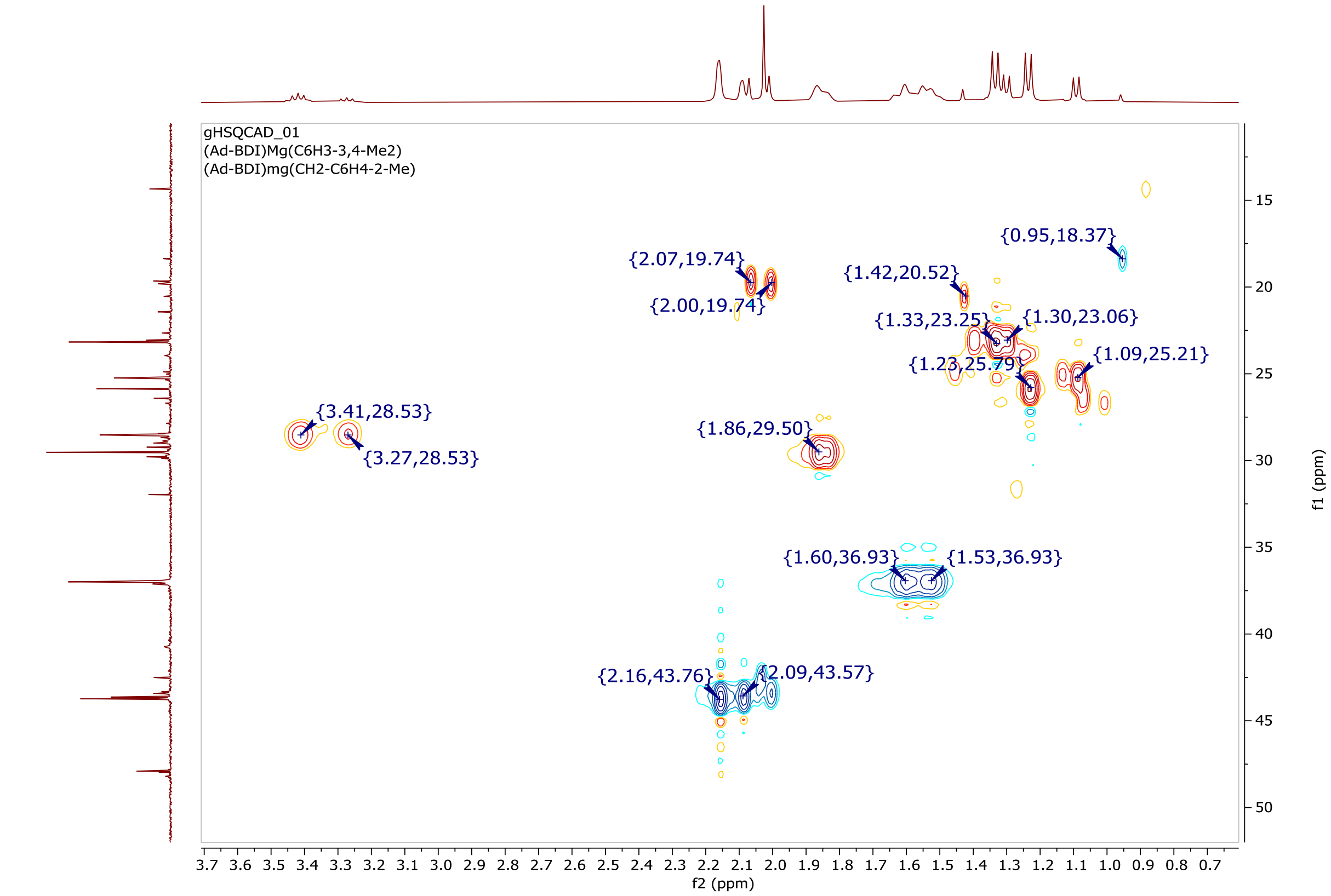

Figure S109: Expanded ^{13}C-^{1}H HSQC NMR (500 MHz, C_6D_6).

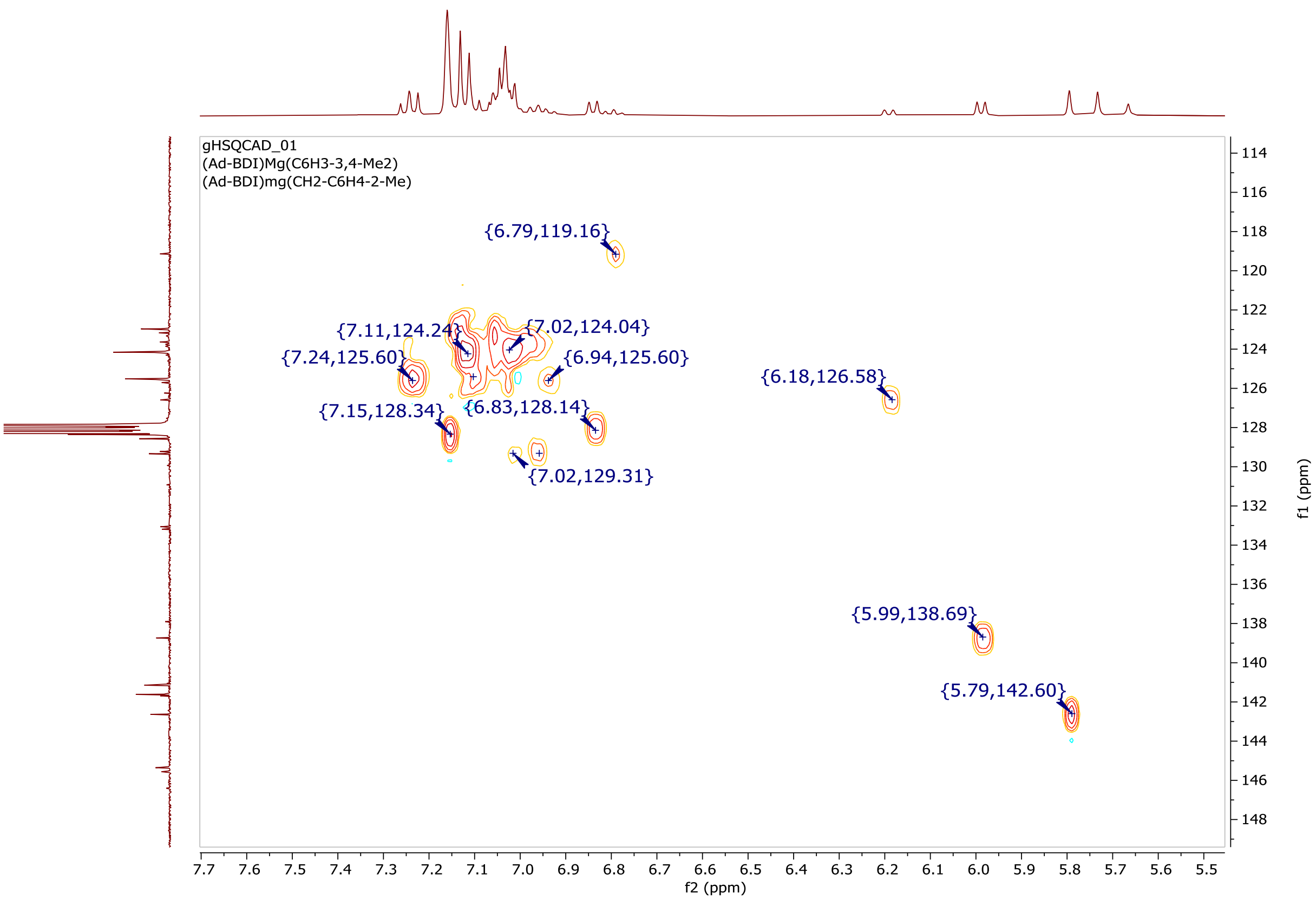

Figure S110: Expanded ^{13}C-^{1}H HSQC NMR (500 MHz, C_6D_6).

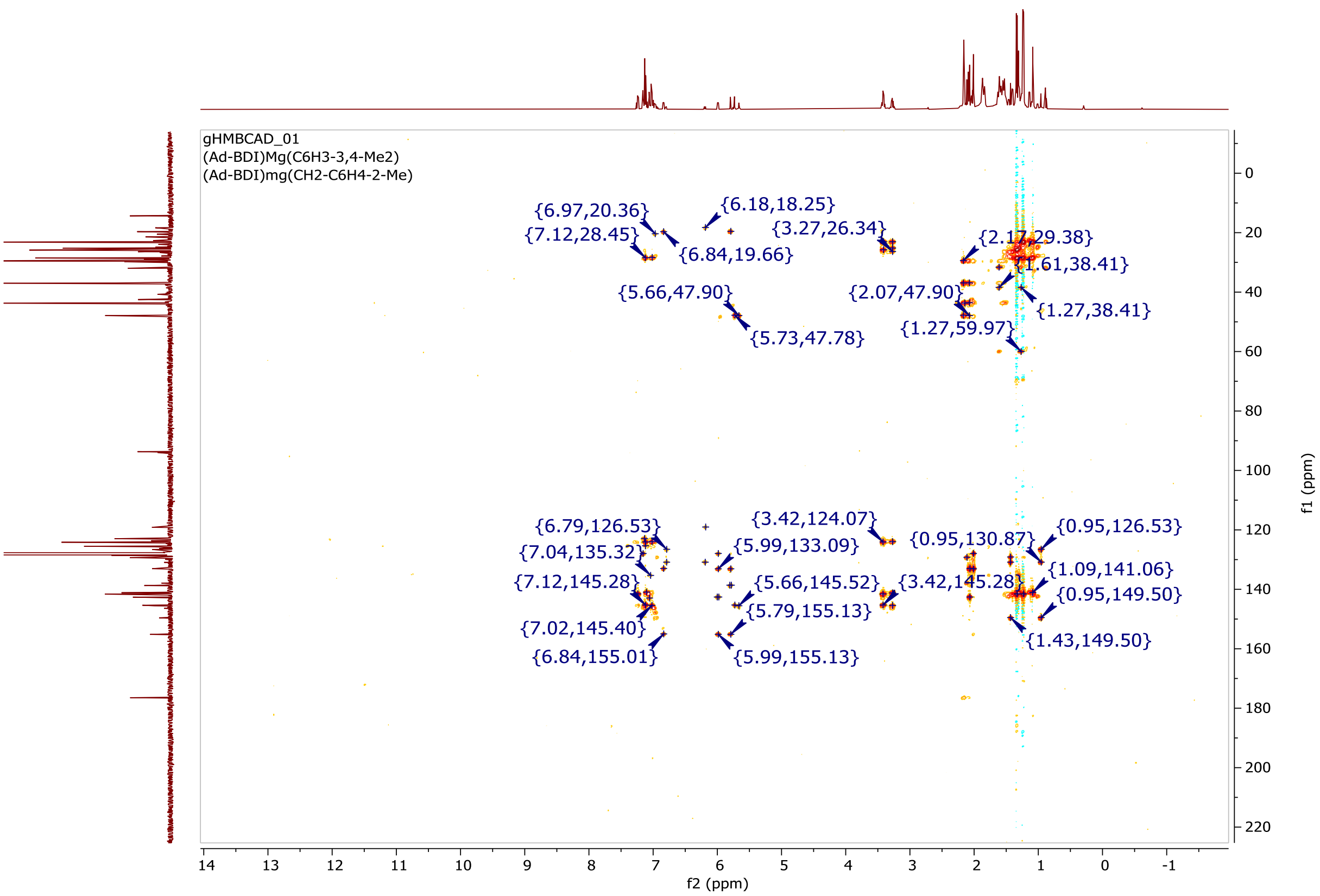

Figure S111: ^{13}C-^{1}H HMBC NMR (500 MHz, C_6D_6).

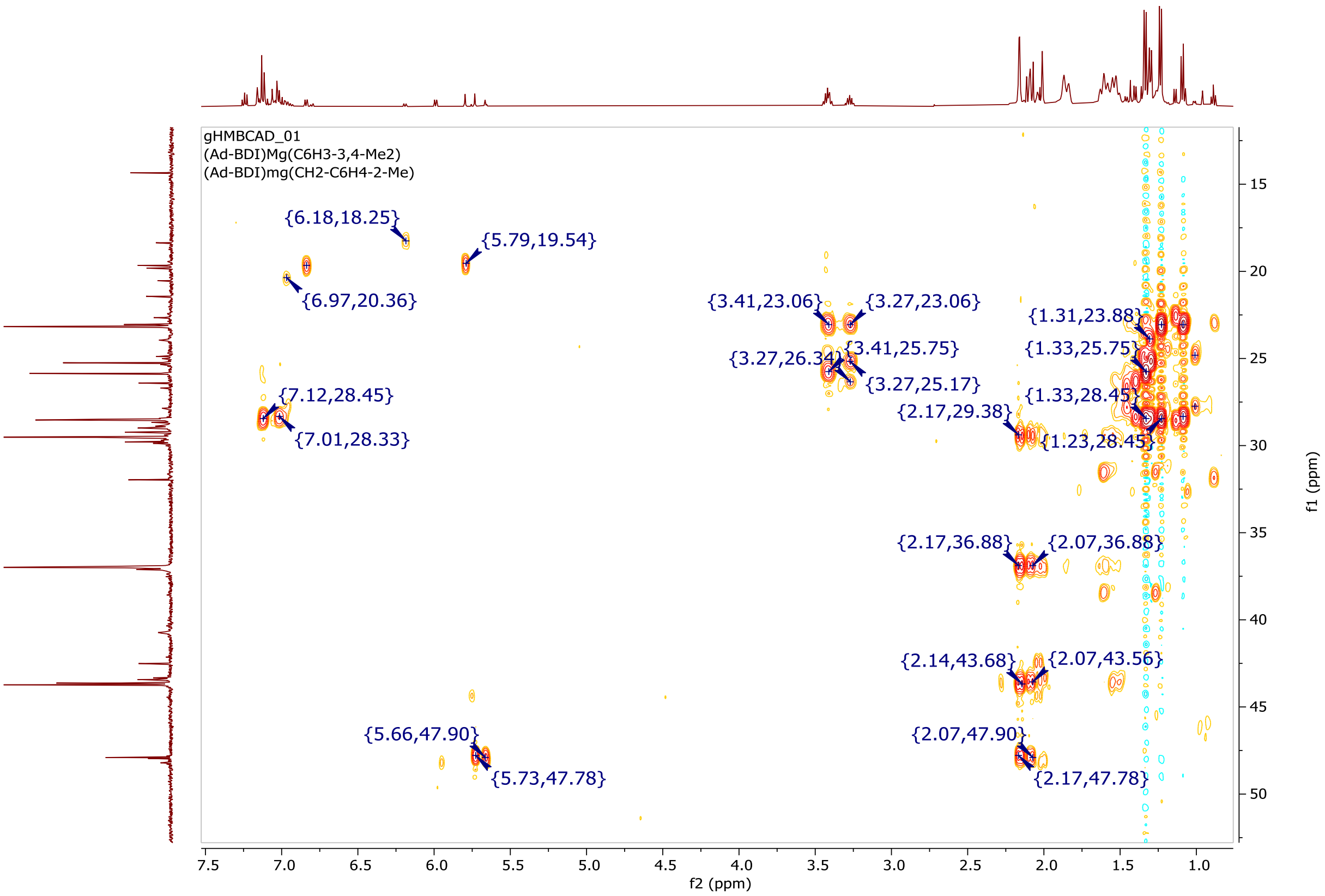

Figure S112: Expanded ^{13}C-^{1}H HMBC NMR (500 MHz, C_6D_6).

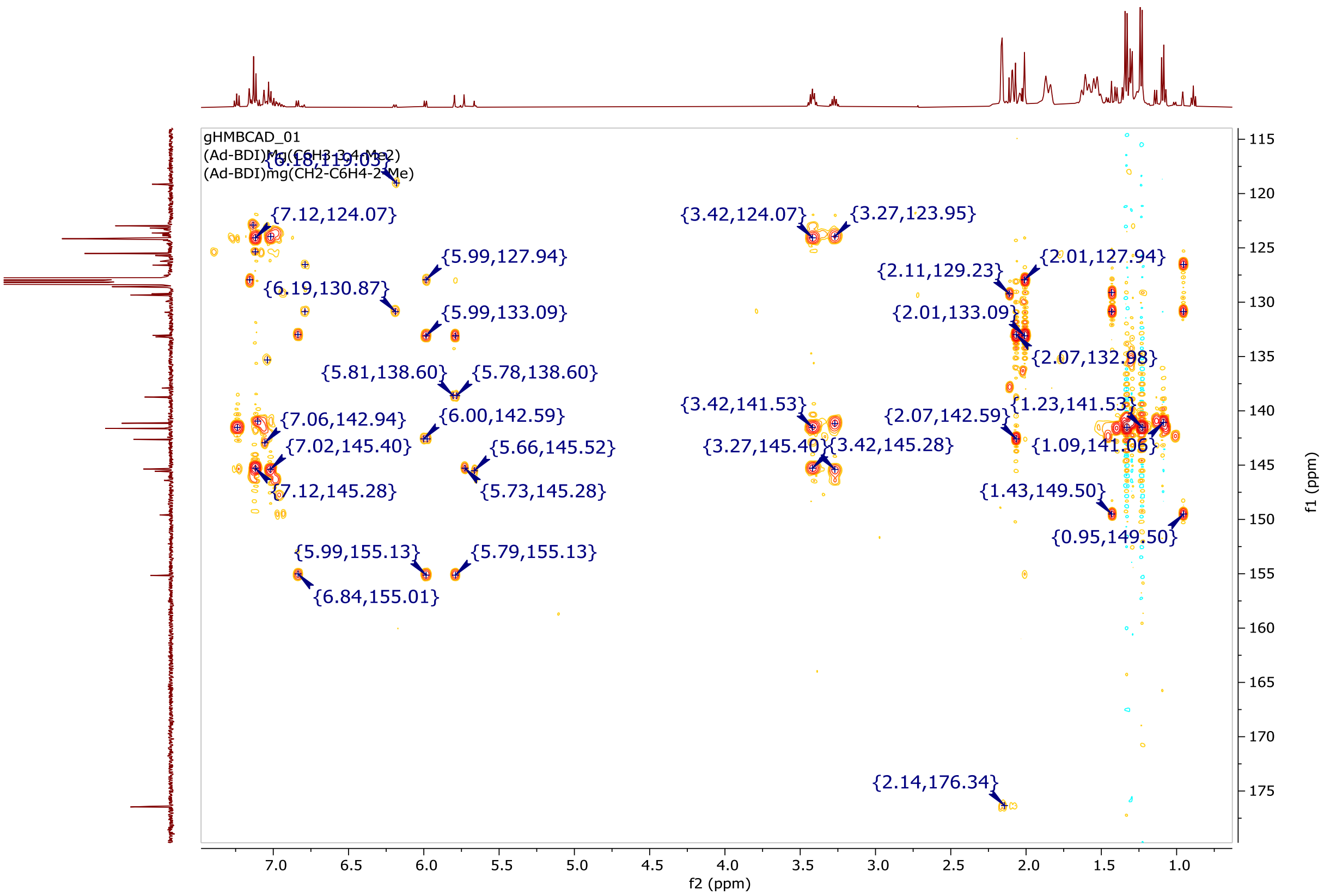

Figure S113: Expanded ^{13}C-^{1}H HMBC NMR (500 MHz, C_6D_6).

2.14 Reaction of [(^{Ad}BDI)Mg^{n}Bu] (1^{Ad}) with *meta*-xylene, synthesis and characterisation of [(^{Ad}BDI)Mg(C_6H_3-3,5-Me_2)] (7) and [(^{Ad}BDI)Mg($CH_2C_6H_4$-3-Me)] (7')

A J. Young's NMR tube was charged with 13 mg **1^{Ad}** (0.018 mmol) and 0.6 ml *meta*-xylene. The pale-yellow solution was heated at 100°C for 11 days. Analysis of the reaction solution by ^{1}H NMR indicated partial conversion of the starting complex and the formation of several BDI-containing products. A few colourless plate-like crystals of **7** suitable for X-ray diffraction analysis formed on the walls of the NMR tube above the meniscus. After a total of 16 days at 100°C, volatiles were removed under vacuum and the crude product redissolved in C_6D_6. Analysis by ^{1}H NMR spectroscopy showed 94% conversion to provide a mixture of **7** and **7'** in respective spectroscopic yields of 27% and 66%.

Alternatively, the reaction mixture was heated to 120°C for 10 days. Analysis of the crude product by ^{1}H NMR spectroscopy after removal of volatiles and dissolution in C_6D_6 indicated quantitative conversion of the starting complex to form a mixture of **7'** and **7** in respective spectroscopic yields of 80% and 20%. Slow evaporation of an *n*-hexane solution of this product mixture at room temperature provided pale-yellow block-shaped single-crystals suitable for X-ray diffraction analysis, the resulting structure solution of which was refined as a disordered co-crystalline mixture of [(^{Ad}BDI)Mg(CH_2-C_6H_4-3-CH_3)] and [(^{Ad}BDI)Mg(C_6H_3-3,5-Me_2)] in a 9:1 ratio. The crystals were isolated in 5.8 mg yield, 66%.

Analysis calculated for $C_{55}H_{73}MgN_2$ (mixture of isomers): C, 83.88; H, 9.47; N, 3.56%. Found: C, 82.17; H, 10.05; N, 3.36%. Despite repeated attempts, more accurate elemental analysis was not obtained.

^{1}H NMR of **7**' (500 MHz, C_6D_6) δ 7.16 – 7.10 (m, 2H, *p*-NC_6*H_3*-*i*Pr_2), 7.04 (d, *J* = 7.6 Hz, 4H, *m*-NC_6*H_3*-*i*Pr_2), 6.90 (t, *J* = 7.5 Hz, 1H, CH_2-*5*-C_6*H_3*-3-Me), 6.58 (d, *J* = 7.3 Hz, 1H, CH_2-*4*-C_6*H_3*-3-Me), 6.09 (d, *J* = 7.6 Hz, 1H, CH_2-*6*-C_6*H_3*-3-Me), 6.03 (d, *J* = 2.0 Hz, 1H, CH_2-*2*-C_6*H_3*-3-Me), 5.65 (s, 1H, *H*CC(Ndipp)), 3.28 (hept, *J* = 6.9 Hz, 4H, *H*C(CH_3)$_2$), 2.19 (s, 3H, $CH_2C_6H_3$-3-C*H_3*), 2.10 (d, *J* = 3.0 Hz, 12H, ^{Ad}CC*H_2*CH), 1.84 (t, *J* = 3.1 Hz, 6H, ^{Ad}C*H*), 1.60 (d, *J* = 11.9 Hz, 8H, ^{Ad}CHC*H_2*CH),* 1.52 (d, *J* = 12.1 Hz, 7H, ^{Ad}CHC*H_2*CH),* 1.31 (d, *J* = 6.9 Hz, 12H, HC(C*H_3*)$_2$), 1.13 (d, *J* = 6.9 Hz, 12H, HC(C*H_3*)$_2$), 1.04 (s, 2H, MgC*H_2*). *Inaccurate integral due to overlap with resonances of [(^{Ad}BDI)Mg(C_6H_3-3,5-Me_2)].

^{13}C NMR of **7**' (126 MHz, C_6D_6) δ 176.31 (N=*C*), 151.57 (CH_2-*1*-*C_6*H_3-3-Me), 145.29 (*ipso*-NC_6H_3-*i*Pr_2), 141.00 (*ortho*-N*C_6*H_3-*i*Pr_2), 136.35 (CH_2-*3*-*C_6*H_3-3-Me), 127.90 (CH_2-*5*-C_6*H_3*-3-Me),* 126.35 (CH_2-*2*-*C_6*H_3-3-Me), 125.43 (*p*-N*C_6*H_3-*i*Pr_2), 123.98 (*m*-N*C_6*H_3-*i*Pr_2), 122.83 (CH_2-*6*-*C_6*H_3-3-Me), 119.54 (CH_2-*4*-*C_6*H_3-3-Me), 93.78 (H*C*C(Ndipp), 47.87 (NC*^{Ad}C*), 43.63 (^{Ad}C*C*H_2CH), 36.96 (^{Ad}CH*C*H_2CH), 29.48 (^{Ad}*C*H), 28.45 ((H*C*(CH_3)$_2$)), 25.23 (HC(*C*H_3)$_2$), 23.21 (HC(*C*H_3)$_2$), 21.99 ($CH_2C_6H_3$-3-*C*H_3), 19.89 (Mg*C*H_2). *Overlaps with solvent resonance, identified *via* HSQC.

^{1}H NMR of **7** (500 MHz, C_6D_6) δ 7.22 (t, *J* = 7.7 Hz, 2H, *p*-NC_6*H_3*-*i*Pr_2), 7.11 (d, *J* = 7.9 Hz, 4H, *m*-NC_6*H_3*-*i*Pr_2), 6.73 (s, 1H, *4*-C_6*H_3*-3,5-Me_2), 5.72 (s, 1H, *H*CC(Ndipp)), 5.70 (d, *J* = 1.9 Hz, 2H, *2,6*-C_6*H_3*-3,5-Me_2), 3.40 (hept, *J* = 6.9 Hz, 4H, *H*C(CH_3)$_2$), 2.17 (s, 3H, C_6H_3-(C*H_3*)$_2$), 2.16 (d, *J* = 2.8 Hz, 12H, ^{Ad}CC*H_2*CH), 1.85 (br, ^{Ad}C*H*),* 1.65 – 1.46 (m, ^{Ad}CHC*H_2*CH),* 1.33 (d, *J* = 6.9 Hz, HC(C*H_3*)$_2$),* 1.21 (d, *J* = 6.9 Hz, 12H, HC(C*H_3*)$_2$). *Integral could not be accurately determined due to overlap with resonance of [(^{Ad}BDI)Mg(CH_2-C_6H_4-3-CH_3)] isomer.

^{13}C NMR of **7** (126 MHz, C_6D_6) δ 176.44 (N=*C*), 158.16 (Mg-*1*-*C_6*H_3-3,5-Me_2), 145.40 (*ipso*-NC_6*H_3*-*i*Pr_2), 141.59 (*ortho*-NC_6*H_3*-*i*Pr_2), 138.86 (*2,6*-*C_6*H_3-3,5-Me_2), 133.96 (*3,5*-*C_6*H_3-3,5-Me_2), 127.74 (*4*-*C_6*H_3-3,5-Me_2), 125.46 (*para*-NC_6H_3-*i*Pr_2), 124.15 (*meta*-NC_6*H_3*-*i*Pr_2), 93.62 (H*C*C(Ndipp)), 43.73 (^{Ad}C*C*H_2CH), 37.00 (^{Ad}CH*C*H_2CH), 29.52 (^{Ad}*C*H), 28.52 (H*C*(CH_3)$_2$), 25.83 (HC(*C*H_3)$_2$), 23.17 (HC(*C*H_3)$_2$), 21.99 (C_6H_3-(*C*H_3)$_2$).

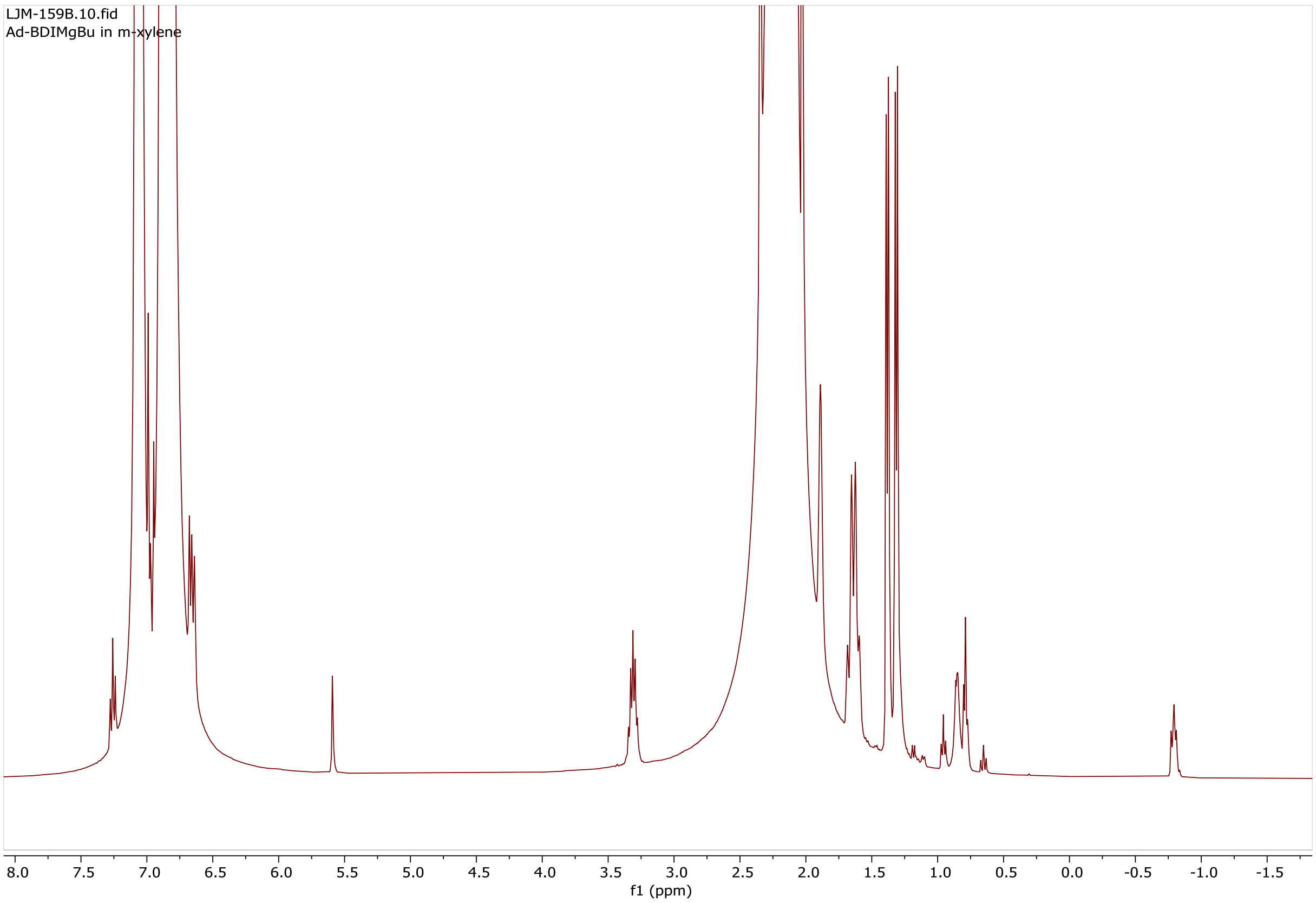

Figure S114: ^{1}H NMR spectrum (400 MHz, *m*-xylene) of the reaction mixture before heating.

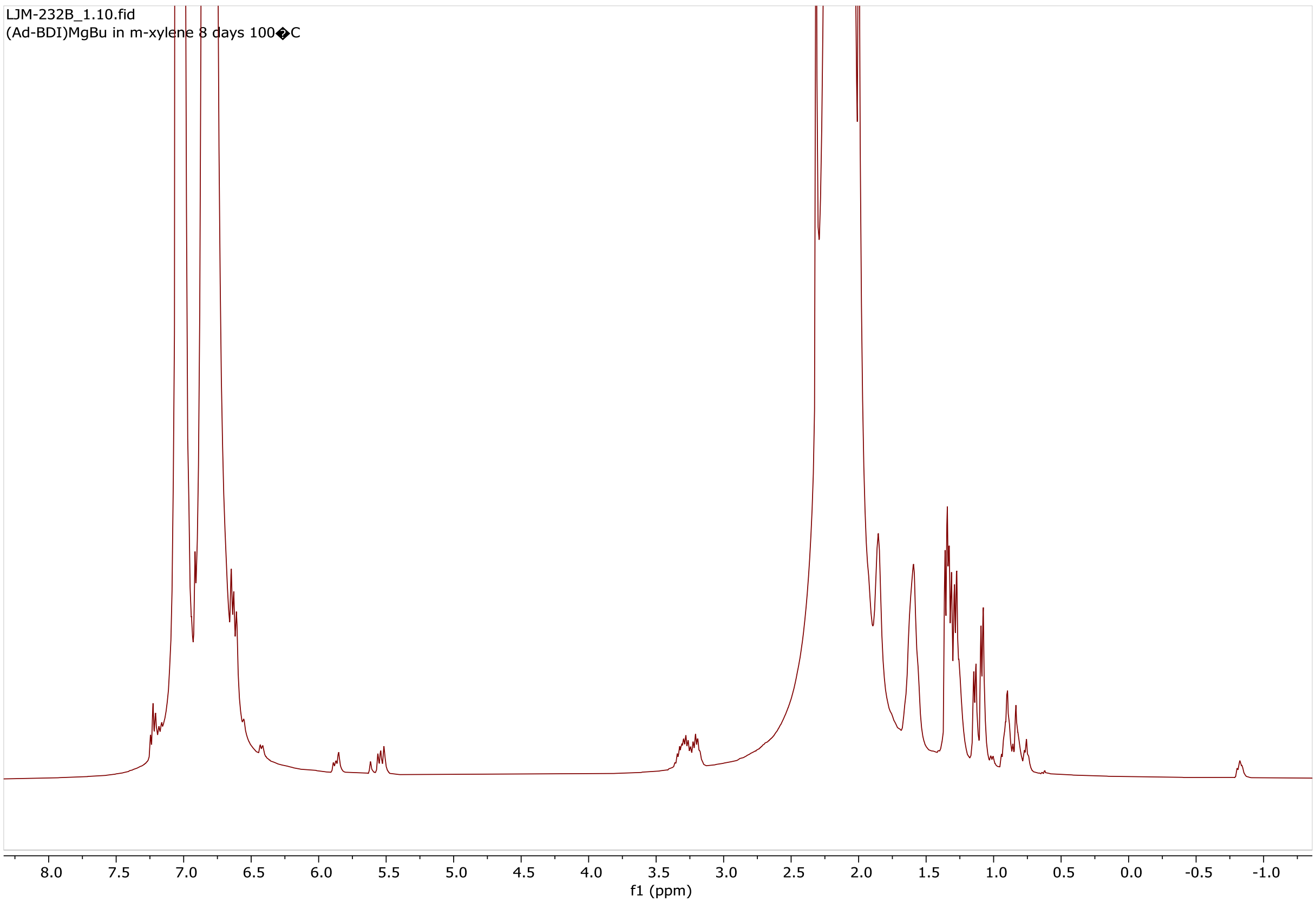

Figure S115: ^{1}H NMR spectrum (400 MHz, *m*-xylene) of the reaction mixture after 8 days at 100°C.

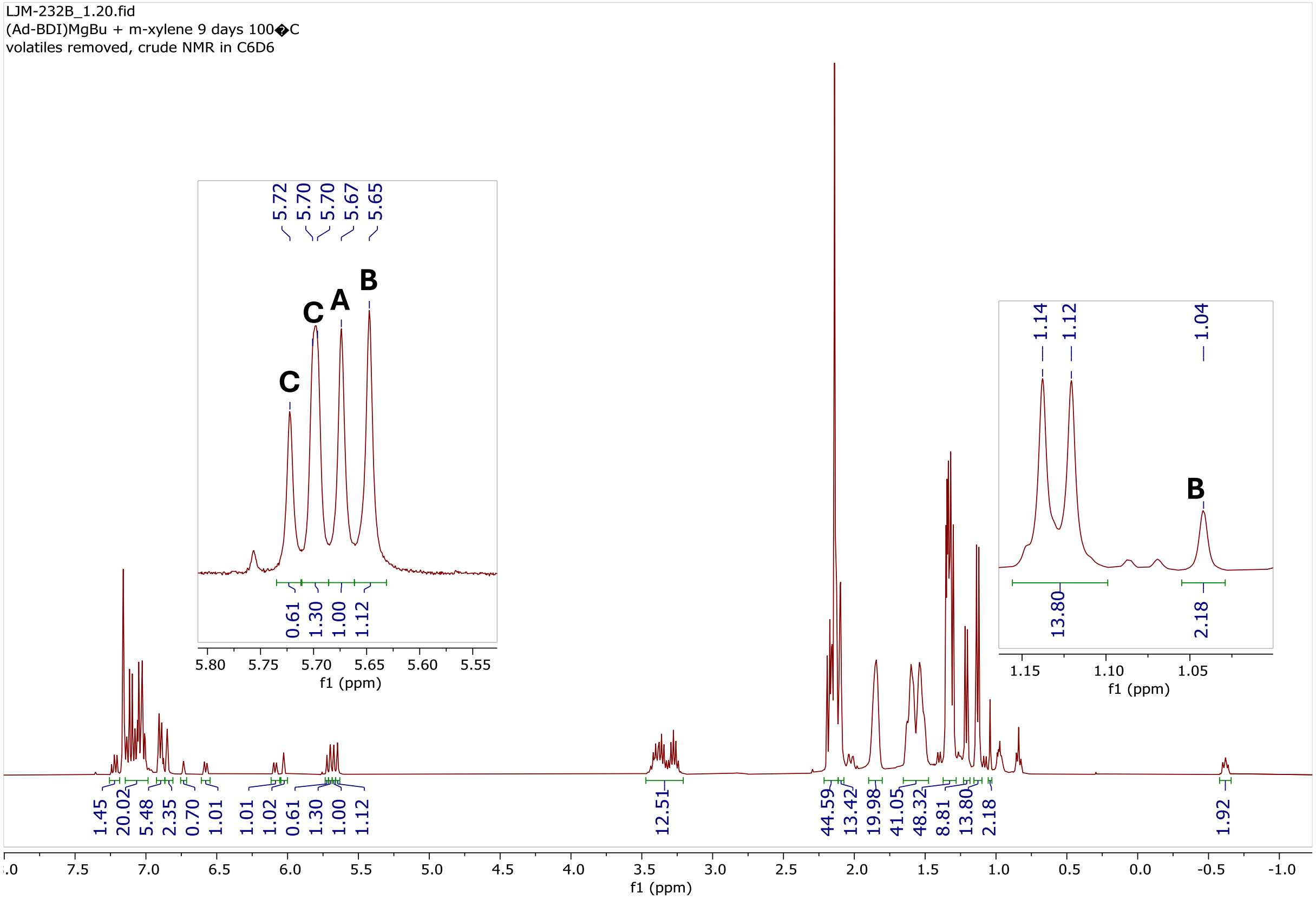

Figure S116: ^{1}H NMR spectrum (400 MHz, C_6D_6) of the product mixture after 8 days at 100°C, with the backbone BDI-methine region and the benzylic methylene highlighted; A = **1^{Ad}**, B = **7'**, C = **7**.

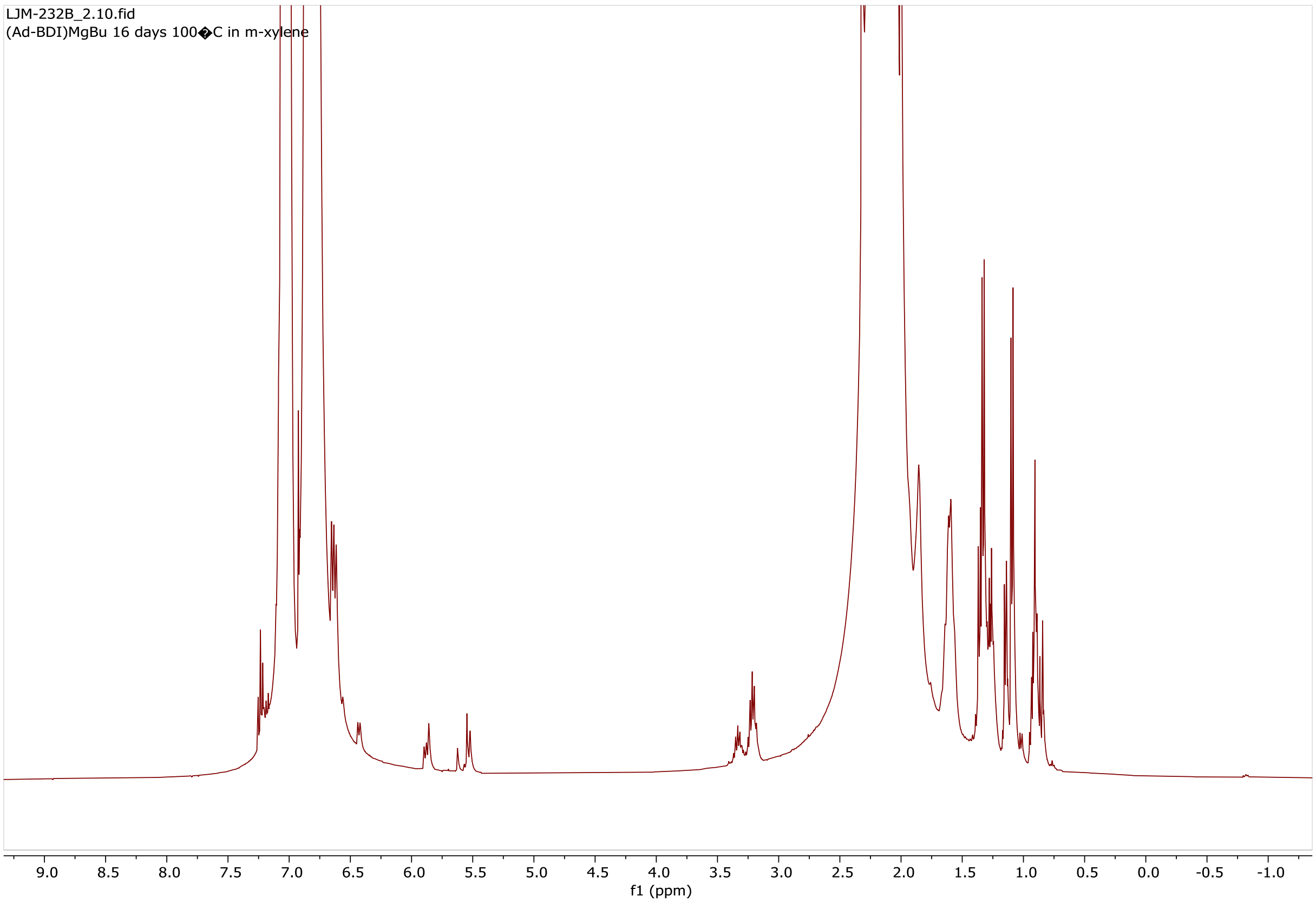

Figure S117: ^{1}H NMR spectrum (400 MHz, *m*-xylene) of the reaction mixture after 16 days at 100°C.

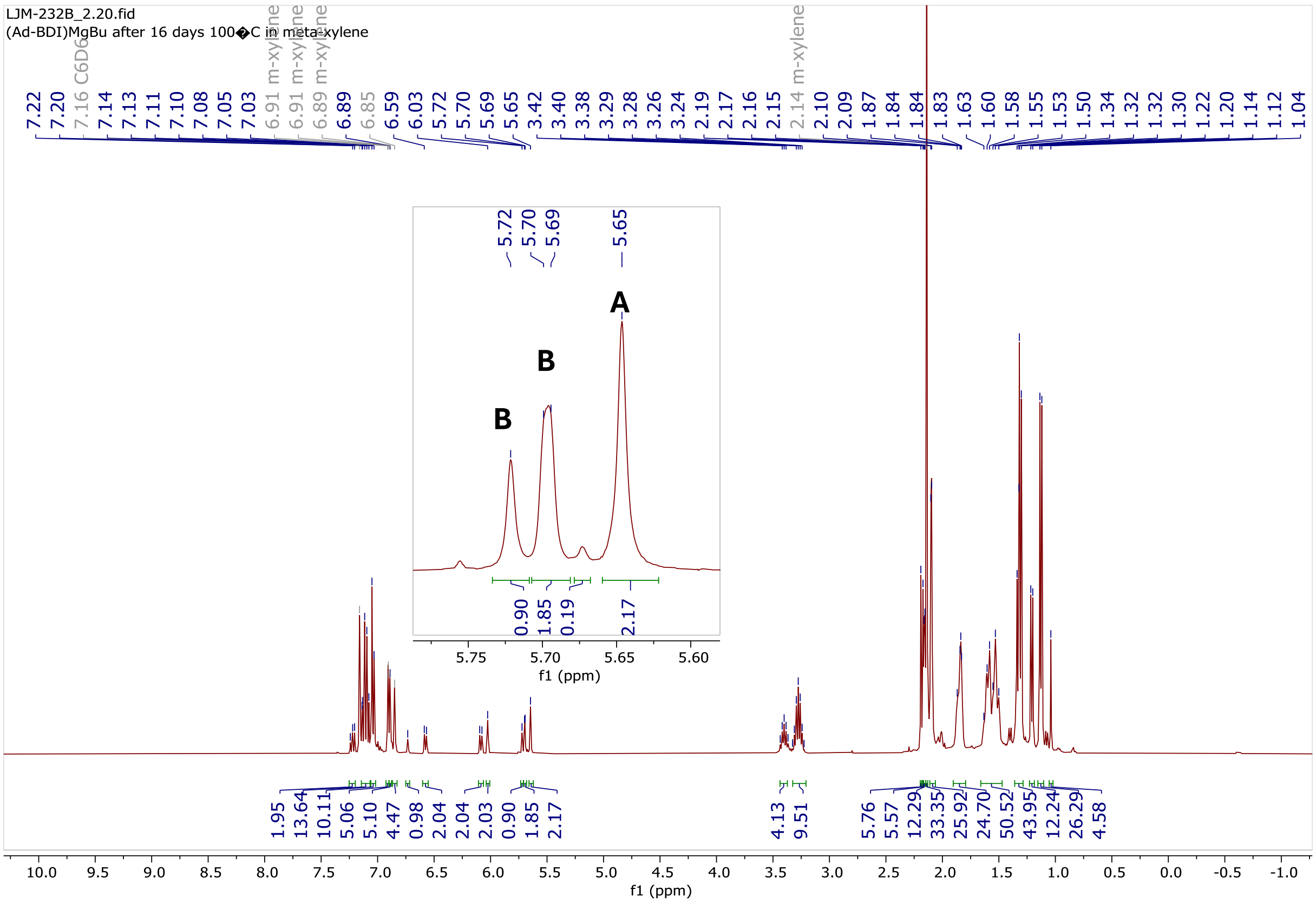

Figure S118: ^{1}H NMR spectrum (500 MHz, C_6D_6) of the product mixture after 16 days at 100°C, showing **7** as minor product (B) and **7'** as major product (A). Integrals are normalised with respect to **7**.

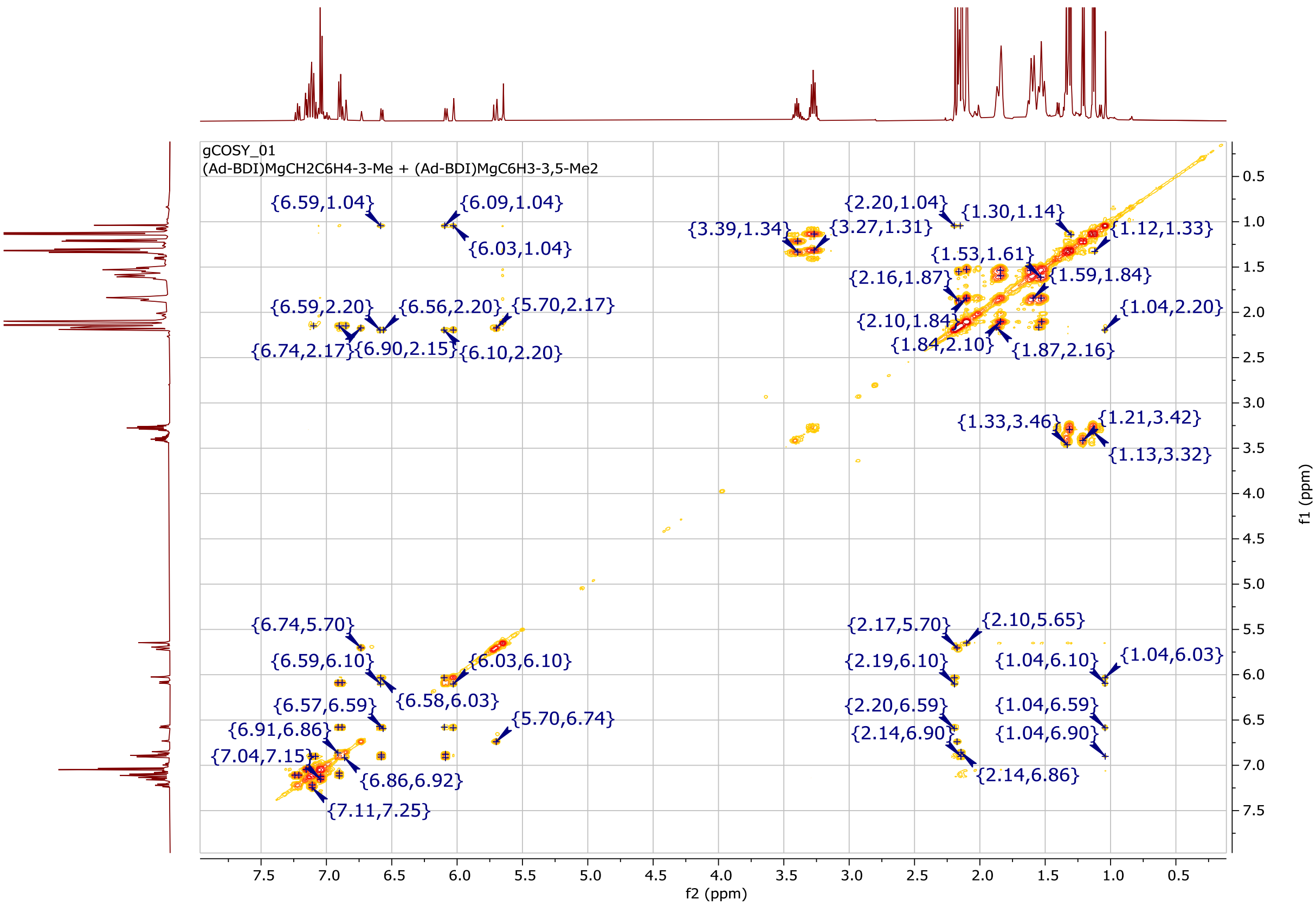

Figure S119: ^{1}H-^{1}H COSY NMR spectrum (500 MHz, C_6D_6) of the product mixture after 16 days at 100°C.

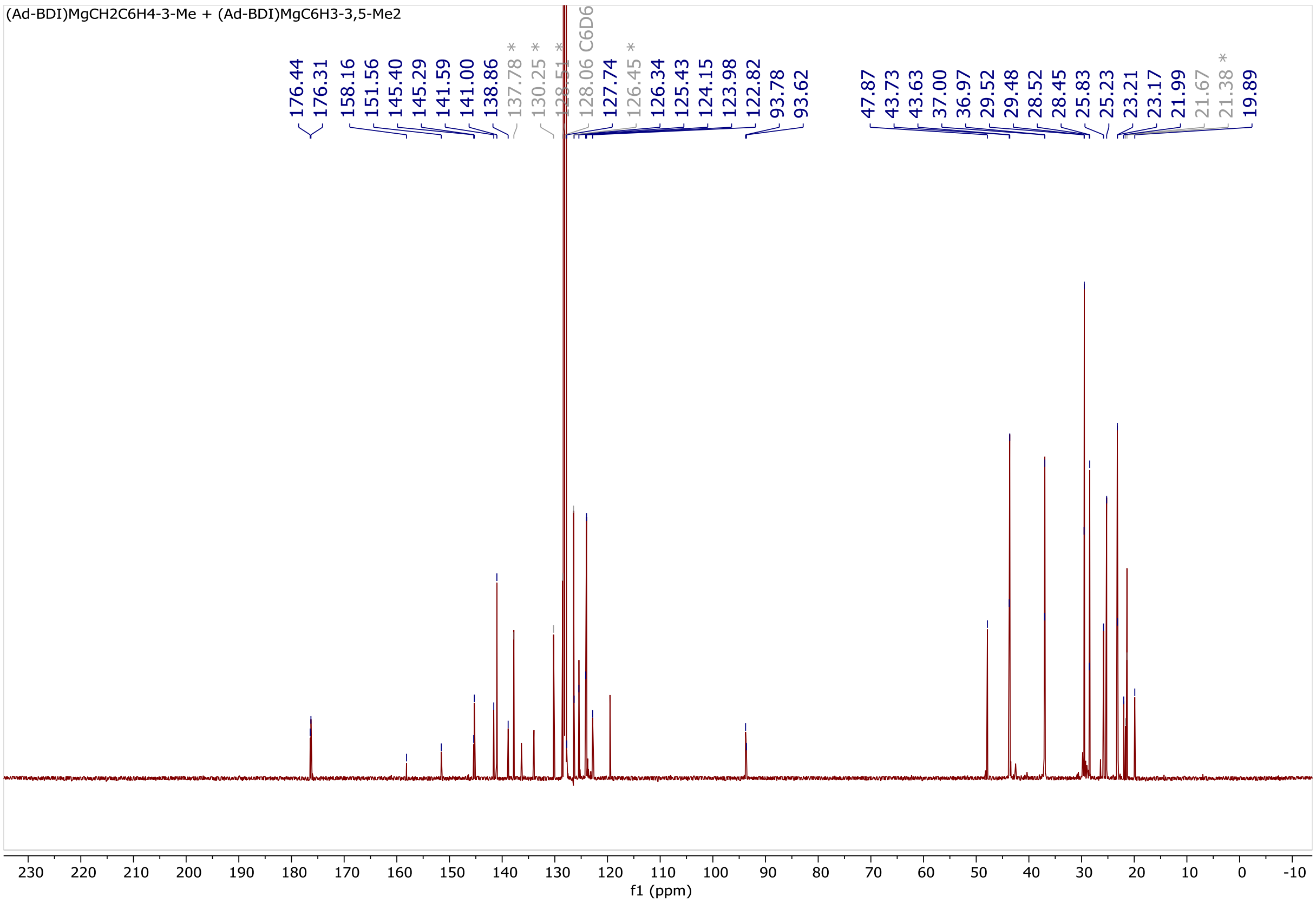

Figure S120: $^{13}C\{^1H\}$ NMR (126 MHz, C_6D_6) of the product mixture after 16 days at 100°C. *Residual *m*-xylene impurity.

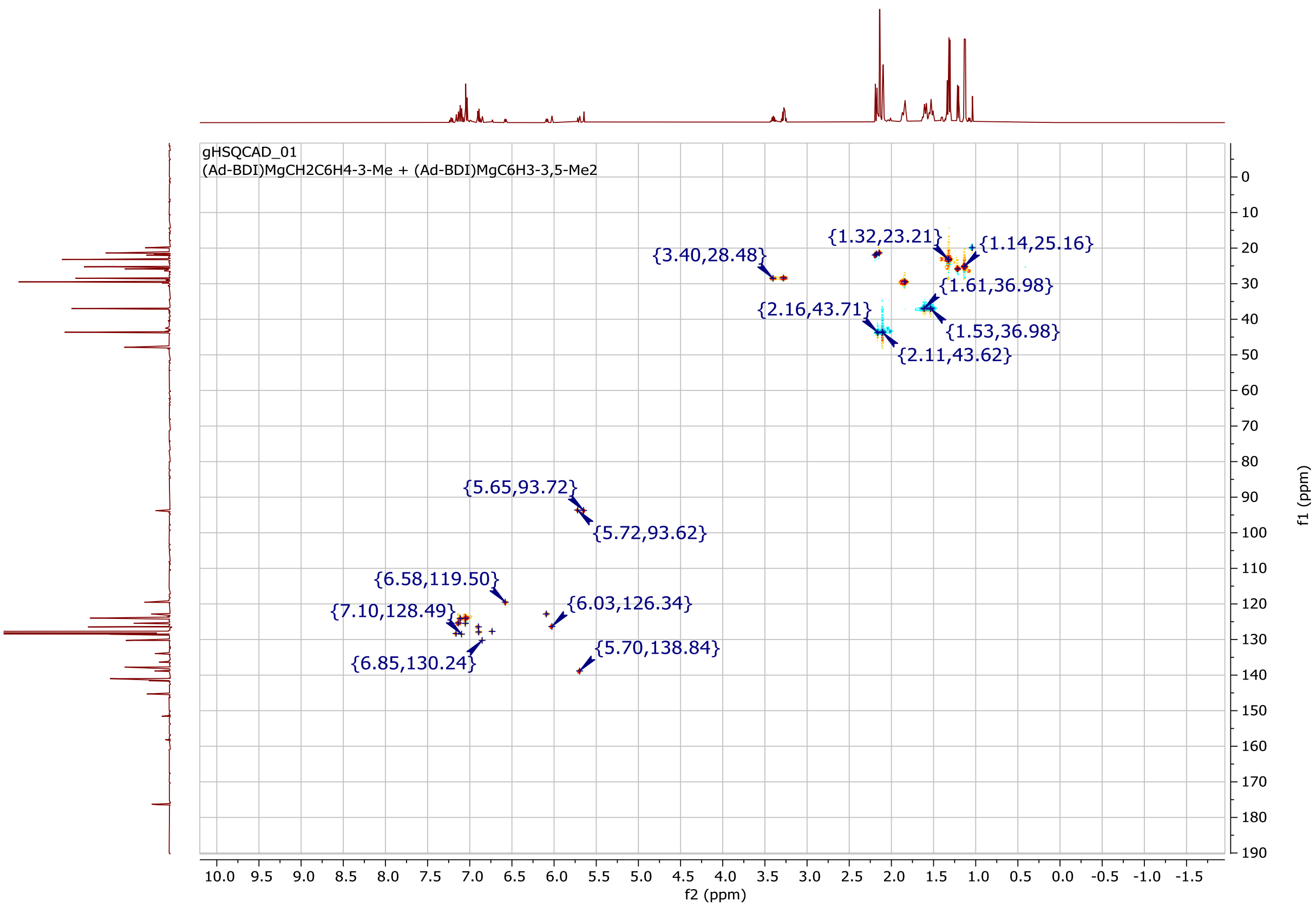

Figure S121: ^{13}C-^1H HSQC NMR (126 MHz, C_6D_6) of the product mixture after 16 days at 100°C.

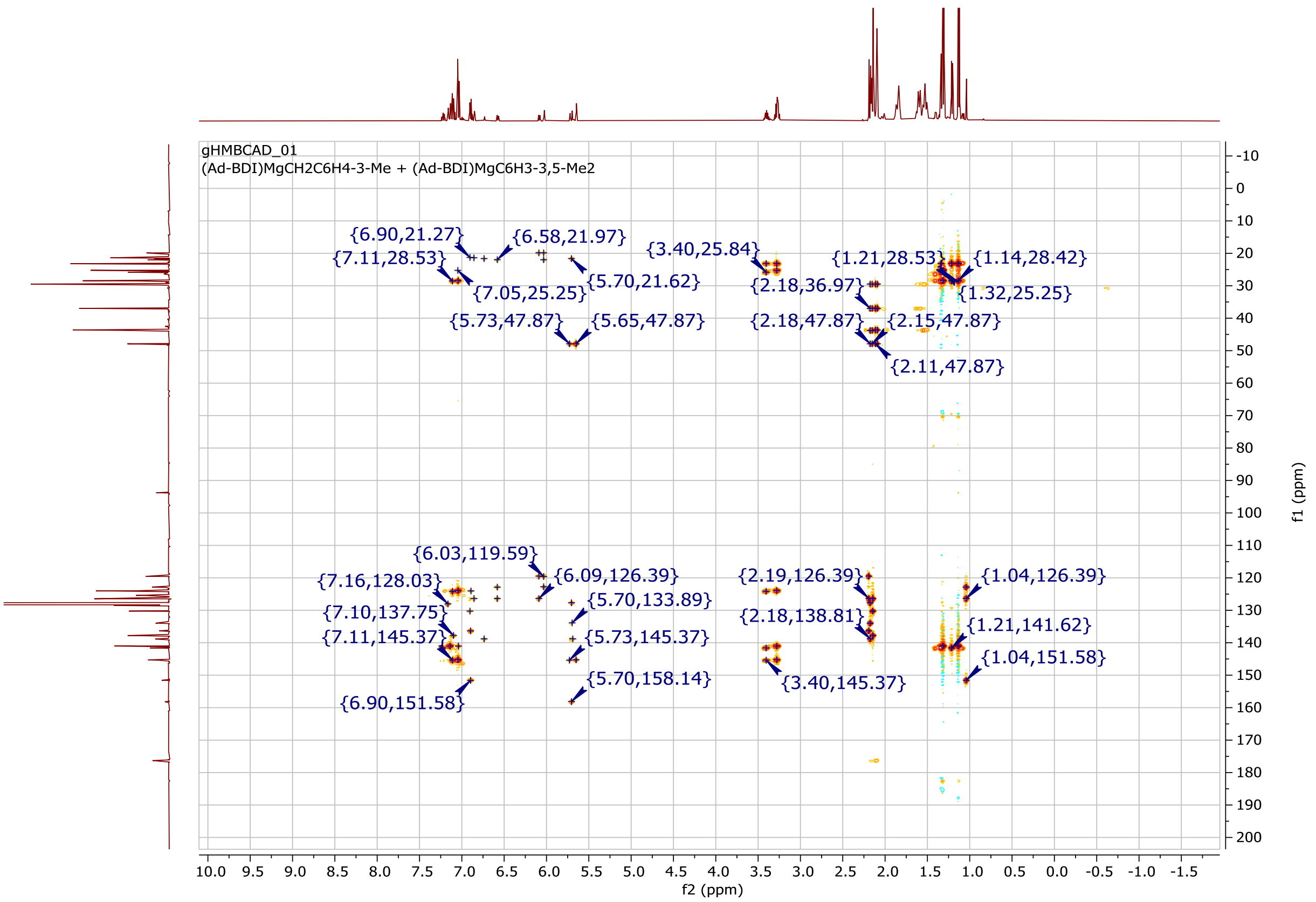

Figure S122: ^{13}C-^{1}H HMBC NMR (126 MHz, C_6D_6) of the product mixture after 16 days at 100°C.

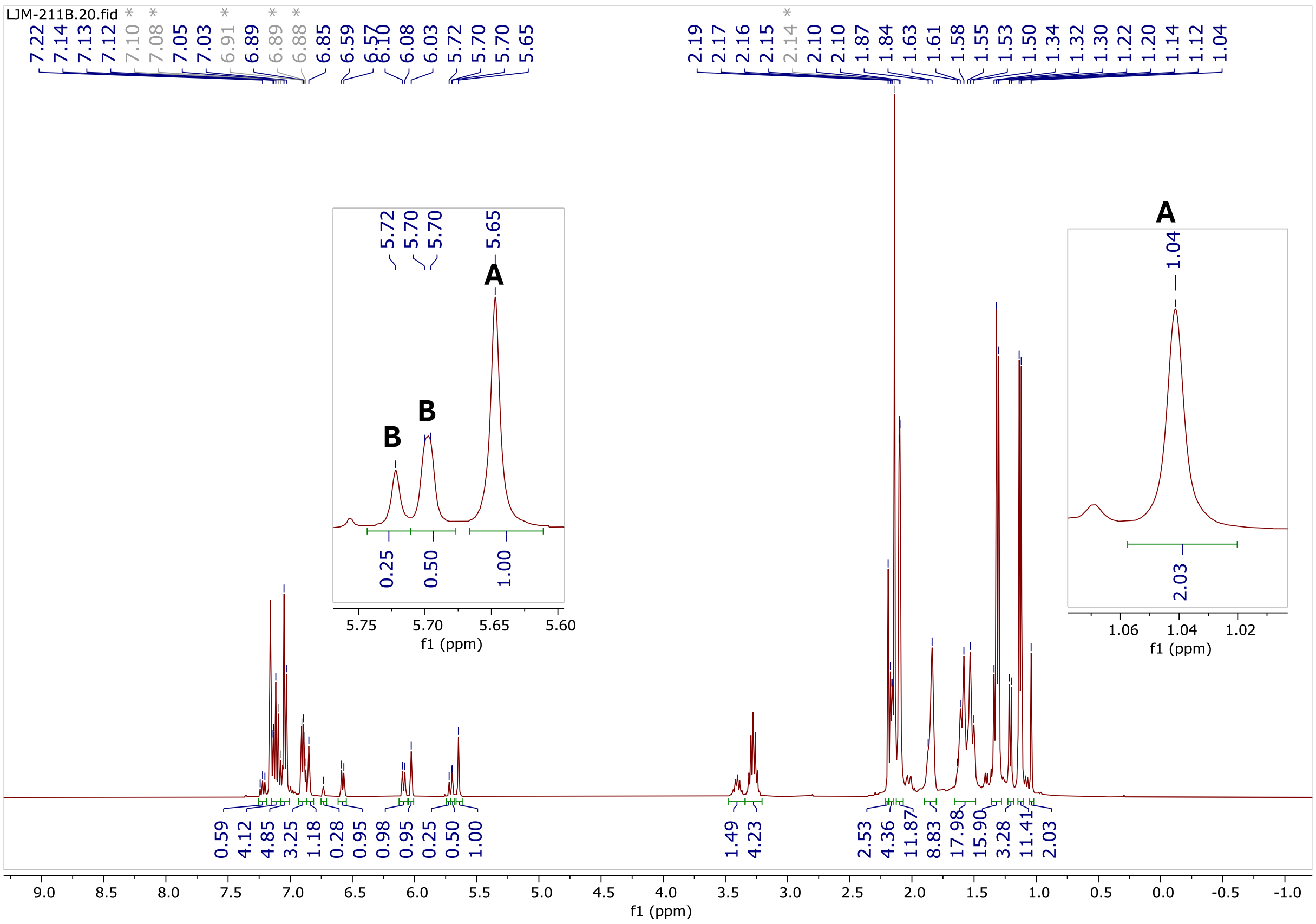

Figure S123: ^{1}H NMR spectrum (400 MHz, C_6D_6) of the product mixture after 10 days at 120°C, showing **7** as minor product (B) and **7'** as major product (A). Integrals are normalised with respect to **7'**. *Residual *meta*-xylene.

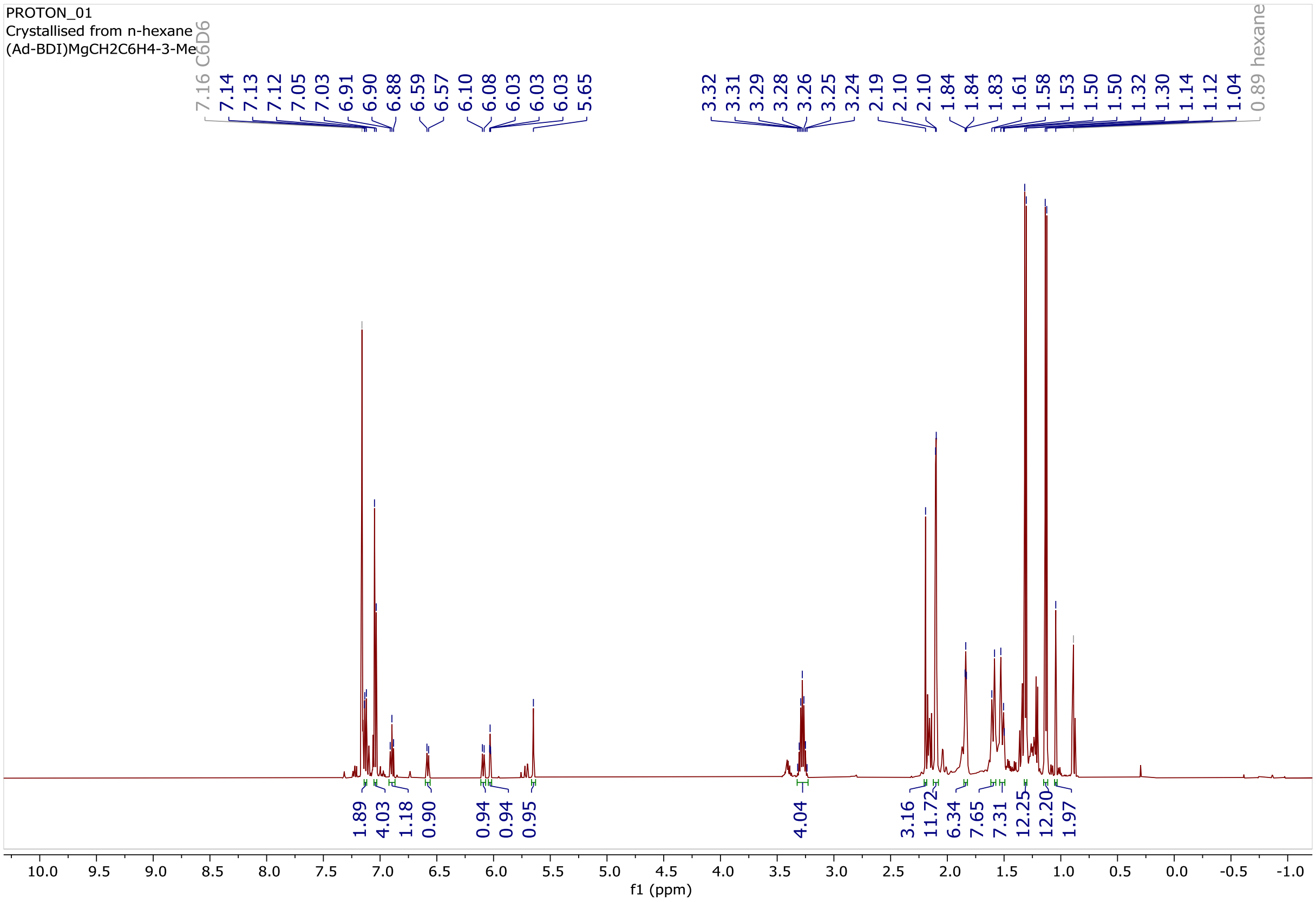

Figure S124: ^{1}H NMR (500 MHz, C_6D_6) of crystals of **7'** (contains 10% **7**).

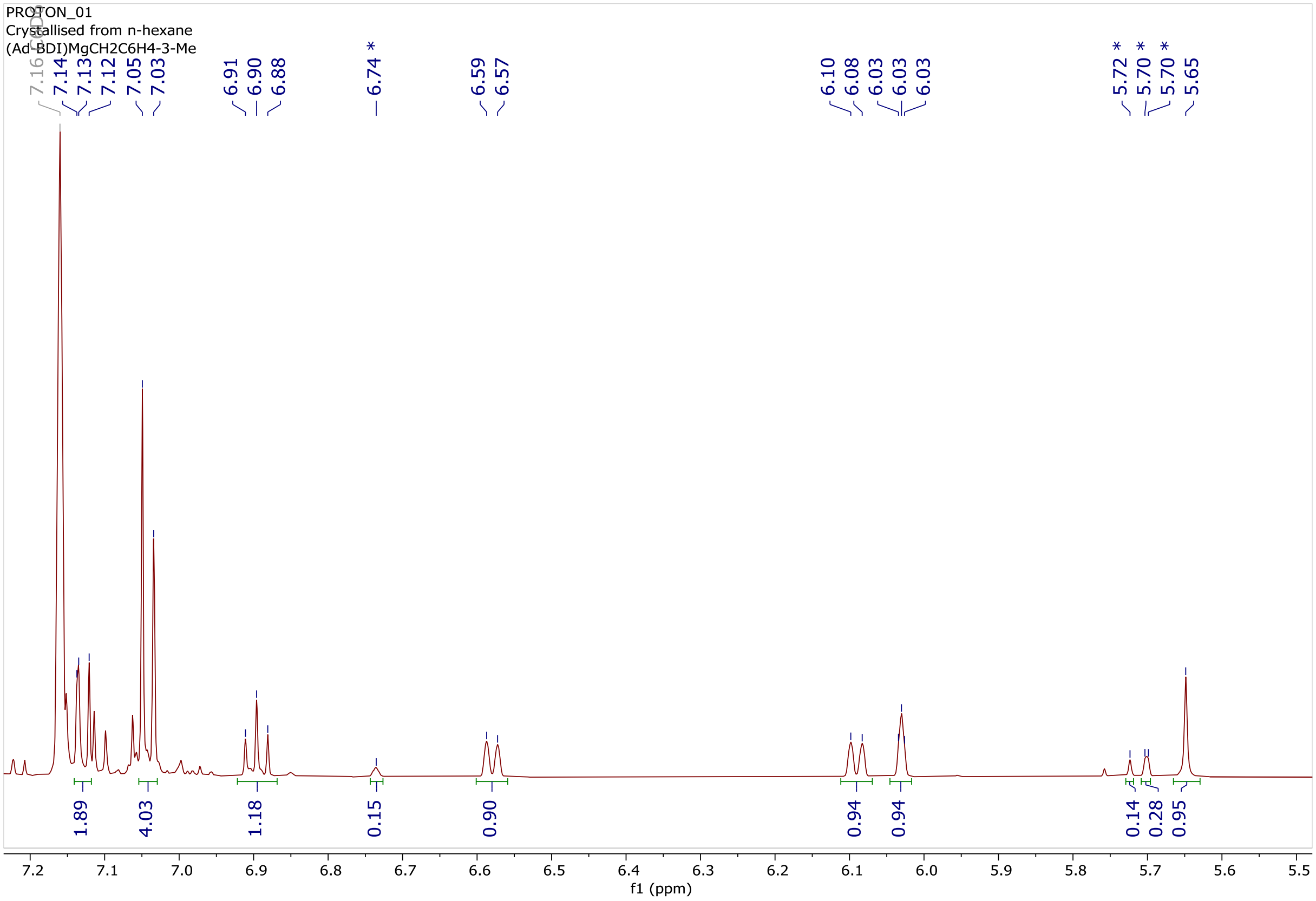

Figure S125: Expanded ^{1}H NMR (500 MHz, C_6D_6) of crystals of **7'**. *Co-crystalline **7**.

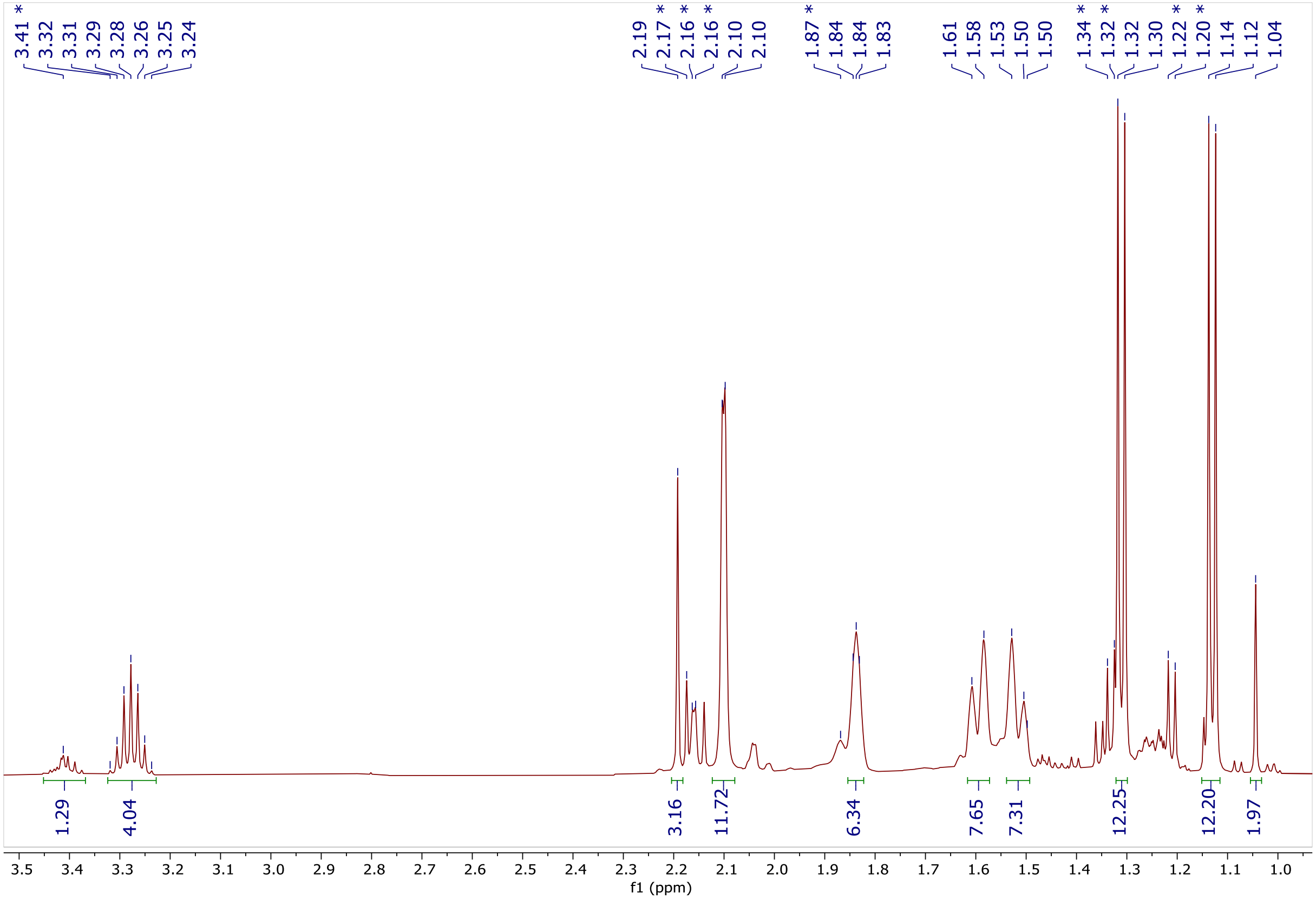

Figure S126: Expanded ^{1}H NMR (500 MHz, C_6D_6) of crystals of **7'**. *Co-crystalline **7**.

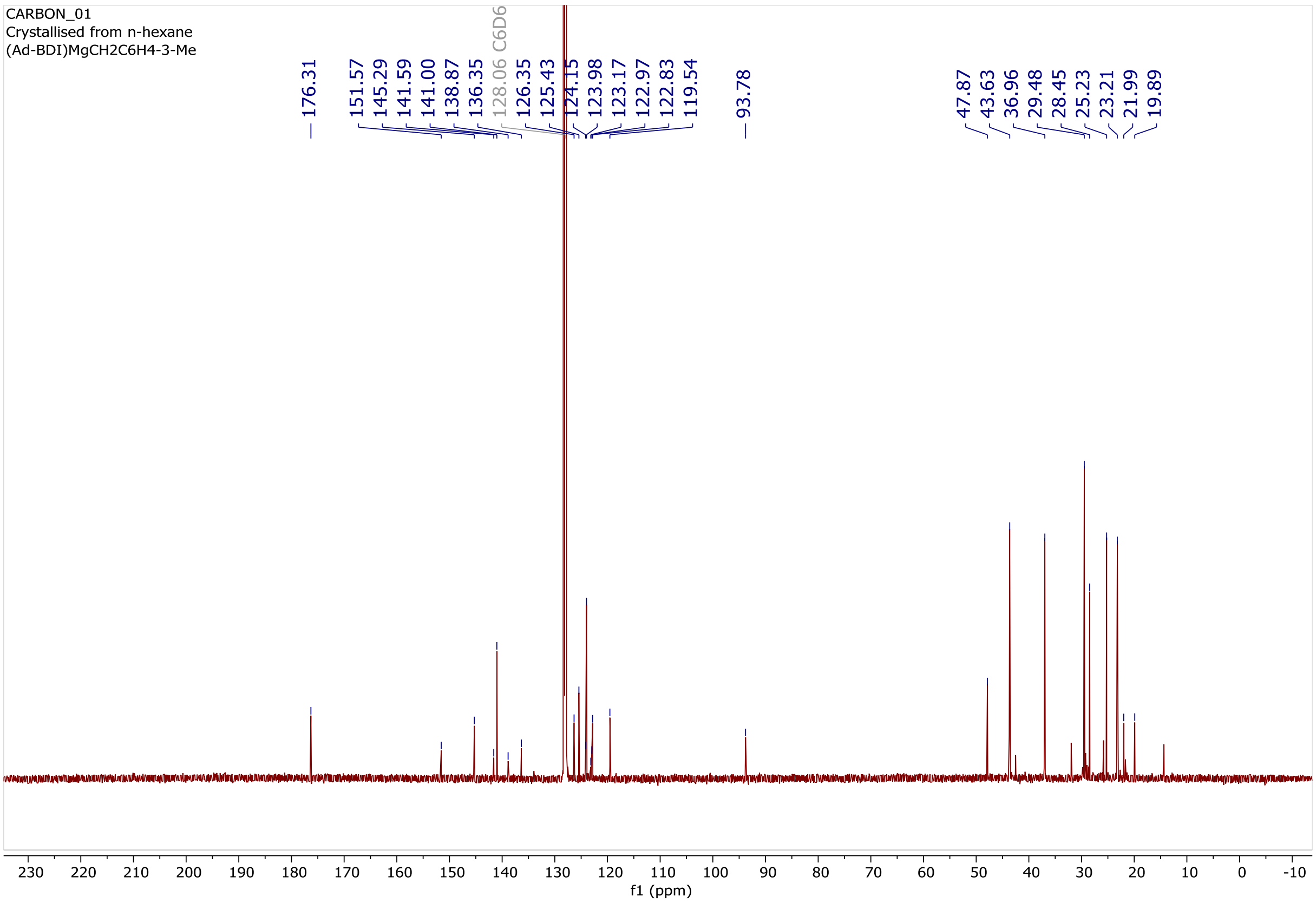

Figure S127: $^{13}C\{^{1}H\}$ NMR of **7'** (126 MHz, C_6D_6).

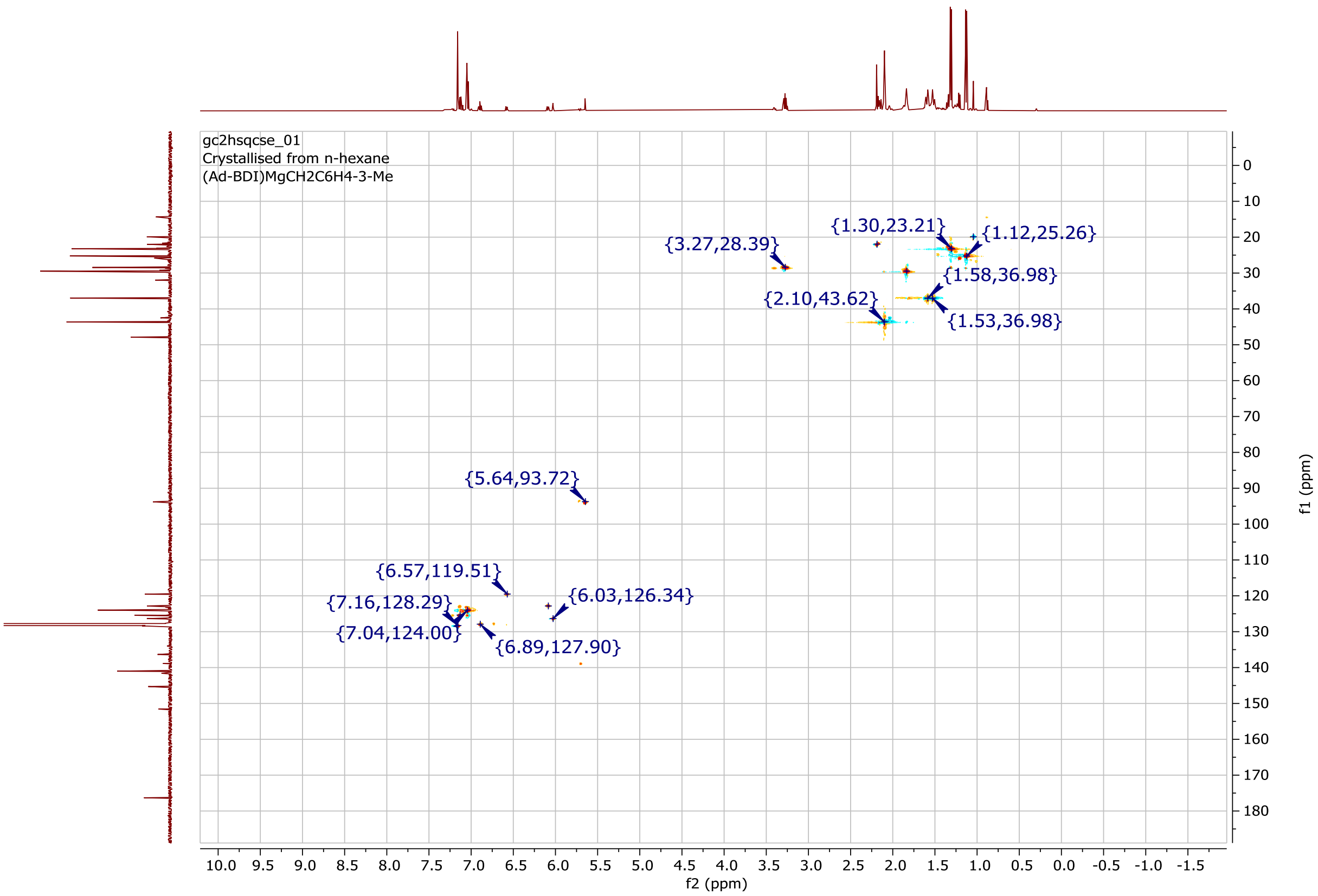

Figure S128: ^{13}C-^{1}H HSQC NMR of **7'** (126 MHz, C_6D_6).

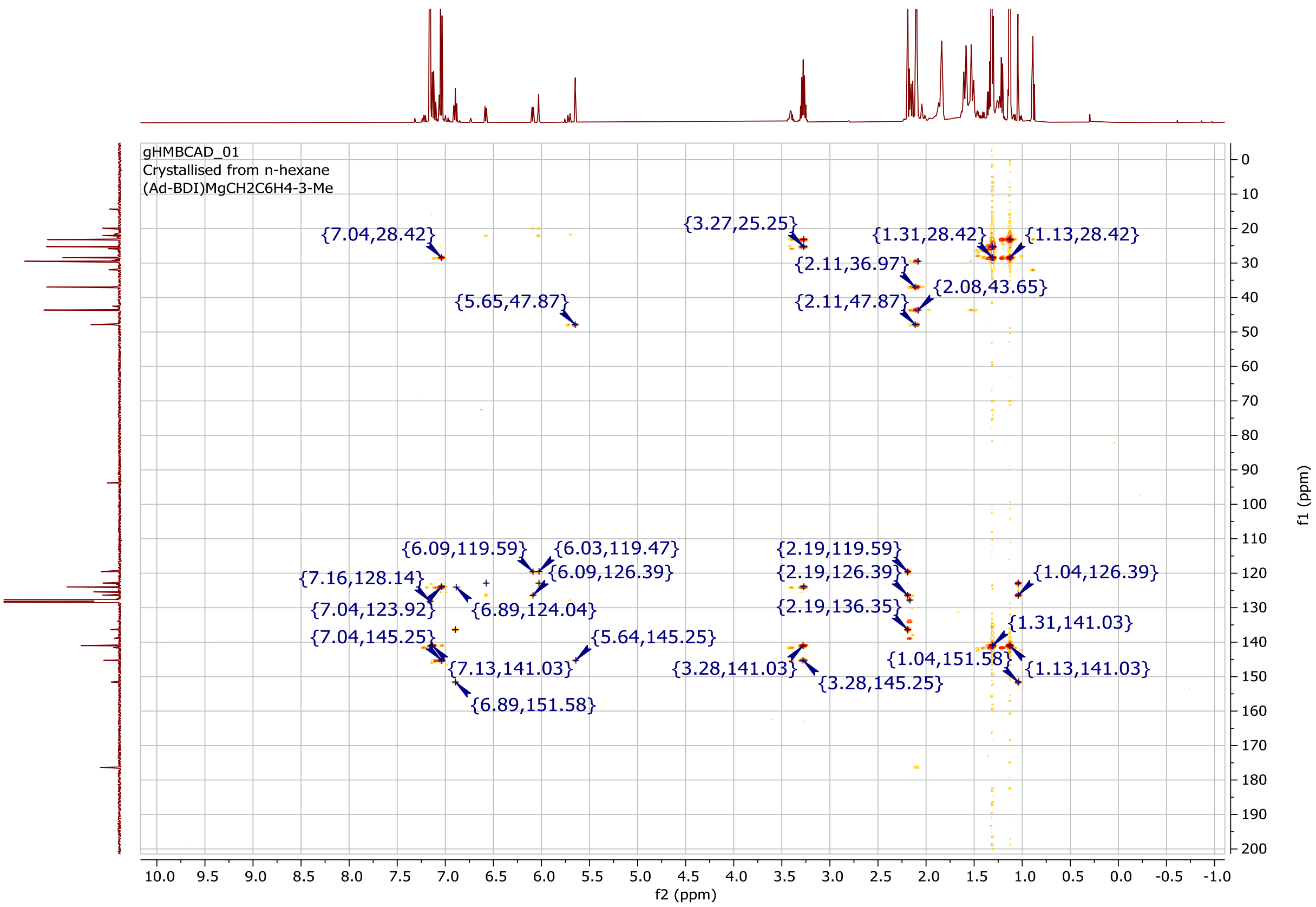

Figure S129: ^{13}C-^{1}H HMBC NMR of **7'** (126 MHz, C_6D_6).

2.15 Reaction of [(^{Ad}BDI)Mg^{n}Bu] (**1^{Ad}**) with *para*-xylene, synthesis and characterisation of [(^{Ad}BDI)Mg($CH_2C_6H_4$-4-Me)] (8)

A J. Young's NMR tube containing a 0.6 ml *para*-xylene solution of 14 mg **1^{Ad}** (0.019 mmol) was heated at 120°C for 10 days. Volatiles were removed under vacuum to provide the product in >95% yield, as determined by ^{1}H NMR in C_6D_6. Recrystallisation from an *n*-hexane/toluene mixture at -30°C provided 6 mg **8** in 66% yield as pale-yellow rod-shaped single crystals suitable for X-ray diffraction analysis.

Analysis calculated for $C_{55}H_{73}MgN_2$: C, 83.88; H, 9.47; N, 3.56%. Found: C, 81.59; H, 9.37; N, 3.17%.

^{1}H NMR (500 MHz, C_6D_6) δ 7.14 (m, *J* = 7.1 Hz,* 2H, *p*-NC_6*H_3*-*i*Pr_2), 7.05 (d, *J* = 6.6 Hz, 4H, *m*-NC_6*H_3*-*i*Pr_2), 6.78 (d, *J* = 7.7 Hz, 2H, Mg-CH_2-*2,6*-C_6*H_4*-4-CH_3), 6.14 (d, *J* = 8.0 Hz, 2H, Mg-CH_2-*3,5*-C_6*H_4*-4-CH_3), 5.65 (s, 1H, *H*CC(Ndipp), 3.28 (hept, *J* = 7.0 Hz, 4H, *H*C(CH_3)$_2$), 2.24 (s, 3H, Mg-CH_2-C_6*H_4*-*4*-C*H_3*), 2.10 (d, *J* = 3.0 Hz, 12H, ^{Ad}CC*H_2*CH), 1.86 – 1.81 (m, 7H, ^{Ad}C*H*),#1.60 (dm, *J* = 12.3 Hz, 9H, ^{Ad}CHC*H_2*CH),# 1.52 (dm, *J* = 12.4 Hz, 8H, ^{Ad}CHC*H_2*CH),# 1.31 (d, *J* = 6.9 Hz, 12H, HC(C*H_3*)$_2$), 1.13 (d, *J* = 6.9 Hz, 12H, HC(C*H_3*)$_2$), 1.03 (s, Mg-C*H_2*). *Overlaps with solvent peak. #Inaccurate integral.

^{13}C NMR (126 MHz, C_6D_6) δ 176.29 (N=*C*), 148.38 (Mg-CH_2-*1*-*C*$_6H_4$-4-CH_3), 145.36 (*ipso*-NC_6H_3-*i*Pr_2), 140.99 (*ortho*-NC_6H_3-*i*Pr_2), 128.65 (Mg-CH_2-*3,5*-*C*$_6H_4$-4-CH_3), 126.80 (Mg-CH_2-*4*-*C*$_6H_4$-4-CH_3), 125.63 (Mg-CH_2-*2,6*-*C*$_6H_4$-4-CH_3), 124.06 (*meta*-N*C*$_6H_3$-*i*Pr_2), 122.97 (*para*-N*C*$_6H_3$-*i*Pr_2), 93.76 (H*C*C(Ndipp)), 47.87 (NC^{Ad}*C*), 43.64 (^{Ad}*C*CH_2CH), 36.97 (^{Ad}CH*C*H_2CH), 29.48 (^{Ad}C*H*), 28.44 (H*C*(CH_3)$_2$), 25.28 (HC(*C*H_3)$_2$), 23.19 (HC(*C*H_3)$_2$), 21.09 (Mg-CH_2-C_6*H_4*-*4*-CH_3), 19.13 (Mg-*C*H_2).

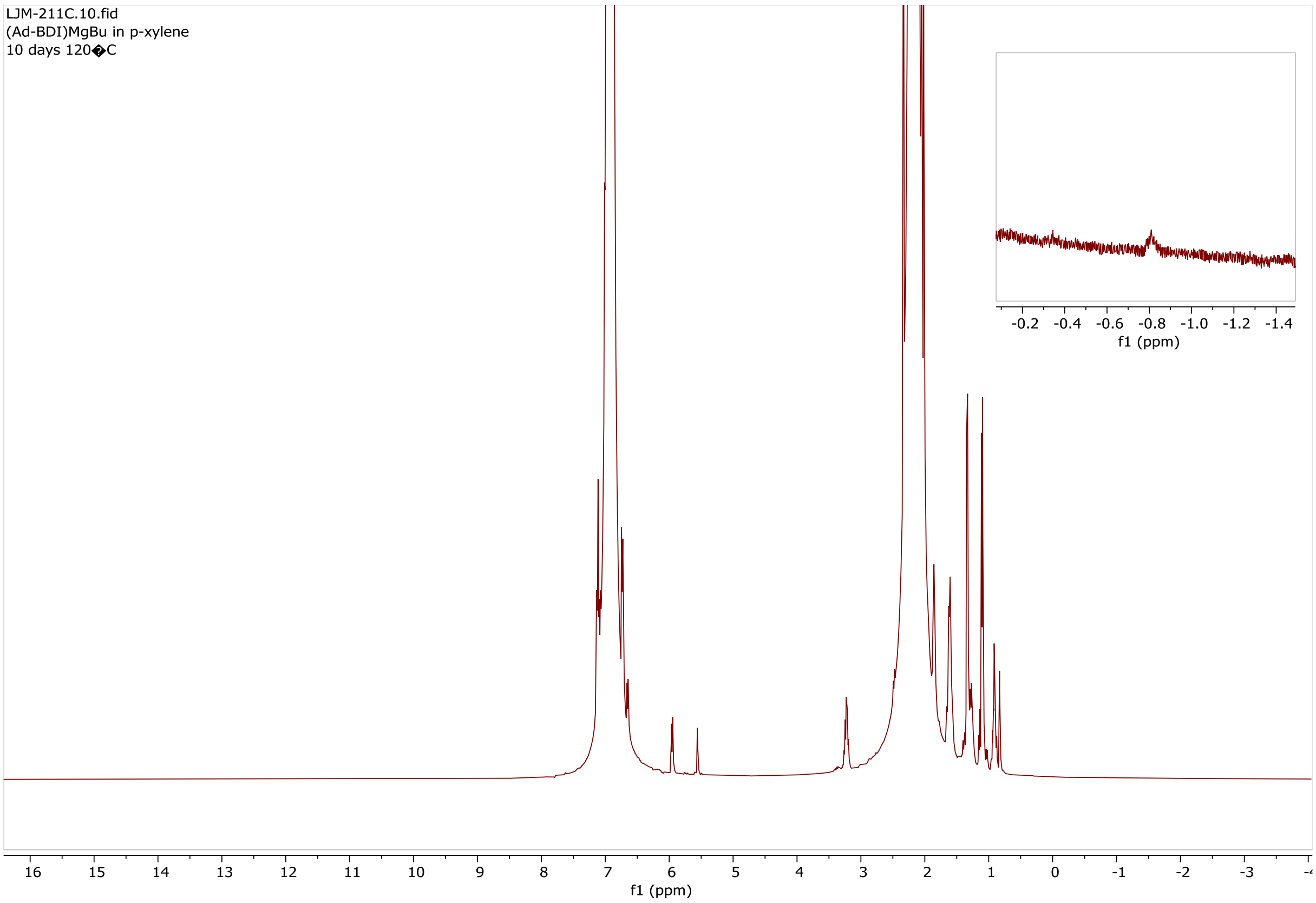

Figure S130: *in situ* ^{1}H NMR (400 MHz, *para*-xylene) after heating at 120°C for 10 days. Expansion shows near-quantitative consumption of butyl.

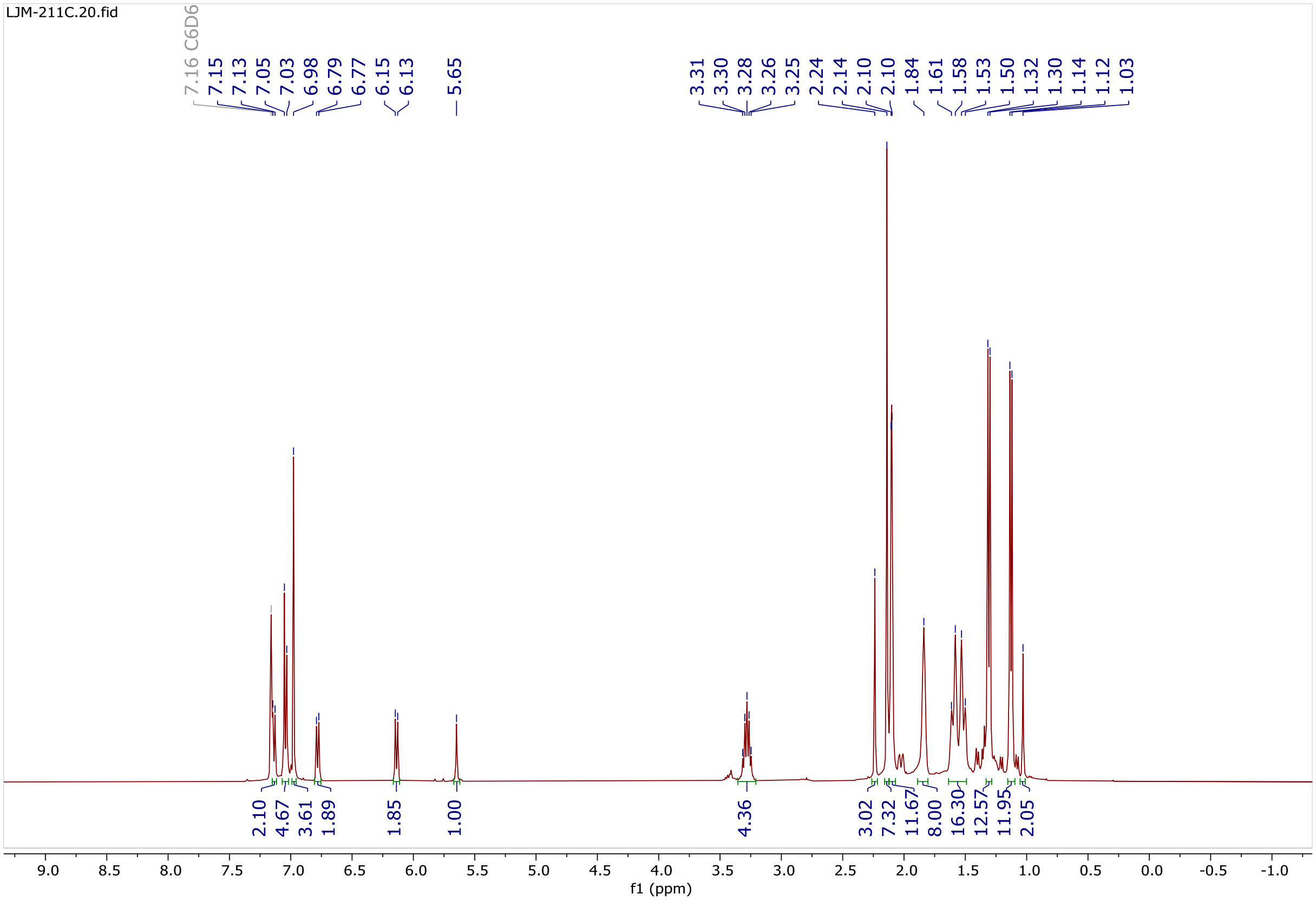

Figure S131: ${}^{1}H$ NMR spectrum (400 MHz, C_6D_6) of crude product after removal of volatiles.

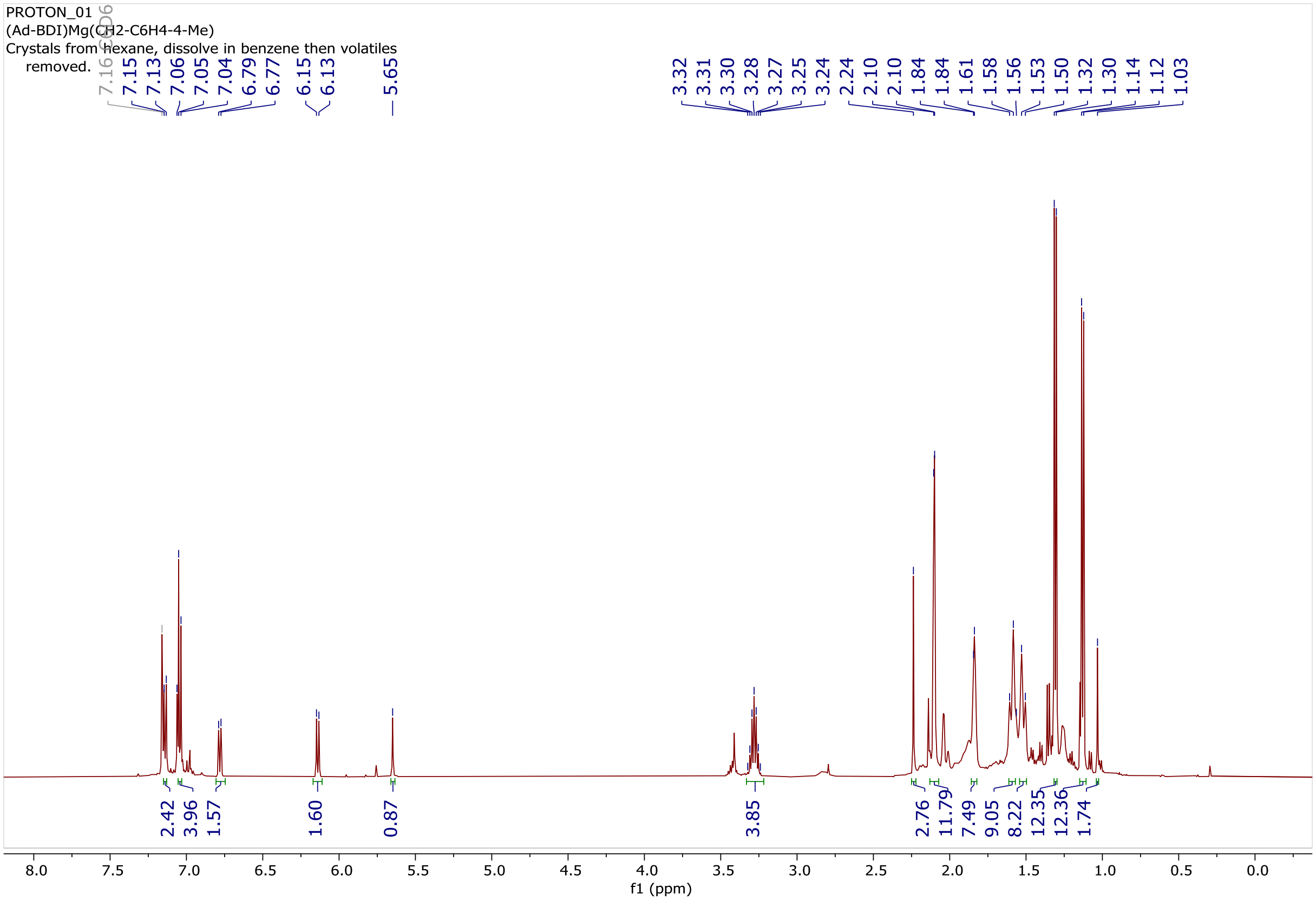

Figure S132: ${}^{1}H$ NMR (500 MHz, C_6D_6) of **8**.

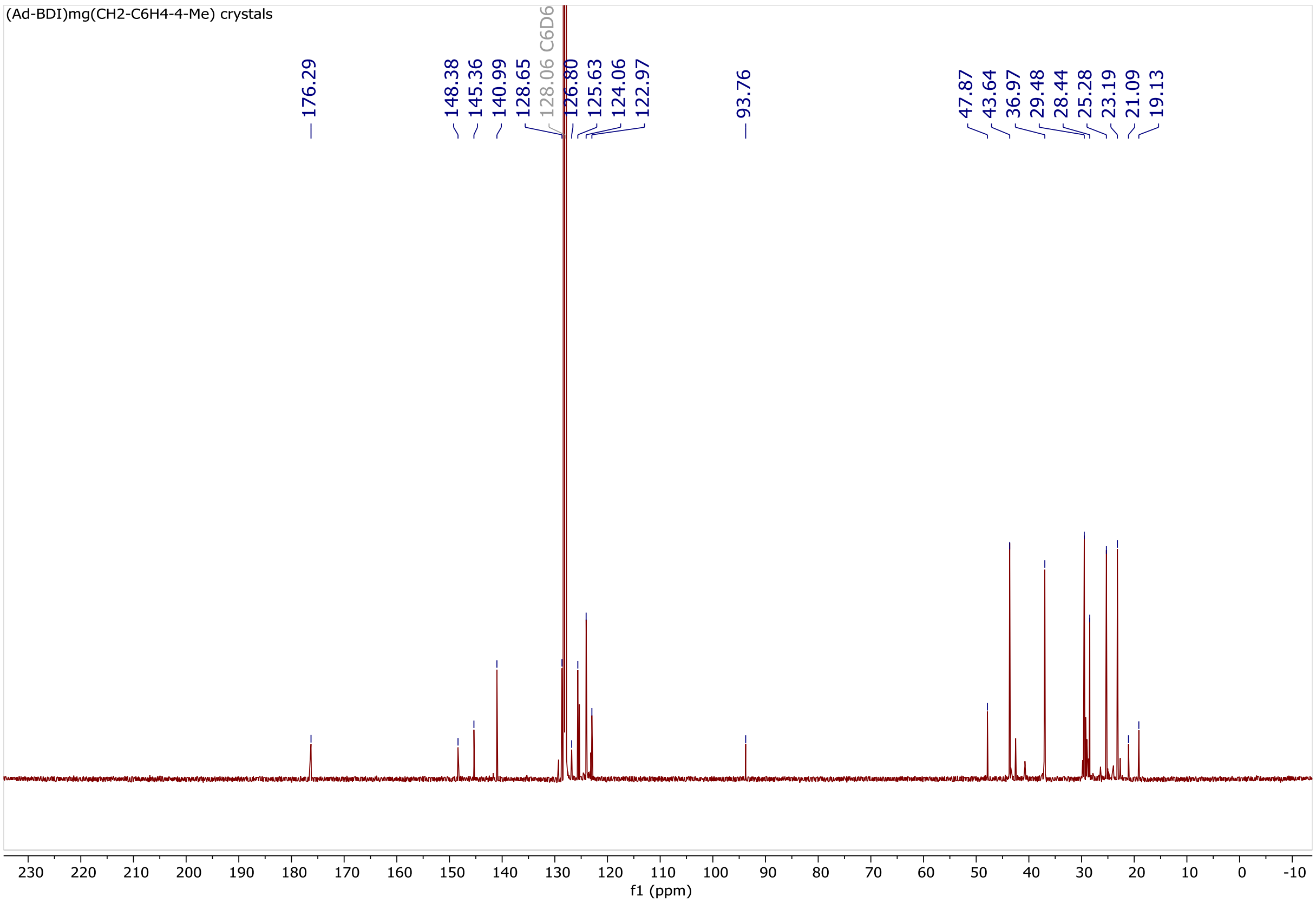

Figure S133: $^{13}C\{^{1}H\}$ NMR (126 MHz, C_6D_6) of **8**.

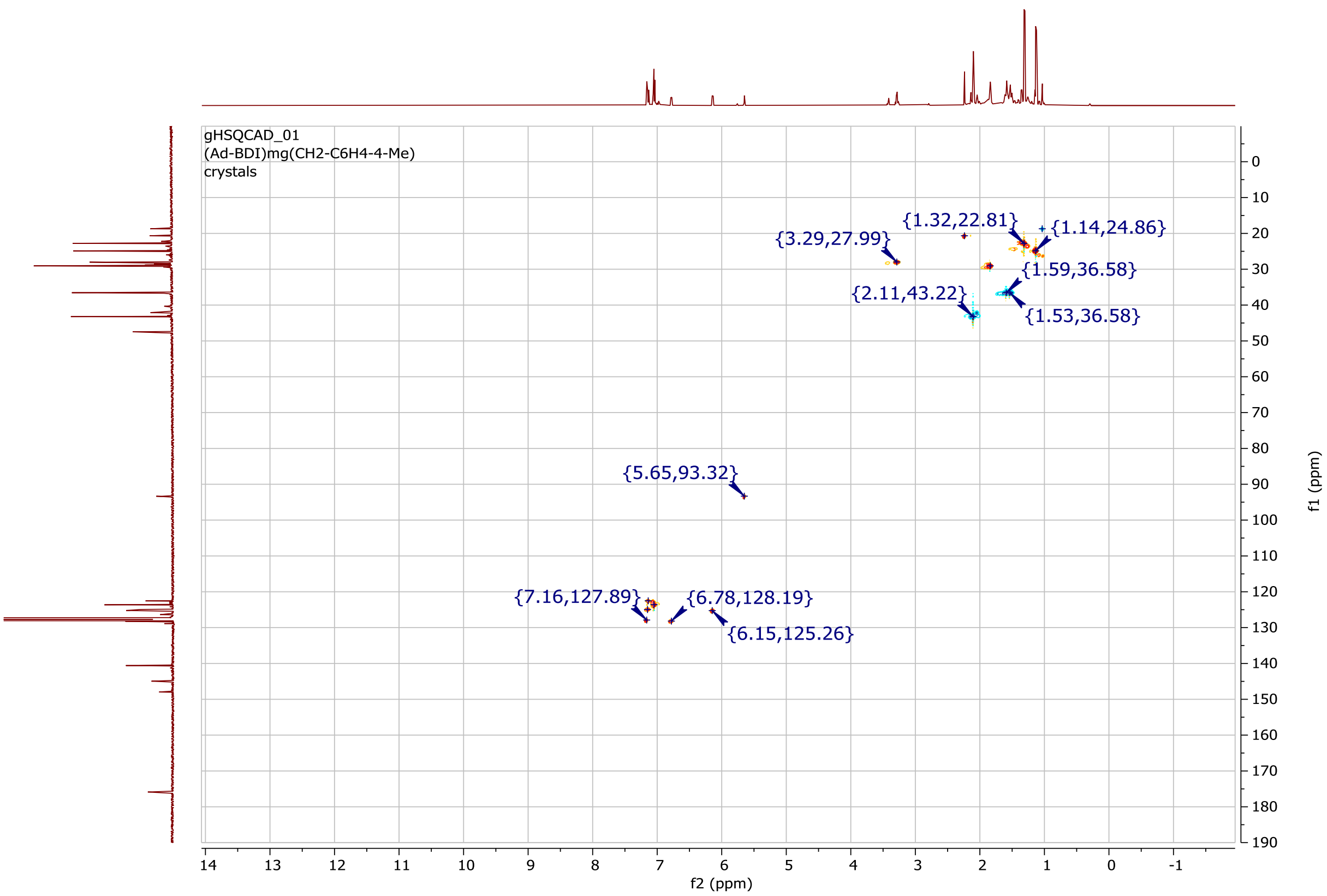

Figure S134: ^{13}C-^{1}H HSQC NMR (126 MHz, C_6D_6) of **8**.

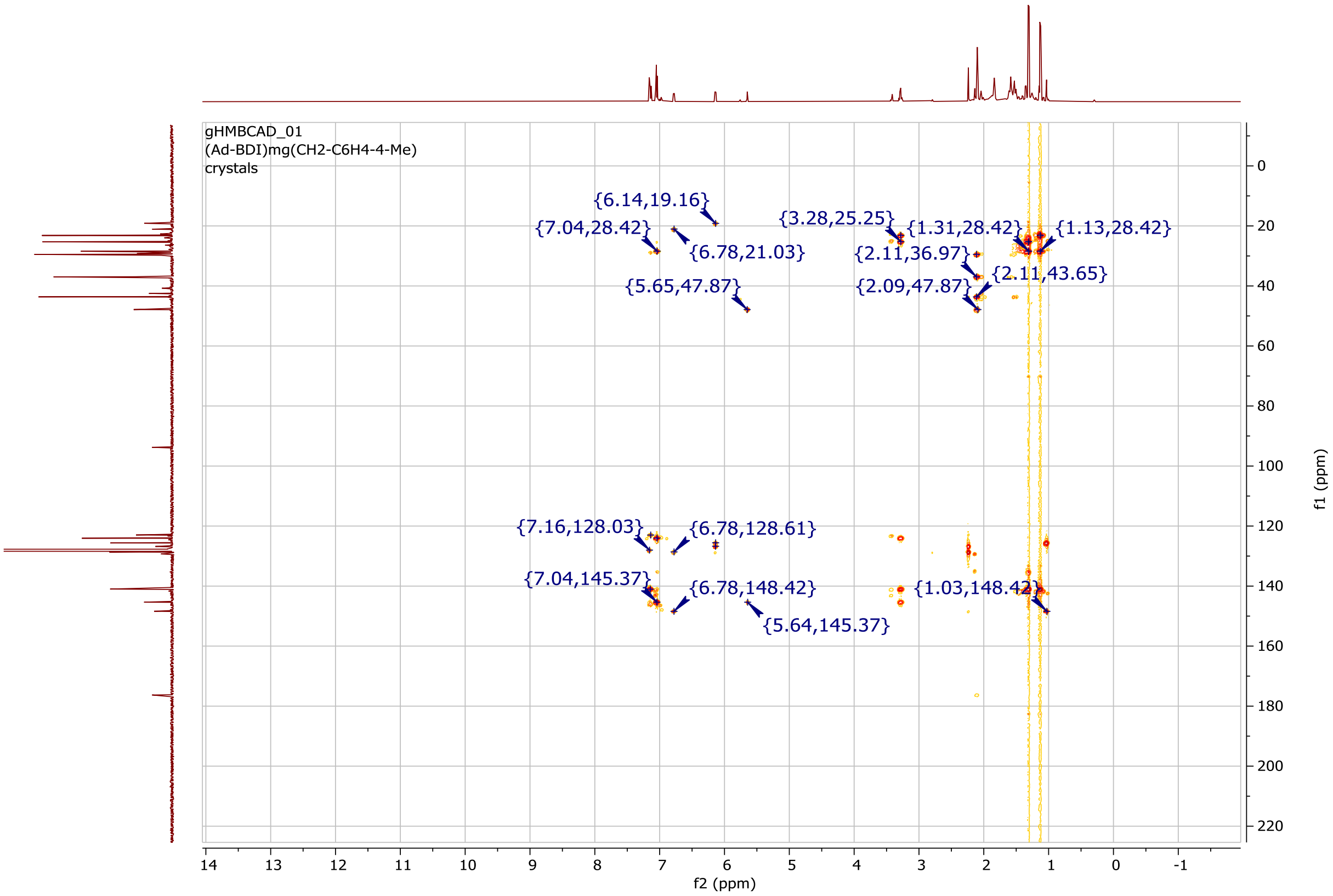

Figure S135: ^{13}C-^{1}H HMBC NMR (126 MHz, C_6D_6) of **8**.

2.16 Reaction of [(^{Ad}BDI)Mg^{n}Bu] (1^{Ad}) with 1,3-di(tert-butyl)benzene, synthesis and characterisation of [(^{Ad}BDI)Mg(C_6H_3-3,5-^{t}Bu$_2$)] (9)

A J. Young's tube was charged with 20 mg **1^{Ad}** (0.027 mmol) and 0.5 ml 1,3-di(*tert*-butyl)benzene. The sealed tube was heated at 120°C for 8 days, after which colourless crystals of **9** suitable for X-ray diffraction analysis had been deposited. Analysis of the reaction mixture by *in situ* ^{1}H NMR spectroscopy showed near-quantitative consumption of the starting complex. The crystals were isolated in 12 mg yield (51%) by decanting the supernatant, washing with three portions of *n*-pentane, and drying under vacuum.

Analysis calculated for $C_{61}H_{86}MgN_2$: C, 84.05; H, 9.94; N, 3.21%. Found: C, 83.50; H, 10.41; N, 3.17%

^{1}H NMR (500 MHz, C_6D_6) δ 7.29 – 7.21 (m, 3H, *para*-NAr + *para*-MgAr), 7.11 (d, *J* = 7.7 Hz, 4H, *meta*-NAr), 6.05 (d, *J* = 2.2 Hz, 2H, *ortho*-MgAr), 5.76 (s, 1H, H̲CC(Me)NAr), 3.45 (hept, *J* = 6.9 Hz, 4H, H̲C(CH$_3$)$_2$), 2.14 (d, *J* = 3.1 Hz, 12H, ^{Ad}CCH̲$_2$CH), 1.89 – 1.84 (m, 6H, ^{Ad}CH̲), 1.61 (d, *J* = 12.1 Hz, 6H, ^{Ad}CHCH̲$_2$CH), 1.53 (d, *J* = 12.3 Hz, 6H, ^{Ad}CHCH̲$_2$CH), 1.33 (d, *J* = 7.0 Hz, 12H, HC(CH̲$_3$)$_2$), 1.32 (s, 18H, C(CH̲$_3$)$_3$), 1.24 (d, *J* = 6.8 Hz, 12H, HC(CH̲$_3$)$_2$).

^{13}C NMR (126 MHz, C_6D_6) δ 176.58 (C̲=N), 157.22 (*ipso*-MgC̲$_6$H$_3$(^{t}Bu)$_2$), 146.40 (*meta*-MgC̲$_6$H$_3$(^{t}Bu)$_2$), 145.10 (*ipso*-NC$_6$H$_3$-*i*Pr$_2$), 141.89 (*ortho*-NC$_6$H$_3$-*i*Pr$_2$), 135.91 (*ortho*-MgC̲$_6$H$_3$(^{t}Bu)$_2$), 126.18 (*para*-NC$_6$H$_3$-*i*Pr$_2$), 124.12 (*meta*-NC$_6$H$_3$-*i*Pr$_2$), 118.74 (*para*-MgC̲$_6$H$_3$(^{t}Bu)$_2$), 93.90 (HC̲C(Ndipp)), 47.90 (NC^{Ad}C̲), 43.69 (^{Ad}CC̲H$_2$CH), 36.97 (^{Ad}CHC̲H$_2$CH), 34.89 (C̲(CH$_3$)$_3$), 32.13 (C(C̲H$_3$)$_3$), 29.53 (^{Ad}C̲H), 28.53 (HC̲(CH$_3$)$_2$), 26.10 (HC(C̲H$_3$)$_2$), 23.09 (HC(C̲H$_3$)$_2$).

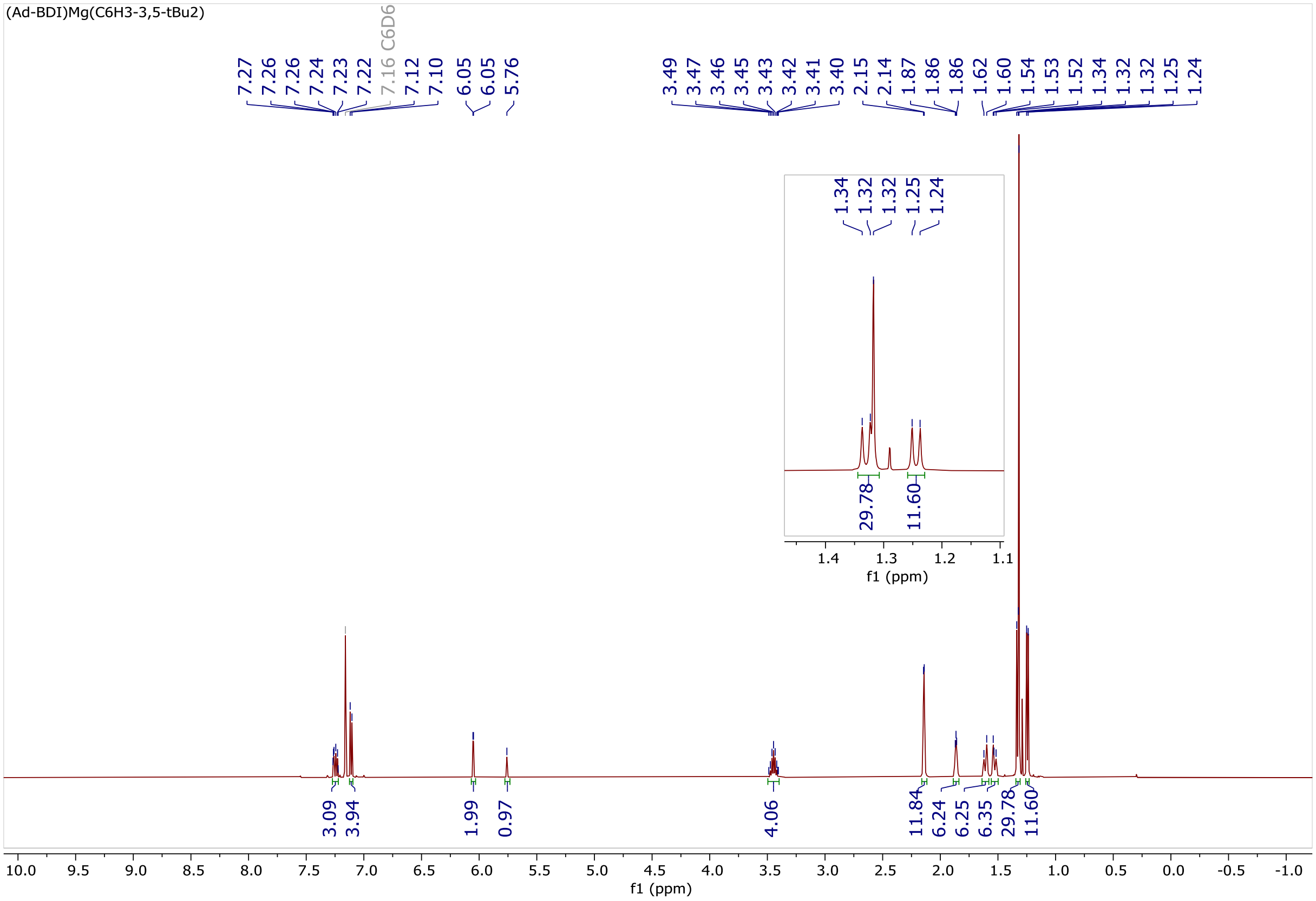

Figure S136: ^{1}H NMR (500 MHz, C_6D_6).

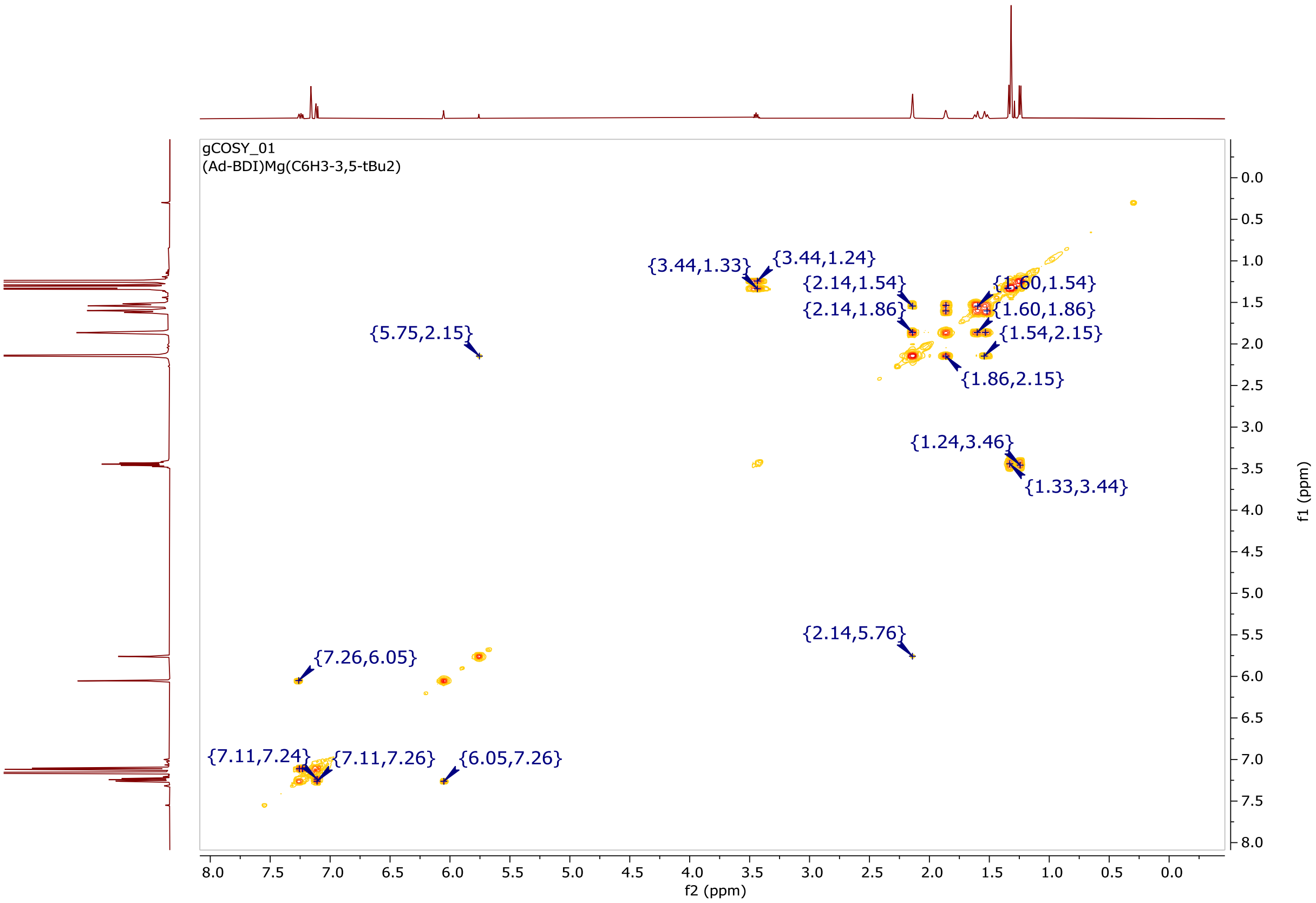

Figure S137: ^{1}H-^{1}H COSY.

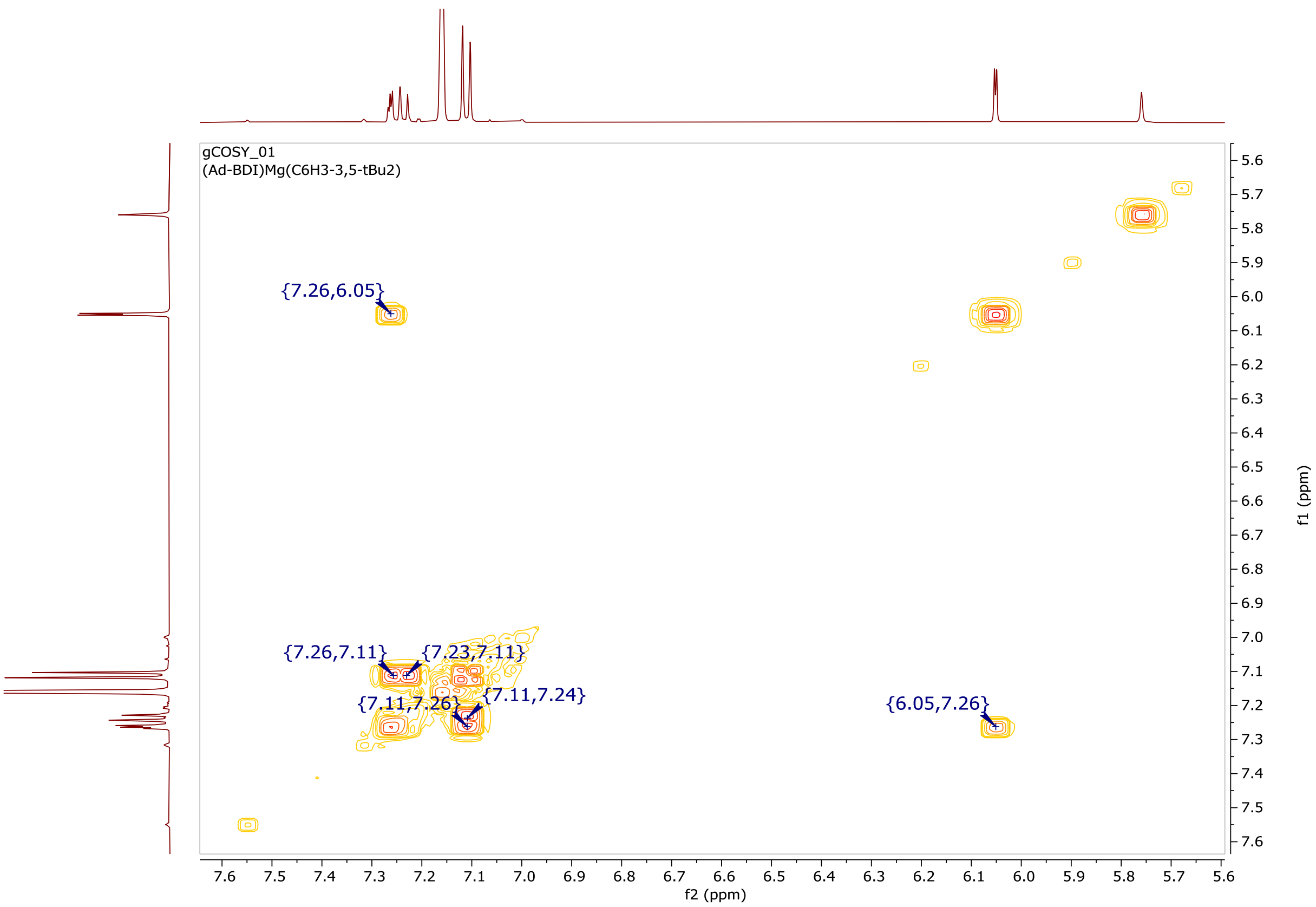

Figure S138: Expanded ^{1}H-^{1}H COSY.

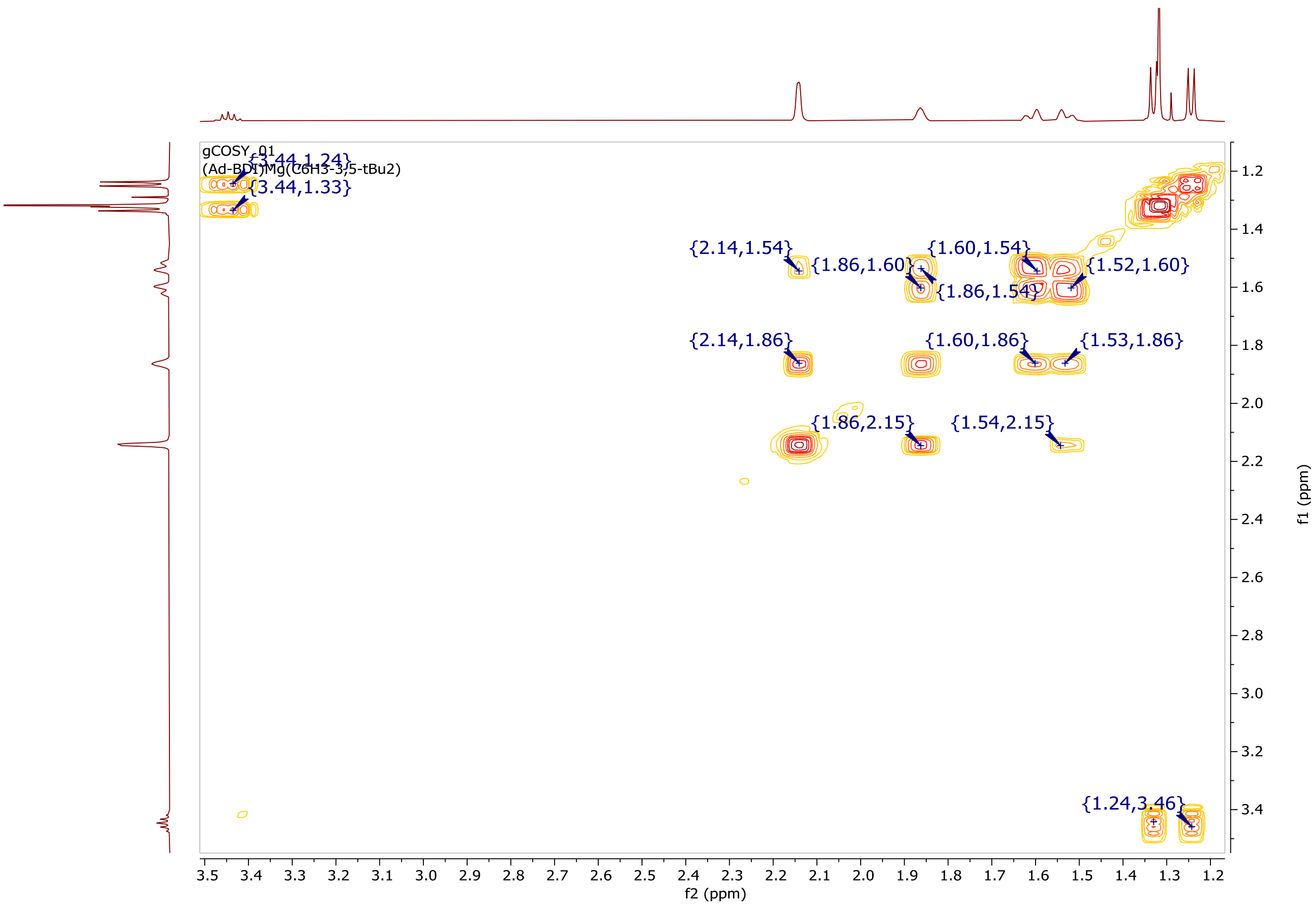

Figure S139: Expanded ^{1}H-^{1}H COSY.

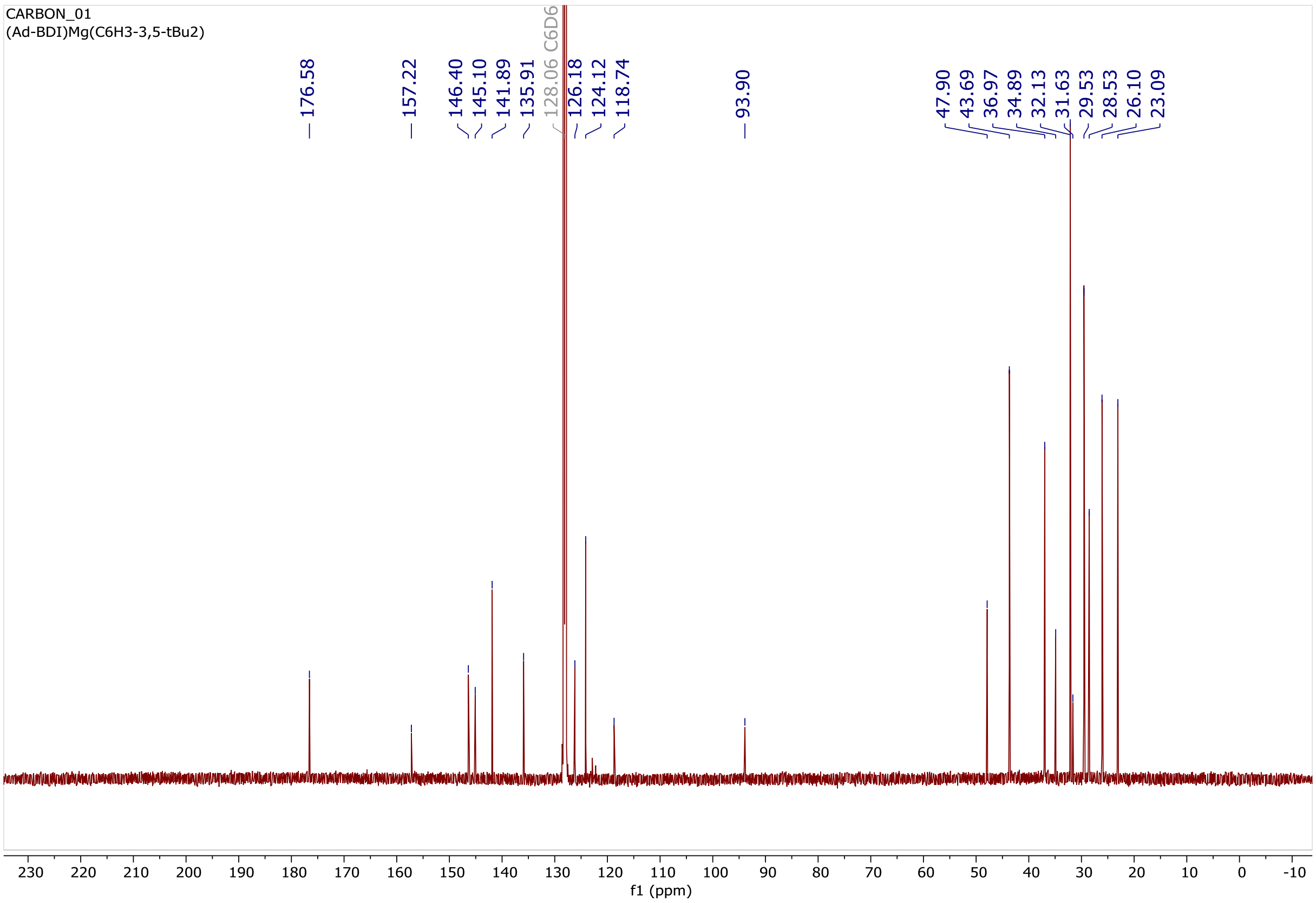

Figure S140: $^{13}C\{^{1}H\}$ NMR (126 MHz, C_6D_6).

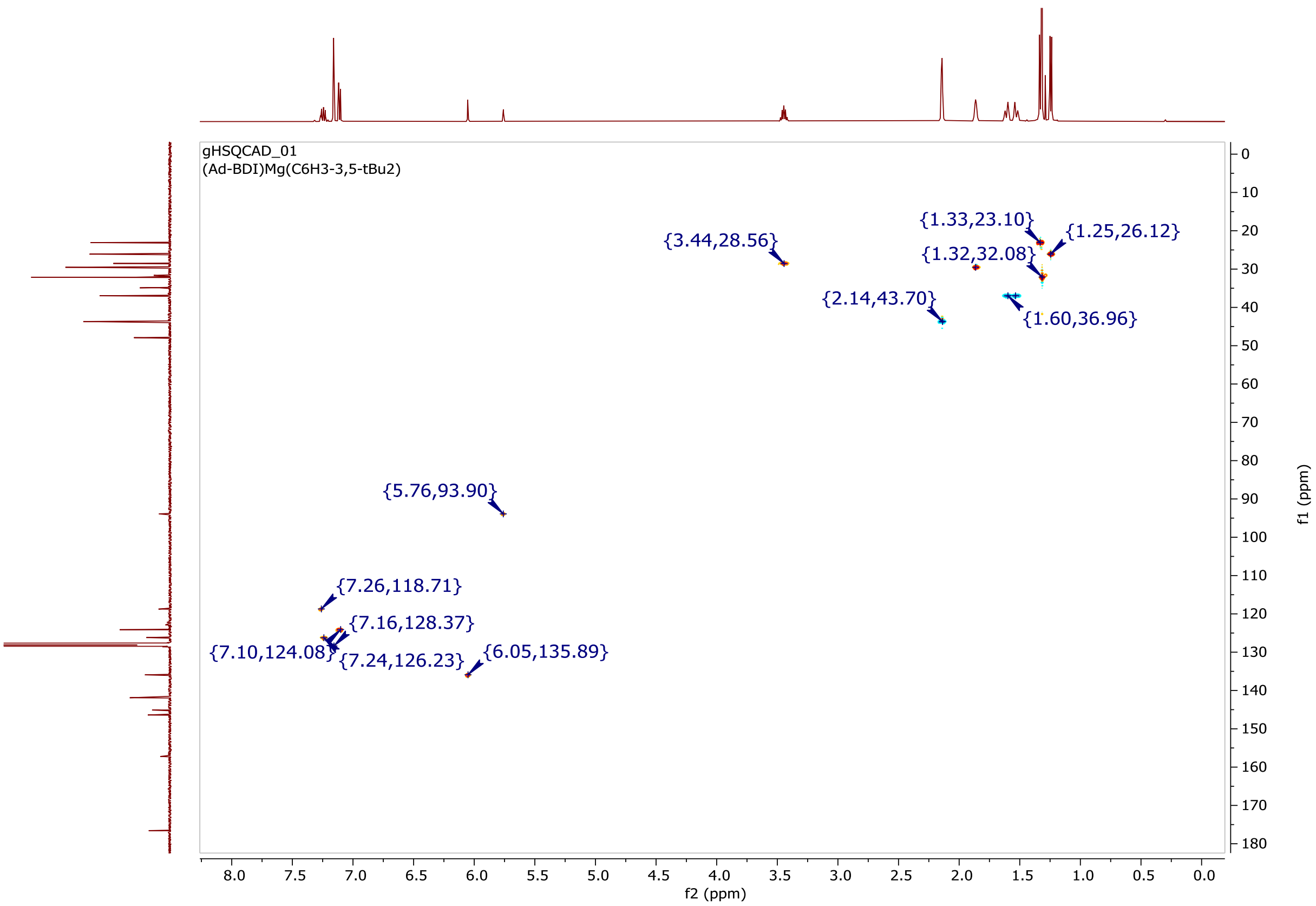

Figure S141: ^{13}C-^{1}H HSQC NMR (126 MHz, C_6D_6).

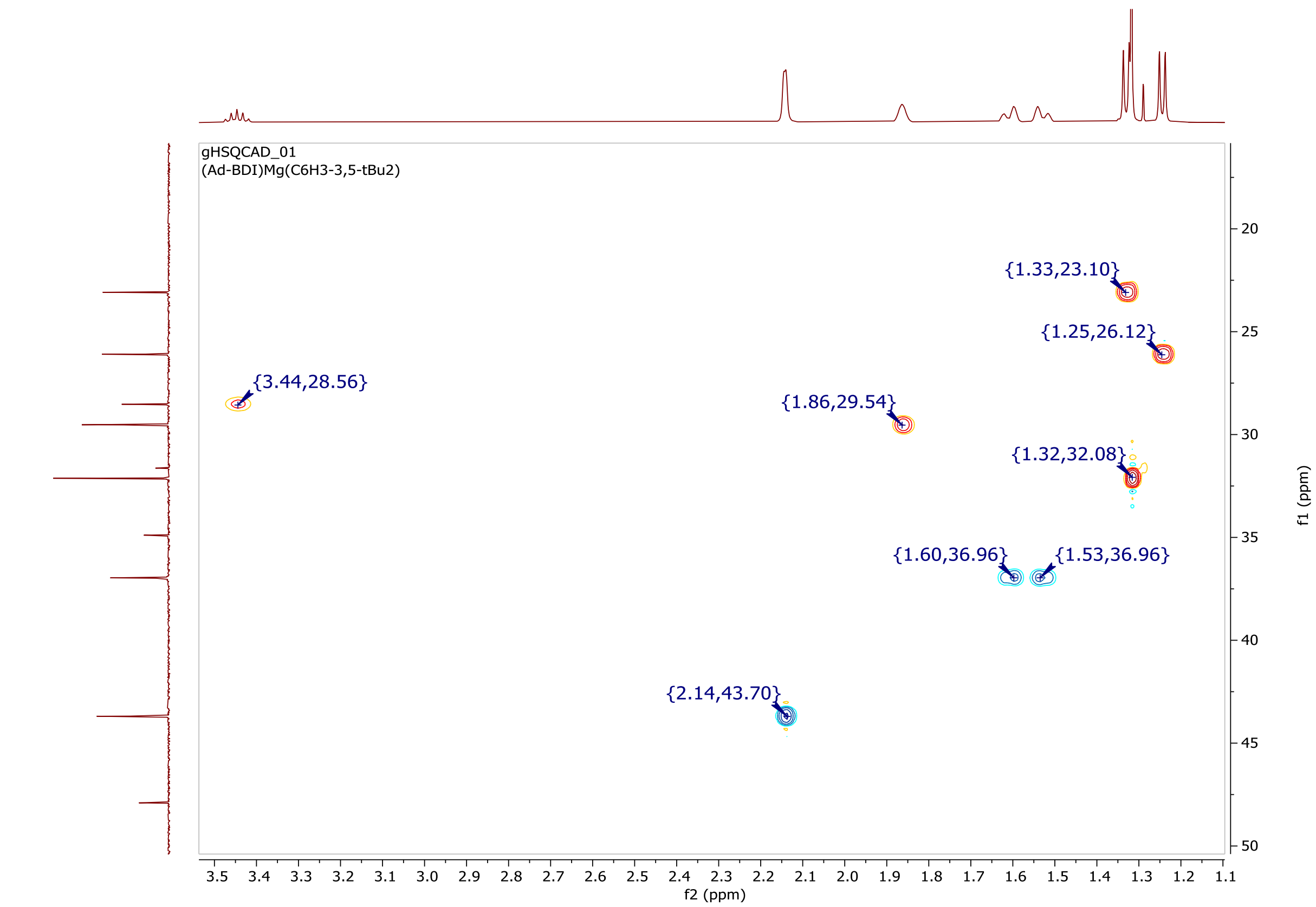

Figure S142: Expanded ^{13}C-^{1}H HSQC NMR (126 MHz, C_6D_6).

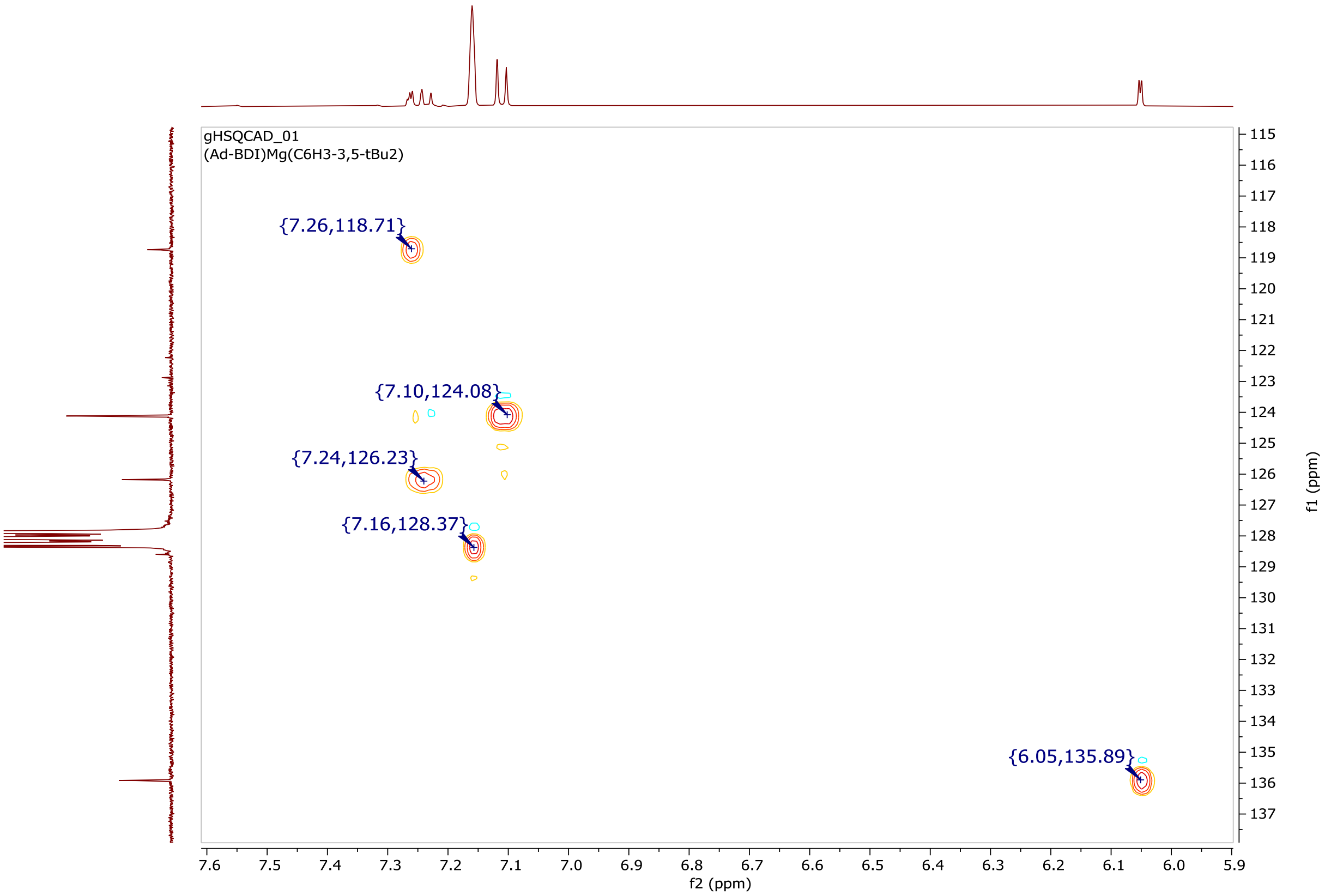

Figure S143: Expanded ^{13}C-^{1}H HSQC NMR (126 MHz, C_6D_6).

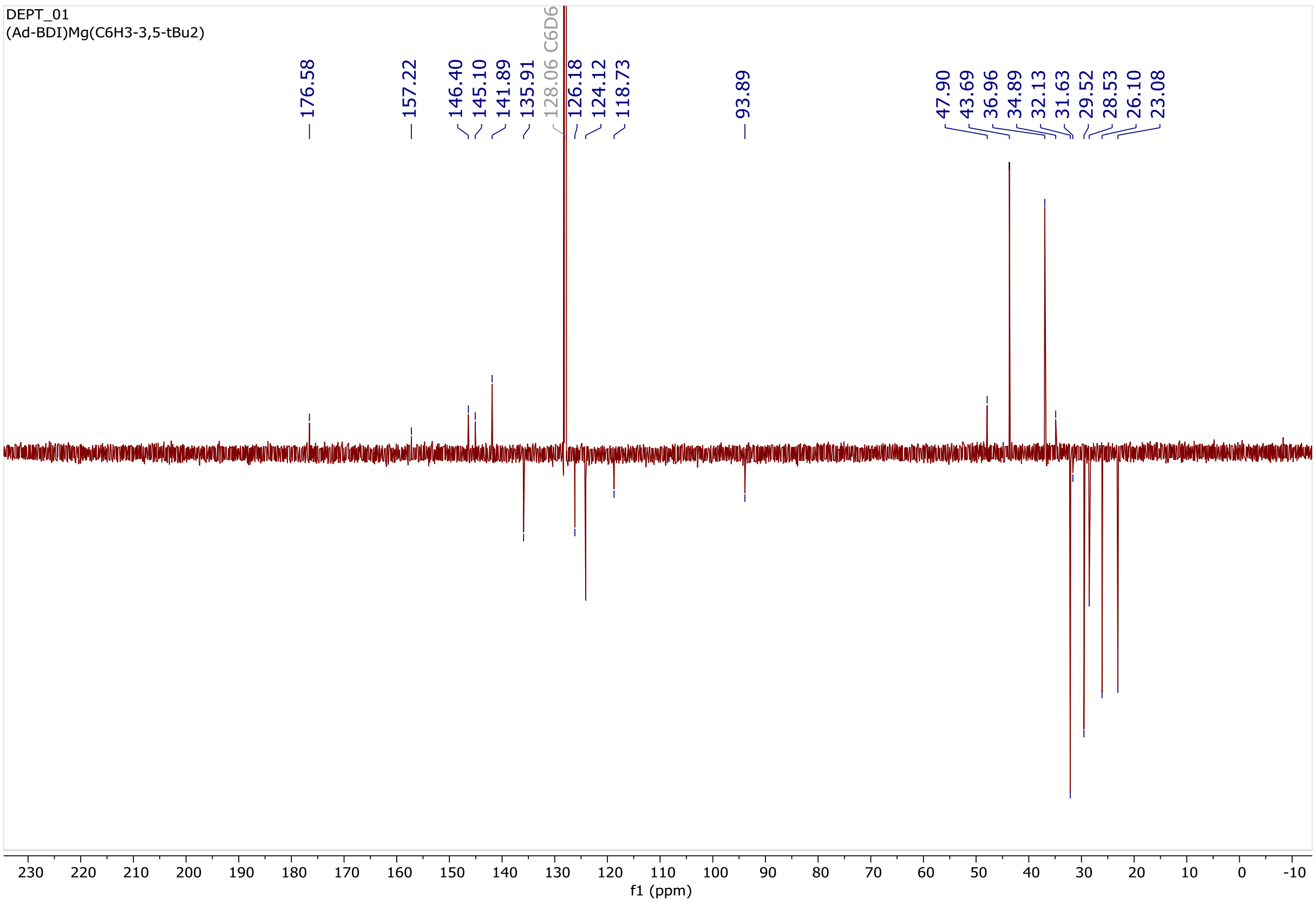

Figure S144: ^{13}C DEPT NMR (126 MHz, C_6D_6).

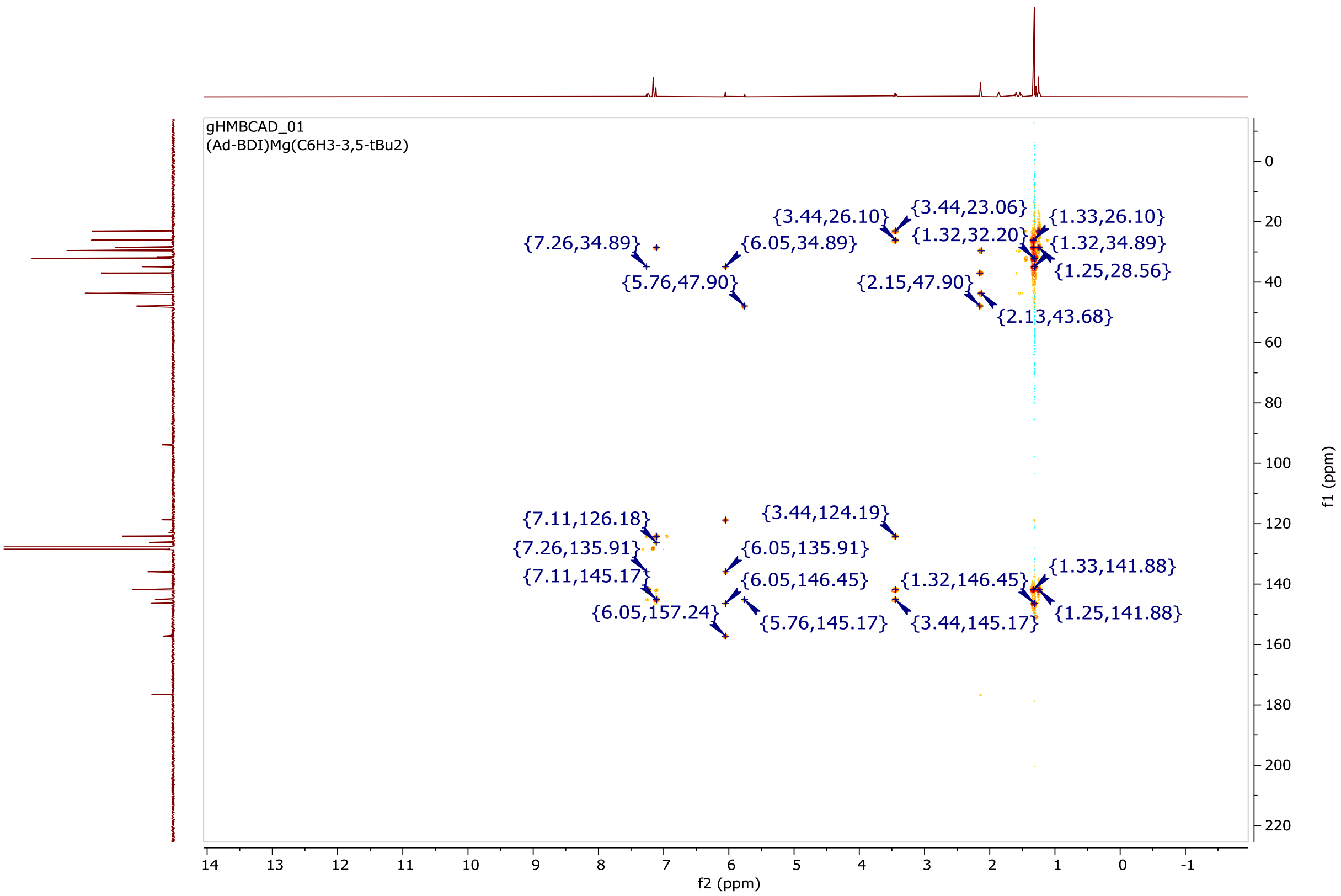

Figure S145: ^{13}C-^{1}H HMBC NMR (126 MHz, C_6D_6).

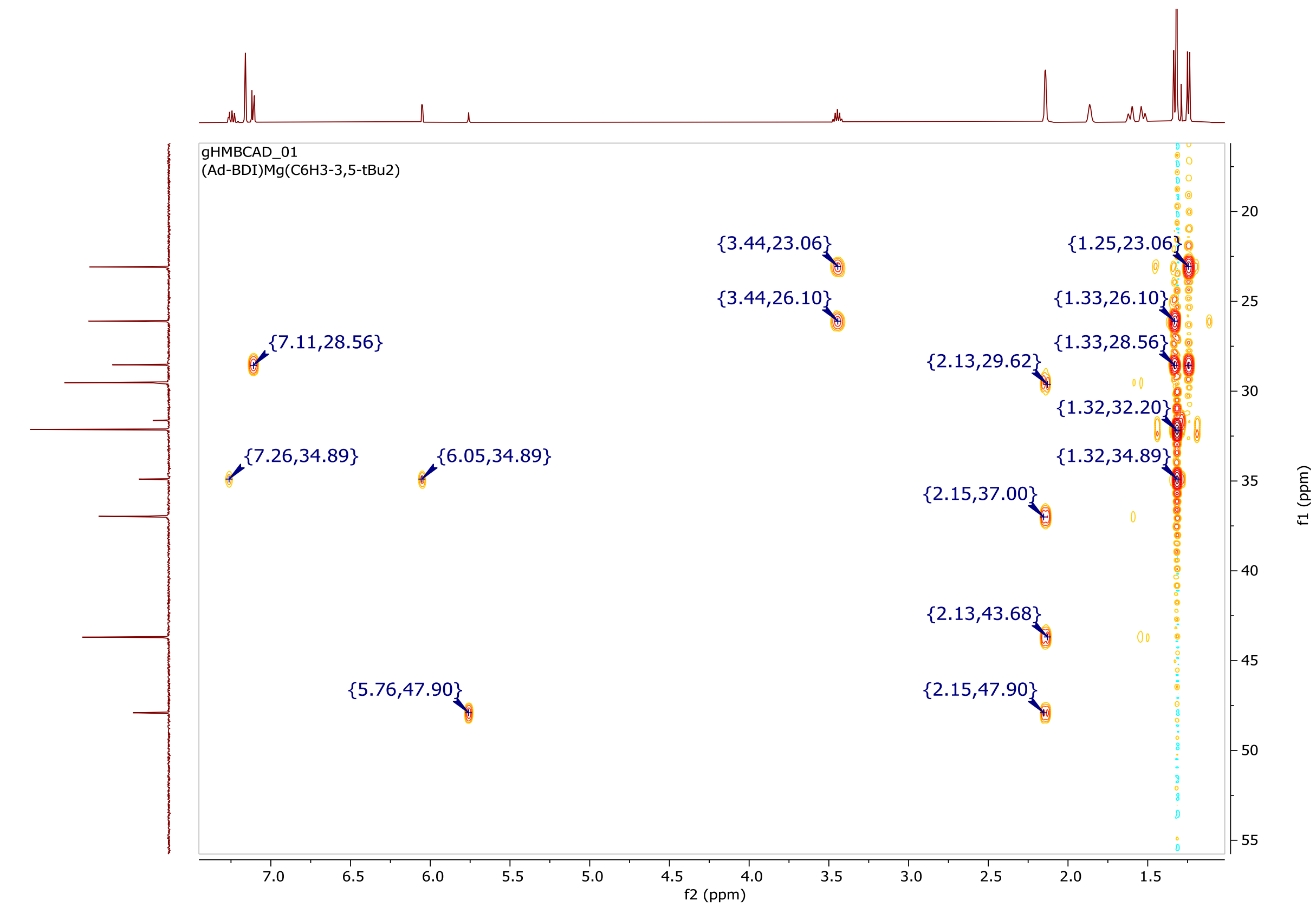

Figure S146: Expanded ^{13}C-^{1}H HMBC NMR (126 MHz, C_6D_6).

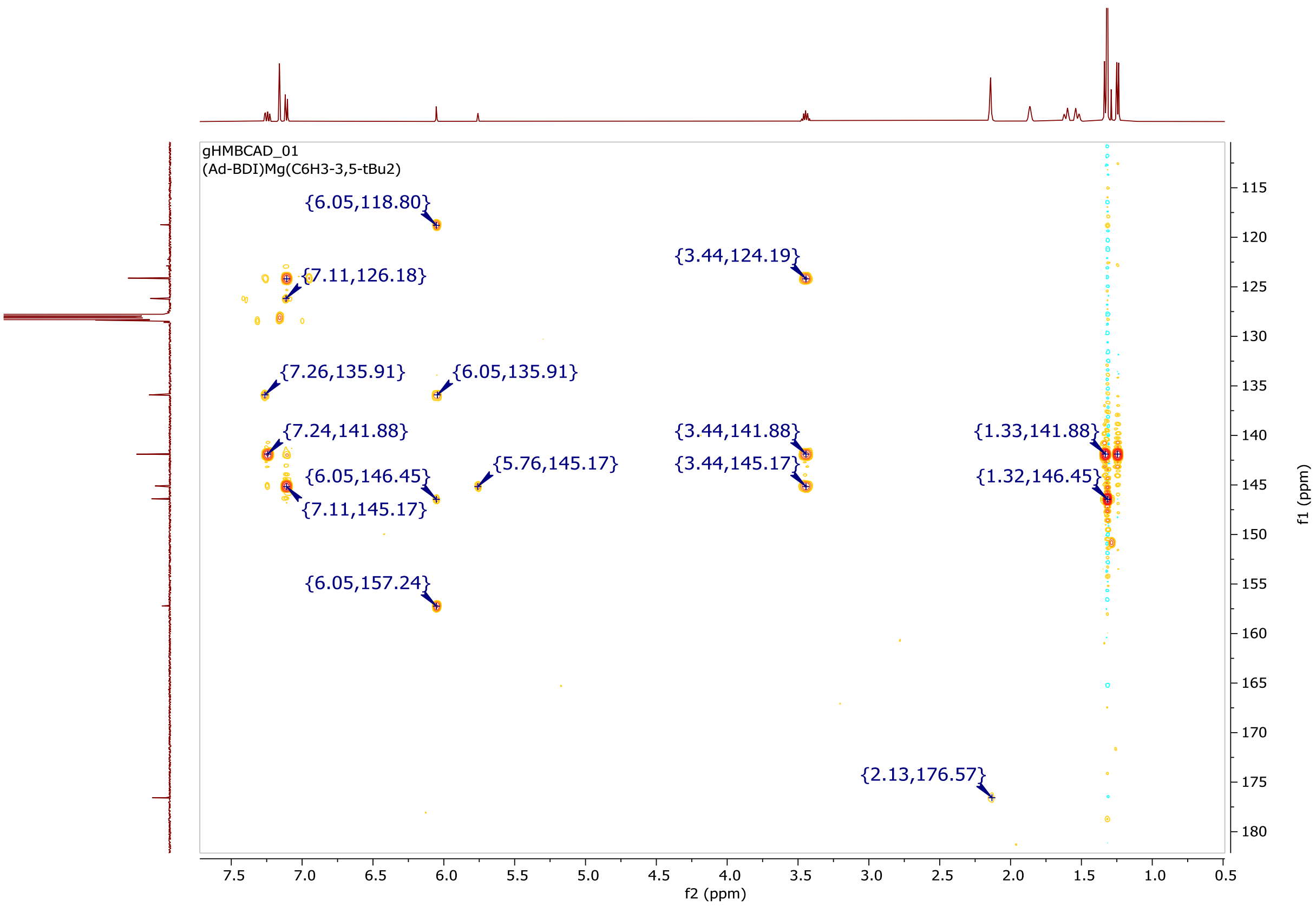

Figure S147: Expanded ^{13}C-^{1}H HMBC NMR (126 MHz, C_6D_6).

2.17 Reaction of [(^{Me}BDI)Mg^{n}Bu]$_2$ [1^{Me}]$_2$ with anisole, synthesis and characterisation of [(^{Me}BDI)Mg(κ^2*C,O*-C_6H_4-2-OMe)(thf)] 10^{Me}·thf

The reaction was repeated using 82 mg **[1^{Me}]$_2$** (0.082 mmol) in 1 ml anisole. After heating the reaction mixture to 100°C for 24 hours, volatiles were removed under vacuum and the crude product was recrystallised and extracted into an *n*-hexane/THF solvent mixture. Storing this solution at -30°C overnight provided colourless plate-like single crystals of **10^{Me}·thf** suitable for X-ray diffraction analysis, which were isolated in 36 mg yield, 35%.

Analysis calculated for $C_{41}H_{56}N_2O_2Mg$: C, 77.34; H, 9.09; N, 4.51%. Found: C, 76.70; H, 9.25; N, 4.53%.

^{1}H NMR (500 MHz, c_6d_6) δ 7.22 (s, 6H, NC_6*H_3*-*i*Pr$_2$), 7.11 (td, *J* = 7.5, 1.8 Hz, 1H, *4*-C_6*H_4*-2-OMe), 6.89 (t, *J* = 6.8 Hz, 1H, *5*-C_6*H_4*-2-OMe), 6.67 (d, br, *J* = 3.7 Hz, 1H, *3*-C_6*H_4*-2-OMe), 6.46 (d, *J* = 7.9 Hz, 1H, 6-C_6*H_4*-2-OMe), 4.88 (s, 1H, *H*CCNAr), 3.60 (m, br, 4H, ^{THF}OC*H_2*), 3.32 (hept, *J* = 7.0 Hz, 4H, *H*C(CH_3)$_2$), 2.78 (s, br, 3H, ^{anisyl}OC*H_3*), 1.73 (s, 6H, *H_3*CCN), 1.26 (d, m, br, *J* = 7.0 Hz, 16H, HC(C*H_3*)$_2$ + ^{THF}OCH_2C*H_2*), 1.13 (d, *J* = 6.9 Hz, 12H, HC(C*H_3*)$_2$).

^{13}C NMR (126 MHz, C_6D_6) δ 168.09 (*C*=N), 167.81 (*2*-*C*$_6$H$_4$-2-OMe), 152.80 (*1*-*C*$_6$H$_4$-2-OMe), 147.09 (*ipso*-*C*$_6$H$_3$-2,6-*i*Pr$_2$), 142.75 (*ortho*-*C*$_6$H$_3$-2,6-*i*Pr$_2$), 141.34 (*3*-*C*$_6$H$_4$-2-OMe), 126.45 (*4*-*C*$_6$H$_4$-2-OMe), 125.04 (*para*-*C*$_6$H$_3$-2,6-*i*Pr$_2$), 123.84 (*meta*-*C*$_6$H$_3$-2,6-*i*Pr$_2$), 121.32 (*5*-*C*$_6$H$_4$-2-OMe), 105.49 (*6*-*C*$_6$H$_4$-2-OMe), 94.66 (H*C*CNAr), 69.41(^{THF}O*C*H$_2$), 53.15 (^{anisyl}O*C*H$_3$), 28.36 (H*C*(CH$_3$)$_2$), 25.38 (^{THF}OCH$_2$*C*H$_2$), 24.82 (HC(*C*H$_3$)$_2$), 24.51 (HC(*C*H$_3$)$_2$), 24.38 (H$_3$*C*CN).

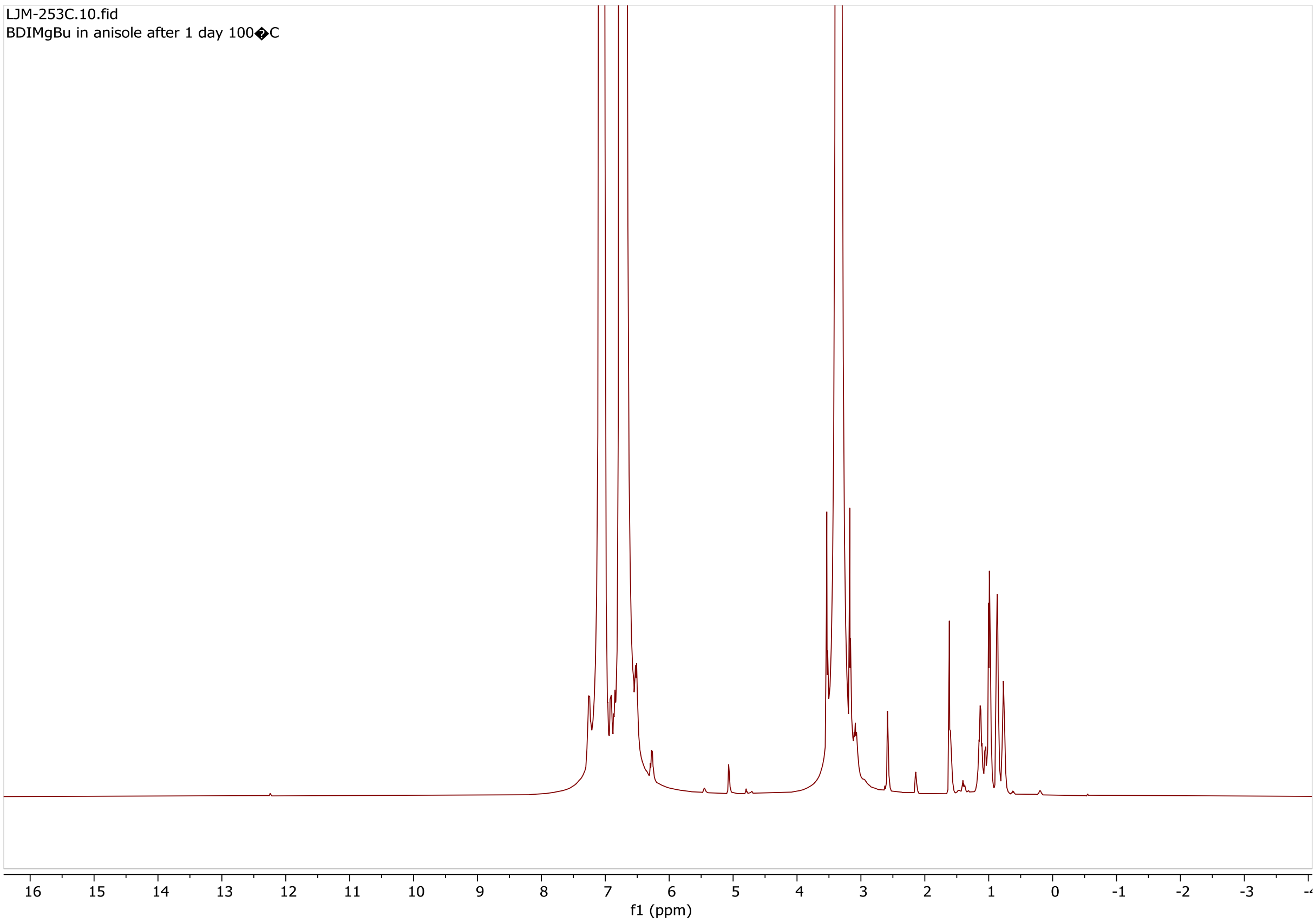

Figure S148: ^{1}H NMR (400 MHz, anisole) of the crude reaction mixture after 24 hours at 100°C.

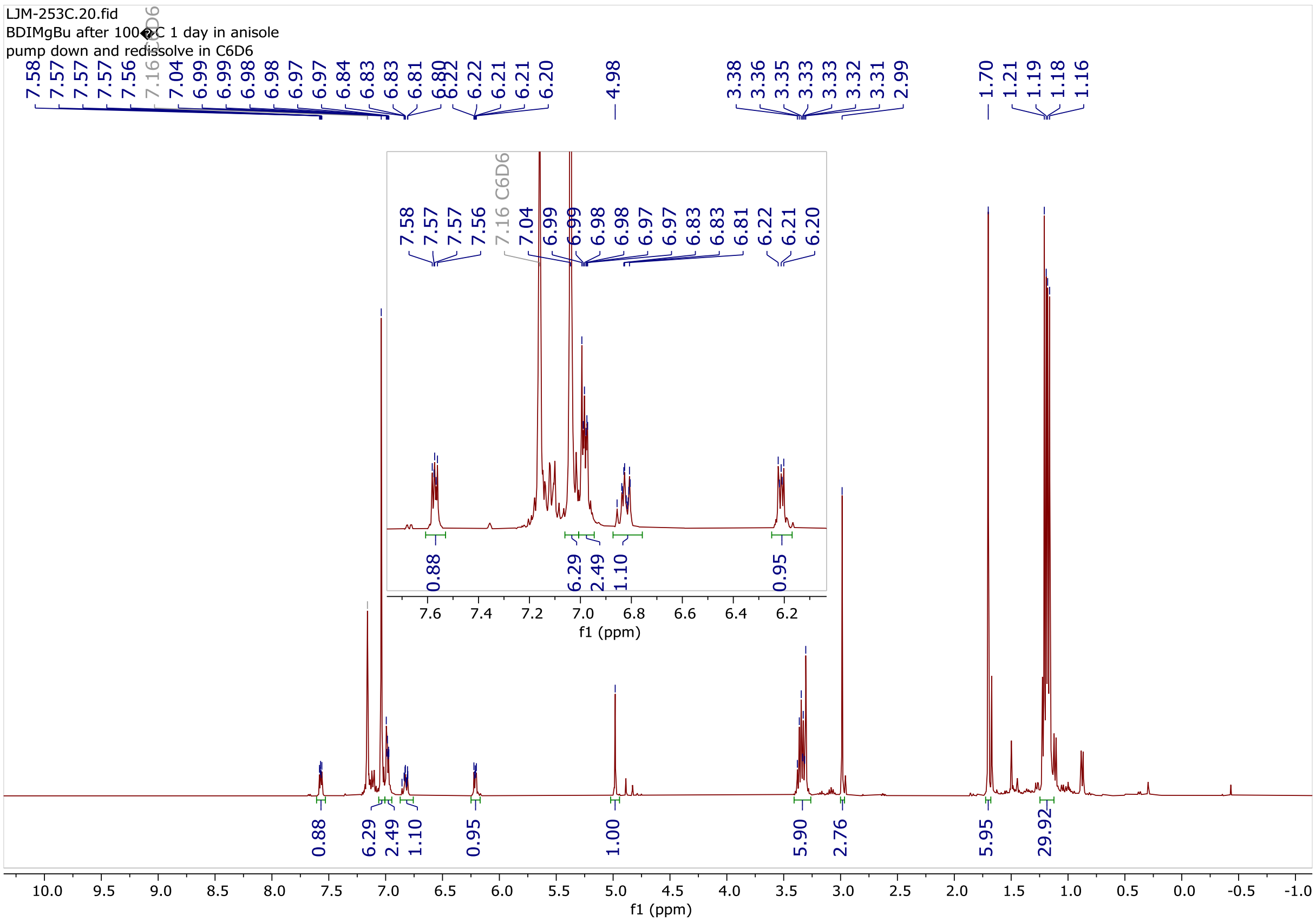

Figure S149: ^{1}H NMR (400 MHz, C_6D_6) of the crude product after removal of volatiles showing formation of THF-free anisyl derivative **10^{Me}**.

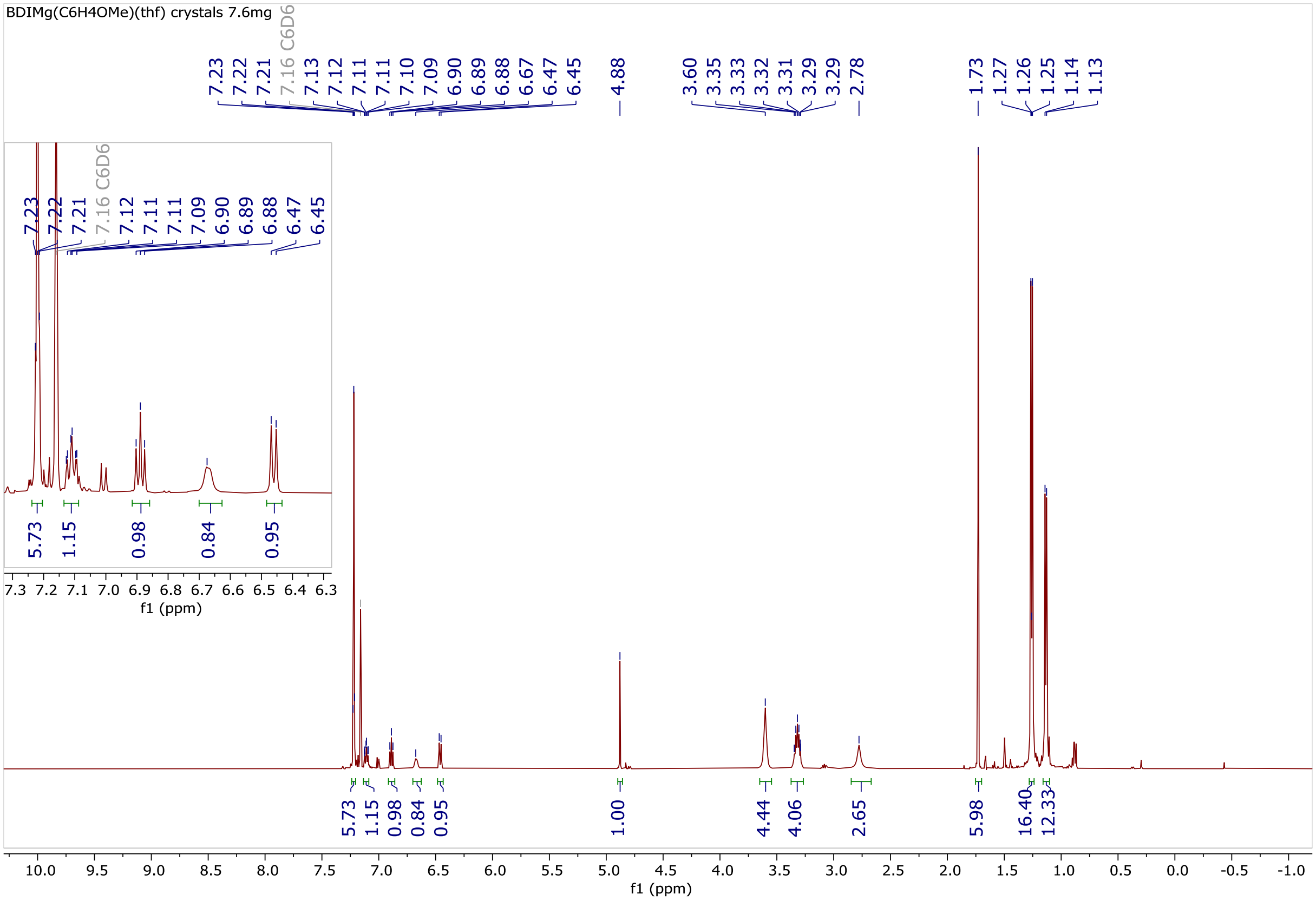

Figure S150: ^{1}H NMR (500 MHz, C_6D_6) of isolated **10^{Me}·thf** after recrystallisation from *n*-hexane/THF.

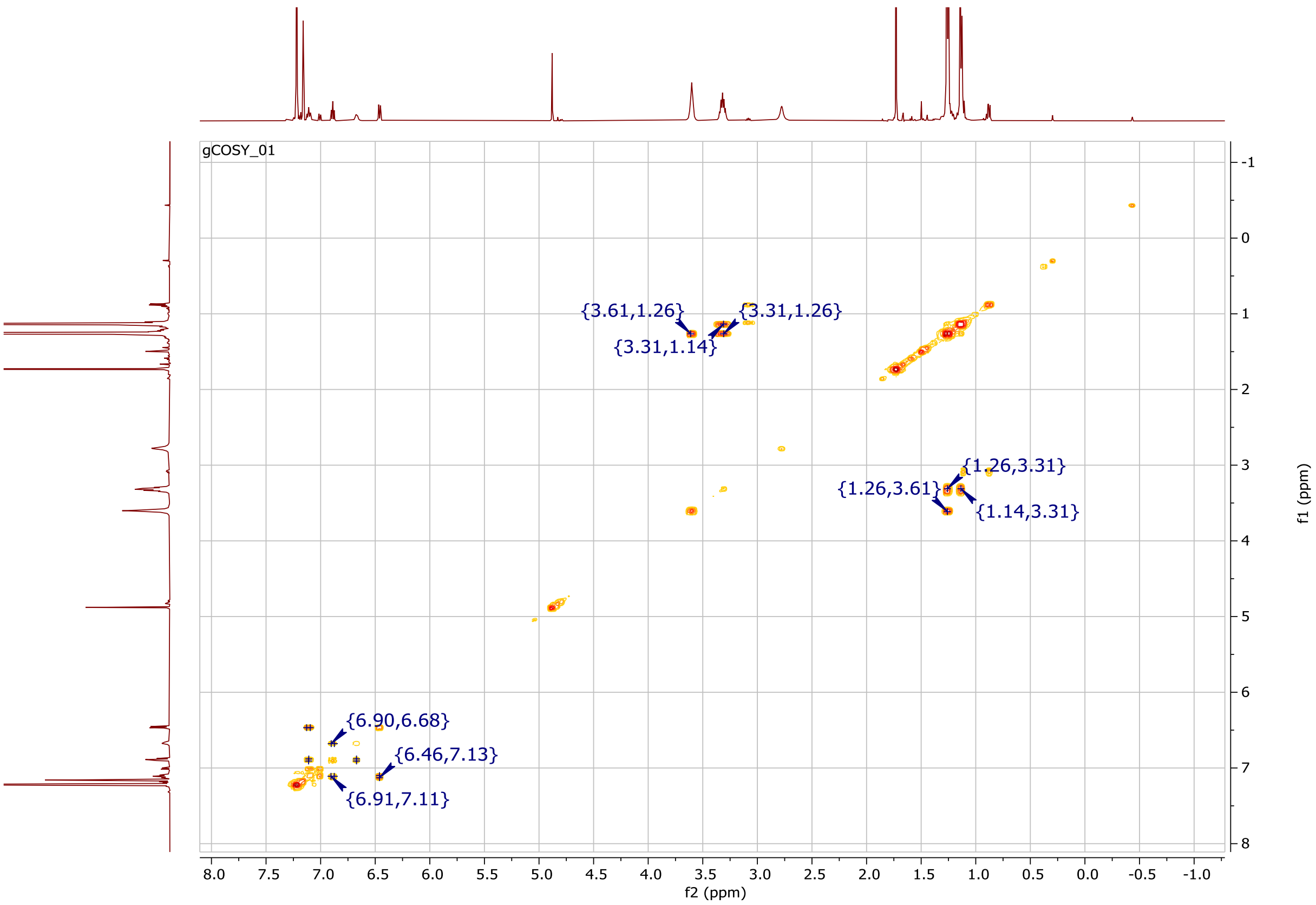

Figure S151: ^{1}H-^{1}H COSY NMR (500 MHz, C_6D_6) of isolated **10^{Me}·thf** after recrystallisation from *n*-hexane/THF.

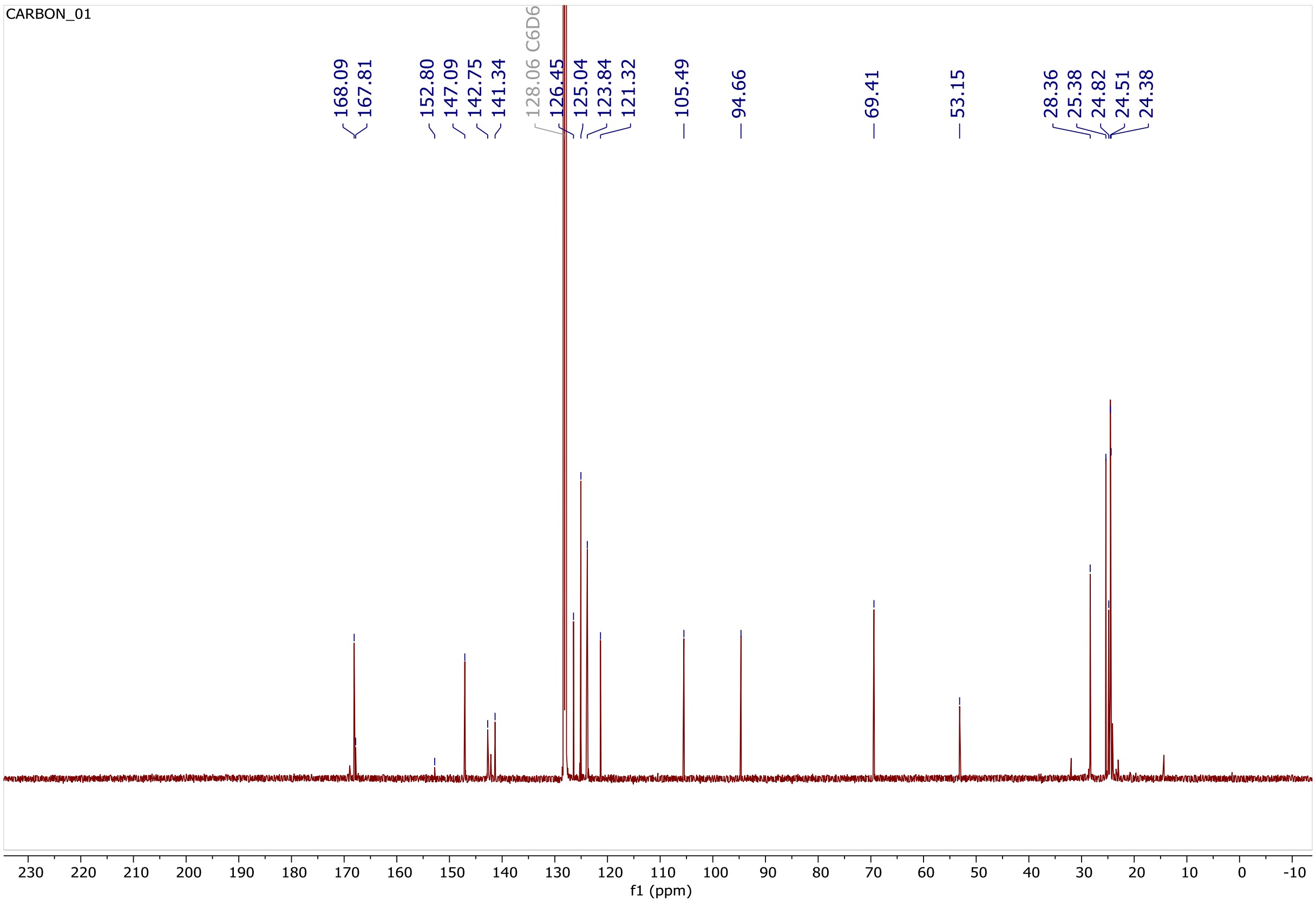

Figure S152: $^{13}C\{^1H\}$ NMR (126 MHz, C_6D_6) of isolated **10^{Me}·thf** after recrystallisation from *n*-hexane/THF.

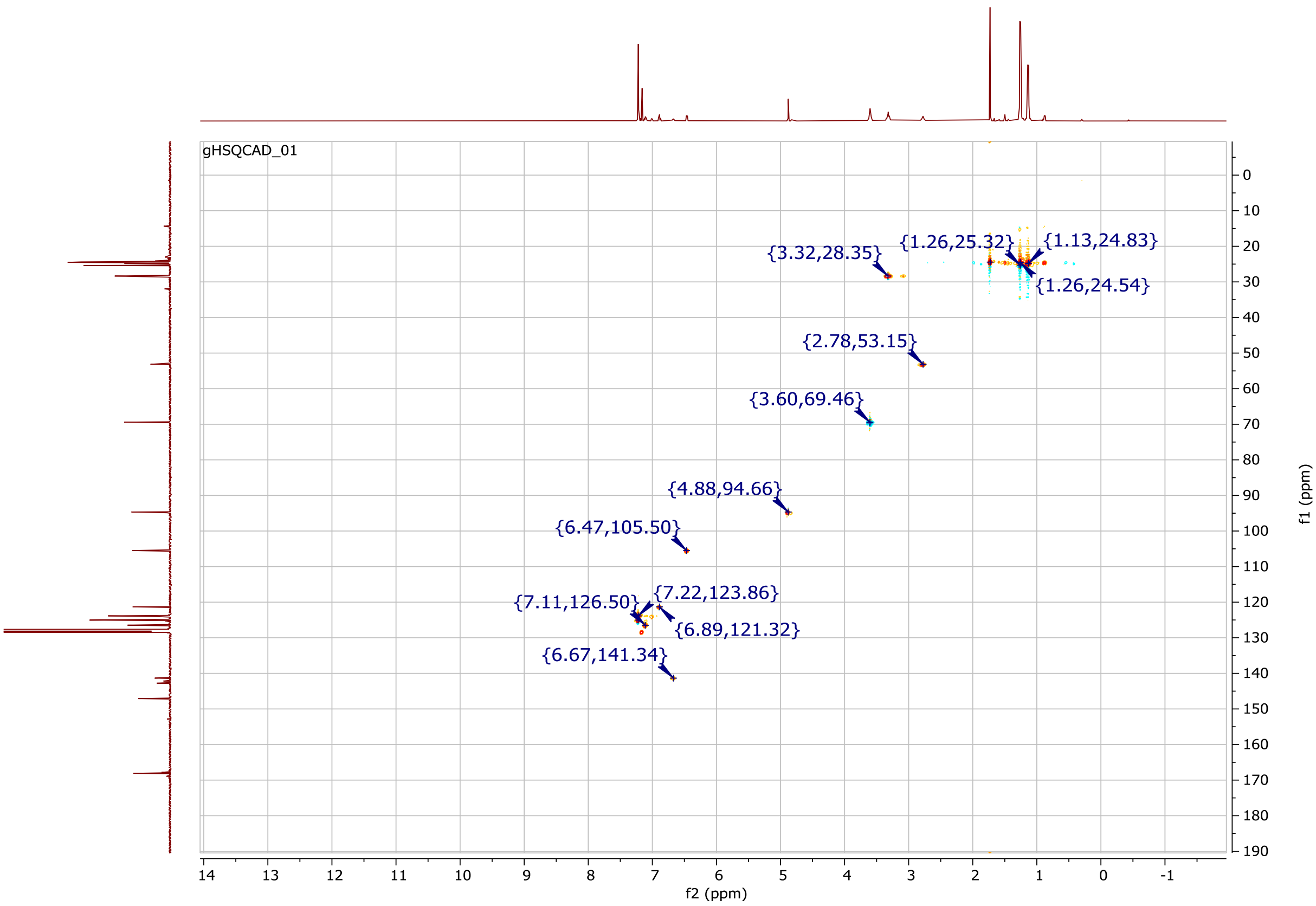

Figure S153: ^{13}C-^1H HSQC NMR (126 MHz, C_6D_6) of isolated **10^{Me}·thf** after recrystallisation from *n*-hexane/THF.

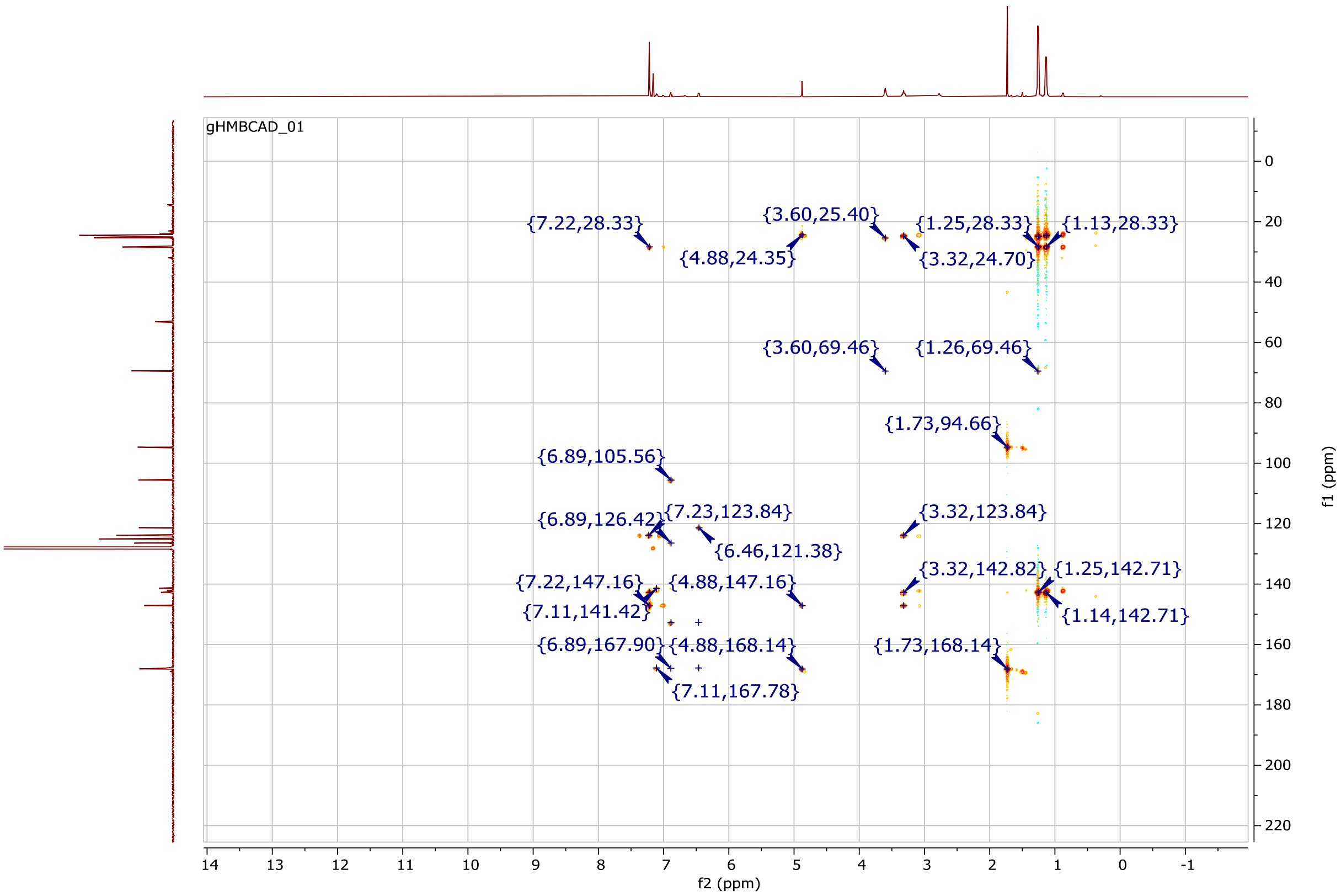

Figure S154: ^{13}C-^{1}H HSQC NMR (126 MHz, C_6D_6) of isolated **10^{Me}·thf** after recrystallisation from *n*-hexane/THF.

2.18 Reaction of [(^{Ad}BDI)Mg^{n}Bu] (1^{Ad}) with anisole, synthesis and characterisation of [(^{Ad}BDI)Mg(C_6H_4-2-OMe)($MeOC_6H_5$)] (10^{Ad}·anisole)

A J. Young's NMR tube was charged with 15 mg **1^{Ad}** (0.02 mmol) and 0.6 ml anisole. The pale-yellow solution was heated at 60°C for 16 hours, but no conversion of the starting complex could be discerned by *in situ* ^{1}H NMR spectroscopy. ^{1}H NMR analysis after heating at 100°C for 7 days, however, indicated consumption of the upfield butyl resonance. Volatiles were removed under vacuum. Analysis of the crude residue by ^{1}H NMR spectroscopy indicated the formation of a major BDI-containing product. Slow evaporation of the benzene solution provided colourless needle-like crystals of **10^{Ad}·anisole** suitable for X-ray diffraction analysis, which were isolated in 5.3 mg yield after washing with *n*-hexane. A further 4.9 mg crystalline product was obtained by storing the *n*-hexane washings at -30°C. Total yield: 10.2 mg, 89%.

Analysis calculated for $C_{61}H_{80}MgN_2O_2$: C, 81.62; H, 8.98; N, 3.12%. Found: C, 80.48; H, 9.63; N, 2.81%. Despite repeated attempts, more accurate elemental analysis was not obtained for this compound.

^{1}H NMR (500 MHz, C_6D_6) δ 7.28 (dd, J = 6.6, 1.7 Hz, 1H, 1-Mg-*3*-C_6H_4-1-OMe), 7.12 (dd, J = 7.9 Hz, 2H, Mg-O(CH_3)-*3,5*-C_6H_5),* 7.09 – 7.03 (m, 2H, 1-Mg-*4*-C_6H_4-1-OMe, Mg-O(CH_3)-*2,6*-C_6H_5), 7.03 – 6.94 (m, 7H, 1-Mg-*5*-C_6H_4-1-OMe, NC_6H_3-*i*Pr_2), 6.84 (t, J = 7.3 Hz, 1H, Mg-O(CH_3)-*4*-C_6H_5), 6.80 (d, J = 8.5 Hz, 1H, Mg-O(CH_3)-*2,6*-C_6H_5), 6.32 (d, J = 7.9 Hz, 1H, 1-Mg-6-C_6H_4-2-OMe), 6.02 (s, 1H, *H*CC(Ndipp)), 3.44 (hept, J = 6.7 Hz, 4H, *H*C(CH_3)$_2$), 3.29 (s, 2H, C_6H_5OCH_3),* 2.86 (s, 3H, C_6H_4OCH_3), 2.14 (d, J = 3.3 Hz, 12H, ^{Ad}CCH_2CH), 1.87 (s, 6H, ^{Ad}C*H*), 1.63 (d, J = 11.1 Hz, 7H, ^{Ad}CHCH_2CH), 1.53 (d, J = 11.7 Hz, 7H, ^{Ad}CHCH_2CH),* 1.37 (d, J = 6.8 Hz, 12H, HC(CH_3)$_2$), 1.12 (d, J = 6.8 Hz, 12H, HC(CH_3)$_2$). *Inaccurate integral. Rotation of the phenyl ring on the coordinated anisole moiety is slow on the NMR timescale at 298 K, breaking the normal C_2 symmetry axis; however, the protons in the 2/6 and the 3/5 positions were not unambiguously assigned.

^{13}C NMR (126 MHz, C_6D_6) δ 178.57 (N=*C*), 165.89 (1-Mg-*2*-*C*$_6$H$_4$-2-OMe), 160.32 (Mg-O(CH$_3$)-*1*-*C*$_6$H$_5$),* 151.61 (1-Mg-*1*-*C*$_6$H$_4$-2-OMe), 146.57 (*ipso*-N*C*$_6$H$_3$-*i*Pr$_2$), 141.09 (*ortho*-N*C*$_6$H$_3$-*i*Pr$_2$), 140.15 (1-Mg-*3*-*C*$_6$H$_4$-1-OMe), 129.71 (Mg-O(CH$_3$)-*3,5*-*C*$_6$H$_5$), 126.91 (1-Mg-*4*-*C*$_6$H$_4$-1-OMe), 124.36 (Mg-O(CH$_3$)-*2,6*-*C*$_6$H$_5$), 123.65 (*meta*-N*C*$_6$H$_3$-*i*Pr$_2$), 122.84 (*para*-N*C*$_6$H$_3$-*i*Pr$_2$), 120.85 (O(CH$_3$)-*4*-*C*$_6$H$_5$), 114.30 (Mg-O(CH$_3$)-*2,6*-*C*$_6$H$_5$), 105.24 (1-Mg-*6*-*C*$_6$H$_4$-2-OMe), 95.14 (*H*CC(Ndipp)), 54.63 (C$_6$H$_5$OC*H$_3$*), 53.75 (MgC$_6$H$_4$OC*H$_3$*), 48.05 (NC^{Ad}*C*), 43.54 (^{Ad}CC*H$_2$*CH), 37.01 (^{Ad}CHC*H$_2$*CH), 29.62 (^{Ad}C*H*), 28.53 (H*C*(CH$_3$)$_2$), 24.61 (HC(*C*H$_3$)$_2$), 23.75 (HC(*C*H$_3$)$_2$). *Detected indirectly via ^{1}H-^{13}C HMBC experiment. Rotation of the phenyl ring on the coordinated anisole moiety is slow on the NMR timescale at 298 K, breaking the normal C_2 symmetry axis; however, the carbon atoms in the 2/6 were not unambiguously assigned. The carbon resonances for the 3/5 positions are believed to be superimposed.

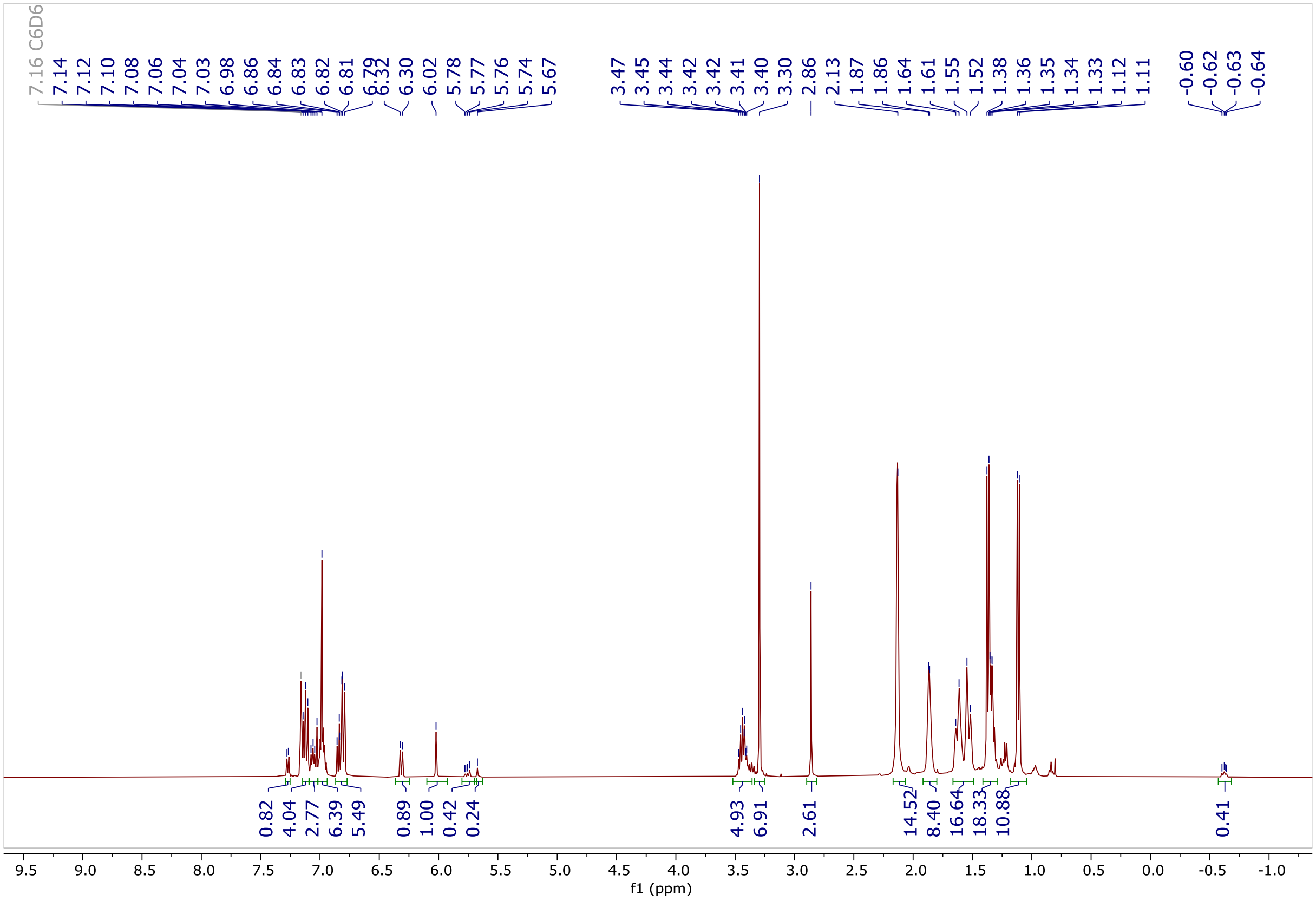

Figure S155: ^{1}H NMR spectrum (C_6D_6, 400 MHz) of crude product **10^{Ad}** after removal of volatiles under vacuum.

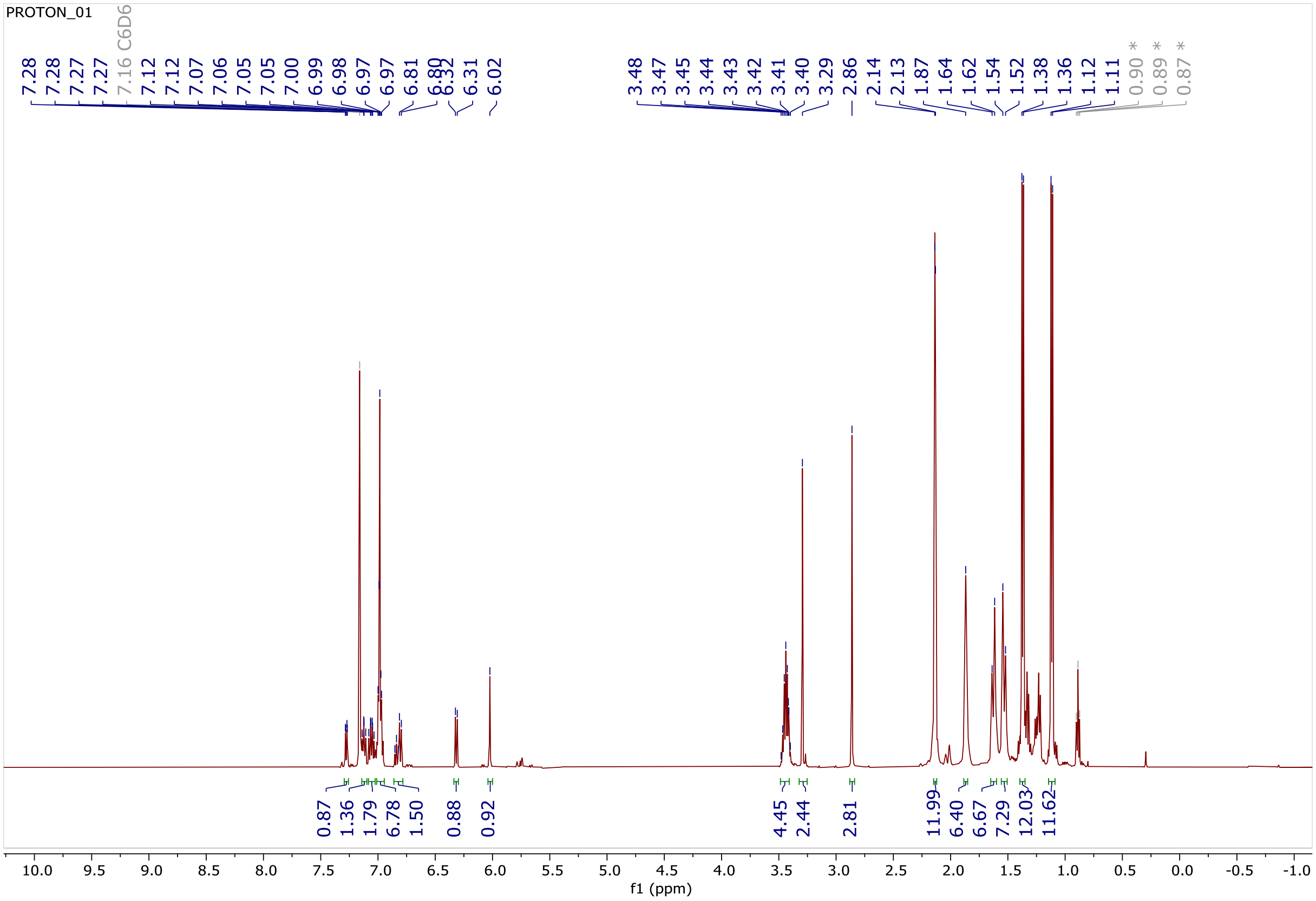

Figure S156: ^{1}H NMR spectrum (C_6D_6, 500 MHz) crystalline product **10^{Ad}·anisole**.

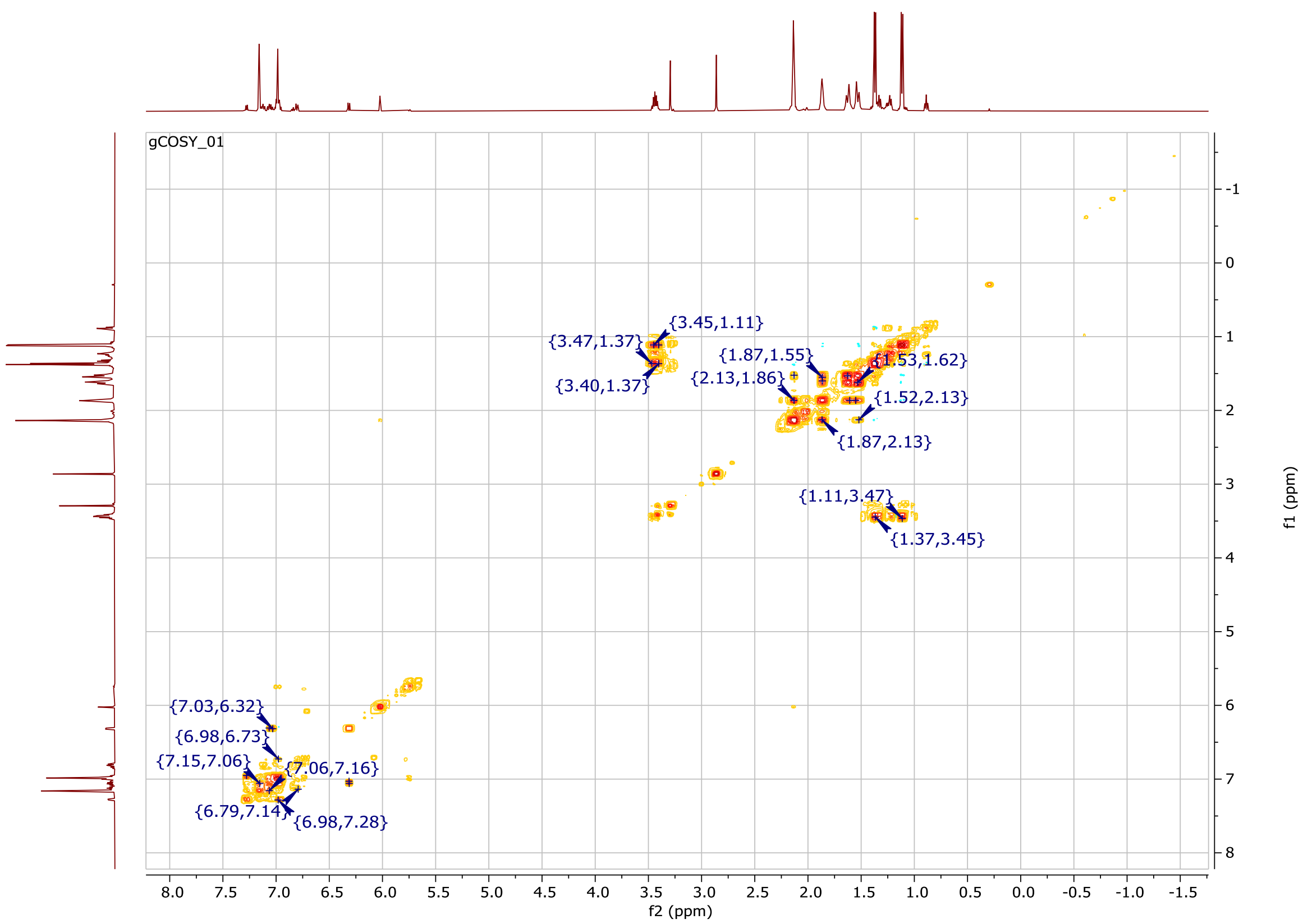

Figure S157: ^{1}H-^{1}H COSY NMR spectrum (C_6D_6, 500 MHz) crystalline product **10^{Ad}·anisole**.

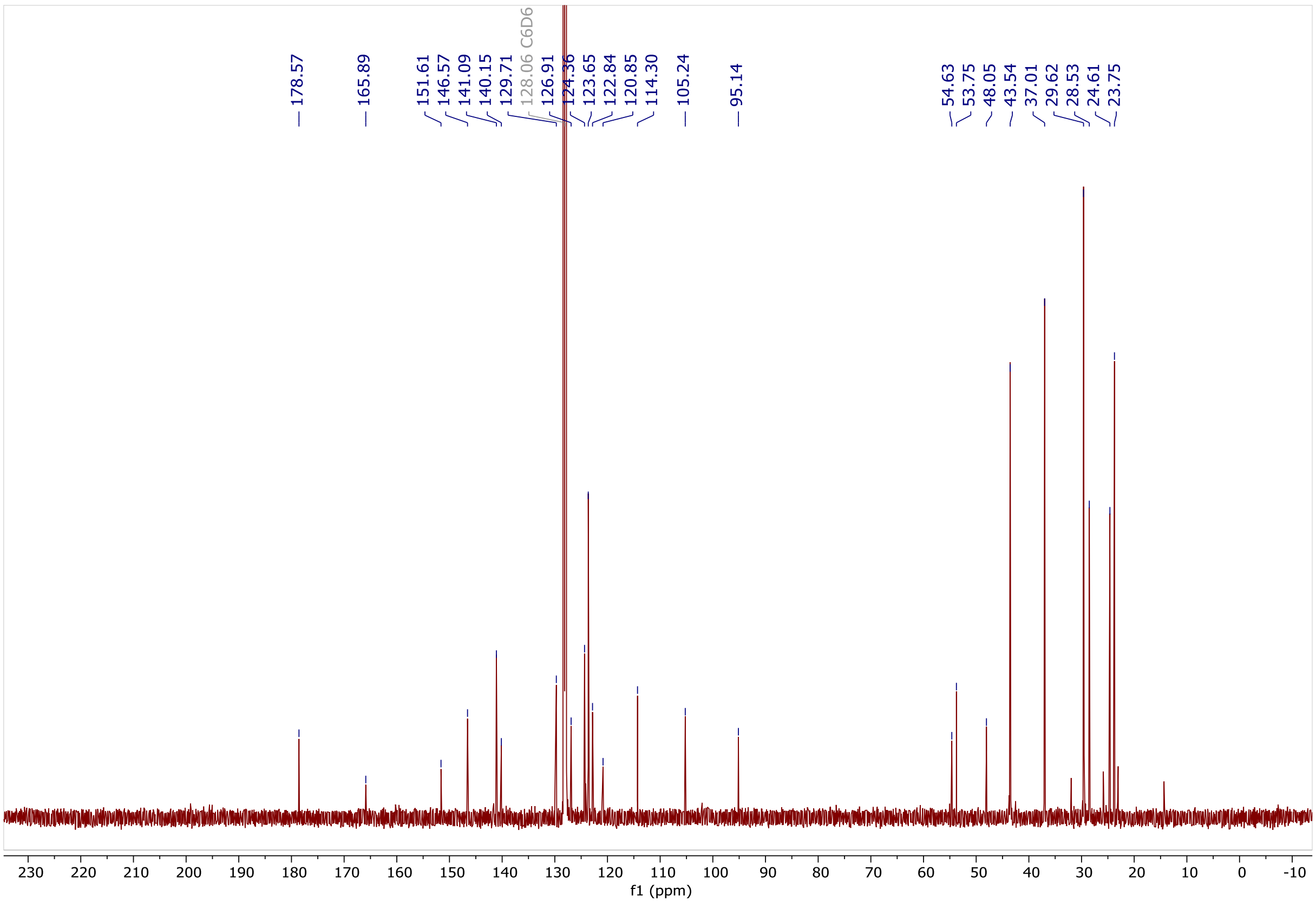

Figure S158: $^{13}C\{^{1}H\}$ NMR spectrum (C_6D_6, 126 MHz) crystalline product **10^{Ad}·anisole**.

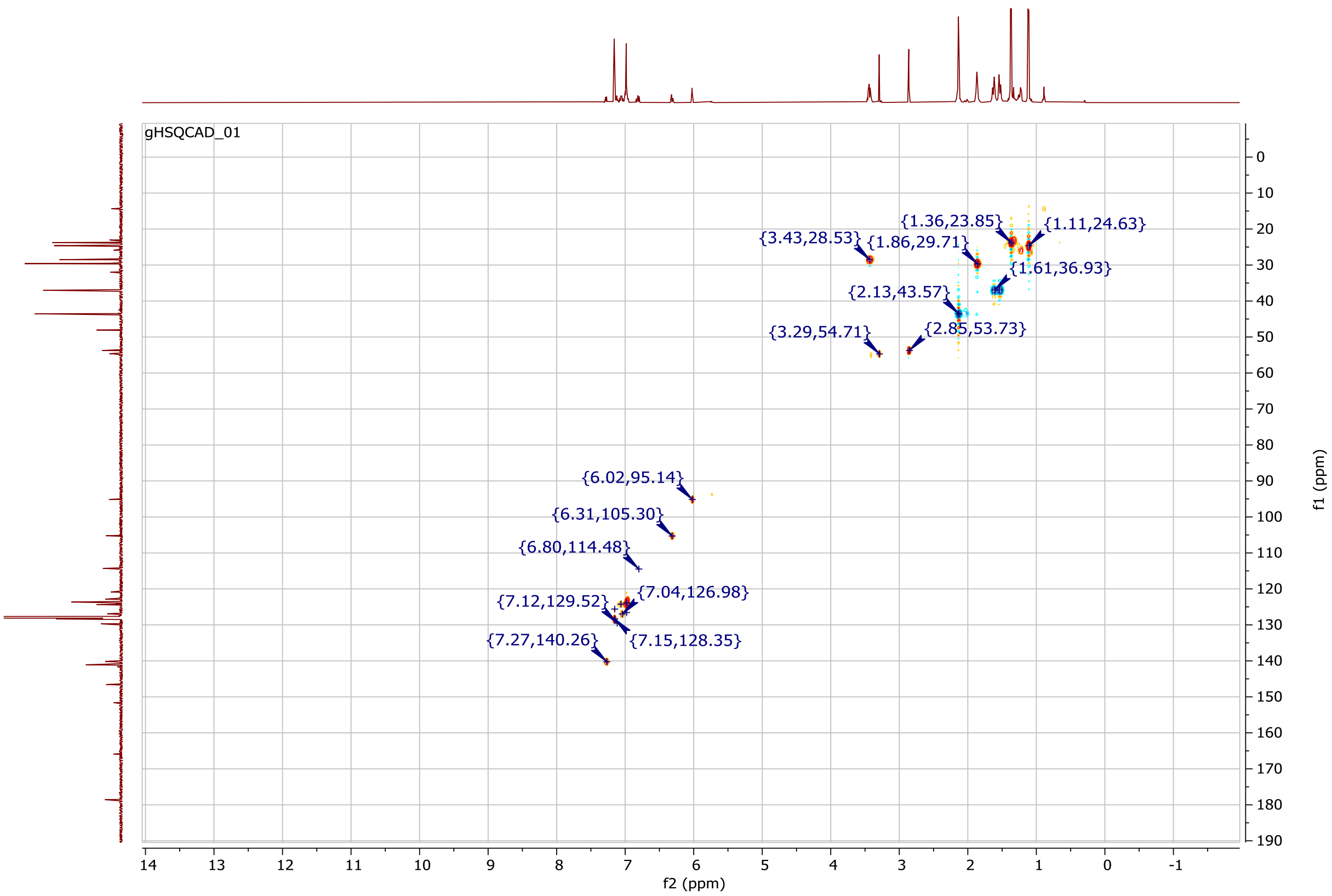

Figure S159: ^{13}C-^{1}H HSQC NMR spectrum (C_6D_6, 126 MHz) crystalline product **10^{Ad}·anisole**.

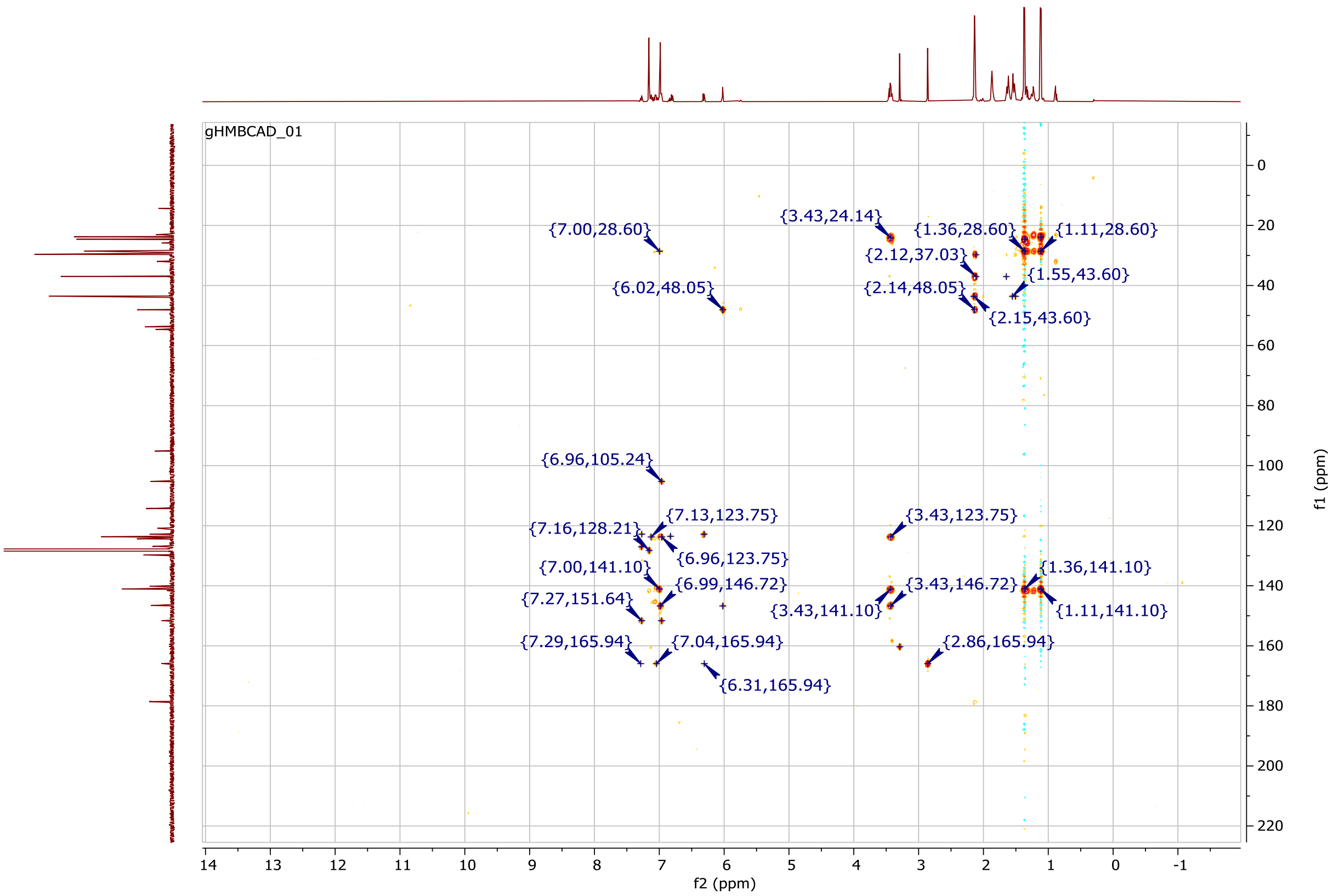

Figure S160: ^{13}C-^{1}H HMBC NMR spectrum (C_6D_6, 126 MHz) crystalline product **10^{Ad}·anisole**.

2.19 Reaction of [(^{Ad}BDI)MgPh] (3^{Ad}) with 1.2 equiv. MeOBpin to form [(^{Ad}BDI)Mg(μ-OMe)$_2$Mg(κ^1-^{Ad}BDI)] (11) and [(^{Ad}BDI)Mg(μ-OMe)$_2$(Bpin)] (12) and crystallisation of 11

A C_6H_6 solution (0.6 ml) of [(^{Ad}BDI)Mg^{n}Bu] (**1^{Ad}**, 13 mg, 0.018 mmol) was heated at 100°C in a sealed J. Young's NMR tube for 8 days to yield **3^{Ad}**. Volatiles were removed under vacuum, then the residue was redissolved in C_6D_6. The addition of 3.6 µl MeOBpin (0.02 mmol) resulted in an immediate colour change from very pale yellow to bright yellow. Analysis by ^{1}H and ^{11}B NMR spectroscopy after 30 minutes indicated partial conversion of **3^{Ad}**. After 64 h, **3^{Ad}** had been fully consumed. ^{1}H NMR showed three ^{Ad}BDI γ-methine resonances of equal intensity at δ 5.99, 5.79, 4.41 ppm, which correspond to the unsymmetrical dimeric magnesium methoxide **11** and the boronate species **12**. ^{11}B NMR showed two major signals at δ 31.2 ppm (identified as PhBpin by comparison to an authentic sample) and δ 7.3 ppm (**12**). The reaction mixture was decanted into a vial and the solvent allowed to slowly evaporate, yielding colourless plate-like single crystals of **11** and a sticky yellow residue. The crystals were washed with *n*-hexane and a suitable plate-shaped crystal of **11**·1.5(C_6D_6)·(C_6H_{14}) was subsequently selected for single crystal X-ray diffraction analysis.

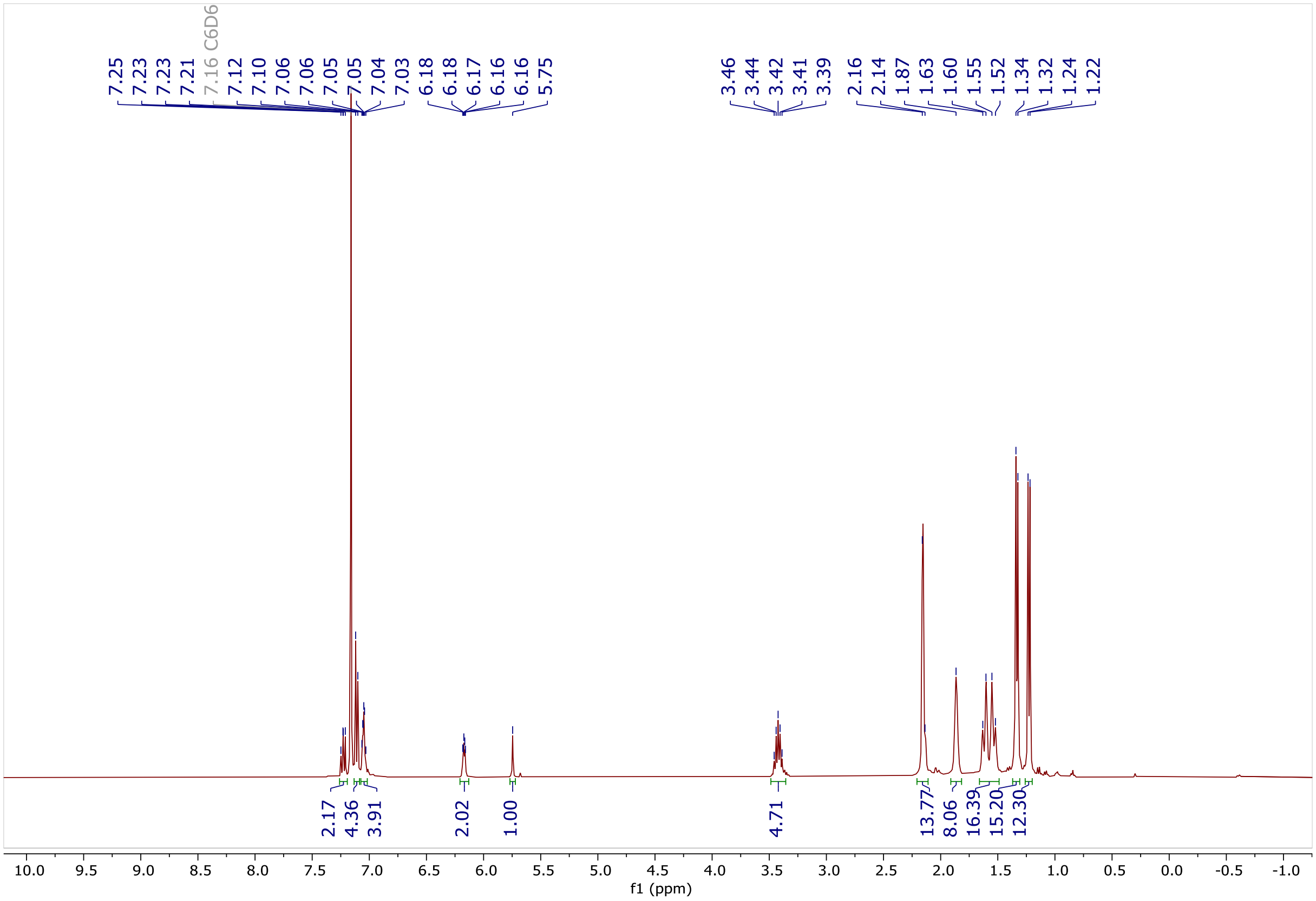

Figure S161: ^{1}H NMR (400 MHz, C_6D_6) of **3^Ad^** prepared *in situ* after removal of volatiles and re-dissolution in C_6D_6 before addition of MeOBpin.

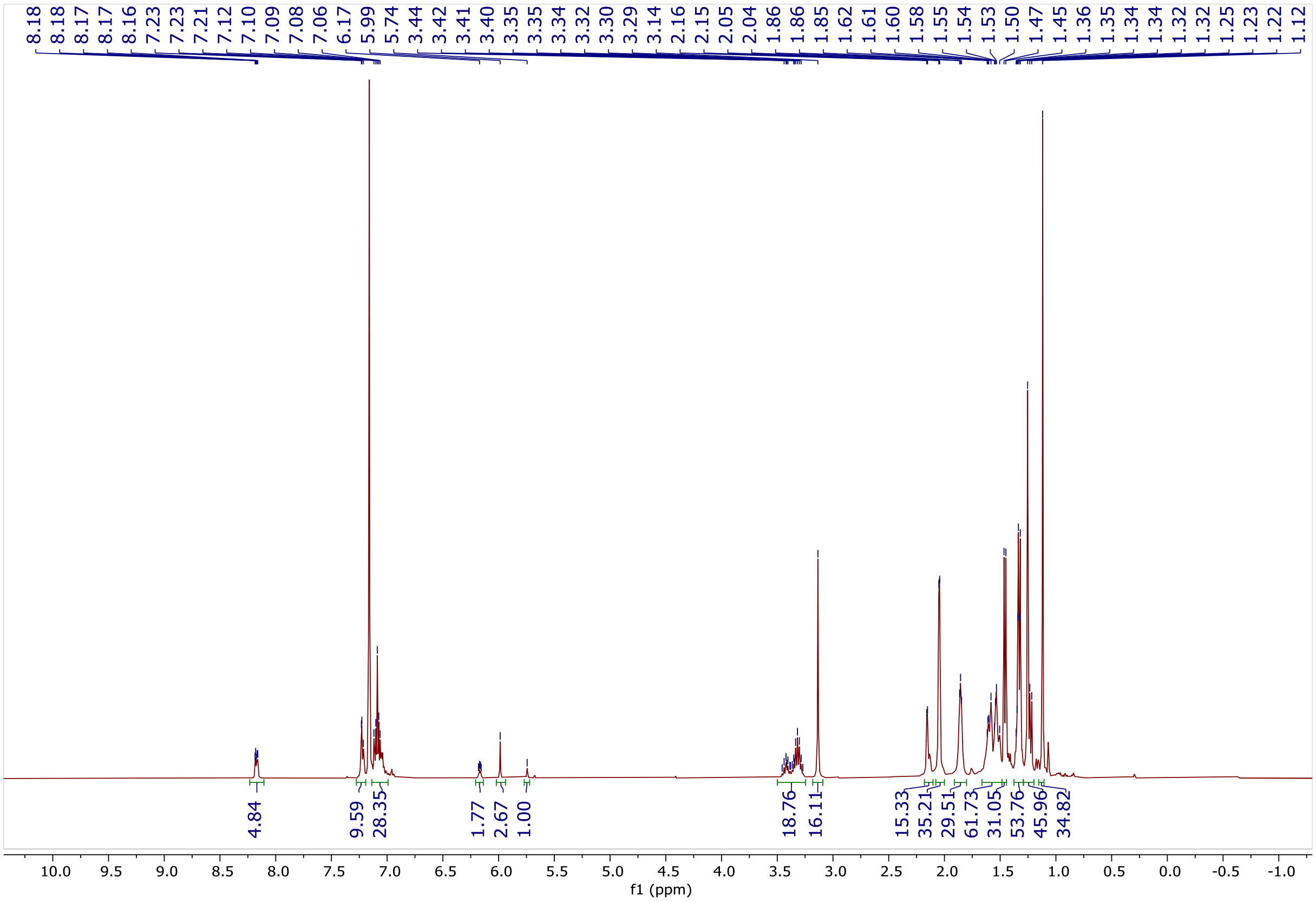

Figure S162: ^{1}H NMR (400 MHz, C_6D_6) 30 minutes after addition of MeOBpin

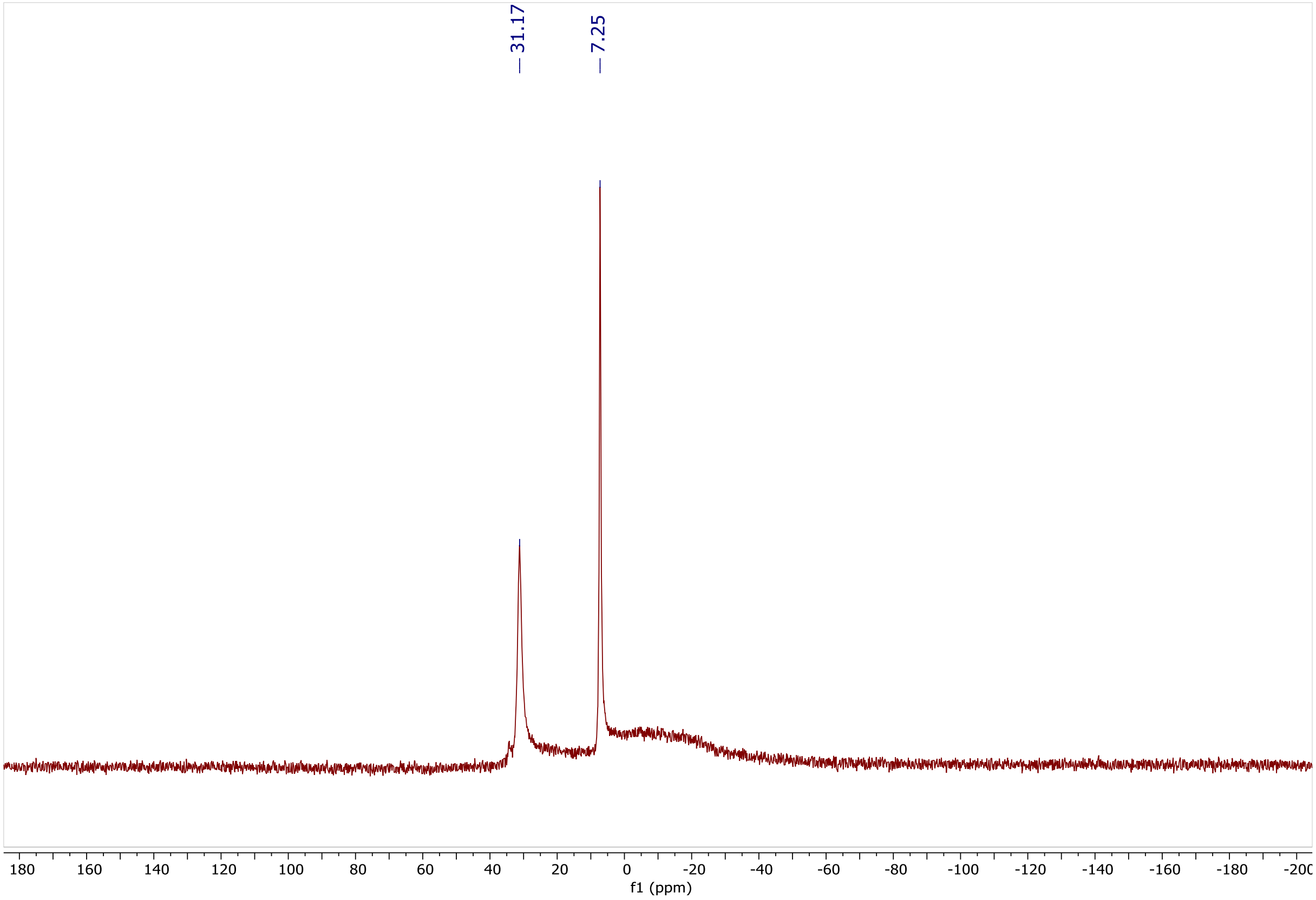

Figure S163: ^{11}B NMR (128 MHz, C_6D_6) 30 minutes after addition of MeOBpin.

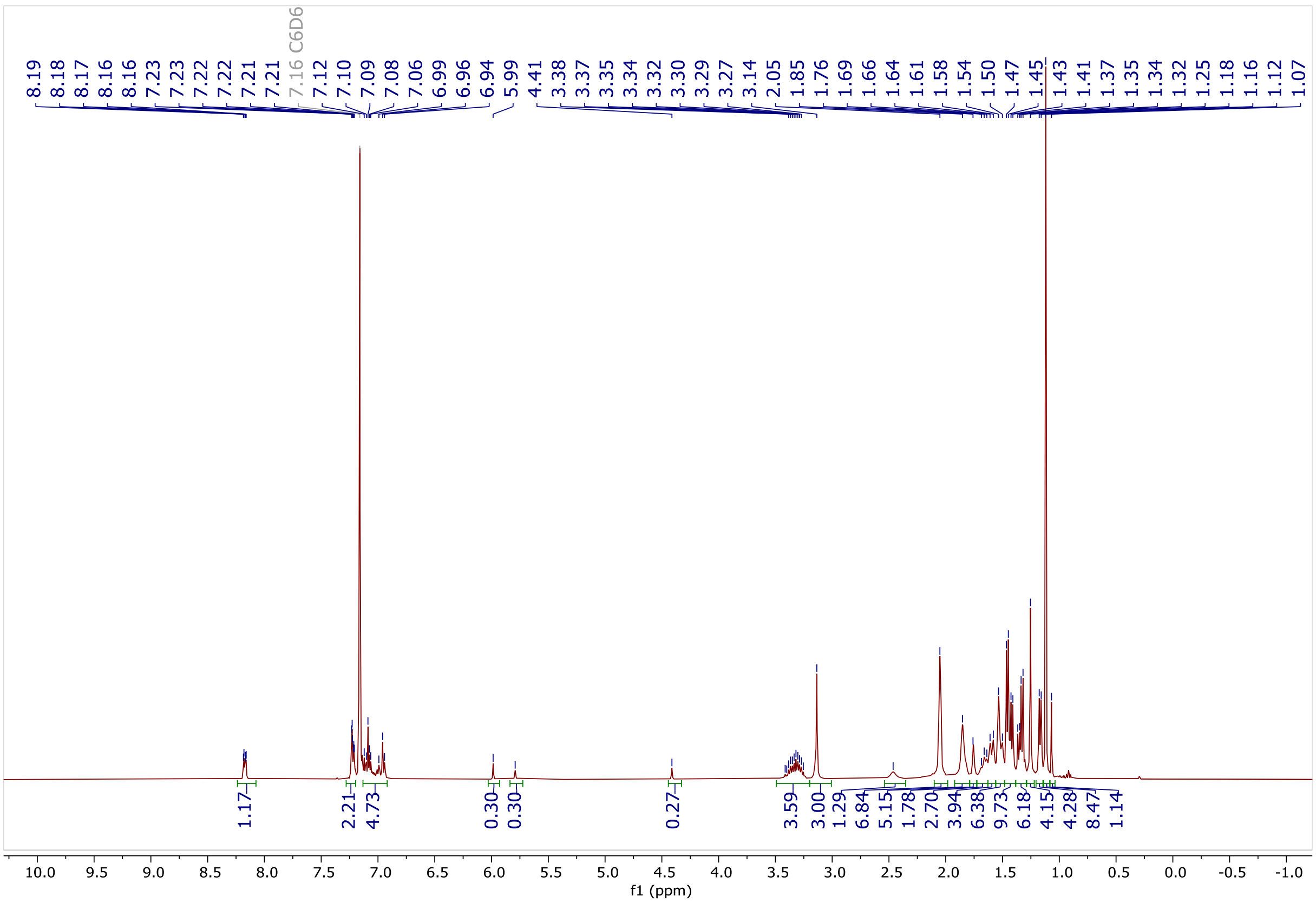

Figure S164: ^{1}H NMR (400 MHz, C_6D_6) after 64 h at room temperature.

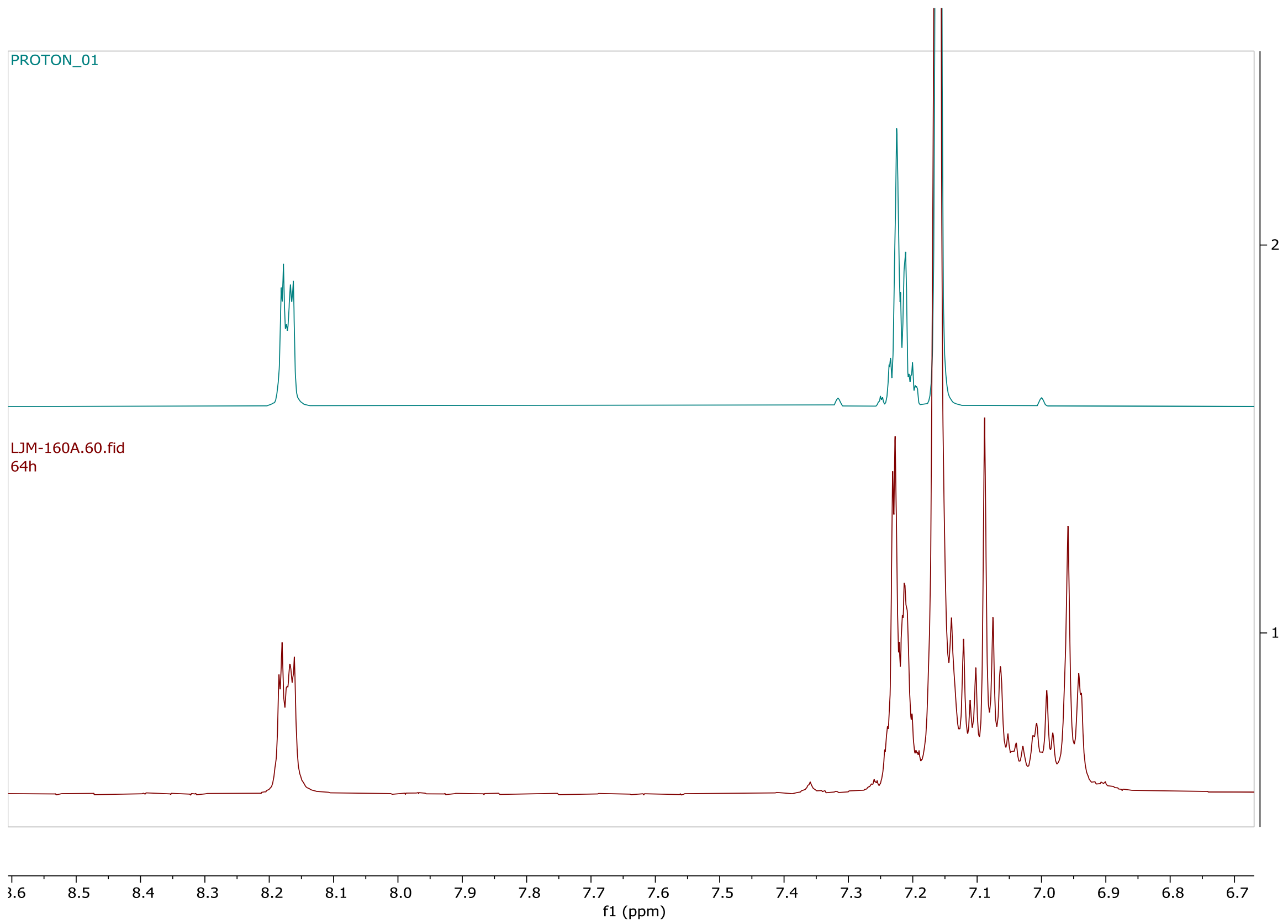

Figure S165: Expanded stacked ^{1}H NMR spectra of PhBpin (top, 500 MHz, C_6D_6) and reaction mixture after 64 h at room temperature (400 MHz, C_6D_6), highlighting formation of PhBpin.

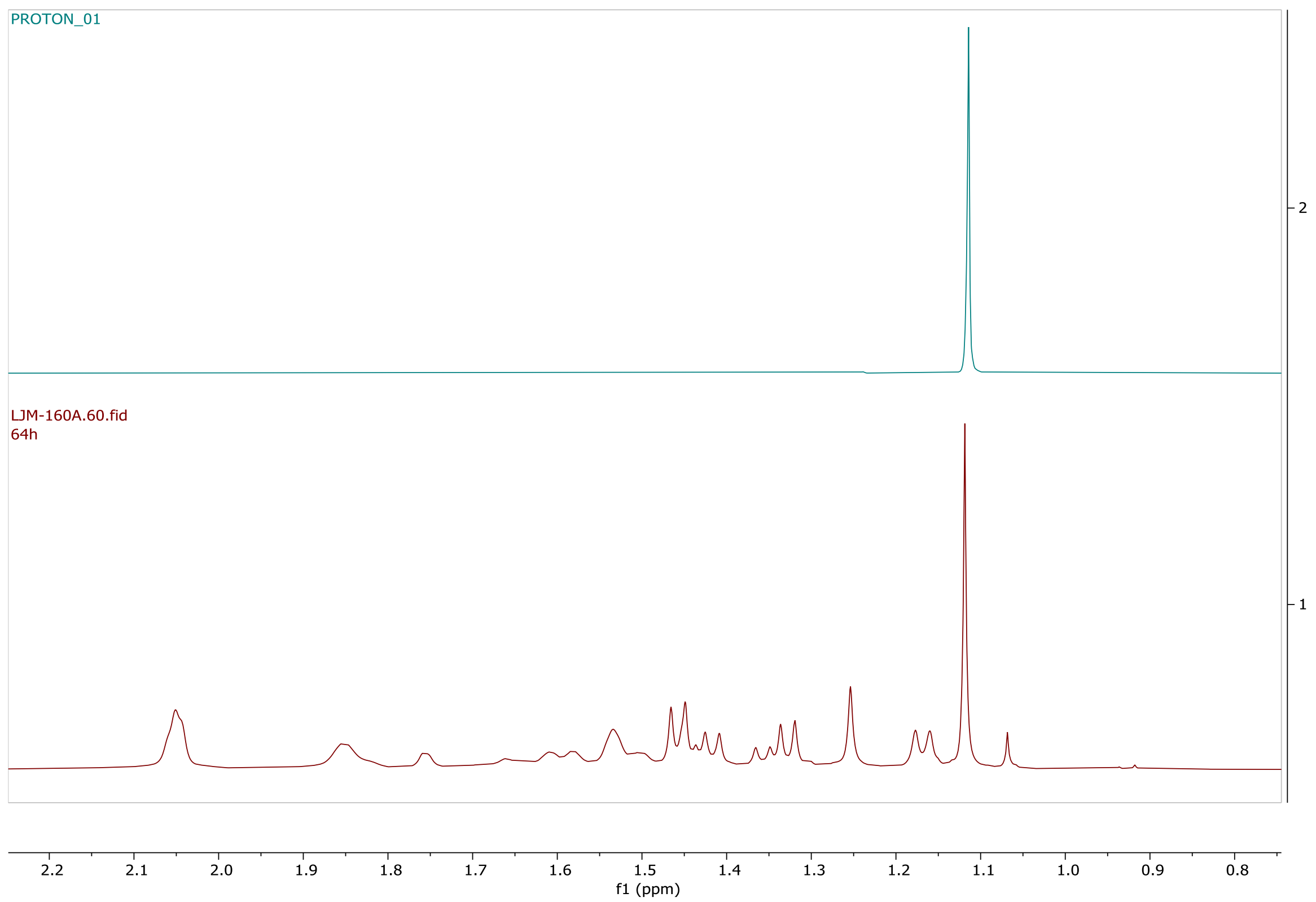

Figure S166: Expanded stacked ^{1}H NMR spectra of PhBpin (top, 500 MHz, C_6D_6) and reaction mixture after 64 h at room temperature (400 MHz, C_6D_6), highlighting formation of PhBpin.

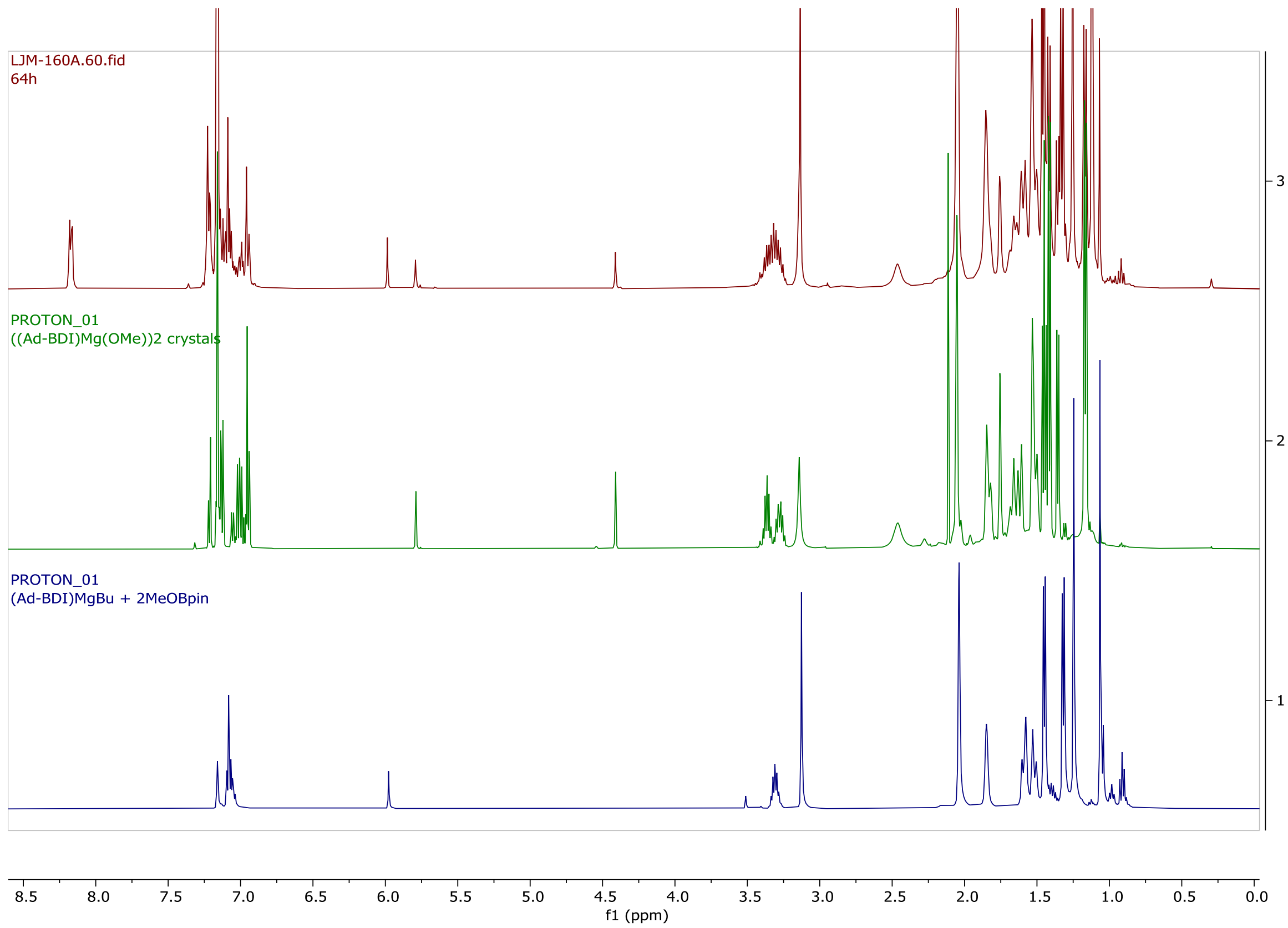

Figure S167: Stacked ^{1}H NMR spectra (C_6D_6) 64 h after addition of MeOBpin (top, 400 MHz), of isolated **11** (centre, 500 MHz), and of isolated **12** (500 MHz, bottom)

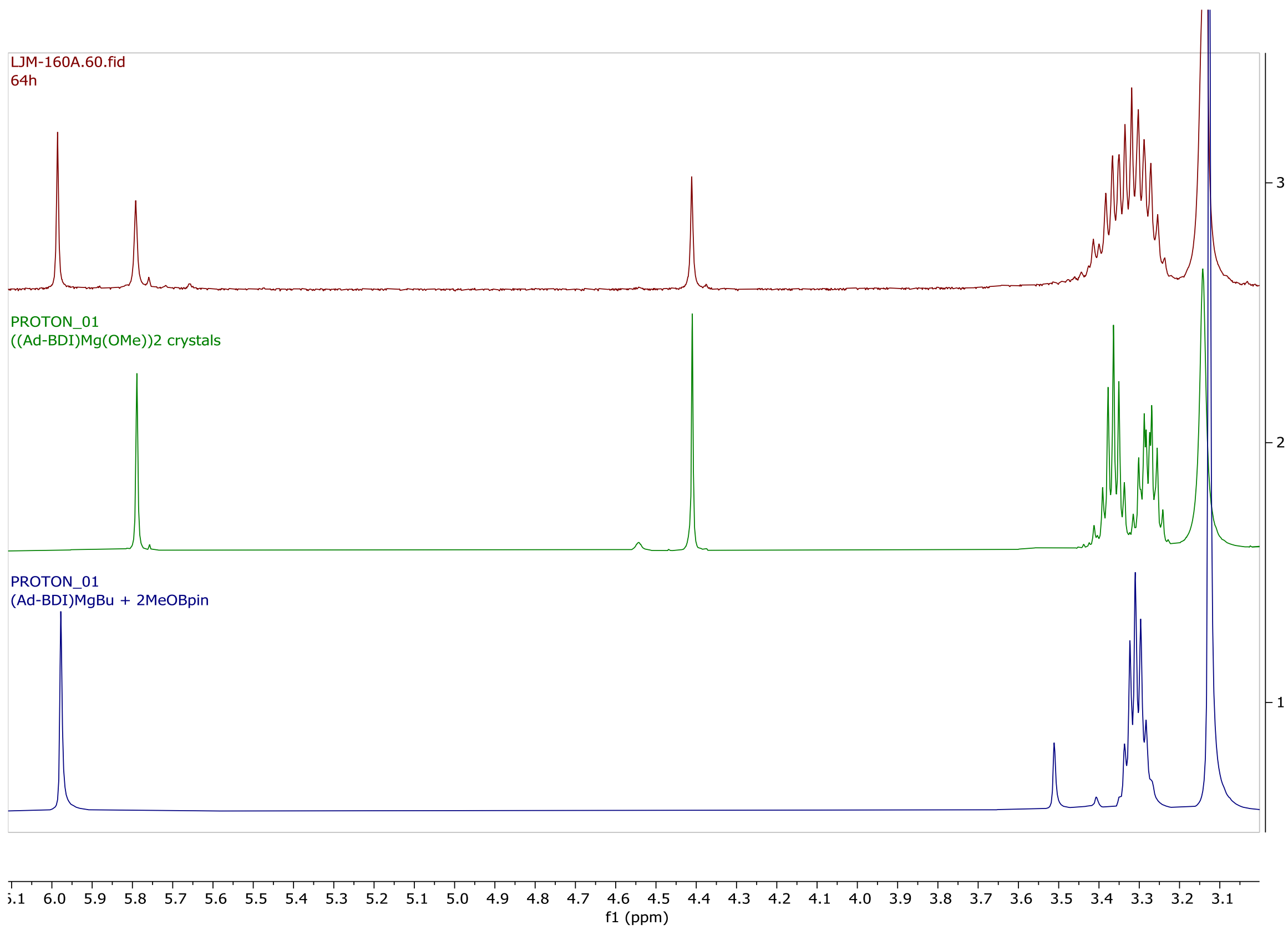

Figure S168: Expanded Stacked ^{1}H NMR spectra (C_6D_6) 64 h after addition of MeOBpin (top, 400 MHz), of isolated **11** (centre, 500 MHz), and of isolated **12** (500 MHz, bottom)

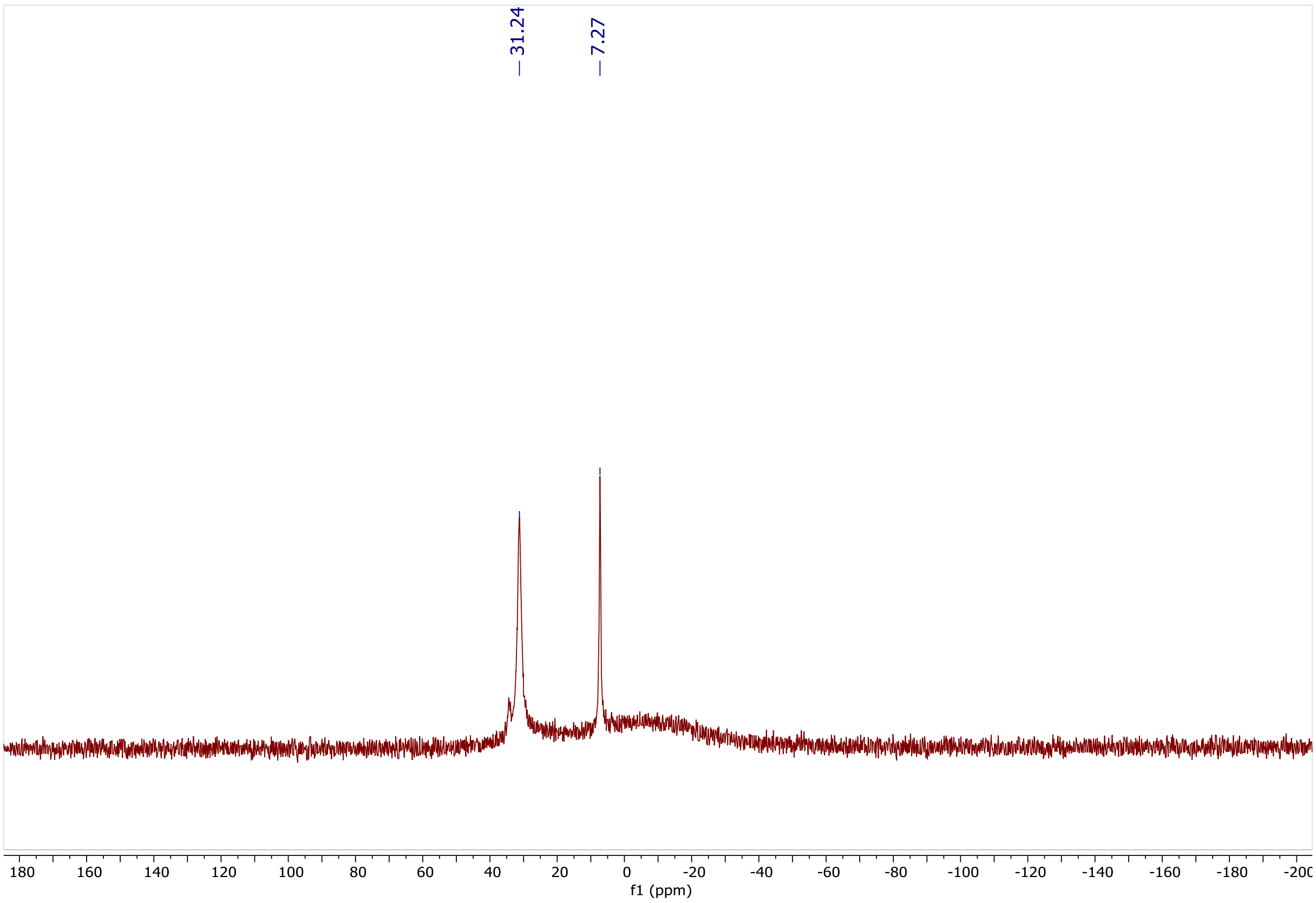

Figure S169: ^{11}B NMR (128 MHz, C_6D_6) after 64 h at room temperature.

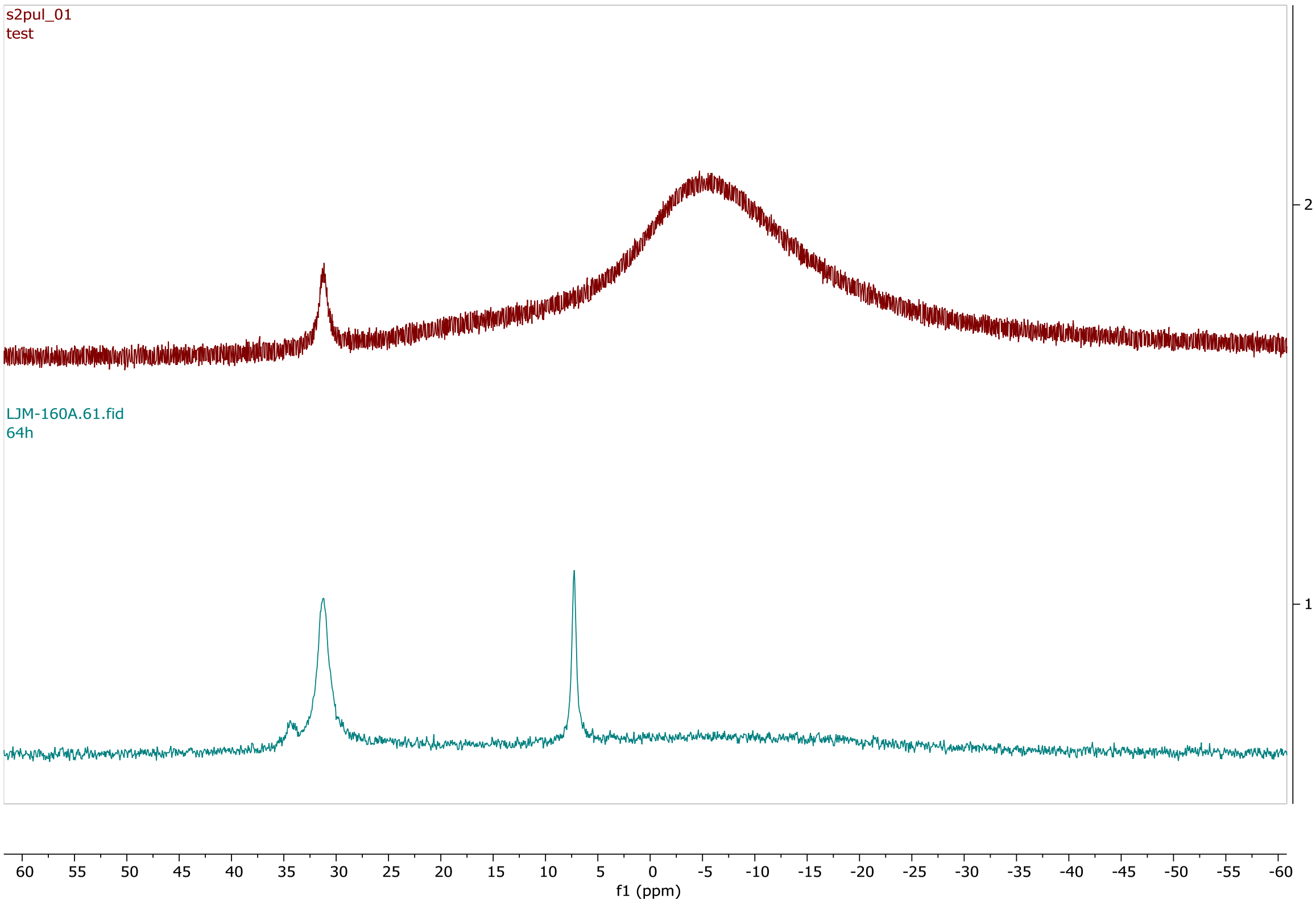

Figure S170: Stacked ^{11}B NMR spectra of PhBpin (top, 160 MHz, C_6D_6) and reaction mixture after 64 h at room temperature (bottom, 128 MHz, C_6D_6), highlighting formation of PhBpin.

2.20 Reaction of [(^{Ad}BDI)MgPh] (3^{Ad}) with 2 equiv. MeOBpin to form [(^{Ad}BDI)Mg(μ-OMe)$_2$(Bpin)] (12)

A C_6H_6 solution (0.6 ml) of [(^{Ad}BDI)Mg^{n}Bu] (**1^{Ad}**, 37 mg, 0.05 mmol) was heated at 100°C in a sealed J. Young's NMR tube for 8 days to afford **3^{Ad}**. Volatiles were removed under vacuum, then the residue was redissolved in C_6D_6. The addition of 3 mg hexamethyl benzene (0.02 mmol) as internal standard and 16 mg MeOBpin (0.1 mmol) resulted in an immediate colour change from very pale yellow to bright yellow. Analysis by ^{1}H and ^{11}B NMR spectroscopy after 30 minutes indicated full conversion of **3^{Ad}** to a single new BDI-containing species, **12.** ^{11}B NMR showed two major signals at δ 31.2 (identified as PhBpin by comparison to an authentic sample) and 7.3 ppm (**12**).

The reaction was repeated, starting from 13 mg **1^{Ad}** (0.018 mmol) in benzene, after **3^{Ad}** had formed, volatiles were removed under vacuum. 0.5 ml *n*-hexane was added to the as-prepared **3^{Ad}**, followed by 3.6 µl MeOBpin (0.022 mmol). The resulting bright-yellow solution was placed in the freezer at -30°C. After 2 days, **12** was isolated as a yellow microcrystalline solid in 3.6 mg yield (23%).

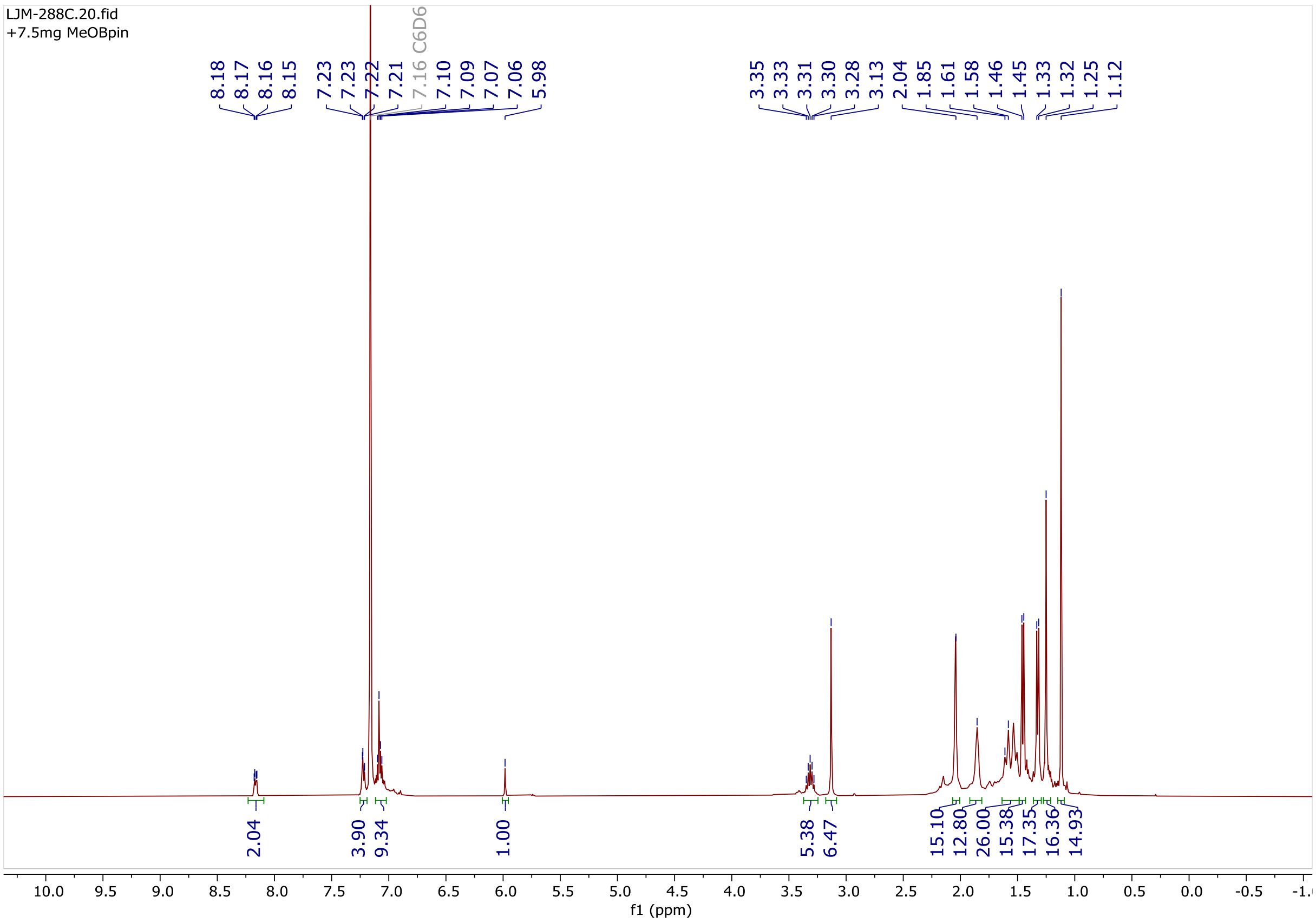

Figure S171: *In situ* ^{1}H NMR spectrum (400 MHz, C_6D_6) of the reaction of **3^{Ad}** with 2 equiv. MeOBpin.

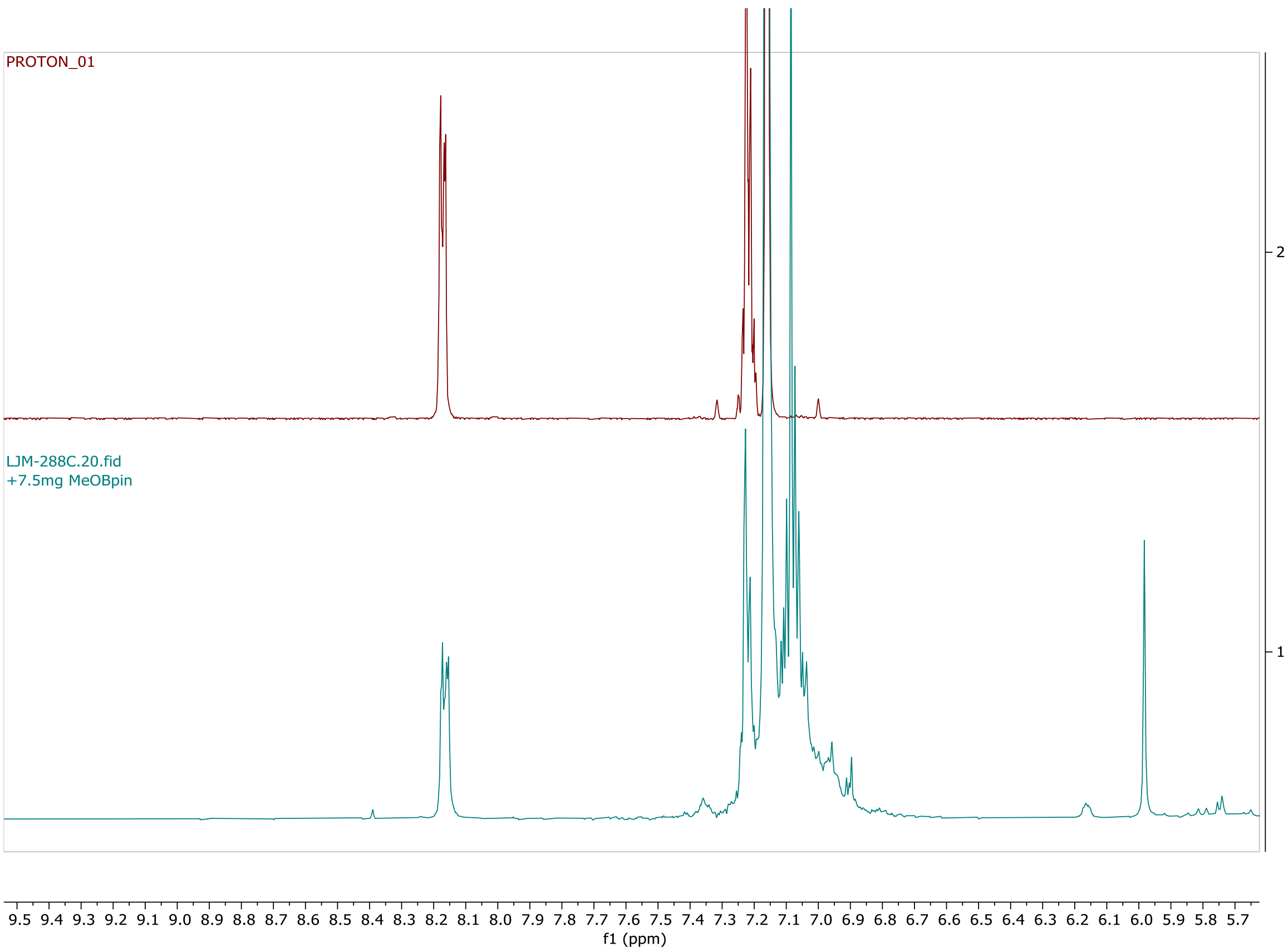

Figure S172: Stacked ^{1}H NMR spectra of PhBpin (top, 500 MHz, C_6D_6) and of the reaction of **3^{Ad}** with 2 equiv. MeOBpin (bottom, 400 MHz, C_6D_6), highlighting formation of PhBpin.

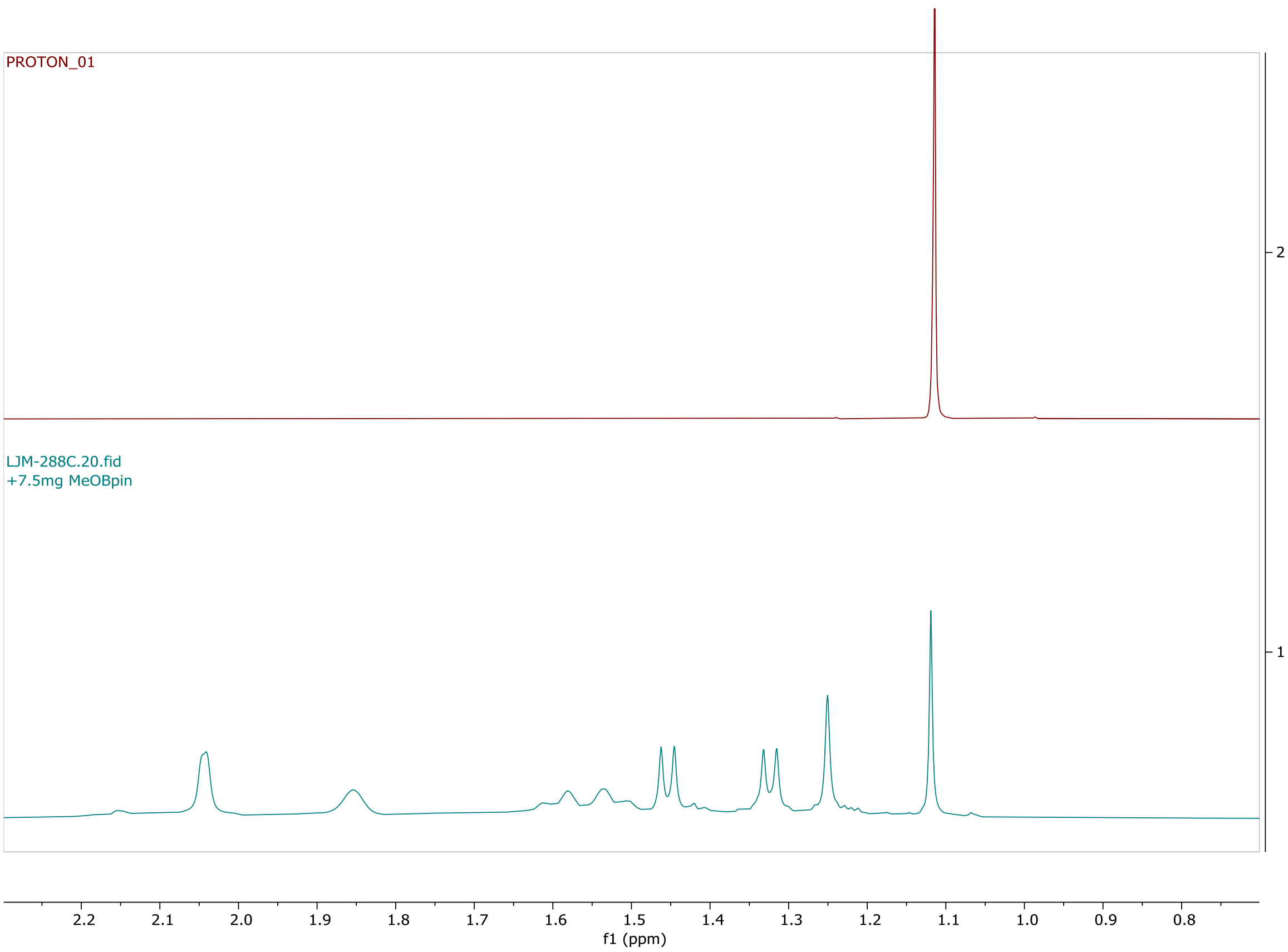

Figure S173: Expanded stacked ^{1}H NMR spectra of PhBpin (top, 500 MHz, C_6D_6) and of the reaction of **3^{Ad}** with 2 equiv. MeOBpin (bottom, 400 MHz, C_6D_6), highlighting formation of PhBpin.

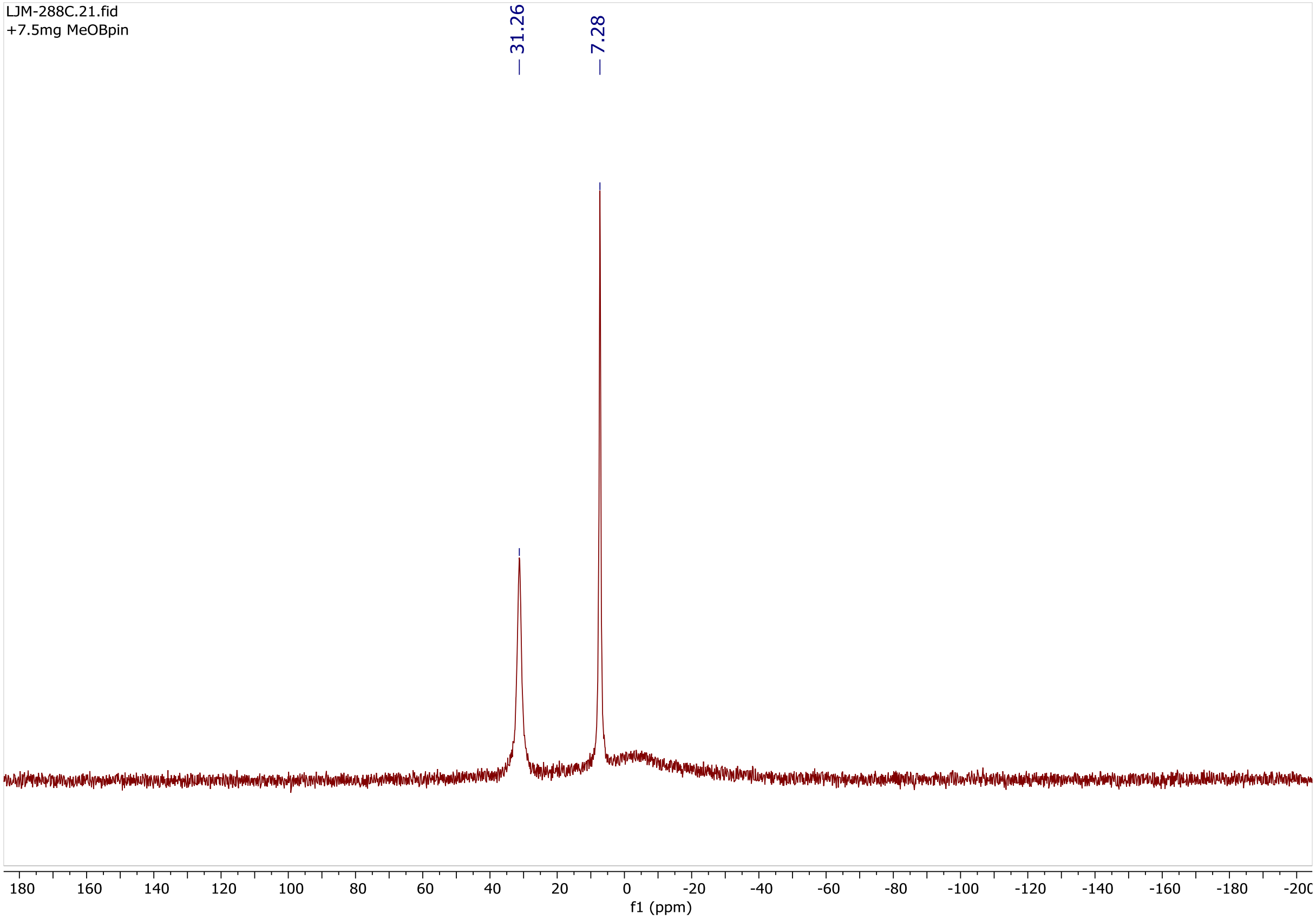

Figure S174: *In situ* $^{11}B\{^1H\}$ NMR spectrum (128 MHz, C_6D_6) of the reaction of **3**^{Ad} with 2 equiv. MeOBpin.

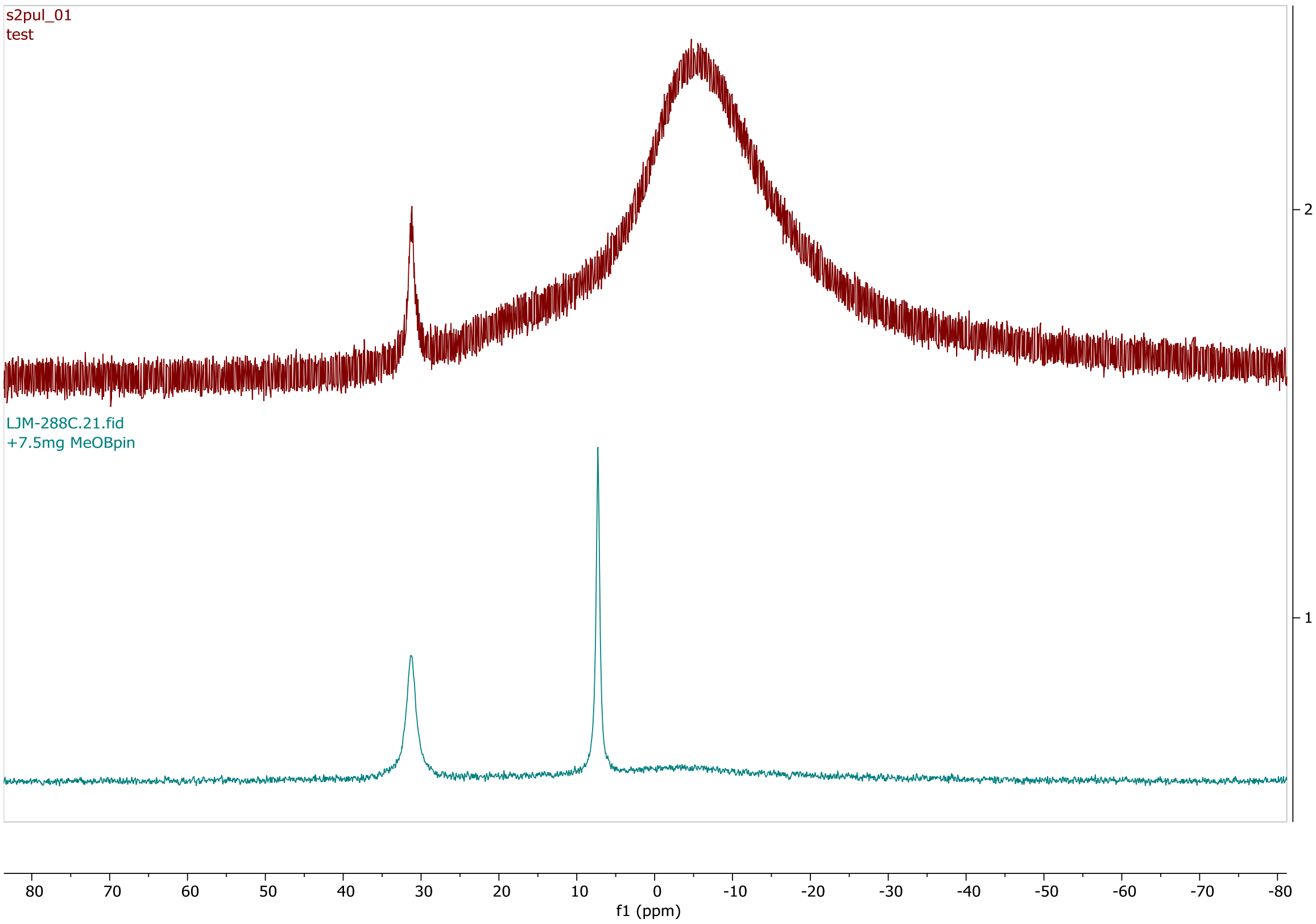

Figure S175: Stacked $^{11}B\{^1H\}$ NMR spectra of PhBpin (top, 160 MHz, C_6D_6) and of the reaction of **3**^{Ad} with 2 equiv. MeOBpin (bottom, 128 MHz, C_6D_6), highlighting formation of PhBpin.

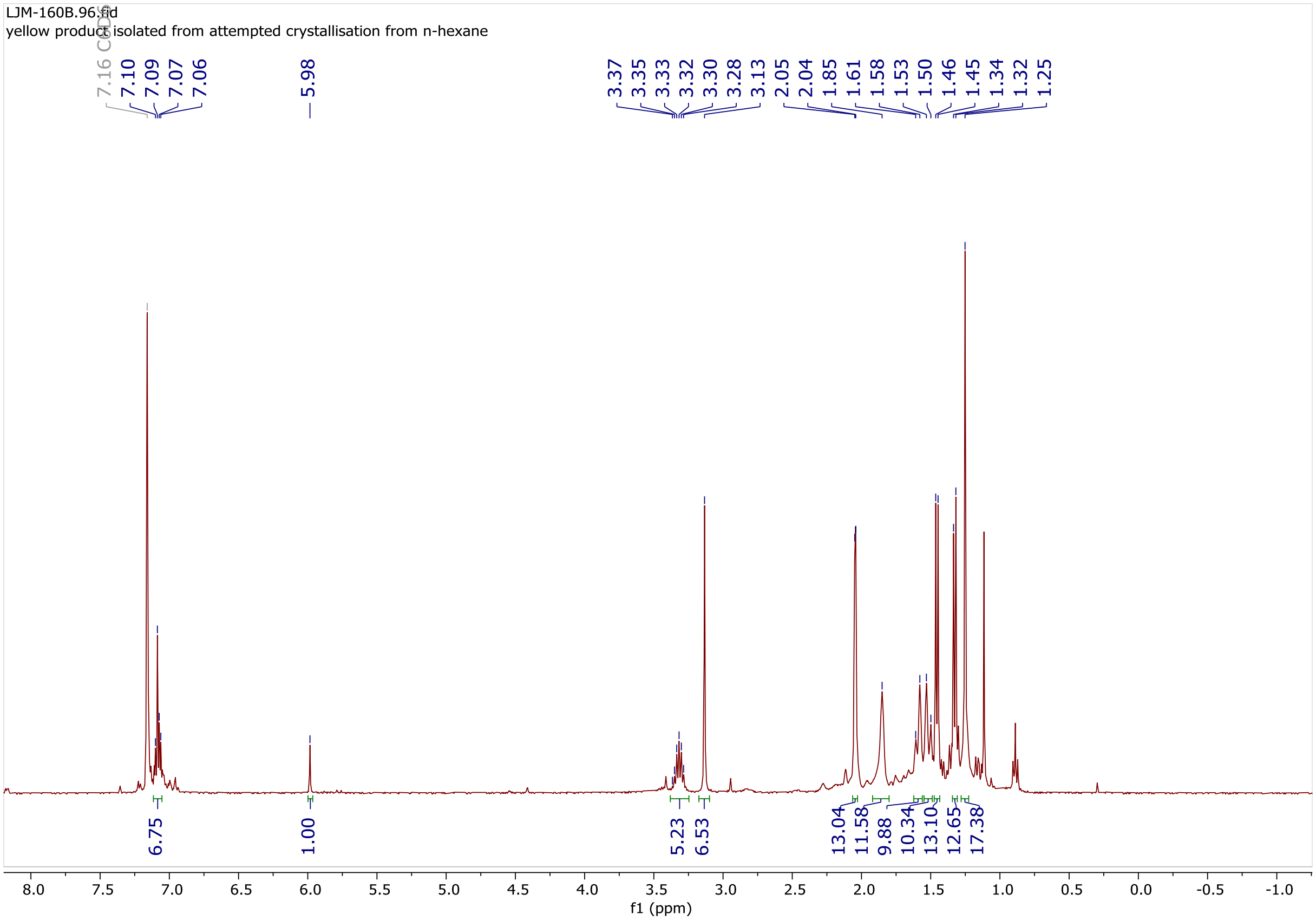

Figure S176: ^{1}H NMR (400 MHz, C_6D_6) of an isolated sample of **12** obtained from the reaction of $\mathbf{3^{Ad}}$ with MeOBpin in *n*-hexane.

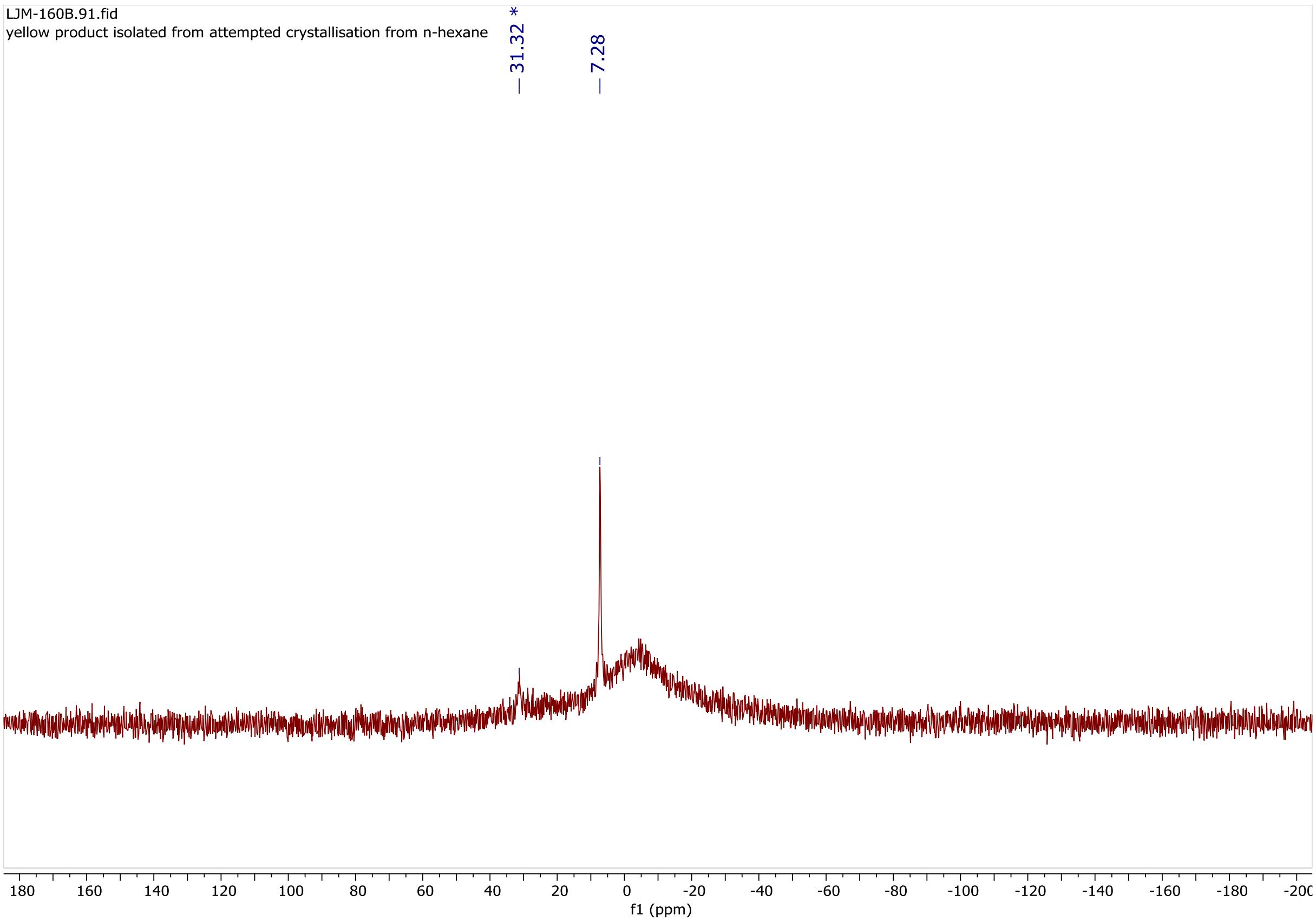

Figure S177: $^{11}B\{^{1}H\}$ NMR spectrum (128 MHz, C_6D_6) of an isolated sample of **12** obtained from the reaction of $\mathbf{3^{Ad}}$ with MeOBpin in *n*-hexane. *Residual PhBpin.

2.21 Synthesis and characterisation of [(^{Ad}BDI)Mg(μ-OMe)$_2$Mg(κ^1-^{Ad}BDI)] (11)

A J. Young's NMR tube was charged with a 0.6 ml C_6D_6 solution of 50 mg [(^{Ad}BDI)Mg^{n}Bu] (**1^{Ad},** 0.06 mmol), then 11 μl MeOBpin (0.068 mmol) was added. The reaction mixture was heated at 40°C for 5 days. **11** crystallised directly from the reaction mixture and was isolated as a colourless crystalline solid in 27 mg yield (55%) after decanting the supernatant, washing with minimal toluene and drying under vacuum.

Analysis calculated for $C_{96}H_{136}Mg_2N_4O_2$: C, 80.82; H, 9.61; N, 3.93%. Found: C, 80.87; H, 9.92; N, 3.33%.

^{1}H NMR (500 MHz, C_6D_6) δ 7.23 – 7.20 (m, 2H, (3,5-C$_6$*H$_3$*^{i}Pr$_2$ (κ^1)), 7.13 (dd, *J* = 7.6, 2.1 Hz, 4H, (3,5-C$_6$*H$_3$*^{i}Pr$_2$ (κ^1)),** 7.03 – 6.98 (m, 4H, (4-*C$_6$*H$_3$^{i}Pr$_2$ (κ^2))*, 6.98 – 6.92 (m, 5H, 4-C$_6$*H$_3$*^{i}Pr$_2$ (κ^1) + 3,5-*C$_6$*H$_3$^{i}Pr$_2$ (κ^2)), 5.79 (s, 1H, *H*C(C(Ad)Ndipp)$_2$ (κ^2)), 4.41 (s, 1H, *H*C=C(Ad)Ndipp (κ^1)), 3.36 (hept, *J* = 6.7 Hz, 4H, *H*C(CH$_3$)$_2$ (κ^2)), 3.29 (d, *J* = 6.8 Hz, 2H, *H*C(CH$_3$)$_2$ (κ^1)), 3.27 (d, *J* = 6.8 Hz, 2H, *H*C(CH$_3$)$_2$ (κ^1)), 3.14 (s, br, 6H, OC*H$_3$*), 2.05 (d, *J* = 3.0 Hz, 12H, ^{Ad}CC*H$_2$*CH (κ^2)), 1.87 – 1.80 (m, 9H, (^{Ad}C*H* (κ^1 + κ^2)), 1.75 (d, *J* = 3.0 Hz, 6H, ^{Ad}CC*H$_2$*CH (κ^1)), 1.70 – 1.59 (m, 13H, ^{Ad}CHC*H$_2$*CH (κ^2) + ^{Ad}C*H* (κ^1)), 1.55 – 1.48 (m, 16H, ^{Ad}CHC*H$_2$*CH (κ^2 + κ^1)), 1.46 (d, *J* = 6.6 Hz, 12H, HC(C*H$_3$*)$_2$ (κ^1)), 1.44 (d, *J* = 6.6 Hz, 12H, HC(C*H$_3$*)$_2$ (κ^1)), 1.41 (d, *J* = 6.7 Hz, 12H, HC(C*H$_3$*)$_2$ (κ^2)), 1.36 (d, *J* = 6.7 Hz, 6H, HC(C*H$_3$*)$_2$ (κ^1)), 1.17 (d, *J* = 6.8 Hz, 18H, HC(C*H$_3$*)$_2$ (κ^2 + κ^1)). κ^n annotations denote whether the resonance corresponds to the chelating (κ^2) BDI ligand, or the (κ^1) β-iminoamide ligand. One of the 4-C$_6$*H$_3$*^{i}Pr$_2$ (κ^1) resonances overlaps with the residual solvent signal. *Measured integral higher than expected due to overlapping toluene impurity. **Measured integral higher than expected.

^{13}C NMR (126 MHz, C_6D_6) δ 179.09 (N=*C*(Ad)CH (κ^2)), 174.75 (N=*C*(Ad)CH (κ^1)), 164.40 (N–*C*(Ad)CH (κ^1)), 148.99 (1-*C$_6$*H$_3$^{i}Pr$_2$ (κ^1)), 147.10 (1-*C$_6$*H$_3$^{i}Pr$_2$ (κ^1)), 145.93 (1-*C$_6$*H$_3$^{i}Pr$_2$ (κ^2)), 144.95 (2,6-*C$_6$*H$_3$^{i}Pr$_2$ (κ^1)), 141.90 (2,6-*C$_6$*H$_3$^{i}Pr$_2$ (κ^2)), 134.08 (2,6-*C$_6$*H$_3$^{i}Pr$_2$ (κ^1)), 124.99 (4-*C$_6$*H$_3$^{i}Pr$_2$ (κ^1)), 124.81 (4-*C$_6$*H$_3$^{i}Pr$_2$ (κ^2)), 124.50 (3,5-*C$_6$*H$_3$^{i}Pr$_2$ (κ^1)), 123.85 (3,5-*C$_6$*H$_3$^{i}Pr$_2$ (κ^2)), 122.90 (3,5-*C$_6$*H$_3$^{i}Pr$_2$ (κ^1)), 119.62 (4-*C$_6$*H$_3$^{i}Pr$_2$ (κ^1)), 96.80 (*H*C=C(Ad)Ndipp (κ^1)), 94.31 (*H*C(C(Ad)Ndipp)$_2$ (κ^2)), 51.06 (O*C*H$_3$), 48.53 (N=C^{Ad}*C* (κ^2)), 48.30 (N=C^{Ad}*C* (κ^1)), 43.26 (^{Ad}C*C*H$_2$CH (κ^2)), 42.24 (N=C^{Ad}C*C*H$_2$CH (κ^1)), 39.78 (N–C^{Ad}*C* (κ^1)), 37.38 (^{Ad}CH*C*H$_2$CH (κ^1)), 37.23 (N–C^{Ad}C*C*H$_2$CH (κ^1)), 37.02 (^{Ad}CH*C*H$_2$CH (κ^1)), 36.90 (^{Ad}CH*C*H$_2$CH (κ^2)), 29.68 (^{Ad}*C*H (κ^2)), 29.43 (^{Ad}*C*H (κ^1)), 28.99 (^{Ad}*C*H (κ^1)), 28.57 (H*C*(CH$_3$)$_2$ (κ^1)), 28.40 (H*C*(CH$_3$)$_2$ (κ^1)), 27.81 (H*C*(CH$_3$)$_2$ (κ^2)), 26.20 (HC(*C*H$_3$)$_2$ (κ^1)), 26.08 (HC(*C*H$_3$)$_2$ (κ^2)), 25.59 (HC(*C*H$_3$)$_2$ (κ^1)), 24.85 (HC(*C*H$_3$)$_2$ (κ^1)), 24.41 (HC(*C*H$_3$)$_2$ (κ^2)), 23.61 (HC(*C*H$_3$)$_2$ (κ^1)). κ^n annotations denote whether the resonance corresponds to the chelating (κ^2) BDI ligand, or the (κ^1) β-iminoamide ligand.

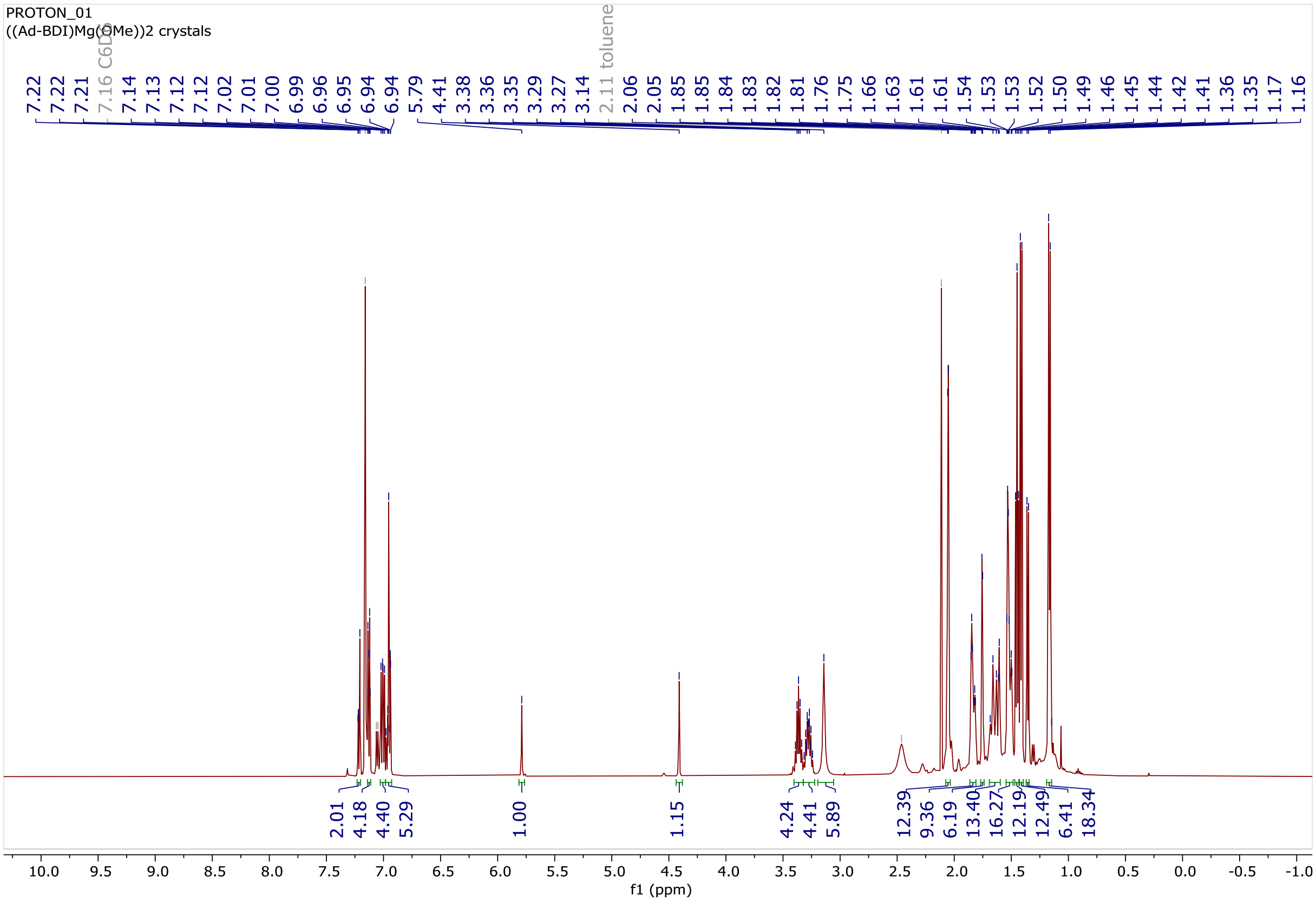

Figure S178: ^{1}H NMR (500 MHz, C_6D_6)

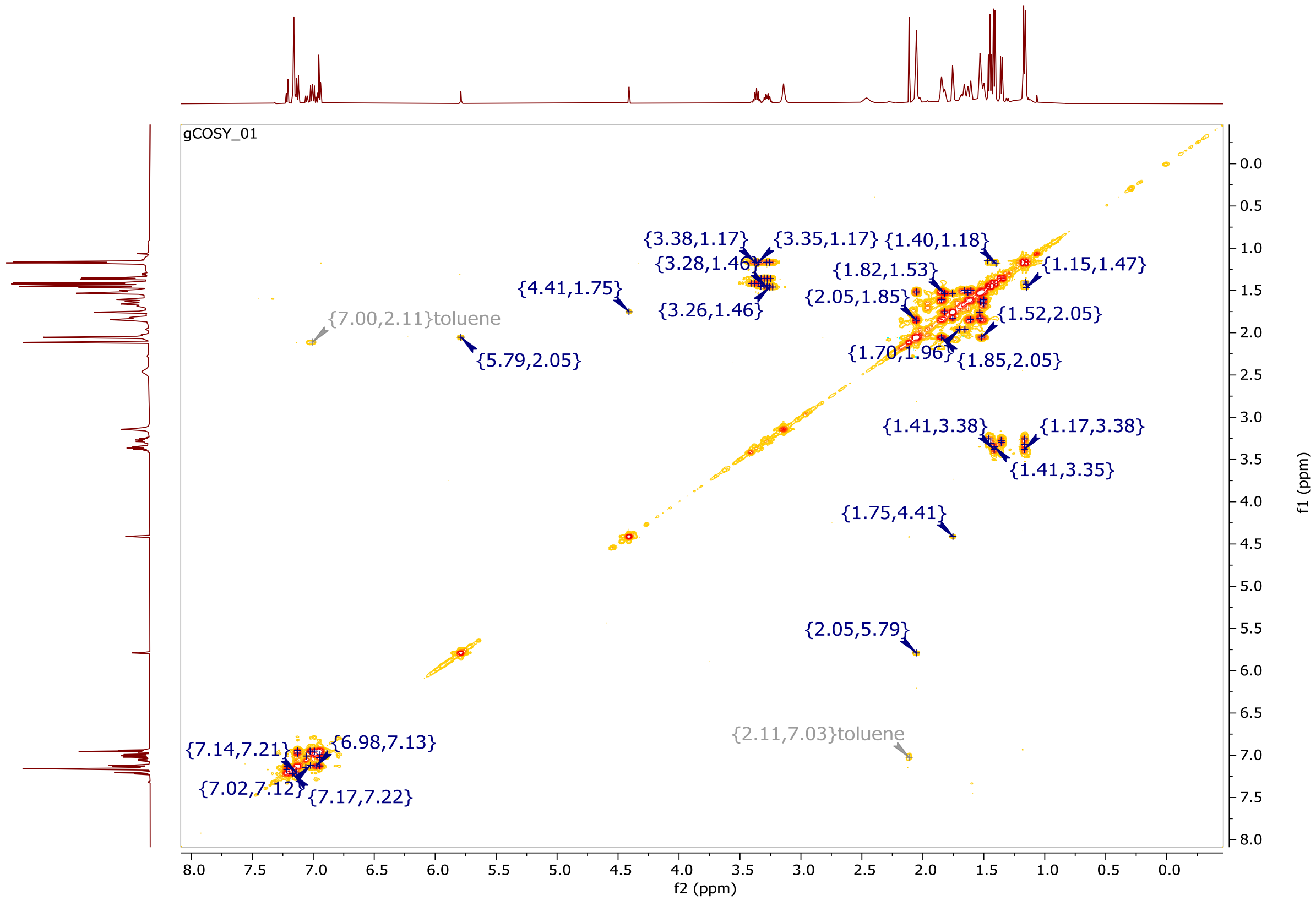

Figure S179: ^{1}H-^{1}H COSY NMR (500 MHz, C_6D_6)

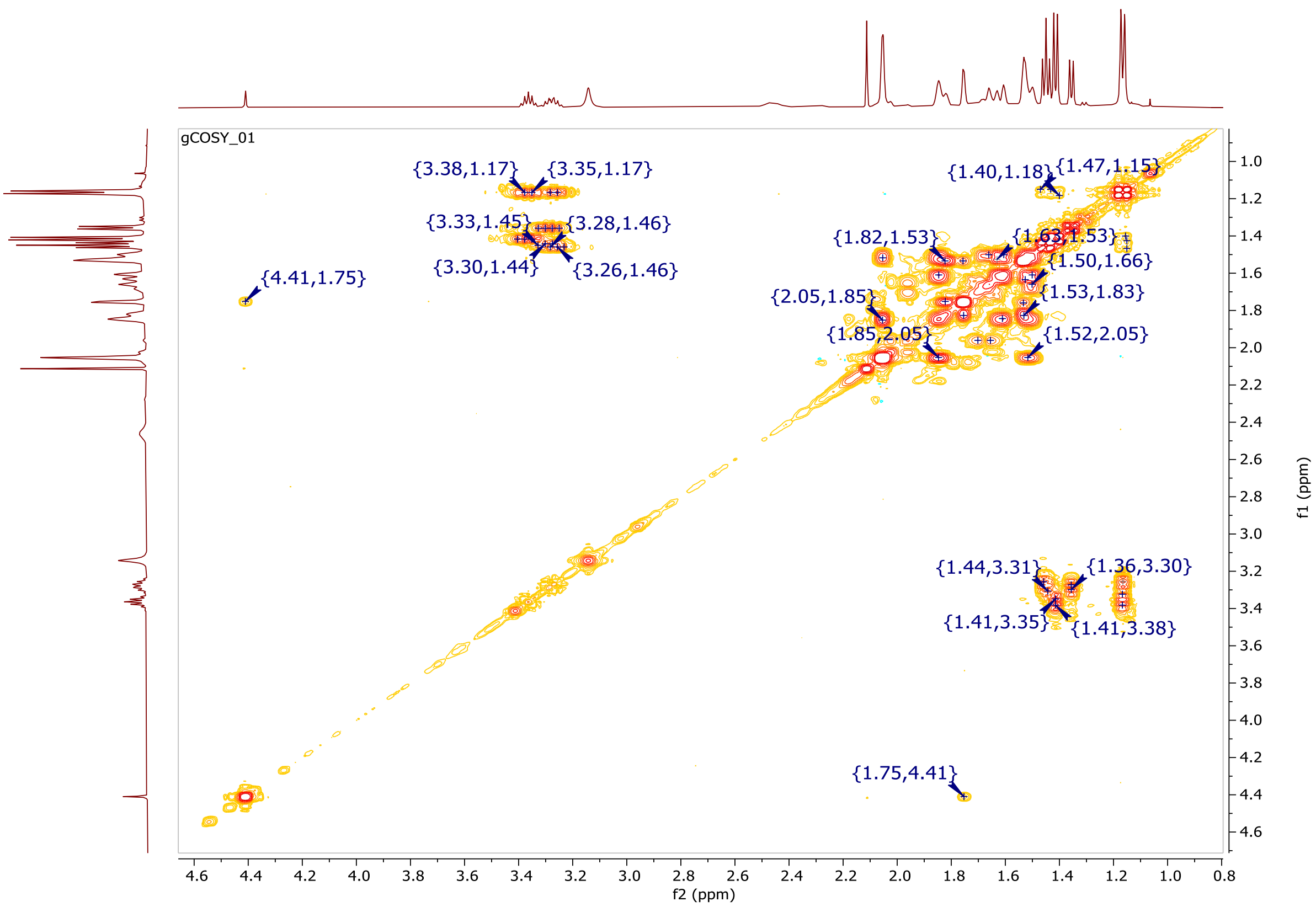

Figure S180: Expanded ^{1}H-^{1}H COSY NMR (500 MHz, C_6D_6)

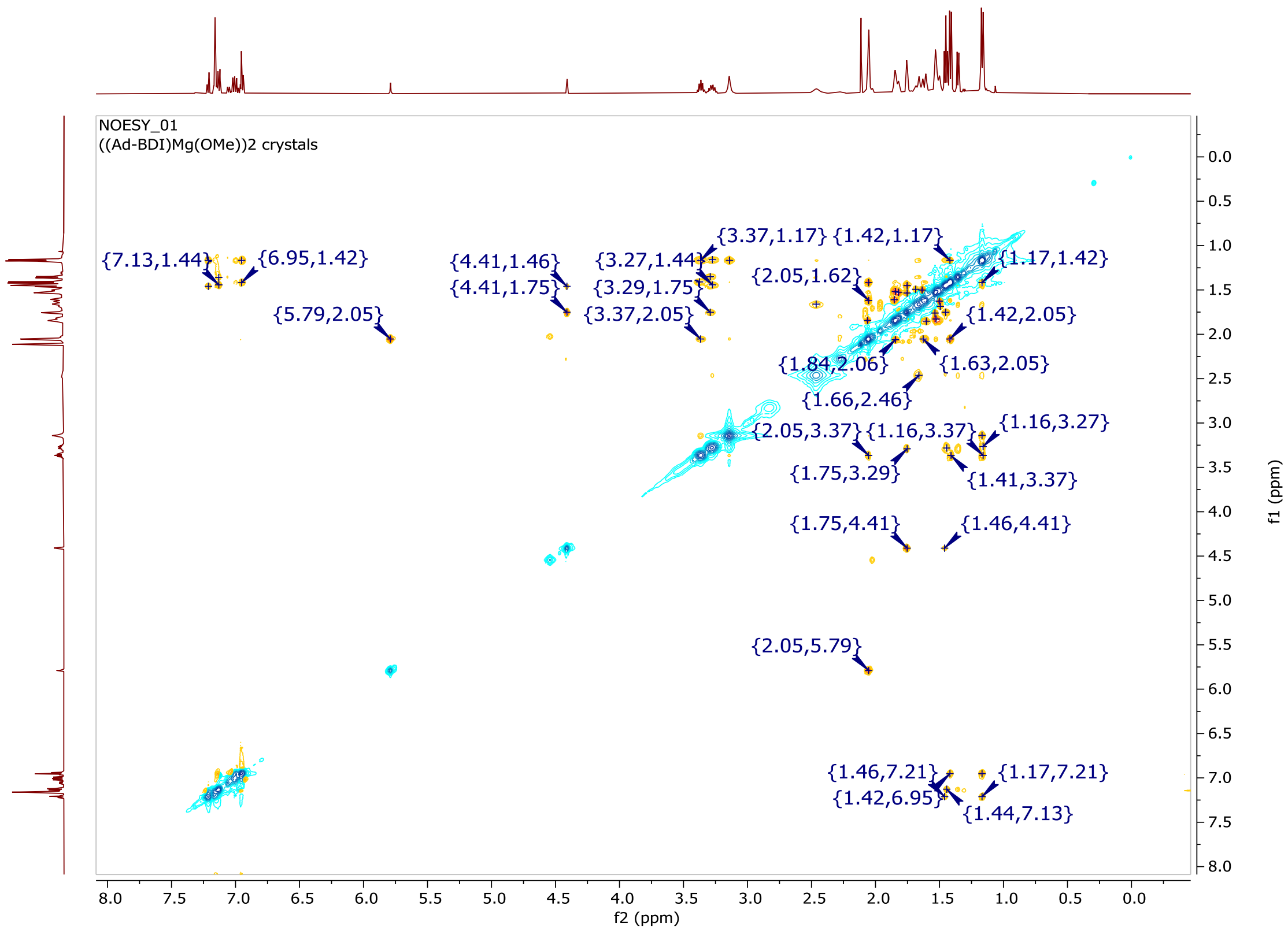

Figure S181: ^{1}H-^{1}H NOESY NMR (500 MHz, C_6D_6)

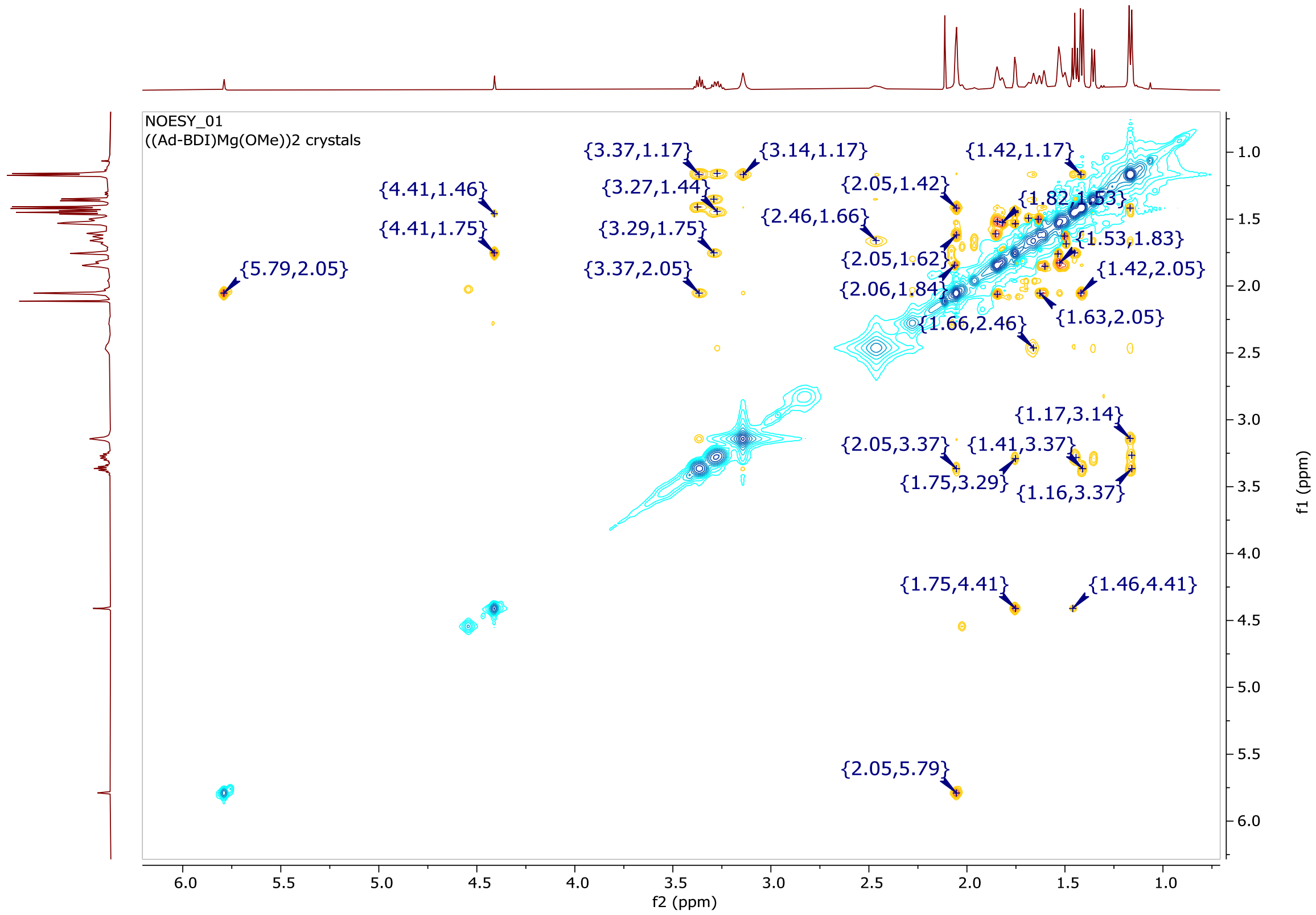

Figure S182: Expanded ^{1}H-^{1}H NOESY NMR (500 MHz, C_6D_6)

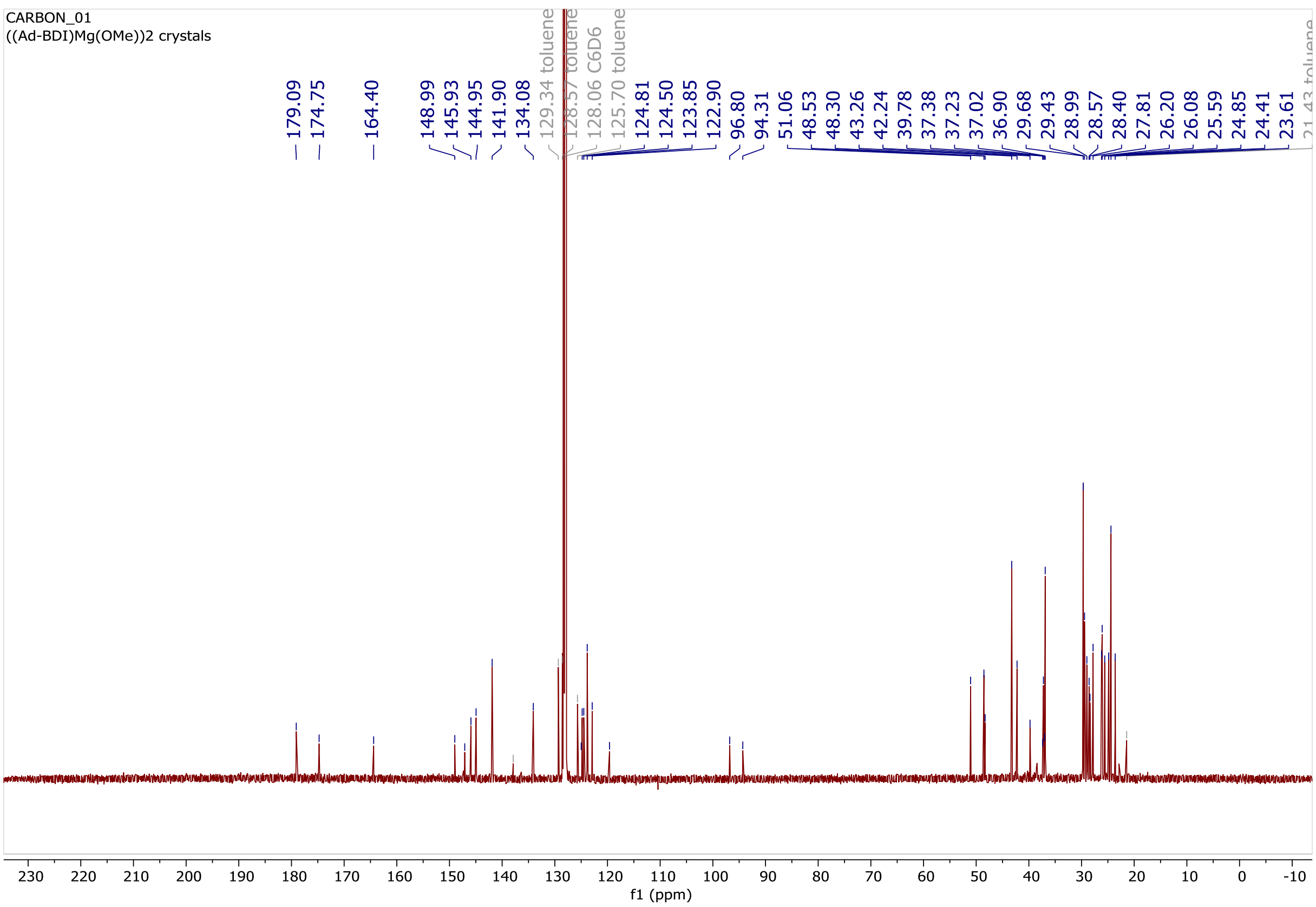

Figure S183: $^{13}C\{^{1}H\}$ NMR (126 MHz, C_6D_6)

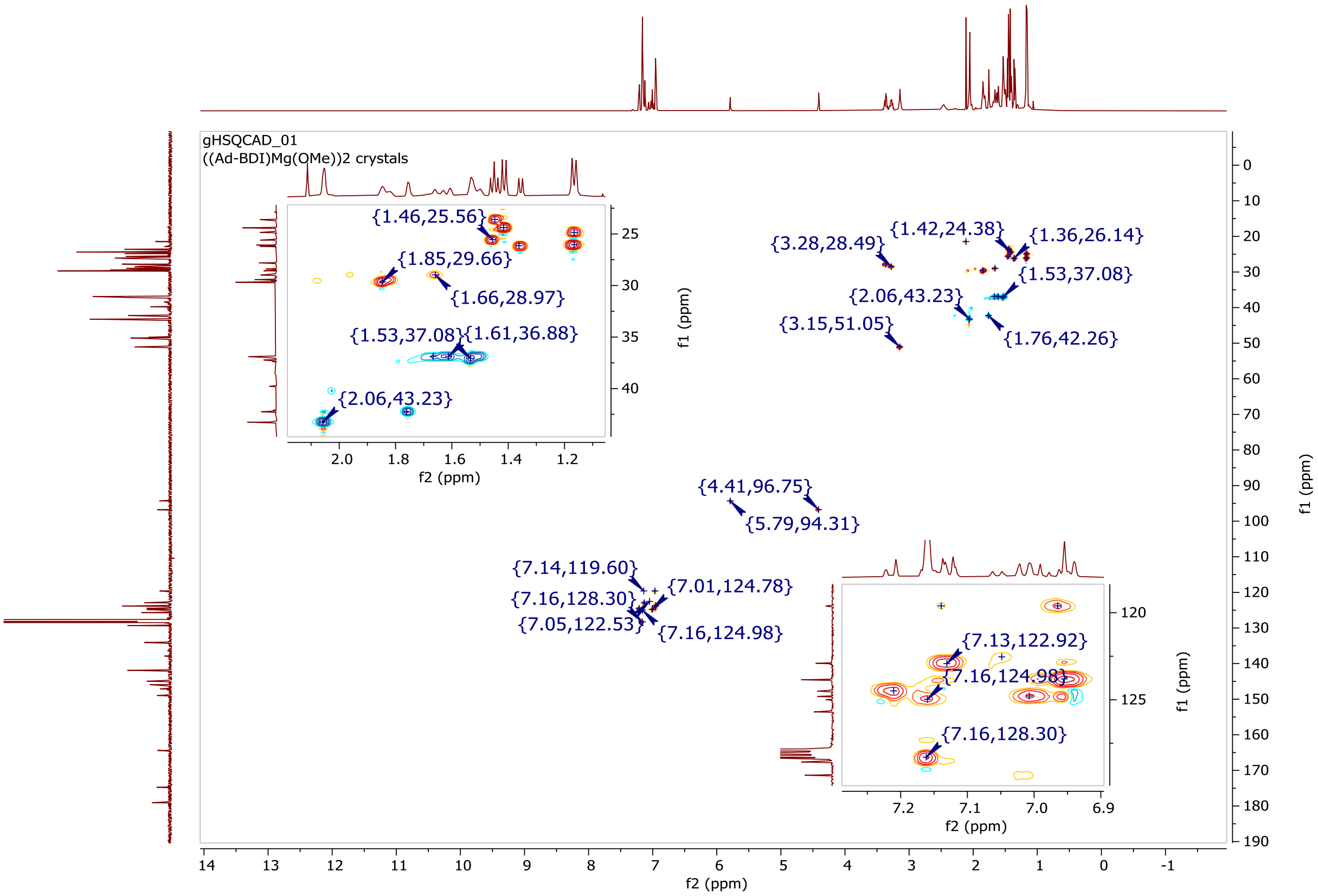

Figure S184: Edited ^{13}C-^{1}H HSQC (126 MHz, C_6D_6) Blue = CH_2, red = CH, CH_3)

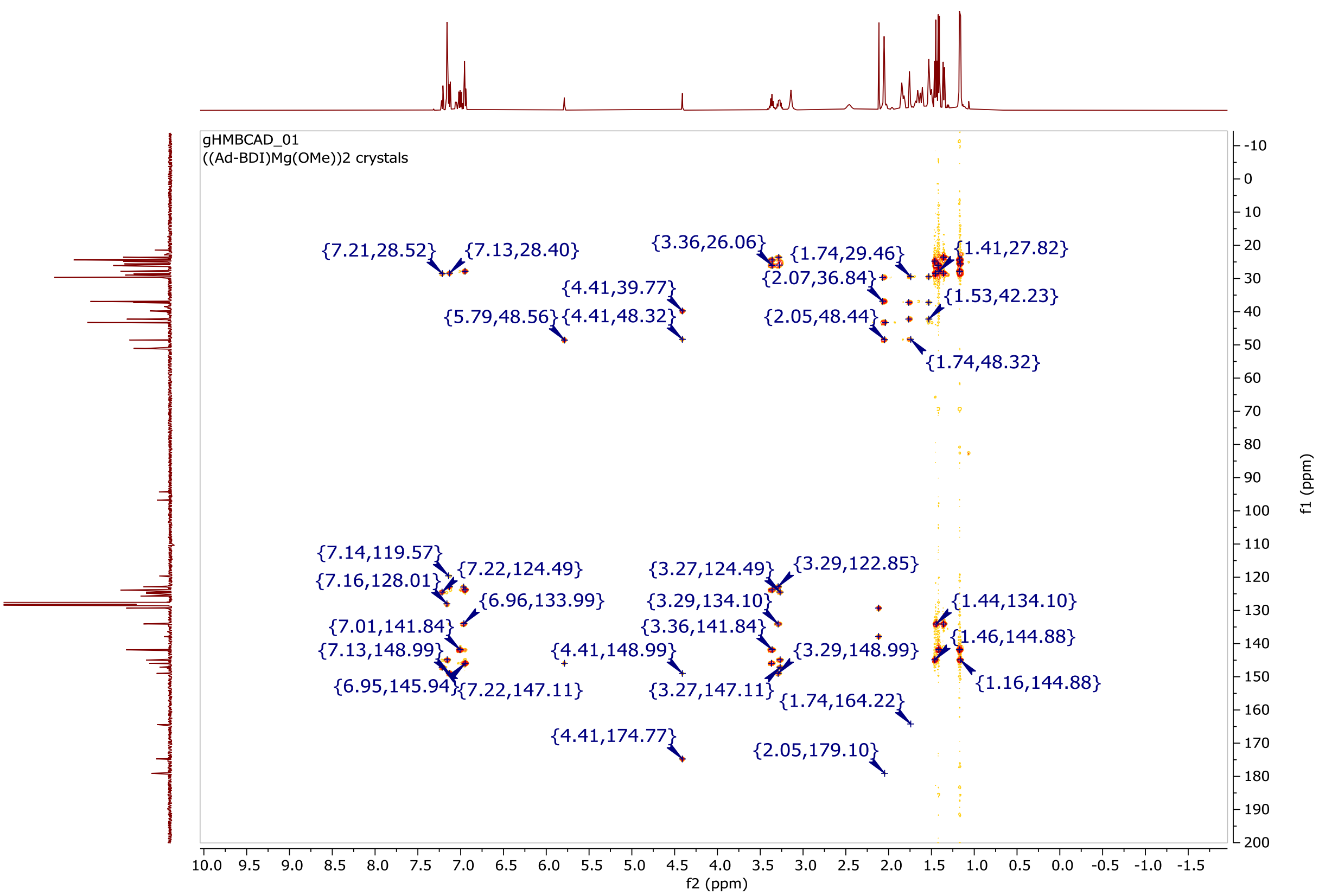

Figure S185: ^{13}C-^{1}H HMBC (126 MHz, C_6D_6).

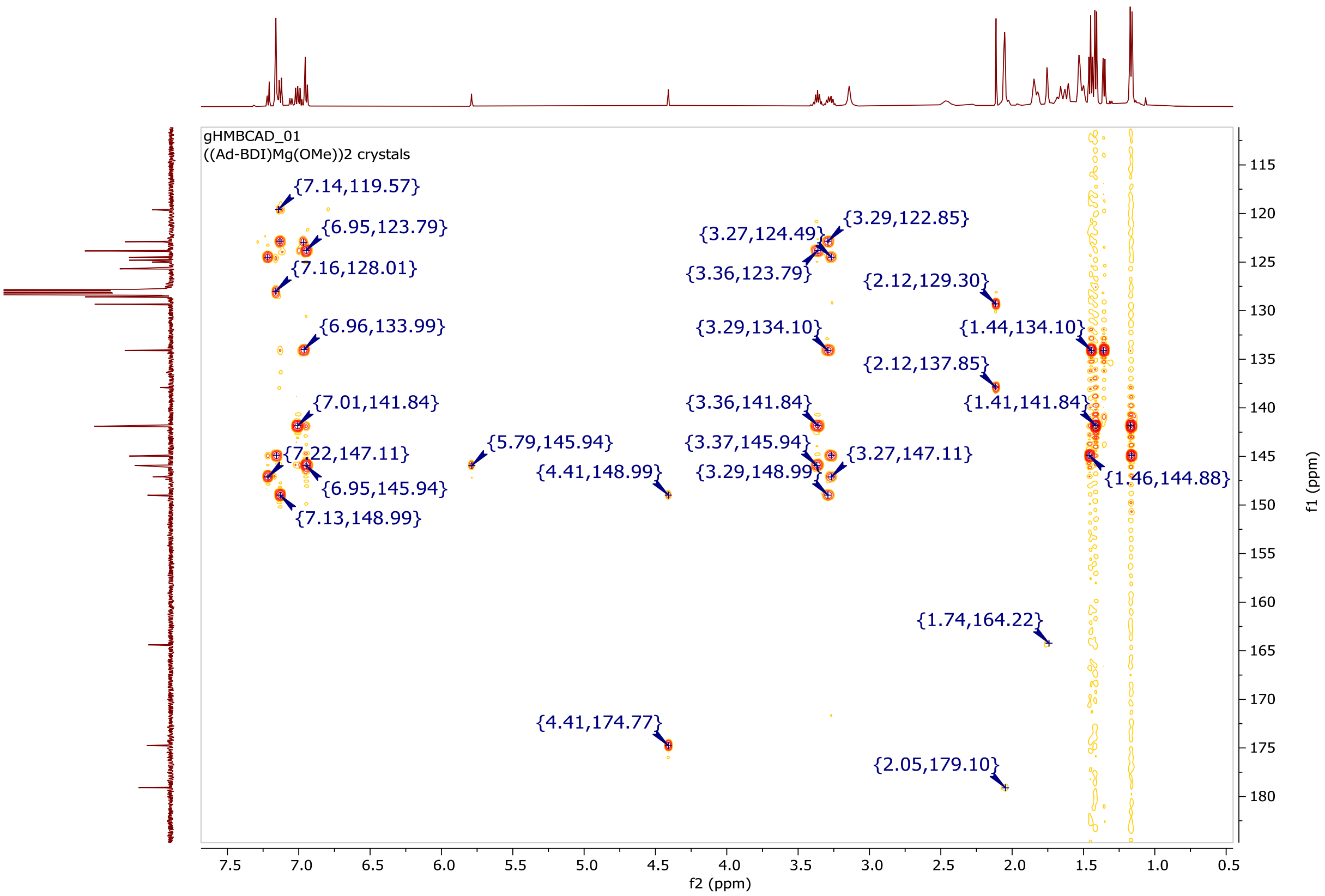

Figure S186: Expanded ^{13}C-^{1}H HMBC (126 MHz, C_6D_6).

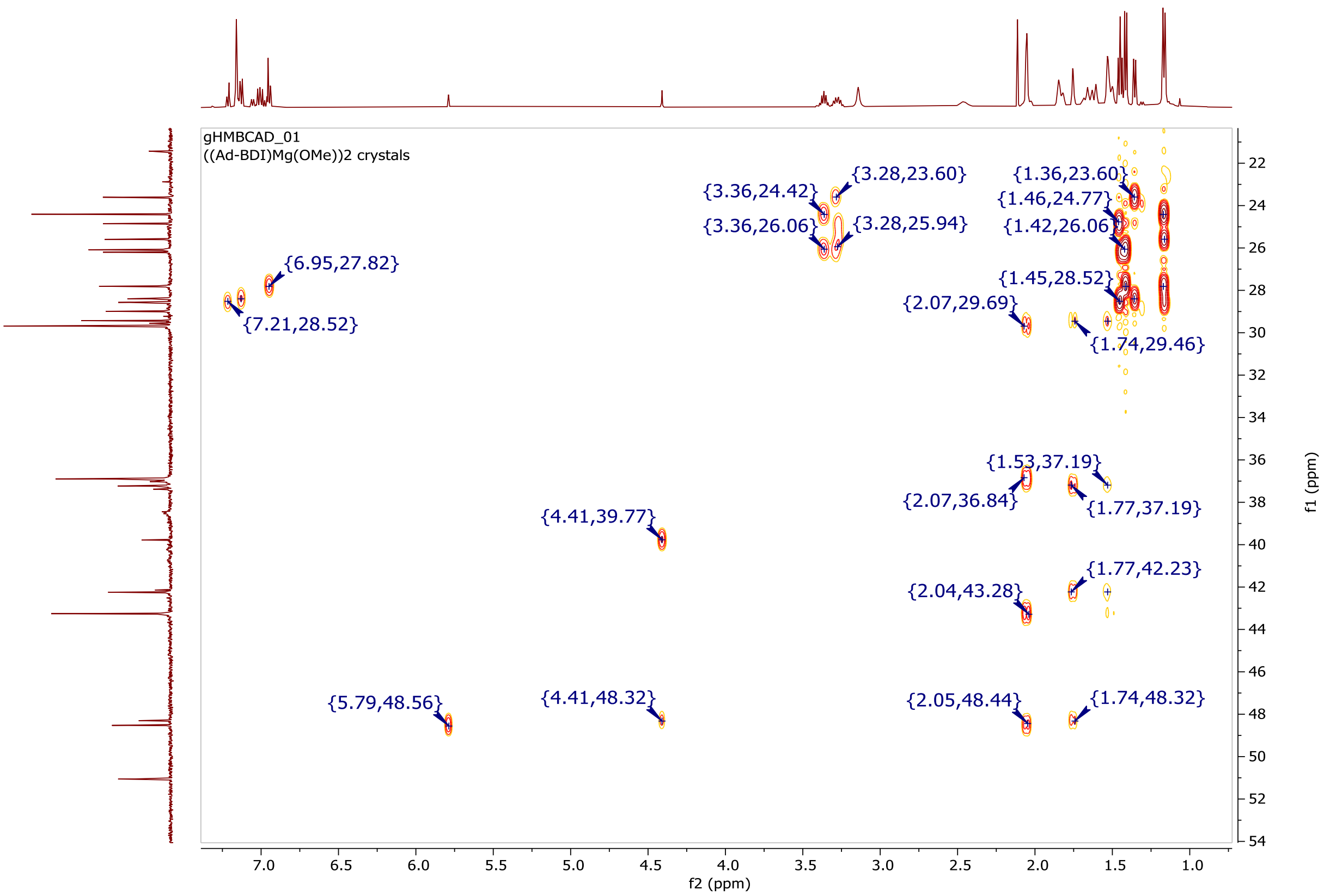

Figure S187: Expanded ^{13}C-^{1}H HMBC (126 MHz, C_6D_6).

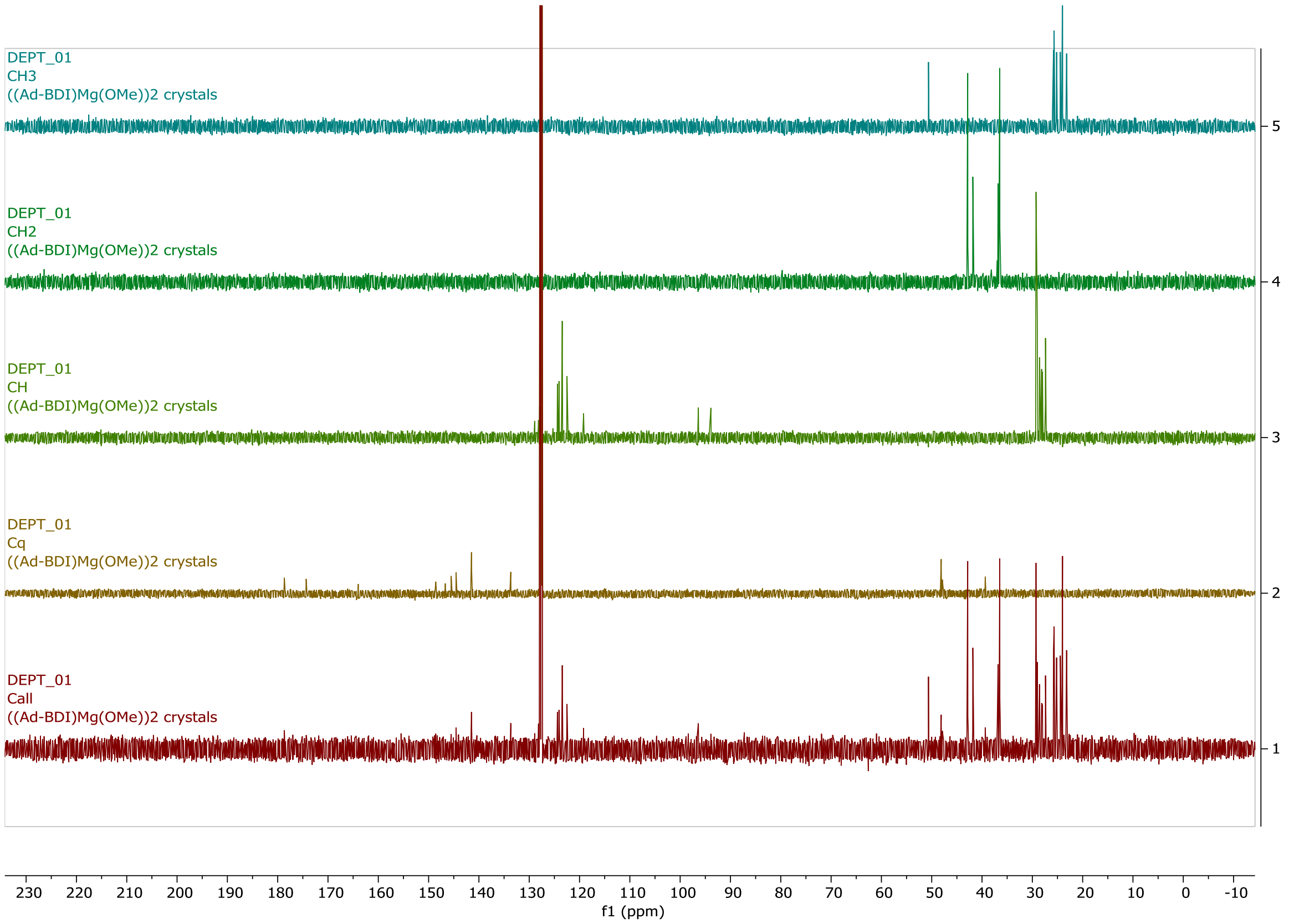

Figure S188: $^{13}C\{^{1}H\}$ DEPT plot (126 MHz, C_6D_6) from top to bottom: CH_3, CH_2, CH, Cq, all C.

2.22 Synthesis and characterisation of [(^{Ad}BDI)Mg(μ-OMe)$_2$Bpin] (12)

To a C_6D_6 solution (0.6 ml) of [(^{Ad}BDI)Mg(^{n}Bu)] (**1^{Ad}**, 23 mg, 0.031 mmol) in a J. Young's NMR tube was added 10 µl MeOBpin (0.062 mmol, 2 equiv.), resulting in the immediate formation of a bright yellow solution. Analysis of the reaction mixture by ^{1}H and ^{11}B NMR spectroscopy indicated stoichiometric conversion of the starting magnesium complex to **12** under elimination of ^{n}BuBpin. Volatiles were removed under vacuum. Recrystallisation of the crude product from *n*-hexane/toluene at -35°C provided bright yellow single crystals of **12**, suitable for X-ray diffraction analysis, in 15 mg yield, 54%.

Analysis calculated for $C_{55}H_{83}BMgN_2O_4$: C, 75.81; H, 9.60; N, 3.21%. Found: C, 75.36; H, 9.96; N, 2.78%.

^{1}H NMR (400 MHz, C_6D_6) δ 7.08 (m, 6H, NC$_6$*H*$_3$-*i*Pr$_2$), 5.98 (s, 1H, *H*CC(Ndipp)), 3.32 (hept, *J* = 6.6 Hz, 4H, *H*C(CH$_3$)$_2$), 3.13 (s, 6H, OC*H*$_3$), 2.04 (d, *J* = 3.0 Hz, 12H, ^{Ad}CC*H*$_2$CH), 1.85 (br, 6H, ^{Ad}C*H*), 1.59 (dm, unresolved, *J* = 11.8 Hz, 6H, ^{Ad}CHC*H*$_2$CH), 1.52 (dm, unresolved, *J* = 11.9 Hz, 6H, ^{Ad}CHC*H*$_2$CH), 1.45 (d, *J* = 6.7 Hz, 12H, HC(C*H*$_3$)$_2$), 1.33 (d, *J* = 6.7 Hz, 12H, HC(C*H*$_3$)$_2$), 1.25 (s, 12H, OC(C*H*$_3$)$_2$).

^{11}B NMR (128 MHz, C_6D_6) δ 7.28.

^{13}C NMR (101 MHz, C_6D_6) δ 179.73 (N=*C*), 145.76 (*ipso*-N*C*$_6$H$_3$-*i*Pr$_2$), 140.78 (*ortho*-N*C*$_6$H$_3$-*i*Pr$_2$), 124.02 (*para*-N*C*$_6$H$_3$-*i*Pr$_2$), 123.89 (*meta*-N*C*$_6$H$_3$-*i*Pr$_2$), 95.47 (H*C*C(Ndipp)), 78.72 (O*C*(CH$_3$)$_2$), 50.10 (O*C*H$_3$), 47.38 (NC^{Ad}*C*), 43.01 (^{Ad}C*C*H$_2$CH), 36.90 (^{Ad}CH*C*H$_2$CH), 29.45 (^{Ad}*C*H), 28.48 (H*C*(CH$_3$)$_2$), 27.83 (OC(*C*H$_3$)$_2$), 26.09 (HC(*C*H$_3$)$_2$), 23.99 (HC(*C*H$_3$)$_2$).

In situ NMR spectra.

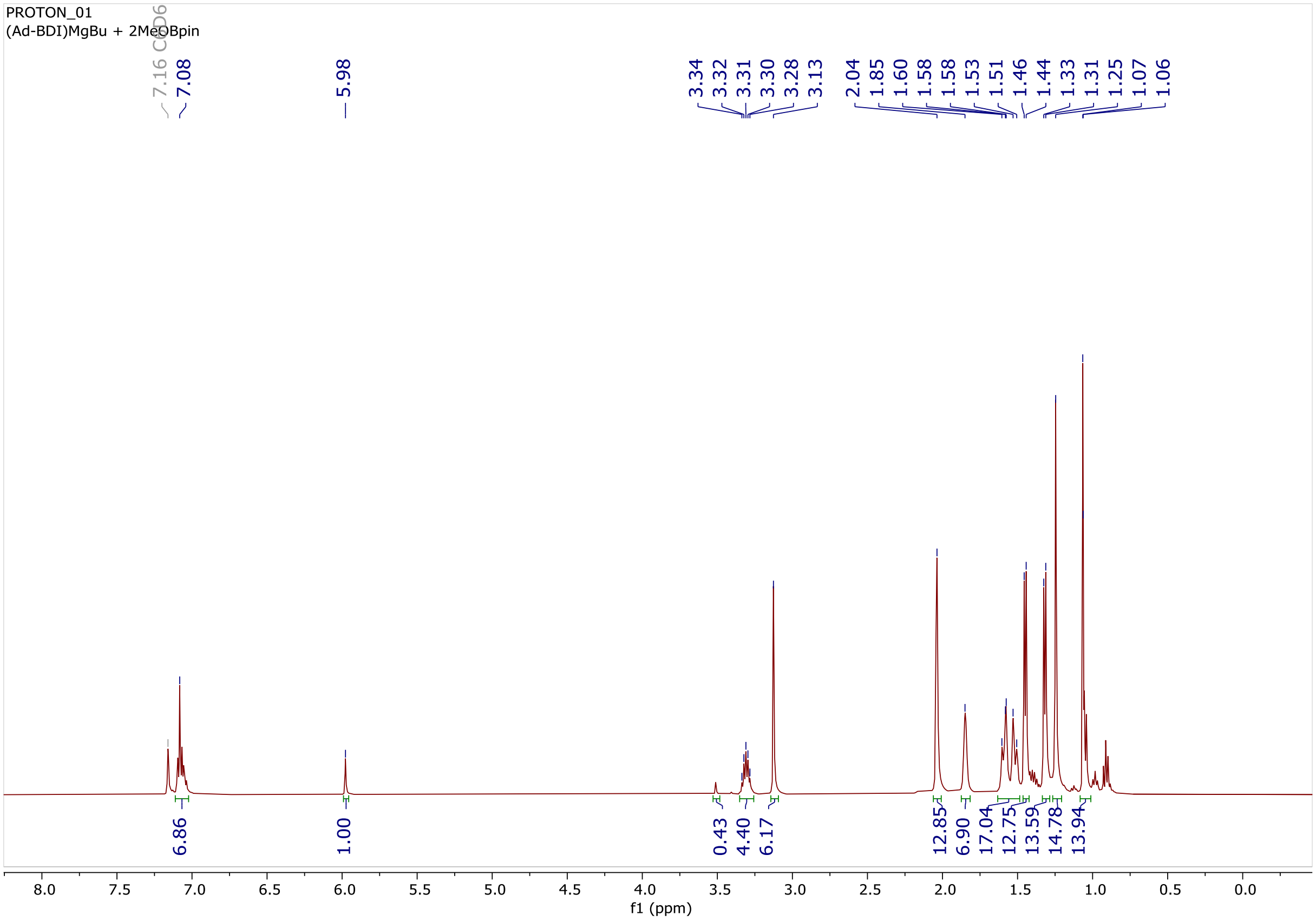

Figure S189: ^{1}H (500 MHz, C_6D_6)

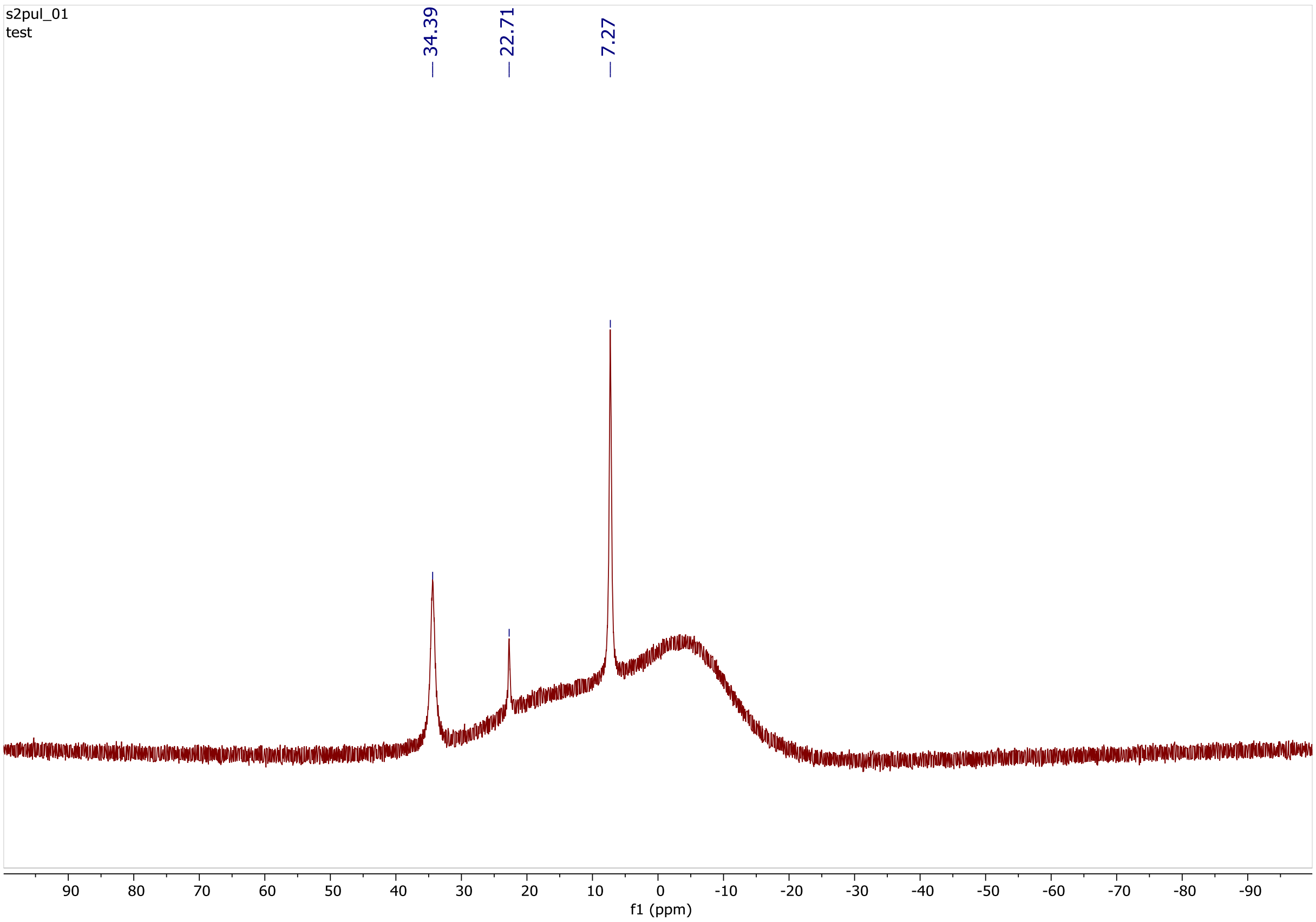

Figure S190: $^{11}B\{^{1}H\}$ (160 MHz, C_6D_6).

NMR of isolated crystals

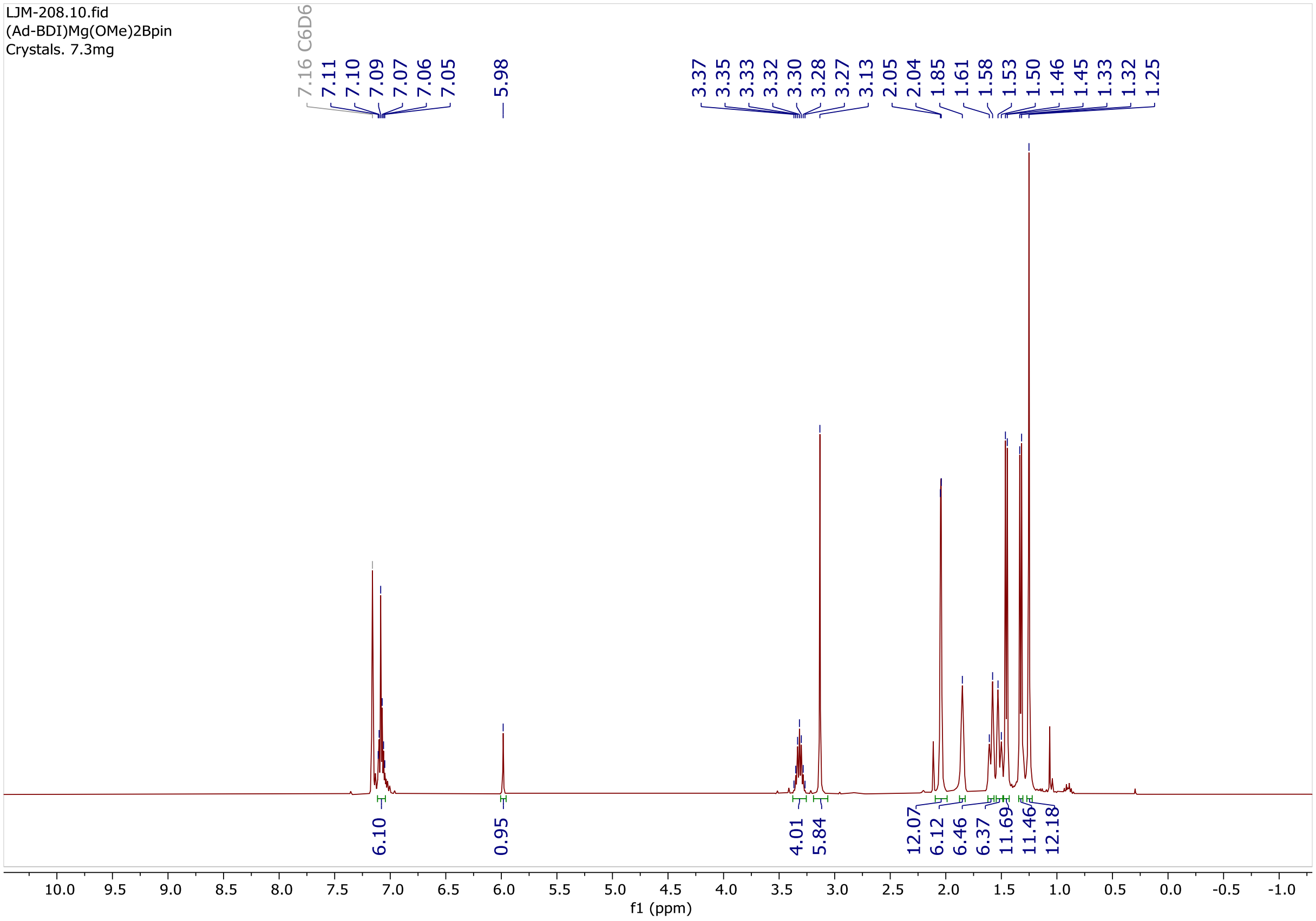

Figure S191: ^{1}H NMR (400 MHz, C_6D_6) spectrum of isolated crystals

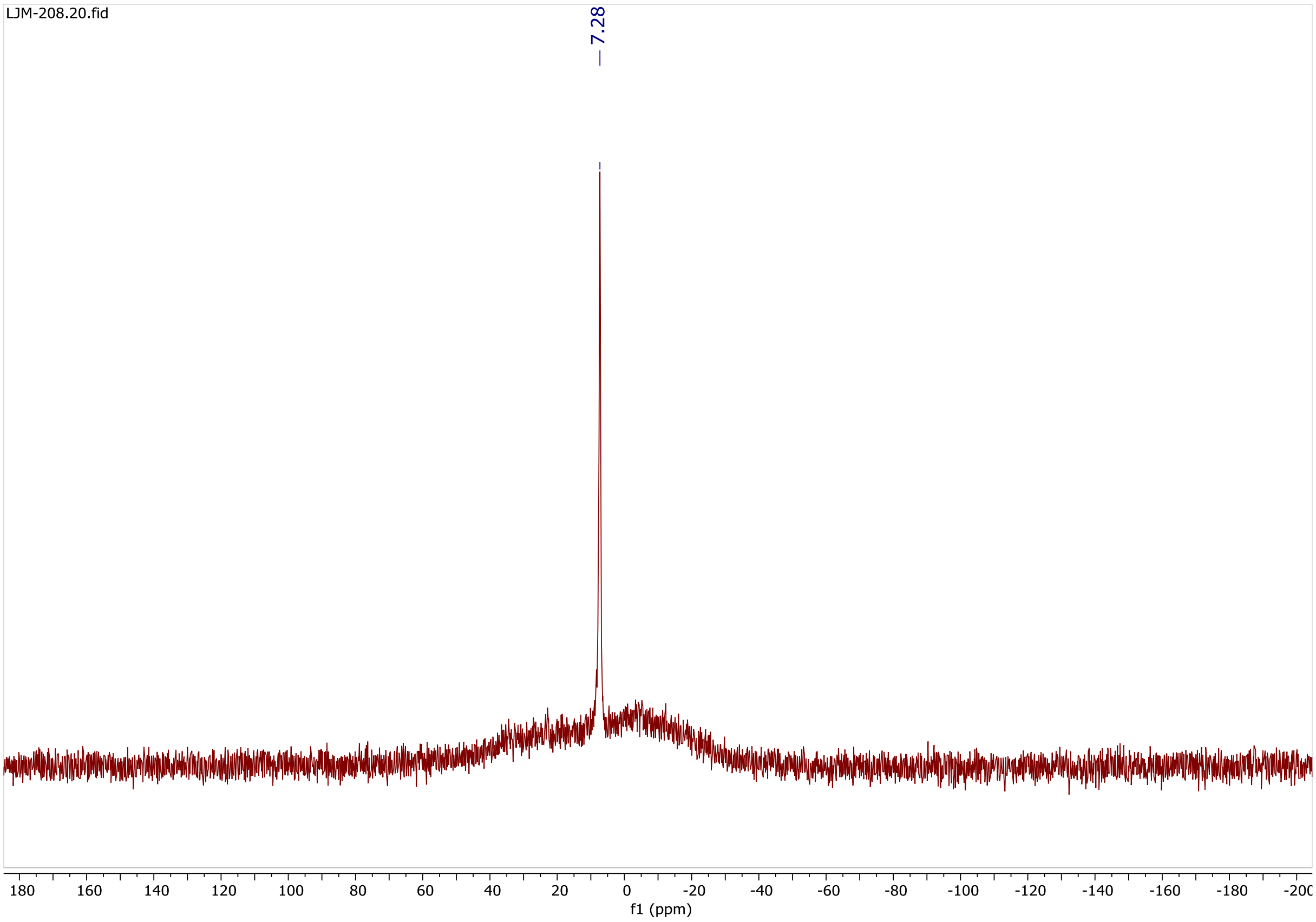

Figure S192: $^{11}B\{^{1}H\}$ NMR (128 MHz, C_6D_6).

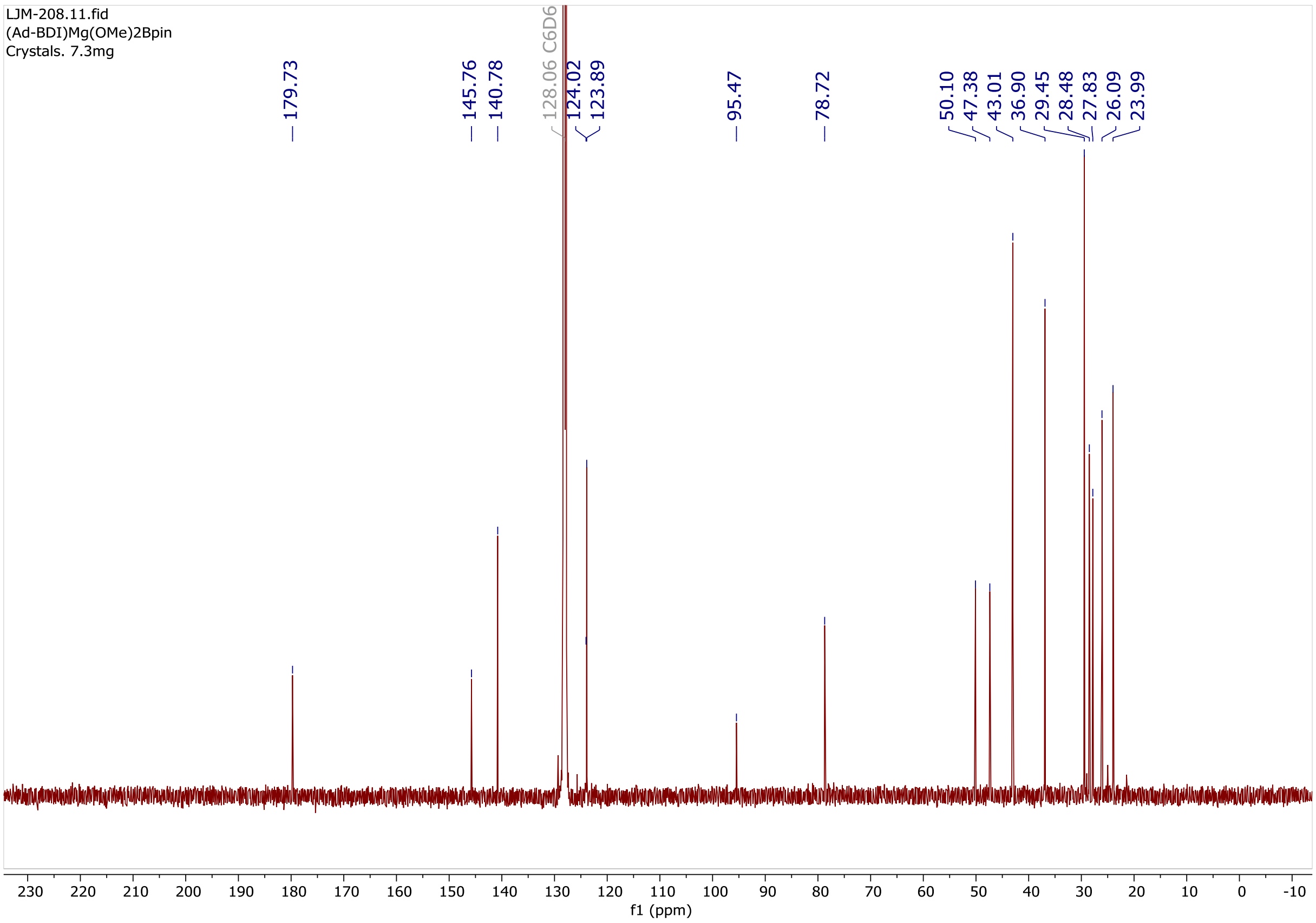

Figure S193: $^{13}C\{^{1}H\}$ NMR (101 MHz, C_6D_6)

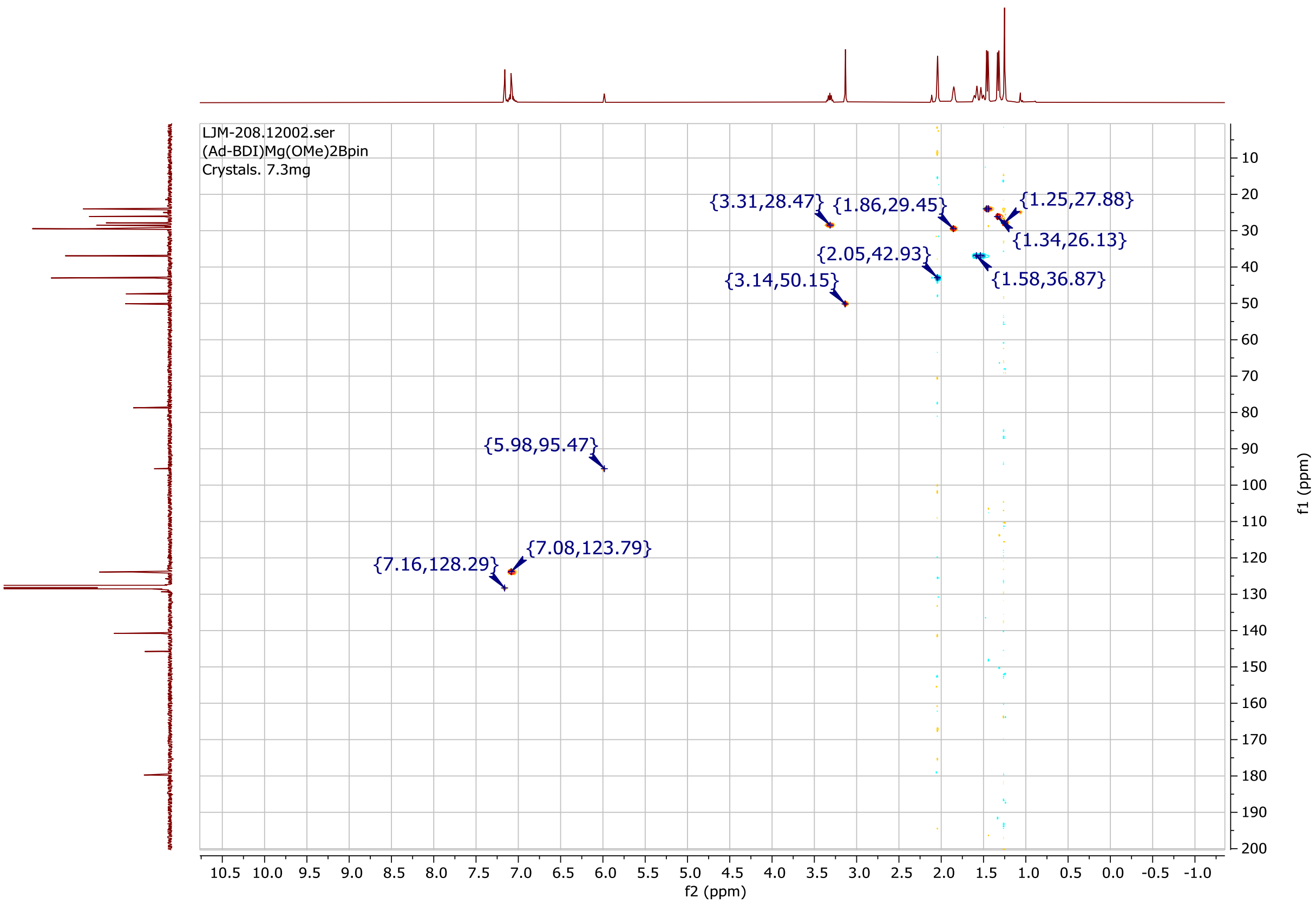

Figure S194: ^{13}C-^{1}H HSQC NMR (101 MHz, C_6D_6)

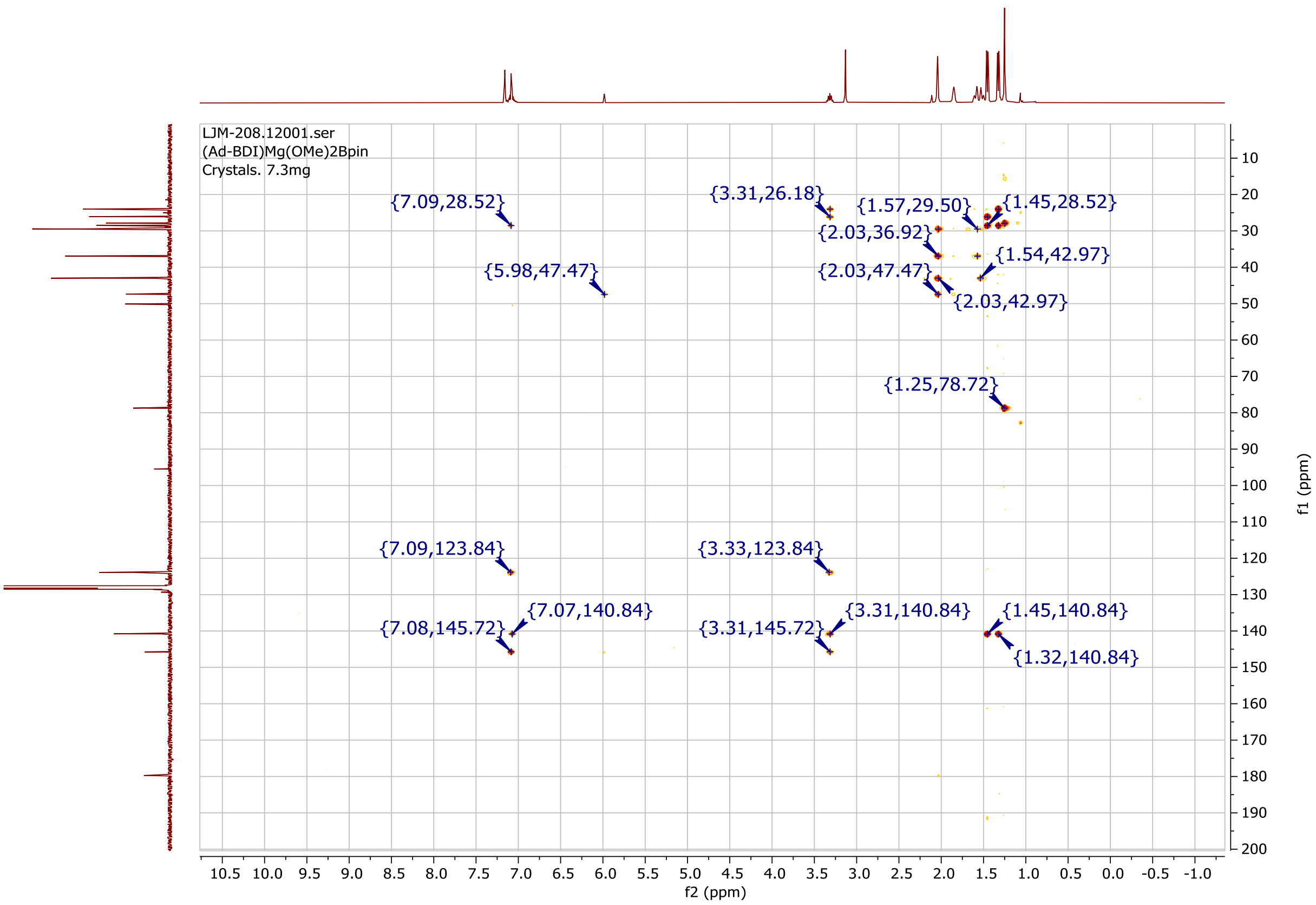

Figure S195: ^{13}C-^{1}H HMBC NMR (101 MHz, C_6D_6)

2.23 Attempted uncatalyzed reaction of [(^{Ad}BDI)MgPh] (3^{Ad}) with bromobenzene

A C_6H_6 solution (0.6 ml) of [(^{Ad}BDI)Mg^{n}Bu] (**1**^{Ad}, 13 mg, 0.018 mmol) was heated at 100°C in a sealed J. Young's NMR tube for 8 days to yield **3**^{Ad}. Volatiles were removed under vacuum, then the residue was redissolved in C_6D_6 and 2 µl bromobenzene (0.02 mmol) was added. After heating at 100°C for two days, biphenyl was not observed in the ^{1}H NMR spectrum, bromobenzene and **3**^{Ad} were not consumed, but the MgC_6H_5 resonances decreased in intensity due to exchange with C_6D_6 and formation of [(^{Ad}BDI)Mg(C_6D_5)] (**3**^{Ad}-d_5).

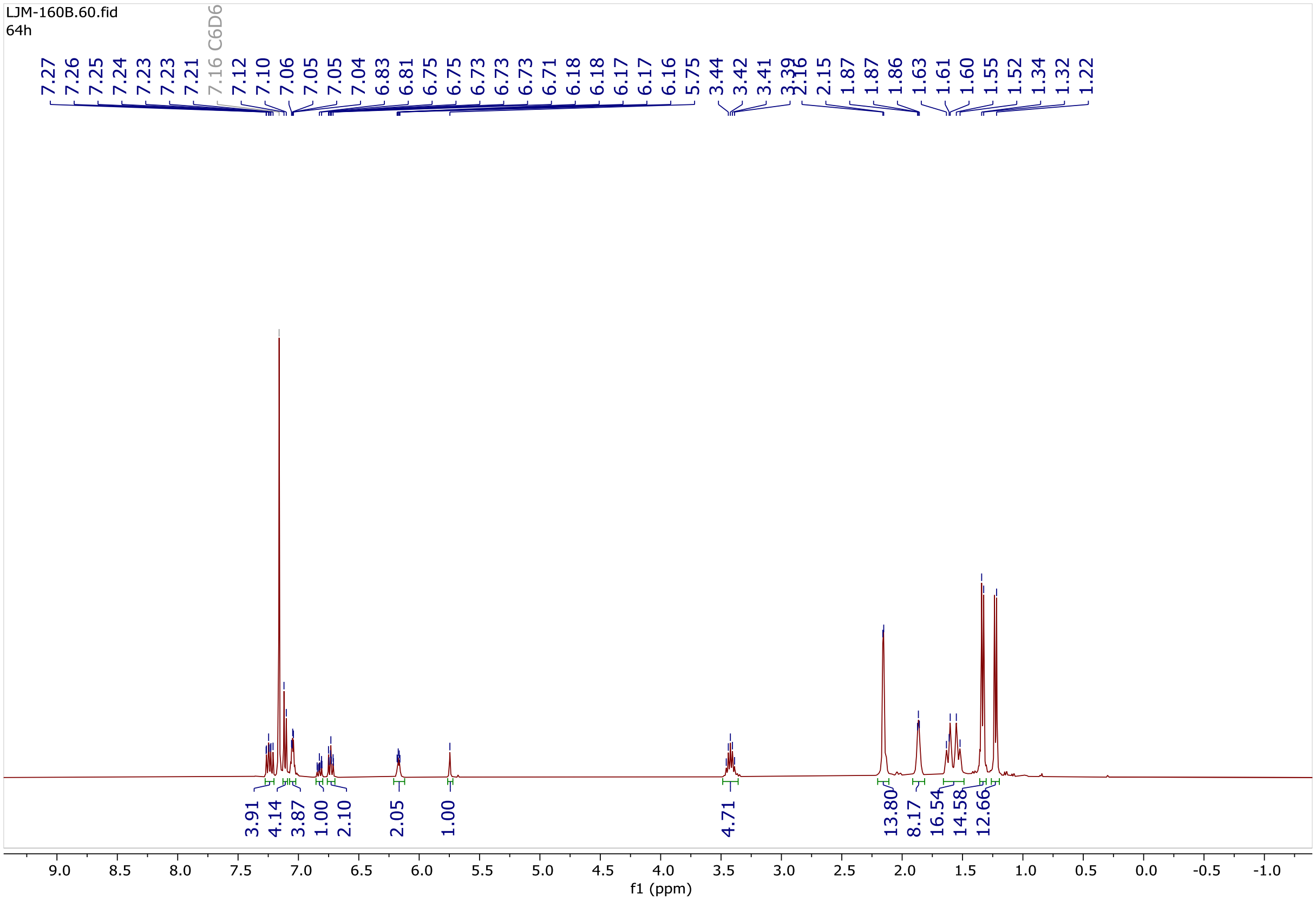

Figure S196: ^{1}H NMR (400 MHz, C_6D_6) before addition of bromobenzene.

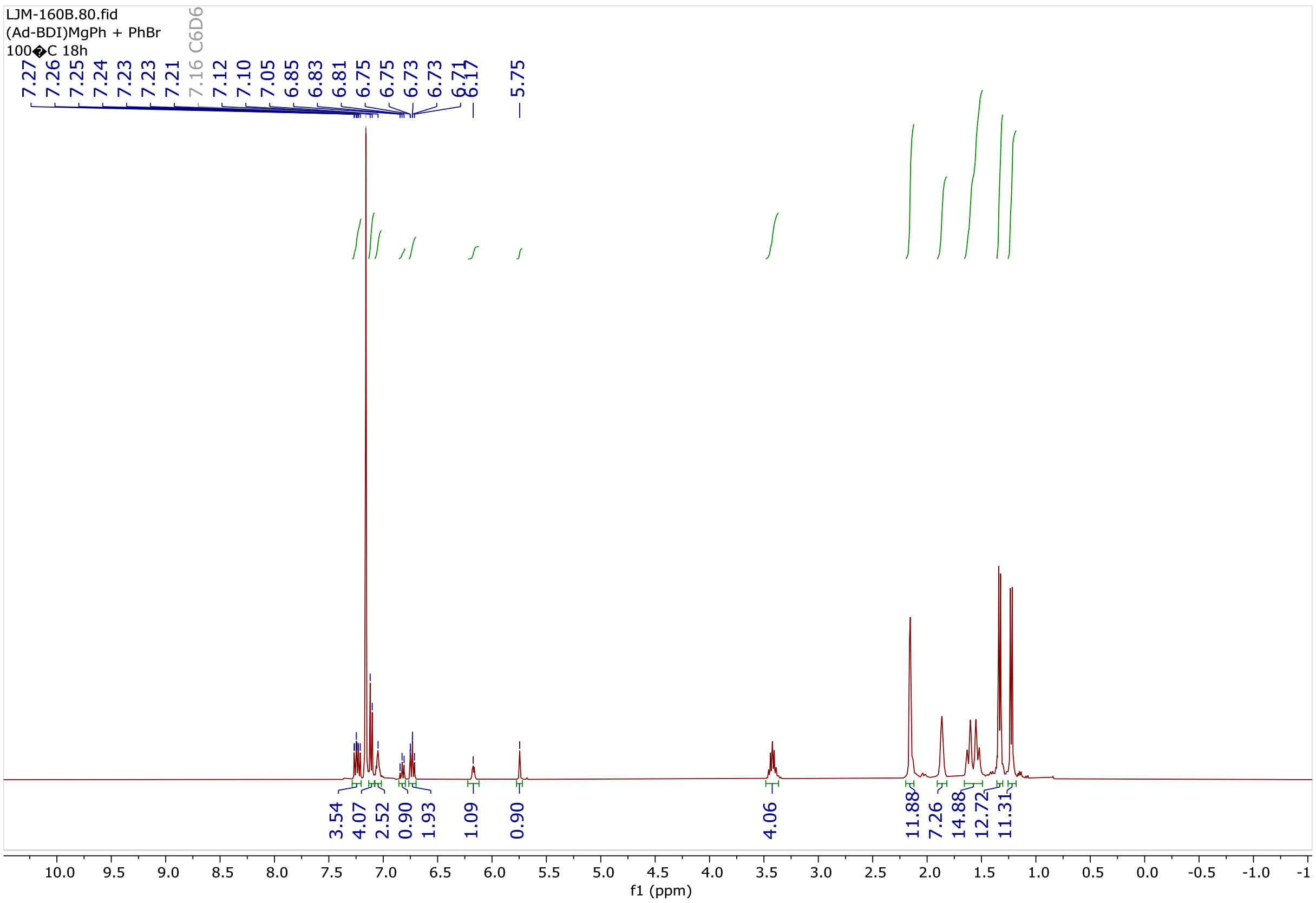

Figure S197: *in situ* ^{1}H NMR (400 MHz, C_6D_6) after 2 days at 100°C.

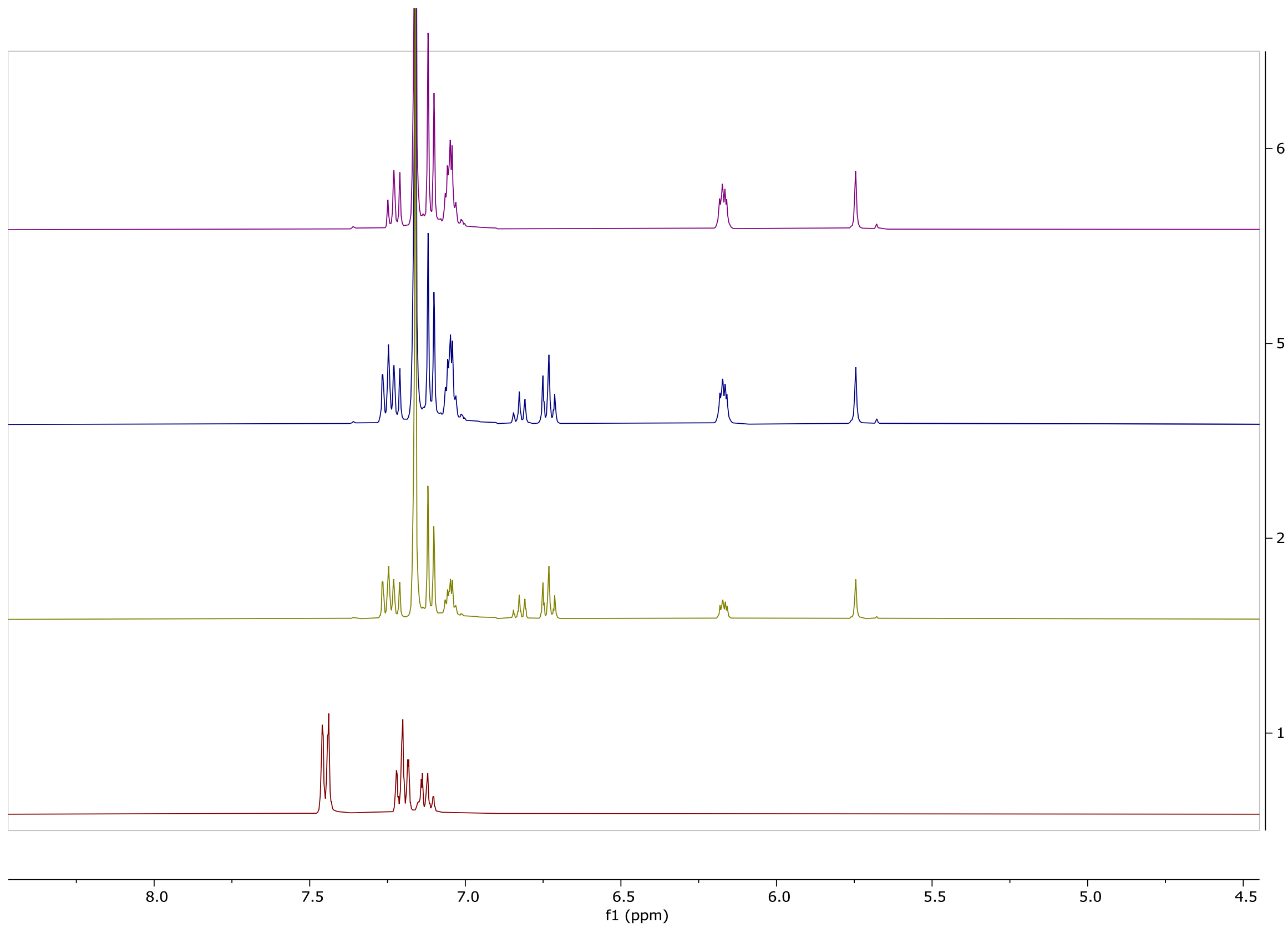

Figure S198: Stacked ^{1}H NMR spectra (400 MHz, C_6D_6) highlighting lack of formation of biphenyl from attempted reaction of **3**^{Ad} with bromobenzene and decrease in intensity of MgC_6H_5 resonances due to exchange with C_6D_6. From top to bottom: **3**^{Ad} before addition of bromobenzene, after addition of bromobenzene, after 2 days at 100°C, pure biphenyl.

2.24 Procedure for telescoped Kumada coupling

*2.24.1 General procedure for cross-coupling via [(^{Ad}BDI)MgPh] (**3**^{Ad})*

A J. Young's NMR tube wash charged with [(^{Ad}BDI)Mg^{n}Bu] (**1**^{Ad}) and 0.6 ml benzene or benzene-*d*$_6$ was added. The NMR tube was sealed, then heated to 100°C for 1 week. Where *proteo*-benzene was used, all volatiles were removed under vacuum and the product redissolved in benzene-*d*$_6$ (0.6 ml). Analysis by ^{1}H NMR at this stage indicated quantitative formation of **3**^{Ad}. The benzene-*d*$_6$ solution was added to the relevant bromoarene and hexamethylbenzene as an internal standard. The reaction mixture was analysed by ^{1}H NMR spectroscopy, then added to [$Pd(PCy_3)_2$]. The NMR tube was sealed and heated to 60°C. [(^{Ad}BDI)MgBr] (**13**) was identified to form by ^{1}H NMR spectroscopy, and by single crystal X-ray diffraction of crystals deposited from the cooled reaction mixture after cross-coupling **3**^{Ad} with bromobenzene (see section 2.22). Products were identified and quantified by *in situ* ^{1}H NMR spectroscopy of the reaction mixture and compared to a commercial sample were available. In each case, a 10 µl aliquot of the reaction mixture was diluted with 1 ml toluene, and the resulting solution analysed by GC-MS to corroborate the products identified by NMR spectroscopy.

2.24.2 Cross-coupling of benzene and bromobenzene to form biphenyl

General procedure using 39 mg **1**^{Ad} (0.053 mmol), 8.2 mg bromobenzene (0.052 mmol), 6.4 mg hexamethylbenzene (0.039 mmol), 1.4 mg [$Pd(PCy_3)_2$] (0.002 mmol, 4.2 mol%).

93% yield of biphenyl after 3 days at 60°C.

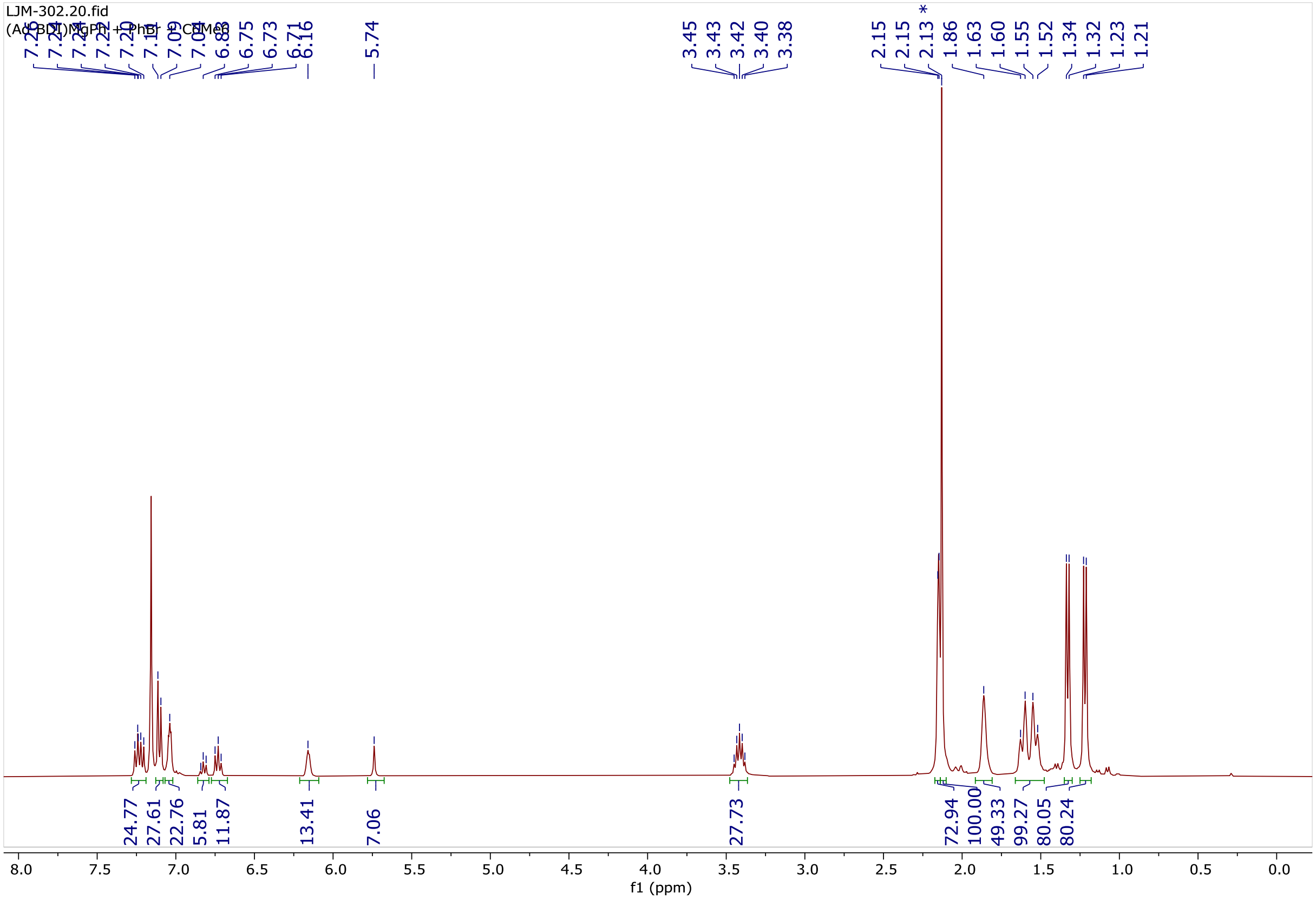

Figure S199: ^{1}H NMR (400 MHz, C_6D_6) before addition of [Pd(PCy$_3$)$_2$]. *hexamethylbenzene.

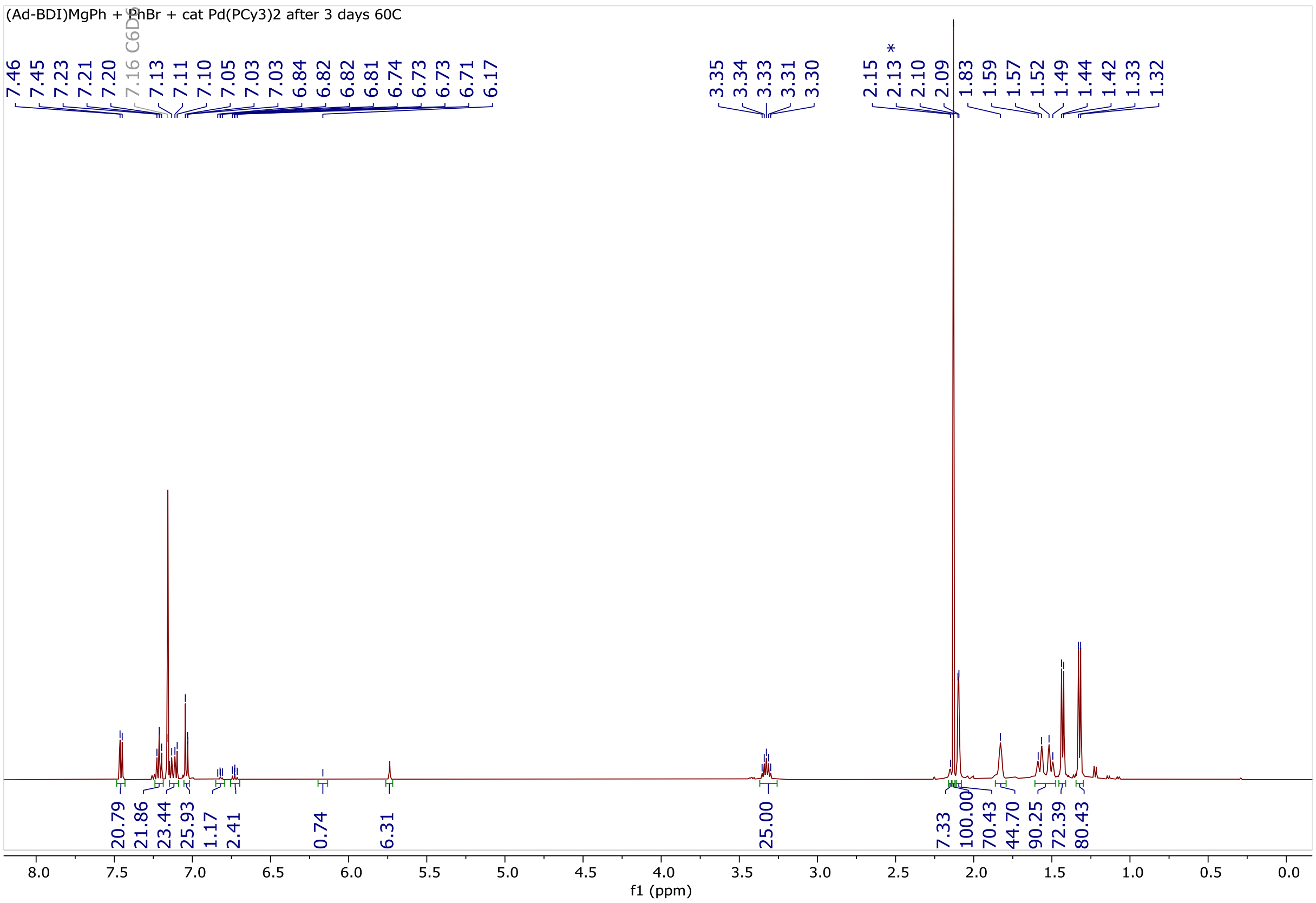

Figure S200: ^{1}H NMR (500 MHz, C_6D_6) after 3 days at 60°C. *hexamethylbenzene.

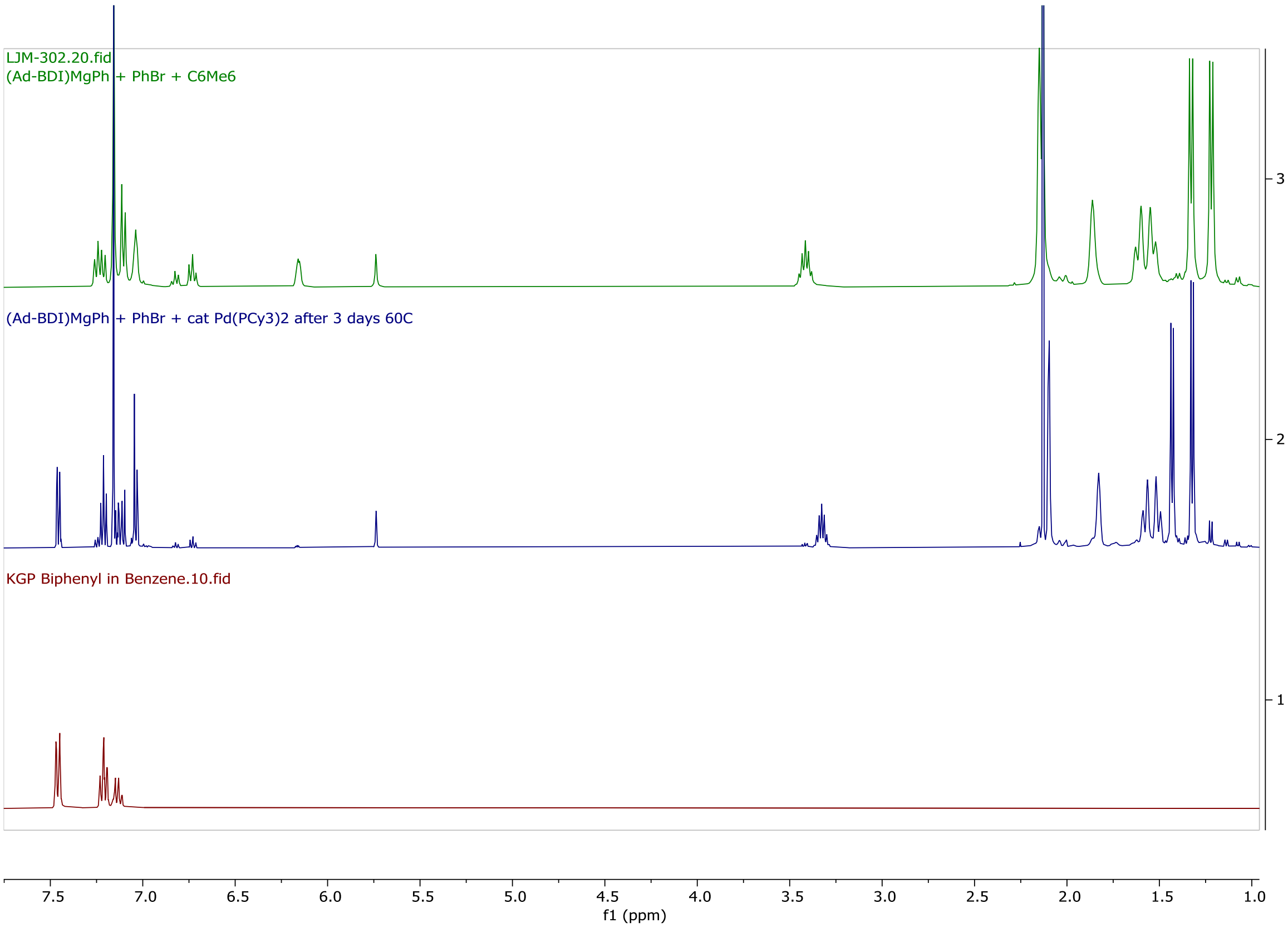

Figure S201: Stacked ^{1}H NMR spectra (C_6D_6) before addition of [Pd(PCy_3)$_2$] (top, 400 MHz), after 3 days at 60°C (centre, 500 MHz), and of a separate sample of pure biphenyl (bottom, 500 MHz).

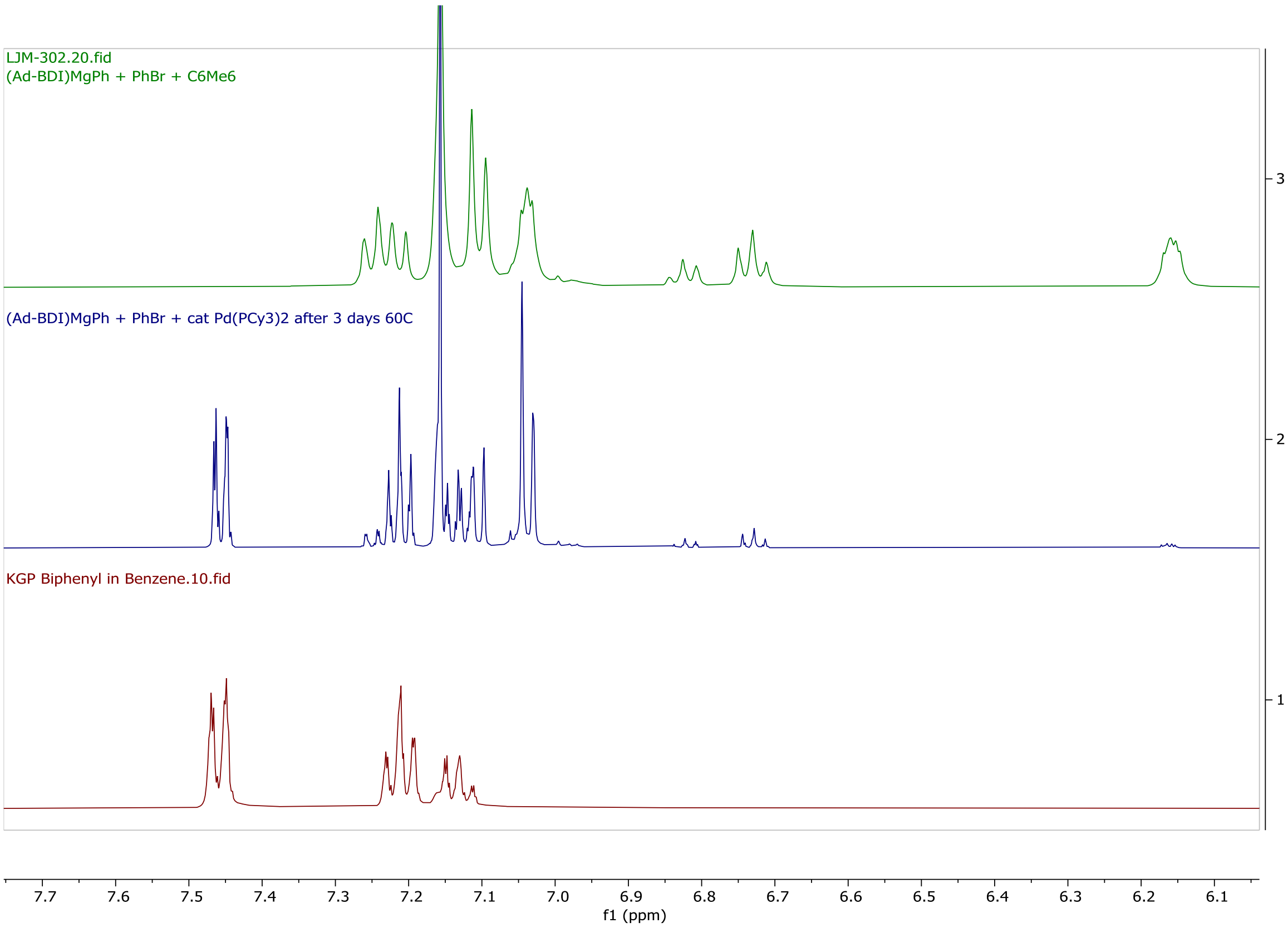

Figure S202: Expanded stacked ^{1}H NMR spectra (C_6D_6) before addition of [Pd(PCy_3)$_2$] (top, 400 MHz), after 3 days at 60°C (centre, 500 MHz), and of a separate sample of pure biphenyl (bottom, 500 MHz) highlighting formation of biphenyl and consumption of **3**^{Ad}.

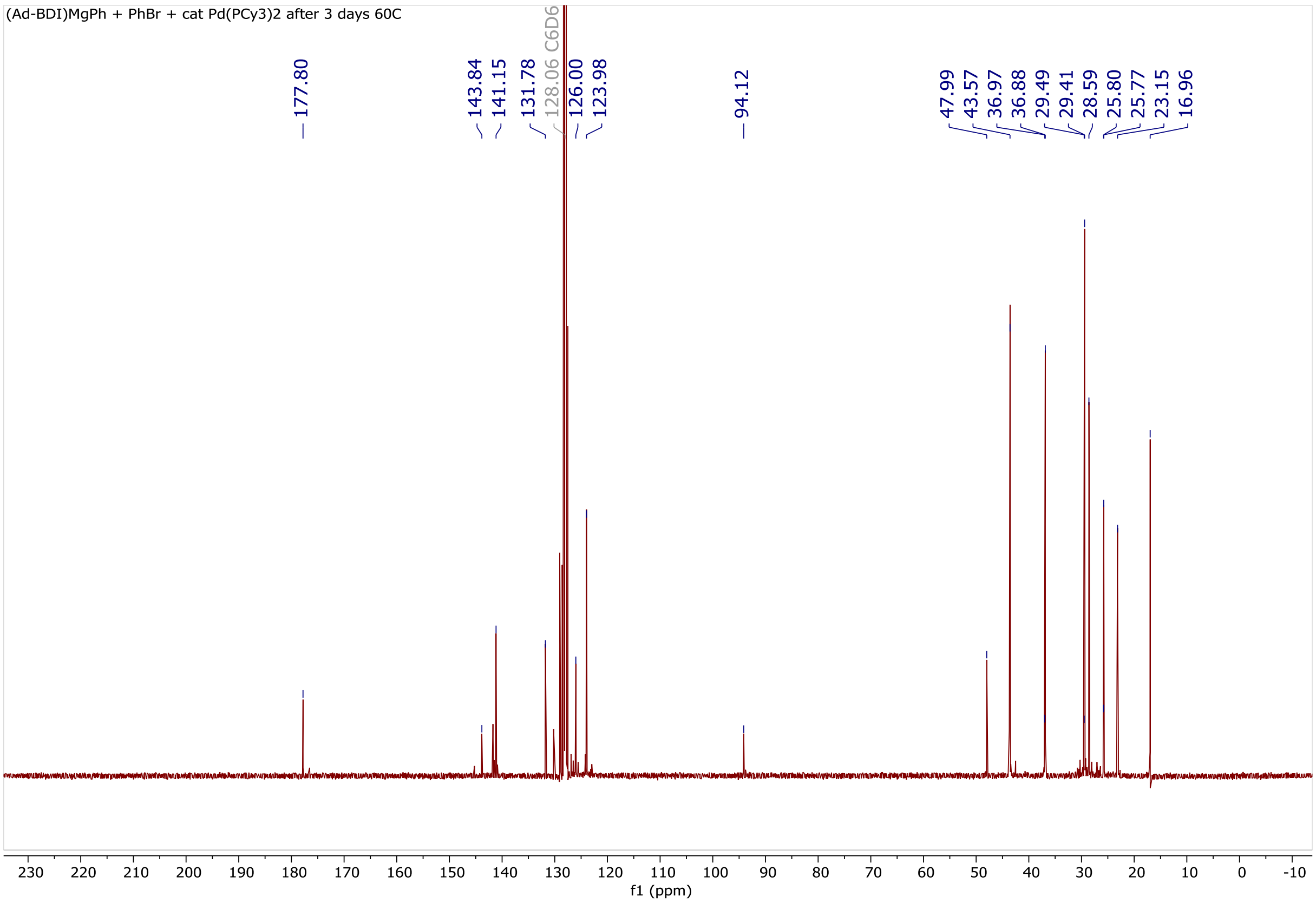

Figure S203: $^{13}C\{^1H\}$ NMR spectrum (126 MHz, C_6D_6) after 3 days at 60°C.

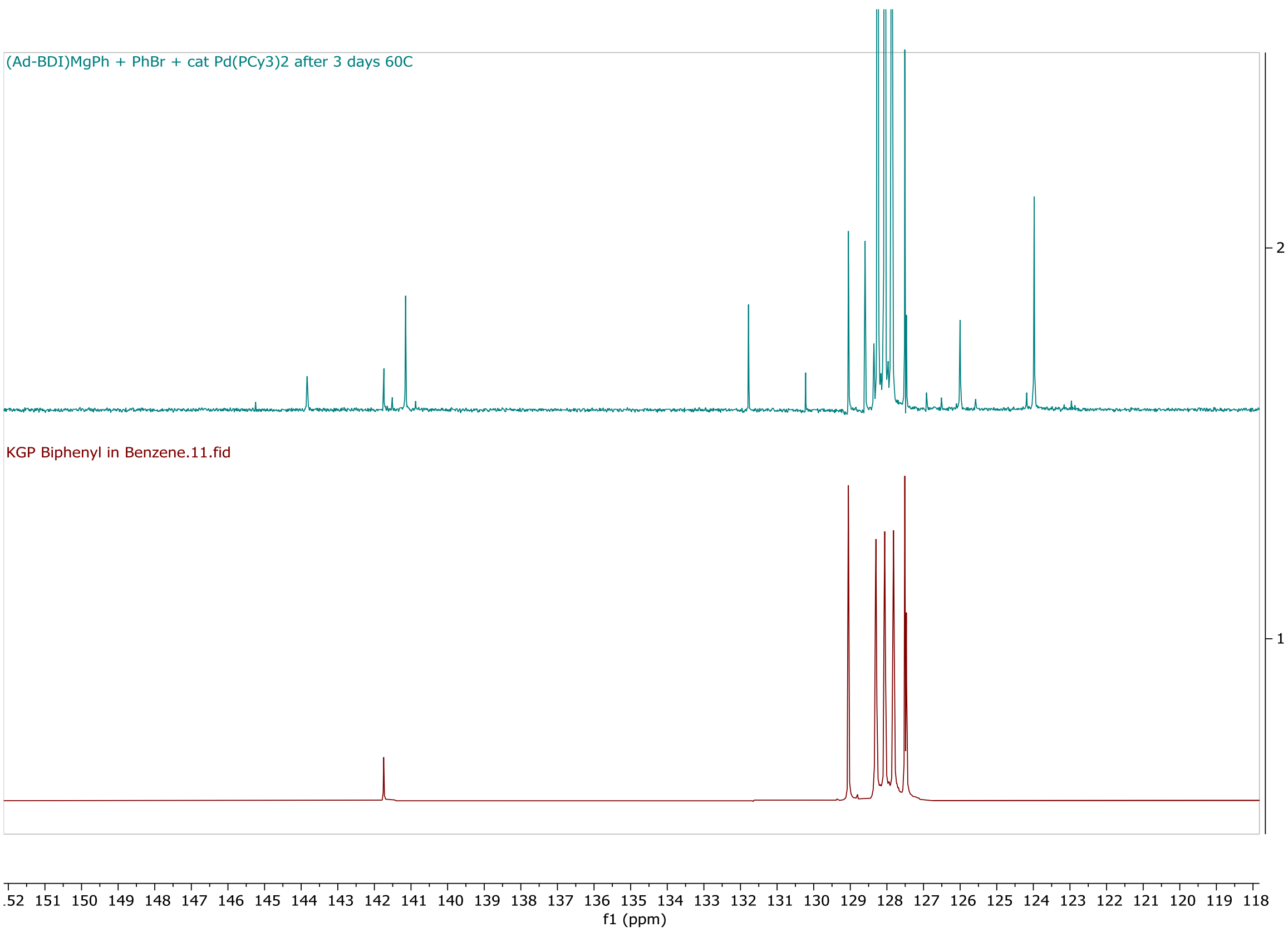

Figure S204: Expanded stacked $^{13}C\{^1H\}$ NMR spectra (126 MHz, C_6D_6) after 3 days at 60°C (top) compared to an authentic sample of biphenyl (bottom).

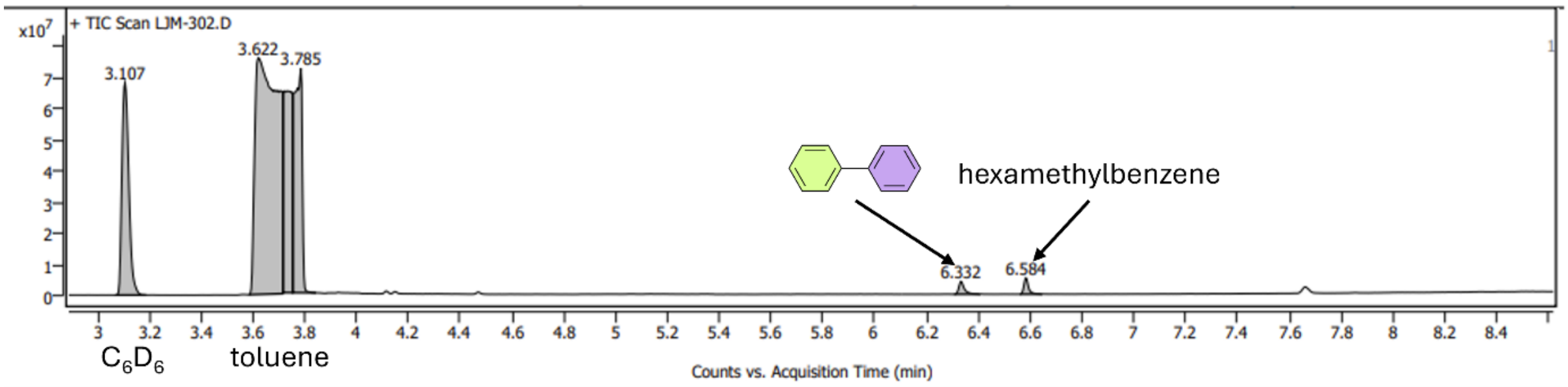

Figure S205: Gas-chromatogram of the diluted reaction mixture.

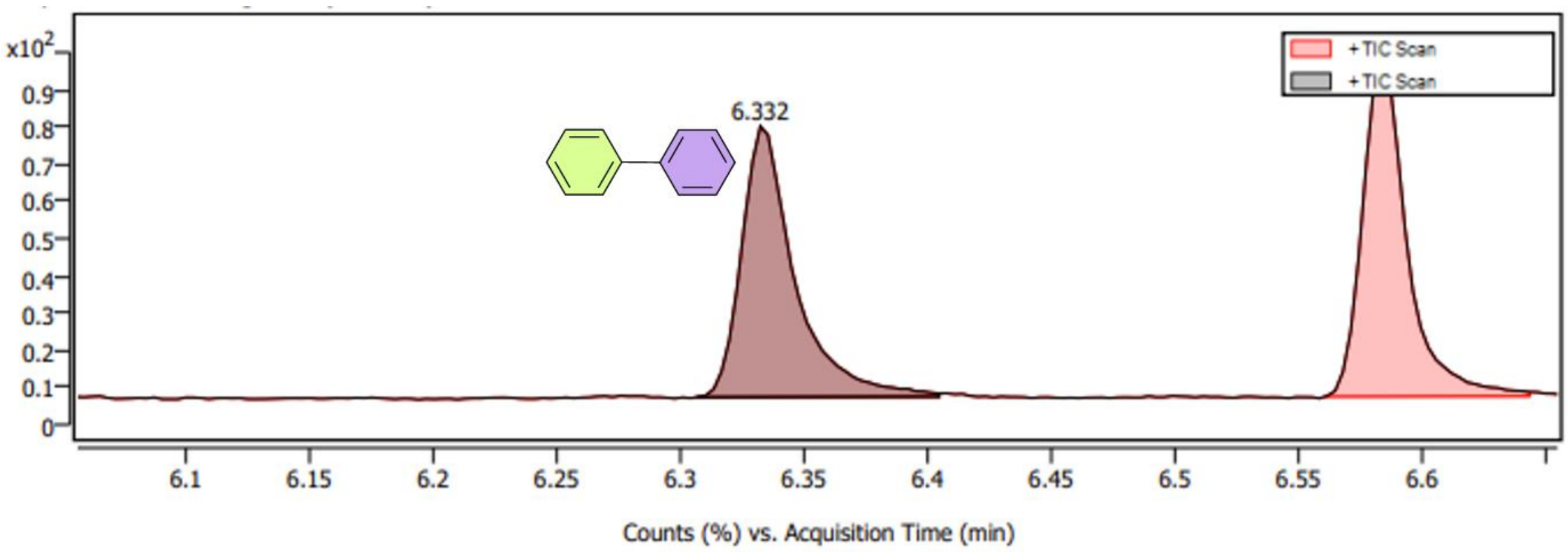

Figure S206: Expanded gas-chromatogram showing peak for biphenyl.

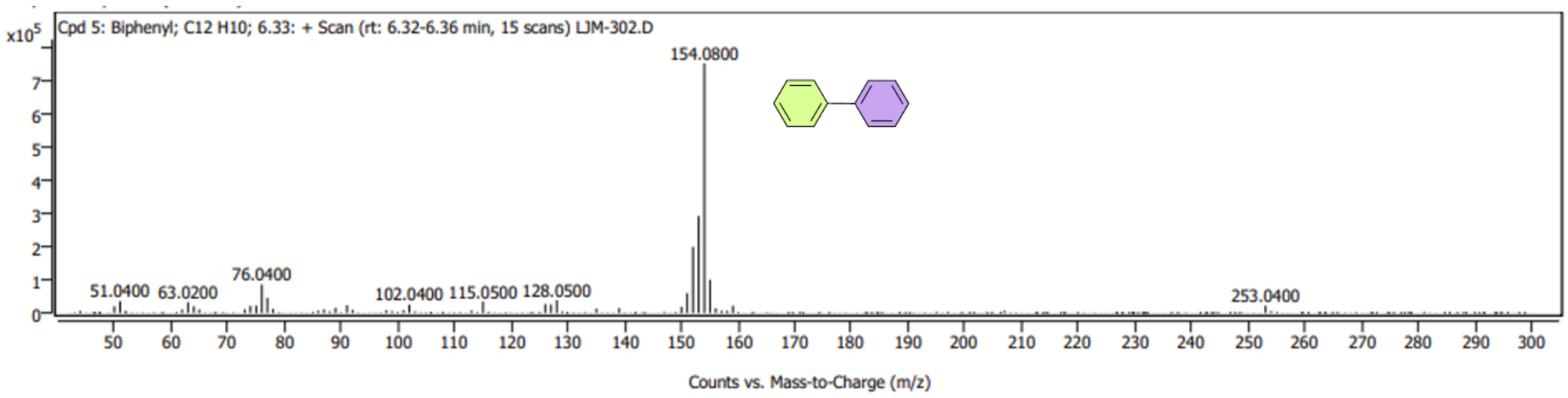

Figure S207: Mass spectrum of biphenyl, showing molecular ion peak, from GC-MS analysis of the reaction mixture.

2.24.3 Cross-coupling of benzene and 4-bromoanisole to form 4-methoxybiphenyl

General procedure using 37 mg **1**^{Ad} (0.050 mmol), 10.7 mg 4-methoxy-1-bromobenzene (0.057 mmol), 5.1 mg hexamethylbenzene (0.031 mmol), 1.7 mg [Pd(PCy$_3$)$_2$] (0.003 mmol, 5.1 mol%).

>99% yield 4-methoxy-1-biphenyl after 2 days at 60°C.

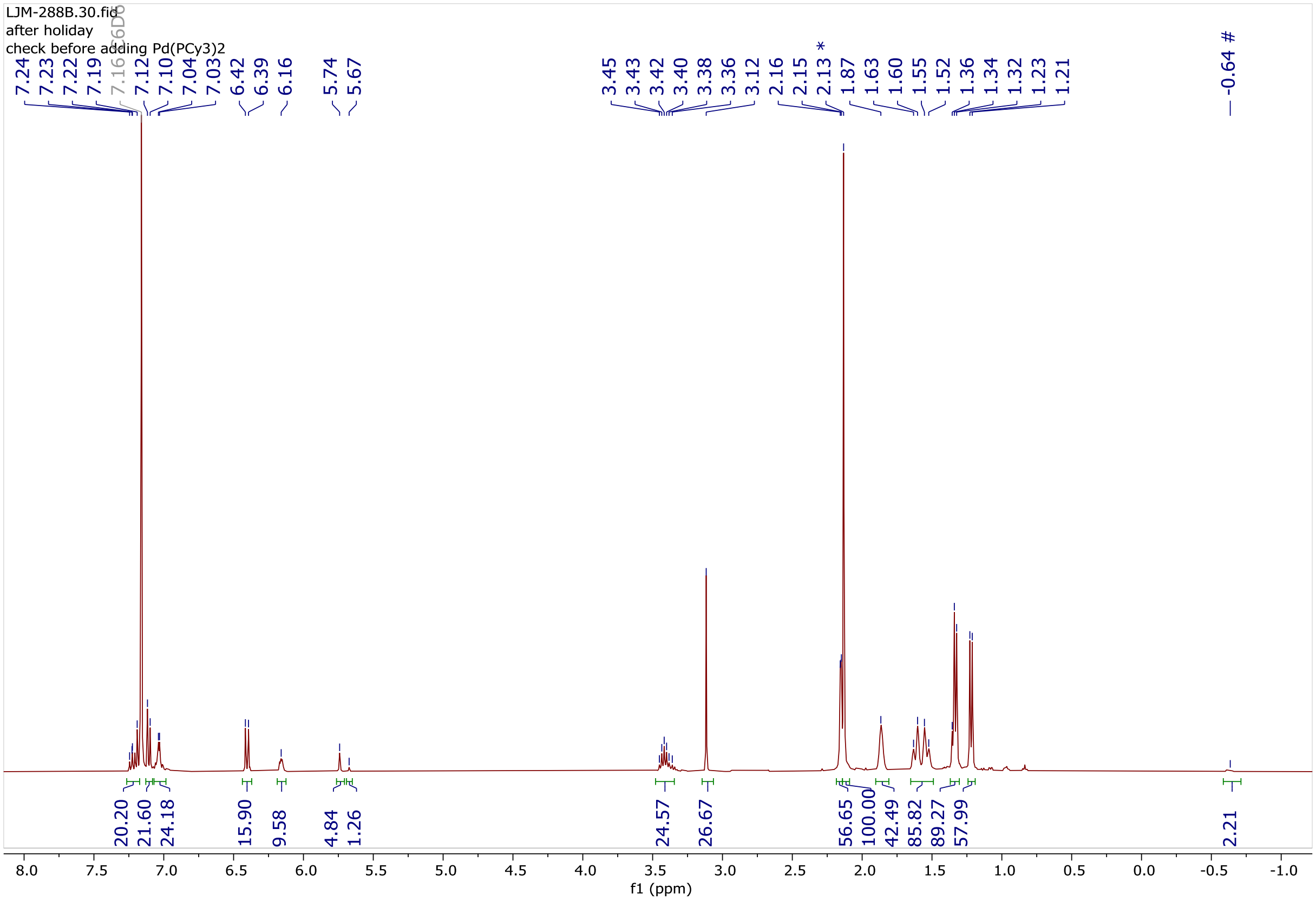

Figure S208: ^{1}H NMR (400 MHz, C_6D_6) before addition of [Pd(PCy$_3$)$_2$]. *Hexamethylbenzene. #Residual **1^{Ad}**.

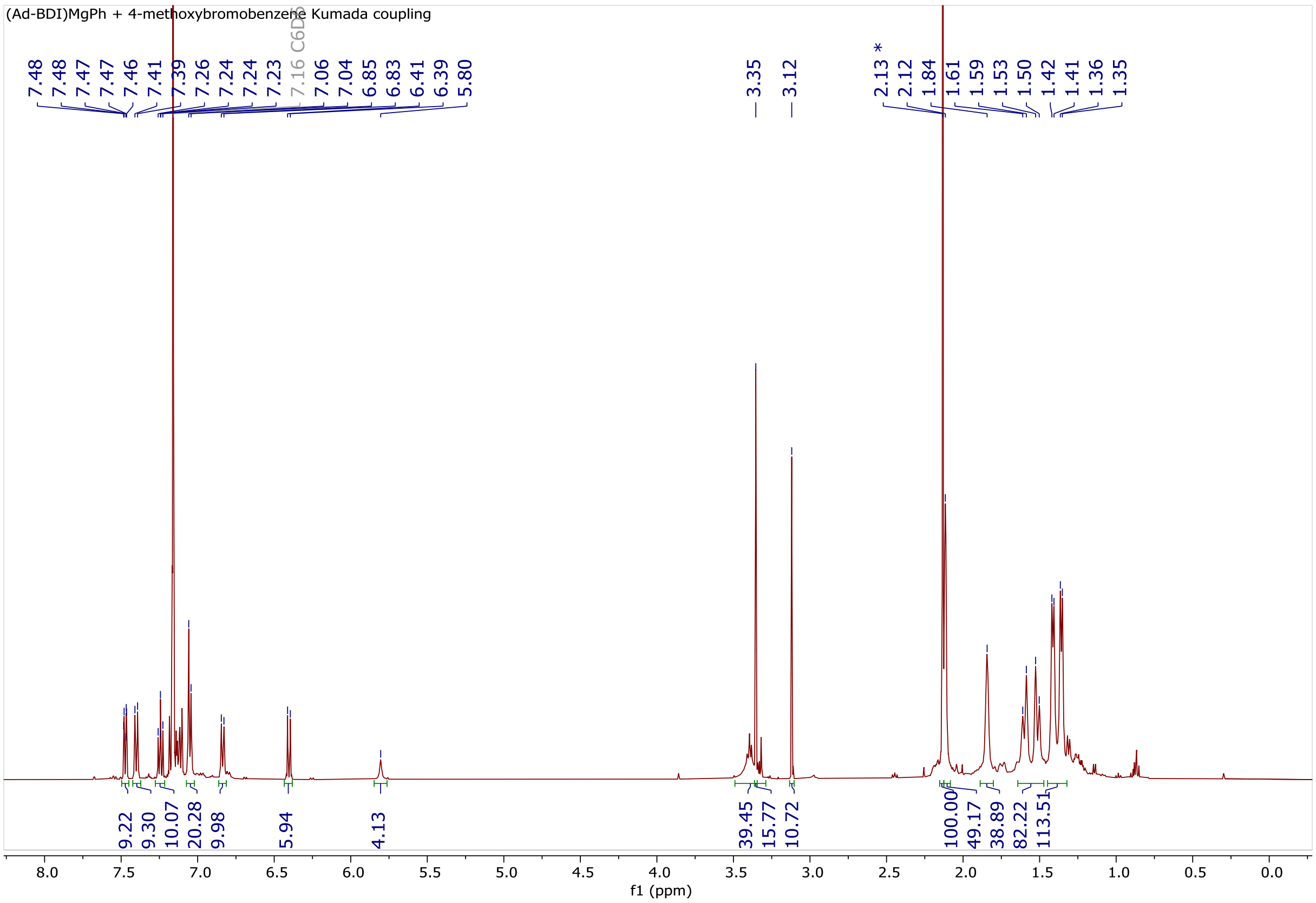

Figure S209: ^{1}H NMR (500 MHz, C_6D_6) after 2 days at 60°C. *Hexamethylbenzene.

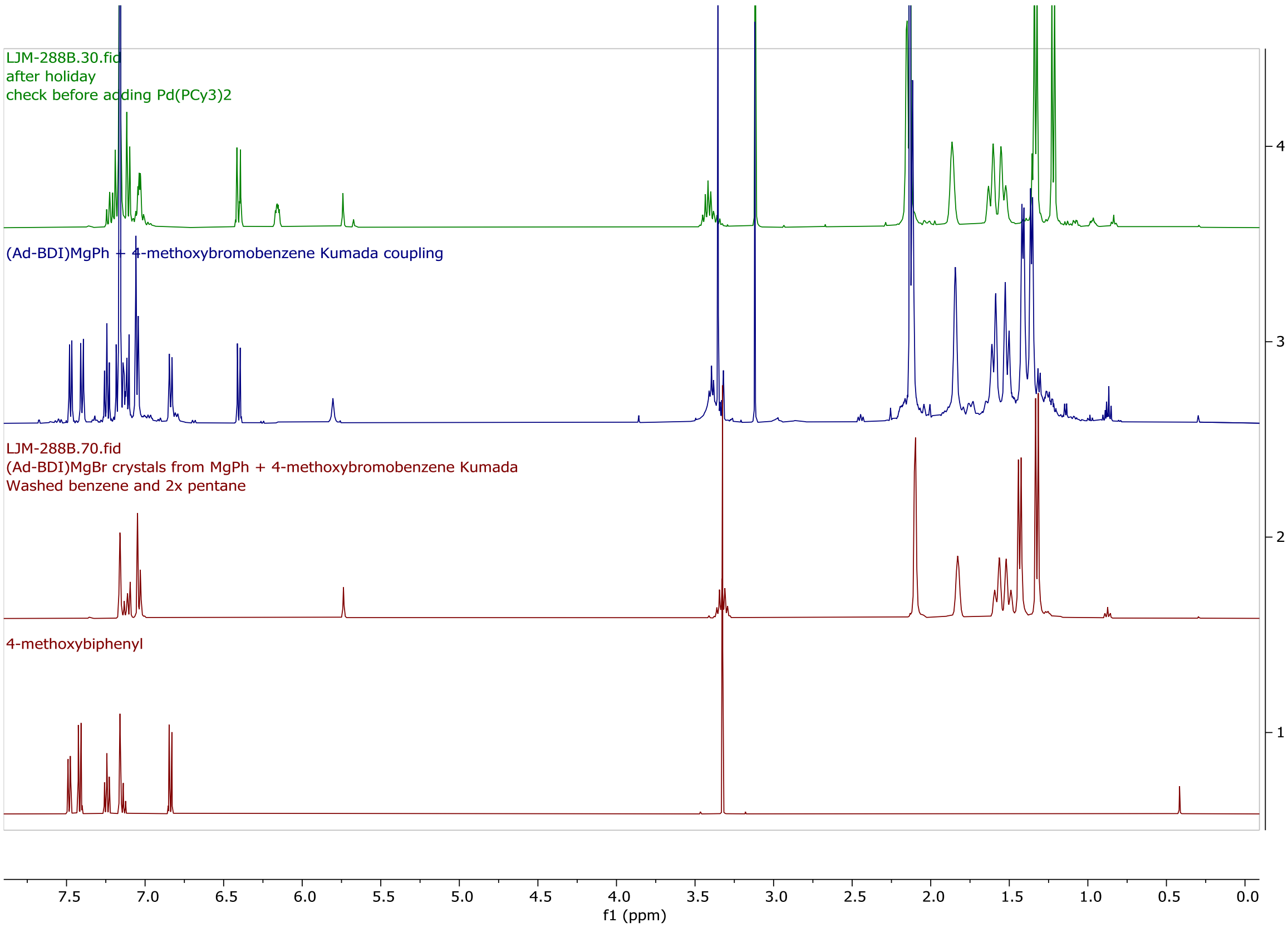

Figure S210: Stacked ^{1}H NMR spectra (C_6D_6) from top to bottom: before addition of [$Pd(PCy_3)_2$] (400 MHz), after 2 days at 60°C (500 MHz), a pure crystalline sample of **13**, a separate sample of pure 4-methoxybiphenyl (500 MHz). Note slight differences in chemical shift between the products in the reaction mixture and in their pure form, especially of the magnesium complex. This is probably due to coordination of 4-methoxybiphenyl to the magnesium centre.

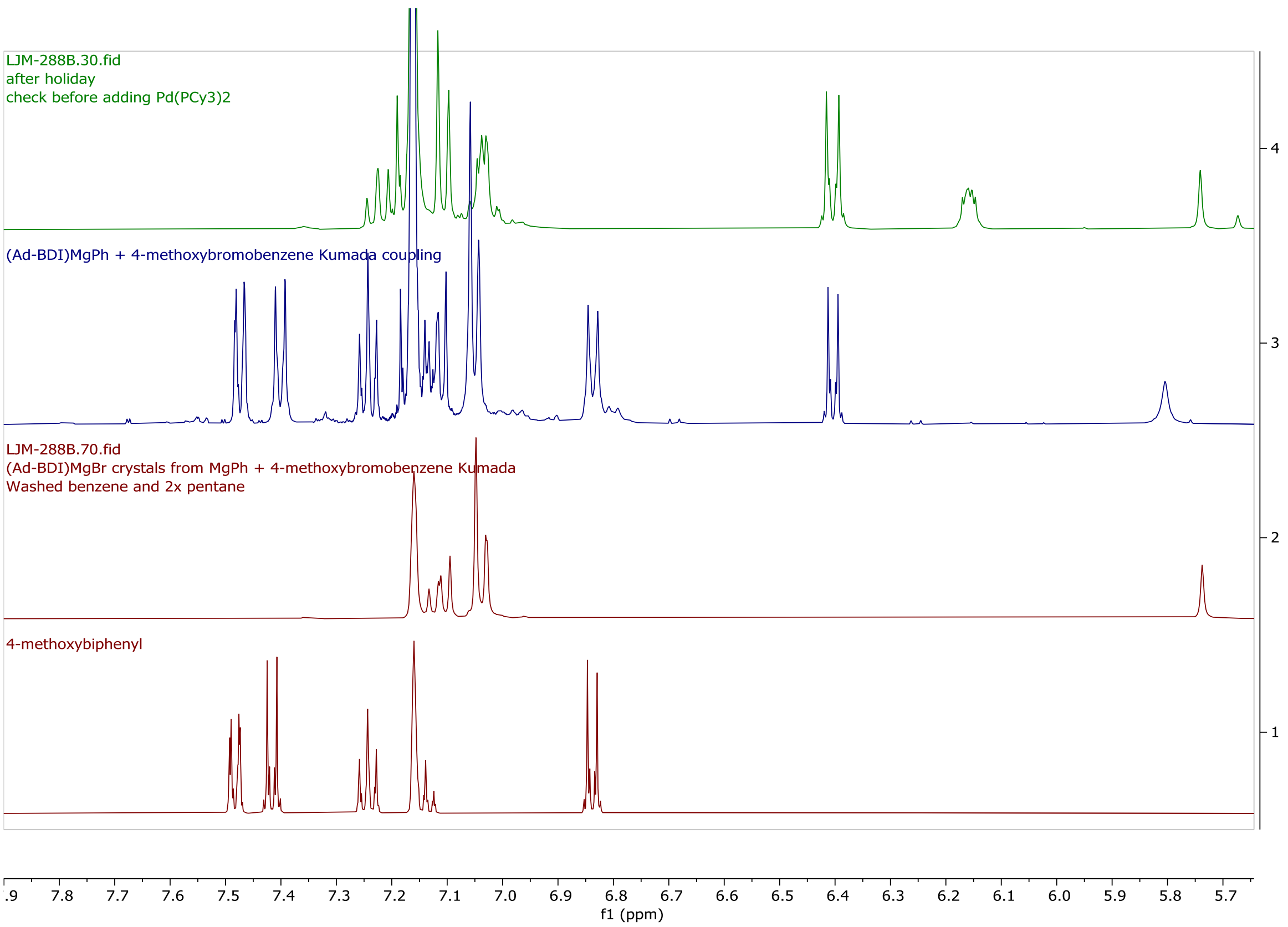

Figure S211: Expanded stacked ^{1}H NMR spectra (C_6D_6) from top to bottom: before addition of [Pd(PCy$_3$)$_2$] (400 MHz), after 2 days at 60°C (500 MHz), a pure crystalline sample of **13**, a separate sample of pure 4-methoxybiphenyl (500 MHz). Note slight differences in chemical shift between the products in the reaction mixture and in their pure form, especially of the magnesium complex. This is probably due to coordination of 4-methoxybiphenyl to the magnesium centre.

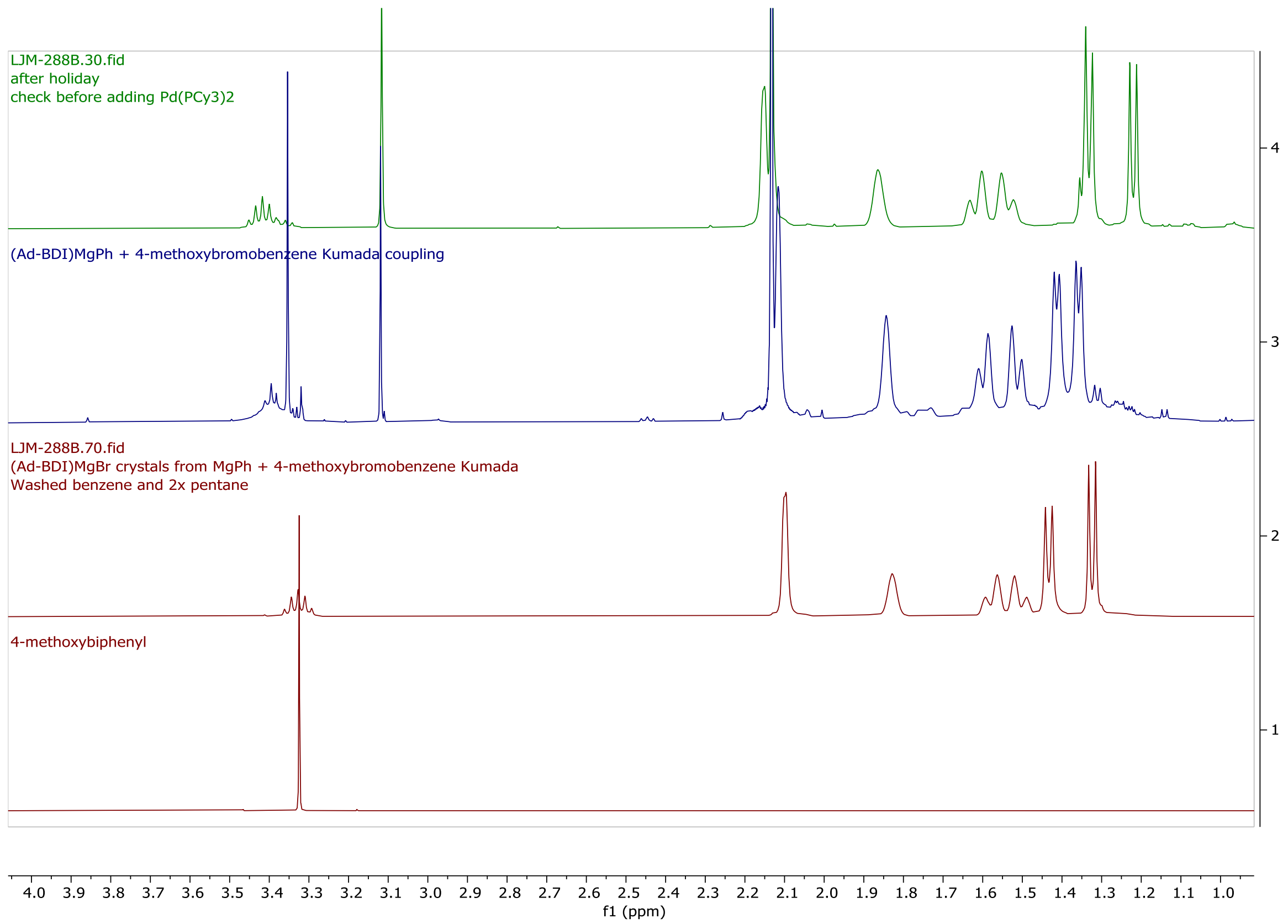

Figure S212: Expanded stacked ^{1}H NMR spectra (C_6D_6) from top to bottom: before addition of [Pd(PCy_3)$_2$] (400 MHz), after 2 days at 60°C (500 MHz), a pure crystalline sample of **13**, a separate sample of pure 4-methoxybiphenyl (500 MHz). Note slight differences in chemical shift between the products in the reaction mixture and in their pure form, especially of the magnesium complex. This is probably due to coordination of 4-methoxybiphenyl to the magnesium centre.

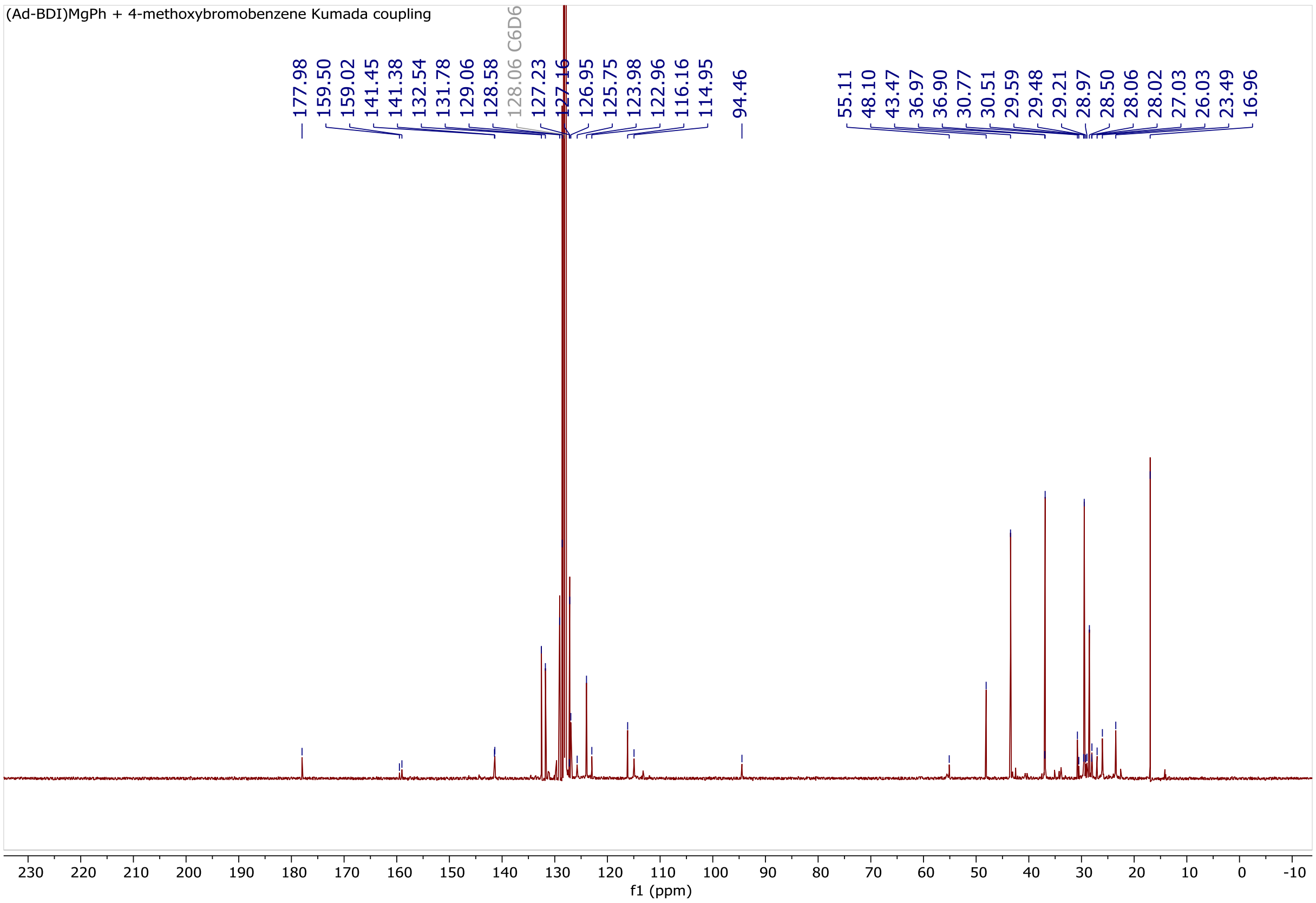

Figure S213: $^{13}C\{^{1}H\}$ NMR spectrum (126 MHz, C_6D_6) after 2 days at 60°C.

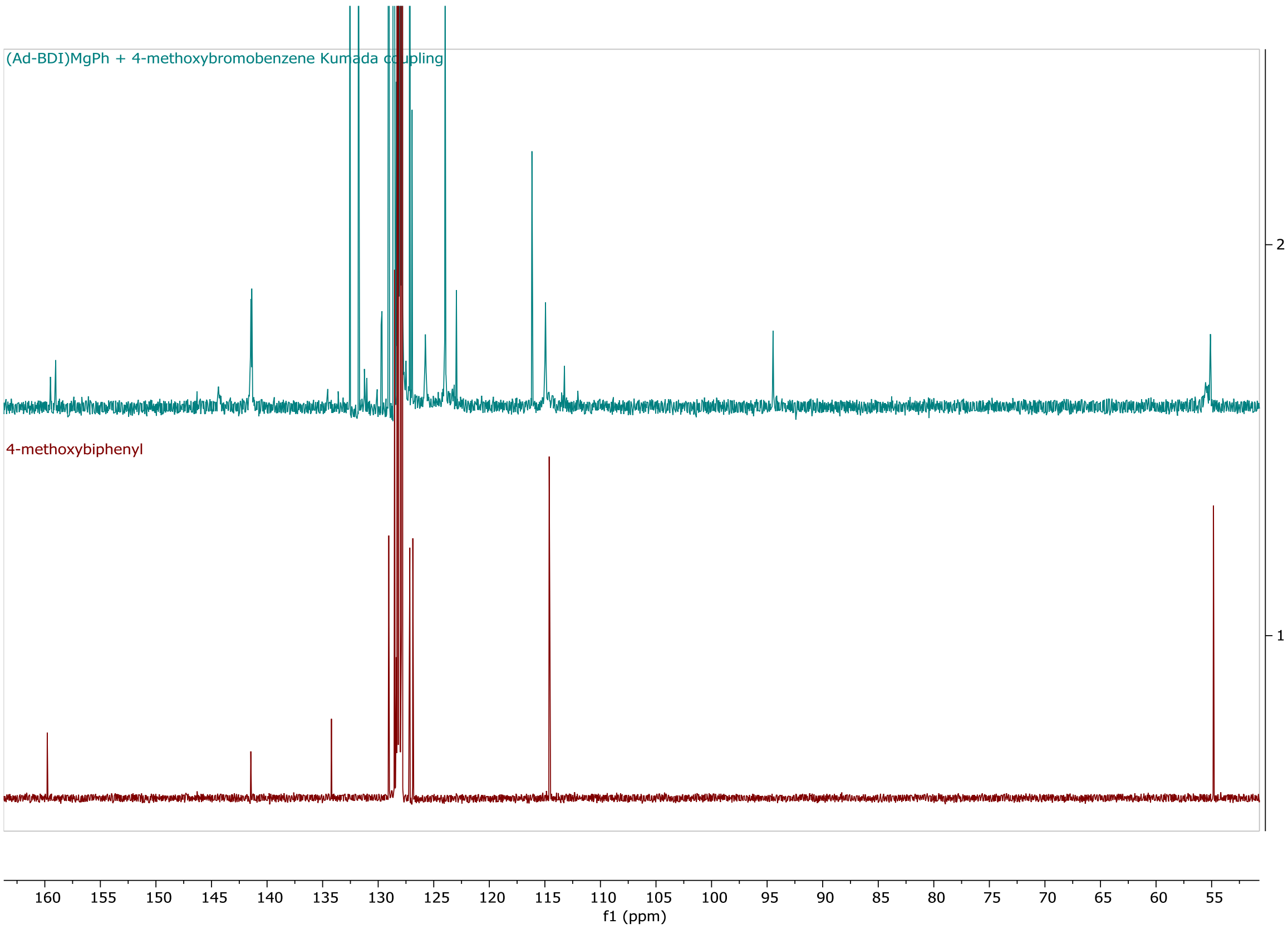

Figure S214: Expanded stacked $^{13}C\{^{1}H\}$ NMR spectra (126 MHz, C_6D_6) after 2 days at 60°C (top) compared to an authentic sample of 4-methoxy-biphenyl (bottom). Note, some slight differences in chemical shift are observed, possibly due to coordination of the product to the magnesium centre.

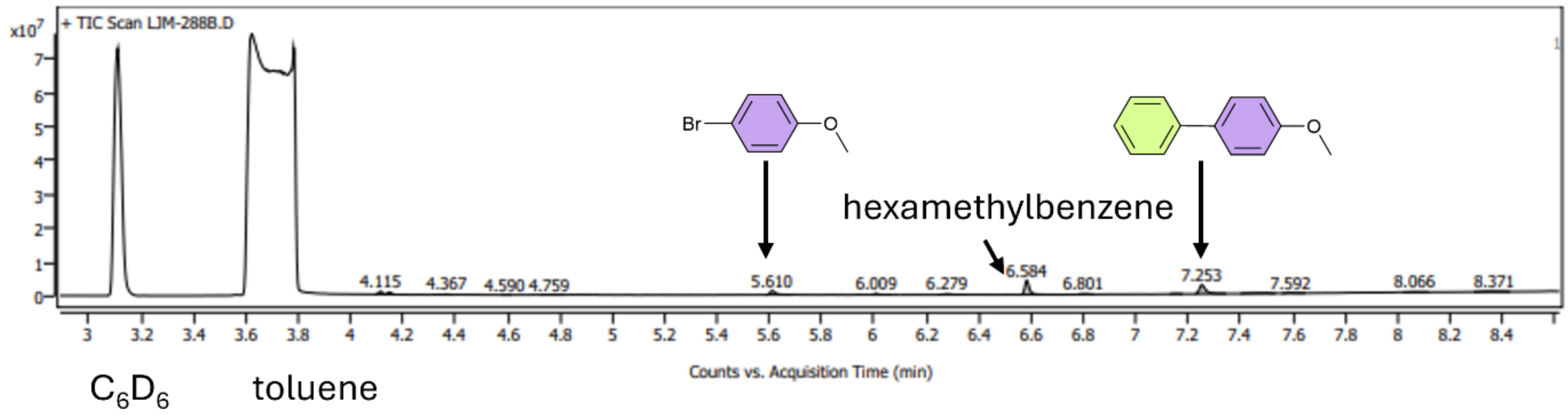

Figure S215: Gas-chromatogram of the diluted reaction mixture.

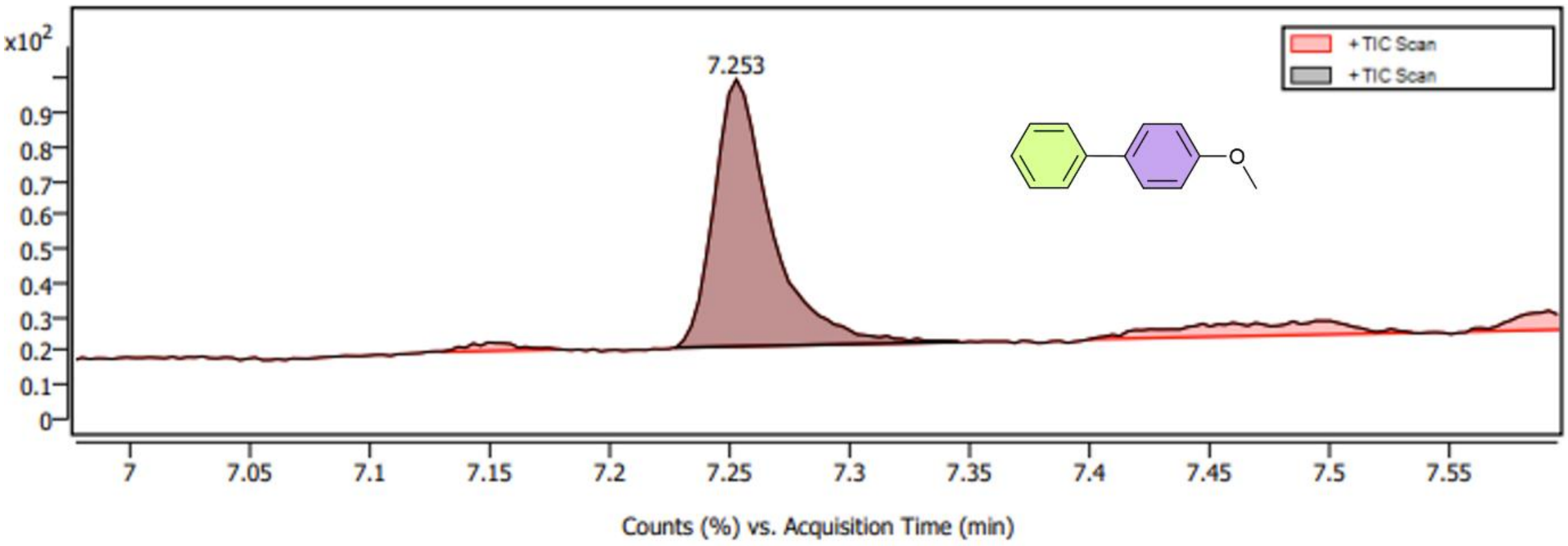

Figure S216: Expanded gas-chromatogram showing peak for 4-methoxybiphenyl.

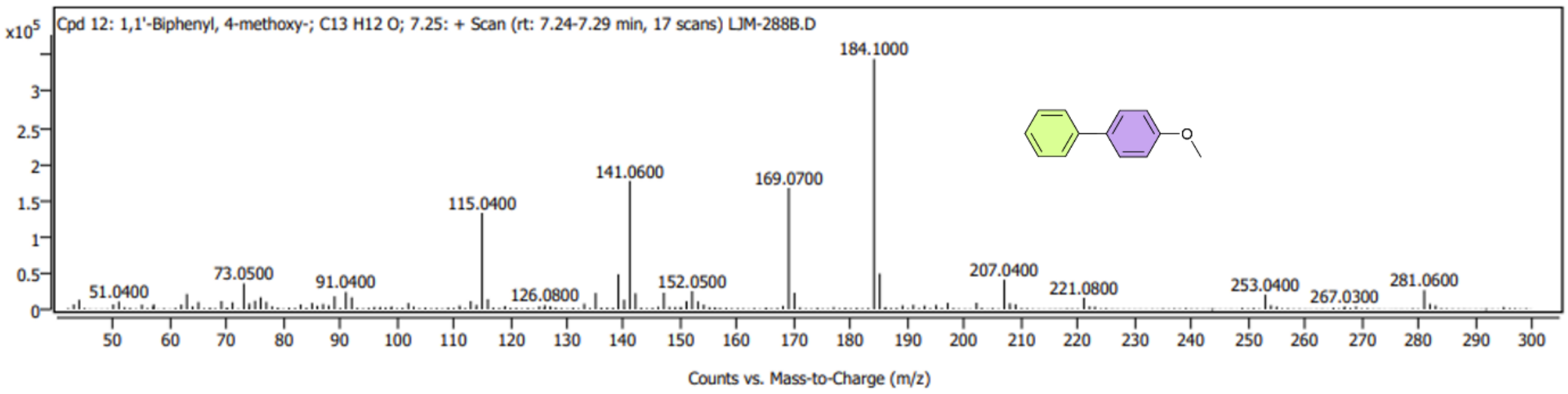

Figure S217: Mass spectrum of 4-methoxybiphenyl, showing molecular ion peak, from GC-MS analysis of the reaction mixture.

2.24.4 Cross-coupling of benzene-d_6 and 4-bromoanisole to form 4-methoxybiphenyl-d_5

General procedure carried out in benzene-d_6 using 34 mg **1**^{Ad} (0.046 mmol), 8.8 mg 4-methoxy-1-bromobenzene (0.047 mmol), 3 mg hexamethylbenzene (0.018 mmol), 1.4 mg [Pd(PCy$_3$)$_2$] (0.002 mmol, 4.2 mol%).

>99% yield 4-methoxybiphenyl-d_5 after 2 days at 60°C.

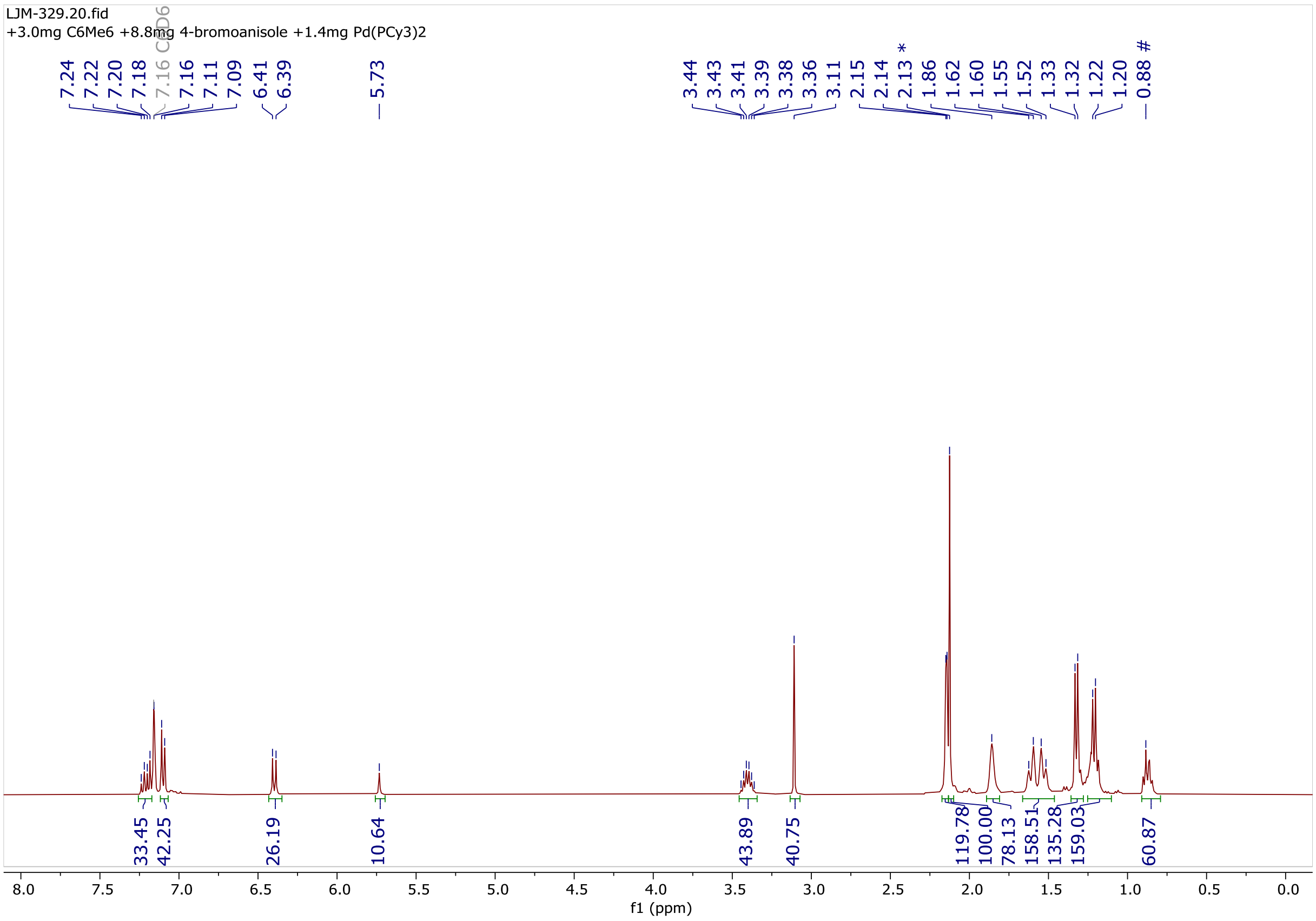

Figure S218: ^{1}H NMR (400 MHz, C_6D_6) immediately after addition of $[Pd(PCy_3)_2]$ before heating. *Hexamethylbenzene. #Butane.

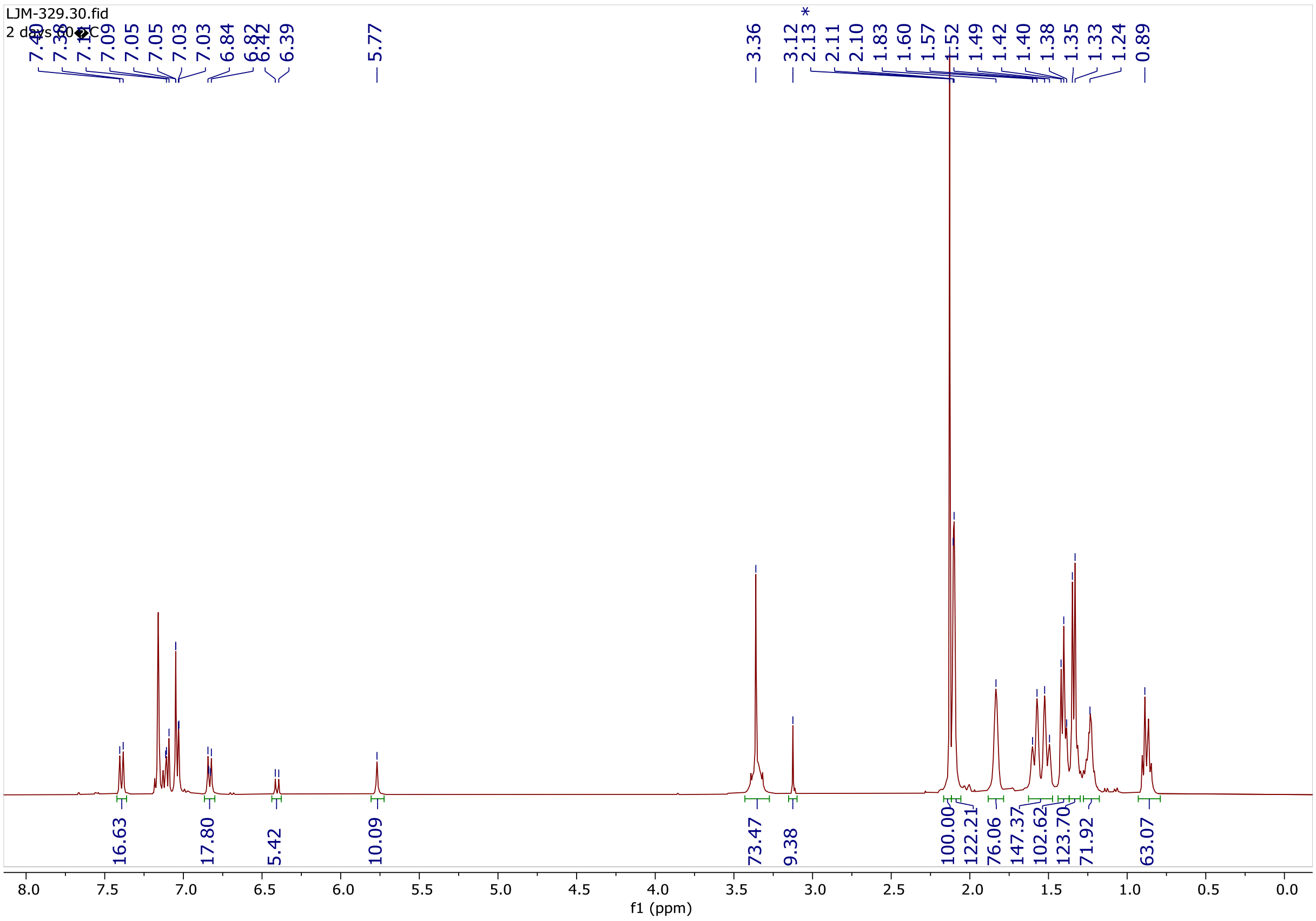

Figure S219: ^{1}H NMR (400 MHz, C_6D_6) after 2 days at 60°C. *Hexamethylbenzene.

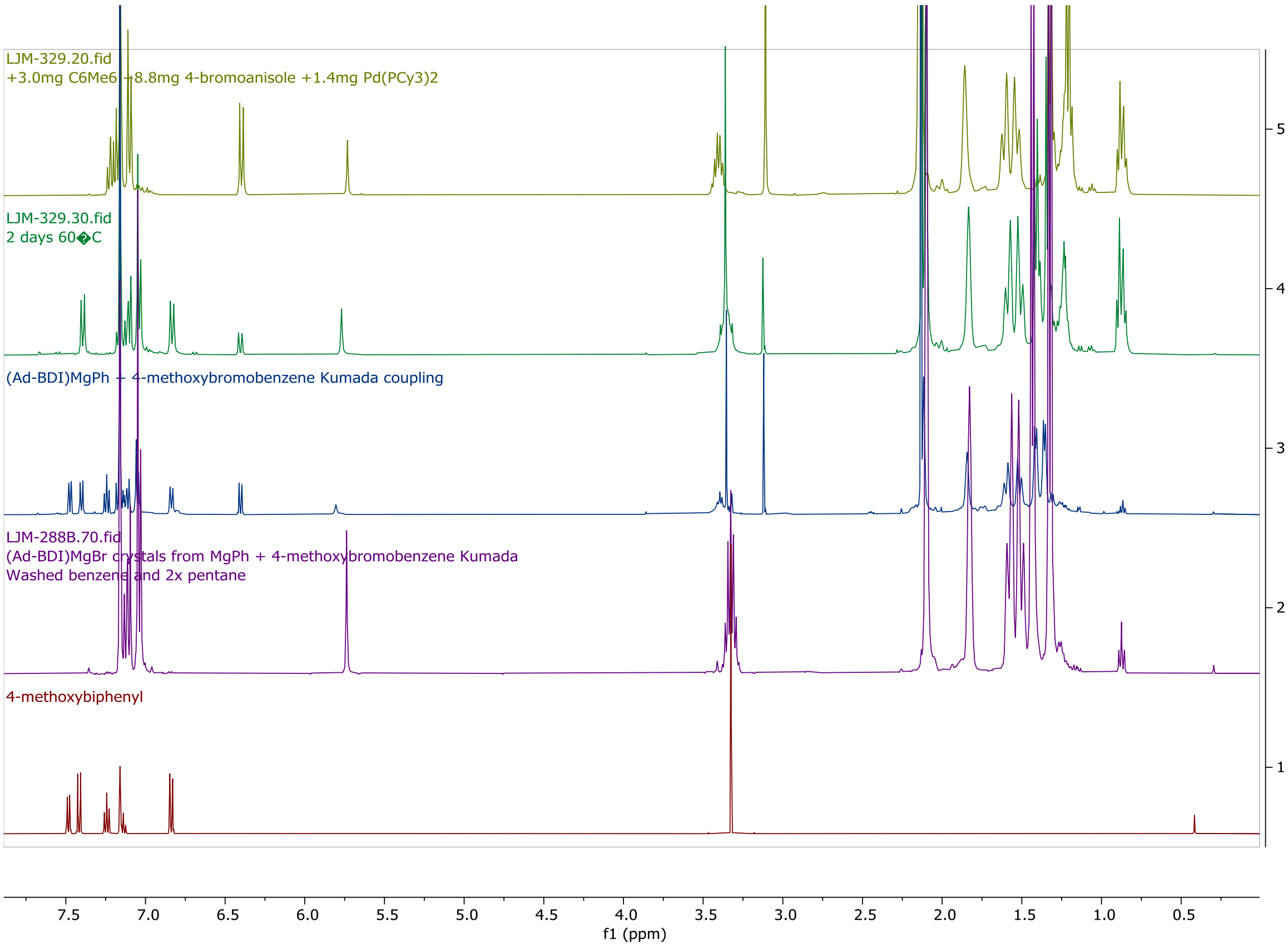

Figure S220: Stacked ^{1}H NMR spectra (C_6D_6) from top to bottom: immediately after addition of [Pd(PCy_3)$_2$] before heating (400 MHz), after 2 days at 60°C (400 MHz), the same reaction after 2 days at 60°C but without isotopic labelling (500 MHz), a pure crystalline sample of **13**, a separate sample of pure 4-methoxybiphenyl (500 MHz). Note slight differences in chemical shift between the products in the reaction mixture and in their pure form, especially of the magnesium complex. This is probably due to coordination of 4-methoxybiphenyl to the magnesium centre.

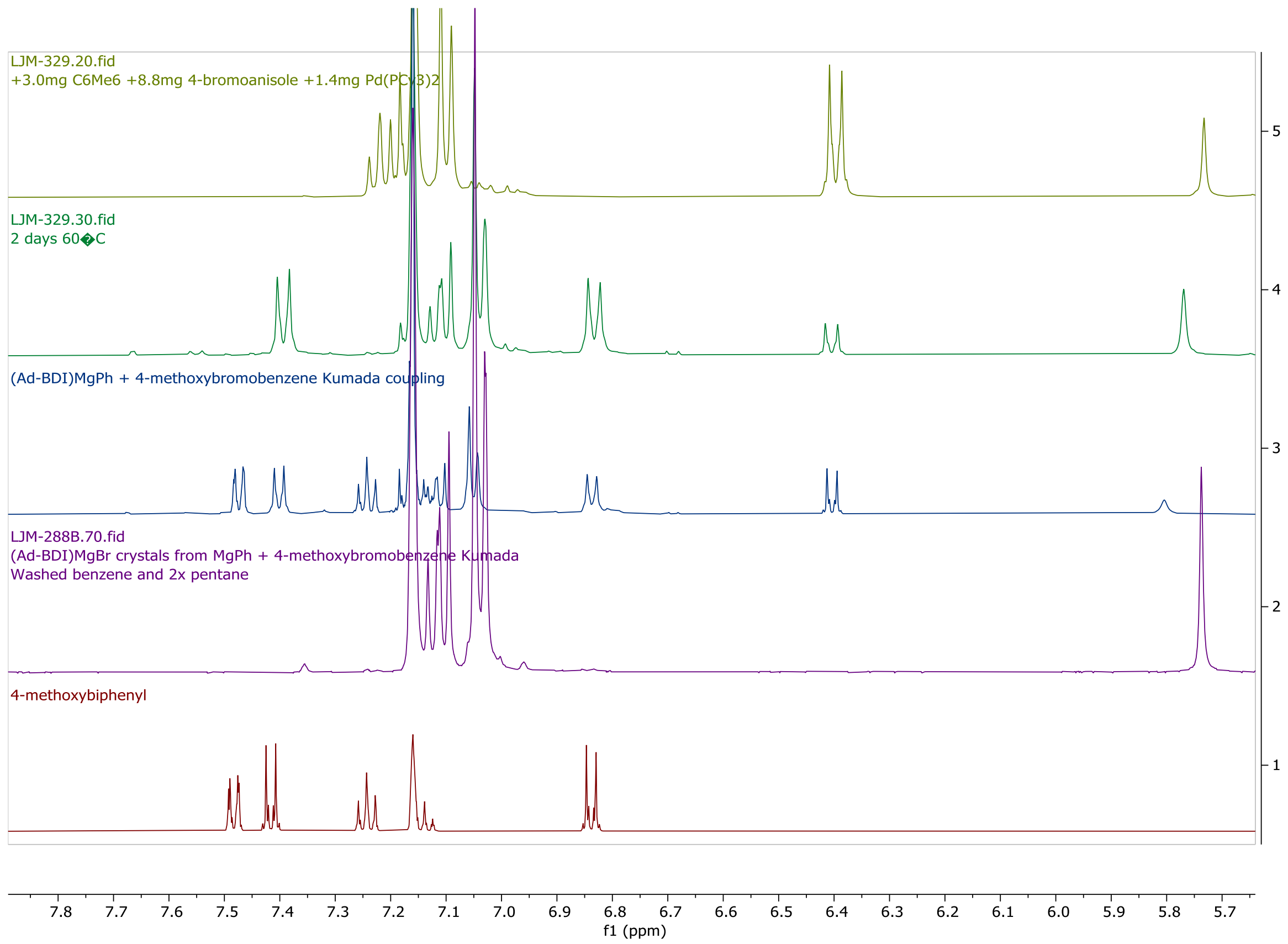

Figure S221: Expanded stacked ^{1}H NMR spectra (C_6D_6) from top to bottom: immediately after addition of [$Pd(PCy_3)_2$] before heating (400 MHz), after 2 days at 60°C (400 MHz), the same reaction after 2 days at 60°C but without isotopic labelling (500 MHz), a pure crystalline sample of **13**, a separate sample of pure 4-methoxybiphenyl (500 MHz). Note slight differences in chemical shift between the products in the reaction mixture and in their pure form, especially of the magnesium complex. This is probably due to coordination of 4-methoxybiphenyl to the magnesium centre.

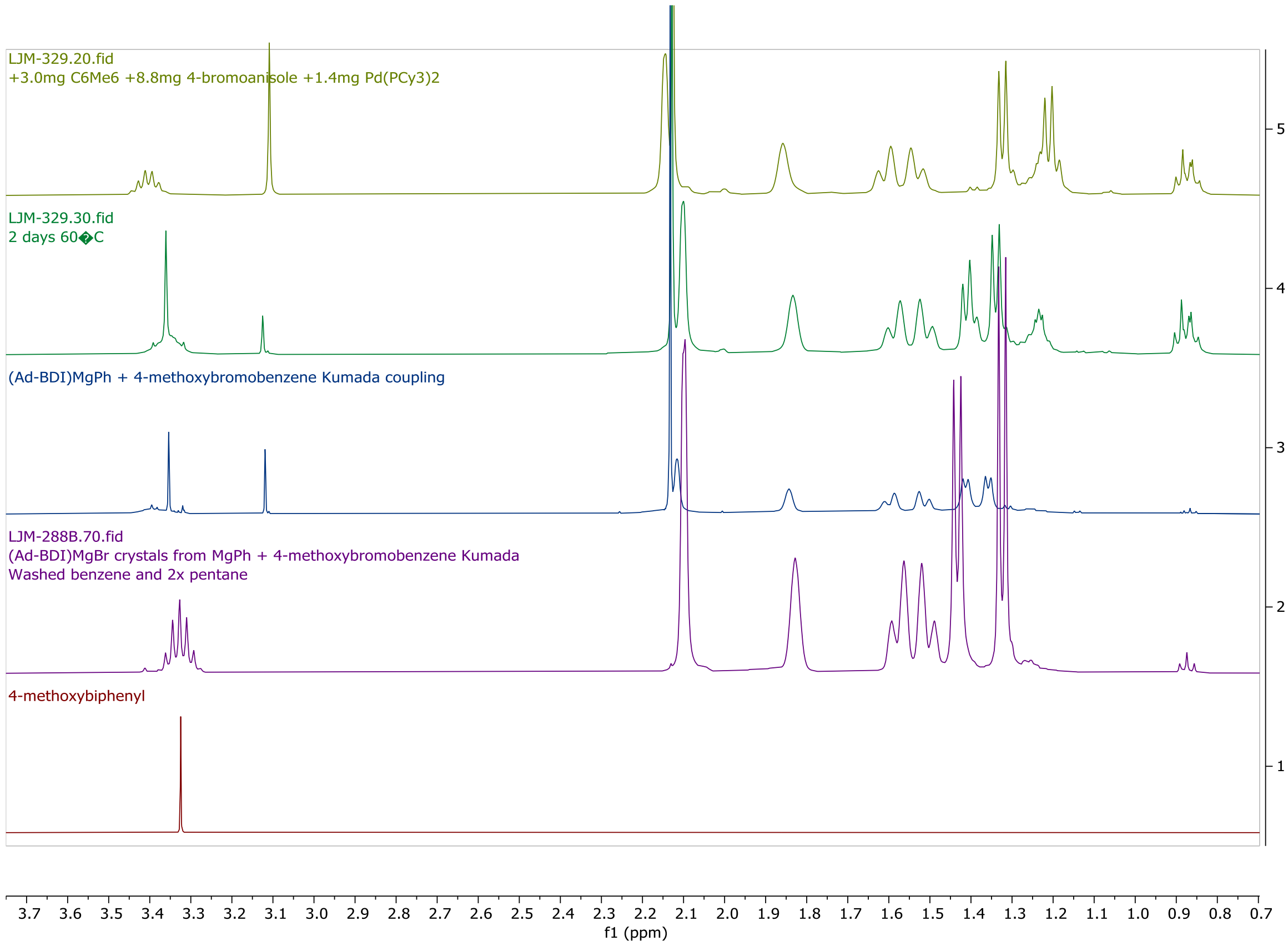

Figure S222: Expanded stacked ^{1}H NMR spectra (C_6D_6) from top to bottom: immediately after addition of $[Pd(PCy_3)_2]$ before heating (400 MHz), after 2 days at 60°C (400 MHz), the same reaction after 2 days at 60°C but without isotopic labelling (500 MHz), a pure crystalline sample of **13**, a separate sample of pure 4-methoxybiphenyl (500 MHz). Note slight differences in chemical shift between the products in the reaction mixture and in their pure form, especially of the magnesium complex. This is probably due to coordination of 4-methoxybiphenyl to the magnesium centre.

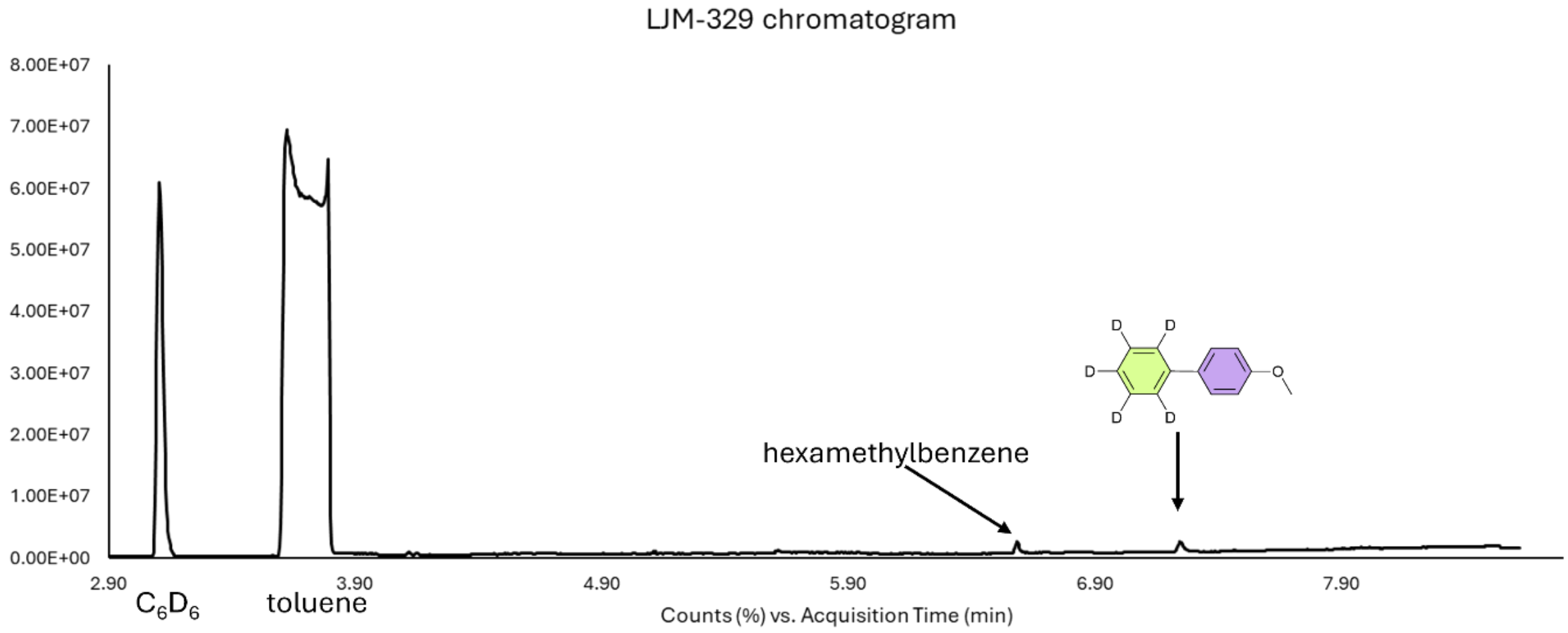

Figure S223: Gas-chromatogram of the diluted reaction mixture.

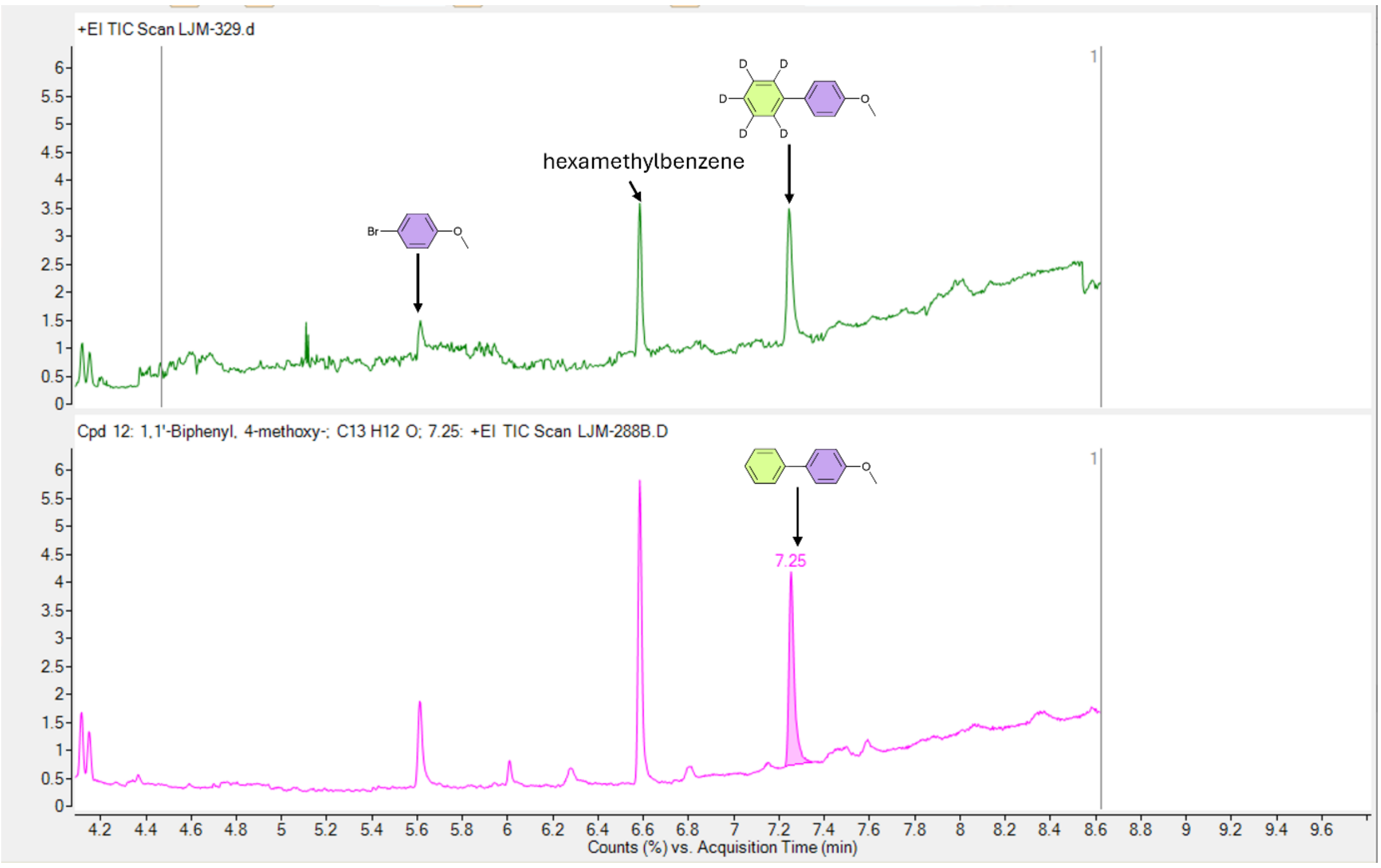

Figure S224: Stacked expanded gas-chromatogram (top) showing peak for 4-methoxybiphenyl-d_5 compared to (bottom) the non-deuterated example.

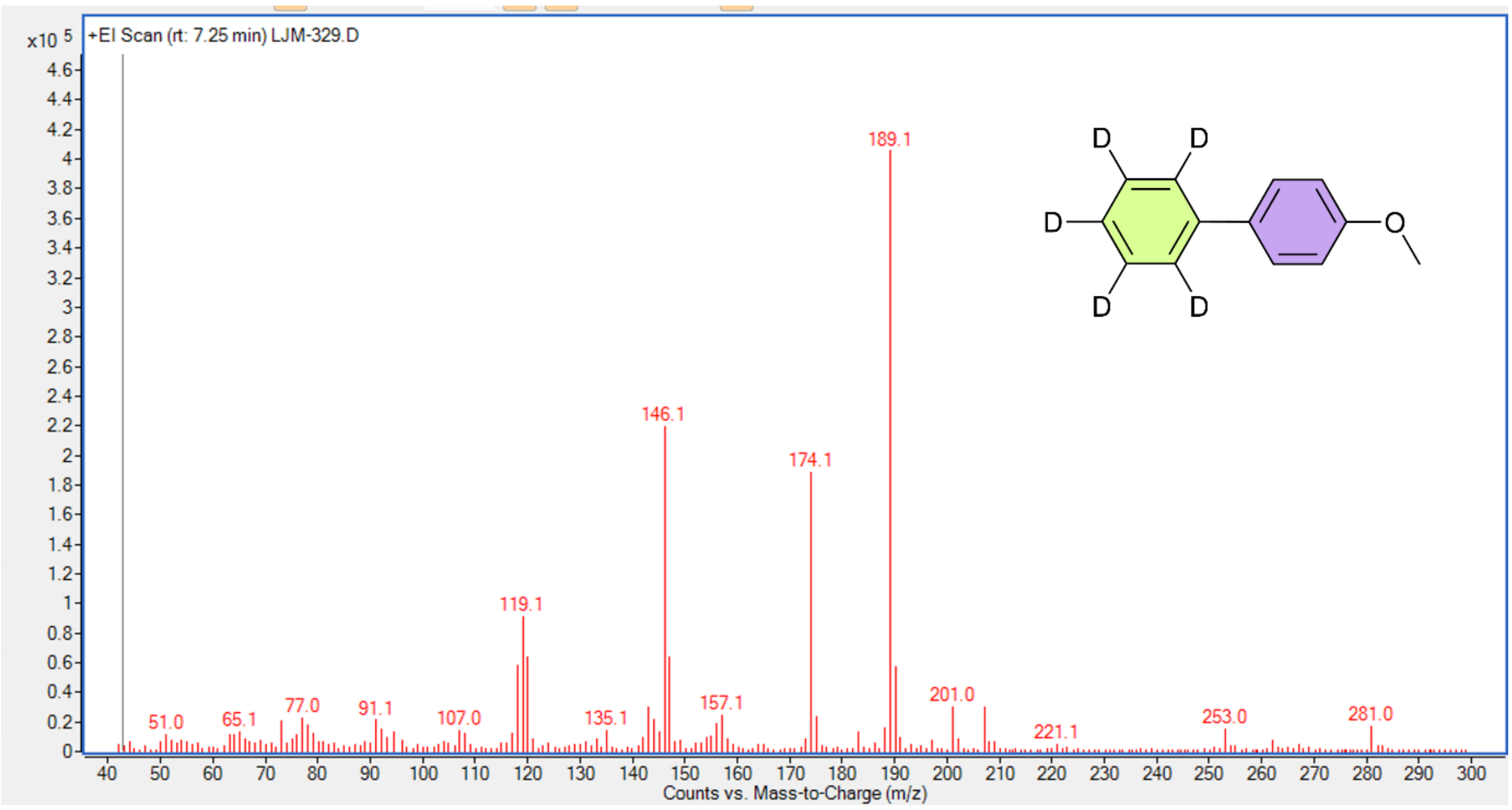

Figure S225: Mass spectrum of 4-methoxybiphenyl-d_5, showing molecular ion peak, from GC-MS analysis of the reaction mixture.

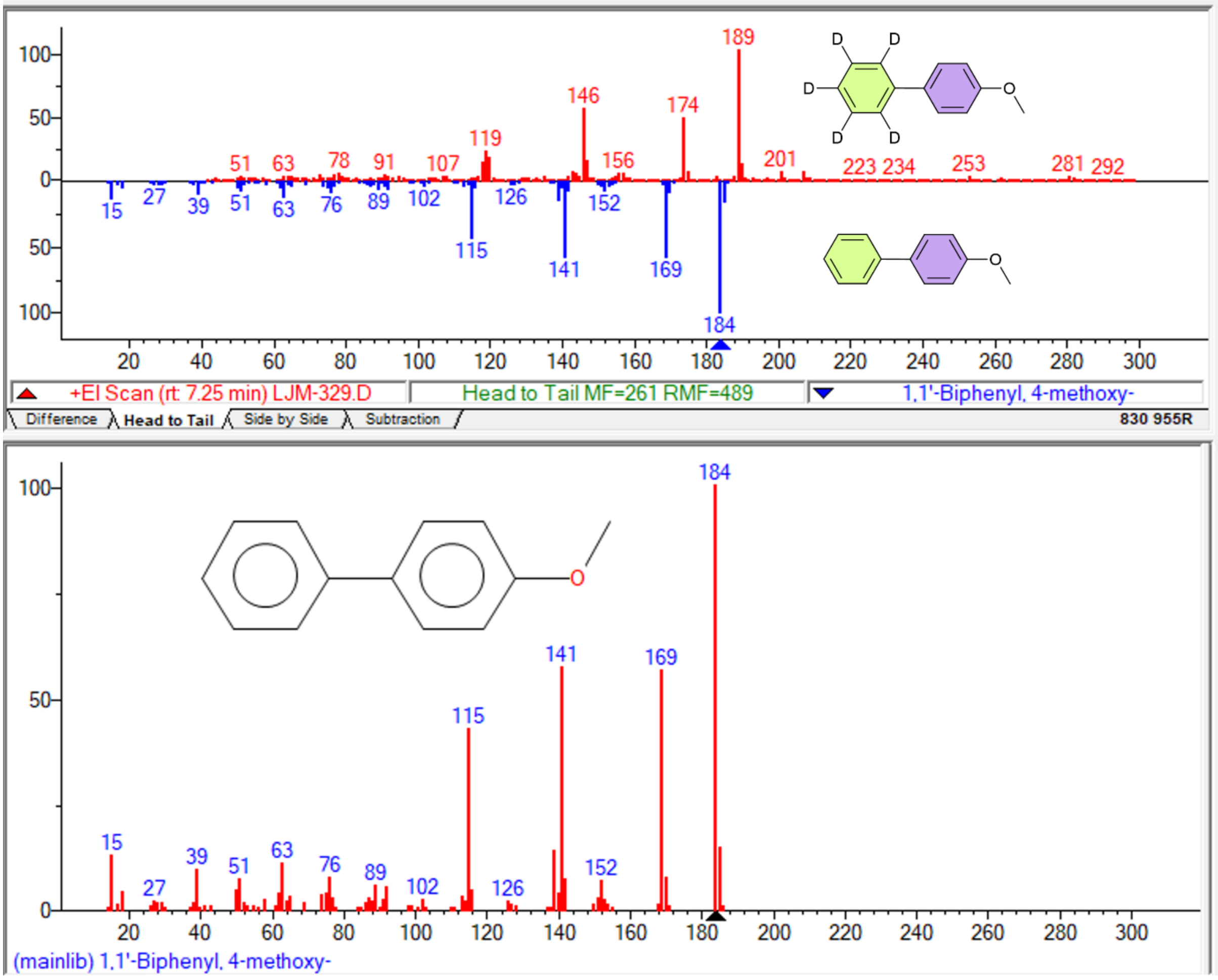

Figure S226: (top) Mirrored mass spectra of 4-methoxybiphenyl-d_5 (red, up) and the non-deuterated compound (blue, down) from GC-MS analysis of the reaction mixture; (bottom) library mass spectrum for 4-methoxybiphenyl.

2.24.5 Cross-coupling of benzene and 2-ethylbromobenzene to form 2-ethylbiphenyl

General procedure using 37 mg **1**^{Ad} (0.050 mmol), 10.1 mg 2-ethyl-1-bromobenzene (0.055 mmol), 7.1 mg hexamethylbenzene (0.044 mmol), 1.3 mg [Pd(PCy$_3$)$_2$] (0.002 mmol, 5.0 mol%).

99% yield 2-ethylbiphenyl after 2 days at 60°C.

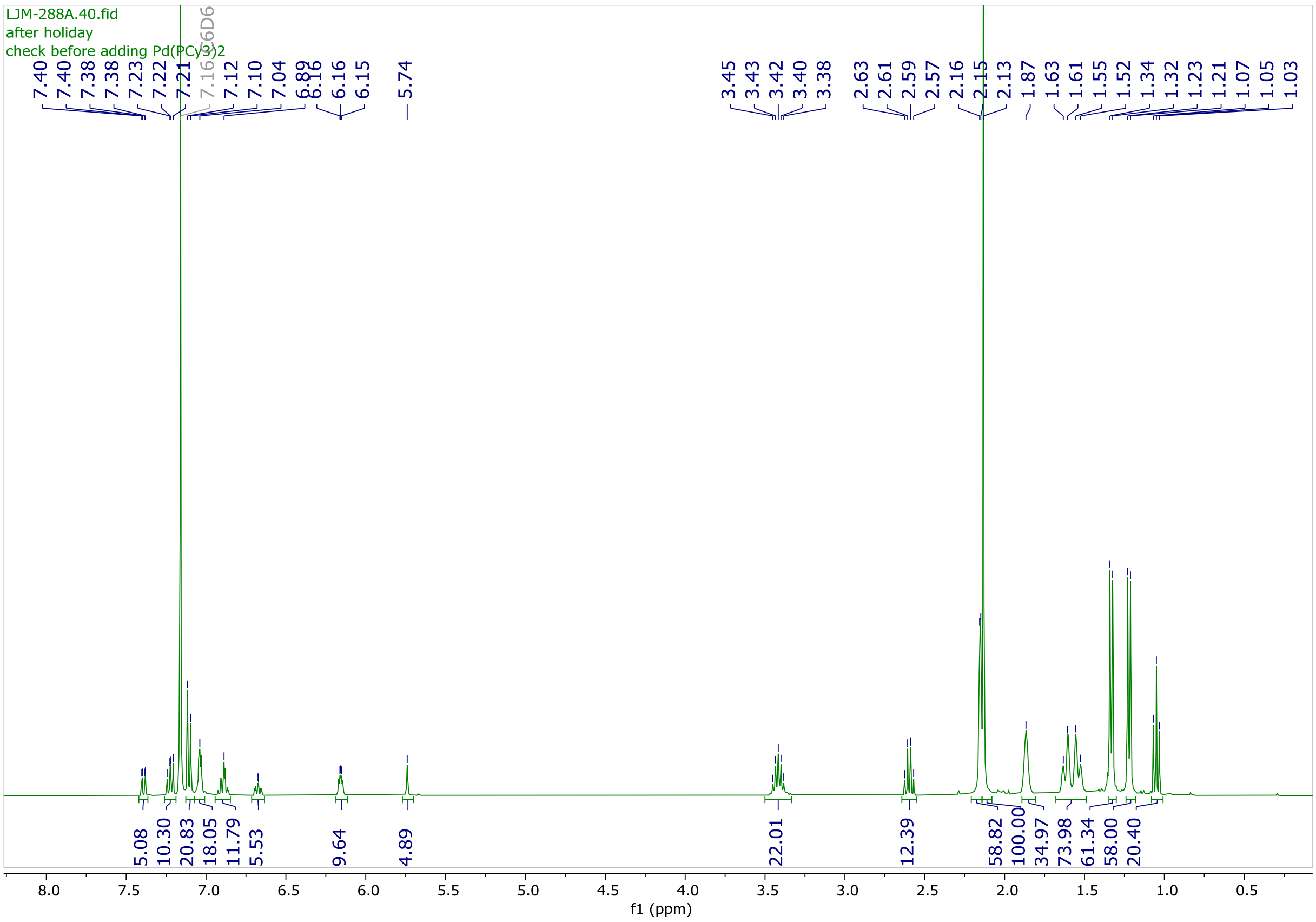

Figure S227: ^{1}H NMR (400 MHz, C_6D_6) before addition of [Pd(PCy_3)$_2$]. *Hexamethylbenzene.

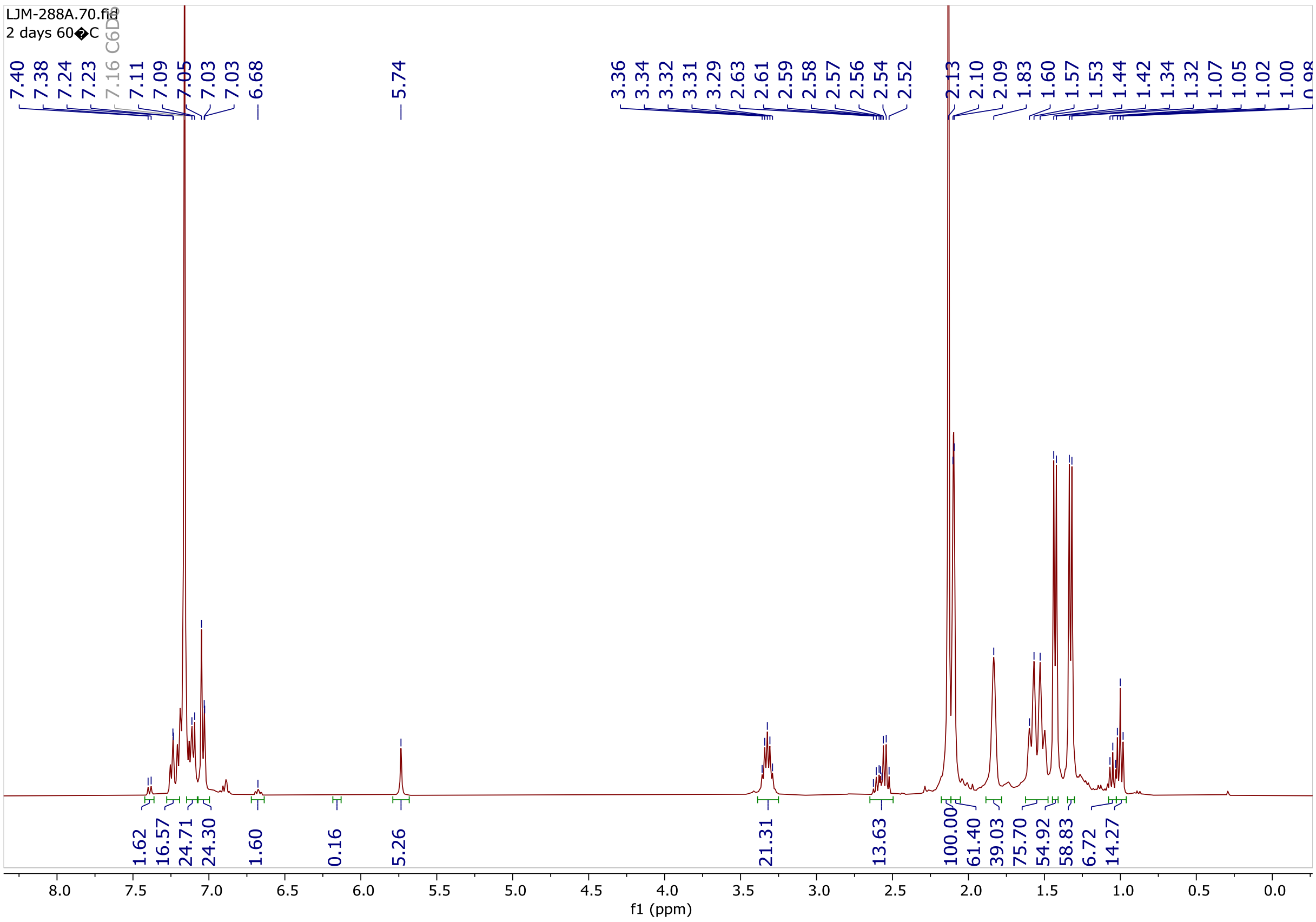

Figure S228: ^{1}H NMR (500 MHz, C_6D_6) after 2 days at 60°C. *Hexamethylbenzene.

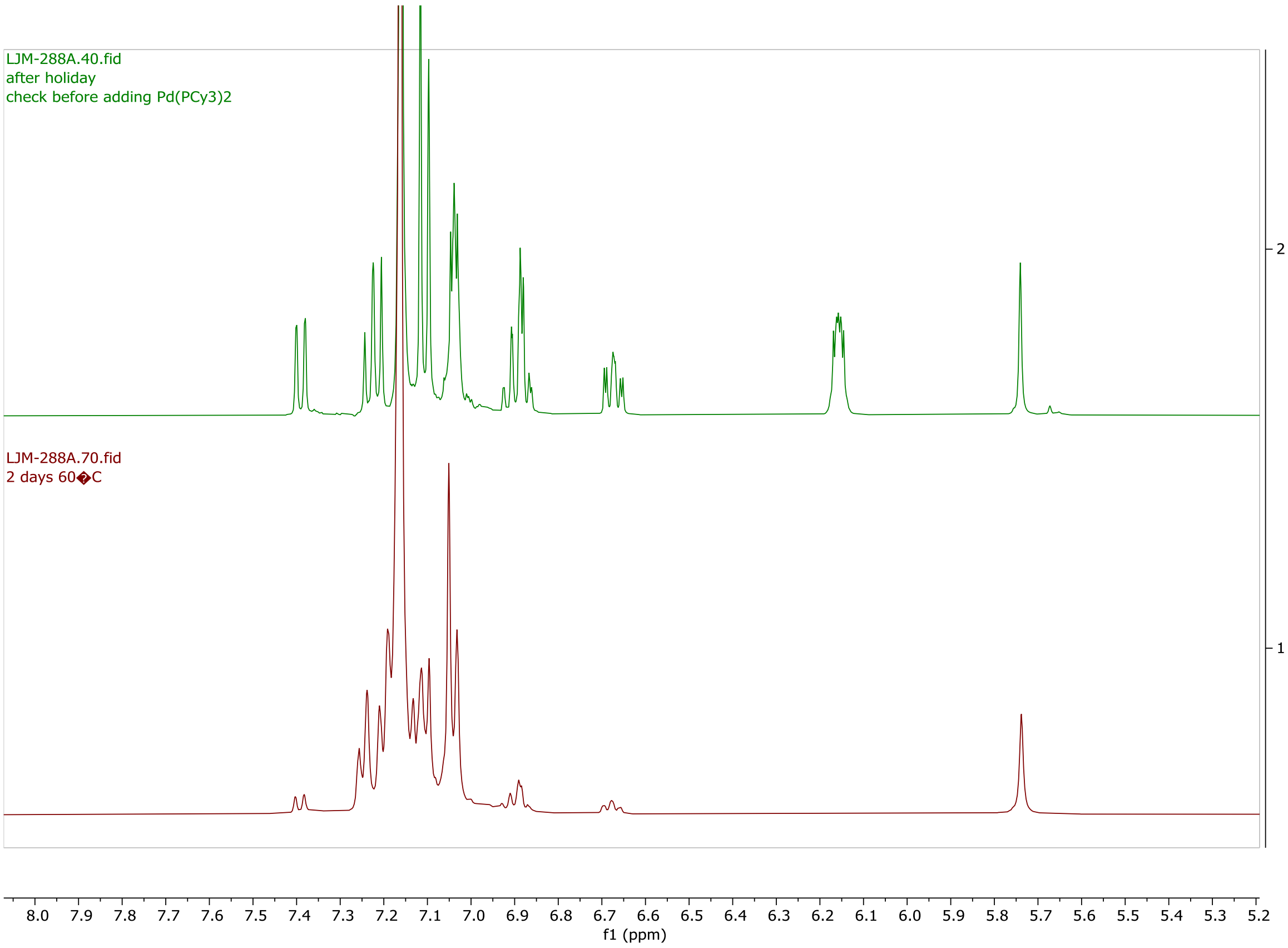

Figure S229: Expanded stacked ^{1}H NMR spectra (400 MHz, C_6D_6) before addition of [Pd(PCy_3)$_2$] (top) and after 2 days at 60°C (bottom), highlighting consumption of phenyl and partial consumption of aryl bromide.

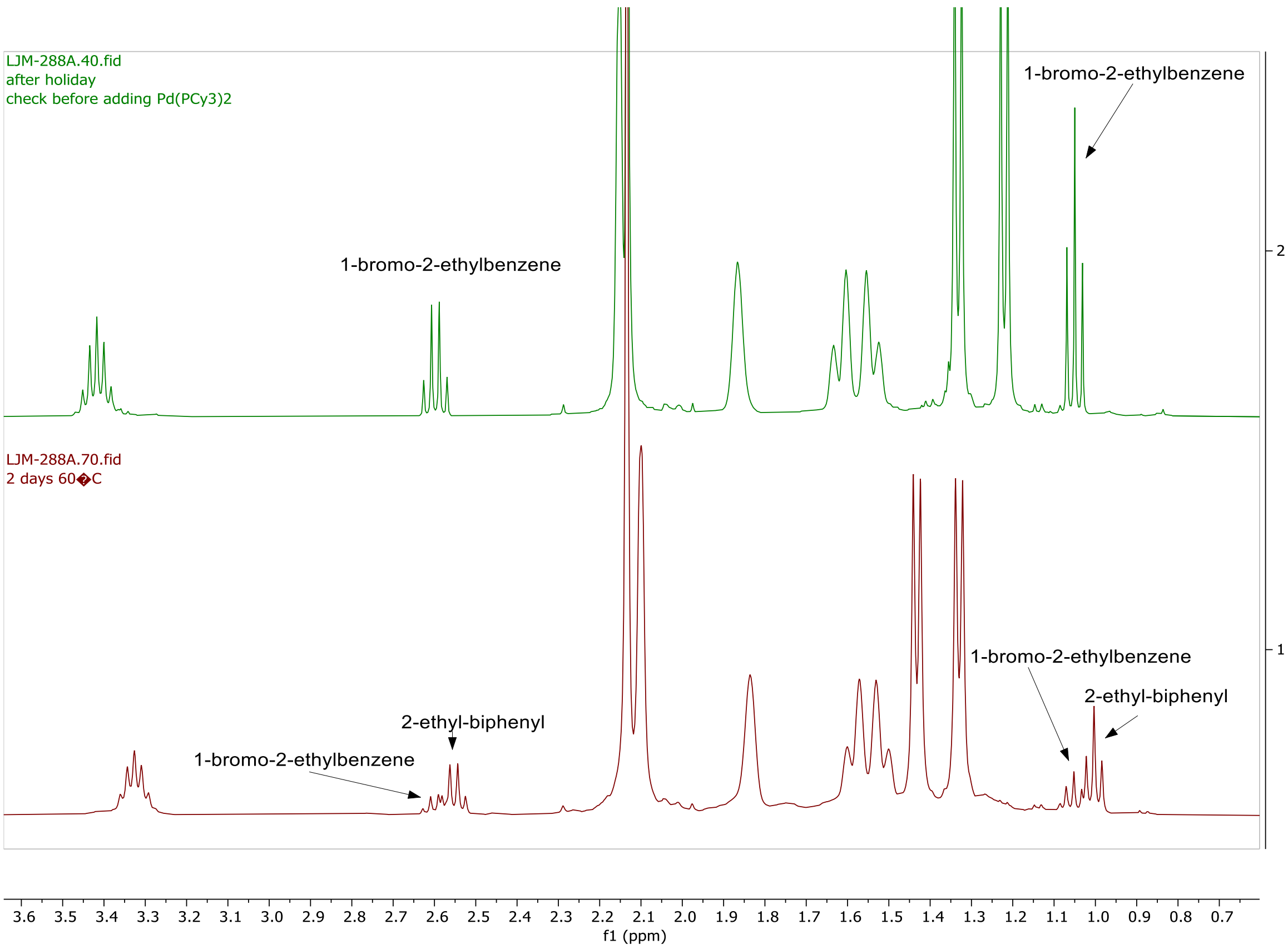

Figure S230: Expanded stacked ^{1}H NMR spectra (400 MHz, C_6D_6) before addition of [Pd(PCy_3)$_2$] (top) and after 2 days

at 60°C (bottom), highlighting consumption of phenyl, partial consumption of aryl bromide, and formation of new ethyl-containing species, assigned to 2-ethyl-biphenyl.

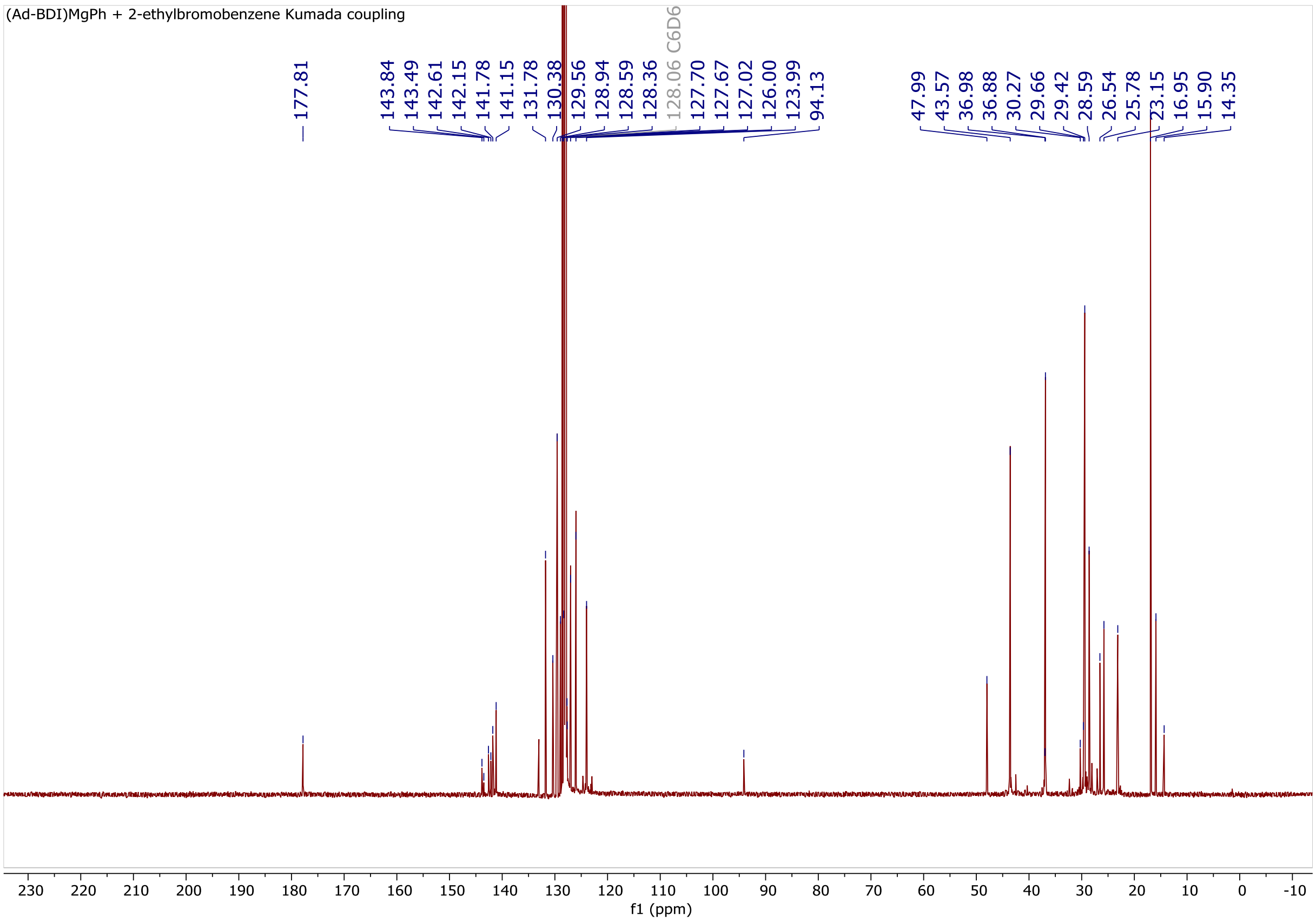

Figure S231: $^{13}C\{^{1}H\}$ NMR spectrum (126 MHz, C_6D_6) after 2 days at 60°C.

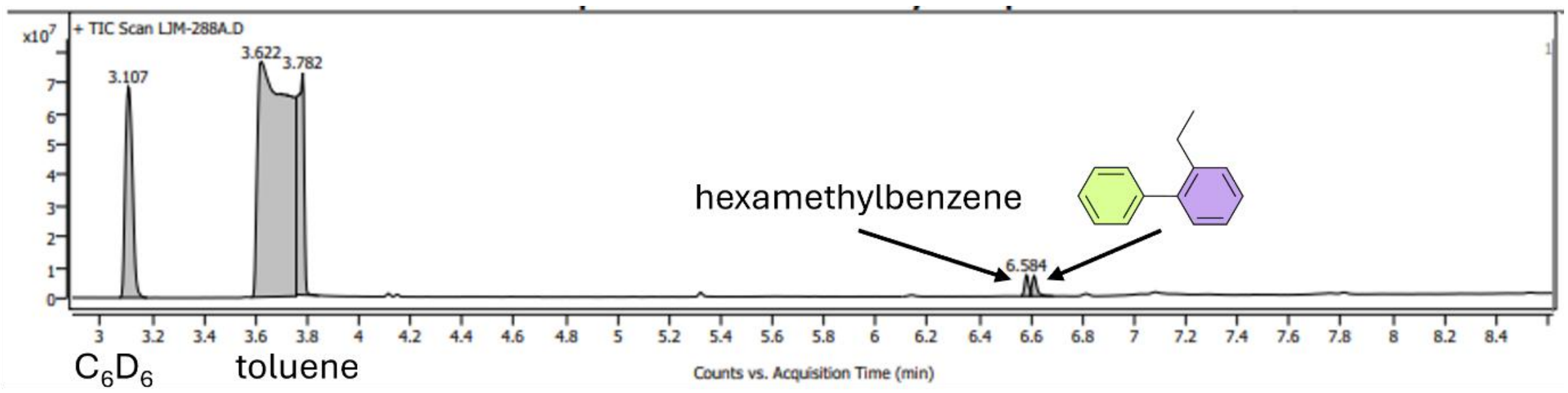

Figure S232: Gas-chromatogram of the diluted reaction mixture.

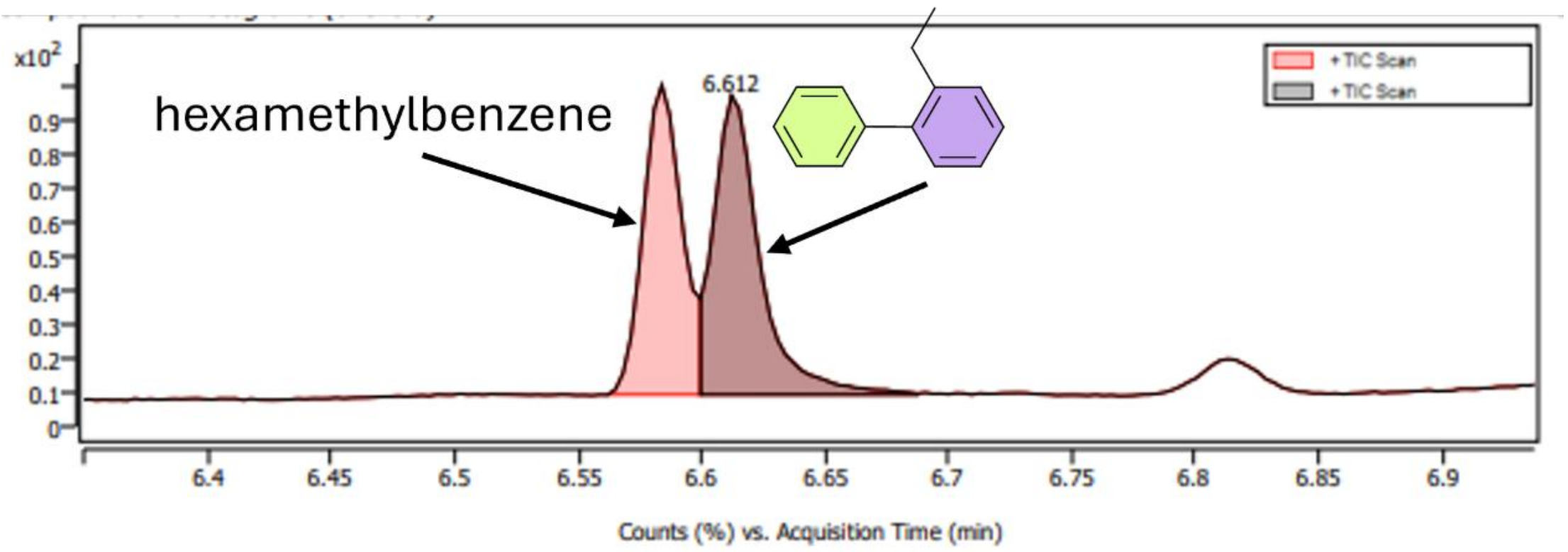

Figure S233: Expanded gas-chromatogram showing peak for 2-ethylbiphenyl.

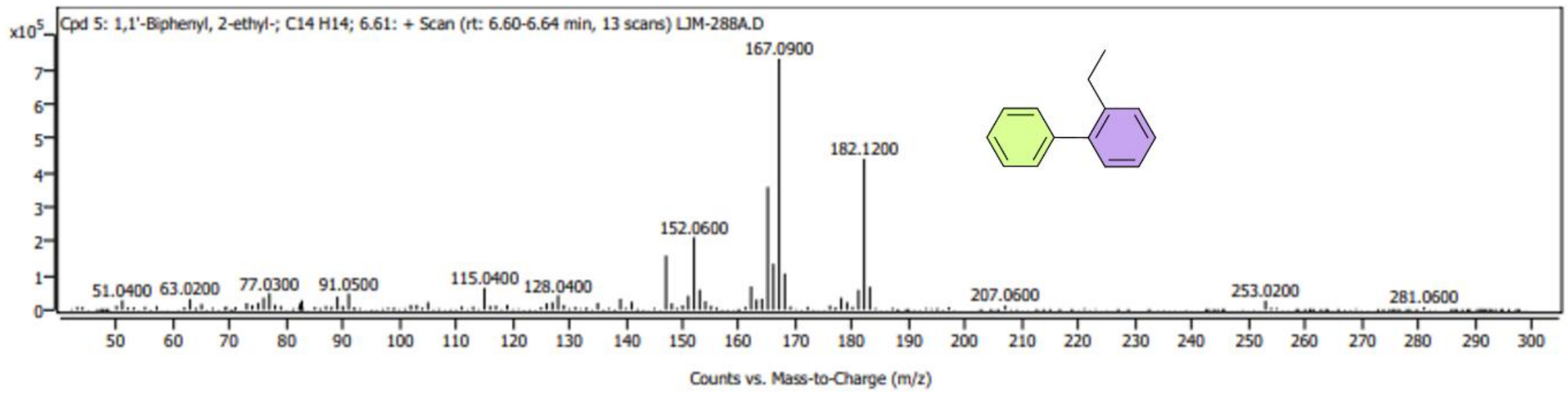

Figure S234: Mass spectrum of 2-ethylbiphenyl, showing molecular ion peak, from GC-MS analysis of the reaction mixture.

2.24.6 *Cross-coupling of anisole and bromobenzene to form 2-methoxybiphenyl*

30 mg [(^{Me}BDI)MgBu]$_2$ (**[1^{Me}]$_2$**, 0.030 mmol) was dissolved in anisole and the sealed J. Young's NMR tube was heated at 100°C for 16 hours. Volatiles were removed under vacuum and the crude product was redissolved in benzene-*d*$_6$. Analysis by ^{1}H NMR spectroscopy indicated the quantitative formation of [(^{Me}BDI)Mg(C_6H_4OMe)] (**10^{Me}**) and the presence of residual excess anisole. The solution was added to 13 mg bromobenzene (0.083 mmol) and 3.6 mg hexamethylbenzene (0.022 mmol), then 2.0 mg [Pd(PCy_3)$_2$] (0.003 mmol, 5.0 mol%). The sealed J. Young's NMR tube was heated to 60°C for 24 hours. Subsequent quantification of the product mixture by *in situ* ^{1}H NMR spectroscopy was consistent with the formation of 2-methoxybiphenyl in 98% yield.

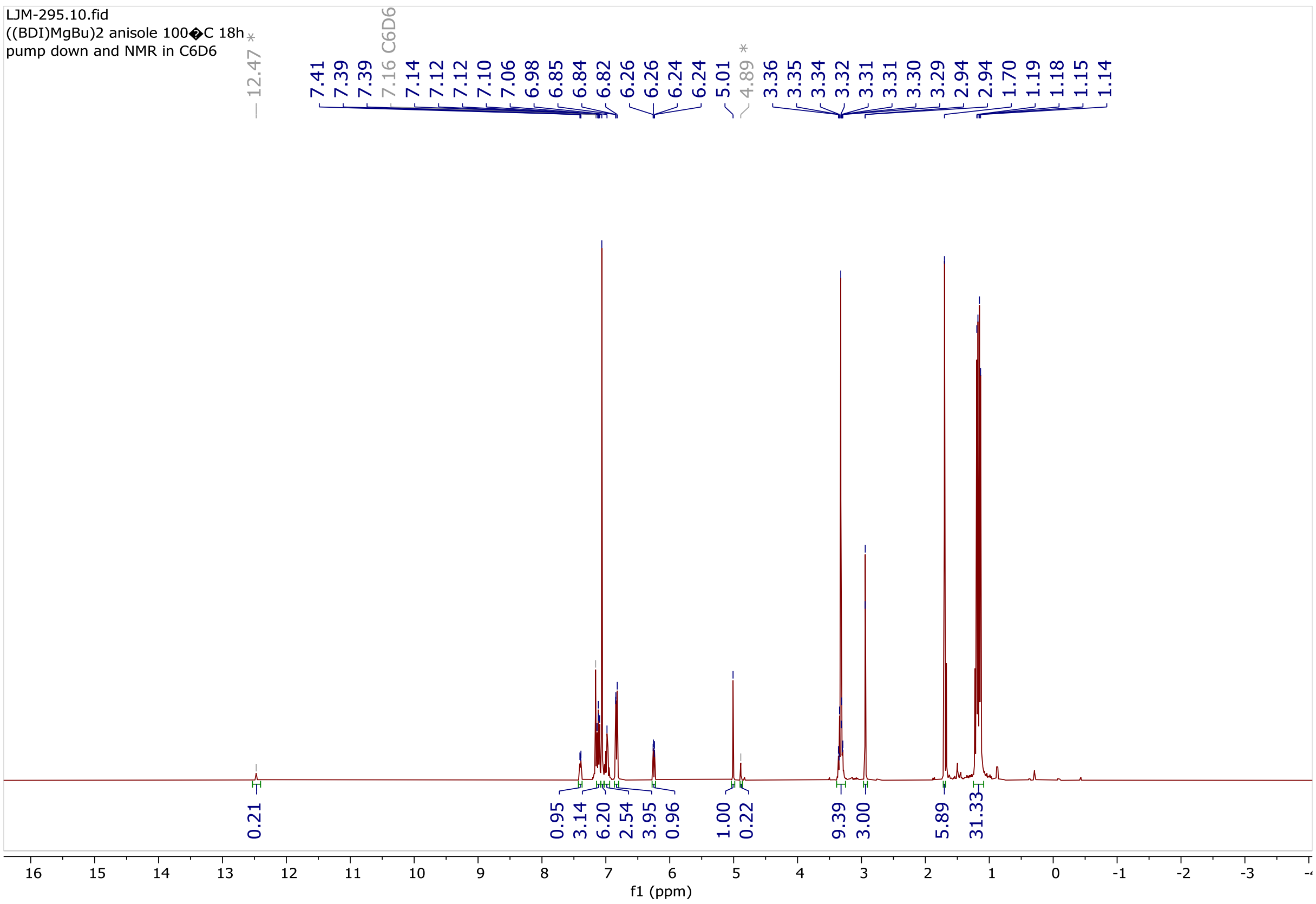

Figure S235: ^{1}H NMR (400 MHz, C_6D_6) of crude magnesium anisyl derivative (**10^{Me}**) after removal of volatiles under vacuum.*BDIH

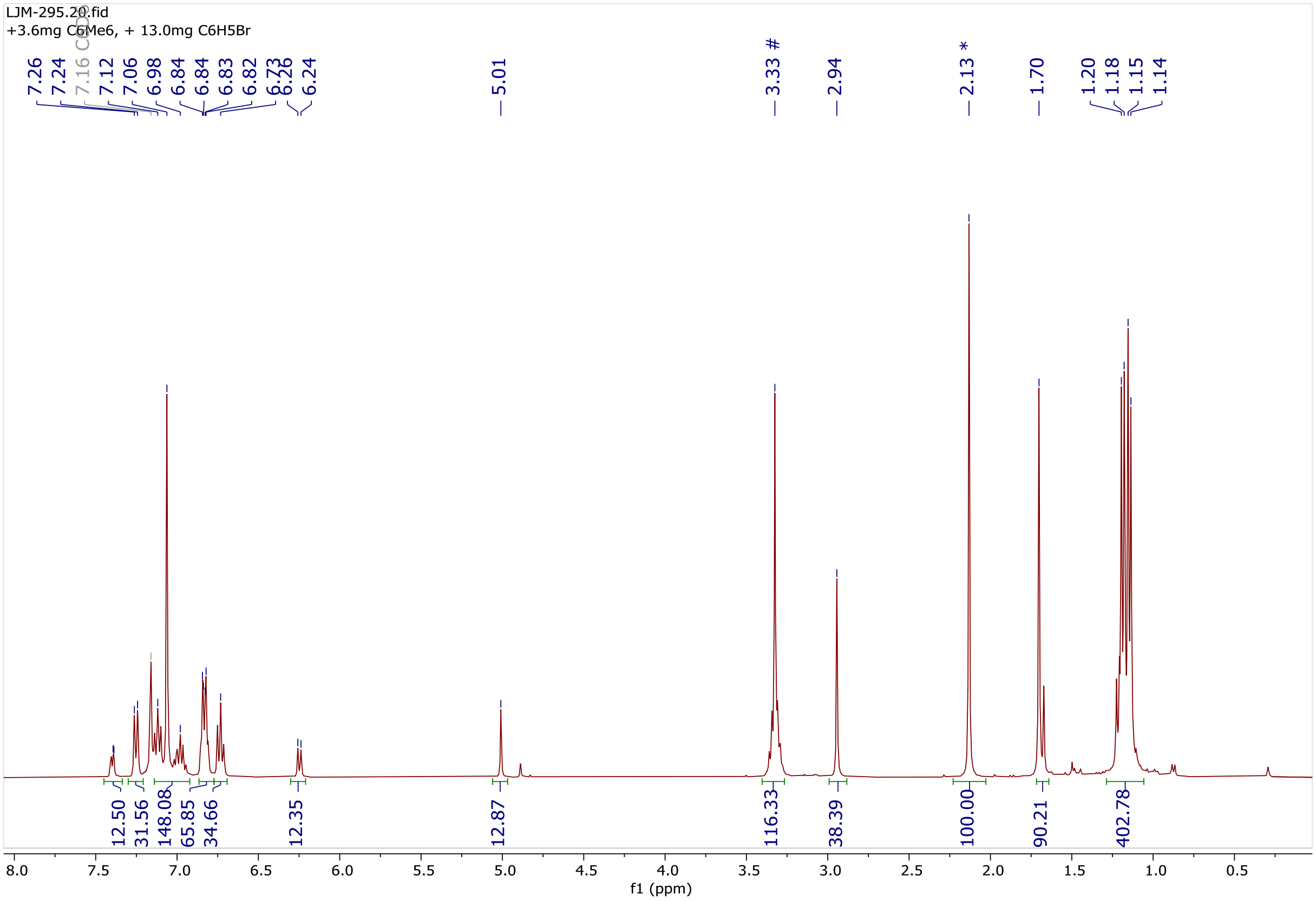

Figure S236: ^{1}H NMR (400 MHz, C_6D_6) before addition of $Pd(PCy_3)_2$. *Hexamethylbenzene. #anisole.

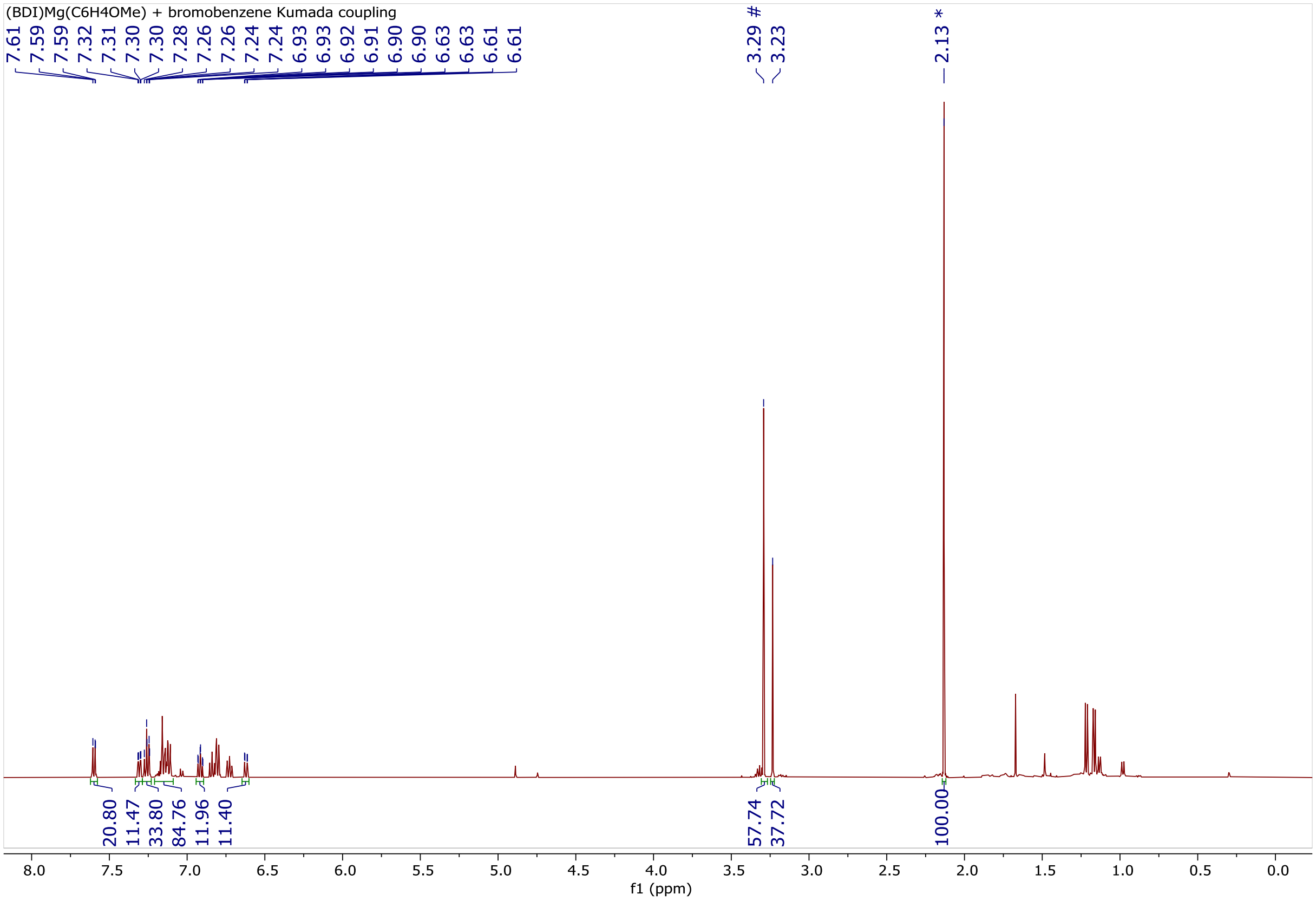

Figure S237: ^{1}H NMR (500 MHz, C_6D_6) after 2 days at 60°C. *Hexamethylbenzene. #anisole.

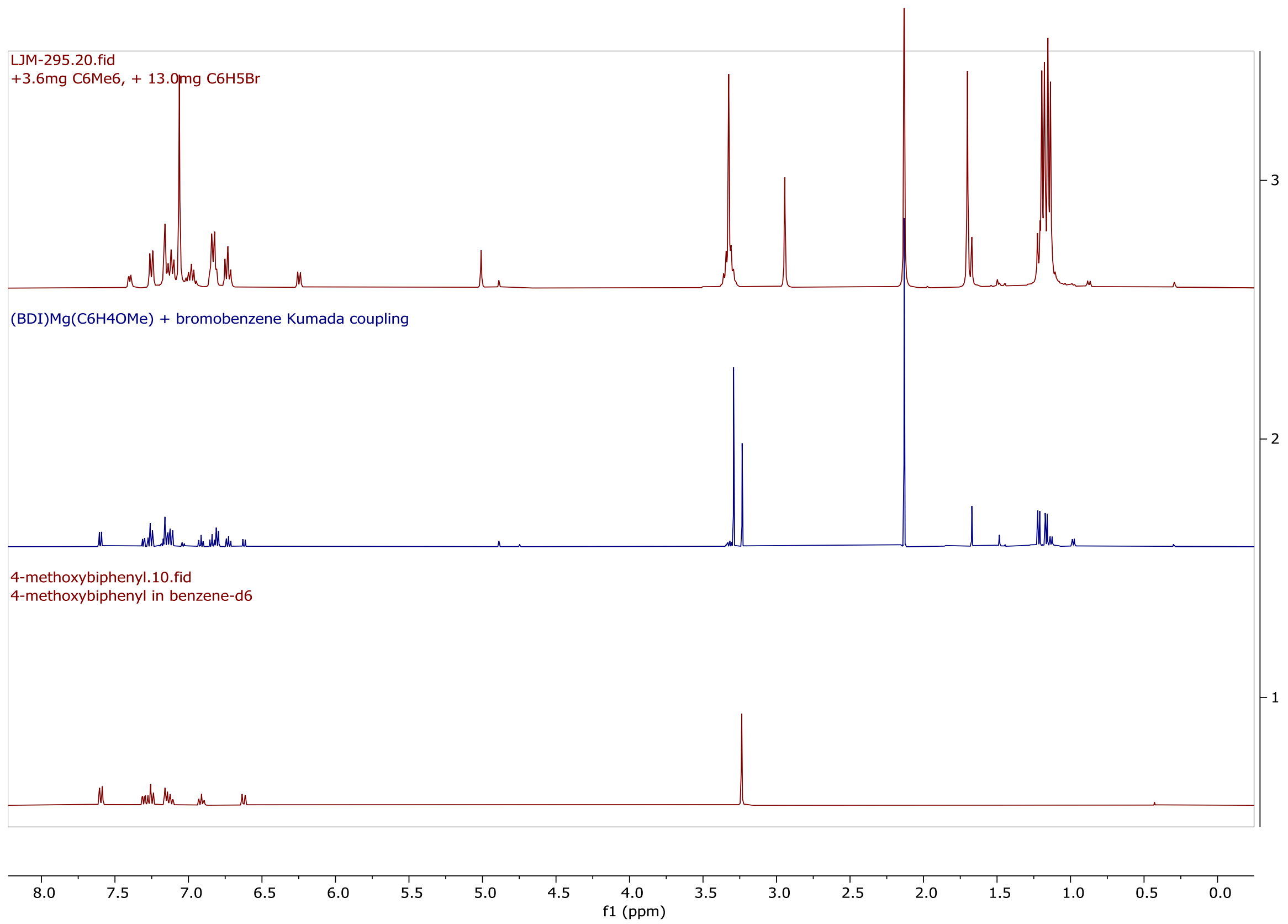

Figure S238: Stacked ^{1}H NMR spectra (C_6D_6) from top to bottom: before addition of $[Pd(PCy_3)_2]$ (400 MHz), after 2 days at 60°C (500 MHz), a separate sample of pure 2-methoxybiphenyl (500 MHz).

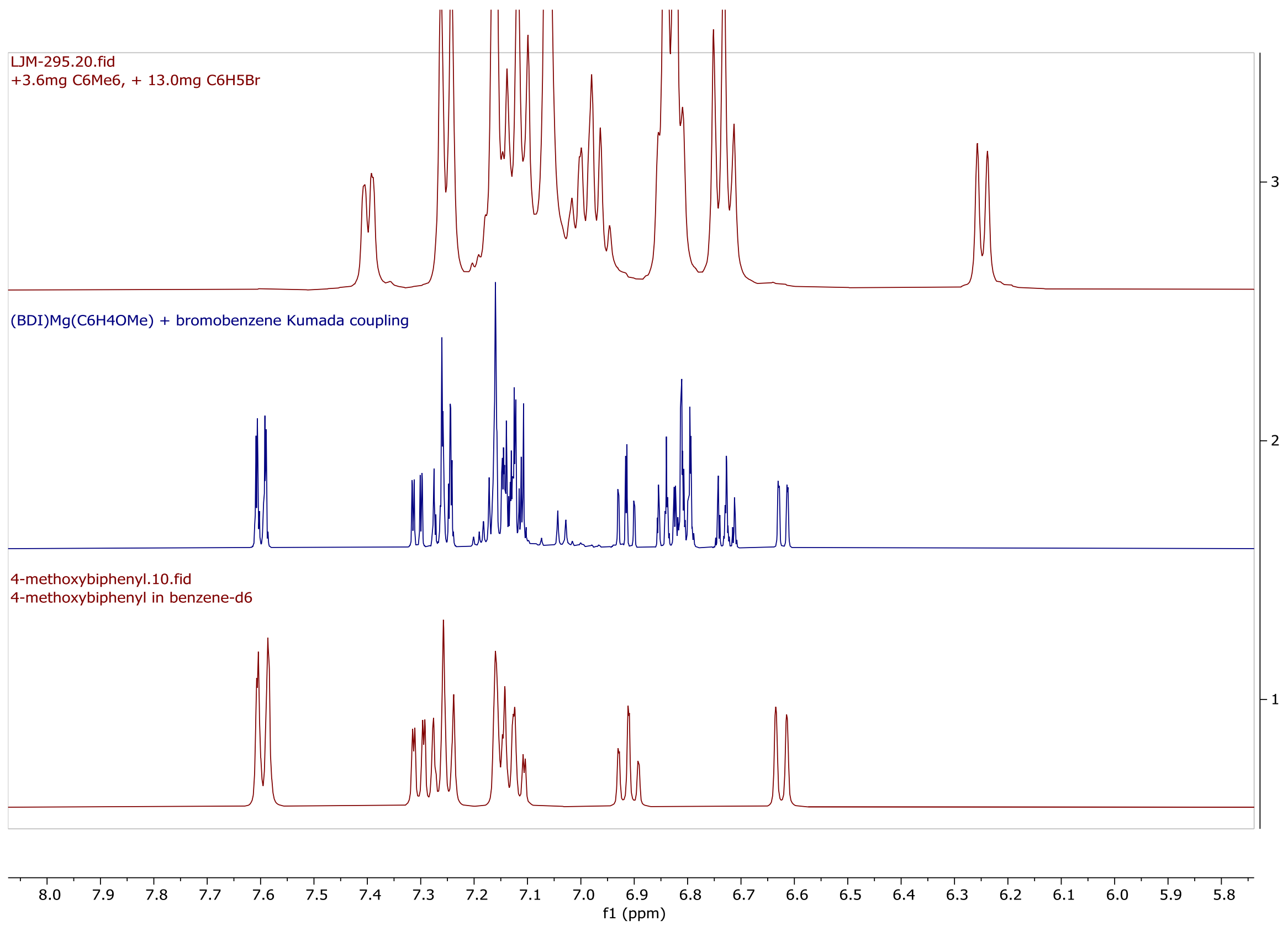

Figure S239: Expanded stacked ^{1}H NMR spectra (C_6D_6) from top to bottom: before addition of $[Pd(PCy_3)_2]$ (400 MHz), after 2 days at 60°C (500 MHz), a separate sample of pure 2-methoxybiphenyl (500 MHz).

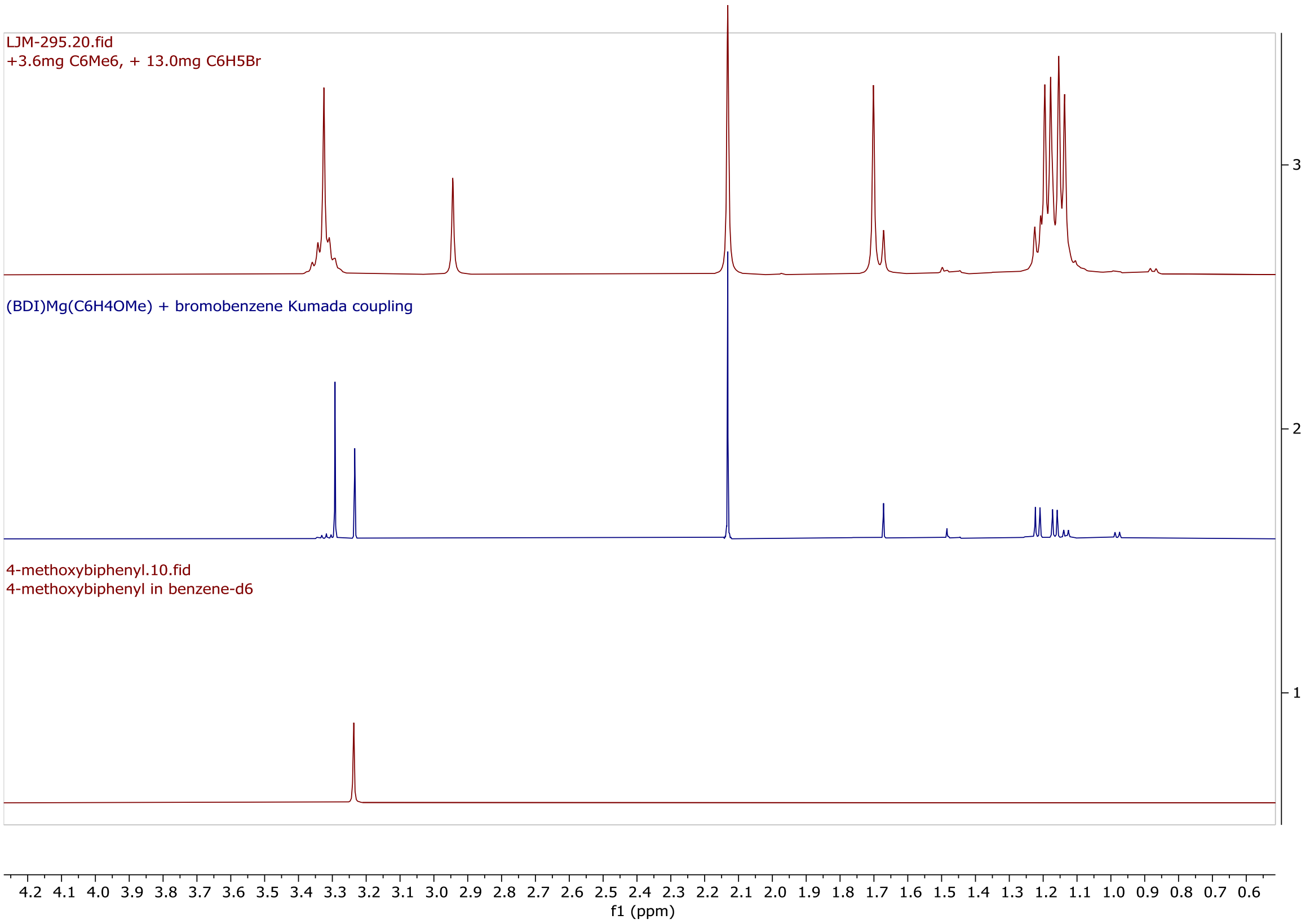

Figure S240: Expanded stacked ^{1}H NMR spectra (C_6D_6) from top to bottom: before addition of [Pd(PCy_3)$_2$] (400 MHz), after 2 days at 60°C (500 MHz), a separate sample of pure 2-methoxybiphenyl (500 MHz).

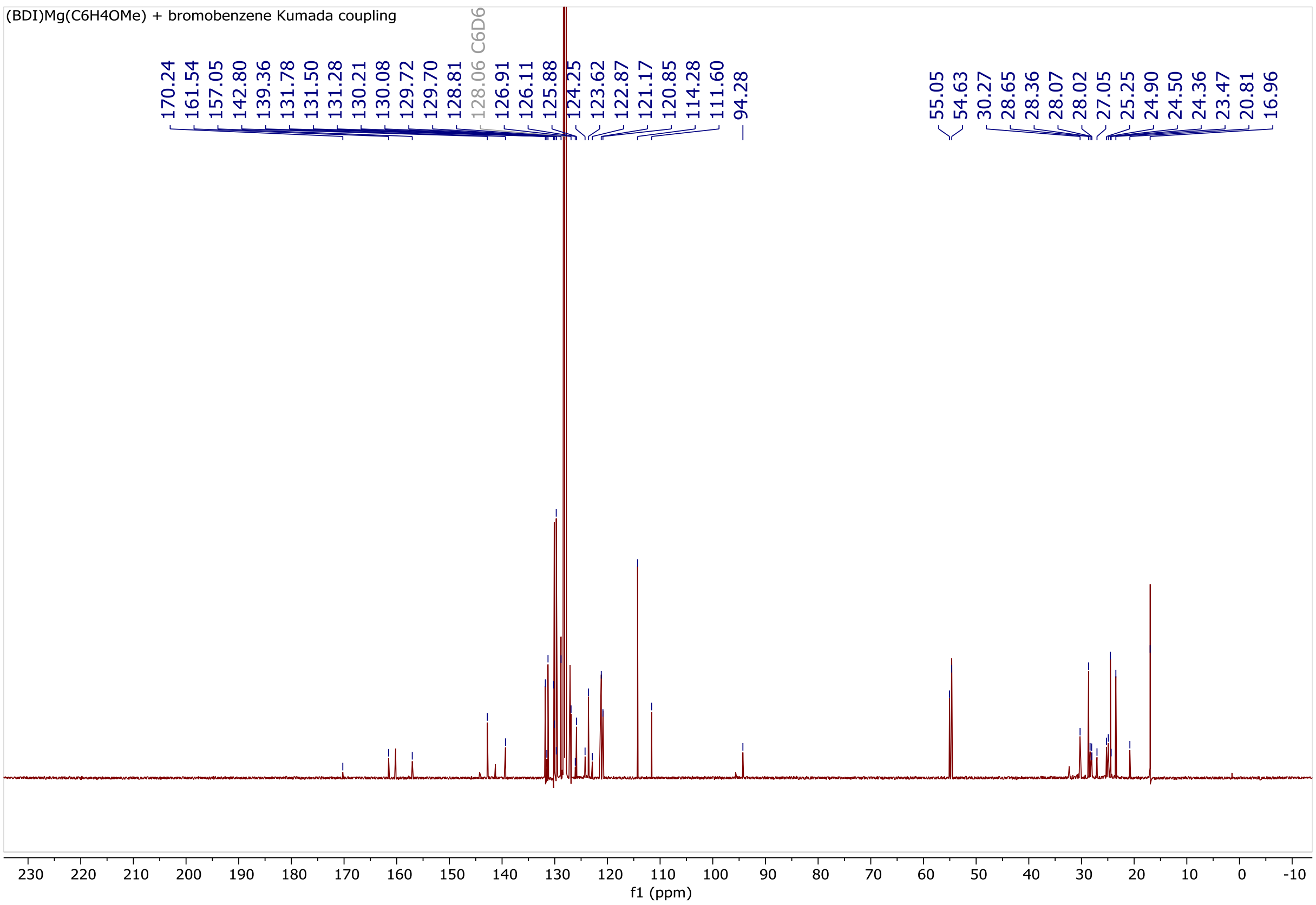

Figure S241: $^{13}C\{^{1}H\}$ NMR spectrum (126 MHz, C_6D_6) after 2 days at 60°C.

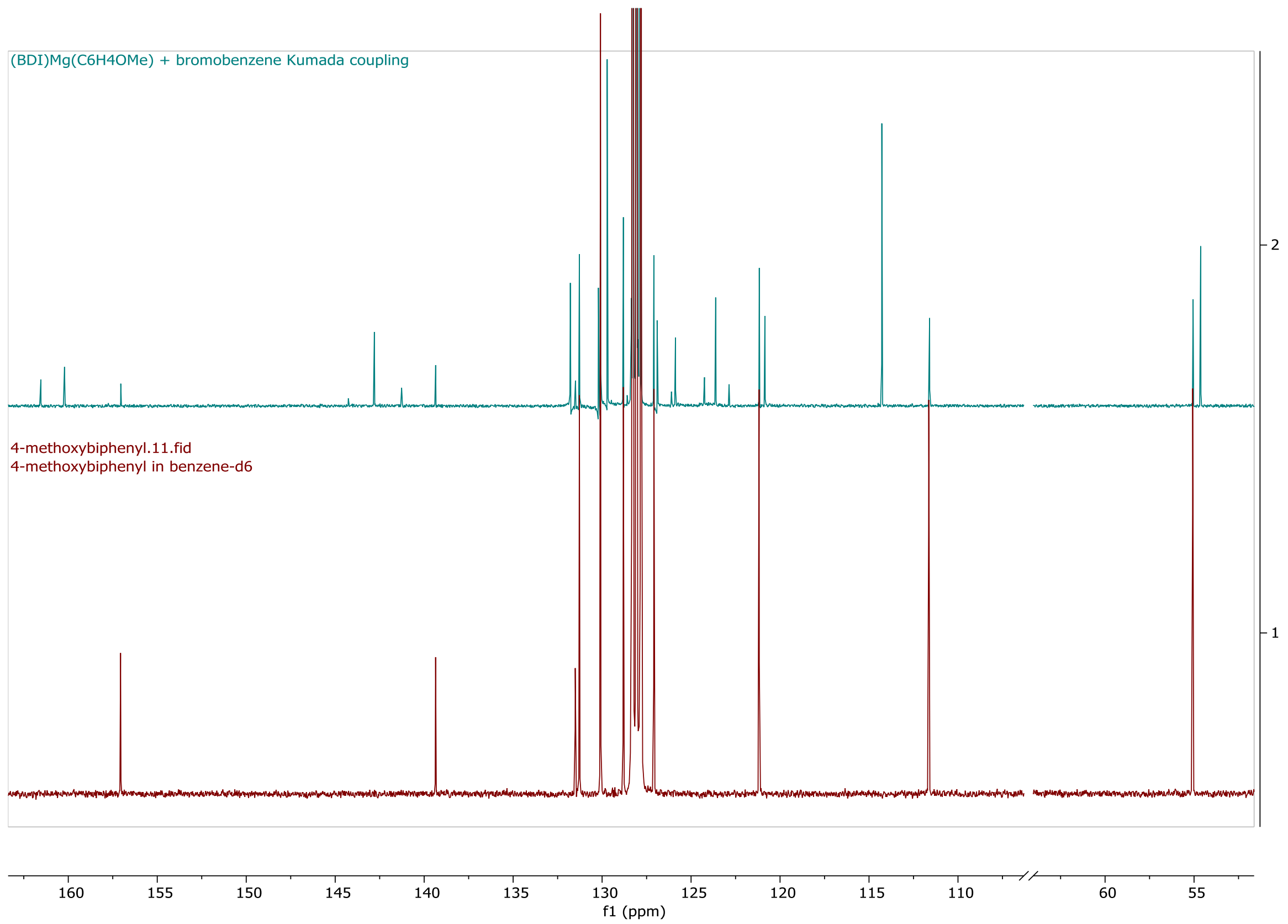

Figure S242: Expanded stacked $^{13}C\{^{1}H\}$ NMR spectra (126 MHz, C_6D_6) after 2 days at 60°C (top) compared to an authentic sample of 2-methoxy-biphenyl (bottom).

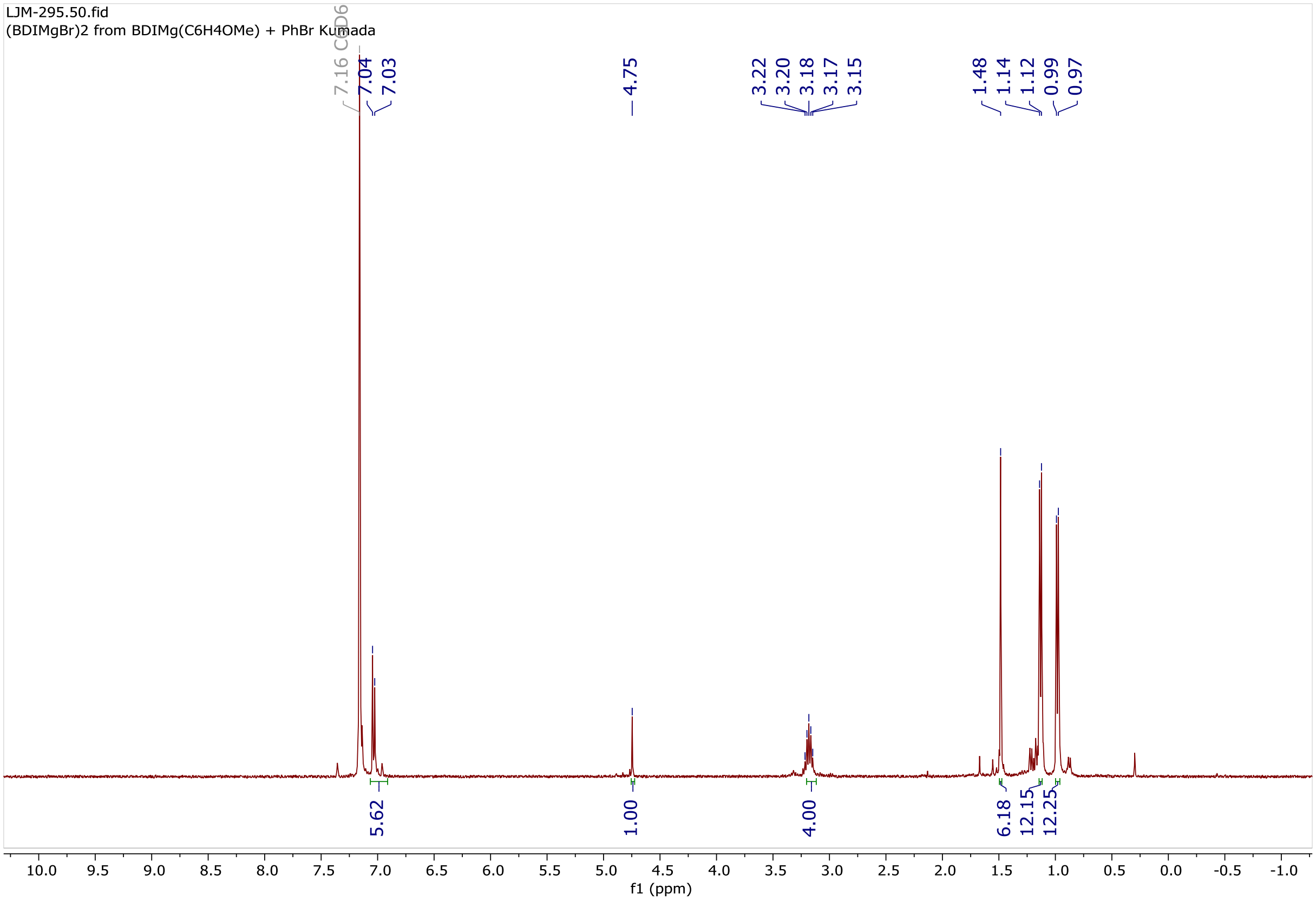

Figure S243: ^{1}H NMR spectrum (400 MHz, C_6D_6) of crystals of **13** isolated from the reaction mixture.

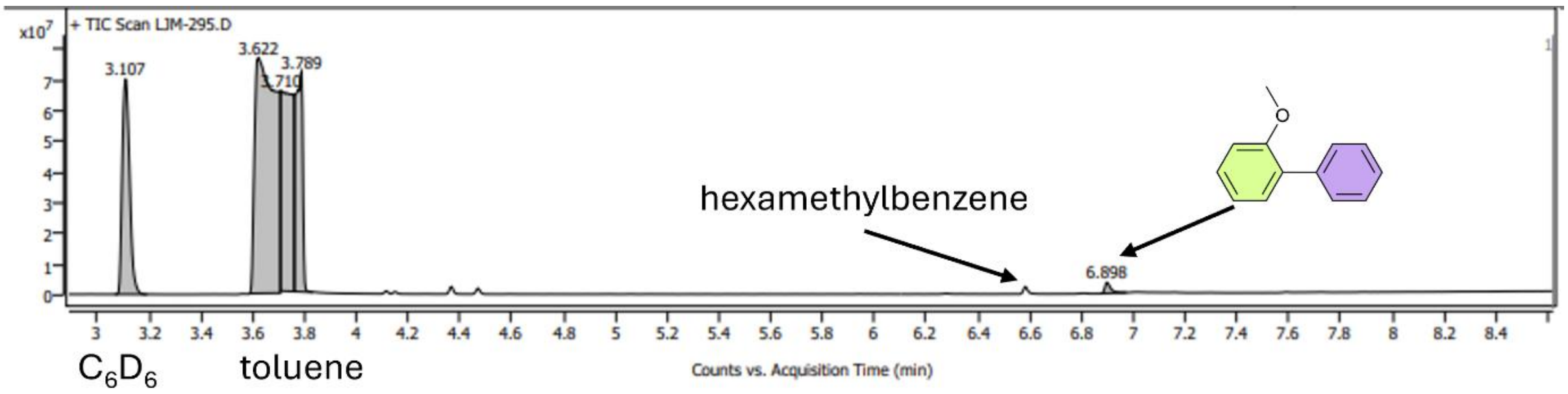

Figure S244: Gas-chromatogram of the diluted reaction mixture.

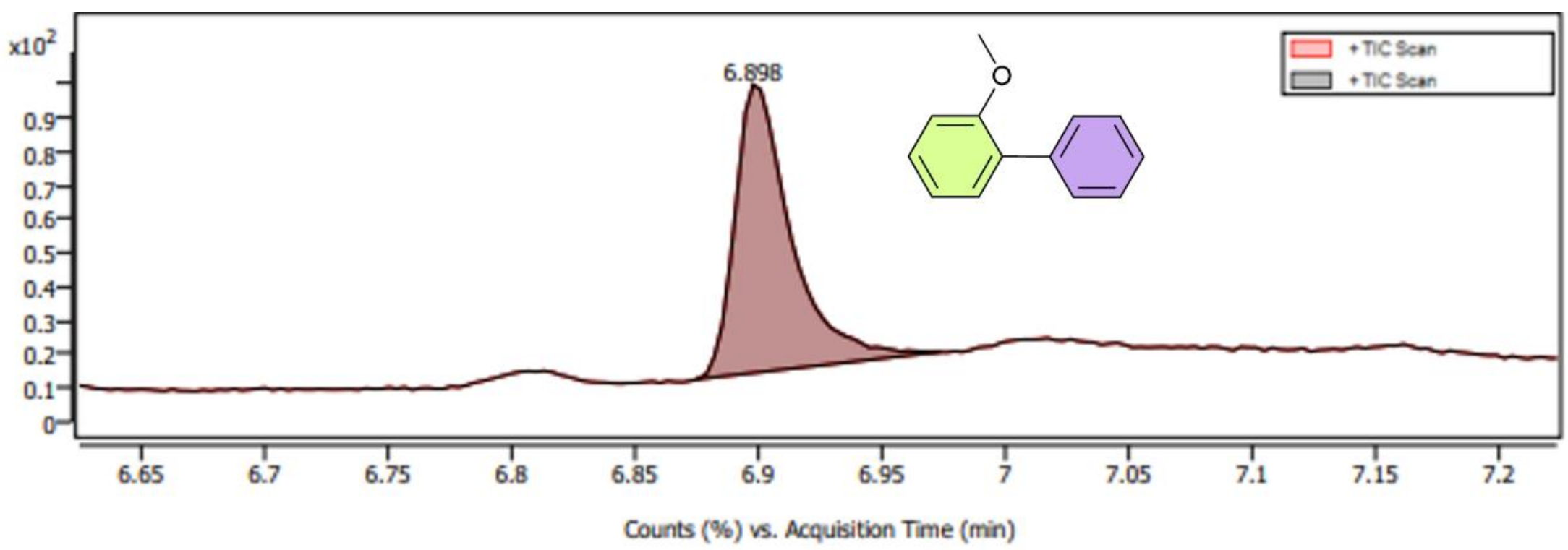

Figure S245: Expanded gas-chromatogram showing peak for 2-methoxybiphenyl.

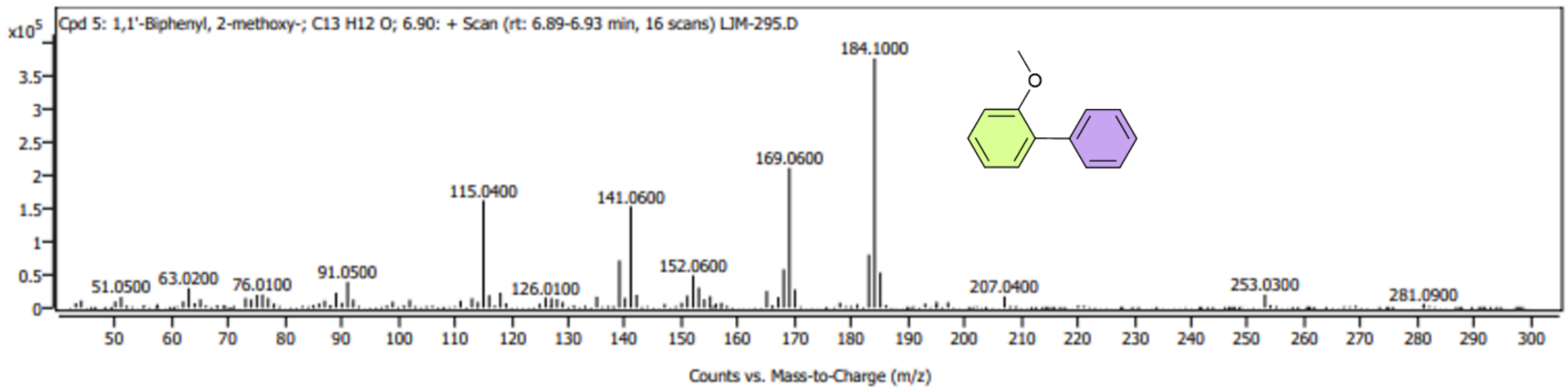

Figure S246: Mass spectrum of 2-methoxybiphenyl, showing molecular ion peak, from GC-MS analysis of the reaction mixture

2.25 Synthesis and characterisation of [(^{Ad}BDI)MgBr] (13)

Compound **13** was formed as the only BDI-containing product from the cross-coupling reactions of [(^{Ad}BDI)MgPh] (**3^{Ad}**) with aryl bromides catalysed by [Pd(PCy$_3$)$_2$]. It crystallises directly from the benzene solution of the reaction mixture. In the case of the reaction of **3^{Ad}** with PhBr catalysed by [Pd(PCy$_3$)$_2$], single crystals suitable for X-ray diffraction analysis were obtained upon cooling the hot benzene-d_6 reaction solution to room temperature and isolated in 16 mg yield (43%).

Analysis calculated for $C_{47}H_{65}BrMgN_2$: C, 74.06; H, 8.60; N, 3.68%. Found: C, 74.46; H, 8.59; N, 3.23%.

^{1}H NMR (500 MHz, C_6D_6) δ 7.11 (dd, *J* = 8.4, 6.9 Hz, 2H, *para*-Ar*H*), 7.04 (d, *J* = 7.2 Hz, 4H, *meta*-Ar*H*), 5.74 (s, 1H, *H*CC(Ad)NAr), 3.33 (hept, *J* = 6.9 Hz, 4H, *H*C(CH$_3$)$_2$), 2.10 (d, *J* = 3.0 Hz, 12H, ^{Ad}CC*H$_2$*CH), 1.85 – 1.80 (m, 6H, ^{Ad}C*H*), 1.61 – 1.54 (dt, *J* = 12.1, 3.5 Hz, 6H, ^{Ad}CHC*H$_2$*CH), 1.50 (dt, *J* = 12.4, 2.9 Hz, 6H, ^{Ad}CHC*H$_2$*CH), 1.44 (d, *J* = 6.9 Hz, 12H, HC(C*H$_3$*)$_2$), 1.32 (d, *J* = 6.8 Hz, 12H, HC(C*H$_3$*)$_2$).

^{13}C NMR (126 MHz, C_6D_6) δ 177.80 (N=*C*), 143.83 (*ipso*-N*C$_6$*H$_3$-*i*Pr$_2$), 141.14 (*ortho*-N*C$_6$*H$_3$-*i*Pr$_2$), 126.01 (*para*-N*C$_6$*H$_3$-*i*Pr$_2$), 123.99 (*meta*-N*C$_6$*H$_3$-*i*Pr$_2$), 94.12 (H*C*C(Ndipp)), 47.99 (NC^{Ad}*C*), 43.57 (^{Ad}C*CH$_2$*CH), 36.87 (^{Ad}CH*C*H$_2$CH), 29.40 (^{Ad}*C*H), 28.59 (H*C*(CH$_3$)$_2$), 25.77 (HC(*C*H$_3$)$_2$), 23.14 (HC(*C*H$_3$)$_2$).

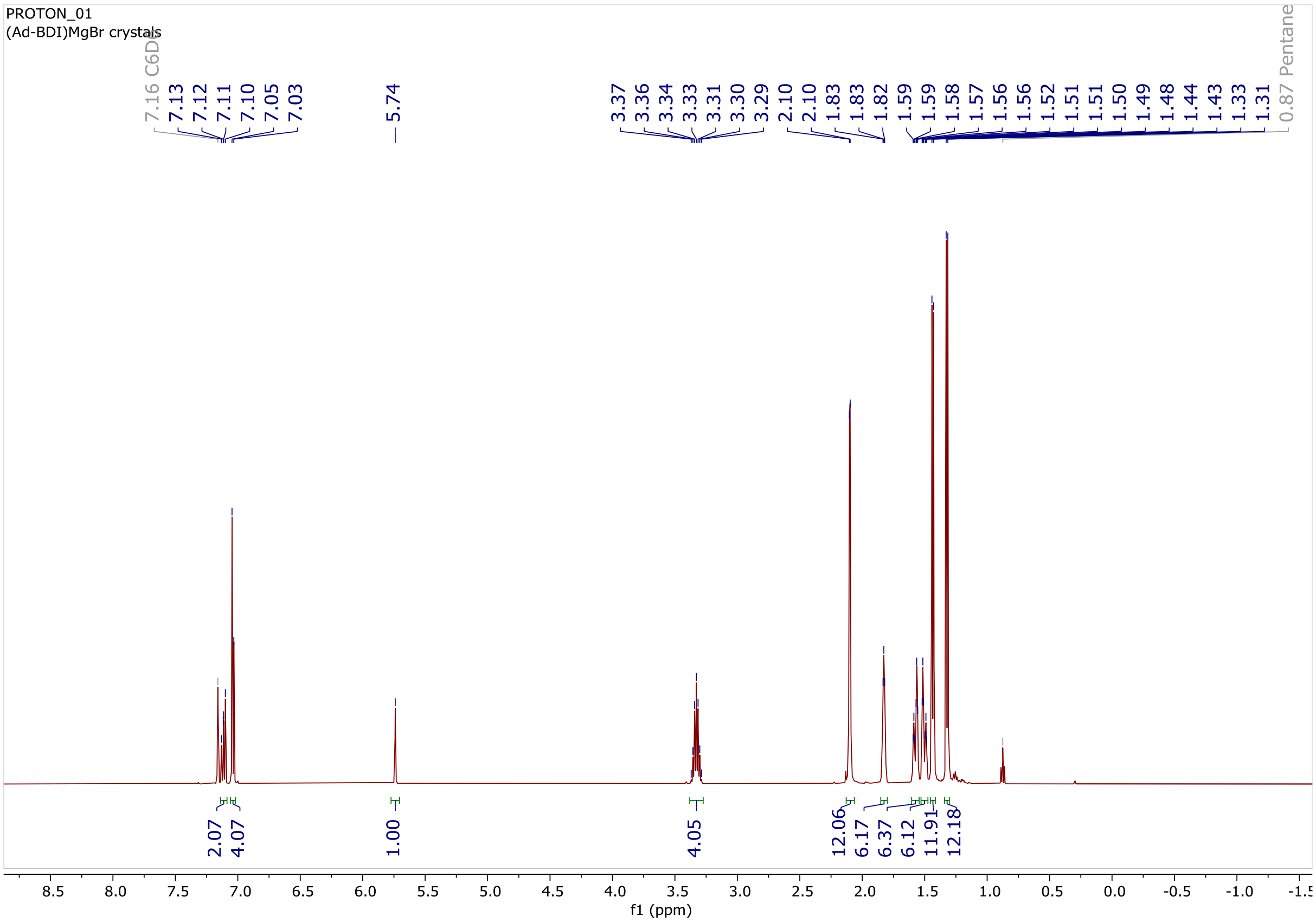

Figure S247: ^{1}H NMR (500 MHz, C_6D_6)

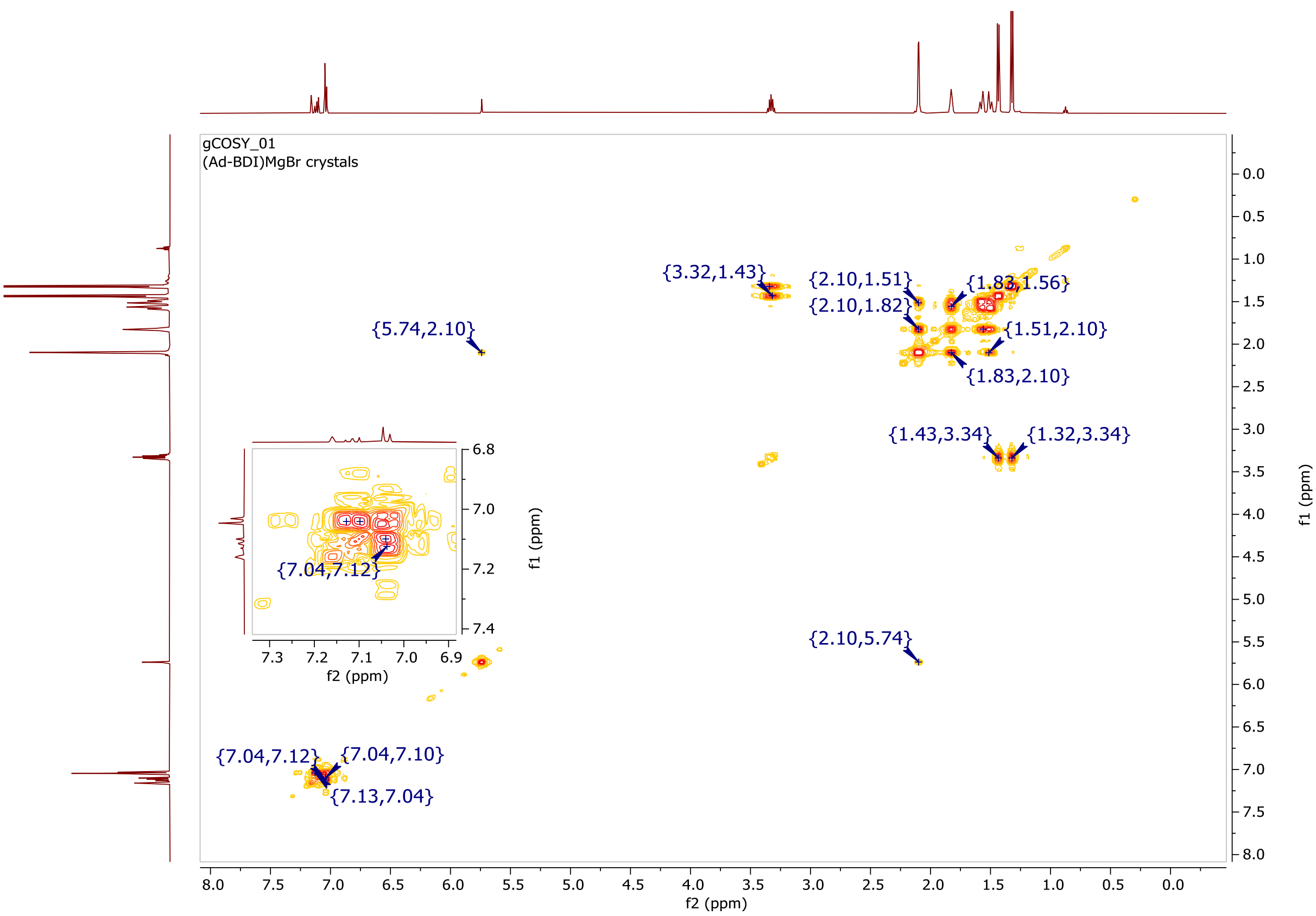

Figure S248: ^{1}H-^{1}H COSY NMR (500 MHz, C_6D_6)

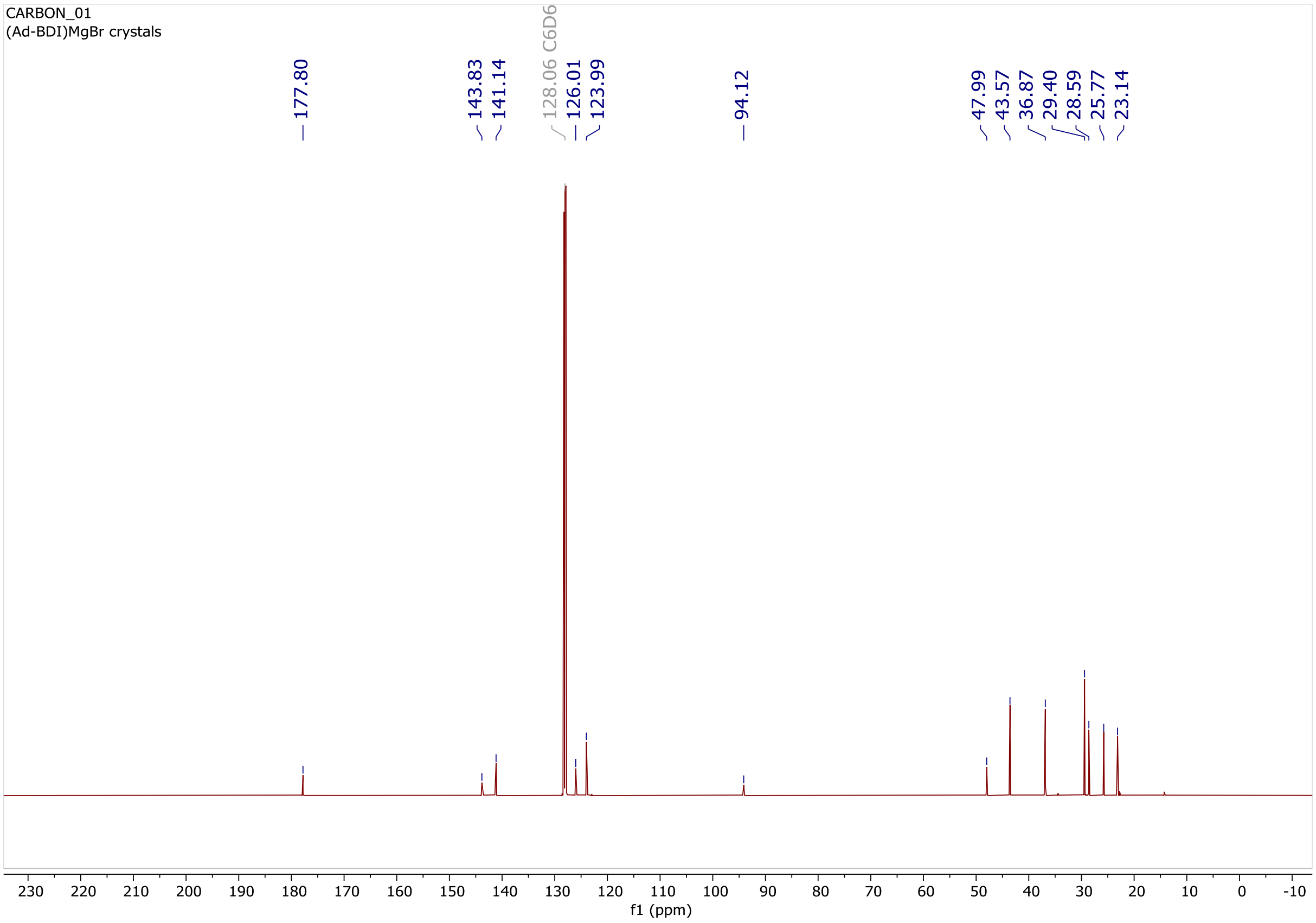

Figure S249: $^{13}C\{^{1}H\}$ NMR (126 MHz, C_6D_6).

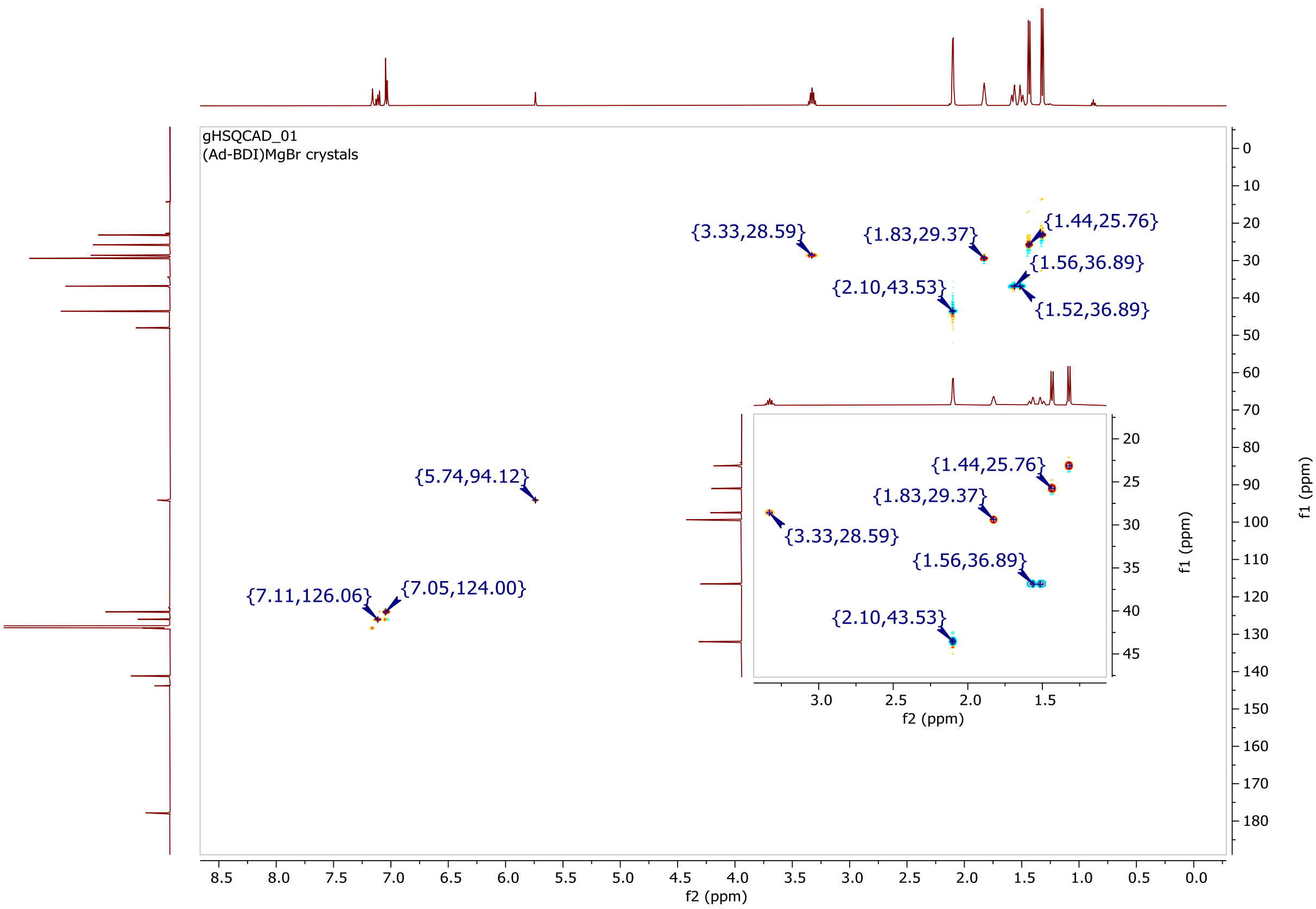

Figure S250: Edited ^{13}C-^{1}H NMR (126 MHz, C_6D_6; CH_3, CH, red; CH_2, blue).

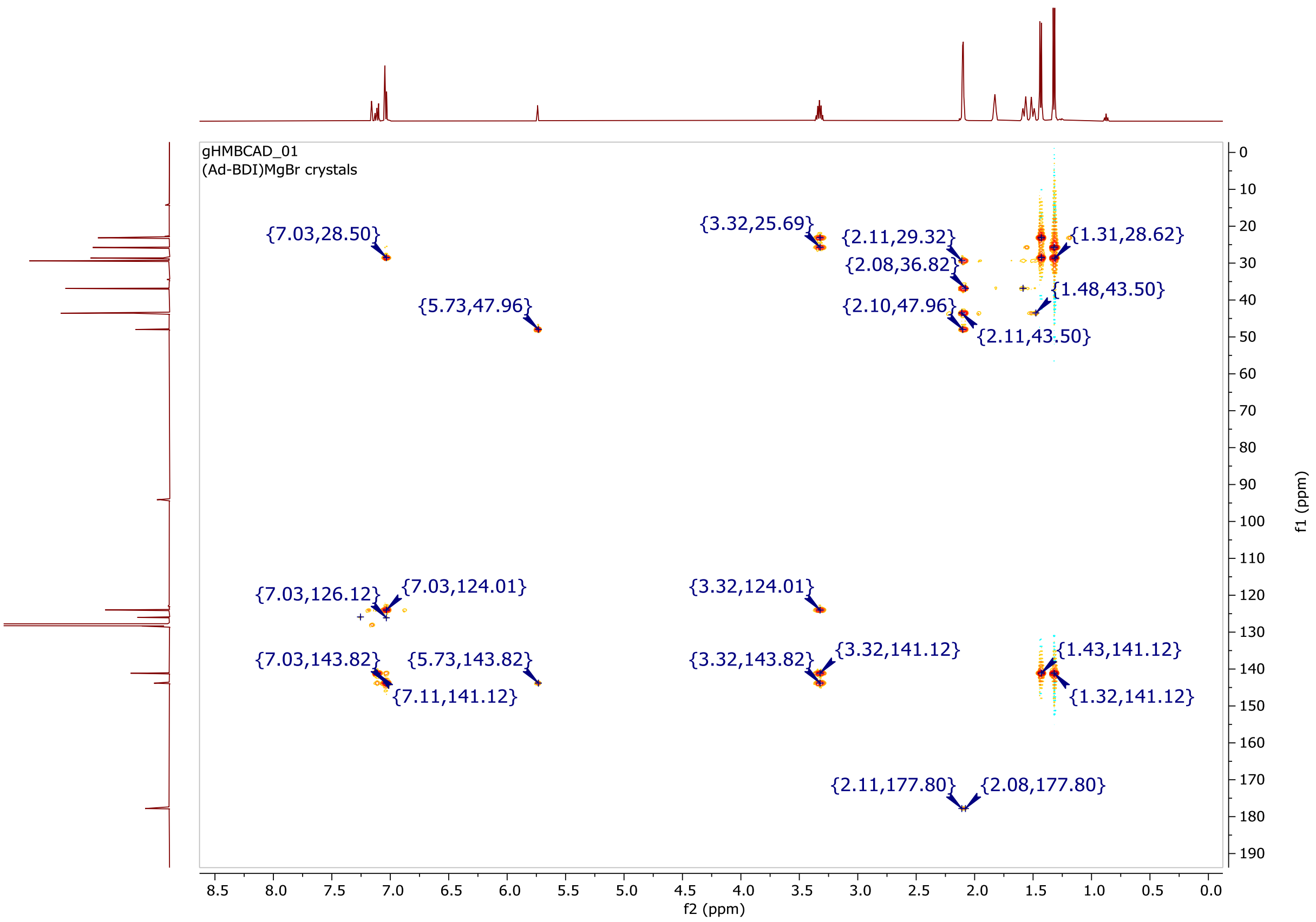

Figure S251: ^{13}C-^{1}H HMBC NMR (126 MHz, C_6D_6).

3 Steric mapping

The steric profiles of different $^{R}BDI^{Ar}$ ligands HC(C(R)NAr)$_2$ were quantified using the SambVca web tool,[7] based on the molecular structures of the corresponding monomeric magnesium *n*-butyl complexes, [($^{R}BDI^{Ar}$)Mg^{n}Bu]. For [($^{Ad}BDI^{dipp}$)Mg^{n}Bu] (**1^{Ad}**), [($^{iPr}BDI^{dipp}$)Mg^{n}Bu] (**1^{dipp,iPr}**),[8] [($^{tBu}BDI^{dipp}$)Mg^{n}Bu] (**1^{dipp,tBu}**),[9] [($^{tBu}BDI^{dipep}$)Mg^{n}Bu] (**1^{dipep,tBu}**),[10] atomic coordinates from the published X-ray crystal structures were used, whilst for [($^{Me}BDI^{dipp}$)Mg^{n}Bu] (**1^{Me}**), the optimised coordinates from the calculated structure were used instead, as the magnesium *n*-butyl complex with this ligand crystallises as a dimer.[11] Atomic radii were used as 1.17 multiplied by the Bondi radii. %V_{bur} were calculated for a 3.5 Å radius sphere and a mesh spacing of 0.10 Å. Hydrogen atoms were included in the calculations. The z-axis was defined by the magnesium centre and the central γ-carbon atom of the BDI ligand backbone, whilst the xz plane was defined by the nitrogen atoms. The centre of the sphere was either defined as the magnesium centre or, in an effort to negate the effects of different Mg-N bond lengths across the series, as a point positioned 1.36 Å perpendicular from the N-N axis, in the positive z-direction. Besides minor differences in the final values, both descriptions provided very similar results.

Table S1: Volume calculations for a 3.5 Å sphere centred on the magnesium centre.

	%V Free	**%V Buried**	% V Tot/V Ex
$^{Me}BDI^{dipp}$	39.7	**60.3**	99.9
$^{iPr}BDI^{dipp}$	37.4	**62.6**	99.9
$^{Ad}BDI^{dipp}$	34.3	**65.7**	99.9
$^{tBu}BDI^{dipp}$	32.6	**67.4**	99.9
$^{tBu}BDI^{dipep}$	34.0	**66.0**	99.9

Table S2: Volume calculations for a 3.5 Å sphere centred on a point positioned 1.36 Å perpendicular from the N-N axis, in the positive z-direction.

	%V Free	**%V Buried**	% V Tot/V Ex
$^{Me}BDI^{dipp}$	39.0	**61.0**	99.9
$^{iPr}BDI^{dipp}$	37.1	**62.9**	99.9
$^{Ad}BDI^{dipp}$	34.2	**65.8**	99.9
$^{tBu}BDI^{dipp}$	32.5	**67.5**	99.9
$^{tBu}BDI^{dipep}$	33.9	**66.1**	99.9

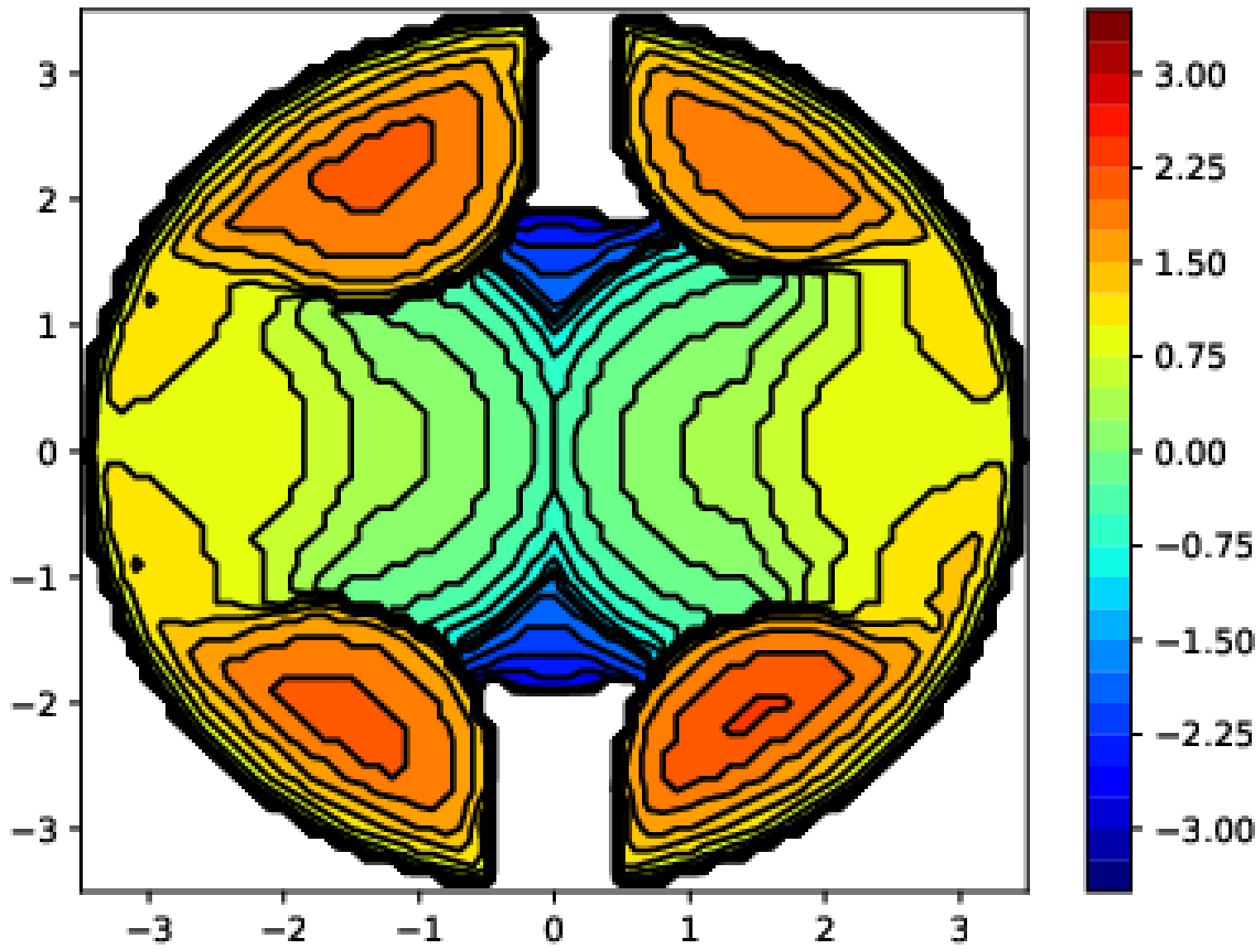

Figure S252: ^{Me}BDI^{dipp} steric map centred on the magnesium centre.

Table S3: Table of parameters for the ^{Me}BDI^{dipp} steric map centred on the magnesium centre

Quadrant	V f	V b	V t	%V f	**%V b**
SW	18.3	26.6	44.9	**40.7**	59.3
NW	16.2	28.7	44.9	**36.0**	64.0
NE	19.1	25.8	44.9	**42.5**	57.5
SE	17.8	27.0	44.9	**39.7**	60.3

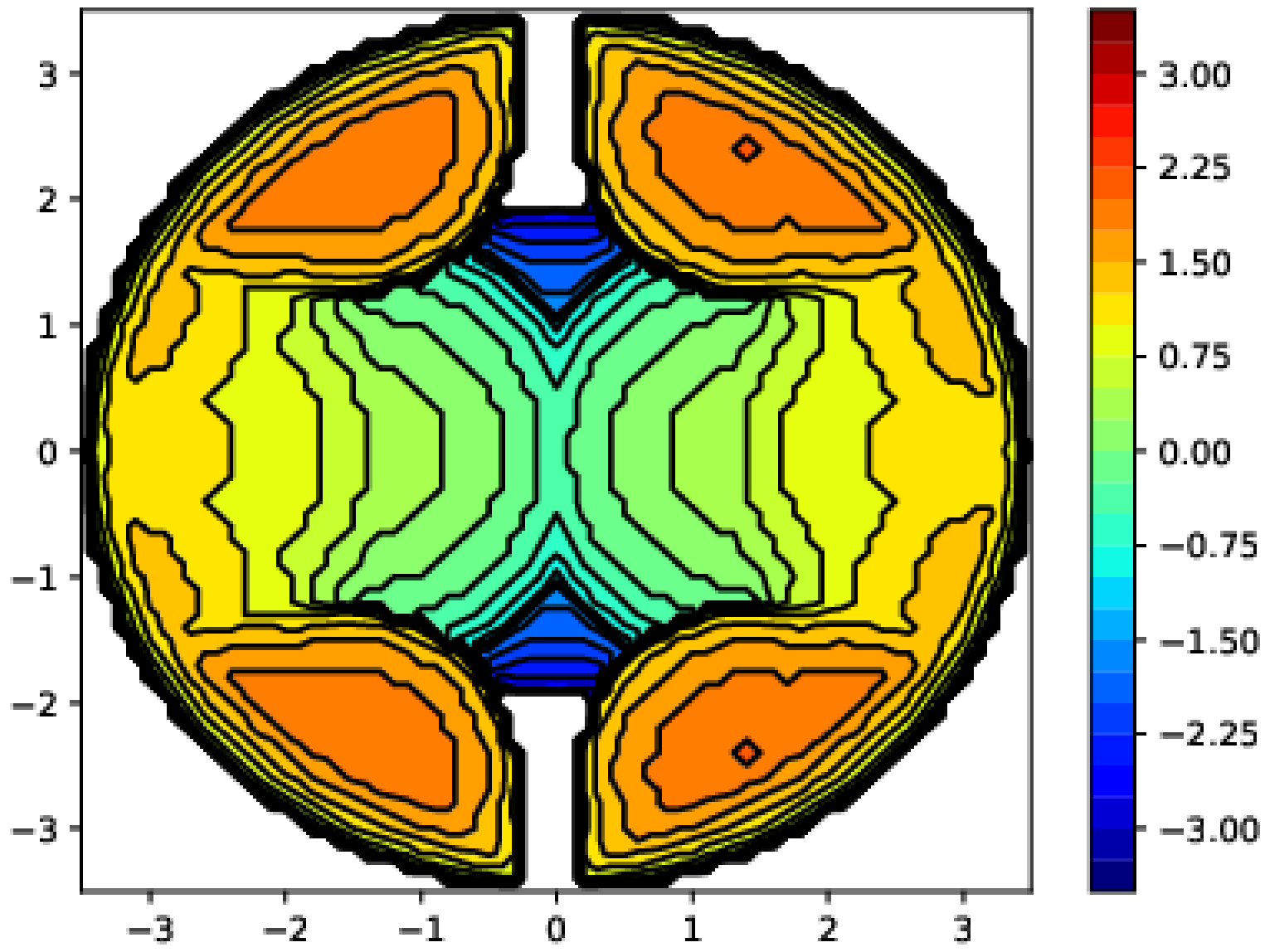

Figure S253: $^{iPr}BDI^{dipp}$ steric map centred on the magnesium centre.

Table S4: Table of parameters for the $^{iPr}BDI^{dipp}$ steric map centred on the magnesium centre

Quadrant	V f	V b	V t	%V f	**%V b**
SW	17.1	27.7	44.9	**38.2**	61.8
NW	17.1	27.7	44.9	**38.2**	61.8
NE	16.4	28.4	44.9	**36.6**	63.4
SE	16.4	28.4	44.9	**36.6**	63.4

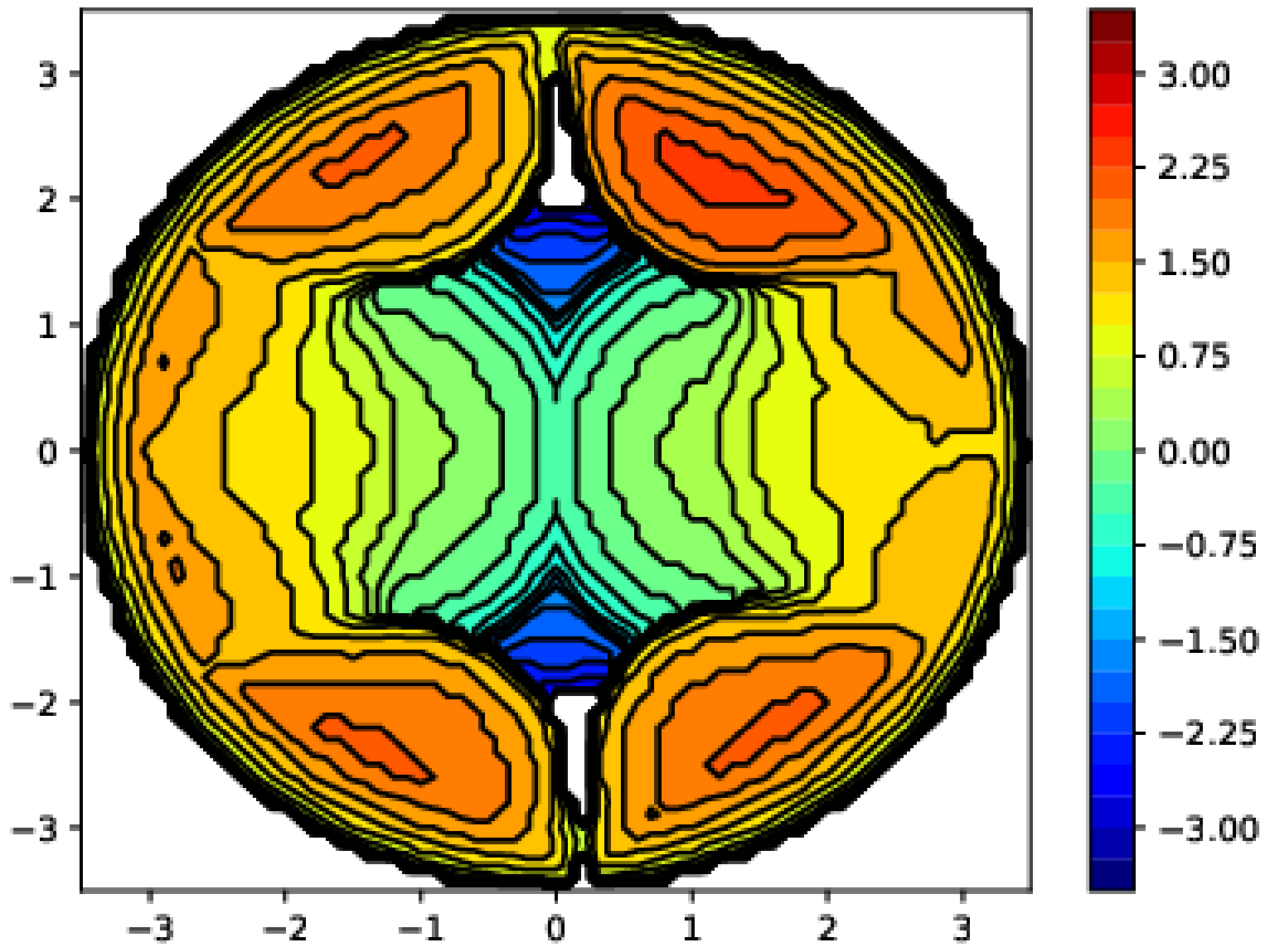

Figure S254: ^{Ad}BDI^{dipp} steric map centred on the magnesium centre.

Table S5: Table of parameters for the ^{Ad}BDI^{dipp} steric map centred on the magnesium centre

Quadrant	V f	V b	V t	%V f	**%V b**
SW	14.6	30.3	44.9	**32.6**	67.4
NW	15.8	29.0	44.9	**35.3**	64.7
NE	14.6	30.2	44.9	**32.6**	67.4
SE	16.5	28.3	44.9	**36.8**	63.2

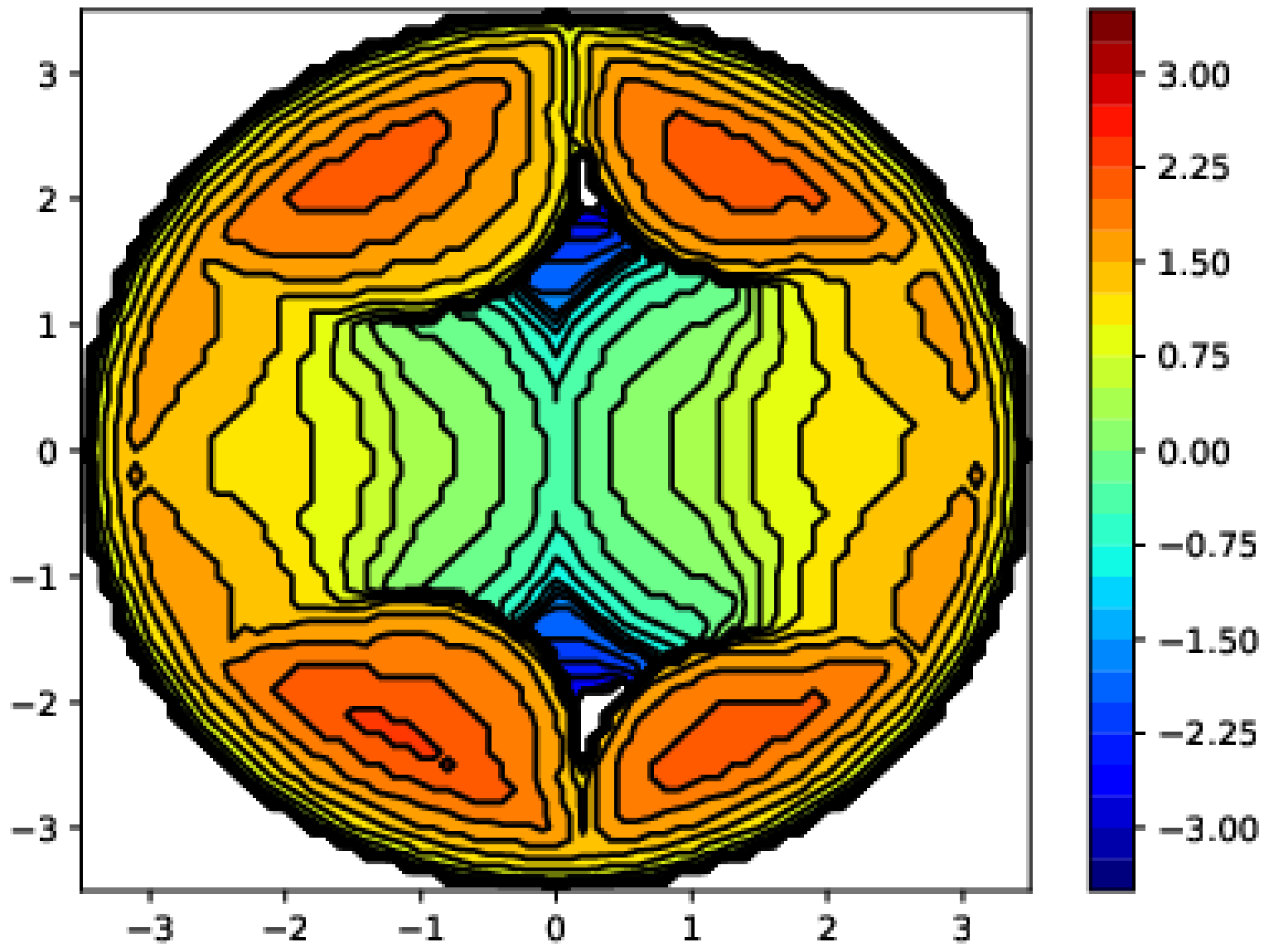

Figure S255: ^{tBu}BDI^{dipp} steric map centred on the magnesium centre.

Table S6: Table of parameters for the ^{tBu}BDI^{dipp} steric map centred on the magnesium centre

Quadrant	V f	V b	V t	%V f	**%V b**
SW	13.0	31.8	44.9	**29.1**	70.9
NW	13.9	30.9	44.9	**31.0**	69.0
NE	15.6	29.2	44.9	**34.8**	65.2
SE	15.9	29.0	44.9	**35.4**	64.6

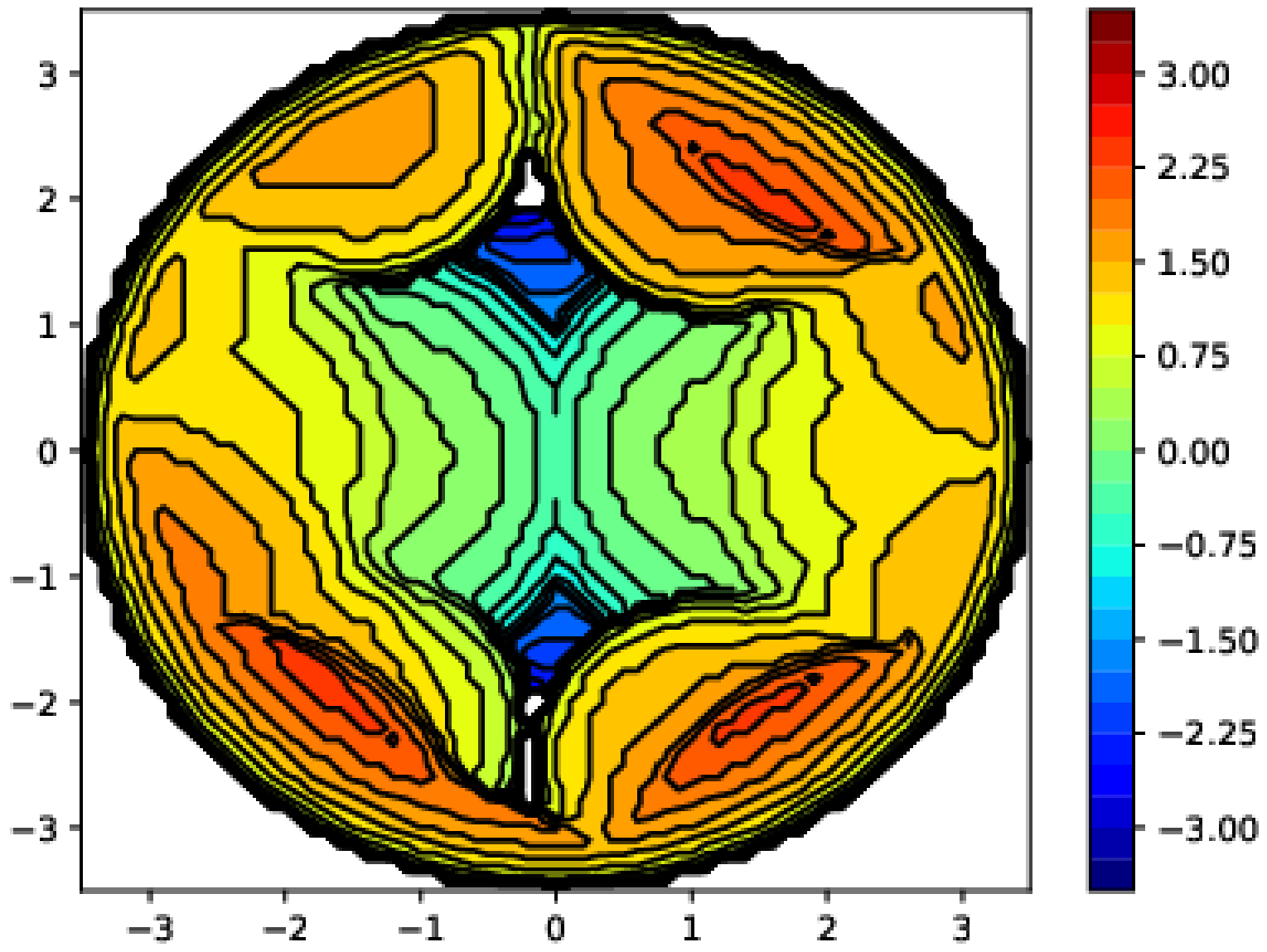

Figure S256: $^{tBu}BDI^{dipep}$ steric map centred on the magnesium centre.

Table S7 Table of parameters for the $^{tBu}BDI^{dipep}$ steric map centred on the magnesium centre

Quadrant	V f	V b	V t	%V f	**%V b**
SW	15.0	29.9	44.9	**33.4**	66.6
NW	17.3	27.6	44.9	**38.6**	61.4
NE	14.0	30.9	44.9	**31.2**	68.8
SE	14.7	30.2	44.9	**32.7**	67.3

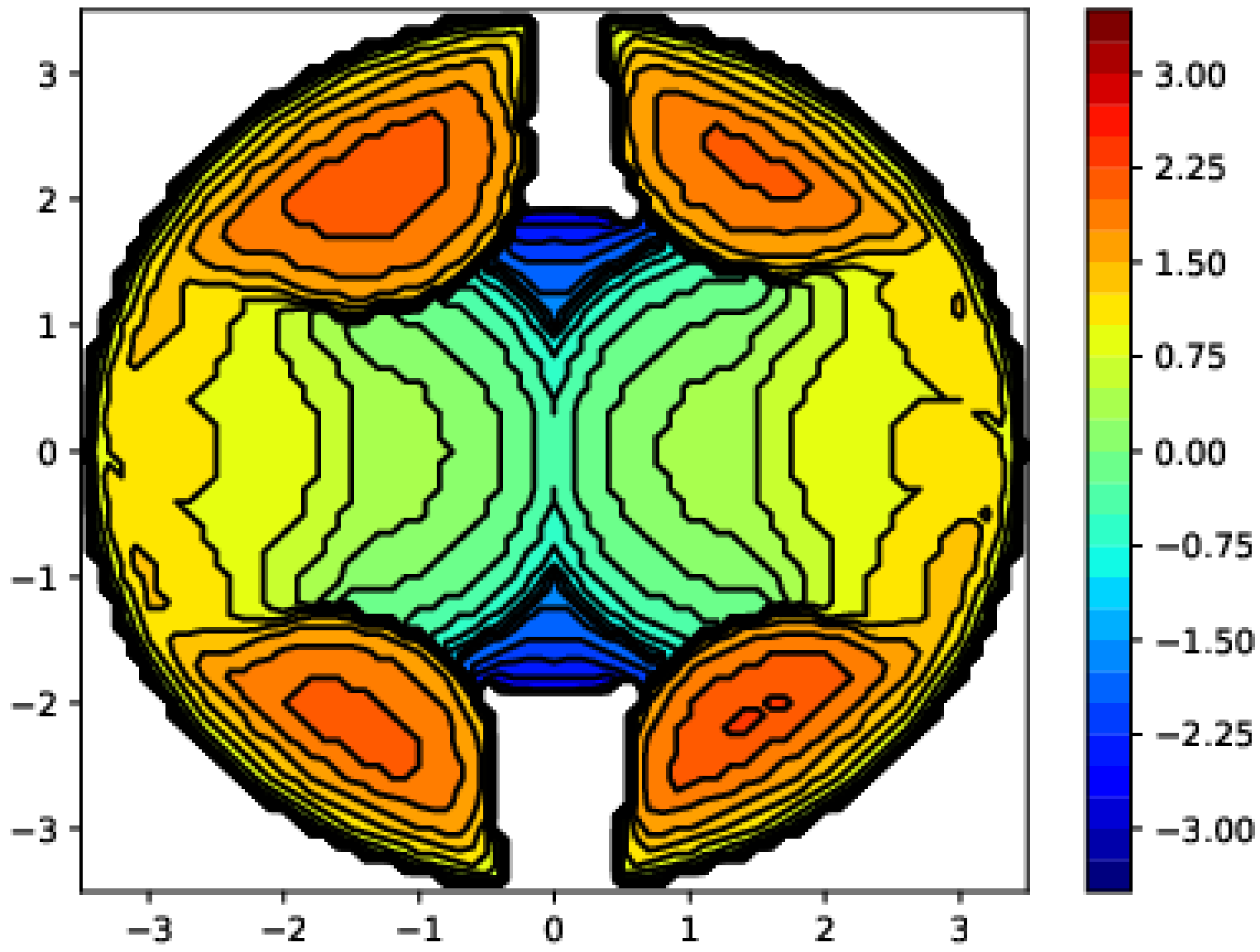

Figure S257: ^{Me}BDI^{dipp} steric map centred on a point positioned 1.36 Å perpendicular from the N-N axis, in the positive z-direction.

Table S8: Table of parameters for the ^{Me}BDI^{dipp} steric map centred on a point positioned 1.36 Å perpendicular from the N-N axis, in the positive z-direction.

Quadrant	V f	V b	V t	%V f	**%V b**
SW	17.9	26.9	44.9	**40.0**	60.0
NW	15.9	29.0	44.9	**35.4**	64.6
NE	18.2	26.7	44.9	**40.6**	59.4
SE	18.0	26.9	44.9	**40.1**	59.9

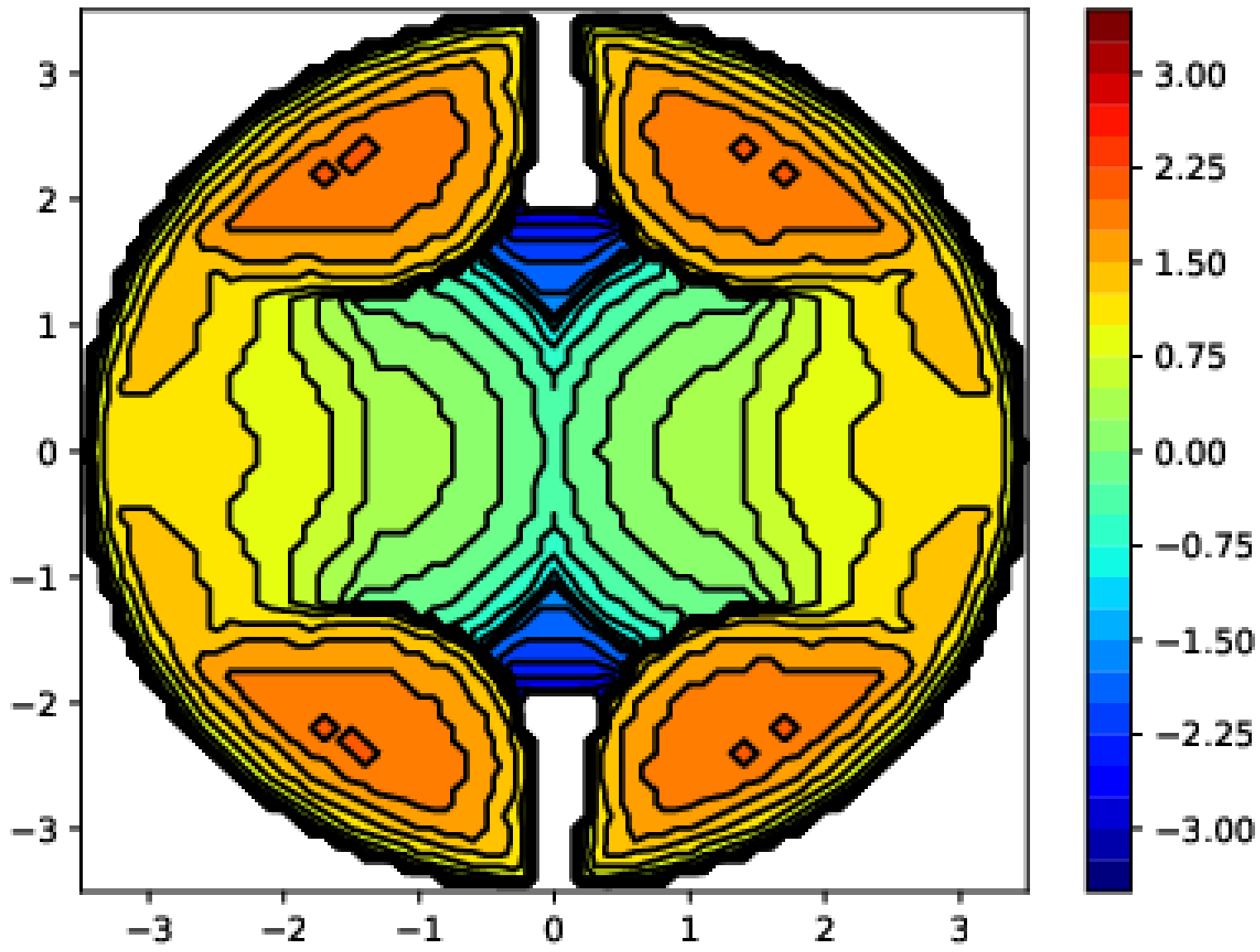

Figure S258: ^{iPr}BDI^{dipp} steric map centred on a point positioned 1.36 Å perpendicular from the N-N axis, in the positive z-direction.

Table S9: Table of parameters for the ^{iPr}BDI^{dipp} steric map centred on a point positioned 1.36 Å perpendicular from the N-N axis, in the positive z-direction.

Quadrant	V f	V b	V t	%V f	**%V b**
SW	16.4	28.4	44.9	**36.7**	63.3
NW	16.4	28.4	44.9	**36.7**	63.3
NE	16.8	28.0	44.9	**37.5**	62.5
SE	16.8	28.0	44.9	**37.5**	62.5

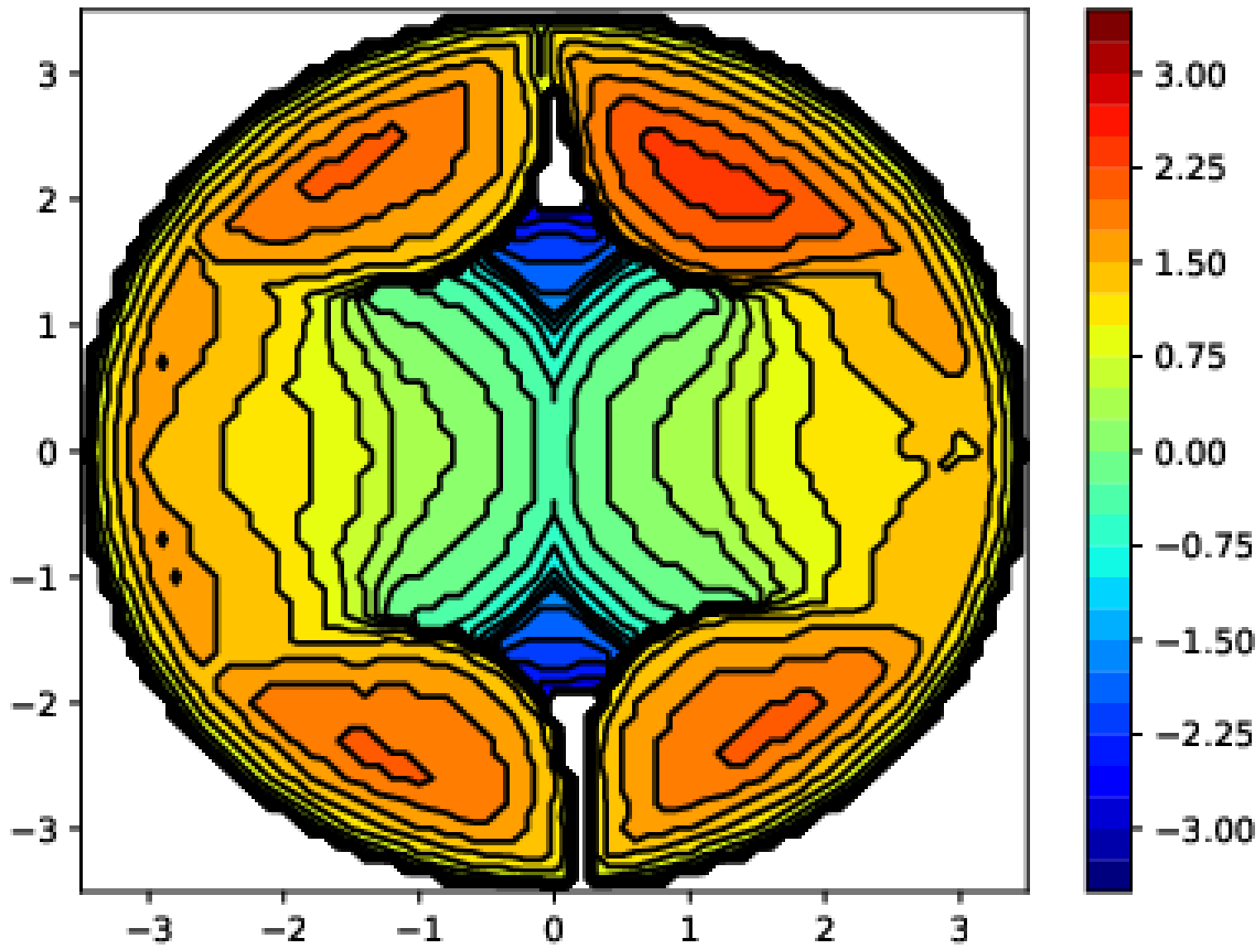

Figure S259: $^{Ad}BDI^{dipp}$ steric map centred on a point positioned 1.36 Å perpendicular from the N-N axis, in the positive z-direction.

Table S10: Table of parameters for the $^{Ad}BDI^{dipp}$ steric map centred on a point positioned 1.36 Å perpendicular from the N-N axis, in the positive z-direction.

Quadrant	V f	V b	V t	%V f	**%V b**
SW	14.7	30.2	44.9	**32.7**	67.3
NW	15.9	29.0	44.9	**35.4**	64.6
NE	14.3	30.5	44.9	**31.9**	68.1
SE	16.6	28.3	44.9	**37.0**	63.0

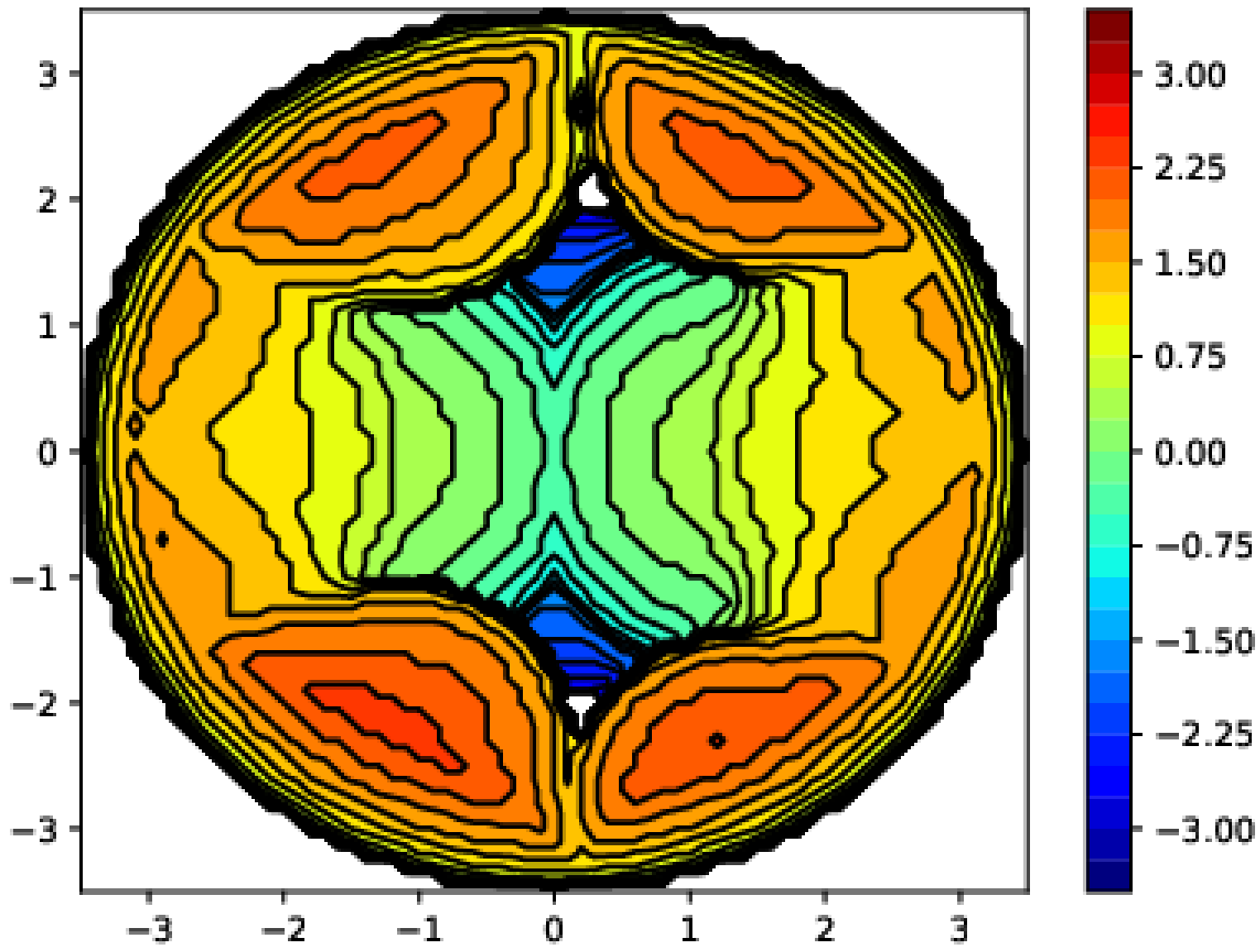

Figure S260: $^{tBu}BDI^{dipp}$ steric map centred on a point positioned 1.36 Å perpendicular from the N-N axis, in the positive z-direction.

Table S11: Table of parameters for the $^{tBu}BDI^{dipp}$ steric map centred on a point positioned 1.36 Å perpendicular from the N-N axis, in the positive z-direction.

Quadrant	V f	V b	V t	%V f	**%V b**
SW	13.0	31.8	44.9	**29.0**	71.0
NW	14.0	30.8	44.9	**31.3**	68.7
NE	16.0	28.9	44.9	**35.6**	64.4
SE	15.2	29.6	44.9	**34.0**	66.0

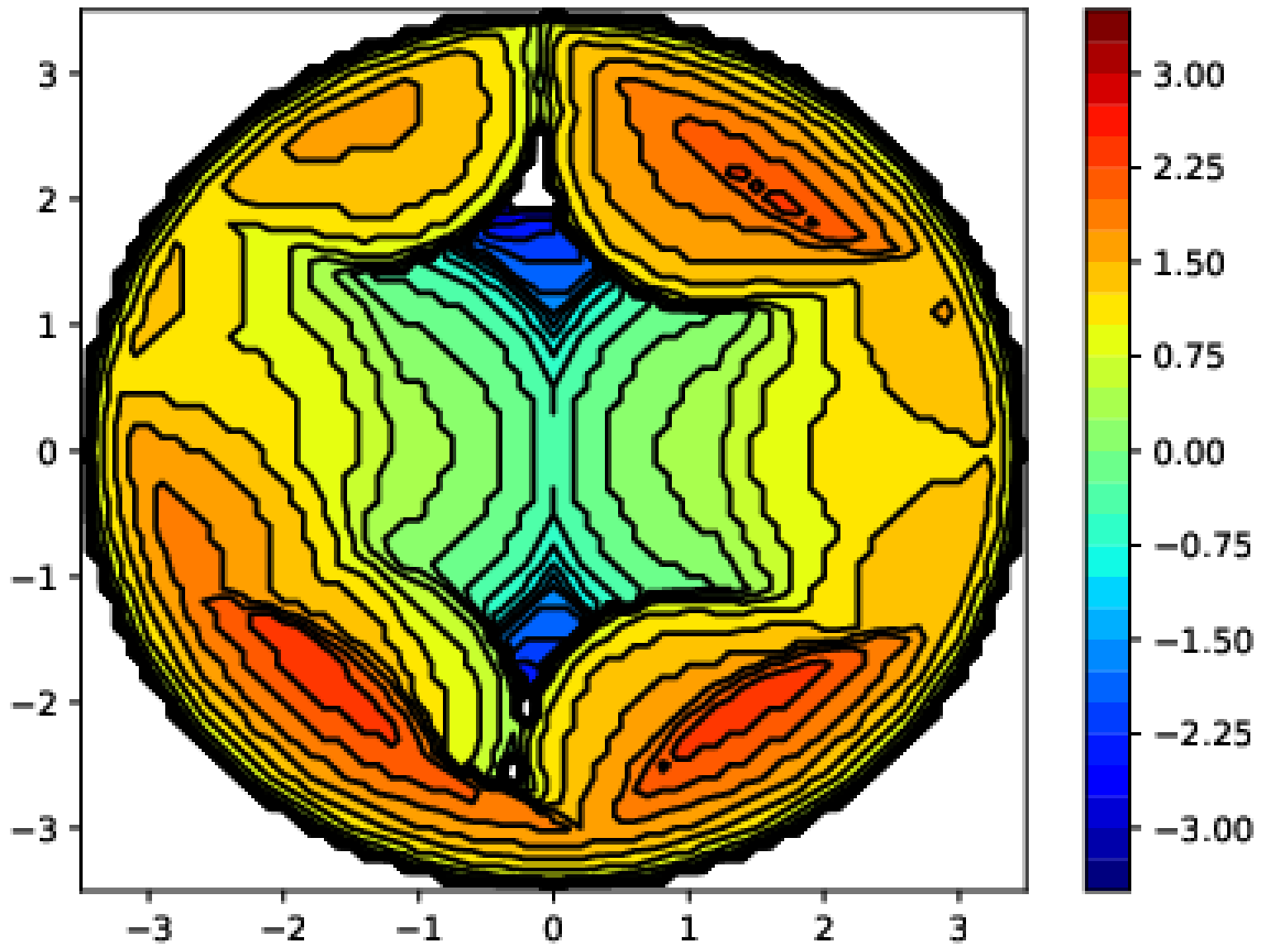

Figure S261: ^{tBu}BDI^{dipep} steric map centred on the magnesium centre.

Table S12 Table of parameters for the ^{tBu}BDI^{dipep} steric map centred on a point positioned 1.36 Å perpendicular from the N-N axis, in the positive z-direction.

Quadrant	V f	V b	V t	%V f	**%V b**
SW	14.9	29.9	44.9	**33.3**	66.7
NW	17.3	27.6	44.9	**38.5**	61.5
NE	14.7	30.2	44.9	**32.8**	67.2
SE	13.9	30.9	44.9	**31.0**	69.0

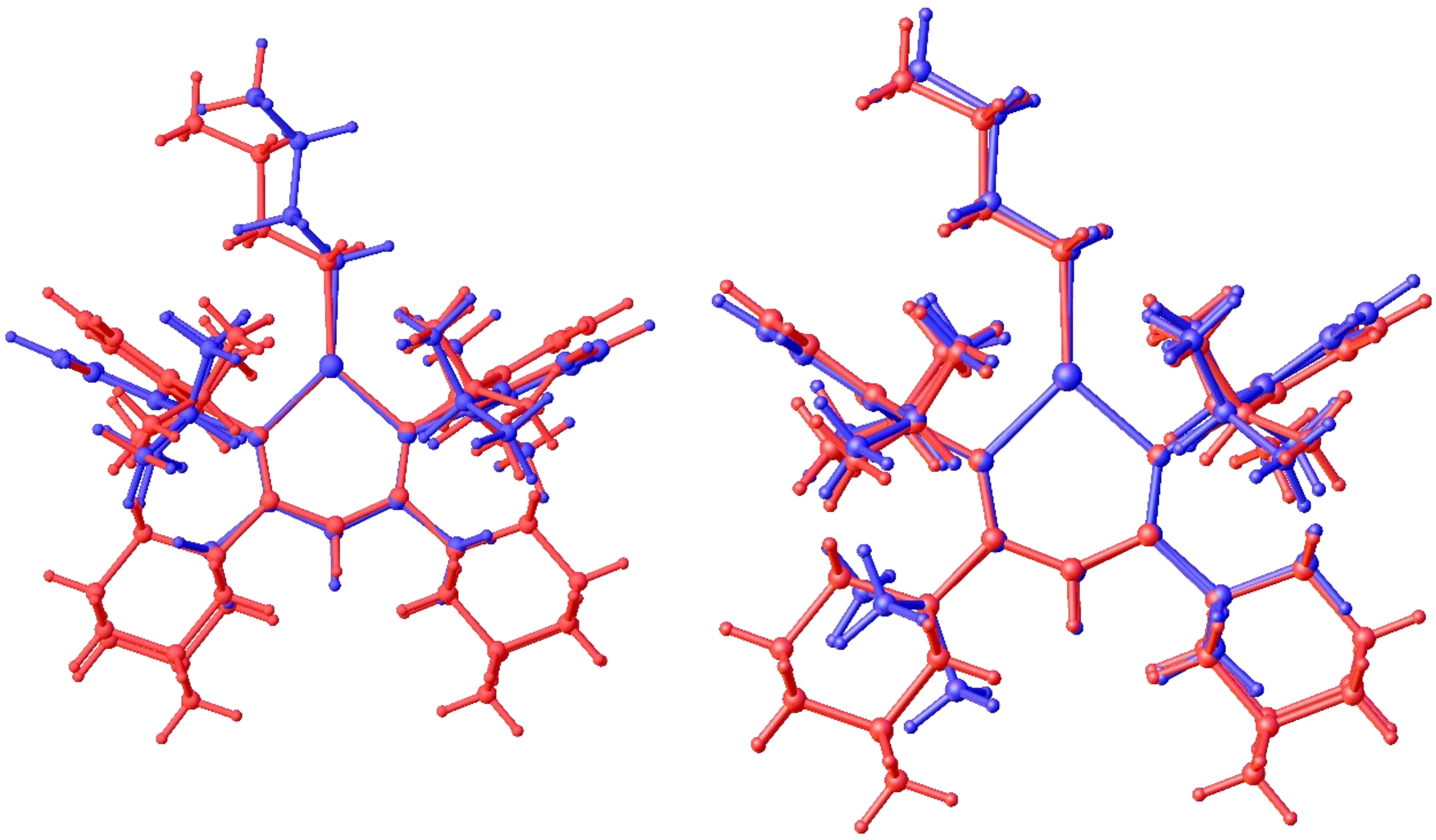

Figure S262: Left: overlay of the crystal structure of $\mathbf{1}^{\mathbf{Ad}}$ (red) and calculated structure of $\mathbf{1}^{\mathbf{Me}}$; Right: overlay of the crystal structures of $\mathbf{1}^{\mathbf{Ad}}$ (red) and $\mathbf{1}^{\mathbf{dipp,tBu}}$ (blue).[10]

Table S13: Comparison of Mg–N distances, N–Mg–N angles, and the fold angle between the benzene rings of the N-aryl groups in monomeric BDI magnesium *n*-butyl complexes [(^{R}BDI^{Ar})Mg^{n}Bu]. For $\mathbf{1}^{\mathbf{dipp,tBu}}$, only values for the Mg1-containing molecule in the asymmetric unit are shown.

Distances (Å) and angles (°)	$\mathbf{1}^{\mathbf{Me}}$	$\mathbf{1}^{\mathbf{dipp,iPr}}$ [ref 8]	$\mathbf{1}^{\mathbf{Ad}}$	$\mathbf{I}^{\mathbf{dipp,tBu}}$ [ref 9]	$\mathbf{1}^{\mathbf{dipep,tBu}}$ [ref 10]
Mg1–N1	2.072	2.0272(13)	2.0257(9)	2.010(5)	2.0323(10)
Mg1–N2	2.073	-	2.0266(9)	2.026(4)	2.0312(10)
N1–Mg1–N2	93.86	93.57(5)	95.29(4)	94.20(17)	95.73(4)
Fold-angle β	131.51	125.89(6)	112.53(4)	110.4(2)	Not measured due to disorder
(C-C)–(C-C) angle α	102.852	101.572	90.244	92.248	90.626
C^{ipso}-N1-C	120.02	120.72(12)	126.31(8)	126.4(4)	126.13(9)
C^{ipso}-N2-C	120.05	120.78(12)	127.81(8)	127.1(4)	126.09(10)
centroid-C^{ipso}-N1	176.25	175.49(12)	176.93(9)	172.1(4)	174.95(9)
centroid-C^{ipso}-N2	176.32	174.86(11)	171.66(8)	174.4(4)	Not measured due to disorder

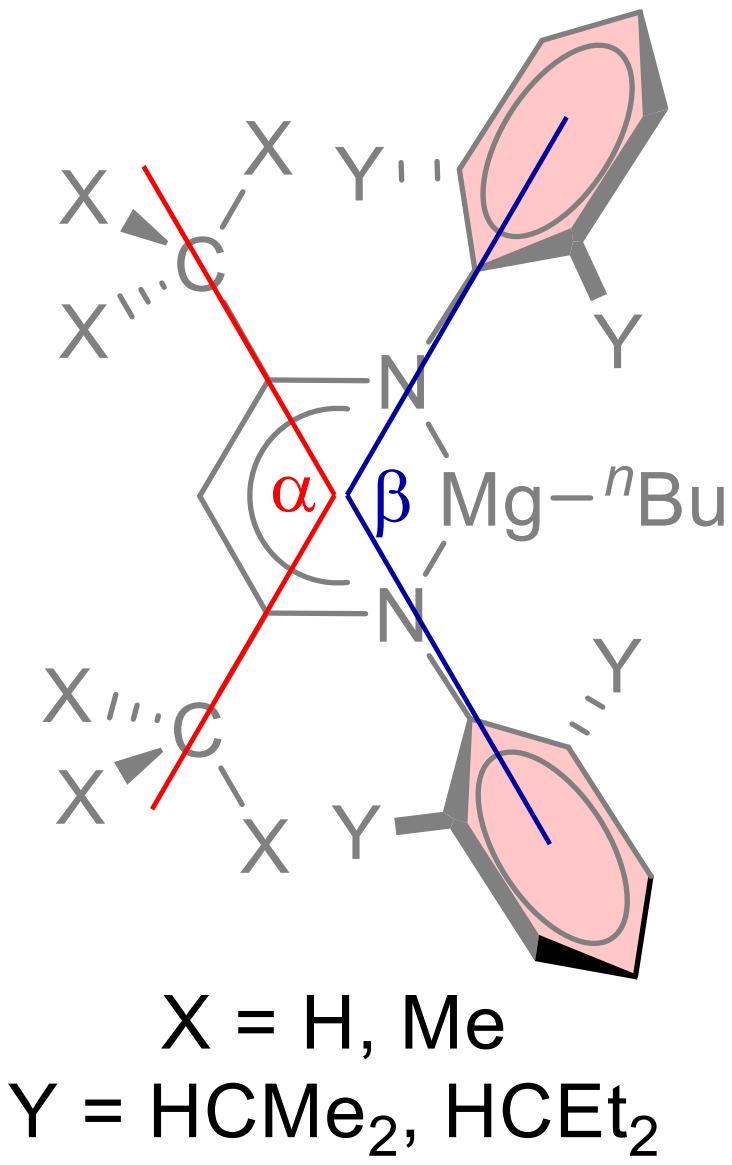

Figure S263: Diagramatic representation of angles α and β used to describe geometry of complexes **1**$^{\mathbf{Me}}$, **1**$^{\mathbf{Ad}}$, and **1**$^{\mathbf{Ar,R}}$.

Bulky backbone groups 'push' the aryl groups forward, resulting in a decreased fold angle (β) and an increased N-Mg-N angle. Additionally, the angle subtended between the linear extension of the backbone C-R bonds (α) is decreases for larger R groups, i.e. as well as the R groups "pushing" the aryl groups forwards, the aryl groups "push" the R groups back. This angle is smaller for **1**$^{\mathbf{Ad}}$ than for **1**$^{\mathbf{dipp,tBu}}$ and **1**$^{\mathbf{dipep,tBu}}$, which may be due to intramolecular attractive dispersion forces between the adamantyl groups. This may explain the smaller N-Mg-N angle, shorter Mg-N distances, and higher %V_{bur} for **1**$^{\mathbf{dipp,tBu}}$ compared to **1**$^{\mathbf{Ad}}$, which has bulkier R, and compared to **1**$^{\mathbf{dipep,tBu}}$, which has bulkier Ar.

4 Kinetic isotope effect experiments

The kinetic isotope effect value for the reaction of **1**$^{\mathbf{Ad}}$ with benzene(-d_6) was determined by periodic ^{1}H NMR spectroscopic analysis of parallel reactions of **1**$^{\mathbf{Ad}}$ with C_6D_6 and with C_6H_6 at 102°C, each run in-duplicate.

Four J. Young's NMR tubes were each charged with 12.5 mg **1**$^{\mathbf{Ad}}$ (0.017 mmol). 600 µl of C_6D_6 was added to two of the tubes, whilst 600 µl C_6H_6 was added to the other two and the Teflon valve was closed (0.028 M concentration). The sealed reaction tubes were placed in an aluminium heating block at 102°C and analysed periodically for a total reaction time of 92 hours (3.8 days). The reaction progress was quantified by the relative integrals of **1**$^{\mathbf{Ad}}$ and the only product, **3**$^{\mathbf{Ad}}$**(-*d*$_6$)**. Plotting the combined ln[**1**$^{\mathbf{Ad}}$] of the duplicate reactions against time provided a linear plot, from which the observed *pseudo*-first order rate constant, k_{obs}, was obtained as the gradient of the least-squares fit.

$k_{obs}(C_6D_6)$ = -0.0153±0.00090

$k_{obs}(C_6H_6)$ = -0.0192±0.00017

KIE = 1.250±0.075

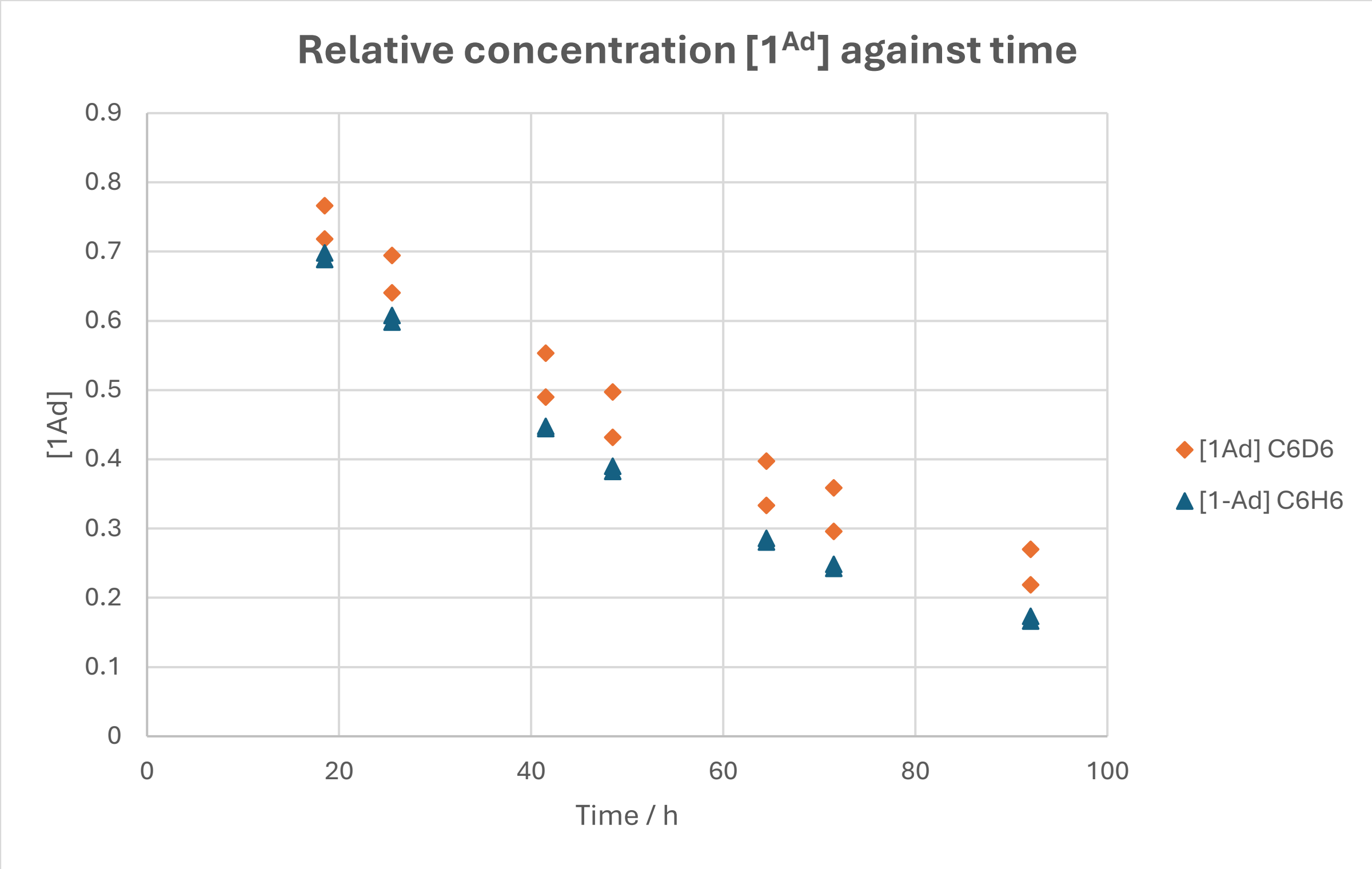

Figure S264: Plot of concentration of **1**[Ad] against time for the reaction of **1**[Ad] with C_6D_6 (orange diamonds) or C_6H_6 (blue triangles).

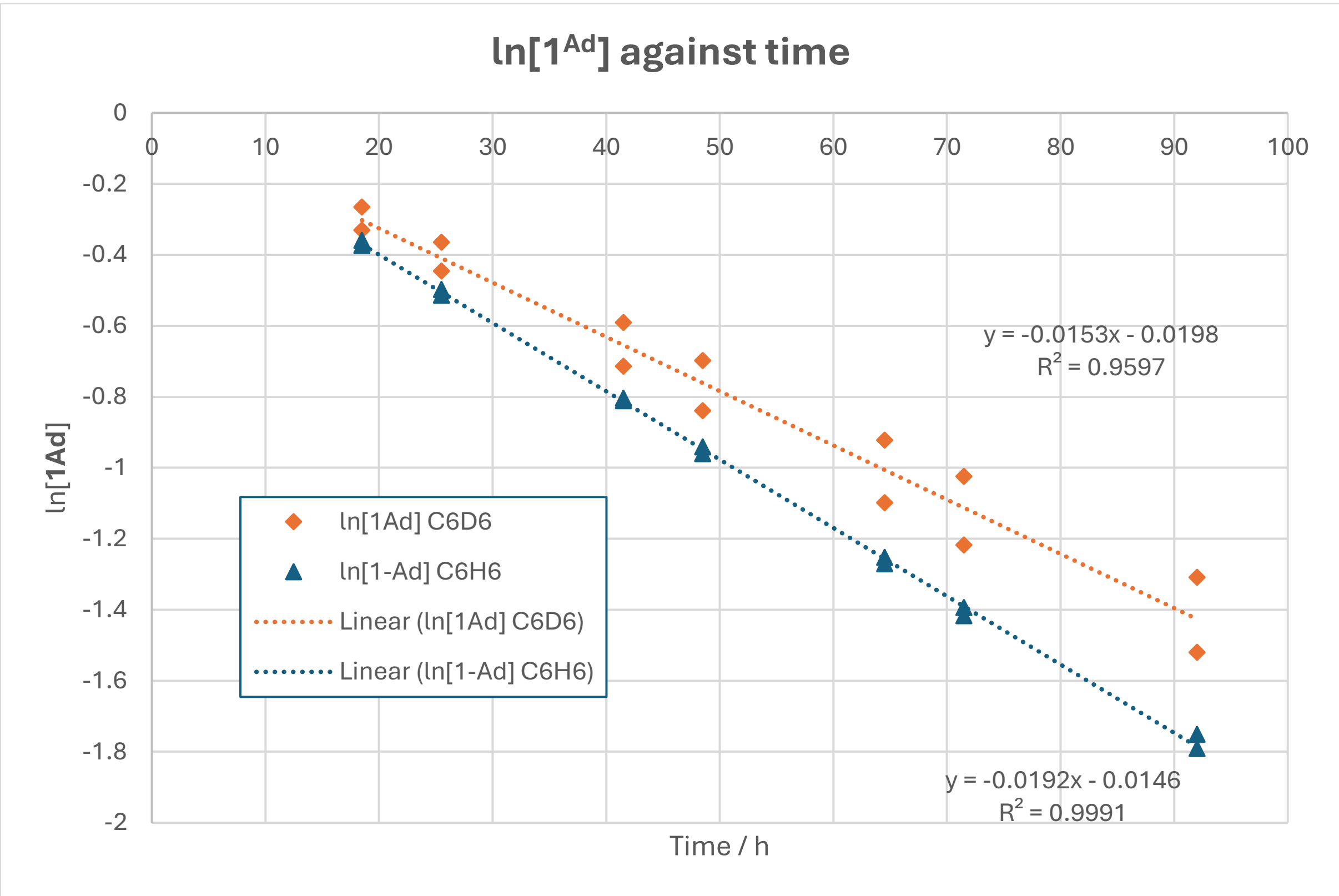

Figure S265: Plot of ln[**1**[Ad]] against time for the reaction of **1**[Ad] with C_6D_6 (orange diamonds) or C_6H_6 (blue triangles).

5 X-ray diffraction

Single Crystal X-ray diffraction data for compounds ^{Ad}BDIH, **1^{Ad}**, **3^{Ad}**, **5-*m*/5-*p***, **6/6'**, **7**, **9**, **10^{Me}·thf**, **12**, and **13** were collected on an Agilent SuperNova EosS2 diffractometer using Cu-Kα (1.54184 Å) radiation, whilst compounds **4**, **5'**, **6'**, **8**, **10^{Ad}·anisole** and **11** were collected on an XtaLAB Synergy, Dualflex, HyPix-Arc 100 diffractometer using Cu-Kα (1.54184 Å) radiation. In each case, the crystals were maintained at 150 K during data collection. Using Olex2,[12] the structures were solved with ShelXT[13] and refined with the ShelXL[14] refinement package using Least−Squares minimisation.

Crystallographic data for all compounds have been deposited with the Cambridge Crystallographic Data Centre as supplementary publications CCDC 2557915-2557930. Copies of these data can be obtained free of charge on application to CCDC, 12 Union Road, Cambridge CB2 1EZ, UK [fax(+44) 1223 336033], e-mail: deposit@ccdc.cam.ac.uk, www.ccdc.cam.ac.uk/structures.

Table S14: Crystal data and structure refinement

Identification code	^{Ad}BDIH	**1^{Ad}**	**3^{Ad}**	**4**
Empirical formula	$C_{47}H_{66}N_2$	$C_{58}H_{88}MgN_2$	$C_{53}H_{70}MgN_2$	$C_{38}H_{54}N_2Mg$
Formula weight	659.01	837.61	759.42	563.14
Temperature/K	150.00(10)	150.00(10)	150.00(10)	150.00(10)
Crystal system	monoclinic	monoclinic	orthorhombic	monoclinic
Space group	$P2_1/n$	$P2_1/n$	Pbca	$P2_1/n$
a/Å	18.39810(10)	11.4469(1)	13.47250(10)	11.37260(10)
b/Å	11.66470(10)	18.5075(1)	24.11050(10)	25.9880(2)
c/Å	18.91450(10)	24.2155(1)	27.80910(10)	12.03910(10)
α/°	90	90	90	90
β/°	105.2520(10)	97.756(1)	90	107.8690(10)
γ/°	90	90	90	90
Volume/Å^3	3916.23(5)	5083.21(6)	9033.19(8)	3386.52(5)
Z	4	4	8	4
ρ_{calc}g/cm^3	1.118	1.094	1.117	1.105
μ/mm^{-1}	0.471	0.569	0.599	0.639
F(000)	1448.0	1848.0	3312.0	1232.0
Crystal size/mm^3	0.18 × 0.15 × 0.1	0.262 × 0.202 × 0.172	0.22 × 0.17 × 0.11	0.16 × 0.16 × 0.04
Radiation	Cu Kα (λ = 1.54184)	CuKα (λ = 1.54184)	Cu Kα (λ = 1.54184)	Cu Kα (λ = 1.54184)
2Θ range for data collection/°	7.808 to 146.542	6.032 to 146.142	7.334 to 146.682	6.802 to 160.47
Index ranges	-22 ≤ h ≤ 22, -14 ≤ k ≤ 14, -23 ≤ l ≤ 19	-14 ≤ h ≤ 13, -22 ≤ k ≤ 22, -27 ≤ l ≤ 30	-16 ≤ h ≤ 16, -29 ≤ k ≤ 28, -34 ≤ l ≤ 32	-14 ≤ h ≤ 11, -32 ≤ k ≤ 33, -15 ≤ l ≤ 15
Reflections collected	53216	66341	116612	48790
Independent reflections	7861 [R_{int} = 0.0268, R_{sigma} = 0.0166]	10148 [R_{int} = 0.0256, R_{sigma} = 0.0161]	9060 [R_{int} = 0.0329, R_{sigma} = 0.0118]	7323 [R_{int} = 0.0270, R_{sigma} = 0.0186]
Data/restraints/parameters	7861/42/568	10148/0/496	9060/29/620	7323/55/470
Goodness-of-fit on F^2	1.023	1.036	1.041	1.040
Final R indexes [I>=2σ (I)]	R_1 = 0.0435, wR_2 = 0.1106	R_1 = 0.0401, wR_2 = 0.1100	R_1 = 0.0409, wR_2 = 0.1107	R_1 = 0.0483, wR_2 = 0.1282
Final R indexes [all data]	R_1 = 0.0474, wR_2 = 0.1137	R_1 = 0.0425, wR_2 = 0.1124	R_1 = 0.0458, wR_2 = 0.1153	R_1 = 0.0529, wR_2 = 0.1316
Largest diff. peak/hole / e Å^{-3}	0.36/-0.31	0.25/-0.27	0.21/-0.23	0.36/-0.29

Table S14: Crystal data and structure refinement

Identification code	**5-*m*/5-*p***	**5'**	**6/6'**	**7**
Empirical formula	$C_{115}H_{152}Mg_2N_4$	$C_{59.5}H_{81}MgN_2$	$C_{55}H_{74}MgN_2$	$C_{55}H_{74}MgN_2$
Formula weight	1639.02	848.57	787.47	787.47
Temperature/K	150.15	150.00(10)	150.00(10)	150.00(10)
Crystal system	monoclinic	triclinic	orthorhombic	monoclinic
Space group	C2/c	P-1	Pbca	$P2_1/n$
a/Å	37.5707(2)	13.0928(3)	13.3268(3)	10.8827(2)
b/Å	24.92530(10)	14.0609(7)	24.1532(5)	17.8000(4)
c/Å	21.20640(10)	14.2101(4)	29.2645(8)	24.4969(4)
α/°	90	80.439(3)	90	90
β/°	100.6010(10)	78.863(2)	90	99.455(2)
γ/°	90	82.422(3)	90	90
Volume/$Å^3$	19520.01(17)	2517.61(16)	9419.8(4)	4680.88(16)
Z	8	2	8	4
ρ_{calc}g/cm^3	1.115	1.119	1.111	1.117
μ/mm^{-1}	0.588	0.585	0.590	0.594
F(000)	7152.0	928.0	3440.0	1720.0
Crystal size/mm^3	0.32 × 0.2 × 0.11	0.15 × 0.09 × 0.07	0.1 × 0.1 × 0.08	0.13 × 0.12 × 0.03
Radiation	Cu Kα (λ = 1.54184)	Cu Kα (λ = 1.54184)	Cu Kα (λ = 1.54184)	Cu Kα (λ = 1.54184)
2Θ range for data collection/°	7.092 to 146.524	6.406 to 140.148	7.32 to 147.264	8.446 to 147.406
Index ranges	-46 ≤ h ≤ 46, -30 ≤ k ≤ 28, -26 ≤ l ≤ 26	-15 ≤ h ≤ 15, -17 ≤ k ≤ 17, -17 ≤ l ≤ 15	-13 ≤ h ≤ 16, -27 ≤ k ≤ 29, -35 ≤ l ≤ 36	-13 ≤ h ≤ 13, -22 ≤ k ≤ 21, -30 ≤ l ≤ 29
Reflections collected	127918	34865	80703	63257
Independent reflections	19509 [R_{int} = 0.0248, R_{sigma} = 0.0136]	9520 [R_{int} = 0.0356, R_{sigma} = 0.0331]	9436 [R_{int} = 0.0592, R_{sigma} = 0.0300]	9369 [R_{int} = 0.0861, R_{sigma} = 0.0504]
Data/restraints/parameters	19509/1367/1398	9520/12/611	9436/237/704	9369/0/537
Goodness-of-fit on F^2	1.048	1.029	1.035	1.019
Final R indexes [I>=2σ (I)]	R_1 = 0.0637, wR_2 = 0.1805	R_1 = 0.0477, wR_2 = 0.1268	R_1 = 0.0690, wR_2 = 0.1745	R_1 = 0.0579, wR_2 = 0.1541
Final R indexes [all data]	R_1 = 0.0698, wR_2 = 0.1871	R_1 = 0.0641, wR_2 = 0.1377	R_1 = 0.1023, wR_2 = 0.1984	R_1 = 0.0677, wR_2 = 0.1660
Largest diff. peak/hole / e $Å^{-3}$	0.46/-0.52	0.36/-0.23	0.32/-0.22	0.65/-0.40

Table S15: Crystal data and structure refinement

Identification code	**7'**	**8**	**9**	**10^{Me}·thf**
Empirical formula	$C_{55}H_{74}N_2Mg$	$C_{61}H_{88}N_2Mg$	$C_{61}H_{86}N_2Mg$	$C_{40}H_{56}MgN_2O_2$
Formula weight	787.47	873.64	871.62	621.17
Temperature/K	150.00(10)	150.00(10)	150.00(10)	150.00(10)
Crystal system	monoclinic	triclinic	orthorhombic	monoclinic
Space group	$P2_1/c$	P-1	$P2_12_12_1$	$P2_1/c$
a/Å	9.94940(10)	13.5887(2)	10.18700(10)	17.2527(2)
b/Å	13.64860(10)	14.7112(2)	21.54160(10)	11.6656(2)
c/Å	34.8915(2)	15.3361(2)	24.25800(10)	18.3717(3)
α/°	90	112.6660(10)	90	90
β/°	96.9110(10)	94.4360(10)	90	92.3100(10)
γ/°	90	106.0660(10)	90	90
Volume/$Å^3$	4703.68(7)	2659.34(7)	5323.28(6)	3694.54(10)
Z	4	2	4	4
$\rho_{calc}g/cm^3$	1.112	1.091	1.088	1.117
μ/mm^{-1}	0.591	0.564	0.563	0.671
F(000)	1720.0	960.0	1912.0	1352.0
Crystal size/mm^3	0.26 × 0.18 × 0.14	0.36 × 0.07 × 0.05	0.24 × 0.18 × 0.12	0.26 × 0.12 × 0.05
Radiation	Cu Kα (λ = 1.54184)	Cu Kα (λ = 1.54184)	Cu Kα (λ = 1.54184)	Cu Kα (λ = 1.54184)
2Θ range for data collection/°	5.102 to 160.742	6.384 to 148.996	7.288 to 146.456	8.982 to 146.346
Index ranges	-12 ≤ h ≤ 12, -17 ≤ k ≤ 16, -42 ≤ l ≤ 44	-16 ≤ h ≤ 16, -18 ≤ k ≤ 17, -17 ≤ l ≤ 19	-12 ≤ h ≤ 12, -26 ≤ k ≤ 25, -30 ≤ l ≤ 18	-21 ≤ h ≤ 19, -14 ≤ k ≤ 14, -22 ≤ l ≤ 22
Reflections collected	79298	38610	72356	50716
Independent reflections	10198 [R_{int} = 0.0318, R_{sigma} = 0.0189]	10792 [R_{int} = 0.0308, R_{sigma} = 0.0302]	10657 [R_{int} = 0.0299, R_{sigma} = 0.0178]	7394 [R_{int} = 0.0402, R_{sigma} = 0.0233]
Data/restraints/parameters	10198/68/606	10792/0/588	10657/3/622	7394/0/490
Goodness-of-fit on F^2	1.044	1.060	1.041	1.100
Final R indexes [I>=2σ (I)]	R_1 = 0.0464, wR_2 = 0.1205	R_1 = 0.0474, wR_2 = 0.1347	R_1 = 0.0307, wR_2 = 0.0811	R_1 = 0.0542, wR_2 = 0.1433
Final R indexes [all data]	R_1 = 0.0513, wR_2 = 0.1243	R_1 = 0.0555, wR_2 = 0.1407	R_1 = 0.0315, wR_2 = 0.0817	R_1 = 0.0588, wR_2 = 0.1468
Largest diff. peak/hole / e $Å^{-3}$	0.45/-0.39	0.28/-0.30	0.30/-0.20	0.30/-0.32
Flack parameter			0.050(9)	

Table S16: Crystal data and structure refinement.

Identification code	**10^{Ad}·anisole**	**11**	**12**	**13**
Empirical formula	$C_{61}H_{80}MgN_2O_2$	$C_{111}H_{159}Mg_2N_4O_2$	$C_{55}H_{83}BMgN_2O_4$	$C_{65}H_{83}BrMgN_2$
Formula weight	897.58	1630.03	871.35	996.55
Temperature/K	149.99(14)	149.99(14)	150.00(10)	150.00(10)
Crystal system	triclinic	triclinic	monoclinic	orthorhombic
Space group	P-1	P-1	$P2_1/c$	Pnma
a/Å	10.8852(11)	10.07720(10)	17.44550(10)	21.05140(10)
b/Å	12.7840(8)	22.3784(2)	13.91910(10)	17.07070(10)
c/Å	20.4936(12)	24.4296(3)	21.29260(10)	15.72880(10)
α/°	99.937(5)	114.4420(10)	90	90
β/°	91.209(6)	98.8080(10)	99.2870(10)	90
γ/°	114.216(8)	97.8680(10)	90	90
Volume/$Å^3$	2548.4(4)	4831.94(10)	5102.62(5)	5652.34(6)
Z	2	2	4	4
$\rho_{calc}g/cm^3$	1.170	1.120	1.134	1.171
μ/mm^{-1}	0.636	0.604	0.644	1.391
F(000)	976.0	1786.0	1904.0	2136.0
Crystal size/mm^3	0.2 × 0.05 × 0.04	0.22 × 0.12 × 0.03	0.16 × 0.14 × 0.1	0.28 × 0.22 × 0.15
Radiation	Cu Kα (λ = 1.54184)	Cu Kα (λ = 1.54184)	Cu Kα (λ = 1.54184)	Cu Kα (λ = 1.54184)
2Θ range for data collection/°	4.4 to 165.6	4.448 to 161.124	7.618 to 146.386	7.016 to 146.402
Index ranges	-13 ≤ h ≤ 13, -16 ≤ k ≤ 16, -25 ≤ l ≤ 26	-12 ≤ h ≤ 11, -23 ≤ k ≤ 28, -31 ≤ l ≤ 30	-21 ≤ h ≤ 21, -11 ≤ k ≤ 17, -26 ≤ l ≤ 26	-20 ≤ h ≤ 26, -21 ≤ k ≤ 21, -19 ≤ l ≤ 19
Reflections collected	22383	79267	69937	74325
Independent reflections	22383 [R_{int} = ?, R_{sigma} = 0.0500]	20566 [R_{int} = 0.0591, R_{sigma} = 0.0502]	10212 [R_{int} = 0.0268, R_{sigma} = 0.0159]	5869 [R_{int} = 0.0279, R_{sigma} = 0.0120]
Data/restraints/parameters	22383/0/606	20566/174/1043	10212/0/856	5869/697/438
Goodness-of-fit on F^2	1.068	1.035	1.028	1.047
Final R indexes [I>=2σ (I)]	R_1 = 0.0946, wR_2 = 0.2737	R_1 = 0.0580, wR_2 = 0.1477	R_1 = 0.0393, wR_2 = 0.1082	R_1 = 0.0336, wR_2 = 0.0978
Final R indexes [all data]	R_1 = 0.1418, wR_2 = 0.3313	R_1 = 0.0778, wR_2 = 0.1591	R_1 = 0.0434, wR_2 = 0.1123	R_1 = 0.0349, wR_2 = 0.0991
Largest diff. peak/hole / e $Å^{-3}$	0.48/-0.66	0.29/-0.44	0.56/-0.32	0.30/-0.40

5.1 Crystal structure determination of ^{Ad}BDIH

CCDC Deposition Number 2557930.

^{Ad}BDIH crystallised as colourless blocks from a saturated ethanol/methylene chloride solution at room temperature.

Crystal Data for $C_{47}H_{66}N_2$ (M =659.01 g/mol): monoclinic, space group P2$_1$/n (no. 14), a = 18.39810(10) Å, b = 11.66470(10) Å, c = 18.91450(10) Å, β = 105.2520(10)°, V = 3916.23(5) Å^3, Z = 4, T = 150.00(10) K, μ(Cu Kα) = 0.471 mm^{-1}, $Dcalc$ = 1.118 g/cm^3, 53216 reflections measured (7.808° ≤ 2Θ ≤ 146.542°), 7861 unique (R_{int} = 0.0268, R_{sigma} = 0.0166) which were used in all calculations. The final R_1 was 0.0435 (I > 2σ(I)) and wR_2 was 0.1137 (all data).

The crystal structure of ^{Ad}BDIH contains one bis-imine molecule in the asymmetric unit. The C14-containing adamantyl moiety was disordered due to rotation about the C13-C14 bond, such that atoms C15-C23 were treated with a split model in two components of relative occupancy 0.646(2):0.354(2). Similarly, the C42-containing *iso*-propyl moiety was disordered such that the associated methyl groups were treated with a split model in two components of relative occupancy 0.809(15):0.191(15). Distance and ADP restraints were employed for the disordered moieties to assist with convergence. The hydrogen atoms H12A and H12B bound to the methylene carbon were located in the difference map and freely refined.

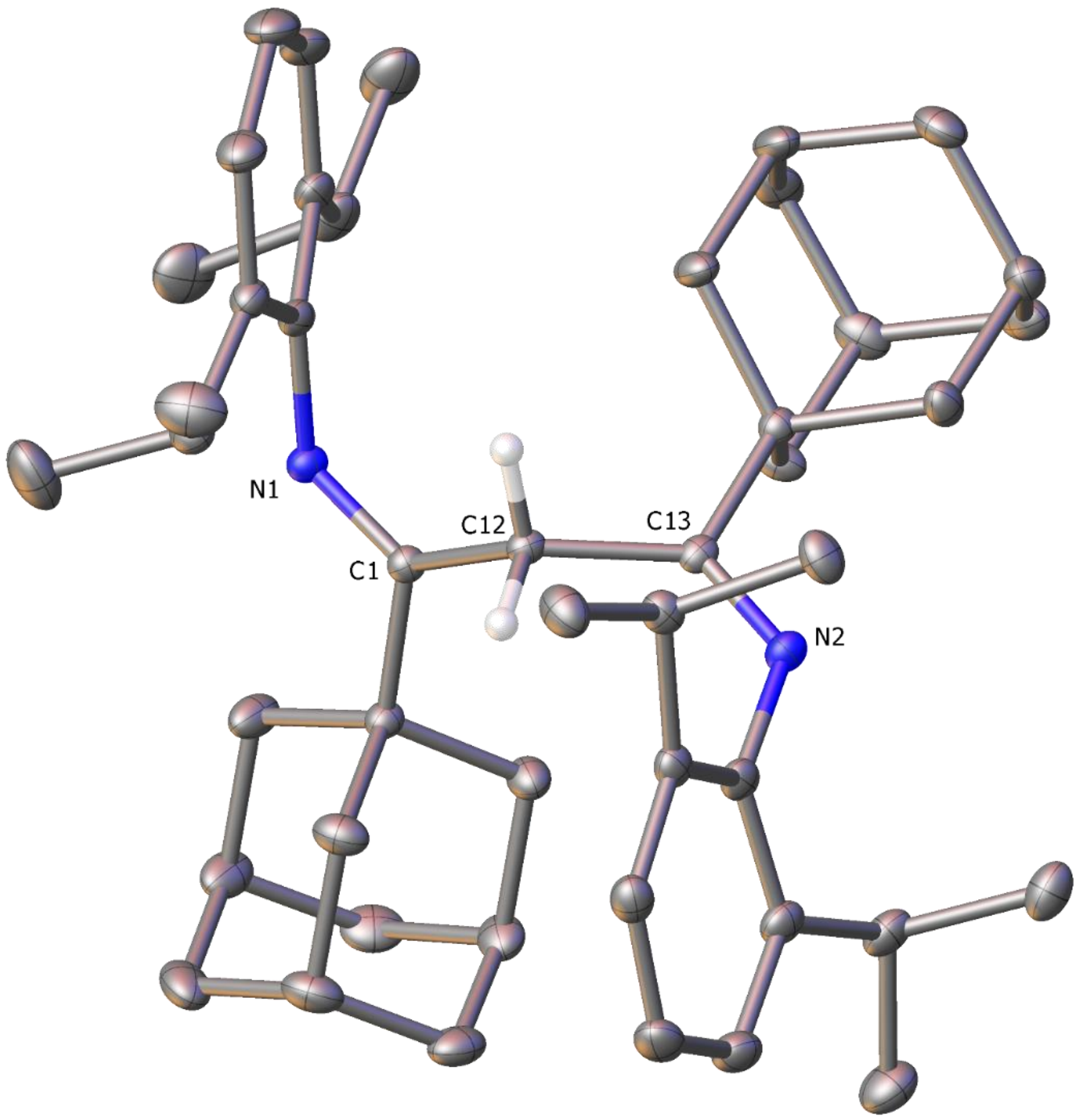

Figure S266: Displacement ellipsoid plot (30%) of ^{Ad}BDIH. Except for those bound to C12, hydrogen atoms are omitted for clarity. Only the major components of the disordered adamantyl and *iso*-propyl moieties are shown.

5.2 Crystal structure determination of [(^{Ad}BDI)Mg^{n}Bu] (1^{Ad})

CCDC Deposition Number 2557915.

1^{Ad} crystallised as colourless blocks from a saturated methylcyclohexane solution at -35°C.

Crystal Data for $C_{58}H_{88}MgN_2$ (M =837.61 g/mol): monoclinic, space group P2$_1$/n (no. 14), a = 11.44690(10) Å, b = 18.50750(10) Å, c = 24.21550(10) Å, β = 97.7560(10)°, V = 5083.21(6) Å^3, Z = 4, T = 150.00(10) K, μ(CuKα) = 0.569 mm^{-1}, $Dcalc$ = 1.094 g/cm^3, 66341 reflections measured (6.032° ≤ 2Θ ≤ 146.142°), 10148 unique (R_{int} = 0.0256, R_{sigma} = 0.0161) which were used in all calculations. The final R_1 was 0.0401 (I > 2σ(I)) and wR_2 was 0.1124 (all data).

The asymmetric unit in **1^{Ad}** contains one molecule of the magnesium complex and one disordered molecule of methylcyclohexane. It was possible to model the latter as two components in a 60:40 ratio. However, satisfactory disorder modelling was precluded by the fact that two of the carbons in the minor component were further disordered over 2 sites. Efforts to model this were not stable even after the inclusion of restraints. Ultimately, therefore, solvent in this structure was addressed using the solvent mask algorithm available in Olex-2 and allowance made for same in the formula as presented.

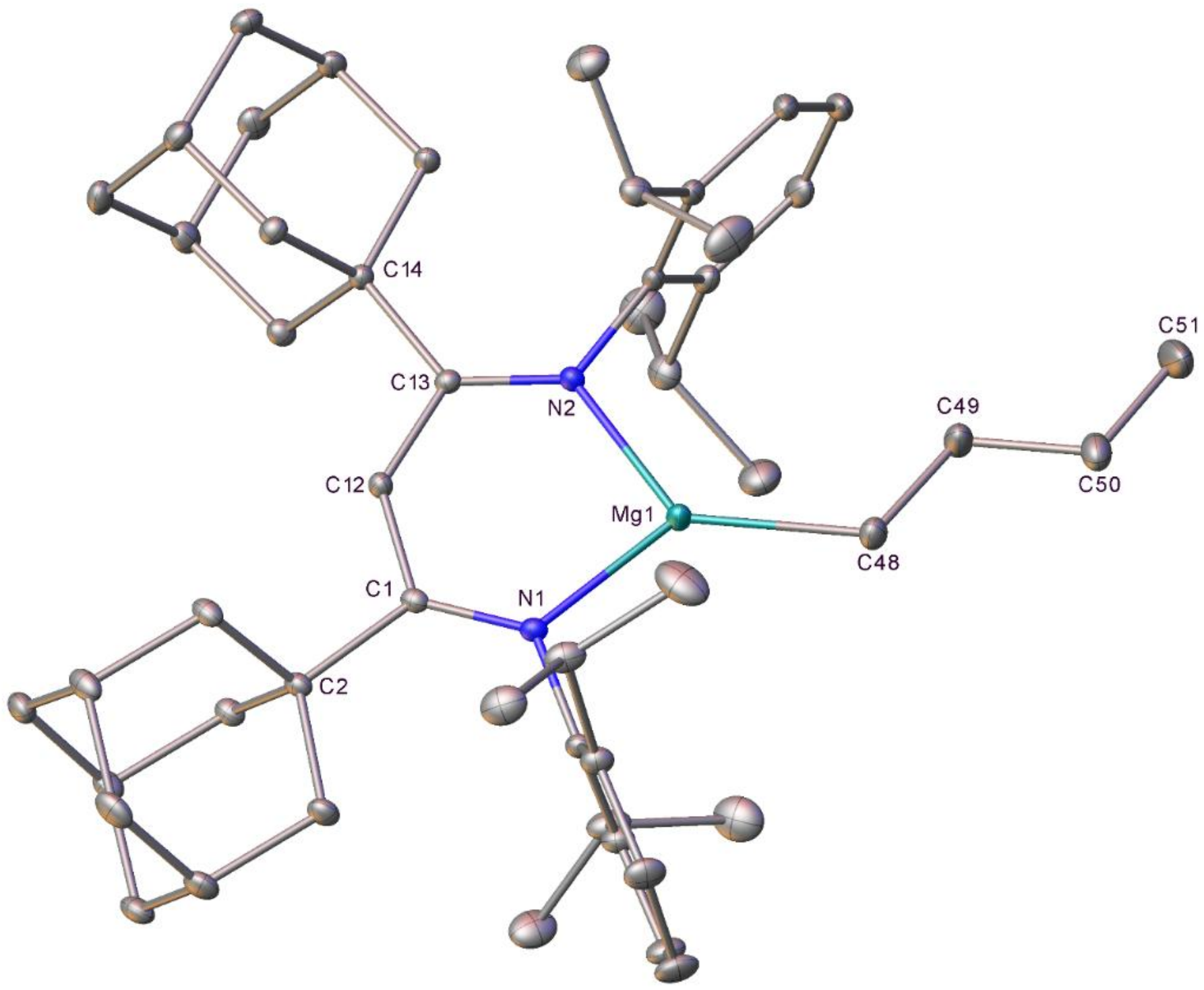

Figure S267: Displacement ellipsoid plot (30% level) of **1^{Ad}** with hydrogen atoms omitted for clarity.

5.3 Crystal structure determination of [(^{Ad}BDI)MgPh] (3^{Ad})

CCDC Deposition Number 2557916.

3^{Ad} crystallised as colourless blocks from slow-evaporation of a saturated *n*-hexane/toluene solution at room temperature.

Crystal Data for $C_{53}H_{70}MgN_2$ (M =759.42 g/mol): orthorhombic, space group Pbca (no. 61), a = 13.47250(10) Å, b = 24.11050(10) Å, c = 27.80910(10) Å, V = 9033.19(8) Å^3, Z = 8, T = 150.00(10) K, μ(Cu Kα) = 0.599 mm^{-1}, $Dcalc$ = 1.117 g/cm^3, 116612 reflections measured (7.334° ≤ 2Θ ≤ 146.682°), 9060 unique (R_{int} = 0.0329, R_{sigma} = 0.0118) which were used in all calculations. The final R_1 was 0.0409 (I > 2σ(I)) and wR_2 was 0.1153 (all data).

The C14-C23 adamantyl moiety was disordered by virtue of rotation about the C13-C14 bond. C15-C23 were modelled split across two components in relative occupancy 0.787(4):0.213(4) with C14 as a pivot atom in part 0.

The C26-C28 *iso*-propyl moiety was disordered and was treated with a split model with C26 as pivot atom in part 0, and C27-C28 split into two components with relative occupancy 0.655(8):0.345(8).

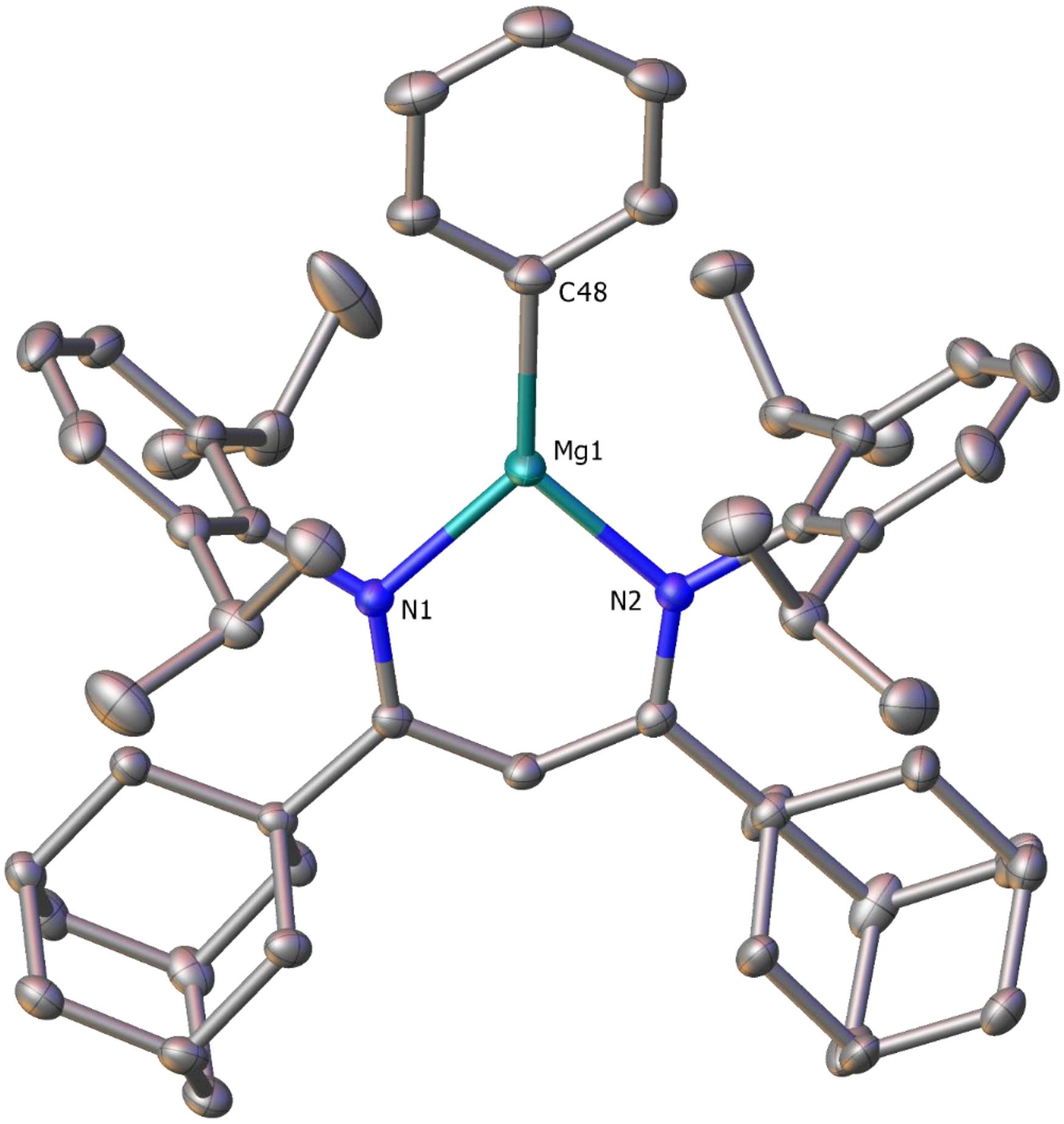

Figure S268: Displacement ellipsoid plot (30%) of **3**^{Ad}. Hydrogen atoms are omitted for clarity and only the major component of disordered *iso*-propyl and adamantyl moieties are shown.

5.4 Crystal structure determination of [(^{Me}BDI)MgCp^{4M}] (4)

CCDC Deposition Number 2557917.

4 crystallised as colourless needles by slow evaporation of a saturated benzene solution.

Crystal Data for $C_{38}H_{54}N_2Mg$ (*M* =563.14 g/mol): monoclinic, space group P2$_1$/n (no. 14), *a* = 11.37260(10) Å, *b* = 25.9880(2) Å, *c* = 12.03910(10) Å, *β* = 107.8690(10)°, *V* = 3386.52(5) Å^3, *Z* = 4, *T* = 150.00(10) K, μ(Cu Kα) = 0.639 mm^{-1}, *Dcalc* = 1.105 g/cm^3, 48790 reflections measured (6.802° ≤ 2Θ ≤ 160.47°), 7323 unique (R_{int} = 0.0270, R_{sigma} = 0.0186) which were used in all calculations. The final R_1 was 0.0483 (I > 2σ(I)) and wR_2 was 0.1316 (all data).

In the crystal structure **4**, the 1,2,3,4-tetramethylcyclopentadienyl moiety was disordered, such that a split model was employed with two components, whose relative occupancies were refined to 0.657(4) and 0.343(4). Distance restraints were employed in the refinement of the C-C and C-Mg bonds associated with the disordered moiety.

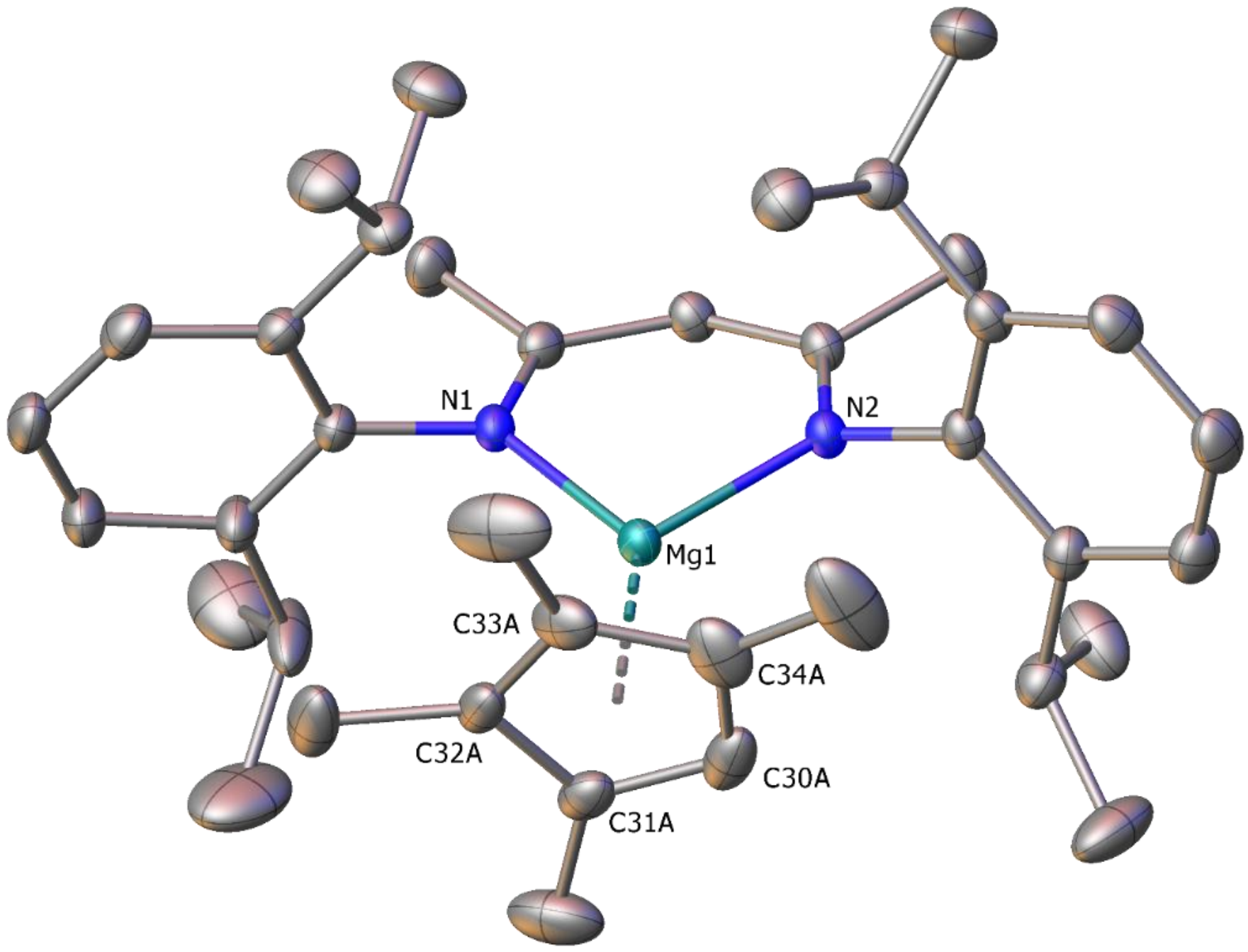

Figure S269: Displacement ellipsoid plot (30% level) of **4** with minor component of the disordered Cp^{4M} moiety omitted. Hydrogen atoms are also omitted for clarity.

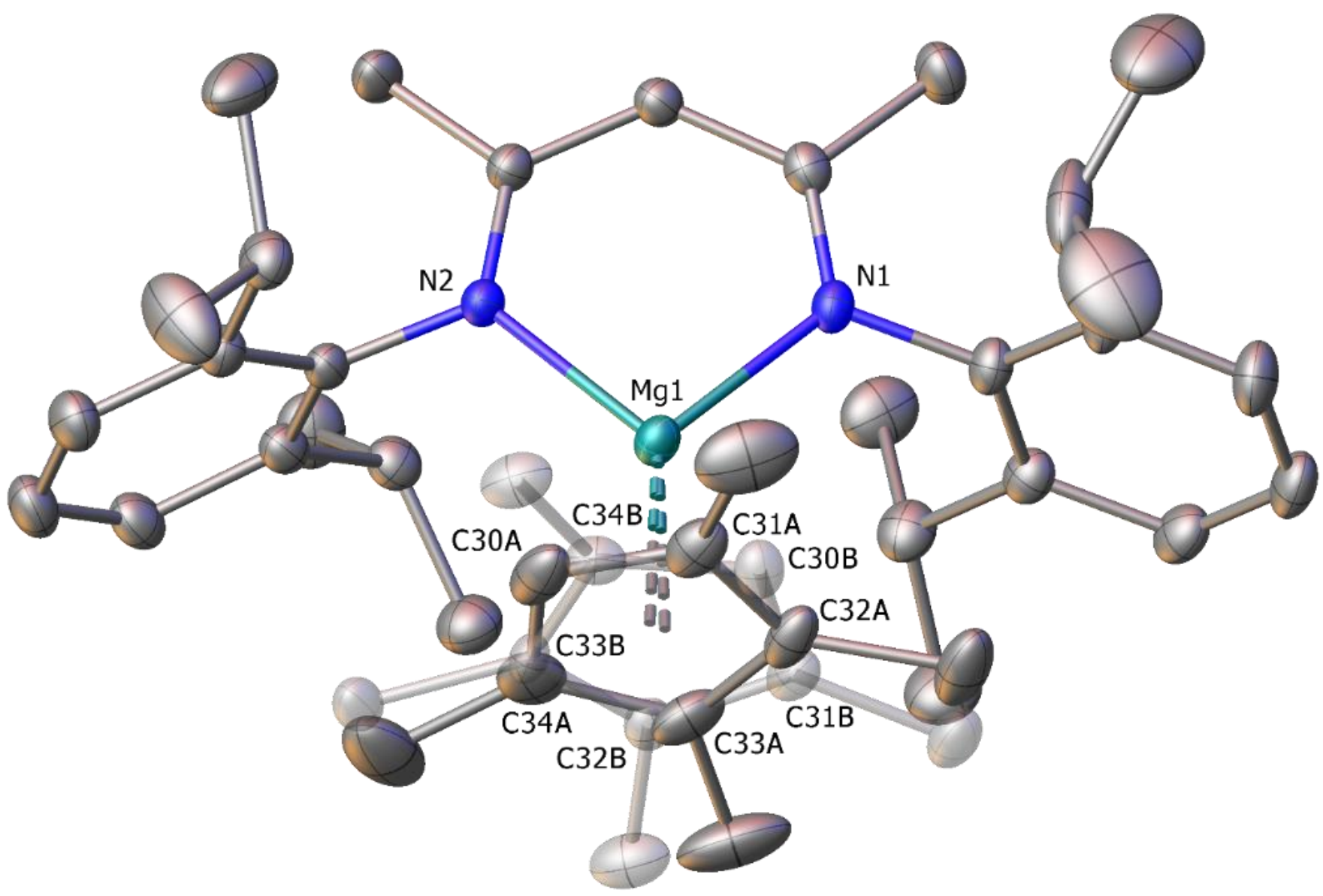

Figure S270: Displacement ellipsoid plot (30% level) of **4** with minor component of the disordered Cp^{4M} moiety shown as semi-transparent. Hydrogen atoms are omitted for clarity.

5.5 Crystal structure determination of [(^{Ad}BDI)Mg(C_6H_4Me)] (5-*m*, 5-*p*)

CCDC Deposition Number 2557919.

5-*m*/5-*p* crystallised as a disordered co-crystal of the *meta*- and *para*-tolyl isomers **5-*m*** and **5-*p*** as colourless blocks from the toluene reaction solution at 100°C.

Crystal Data for $C_{115}H_{152}Mg_2N_4$ (M =1639.02 g/mol): monoclinic, space group C2/c (no. 15), a = 37.5707(2) Å, b = 24.92530(10) Å, c = 21.20640(10) Å, β = 100.6010(10)°, V = 19520.01(17) Å^3, Z = 8, T = 150.15 K, μ(Cu Kα) = 0.588 mm^{-1}, $Dcalc$ = 1.115 g/cm^3, 127918 reflections measured

(7.092° ≤ 2Θ ≤ 146.524°), 19509 unique (R_{int} = 0.0248, R_{sigma} = 0.0136) which were used in all calculations. The final R_1 was 0.0637 (I > 2σ(I)) and wR_2 was 0.1871 (all data).

In the crystal structure of **5-*m*/5-*p***, the asymmetric unit contains 2 x Mg complexes and 1 molecule of toluene. In the molecule based on Mg1, the tolyl ligand based on C48 was treated for 70:30 disorder, as was the toluene solvent in the lattice. Disorder was rampant in the complex containing Mg2, such that all adamantyl carbons (barring C56) plus the tolyl ligand were treated for 82:18 disorder while the Dipp unit based on C55 was modelled to take account of 55:45 disorder. Appropriate distance and ADP restraints were included in disordered regions (to assist convergence) and all fractional occupancy rings were refined as rigid hexagons. Further residual electron density associated with each of the Mg complexes may arise from the presence of the isomeric magnesium benzyl complex [(^{Ad}BDI)Mg(CH_2Ph)] (**5'**) as a further minor component. Given that this component may be present in only minor quantities and the existing solution already involves a significant degree of disorder over two components, any attempts to model the benzyl isomer as a third component were abandoned.

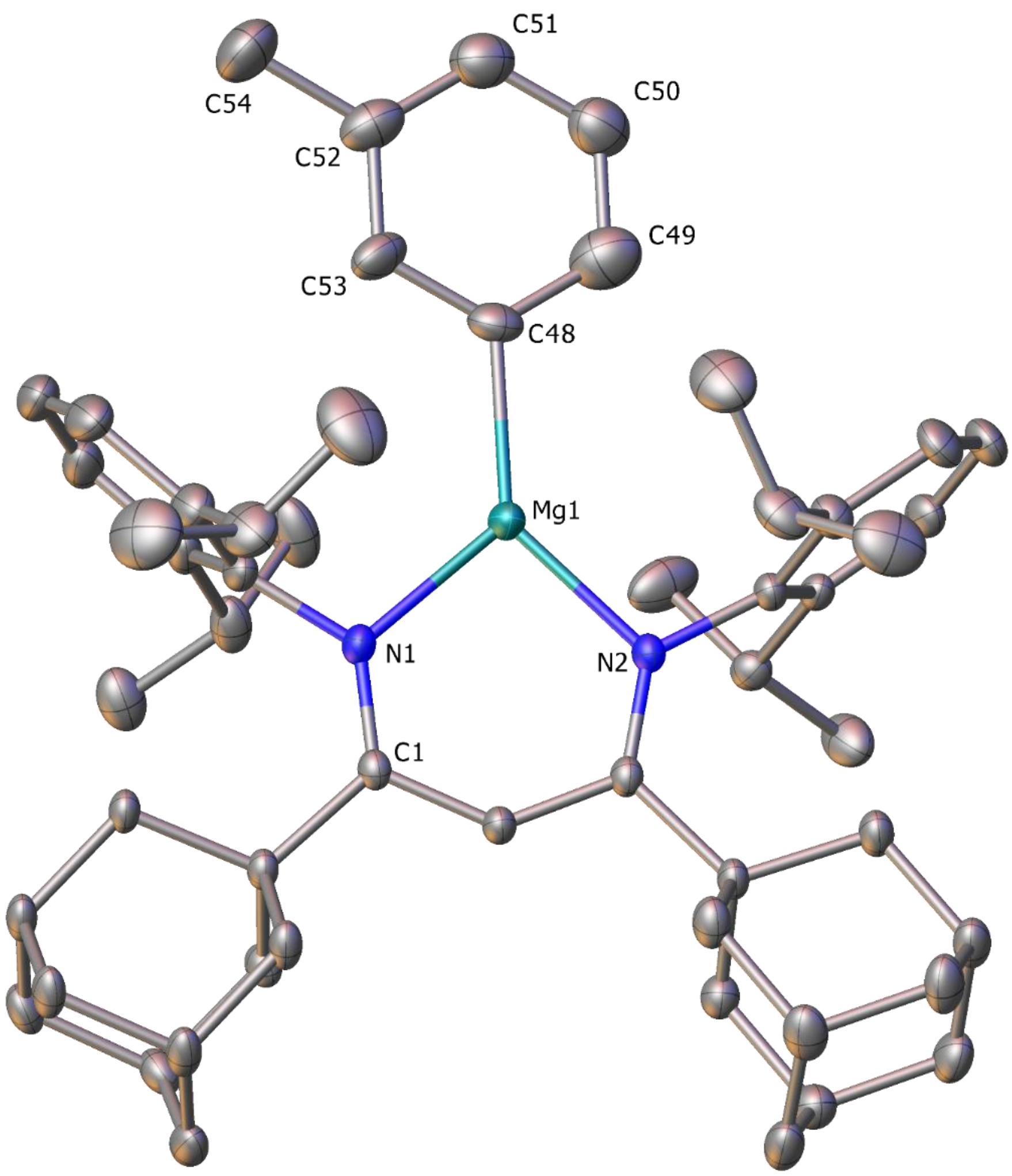

Figure S271: Displacement ellipsoid plot (30% level) of **5-*m*/5-*p***, showing the *meta*-isomer **5-*m*** of the Mg1-containing molecule.

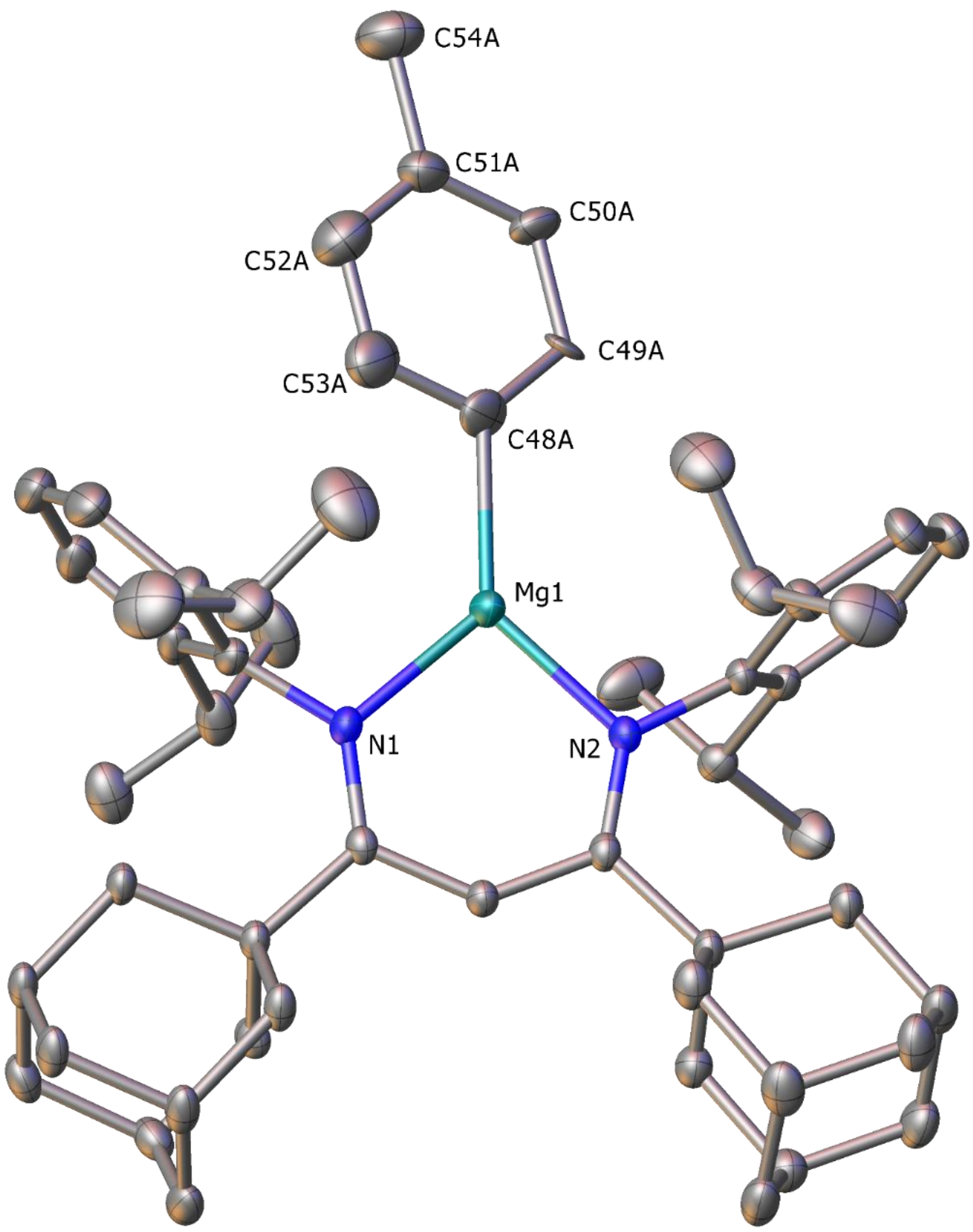

Figure S272: Displacement ellipsoid plot (30% level) of **5-*m*/5-*p***, showing the *para*-isomer **5-*p*** of the Mg1-containing molecule.

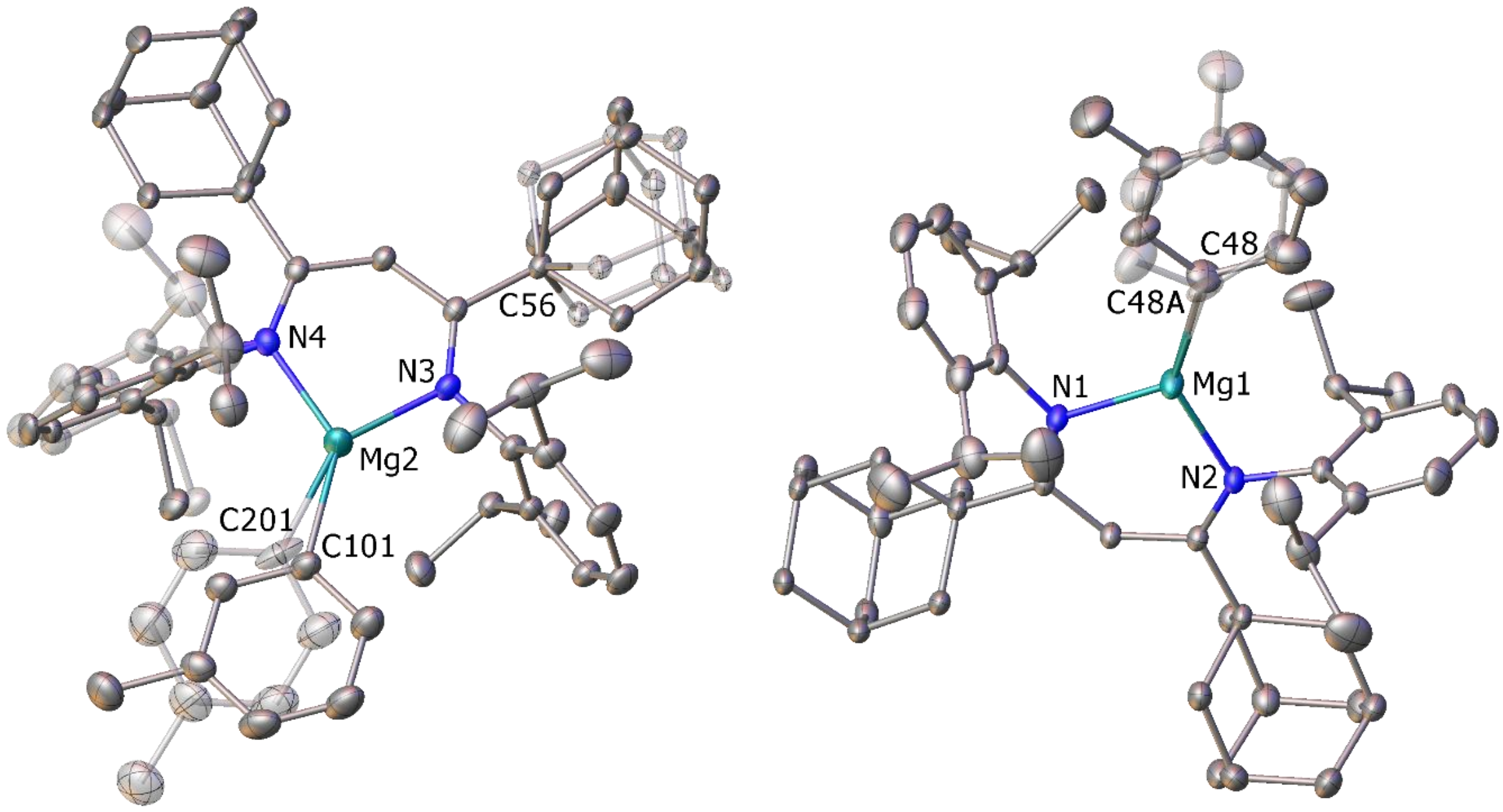

Figure S273: Displacement ellipsoid plot (30% level) of **5-*m*/5-*p***, with the minor components of disordered moieties shown as semi-transparent. The disordered molecule of toluene is not shown. Hydrogen atoms are omitted for clarity.

5.6 Crystal structure determination of [(^{Ad}BDI)Mg(CH_2Ph)] (5')

CCDC Deposition Number 2557918.

5' crystallised as pale-yellow blocks from a saturated *n*-pentane/benzene solution at -35°C.

Crystal Data for $C_{59.5}H_{81}MgN_2$ (M =848.57 g/mol): triclinic, space group P-1 (no. 2), a = 13.0928(3) Å, b = 14.0609(7) Å, c = 14.2101(4) Å, α = 80.439(3)°, β = 78.863(2)°, γ = 82.422(3)°, V = 2517.61(16) Å^3, Z = 2, T = 150.00(10) K, μ(Cu Kα) = 0.585 mm^{-1}, $Dcalc$ = 1.119 g/cm^3, 34865 reflections measured (6.406° ≤ 2Θ ≤ 140.148°), 9520 unique (R_{int} = 0.0356, R_{sigma} = 0.0331) which were used in all calculations. The final R_1 was 0.0477 (I > 2σ(I)) and wR_2 was 0.1377 (all data).

The crystal structure **5'** contains one magnesium complex and one region of disordered solvent. The latter moiety was modelled in two parts as superimposed *n*-pentane and benzene with relative occupancy of 0.5 each. Distance and ADP restraints were employed to assist with convergence, then relaxed. AFIX 66 and SIMU restraints were employed for the benzene molecule, and SIMU restraints for the *n*-pentane in the final model.

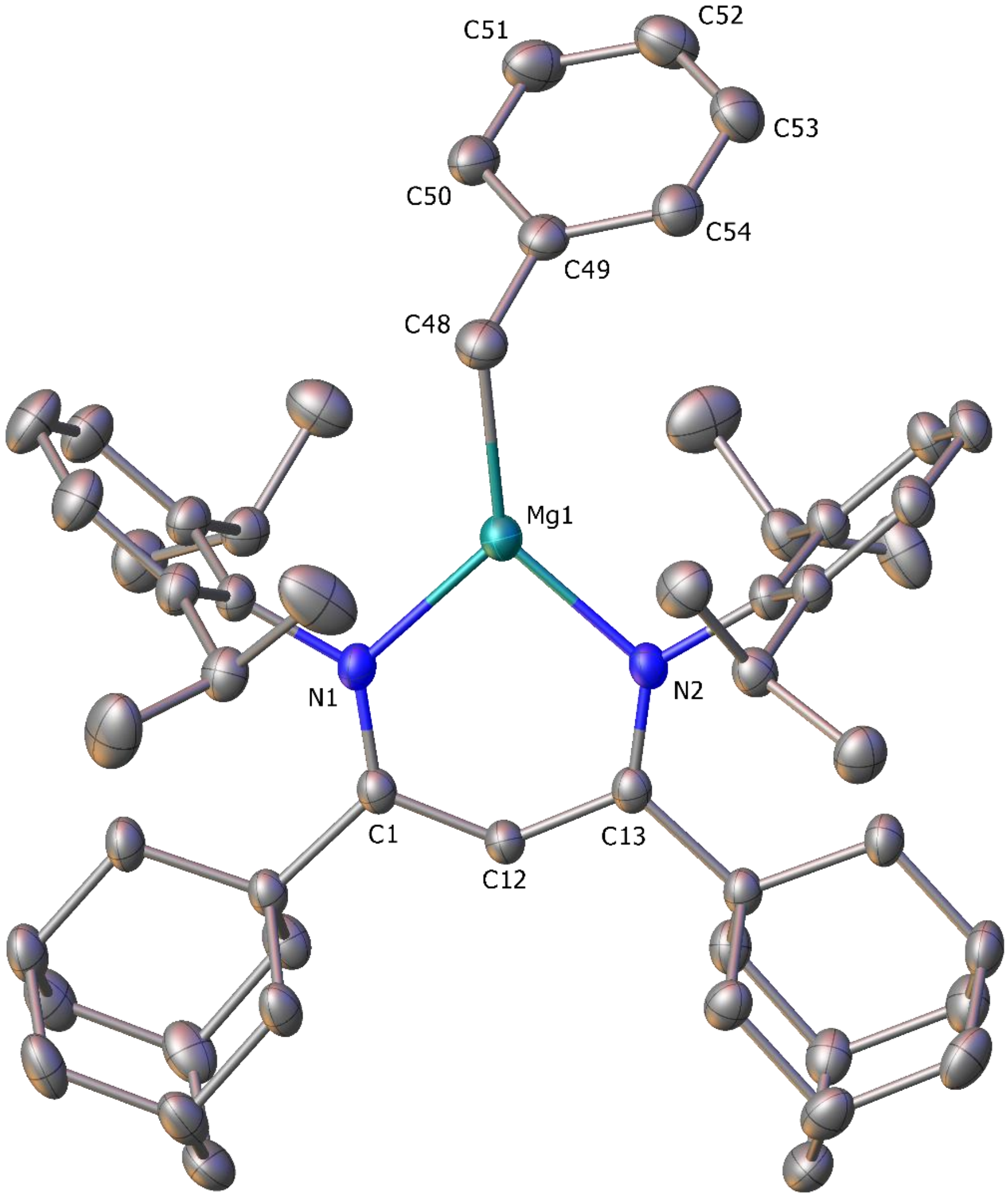

Figure S274: Displacement ellipsoid plot (30% level) of **5'**, with the minor components of disordered moieties shown as semi-transparent. The disordered solvent molecules are not shown. Hydrogen atoms are omitted for clarity.

5.7 Crystal structure determination of [(^{Ad}BDI)Mg(C_6H_3-3,4-Me_2)]/ [(^{Ad}BDI)Mg($CH_2C_6H_4$-2-Me)] (6/6')

CCDC Deposition Number 2557920.

6/6' crystallised as pale-yellow blocks of a respective 0.8:0.2 disordered co-crystal from *n*-hexane at 80°C.

Crystal Data for $C_{55}H_{74}MgN_2$ (M =787.47 g/mol): orthorhombic, space group Pbca (no. 61), a = 13.3268(3) Å, b = 24.1532(5) Å, c = 29.2645(8) Å, V = 9419.8(4) Å^3, Z = 8, T = 150.00(10) K, μ(Cu Kα) = 0.590 mm^{-1}, $Dcalc$ = 1.111 g/cm^3, 80703 reflections measured (7.32° ≤ 2Θ ≤ 147.264°), 9436 unique (R_{int} = 0.0592, R_{sigma} = 0.0300) which were used in all calculations. The final R_1 was 0.0690 (I > 2σ(I)) and wR_2 was 0.1984 (all data).

The X-ray crystal structure **6/6'** was solved as a co-crystalline mixture of the regioisomers **6** and **6'**. A split model was employed with an occupancy of 0.8 the major component, **6**, and 0.2 for the minor **6'** component. Additionally, the meta-methyl moiety in the major component, **6**, was disordered over two positions related by rotation about the Mg1-C48 bond and was resolved using a split model with relative occupancies of 0.5 and 0.3 for C55 and C55a, respectively. The corresponding disordered aromatic hydrogen atoms (H50 and H52) with respective fractional occupancies of 0.5 and 0.3 positions were included with appropriate distance and planarity

restraints. The C1-bound adamantyl group was disordered with respect to rotation about the C1-C2 bond and was refined using a two-component split model with relative occupancy 0.878/0.122. Besides the specific restraints outlined herein, additional, appropriate distance and ADP restraints were employed in the disordered regions, to assist with convergence.

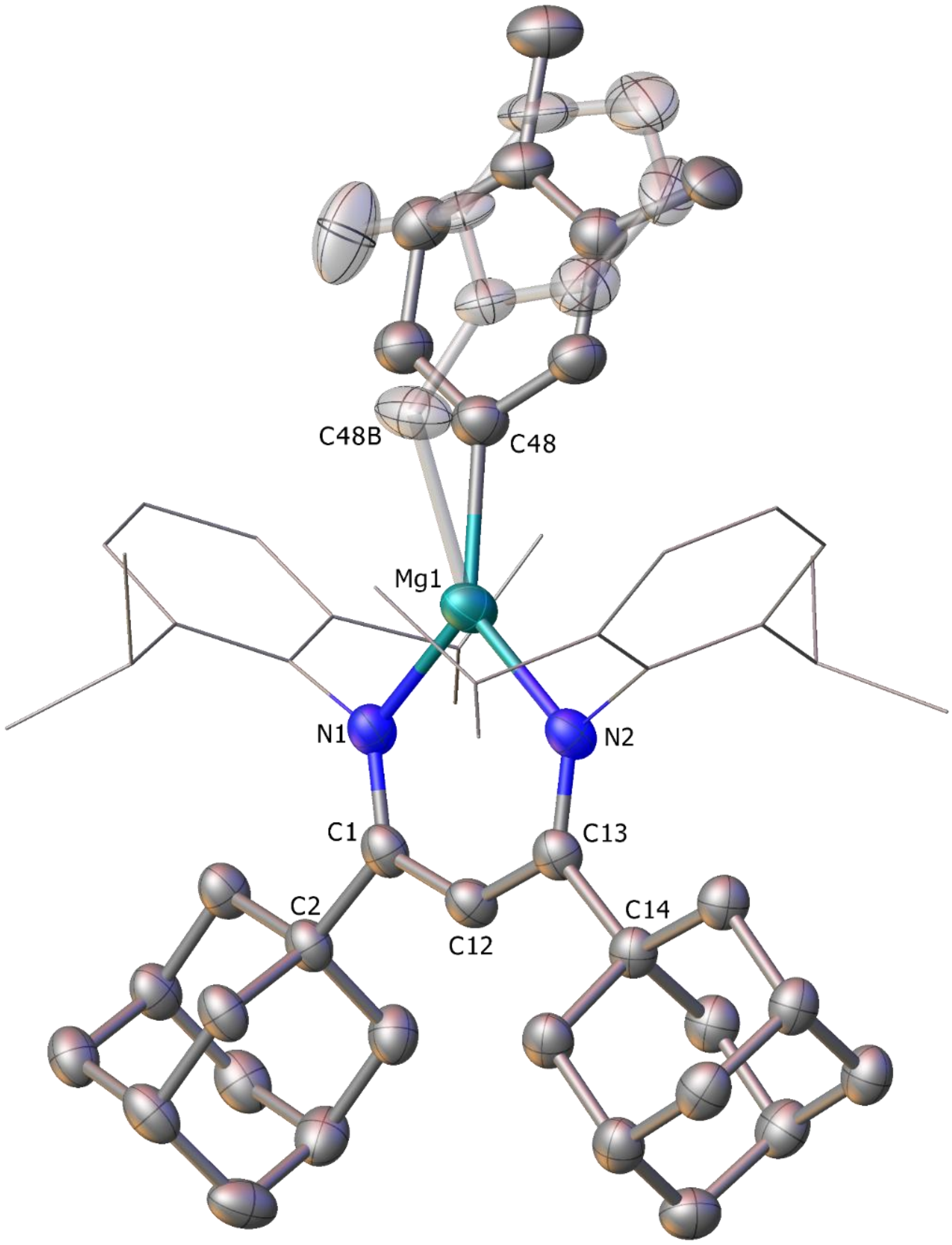

Figure S275: Displacement ellipsoid plot (30% level) of the co-crystal of **6** (80%) and **6'** (20%). For clarity, hydrogen atoms are omitted, dipp groups shown as wireframes, and only the major component of the disordered adamantyl and xylyl moieties are shown.

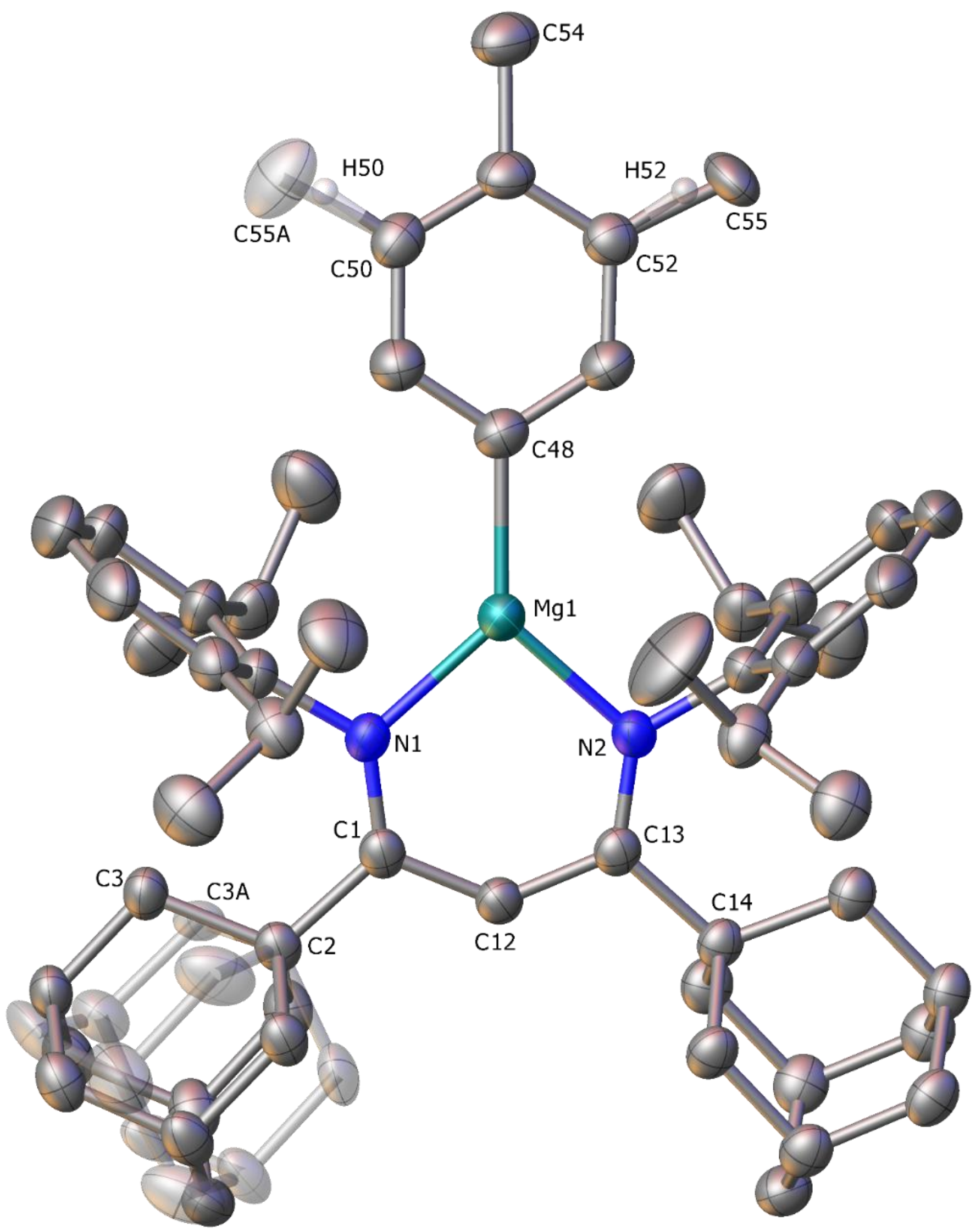

Figure S276: Displacement ellipsoid plot (30% level) showing the major **6** (80%) component of the co-crystal of **6** (80%) and **6'** (20%). For clarity, hydrogen atoms are omitted, dipp groups shown as wireframes. The minor components of the disordered xylyl (0.3:0.5) and adamantyl (0.88:0.12) moieties are shown semi-transparent.

5.8 Crystal structure determination of [(^{Ad}BDI)Mg(C_6H_3-3,5-Me_2)] (7)

CCDC Deposition Number 2557921.

7 crystallised as colourless plates from the *meta*-xylene reaction solution at 100°C.

Crystal Data for $C_{55}H_{74}MgN_2$ (M =787.47 g/mol): monoclinic, space group P2_1/n (no. 14), a = 10.8827(2) Å, b = 17.8000(4) Å, c = 24.4969(4) Å, β = 99.455(2)°, V = 4680.88(16) Å^3, Z = 4, T = 150.00(10) K, μ(Cu Kα) = 0.594 mm^{-1}, $Dcalc$ = 1.117 g/cm^3, 63257 reflections measured (8.446° ≤ 2Θ ≤ 147.406°), 9369 unique (R_{int} = 0.0861, R_{sigma} = 0.0504) which were used in all calculations. The final R_1 was 0.0579 (I > 2σ(I)) and wR_2 was 0.1660 (all data).

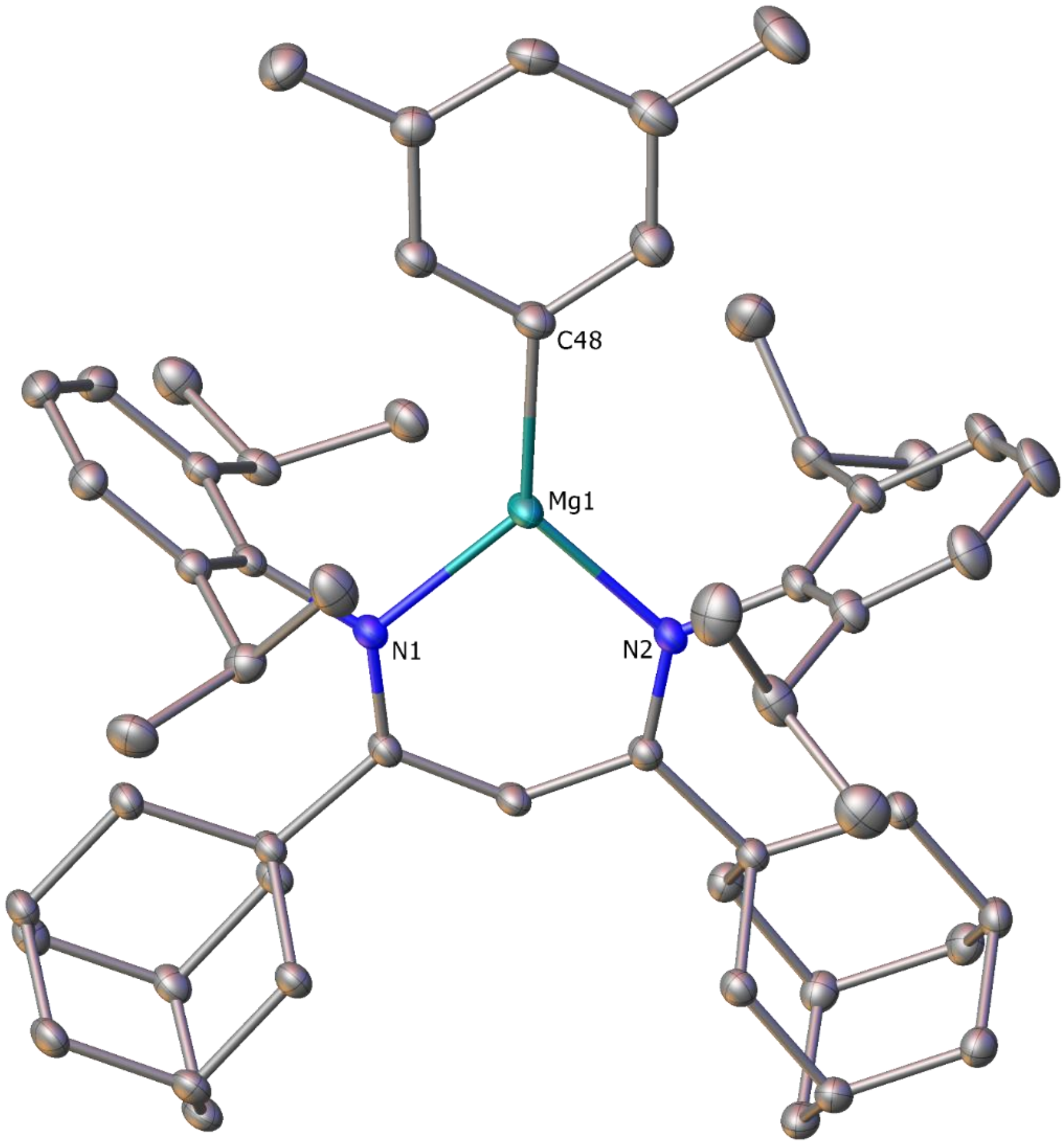

Figure S277: Displacement ellipsoid plot (30%) of the crystal structure of **7**. Hydrogen atoms are omitted for clarity.

5.9 Crystal structure determination of [(^{Ad}BDI)Mg($CH_2C_6H_4$-3-Me)] (7')

CCDC Deposition Number 2557922.

7' crystallised by slow evaporation of a saturated *n*-hexane solution at room temperature as pale-yellow blocks.

Crystal Data for $C_{55}H_{74}N_2Mg$ (M =787.47 g/mol): monoclinic, space group P2$_1$/c (no. 14), a = 9.94940(10) Å, b = 13.64860(10) Å, c = 34.8915(2) Å, β = 96.9110(10)°, V = 4703.68(7) Å^3, Z = 4, T = 150.00(10) K, μ(Cu Kα) = 0.591 mm^{-1}, $Dcalc$ = 1.112 g/cm^3, 79298 reflections measured (5.102° ≤ 2Θ ≤ 160.742°), 10198 unique (R_{int} = 0.0318, R_{sigma} = 0.0189) which were used in all calculations. The final R_1 was 0.0464 (I > 2σ(I)) and wR_2 was 0.1243 (all data).

The X-ray crystal structure of **7'** was found to contain a co-crystalline mixture of the two isomers **7'** and **7** in a relative occupancy ratio of 0.9:0.1. A split model was employed to model this disorder. To aid convergence, it was found to be necessary to apply DFIX and RIGU restraints to the minor component. Distances and angles for this component should, thus, be treated with caution.

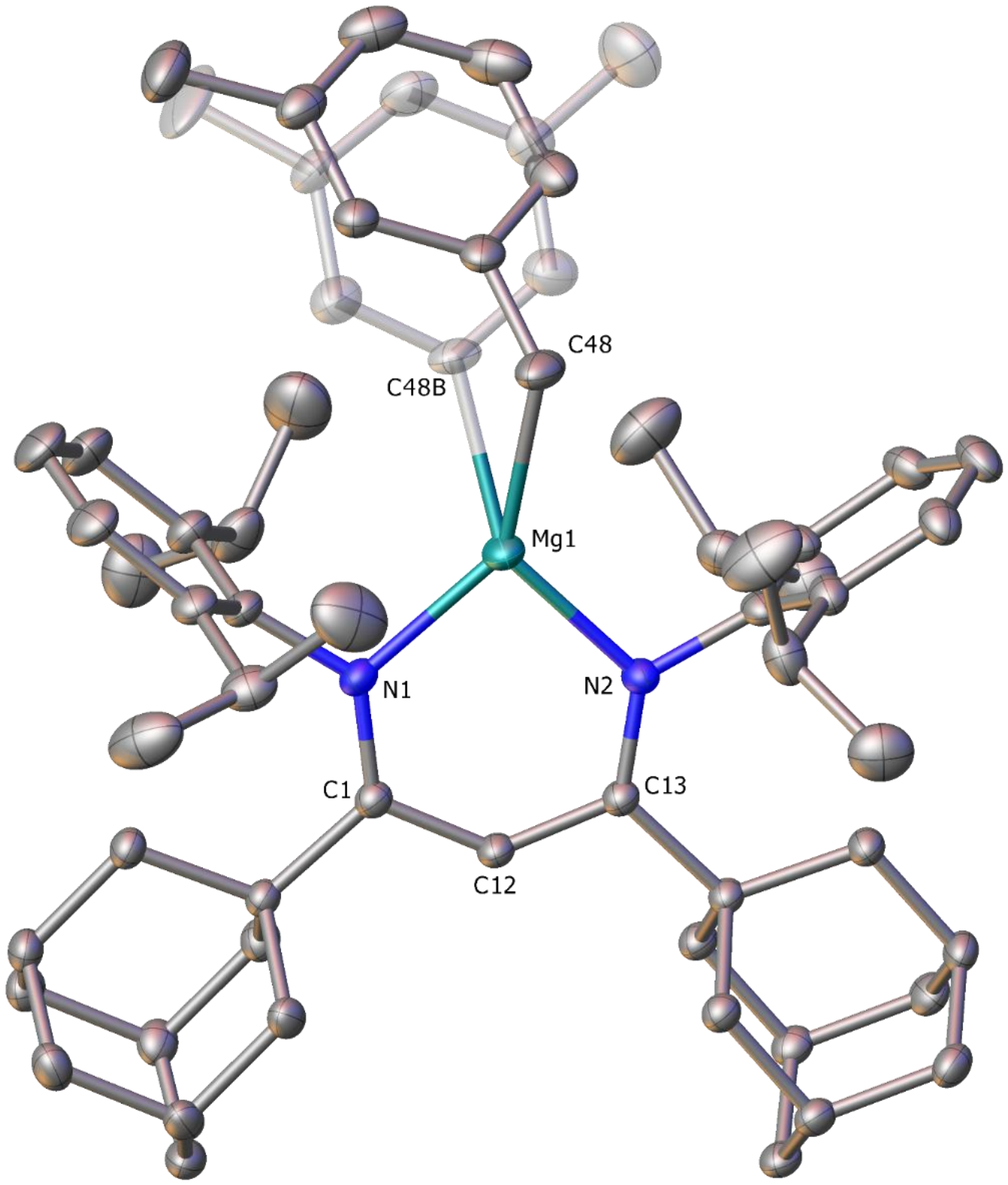

Figure S278: Displacement ellipsoid plot (30%) of the crystal structure of **7'**, showing the minor (10%) co-crystalline component of **7** (semi-transparent). Hydrogen atoms are omitted for clarity.

5.10 Crystal structure determination of [(^{Ad}BDI)Mg($CH_2C_6H_4$-4-Me)] (8)

CCDC Deposition Number 2557923.

8 crystallised from an *n*-hexane/toluene solution at -30°C as pale-yellow rods.

Crystal Data for $C_{61}H_{88}N_2Mg$ (M =873.64 g/mol): triclinic, space group P-1 (no. 2), a = 13.5887(2) Å, b = 14.7112(2) Å, c = 15.3361(2) Å, α = 112.6660(10)°, β = 94.4360(10)°, γ = 106.0660(10)°, V = 2659.34(7) Å^3, Z = 2, T = 150.00(10) K, μ(Cu Kα) = 0.564 mm^{-1}, $Dcalc$ = 1.091 g/cm^3, 38610 reflections measured (6.384° ≤ 2Θ ≤ 148.996°), 10792 unique (R_{int} = 0.0308, R_{sigma} = 0.0302) which were used in all calculations. The final R_1 was 0.0474 (I > 2σ(I)) and wR_2 was 0.1407 (all data).

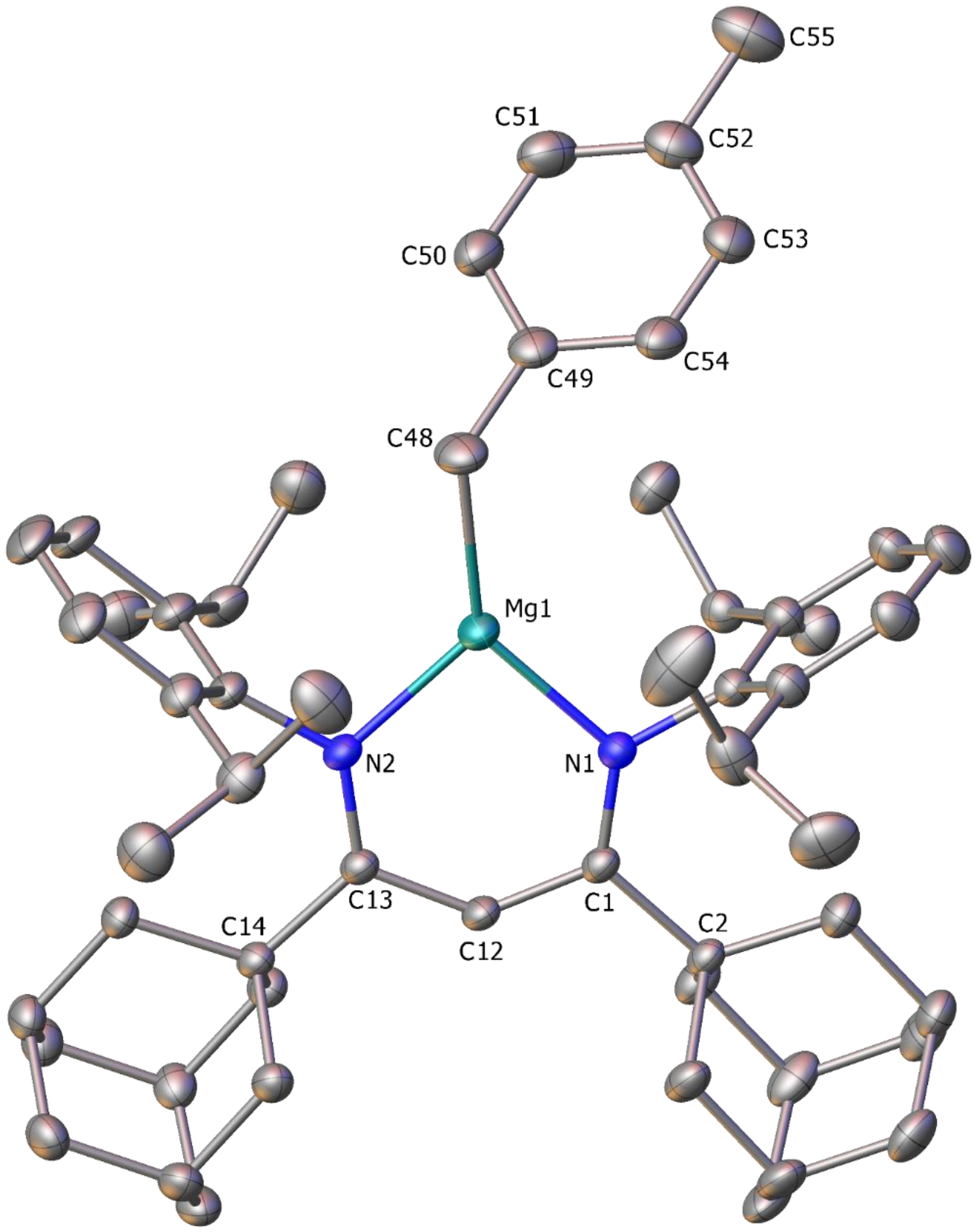

Figure S279: Displacement ellipsoid plot (30% level) of the crystal structure of **8**. Hydrogen atoms are omitted for clarity.

5.11 Crystal structure determination of [(^{Ad}BDI)Mg(C_6H_3-3,5-$^tBu_2$)] (9)

CCDC Deposition Number 2557924.

9 crystallised as colourless blocks from the 1,3-di(tert-butyl)benzene reaction solution at 120°C.

Crystal Data for $C_{61}H_{86}N_2Mg$ (M =871.62 g/mol): orthorhombic, space group $P2_12_12_1$ (no. 19), a = 10.18700(10) Å, b = 21.54160(10) Å, c = 24.25800(10) Å, V = 5323.28(6) Å^3, Z = 4, T = 150.00(10) K, μ(Cu Kα) = 0.563 mm^{-1}, $Dcalc$ = 1.088 g/cm^3, 72356 reflections measured (7.288° ≤ 2Θ ≤ 146.456°), 10657 unique (R_{int} = 0.0299, R_{sigma} = 0.0178) which were used in all calculations. The final R_1 was 0.0307 (I > 2σ(I)) and wR_2 was 0.0817 (all data).

The *tert*-butyl methyl groups attached to C58 were split in two components of relative occupancy 0.781(14):0.219(14). Restraints were employed to assist with modelling disorder.

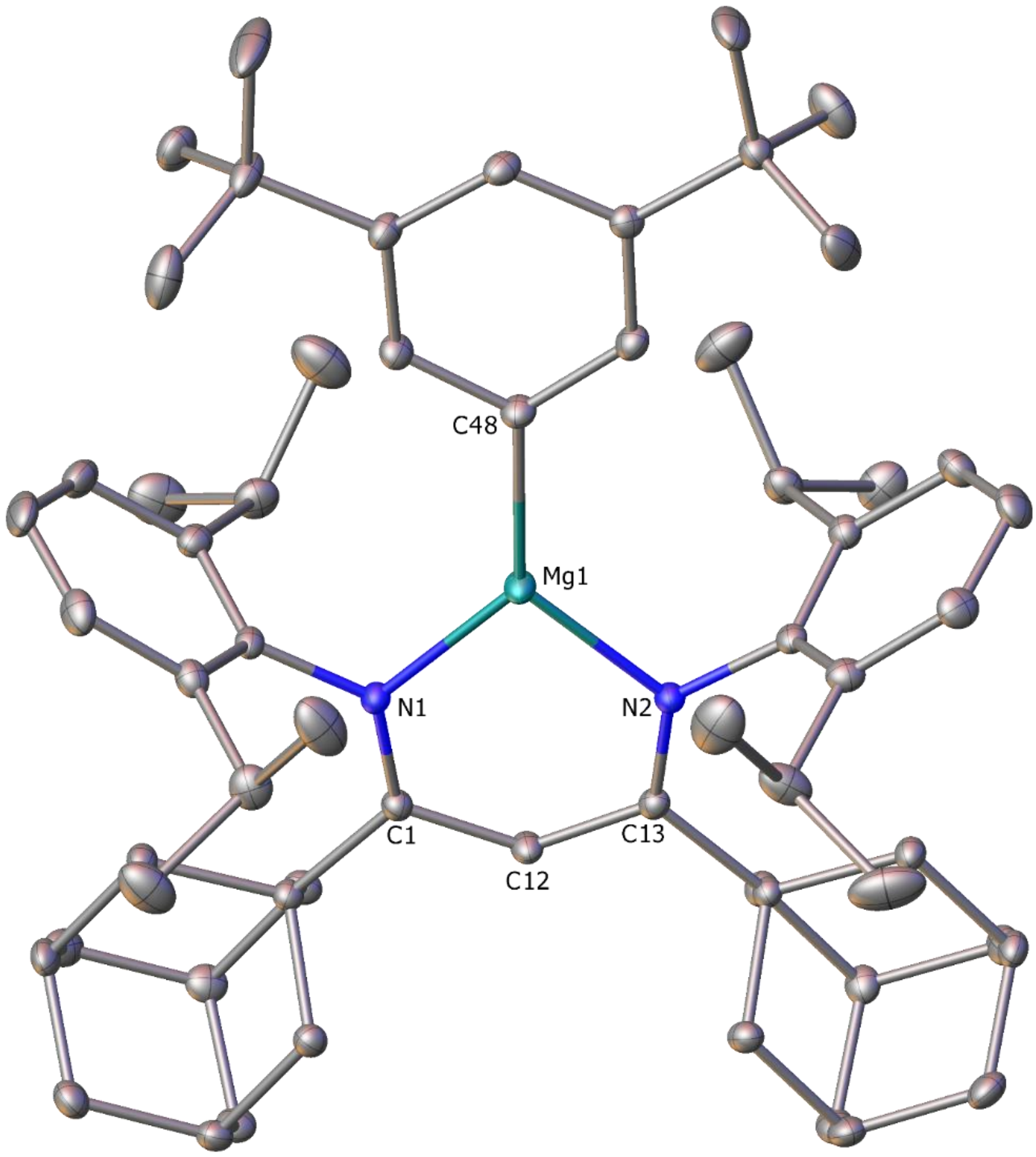

Figure S280: Displacement ellipsoid plot (30% level) of **9**. Hydrogen atoms are omitted for clarity and only the major component of the disordered *tert*-butyl group is shown.

5.12 Crystal structure determination of [(^{Me}BDI)Mg(κ^2*C,O*-C_6H_4-2-OMe)(thf)] (10^{Me}·thf)

CCDC Deposition Number 2557926.

10^{Me}·thf crystallised as colourless plates from an *n*-hexane/THF solution at -30°C.

Crystal Data for $C_{40}H_{56}MgN_2O_2$ (*M* =621.17 g/mol): monoclinic, space group P2$_1$/c (no. 14), *a* = 17.2527(2) Å, *b* = 11.6656(2) Å, *c* = 18.3717(3) Å, *β* = 92.3100(10)°, *V* = 3694.54(10) Å^3, *Z* = 4, *T* = 150.00(10) K, μ(Cu Kα) = 0.671 mm^{-1}, *Dcalc* = 1.117 g/cm^3, 50716 reflections measured (8.982° ≤ 2Θ ≤ 146.346°), 7394 unique (R_{int} = 0.0402, R_{sigma} = 0.0233) which were used in all calculations. The final R_1 was 0.0542 (I > 2σ(I)) and wR_2 was 0.1468 (all data).

The crystal structure of **10^{Me}·thf** contains one magnesium complex per asymmetric unit, in which the κ$_2$-anisyl ligand is disordered over two sites. The disorder was resolved by employing a split model with the anisyl in two components of relative occupancy 0.8:0.2, without any further restraints.

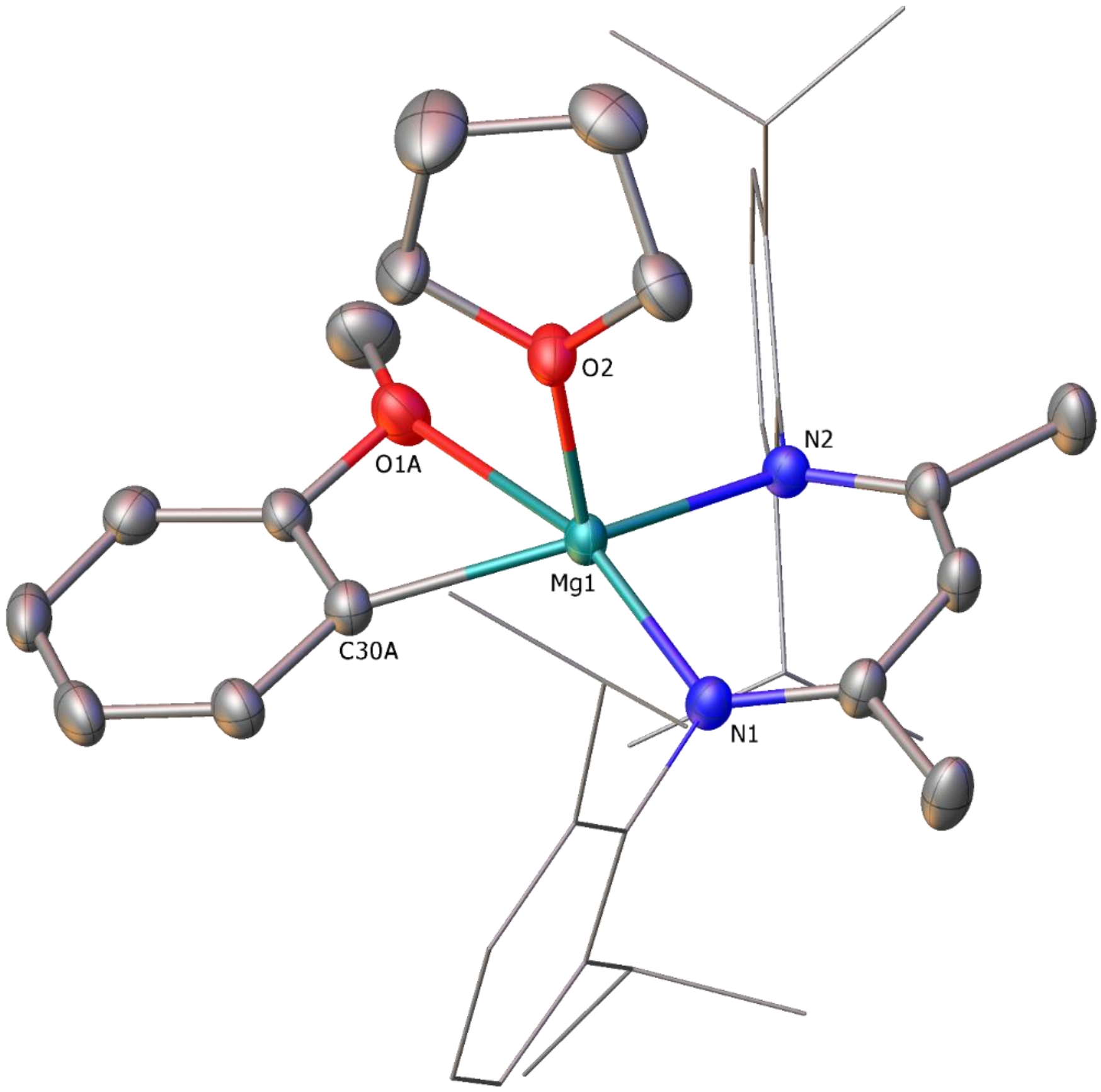

Figure S281: Displacement ellipsoid plot (30% level) of the crystal structure of **10^{Me}·thf**. Only the major component of the disordered anisyl moiety is shown, dipp groups are shown as wireframes, and hydrogen atoms are omitted for clarity.

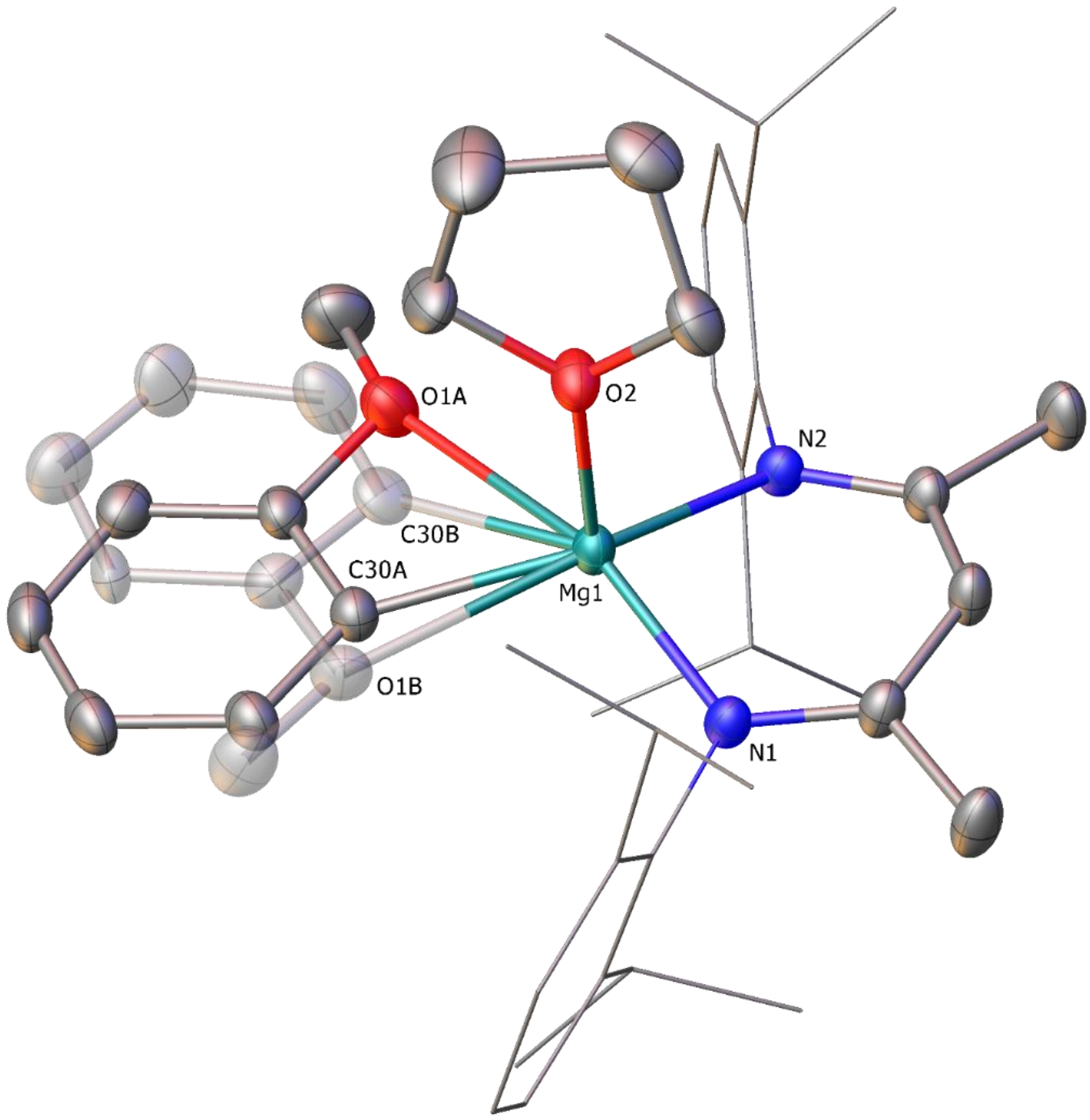

Figure S282: Displacement ellipsoid plot (30% level) of the crystal structure of **10^{Me}·thf**. The minor component of the disordered anisyl moiety is shown semi-transparent, dipp groups are shown as wireframes, and hydrogen atoms are omitted for clarity.

5.13 Crystal structure determination of [(^{Me}BDI)Mg(κ^1,*C*-C_6H_4-2-OMe)(κ^1,*O*-C_6H_5OMe)] (10^{Ad}·anisole)

CCDC Deposition Number 2557925.

10^{Ad}·anisole crystallised as colourless needles after slow evaporation of a saturated benzene solution at room temperature.

Crystal Data for $C_{61}H_{80}MgN_2O_2$ (*M* =897.58 g/mol): triclinic, space group P-1 (no. 2), *a* = 10.8852(11) Å, *b* = 12.7840(8) Å, *c* = 20.4936(12) Å, *α* = 99.937(5)°, *β* = 91.209(6)°, *γ* = 114.216(8)°, *V* = 2548.4(4) Å^3, *Z* = 2, *T* = 149.99(14) K, μ(Cu Kα) = 0.636 mm^{-1}, *Dcalc* = 1.170 g/cm^3, 22383 reflections measured (4.4° ≤ 2Θ ≤ 165.6°), 22383 unique (R_{int} = ?, R_{sigma} = 0.0500) which were used in all calculations. The final R_1 was 0.0946 (I > 2σ(I)) and wR_2 was 0.3313 (all data).

The crystal structure **10^{Ad}·anisole** was refined as a two-component twin 0.6723(16):0.3277(16) with twin law of 1.0209 0.0453 0.0310 -0.0532 0.9684 -0.0374 -0.0637 0.0636 1.0044 for the second component.

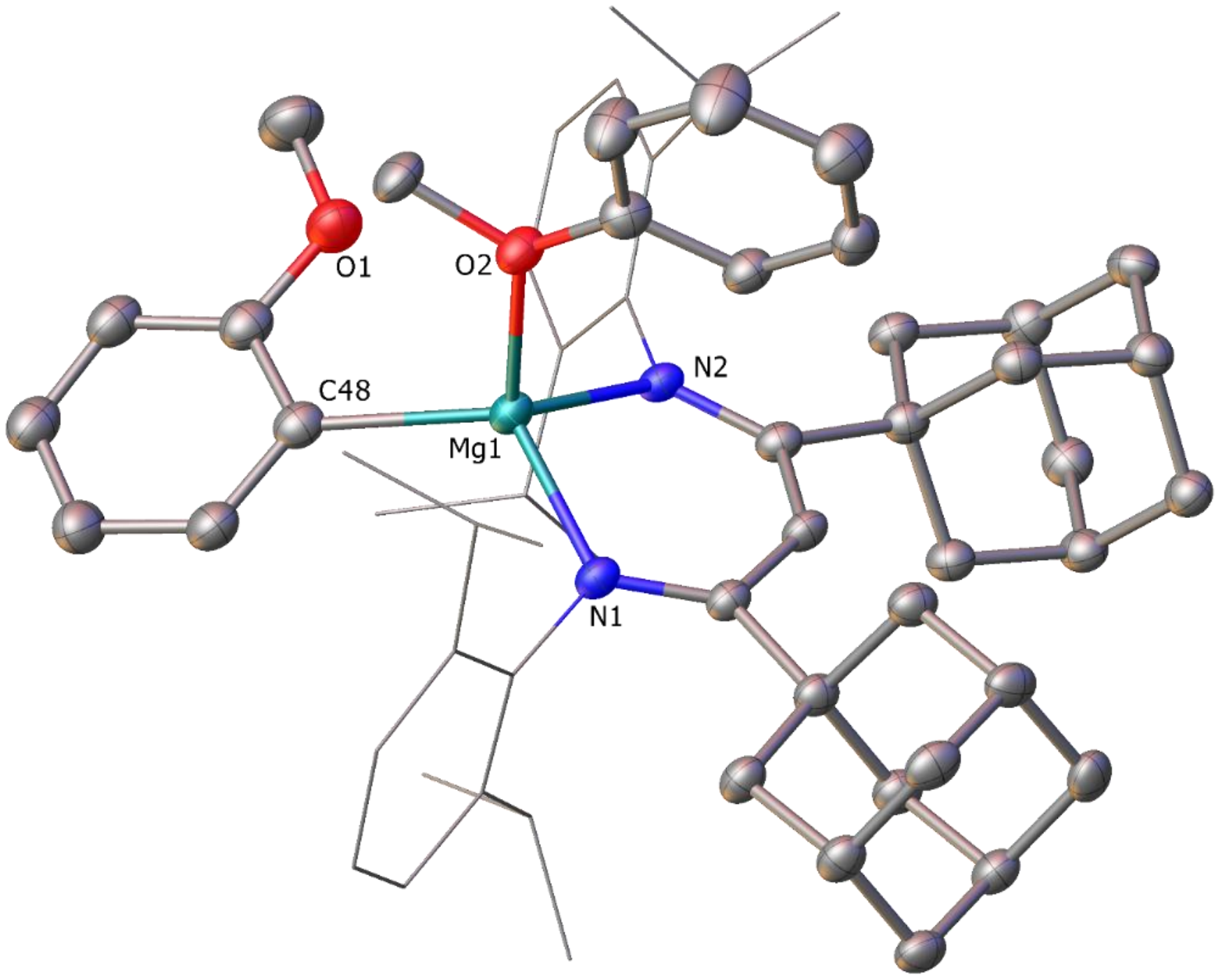

Figure S283: Displacement ellipsoid plot (30% level) of the crystal structure of **9^{Ad}·anisole**. Dipp groups are shown as wireframes and hydrogen atoms are omitted for clarity.

5.14 Crystal structure determination of [(κ^2-^{Ad}BDI)Mg(μ-OMe)$_2$Mg(κ^1-^{Ad}BDI)] (11)

CCDC Deposition Number 2557927.

11 crystallised as colourless plates from saturated benzene/n-hexane solution at room temperature.

Crystal Data for $C_{111}H_{159}Mg_2N_4O_2$ (M =1630.03 g/mol): triclinic, space group P-1 (no. 2), a = 10.07720(10) Å, b = 22.3784(2) Å, c = 24.4296(3) Å, α = 114.4420(10)°, β = 98.8080(10)°, γ = 97.8680(10)°, V = 4831.94(10) Å^3, Z = 2, T = 149.99(14) K, μ(Cu Kα) = 0.604 mm^{-1}, $Dcalc$ = 1.120 g/cm^3, 79267 reflections measured (4.448° ≤ 2Θ ≤ 161.124°), 20566 unique (R_{int} = 0.0591, R_{sigma} = 0.0502) which were used in all calculations. The final R_1 was 0.0580 (I > 2σ(I)) and wR_2 was 0.1591 (all data).

In the crystal structure of **11** there is one dinuclear magnesium complex and two regions of disordered solvent per asymmetric unit. One region of solvent was modelled as one molecule of benzene, which was treated as a rigid hexagon and split over two positions with relative occupancy 0.56:0.44. A SIMU restraint was also imposed on this moiety during refinement. Although some peaks of electron density in the remaining region of lattice solvent could be reasonably refined as half a molecule of benzene when treated as a rigid hexagon, significant residual electron density in the same region remained and could not be satisfactorily modelled as any solvent molecule. Instead, a solvent mask was calculated and 140 electrons were found in a volume of 742 Å^3 in 2 voids per unit cell. This is consistent with the presence of 0.5 molecules of benzene, and one molecule of n-hexane per Asymmetric Unit, which account for 142 electrons per unit cell.

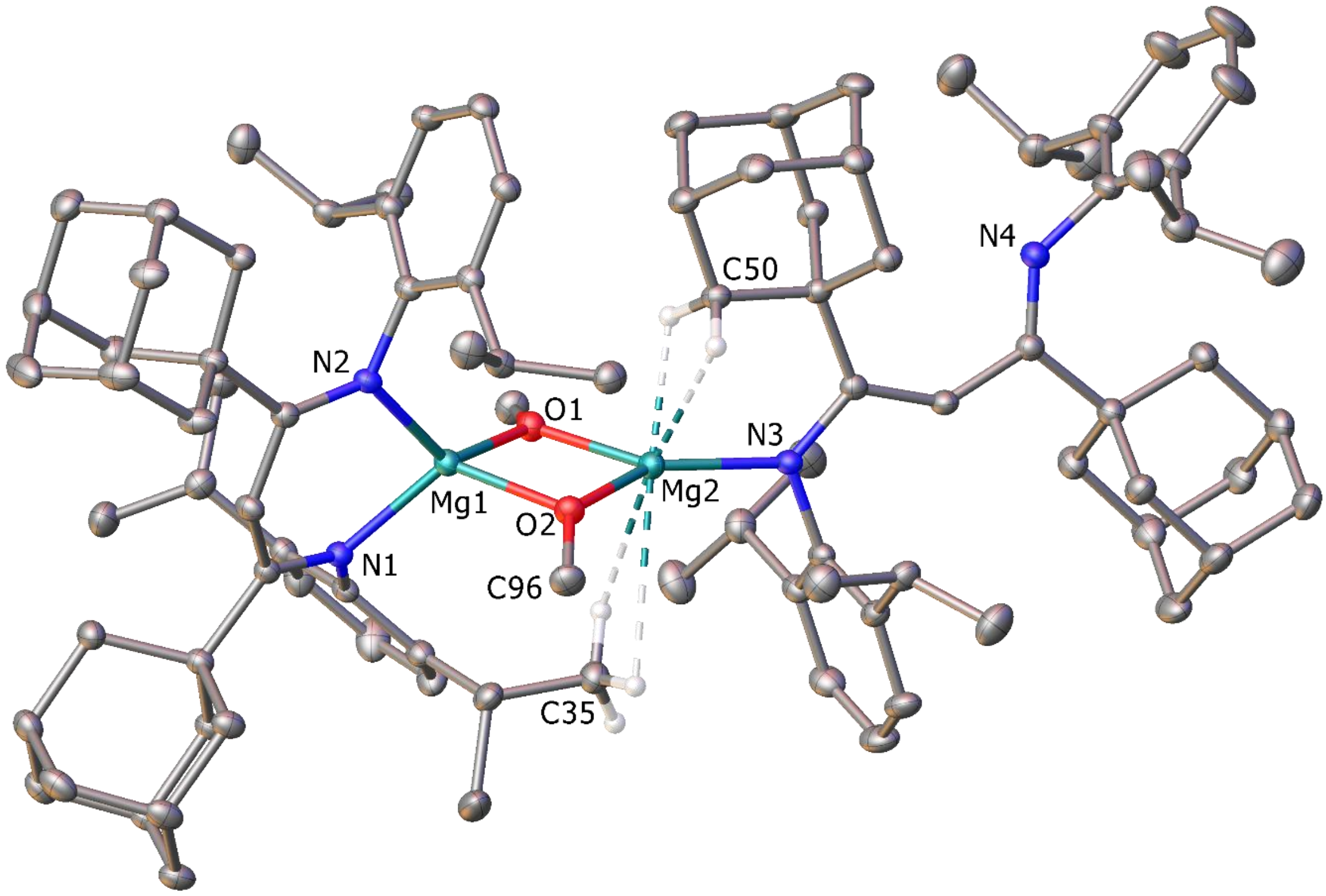

Figure S284: Displacement ellipsoid plot (30%) of the crystal structure of **11**. Hydrogen atoms are omitted for clarity, except for those bound to C35 and C50. Solvent molecules are also omitted for clarity.

5.15 Crystal structure determination of [(AdBDI)Mg(μ-OMe)$_2$(Bpin)] (12)

CCDC Deposition Number 2557928.

12 crystallised as bright yellow blocks from a saturated *n*-hexane/toluene solution at -35°C.

Crystal Data for $C_{55}H_{83}BMgN_2O_4$ (*M* =871.35 g/mol): monoclinic, space group P2$_1$/c (no. 14), a = 17.44550(10) Å, b = 13.91910(10) Å, c = 21.29260(10) Å, β = 99.2870(10)°, V = 5102.62(5) Å^3, Z = 4, T = 150.00(10) K, μ(Cu Kα) = 0.644 mm^{-1}, *Dcalc* = 1.134 g/cm^3, 69937 reflections measured (7.618° ≤ 2Θ ≤ 146.386°), 10212 unique (R_{int} = 0.0268, R_{sigma} = 0.0159) which were used in all calculations. The final R_1 was 0.0393 (I > 2σ(I)) and wR_2 was 0.1123 (all data).

Residual electron density was observed close to the pinacolate moiety, most likely due to disorder in the pinacolate backbone. Whilst modelling the residual electron density as a second component with relative occupancy 0.1 did provide an improved R1 (3.58%), the resulting model required a significant level of restraint to converge with realistic bond lengths, angles, and ADPs. As such, the structure solution is presented without modelling the minor disordered component.

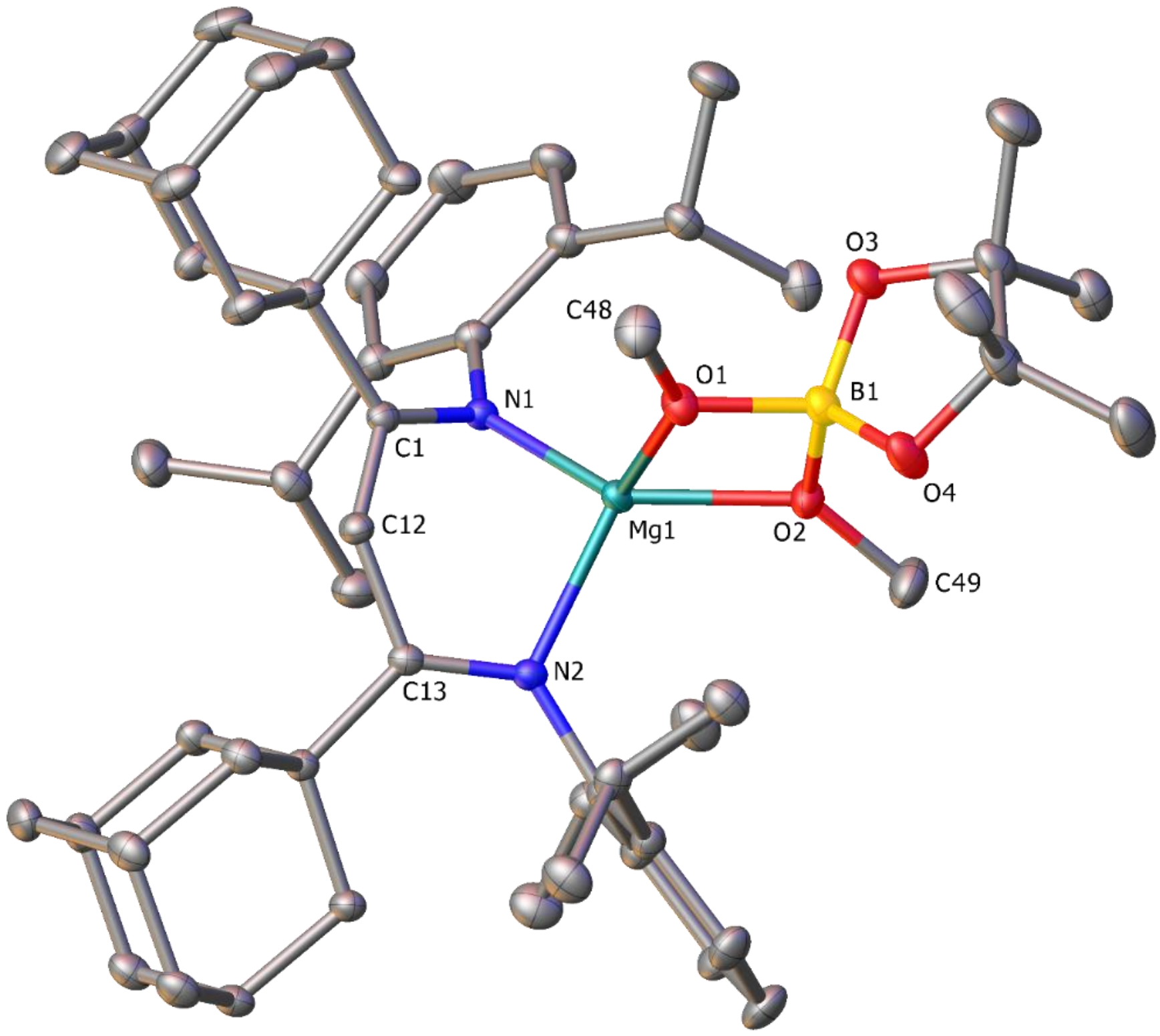

Figure S285: Displacement ellipsoid plot (30%) of the crystal structure of **12**. Hydrogen atoms are omitted for clarity.

5.16 Crystal structure determination of [(^{Ad}BDI)MgBr] (13)

CCDC Deposition Number 2557929.

13 crystallised from the benzene-d_6 solution of a palladium-catalysed Kumada cross-coupling of **3^{Ad}** with bromobenzene, after cooling the reaction mixture from 60°C to room temperature.

Crystal Data for $C_{65}H_{83}BrMgN_2$ (*M* =996.55 g/mol): orthorhombic, space group Pnma (no. 62), *a* = 21.05140(10) Å, *b* = 17.07070(10) Å, *c* = 15.72880(10) Å, *V* = 5652.34(6) Å^3, *Z* = 4, *T* = 150.00(10) K, μ(Cu Kα) = 1.391 mm^{-1}, *Dcalc* = 1.171 g/cm^3, 74325 reflections measured (7.016° ≤ 2Θ ≤ 146.402°), 5869 unique (R_{int} = 0.0279, R_{sigma} = 0.0120) which were used in all calculations. The final R_1 was 0.0336 (I > 2σ(I)) and wR_2 was 0.0991 (all data).

In the crystal structure of **13**, the asymmetric unit contains half a magnesium complex, and three regions of regions of disordered benzene solvent. The magnesium complex straddles a crystallographic mirror plane, upon which Mg1, C12, H12, and Br1a/Br1b reside. The bromide ligand was found to be disordered and was split across two sites (parts 1 and 2) with relative occupancy 0.59(3):0.41(3). The benzene moiety C31-C36 also resides on the crystallographic mirror plane, and displayed substantial translational disorder, which was modelled as three components (parts 1, 2, and 3) with relative occupancy 0.5:0.3:0.2. The benzene moiety C25-C30 was disordered across a symmetry element, such that half of the molecule is present per asymmetric unit, this was modelled in two components (parts -1 and -2) of relative occupancy 0.35:0.15. The third region of disordered solvent was highly disordered over a symmetry element, and in preference to a heavily restrained model, a solvent mask was calculated and 172 electrons were found in a volume of 744 Å^3 in 1 void per unit cell. This is consistent with the presence of half a benzene molecule per asymmetric unit, which accounts for 168 electrons per unit cell.

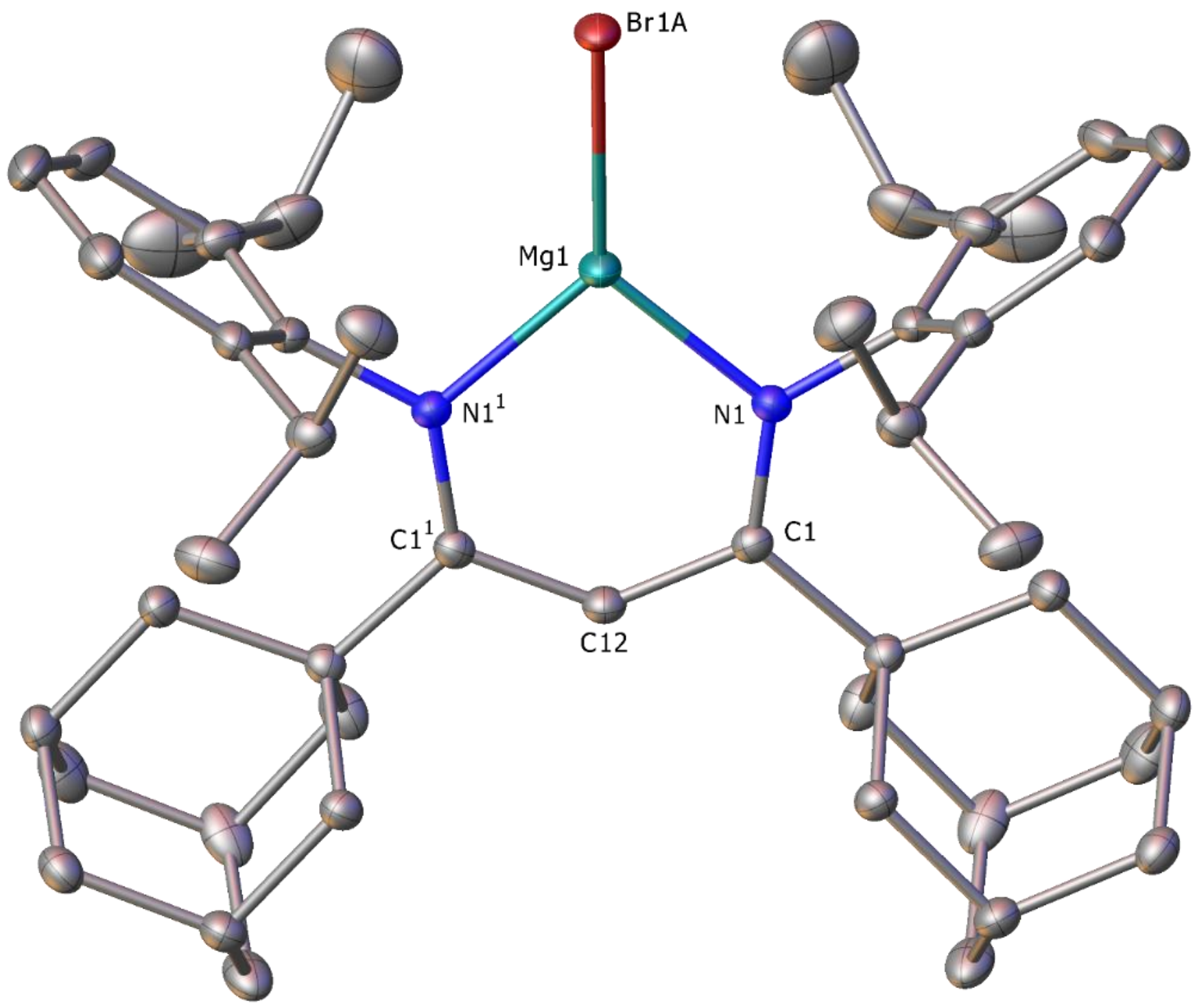

Figure S286: Displacement ellipsoid plot (30%) of the crystal structure of **13**. Only the major component of the disordered bromide is shown. Hydrogen atoms are omitted for clarity. [1]+X,1/2-Y,+Z.

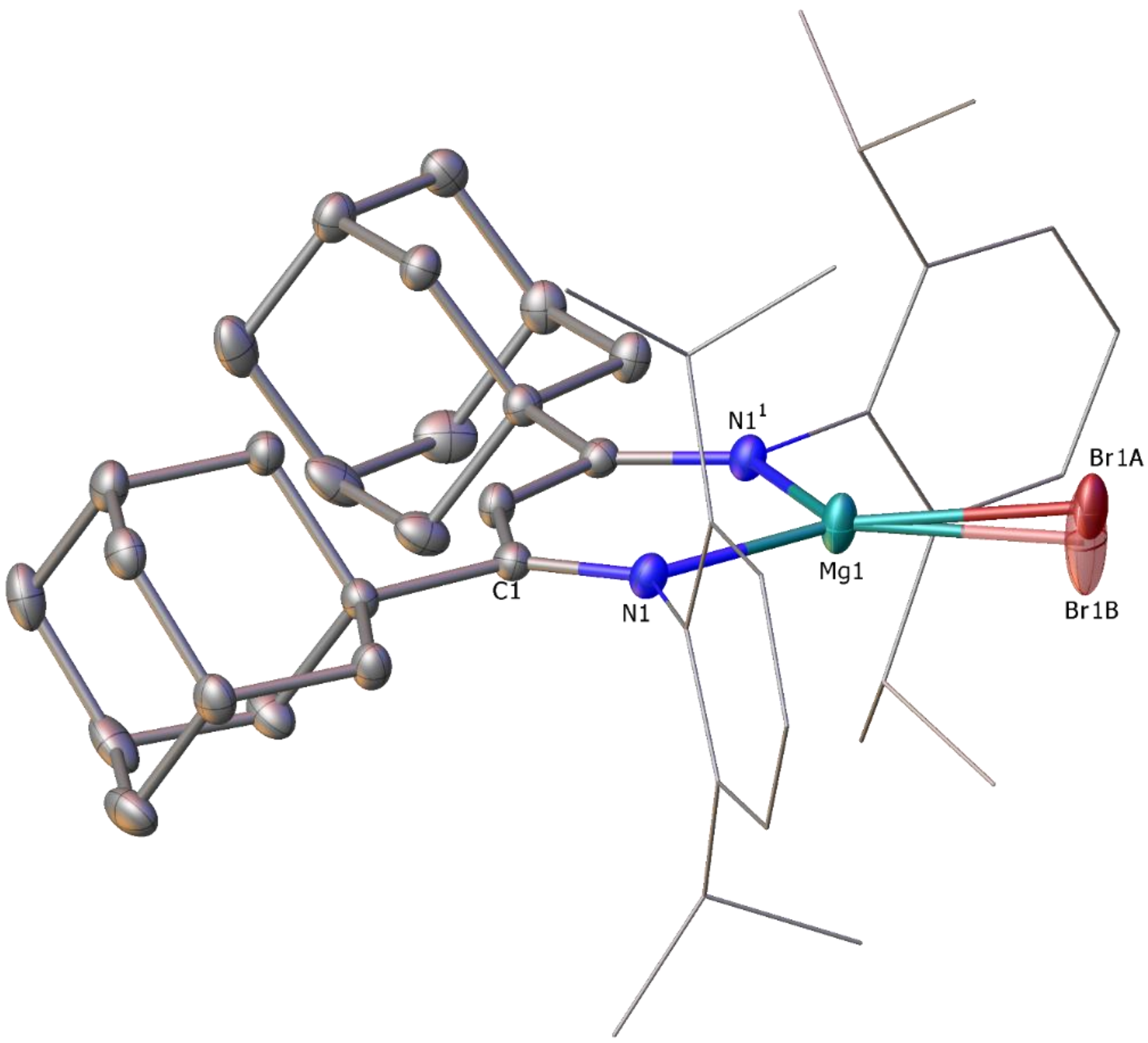

Figure S287: Displacement ellipsoid plot (30%) of the crystal structure of **13** showing the minor component of the disordered bromide semi-transparent. Hydrogen atoms are omitted and Dipp groups shown as wireframes for clarity. [1]+X,1/2-Y,+Z.

6 Computational details

6.1 Computational details

DFT calculations were performed with Gaussian 16 (C.01).[15] Initial BP86[16] optimisations were performed using the 'grid = ultrafine' option with BS1 for all stationary points. BS1 uses the def2-SVP basis set for all atoms (H, C, N and Mg atoms).[17] Dispersion corrections were applied using Grimme's D3 parameters with Becke–Johnson damping ($D3^{BJ}$).[18] Corrections for the effect of benzene (ε = 2.2706) and toluene (ε = 2.3741) solvents were introduced using SMD continuum universal solvation model.[19] All energies were corrected by single-point calculations with a larger basis set (BS2) involving def2TZVPP for all atoms.[17] Analytical frequency calculations were performed to characterise the stationary points, either as minima (all eigenvalues positive) or transition states (one negative eigenvalue). The connectivity of all located transition states to their corresponding minima was verified by intrinsic reaction coordinate (IRC) calculations. In each case, the transition-state geometry was displaced in both directions along the transition vector, followed by full geometry optimisation until the adjacent minima were reached. A modified version of the PyMOL ball-and-stick style provided by the Paton group was used to generate molecular structure images.[20] In the figures, the N-Dipp substituents and the backbone substituents of the BDI ligand (R = methyl and 1-adamantyl) are shown in wireframe representation. Atom colours are assigned as follows: carbon (grey), magnesium (green), and nitrogen (blue).

6.2 Breakdown of Energy Contribution

$\Delta\mathbf{E}_{\mathbf{BS1}}$	SCF energy computed with the BP86 functional and BS1
$\Delta\mathbf{G}_{\mathbf{BS1}}$	Free energy at 298.15 K and 1 atm with the BP86 functional and BS1
$\Delta\mathbf{G}_{\mathbf{BS1/C_6H_6}}$	Free energy corrected for Benzene solvent (SMD) with the BP86 functional and BS1
$\Delta\mathbf{G}_{\mathbf{BS1/C_7H_8}}$	Free energy corrected for Toluene solvent (SMD) with the BP86 functional and BS1
$\Delta\mathbf{G}_{\mathbf{BS1/D3^{BJ}/C_6H_6}}$	Free energy corrected for Benzene solvent (SMD) and dispersion ($D3^{BJ}$) with the BP86 functional and BS1
$\Delta\mathbf{G}_{\mathbf{BS1/D3^{BJ}/C_7H_8}}$	Free energy corrected for Toluene solvent (SMD) and dispersion ($D3^{BJ}$) with the BP86 functional and BS1
$\Delta\mathbf{E}_{\mathbf{BS2}}$	SCF energy computed with the BP86 functional and BS2
$\Delta\mathbf{G}_{bnz}$	Overall Free energy computed at the BP86-$D3^{BJ}$; SMD=Benzene/BS2//BP86/ BS1 level
$\Delta\mathbf{G}_{\mathbf{tol}}$	Overall Free energy computed at the BP86-$D3^{BJ}$; SMD=Toluene/BS2//BP86/ BS1 level
$\Delta\mathbf{H}_{\mathbf{BS1}}$	Zero-point energy (ZPE) at 298.15 K and 1 atm with the BP86 functional and BS1

6.3 Optimised structures and transition states

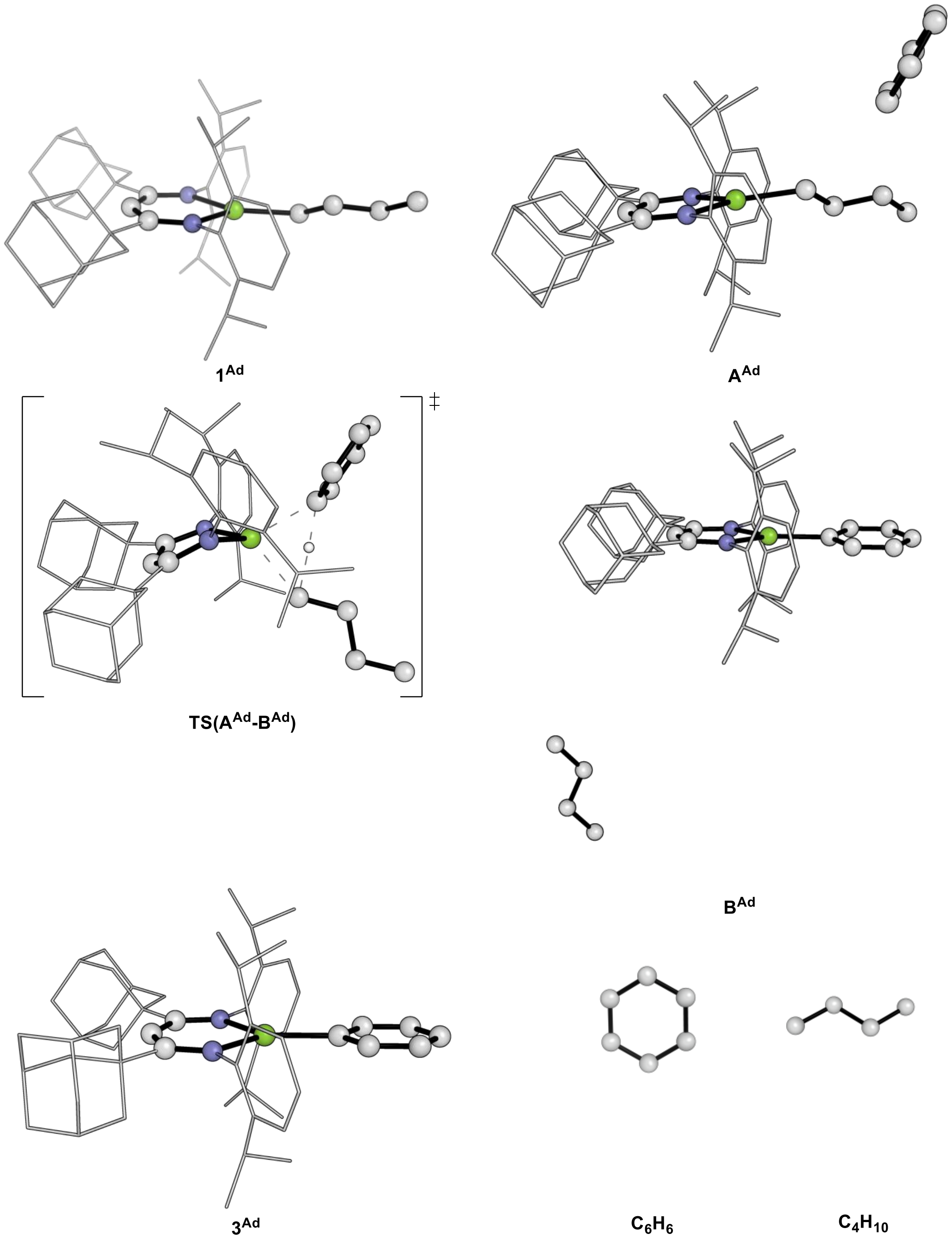

Figure S288. BP86/BS1 optimised geometries for the deprotonation of benzene by $\mathbf{1^{Ad}}$. For clarity, hydrogen atoms from BDI ligand are omitted.

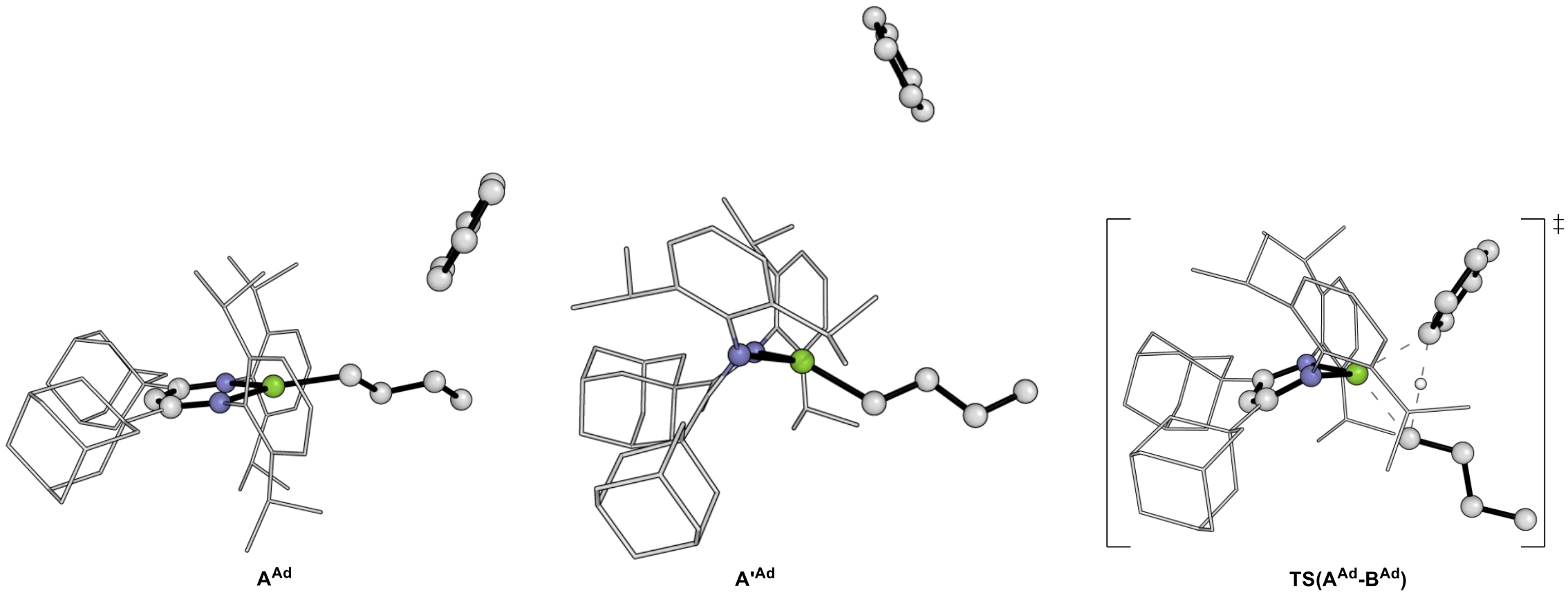

Figure S289. BP86/BS1 optimised geometries illustrating distortion of the ligand *via* structure **A'^{Ad}** during the deprotonation of benzene by **1^{Ad}**. Note that the structure of **A'^{Ad}** is not an optimised stationary point on the potential energy surface. For clarity, hydrogen atoms from BDI ligand are omitted.

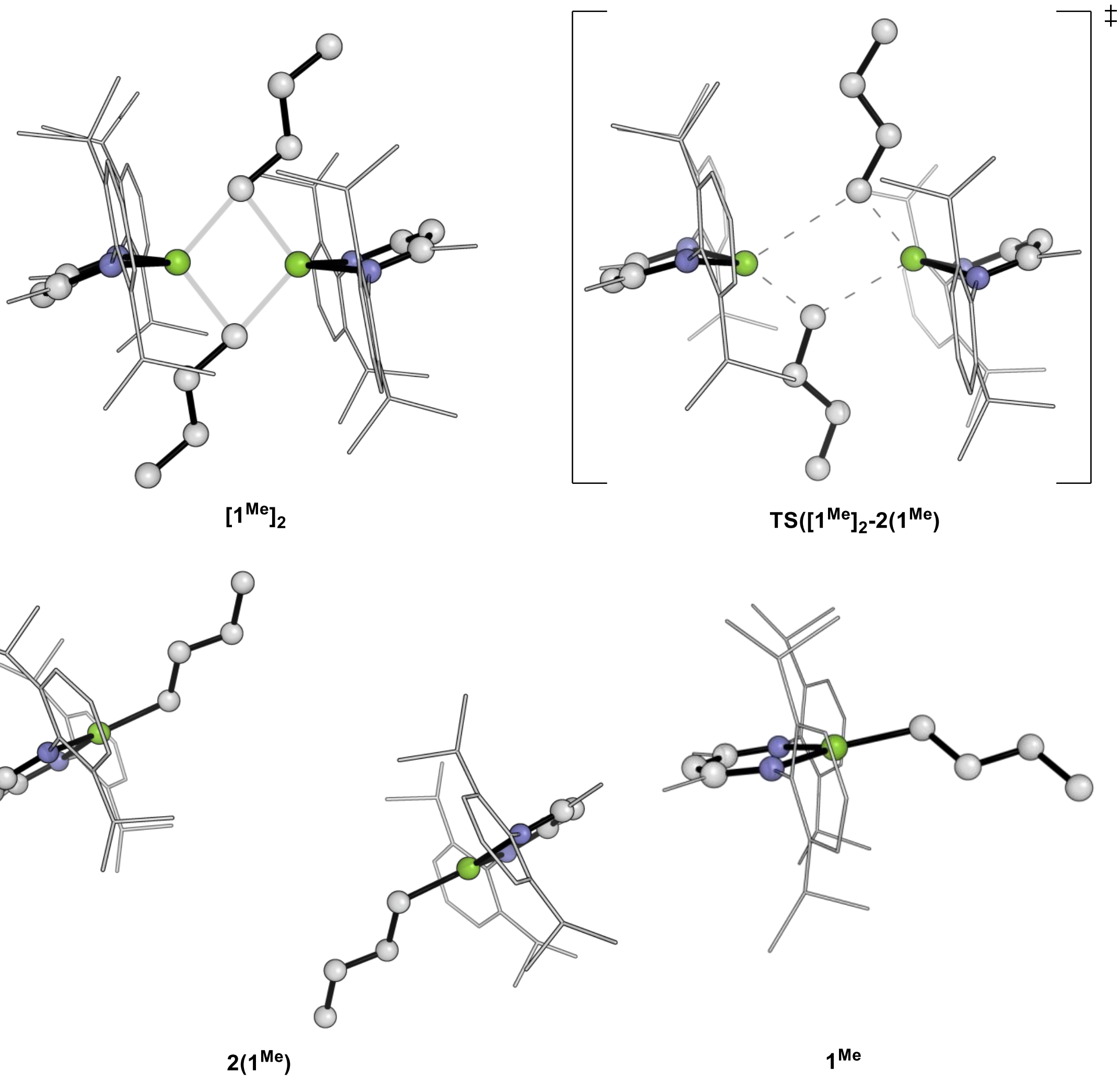

Figure S290. BP86/BS1 optimised geometries for the dissociation of dimeric **[1^{Me}]$_2$**. For clarity, hydrogen atoms from BDI ligand are omitted.

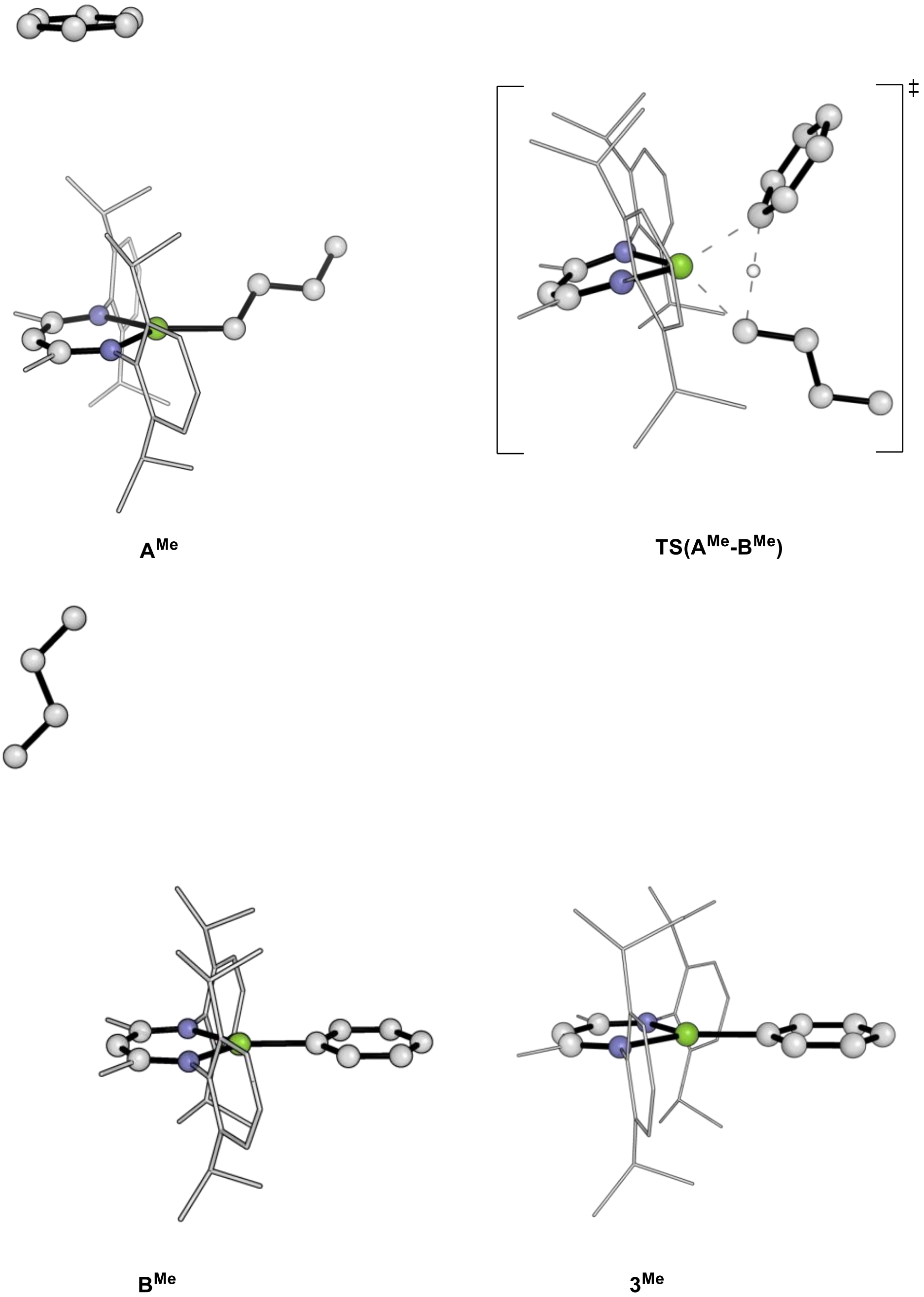

Figure S291. BP86/BS1 optimised geometries for the deprotonation of benzene by $\mathbf{1^{Me}}$. For clarity, hydrogen atoms from BDI ligand are omitted.

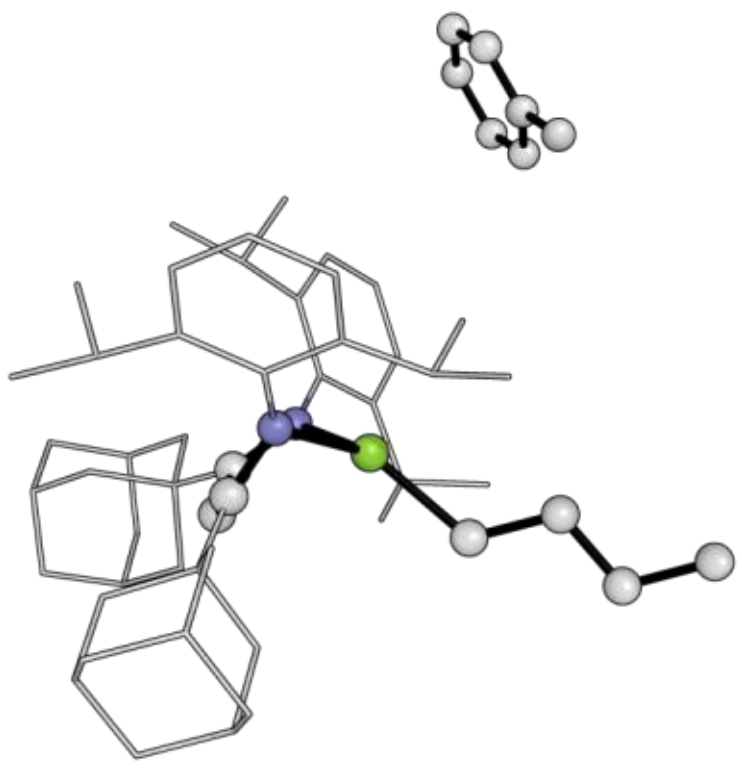

C^{Ad}

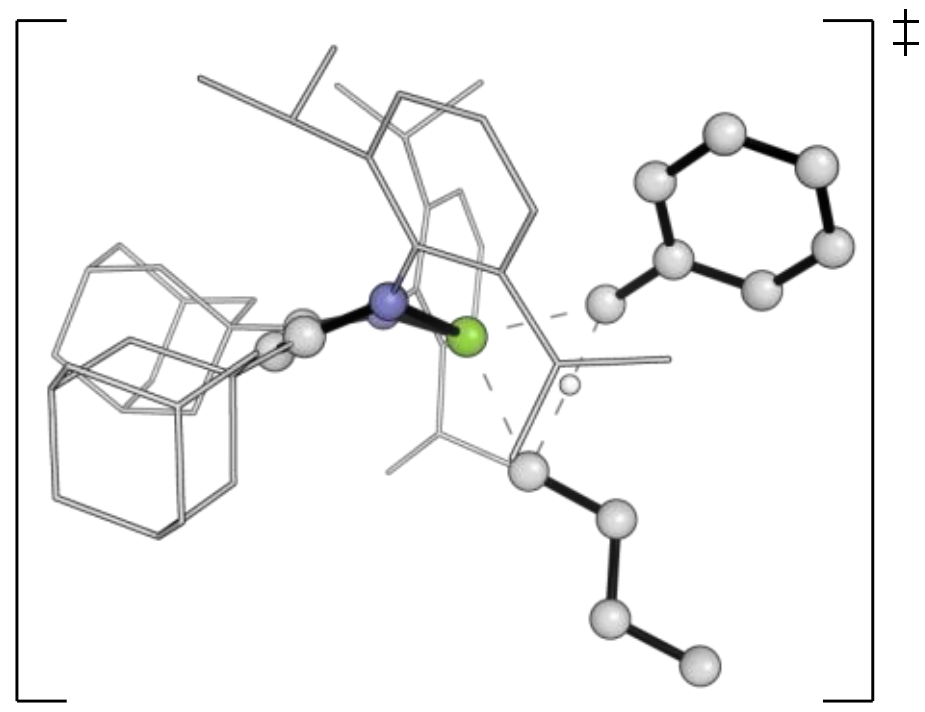

TS(C^{Ad}-D^{Ad})

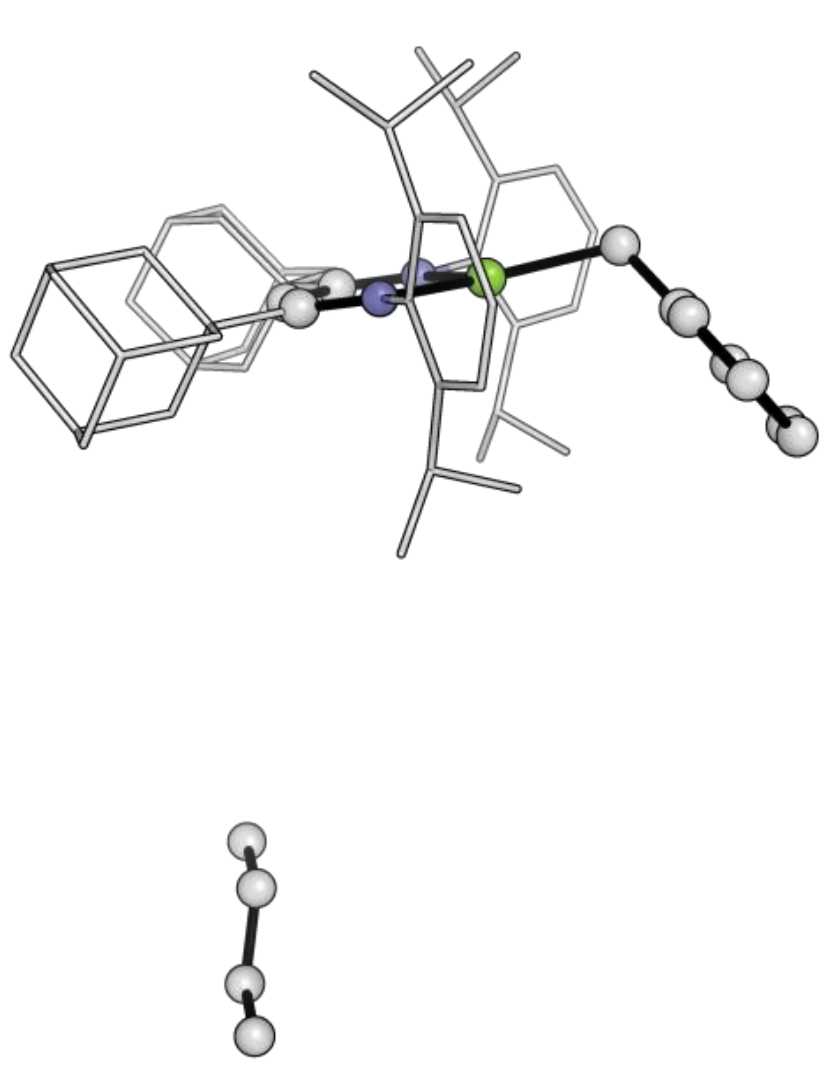

D^{Ad}

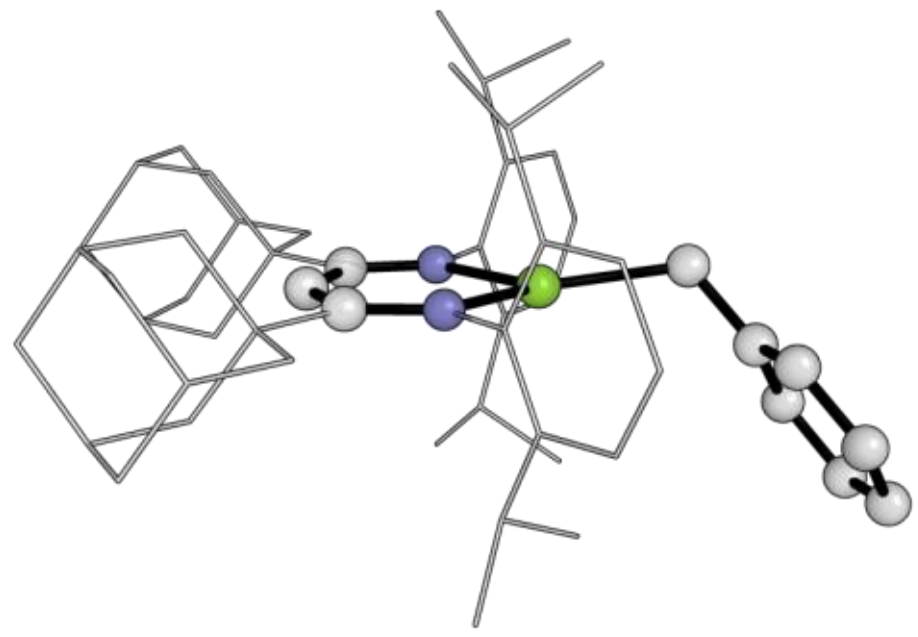

5'

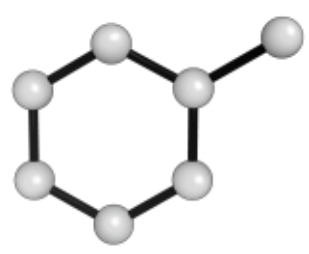

C_7H_8

Figure S292. BP86/BS1 optimised geometries for the deprotonation of toluene at the CH_3 position by **1^{Ad}**. For clarity, hydrogen atoms from BDI ligand are omitted.

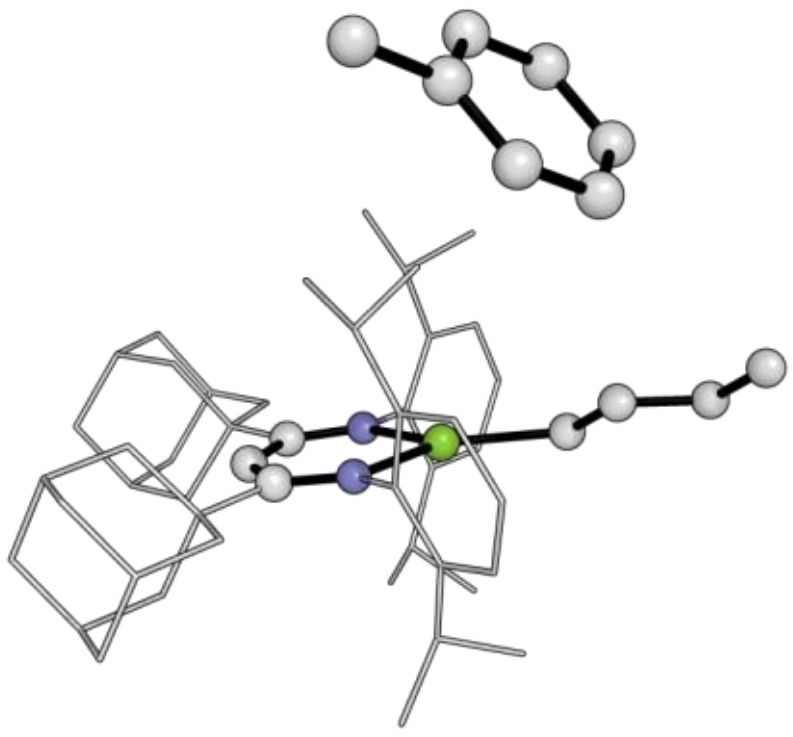

C

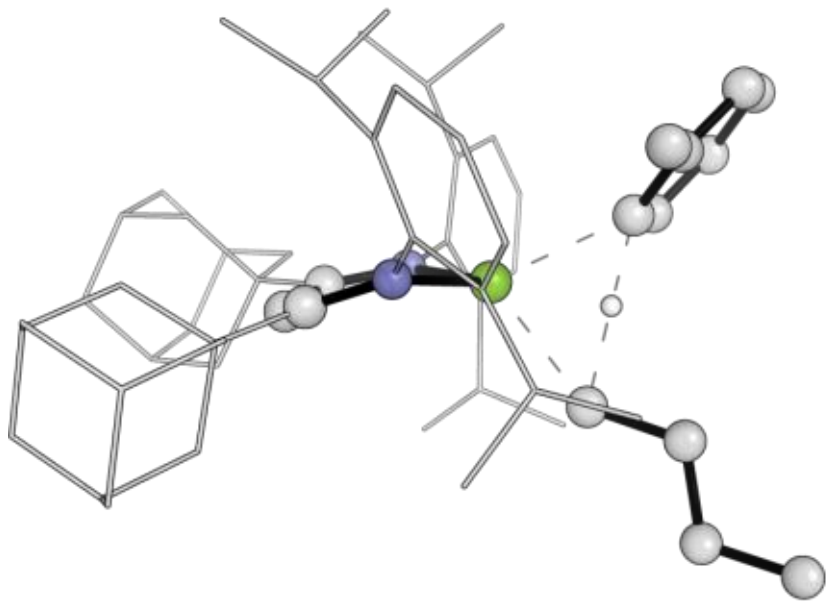

TS(C-D^{o})

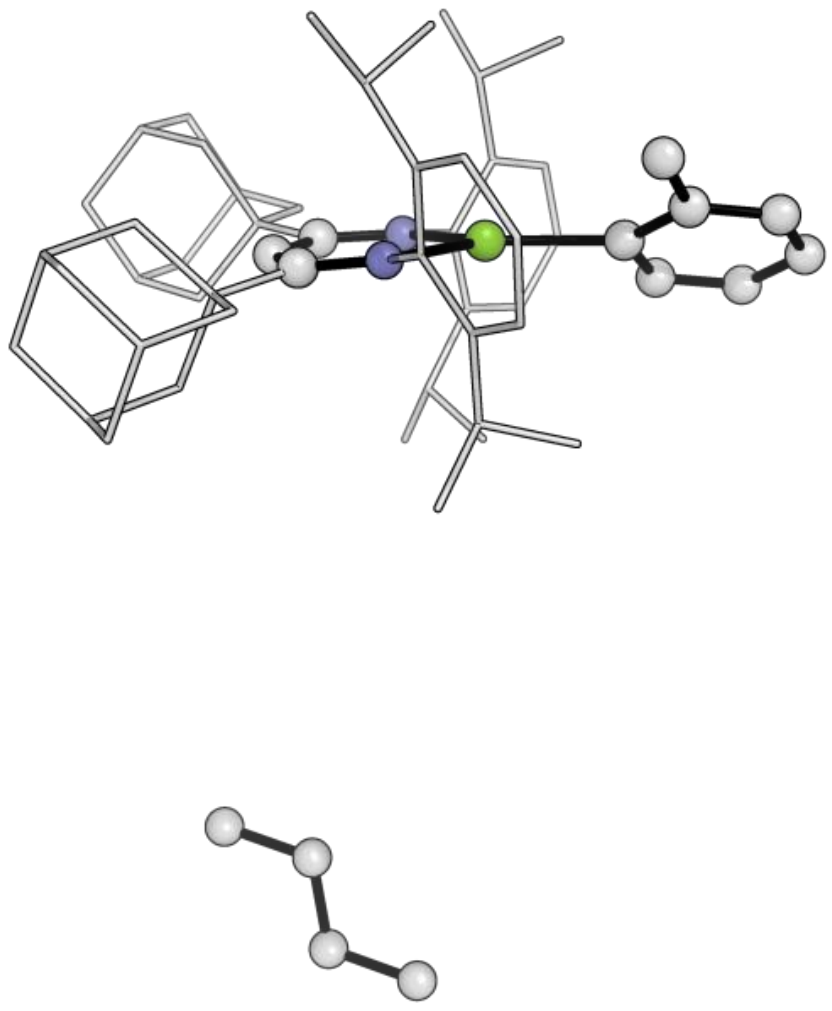

D^{o}

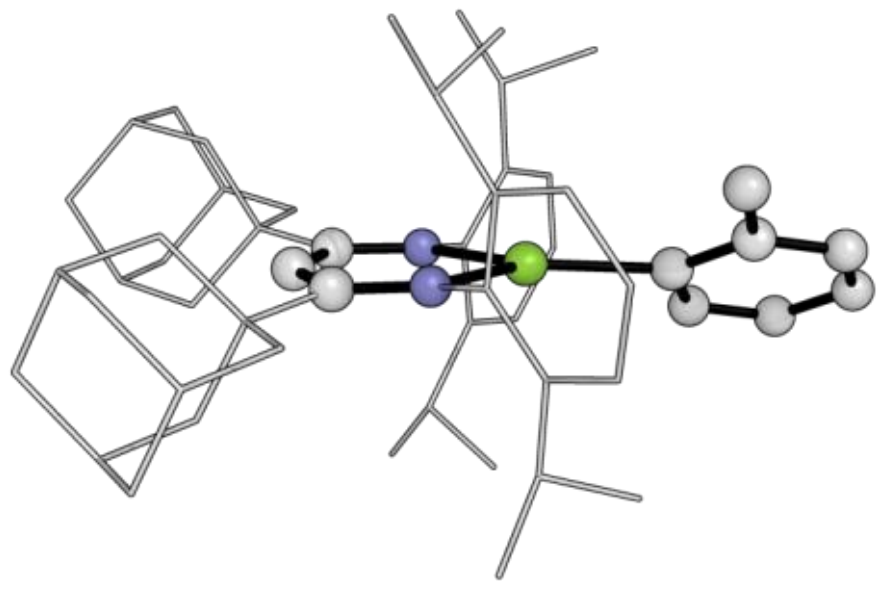

5-*o*

Figure S293. BP86/BS1 optimised geometries for the deprotonation of toluene at the *ortho*-position by **1**^{Ad}. For clarity, hydrogen atoms from BDI ligand are omitted.

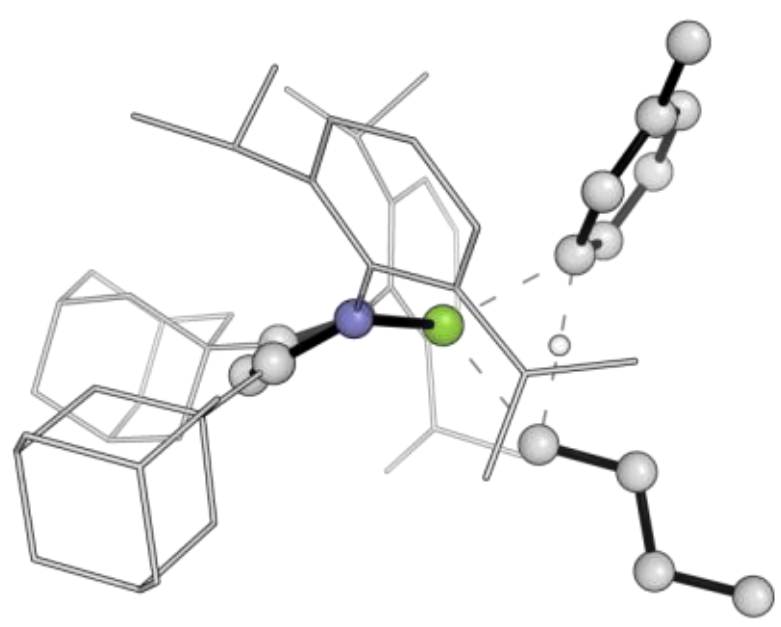

TS(C-D^{m})

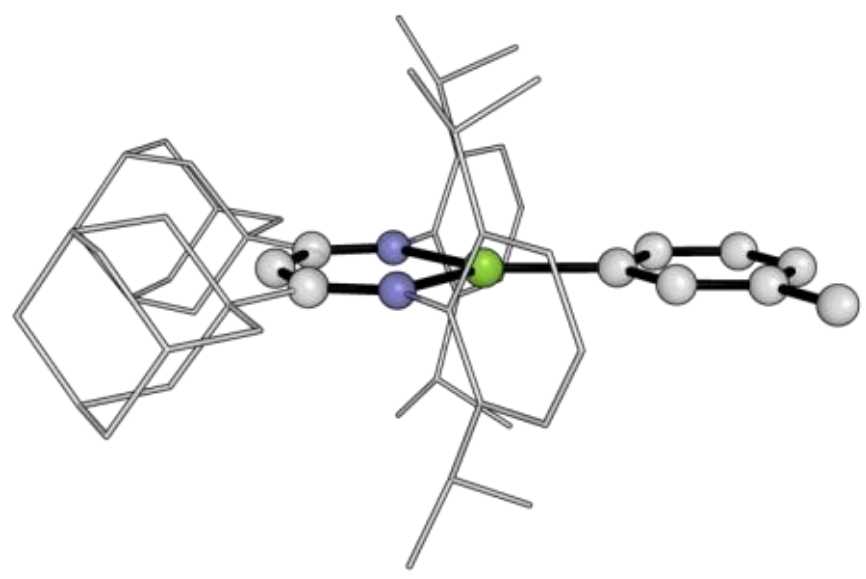

5-*m*

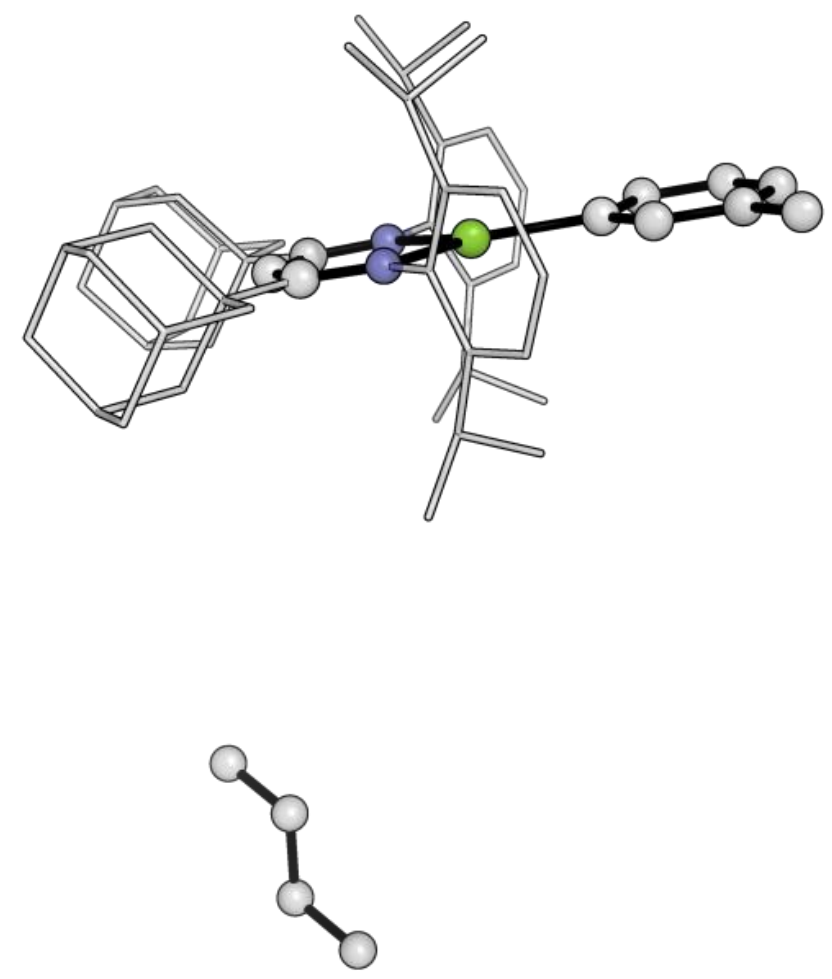

D^{m}

Figure S294. BP86/BS1 optimised geometries for the deprotonation of toluene at the *meta*-position by **1^{Ad}**. For clarity, hydrogen atoms from BDI ligand are omitted.

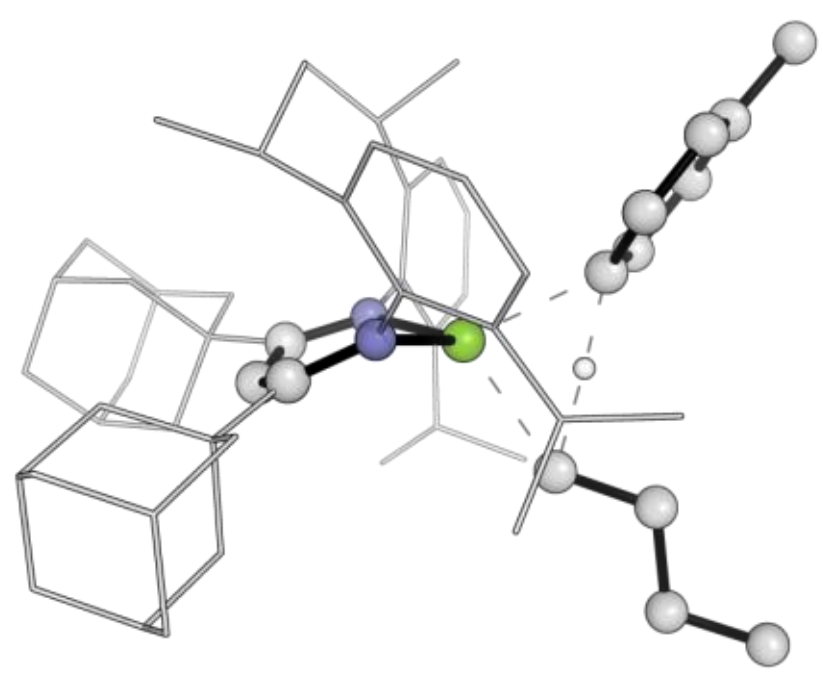

TS(C-D^{p})

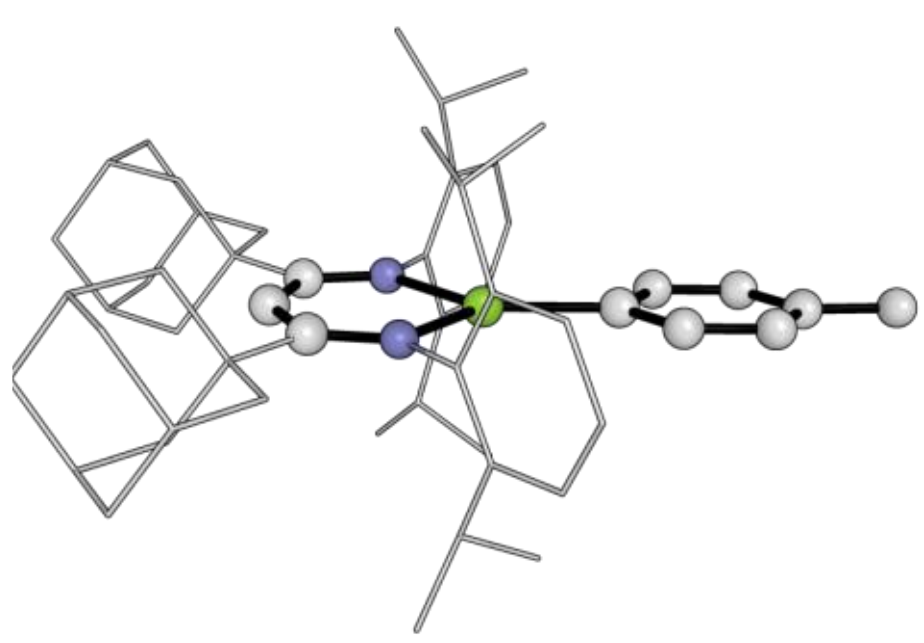

5-*p*

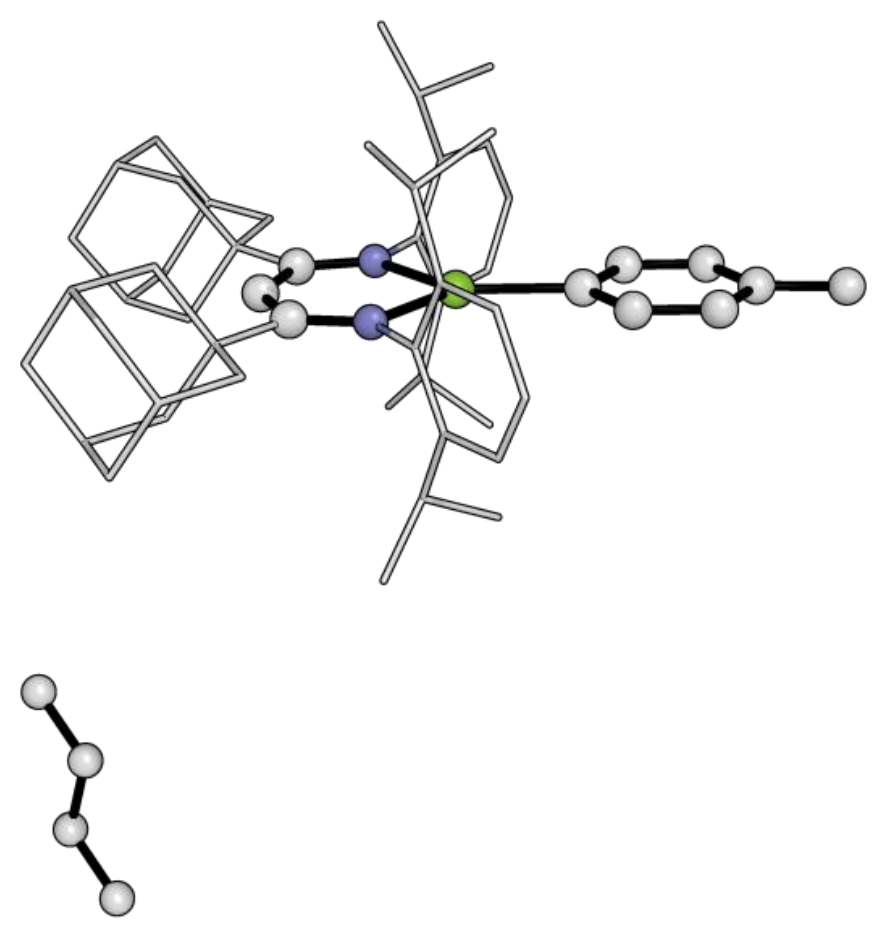

D^{p}

Figure S295. BP86/BS1 optimised geometries for the deprotonation of toluene at the *para*-position by **1^{Ad}**. For clarity, hydrogen atoms from BDI ligand are omitted.

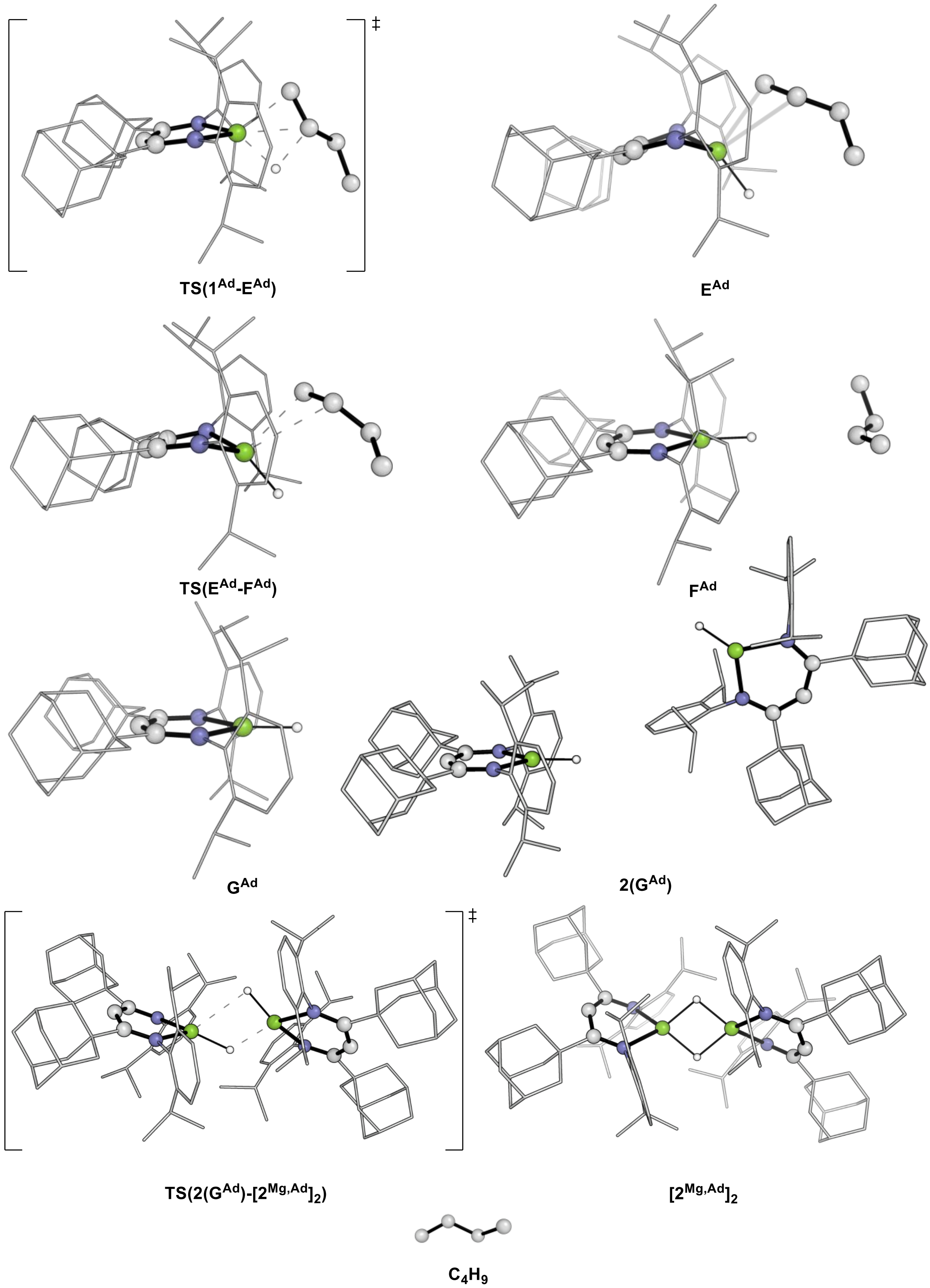

Figure S296. BP86/BS1 optimised geometries for the β-hydride elimination of 1-butene from **1^{Ad}**. For clarity, hydrogen atoms from BDI ligand are omitted.

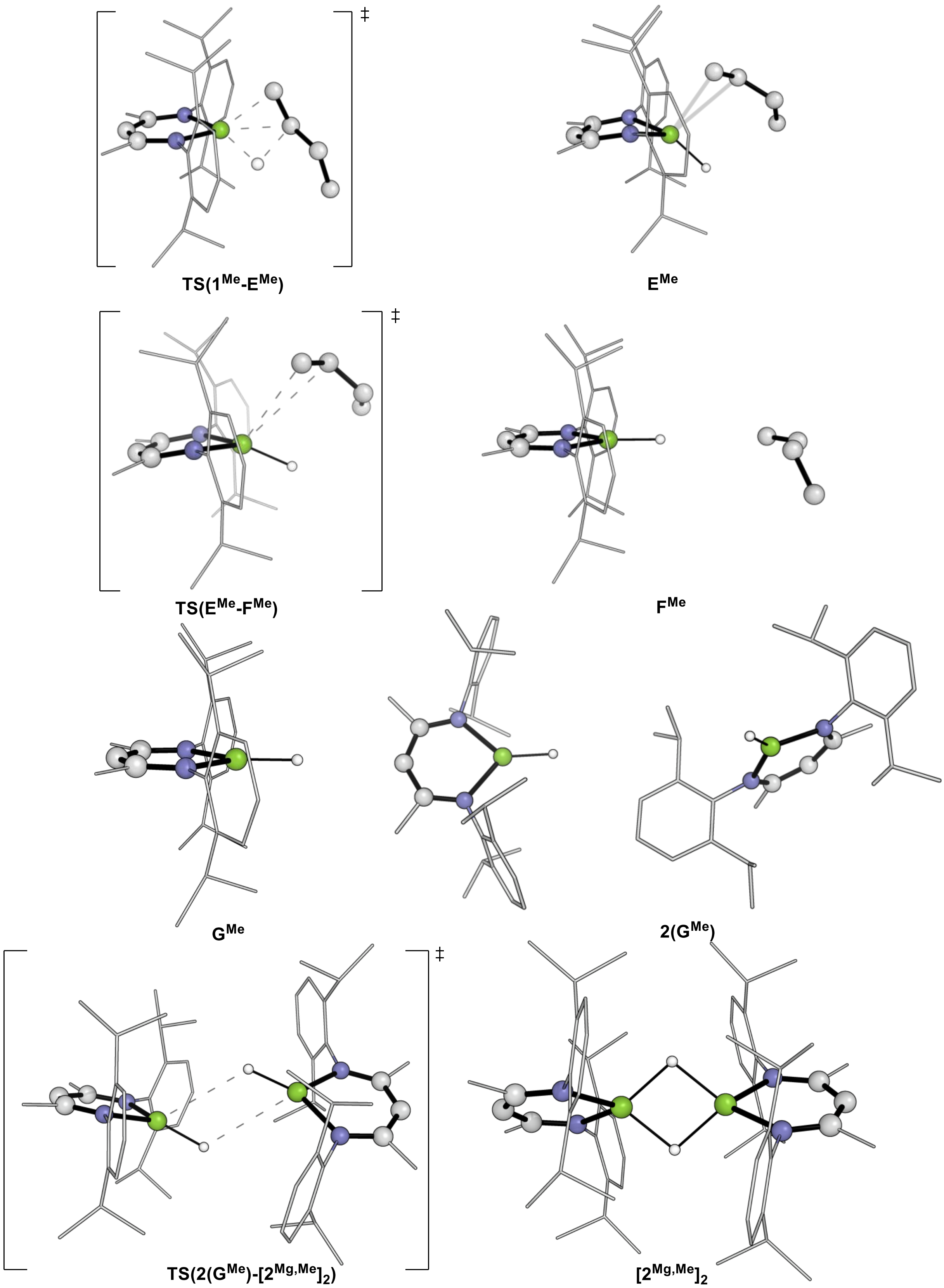

Figure S297. BP86/BS1 optimised geometries for the β-hydride elimination of 1-butene from **1**^{Me}. For clarity, hydrogen atoms from BDI ligand are omitted.

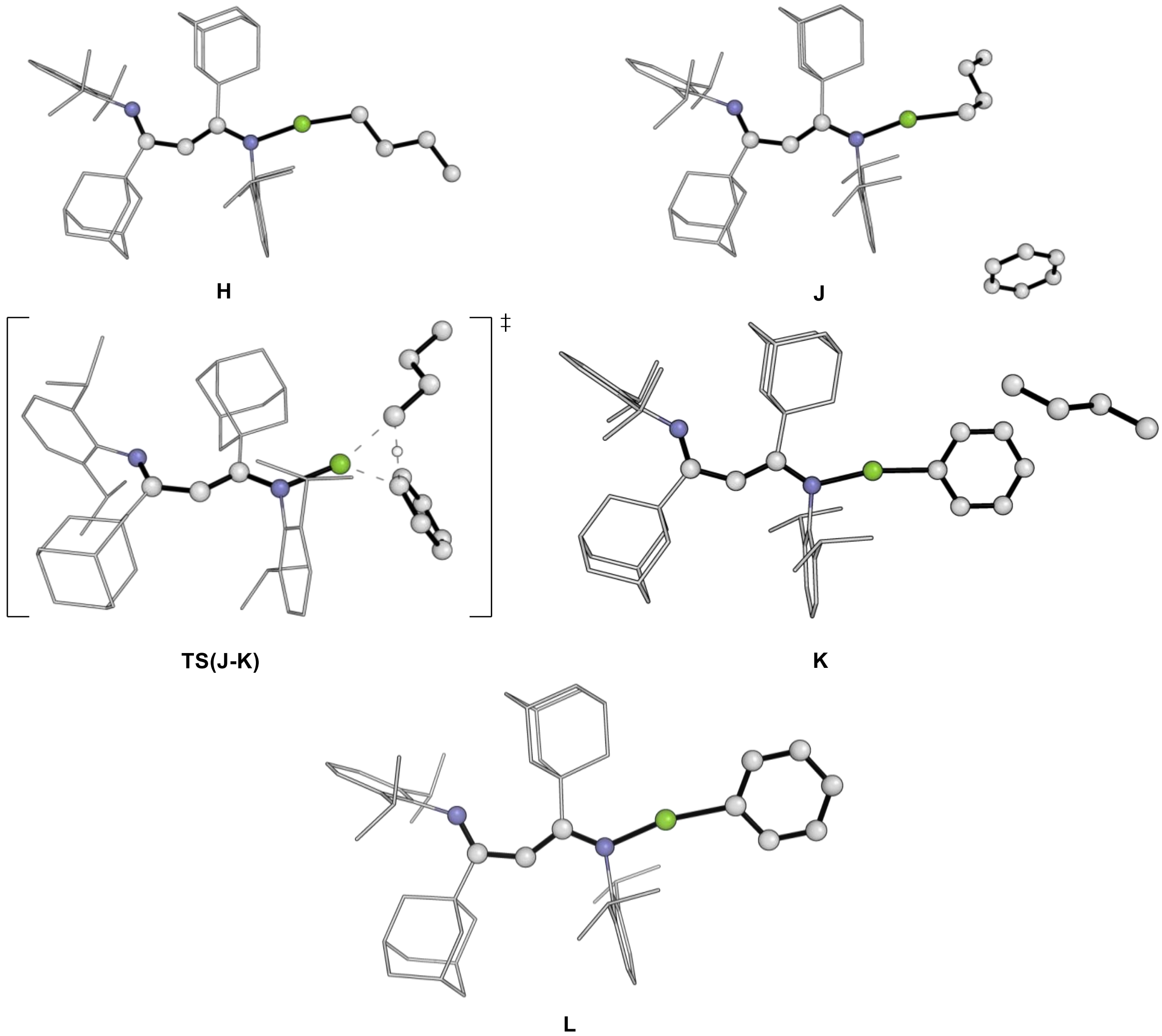

Figure S298. BP86/BS1 optimised geometries for the deprotonation of benzene by a hypothetical open-chain isomer of **1**^{Ad}. For clarity, hydrogen atoms from BDI ligand are omitted.

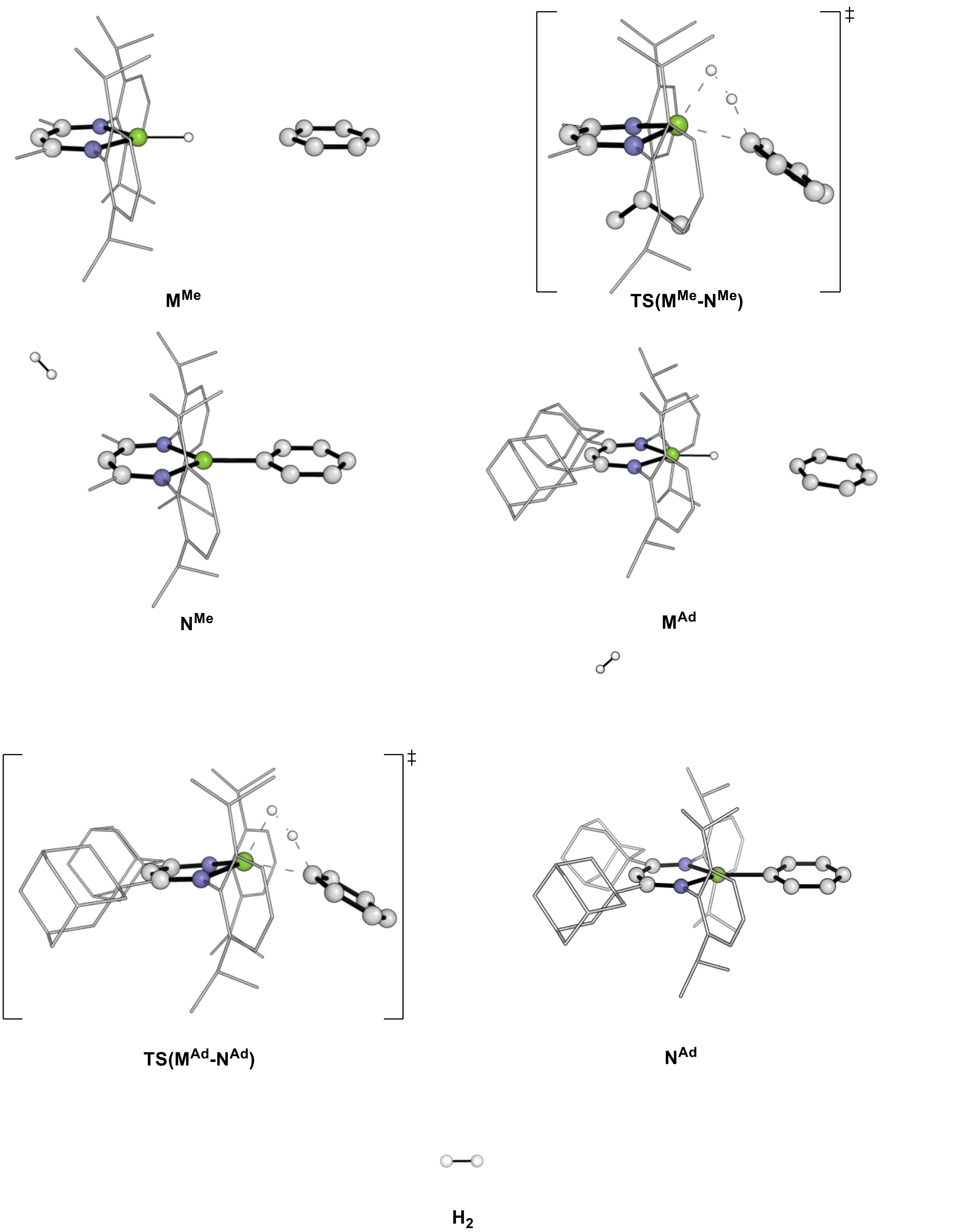

Figure S299. BP86/BS1 optimised geometries for the deprotonation of benzene by hypothetical monomeric hydride complexes $\mathbf{2}^{Mg,R}$. For clarity, hydrogen atoms from BDI ligand are omitted.

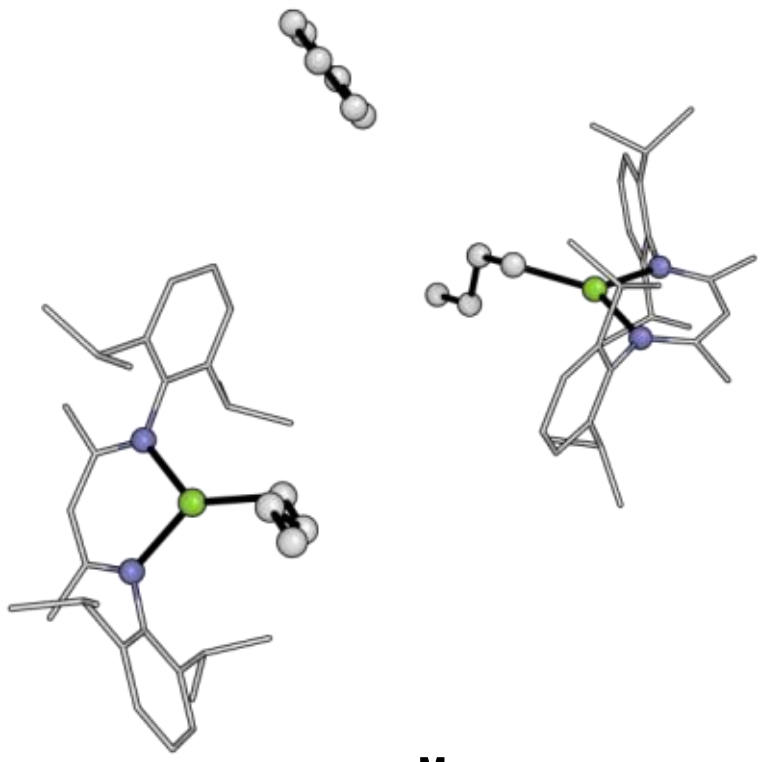

O^{Me}

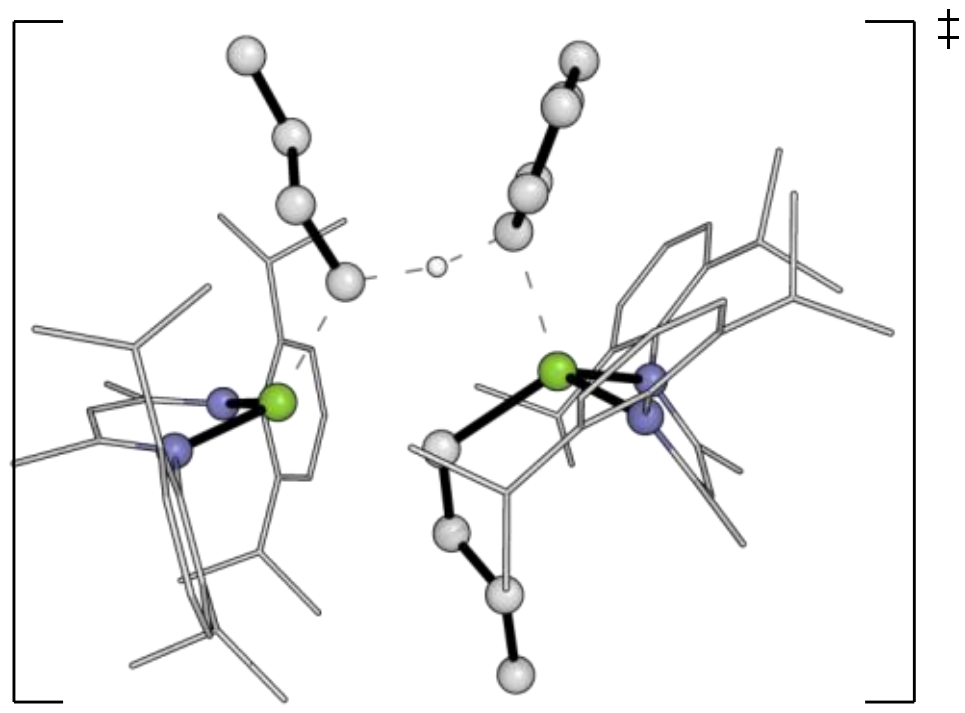

TS(O^{Me}-P^{Me})

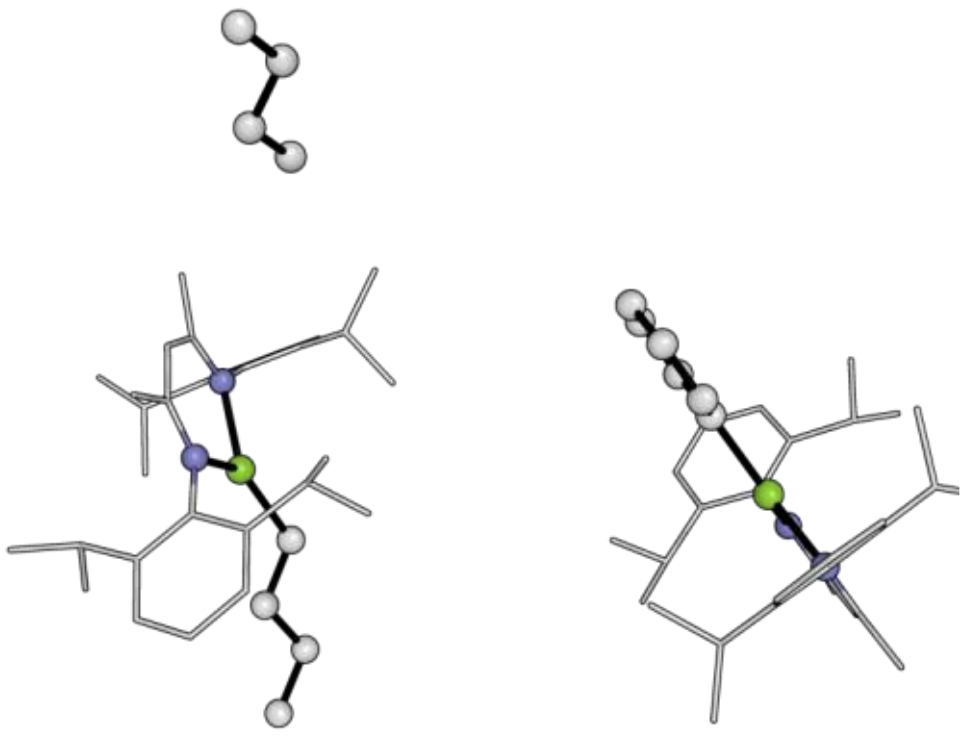

P^{Me}

Figure S300: BP86/BS1 optimised geometries for the deprotonation of benzene by 2×**1**^{Me}. For clarity, hydrogen atoms from BDI ligand are omitted.

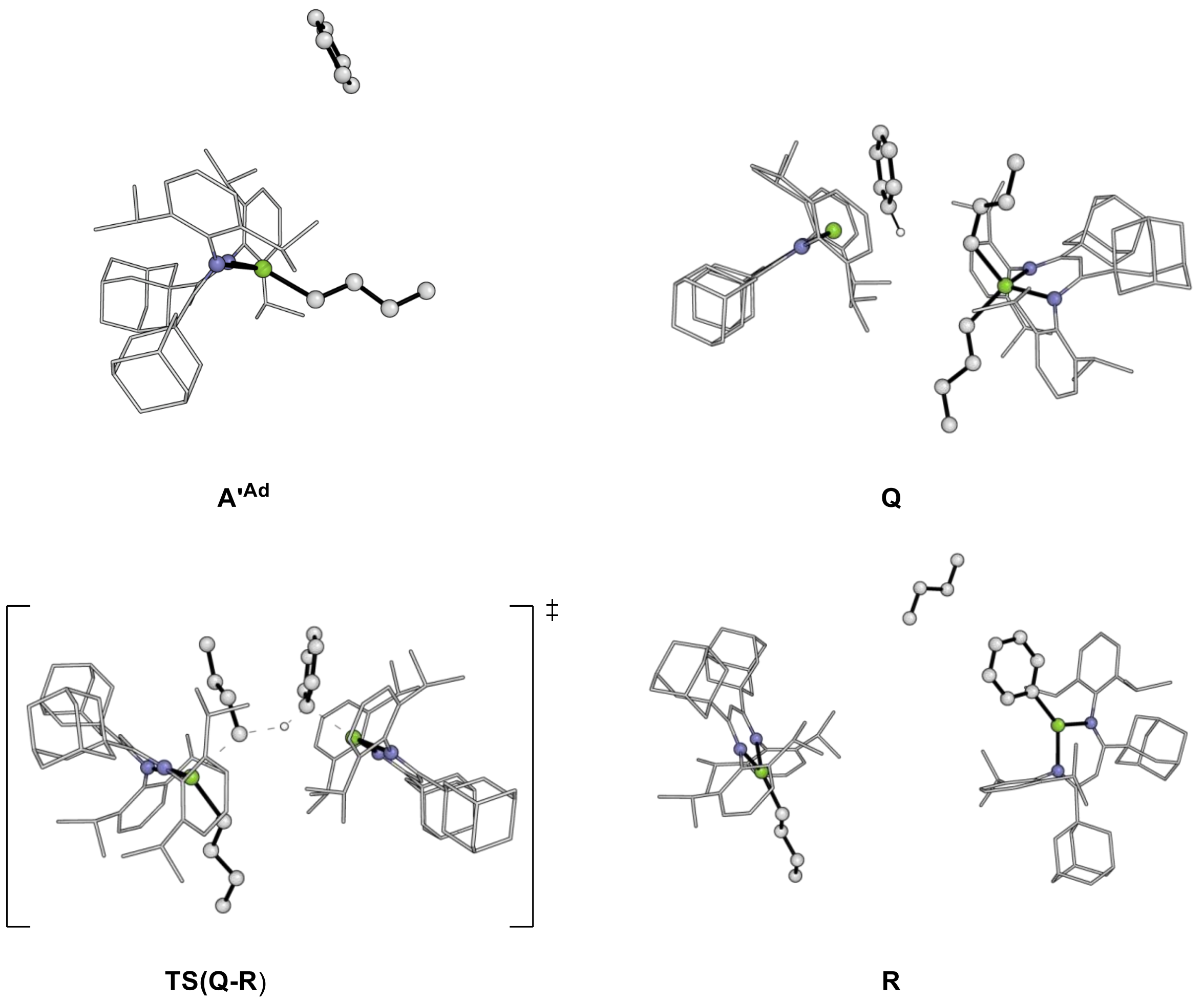

Figure S301: BP86/BS1 optimised geometries for the deprotonation of benzene by 2×**1^Ad**. For clarity, hydrogen atoms from BDI ligand are omitted.

6.4 Calculated potential energy surfaces

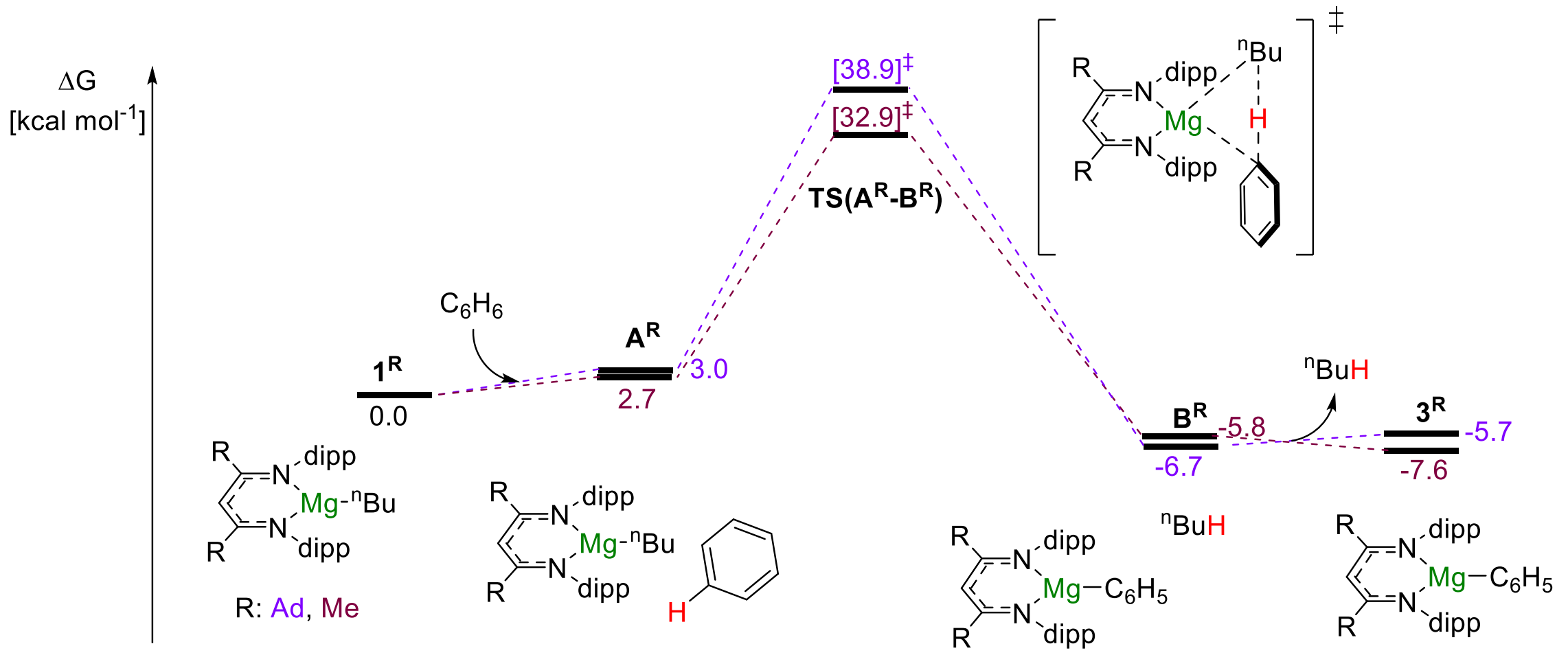

Figure S302. C(sp²)−H deprotonation of benzene from the monomeric ^RBDIMg−^nBu (R: Me (**1^Me**), Ad (**1^Ad**)) complex.

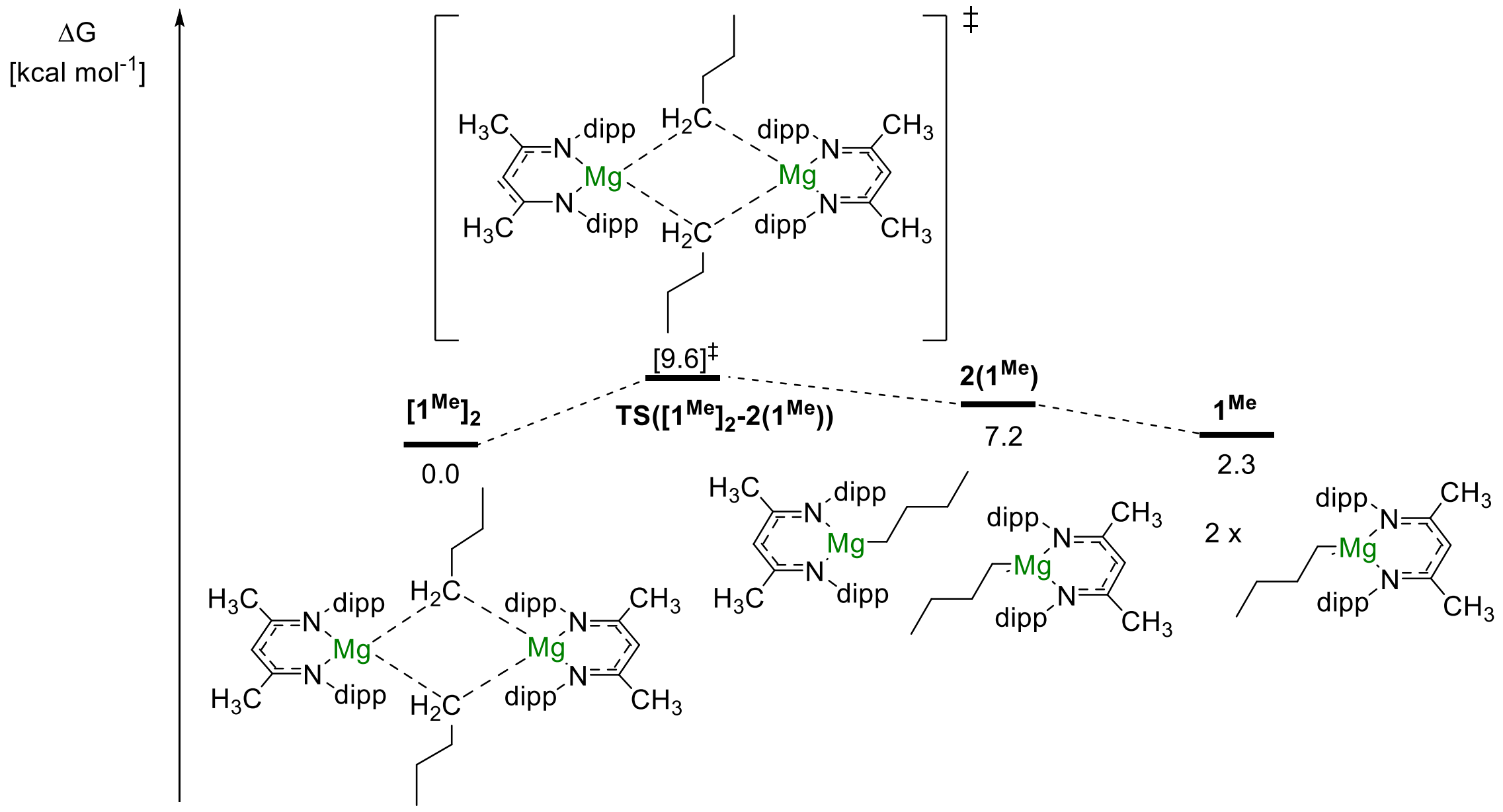

Figure S303. Dissociation of **$[1^{Me}]_2$** into 2 **1^{Me}**.

Table S14: Relative Gibbs free energy associated to the bending of the BDI ligand in A^{Ad} prior to deprotonation of benzene in kcal/mol.

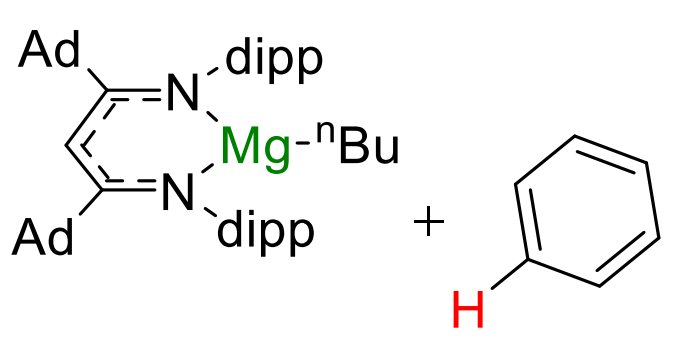	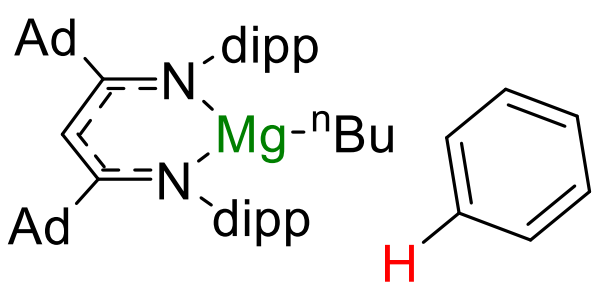	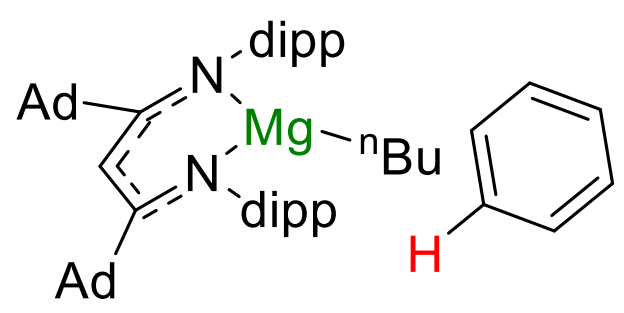	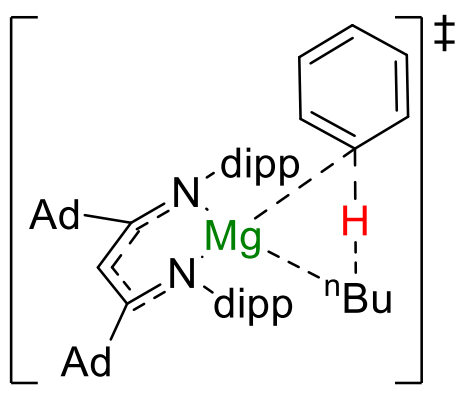
**1^{Ad}(+C_6H_6)**	**A^{Ad}**	**A'^{Ad}**	**TS(A^{Ad}-B^{Ad})**
0.0	3.0	20.1	38.9

Attempts were made to locate the bent BDI conformation of **A^{Ad}** as a stationary point on the potential energy surface prior to deprotonation. However, all unconstrained optimisations consistently relaxed to the planar form (**A^{Ad}**). To examine the bent geometry, a structure was extracted from the IRC pathway and subjected to a single-point frequency calculation. Although all vibrational frequencies were positive, the geometry was not converged. The corresponding structure (**A'^{Ad}**) lay 20.1 kcal mol^{-1} higher in energy than the fully optimised planar analogue (**A^{Ad}**). This intermediate is therefore not a true stationary point and is reported only to illustrate the energetic cost associated with predisposing the BDI ligand toward bending prior to deprotonation of benzene.

Table S15: Relative Gibbs free energy associated to the deprotonation of benzene by **$[1^{Me}]_2$** in kcal/mol.

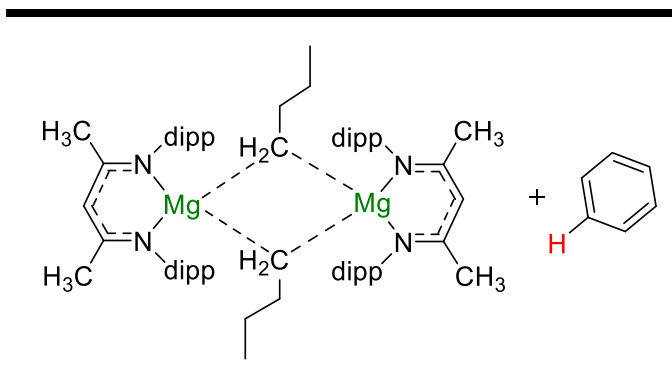	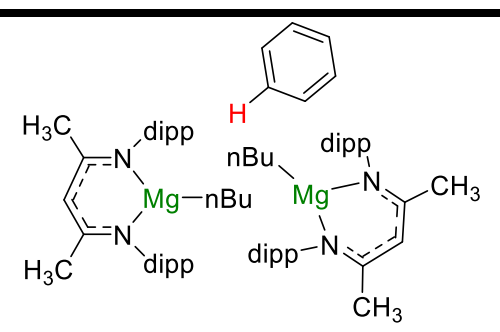	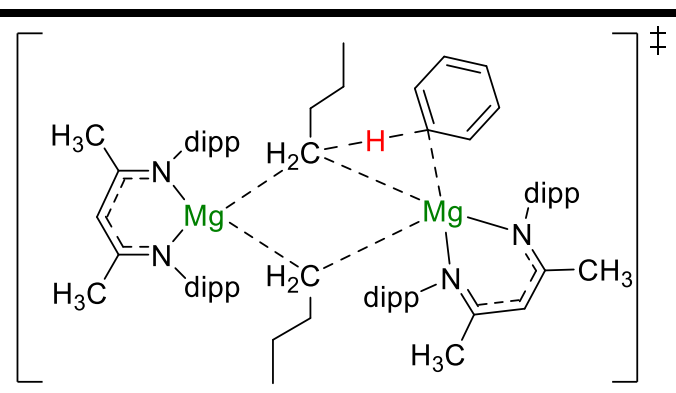	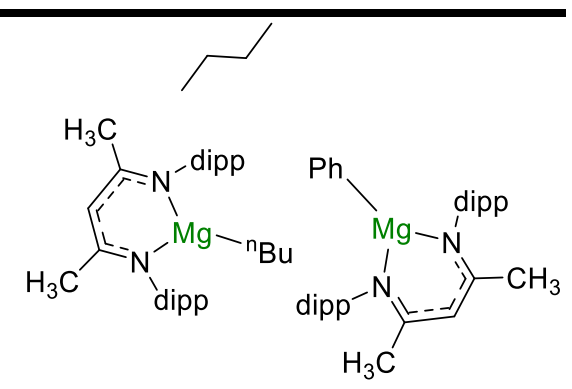
**$[1^{Me}]_2$(+C_6H_6)**	**O^{Me}**	**TS(O^{Me}-P^{Me})**	**P^{Me}**
0.0	11.7	49.2	3.7

The attempted C(sp^2)-H deprotonation of benzene by the dimeric **[1^{Me}]$_2$** yielded a highly elevated transition state (ΔG‡ = +49.2 kcal/mol), rendering this pathway overwhelmingly unfeasible compared with the monomeric mechanism reported in the manuscript. Importantly, IRC analysis starting from the distorted dimer-based TS (**TS(O^{Me}-P^{Me})**) does not return to the intact **[1^{Me}]$_2$** dimer; instead, the reaction coordinate relaxes directly to the monomeric species (**O^{Me}**). This behaviour demonstrates that dissociation of the dimer precedes any benzene deprotonation event, confirming that the dimeric route is not connected to the C-H activation surface. Taken together, the prohibitively high barrier and the IRC connectivity both rule out dimer involvement and reinforce that benzene activation must proceed exclusively via the monomeric species.

Table S16. Relative Gibbs free energy associated to the deprotonation of benzene by 2×**1^{Ad}** in kcal/mol.

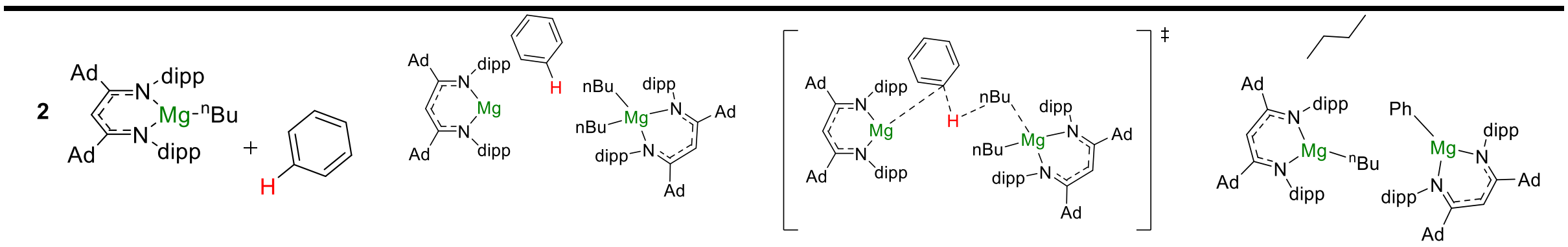

2×**1^{Ad}**(+C_6H_6)	**Q**	**TS(Q-R)**	**R**
0.0	47.6	52.0	1.2

Attempts to isolate the dimeric form of **1^{Ad}** were unsuccessful, most likely due to the severe steric congestion imposed by the Dipp substituents, which are further forced outward by the Ad groups on the BDI backbone. A transition state analogous to **TS(O^{Me}-P^{Me})** (TS(O^{Ad}-P^{Ad})), was also explored, but the restricted space inside the pocket prevents the ^{n}Bu chain and the benzene ring from occupying the same side of the dimer also precluding the isolation of the equivalent transition state. As a result, the putative dimer collapses into a species in which one Mg centre binds the arene while the other retains both ^{n}Bu groups, effectively yielding two monomeric fragments rather than a true dimer. Considering 2×**1^{Ad}**(+C_6H_6), the process is prohibitively endergonic, indicating that formation of this adduct is highly unlikely under any reasonable conditions.

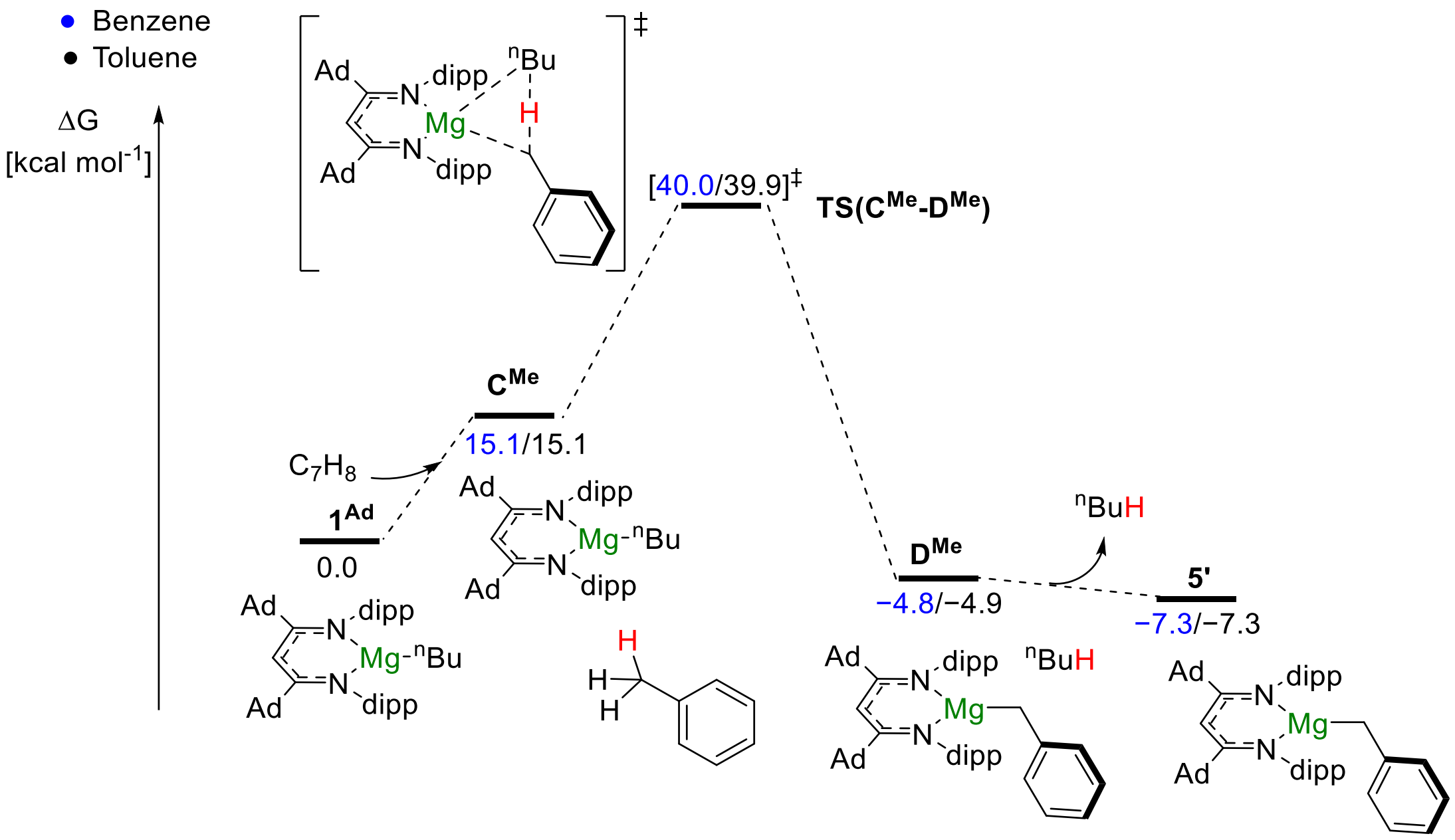

Figure S304. C(sp^3)−H deprotonation of toluene from the monomeric ^{Ad}BDIMg−^{n}Bu (**1^{Ad}**) complex. DFT-calculated Gibbs free energies (blue text) were computed in benzene or in toluene (black text).

Table S17. Relative Gibbs free energies (ΔGtol) of backward IRC structures for the toluene deprotonation steps TS(C−D) with respect to “Cp”, the lowest C isomer. (BP86− D3[BJ](SMD=toluene)/def2−TZVPP//BP86/def2−SVP level, quoted in kcal mol^{-1}).

C

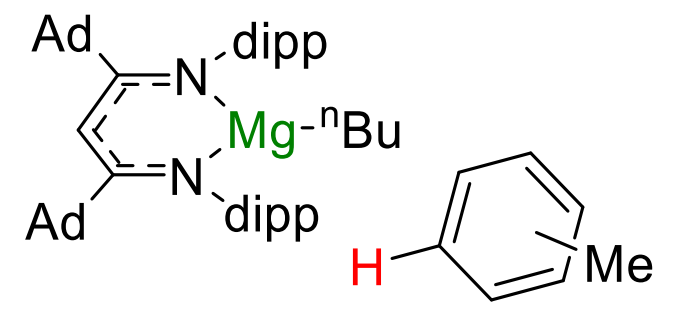

**C**	
**C^o**	+0.3
**C^m**	+0.5
**C^p**	0.0

Note: If benzene is used as solvent, it does not influence the stability of the obtained backward IRC, **$\Delta G_{tol} = \Delta G_{bnz}$.**

Table S18. DFT−calculated Gibbs free energies (BP86−D3[BJ](SMD = benzene or toluene)/def2−TZVPP//BP86/def2−SVP level, in kcal mol^{-1}) for the formation of **D** and ^{n}BuH through the C(sp^2)−H deprotonation of toluene by **C**. The values in blue correspond to Gibbs free energies calculated in benzene and in black, in toluene.

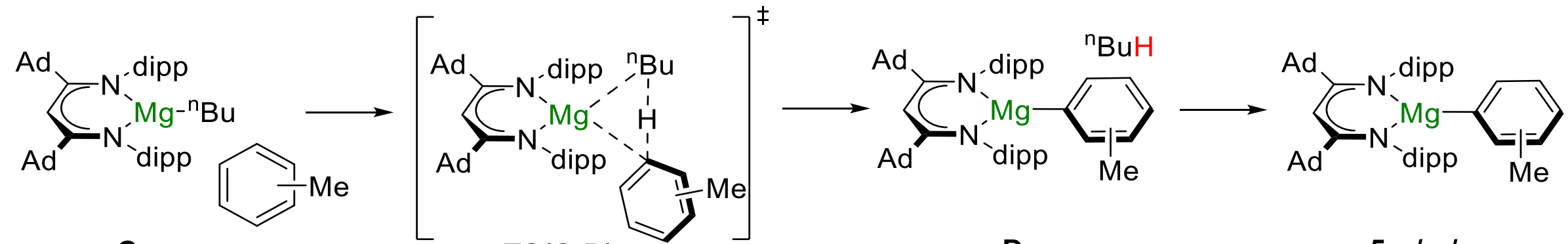

C **TS(C-D)** **D** **5-*o*/*m*/*p***

**TS(C−D^o)**	42.8/42.8	**D^o**	−1.7/−1.8	**5-*o***(+^{n}BuH)	−4.2/−4.2
**TS(C−D^m)**	40.0/40.0	**D^m**	−3.9/−3.9	**5-*m***(+^{n}BuH)	−6.6/−6.6
**TS(C−D^p)**	39.9/39.9	**D^p**	−4.0/−4.1	**5-*p***(+^{n}BuH)	−7.0/−7.0

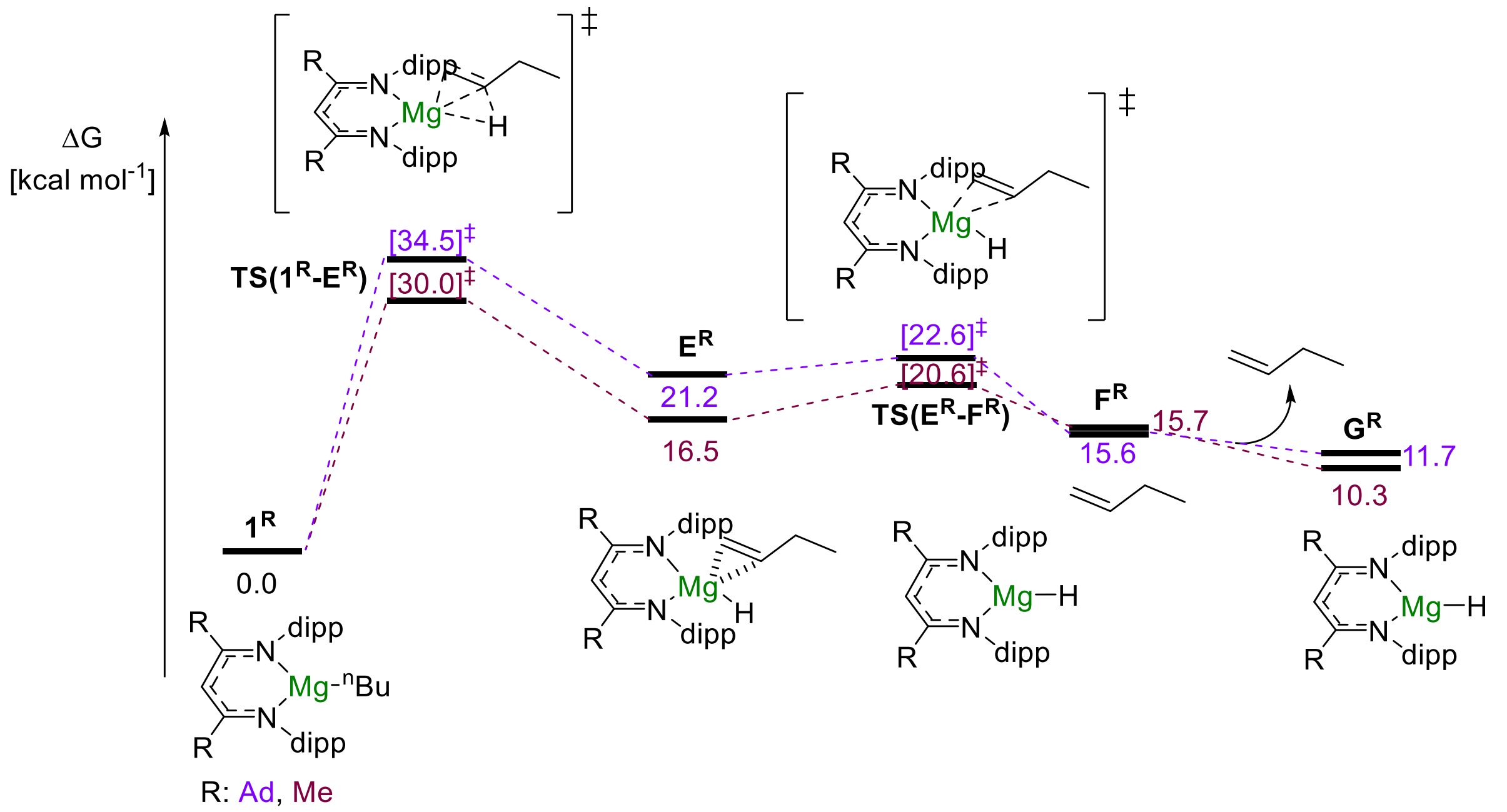

Figure S305. β−H elimination of n−butyl from the monomeric ^{R}BDIMg−^{n}Bu (**1^R**) complex to the monomeric form of hydride magnesium complex (**G^R**).

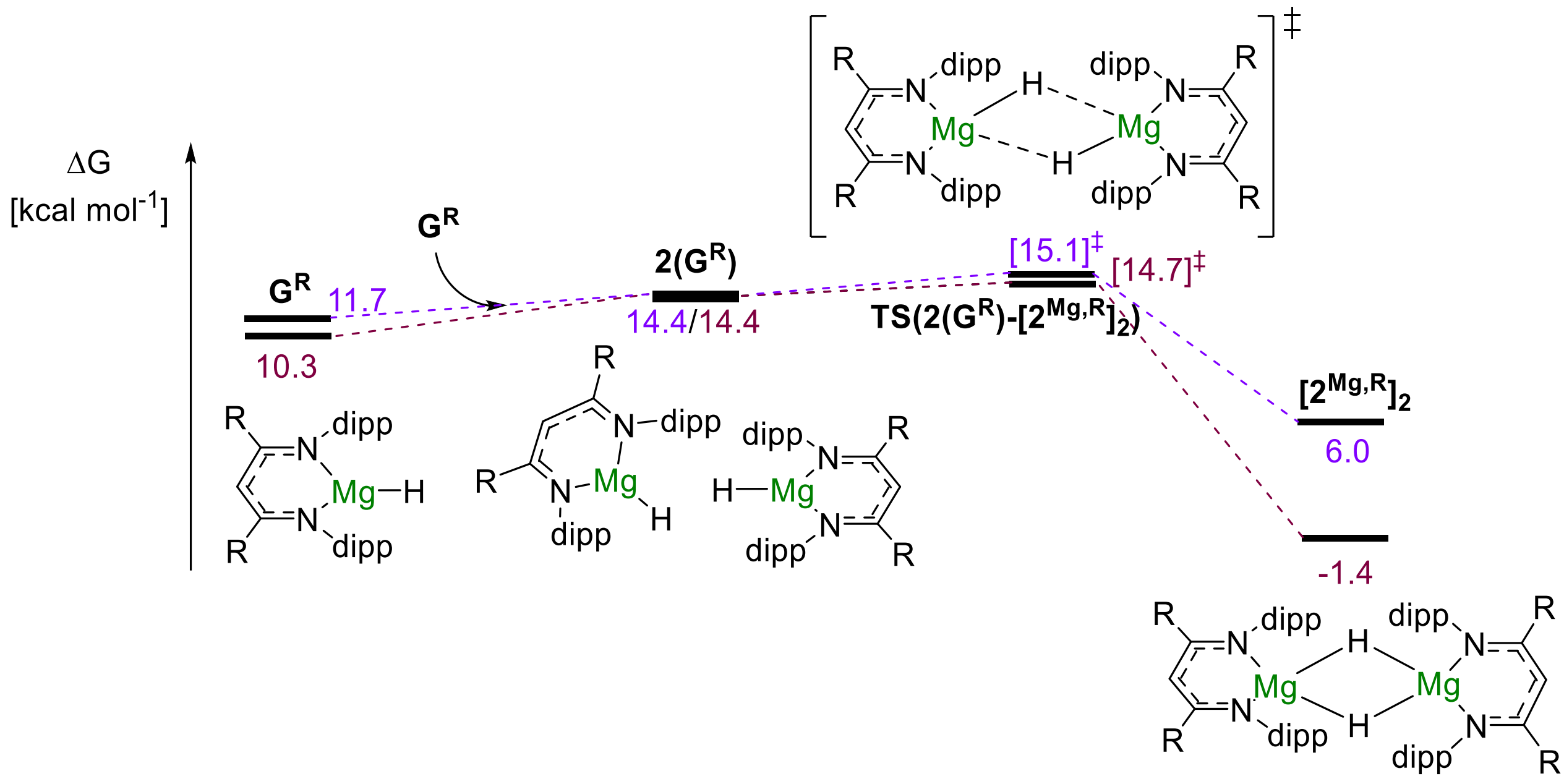

Figure S306. Dimersisation of monomeric form of hydride magnesium complex ($\mathbf{G^R}$) to $\mathbf{[2^{Mg,R}]_2}$.

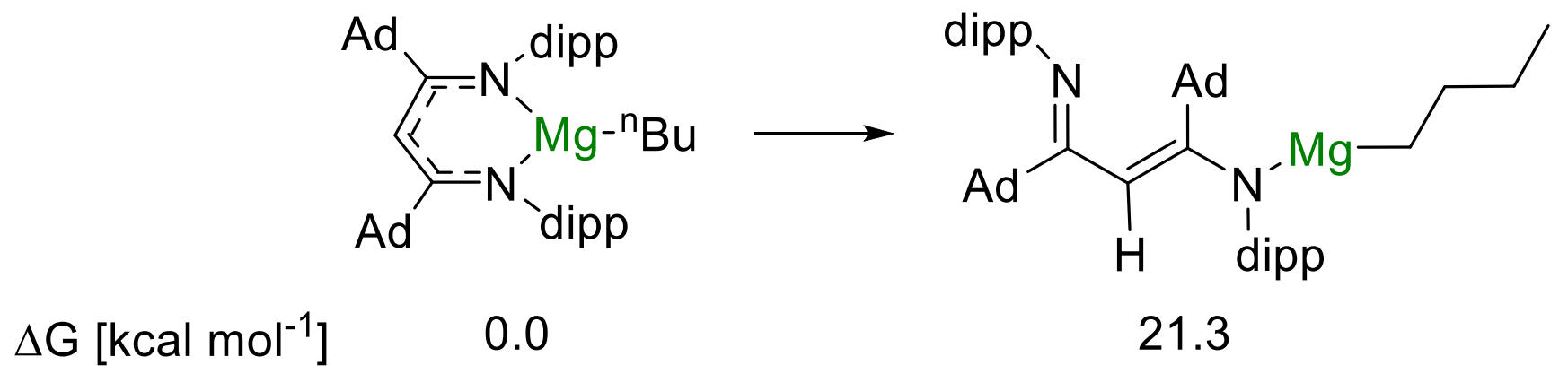

Figure S307. Gibbs free energy difference between the open [(κ^1-^{Ad}BDI)Mg–^{n}Bu] (**H**) and [((κ^2-^{Ad}BDI)–Mg–^{n}Bu (**1^{Ad}**) complex.

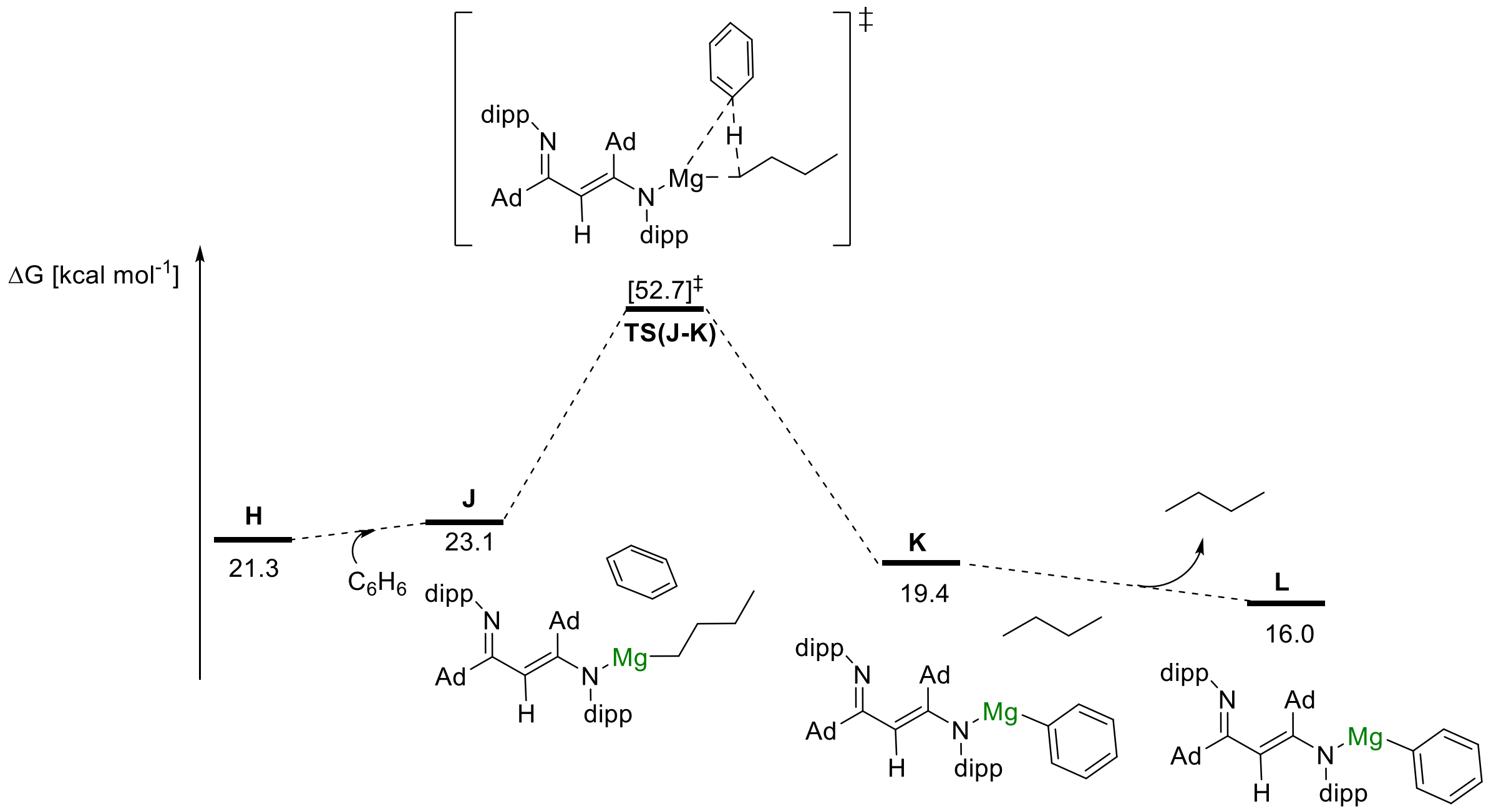

Figure S308. C(sp^2)–H deprotonation of benzene from the monomeric open [(κ^1-^{Ad}BDI)Mg–^{n}Bu] (**H**) complex to yield **L** and ^{n}BuH.

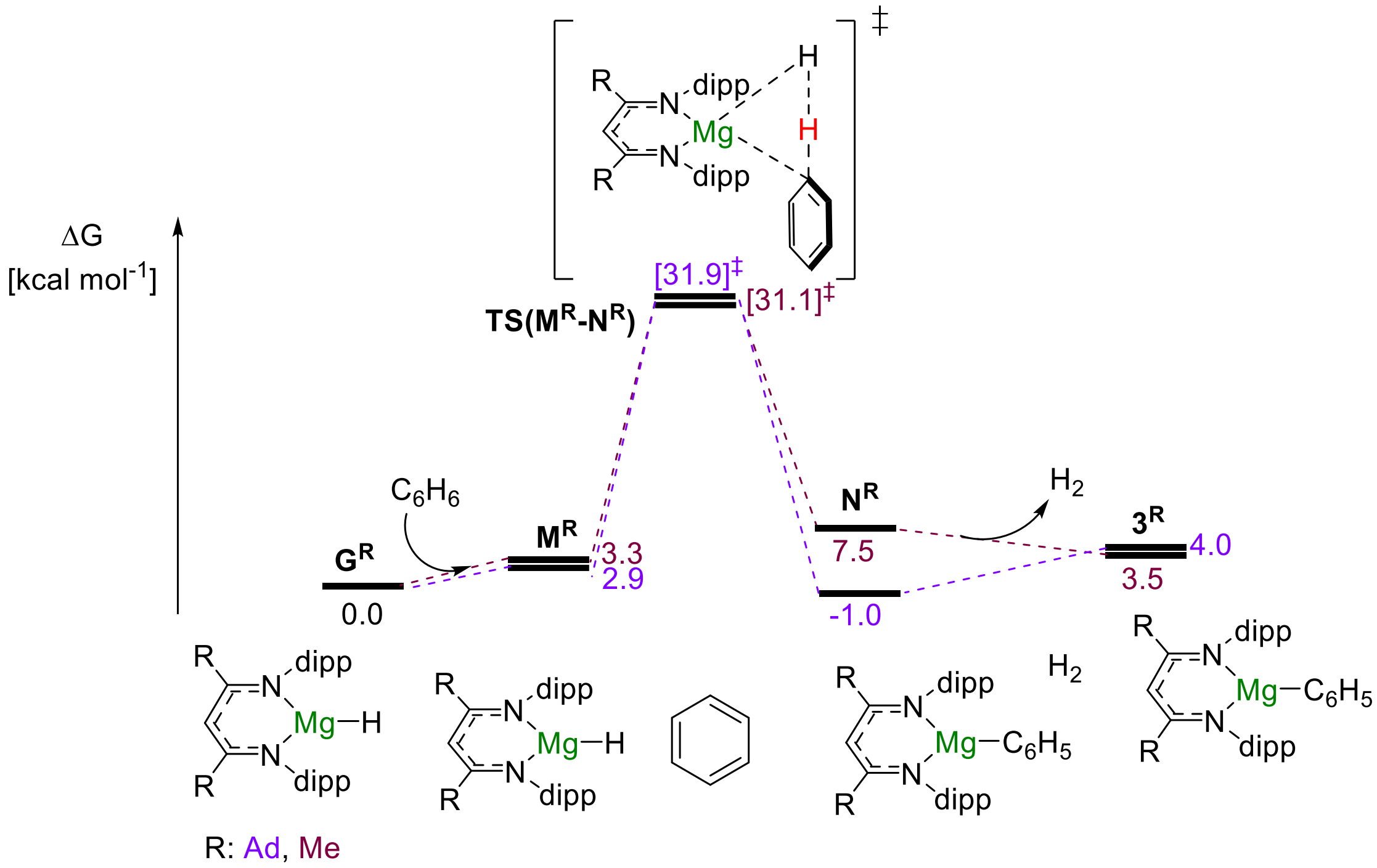

Figure S309. H_2 evolution through C(sp[2])–H deprotonation by **G**[R] complex to yield **3**[R] and H_2.

6.5 Energy contributions for calculated structures and transition states

Table S17. Relative energies (kcal mol^{-1}) for the formation of **3**^{Ad} or **3**^{Me} with ^{n}BuH through the C(sp^2)-H deprotonation of benzene by **1**^{Ad} or **1**^{Me}, respectively. Free energies are quoted relative to **1**^{Ad}(+C_6H_6) or **1**^{Me}(+C_6H_6).

	ΔE_{BS1}	ΔH_{BS1}	ΔG_{BS1}	$\Delta G_{BS1/C_6H_6}$	$\Delta G_{BS1/D3^{BJ}/C_6H_6}$	ΔE_{BS2}	ΔG_{bnz}
**1**^{Ad}(+C_6H_6)	0.0	0.0	0.0	0.0	0.0	0.0	**0.0**
**A**^{Ad}	−0.3	−0.1	5.5	6.0	2.4	0.3	**3.0**
**TS(A**^{Ad}**–B**^{Ad}**)**	39.1	37.7	52.1	55.7	37.8	40.2	**38.9**
**B**^{Ad}	−6.3	−5.8	−4.8	−3.8	−7.3	−5.7	**−6.7**
**3**^{Ad}(+^{n}BuH)	−5.1	−4.0	−3.0	−2.1	−6.5	−4.3	**−5.7**
**1**^{Me}(+C_6H_6)	0.0	0.0	0.0	0.0	0.0	0.0	**0.0**
**A**^{Me}	−0.1	0.0	3.6	3.7	2.4	0.2	**2.7**
**TS(A**^{Me}**–B**^{Me}**)**	33.1	31.0	43.6	46.7	32.0	34.0	**32.9**
**B**^{Me}	−6.1	−5.6	−3.9	−3.4	−6.5	−5.4	**−5.8**
**3**^{Me}(+^{n}BuH)	−6.1	−5.6	−5.7	−5.3	−8.2	−5.4	**−7.6**

Table S18. Relative energies (kcal mol^{-1}) for the bending of the BDI ligand in **A**^{Ad} prior to deprotonation of benzene.

	ΔE_{BS1}	ΔH_{BS1}	ΔG_{BS1}	$\Delta G_{BS1/C_6H_6}$	$\Delta G_{BS1/D3^{BJ}/C_6H_6}$	ΔE_{BS2}	ΔG_{bnz}
**1**^{Ad}(+C_6H_6)	0.0	0.0	0.0	0.0	0.0	0.0	**0.0**
**A**^{Ad}	−0.3	−0.1	5.5	6.0	2.4	0.3	**3.0**
**A'**^{Ad}	13.1	12.5	21.2	20.6	19.1	14.2	**20.1**

Table S19. Relative energies (kcal mol^{-1}) for the deprotonation of benzene by **[1**^{Me}**]**$_2$ in kcal/mol.

	ΔE_{BS1}	ΔH_{BS1}	ΔG_{BS1}	$\Delta G_{BS1/C_6H_6}$	$\Delta G_{BS1/D3^{BJ}/C_6H_6}$	ΔE_{BS2}	ΔG_{bnz}
**[1**^{Me}**]**$_2$(+C_6H_6)	**0.0**	**0.0**	**0.0**	**0.0**	**0.0**	**0.0**	**0.0**
**O**^{Me}	**−11.1**	**−12.4**	**−25.1**	**−35.3**	**14.8**	**−14.2**	**11.7**
**TS(O**^{Me}**-P**^{Me}**)**	**44.0**	**41.3**	**53.0**	**51.5**	**47.5**	**45.7**	**49.2**
**P**^{Me}	**−19.9**	**−21.1**	**−33.4**	**−43.3**	**6.3**	**−22.5**	**3.7**

Table S20: Relative energies (kcal mol^{-1}) for the deprotonation of benzene by 2× **1**^{Ad} in kcal/mol.

	ΔE_{BS1}	ΔH_{BS1}	ΔG_{BS1}	$\Delta G_{BS1/C_6H_6}$	$\Delta G_{BS1/D3^{BJ}/C_6H_6}$	ΔE_{BS2}	ΔG_{bnz}
2×**1**^{Ad}(+C_6H_6)	**0.0**	**0.0**	**0.0**	**0.0**	**0.0**	**0.0**	**0.0**
**Q**	**57.2**	**57.2**	**86.0**	**88.6**	**44.5**	**60.2**	**47.6**
**TS(Q-R)**	**61.8**	**60.9**	**91.7**	**97.8**	**47.0**	**66.7**	**52.0**
**R**	**−6.5**	**−5.8**	**4.2**	**5.5**	**0.3**	**−5.6**	**1.2**

Table S20. Relative energies (kcal mol^{-1}) for the dissociation of **[1**^{Me}**]**$_2$ resulting into the formation of two molecules of **1**^{Me}. Free energies are quoted relative to **[1**^{Me}**]**$_2$.

	ΔE_{BS1}	ΔH_{BS1}	ΔG_{BS1}	$\Delta G_{BS1/C_6H_6}$	$\Delta G_{BS1/D3^{BJ}/C_6H_6}$	ΔE_{BS2}	ΔG_{bnz}
**[1**^{Me}**]**$_2$	0.0	0.0	0.0	0.0	0.0	0.0	**0.0**
**TS([1**^{Me}**]**$_2$**–2(1**^{Me}**))**	5.0	3.6	0.0	−6.2	11.0	3.5	**9.6**
**2(1**^{Me}**)**	−13.7	−15.4	−32.3	−42.9	10.7	−17.2	**7.2**
**1**^{Me} **(+1**^{Me}**)**	−13.7	−15.6	−38.4	−48.9	5.8	−17.2	**2.3**

Table S21. Relative energies (kcal mol^{-1}) for the formation of **5'** or **5-*o*/*m*/*p*** through the C(sp^3)-H or C(sp^2)-H deprotonation of toluene by **1^{Ad}**, respectively. Free energies are quoted relative to **1^{Ad}**(+C_7H_8).

	ΔE_{BS1}	ΔH_{BS1}	ΔG_{BS1}	$\Delta G_{BS1/C_6H_6}$	$\Delta G_{BS1/C_7H_8}$	$\Delta G_{BS1/D3^{BJ}/C_6H_6}$	$\Delta G_{BS1/D3^{BJ}/C_7H_8}$	ΔE_{BS2}	ΔG_{bnz}	ΔG_{tol}
**1^{Ad}**(+C_7H_8)	0.0	0.0	0.0	0.0	0.0	0.0	0.0	0.0	**0.0**	**0.0**
**C^{Me}**	9.1	9.2	17.3	17.7	17.7	15.8	15.8	8.4	**15.1**	**15.1**
**TS(C^{Me}–D^{Me})**	40.1	38.8	55.6	58.2	58.2	39.0	39.0	41.1	**40.0**	**39.9**
**D^{Me}**	−5.9	−5.4	−0.9	−0.6	−0.7	−5.3	−5.3	−5.5	**−4.8**	**−4.9**
**5'**(+^{n}BuH)	−5.9	−5.5	−3.6	−3.3	−3.4	−7.7	−7.8	−5.5	**−7.3**	**−7.3**
**C**	−0.2	0.0	5.0	5.1	5.1	3.8	3.8	0.1	**4.0**	**4.0**
**TS(C–D^o)**	43.1	42.2	59.5	63.5	63.4	41.0	40.9	45.0	**42.8**	**42.8**
**D^o**	−2.5	−1.8	4.0	4.9	4.9	−2.9	−3.0	−1.3	**−1.7**	**−1.8**
**5–*o*** (+^{n}BuH)	−2.5	−1.8	1.5	2.2	2.2	−5.3	−5.4	−1.3	**−4.2**	**−4.2**
**TS(C–D^m)**	39.1	37.8	53.9	57.5	57.5	38.8	38.8	40.4	**40.0**	**40.0**
**D^m**	−6.1	−5.6	−1.7	−0.4	−0.4	−4.5	−4.5	−5.5	**−3.9**	**−3.9**
**5–*m***(+^{n}BuH)	−6.1	−5.7	−4.7	−3.4	−3.4	−7.3	−7.3	−5.5	**−6.6**	**−6.6**
**TS(C–D^p)**	38.9	37.4	53.3	56.9	56.9	38.7	38.7	40.0	**39.9**	**39.9**
**D^p**	−6.2	−5.7	−2.5	−1.2	−1.2	−4.6	−4.6	−5.6	**−4.0**	**−4.1**
**5–*p***(+^{n}BuH)	−6.2	−5.8	−5.5	−4.3	−4.3	−7.6	−7.6	−5.6	**−7.0**	**−7.0**

Table S22. Relative energies (kcal mol^{-1}) for the formation of **[2^{Mg,R}]$_2$** (R = Ad and Me) through the β-H elimination of **1^{Ad}** or **1^{Me}**, respectively. Free energies are quoted relative to **1^{Ad}** or **1^{Me}**, respectively.

	ΔE_{BS1}	ΔH_{BS1}	ΔG_{BS1}	$\Delta G_{BS1/C_6H_6}$	$\Delta G_{BS1/D3^{BJ}/C_6H_6}$	ΔE_{BS2}	ΔG_{bnz}
**1^{Ad}**	0.0	0.0	0.0	0.0	0.0	0.0	**0.0**
**TS(1^{Ad}–E^{Ad})**	38.7	35.7	38.6	40.2	36.2	37.0	**34.5**
**E^{Ad}**	28.4	24.4	27.2	27.5	22.7	26.9	**21.2**
**TS(E^{Ad}–F^{Ad})**	28.7	25.5	27.8	27.9	23.9	27.3	**22.6**
**F^{Ad}**	22.1	18.1	12.4	10.0	17.7	20.0	**15.6**
**G^{Ad}**(+C_4H_8)	22.6	18.5	7.1	4.3	14.2	20.1	**11.7**
**2G^{Ad}**	21.9	17.9	13.5	11.8	16.3	20.0	**14.4**
**TS(2G^{Ad}–[2^{Mg,R}]$_2$)**	36.4	34.3	42.7	46.8	14.2	37.3	**15.1**
**[2^{Mg,Ad}]$_2$**	30.2	28.6	36.2	41.6	5.2	31.0	**6.0**
**1^{Me}**	0.0	0.0	0.0	0.0	0.0	0.0	**0.0**
**TS(1^{Me}–E^{Me})**	35.3	31.9	34.3	35.3	31.9	33.4	**30.0**
**E^{Me}**	25.8	22.3	22.6	22.1	18.2	24.1	**16.5**
**TS(E^{Me}–F^{Me})**	26.3	22.9	24.5	23.5	22.3	24.7	**20.6**
**F^{Me}**	22.4	18.6	13.9	11.7	17.5	20.5	**15.7**
**G^{Me}**(+C_4H_8)	22.8	18.7	7.8	5.2	12.5	20.7	**10.3**
**2G^{Me}**	21.9	18.0	15.1	13.7	16.0	20.3	**14.4**
**TS(2G^{Me}–[2^{Mg,Me}]$_2$)**	24.7	21.2	25.0	24.8	15.7	23.6	**14.7**
**[2^{Mg,Me}]$_2$**	11.5	10.3	19.0	23.7	0.0	10.1	**−1.4**

Table S23. Relative energies (kcal mol^{-1}) for the formation of **L** and ^{n}BuH through the BDI opening and C(sp^2)-H deprotonation of benzene by **J**. Free energies are quoted relative to **1^{Ad}**.

	ΔE_{BS1}	ΔH_{BS1}	ΔG_{BS1}	$\Delta G_{BS1/C_6H_6}$	$\Delta G_{BS1/D3^{BJ}/C_6H_6}$	ΔE_{BS2}	ΔG_{bnz}
**1^{Ad}**	0.0	0.0	0.0	0.0	0.0	0.0	**0.0**
**H**	23.0	22.2	20.9	17.7	22.6	21.7	**21.3**
**H**(+C_6H_6)	23.0	22.2	20.9	17.7	22.6	21.7	**21.3**
**J**	23.0	22.2	24.5	21.9	23.9	22.1	**23.1**
**TS(J–K)**	55.5	52.8	63.1	62.6	52.9	55.3	**52.7**
**K**	19.4	19.0	20.8	17.6	20.3	18.5	**19.4**
**L**(+^{n}BuH)	19.4	18.9	16.9	13.4	17.1	18.3	**16.0**

Table S24. Relative energies (kcal mol^{-1}) for the formation of **3^{Ad}** or **3^{Me}** with H_2 evolution through the C(sp^2)-H deprotonation of benzene by **G^{Mg,R}** (R=Ad and Me), respectively. Free energies are quoted relative to **G^{Mg,R}**(+C_6H_6), respectively.

	ΔE_{BS1}	ΔH_{BS1}	ΔG_{BS1}	$\Delta G_{BS1/C_6H_6}$	$\Delta G_{BS1/D3^{BJ}/C_6H_6}$	ΔE_{BS2}	ΔG_{bnz}
**G^{Ad}**(+C_6H_6)	0.0	0.0	0.0	0.0	0.0	0.0	**0.0**
**M^{Ad}**	−1.0	−0.7	4.1	4.6	2.5	−0.6	**2.9**
**TS(M^{Ad}–N^{Ad})**	31.5	30.0	41.6	46.6	31.2	32.2	**31.9**
**N^{Ad}**	9.0	5.4	6.6	10.9	−1.4	9.5	**−1.0**
**3^{Ad}**(+H_2)	10.2	7.1	12.5	16.8	3.3	10.9	**4.0**
**G^{Me}**(+C_6H_6)	0.0	0.0	0.0	0.0	0.0	0.0	**0.0**
**M^{Me}**	−0.9	−0.6	3.9	4.5	3.0	−0.6	**3.3**
**TS(M^{Me}–N^{Me})**	29.5	27.5	38.7	42.8	30.8	29.8	**31.1**
**N^{Me}**	9.1	6.0	13.5	17.1	7.1	9.4	**7.5**
**3^{Me}**(+H_2)	9.0	5.3	9.1	12.6	3.4	9.2	**3.5**

6.6 Cartesian Coordinates and Raw Electronic Energies (in Hartrees)

1^{Ad}

SCF (BP86) Energy = -2296.052513
Enthalpy 0K = -2294.942376
Enthalpy 298K = -2294.885108
Free Energy 298K = -2295.034709
Lowest Frequency = 12.2071 cm^{-1}
Second Frequency = 16.0015 cm^{-1}
SCF (BP86-D3^{BJ}) Energy = -2296.378676
SCF (C_6H_6) Energy = -2296.090194
SCF ($C_6H_5CH_3$) Energy = -2296.089232
SCF (BS2) Energy = -2298.352815

Mg	0.38777	-1.77224	0.14224
N	-1.45222	-0.80754	0.06356
N	1.51317	-0.02717	0.06545
C	-1.58328	0.53122	-0.00620
C	-2.94802	1.31344	-0.11116
C	-3.07551	2.32517	1.07689
H	-3.10089	1.76076	2.03528
H	-2.18488	2.98472	1.12666
C	-4.24009	0.44747	-0.09833
H	-4.21796	-0.28969	-0.92411
H	-4.30185	-0.13940	0.83820
C	-2.96191	2.12212	-1.45325
H	-2.06277	2.76796	-1.52539
H	-2.90459	1.41020	-2.30632
C	-4.34383	3.19917	0.94097
H	-4.37916	3.91019	1.79609
C	-5.59863	2.30229	0.95359
H	-6.51978	2.92230	0.87878
H	-5.66294	1.74594	1.91510
C	-5.51609	1.31592	-0.22938
H	-6.39830	0.63890	-0.21521
C	-5.48420	2.10058	-1.55681
H	-6.40449	2.71683	-1.66569
H	-5.46443	1.39787	-2.41939
C	-4.22987	2.99789	-1.57346
H	-4.18248	3.56369	-2.53038
C	-4.28590	3.98763	-0.38739
H	-3.39335	4.65256	-0.40026
H	-5.17712	4.64718	-0.48043
C	-0.44669	1.39261	-0.01358
H	-0.72474	2.44876	-0.05736
C	0.96564	1.20383	0.01943
C	1.76736	2.56165	0.02805
C	1.35725	3.37697	1.30218
H	0.25570	3.50193	1.34309
H	1.64017	2.79858	2.20949
C	1.40331	3.40811	-1.23746
H	1.71921	2.85532	-2.14980
H	0.30460	3.53959	-1.31727
C	3.31805	2.44393	0.05430
H	3.64395	1.85900	0.93605
H	3.67886	1.89117	-0.83423
C	2.02944	4.76862	1.32490
H	1.69209	5.31210	2.23533
C	1.61489	5.56085	0.06390
H	0.51089	5.70143	0.04494
H	2.06615	6.57763	0.08470
C	2.07694	4.79946	-1.19964
H	1.77476	5.36589	-2.10839
C	3.61046	4.63597	-1.17033
H	3.95768	4.10786	-2.08614
H	4.10696	5.63193	-1.16566
C	4.00054	3.83388	0.08821
H	5.10159	3.67832	0.10705
C	3.56274	4.60453	1.35041
H	4.05964	5.59955	1.38971
H	3.87437	4.05368	2.26567
C	-2.46381	-1.81648	0.16437
C	-2.94380	-2.21015	1.45421
C	-2.49796	-1.48679	2.72892
H	-2.05587	-0.51607	2.42107
C	-3.66075	-1.17624	3.69281
H	-4.47483	-0.61512	3.18878
H	-3.30278	-0.56330	4.54670
H	-4.10573	-2.09958	4.12038
C	-1.39130	-2.28354	3.45442
H	-1.76816	-3.27284	3.79033
H	-1.02366	-1.73513	4.34764
H	-0.52044	-2.48108	2.79380
C	-3.81913	-3.31014	1.54523
H	-4.19582	-3.61712	2.53408
C	-4.22040	-4.02408	0.40697
H	-4.90640	-4.88064	0.50123
C	-3.73176	-3.64318	-0.84935
H	-4.04040	-4.20996	-1.74305
C	-2.85047	-2.55270	-0.99840
C	-2.32689	-2.20023	-2.39432
H	-1.69812	-1.29006	-2.28839
C	-3.46187	-1.86297	-3.38485
H	-4.11909	-2.73979	-3.56649
H	-3.04538	-1.55319	-4.36673
H	-4.10330	-1.03760	-3.01299
C	-1.43300	-3.32576	-2.96001
H	-0.59919	-3.57349	-2.27066
H	-0.99866	-3.03276	-3.93917
H	-2.01404	-4.25940	-3.11720
C	2.89091	-0.41147	-0.00668
C	3.59099	-0.77624	1.18509
C	2.96649	-0.61769	2.57476
H	1.97237	-0.14119	2.43386
C	3.79423	0.30669	3.49331
H	4.79688	-0.12127	3.70612
H	3.28209	0.44929	4.46842
H	3.94879	1.30828	3.04177
C	2.73999	-1.98808	3.24993
H	2.13497	-2.66448	2.61144
H	2.21681	-1.86893	4.22235
H	3.70450	-2.50170	3.44961
C	4.89487	-1.30154	1.07194
H	5.44333	-1.57789	1.98734
C	5.50756	-1.48005	-0.17499
H	6.52641	-1.89323	-0.24060
C	4.80772	-1.13493	-1.34036
H	5.28809	-1.28342	-2.32058
C	3.50409	-0.60448	-1.28610
C	2.75812	-0.27797	-2.58346
H	1.91977	0.40074	-2.32106
C	3.62970	0.45164	-3.62490
H	4.10344	1.36094	-3.20028
H	3.01378	0.76287	-4.49474

H	4.44124	-0.19639	-4.01825
C	2.13651	-1.55175	-3.19586
H	2.92303	-2.28239	-3.47980
H	1.54447	-1.31210	-4.10447
H	1.46128	-2.07030	-2.48163
C	0.82190	-3.85675	0.33340
H	0.79901	-4.10103	1.42481
H	-0.05162	-4.42011	-0.07558
C	2.12388	-4.42065	-0.26867
H	2.14572	-4.24583	-1.37068
H	3.00614	-3.86083	0.12256
C	2.35308	-5.92512	-0.01832
H	1.47877	-6.49325	-0.41232
H	2.35331	-6.10966	1.08102
C	3.64741	-6.47296	-0.63438
H	3.65826	-6.33987	-1.73823
H	3.78049	-7.55652	-0.42956
H	4.54050	-5.94564	-0.23421

C_6H_6

SCF (BP86) Energy = -232.076218
Enthalpy 0K = -231.978537
Enthalpy 298K = -231.973085
Free Energy 298K = -232.004380
Lowest Frequency = 401.2144 cm^{-1}
Second Frequency = 401.2147 cm^{-1}
SCF (BP86-$D3^{BJ}$) Energy = -232.093114
SCF (C_6H_6) Energy = -232.083921
SCF (BS2) Energy = -232.326587

C	0.00000	1.40669	-0.00000
C	-1.21823	0.70334	0.00000
C	1.21823	0.70334	-0.00000
H	-2.17256	1.25433	0.00000
H	2.17256	1.25433	-0.00000
C	-1.21823	-0.70334	0.00000
C	1.21823	-0.70334	-0.00000
H	-2.17256	-1.25433	0.00000
H	2.17256	-1.25433	-0.00000
C	0.00000	-1.40669	0.00000
H	0.00000	-2.50866	0.00000
H	-0.00000	2.50866	0.00000

A^{Ad}

SCF (BP86) Energy = -2528.129237
Enthalpy 0K = -2526.921045
Enthalpy 298K = -2526.856720
Free Energy 298K = -2527.030263
Lowest Frequency = 5.0718 cm^{-1}
Second Frequency = 7.8256 cm^{-1}
SCF (BP86-$D3^{BJ}$) Energy = -2528.477997
SCF (C_6H_6) Energy = -2528.173945
SCF (BS2) Energy = -2530.678934

Mg	0.75671	-0.79753	0.51991
N	0.36420	1.23926	0.39651
N	-1.17885	-1.38850	0.05041
C	-0.84693	1.74082	0.08598
C	-1.21373	3.27276	0.02338
C	-0.05552	4.26958	0.31429
H	0.37975	4.07268	1.31315
H	0.76612	4.13009	-0.41438
C	-2.33325	3.56309	1.08086
H	-3.19675	2.88393	0.92643
H	-1.93859	3.34056	2.09703
C	-1.76884	3.63327	-1.39540
H	-0.97183	3.46394	-2.15295
H	-2.60805	2.96156	-1.66976
C	-0.53393	5.74148	0.25204
H	0.33706	6.39785	0.46973
C	-1.63974	5.98032	1.30034
H	-1.97928	7.03976	1.27186
H	-1.24569	5.79679	2.32469
C	-2.81739	5.02902	1.00548
H	-3.62161	5.17764	1.75994
C	-3.37250	5.31515	-0.40848
H	-3.75502	6.35834	-0.46611
H	-4.23529	4.64590	-0.62449
C	-2.25562	5.09971	-1.45521
H	-2.65497	5.30054	-2.47412
C	-1.07955	6.05206	-1.15686
H	-0.27788	5.92182	-1.91744
H	-1.41299	7.11197	-1.21944
C	-1.95576	0.89091	-0.19976
H	-2.85832	1.45419	-0.45027
C	-2.16793	-0.51815	-0.23414
C	-3.63129	-0.90943	-0.67232
C	-3.87250	-0.36744	-2.12285
H	-3.66040	0.72068	-2.16762
H	-3.15259	-0.85519	-2.81679
C	-4.68003	-0.24705	0.28282
H	-4.54259	-0.64877	1.31121
H	-4.51298	0.84744	0.35075
C	-3.95679	-2.43017	-0.70168
H	-3.25162	-2.96215	-1.36878
H	-3.82469	-2.87283	0.30449
C	-5.32286	-0.62591	-2.59017
H	-5.44712	-0.20847	-3.61403
C	-6.30645	0.07031	-1.62219
H	-6.12639	1.16871	-1.61668
H	-7.35380	-0.08011	-1.96618
C	-6.12619	-0.50653	-0.19946
H	-6.82986	-0.00246	0.49966
C	-6.40481	-2.02345	-0.21909
H	-6.30020	-2.44611	0.80491
H	-7.45065	-2.22195	-0.54363
C	-5.40532	-2.69920	-1.17993
H	-5.57091	-3.79894	-1.18073
C	-5.60036	-2.14322	-2.60535
H	-6.63481	-2.34557	-2.96237
H	-4.91070	-2.65203	-3.31506
C	1.60177	1.92400	0.61965
C	2.45220	2.22579	-0.49169
C	2.01085	1.94597	-1.93162
H	0.90469	1.85149	-1.92544
C	2.36761	3.07945	-2.91375
H	1.98684	4.06180	-2.56480
H	1.92615	2.87800	-3.91238
H	3.46421	3.17646	-3.05903
C	2.58165	0.59974	-2.42941
H	3.69156	0.61940	-2.45115
H	2.22073	0.36677	-3.45362
H	2.29147	-0.24565	-1.76907
C	3.73081	2.76119	-0.24085
H	4.38902	3.00190	-1.09059
C	4.18454	2.99384	1.06609
H	5.18586	3.41898	1.23954
C	3.35390	2.67558	2.14837

H	3.71424	2.85053	3.17534
C	2.06571	2.13602	1.95504
C	1.21041	1.78618	3.17668
H	0.22656	1.43483	2.79779
C	0.94487	3.00736	4.08299
H	1.88459	3.39751	4.52847
H	0.27077	2.73290	4.92186
H	0.47002	3.84047	3.52491
C	1.83896	0.63446	3.99173
H	2.01911	-0.26295	3.36392
H	1.17980	0.34053	4.83569
H	2.81892	0.93412	4.42051
C	-1.23095	-2.81340	0.18246
C	-0.73760	-3.64413	-0.87131
C	-0.31118	-3.07221	-2.22713
H	-0.48089	-1.97443	-2.18667
C	-1.16035	-3.62700	-3.39157
H	-1.02113	-4.72271	-3.50948
H	-0.86770	-3.15046	-4.35111
H	-2.24373	-3.44387	-3.23867
C	1.19055	-3.30794	-2.49824
H	1.82312	-2.90578	-1.68059
H	1.50148	-2.82238	-3.44737
H	1.41815	-4.39149	-2.58799
C	-0.64605	-5.03442	-0.65233
H	-0.27342	-5.68172	-1.46309
C	-1.01689	-5.61008	0.56949
H	-0.93546	-6.69815	0.71981
C	-1.48734	-4.78647	1.60286
H	-1.77251	-5.24050	2.56513
C	-1.60152	-3.39242	1.43876
C	-2.08279	-2.52170	2.60359
H	-2.42056	-1.55445	2.17618
C	-3.27671	-3.12539	3.36934
H	-4.11518	-3.38110	2.68876
H	-3.65699	-2.40384	4.12265
H	-2.99715	-4.04908	3.91877
C	-0.92321	-2.20757	3.57320
H	-0.52658	-3.13612	4.03510
H	-1.25480	-1.53042	4.38868
H	-0.06996	-1.71727	3.05709
C	2.59260	-1.81984	0.92022
H	3.06905	-2.03575	-0.06877
H	3.28884	-1.08865	1.39809
C	2.56227	-3.12112	1.74593
H	2.14036	-2.92233	2.76014
H	1.85835	-3.85434	1.28595
C	3.93081	-3.80906	1.92572
H	4.64046	-3.08519	2.38902
H	4.35325	-4.03447	0.91902
C	3.88147	-5.09406	2.76315
H	3.50209	-4.89392	3.78895
H	4.88302	-5.56305	2.86559
H	3.20456	-5.84844	2.30658
C	8.23775	0.56415	-1.50130
C	7.08364	0.13112	-0.82303
C	8.90911	-0.30903	-2.37649
H	6.55419	0.81203	-0.13752
H	9.81359	0.02983	-2.90720
C	6.60004	-1.17579	-1.01927
C	8.42589	-1.61550	-2.57329
H	5.69669	-1.51269	-0.48547
H	8.95208	-2.29991	-3.25834
C	7.27200	-2.04834	-1.89486
H	6.89426	-3.07215	-2.04786
H	8.61742	1.58720	-1.34643

TS (A^{Ad}–B^{Ad})

SCF (BP86) Energy = -2528.066460
Enthalpy 0K = -2526.860773
Enthalpy 298K = -2526.798421
Free Energy 298K = -2526.956010
Lowest Frequency = -1286.5732 cm^{-1}
Second Frequency = 13.4261 cm^{-1}
SCF (BP86-D3^{BJ}) Energy = -2528.438000
SCF (C_6H_6) Energy = -2528.106215
SCF (BS2) Energy = -2530.615347

Mg	-0.14036	1.46589	-0.23482
N	1.56750	0.40810	0.28858
N	-1.59479	-0.01442	-0.06527
C	1.49740	-0.87258	-0.17100
C	2.75389	-1.75887	-0.55667
C	4.14707	-1.18578	-0.16491
H	4.26593	-0.15909	-0.55663
H	4.23434	-1.10839	0.93568
C	2.73342	-1.89129	-2.12179
H	1.76098	-2.31150	-2.45366
H	2.80574	-0.87912	-2.57667
C	2.68291	-3.20121	0.04313
H	2.71461	-3.14302	1.15180
H	1.72326	-3.69330	-0.21633
C	5.30381	-2.06983	-0.69288
H	6.26424	-1.59951	-0.38762
C	5.23925	-2.16847	-2.23074
H	6.07824	-2.79063	-2.61488
H	5.35167	-1.15994	-2.68655
C	3.88455	-2.78622	-2.63369
H	3.81419	-2.85465	-3.74206
C	3.75343	-4.19630	-2.01560
H	4.55954	-4.86025	-2.39945
H	2.78701	-4.65902	-2.31713
C	3.83772	-4.09105	-0.47623
H	3.73316	-5.10347	-0.02660
C	5.19607	-3.47972	-0.07711
H	5.28221	-3.42273	1.03078
H	6.03027	-4.12552	-0.43162
C	0.26467	-1.52020	-0.46401
H	0.42593	-2.53081	-0.84752
C	-1.13256	-1.26488	-0.24044
C	-1.99003	-2.58901	-0.22685
C	-1.40988	-3.54785	0.86966
H	-0.32461	-3.70449	0.70830
H	-1.51002	-3.06123	1.86494
C	-1.87973	-3.30767	-1.61499
H	-2.31442	-2.65436	-2.40228
H	-0.81755	-3.46654	-1.89383
C	-3.50727	-2.43319	0.08212
H	-3.64890	-1.94323	1.06448
H	-3.99169	-1.77427	-0.66155
C	-2.13075	-4.91493	0.86903
H	-1.66890	-5.55985	1.64920
C	-1.96854	-5.58000	-0.51680
H	-0.89072	-5.74805	-0.73810
H	-2.45561	-6.58027	-0.52171
C	-2.60055	-4.67592	-1.59937
H	-2.48141	-5.14998	-2.59886
C	-4.09847	-4.47403	-1.29083

H	-4.57165	-3.84428	-2.07657
H	-4.63074	-5.45129	-1.30040
C	-4.23595	-3.80025	0.08998
H	-5.31008	-3.61678	0.31208
C	-3.62905	-4.71414	1.17367
H	-4.15647	-5.69390	1.19563
H	-3.75833	-4.25709	2.17988
C	2.61863	0.94082	1.10165
C	2.87657	0.38573	2.40038
C	2.02359	-0.72986	3.01363
H	1.42704	-1.19944	2.20574
C	2.85037	-1.84516	3.68645
H	3.62048	-2.26281	3.00608
H	2.18900	-2.67847	4.00449
H	3.37354	-1.48290	4.59666
C	1.02968	-0.12060	4.02582
H	1.56814	0.32684	4.88865
H	0.33618	-0.89308	4.42146
H	0.42552	0.68310	3.56178
C	3.90089	0.94012	3.19486
H	4.10038	0.50648	4.18778
C	4.66057	2.03092	2.75986
H	5.46370	2.44273	3.39122
C	4.36382	2.61125	1.52019
H	4.94147	3.48746	1.19115
C	3.34899	2.10536	0.68199
C	3.07691	2.80976	-0.65315
H	1.98146	2.76316	-0.83263
C	3.73934	2.10930	-1.85863
H	4.84246	2.06372	-1.73399
H	3.52648	2.66459	-2.79627
H	3.36511	1.07619	-1.99140
C	3.45740	4.30334	-0.65706
H	3.03110	4.84919	0.20877
H	3.07622	4.78578	-1.58039
H	4.55811	4.45283	-0.64756
C	-2.94384	0.41415	0.19282
C	-3.37843	0.62369	1.53997
C	-2.48417	0.35984	2.75466
H	-1.50568	-0.00460	2.37664
C	-3.05795	-0.73236	3.68364
H	-4.02710	-0.42056	4.12813
H	-2.36009	-0.93534	4.52323
H	-3.23002	-1.68831	3.14794
C	-2.23194	1.65918	3.55062
H	-1.76090	2.44224	2.92321
H	-1.56977	1.46510	4.41997
H	-3.18113	2.07761	3.94711
C	-4.68464	1.10182	1.76953
H	-5.02409	1.24847	2.80769
C	-5.55731	1.39538	0.71540
H	-6.57691	1.75978	0.91780
C	-5.10767	1.23737	-0.60100
H	-5.78393	1.48836	-1.43424
C	-3.81072	0.76592	-0.89351
C	-3.40571	0.66304	-2.36920
H	-2.33924	0.35387	-2.40309
C	-4.22556	-0.39158	-3.14565
H	-4.14038	-1.40303	-2.70170
H	-3.87918	-0.45519	-4.19888
H	-5.30433	-0.12726	-3.16558
C	-3.53025	2.02787	-3.08288
H	-4.59117	2.34715	-3.15964
H	-3.12842	1.96711	-4.11606
H	-2.97733	2.82668	-2.55028
C	-0.05804	2.60591	-2.27016
H	0.87132	2.05576	-2.56048
H	-0.89639	2.03141	-2.72801
C	-0.01588	4.00866	-2.90277
H	-0.94930	4.56290	-2.64459
H	0.80111	4.60760	-2.43478
C	0.16695	4.03669	-4.43168
H	-0.65244	3.44728	-4.90364
H	1.10652	3.49756	-4.69353
C	0.19716	5.45010	-5.02834
H	-0.74566	5.99912	-4.81605
H	0.33091	5.43189	-6.13049
H	1.02845	6.05241	-4.60198
C	0.19731	4.18139	1.40646
C	-0.68288	3.59936	0.45570
C	-0.24959	5.13795	2.33744
H	-0.31395	3.15204	-0.83883
H	0.45241	5.56510	3.07286
C	-2.03187	4.03851	0.47250
C	-1.59472	5.55098	2.32761
H	-2.74957	3.61868	-0.25564
H	-1.94601	6.30600	3.04992
C	-2.48905	4.99981	1.39139
H	-3.54386	5.32040	1.38084
H	1.25858	3.87696	1.43935

B^{Ad}

SCF (BP86) Energy = -2528.138775
Enthalpy 0K = -2526.930112
Enthalpy 298K = -2526.865346
Free Energy 298K = -2527.046712
Lowest Frequency = 0.8775 cm^{-1}
Second Frequency = 1.8534 cm^{-1}
SCF (BP86-D3^{BJ}) Energy = -2528.487430
SCF (C_6H_6) Energy = -2528.182633
SCF (BS2) Energy = -2530.688486

Mg	-2.04592	-0.13339	0.83879
N	-0.89174	-1.57244	-0.09188
N	-1.11598	1.48839	-0.04131
C	0.09611	-1.26933	-0.95754
C	0.99091	-2.31396	-1.72693
C	0.71835	-3.81434	-1.41953
H	0.87071	-4.02091	-0.34258
H	-0.33727	-4.06685	-1.63708
C	2.50174	-2.05877	-1.40369
H	2.77199	-1.00176	-1.60434
H	2.67474	-2.22505	-0.31729
C	0.77487	-2.12391	-3.26734
H	-0.28944	-2.33487	-3.51312
H	0.95846	-1.06883	-3.55700
C	1.63967	-4.74711	-2.24456
H	1.39359	-5.79880	-1.97967
C	3.11590	-4.45691	-1.90481
H	3.78709	-5.13147	-2.48179
H	3.30933	-4.65586	-0.82723
C	3.42113	-2.98191	-2.23634
H	4.47984	-2.74959	-1.98519
C	3.17769	-2.72991	-3.74207
H	3.85901	-3.36439	-4.35123
H	3.41255	-1.67222	-3.99720
C	1.70486	-3.04667	-4.08727
H	1.52637	-2.86087	-5.16969

C	1.40104	-4.52103	-3.75173
H	0.34814	-4.76538	-4.01604
H	2.04838	-5.19779	-4.35291
C	0.41595	0.08202	-1.28107
H	1.24503	0.15446	-1.98945
C	-0.08883	1.36132	-0.90511
C	0.68852	2.55268	-1.58352
C	0.62563	2.40850	-3.14167
H	0.97964	1.40561	-3.45666
H	-0.43412	2.48385	-3.47185
C	2.19042	2.49189	-1.14111
H	2.24984	2.62522	-0.03824
H	2.61746	1.49099	-1.35784
C	0.18126	3.98484	-1.24982
H	-0.87732	4.09902	-1.55336
H	0.21043	4.15960	-0.15719
C	1.48056	3.48826	-3.84427
H	1.41837	3.33090	-4.94397
C	2.95107	3.36151	-3.38437
H	3.35316	2.35976	-3.65597
H	3.58233	4.11222	-3.90956
C	3.03417	3.57230	-1.85546
H	4.09129	3.47672	-1.52179
C	2.49653	4.97322	-1.49805
H	2.57094	5.14430	-0.40106
H	3.11047	5.76139	-1.98828
C	1.02690	5.07381	-1.95565
H	0.61435	6.06777	-1.67525
C	0.94659	4.88979	-3.48475
H	1.53993	5.67628	-4.00229
H	-0.10500	5.00024	-3.83136
C	-1.31878	-2.85391	0.38760
C	-2.45436	-3.49035	-0.20388
C	-3.16426	-2.91001	-1.43091
H	-2.58238	-2.02107	-1.75648
C	-3.20007	-3.89618	-2.61786
H	-2.18351	-4.23497	-2.90583
H	-3.66394	-3.41972	-3.50733
H	-3.79815	-4.80122	-2.37929
C	-4.59119	-2.43490	-1.07768
H	-5.23539	-3.28922	-0.77933
H	-5.07090	-1.94240	-1.94988
H	-4.59126	-1.71882	-0.22911
C	-2.94375	-4.67851	0.37657
H	-3.81448	-5.17861	-0.07796
C	-2.35304	-5.23339	1.51950
H	-2.75214	-6.16212	1.95695
C	-1.25700	-4.58739	2.10940
H	-0.80273	-5.01647	3.01695
C	-0.72500	-3.39931	1.57015
C	0.43957	-2.70039	2.27796
H	0.84478	-1.94151	1.57607
C	1.59540	-3.65559	2.63820
H	1.29337	-4.40500	3.40009
H	2.44967	-3.08714	3.06252
H	1.96364	-4.21061	1.75054
C	-0.05623	-1.94989	3.53405
H	-0.87617	-1.23733	3.30016
H	0.76917	-1.37926	4.01014
H	-0.46011	-2.65888	4.28758
C	-1.78356	2.67407	0.40954
C	-2.92376	3.15828	-0.30693
C	-3.38562	2.50938	-1.61516
H	-2.56542	1.84649	-1.96253
C	-3.65702	3.53146	-2.73787
H	-4.51808	4.19113	-2.49960
H	-3.90029	3.00915	-3.68709
H	-2.77819	4.18302	-2.92407
C	-4.62777	1.62228	-1.37668
H	-4.45292	0.86053	-0.58697
H	-4.92361	1.09174	-2.30651
H	-5.49380	2.23174	-1.04178
C	-3.65513	4.23568	0.23154
H	-4.53162	4.61746	-0.31641
C	-3.29520	4.82972	1.44963
H	-3.88040	5.67171	1.85183
C	-2.19194	4.33301	2.15640
H	-1.91794	4.79077	3.12087
C	-1.42567	3.25598	1.66577
C	-0.25321	2.73050	2.49956
H	0.25148	1.94511	1.89701
C	0.79551	3.82116	2.80466
H	1.18200	4.29469	1.87859
H	1.66011	3.38904	3.35151
H	0.37358	4.62798	3.44080
C	-0.74939	2.07405	3.80712
H	-1.23307	2.82155	4.47138
H	0.09589	1.62325	4.36903
H	-1.50199	1.28087	3.61334
C	8.57050	-0.20664	1.61725
H	8.91679	-1.25963	1.53728
H	9.24652	0.41013	0.98661
C	8.57199	0.27331	3.07386
H	8.18399	1.31676	3.12377
H	7.85616	-0.33875	3.66950
C	9.95565	0.21936	3.74166
H	10.67153	0.83133	3.14601
H	10.34356	-0.82411	3.69179
C	9.95698	0.69935	5.19822
H	9.61134	1.75254	5.27813
H	10.97048	0.64681	5.64765
H	9.28065	0.08285	5.82875
C	-4.04741	-1.51511	2.79264
C	-3.64869	-0.27422	2.22299
C	-5.11050	-1.61601	3.71171
H	7.55682	-0.15461	1.16799
H	-5.38649	-2.59890	4.13029
C	-4.39083	0.86653	2.63673
C	-5.82074	-0.46484	4.09524
H	-4.13647	1.86553	2.23832
H	-6.65416	-0.53799	4.81363
C	-5.45737	0.78054	3.55331
H	-6.00737	1.69094	3.84676
H	-3.51883	-2.44544	2.51596

3[Ad]

SCF (BP86) Energy = -2369.807865
Enthalpy 0K = -2368.726043
Enthalpy 298K = -2368.670293
Free Energy 298K = -2368.814593
Lowest Frequency = 7.2203 cm^{-1}
Second Frequency = 21.4932 cm^{-1}
SCF (BP86-D3^{BJ}) Energy = -2370.145358
SCF (C6H6) Energy = -2369.847350
SCF (BS2) Energy = -2372.179688

Mg	-0.00709	1.65259	-0.00752
N	1.52129	0.26482	-0.00126

N	-1.55586	0.28145	-0.00859
C	1.29531	-1.07221	-0.00740
C	2.45932	-2.13544	-0.02386
C	1.94922	-3.61111	-0.05631
H	1.31851	-3.81478	0.83569
H	1.31306	-3.77275	-0.95302
C	3.36170	-2.00439	1.24213
H	3.78362	-0.98379	1.29656
H	2.74248	-2.15627	2.15368
C	3.36024	-1.94725	-1.28289
H	2.73990	-2.05505	-2.19998
H	3.78183	-0.92541	-1.28879
C	3.11142	-4.63089	-0.08160
H	2.67784	-5.65520	-0.10469
C	3.97626	-4.45935	1.18438
H	3.36822	-4.65833	2.09502
H	4.81015	-5.19621	1.18666
C	4.52850	-3.02030	1.21520
H	5.14558	-2.87290	2.12925
C	5.39457	-2.77245	-0.04043
H	6.25650	-3.47606	-0.05697
H	5.81801	-1.74422	-0.01733
C	4.52676	-2.96277	-1.30493
H	5.14263	-2.77351	-2.21212
C	3.97403	-4.40147	-1.33975
H	4.80753	-5.13782	-1.37738
H	3.36440	-4.55789	-2.25758
C	-0.02503	-1.60043	-0.00598
H	-0.04502	-2.68854	-0.00493
C	-1.34399	-1.04717	-0.00209
C	-2.46565	-2.15416	0.02144
C	2.76793	0.97564	0.04092
C	3.30696	1.38757	1.30560
C	2.62023	1.03660	2.63105
H	1.92851	0.19192	2.43172
C	4.45942	2.19920	1.32315
H	4.88178	2.50917	2.29212
C	5.07174	2.63067	0.13890
H	5.97317	3.26251	0.17653
C	4.51059	2.27019	-1.09287
H	4.97295	2.63629	-2.02334
C	3.35936	1.45968	-1.17302
C	2.73693	1.18755	-2.54792
H	2.00722	0.36034	-2.42117
C	3.76201	0.75152	-3.61524
H	4.36782	-0.11675	-3.28657
H	4.46365	1.57342	-3.87116
H	3.24177	0.46836	-4.55435
C	1.95903	2.42656	-3.04652
H	1.19751	2.77394	-2.31607
H	1.44413	2.21069	-4.00651
H	2.64517	3.28373	-3.21358
C	-2.79709	0.99999	-0.04230
C	-3.36797	1.48811	1.17460
C	-2.75664	1.17932	2.54493
H	-1.90317	0.48794	2.37717
C	-3.74603	0.46711	3.49193
H	-4.61517	1.11424	3.73593
H	-3.24961	0.20673	4.45061
H	-4.14220	-0.47014	3.04978
C	-2.20260	2.46068	3.20676
H	-1.48497	2.99068	2.54631
H	-1.68761	2.22119	4.16118
H	-3.01976	3.17659	3.43830
C	-4.51863	2.29982	1.10484
H	-4.96934	2.67213	2.03919
C	-5.09715	2.64873	-0.12274
H	-5.99657	3.28375	-0.15367
C	-4.50947	2.19502	-1.31184
H	-4.95310	2.48527	-2.27801
C	-3.36055	1.37891	-1.30173
C	-2.73018	0.95546	-2.63204
H	-1.94478	0.20486	-2.40230
C	-3.73685	0.28877	-3.59248
H	-4.52676	0.99762	-3.91965
H	-4.24212	-0.58090	-3.12382
H	-3.22087	-0.07075	-4.50769
C	-2.04108	2.15648	-3.31748
H	-1.51705	1.83847	-4.24373
H	-1.30016	2.64707	-2.65163
H	-2.78283	2.93407	-3.59846
C	-0.04553	3.77860	-0.01185
C	1.14433	4.55328	0.07547
H	2.12658	4.05090	0.14675
C	1.13039	5.96176	0.07329
H	2.07759	6.52335	0.14341
C	-0.09076	6.65291	-0.01898
H	-0.10809	7.75548	-0.02167
C	-1.28897	5.92330	-0.10788
H	-2.25348	6.45413	-0.18088
C	-1.25916	4.51439	-0.10305
H	-2.22458	3.98131	-0.17259
C	-2.29152	-3.02766	1.31133
H	-2.43279	-2.38338	2.20729
H	-1.25847	-3.42666	1.37367
C	-2.31194	-3.08379	-1.23068
H	-1.28075	-3.48735	-1.29336
H	-2.46870	-2.48083	-2.15245
C	-3.93691	-1.64902	0.02099
H	-4.11858	-0.99020	0.89165
H	-4.13328	-1.03429	-0.87827
C	-3.29405	-4.20363	1.33719
H	-3.11728	-4.80100	2.25910
C	-3.07561	-5.09372	0.09234
H	-2.04254	-5.50786	0.09314
H	-3.76886	-5.96357	0.11724
C	-3.31477	-4.25982	-1.18704
H	-3.15308	-4.89769	-2.08424
C	-4.75770	-3.71501	-1.18501
H	-4.94874	-3.13112	-2.11281
H	-5.48946	-4.55342	-1.17664
C	-4.94885	-2.82100	0.05729
H	-5.97480	-2.39178	0.05616
C	-4.73664	-3.65811	1.33509
H	-5.46877	-4.49521	1.37695
H	-4.91177	-3.03298	2.23885
C	3.59734	0.59036	3.73760
H	4.25108	1.42348	4.07202
H	4.25347	-0.23986	3.40682
H	3.03460	0.24524	4.63030
C	1.76935	2.22552	3.13167
H	1.03376	2.57452	2.37520
H	2.41056	3.10113	3.36702
H	1.21070	1.95381	4.05217

C_4H_{10}

SCF (BP86) Energy = -158.328928

Enthalpy 0K = -158.201245

Enthalpy 298K = -158.194427
Free Energy 298K = -158.229314
Lowest Frequency = 121.6464 cm^{-1}
Second Frequency = 223.4482 cm^{-1}
SCF (BP86-D3^{BJ}) Energy = -158.341577
SCF (C_6H_6) Energy = -158.333280
SCF ($C_6H_5CH_3$) Energy = -158.333050
SCF (BS2) Energy = -158.506517

C	1.96908	0.12021	0.00002
H	2.12322	0.76165	-0.89436
H	2.12334	0.76134	0.89459
C	0.57231	-0.51312	-0.00002
H	0.46423	-1.17919	-0.88684
H	0.46420	-1.17919	0.88681
H	2.76948	-0.64879	-0.00019
C	-0.57232	0.51307	-0.00002
H	-0.46422	1.17916	0.88679
H	-0.46424	1.17912	-0.88687
C	-1.96908	-0.12018	0.00001
H	-2.12310	-0.76184	0.89425
H	-2.76939	0.64891	0.00053
H	-2.12350	-0.76106	-0.89471

1^{Me}

SCF (BP86) Energy = -1596.189748
Enthalpy 0K = -1595.458216
Enthalpy 298K = -1595.413154
Free Energy 298K = -1595.539909
Lowest Frequency = 12.2121 cm^{-1}
Second Frequency = 15.2535 cm^{-1}
SCF (BP86-D3^{BJ}) Energy = -1596.381371
SCF (C_6H_6) Energy = -1596.218435
SCF (BS2) Energy = -1597.765485

N	-1.34550	-0.80552	0.77537
C	-2.14139	-2.30903	2.56869
H	-2.81106	-1.55971	3.03947
H	-1.74295	-2.97279	3.35810
H	-2.78425	-2.90690	1.88993
C	-1.02608	-1.62824	1.79405
C	-2.72450	-0.56294	0.45272
C	-3.42034	0.51471	1.08177
C	-4.74271	0.79190	0.67914
H	-5.28725	1.62408	1.15419
C	-5.37801	0.03388	-0.31422
H	-6.41138	0.26849	-0.61524
C	-4.68828	-1.02361	-0.92228
H	-5.19008	-1.61769	-1.70344
C	-3.36442	-1.34408	-0.55703
C	-2.75427	1.38929	2.14776
H	-1.79855	0.89888	2.42765
C	-2.40579	2.78283	1.58006
H	-1.76812	2.71046	0.67425
H	-1.86594	3.39587	2.33280
H	-3.32284	3.33707	1.28707
C	-3.60008	1.51419	3.43181
H	-4.55269	2.05540	3.24950
H	-3.04575	2.08031	4.20988
H	-3.85745	0.52036	3.85365
C	-2.64446	-2.49620	-1.26293
H	-1.67579	-2.64694	-0.74120
C	-3.42493	-3.82425	-1.17244
H	-3.64998	-4.09808	-0.12072
H	-2.83839	-4.65345	-1.62154
H	-4.39152	-3.77250	-1.71720
C	-2.32793	-2.13956	-2.73218
H	-3.25896	-1.97943	-3.31662
H	-1.75741	-2.95564	-3.22459
H	-1.72857	-1.20803	-2.80625
C	0.04107	1.36340	-2.16081
H	1.09317	1.69137	-2.34603
H	-0.18812	0.69452	-3.02784
C	-0.88912	2.59155	-2.23699
H	-0.65236	3.30591	-1.41169
H	-1.94779	2.28812	-2.05814
C	-0.82729	3.36711	-3.56861
H	0.22615	3.68152	-3.75186
H	-1.07618	2.66848	-4.40063
C	-1.75144	4.59073	-3.62609
H	-2.81530	4.30200	-3.48172
H	-1.68011	5.12055	-4.59958
H	-1.50149	5.32507	-2.82951
Mg	0.06595	0.15296	-0.40196
N	1.67352	-0.59998	0.66653
C	2.79627	-1.93746	2.41557
H	3.38648	-1.08605	2.81401
H	2.55064	-2.62038	3.24976
H	3.46700	-2.46645	1.70708
C	1.54579	-1.44265	1.70937
C	0.30179	-1.91192	2.20781
H	0.37949	-2.60319	3.05823
C	2.97799	-0.17860	0.23766
C	3.53920	1.02386	0.76544
C	4.77970	1.46753	0.26386
H	5.22145	2.39588	0.66010
C	5.46156	0.75301	-0.73113
H	6.42965	1.11723	-1.11021
C	4.90236	-0.42606	-1.24202
H	5.43966	-0.98275	-2.02682
C	3.66445	-0.91409	-0.77507
C	2.79554	1.85284	1.81581
H	2.01379	1.19945	2.25721
C	2.07289	3.04685	1.15223
H	1.38728	2.71557	0.34234
H	1.47641	3.61782	1.89512
H	2.80193	3.74569	0.68965
C	3.69995	2.32774	2.97011
H	4.46474	3.05713	2.62866
H	3.09616	2.83352	3.75273
H	4.23498	1.48045	3.44719
C	3.06691	-2.18315	-1.38824
H	2.21297	-2.48785	-0.74739
C	4.06051	-3.36220	-1.41802
H	4.47358	-3.57714	-0.41051
H	3.56018	-4.28344	-1.78421
H	4.91849	-3.16560	-2.09541
C	2.50402	-1.89721	-2.79861
H	3.31256	-1.59159	-3.49644
H	2.01346	-2.79930	-3.22242
H	1.75863	-1.07415	-2.78427

A^{Me}

SCF (BP86) Energy = -1828.266163
Enthalpy 0K = -1827.436687
Enthalpy 298K = -1827.384468
Free Energy 298K = -1827.538481
Lowest Frequency = 2.1802 cm^{-1}

Second Frequency = 4.7472 cm^{-1}
SCF (BP86-D3^{BJ}) Energy = -1828.476730
SCF (C_6H_6) Energy = -1828.302518
SCF (BS2) Energy = -1830.091800

Mg	-1.28524	0.06360	0.43395
N	-1.09134	1.67180	-0.85748
N	-1.13368	-1.34751	-1.07647
C	-0.96176	1.54442	-2.19193
C	-0.93722	0.30014	-2.87551
H	-0.83655	0.37787	-3.96681
C	-1.01152	-1.02802	-2.38017
C	-1.09574	2.97621	-0.25632
C	0.13148	3.54432	0.20287
C	1.46143	2.80613	0.02992
H	1.30773	2.02834	-0.74739
C	2.60742	3.71580	-0.45463
H	2.33671	4.25537	-1.38600
H	3.51765	3.11442	-0.66010
H	2.88554	4.47731	0.30467
C	1.85037	2.07636	1.33552
H	2.02043	2.80104	2.16009
H	2.78065	1.48486	1.20176
H	1.04883	1.38548	1.67647
C	0.09308	4.78542	0.87020
H	1.03266	5.23265	1.23225
C	-1.11664	5.46070	1.08533
H	-1.12497	6.42941	1.60985
C	-2.31527	4.89412	0.63120
H	-3.26445	5.42609	0.80599
C	-2.33286	3.65544	-0.04261
C	-3.66651	3.04882	-0.48632
H	-3.43260	2.19640	-1.15820
C	-4.54040	4.03635	-1.28585
H	-4.87668	4.89204	-0.66265
H	-5.45326	3.52977	-1.66427
H	-3.99611	4.45306	-2.15876
C	-4.43946	2.48026	0.72496
H	-3.83372	1.73866	1.28740
H	-5.37872	1.98348	0.40082
H	-4.71146	3.28674	1.43890
C	-1.17756	-2.72679	-0.67588
C	0.03527	-3.41179	-0.35832
C	1.40264	-2.73626	-0.49467
H	1.24261	-1.76750	-1.01244
C	2.38924	-3.55712	-1.35107
H	2.64724	-4.52476	-0.87049
H	3.33762	-2.99798	-1.49516
H	1.97288	-3.78496	-2.35440
C	2.00735	-2.42201	0.89142
H	1.32282	-1.80246	1.50764
H	2.96946	-1.87658	0.79048
H	2.20477	-3.35369	1.46352
C	-0.04204	-4.73555	0.12099
H	0.88733	-5.27180	0.37311
C	-1.27456	-5.38218	0.28637
H	-1.31244	-6.41636	0.66395
C	-2.45842	-4.70245	-0.03066
H	-3.42637	-5.21292	0.10148
C	-2.43837	-3.37805	-0.51435
C	-3.75867	-2.66824	-0.82611
H	-3.50576	-1.70169	-1.31079
C	-4.63780	-3.46074	-1.81596
H	-4.09408	-3.69098	-2.75572
H	-5.54626	-2.88024	-2.08243
H	-4.97932	-4.42534	-1.38393
C	-4.53654	-2.34611	0.46903
H	-4.82631	-3.27495	1.00505
H	-5.46697	-1.78261	0.24378
H	-3.92767	-1.73795	1.17043
C	-1.65964	0.03406	2.53599
H	-1.56693	1.08673	2.89935
H	-2.74216	-0.20693	2.68364
C	-0.82865	-0.88890	3.45084
H	-0.90740	-1.94771	3.10794
H	0.25680	-0.63979	3.36561
C	-1.21227	-0.83495	4.94369
H	-2.29113	-1.09598	5.04483
H	-1.12869	0.21859	5.29758
C	-0.37146	-1.75224	5.84161
H	-0.46399	-2.81637	5.53328
H	-0.67863	-1.68703	6.90693
H	0.70796	-1.49036	5.79079
C	7.15815	0.89298	0.84259
C	7.08341	-0.49867	1.03449
C	7.33987	1.41595	-0.45081
H	6.94309	-0.90837	2.04770
H	7.40130	2.50591	-0.60068
C	7.19076	-1.36725	-0.06705
C	7.44630	0.54736	-1.55233
H	7.13539	-2.45738	0.08374
H	7.58968	0.95703	-2.56517
C	7.37198	-0.84420	-1.36044
H	7.45766	-1.52465	-2.22294
H	7.07662	1.57318	1.70563
C	-0.83082	2.79556	-3.04329
H	-1.70796	3.46129	-2.90510
H	0.05375	3.39077	-2.73463
H	-0.73623	2.55013	-4.11726
C	-0.94634	-2.14353	-3.40915
H	-0.08120	-2.81083	-3.21526
H	-1.84726	-2.78865	-3.34814
H	-0.86304	-1.74521	-4.43721

TS (A^{Me}-B^{Me})
SCF (BP86) Energy = -1828.213280
Enthalpy 0K = -1827.387341
Enthalpy 298K = -1827.336631
Free Energy 298K = -1827.474862
Lowest Frequency = -1218.2532 cm^{-1}
Second Frequency = 8.7542 cm^{-1}
SCF (BP86-D3BJ) Energy = -1828.445334
SCF (C_6H_6) Energy = -1828.244599
SCF (BS2) Energy = -1830.037957

Mg	0.00023	0.40426	-0.12208
N	1.55435	-0.52824	-1.12341
N	-1.55409	-0.52731	-1.12401
C	1.29695	-1.07215	-2.33027
C	0.00026	-1.21884	-2.89488
H	0.00033	-1.63653	-3.91090
C	-1.29656	-1.07130	-2.33080
C	2.88193	-0.62550	-0.57359
C	3.23636	-1.77710	0.19825
C	2.26583	-2.93733	0.44050
H	1.31029	-2.68359	-0.06442
C	2.77815	-4.25905	-0.17331
H	2.98105	-4.16248	-1.25985

H	2.02967	-5.06872	-0.04086
H	3.71965	-4.59438	0.31176
C	1.96529	-3.11883	1.94370
H	2.88192	-3.38667	2.51133
H	1.23008	-3.93596	2.09989
H	1.55065	-2.19379	2.39307
C	4.52714	-1.84698	0.76137
H	4.80567	-2.73262	1.35556
C	5.46146	-0.81872	0.58290
H	6.46591	-0.89511	1.02880
C	5.10329	0.30939	-0.16539
H	5.83693	1.12024	-0.30347
C	3.82545	0.43412	-0.75083
C	3.50952	1.69810	-1.55509
H	2.44751	1.63033	-1.87289
C	4.37081	1.80953	-2.83290
H	5.45006	1.90176	-2.58634
H	4.08456	2.70738	-3.42051
H	4.25581	0.92544	-3.49294
C	3.66558	2.97126	-0.69589
H	3.05823	2.91776	0.22993
H	3.34246	3.86936	-1.26315
H	4.72286	3.13185	-0.39552
C	-2.88189	-0.62391	-0.57461
C	-3.23740	-1.77568	0.19648
C	-2.26784	-2.93680	0.43833
H	-1.31217	-2.68380	-0.06669
C	-2.78140	-4.25798	-0.17562
H	-3.72302	-4.59270	0.30963
H	-2.03351	-5.06826	-0.04357
H	-2.98454	-4.16101	-1.26209
C	-1.96722	-3.11883	1.94145
H	-1.55171	-2.19424	2.39093
H	-1.23273	-3.93667	2.09738
H	-2.88401	-3.38595	2.50916
C	-4.52836	-1.84483	0.75931
H	-4.80772	-2.73057	1.35295
C	-5.46180	-0.81572	0.58125
H	-6.46640	-0.89154	1.02693
C	-5.10259	0.31251	-0.16637
H	-5.83557	1.12401	-0.30418
C	-3.82454	0.43652	-0.75150
C	-3.50749	1.70057	-1.55523
H	-2.44538	1.63225	-1.87256
C	-4.36810	1.81279	-2.83343
H	-4.25322	0.92880	-3.49363
H	-4.08112	2.71064	-3.42066
H	-5.44743	1.90549	-2.58735
C	-3.66319	2.97360	-0.69578
H	-4.72051	3.13477	-0.39586
H	-3.33926	3.87164	-1.26266
H	-3.05630	2.91950	0.23031
C	0.00104	2.72652	-0.05812
H	0.89141	2.79610	-0.72859
H	-0.88922	2.79731	-0.72858
C	0.00189	3.93832	0.89151
H	-0.88027	3.88091	1.57285
H	0.88405	3.87975	1.57276
C	0.00275	5.31592	0.20336
H	-0.88359	5.38431	-0.46849
H	0.88912	5.38318	-0.46857
C	0.00355	6.49850	1.18115
H	-0.89043	6.47850	1.84139
H	0.00415	7.47434	0.65126
H	0.89756	6.47736	1.84131
C	1.21033	0.62385	2.88492
C	-0.00098	0.78716	2.16405
C	1.21607	0.28216	4.24980
H	-0.00008	1.71049	1.11537
H	2.17047	0.15494	4.78736
C	-1.21332	0.62383	2.88319
C	-0.00295	0.10856	4.93186
H	-2.18049	0.77123	2.36785
H	-0.00371	-0.15304	6.00270
C	-1.22100	0.28214	4.24806
H	-2.17615	0.15487	4.78425
H	2.17823	0.77129	2.37096
C	2.44724	-1.62211	-3.15698
H	3.26707	-0.88262	-3.24956
H	2.89147	-2.51024	-2.66152
H	2.11281	-1.91983	-4.16787
C	-2.44688	-1.62045	-3.15802
H	-2.89131	-2.50886	-2.66326
H	-3.26656	-0.88074	-3.25018
H	-2.11238	-1.91754	-4.16908

B^{Me}

SCF (BP86) Energy = -1828.275633
Enthalpy 0K = -1827.445599
Enthalpy 298K = -1827.393060
Free Energy 298K = -1827.550515
Lowest Frequency = 1.0539 cm^{-1}
Second Frequency = 2.4030 cm^{-1}
SCF (BP86-D3^{BJ}) Energy = -1828.489095
SCF (C_6H_6) Energy = -1828.311143
SCF (BS2) Energy = -1830.100709

Mg	1.29757	0.00034	0.26518
N	0.69194	1.51242	-0.99285
N	0.69294	-1.51309	-0.99166
C	0.11734	1.28930	-2.19095
C	-0.13846	-0.00129	-2.72540
H	-0.61356	-0.00183	-3.71604
C	0.11811	-1.29129	-2.18991
C	0.91250	2.85507	-0.53087
C	2.15815	3.49966	-0.80011
C	3.25203	2.82023	-1.62837
H	2.79851	1.92311	-2.09981
C	3.78274	3.71602	-2.76642
H	2.96386	4.07258	-3.42539
H	4.50704	3.15715	-3.39578
H	4.31144	4.61172	-2.37682
C	4.40599	2.33176	-0.72415
H	4.91120	3.18568	-0.22454
H	5.17102	1.78622	-1.31655
H	4.04523	1.65399	0.07869
C	2.38828	4.77804	-0.25141
H	3.34739	5.28400	-0.44791
C	1.42454	5.41658	0.54129
H	1.62516	6.41500	0.96132
C	0.20495	4.77513	0.79839
H	-0.54868	5.27874	1.42520
C	-0.07699	3.49662	0.27474
C	-1.40523	2.81346	0.61137
H	-1.49055	1.91938	-0.04145
C	-2.62770	3.70871	0.32280
H	-2.65381	4.60066	0.98410
H	-3.57017	3.14719	0.49448

H	-2.63387	4.07106	-0.72629
C	-1.41511	2.31759	2.07489
H	-0.56088	1.64038	2.28905
H	-2.35390	1.76910	2.30217
H	-1.34007	3.16814	2.78551
C	0.91428	-2.85524	-0.52860
C	2.16019	-3.49944	-0.79761
C	3.25355	-2.82003	-1.62658
H	2.79926	-1.92395	-2.09922
C	3.78538	-3.71671	-2.76340
H	4.31504	-4.61133	-2.37261
H	4.50913	-3.15785	-3.39339
H	2.96696	-4.07501	-3.42201
C	4.40685	-2.32931	-0.72274
H	4.04528	-1.65089	0.07919
H	5.17148	-1.78375	-1.31565
H	4.91275	-3.18212	-0.22194
C	2.39109	-4.77724	-0.24789
H	3.35041	-5.28289	-0.44418
C	1.42785	-5.41558	0.54559
H	1.62908	-6.41355	0.96641
C	0.20800	-4.77453	0.80244
H	-0.54523	-5.27799	1.42985
C	-0.07470	-3.49661	0.27776
C	-1.40323	-2.81386	0.61408
H	-1.48907	-1.92029	-0.03939
C	-2.62529	-3.70991	0.32630
H	-2.63143	-4.07298	-0.72255
H	-3.56802	-3.14875	0.49773
H	-2.65086	-4.60142	0.98821
C	-1.41318	-2.31693	2.07724
H	-1.33763	-3.16692	2.78846
H	-2.35223	-1.76876	2.30423
H	-0.55928	-1.63911	2.29081
C	-8.45422	-0.01946	-1.56184
H	-8.80053	0.87985	-2.11540
H	-8.82467	-0.90887	-2.11575
C	-8.95804	-0.01296	-0.11331
H	-8.56207	-0.90536	0.42396
H	-8.53816	0.86820	0.42434
C	-10.49034	0.00768	0.00866
H	-10.91026	-0.87344	-0.52899
H	-10.88634	0.90007	-0.52855
C	-10.99414	0.01412	1.45717
H	-10.64802	-0.88525	2.01071
H	-12.10285	0.02908	1.50689
H	-10.62387	0.90354	2.01115
C	2.54501	1.20792	2.85122
C	2.21729	0.00171	2.17235
C	3.15804	1.21696	4.11974
H	-7.34544	-0.03441	-1.61191
H	3.39575	2.17532	4.61205
C	2.54611	-1.20348	2.85249
C	3.46738	0.00364	4.75928
H	2.32099	-2.18051	2.38620
H	3.94714	0.00439	5.75205
C	3.15915	-1.21063	4.12101
H	3.39773	-2.16825	4.61434
H	2.31900	2.18425	2.38390
C	-0.29212	2.47786	-3.04260
H	-1.01134	3.12397	-2.49765
H	0.58366	3.12110	-3.26849
H	-0.75286	2.15794	-3.99538
C	-0.29069	-2.48079	-3.04055
H	0.58544	-3.12371	-3.26596
H	-1.00951	-3.12685	-2.49501
H	-0.75168	-2.16193	-3.99356

3^Me

SCF (BP86) Energy = -1669.946705
Enthalpy 0K = -1669.244492
Enthalpy 298K = -1669.200565
Free Energy 298K = -1669.324078
Lowest Frequency = 17.1203 cm^{-1}
Second Frequency = 18.4507 cm^{-1}
SCF (BP86-D3^{BJ}) Energy = -1670.147215
SCF (C_6H_6) Energy = -1669.978081
SCF (BS2) Energy = -1671.594202

Mg	-0.00007	0.28706	0.00053
N	1.51276	-1.10844	-0.00342
N	-1.51300	-1.10830	-0.00343
C	1.29016	-2.43725	-0.00697
C	-0.00021	-3.03026	-0.00856
H	-0.00026	-4.12893	-0.01161
C	-1.29053	-2.43714	-0.00699
C	2.85506	-0.59563	-0.00202
C	3.49818	-0.30156	1.23877
C	2.81761	-0.57864	2.58198
H	1.92198	-1.20138	2.37493
C	3.71380	-1.37481	3.55268
H	4.07248	-2.32194	3.09855
H	3.15446	-1.63112	4.47707
H	4.60819	-0.79369	3.86246
C	2.32573	0.73382	3.23267
H	3.17810	1.40387	3.47464
H	1.77896	0.52809	4.17741
H	1.64812	1.30172	2.56000
C	4.77604	0.29391	1.21151
H	5.28069	0.52989	2.16236
C	5.41551	0.59496	0.00100
H	6.41350	1.06134	0.00219
C	4.77548	0.30129	-1.21105
H	5.27966	0.54309	-2.16068
C	3.49760	-0.29394	-1.24130
C	2.81599	-0.56242	-2.58573
H	1.92122	-1.18749	-2.38209
C	3.71186	-1.35083	-3.56301
H	4.60520	-0.76661	-3.86997
H	3.15161	-1.60134	-4.48843
H	4.07212	-2.30068	-3.11588
C	2.32199	0.75410	-3.22653
H	1.64477	1.31663	-2.54895
H	1.77412	0.55454	-4.17197
H	3.17340	1.42667	-3.46482
C	-2.85527	-0.59538	-0.00202
C	-3.49841	-0.30140	1.23878
C	-2.81783	-0.57862	2.58196
H	-1.92251	-1.20180	2.37491
C	-3.71425	-1.37423	3.55291
H	-4.60829	-0.79263	3.86280
H	-3.15487	-1.63070	4.47722
H	-4.07348	-2.32123	3.09895
C	-2.32528	0.73373	3.23238
H	-1.64749	1.30122	2.55954
H	-1.77849	0.52792	4.17709
H	-3.17733	1.40420	3.47431
C	-4.77623	0.29415	1.21155

H	-5.28089	0.53006	2.16240
C	-5.41562	0.59539	0.00104
H	-6.41359	1.06184	0.00225
C	-4.77558	0.30182	-1.21101
H	-5.27971	0.54376	-2.16064
C	-3.49773	-0.29350	-1.24129
C	-2.81613	-0.56194	-2.58573
H	-1.92134	-1.18699	-2.38211
C	-3.71199	-1.35036	-3.56300
H	-4.07220	-2.30024	-3.11588
H	-3.15176	-1.60083	-4.48845
H	-4.60537	-0.76617	-3.86991
C	-2.32218	0.75460	-3.22653
H	-3.17362	1.42716	-3.46479
H	-1.77433	0.55507	-4.17198
H	-1.64495	1.31715	-2.54895
C	1.20609	3.15891	0.00993
C	0.00026	2.40446	0.00740
C	1.21444	4.56779	0.01483
H	2.17253	5.11496	0.01667
C	-1.20530	3.15935	0.01005
C	0.00078	5.27764	0.01737
H	-2.18208	2.64112	0.00825
H	0.00098	6.38025	0.02120
C	-1.21313	4.56823	0.01495
H	-2.17103	5.11574	0.01689
H	2.18268	2.64033	0.00804
C	2.47899	-3.38189	-0.00932
H	3.12467	-3.20080	-0.89362
H	3.12256	-3.20755	0.87789
H	2.15932	-4.44029	-0.01362
C	-2.47944	-3.38166	-0.00942
H	-3.12303	-3.20731	0.87778
H	-3.12507	-3.20047	-0.89373
H	-2.15987	-4.44009	-0.01376

[1^{Me}]$_2$

SCF (BP86) Energy = -3192.357739
Enthalpy 0K = -3190.891633
Enthalpy 298K = -3190.802237
Free Energy 298K = -3191.018615
Lowest Frequency = 10.7901 cm^{-1}
Second Frequency = 16.9021 cm^{-1}
SCF (BP86-D3^{BJ}) Energy = -3192.828168
SCF (C_6H_6) Energy = -3192.398343
SCF (BS2) Energy = -3195.503596

N	0.99540	-1.53671	2.73954
C	0.96647	-2.44236	5.06298
H	0.09956	-3.12710	4.96569
H	0.99069	-2.06275	6.10099
H	1.86867	-3.06249	4.89113
C	0.87393	-1.29551	4.06285
C	1.43525	-2.86184	2.37911
C	0.51484	-3.95202	2.30067
C	1.00888	-5.23822	1.99846
H	0.30369	-6.08221	1.93659
C	2.37457	-5.46877	1.79271
H	2.74037	-6.48426	1.57173
C	3.27162	-4.39540	1.86904
H	4.34583	-4.57845	1.70970
C	2.83067	-3.08702	2.15587
C	-0.98443	-3.77326	2.54680
H	-1.12356	-2.78237	3.02711
C	-1.76228	-3.75141	1.21810
H	-1.40900	-2.94112	0.54895
H	-2.84748	-3.59777	1.39630
H	-1.64352	-4.70487	0.66412
C	-1.57603	-4.83763	3.49514
H	-1.58668	-5.84626	3.03057
H	-2.62780	-4.58645	3.74718
H	-1.00759	-4.91642	4.44534
C	3.85773	-1.96009	2.29474
H	3.30593	-1.00473	2.16378
C	4.47907	-1.93525	3.70991
H	3.71543	-1.77245	4.49595
H	5.22639	-1.11800	3.79644
H	4.99794	-2.89278	3.93113
C	4.96505	-2.01351	1.22556
H	5.62902	-2.89468	1.35411
H	5.61113	-1.11349	1.29640
H	4.54371	-2.04864	0.20108
Mg	0.46469	0.03553	1.36522
N	0.94140	1.58715	2.79331
C	1.02683	2.38652	5.15115
H	0.25431	3.17143	5.04183
H	0.96935	1.97411	6.17508
H	2.00055	2.90451	5.03536
C	0.86858	1.28787	4.10807
C	0.70006	-0.01446	4.64451
H	0.58794	-0.03369	5.73666
C	1.30819	2.94736	2.48088
C	0.32667	3.98759	2.44889
C	0.74855	5.31096	2.20097
H	-0.00353	6.11627	2.17891
C	2.09738	5.62737	1.99616
H	2.40424	6.67044	1.81802
C	3.05157	4.60194	2.01677
H	4.11323	4.84872	1.85395
C	2.68536	3.26067	2.25139
C	-1.16631	3.73081	2.67757
H	-1.28492	2.65467	2.92180
C	-1.97613	4.02145	1.39938
H	-1.58598	3.46935	0.52228
H	-3.04226	3.74221	1.53385
H	-1.94613	5.10060	1.14265
C	-1.75161	4.55227	3.84873
H	-1.72376	5.64163	3.63371
H	-2.81438	4.27865	4.01795
H	-1.20892	4.39054	4.80244
C	3.78226	2.19326	2.30626
H	3.27448	1.20563	2.32531
C	4.61939	2.30233	3.60039
H	3.99408	2.18992	4.50874
H	5.40069	1.51351	3.63192
H	5.13237	3.28609	3.66323
C	4.70148	2.22742	1.06877
H	5.29633	3.16421	1.01946
H	5.42332	1.38439	1.09725
H	4.12192	2.14813	0.12711
C	-1.70801	0.24227	0.62029
H	-2.37869	-0.28317	-0.11212
H	-1.91646	1.32447	0.43236
C	-2.28788	-0.10964	2.01841
H	-2.21410	-1.20614	2.18802
H	-1.67168	0.34334	2.83434
C	-3.74901	0.31746	2.26378
H	-4.39080	-0.12797	1.47053

H	-3.82863	1.41995	2.12871
C	-4.28345	-0.07394	3.64773
H	-3.67262	0.37609	4.45977
H	-5.33235	0.25883	3.79493
H	-4.26038	-1.17542	3.79428
N	-0.94140	-1.58715	-2.79331
C	-1.02683	-2.38652	-5.15115
H	-0.25431	-3.17143	-5.04183
H	-0.96935	-1.97411	-6.17508
H	-2.00055	-2.90451	-5.03536
C	-0.86858	-1.28787	-4.10807
C	-1.30819	-2.94736	-2.48088
C	-0.32667	-3.98759	-2.44889
C	-0.74855	-5.31096	-2.20097
H	0.00353	-6.11627	-2.17891
C	-2.09738	-5.62737	-1.99616
H	-2.40424	-6.67044	-1.81802
C	-3.05157	-4.60194	-2.01677
H	-4.11323	-4.84872	-1.85395
C	-2.68536	-3.26067	-2.25139
C	1.16631	-3.73081	-2.67757
H	1.28492	-2.65467	-2.92180
C	1.97613	-4.02145	-1.39938
H	1.58598	-3.46935	-0.52228
H	3.04226	-3.74221	-1.53385
H	1.94613	-5.10060	-1.14265
C	1.75161	-4.55227	-3.84873
H	1.72376	-5.64163	-3.63371
H	2.81438	-4.27865	-4.01795
H	1.20892	-4.39054	-4.80244
C	-3.78226	-2.19326	-2.30626
H	-3.27448	-1.20563	-2.32531
C	-4.61939	-2.30233	-3.60039
H	-3.99408	-2.18992	-4.50874
H	-5.40069	-1.51351	-3.63192
H	-5.13237	-3.28609	-3.66323
C	-4.70148	-2.22742	-1.06877
H	-5.29633	-3.16421	-1.01946
H	-5.42332	-1.38439	-1.09725
H	-4.12192	-2.14813	-0.12711
C	1.70801	-0.24227	-0.62029
H	2.37869	0.28317	0.11212
H	1.91646	-1.32447	-0.43236
C	2.28788	0.10964	-2.01841
H	2.21410	1.20614	-2.18802
H	1.67168	-0.34334	-2.83434
C	3.74901	-0.31746	-2.26378
H	4.39080	0.12797	-1.47053
H	3.82863	-1.41995	-2.12871
C	4.28345	0.07394	-3.64773
H	3.67262	-0.37609	-4.45977
H	5.33235	-0.25883	-3.79493
H	4.26038	1.17542	-3.79428
Mg	-0.46469	-0.03553	-1.36522
N	-0.99540	1.53671	-2.73954
C	-0.96647	2.44236	-5.06298
H	-0.09956	3.12710	-4.96569
H	-0.99069	2.06275	-6.10099
H	-1.86867	3.06249	-4.89113
C	-0.87393	1.29551	-4.06285
C	-0.70006	0.01446	-4.64451
H	-0.58794	0.03369	-5.73666
C	-1.43525	2.86184	-2.37911
C	-0.51484	3.95202	-2.30067

C	-1.00888	5.23822	-1.99846
H	-0.30369	6.08221	-1.93659
C	-2.37457	5.46877	-1.79271
H	-2.74037	6.48426	-1.57173
C	-3.27162	4.39540	-1.86904
H	-4.34583	4.57845	-1.70970
C	-2.83067	3.08702	-2.15587
C	0.98443	3.77326	-2.54680
H	1.12356	2.78237	-3.02711
C	1.76228	3.75141	-1.21810
H	1.40900	2.94112	-0.54895
H	2.84748	3.59777	-1.39630
H	1.64352	4.70487	-0.66412
C	1.57603	4.83763	-3.49514
H	1.58668	5.84626	-3.03057
H	2.62780	4.58645	-3.74718
H	1.00759	4.91642	-4.44534
C	-3.85773	1.96009	-2.29474
H	-3.30593	1.00473	-2.16378
C	-4.47907	1.93525	-3.70991
H	-3.71543	1.77245	-4.49595
H	-5.22639	1.11800	-3.79644
H	-4.99794	2.89278	-3.93113
C	-4.96505	2.01351	-1.22556
H	-5.62902	2.89468	-1.35411
H	-5.61113	1.11349	-1.29640
H	-4.54371	2.04864	-0.20108

TS([1^{Me}]$_2$-1^{Me})

SCF (BP86) Energy = -3192.349776
Enthalpy 0K = -3190.885900
Enthalpy 298K = -3190.796529
Free Energy 298K = -3191.018563
Lowest Frequency = -22.2878 cm^{-1}
Second Frequency = 7.3954 cm^{-1}
SCF (BP86-D3^{BJ}) Energy = -3192.792750
SCF (C_6H_6) Energy = -3192.400321
SCF (BS2) Energy = -3195.497963

N	3.33301	1.55394	-1.01478
C	5.22528	2.49548	-2.31904
H	4.63940	3.06814	-3.06748
H	6.16093	2.15375	-2.79882
H	5.47644	3.21279	-1.51269
C	4.41257	1.32248	-1.79137
C	3.09800	2.89861	-0.55888
C	2.36116	3.82966	-1.35258
C	2.19208	5.14440	-0.86880
H	1.62910	5.87064	-1.47707
C	2.72566	5.54741	0.36126
H	2.59315	6.58326	0.71229
C	3.41781	4.61750	1.14945
H	3.82069	4.93165	2.12499
C	3.60851	3.28851	0.72007
C	1.73460	3.45528	-2.69848
H	2.04200	2.41317	-2.93044
C	0.19359	3.48515	-2.61623
H	-0.18857	2.81867	-1.81785
H	-0.25894	3.16424	-3.57831
H	-0.18158	4.50578	-2.39418
C	2.21908	4.35675	-3.85543
H	1.88160	5.40650	-3.72239
H	1.80970	4.00011	-4.82409
H	3.32518	4.37461	-3.94156

C	4.37794	2.30640	1.60869
H	4.08481	1.28367	1.28466
C	5.90594	2.42197	1.40823
H	6.20675	2.20238	0.36490
H	6.44360	1.70820	2.06799
H	6.26062	3.44539	1.65689
C	4.02301	2.44446	3.10168
H	4.40413	3.39329	3.53561
H	4.47972	1.61766	3.68454
H	2.92685	2.41314	3.26619
Mg	2.00553	-0.04127	-0.71454
N	3.46538	-1.53814	-0.92223
C	5.40772	-2.39483	-2.21707
H	4.90297	-2.94160	-3.04169
H	6.36921	-2.00961	-2.60463
H	5.60969	-3.14104	-1.42448
C	4.51621	-1.26437	-1.72331
C	4.87419	0.03467	-2.17304
H	5.73660	0.05117	-2.85344
C	3.34901	-2.85931	-0.36245
C	2.67899	-3.90824	-1.06236
C	2.60757	-5.18341	-0.46162
H	2.09767	-5.99998	-0.99822
C	3.17027	-5.43510	0.79491
H	3.10831	-6.44045	1.24111
C	3.80659	-4.39401	1.48535
H	4.23997	-4.59231	2.47780
C	3.90661	-3.10054	0.93456
C	2.03156	-3.70935	-2.43527
H	2.23923	-2.66486	-2.75208
C	0.49978	-3.88404	-2.36280
H	0.03994	-3.19690	-1.62535
H	0.03244	-3.68754	-3.35050
H	0.22449	-4.91726	-2.06437
C	2.61961	-4.65285	-3.50803
H	2.38051	-5.71514	-3.28863
H	2.19520	-4.41970	-4.50736
H	3.72362	-4.57222	-3.57927
C	4.64014	-1.99846	1.70546
H	4.23406	-1.02872	1.34183
C	6.15545	-1.99596	1.39935
H	6.36555	-1.82198	0.32614
H	6.66922	-1.19602	1.97371
H	6.61560	-2.96654	1.68426
C	4.41004	-2.06327	3.22675
H	4.90617	-2.94411	3.68698
H	4.83581	-1.16335	3.71756
H	3.33160	-2.10804	3.47883
C	-0.02887	-0.17967	-1.43079
H	-0.61063	0.72423	-1.10418
H	-0.49504	-1.08797	-0.96660
C	-0.10401	-0.29483	-2.96775
H	0.25495	0.65699	-3.42340
H	0.61869	-1.06796	-3.32148
C	-1.48132	-0.63540	-3.56300
H	-2.22197	0.11746	-3.21122
H	-1.81667	-1.60837	-3.13757
C	-1.49882	-0.70396	-5.09551
H	-0.78444	-1.46672	-5.47451
H	-2.50370	-0.96666	-5.48782
H	-1.20921	0.26894	-5.54829
N	-3.23036	1.60268	0.88079
C	-5.06101	2.58765	2.24447
H	-4.47043	3.18278	2.97188
H	-5.98925	2.25583	2.74566
H	-5.32490	3.27893	1.41985
C	-4.24867	1.40232	1.74203
C	-3.04171	2.91344	0.32399
C	-2.30676	3.91738	1.02661
C	-2.16459	5.19144	0.43677
H	-1.60231	5.97100	0.97592
C	-2.72244	5.48743	-0.81245
H	-2.60961	6.49289	-1.24869
C	-3.41804	4.48895	-1.50905
H	-3.84358	4.72053	-2.49756
C	-3.58442	3.19691	-0.97054
C	-1.64832	3.65959	2.38383
H	-1.94979	2.64278	2.71283
C	-0.11050	3.67183	2.25990
H	0.24408	2.94774	1.49992
H	0.36465	3.41034	3.22894
H	0.26439	4.67239	1.95980
C	-2.09915	4.66140	3.46930
H	-1.75478	5.69254	3.24127
H	-1.67189	4.38359	4.45592
H	-3.20298	4.69917	3.57579
C	-4.36103	2.13510	-1.75380
H	-3.95256	1.14908	-1.43878
C	-5.86419	2.13463	-1.39416
H	-6.03643	1.90974	-0.32363
H	-6.40823	1.36862	-1.98691
H	-6.32260	3.12243	-1.61583
C	-4.18485	2.25461	-3.27949
H	-4.70917	3.14353	-3.69092
H	-4.61447	1.36575	-3.78619
H	-3.11668	2.32962	-3.56827
C	-0.02169	-0.08672	1.92816
H	0.50868	-1.04095	1.67915
H	0.67342	0.74103	1.62362
C	-0.23907	-0.01497	3.45465
H	-0.99862	-0.77453	3.76214
H	-0.70427	0.96435	3.71857
C	1.00329	-0.21180	4.34438
H	1.44468	-1.21012	4.12181
H	1.78082	0.52845	4.04807
C	0.72234	-0.09675	5.84846
H	0.31786	0.90551	6.10888
H	1.63774	-0.25399	6.45726
H	-0.02941	-0.84656	6.17808
Mg	-1.85522	-0.00320	0.78112
N	-3.35744	-1.48844	0.93016
C	-5.14148	-2.28900	2.48098
H	-4.69413	-2.52776	3.47033
H	-6.18333	-1.96328	2.66672
H	-5.15214	-3.22223	1.88825
C	-4.32116	-1.18636	1.82165
C	-4.64241	0.13214	2.24526
H	-5.43430	0.17659	3.00656
C	-3.29579	-2.80453	0.35836
C	-2.45374	-3.81277	0.91889
C	-2.40411	-5.07906	0.29757
H	-1.76180	-5.86280	0.73111
C	-3.15699	-5.36309	-0.84807
H	-3.10703	-6.36001	-1.31451
C	-3.97644	-4.36640	-1.39685
H	-4.56731	-4.59192	-2.29844
C	-4.06359	-3.08340	-0.81951
C	-1.63449	-3.58181	2.19005

H	-1.74173	-2.51039	2.46177
C	-0.13425	-3.86198	1.97470
H	0.27572	-3.29095	1.11780
H	0.44903	-3.58404	2.87697
H	0.05981	-4.93692	1.77635
C	-2.16627	-4.42194	3.37338
H	-2.04923	-5.50928	3.17660
H	-1.60496	-4.18907	4.30314
H	-3.24130	-4.23464	3.56705
C	-5.00268	-2.03810	-1.43044
H	-4.63018	-1.04378	-1.10185
C	-6.44665	-2.18943	-0.89888
H	-6.50146	-2.08728	0.20160
H	-7.11012	-1.41612	-1.34123
H	-6.86121	-3.18534	-1.16649
C	-5.01565	-2.05385	-2.97146
H	-5.49527	-2.97099	-3.37511
H	-5.59994	-1.19249	-3.35803
H	-3.99340	-1.99102	-3.39372

2(1^{Me})

SCF (BP86) Energy = -3192.379648
Enthalpy 0K = -3190.916213
Enthalpy 298K = -3190.824445
Free Energy 298K = -3191.070161
Lowest Frequency = 1.1640 cm^{-1}
Second Frequency = 2.6539 cm^{-1}
SCF (BP86-D3^{BJ}) Energy = -3192.764681
SCF (C_6H_6) Energy = -3192.437072
SCF (BS2) Energy = -3195.531000

N	6.44626	1.39167	0.87570
C	7.98598	2.21945	2.62306
H	8.59933	2.83526	1.93317
H	8.63768	1.83661	3.43004
H	7.23892	2.91099	3.06493
C	7.29832	1.09002	1.87550
C	6.21866	2.76364	0.51452
C	7.04530	3.38397	-0.47186
C	6.74257	4.70010	-0.87683
H	7.37092	5.18656	-1.64063
C	5.65823	5.40119	-0.33142
H	5.43811	6.42833	-0.66325
C	4.85564	4.78511	0.63820
H	4.00319	5.33830	1.06514
C	5.11429	3.47102	1.07966
C	8.22161	2.64619	-1.11747
H	8.36718	1.70045	-0.55477
C	7.90232	2.26578	-2.57992
H	6.97234	1.66441	-2.65455
H	8.73052	1.67600	-3.02735
H	7.75492	3.17073	-3.20716
C	9.54157	3.44038	-1.03180
H	9.49854	4.37926	-1.62368
H	10.38523	2.83903	-1.43161
H	9.78825	3.71776	0.01416
C	4.19821	2.83230	2.12680
H	4.65383	1.86084	2.41315
C	4.08583	3.68121	3.41050
H	5.08192	3.89502	3.85085
H	3.48168	3.15152	4.17704
H	3.59030	4.65647	3.21800
C	2.80225	2.53282	1.53759
H	2.28602	3.46767	1.23179
H	2.15774	2.02113	2.28345
H	2.86898	1.88443	0.63882
Mg	5.39477	-0.03771	-0.19576
N	6.25556	-1.62987	0.81210
C	7.70860	-2.72488	2.48478
H	8.21626	-3.37868	1.74532
H	8.42855	-2.46524	3.28280
H	6.89495	-3.33850	2.92434
C	7.14654	-1.48466	1.81160
C	7.60803	-0.23096	2.29258
H	8.32620	-0.29369	3.12167
C	5.84948	-2.94316	0.39568
C	6.54422	-3.59139	-0.67033
C	6.07188	-4.83973	-1.12410
H	6.59695	-5.34828	-1.94852
C	4.94628	-5.44612	-0.54904
H	4.59295	-6.42193	-0.91859
C	4.27261	-4.80220	0.49768
H	3.38629	-5.28071	0.94473
C	4.70333	-3.55273	0.98944
C	7.74704	-2.93263	-1.35064
H	8.10759	-2.12924	-0.67431
C	7.32069	-2.25904	-2.67457
H	6.49742	-1.52844	-2.51907
H	8.17244	-1.72222	-3.14376
H	6.94895	-3.01175	-3.40199
C	8.92430	-3.90106	-1.57876
H	8.67534	-4.69504	-2.31440
H	9.80457	-3.35412	-1.97736
H	9.23194	-4.40168	-0.63722
C	3.91581	-2.86015	2.10459
H	4.53987	-2.02010	2.47559
C	3.63086	-3.78431	3.30567
H	4.56235	-4.23237	3.71009
H	3.14226	-3.21645	4.12521
H	2.94858	-4.61816	3.03599
C	2.60740	-2.25118	1.55263
H	1.92990	-3.04363	1.16924
H	2.06255	-1.69274	2.34307
H	2.80166	-1.55425	0.70998
C	3.87812	-0.02981	-1.69902
H	2.91170	0.23310	-1.20059
H	3.73248	-1.08933	-2.02312
C	4.03755	0.86206	-2.94735
H	4.18007	1.92707	-2.64688
H	4.97119	0.59224	-3.49788
C	2.86299	0.79421	-3.94482
H	1.92477	1.07100	-3.41059
H	2.72221	-0.26522	-4.26138
C	3.03701	1.68569	-5.18168
H	3.94720	1.40772	-5.75660
H	2.17191	1.61045	-5.87423
H	3.14424	2.75531	-4.89814
N	-6.47263	1.62315	-0.63851
C	-8.05156	2.68702	-2.21476
H	-8.65369	3.17606	-1.42084
H	-8.71993	2.42140	-3.05457
H	-7.32870	3.45376	-2.56306
C	-7.32640	1.46900	-1.66884
C	-6.26348	2.92439	-0.06746
C	-7.05986	3.34354	1.04157
C	-6.77877	4.58676	1.64361
H	-7.38379	4.91988	2.50202
C	-5.74333	5.40690	1.17357

H	-5.53957	6.37486	1.65848
C	-4.96817	4.98641	0.08443
H	-4.15292	5.63234	-0.27969
C	-5.20755	3.75218	-0.55445
C	-8.16401	2.44755	1.60888
H	-8.40463	1.69089	0.83271
C	-7.65610	1.68228	2.85174
H	-6.73728	1.09732	2.62931
H	-8.42698	0.97916	3.23271
H	-7.39820	2.38456	3.67280
C	-9.46653	3.20869	1.92473
H	-9.33642	3.93233	2.75721
H	-10.26421	2.50041	2.23280
H	-9.83480	3.77447	1.04379
C	-4.31224	3.30894	-1.71429
H	-4.79797	2.42978	-2.18752
C	-4.15944	4.38931	-2.80400
H	-5.14429	4.73190	-3.18432
H	-3.58081	3.99361	-3.66513
H	-3.61660	5.28313	-2.42979
C	-2.93219	2.84715	-1.19471
H	-2.38675	3.68599	-0.71213
H	-2.30066	2.46548	-2.02487
H	-3.02723	2.04186	-0.43592
C	-3.90139	0.09381	1.72238
H	-2.90874	-0.00024	1.21511
H	-3.88746	1.12592	2.15037
C	-3.95511	-0.92910	2.87558
H	-3.97443	-1.96884	2.47123
H	-4.91304	-0.82246	3.43998
C	-2.79488	-0.82712	3.88676
H	-1.83109	-0.95236	3.33974
H	-2.76817	0.20801	4.29906
C	-2.87222	-1.84195	5.03516
H	-3.80662	-1.71620	5.62451
H	-2.01984	-1.73927	5.73982
H	-2.86491	-2.88633	4.65408
Mg	-5.40228	0.06204	0.20357
N	-6.24148	-1.37074	-1.03666
C	-7.63913	-2.20159	-2.89856
H	-8.34051	-1.82546	-3.66606
H	-6.80116	-2.72305	-3.40610
H	-8.15542	-2.97459	-2.29266
C	-7.11914	-1.07909	-2.01709
C	-7.60476	0.22549	-2.29507
H	-8.31827	0.28120	-3.12866
C	-5.82923	-2.73056	-0.82241
C	-6.56126	-3.55865	0.08274
C	-6.08314	-4.85851	0.34697
H	-6.63799	-5.50447	1.04685
C	-4.91682	-5.34502	-0.25921
H	-4.55976	-6.36336	-0.03773
C	-4.20767	-4.52588	-1.14826
H	-3.29021	-4.91054	-1.62267
C	-4.64184	-3.21831	-1.44862
C	-7.82581	-3.06101	0.78854
H	-8.09051	-2.08113	0.33896
C	-7.56599	-2.82244	2.29222
H	-6.72260	-2.12015	2.45762
H	-8.46631	-2.40256	2.78933
H	-7.30597	-3.77019	2.81019
C	-9.03062	-4.00260	0.58193
H	-8.86619	-4.99375	1.05554
H	-9.94689	-3.56958	1.03608
H	-9.23618	-4.17853	-0.49445
C	-3.82150	-2.35098	-2.40728
H	-4.41527	-1.43514	-2.61177
C	-3.56559	-3.04251	-3.76254
H	-4.51177	-3.36309	-4.24618
H	-3.04374	-2.35310	-4.45924
H	-2.92535	-3.94338	-3.65245
C	-2.49413	-1.90496	-1.75454
H	-1.84577	-2.77766	-1.52675
H	-1.92558	-1.23044	-2.42925
H	-2.66938	-1.36755	-0.79881

C_7H_8

SCF (BP86) Energy = -271.362925
Enthalpy 0K = -271.238965
Enthalpy 298K = -271.231609
Free Energy 298K = -271.270554
Lowest Frequency = 14.4317 cm^{-1}
Second Frequency = 205.6771 cm^{-1}
SCF (BP86-D3^{BJ}) Energy = -271.384695
SCF (C_6H_6) Energy = -271.371253
SCF ($C_6H_5CH_3$) Energy = -271.371067
SCF (BS2) Energy = -271.656055

C	-1.21419	1.21098	0.00095
C	0.19218	1.21334	-0.00383
C	-1.91812	-0.00480	0.00357
H	0.73579	2.17301	-0.00749
H	-3.01980	-0.00950	0.00608
C	0.92398	0.00627	-0.00533
C	-1.20351	-1.21663	0.00095
H	-1.74517	-2.17642	0.00094
C	0.20067	-1.20859	-0.00386
H	0.75047	-2.16501	-0.00755
H	-1.76248	2.16693	0.00090
C	2.43727	0.00313	0.00384
H	2.84966	1.02283	-0.13206
H	2.83396	-0.39320	0.96398
H	2.84782	-0.64087	-0.80253

C^{Me}

SCF (BP86) Energy = -2567.400913
Enthalpy 0K = -2566.166660
Enthalpy 298K = -2566.100824
Free Energy 298K = -2566.277764
Lowest Frequency = 3.9199 cm^{-1}
Second Frequency = 4.4798 cm^{-1}
SCF (BP86-D3^{BJ}) Energy = -2567.751874
SCF (C_6H_6) Energy = -2567.446185
SCF ($C_6H_5CH_3$) Energy = -2567.445010
SCF (BS2) Energy = -2569.995530

Mg	0.52772	-1.25363	0.94794
N	1.86129	-1.06245	-0.65205
N	-0.48067	0.54555	0.45195
C	2.54579	-0.01542	-0.10444
C	4.09466	-0.04523	0.16492
C	4.82744	-1.24897	-0.48922
H	4.33093	-2.19612	-0.19155
H	4.75331	-1.19665	-1.59260
C	4.26384	-0.20376	1.71833
H	3.75644	0.63407	2.24117
H	3.75281	-1.13543	2.04708
C	4.83196	1.26107	-0.25930

H	4.74500	1.40379	-1.35662
H	4.35606	2.14617	0.21450
C	6.31986	-1.29126	-0.08273
H	6.79622	-2.16120	-0.58585
C	6.44559	-1.44187	1.44736
H	7.51662	-1.49095	1.74571
H	5.97461	-2.39328	1.78010
C	5.75406	-0.24080	2.12496
H	5.81710	-0.34247	3.23110
C	6.43919	1.07113	1.68175
H	7.50885	1.06876	1.98814
H	5.96364	1.94207	2.18601
C	6.32469	1.21486	0.14772
H	6.81249	2.16070	-0.17692
C	7.01398	0.01258	-0.53317
H	6.95942	0.11453	-1.63984
H	8.09426	-0.01243	-0.26714
C	1.85826	1.08355	0.47435
H	2.52541	1.83778	0.90869
C	0.46360	1.48862	0.44361
C	0.24909	3.03591	0.45676
C	1.07634	3.67485	-0.70822
H	2.13922	3.36626	-0.63433
H	0.70175	3.28033	-1.67858
C	0.77634	3.62197	1.81093
H	0.18715	3.19397	2.65103
H	1.83217	3.32390	1.98082
C	-1.22141	3.51105	0.29114
H	-1.64107	3.12380	-0.65865
H	-1.85561	3.09965	1.09951
C	0.98006	5.21687	-0.68428
H	1.59138	5.62542	-1.51917
C	1.52348	5.74396	0.66332
H	2.59161	5.45609	0.78557
H	1.48763	6.85574	0.68293
C	0.67890	5.16541	1.82135
H	1.07097	5.53852	2.79339
C	-0.79281	5.59728	1.65046
H	-1.40813	5.20717	2.49140
H	-0.87527	6.70654	1.68116
C	-1.32171	5.05612	0.30544
H	-2.39069	5.33706	0.18305
C	-0.49180	5.64791	-0.85277
H	-0.57279	6.75776	-0.85934
H	-0.88630	5.29124	-1.83014
C	2.20995	-1.84694	-1.76908
C	2.65054	-1.27801	-3.01592
C	2.79918	0.22997	-3.25157
H	2.77367	0.74093	-2.26763
C	4.12859	0.61221	-3.93693
H	5.00812	0.19477	-3.40560
H	4.24401	1.71616	-3.97495
H	4.17162	0.24699	-4.98479
C	1.61013	0.77555	-4.07095
H	1.57642	0.31335	-5.08072
H	1.68883	1.87603	-4.20421
H	0.64717	0.55797	-3.57044
C	2.88360	-2.13359	-4.11290
H	3.21832	-1.69259	-5.06584
C	2.69672	-3.51845	-4.03551
H	2.89563	-4.16139	-4.90719
C	2.24200	-4.07016	-2.82949
H	2.08279	-5.15735	-2.76214
C	1.98849	-3.26762	-1.70351
C	1.42944	-3.90168	-0.43102
H	1.81277	-3.30867	0.43272
C	1.87930	-5.35253	-0.18167
H	1.45860	-6.05623	-0.93069
H	1.53370	-5.69573	0.81546
H	2.98419	-5.44497	-0.21179
C	-0.11663	-3.81455	-0.42785
H	-0.48714	-2.78919	-0.66638
H	-0.55051	-4.13661	0.54146
H	-0.53969	-4.45882	-1.22706
C	-1.90662	0.65056	0.37399
C	-2.56132	0.59088	-0.89610
C	-1.78799	0.50660	-2.21283
H	-0.71068	0.61978	-1.97056
C	-2.17351	1.62769	-3.20098
H	-3.23169	1.54179	-3.52683
H	-1.54386	1.57804	-4.11403
H	-2.04370	2.63512	-2.75427
C	-1.96609	-0.87852	-2.87232
H	-1.65866	-1.69693	-2.19043
H	-1.35197	-0.96198	-3.79345
H	-3.02578	-1.05749	-3.15332
C	-3.97054	0.58322	-0.93531
H	-4.47804	0.54712	-1.91284
C	-4.74063	0.61882	0.23442
H	-5.84061	0.61881	0.17858
C	-4.08982	0.64361	1.47488
H	-4.69090	0.65864	2.39871
C	-2.68363	0.64872	1.57660
C	-2.05323	0.64263	2.97275
H	-0.94990	0.65293	2.84517
C	-2.43859	1.88780	3.80105
H	-2.17827	2.83167	3.27988
H	-1.91326	1.88375	4.77942
H	-3.52905	1.91505	4.01063
C	-2.41889	-0.64407	3.74298
H	-3.51241	-0.70577	3.92798
H	-1.90924	-0.67136	4.72888
H	-2.11792	-1.55290	3.18417
C	0.78698	-2.14752	2.88017
H	1.89615	-2.19242	3.02123
H	0.45617	-1.38671	3.62828
C	0.19917	-3.51484	3.27539
H	-0.90976	-3.52020	3.14147
H	0.57257	-4.30850	2.58505
C	0.50665	-3.96200	4.71924
H	0.12372	-3.18729	5.42289
H	1.61186	-3.97073	4.86308
C	-0.07572	-5.33211	5.09100
H	-1.18296	-5.34285	4.99076
H	0.16526	-5.61658	6.13710
H	0.31968	-6.13401	4.43002
C	-8.72149	-1.72733	-1.93636
C	-8.29759	-1.90782	-0.60154
C	-9.68769	-0.76108	-2.26821
H	-10.00339	-0.64191	-3.31733
C	-8.87817	-1.08952	0.39490
C	-10.25249	0.04666	-1.26665
H	-8.56963	-1.21805	1.44613
H	-11.01156	0.80251	-1.52381
C	-9.84352	-0.12240	0.06850
H	-10.28351	0.50131	0.86349
H	-8.28709	-2.35858	-2.72961
C	-7.23825	-2.92735	-0.24477

H	-6.25669	-2.43614	-0.06473
H	-7.49795	-3.47895	0.68268
H	-7.09371	-3.66893	-1.05589

TS (C^{Me}–D^{Me})

SCF (BP86) Energy = -2567.351464
Enthalpy 0K = -2566.119549
Enthalpy 298K = -2566.055859
Free Energy 298K = -2566.216709
Lowest Frequency = -1370.2162 cm^{-1}
Second Frequency = 13.5257 cm^{-1}
SCF (BP86-D3^{BJ}) Energy = -2567.729978
SCF (C_6H_6) Energy = -2567.393245
SCF ($C_6H_5CH_3$) Energy = -2567.392208
SCF (BS2) Energy = -2569.943329

Mg	0.85277	-0.98835	0.37106
N	1.08623	0.95357	-0.29114
N	-1.17295	-1.28500	-0.03268
C	0.02450	1.67711	0.16557
C	0.09827	3.19819	0.59938
C	1.41354	3.94899	0.24062
H	2.29046	3.39335	0.62102
H	1.53715	4.00365	-0.85777
C	-0.02683	3.21081	2.16567
H	-0.96109	2.69689	2.47491
H	0.81054	2.62586	2.60550
C	-1.07726	4.05244	0.02336
H	-1.00934	4.07792	-1.08481
H	-2.05767	3.59350	0.26625
C	1.43074	5.38654	0.81569
H	2.39559	5.86055	0.53092
C	1.30564	5.34577	2.35249
H	1.33371	6.37646	2.77127
H	2.16545	4.79555	2.79461
C	-0.01987	4.65093	2.72599
H	-0.12243	4.60061	3.83281
C	-1.20568	5.44082	2.12845
H	-1.22960	6.47170	2.54632
H	-2.16872	4.95892	2.41014
C	-1.06399	5.49242	0.59050
H	-1.92173	6.05162	0.15507
C	0.25906	6.19347	0.22035
H	0.36231	6.26213	-0.88539
H	0.26602	7.23631	0.60893
C	-1.24675	1.09222	0.42545
H	-1.95334	1.82496	0.82423
C	-1.89225	-0.16294	0.14354
C	-3.46182	-0.03693	0.09007
C	-3.84413	1.05682	-0.96567
H	-3.31442	2.00532	-0.74628
H	-3.49329	0.72917	-1.96919
C	-3.99989	0.42178	1.48883
H	-3.76374	-0.35707	2.24603
H	-3.48811	1.34786	1.82332
C	-4.24979	-1.32182	-0.29667
H	-3.91897	-1.69080	-1.28674
H	-4.04120	-2.13729	0.41986
C	-5.36776	1.31444	-0.99130
H	-5.58068	2.10892	-1.74048
C	-5.83284	1.78143	0.40679
H	-5.31903	2.72860	0.68567
H	-6.92339	2.00112	0.39488
C	-5.52421	0.68058	1.44683
H	-5.85376	1.01571	2.45527
C	-6.26466	-0.61563	1.05796
H	-6.07165	-1.41030	1.81238
H	-7.36422	-0.44475	1.04772
C	-5.77780	-1.06909	-0.33381
H	-6.27790	-2.02218	-0.61346
C	-6.10815	0.01775	-1.37665
H	-7.20610	0.19432	-1.41888
H	-5.79692	-0.31480	-2.39185
C	2.16438	1.45919	-1.08327
C	1.90865	2.05410	-2.36485
C	0.51087	2.13197	-2.98945
H	-0.23905	1.92428	-2.19951
C	0.17868	3.51282	-3.59313
H	0.33725	4.33598	-2.86700
H	-0.88161	3.54741	-3.92126
H	0.80070	3.73474	-4.48594
C	0.35998	1.03662	-4.06703
H	1.05836	1.21384	-4.91271
H	-0.67229	1.01724	-4.47721
H	0.58747	0.03414	-3.65513
C	2.99526	2.51727	-3.13484
H	2.79634	2.98016	-4.11474
C	4.31663	2.39401	-2.69280
H	5.15028	2.77094	-3.30614
C	4.56605	1.75578	-1.47133
H	5.60631	1.62794	-1.13877
C	3.52312	1.26975	-0.65794
C	3.88069	0.57822	0.66291
H	3.14670	-0.24471	0.81607
C	3.75631	1.51502	1.88334
H	4.43580	2.38749	1.77678
H	4.03473	0.98089	2.81593
H	2.72582	1.89862	2.01180
C	5.26925	-0.08754	0.66146
H	5.41390	-0.74733	-0.21652
H	5.38877	-0.70695	1.57390
H	6.08695	0.66437	0.67552
C	-1.64132	-2.60758	-0.35112
C	-1.67320	-3.04577	-1.71289
C	-1.27349	-2.14707	-2.88619
H	-0.93679	-1.17711	-2.46285
C	-2.45619	-1.85444	-3.83510
H	-2.83802	-2.78577	-4.30502
H	-2.14113	-1.17415	-4.65408
H	-3.30538	-1.37617	-3.30600
C	-0.09675	-2.75643	-3.68013
H	0.78259	-2.95685	-3.03460
H	0.22527	-2.07468	-4.49479
H	-0.38273	-3.71966	-4.15278
C	-2.08254	-4.36526	-1.99710
H	-2.12435	-4.69680	-3.04726
C	-2.43239	-5.26223	-0.98172
H	-2.75442	-6.28664	-1.22655
C	-2.34487	-4.84409	0.35155
H	-2.59476	-5.55480	1.15596
C	-1.94348	-3.53743	0.69695
C	-1.84177	-3.19178	2.18673
H	-1.49923	-2.13818	2.26407
C	-3.19469	-3.29930	2.92459
H	-3.97490	-2.65771	2.46852
H	-3.08354	-2.99330	3.98628
H	-3.57721	-4.34204	2.91907
C	-0.79772	-4.08505	2.89287

H	-1.12282	-5.14692	2.90650
H	-0.65671	-3.76713	3.94739
H	0.18796	-4.04349	2.38952
C	1.55710	-1.45216	2.51393
H	1.69360	-0.34842	2.63891
H	0.56406	-1.68353	2.96416
C	2.65782	-2.16933	3.31443
H	2.54365	-3.27368	3.20033
H	3.65446	-1.93952	2.87028
C	2.69508	-1.83702	4.81778
H	1.70027	-2.06936	5.26273
H	2.82445	-0.73732	4.94193
C	3.79417	-2.57841	5.59014
H	3.66783	-3.68027	5.51700
H	3.79136	-2.31421	6.66849
H	4.80308	-2.33758	5.19093
C	3.57431	-2.33741	-1.83894
C	3.31970	-3.06695	-0.64676
C	4.83504	-2.35119	-2.45705
H	4.98942	-1.77538	-3.38386
C	4.40650	-3.79962	-0.09849
C	5.89478	-3.08961	-1.89913
H	4.24635	-4.38118	0.82497
H	6.88391	-3.10315	-2.38338
C	5.66870	-3.81132	-0.71188
H	6.48570	-4.39812	-0.26043
H	2.76281	-1.74689	-2.29876
C	1.98561	-3.05149	0.01655
H	1.16369	-3.04789	-0.74363
H	1.80974	-3.97858	0.60117
H	1.83497	-2.23039	1.22964

D^Me

SCF (BP86) Energy = -2567.424885
Enthalpy 0K = -2566.189931
Enthalpy 298K = -2566.123789
Free Energy 298K = -2566.306724
Lowest Frequency = 1.8431 cm^{-1}
Second Frequency = 3.0644 cm^{-1}
SCF (BP86-D3^{BJ}) Energy = -2567.780253
SCF (C_6H_6) Energy = -2567.470432
SCF ($C_6H_5CH_3$) Energy = -2567.469342
SCF (BS2) Energy = -2570.017596

Mg	-1.35415	-1.47336	0.51733
N	0.03201	-1.26852	-1.00788
N	-1.90854	0.52469	0.56193
C	0.33006	-0.07939	-1.56998
C	1.39675	0.15507	-2.70791
C	2.17736	-1.10014	-3.19326
H	2.72336	-1.56402	-2.34941
H	1.47665	-1.86994	-3.56974
C	2.46971	1.18252	-2.21047
H	1.98309	2.11193	-1.85012
H	3.00794	0.75252	-1.33679
C	0.68273	0.75604	-3.96617
H	-0.05964	0.02119	-4.34876
H	0.10846	1.66416	-3.68991
C	3.18981	-0.75102	-4.31242
H	3.70871	-1.68753	-4.61321
C	4.22038	0.26789	-3.78465
H	4.96257	0.51514	-4.57614
H	4.79035	-0.16676	-2.93363
C	3.47382	1.53887	-3.33086
H	4.20005	2.28245	-2.93385
C	2.71187	2.14682	-4.53064
H	3.42785	2.43906	-5.33053
H	2.18399	3.07598	-4.21922
C	1.69782	1.11503	-5.07523
H	1.14473	1.55294	-5.93564
C	2.44655	-0.15585	-5.52574
H	1.72814	-0.89807	-5.93956
H	3.16468	0.08472	-6.34113
C	-0.32904	1.12069	-1.17338
H	0.02486	1.99169	-1.73048
C	-1.34943	1.45633	-0.23602
C	-1.72273	2.98782	-0.25814
C	-2.21785	3.37733	-1.69246
H	-1.46786	3.08433	-2.45553
H	-3.14193	2.80377	-1.92637
C	-0.44900	3.83726	0.07367
H	-0.10771	3.59384	1.10419
H	0.38719	3.56730	-0.60351
C	-2.83077	3.44216	0.73453
H	-3.76892	2.88529	0.54707
H	-2.53510	3.20819	1.77511
C	-2.48954	4.89496	-1.80432
H	-2.81933	5.12179	-2.84247
C	-1.19231	5.67450	-1.48978
H	-0.39512	5.40246	-2.21741
H	-1.36616	6.76783	-1.60036
C	-0.73203	5.35213	-0.04968
H	0.20461	5.90843	0.17653
C	-1.83635	5.75826	0.94747
H	-1.50699	5.55116	1.98996
H	-2.03381	6.85163	0.88432
C	-3.11597	4.96062	0.62322
H	-3.91322	5.21717	1.35492
C	-3.59108	5.30122	-0.80409
H	-3.81256	6.38839	-0.89047
H	-4.53657	4.76168	-1.03459
C	0.57474	-2.56589	-1.28910
C	-0.11111	-3.44323	-2.18552
C	-1.32794	-2.98998	-2.99907
H	-1.48494	-1.91154	-2.78347
C	-1.10342	-3.12960	-4.52035
H	-0.19853	-2.58313	-4.85672
H	-1.97227	-2.72645	-5.08249
H	-0.98072	-4.19231	-4.81909
C	-2.60765	-3.74297	-2.57559
H	-2.52199	-4.83118	-2.78095
H	-3.49168	-3.36561	-3.13187
H	-2.81188	-3.63013	-1.49117
C	0.35585	-4.76701	-2.32545
H	-0.16262	-5.44871	-3.01947
C	1.46365	-5.23272	-1.60548
H	1.81118	-6.27047	-1.72979
C	2.12329	-4.36758	-0.72032
H	2.99002	-4.73795	-0.15103
C	1.70034	-3.03653	-0.54113
C	2.42808	-2.12445	0.45092
H	2.18915	-1.07742	0.16713
C	3.96197	-2.27139	0.40709
H	4.29647	-3.26273	0.77892
H	4.44067	-1.50631	1.05365
H	4.35982	-2.14608	-0.62147
C	1.90848	-2.33991	1.88821
H	0.80869	-2.19783	1.96682

H	2.38824	-1.63258	2.59703
H	2.10404	-3.37416	2.23907
C	-2.94202	0.67224	1.54493
C	-4.30196	0.41068	1.18754
C	-4.72124	0.12366	-0.25783
H	-3.82614	0.27706	-0.89736
C	-5.81865	1.08511	-0.76151
H	-6.76710	0.95660	-0.19807
H	-6.04350	0.89395	-1.83213
H	-5.51424	2.14745	-0.66321
C	-5.17117	-1.34386	-0.42929
H	-4.38994	-2.05784	-0.09648
H	-5.40955	-1.56454	-1.49132
H	-6.08098	-1.55699	0.17123
C	-5.28037	0.40225	2.20253
H	-6.33117	0.20586	1.93371
C	-4.94767	0.63496	3.54373
H	-5.72797	0.62207	4.32098
C	-3.61019	0.87620	3.88702
H	-3.34768	1.05291	4.94273
C	-2.59154	0.89717	2.91332
C	-1.14138	1.13268	3.34668
H	-0.54011	1.27371	2.42380
C	-0.96796	2.40042	4.20897
H	-1.36055	3.30438	3.69913
H	0.10589	2.57502	4.43128
H	-1.49334	2.31196	5.18342
C	-0.57834	-0.10130	4.08393
H	-1.12344	-0.28051	5.03498
H	0.49570	0.03531	4.33073
H	-0.67328	-1.02991	3.48263
C	6.80732	3.67746	3.49348
H	7.16561	4.63643	3.06088
H	6.62107	3.85424	4.57474
C	7.82621	2.55152	3.27843
H	7.42105	1.59777	3.68830
H	7.96053	2.37267	2.18672
C	9.19803	2.82873	3.91445
H	9.06368	3.00765	5.00609
H	9.60333	3.78245	3.50463
C	10.21674	1.70263	3.69964
H	9.85850	0.74398	4.13291
H	11.19378	1.93641	4.17153
H	10.40297	1.52530	2.61844
C	-0.66358	-5.07056	1.80640
C	-1.43858	-4.02488	2.37787
C	0.14712	-5.90200	2.59459
H	0.72699	-6.70643	2.11213
C	-1.35473	-3.86668	3.78774
C	0.22352	-5.71898	3.98851
H	-1.96329	-3.08504	4.27265
H	0.85918	-6.37232	4.60716
C	-0.53729	-4.69287	4.57719
H	-0.50486	-4.53998	5.66897
H	-0.69863	-5.22780	0.71503
C	-2.28883	-3.13116	1.53709
H	-2.76641	-3.71913	0.72081
H	-3.10983	-2.68130	2.13689
H	5.83025	3.44402	3.02141

5′

SCF (BP86) Energy = -2409.095923
Enthalpy 0K = -2407.988848
Enthalpy 298K = -2407.931291
Free Energy 298K = -2408.081675
Lowest Frequency = 10.9981 cm^{-1}
Second Frequency = 13.4456 cm^{-1}
SCF (BP86-D3^{BJ}) Energy = -2409.438217
SCF (C_6H_6) Energy = -2409.137183
SCF ($C_6H_5CH_3$) Energy = -2409.136325
SCF (BS2) Energy = -2411.511064

Mg	-0.63354	-1.45462	0.40358
N	-1.36533	0.47430	0.22339
N	1.36288	-0.94042	0.17194
C	-0.57178	1.53813	-0.01636
C	-1.06300	3.02578	-0.19813
C	-2.59941	3.26118	-0.12604
H	-3.11658	2.66837	-0.90503
H	-2.99930	2.91268	0.84543
C	-0.59647	3.56039	-1.59518
H	0.49783	3.42799	-1.71867
H	-1.07664	2.95430	-2.39525
C	-0.41697	3.91951	0.91428
H	-0.76761	3.57033	1.91055
H	0.68640	3.80291	0.91358
C	-2.96178	4.75570	-0.31206
H	-4.06769	4.85352	-0.24720
C	-2.47668	5.24339	-1.69237
H	-2.74502	6.31288	-1.84276
H	-2.97886	4.66880	-2.50213
C	-0.94820	5.05478	-1.77576
H	-0.57991	5.38876	-2.77113
C	-0.26357	5.88286	-0.66439
H	-0.48176	6.96491	-0.80366
H	0.84194	5.76911	-0.72668
C	-0.77067	5.41144	0.71764
H	-0.27535	6.00317	1.51920
C	-2.29964	5.59830	0.79724
H	-2.67339	5.28209	1.79652
H	-2.56631	6.67236	0.68012
C	0.84354	1.40587	-0.12161
H	1.33681	2.36344	-0.30589
C	1.77161	0.32714	-0.03674
C	3.26824	0.79069	-0.21083
C	3.61668	1.83957	0.89944
H	2.88310	2.67175	0.89285
H	3.53100	1.35503	1.89727
C	3.43930	1.47513	-1.60941
H	3.22660	0.72995	-2.40759
H	2.69730	2.28979	-1.73777
C	4.34979	-0.32387	-0.12524
H	4.29201	-0.84267	0.85090
H	4.16933	-1.09632	-0.89712
C	5.03847	2.41538	0.70723
H	5.22941	3.16558	1.50642
C	5.13529	3.09582	-0.67733
H	4.40226	3.93057	-0.74672
H	6.14419	3.54449	-0.81387
C	4.86222	2.05335	-1.78549
H	4.92638	2.54258	-2.78273
C	5.90094	0.91700	-1.69173
H	5.72762	0.17120	-2.49912
H	6.92876	1.31721	-1.83951
C	5.77876	0.24530	-0.30868
H	6.49905	-0.59899	-0.23697
C	6.07676	1.27845	0.79708
H	7.10706	1.68323	0.68272

H	6.03088	0.79529	1.79841
C	-2.79079	0.42286	0.37384
C	-3.36887	0.45508	1.68098
C	-2.53114	0.71223	2.93805
H	-1.48986	0.90966	2.60532
C	-3.01011	1.95428	3.72032
H	-3.02678	2.86278	3.08399
H	-2.33984	2.15576	4.58263
H	-4.03484	1.81222	4.12450
C	-2.50037	-0.52378	3.86297
H	-3.51440	-0.76586	4.24622
H	-1.84396	-0.34550	4.74089
H	-2.12749	-1.42467	3.33408
C	-4.75803	0.24707	1.81120
H	-5.21374	0.27684	2.81452
C	-5.57044	0.00681	0.69549
H	-6.65301	-0.15333	0.82022
C	-4.99229	-0.03407	-0.58159
H	-5.63193	-0.22976	-1.45630
C	-3.61136	0.16645	-0.77017
C	-3.00779	0.08982	-2.17554
H	-2.03075	0.61704	-2.14026
C	-3.86534	0.78703	-3.25017
H	-4.82631	0.25861	-3.42397
H	-3.32682	0.80456	-4.22089
H	-4.10310	1.83516	-2.97304
C	-2.72570	-1.37322	-2.57805
H	-2.07771	-1.89741	-1.84229
H	-2.22202	-1.42648	-3.56614
H	-3.66179	-1.96664	-2.62997
C	2.14161	-2.13895	0.28727
C	2.53116	-2.60971	1.58059
C	2.27855	-1.79131	2.85089
H	1.91476	-0.79163	2.53181
C	3.55823	-1.57204	3.68518
H	3.95481	-2.52733	4.08962
H	3.34930	-0.91037	4.55223
H	4.36610	-1.10261	3.08683
C	1.17377	-2.43354	3.71841
H	0.23210	-2.57820	3.14966
H	0.94729	-1.80347	4.60452
H	1.48507	-3.43438	4.08618
C	3.14954	-3.87214	1.68474
H	3.45570	-4.23989	2.67765
C	3.38185	-4.67030	0.55637
H	3.86493	-5.65459	0.66101
C	2.98647	-4.20618	-0.70562
H	3.16456	-4.83630	-1.59223
C	2.36433	-2.95187	-0.86816
C	1.92624	-2.50649	-2.26674
H	1.59040	-1.45098	-2.18647
C	3.07436	-2.54902	-3.29706
H	3.94826	-1.94988	-2.96779
H	2.73485	-2.14592	-4.27450
H	3.42907	-3.58616	-3.47575
C	0.72581	-3.34011	-2.76361
H	1.00577	-4.40747	-2.89074
H	0.36004	-2.96877	-3.74417
H	-0.12582	-3.31567	-2.05186
C	-3.86379	-3.60800	0.22418
C	-2.49793	-3.99093	0.13195
C	-4.84743	-4.18419	-0.59449
H	-5.89691	-3.86341	-0.48582
C	-2.17965	-4.99046	-0.82697
C	-4.50660	-5.16418	-1.54619
H	-1.13545	-5.33609	-0.90871
H	-5.27903	-5.61699	-2.18805
C	-3.16190	-5.56252	-1.65281
H	-2.87342	-6.33927	-2.38071
H	-4.15531	-2.83539	0.95567
C	-1.44928	-3.36605	0.99135
H	-1.85187	-3.18960	2.01450
H	-0.56998	-4.03782	1.09991

C

SCF (BP86) Energy = -2567.415683
Enthalpy 0K = -2566.181400
Enthalpy 298K = -2566.115046
Free Energy 298K = -2566.297292
Lowest Frequency = 2.9887 cm^{-1}
Second Frequency = 4.3912 cm^{-1}
SCF (BP86-D3^{BJ}) Energy = -2567.765612
SCF (C_6H_6) Energy = -2567.461590
SCF ($C_6H_5CH_3$) Energy = -2567.460433
SCF (BS2) Energy = -2570.008772

Mg	0.53026	1.60418	0.74499
N	-0.24949	-0.31483	0.90075
N	2.31809	1.00349	-0.13022
C	0.41812	-1.41895	0.51127
C	-0.09297	-2.90178	0.67533
C	-1.50859	-3.07599	1.29619
H	-1.55415	-2.59308	2.29138
H	-2.26929	-2.56958	0.67157
C	0.90472	-3.67069	1.60801
H	1.93983	-3.58902	1.21725
H	0.90975	-3.18761	2.61023
C	-0.12192	-3.62024	-0.71512
H	-0.85198	-3.10467	-1.37756
H	0.86439	-3.54194	-1.21694
C	-1.89433	-4.56916	1.44005
H	-2.91053	-4.62233	1.88877
C	-0.88308	-5.28832	2.35594
H	-1.16122	-6.35922	2.47600
H	-0.89595	-4.83727	3.37311
C	0.52405	-5.16313	1.73690
H	1.27089	-5.66590	2.39079
C	0.53414	-5.81929	0.33743
H	0.29354	-6.90243	0.42044
H	1.55068	-5.75097	-0.11086
C	-0.49788	-5.11332	-0.57141
H	-0.48851	-5.58130	-1.58083
C	-1.90371	-5.23959	0.05109
H	-2.65919	-4.75363	-0.60555
H	-2.19455	-6.31027	0.13835
C	1.71406	-1.33955	-0.07724
H	2.11108	-2.32205	-0.34515
C	2.61454	-0.28239	-0.40086
C	3.92829	-0.78838	-1.10983
C	3.53219	-1.50574	-2.44546
H	2.78767	-2.30443	-2.24882
H	3.03362	-0.77208	-3.11706
C	4.66774	-1.82169	-0.19511
H	4.98451	-1.31645	0.74409
H	3.98116	-2.63908	0.10677
C	4.97223	0.30304	-1.48163
H	4.51328	1.06619	-2.13940
H	5.30925	0.83974	-0.57394

C	4.76575	-2.11851	-3.14688
H	4.42971	-2.62965	-4.07633
C	5.43691	-3.14338	-2.20424
H	4.72514	-3.96357	-1.96001
H	6.30783	-3.61625	-2.71005
C	5.89517	-2.43225	-0.91041
H	6.37362	-3.16988	-0.22847
C	6.90069	-1.31738	-1.26398
H	7.25501	-0.81227	-0.33797
H	7.79947	-1.74792	-1.75932
C	6.20771	-0.30085	-2.19434
H	6.90949	0.52842	-2.43243
C	5.77203	-1.00309	-3.49658
H	6.65572	-1.42782	-4.02315
H	5.30562	-0.27005	-4.19184
C	-1.58787	-0.19048	1.39570
C	-2.67484	-0.07089	0.47115
C	-2.45402	-0.20680	-1.03852
H	-1.48757	-0.73262	-1.18604
C	-3.54205	-1.03943	-1.74535
H	-3.67042	-2.03468	-1.27133
H	-3.27232	-1.20205	-2.81016
H	-4.53005	-0.53325	-1.73500
C	-2.32078	1.18160	-1.70093
H	-3.25626	1.76861	-1.58640
H	-2.10249	1.08864	-2.78598
H	-1.50609	1.78444	-1.24497
C	-3.96219	0.20859	0.96967
H	-4.80528	0.29865	0.26654
C	-4.19476	0.37673	2.34266
H	-5.20985	0.59383	2.71081
C	-3.12337	0.27371	3.23900
H	-3.30617	0.41155	4.31737
C	-1.81369	-0.00275	2.79478
C	-0.67978	-0.09162	3.82103
H	0.24678	-0.34747	3.26290
C	-0.91275	-1.20765	4.86231
H	-1.81338	-1.00583	5.48036
H	-0.04706	-1.28466	5.55382
H	-1.05319	-2.19841	4.38325
C	-0.44668	1.26249	4.52590
H	-0.26208	2.07782	3.79653
H	0.42533	1.20647	5.21149
H	-1.32956	1.55739	5.13213
C	3.12163	2.17710	-0.29705
C	2.90744	3.03071	-1.42359
C	1.94336	2.65640	-2.55376
H	1.56196	1.63595	-2.33438
C	2.63768	2.60222	-3.93145
H	3.01827	3.60006	-4.23675
H	1.92619	2.26623	-4.71528
H	3.49899	1.90283	-3.93290
C	0.73045	3.61180	-2.59734
H	0.20408	3.65219	-1.62102
H	0.00152	3.29217	-3.37178
H	1.04523	4.64900	-2.84055
C	3.59793	4.25844	-1.48587
H	3.44315	4.91792	-2.35557
C	4.47219	4.66002	-0.46766
H	4.99995	5.62444	-0.53523
C	4.66108	3.82625	0.64388
H	5.34106	4.14778	1.44902
C	3.99988	2.58744	0.75621
C	4.20205	1.72893	2.00876
H	3.83207	0.70890	1.77411
C	5.68056	1.59576	2.42569
H	6.31181	1.22513	1.59154
H	5.78165	0.88359	3.27161
H	6.10731	2.56368	2.76386
C	3.35380	2.26347	3.18378
H	3.67223	3.28806	3.47087
H	3.45129	1.61079	4.07724
H	2.27565	2.32538	2.92349
C	-0.10497	3.56304	1.31829
H	0.27301	3.74691	2.35487
H	0.46876	4.29509	0.69977
C	-1.60459	3.91629	1.27252
H	-1.99677	3.79677	0.23440
H	-2.19265	3.18910	1.88091
C	-1.94833	5.34110	1.75253
H	-1.36990	6.07538	1.14534
H	-1.57909	5.46630	2.79684
C	-3.44368	5.68009	1.68922
H	-3.83204	5.60450	0.65005
H	-3.65322	6.71039	2.04752
H	-4.04326	4.98160	2.31251
C	-9.01620	1.19947	-0.60218
C	-8.21153	2.04844	-1.38285
C	-9.54417	0.02042	-1.15505
H	-7.79578	2.97257	-0.95085
C	-7.94036	1.70507	-2.71899
C	-9.28397	-0.33680	-2.49711
H	-7.30890	2.36072	-3.34014
C	-8.47102	0.52435	-3.26716
H	-8.24982	0.26324	-4.31574
H	-9.23284	1.45606	0.44743
H	-10.17124	-0.63966	-0.53235
C	-9.88297	-1.58951	-3.09841
H	-9.27169	-1.97307	-3.94040
H	-9.97984	-2.39920	-2.34641
H	-10.90297	-1.39389	-3.49777

TS (C–D°)

SCF (BP86) Energy = -2567.346694
Enthalpy 0K = -2566.114150
Enthalpy 298K = -2566.050232
Free Energy 298K = -2566.210483
Lowest Frequency = -1274.1511 cm^{-1}
Second Frequency = 11.4717 cm^{-1}
SCF (BP86-D3^{BJ}) Energy = -2567.730464
SCF (C_6H_6) Energy = -2567.386306
SCF ($C_6H_5CH_3$) Energy = -2567.385244
SCF (BS2) Energy = -2569.937204

Mg	-0.30904	-1.43720	0.21494
N	1.54423	-0.52649	-0.14949
N	-1.56758	0.24320	0.12995
C	1.60138	0.78922	0.19245
C	2.93732	1.61956	0.43750
C	4.28511	0.88857	0.16125
H	4.33811	-0.05344	0.73586
H	4.35852	0.60739	-0.90607
C	2.95042	2.03620	1.95260
H	2.01830	2.57995	2.21014
H	2.96021	1.12252	2.58556
C	2.96647	2.92997	-0.41773
H	2.98783	2.66535	-1.49630
H	2.04314	3.52412	-0.26144

C	5.50743	1.77020	0.51958
H	6.42745	1.18333	0.30497
C	5.47081	2.15219	2.01277
H	6.35562	2.77348	2.27691
H	5.51818	1.23861	2.64586
C	4.16700	2.92609	2.29314
H	4.11336	3.20025	3.37023
C	4.12614	4.20567	1.42815
H	4.98036	4.87069	1.68519
H	3.19738	4.78181	1.63914
C	4.18514	3.81616	-0.06600
H	4.14620	4.73448	-0.69316
C	5.49275	3.04763	-0.34414
H	5.56180	2.78278	-1.42265
H	6.37438	3.68614	-0.11242
C	0.44121	1.57596	0.43648
H	0.71054	2.59508	0.72056
C	-0.97573	1.44646	0.26823
C	-1.69618	2.85800	0.25205
C	-1.08603	3.71557	-0.91074
H	0.01750	3.75985	-0.81848
H	-1.29624	3.21383	-1.88055
C	-1.42511	3.60802	1.60238
H	-1.87601	3.03256	2.43952
H	-0.33780	3.66013	1.81538
C	-3.23907	2.86709	0.03860
H	-3.49770	2.37087	-0.91540
H	-3.74705	2.28875	0.83153
C	-1.65326	5.15365	-0.92010
H	-1.17304	5.71793	-1.75016
C	-1.33455	5.84068	0.42713
H	-0.23329	5.89560	0.57933
H	-1.70917	6.88829	0.42171
C	-1.99294	5.04637	1.57783
H	-1.75938	5.53432	2.55015
C	-3.51988	5.00346	1.36668
H	-4.00887	4.45475	2.20188
H	-3.94074	6.03372	1.37008
C	-3.81366	4.30589	0.02332
H	-4.91290	4.23384	-0.12928
C	-3.18048	5.11219	-1.12835
H	-3.59743	6.14371	-1.15642
H	-3.42249	4.64139	-2.10698
C	2.59191	-1.22029	-0.84420
C	2.87398	-0.88381	-2.20910
C	2.04804	0.14275	-2.99103
H	1.50962	0.78324	-2.26347
C	2.89513	1.07196	-3.88406
H	3.71724	1.55696	-3.31855
H	2.26059	1.87344	-4.31720
H	3.35370	0.52790	-4.73675
C	0.98258	-0.58580	-3.83797
H	1.46078	-1.24139	-4.59688
H	0.33448	0.13936	-4.37434
H	0.33085	-1.22738	-3.21119
C	3.90087	-1.57205	-2.88798
H	4.12257	-1.30934	-3.93435
C	4.64337	-2.57849	-2.26154
H	5.45463	-3.09317	-2.80034
C	4.32023	-2.94728	-0.94896
H	4.88334	-3.76396	-0.47488
C	3.29087	-2.31062	-0.22589
C	2.93356	-2.82049	1.17701
H	1.82298	-2.83568	1.22747
C	3.40534	-1.90580	2.32646
H	4.51264	-1.81372	2.33656
H	3.09497	-2.32796	3.30549
H	2.97326	-0.89060	2.24981
C	3.40911	-4.25962	1.45313
H	3.09425	-4.96938	0.66082
H	2.98735	-4.61722	2.41464
H	4.51468	-4.32192	1.54419
C	-2.96786	-0.00752	-0.08994
C	-3.46727	-0.13705	-1.42540
C	-2.58271	0.00715	-2.66858
H	-1.54542	0.19954	-2.32094
C	-3.00304	1.19825	-3.55802
H	-4.02264	1.05259	-3.97426
H	-2.30826	1.30669	-4.41768
H	-3.00503	2.15764	-3.00224
C	-2.57240	-1.29275	-3.50263
H	-2.21796	-2.16036	-2.91158
H	-1.91476	-1.18241	-4.38963
H	-3.58912	-1.53809	-3.87577
C	-4.83643	-0.41254	-1.61759
H	-5.22519	-0.49525	-2.64546
C	-5.71159	-0.58348	-0.53872
H	-6.78038	-0.78608	-0.71203
C	-5.20383	-0.51719	0.76442
H	-5.88473	-0.68010	1.61531
C	-3.84227	-0.25087	1.02035
C	-3.37843	-0.24716	2.48338
H	-2.26925	-0.18309	2.48000
C	-3.91500	0.95760	3.28816
H	-3.59129	1.92781	2.86433
H	-3.55637	0.91370	4.33834
H	-5.02574	0.95814	3.31383
C	-3.78278	-1.55008	3.21051
H	-4.88082	-1.60129	3.36864
H	-3.30506	-1.60067	4.21119
H	-3.48561	-2.45566	2.64522
C	-0.45771	-2.73007	2.16372
H	0.39038	-2.13427	2.58606
H	-1.37814	-2.27268	2.58871
C	-0.34877	-4.17973	2.66952
H	-1.21341	-4.76915	2.28329
H	0.54787	-4.67220	2.22445
C	-0.28741	-4.34029	4.20015
H	-1.18314	-3.85371	4.64987
H	0.58600	-3.76828	4.59037
C	-0.20186	-5.79904	4.66872
H	-1.08216	-6.38553	4.32758
H	-0.15807	-5.87700	5.77551
H	0.70245	-6.30281	4.26353
C	-0.63409	-4.33718	-1.54329
C	-1.21928	-3.46255	-0.57491
C	-1.46543	-5.15590	-2.33881
H	-0.69849	-3.14406	0.69527
H	-1.00685	-5.82622	-3.08621
C	-2.63052	-3.49232	-0.43564
C	-2.86472	-5.13953	-2.19389
H	-3.10831	-2.86249	0.33654
H	-3.48939	-5.79134	-2.82653
C	-3.45624	-4.30488	-1.23253
H	-4.54991	-4.29028	-1.09916
C	0.86601	-4.40547	-1.72904
H	1.15866	-5.20454	-2.43941
H	1.28408	-3.45064	-2.11301

H	1.37950	-4.60577	-0.76582

D°

SCF (BP86) Energy = -2567.419416
Enthalpy 0K = -2566.184161
Enthalpy 298K = -2566.117864
Free Energy 298K = -2566.298959
Lowest Frequency = 2.0918 cm^{-1}
Second Frequency = 3.0053 cm^{-1}
SCF (BP86-D3^{BJ}) Energy = -2567.779822
SCF (C_6H_6) Energy = -2567.463926
SCF ($C_6H_5CH_3$) Energy = -2567.462824
SCF (BS2) Energy = -2570.010952

Mg	-1.39671	-0.61625	1.25292
N	-0.30668	-1.70709	-0.14963
N	-1.37791	1.15400	0.15182
C	0.18601	-1.16401	-1.28395
C	1.17002	-1.88033	-2.29099
C	1.51873	-3.36547	-1.98556
H	1.93417	-3.45933	-0.96393
H	0.60579	-3.99102	-2.00765
C	2.52409	-1.08816	-2.26962
H	2.34371	-0.01512	-2.48601
H	2.95221	-1.13255	-1.24368
C	0.60400	-1.84649	-3.74992
H	-0.33891	-2.43527	-3.79134
H	0.33869	-0.81130	-4.04735
C	2.53844	-3.93940	-3.00060
H	2.74334	-4.99674	-2.72333
C	3.84780	-3.12598	-2.95140
H	4.59025	-3.54185	-3.66854
H	4.30580	-3.19379	-1.93955
C	3.53190	-1.65607	-3.29427
H	4.46315	-1.04866	-3.25129
C	2.92309	-1.57434	-4.71253
H	3.65158	-1.95021	-5.46479
H	2.70820	-0.51480	-4.97736
C	1.62453	-2.41128	-4.76568
H	1.18207	-2.34925	-5.78466
C	1.94440	-3.88055	-4.42266
H	1.02056	-4.49814	-4.47976
H	2.66081	-4.30438	-5.16128
C	-0.09911	0.17944	-1.65656
H	0.33679	0.44539	-2.62270
C	-0.81516	1.26142	-1.06808
C	-0.91726	2.51803	-2.01718
C	-1.70192	2.07559	-3.30166
H	-1.21616	1.19078	-3.76209
H	-2.72518	1.75318	-3.00685
C	0.50267	3.01007	-2.45422
H	1.06088	3.36130	-1.55844
H	1.09494	2.17632	-2.88423
C	-1.66136	3.75839	-1.44534
H	-2.68178	3.47682	-1.12173
H	-1.14459	4.13885	-0.54329
C	-1.78050	3.21663	-4.34071
H	-2.32796	2.84562	-5.23556
C	-0.35092	3.64191	-4.74569
H	0.18956	2.78317	-5.20332
H	-0.39297	4.44136	-5.51835
C	0.40962	4.14736	-3.49840
H	1.44086	4.44941	-3.78692
C	-0.33839	5.35249	-2.89251
H	0.21201	5.73998	-2.00651
H	-0.39125	6.18583	-3.62816
C	-1.75640	4.90282	-2.48486
H	-2.29842	5.75250	-2.01472
C	-2.52587	4.42146	-3.73206
H	-2.61021	5.24365	-4.47730
H	-3.56380	4.13047	-3.45600
C	-0.30279	-3.08686	0.23165
C	-1.27034	-3.97580	-0.34122
C	-2.23302	-3.50041	-1.43541
H	-1.74547	-2.65305	-1.96072
C	-2.54071	-4.57414	-2.49684
H	-1.61303	-5.00568	-2.92672
H	-3.13002	-4.13486	-3.32881
H	-3.14145	-5.41208	-2.08409
C	-3.54485	-2.95329	-0.83152
H	-4.09315	-3.74692	-0.28176
H	-4.21376	-2.55770	-1.62473
H	-3.36094	-2.12627	-0.11233
C	-1.33952	-5.29803	0.13816
H	-2.07415	-5.99233	-0.29859
C	-0.49482	-5.75121	1.16325
H	-0.56747	-6.78960	1.52320
C	0.43422	-4.86976	1.72966
H	1.09288	-5.22546	2.53862
C	0.54950	-3.53475	1.28775
C	1.59087	-2.61917	1.93988
H	1.53433	-1.63877	1.41791
C	3.03028	-3.14958	1.75986
H	3.17322	-4.12003	2.28098
H	3.76505	-2.43426	2.18603
H	3.28736	-3.30373	0.69162
C	1.28845	-2.37860	3.43510
H	0.27174	-1.96084	3.59023
H	2.02294	-1.67031	3.87386
H	1.35180	-3.32392	4.01504
C	-1.91431	2.19897	0.97088
C	-3.32544	2.29671	1.16909
C	-4.31820	1.42556	0.39208
H	-3.72203	0.79979	-0.30801
C	-5.29061	2.26717	-0.46323
H	-5.94304	2.90313	0.17204
H	-5.95393	1.60819	-1.06260
H	-4.75406	2.93772	-1.16575
C	-5.10425	0.47717	1.32374
H	-4.42555	-0.14917	1.93968
H	-5.76698	-0.19147	0.73406
H	-5.74768	1.04781	2.02669
C	-3.81403	3.23385	2.10256
H	-4.90165	3.31738	2.26016
C	-2.94973	4.05881	2.83267
H	-3.35284	4.78361	3.55744
C	-1.56427	3.95010	2.63839
H	-0.88767	4.59693	3.21857
C	-1.01982	3.02902	1.72215
C	0.50060	2.90196	1.57153
H	0.70037	2.47192	0.56813
C	1.24034	4.25215	1.64078
H	0.81844	4.98989	0.92701
H	2.31455	4.11575	1.39577
H	1.19597	4.70268	2.65485
C	1.08729	1.91448	2.60447
H	0.91904	2.27228	3.64194
H	2.18065	1.78776	2.45635

H	0.62343	0.90740	2.53315
C	8.36346	2.73971	1.02321
H	9.21969	2.31626	0.45520
H	8.46912	3.84580	1.00032
C	8.33069	2.21068	2.46238
H	7.44074	2.62253	2.99168
H	8.18486	1.10596	2.45103
C	9.59455	2.54212	3.27235
H	9.74040	3.64683	3.28369
H	10.48453	2.13027	2.74312
C	9.56164	2.01306	4.71146
H	8.70561	2.43671	5.27961
H	10.48842	2.27020	5.26543
H	9.45592	0.90700	4.73435
C	-2.75855	-2.03208	3.82304
C	-2.17870	-0.86313	3.23699
C	-3.18641	-2.02355	5.17030
H	7.43679	2.48241	0.46893
H	-3.62804	-2.93907	5.60150
C	-2.08556	0.28159	4.07445
C	-3.06347	-0.87499	5.97099
H	-1.67156	1.22388	3.67496
H	-3.40133	-0.89343	7.02044
C	-2.51195	0.28950	5.41758
H	-2.41359	1.20505	6.02451
C	-2.95730	-3.29575	3.01064
H	-3.24033	-4.15988	3.64605
H	-3.76757	-3.16538	2.25983
H	-2.04182	-3.57671	2.45025

5-*o*

SCF (BP86) Energy = -2567.419416
Enthalpy 0K = -2566.184161
Enthalpy 298K = -2566.117864
Free Energy 298K = -2566.298959
Lowest Frequency = 2.0918 cm^{-1}
Second Frequency = 3.0053 cm^{-1}
SCF (BP86-D3^{BJ}) Energy = -2567.779822
SCF (C_6H_6) Energy = -2567.463926
SCF ($C_6H_5CH_3$) Energy = -2567.462824
SCF (BS2) Energy = -2570.010952

Mg	-1.39671	-0.61625	1.25292
N	-0.30668	-1.70709	-0.14963
N	-1.37791	1.15400	0.15182
C	0.18601	-1.16401	-1.28395
C	1.17002	-1.88033	-2.29099
C	1.51873	-3.36547	-1.98556
H	1.93417	-3.45933	-0.96393
H	0.60579	-3.99102	-2.00765
C	2.52409	-1.08816	-2.26962
H	2.34371	-0.01512	-2.48601
H	2.95221	-1.13255	-1.24368
C	0.60400	-1.84649	-3.74992
H	-0.33891	-2.43527	-3.79134
H	0.33869	-0.81130	-4.04735
C	2.53844	-3.93940	-3.00060
H	2.74334	-4.99674	-2.72333
C	3.84780	-3.12598	-2.95140
H	4.59025	-3.54185	-3.66854
H	4.30580	-3.19379	-1.93955
C	3.53190	-1.65607	-3.29427
H	4.46315	-1.04866	-3.25129
C	2.92309	-1.57434	-4.71253
H	3.65158	-1.95021	-5.46479
H	2.70820	-0.51480	-4.97736
C	1.62453	-2.41128	-4.76568
H	1.18207	-2.34925	-5.78466
C	1.94440	-3.88055	-4.42266
H	1.02056	-4.49814	-4.47976
H	2.66081	-4.30438	-5.16128
C	-0.09911	0.17944	-1.65656
H	0.33679	0.44539	-2.62270
C	-0.81516	1.26142	-1.06808
C	-0.91726	2.51803	-2.01718
C	-1.70192	2.07559	-3.30166
H	-1.21616	1.19078	-3.76209
H	-2.72518	1.75318	-3.00685
C	0.50267	3.01007	-2.45422
H	1.06088	3.36130	-1.55844
H	1.09494	2.17632	-2.88423
C	-1.66136	3.75839	-1.44534
H	-2.68178	3.47682	-1.12173
H	-1.14459	4.13885	-0.54329
C	-1.78050	3.21663	-4.34071
H	-2.32796	2.84562	-5.23556
C	-0.35092	3.64191	-4.74569
H	0.18956	2.78317	-5.20332
H	-0.39297	4.44136	-5.51835
C	0.40962	4.14736	-3.49840
H	1.44086	4.44941	-3.78692
C	-0.33839	5.35249	-2.89251
H	0.21201	5.73998	-2.00651
H	-0.39125	6.18583	-3.62816
C	-1.75640	4.90282	-2.48486
H	-2.29842	5.75250	-2.01472
C	-2.52587	4.42146	-3.73206
H	-2.61021	5.24365	-4.47730
H	-3.56380	4.13047	-3.45600
C	-0.30279	-3.08686	0.23165
C	-1.27034	-3.97580	-0.34122
C	-2.23302	-3.50041	-1.43541
H	-1.74547	-2.65305	-1.96072
C	-2.54071	-4.57414	-2.49684
H	-1.61303	-5.00568	-2.92672
H	-3.13002	-4.13486	-3.32881
H	-3.14145	-5.41208	-2.08409
C	-3.54485	-2.95329	-0.83152
H	-4.09315	-3.74692	-0.28176
H	-4.21376	-2.55770	-1.62473
H	-3.36094	-2.12627	-0.11233
C	-1.33952	-5.29803	0.13816
H	-2.07415	-5.99233	-0.29859
C	-0.49482	-5.75121	1.16325
H	-0.56747	-6.78960	1.52320
C	0.43422	-4.86976	1.72966
H	1.09288	-5.22546	2.53862
C	0.54950	-3.53475	1.28775
C	1.59087	-2.61917	1.93988
H	1.53433	-1.63877	1.41791
C	3.03028	-3.14958	1.75986
H	3.17322	-4.12003	2.28098
H	3.76505	-2.43426	2.18603
H	3.28736	-3.30373	0.69162
C	1.28845	-2.37860	3.43510
H	0.27174	-1.96084	3.59023
H	2.02294	-1.67031	3.87386
H	1.35180	-3.32392	4.01504

C	-1.91431	2.19897	0.97088
C	-3.32544	2.29671	1.16909
C	-4.31820	1.42556	0.39208
H	-3.72203	0.79979	-0.30801
C	-5.29061	2.26717	-0.46323
H	-5.94304	2.90313	0.17204
H	-5.95393	1.60819	-1.06260
H	-4.75406	2.93772	-1.16575
C	-5.10425	0.47717	1.32374
H	-4.42555	-0.14917	1.93968
H	-5.76698	-0.19147	0.73406
H	-5.74768	1.04781	2.02669
C	-3.81403	3.23385	2.10256
H	-4.90165	3.31738	2.26016
C	-2.94973	4.05881	2.83267
H	-3.35284	4.78361	3.55744
C	-1.56427	3.95010	2.63839
H	-0.88767	4.59693	3.21857
C	-1.01982	3.02902	1.72215
C	0.50060	2.90196	1.57153
H	0.70037	2.47192	0.56813
C	1.24034	4.25215	1.64078
H	0.81844	4.98989	0.92701
H	2.31455	4.11575	1.39577
H	1.19597	4.70268	2.65485
C	1.08729	1.91448	2.60447
H	0.91904	2.27228	3.64194
H	2.18065	1.78776	2.45635
H	0.62343	0.90740	2.53315
C	8.36346	2.73971	1.02321
H	9.21969	2.31626	0.45520
H	8.46912	3.84580	1.00032
C	8.33069	2.21068	2.46238
H	7.44074	2.62253	2.99168
H	8.18486	1.10596	2.45103
C	9.59455	2.54212	3.27235
H	9.74040	3.64683	3.28369
H	10.48453	2.13027	2.74312
C	9.56164	2.01306	4.71146
H	8.70561	2.43671	5.27961
H	10.48842	2.27020	5.26543
H	9.45592	0.90700	4.73435
C	-2.75855	-2.03208	3.82304
C	-2.17870	-0.86313	3.23699
C	-3.18641	-2.02355	5.17030
H	7.43679	2.48241	0.46893
H	-3.62804	-2.93907	5.60150
C	-2.08556	0.28159	4.07445
C	-3.06347	-0.87499	5.97099
H	-1.67156	1.22388	3.67496
H	-3.40133	-0.89343	7.02044
C	-2.51195	0.28950	5.41758
H	-2.41359	1.20505	6.02451
C	-2.95730	-3.29575	3.01064
H	-3.24033	-4.15988	3.64605
H	-3.76757	-3.16538	2.25983
H	-2.04182	-3.57671	2.45025

TS(C–D^{m})

SCF (BP86) Energy = -2567.353080
Enthalpy 0K = -2566.121175
Enthalpy 298K = -2566.056897
Free Energy 298K = -2566.219391
Lowest Frequency = -1289.4974 cm^{-1}
Second Frequency = 12.8929 cm^{-1}
SCF (BP86-D3^{BJ}) Energy = -2567.730891
SCF (C_6H_6) Energy = -2567.393256
SCF ($C_6H_5CH_3$) Energy = -2567.392148
SCF (BS2) Energy = -2569.944561

Mg	0.46528	-1.27112	0.38754
N	1.52847	0.41562	-0.19593
N	-1.49373	-0.61813	0.11096
C	0.87175	1.55069	0.17371
C	1.57907	2.93020	0.50528
C	3.09536	3.02410	0.16656
H	3.64686	2.18662	0.63128
H	3.25134	2.92853	-0.92501
C	1.43986	3.13287	2.05679
H	0.37001	3.08893	2.34964
H	1.94113	2.29142	2.58345
C	0.89143	4.14693	-0.19608
H	0.98926	4.04340	-1.29754
H	-0.19635	4.16774	0.02049
C	3.71027	4.36425	0.64005
H	4.79086	4.35985	0.37705
C	3.54781	4.51516	2.16649
H	4.00252	5.47036	2.51209
H	4.08289	3.69482	2.69402
C	2.04529	4.47947	2.51301
H	1.90789	4.57515	3.61306
C	1.31982	5.63937	1.79498
H	1.72647	6.61693	2.13705
H	0.23753	5.63441	2.05506
C	1.50273	5.49116	0.26767
H	0.97269	6.31888	-0.25390
C	3.00569	5.53456	-0.07548
H	3.15206	5.45622	-1.17572
H	3.44642	6.50693	0.23897
C	-0.53029	1.58711	0.41303
H	-0.85647	2.58262	0.72455
C	-1.65243	0.71391	0.19876
C	-3.01301	1.50332	0.07931
C	-2.88234	2.54355	-1.08650
H	-1.99084	3.18366	-0.93177
H	-2.71263	1.99764	-2.04078
C	-3.29409	2.28717	1.40683
H	-3.41949	1.56336	2.24113
H	-2.42951	2.92762	1.67792
C	-4.28452	0.65880	-0.22374
H	-4.15169	0.09162	-1.16485
H	-4.45027	-0.09511	0.56757
C	-4.14059	3.43421	-1.19780
H	-3.98714	4.16335	-2.02408
C	-4.35104	4.19358	0.13192
H	-3.47394	4.84494	0.34487
H	-5.23568	4.86392	0.05512
C	-4.55213	3.17785	1.27934
H	-4.69908	3.72172	2.23879
C	-5.78552	2.30118	0.98021
H	-5.95619	1.58083	1.81078
H	-6.70022	2.93113	0.90975
C	-5.54932	1.54558	-0.34357
H	-6.41610	0.88204	-0.55615
C	-5.37481	2.55831	-1.49333
H	-6.28513	3.19026	-1.59589
H	-5.24379	2.02423	-2.46063
C	2.73444	0.36800	-0.96558

C	2.76107	0.90306	-2.29780
C	1.52155	1.48316	-2.98730
H	0.75251	1.68394	-2.21447
C	1.78806	2.80876	-3.73023
H	2.27294	3.56337	-3.07764
H	0.83564	3.24171	-4.10227
H	2.44417	2.66474	-4.61465
C	0.93460	0.43753	-3.95967
H	1.64378	0.22587	-4.78848
H	-0.01580	0.79688	-4.40894
H	0.73350	-0.52182	-3.44458
C	3.95104	0.82031	-3.04934
H	3.96902	1.23830	-4.06852
C	5.10296	0.21293	-2.53891
H	6.02616	0.16830	-3.13805
C	5.05592	-0.36307	-1.26291
H	5.95349	-0.86448	-0.87230
C	3.89463	-0.31666	-0.46434
C	3.92191	-0.98516	0.91606
H	2.91911	-1.43265	1.08496
C	4.14662	0.01155	2.07304
H	5.11117	0.54957	1.95207
H	4.17573	-0.52262	3.04595
H	3.33715	0.76408	2.13093
C	4.93901	-2.13744	1.02971
H	4.83904	-2.87054	0.20394
H	4.78457	-2.68188	1.98379
H	5.98700	-1.76912	1.03617
C	-2.49426	-1.62585	-0.12037
C	-2.73881	-2.09332	-1.45039
C	-2.01647	-1.52892	-2.67678
H	-1.33283	-0.72846	-2.32315
C	-2.98850	-0.89217	-3.69395
H	-3.68273	-1.64678	-4.12124
H	-2.42695	-0.44404	-4.54069
H	-3.60676	-0.09261	-3.23676
C	-1.15996	-2.61538	-3.36357
H	-0.40554	-3.04279	-2.67284
H	-0.62918	-2.19847	-4.24480
H	-1.79127	-3.45416	-3.72564
C	-3.67820	-3.12536	-1.64968
H	-3.87674	-3.47517	-2.67572
C	-4.36140	-3.71563	-0.58017
H	-5.09785	-4.51493	-0.75933
C	-4.08001	-3.28814	0.72293
H	-4.59944	-3.76541	1.56969
C	-3.14733	-2.26288	0.98544
C	-2.88559	-1.89520	2.45131
H	-2.07333	-1.13753	2.46665
C	-4.11916	-1.27525	3.14498
H	-4.47974	-0.36365	2.62885
H	-3.87723	-0.99653	4.19241
H	-4.96505	-1.99455	3.17889
C	-2.41059	-3.12139	3.26290
H	-3.21801	-3.87773	3.36003
H	-2.11572	-2.82004	4.28999
H	-1.53985	-3.61923	2.79225
C	0.98057	-2.11631	2.50296
H	1.54043	-1.18134	2.75441
H	-0.04425	-1.96630	2.91448
C	1.64123	-3.29715	3.23668
H	1.07703	-4.23466	3.01805
H	2.65801	-3.48031	2.81495
C	1.76318	-3.13959	4.76382
H	0.74859	-2.96674	5.19074
H	2.33887	-2.21174	4.98688
C	2.42085	-4.33751	5.46178
H	1.84535	-5.27255	5.28883
H	2.49294	-4.18917	6.55987
H	3.45044	-4.51310	5.08129
C	2.04667	-3.63673	-1.05065
C	0.96784	-3.45423	-0.14585
C	2.13076	-4.74190	-1.92696
H	1.05252	-2.80806	1.11575
C	-0.04205	-4.44792	-0.13332
C	1.09668	-5.70528	-1.87823
H	-0.89723	-4.35271	0.55993
H	1.14040	-6.58161	-2.54760
C	0.01851	-5.56220	-0.98913
H	-0.77859	-6.32374	-0.96743
H	2.85835	-2.88743	-1.09766
C	3.29178	-4.90387	-2.88460
H	3.90379	-5.79708	-2.63146
H	2.94228	-5.04189	-3.93024
H	3.96105	-4.02063	-2.86502

D^m

SCF (BP86) Energy = -2567.425164
Enthalpy 0K = -2566.190224
Enthalpy 298K = -2566.123545
Free Energy 298K = -2566.307932
Lowest Frequency = 1.4829 cm^{-1}
Second Frequency = 2.0093 cm^{-1}
SCF (BP86-D3^{BJ}) Energy = -2567.779631
SCF (C_6H_6) Energy = -2567.469177
SCF ($C_6H_5CH_3$) Energy = -2567.468047
SCF (BS2) Energy = -2570.017556

Mg	1.78070	-0.26209	0.79169
N	1.08043	1.31162	-0.35147
N	0.55189	-1.69729	-0.04711
C	0.11465	1.16626	-1.28092
C	-0.45875	2.31668	-2.19329
C	0.15324	3.73171	-1.98525
H	0.00776	4.06488	-0.93975
H	1.24724	3.70676	-2.15283
C	-1.99993	2.45514	-1.95141
H	-2.50558	1.47716	-2.08815
H	-2.17691	2.75347	-0.89434
C	-0.22454	1.94394	-3.69693
H	0.86935	1.87562	-3.88794
H	-0.64277	0.94076	-3.91890
C	-0.47845	4.77778	-2.93712
H	0.00134	5.76104	-2.73758
C	-1.99480	4.87584	-2.67283
H	-2.45739	5.63546	-3.34175
H	-2.18283	5.21049	-1.62839
C	-2.63116	3.49092	-2.91010
H	-3.72494	3.53884	-2.71175
C	-2.38764	3.05436	-4.37286
H	-2.86791	3.77424	-5.07212
H	-2.85837	2.06343	-4.56110
C	-0.86759	2.98242	-4.64423
H	-0.69033	2.66302	-5.69521
C	-0.23387	4.36778	-4.40376
H	0.85768	4.33246	-4.61701
H	-0.67212	5.12111	-5.09580
C	-0.49310	-0.09830	-1.53438

H	-1.25778	-0.04284	-2.31325
C	-0.34164	-1.41834	-1.01694
C	-1.31491	-2.45812	-1.69186
C	-1.05344	-2.49107	-3.23585
H	-1.11743	-1.46991	-3.66457
H	-0.01455	-2.84333	-3.42127
C	-2.79546	-2.00719	-1.44948
H	-3.00245	-2.01231	-0.35641
H	-2.94409	-0.96187	-1.79015
C	-1.20855	-3.92739	-1.19195
H	-0.18331	-4.31427	-1.34907
H	-1.39495	-3.97854	-0.10194
C	-2.06431	-3.40906	-3.96061
H	-1.84906	-3.38320	-5.05194
C	-3.50047	-2.89870	-3.70287
H	-3.61633	-1.86383	-4.09604
H	-4.23524	-3.53172	-4.24811
C	-3.79482	-2.92859	-2.18569
H	-4.82655	-2.55687	-1.99772
C	-3.65457	-4.37234	-1.66149
H	-3.88541	-4.40952	-0.57359
H	-4.38501	-5.04207	-2.16784
C	-2.21277	-4.85665	-1.91823
H	-2.08551	-5.88543	-1.51554
C	-1.92719	-4.85218	-3.43401
H	-2.63443	-5.52810	-3.96451
H	-0.90282	-5.23787	-3.63420
C	1.78025	2.49559	0.05251
C	3.05892	2.79223	-0.51547
C	3.66386	1.94485	-1.63893
H	2.88964	1.21153	-1.95030
C	4.03492	2.77871	-2.88341
H	3.16618	3.34710	-3.27511
H	4.40484	2.12021	-3.69753
H	4.84018	3.51084	-2.66160
C	4.88835	1.14894	-1.13463
H	5.71468	1.83089	-0.84124
H	5.27534	0.47355	-1.92685
H	4.64579	0.53502	-0.24161
C	3.79067	3.88354	-0.00475
H	4.77339	4.12282	-0.44269
C	3.29962	4.66682	1.04869
H	3.88702	5.51660	1.43087
C	2.05865	4.35037	1.61952
H	1.68037	4.95667	2.45843
C	1.28421	3.27178	1.14751
C	-0.04210	2.93786	1.83691
H	-0.56051	2.18468	1.20677
C	-0.98060	4.15551	1.96436
H	-0.55977	4.93363	2.63584
H	-1.95833	3.84916	2.39247
H	-1.17466	4.63225	0.98124
C	0.20499	2.29536	3.22012
H	0.86721	1.40580	3.15503
H	-0.75135	1.98098	3.68935
H	0.70137	3.01155	3.90894
C	0.85432	-2.95239	0.57608
C	1.90801	-3.76650	0.05268
C	2.65236	-3.39212	-1.23243
H	2.08058	-2.57305	-1.71761
C	2.73769	-4.55441	-2.24303
H	3.35381	-5.39405	-1.85727
H	3.20820	-4.21254	-3.18898
H	1.73527	-4.96148	-2.48996
C	4.06190	-2.84740	-0.91081
H	4.02838	-1.99738	-0.19625
H	4.57609	-2.50345	-1.83326
H	4.69406	-3.63093	-0.44155
C	2.28712	-4.92330	0.76270
H	3.09474	-5.55810	0.36373
C	1.66331	-5.28192	1.96598
H	1.97467	-6.19106	2.50422
C	0.64960	-4.46479	2.48427
H	0.17018	-4.73898	3.43810
C	0.23184	-3.29521	1.81714
C	-0.85031	-2.41645	2.45170
H	-1.09197	-1.61442	1.72195
C	-2.15585	-3.18860	2.73635
H	-2.55882	-3.67153	1.82218
H	-2.93589	-2.50345	3.13054
H	-2.00427	-3.98520	3.49537
C	-0.32934	-1.73786	3.73822
H	-0.10245	-2.49043	4.52310
H	-1.08657	-1.03847	4.15175
H	0.60776	-1.16990	3.55772
C	-8.18930	0.51164	2.63921
H	-9.01337	0.87483	1.98797
H	-8.49316	-0.48377	3.02897
C	-7.90349	1.49557	3.78041
H	-7.04870	1.12232	4.39020
H	-7.56438	2.46938	3.35789
C	-9.10892	1.74063	4.70245
H	-9.44800	0.76685	5.12498
H	-9.96376	2.11383	4.09273
C	-8.82299	2.72453	5.84360
H	-7.99903	2.36130	6.49492
H	-9.71374	2.87713	6.48796
H	-8.51929	3.72002	5.45390
C	3.94726	0.78471	2.77384
C	3.25730	-0.36894	2.31103
C	4.93598	0.74848	3.78412
H	-7.29855	0.35892	1.99474
C	3.61485	-1.59883	2.92534
C	5.25295	-0.50173	4.36017
H	3.12158	-2.53905	2.61965
H	6.02163	-0.56049	5.15041
C	4.59678	-1.66678	3.93335
H	4.85291	-2.63787	4.39057
H	3.71697	1.77411	2.33568
C	5.63877	2.00916	4.24270
H	5.45778	2.20877	5.32164
H	6.74055	1.93353	4.11398
H	5.29229	2.89538	3.67370

5-*m*

SCF (BP86) Energy = -2409.096229
Enthalpy 0K = -2407.989142
Enthalpy 298K = -2407.931035
Free Energy 298K = -2408.083432
Lowest Frequency = 4.3888 cm^{-1}
Second Frequency = 19.9103 cm^{-1}
SCF (BP86-D3^{BJ}) Energy = -2409.437600
SCF (C_6H_6) Energy = -2409.135872
SCF ($C_6H_5CH_3$) Energy = -2409.134972
SCF (BS2) Energy = -2411.511044

Mg	-0.58423	-1.46787	0.00003
N	-1.43698	0.41501	0.00002

N	1.37681	-0.81517	-0.00001
C	-0.70542	1.54760	-0.00002
C	-1.28668	3.01281	0.00001
C	-2.83692	3.13858	0.00003
H	-3.26489	2.63061	-0.88550
H	-3.26488	2.63055	0.88553
C	-0.77536	3.77287	-1.27065
H	0.33140	3.72787	-1.33230
H	-1.16023	3.26055	-2.18012
C	-0.77532	3.77281	1.27068
H	-1.16017	3.26044	2.18014
H	0.33144	3.72780	1.33230
C	-3.29473	4.61842	0.00008
H	-4.40670	4.63624	0.00009
C	-2.76323	5.33074	-1.26042
H	-3.09850	6.39178	-1.27741
H	-3.17292	4.84975	-2.17639
C	-1.22287	5.25274	-1.26252
H	-0.82046	5.75097	-2.17236
C	-0.66819	5.95120	0.00007
H	-0.95486	7.02622	0.00010
H	0.44438	5.91716	0.00005
C	-1.22283	5.25268	1.26264
H	-0.82039	5.75087	2.17250
C	-2.76320	5.33068	1.26059
H	-3.17286	4.84964	2.17655
H	-3.09847	6.39171	1.27765
C	0.71994	1.51530	-0.00006
H	1.15800	2.51656	-0.00009
C	1.71159	0.49053	-0.00004
C	3.18167	1.05854	-0.00006
C	3.39200	1.95004	1.27068
H	2.60700	2.73153	1.33264
H	3.27767	1.31951	2.18011
C	3.39198	1.94997	-1.27085
H	3.27765	1.31938	-2.18025
H	2.60699	2.73145	-1.33286
C	4.32707	0.00606	-0.00003
H	4.24449	-0.65315	0.88539
H	4.24448	-0.65321	-0.88541
C	4.78188	2.62685	1.26241
H	4.87428	3.26060	2.17225
C	4.91754	3.50844	-0.00014
H	4.13657	4.30156	-0.00016
H	5.90115	4.02841	-0.00016
C	4.78187	2.62678	-1.26263
H	4.87426	3.26048	-2.17252
C	5.88545	1.54942	-1.26058
H	5.81079	0.92205	-2.17656
H	6.89184	2.02417	-1.27768
C	5.72401	0.67529	-0.00006
H	6.49256	-0.12851	-0.00005
C	5.88547	1.54949	1.26040
H	6.89186	2.02424	1.27746
H	5.81082	0.92217	2.17642
C	-2.86134	0.25408	0.00001
C	-3.55484	0.08299	1.23944
C	-2.83605	0.18069	2.58841
H	-1.81022	0.55648	2.38823
C	-3.50904	1.17447	3.55800
H	-3.60742	2.18552	3.11136
H	-2.91399	1.27137	4.49057
H	-4.52609	0.83886	3.85216
C	-2.70357	-1.21223	3.24361
H	-3.70128	-1.63084	3.49498
H	-2.11463	-1.15478	4.18351
H	-2.21076	-1.94452	2.56932
C	-4.93262	-0.21283	1.20993
H	-5.47506	-0.33932	2.16094
C	-5.62638	-0.35360	-0.00003
H	-6.70389	-0.58205	-0.00005
C	-4.93263	-0.21258	-1.20998
H	-5.47508	-0.33888	-2.16100
C	-3.55486	0.08325	-1.23944
C	-2.83604	0.18119	-2.58838
H	-1.81036	0.55738	-2.38817
C	-3.50935	1.17466	-3.55807
H	-4.52621	0.83862	-3.85237
H	-2.91421	1.27181	-4.49056
H	-3.60822	2.18568	-3.11145
C	-2.70299	-1.21171	-3.24350
H	-2.20994	-1.94378	-2.56915
H	-2.11402	-1.15409	-4.18338
H	-3.70053	-1.63071	-3.49488
C	2.22513	-1.97115	0.00005
C	2.56836	-2.59750	1.23944
C	2.15395	-2.00266	2.58864
H	1.73109	-0.99533	2.38842
C	3.34251	-1.81924	3.55530
H	3.78968	-2.79291	3.84774
H	3.01087	-1.31765	4.48890
H	4.14900	-1.20297	3.10679
C	1.04433	-2.85222	3.24750
H	0.17153	-2.99099	2.57473
H	0.68717	-2.37955	4.18698
H	1.41674	-3.86765	3.50055
C	3.28139	-3.81304	1.20997
H	3.55398	-4.29862	2.16107
C	3.64582	-4.41999	0.00018
H	4.20303	-5.37010	0.00023
C	3.28135	-3.81320	-1.20968
H	3.55391	-4.29890	-2.16072
C	2.56831	-2.59767	-1.23928
C	2.15387	-2.00301	-2.58855
H	1.73081	-0.99575	-2.38843
C	3.34245	-1.81945	-3.55515
H	4.14878	-1.20297	-3.10665
H	3.01078	-1.31804	-4.48884
H	3.78984	-2.79307	-3.84744
C	1.04444	-2.85284	-3.24741
H	1.41708	-3.86821	-3.50039
H	0.68724	-2.38029	-4.18693
H	0.17165	-2.99173	-2.57466
C	-2.83976	-3.62095	-0.00013
C	-1.43352	-3.41202	0.00003
C	-3.43723	-4.90256	-0.00020
C	-0.62833	-4.58231	0.00012
C	-2.58891	-6.03206	-0.00013
H	0.47355	-4.50089	0.00024
H	-3.02745	-7.04521	-0.00020
C	-1.19443	-5.87245	0.00003
H	-0.54172	-6.76219	0.00008
H	-3.52361	-2.75130	-0.00020
C	-4.94252	-5.06873	-0.00028
H	-5.29166	-5.63467	-0.89151
H	-5.29188	-5.63341	0.89165
H	-5.45787	-4.08710	-0.00101

TS(C-D^P)

SCF (BP86) Energy = -2567.353525
Enthalpy 0K = -2566.121692
Enthalpy 298K = -2566.057369
Free Energy 298K = -2566.220362
Lowest Frequency = -1289.3296 cm^{-1}
Second Frequency = 11.3730 cm^{-1}
SCF (BP86-D3^{BJ}) Energy = -2567.730356
SCF (C_6H_6) Energy = -2567.393787
SCF ($C_6H_5CH_3$) Energy = -2567.392683
SCF (BS2) Energy = -2569.945120

Mg	-0.99958	-0.87172	0.39370
N	0.91712	-1.34322	-0.25280
N	-0.99979	1.19759	0.15144
C	1.79906	-0.37248	0.11614
C	3.33670	-0.62158	0.40971
C	3.89299	-2.02326	0.02376
H	3.27872	-2.82098	0.47978
H	3.82970	-2.17280	-1.07109
C	3.51512	-0.46640	1.96264
H	3.14489	0.52827	2.28815
H	2.88195	-1.21878	2.48196
C	4.26448	0.42986	-0.28220
H	4.17590	0.33323	-1.38516
H	3.94909	1.46368	-0.03287
C	5.36700	-2.20002	0.46503
H	5.69543	-3.22047	0.16889
C	5.48833	-2.03875	1.99412
H	6.54406	-2.18093	2.31633
H	4.88608	-2.81722	2.51260
C	4.99164	-0.63231	2.38707
H	5.06030	-0.50079	3.48985
C	5.85317	0.43810	1.68055
H	6.91513	0.34870	2.00031
H	5.51537	1.45732	1.97394
C	5.74077	0.25816	0.14992
H	6.35152	1.03397	-0.36310
C	6.24497	-1.14628	-0.23983
H	6.19531	-1.28085	-1.34323
H	7.31214	-1.26905	0.05127
C	1.40210	0.96587	0.39153
H	2.25273	1.58092	0.69600
C	0.21787	1.76299	0.21898
C	0.54005	3.30375	0.11768
C	1.54709	3.52449	-1.06375
H	2.43729	2.87677	-0.93550
H	1.06441	3.20665	-2.01427
C	1.22155	3.79545	1.44017
H	0.51169	3.67383	2.28679
H	2.10614	3.17130	1.68359
C	-0.66619	4.25020	-0.14874
H	-1.17985	3.95972	-1.08517
H	-1.41972	4.15930	0.65504
C	1.99618	5.00019	-1.16063
H	2.72127	5.09608	-1.99906
C	2.67535	5.41826	0.16334
H	3.57326	4.78751	0.35000
H	3.03200	6.46999	0.09616
C	1.66928	5.27164	1.32741
H	2.15702	5.56730	2.28273
C	0.44403	6.17152	1.06464
H	-0.27825	6.09404	1.90738
H	0.75345	7.23888	1.00549
C	-0.22322	5.73086	-0.25424
H	-1.12886	6.34882	-0.44061
C	0.77182	5.90130	-1.41988
H	1.08417	6.96554	-1.51164
H	0.28817	5.62416	-2.38287
C	1.23019	-2.48635	-1.05531
C	1.71646	-2.31500	-2.39541
C	1.86040	-0.94159	-3.05997
H	1.82547	-0.16419	-2.27034
C	3.18522	-0.76006	-3.82938
H	4.06887	-0.99225	-3.20033
H	3.28574	0.28714	-4.18468
H	3.23692	-1.41182	-4.72714
C	0.66096	-0.69614	-4.00058
H	0.66594	-1.41829	-4.84507
H	0.69038	0.32843	-4.42913
H	-0.30064	-0.82095	-3.46578
C	1.99401	-3.45434	-3.17805
H	2.37333	-3.31811	-4.20339
C	1.78952	-4.74940	-2.69098
H	2.02231	-5.62677	-3.31489
C	1.25536	-4.91297	-1.40660
H	1.06720	-5.93067	-1.03430
C	0.95349	-3.81330	-0.57720
C	0.35729	-4.07940	0.81082
H	-0.36888	-3.26482	1.01909
C	1.40308	-4.02238	1.94475
H	2.20433	-4.77507	1.78361
H	0.92679	-4.23805	2.92427
H	1.87752	-3.02514	2.01809
C	-0.42888	-5.40164	0.90728
H	-1.17410	-5.50941	0.09348
H	-0.97503	-5.44435	1.87190
H	0.24083	-6.28743	0.87793
C	-2.27540	1.83563	-0.03592
C	-2.83219	1.94245	-1.34965
C	-2.10523	1.45065	-2.60427
H	-1.12303	1.04363	-2.28374
C	-1.82840	2.58826	-3.61126
H	-2.77213	3.02308	-4.00409
H	-1.25406	2.20632	-4.48151
H	-1.24480	3.41518	-3.15720
C	-2.89131	0.31064	-3.28847
H	-3.05138	-0.54751	-2.60521
H	-2.35108	-0.05536	-4.18642
H	-3.89080	0.65898	-3.62467
C	-4.11034	2.51649	-1.50490
H	-4.53226	2.61273	-2.51845
C	-4.85460	2.96461	-0.40746
H	-5.84783	3.41828	-0.55249
C	-4.32561	2.80948	0.87948
H	-4.91763	3.14047	1.74813
C	-3.05397	2.23944	1.09809
C	-2.58207	2.08193	2.54880
H	-1.60665	1.55072	2.52735
C	-2.36419	3.43604	3.26018
H	-1.63103	4.07692	2.73179
H	-1.99012	3.27680	4.29365
H	-3.31319	4.00865	3.33450
C	-3.57233	1.22917	3.37325
H	-4.54325	1.75244	3.50343
H	-3.16644	1.02964	4.38721
H	-3.77945	0.25258	2.89266
C	-1.57438	-1.66159	2.51552

H	-0.50582	-1.91414	2.72787
H	-1.72893	-0.64570	2.94773
C	-2.47344	-2.66074	3.26546
H	-3.54502	-2.40778	3.08415
H	-2.35048	-3.67942	2.82674
C	-2.23781	-2.74777	4.78496
H	-2.36929	-1.73423	5.22882
H	-1.17195	-3.01499	4.97088
C	-3.15505	-3.74861	5.50025
H	-4.22613	-3.48438	5.36459
H	-2.95692	-3.78531	6.59223
H	-3.02008	-4.77786	5.10267
C	-2.79999	-3.08413	-1.03371
C	-2.92820	-2.02148	-0.10045
C	-3.86479	-3.47274	-1.86540
H	-2.25361	-1.92369	1.14341
C	-4.19379	-1.38397	-0.04014
C	-5.12038	-2.82843	-1.78911
H	-4.35165	-0.55294	0.67129
C	-5.26499	-1.77217	-0.86154
H	-6.23250	-1.24641	-0.78755
H	-1.83957	-3.61982	-1.13762
H	-3.72230	-4.29272	-2.59062
C	-6.28041	-3.27643	-2.64954
H	-6.98348	-2.44378	-2.85615
H	-5.93606	-3.68891	-3.61999
H	-6.86508	-4.07805	-2.14536

D^{P}

SCF (BP86) Energy = -2567.425355
Enthalpy 0K = -2566.190434
Enthalpy 298K = -2566.123690
Free Energy 298K = -2566.309201
Lowest Frequency = 1.9225 cm^{-1}
Second Frequency = 1.9708 cm^{-1}
SCF (BP86-D3^{BJ}) Energy = -2567.778784
SCF (C_6H_6) Energy = -2567.469295
SCF ($C_6H_5CH_3$) Energy = -2567.468164
SCF (BS2) Energy = -2570.017858

Mg	1.99053	0.04569	0.57066
N	0.84197	1.53447	-0.28751
N	0.89662	-1.53418	-0.19032
C	-0.23820	1.27454	-1.05100
C	-1.16016	2.35698	-1.73117
C	-0.76514	3.84326	-1.49727
H	-0.75588	4.07390	-0.41468
H	0.26296	4.03080	-1.86249
C	-2.62554	2.19329	-1.20271
H	-2.97547	1.14996	-1.34423
H	-2.63998	2.38223	-0.10644
C	-1.16523	2.13385	-3.28176
H	-0.13436	2.28069	-3.67353
H	-1.44477	1.08811	-3.52419
C	-1.73746	4.81618	-2.20934
H	-1.39976	5.85541	-2.00256
C	-3.16721	4.61651	-1.66662
H	-3.87231	5.32119	-2.16132
H	-3.20047	4.84085	-0.57723
C	-3.59680	3.15721	-1.92246
H	-4.62288	2.99024	-1.52591
C	-3.57417	2.87105	-3.44143
H	-4.29522	3.53560	-3.96710
H	-3.90009	1.82540	-3.63986
C	-2.14631	3.09701	-3.98850
H	-2.12705	2.88590	-5.08078
C	-1.71924	4.55623	-3.72950
H	-0.70034	4.73616	-4.13897
H	-2.40448	5.26034	-4.25217
C	-0.65732	-0.06139	-1.32100
H	-1.54861	-0.09725	-1.95256
C	-0.18909	-1.36179	-0.97055
C	-1.06336	-2.51756	-1.59014
C	-1.06154	-2.38520	-3.15124
H	-1.37993	-1.36725	-3.45592
H	-0.02165	-2.51250	-3.52563
C	-2.53919	-2.38196	-1.08378
H	-2.55766	-2.50732	0.02148
H	-2.92882	-1.36358	-1.28896
C	-0.61245	-3.97086	-1.26678
H	0.42606	-4.13789	-1.61203
H	-0.60556	-4.13831	-0.17257
C	-1.99642	-3.42614	-3.80852
H	-1.97439	-3.27794	-4.91104
C	-3.43751	-3.22602	-3.28660
H	-3.80233	-2.20748	-3.54823
H	-4.12657	-3.94818	-3.77809
C	-3.46413	-3.42422	-1.75372
H	-4.49999	-3.27587	-1.37567
C	-2.97987	-4.84797	-1.40989
H	-3.01507	-5.00991	-0.30956
H	-3.65171	-5.60768	-1.86807
C	-1.53807	-5.02145	-1.92900
H	-1.16176	-6.03263	-1.65932
C	-1.51472	-4.84962	-3.46152
H	-2.16627	-5.60980	-3.94742
H	-0.48540	-5.01211	-3.85177
C	1.41061	2.79739	0.08293
C	2.47751	3.34864	-0.69433
C	2.96102	2.68767	-1.98864
H	2.24386	1.87491	-2.23101
C	2.97987	3.65763	-3.18845
H	1.98831	4.12644	-3.35682
H	3.26415	3.12095	-4.11817
H	3.71633	4.47649	-3.04474
C	4.35049	2.04172	-1.79095
H	5.12105	2.81182	-1.57401
H	4.66774	1.49433	-2.70380
H	4.36120	1.33014	-0.93822
C	3.11849	4.51661	-0.23406
H	3.93764	4.94998	-0.83063
C	2.74160	5.13518	0.96592
H	3.25578	6.04764	1.30685
C	1.71337	4.57280	1.73482
H	1.42774	5.05039	2.68610
C	1.03809	3.40625	1.32268
C	-0.04594	2.80795	2.22424
H	-0.53544	1.99108	1.65262
C	-1.14210	3.82556	2.60314
H	-0.73917	4.65259	3.22541
H	-1.94338	3.33247	3.19306
H	-1.61165	4.28071	1.70663
C	0.57641	2.18284	3.49270
H	1.36360	1.43889	3.24661
H	-0.19837	1.67840	4.10822
H	1.05744	2.95937	4.12476
C	1.50043	-2.74944	0.27218
C	2.59855	-3.31495	-0.44911

C	3.07924	-2.72982	-1.78043
H	2.33745	-1.96299	-2.08930
C	3.15086	-3.78091	-2.90784
H	3.91276	-4.56073	-2.69604
H	3.43145	-3.30144	-3.86939
H	2.17893	-4.29545	-3.05598
C	4.44191	-2.02169	-1.61119
H	4.41439	-1.25183	-0.81100
H	4.75392	-1.52923	-2.55654
H	5.23548	-2.74622	-1.32986
C	3.27150	-4.42468	0.10096
H	4.11517	-4.86871	-0.45195
C	2.89579	-4.97173	1.33550
H	3.43567	-5.83912	1.74703
C	1.83459	-4.39555	2.04744
H	1.54869	-4.81663	3.02495
C	1.12630	-3.28557	1.54465
C	0.00524	-2.66559	2.38400
H	-0.51084	-1.91909	1.74384
C	-1.05147	-3.69628	2.83267
H	-1.48598	-4.24440	1.97117
H	-1.88289	-3.19223	3.36918
H	-0.62383	-4.45030	3.52698
C	0.58318	-1.91384	3.60374
H	1.09138	-2.61484	4.29957
H	-0.22066	-1.39768	4.17033
H	1.33917	-1.15632	3.30627
C	-8.67638	-0.74606	2.39475
H	-9.59347	-0.49286	1.82036
H	-8.74014	-1.82422	2.65666
C	-8.54295	0.12702	3.64857
H	-7.59604	-0.12424	4.17965
H	-8.44182	1.19597	3.35034
C	-9.72006	-0.01218	4.62751
H	-9.82134	-1.08115	4.92556
H	-10.66692	0.23928	4.09649
C	-9.58640	0.86064	5.88145
H	-8.66961	0.60684	6.45604
H	-10.45238	0.73496	6.56425
H	-9.52223	1.93883	5.61979
C	4.30897	1.34190	2.20705
C	3.70407	0.11618	1.81630
C	5.44748	1.39424	3.03343
H	-7.81054	-0.62016	1.71171
C	4.32955	-1.05843	2.31729
C	6.04854	0.21442	3.52175
H	3.92654	-2.05419	2.05737
C	5.46762	-1.01554	3.14414
H	5.91766	-1.95822	3.50312
H	3.88878	2.30280	1.85833
H	5.88140	2.37318	3.30425
C	7.25175	0.26527	4.43825
H	7.91878	-0.60907	4.28891
H	7.85050	1.18566	4.27882
H	6.94534	0.25835	5.50851

5-*p*

SCF (BP86) Energy = -2409.096436
Enthalpy 0K = -2407.989407
Enthalpy 298K = -2407.931227
Free Energy 298K = -2408.084671
Lowest Frequency = 5.7421 cm^{-1}
Second Frequency = 11.0106 cm^{-1}
SCF (BP86-D3^{BJ}) Energy = -2409.436930
SCF (C_6H_6) Energy = -2409.136275
SCF ($C_6H_5CH_3$) Energy = -2409.135377
SCF (BS2) Energy = -2411.511317

Mg	0.11604	1.54316	0.00534
N	1.54276	0.04794	0.00506
N	-1.51860	0.27818	0.00497
C	1.22752	-1.26271	0.00269
C	2.26080	-2.45259	-0.01845
C	3.76579	-2.05880	-0.01567
H	3.99776	-1.41620	-0.88665
H	4.00583	-1.45952	0.88366
C	2.02314	-3.31072	-1.30746
H	0.96288	-3.63110	-1.37042
H	2.21264	-2.67963	-2.20385
C	2.03534	-3.36663	1.23336
H	2.23415	-2.77653	2.15531
H	0.97645	-3.69191	1.29369
C	4.68653	-3.30396	-0.04950
H	5.74202	-2.95363	-0.04698
C	4.41367	-4.12338	-1.32737
H	5.08093	-5.01312	-1.36782
H	4.63653	-3.51354	-2.23105
C	2.93417	-4.55926	-1.33169
H	2.71457	-5.14225	-2.25358
C	2.64776	-5.42939	-0.08657
H	3.27357	-6.34902	-0.10967
H	1.58643	-5.76489	-0.08882
C	2.94661	-4.61494	1.19276
H	2.73602	-5.23828	2.09004
C	4.42637	-4.17986	1.19305
H	4.65883	-3.61106	2.12075
H	5.09316	-5.07085	1.18684
C	-0.12832	-1.70415	0.00737
H	-0.21036	-2.79402	0.00779
C	-1.40284	-1.06486	0.00857
C	-2.60277	-2.08661	0.02239
C	-2.50150	-2.97899	1.30616
H	-1.50190	-3.45598	1.37003
H	-2.59756	-2.33257	2.20654
C	-2.51244	-3.01625	-1.23481
H	-2.61684	-2.39740	-2.15344
H	-1.51408	-3.49637	-1.29405
C	-4.03137	-1.47121	0.01890
H	-4.16679	-0.80685	0.89400
H	-4.17557	-0.83676	-0.87668
C	-3.58998	-4.07648	1.31914
H	-3.46384	-4.69176	2.23758
C	-3.43310	-4.97199	0.06890
H	-2.43438	-5.46344	0.07176
H	-4.19029	-5.78703	0.08421
C	-3.60115	-4.11358	-1.20567
H	-3.48319	-4.75559	-2.10672
C	-4.99861	-3.46075	-1.20688
H	-5.13935	-2.85732	-2.13114
H	-5.79191	-4.24123	-1.20880
C	-5.12889	-2.56383	0.04117
H	-6.11972	-2.05881	0.03815
C	-4.98724	-3.42340	1.31394
H	-5.78073	-4.20303	1.34636
H	-5.11933	-2.79287	2.22125
C	2.83273	0.67259	0.03240
C	3.42556	1.01595	1.28865
C	2.77111	0.63183	2.61899

H	1.96891	-0.10165	2.39217
C	3.74813	-0.04474	3.60227
H	4.24062	-0.93087	3.15088
H	3.21032	-0.38192	4.51339
H	4.54861	0.64965	3.93466
C	2.10394	1.86108	3.27545
H	2.86111	2.62680	3.54766
H	1.56280	1.57368	4.20183
H	1.38137	2.35850	2.59366
C	4.62359	1.75792	1.29273
H	5.08875	2.02183	2.25624
C	5.23390	2.17096	0.09993
H	6.17089	2.74938	0.12606
C	4.63246	1.85267	-1.12505
H	5.10411	2.19090	-2.06205
C	3.43318	1.11413	-1.18815
C	2.79885	0.83217	-2.55354
H	1.92308	0.17127	-2.37871
C	3.75246	0.08836	-3.51237
H	4.63932	0.70578	-3.76913
H	3.23545	-0.15625	-4.46430
H	4.12301	-0.86113	-3.07377
C	2.28193	2.13440	-3.20459
H	1.58799	2.68563	-2.53540
H	1.75079	1.91782	-4.15564
H	3.12040	2.82475	-3.43768
C	-2.70052	1.08888	-0.02605
C	-3.23372	1.61233	1.19332
C	-2.65428	1.23478	2.55994
H	-1.89222	0.44518	2.38666
C	-3.71393	0.64962	3.51727
H	-4.49294	1.39847	3.77415
H	-3.24259	0.32607	4.46933
H	-4.22869	-0.22954	3.07753
C	-1.94125	2.44058	3.21151
H	-1.16858	2.87646	2.54347
H	-1.45196	2.14441	4.16357
H	-2.66193	3.25370	3.44252
C	-4.30821	2.52263	1.12757
H	-4.72830	2.92533	2.06360
C	-4.84915	2.93094	-0.09901
H	-5.68834	3.64379	-0.12727
C	-4.30234	2.43384	-1.29045
H	-4.71789	2.76725	-2.25510
C	-3.22947	1.52010	-1.28369
C	-2.63545	1.04439	-2.61279
H	-1.94655	0.20406	-2.38410
C	-3.69895	0.51262	-3.59543
H	-4.31179	-0.29462	-3.14344
H	-3.21477	0.10227	-4.50661
H	-4.39319	1.31333	-3.92758
C	-1.79888	2.16410	-3.27095
H	-2.43813	3.03023	-3.54469
H	-1.30471	1.80044	-4.19678
H	-1.01241	2.55310	-2.58950
C	1.52893	4.32349	0.08594
C	0.27612	3.65661	0.00355
C	1.64040	5.72679	0.08814
C	-0.86164	4.50569	-0.07618
C	0.49641	6.54899	0.00345
H	-1.87514	4.06901	-0.13664
C	-0.75954	5.90942	-0.07602
H	-1.67558	6.52365	-0.13621
H	2.46414	3.73852	0.15470
H	2.63806	6.19544	0.15896
C	0.61260	8.05751	-0.03046
H	-0.26504	8.54755	0.43986
H	1.52503	8.41071	0.49282
H	0.67143	8.43503	-1.07613

TS(1^{Ad}–E^{Ad})

SCF (BP86) Energy = -2295.990857
Enthalpy 0K = -2294.885463
Enthalpy 298K = -2294.829240
Free Energy 298K = -2294.973150
Lowest Frequency = -542.3557 cm^{-1}
Second Frequency = 8.7087 cm^{-1}
SCF (BP86-$D3^{BJ}$) Energy = -2296.323454
SCF (C_6H_6) Energy = -2296.026044
SCF (BS2) Energy = -2298.293780

Mg	0.05430	-1.90986	-0.15499
N	-1.54964	-0.58353	0.04908
N	1.56977	-0.49458	-0.00799
C	-1.35433	0.74539	-0.05392
C	-2.48633	1.85017	-0.07170
C	-2.20701	2.91705	1.04317
H	-2.23999	2.42544	2.03977
H	-1.18408	3.33148	0.94092
C	-3.94367	1.34997	0.14765
H	-4.21840	0.61269	-0.62748
H	-4.03161	0.82203	1.11687
C	-2.46191	2.58399	-1.45605
H	-1.44537	2.97744	-1.66535
H	-2.68680	1.85332	-2.26287
C	-3.22308	4.08030	0.99109
H	-2.96699	4.81023	1.79095
C	-4.64897	3.53738	1.21429
H	-5.38861	4.36885	1.19821
H	-4.72487	3.05795	2.21539
C	-4.96556	2.51370	0.10584
H	-5.98048	2.08807	0.26633
C	-4.90560	3.20831	-1.27079
H	-5.64568	4.03815	-1.31891
H	-5.17663	2.49132	-2.07697
C	-3.47840	3.74931	-1.49622
H	-3.41447	4.24157	-2.49207
C	-3.13862	4.77165	-0.38848
H	-2.11802	5.18630	-0.54768
H	-3.84362	5.63142	-0.43119
C	-0.04582	1.30783	-0.17264
H	-0.07728	2.39210	-0.30326
C	1.29185	0.82059	-0.10397
C	2.36785	1.96973	-0.19271
C	2.13820	3.02245	0.94179
H	1.09359	3.39493	0.92301
H	2.27644	2.53104	1.92991
C	2.21105	2.69116	-1.57511
H	2.40292	1.95947	-2.39094
H	1.16818	3.04456	-1.71140
C	3.85142	1.51892	-0.09089
H	4.03529	1.00992	0.87450
H	4.08061	0.77924	-0.88039
C	3.10489	4.22157	0.80394
H	2.89176	4.94658	1.62061
C	2.88652	4.90397	-0.56581
H	1.84179	5.27975	-0.64493
H	3.55304	5.78955	-0.66336

C	3.17607	3.89262	-1.69837
H	3.01359	4.37988	-2.68541
C	4.63398	3.40086	-1.59064
H	4.86002	2.68842	-2.41483
H	5.34074	4.25356	-1.70001
C	4.82735	2.71467	-0.22279
H	5.86614	2.32541	-0.14215
C	4.56339	3.73026	0.90783
H	5.26858	4.58789	0.83178
H	4.73914	3.25715	1.89949
C	-2.77190	-1.32107	0.16286
C	-3.17966	-1.80827	1.45003
C	-2.40046	-1.48081	2.73104
H	-1.35657	-1.25132	2.42107
C	-2.93904	-0.22958	3.45861
H	-2.86879	0.67895	2.83104
H	-2.36217	-0.03914	4.38858
H	-4.00481	-0.36496	3.74229
C	-2.35191	-2.65838	3.72739
H	-3.33255	-2.81917	4.22352
H	-1.61546	-2.44991	4.53107
H	-2.06651	-3.61393	3.24196
C	-4.33474	-2.61284	1.53610
H	-4.66594	-2.97242	2.52270
C	-5.06593	-2.97470	0.39718
H	-5.96468	-3.60491	0.48945
C	-4.62801	-2.54379	-0.86130
H	-5.18839	-2.84858	-1.76012
C	-3.48943	-1.72606	-1.01228
C	-3.07611	-1.31140	-2.43106
H	-2.23116	-0.59669	-2.33700
C	-4.21320	-0.60232	-3.20042
H	-5.05703	-1.29402	-3.40796
H	-3.84450	-0.23400	-4.18098
H	-4.62454	0.26519	-2.64595
C	-2.57348	-2.51941	-3.25209
H	-1.70219	-2.99906	-2.75932
H	-2.25953	-2.19558	-4.26761
H	-3.37374	-3.28052	-3.37530
C	2.80658	-1.16179	0.26740
C	3.21360	-1.36940	1.62611
C	2.52103	-0.67496	2.80525
H	1.77322	0.02880	2.38404
C	3.51242	0.15117	3.65440
H	4.25097	-0.49897	4.16974
H	2.97161	0.72087	4.43957
H	4.08239	0.87761	3.03968
C	1.75772	-1.66925	3.70602
H	0.98821	-2.22587	3.13692
H	1.25213	-1.13460	4.53814
H	2.44625	-2.41537	4.15743
C	4.29951	-2.23042	1.88394
H	4.61345	-2.39886	2.92722
C	4.98863	-2.87669	0.84823
H	5.82804	-3.55302	1.07481
C	4.60777	-2.63928	-0.47925
H	5.16769	-3.12384	-1.29585
C	3.53310	-1.78389	-0.79945
C	3.21274	-1.51196	-2.27485
H	2.43284	-0.72069	-2.29842
C	4.44242	-0.98293	-3.04734
H	4.89449	-0.09417	-2.56106
H	4.15553	-0.69598	-4.08108
H	5.23604	-1.75546	-3.12980
C	2.63571	-2.74974	-2.99174
H	3.35441	-3.59701	-2.97360
H	2.41643	-2.51871	-4.05612
H	1.68745	-3.08168	-2.51814
C	0.20668	-3.83369	1.12938
H	1.24575	-3.93016	1.48771
H	-0.57550	-3.64602	1.88114
C	-0.15225	-4.37228	-0.11914
H	-1.23406	-4.50748	-0.30983
H	-0.14172	-3.14912	-1.47346
C	0.74162	-5.33679	-0.87880
H	0.78912	-6.24956	-0.23839
H	1.78145	-4.94490	-0.90113
C	0.26886	-5.72998	-2.28065
H	-0.75148	-6.16819	-2.25230
H	0.94464	-6.48522	-2.73195
H	0.23453	-4.85091	-2.95571

E^Ad

SCF (BP86) Energy = -2296.007282
Enthalpy 0K = -2294.901940
Enthalpy 298K = -2294.844292
Free Energy 298K = -2294.991389
Lowest Frequency = 14.8250 cm^{-1}
Second Frequency = 22.8017 cm^{-1}
SCF (BP86-D3^{BJ}) Energy = -2296.340993
SCF (C_6H_6) Energy = -2296.044488
SCF (BS2) Energy = -2298.309978

Mg	-0.02932	-1.69312	-0.92060
N	-1.55271	-0.45632	-0.14436
N	1.57915	-0.64389	-0.07999
C	-1.23534	0.85159	-0.15015
C	-2.24879	2.05645	-0.03857
C	-1.82302	2.99440	1.14129
H	-1.87227	2.42695	2.09629
H	-0.76583	3.30723	1.02455
C	-3.74054	1.68283	0.19670
H	-4.10971	1.03872	-0.62212
H	-3.85062	1.09032	1.12462
C	-2.20001	2.89163	-1.36504
H	-1.15947	3.20317	-1.59182
H	-2.52615	2.25092	-2.21297
C	-2.71871	4.25148	1.22127
H	-2.36546	4.88684	2.06366
C	-4.18408	3.83296	1.45919
H	-4.83654	4.73102	1.53962
H	-4.27486	3.28276	2.42204
C	-4.64124	2.93963	0.28799
H	-5.68758	2.60351	0.45874
C	-4.55986	3.73545	-1.03106
H	-5.21406	4.63455	-0.98319
H	-4.92849	3.11617	-1.87859
C	-3.09388	4.15049	-1.27201
H	-3.01325	4.71326	-2.22849
C	-2.61349	5.04065	-0.10350
H	-1.56249	5.36488	-0.27425
H	-3.22892	5.96603	-0.04924
C	0.12139	1.29031	-0.30759
H	0.18477	2.36963	-0.46863
C	1.41137	0.69347	-0.19747
C	2.58769	1.73065	-0.36806
C	2.45263	2.94433	0.60639
H	1.45218	3.41450	0.51248

H	2.53168	2.58623	1.65548
C	2.50440	2.27287	-1.83880
H	2.62454	1.42232	-2.54558
H	1.49880	2.70089	-2.03189
C	4.02096	1.15848	-0.18596
H	4.15526	0.76550	0.84014
H	4.17467	0.30045	-0.86746
C	3.53330	4.01543	0.32675
H	3.38898	4.86042	1.03615
C	3.38830	4.52643	-1.12479
H	2.38585	4.98767	-1.27090
H	4.13912	5.32211	-1.32794
C	3.58109	3.34891	-2.10713
H	3.46824	3.71137	-3.15307
C	4.98421	2.73891	-1.91204
H	5.14213	1.90354	-2.62983
H	5.77056	3.49752	-2.12373
C	5.10682	2.22727	-0.46201
H	6.10345	1.75634	-0.31372
C	4.93720	3.40543	0.51933
H	5.72162	4.17481	0.34212
H	5.06331	3.05443	1.56775
C	-2.82799	-1.08046	0.04176
C	-3.23959	-1.50619	1.34909
C	-2.40154	-1.25722	2.61033
H	-1.39286	-0.93254	2.27355
C	-2.97839	-0.13240	3.49889
H	-3.03462	0.83647	2.96598
H	-2.34715	0.01645	4.40061
H	-4.00384	-0.38506	3.84428
C	-2.24094	-2.53512	3.46380
H	-3.20599	-2.84707	3.91604
H	-1.53621	-2.35573	4.30208
H	-1.85964	-3.39246	2.87400
C	-4.47020	-2.18166	1.48916
H	-4.80026	-2.48775	2.49486
C	-5.28015	-2.47753	0.38573
H	-6.23892	-3.00274	0.52128
C	-4.84412	-2.11187	-0.89369
H	-5.46793	-2.36189	-1.76734
C	-3.62967	-1.42534	-1.09913
C	-3.23924	-1.07503	-2.54178
H	-2.25703	-0.55678	-2.51117
C	-4.25486	-0.12532	-3.21650
H	-5.24889	-0.60863	-3.32868
H	-3.90637	0.15298	-4.23339
H	-4.40540	0.81155	-2.64349
C	-3.06391	-2.34093	-3.40692
H	-2.28587	-3.00758	-2.98768
H	-2.74070	-2.06439	-4.43266
H	-4.01568	-2.90764	-3.49451
C	2.70161	-1.35327	0.44155
C	2.99524	-1.31364	1.84651
C	2.21411	-0.44162	2.83641
H	1.50982	0.18819	2.25517
C	3.12663	0.50228	3.64948
H	3.80183	-0.06341	4.32615
H	2.51910	1.18124	4.28467
H	3.76483	1.13015	2.99538
C	1.37311	-1.30823	3.79825
H	0.69342	-1.98824	3.24857
H	0.75433	-0.67229	4.46660
H	2.02157	-1.94012	4.44212
C	4.02336	-2.13407	2.35432
H	4.24785	-2.10193	3.43321
C	4.76189	-2.99110	1.52822
H	5.56131	-3.62178	1.94830
C	4.46573	-3.03353	0.15979
H	5.04698	-3.70166	-0.49682
C	3.45040	-2.23610	-0.40689
C	3.21886	-2.32757	-1.91997
H	2.37609	-1.65000	-2.17733
C	4.44988	-1.86040	-2.72873
H	4.75236	-0.82574	-2.46776
H	4.23079	-1.88426	-3.81707
H	5.32745	-2.51792	-2.55024
C	2.81008	-3.74855	-2.35906
H	3.61719	-4.48548	-2.15848
H	2.59002	-3.77026	-3.44663
H	1.89269	-4.08739	-1.83750
C	0.11970	-3.73286	0.81321
H	1.19435	-3.97755	0.75699
H	-0.16173	-2.88708	1.46332
C	-0.82365	-4.48985	0.20432
H	-1.88751	-4.19934	0.31723
H	-0.06692	-2.36713	-2.51345
C	-0.56030	-5.73343	-0.60005
H	-0.96321	-6.59323	-0.01343
H	0.53544	-5.90173	-0.67920
C	-1.21183	-5.73015	-1.99402
H	-2.31157	-5.59497	-1.92537
H	-1.02154	-6.68893	-2.51820
H	-0.80801	-4.90491	-2.61529

TS(E^{Ad}–F^{Ad})

SCF (BP86) Energy = -2296.006821
Enthalpy 0K = -2294.901783
Enthalpy 298K = -2294.844871
Free Energy 298K = -2294.990450
Lowest Frequency = -14.4061 cm^{-1}
Second Frequency = 16.2527 cm^{-1}
SCF (BP86-D3^{BJ}) Energy = -2296.339388
SCF (C_6H_6) Energy = -2296.044246
SCF (BS2) Energy = -2298.309279

Mg	0.09926	1.77549	-0.78001
N	1.55147	0.38597	-0.14854
N	-1.56634	0.68981	-0.10634
C	1.18812	-0.90953	-0.13540
C	2.16188	-2.14506	-0.01293
C	1.73916	-3.02036	1.21566
H	1.84190	-2.41786	2.14487
H	0.66756	-3.29619	1.14344
C	3.67155	-1.81839	0.16721
H	4.03933	-1.21562	-0.68310
H	3.82781	-1.19938	1.07036
C	2.04689	-3.02530	-1.30512
H	0.99047	-3.30671	-1.49417
H	2.37334	-2.42774	-2.18414
C	2.59041	-4.30661	1.31648
H	2.24009	-4.89753	2.19182
C	4.07668	-3.93529	1.49598
H	4.69808	-4.85372	1.59100
H	4.21676	-3.35491	2.43499
C	4.53049	-3.10258	0.27952
H	5.59244	-2.79822	0.40841
C	4.38290	-3.94312	-1.00541
H	5.00586	-4.86316	-0.94139

H	4.74807	-3.36956	-1.88598
C	2.89659	-4.31208	-1.18911
H	2.76770	-4.90704	-2.12049
C	2.41753	-5.13919	0.02565
H	1.35067	-5.42904	-0.10370
H	2.99929	-6.08485	0.09830
C	-0.18266	-1.30476	-0.26254
H	-0.28623	-2.38641	-0.37961
C	-1.44786	-0.65522	-0.18527
C	-2.66124	-1.65415	-0.33609
C	-2.57854	-2.84194	0.67646
H	-1.59414	-3.34914	0.61115
H	-2.65942	-2.44906	1.71328
C	-2.58483	-2.24233	-1.78917
H	-2.67004	-1.40915	-2.52127
H	-1.59257	-2.70904	-1.96018
C	-4.07588	-1.03003	-0.18132
H	-4.20484	-0.60911	0.83407
H	-4.19625	-0.18383	-0.88355
C	-3.69183	-3.88435	0.41616
H	-3.58239	-4.71247	1.15122
C	-3.55039	-4.44235	-1.01841
H	-2.56251	-4.94042	-1.14097
H	-4.32528	-5.21858	-1.20571
C	-3.69504	-3.28853	-2.03653
H	-3.58574	-3.68541	-3.07030
C	-5.07789	-2.62546	-1.87284
H	-5.20049	-1.80657	-2.61609
H	-5.88825	-3.36232	-2.07032
C	-5.19568	-2.06810	-0.43942
H	-6.17658	-1.55887	-0.31469
C	-5.07614	-3.22290	0.57639
H	-5.88436	-3.97024	0.41239
H	-5.20032	-2.83812	1.61308
C	2.85384	0.96858	-0.01639
C	3.32307	1.39508	1.27039
C	2.54673	1.13391	2.56816
H	1.56159	0.70667	2.28281
C	3.25449	0.11352	3.48835
H	3.41454	-0.86255	2.98970
H	2.65213	-0.07097	4.40307
H	4.24869	0.48865	3.81280
C	2.29230	2.43218	3.36420
H	3.24237	2.88571	3.71799
H	1.67465	2.22627	4.26307
H	1.76707	3.19765	2.76000
C	4.55866	2.06981	1.35559
H	4.93128	2.38107	2.34528
C	5.32325	2.35380	0.21698
H	6.28582	2.88165	0.30802
C	4.84340	1.96345	-1.03958
H	5.43932	2.19245	-1.93813
C	3.62080	1.27724	-1.19074
C	3.19294	0.87079	-2.60880
H	2.21975	0.34065	-2.52698
C	4.20298	-0.09670	-3.26839
H	5.18399	0.39646	-3.43710
H	3.82741	-0.43061	-4.25860
H	4.38887	-1.00144	-2.65534
C	2.97826	2.09336	-3.52660
H	2.16581	2.73999	-3.13807
H	2.68071	1.76196	-4.54424
H	3.90799	2.69295	-3.62877
C	-2.67556	1.45291	0.36875
C	-3.00940	1.44448	1.76570
C	-2.32365	0.51526	2.77550
H	-1.73996	-0.23660	2.20621
C	-3.32909	-0.25325	3.66000
H	-3.87805	0.42422	4.34789
H	-2.79996	-0.99853	4.29075
H	-4.08477	-0.79513	3.05527
C	-1.32400	1.28524	3.66325
H	-0.53334	1.76926	3.05721
H	-0.82329	0.60204	4.38194
H	-1.83378	2.07995	4.24877
C	-3.99128	2.33802	2.23864
H	-4.24305	2.33631	3.31168
C	-4.65519	3.22682	1.38247
H	-5.42011	3.91506	1.77550
C	-4.33498	3.22245	0.01922
H	-4.86576	3.90891	-0.66055
C	-3.35676	2.35699	-0.51367
C	-3.10634	2.39241	-2.02750
H	-2.25372	1.71311	-2.24484
C	-4.32665	1.87757	-2.82517
H	-4.61589	0.84796	-2.53242
H	-4.10372	1.86886	-3.91303
H	-5.21367	2.52841	-2.67020
C	-2.71312	3.79648	-2.52990
H	-3.52786	4.53416	-2.36757
H	-2.49569	3.76888	-3.61805
H	-1.79575	4.16381	-2.02865
C	-0.21461	3.96230	0.86138
H	-1.20429	4.12364	0.39857
H	-0.18100	3.28112	1.72774
C	0.87601	4.66603	0.47684
H	1.83298	4.48831	1.00649
H	0.20886	2.67526	-2.25115
C	0.91140	5.71642	-0.59919
H	0.86862	6.70844	-0.08815
H	-0.01010	5.64891	-1.21620
C	2.16642	5.66477	-1.48411
H	3.09249	5.76148	-0.87880
H	2.16058	6.48915	-2.22604
H	2.22239	4.70687	-2.03841

F^{Ad}

SCF (BP86) Energy = -2296.017323
Enthalpy 0K = -2294.913499
Enthalpy 298K = -2294.854571
Free Energy 298K = -2295.014950
Lowest Frequency = 4.9935 cm^{-1}
Second Frequency = 6.1814 cm^{-1}
SCF (BP86-D3^{BJ}) Energy = -2296.331155
SCF (C_6H_6) Energy = -2296.058847
SCF (BS2) Energy = -2298.321012

Mg	-0.76266	1.45609	-0.10501
N	1.23843	0.95477	-0.05686
N	-1.47965	-0.47789	-0.04522
C	1.66667	-0.32143	0.01268
C	3.17126	-0.77435	0.13206
C	3.35006	-1.55337	1.48035
H	3.12142	-0.86902	2.32725
H	2.61941	-2.38557	1.54588
C	4.23401	0.36156	0.12910
H	4.17433	0.94394	-0.81046
H	4.03393	1.07739	0.94996

C	3.53936	-1.73621	-1.04701
H	2.82179	-2.58049	-1.10278
H	3.44958	-1.18514	-2.00929
C	4.78148	-2.11871	1.62218
H	4.85030	-2.67644	2.58251
C	5.80040	-0.96115	1.61406
H	6.83469	-1.35274	1.73819
H	5.61081	-0.27742	2.47127
C	5.67133	-0.19531	0.28153
H	6.37771	0.66359	0.27269
C	5.98993	-1.14260	-0.89356
H	7.02630	-1.53798	-0.80380
H	5.93910	-0.59001	-1.85804
C	4.97056	-2.30002	-0.88928
H	5.17712	-2.98830	-1.73875
C	5.07517	-3.07536	0.44396
H	4.35636	-3.92520	0.45135
H	6.09127	-3.51555	0.55197
C	0.75245	-1.41643	0.01418
H	1.26165	-2.38292	0.05265
C	-0.66660	-1.55328	-0.02028
C	-1.14010	-3.05584	-0.05759
C	-0.60052	-3.82146	1.19659
H	0.49891	-3.70152	1.28528
H	-1.03968	-3.37290	2.11503
C	-0.54908	-3.73188	-1.34234
H	-0.95186	-3.21489	-2.24149
H	0.55221	-3.60277	-1.37588
C	-2.67734	-3.29162	-0.09747
H	-3.15912	-2.85208	0.79703
H	-3.12200	-2.77832	-0.97222
C	-0.94043	-5.32831	1.12660
H	-0.52299	-5.82929	2.02812
C	-0.31055	-5.94034	-0.14546
H	0.79642	-5.82699	-0.11832
H	-0.51891	-7.03241	-0.18835
C	-0.88748	-5.23883	-1.39620
H	-0.43062	-5.67368	-2.31283
C	-2.41799	-5.42592	-1.43396
H	-2.84110	-4.94215	-2.34236
H	-2.67623	-6.50666	-1.49569
C	-3.02626	-4.79920	-0.16285
H	-4.13373	-4.89714	-0.18950
C	-2.47095	-5.51616	1.08498
H	-2.72837	-6.59849	1.05782
H	-2.93405	-5.09876	2.00667
C	1.99796	2.16368	-0.18146
C	2.20175	2.99458	0.96302
C	1.73525	2.57978	2.36155
H	1.31690	1.55334	2.27630
C	2.89307	2.51823	3.38038
H	3.70073	1.83425	3.04705
H	2.52861	2.15861	4.36592
H	3.34843	3.51844	3.54160
C	0.60978	3.50818	2.87030
H	0.97945	4.54616	3.01080
H	0.21829	3.15692	3.84847
H	-0.23707	3.55999	2.15347
C	2.83459	4.24293	0.79091
H	3.00442	4.88430	1.67111
C	3.24679	4.68726	-0.47170
H	3.73984	5.66578	-0.58387
C	3.01072	3.88116	-1.59476
H	3.31770	4.24056	-2.58990
C	2.38542	2.62425	-1.48019
C	2.08485	1.80576	-2.73914
H	1.87683	0.76409	-2.41680
C	3.26156	1.75005	-3.73337
H	3.47976	2.74383	-4.17843
H	3.02598	1.06367	-4.57376
H	4.19229	1.38831	-3.24907
C	0.81035	2.33403	-3.43488
H	-0.06298	2.35593	-2.74759
H	0.53994	1.70438	-4.30904
H	0.95594	3.37522	-3.79290
C	-2.90846	-0.41616	0.04437
C	-3.53440	-0.38617	1.33120
C	-2.71731	-0.51521	2.62000
H	-1.72711	-0.93503	2.34544
C	-3.34808	-1.46893	3.65401
H	-4.30859	-1.07779	4.05115
H	-2.66895	-1.60295	4.52218
H	-3.54829	-2.47081	3.22068
C	-2.46807	0.87598	3.24419
H	-1.98236	1.57410	2.52860
H	-1.81802	0.80214	4.14181
H	-3.42392	1.35114	3.55099
C	-4.92675	-0.18466	1.40322
H	-5.41716	-0.16606	2.38968
C	-5.70150	-0.00272	0.24859
H	-6.78929	0.15105	0.32780
C	-5.07605	-0.00324	-1.00472
H	-5.68283	0.15554	-1.91115
C	-3.68567	-0.19815	-1.13487
C	-3.04950	-0.14723	-2.52706
H	-1.97405	-0.40025	-2.40480
C	-3.65541	-1.18229	-3.49832
H	-3.58156	-2.21523	-3.09972
H	-3.12824	-1.15682	-4.47547
H	-4.72875	-0.97743	-3.69746
C	-3.13501	1.27575	-3.12338
H	-4.19099	1.57147	-3.30099
H	-2.60273	1.33129	-4.09657
H	-2.69595	2.03504	-2.44152
C	-1.84561	7.35143	-0.26918
H	-2.19605	8.39935	-0.26625
H	-0.75600	7.18920	-0.30855
C	-2.70897	6.31902	-0.23176
H	-2.30873	5.28509	-0.23303
H	-1.56310	2.97732	-0.16659
C	-4.21088	6.43950	-0.17240
H	-4.64549	5.95797	-1.07978
H	-4.50533	7.51161	-0.21781
C	-4.82214	5.77286	1.07465
H	-4.55316	4.69673	1.12635
H	-5.93016	5.84381	1.06802
H	-4.45453	6.25365	2.00545

G^{Ad}

SCF (BP86) Energy = -2138.920336
Enthalpy 0K = -2137.921522
Enthalpy 298K = -2137.870582
Free Energy 298K = -2138.004385
Lowest Frequency = 11.0790 cm^{-1}
Second Frequency = 23.1507 cm^{-1}
SCF (BP86-D3^{BJ}) Energy = -2139.219586
SCF (C_6H_6) Energy = -2138.958522
SCF (BS2) Energy = -2141.048164

Mg	-0.00004	-2.27515	-0.00003
N	-1.53748	-0.89657	-0.01799
N	1.53744	-0.89655	0.01797
C	-1.31965	0.43359	0.00688
C	-2.43895	1.54098	0.07817
C	-2.24085	2.36244	1.39808
H	-2.36452	1.68235	2.26991
H	-1.20616	2.75838	1.45562
C	-3.90940	1.03340	0.08580
H	-4.12364	0.45578	-0.83398
H	-4.07144	0.33831	0.93246
C	-2.30780	2.51838	-1.13740
H	-1.27829	2.92676	-1.20242
H	-2.48053	1.95368	-2.08023
C	-3.24214	3.53605	1.49055
H	-3.04761	4.09674	2.43173
C	-4.68405	2.98877	1.49477
H	-5.41606	3.82229	1.58387
H	-4.84081	2.32803	2.37630
C	-4.92046	2.20237	0.18939
H	-5.94642	1.77318	0.19069
C	-4.75263	3.14424	-1.02068
H	-5.48440	3.98090	-0.96612
H	-4.96072	2.59629	-1.96652
C	-3.31024	3.69101	-1.02896
H	-3.16644	4.36393	-1.90330
C	-3.04779	4.47433	0.27754
H	-2.01504	4.88957	0.27529
H	-3.74072	5.34185	0.34933
C	-0.00003	0.97493	0.00000
H	-0.00003	2.06796	-0.00001
C	1.31961	0.43360	-0.00681
C	2.43890	1.54099	-0.07806
C	2.30756	2.51859	1.13733
H	1.27804	2.92696	1.20216
H	2.48019	1.95404	2.08027
C	2.24092	2.36224	-1.39813
H	2.36468	1.68200	-2.26984
H	1.20622	2.75814	-1.45584
C	3.90937	1.03346	-0.08541
H	4.12349	0.45600	0.83449
H	4.07154	0.33824	-0.93193
C	3.30998	3.69123	1.02882
H	3.16605	4.36430	1.90303
C	3.04766	4.47433	-0.27784
H	2.01490	4.88954	-0.27578
H	3.74058	5.34186	-0.34968
C	3.24218	3.53585	-1.49067
H	3.04776	4.09638	-2.43197
C	4.68411	2.98861	-1.49463
H	4.84100	2.32773	-2.37602
H	5.41612	3.82214	-1.58378
C	4.92040	2.20244	-0.18908
H	5.94637	1.77329	-0.19018
C	4.75239	3.14451	1.02081
H	5.48414	3.98120	0.96618
H	4.96039	2.59674	1.96677
C	-2.77359	-1.61221	-0.13456
C	-3.36328	-2.20530	1.02408
C	-2.77741	-2.00516	2.42476
H	-1.94062	-1.27982	2.32897
C	-3.79429	-1.40153	3.41640
H	-4.20609	-0.43831	3.05029
H	-3.31619	-1.21414	4.40115
H	-4.65150	-2.08591	3.59114
C	-2.19603	-3.32681	2.97533
H	-2.99570	-4.08344	3.12415
H	-1.70065	-3.16444	3.95606
H	-1.45410	-3.77174	2.27829
C	-4.50552	-3.01637	0.86455
H	-4.97095	-3.46888	1.75541
C	-5.05804	-3.26313	-0.39860
H	-5.95119	-3.89954	-0.50089
C	-4.45289	-2.70529	-1.53394
H	-4.87682	-2.91571	-2.52888
C	-3.31168	-1.88583	-1.43223
C	-2.64299	-1.35318	-2.70280
H	-1.96137	-0.53085	-2.40046
C	-3.63974	-0.76882	-3.72339
H	-4.30836	-1.54781	-4.14706
H	-3.09652	-0.30944	-4.57593
H	-4.28291	0.01301	-3.26870
C	-1.77562	-2.45178	-3.35719
H	-1.02744	-2.87014	-2.64970
H	-1.22795	-2.05806	-4.23972
H	-2.40274	-3.30422	-3.69443
C	2.77355	-1.61221	0.13456
C	3.31162	-1.88588	1.43221
C	2.64300	-1.35326	2.70284
H	1.96093	-0.53131	2.40050
C	3.63978	-0.76820	3.72300
H	4.30895	-1.54678	4.14656
H	3.09663	-0.30892	4.57563
H	4.28239	0.01387	3.26792
C	1.77626	-2.45211	3.35768
H	1.02807	-2.87083	2.65044
H	1.22869	-2.05842	4.24029
H	2.40381	-3.30421	3.69496
C	4.45279	-2.70542	1.53390
H	4.87667	-2.91590	2.52885
C	5.05792	-3.26324	0.39855
H	5.95103	-3.89972	0.50084
C	4.50543	-3.01640	-0.86460
H	4.97083	-3.46891	-1.75547
C	3.36322	-2.20526	-1.02411
C	2.77744	-2.00504	-2.42481
H	1.94047	-1.27991	-2.32899
C	3.79432	-1.40097	-3.41621
H	4.20586	-0.43772	-3.04987
H	3.31631	-1.21351	-4.40099
H	4.65171	-2.08513	-3.59093
C	2.19644	-3.32668	-2.97576
H	2.99629	-4.08307	-3.12479
H	1.70107	-3.16415	-3.95648
H	1.45457	-3.77197	-2.27889
H	0.00039	-3.99260	-0.00011

C_4H_8

SCF (BP86) Energy = -157.096141
Enthalpy 0K = -156.991357
Enthalpy 298K = -156.985030
Free Energy 298K = -157.018956
Lowest Frequency = 104.3452 cm^{-1}
Second Frequency = 230.4770 cm^{-1}
SCF (BP86-D3^{BJ}) Energy = -157.107237
SCF (C_6H_6) Energy = -157.100190
SCF (BS2) Energy = -157.272616

C	0.72394	0.30738	0.32781
C	-0.54299	-0.50956	0.31805
H	-0.36628	-1.46469	-0.22358
H	-0.79856	-0.79004	1.36686
H	0.67100	1.26801	0.87761
C	-1.74197	0.23529	-0.29864
H	-1.94916	1.18497	0.23939
H	-1.55131	0.49077	-1.36186
H	-2.66400	-0.38097	-0.25520
C	1.87917	-0.02685	-0.27626
H	1.98291	-0.97089	-0.83975
H	2.76651	0.62525	-0.22921

$2G^{Ad}$

SCF (BP86) Energy = -4277.841760
Enthalpy 0K = -4275.843996
Enthalpy 298K = -4275.740293
Free Energy 298K = -4275.998647
Lowest Frequency = 1.4305 cm^{-1}
Second Frequency = 3.3811 cm^{-1}
SCF (BP86-D3^{BJ}) Energy = -4278.448881
SCF (C_6H_6) Energy = -4277.916331
SCF (BS2) Energy = -4282.096435

Mg	4.72217	0.29824	-1.56732
N	6.70753	0.82937	-1.34206
N	4.58217	-0.79556	0.17706
C	7.44792	0.42851	-0.28763
C	8.98366	0.72398	-0.08685
C	9.21858	1.46132	1.27333
H	8.70564	2.44818	1.24749
H	8.76177	0.89364	2.10986
C	9.66672	1.58639	-1.18683
H	9.54332	1.11049	-2.17908
H	9.18083	2.57850	-1.25741
C	9.75149	-0.64151	-0.04739
H	9.31565	-1.30873	0.72440
H	9.61737	-1.16122	-1.02197
C	10.72645	1.65350	1.55836
H	10.84057	2.16537	2.53977
C	11.36259	2.51019	0.44443
H	12.44369	2.67489	0.65138
H	10.88487	3.51467	0.41301
C	11.17652	1.78848	-0.90601
H	11.60062	2.40985	-1.72504
C	11.89064	0.42214	-0.86593
H	12.97930	0.55766	-0.67804
H	11.79545	-0.09110	-1.84869
C	11.25525	-0.43725	0.24605
H	11.74927	-1.43376	0.28188
C	11.42127	0.27354	1.60844
H	10.98262	-0.34762	2.42123
H	12.50102	0.39648	1.84732
C	6.89124	-0.35846	0.76190
H	7.61979	-0.58031	1.54615
C	5.61101	-0.92780	1.03558
C	5.55207	-1.67180	2.42383
C	5.88861	-0.64173	3.55630
H	6.85882	-0.14417	3.35150
H	5.11865	0.16121	3.55663
C	6.61849	-2.81636	2.46606
H	6.37438	-3.57284	1.68762
H	7.62517	-2.42306	2.21468
C	4.18757	-2.31659	2.79969
H	3.38717	-1.55142	2.79619
H	3.89561	-3.07191	2.04508
C	5.94893	-1.32334	4.94228
H	6.20895	-0.55598	5.70505
C	7.03104	-2.42697	4.92648
H	8.02831	-1.98381	4.70703
H	7.10686	-2.90344	5.92913
C	6.67255	-3.48651	3.85936
H	7.45361	-4.27890	3.84227
C	5.30093	-4.10897	4.19204
H	5.04130	-4.88830	3.44133
H	5.33447	-4.61481	5.18285
C	4.23571	-2.99342	4.19180
H	3.23435	-3.43113	4.39790
C	4.57858	-1.94871	5.27339
H	4.60102	-2.42378	6.27972
H	3.79499	-1.15944	5.30774
C	7.05749	1.71537	-2.41156
C	6.89812	3.12720	-2.24151
C	6.44506	3.72390	-0.90591
H	6.59890	2.94858	-0.12648
C	7.25420	4.96560	-0.48184
H	8.34466	4.75957	-0.46445
H	6.95357	5.29502	0.53504
H	7.08672	5.82594	-1.16392
C	4.93452	4.04611	-0.94102
H	4.71302	4.82865	-1.69753
H	4.57951	4.41188	0.04579
H	4.32276	3.15964	-1.21517
C	7.11967	3.97298	-3.34633
H	7.00441	5.06162	-3.22180
C	7.47917	3.46034	-4.60080
H	7.65078	4.13990	-5.45056
C	7.60252	2.07465	-4.76637
H	7.86863	1.67121	-5.75709
C	7.38966	1.18250	-3.69590
C	7.49414	-0.32493	-3.94435
H	7.37046	-0.82724	-2.96065
C	8.87010	-0.74411	-4.50331
H	9.05502	-0.30586	-5.50707
H	8.92929	-1.84805	-4.60996
H	9.70033	-0.42224	-3.84099
C	6.35626	-0.81238	-4.86920
H	5.35755	-0.52654	-4.47553
H	6.38384	-1.91686	-4.98400
H	6.44688	-0.36663	-5.88269
C	3.27622	-1.38811	0.22220
C	2.15681	-0.61086	0.65277
C	2.32021	0.81567	1.18707
H	3.41255	1.01312	1.24881
C	1.73390	0.98970	2.60344
H	0.63517	0.83001	2.60930
H	1.92230	2.01711	2.98125
H	2.18092	0.27524	3.32553
C	1.70181	1.84215	0.21177
H	2.11639	1.73649	-0.81383
H	1.88680	2.88104	0.55915
H	0.60268	1.70277	0.13570
C	0.86514	-1.16963	0.56050
H	-0.01163	-0.58703	0.89026
C	0.66316	-2.45711	0.04559
H	-0.35496	-2.87282	-0.01550
C	1.76386	-3.20031	-0.40531

H	1.60028	-4.20502	-0.82756
C	3.07502	-2.68984	-0.33634
C	4.24062	-3.50596	-0.90335
H	5.18011	-3.05869	-0.51637
C	4.22483	-4.98424	-0.46638
H	4.18868	-5.08550	0.63825
H	5.13677	-5.50568	-0.82670
H	3.35222	-5.53225	-0.88090
C	4.28000	-3.39451	-2.44382
H	3.36694	-3.83644	-2.89652
H	5.16213	-3.92364	-2.86347
H	4.32140	-2.33722	-2.78399
H	-2.37462	-0.03288	0.92138
Mg	-4.03343	0.05491	0.47157
N	-5.42285	1.57018	0.65854
N	-5.31952	-1.33039	-0.35421
C	-6.71373	1.41809	0.29954
C	-7.84491	2.50142	0.47729
C	-8.49504	2.83013	-0.90838
H	-7.72524	3.27727	-1.57568
H	-8.84052	1.90229	-1.40895
C	-7.40503	3.85932	1.09587
H	-6.94211	3.69776	2.08856
H	-6.62949	4.33700	0.46670
C	-8.95403	1.91759	1.41847
H	-9.31726	0.94387	1.03037
H	-8.50989	1.71024	2.41729
C	-9.69197	3.79711	-0.75545
H	-10.12842	3.98440	-1.76159
C	-9.21251	5.12617	-0.13677
H	-10.06158	5.83941	-0.04319
H	-8.45945	5.60811	-0.79922
C	-8.59742	4.83601	1.24749
H	-8.21589	5.78052	1.69368
C	-9.66726	4.21946	2.17170
H	-10.52223	4.92053	2.29802
H	-9.24451	4.04241	3.18569
C	-10.14803	2.88928	1.55652
H	-10.91275	2.42237	2.21629
C	-10.75844	3.15552	0.16157
H	-11.12581	2.20420	-0.28449
H	-11.64041	3.82774	0.25152
C	-7.19124	0.20410	-0.27656
H	-8.25442	0.25362	-0.52576
C	-6.61526	-1.05789	-0.60855
C	-7.63635	-2.03782	-1.30171
C	-8.15782	-1.36772	-2.61871
H	-8.57024	-0.36047	-2.40458
H	-7.30055	-1.21337	-3.31099
C	-8.85982	-2.28985	-0.35733
H	-8.50758	-2.79701	0.56818
H	-9.30885	-1.32791	-0.03491
C	-7.08608	-3.43706	-1.70209
H	-6.21788	-3.32873	-2.38086
H	-6.71602	-3.97733	-0.80970
C	-9.24619	-2.22801	-3.30047
H	-9.59506	-1.70013	-4.21569
C	-10.43253	-2.42124	-2.32839
H	-10.87556	-1.43524	-2.06300
H	-11.23771	-3.01191	-2.81891
C	-9.94263	-3.14590	-1.05381
H	-10.79454	-3.28263	-0.35123
C	-9.35472	-4.51966	-1.43663
H	-9.01855	-5.06090	-0.52428
H	-10.13227	-5.15395	-1.91786
C	-8.16706	-4.30260	-2.39646
H	-7.71061	-5.28310	-2.65541
C	-8.65903	-3.60264	-3.67957
H	-9.42613	-4.22531	-4.19182
H	-7.81746	-3.47585	-4.39640
C	-4.74663	2.73623	1.14515
C	-4.18171	3.66331	0.21267
C	-4.36678	3.48830	-1.29738
H	-5.21427	2.78752	-1.44881
C	-4.72014	4.79822	-2.02952
H	-5.61022	5.29016	-1.58515
H	-4.94073	4.59618	-3.09876
H	-3.88464	5.52921	-2.00375
C	-3.11403	2.83645	-1.92363
H	-2.22583	3.49377	-1.81379
H	-3.26258	2.63877	-3.00638
H	-2.85501	1.87212	-1.43514
C	-3.40249	4.72914	0.70396
H	-2.96939	5.45087	-0.00687
C	-3.16194	4.88863	2.07615
H	-2.55093	5.73015	2.43917
C	-3.69371	3.95912	2.97909
H	-3.49120	4.07604	4.05636
C	-4.48001	2.87333	2.54241
C	-5.00195	1.86534	3.57039
H	-5.65850	1.15257	3.02625
C	-5.85651	2.52514	4.67305
H	-5.25829	3.22985	5.28895
H	-6.26926	1.75633	5.35999
H	-6.70934	3.09452	4.24913
C	-3.84058	1.05678	4.19118
H	-3.21879	0.56602	3.41220
H	-4.22445	0.27207	4.87700
H	-3.16535	1.71540	4.77803
C	-4.59938	-2.55758	-0.52765
C	-3.74866	-2.72717	-1.66309
C	-3.66266	-1.67995	-2.77709
H	-4.42197	-0.89997	-2.55207
C	-4.00017	-2.25861	-4.16760
H	-3.26295	-3.02846	-4.47962
H	-3.98578	-1.45822	-4.93721
H	-5.00434	-2.73046	-4.18674
C	-2.27540	-1.00043	-2.79355
H	-2.01322	-0.56724	-1.80495
H	-2.24069	-0.18716	-3.54885
H	-1.47393	-1.72609	-3.04623
C	-2.94895	-3.88621	-1.74346
H	-2.29874	-4.02894	-2.62185
C	-2.96053	-4.85586	-0.73221
H	-2.33061	-5.75549	-0.81647
C	-3.77175	-4.66356	0.39570
H	-3.76514	-5.41741	1.19917
C	-4.59234	-3.52608	0.52616
C	-5.41505	-3.32583	1.80213
H	-6.16938	-2.53926	1.59053
C	-6.18114	-4.58935	2.24169
H	-6.82292	-4.98531	1.42748
H	-6.83434	-4.36392	3.11087
H	-5.49547	-5.40521	2.55416
C	-4.51796	-2.80654	2.94804
H	-3.74347	-3.55585	3.21707
H	-5.11650	-2.59034	3.85846
H	-3.97687	-1.87785	2.66554

H	3.59554	0.62602	-2.82408

TS(2G^{Ad}–[2^{Mg,Ad}]$_2$)

SCF (BP86) Energy = -4277.818705
Enthalpy 0K = -4275.817943
Enthalpy 298K = -4275.717413
Free Energy 298K = -4275.952020
Lowest Frequency = -77.6996 cm^{-1}
Second Frequency = 7.6649 cm^{-1}
SCF (BP86-D3^{BJ}) Energy = -4278.484979
SCF (C_6H_6) Energy = -4277.884059
SCF (BS2) Energy = -4282.068909

Mg	1.73774	-0.16407	-0.20982
N	3.06741	1.37386	-0.80454
N	3.28043	-1.49571	0.37347
C	4.37878	1.34607	-0.48842
C	5.49299	2.36636	-0.97851
C	6.28759	2.96536	0.23166
H	5.60317	3.58777	0.84904
H	6.66648	2.16419	0.89867
C	5.03349	3.57835	-1.84226
H	4.46977	3.22765	-2.72731
H	4.34259	4.22543	-1.26993
C	6.49669	1.54722	-1.86671
H	6.87090	0.66681	-1.30648
H	5.95094	1.14634	-2.74952
C	7.48715	3.81957	-0.24283
H	8.02323	4.20267	0.65372
C	6.98359	5.00109	-1.09633
H	7.83764	5.63579	-1.42233
H	6.31059	5.65095	-0.49405
C	6.22970	4.44010	-2.31852
H	5.82538	5.27872	-2.92691
C	7.18917	3.59107	-3.17588
H	8.04373	4.20908	-3.53152
H	6.66540	3.21489	-4.08276
C	7.69442	2.40903	-2.32519
H	8.37840	1.77196	-2.92892
C	8.44414	2.94571	-1.08495
H	8.82965	2.09919	-0.47362
H	9.32936	3.54175	-1.39960
C	4.93285	0.28929	0.29054
H	5.96411	0.49314	0.58560
C	4.51878	-1.01351	0.66323
C	5.64211	-1.77774	1.49482
C	5.85860	-0.95476	2.81676
H	6.10100	0.10028	2.57323
H	4.90964	-0.92922	3.39444
C	7.01086	-1.81665	0.73466
H	6.90363	-2.41946	-0.19180
H	7.31544	-0.79958	0.41280
C	5.33748	-3.24564	1.91827
H	4.37780	-3.29920	2.46215
H	5.22549	-3.88827	1.02471
C	6.98868	-1.54984	3.68648
H	7.10151	-0.92060	4.59728
C	8.30946	-1.54705	2.88533
H	8.58872	-0.50541	2.60943
H	9.13932	-1.94585	3.51016
C	8.13800	-2.40876	1.61467
H	9.08331	-2.40123	1.02767
C	7.78519	-3.85440	2.01958
H	7.68544	-4.49420	1.11470
H	8.60100	-4.29357	2.63628
C	6.45983	-3.83675	2.80749
H	6.17083	-4.87607	3.07800
C	6.62788	-2.99434	4.08786
H	7.42258	-3.42705	4.73589
H	5.68724	-3.00373	4.68162
C	2.40465	2.50568	-1.37903
C	2.10615	3.62655	-0.53751
C	2.49634	3.61332	0.94510
H	3.50670	3.15551	1.01148
C	2.60079	5.01219	1.57818
H	3.27064	5.68166	0.99914
H	3.00742	4.93329	2.60776
H	1.60919	5.50443	1.65954
C	1.54912	2.72755	1.77983
H	0.50470	3.09105	1.73162
H	1.85960	2.70717	2.84493
H	1.54064	1.67041	1.43803
C	1.48483	4.75052	-1.11535
H	1.27325	5.63095	-0.49127
C	1.14747	4.78052	-2.47823
H	0.68513	5.68011	-2.91441
C	1.38300	3.65261	-3.27304
H	1.09178	3.66898	-4.33593
C	1.99270	2.49554	-2.74410
C	2.22316	1.28965	-3.65753
H	2.72257	0.50828	-3.04477
C	3.16255	1.60890	-4.84031
H	2.72507	2.37922	-5.51068
H	3.34248	0.69998	-5.45245
H	4.14779	1.98576	-4.49714
C	0.88651	0.71013	-4.16752
H	0.22005	0.43756	-3.32394
H	1.06233	-0.19787	-4.78287
H	0.34585	1.44183	-4.80497
C	3.00092	-2.89543	0.25001
C	2.04628	-3.51307	1.11977
C	1.32225	-2.71943	2.21147
H	0.96616	-1.76171	1.75425
C	2.20078	-2.31630	3.41309
H	2.58084	-3.21033	3.95301
H	1.60013	-1.72017	4.13161
H	3.06593	-1.69807	3.11028
C	0.04931	-3.40291	2.72843
H	-0.63223	-3.70267	1.90800
H	-0.50180	-2.69836	3.38204
H	0.27969	-4.30598	3.33446
C	1.76570	-4.88383	0.94123
H	1.04479	-5.37577	1.60893
C	2.37581	-5.63513	-0.07168
H	2.14174	-6.70560	-0.18555
C	3.27150	-5.01021	-0.94854
H	3.73155	-5.59913	-1.75710
C	3.60095	-3.64626	-0.81557
C	4.55897	-3.00916	-1.83087
H	5.03898	-2.13581	-1.34528
C	5.69141	-3.95116	-2.28629
H	6.22187	-4.41025	-1.42650
H	6.43739	-3.39106	-2.88827
H	5.31697	-4.77724	-2.92734
C	3.79831	-2.46457	-3.05810
H	3.26997	-3.28001	-3.59574
H	4.49663	-1.97781	-3.77176
H	3.03948	-1.71197	-2.76406

H	-0.48326	-0.35654	1.73102
Mg	-1.71043	-0.16921	0.46336
N	-3.17888	1.36480	0.85531
N	-3.17562	-1.53058	-0.30954
C	-4.46073	1.26901	0.45609
C	-5.65245	2.24574	0.85401
C	-6.44472	2.73039	-0.40809
H	-5.78155	3.36090	-1.04077
H	-6.75551	1.87515	-1.04160
C	-5.29542	3.52983	1.66105
H	-4.73662	3.26696	2.57867
H	-4.62989	4.18810	1.07123
C	-6.63389	1.41278	1.75316
H	-6.93439	0.48464	1.22619
H	-6.09518	1.08902	2.67079
C	-7.70893	3.53143	-0.01642
H	-8.23836	3.83216	-0.94782
C	-7.30640	4.78564	0.78484
H	-8.20755	5.38156	1.05248
H	-6.65647	5.44401	0.16653
C	-6.55702	4.33847	2.05597
H	-6.22518	5.23173	2.62959
C	-7.48879	3.47594	2.93065
H	-8.39035	4.05693	3.22834
H	-6.97149	3.18135	3.87063
C	-7.89500	2.22143	2.13156
H	-8.55773	1.57619	2.75027
C	-8.63738	2.64507	0.84426
H	-8.95110	1.74529	0.26900
H	-9.56664	3.20063	1.10125
C	-4.92061	0.16442	-0.31933
H	-5.94630	0.30598	-0.66358
C	-4.42467	-1.12347	-0.64487
C	-5.48117	-1.96503	-1.48751
C	-5.65886	-1.21400	-2.85450
H	-5.93328	-0.15419	-2.67362
H	-4.68229	-1.19418	-3.38683
C	-6.87767	-2.02253	-0.78048
H	-6.78278	-2.58404	0.17410
H	-7.23173	-1.00729	-0.51013
C	-5.11692	-3.44060	-1.82724
H	-4.13409	-3.49230	-2.32746
H	-5.02228	-4.03699	-0.89986
C	-6.73578	-1.88029	-3.74011
H	-6.83111	-1.29731	-4.68326
C	-8.08808	-1.88283	-2.99192
H	-8.40603	-0.83903	-2.77170
H	-8.88040	-2.33006	-3.63261
C	-7.94821	-2.68623	-1.67921
H	-8.91851	-2.68214	-1.13439
C	-7.53762	-4.13608	-2.00542
H	-7.45915	-4.73323	-1.06984
H	-8.31189	-4.62641	-2.63717
C	-6.17995	-4.10891	-2.73511
H	-5.84611	-5.14883	-2.94545
C	-6.31901	-3.33033	-4.05902
H	-7.07366	-3.81620	-4.71740
H	-5.35527	-3.33891	-4.61492
C	-2.59347	2.52712	1.44446
C	-2.30204	3.65447	0.60530
C	-2.69030	3.64648	-0.87975
H	-3.72360	3.24065	-0.93680
C	-2.73664	5.04538	-1.51939
H	-3.38135	5.74499	-0.94735
H	-3.14339	4.97677	-2.54977
H	-1.72620	5.49797	-1.59930
C	-1.80682	2.70294	-1.72090
H	-0.73388	2.95832	-1.62776
H	-2.08295	2.76300	-2.79436
H	-1.93531	1.64384	-1.41966
C	-1.71354	4.79237	1.19086
H	-1.50591	5.67447	0.56784
C	-1.40560	4.83711	2.56033
H	-0.96492	5.74627	2.99966
C	-1.65497	3.71530	3.35942
H	-1.40234	3.74831	4.43169
C	-2.23216	2.54273	2.82698
C	-2.51450	1.36540	3.76510
H	-2.86046	0.52078	3.13206
C	-3.64243	1.68431	4.77177
H	-3.35974	2.52424	5.44224
H	-3.85415	0.80232	5.41290
H	-4.58755	1.96670	4.26513
C	-1.24894	0.89879	4.51195
H	-0.45804	0.60684	3.79244
H	-1.47922	0.01534	5.14494
H	-0.85150	1.68959	5.18386
C	-2.80230	-2.90622	-0.19777
C	-1.84611	-3.47285	-1.09661
C	-1.23046	-2.66594	-2.24379
H	-1.41864	-1.59176	-2.02776
C	-1.86163	-2.97062	-3.62123
H	-1.78948	-4.05246	-3.86450
H	-1.33210	-2.40924	-4.42006
H	-2.92990	-2.68338	-3.66920
C	0.29155	-2.86144	-2.33054
H	0.78129	-2.74445	-1.34243
H	0.73116	-2.12638	-3.03438
H	0.56291	-3.87239	-2.70048
C	-1.48561	-4.82770	-0.94096
H	-0.76227	-5.27470	-1.64075
C	-2.02784	-5.61789	0.08009
H	-1.73790	-6.67608	0.17850
C	-2.93219	-5.04438	0.98594
H	-3.34189	-5.66265	1.79992
C	-3.33195	-3.69798	0.87691
C	-4.29997	-3.11178	1.91212
H	-4.80976	-2.24534	1.44377
C	-5.39882	-4.09691	2.35753
H	-5.93417	-4.54178	1.49302
H	-6.14750	-3.57579	2.99049
H	-4.99135	-4.93285	2.96507
C	-3.53963	-2.56813	3.14112
H	-3.02527	-3.38944	3.68382
H	-4.23827	-2.07474	3.84996
H	-2.76653	-1.82713	2.85056
H	0.05031	-0.07470	-0.85519

[$2^{Mg,Ad}$]$_2$

SCF (BP86) Energy = -4277.828574
Enthalpy 0K = -4275.826925
Enthalpy 298K = -4275.725985
Free Energy 298K = -4275.962474
Lowest Frequency = 10.0164 cm^{-1}
Second Frequency = 10.8542 cm^{-1}
SCF (BP86-D3^{BJ}) Energy = -4278.500889
SCF (C_6H_6) Energy = -4277.891731
SCF (BS2) Energy = -4282.078982

Mg	-1.47536	-0.08952	0.13776
N	-2.85978	1.32219	0.98508
N	-3.02117	-1.44495	-0.52326
C	-4.15711	1.29025	0.62459
C	-5.35025	2.09887	1.29874
C	-6.23881	2.84502	0.24632
H	-5.64278	3.64664	-0.24190
H	-6.56606	2.16149	-0.56347
C	-4.98446	3.15239	2.38602
H	-4.35543	2.69283	3.17160
H	-4.38279	3.97059	1.94765
C	-6.24716	1.01859	2.00537
H	-6.55056	0.24578	1.27045
H	-5.63971	0.49559	2.77666
C	-7.49997	3.46014	0.89963
H	-8.09734	3.96149	0.10600
C	-7.08707	4.48855	1.97169
H	-7.98829	4.95342	2.43032
H	-6.49988	5.31226	1.50825
C	-6.24344	3.77072	3.04413
H	-5.90445	4.50466	3.80791
C	-7.08776	2.67097	3.71869
H	-7.98581	3.11364	4.20488
H	-6.50051	2.17227	4.52146
C	-7.50531	1.64316	2.64833
H	-8.10446	0.83037	3.11595
C	-8.34303	2.34304	1.55502
H	-8.66567	1.60429	0.78758
H	-9.27008	2.77219	1.99601
C	-4.62754	0.38357	-0.37323
H	-5.62530	0.64957	-0.72871
C	-4.20413	-0.87138	-0.87230
C	-5.22275	-1.46718	-1.93209
C	-5.14051	-0.51811	-3.18002
H	-5.35503	0.52711	-2.87345
H	-4.09856	-0.52377	-3.56971
C	-6.70304	-1.47555	-1.42691
H	-6.79313	-2.16398	-0.55914
H	-7.01081	-0.47334	-1.06517
C	-4.92731	-2.90509	-2.43832
H	-3.89125	-2.96155	-2.81512
H	-4.99665	-3.63182	-1.60552
C	-6.12841	-0.94724	-4.28823
H	-6.04102	-0.23456	-5.13841
C	-7.56991	-0.91932	-3.73220
H	-7.83404	0.11077	-3.40320
H	-8.29459	-1.19589	-4.53003
C	-7.68301	-1.90351	-2.54581
H	-8.71806	-1.87868	-2.13806
C	-7.34473	-3.32943	-3.02579
H	-7.44671	-4.05223	-2.18599
H	-8.05802	-3.65195	-3.81699
C	-5.89968	-3.33793	-3.56312
H	-5.62355	-4.36716	-3.88198
C	-5.78588	-2.37428	-4.76234
H	-6.47382	-2.68835	-5.57897
H	-4.75583	-2.40105	-5.18235
C	-2.27234	2.39810	1.72331
C	-2.15352	3.68294	1.09279
C	-2.64275	3.90313	-0.34374
H	-3.62213	3.38862	-0.43891
C	-2.88720	5.37848	-0.70648
H	-3.56483	5.88088	0.01526

H	-3.35114	5.44900	-1.71223
H	-1.94138	5.95934	-0.74690
C	-1.70345	3.25269	-1.37744
H	-0.69363	3.70541	-1.34583
H	-2.10203	3.37721	-2.40565
H	-1.58071	2.16333	-1.20805
C	-1.60217	4.74594	1.83424
H	-1.52939	5.74349	1.37651
C	-1.15148	4.56616	3.15170
H	-0.73724	5.41615	3.71678
C	-1.20963	3.29345	3.72980
H	-0.83603	3.14926	4.75628
C	-1.75052	2.19044	3.03481
C	-1.82299	0.83391	3.74022
H	-2.09100	0.08086	2.96670
C	-2.92944	0.79567	4.81740
H	-2.73879	1.54796	5.61260
H	-2.97186	-0.20309	5.30124
H	-3.93135	1.00514	4.39159
C	-0.46896	0.41940	4.34780
H	0.32441	0.40835	3.57406
H	-0.53810	-0.59737	4.78917
H	-0.15504	1.10462	5.16356
C	-2.84336	-2.86612	-0.44473
C	-1.89268	-3.54191	-1.27499
C	-1.12629	-2.83742	-2.39746
H	-1.43018	-1.76900	-2.38954
C	-1.43574	-3.41445	-3.79824
H	-1.13668	-4.48172	-3.87039
H	-0.87045	-2.85887	-4.57606
H	-2.51085	-3.35254	-4.05924
C	0.39213	-2.89940	-2.16206
H	0.66801	-2.56048	-1.14386
H	0.92776	-2.26543	-2.89663
H	0.78228	-3.93400	-2.25889
C	-1.66133	-4.91850	-1.07655
H	-0.92744	-5.43410	-1.71661
C	-2.34436	-5.64613	-0.09521
H	-2.14869	-6.72145	0.04229
C	-3.28873	-4.98729	0.70356
H	-3.83489	-5.55905	1.47062
C	-3.56196	-3.61278	0.55180
C	-4.60656	-2.97425	1.47722
H	-4.92908	-2.01783	1.01983
C	-5.87122	-3.84239	1.64696
H	-6.29000	-4.16040	0.66978
H	-6.65777	-3.27509	2.18781
H	-5.67302	-4.76148	2.23836
C	-4.01154	-2.62639	2.85782
H	-3.62728	-3.53441	3.36996
H	-4.78044	-2.16506	3.51364
H	-3.17154	-1.90960	2.76757
H	-0.11127	-0.04205	-1.22788
Mg	1.48133	-0.08391	-0.12903
N	2.86936	1.32222	-0.98156
N	3.01655	-1.44788	0.52330
C	4.16878	1.27744	-0.62983
C	5.36544	2.07254	-1.31300
C	6.26596	2.81554	-0.26854
H	5.67958	3.62401	0.21989
H	6.59213	2.13216	0.54187
C	5.00227	3.12397	-2.40303
H	4.36501	2.66576	-3.18277
H	4.40986	3.94912	-1.96514

C	6.24964	0.98158	−2.01898
H	6.55084	0.21014	−1.28162
H	5.63378	0.45950	−2.78417
C	7.52832	3.41768	−0.93152
H	8.13396	3.91786	−0.14344
C	7.11780	4.44451	−2.00605
H	8.02025	4.90005	−2.47156
H	6.53974	5.27500	−1.54324
C	6.26266	3.72874	−3.07082
H	5.92575	4.46195	−3.83621
C	7.09430	2.61892	−3.74461
H	7.99326	3.05190	−4.23778
H	6.49862	2.12125	−4.54178
C	7.50937	1.59276	−2.67167
H	8.09925	0.77290	−3.13870
C	8.35882	2.29097	−1.58629
H	8.67990	1.55322	−0.81722
H	9.28675	2.71063	−2.03449
C	4.63542	0.36952	0.36899
H	5.63668	0.62786	0.72167
C	4.20039	−0.87777	0.87624
C	5.20776	−1.47292	1.94596
C	5.15818	−0.49229	3.17133
H	5.39905	0.53937	2.84011
H	4.11846	−0.46062	3.56584
C	6.68361	−1.53407	1.43321
H	6.75030	−2.24752	0.58378
H	7.01369	−0.54954	1.04272
C	4.87275	−2.88788	2.49031
H	3.83730	−2.90127	2.87464
H	4.91598	−3.63794	1.67686
C	6.14010	−0.92090	4.28486
H	6.07708	−0.18515	5.11735
C	7.57845	−0.94602	3.72064
H	7.86875	0.06822	3.36548
H	8.30016	−1.22327	4.52091
C	7.65729	−1.96107	2.55788
H	8.69052	−1.97592	2.14506
C	7.28098	−3.36508	3.07387
H	7.35711	−4.11018	2.25101
H	7.98985	−3.68891	3.86848
C	5.83958	−3.32039	3.62002
H	5.53753	−4.33377	3.96514
C	5.76085	−2.32560	4.79609
H	6.44508	−2.63907	5.61608
H	4.73310	−2.31304	5.22239
C	2.28679	2.40555	−1.71288
C	2.18912	3.69140	−1.08004
C	2.69530	3.90578	0.35148
H	3.66445	3.37141	0.43964
C	2.97385	5.37690	0.70697
H	3.65395	5.86329	−0.02338
H	3.44955	5.44075	1.70768
H	2.04076	5.97743	0.75560
C	1.75230	3.27774	1.39561
H	0.75414	3.75651	1.37747
H	2.16689	3.39043	2.41897
H	1.59735	2.19261	1.22579
C	1.64199	4.76132	−1.81469
H	1.58485	5.75894	−1.35484
C	1.17658	4.58848	−3.12791
H	0.76660	5.44382	−3.68799
C	1.21485	3.31591	−3.70795
H	0.82972	3.17722	−4.73089
C	1.74925	2.20558	−3.01948
C	1.79757	0.84921	−3.72752
H	2.05799	0.09046	−2.95721
C	2.89712	0.79608	−4.81112
H	2.71417	1.55347	−5.60331
H	2.92052	−0.20199	−5.29763
H	3.90476	0.98794	−4.39058
C	0.43428	0.45542	−4.32800
H	−0.35532	0.45513	−3.55042
H	0.48586	−0.56247	−4.76905
H	0.12618	1.14506	−5.14224
C	2.83845	−2.86862	0.44329
C	1.88230	−3.54310	1.26709
C	1.10576	−2.83154	2.37677
H	1.44252	−1.77290	2.39307
C	1.35402	−3.43609	3.77760
H	1.00685	−4.48966	3.83155
H	0.79491	−2.86415	4.54791
H	2.42475	−3.42446	4.06336
C	−0.40434	−2.84362	2.08888
H	−0.62895	−2.48245	1.06529
H	−0.94776	−2.20442	2.81303
H	−0.82844	−3.86736	2.15070
C	1.65241	−4.92016	1.07099
H	0.91383	−5.43445	1.70670
C	2.34462	−5.64956	0.09765
H	2.15163	−6.72554	−0.03842
C	3.29497	−4.99202	−0.69544
H	3.84740	−5.56586	−1.45611
C	3.56540	−3.61679	−0.54618
C	4.60993	−2.97397	−1.46820
H	4.96277	−2.04115	−0.98507
C	5.85160	−3.86188	−1.69140
H	6.27962	−4.22445	−0.73382
H	6.64079	−3.29106	−2.22451
H	5.62477	−4.75374	−2.31347
C	3.99687	−2.56325	−2.82320
H	3.57830	−3.44292	−3.35714
H	4.76415	−2.09911	−3.47891
H	3.17879	−1.82799	−2.68950
H	0.12392	0.02862	1.23508

TS(1^{Me}–E^{Me})

SCF (BP86) Energy = −1596.133501
Enthalpy 0K = −1595.407421
Enthalpy 298K = −1595.363067
Free Energy 298K = −1595.485301
Lowest Frequency = −550.6330 cm^{-1}
Second Frequency = 8.7043 cm^{-1}
SCF (BP86-$D3^{BJ}$) Energy = −1596.330472
SCF (C_6H_6) Energy = −1596.160610
SCF (BS2) Energy = −1597.712189

N	1.60890	0.06621	−0.76864
C	2.59450	0.02448	−3.04376
H	2.34428	0.45023	−4.03408
H	2.89318	−1.03458	−3.20089
H	3.48150	0.54622	−2.63544
C	1.40492	0.09173	−2.09771
C	2.93428	0.04032	−0.22237
C	3.51211	1.26187	0.25075
C	4.78928	1.21855	0.84595
H	5.24862	2.15198	1.20692
C	5.48689	0.01078	0.99093

H	6.48356	-0.00066	1.46026
C	4.90458	-1.18114	0.54144
H	5.45157	-2.12991	0.66631
C	3.63202	-1.19684	-0.06744
C	2.78079	2.59860	0.09104
H	1.69520	2.36267	0.03669
C	2.99195	3.55718	1.27904
H	2.78283	3.06675	2.25222
H	2.31947	4.43567	1.18825
H	4.03028	3.94992	1.31885
C	3.15623	3.30198	-1.23300
H	4.24381	3.52787	-1.26523
H	2.60665	4.26165	-1.33827
H	2.91396	2.67934	-2.11643
C	3.03629	-2.53439	-0.51710
H	2.09994	-2.31326	-1.07089
C	3.97372	-3.30211	-1.47388
H	4.27454	-2.68373	-2.34502
H	3.47371	-4.21575	-1.85919
H	4.90447	-3.62904	-0.96324
C	2.65048	-3.41205	0.69409
H	3.53824	-3.65035	1.31839
H	2.20816	-4.37353	0.35596
H	1.90057	-2.90195	1.33427
C	0.05854	1.08959	2.52020
H	0.98838	1.67509	2.43681
H	-0.88975	1.65191	2.56473
C	0.10831	-0.23017	3.01269
H	1.10861	-0.63181	3.25957
H	0.02816	-1.51357	1.73334
C	-1.02736	-0.83740	3.82065
H	-1.05087	-0.25290	4.77113
H	-1.99792	-0.62254	3.32295
C	-0.89950	-2.32716	4.14870
H	-0.89789	-2.94096	3.22521
H	-1.73826	-2.66674	4.79040
H	0.04555	-2.53978	4.69259
Mg	0.02834	-0.11648	0.56035
N	-1.46710	0.13395	-0.85193
C	-2.32432	0.19491	-3.17728
H	-3.16936	0.81998	-2.82834
H	-1.97891	0.56686	-4.16035
H	-2.73093	-0.82816	-3.32816
C	-1.18553	0.16563	-2.16849
C	0.12550	0.14485	-2.71537
H	0.15626	0.17235	-3.81379
C	-2.82442	0.15311	-0.39081
C	-3.36995	1.37771	0.11082
C	-4.68473	1.37252	0.61995
H	-5.11961	2.30921	1.00188
C	-5.45097	0.19863	0.65130
H	-6.47636	0.21682	1.05378
C	-4.90151	-0.99821	0.17358
H	-5.50349	-1.92066	0.20986
C	-3.59334	-1.05093	-0.35151
C	-2.56215	2.67858	0.07006
H	-1.48892	2.38723	0.09862
C	-2.82615	3.60170	1.27486
H	-2.72926	3.06315	2.24010
H	-2.10260	4.44345	1.27976
H	-3.84166	4.05086	1.24212
C	-2.78365	3.44918	-1.25118
H	-3.85190	3.73147	-1.36945
H	-2.18166	4.38267	-1.26751
H	-2.49186	2.84826	-2.13477
C	-3.02911	-2.39431	-0.82437
H	-2.08020	-2.18858	-1.36182
C	-3.97081	-3.12288	-1.80611
H	-4.25159	-2.47989	-2.66627
H	-3.48252	-4.03605	-2.20693
H	-4.91216	-3.44659	-1.31336
C	-2.67825	-3.30121	0.37581
H	-3.57896	-3.52645	0.98609
H	-2.25347	-4.26724	0.02840
H	-1.92593	-2.81890	1.03484

E^{Me}

SCF (BP86) Energy = -1596.148606
Enthalpy 0K = -1595.422626
Enthalpy 298K = -1595.376808
Free Energy 298K = -1595.503914
Lowest Frequency = 11.5122 cm^{-1}
Second Frequency = 15.9484 cm^{-1}
SCF (BP86-D3^{BJ}) Energy = -1596.346335
SCF (C_6H_6) Energy = -1596.178144
SCF (BS2) Energy = -1597.727009

N	1.42555	-0.07645	-0.90382
C	2.24578	0.07149	-3.24555
H	1.91766	0.60861	-4.15622
H	2.53854	-0.95569	-3.55445
H	3.15460	0.55695	-2.84242
C	1.12833	-0.00760	-2.21511
C	2.77155	0.07586	-0.43506
C	3.23147	1.37404	-0.04133
C	4.52838	1.49917	0.49725
H	4.89513	2.49460	0.79495
C	5.36234	0.38379	0.66122
H	6.37164	0.50310	1.08648
C	4.90191	-0.88240	0.27822
H	5.55980	-1.75757	0.40633
C	3.61640	-1.06567	-0.27371
C	2.36311	2.62180	-0.23233
H	1.31816	2.26634	-0.36393
C	2.39325	3.57504	0.97946
H	2.15420	3.05237	1.92829
H	1.65547	4.39417	0.84676
H	3.38684	4.05500	1.10646
C	2.74805	3.38979	-1.51752
H	3.79807	3.74959	-1.46324
H	2.09306	4.27581	-1.65929
H	2.65724	2.75616	-2.42152
C	3.18121	-2.47263	-0.69397
H	2.16014	-2.38970	-1.12299
C	4.10200	-3.04995	-1.79215
H	4.17928	-2.37170	-2.66681
H	3.71757	-4.02858	-2.14985
H	5.13230	-3.21872	-1.41222
C	3.09418	-3.43531	0.50910
H	4.07972	-3.55370	1.00829
H	2.76248	-4.44227	0.17802
H	2.36062	-3.07219	1.25731
C	-0.25740	1.10874	2.37220
H	-0.05119	1.92988	1.66686
H	-1.30614	0.76916	2.45974
C	0.71099	0.62537	3.18657
H	1.73662	1.03092	3.07714
H	0.00398	-2.16588	1.43216

C	0.52558	-0.40533	4.26563
H	0.48494	0.14510	5.23646
H	-0.46309	-0.89783	4.14557
C	1.65005	-1.45130	4.32847
H	1.67883	-2.04786	3.39448
H	1.49122	-2.14909	5.17562
H	2.64263	-0.97240	4.46631
Mg	-0.02488	-0.73533	0.45072
N	-1.63035	-0.14119	-0.76221
C	-2.66187	-0.07989	-3.02665
H	-3.58561	0.26382	-2.52526
H	-2.47855	0.54342	-3.92354
H	-2.84655	-1.11699	-3.38225
C	-1.45720	-0.07649	-2.09491
C	-0.19059	-0.05133	-2.73996
H	-0.24277	-0.02008	-3.83820
C	-2.93541	-0.04260	-0.17549
C	-3.44555	1.24575	0.18941
C	-4.70132	1.32136	0.82614
H	-5.10634	2.30781	1.10295
C	-5.44526	0.16892	1.11600
H	-6.42413	0.25093	1.61482
C	-4.93148	-1.08685	0.76903
H	-5.51623	-1.99231	0.99993
C	-3.68328	-1.22300	0.12543
C	-2.67644	2.53364	-0.12430
H	-1.62255	2.23813	-0.31748
C	-2.68295	3.54255	1.04267
H	-2.35414	3.08165	1.99614
H	-2.00420	4.39317	0.82161
H	-3.69253	3.97382	1.21172
C	-3.20241	3.21434	-1.40890
H	-4.26637	3.51234	-1.28992
H	-2.61768	4.13098	-1.63700
H	-3.13850	2.54613	-2.28960
C	-3.19036	-2.62390	-0.24789
H	-2.18235	-2.51164	-0.70090
C	-4.10530	-3.28180	-1.30519
H	-4.21875	-2.64627	-2.20729
H	-3.69203	-4.26160	-1.62585
H	-5.12339	-3.46692	-0.90040
C	-3.03632	-3.53465	0.98778
H	-4.00684	-3.69058	1.50581
H	-2.65428	-4.53310	0.68666
H	-2.31283	-3.10556	1.71007

TS(E^{Me}-F^{Me})

SCF (BP86) Energy = -1596.147802
Enthalpy 0K = -1595.421671
Enthalpy 298K = -1595.376744
Free Energy 298K = -1595.500870
Lowest Frequency = -35.2351 cm^{-1}
Second Frequency = 11.8374 cm^{-1}
SCF (BP86-D3^{BJ}) Energy = -1596.341329
SCF (C_6H_6) Energy = -1596.178151
SCF (BS2) Energy = -1597.726165

N	-1.37403	0.39939	-0.89804
C	-2.15491	0.97009	-3.17558
H	-1.74828	1.29809	-4.15006
H	-2.85338	1.74505	-2.79834
H	-2.76789	0.06044	-3.34018
C	-1.04729	0.70636	-2.16944
C	-2.75374	0.28361	-0.51550
C	-3.45410	-0.94739	-0.71505
C	-4.78029	-1.05510	-0.24881
H	-5.32665	-2.00120	-0.39593
C	-5.41810	0.01169	0.39874
H	-6.45475	-0.09485	0.75602
C	-4.72602	1.21522	0.58583
H	-5.23024	2.05574	1.09013
C	-3.39830	1.37973	0.13872
C	-2.80500	-2.15539	-1.39837
H	-1.81109	-1.83288	-1.77201
C	-2.56570	-3.30379	-0.39459
H	-1.94826	-2.96676	0.46195
H	-2.04493	-4.15512	-0.88281
H	-3.52465	-3.68721	0.01502
C	-3.61963	-2.65805	-2.60960
H	-4.60795	-3.06060	-2.30159
H	-3.07921	-3.47573	-3.13183
H	-3.80761	-1.85116	-3.34806
C	-2.70670	2.73114	0.34367
H	-1.66903	2.63322	-0.04165
C	-3.39185	3.84769	-0.47567
H	-3.43906	3.59859	-1.55607
H	-2.83941	4.80564	-0.37226
H	-4.43272	4.02356	-0.12934
C	-2.61217	3.11955	1.83389
H	-3.61800	3.23659	2.29079
H	-2.07991	4.08752	1.94982
H	-2.05202	2.35596	2.41122
C	0.32381	-2.42802	2.23914
H	0.37097	-3.15461	1.41217
H	1.19981	-1.77154	2.38839
C	-0.72366	-2.40279	3.09201
H	-1.55990	-3.10894	2.91765
H	0.03675	0.88281	2.22567
C	-0.86723	-1.50167	4.28836
H	-0.68230	-2.11278	5.20397
H	-0.07684	-0.72156	4.25980
C	-2.25609	-0.85009	4.39512
H	-2.45460	-0.19373	3.52319
H	-2.33421	-0.22883	5.31083
H	-3.06280	-1.61304	4.43648
Mg	0.06272	0.30454	0.59933
N	1.67867	0.26369	-0.71684
C	2.76511	0.92501	-2.84906
H	3.61278	0.25708	-2.60384
H	2.54018	0.84912	-3.92984
H	3.11289	1.96209	-2.65080
C	1.53408	0.62950	-2.00517
C	0.28276	0.82162	-2.65112
H	0.35913	1.11267	-3.70802
C	2.99096	0.07078	-0.16542
C	3.53641	-1.25181	-0.10886
C	4.80871	-1.43156	0.47168
H	5.24477	-2.44146	0.51514
C	5.52986	-0.35208	1.00149
H	6.52188	-0.51628	1.45157
C	4.97490	0.93291	0.96520
H	5.53762	1.77678	1.39647
C	3.70952	1.17337	0.38982
C	2.77323	-2.45395	-0.67656
H	1.69106	-2.20445	-0.60402
C	3.01356	-3.75595	0.11263
H	2.87055	-3.61664	1.20330
H	2.31210	-4.54635	-0.22786

H	4.03949	-4.15193	-0.04430
C	3.07749	-2.68732	-2.17421
H	4.16114	-2.87690	-2.33066
H	2.52068	-3.57074	-2.55319
H	2.79034	-1.82020	-2.79954
C	3.14498	2.59709	0.40359
H	2.20488	2.58895	-0.18799
C	4.09817	3.61373	-0.26100
H	4.37961	3.30846	-1.29031
H	3.62075	4.61462	-0.31975
H	5.03880	3.73287	0.31769
C	2.78189	3.04120	1.83828
H	3.68099	3.06257	2.49069
H	2.34621	4.06323	1.83281
H	2.03909	2.35590	2.29680

F^Me

SCF (BP86) Energy = -1596.154028
Enthalpy 0K = -1595.428579
Enthalpy 298K = -1595.382015
Free Energy 298K = -1595.517735
Lowest Frequency = 5.8538 cm^{-1}
Second Frequency = 6.3567 cm^{-1}
SCF (BP86-D3^{BJ}) Energy = -1596.336369
SCF (C_6H_6) Energy = -1596.186205
SCF (BS2) Energy = -1597.732778

N	-0.96823	1.55485	-0.11635
C	-1.29632	3.98815	-0.39414
H	-0.70715	4.91555	-0.51765
H	-1.94243	4.09236	0.50221
H	-1.98766	3.88714	-1.25656
C	-0.40104	2.76725	-0.27503
C	-2.39900	1.43023	-0.06085
C	-3.13352	1.17778	-1.25873
C	-4.52610	0.97706	-1.16700
H	-5.10372	0.77830	-2.08407
C	-5.18964	1.02134	0.06683
H	-6.27849	0.86179	0.11669
C	-4.45821	1.26498	1.23740
H	-4.98263	1.29209	2.20613
C	-3.06412	1.47269	1.20170
C	-2.43435	1.06964	-2.61618
H	-1.41245	1.48658	-2.49422
C	-2.28094	-0.41134	-3.03092
H	-1.74910	-1.00471	-2.25612
H	-1.71608	-0.50350	-3.98294
H	-3.27350	-0.88834	-3.17689
C	-3.13131	1.88488	-3.72373
H	-4.14007	1.48607	-3.96225
H	-2.53828	1.85161	-4.66188
H	-3.25258	2.94976	-3.43533
C	-2.29008	1.68393	2.50540
H	-1.27964	2.05447	2.23218
C	-2.93102	2.74145	3.42620
H	-3.07653	3.70873	2.90175
H	-2.28664	2.92754	4.31112
H	-3.92181	2.41577	3.80805
C	-2.10238	0.34195	3.24891
H	-3.08149	-0.08233	3.55767
H	-1.48575	0.47452	4.16329
H	-1.60854	-0.42022	2.60829
C	-0.94723	-5.36260	0.71328
H	-0.06595	-6.02299	0.77188
H	-0.75732	-4.28307	0.57158
C	-2.19646	-5.85385	0.81561
H	-2.33295	-6.94538	0.95347
H	-0.32881	-1.83006	0.26232
C	-3.46510	-5.04140	0.74315
H	-4.01621	-5.14383	1.70777
H	-3.20969	-3.96419	0.64314
C	-4.39840	-5.47335	-0.40355
H	-3.90766	-5.33900	-1.39020
H	-5.33679	-4.88011	-0.40548
H	-4.68048	-6.54478	-0.31631
Mg	0.12635	-0.18248	0.05588
N	1.95590	0.75169	-0.10670
C	3.48259	2.67623	-0.37363
H	4.03411	2.23334	-1.22889
H	3.45024	3.77397	-0.50148
H	4.08262	2.43966	0.52963
C	2.08854	2.08348	-0.26555
C	0.99955	2.99195	-0.34083
H	1.28890	4.04367	-0.47222
C	3.12207	-0.08607	-0.04187
C	3.63547	-0.67668	-1.23587
C	4.72995	-1.55982	-1.13519
H	5.13338	-2.02480	-2.04906
C	5.31195	-1.86181	0.10359
H	6.16629	-2.55490	0.16035
C	4.79642	-1.28086	1.27031
H	5.25197	-1.52713	2.24292
C	3.70383	-0.39083	1.22572
C	2.98970	-0.41340	-2.59854
H	2.32434	0.46808	-2.48311
C	2.10267	-1.60779	-3.01761
H	1.33656	-1.84407	-2.24786
H	1.57716	-1.39945	-3.97383
H	2.71200	-2.52601	-3.15733
C	4.01367	-0.07184	-3.69932
H	4.67711	-0.93153	-3.93242
H	3.49394	0.20250	-4.64143
H	4.66152	0.78073	-3.40733
C	3.13252	0.18276	2.52485
H	2.45623	1.01837	2.24651
C	4.21342	0.76195	3.45920
H	4.83856	1.52037	2.94358
H	3.74472	1.24892	4.34018
H	4.89431	-0.02526	3.84675
C	2.27788	-0.87748	3.25598
H	2.90006	-1.74271	3.56948
H	1.80473	-0.45127	4.16614
H	1.47186	-1.27991	2.60508

G^Me

SCF (BP86) Energy = -1439.057279
Enthalpy 0K = -1438.436997
Enthalpy 298K = -1438.398334
Free Energy 298K = -1438.508460
Lowest Frequency = 14.7575 cm^{-1}
Second Frequency = 18.6340 cm^{-1}
SCF (BP86-D3^{BJ}) Energy = -1439.226213
SCF (C_6H_6) Energy = -1439.086140
SCF (BS2) Energy = -1440.459913

N	-1.51662	-0.00021	0.45834
C	-2.47774	-0.00038	2.73493
H	-2.15511	-0.00093	3.79246

H	-3.12344	-0.88567	2.55839
H	-3.12282	0.88553	2.55918
C	-1.29107	-0.00035	1.78707
C	-2.86317	0.00002	-0.04401
C	-3.50984	1.23954	-0.33303
C	-4.79950	1.21154	-0.90208
H	-5.30764	2.16161	-1.13288
C	-5.44620	0.00044	-1.18406
H	-6.45406	0.00061	-1.62878
C	-4.79990	-1.21088	-0.90207
H	-5.30838	-2.16078	-1.13281
C	-3.51024	-1.23930	-0.33303
C	-2.81119	2.57986	-0.09101
H	-1.93780	2.38228	0.56561
C	-2.26813	3.15453	-1.41914
H	-1.59599	2.43599	-1.93606
H	-1.70090	4.09384	-1.24618
H	-3.09755	3.38241	-2.12213
C	-3.70352	3.60771	0.63296
H	-4.56923	3.91931	0.01084
H	-3.12534	4.52592	0.86870
H	-4.10477	3.20419	1.58587
C	-2.81198	-2.57982	-0.09094
H	-1.93888	-2.38253	0.56614
C	-3.70479	-3.60771	0.63238
H	-4.10650	-3.20431	1.58515
H	-3.12677	-4.52597	0.86828
H	-4.57017	-3.91919	0.00977
C	-2.26842	-3.15428	-1.41896
H	-3.09761	-3.38181	-2.12235
H	-1.70144	-4.09374	-1.24600
H	-1.59596	-2.43574	-1.93548
H	-0.00070	0.00012	-2.65788
Mg	0.00005	-0.00006	-0.93870
N	1.51670	-0.00012	0.45828
C	2.47783	-0.00053	2.73486
H	3.12334	0.88499	2.55878
H	2.15522	-0.00059	3.79239
H	3.12309	-0.88621	2.55863
C	1.29113	-0.00032	1.78704
C	0.00005	-0.00043	2.37870
H	0.00006	-0.00061	3.47740
C	2.86327	0.00000	-0.04401
C	3.51014	1.23951	-0.33276
C	4.79986	1.21142	-0.90165
H	5.30815	2.16146	-1.13226
C	5.44644	0.00028	-1.18376
H	6.45431	0.00038	-1.62842
C	4.79996	-1.21100	-0.90200
H	5.30835	-2.16093	-1.13284
C	3.51024	-1.23934	-0.33312
C	2.81145	2.57979	-0.09067
H	1.93876	2.38234	0.56690
C	2.26697	3.15367	-1.41857
H	1.59460	2.43471	-1.93466
H	1.69958	4.09288	-1.24557
H	3.09572	3.38141	-2.12240
C	3.70418	3.60831	0.63183
H	4.56899	3.92013	0.00858
H	3.12583	4.52632	0.86789
H	4.10674	3.20535	1.58443
C	2.81180	-2.57980	-0.09127
H	1.93869	-2.38250	0.56581
C	3.70447	-3.60789	0.63191
H	4.10626	-3.20468	1.58472
H	3.12635	-4.52613	0.86769
H	4.56981	-3.91940	0.00924
C	2.26821	-3.15402	-1.41938
H	3.09737	-3.38162	-2.12276
H	1.70105	-4.09340	-1.24653
H	1.59585	-2.43534	-1.93585

2G^{Me}

SCF (BP86) Energy = -2878.116040
Enthalpy 0K = -2876.875142
Enthalpy 298K = -2876.796126
Free Energy 298K = -2877.005282
Lowest Frequency = 2.7408 cm^{-1}
Second Frequency = 4.5340 cm^{-1}
SCF (BP86-D3^{BJ}) Energy = -2878.461930
SCF (C_6H_6) Energy = -2878.171770
SCF (BS2) Energy = -2880.920498

N	-4.04587	0.85322	-1.09702
C	-3.83096	1.28927	-3.51903
H	-3.65059	2.37698	-3.39245
H	-4.38376	1.12656	-4.46275
H	-2.82730	0.82392	-3.60791
C	-4.58188	0.72522	-2.32550
C	-2.77691	1.51046	-0.92959
C	-2.73893	2.90811	-0.64383
C	-1.48784	3.51224	-0.40259
H	-1.44482	4.59029	-0.17706
C	-0.29936	2.76976	-0.44032
H	0.66850	3.25977	-0.24842
C	-0.34732	1.39600	-0.71847
H	0.59122	0.81751	-0.73949
C	-1.57323	0.74368	-0.96575
C	-4.02079	3.73935	-0.54510
H	-4.84145	3.13256	-0.98285
C	-4.38427	4.00671	0.93307
H	-4.47349	3.06270	1.51276
H	-5.34731	4.55449	1.01622
H	-3.60128	4.61806	1.43051
C	-3.95122	5.05604	-1.34437
H	-3.19615	5.75590	-0.92754
H	-4.93029	5.57968	-1.31878
H	-3.68860	4.87684	-2.40776
C	-1.59738	-0.76723	-1.20968
H	-2.58831	-1.01784	-1.64429
C	-0.52106	-1.23777	-2.20831
H	-0.59229	-0.69179	-3.17236
H	-0.64026	-2.32115	-2.42193
H	0.50626	-1.09122	-1.81266
C	-1.47645	-1.53015	0.12943
H	-0.49465	-1.33044	0.60809
H	-1.56335	-2.62670	-0.02633
H	-2.25981	-1.22215	0.85605
Mg	-4.94895	0.12714	0.60651
N	-6.59573	-0.62517	-0.38331
C	-8.00205	-1.12062	-2.35452
H	-8.91709	-0.65802	-1.92952
H	-8.00772	-0.97734	-3.45090
H	-8.08128	-2.20597	-2.13622
C	-6.75414	-0.53173	-1.71940
C	-5.82389	0.08833	-2.59357
H	-6.11155	0.07370	-3.65389
C	-7.59932	-1.27021	0.41687

C	-8.65208	-0.49547	0.99136
C	-9.57837	-1.13713	1.83882
H	-10.39294	-0.54869	2.29108
C	-9.48152	-2.50653	2.12146
H	-10.21573	-2.98877	2.78632
C	-8.44074	-3.25704	1.55742
H	-8.36294	-4.33164	1.78861
C	-7.48796	-2.66447	0.70368
C	-8.75634	1.01239	0.74960
H	-8.09223	1.25494	-0.10657
C	-8.23228	1.79510	1.97507
H	-7.19457	1.49907	2.24072
H	-8.24042	2.88935	1.78365
H	-8.86225	1.60145	2.86943
C	-10.17884	1.46863	0.36839
H	-10.89906	1.32738	1.20210
H	-10.18475	2.54929	0.11300
H	-10.56836	0.90978	-0.50791
C	-6.33191	-3.50206	0.15153
H	-5.85984	-2.91445	-0.66376
C	-6.78959	-4.84251	-0.45718
H	-7.56628	-4.69580	-1.23639
H	-5.93173	-5.36870	-0.92643
H	-7.21056	-5.52654	0.31005
C	-5.25730	-3.72523	1.23985
H	-5.66571	-4.31869	2.08559
H	-4.38153	-4.27264	0.83083
H	-4.89483	-2.76469	1.66548
N	5.38453	1.40567	0.69420
C	7.34254	2.55788	1.66537
H	7.37539	3.34726	0.88586
H	8.37489	2.34360	1.99816
H	6.77693	2.99360	2.51515
C	6.65240	1.31077	1.14159
C	4.70386	2.67131	0.71711
C	4.74968	3.51909	-0.43066
C	4.00391	4.71564	-0.41573
H	4.02873	5.37761	-1.29619
C	3.23011	5.07857	0.69552
H	2.65659	6.01907	0.68809
C	3.18895	4.23642	1.81576
H	2.57403	4.52248	2.68395
C	3.91586	3.02883	1.85277
C	5.54342	3.12946	-1.68028
H	6.22583	2.30229	-1.39203
C	4.59905	2.58607	-2.77656
H	3.98576	1.73487	-2.40965
H	5.17278	2.24000	-3.66256
H	3.88965	3.37062	-3.11590
C	6.41922	4.27602	-2.22377
H	5.80861	5.12116	-2.60641
H	7.04577	3.91915	-3.06816
H	7.09745	4.67995	-1.44362
C	3.80686	2.10730	3.07019
H	4.63443	1.37019	3.00034
C	3.96927	2.85060	4.41102
H	4.91356	3.43285	4.44658
H	3.98187	2.12903	5.25476
H	3.13290	3.55624	4.60039
C	2.48031	1.31505	3.03336
H	1.60584	1.99591	3.10345
H	2.42067	0.59568	3.87749
H	2.36439	0.74461	2.08651
Mg	4.33046	-0.20513	-0.03609
N	5.86284	-1.55353	0.23497
C	8.12627	-2.27749	0.91202
H	8.36524	-2.74727	-0.06473
H	9.05596	-1.86389	1.34444
H	7.76447	-3.09970	1.56385
C	7.05998	-1.20843	0.74984
C	7.40503	0.10664	1.15973
H	8.42398	0.20963	1.55748
C	5.60979	-2.91478	-0.15137
C	5.86491	-3.32751	-1.49402
C	5.53080	-4.64465	-1.87011
H	5.71980	-4.97459	-2.90433
C	4.95979	-5.54134	-0.95663
H	4.70606	-6.56623	-1.27086
C	4.70930	-5.12441	0.35787
H	4.25413	-5.83042	1.07093
C	5.02449	-3.81852	0.78609
C	6.43791	-2.35925	-2.53204
H	6.83945	-1.48367	-1.97967
C	5.32054	-1.84115	-3.46578
H	4.48990	-1.37201	-2.89553
H	5.71454	-1.08853	-4.18150
H	4.87758	-2.67201	-4.05527
C	7.60250	-2.96032	-3.34411
H	7.27057	-3.80243	-3.98769
H	8.04490	-2.19353	-4.01435
H	8.40926	-3.34188	-2.68430
C	4.68892	-3.38160	2.21428
H	5.22576	-2.42789	2.40186
C	5.15970	-4.38849	3.28273
H	6.24349	-4.60900	3.18993
H	4.98014	-3.98548	4.30163
H	4.61482	-5.35383	3.21418
C	3.17675	-3.09357	2.35243
H	2.57919	-4.01427	2.18181
H	2.93285	-2.71299	3.36697
H	2.82950	-2.34217	1.61073
H	-4.52875	0.15243	2.27437
H	2.73256	-0.35677	-0.66533

TS(2G^{Me}–[2^{Mg,Me}]$_2$)

SCF (BP86) Energy = -2878.111588
Enthalpy 0K = -2876.870148
Enthalpy 298K = -2876.792680
Free Energy 298K = -2876.989627
Lowest Frequency = -9.0425 cm^{-1}
Second Frequency = 8.7667 cm^{-1}
SCF (BP86-D3^{BJ}) Energy = -2878.475627
SCF (C_6H_6) Energy = -2878.165320
SCF (BS2) Energy = -2880.915109

N	-3.56231	1.41577	-0.77922
C	-5.71737	2.17386	-1.72669
H	-5.75744	3.07929	-1.08654
H	-6.74697	1.80488	-1.88868
H	-5.30386	2.50803	-2.70017
C	-4.83591	1.10869	-1.09719
C	-3.04845	2.72194	-1.09326
C	-3.05075	3.74042	-0.09220
C	-2.47195	4.98939	-0.39726
H	-2.46528	5.78091	0.36968
C	-1.90746	5.24472	-1.65389
H	-1.46124	6.22798	-1.87233
C	-1.91388	4.23955	-2.63057

H	-1.46751	4.44241	-3.61776	H	5.30599	2.51028	2.69658
C	-2.47647	2.97150	-2.37864	C	4.83666	1.10921	1.09556
C	-3.65508	3.51102	1.29591	C	3.04869	2.72192	1.09297
H	-4.13961	2.51146	1.28267	C	3.05037	3.73972	0.09122
C	-2.56394	3.48368	2.38831	C	2.47173	4.98887	0.39578
H	-1.80310	2.70172	2.18460	H	2.46449	5.77987	-0.37169
H	-3.00994	3.27818	3.38496	C	1.90802	5.24503	1.65261
H	-2.03266	4.45715	2.45005	H	1.46189	6.22841	1.87065
C	-4.75220	4.54288	1.63399	C	1.91507	4.24052	2.62995
H	-4.33905	5.57101	1.71260	H	1.46930	4.44403	3.61727
H	-5.22718	4.30319	2.60892	C	2.47751	2.97229	2.37852
H	-5.55050	4.56337	0.86310	C	3.65351	3.50909	-1.29720
C	-2.44387	1.90492	-3.47751	H	4.13969	2.51036	-1.28293
H	-3.00681	1.02493	-3.10228	C	2.56098	3.47809	-2.38813
C	-3.13621	2.37800	-4.77416	H	1.80150	2.69539	-2.18208
H	-4.17951	2.71084	-4.59247	H	3.00591	3.27158	-3.38505
H	-3.16830	1.55671	-5.52107	H	2.02801	4.45058	-2.45074
H	-2.59408	3.22734	-5.24180	C	4.74847	4.54216	-1.63851
C	-1.00110	1.43924	-3.76981	H	4.33357	5.56945	-1.71868
H	-0.38599	2.27205	-4.17316	H	5.22273	4.30138	-2.61352
H	-0.99807	0.62631	-4.52714	H	5.54760	4.56546	-0.86858
H	-0.48843	1.06161	-2.85982	C	2.44572	1.90651	3.47818
Mg	-2.31983	0.11114	0.24453	H	3.00749	1.02585	3.10272
N	-3.64833	-1.47618	0.14604	C	3.14046	2.38008	4.77338
C	-5.82358	-2.56266	-0.30553	H	4.18381	2.71176	4.58986
H	-5.93790	-2.94622	0.72946	H	3.17289	1.55942	5.52099
H	-6.82418	-2.31950	-0.70831	H	2.59981	3.23036	5.24104
H	-5.39806	-3.40123	-0.89519	C	1.00307	1.44214	3.77310
C	-4.90049	-1.35635	-0.33708	H	0.38924	2.27565	4.17695
C	-5.43521	-0.15992	-0.88139	H	1.00067	0.62972	4.53099
H	-6.48325	-0.23143	-1.20304	H	0.48849	1.06433	2.86425
C	-3.20051	-2.74410	0.64986	Mg	2.31830	0.10934	-0.24000
C	-3.30742	-3.03644	2.04241	N	3.64859	-1.47647	-0.14550
C	-2.79436	-4.26286	2.51487	C	5.82490	-2.56190	0.30344
H	-2.87023	-4.50031	3.58856	H	5.93908	-2.94479	-0.73181
C	-2.19496	-5.18532	1.64720	H	6.82554	-2.31857	0.70603
H	-1.80646	-6.14024	2.03594	H	5.39987	-3.40101	0.89270
C	-2.09457	-4.88734	0.27971	C	4.90132	-1.35600	0.33602
H	-1.62045	-5.61403	-0.39809	C	5.43616	-0.15926	0.87951
C	-2.58825	-3.67579	-0.24401	H	6.48462	-0.23026	1.19992
C	-3.94622	-2.05438	3.02790	C	3.20102	-2.74457	-0.64913
H	-4.38410	-1.22785	2.42919	C	3.30842	-3.03731	-2.04153
C	-2.88717	-1.43409	3.96584	C	2.79595	-4.26409	-2.51369
H	-2.08759	-0.92188	3.39083	H	2.87212	-4.50191	-3.58728
H	-3.35189	-0.69022	4.64777	C	2.19669	-5.18646	-1.64582
H	-2.40484	-2.21207	4.59571	H	1.80870	-6.14168	-2.03432
C	-5.09552	-2.69373	3.83537	C	2.09567	-4.88800	-0.27848
H	-4.72970	-3.50116	4.50488	H	1.62160	-5.61461	0.39942
H	-5.59110	-1.93453	4.47673	C	2.58868	-3.67602	0.24490
H	-5.86898	-3.13570	3.17322	C	3.94638	-2.05480	-3.02709
C	-2.43022	-3.35036	-1.73144	H	4.38666	-1.22966	-2.42824
H	-3.20527	-2.59744	-1.98703	C	2.88570	-1.43182	-3.96144
C	-2.65219	-4.56450	-2.65384	H	2.08785	-0.91985	-3.38377
H	-3.62124	-5.06616	-2.45011	H	3.34945	-0.68730	-4.64333
H	-2.65020	-4.24461	-3.71691	H	2.40100	-2.20835	-4.59128
H	-1.84911	-5.32383	-2.54515	C	5.09291	-2.69419	-3.83837
C	-1.05573	-2.70083	-2.00549	H	4.72459	-3.49982	-4.50866
H	-0.22668	-3.40104	-1.77051	H	5.58805	-1.93442	-4.47940
H	-0.96070	-2.40278	-3.07085	H	5.86728	-3.13844	-3.17879
H	-0.88403	-1.78938	-1.39277	C	2.42984	-3.34951	1.73199
N	3.56243	1.41555	0.77942	H	3.20647	-2.59834	1.98799
C	5.71862	2.17512	1.72307	C	2.64770	-4.56353	2.65546
H	5.75797	3.07988	1.08192	H	3.61566	-5.06783	2.45309
H	6.74843	1.80644	1.88442	H	2.64543	-4.24286	3.71829

H	1.84272	-5.32083	2.54651
C	1.05659	-2.69650	2.00411
H	0.22618	-3.39515	1.76931
H	0.96127	-2.39672	3.06893
H	0.88723	-1.78554	1.38992
H	-0.94537	0.28046	1.27744
H	0.93726	0.27577	-1.26483

[2^{Mg,Me}]$_2$

SCF (BP86) Energy = -2878.132575
Enthalpy 0K = -2876.887408
Enthalpy 298K = -2876.811220
Free Energy 298K = -2876.999110
Lowest Frequency = 14.8193 cm^{-1}
Second Frequency = 16.9809 cm^{-1}
SCF (BP86-D3^{BJ}) Energy = -2878.519870
SCF (C_6H_6) Energy = -2878.178548
SCF (BS2) Energy = -2880.936697

N	-1.49537	-2.94914	-0.28120
C	-2.38985	-5.27071	-0.26956
H	-3.30642	-5.08291	0.32680
H	-2.03957	-6.29705	-0.05439
H	-2.70342	-5.21603	-1.33135
C	-1.30428	-4.24712	0.03814
C	-2.74656	-2.62174	-0.91024
C	-3.91257	-2.43493	-0.10336
C	-5.13718	-2.16236	-0.74515
H	-6.04550	-2.02192	-0.13971
C	-5.22705	-2.08407	-2.14369
H	-6.19783	-1.88488	-2.62515
C	-4.07634	-2.25831	-2.92254
H	-4.15132	-2.19345	-4.02018
C	-2.82191	-2.52048	-2.33120
C	-3.83410	-2.53505	1.42350
H	-3.04315	-3.27593	1.66544
C	-3.39313	-1.19479	2.04479
H	-2.41187	-0.85446	1.65254
H	-3.29449	-1.28301	3.14747
H	-4.12679	-0.39195	1.83027
C	-5.13510	-3.03164	2.08140
H	-5.95361	-2.28483	2.00420
H	-4.96922	-3.21470	3.16354
H	-5.49684	-3.97791	1.62721
C	-1.59738	-2.73649	-3.22487
H	-0.70967	-2.76518	-2.55765
C	-1.66006	-4.09097	-3.96561
H	-1.73505	-4.94535	-3.26269
H	-0.74969	-4.24371	-4.58342
H	-2.53862	-4.13656	-4.64453
C	-1.39091	-1.58072	-4.22330
H	-2.22535	-1.51073	-4.95349
H	-0.45794	-1.73350	-4.80541
H	-1.30939	-0.60807	-3.69867
Mg	-0.04288	-1.46260	0.11175
N	1.39465	-2.85678	0.78296
C	2.12492	-5.08624	1.61515
H	2.20281	-4.89275	2.70543
H	1.83768	-6.14564	1.47965
H	3.13920	-4.93137	1.19889
C	1.09580	-4.15746	0.98381
C	-0.13832	-4.76786	0.64994
H	-0.18281	-5.84373	0.86601
C	2.73638	-2.44151	1.08815
C	3.12109	-2.13723	2.42923
C	4.46459	-1.78619	2.67830
H	4.77908	-1.56092	3.71016
C	5.40761	-1.72223	1.64424
H	6.45285	-1.45159	1.86317
C	5.01206	-1.99501	0.32704
H	5.75550	-1.93004	-0.48135
C	3.68460	-2.35753	0.02026
C	2.12792	-2.17574	3.59544
H	1.19916	-2.65784	3.22505
C	1.74861	-0.75084	4.05298
H	1.31482	-0.16218	3.21952
H	0.99788	-0.78971	4.87071
H	2.63721	-0.20561	4.43634
C	2.64039	-3.00273	4.79409
H	3.51294	-2.52061	5.28400
H	1.84722	-3.09974	5.56510
H	2.95261	-4.02549	4.49682
C	3.28657	-2.69934	-1.42102
H	2.22457	-2.38551	-1.54372
C	3.33914	-4.21930	-1.70096
H	2.63082	-4.79091	-1.07131
H	4.36117	-4.61631	-1.52022
C	4.11448	-1.94944	-2.47857
H	5.16321	-2.31488	-2.51695
H	3.68148	-2.11628	-3.48673
H	4.13924	-0.85795	-2.29060
H	-0.02775	-0.11293	-1.25143
N	-1.49405	2.94969	0.28117
C	-2.38767	5.27161	0.26953
H	-3.30427	5.08426	-0.32693
H	-2.03694	6.29782	0.05446
H	-2.70134	5.21697	1.33128
C	-1.30250	4.24759	-0.03821
C	-2.74535	2.62276	0.91026
C	-3.91146	2.43642	0.10345
C	-5.13616	2.16441	0.74530
H	-6.04457	2.02435	0.13990
C	-5.22601	2.08619	2.14384
H	-6.19686	1.88745	2.62535
C	-4.07518	2.25992	2.92264
H	-4.15014	2.19511	4.02027
C	-2.82067	2.52153	2.33123
C	-3.83302	2.53646	-1.42343
H	-3.04184	3.27706	-1.66544
C	-3.39249	1.19601	-2.04463
H	-2.41132	0.85542	-1.65240
H	-3.29388	1.28410	-3.14732
H	-4.12639	0.39341	-1.83001
C	-5.13388	3.03342	-2.08132
H	-5.95262	2.28687	-2.00406
H	-4.96797	3.21637	-3.16347
H	-5.49531	3.97983	-1.62716
C	-1.59598	2.73705	3.22481
H	-0.70828	2.76505	2.55755
C	-1.65785	4.09178	3.96516
H	-1.73228	4.94599	3.26198
H	-0.74740	4.24413	4.58296
H	-2.53640	4.13811	4.64402
C	-1.39016	1.58142	4.22353
H	-2.22455	1.51217	4.95384
H	-0.45702	1.73375	4.80549
H	-1.30932	0.60858	3.69916
Mg	-0.04210	1.46260	-0.11171

N 1.39590 2.85624 -0.78301
C 2.12698 5.08539 -1.61532
H 2.20502 4.89168 -2.70554
H 1.84000 6.14490 -1.48005
H 3.14114 4.93032 -1.19884
C 1.09751 4.15702 -0.98395
C -0.13639 4.76788 -0.65009
H -0.18048 5.84375 -0.86619
C 2.73751 2.44051 -1.08817
C 3.12211 2.13605 -2.42925
C 4.46549 1.78459 -2.67831
H 4.77990 1.55916 -3.71016
C 5.40850 1.72036 -1.64426
H 6.45365 1.44937 -1.86317
C 5.01304 1.99330 -0.32706
H 5.75646 1.92809 0.48133
C 3.68569 2.35625 -0.02028
C 2.12891 2.17475 -3.59542
H 1.20030 2.65716 -3.22505
C 1.74915 0.74992 -4.05281
H 1.31522 0.16145 -3.21928
H 0.99838 0.78895 -4.87050
H 2.63756 0.20438 -4.43618
C 2.64159 3.00147 -4.79418
H 3.51395 2.51903 -5.28408
H 1.84840 3.09866 -5.56515
H 2.95414 4.02416 -4.49701
C 3.28780 2.69816 1.42101
H 2.22568 2.38471 1.54370
C 3.34092 4.21808 1.70103
H 2.63294 4.79000 1.07126
H 4.36314 4.61469 1.52050
C 4.11543 1.94790 2.47852
H 5.16430 2.31291 2.51690
H 3.68250 2.11486 3.48669
H 4.13975 0.85640 2.29051
H -0.02791 0.11290 1.25147
H 3.08162 4.42411 2.76131
H 3.07997 -4.42527 -2.76128

H
SCF (BP86) Energy = -2296.015830
Enthalpy 0K = -2294.907051
Enthalpy 298K = -2294.849408
Free Energy 298K = -2295.001417
Lowest Frequency = 7.4715 cm^{-1}
Second Frequency = 10.8655 cm^{-1}
SCF (BP86-D3^{BJ}) Energy = -2296.334217
SCF (C6H6) Energy = -2296.058580
SCF (BS2) Energy = -2298.318173

Mg -3.99054 0.94130 -0.00002
N -2.21017 0.03085 -0.00001
N 2.28438 0.71677 0.00017
C -0.91074 0.49762 0.00009
C -0.77629 2.03996 0.00016
C -0.00768 2.51377 1.27133
H -0.57068 2.19933 2.17938
H 0.97587 2.00355 1.28575
C -2.14080 2.78921 0.00007
H -2.72370 2.52682 -0.92178
C -0.00749 2.51389 -1.27086
H 0.97605 2.00367 -1.28518
H -0.57036 2.19953 -2.17902
C 0.18706 4.04683 1.26301
H 0.75603 4.34092 2.17218
C -1.18637 4.74975 1.26371
H -1.75959 4.48013 2.17933
H -1.06264 5.85511 1.27745
C -1.96700 4.32848 0.00017
H -2.97433 4.80004 0.00012
C -1.18618 4.74988 -1.26321
H -1.06243 5.85524 -1.27682
H -1.75927 4.48035 -2.17894
C 0.18724 4.04695 -1.26237
H 0.75636 4.34112 -2.17143
C 0.97843 4.45915 0.00039
H 1.15235 5.55835 0.00046
H 1.97675 3.97183 0.00044
C 0.14769 -0.42588 0.00018
H -0.24061 -1.45414 0.00020
C 1.60804 -0.40140 0.00018
C 2.23449 -1.84790 0.00038
C 1.76384 -2.63275 1.26789
H 2.11039 -2.09617 2.17902
H 0.65531 -2.65885 1.31689
C 3.78703 -1.86745 0.00052
H 4.17625 -1.32581 0.88505
H 4.17644 -1.32610 -0.88409
C 1.76399 -2.63318 -1.26691
H 0.65546 -2.65928 -1.31601
H 2.11064 -2.09691 -2.17818
C 2.30741 -4.08043 1.26312
H 1.93910 -4.60484 2.17303
C 3.85032 -4.05726 1.26164
H 4.25317 -5.09478 1.28070
H 4.22709 -3.54932 2.17728
C 4.33898 -3.31352 0.00080
H 5.45020 -3.26607 0.00087
C 3.85049 -4.05766 -1.25986
H 4.25335 -5.09518 -1.27854
H 4.22739 -3.55001 -2.17561
C 2.30757 -4.08084 -1.26157
H 1.93942 -4.60557 -2.17136
C 1.80034 -4.81502 0.00086
H 0.68829 -4.85362 0.00080
H 2.15926 -5.86847 0.00105
C -2.49676 -1.37605 -0.00007
C -2.69845 -2.05780 -1.24117
C -2.50463 -1.32517 -2.57101
H -1.83811 -0.46194 -2.36447
C -1.82240 -2.18422 -3.65255
H -0.86196 -2.60221 -3.28917
H -1.60720 -1.57108 -4.55260
H -2.46080 -3.03134 -3.98212
C -3.84731 -0.76806 -3.09572
H -4.56485 -1.58707 -3.31454
H -3.70469 -0.17802 -4.02573
H -4.35158 -0.09875 -2.35942
C -3.12197 -3.40265 -1.21355
H -3.28897 -3.93752 -2.16193
C -3.33482 -4.07322 -0.00017
H -3.66704 -5.12354 -0.00022
C -3.12214 -3.40270 1.21325
H -3.28925 -3.93761 2.16159
C -2.69862 -2.05785 1.24098
C -2.50501 -1.32525 2.57087
C -1.82309 -2.18434 3.65258

H	-2.46170	-3.03130	3.98214
H	-1.60788	-1.57117	4.55259
H	-0.86269	-2.60256	3.28935
C	-3.84776	-0.76801	3.09529
H	-4.35183	-0.09870	2.35886
H	-3.70529	-0.17797	4.02532
H	-4.56541	-1.58698	3.31395
C	3.63488	1.06027	-0.00036
C	4.31353	1.33057	1.23819
C	3.62424	1.12313	2.59001
H	2.63288	0.66568	2.38720
C	4.39752	0.15976	3.51561
H	4.56825	-0.82482	3.03320
H	3.83756	-0.01623	4.45904
H	5.39199	0.56919	3.79408
C	3.37380	2.46956	3.30395
H	4.33120	2.97195	3.56022
H	2.80830	2.32243	4.24944
H	2.79671	3.16613	2.66252
C	5.63424	1.81652	1.20558
H	6.15292	2.01269	2.15911
C	6.30680	2.05728	-0.00143
H	7.34146	2.43461	-0.00184
C	5.63362	1.81559	-1.20790
H	6.15181	2.01103	-2.16185
C	4.31288	1.32961	-1.23948
C	3.62293	1.12129	-2.59083
H	2.63164	0.66402	-2.38728
C	4.39574	0.15729	-3.51617
H	5.39007	0.56651	-3.79539
H	3.83530	-0.01931	-4.45921
H	4.56667	-0.82698	-3.03320
C	3.37221	2.46726	-3.30555
H	2.79545	3.16429	-2.66431
H	2.80626	2.31952	-4.25068
H	4.32951	2.96944	-3.56260
C	-6.02085	1.53037	0.00006
H	-6.17998	2.19429	0.88379
H	-6.18015	2.19514	-0.88298
C	-7.07999	0.40884	-0.00036
H	-6.93828	-0.25714	0.88342
H	-6.93861	-0.25612	-0.88495
C	-8.53735	0.91143	0.00020
H	-8.69038	1.56930	-0.88614
H	-8.69006	1.56829	0.88735
C	-9.58302	-0.21124	-0.00025
H	-9.47811	-0.86344	0.89370
H	-10.61859	0.18889	0.00015
H	-9.47841	-0.86244	-0.89497
H	-2.72390	2.52673	0.92177
H	-1.83837	-0.46208	2.36449

J

SCF (BP86) Energy = -2528.092114
Enthalpy 0K = -2526.885456
Enthalpy 298K = -2526.820695
Free Energy 298K = -2527.000011
Lowest Frequency = 2.0228 cm^{-1}
Second Frequency = 3.3921 cm^{-1}
SCF (BP86-D3^{BJ}) Energy = -2528.431927
SCF (C_6H_6) Energy = -2528.141678
SCF (BS2) Energy = -2530.644163

Mg	2.72342	-2.05383	-0.71651
N	1.25922	-0.70366	-0.49319
N	-3.21472	-0.18990	0.14632
C	-0.10427	-0.81477	-0.30178
C	-0.64089	-2.26653	-0.27591
C	-1.33188	-2.56418	1.08952
H	-0.59032	-2.44019	1.91115
H	-2.13370	-1.81383	1.23718
C	0.46379	-3.34692	-0.46067
H	0.96813	-3.21631	-1.45410
C	-1.66899	-2.47905	-1.42889
H	-2.47548	-1.72845	-1.31027
H	-1.16813	-2.29313	-2.40607
C	-1.92563	-3.99078	1.11184
H	-2.42679	-4.15541	2.09089
C	-0.79949	-5.02927	0.92729
H	-0.05959	-4.95022	1.75541
H	-1.20823	-6.06330	0.96077
C	-0.10926	-4.78548	-0.43183
H	0.72855	-5.50434	-0.56749
C	-1.13330	-4.94504	-1.57586
H	-1.54576	-5.97810	-1.56955
H	-0.63560	-4.80487	-2.56192
C	-2.25945	-3.90659	-1.38990
H	-3.00097	-4.01053	-2.21223
C	-2.95752	-4.13853	-0.02998
H	-3.41438	-5.15295	-0.00312
H	-3.78227	-3.40657	0.10454
C	-0.87181	0.35247	-0.15344
H	-0.23062	1.24317	-0.21030
C	-2.27280	0.71099	0.05216
C	-2.48845	2.27010	0.14061
C	-1.66076	2.85657	1.33002
H	-2.00972	2.39508	2.28055
H	-0.58854	2.59064	1.22270
C	-3.96607	2.69452	0.35776
H	-4.36160	2.24038	1.28748
H	-4.59957	2.30751	-0.46455
C	-2.00254	2.95224	-1.17941
H	-0.94327	2.69056	-1.38284
H	-2.59765	2.55960	-2.03378
C	-1.79859	4.39515	1.40247
H	-1.18579	4.77052	2.25229
C	-3.27859	4.77554	1.61720
H	-3.38718	5.88074	1.69352
H	-3.64886	4.34922	2.57610
C	-4.11166	4.23338	0.43660
H	-5.18546	4.47787	0.59230
C	-3.61878	4.87104	-0.87950
H	-3.73234	5.97771	-0.84092
H	-4.23621	4.51423	-1.73378
C	-2.13930	4.49054	-1.09714
H	-1.77189	4.93455	-2.04920
C	-1.29081	5.01881	0.08221
H	-0.21856	4.76542	-0.07371
H	-1.35555	6.12867	0.13338
C	1.89268	0.58281	-0.56719
C	2.05802	1.21981	-1.83758
C	1.47857	0.58941	-3.10626
H	0.65511	-0.08024	-2.78180
C	0.87670	1.61625	-4.08415
H	0.12404	2.25799	-3.58323
H	0.37355	1.09658	-4.92622
H	1.64993	2.27879	-4.52803
C	2.53696	-0.27973	-3.82239

H	3.39541	0.33492	-4.16739
H	2.10534	-0.79408	-4.70705
H	2.96136	-1.06830	-3.15863
C	2.80326	2.41490	-1.90329
H	2.94502	2.91190	-2.87601
C	3.36750	2.98518	-0.75226
H	3.94336	3.92155	-0.82523
C	3.19073	2.36219	0.49196
H	3.63320	2.81815	1.39184
C	2.45420	1.16525	0.61187
C	2.29831	0.47682	1.96973
C	2.02127	1.45449	3.12756
H	2.88882	2.11574	3.33699
H	1.80754	0.89453	4.06195
H	1.14567	2.09895	2.91025
C	3.53203	-0.39749	2.28806
H	3.74104	-1.14972	1.49118
H	3.39762	-0.95782	3.23736
H	4.45092	0.21949	2.37923
C	-4.59512	-0.16896	0.33508
C	-5.14779	-0.29762	1.65639
C	-4.25368	-0.32414	2.89960
H	-3.21309	-0.13587	2.56066
C	-4.61410	0.77667	3.92004
H	-4.58279	1.78710	3.46257
H	-3.90603	0.76877	4.77631
H	-5.63452	0.63227	4.33478
C	-4.27528	-1.71192	3.57689
H	-5.28839	-1.95323	3.96424
H	-3.57000	-1.74970	4.43511
H	-3.99436	-2.51424	2.86489
C	-6.54175	-0.42150	1.80848
H	-6.96046	-0.51035	2.82513
C	-7.41030	-0.43482	0.70738
H	-8.49802	-0.52955	0.85156
C	-6.86627	-0.33235	-0.58125
H	-7.54140	-0.35050	-1.45341
C	-5.48095	-0.20634	-0.79708
C	-4.94979	-0.13351	-2.23166
H	-3.85537	0.04381	-2.16888
C	-5.56144	1.03186	-3.03828
H	-6.65620	0.90384	-3.17695
H	-5.10607	1.09398	-4.04990
H	-5.40361	2.00762	-2.53406
C	-5.15915	-1.47041	-2.97608
H	-4.70274	-2.31692	-2.42401
H	-4.70867	-1.43804	-3.99167
H	-6.24024	-1.69771	-3.09547
C	4.53901	-3.06124	-1.14762
H	4.28715	-3.86838	-1.87709
H	5.19974	-2.36185	-1.70891
C	5.30434	-3.66588	0.04604
H	5.72865	-2.85267	0.67997
H	6.19087	-4.24679	-0.30876
C	4.46098	-4.59081	0.93857
H	3.99862	-5.38439	0.30679
H	3.60179	-4.00766	1.35709
C	5.23803	-5.23827	2.09159
H	5.68572	-4.47093	2.75895
H	4.58854	-5.88716	2.71535
H	6.07013	-5.86778	1.71026
H	1.21198	-3.27755	0.37226
H	1.41923	-0.19589	1.88659
C	8.68424	3.04522	0.92762
C	10.03918	2.90927	1.28045
C	8.09898	2.14339	0.02022
H	10.49730	3.61618	1.99088
H	7.03759	2.24924	-0.25625
C	10.80950	1.87108	0.72593
C	8.86988	1.10538	-0.53427
H	11.87094	1.76458	1.00209
H	8.41202	0.39916	-1.24546
C	10.22477	0.96914	-0.18148
H	10.82822	0.15599	-0.61612
H	8.08156	3.85922	1.36190

TS(J-K)

SCF (BP86) Energy = -2528.040238
Enthalpy 0K = -2526.836792
Enthalpy 298K = -2526.773448
Free Energy 298K = -2526.938513
Lowest Frequency = -1332.6096 cm^{-1}
Second Frequency = 8.7090 cm^{-1}
SCF (BP86-D3^{BJ}) Energy = -2528.398751
SCF (C_6H_6) Energy = -2528.086431
SCF (BS2) Energy = -2530.591294

Mg	-3.52757	0.06845	0.39014
N	-1.62958	0.67230	0.17431
N	2.59364	-0.99829	-0.09875
C	-0.46859	-0.06975	0.10007
C	-0.67882	-1.59591	-0.07121
C	-0.08120	-2.05193	-1.43819
H	-0.59888	-1.51179	-2.26278
H	0.98821	-1.76206	-1.45531
C	-2.17346	-2.03732	-0.07083
H	-2.63867	-1.80861	0.92468
C	0.01266	-2.38282	1.08195
H	1.08328	-2.09830	1.09553
H	-0.43580	-2.07864	2.05492
C	-0.22031	-3.57908	-1.62572
H	0.23838	-3.86385	-2.59796
C	-1.71250	-3.97093	-1.62495
H	-2.24545	-3.46779	-2.46297
H	-1.83255	-5.06592	-1.77965
C	-2.33572	-3.56570	-0.27255
H	-3.42056	-3.81119	-0.26304
C	-1.61933	-4.30414	0.87821
H	-1.73502	-5.40287	0.74888
H	-2.08893	-4.04518	1.85391
C	-0.12823	-3.90824	0.87622
H	0.39604	-4.43171	1.70552
C	0.50909	-4.30879	-0.47435
H	0.44189	-5.41050	-0.61664
H	1.58815	-4.04507	-0.47962
C	0.77611	0.58403	0.16316
H	0.63436	1.66547	0.30174
C	2.19164	0.23271	0.08161
C	3.13907	1.48534	0.22692
C	2.82377	2.52774	-0.89400
H	2.99772	2.05895	-1.88811
H	1.75331	2.81853	-0.86173
C	4.65088	1.14739	0.12428
H	4.86826	0.65951	-0.84625
H	4.93288	0.41353	0.90442
C	2.91288	2.17358	1.61154
H	1.84570	2.44906	1.73699
H	3.15185	1.45098	2.42331

C	3.69655	3.79546	-0.74290
H	3.43025	4.51201	-1.55170
C	5.18861	3.41735	-0.84925
H	5.82573	4.32610	-0.76329
H	5.40063	2.96901	-1.84546
C	5.53060	2.41211	0.27049
H	6.59789	2.10964	0.18957
C	5.27798	3.06446	1.64616
H	5.91632	3.96788	1.77062
H	5.55551	2.35999	2.46167
C	3.78584	3.44240	1.75477
H	3.58541	3.90485	2.74704
C	3.42438	4.44372	0.63384
H	2.35437	4.73849	0.71318
H	4.02410	5.37476	0.74409
C	-1.60930	2.10678	0.18029
C	-1.88022	2.81368	1.39636
C	-2.04805	2.07673	2.73026
H	-2.43970	1.05788	2.50208
C	-0.68911	1.85466	3.43116
H	0.01151	1.29032	2.78466
H	-0.82181	1.28750	4.37710
H	-0.21738	2.82873	3.68197
C	-3.05274	2.74690	3.68603
H	-2.66237	3.70408	4.09182
H	-3.25410	2.08762	4.55559
H	-4.02123	2.96321	3.18894
C	-1.95785	4.22350	1.35861
H	-2.16220	4.77896	2.28679
C	-1.77342	4.93093	0.16477
H	-1.83368	6.03090	0.15804
C	-1.51924	4.23136	-1.02505
H	-1.38763	4.79493	-1.96152
C	-1.43811	2.82567	-1.04925
C	-1.22627	2.08303	-2.37187
C	-0.35421	2.84909	-3.38402
H	-0.86158	3.75726	-3.77413
H	-0.13378	2.20411	-4.26001
H	0.61322	3.16084	-2.94138
C	-2.57996	1.70769	-3.01487
H	-3.21489	1.10484	-2.33269
H	-2.42740	1.11430	-3.94135
H	-3.15946	2.61629	-3.28351
C	3.82480	-1.63963	-0.21277
C	4.38677	-1.90078	-1.51078
C	3.73136	-1.36780	-2.78825
H	2.87721	-0.72970	-2.47764
C	4.68348	-0.48953	-3.62807
H	5.08736	0.35895	-3.03801
H	4.15707	-0.06916	-4.51170
H	5.55036	-1.07200	-4.00682
C	3.16146	-2.51631	-3.64912
H	3.97156	-3.18189	-4.01708
H	2.62233	-2.12152	-4.53730
H	2.45560	-3.14496	-3.06933
C	5.55594	-2.67832	-1.60989
H	5.98678	-2.86678	-2.60771
C	6.18660	-3.21779	-0.47924
H	7.10260	-3.82066	-0.58130
C	5.62405	-2.98027	0.78335
H	6.10888	-3.40756	1.67733
C	4.45655	-2.21086	0.94642
C	3.87772	-2.01852	2.35135
H	3.01430	-1.32614	2.25998
C	4.88361	-1.37678	3.33089
H	5.76254	-2.03386	3.50372
H	4.41007	-1.19259	4.31903
H	5.26536	-0.40665	2.95106
C	3.34416	-3.34887	2.92637
H	2.60143	-3.81444	2.24732
H	2.85828	-3.19205	3.91364
H	4.16652	-4.08222	3.06985
C	-5.00563	-0.33482	2.13341
H	-4.12836	-0.77132	2.68027
H	-5.19368	0.64861	2.62186
C	-6.21150	-1.27351	2.34484
H	-7.10006	-0.86401	1.80872
H	-6.50657	-1.30747	3.41996
C	-5.96426	-2.71106	1.86034
H	-5.07714	-3.12604	2.39316
H	-5.68377	-2.69033	0.78085
C	-7.16362	-3.64688	2.05559
H	-8.05456	-3.28212	1.50111
H	-6.94615	-4.67560	1.70023
H	-7.44849	-3.71763	3.12714
H	-2.72526	-1.53290	-0.90763
H	-0.69341	1.14029	-2.13027
C	-6.73287	2.05603	-1.93264
C	-7.12090	1.10751	-2.89701
C	-5.93889	1.65508	-0.84366
H	-7.74844	1.41387	-3.74963
H	-5.66306	2.41024	-0.08331
C	-6.71771	-0.23519	-2.76764
C	-5.49753	0.31381	-0.69616
H	-7.03123	-0.97823	-3.51936
H	-5.28003	-0.07296	0.66634
C	-5.91990	-0.62201	-1.67649
H	-5.62342	-1.68392	-1.59028
H	-7.05688	3.10516	-2.02990

K

SCF (BP86) Energy = -2528.097797
Enthalpy 0K = -2526.890695
Enthalpy 298K = -2526.825533
Free Energy 298K = -2527.005905
Lowest Frequency = 2.9331 cm^{-1}
Second Frequency = 3.3508 cm^{-1}
SCF (BP86-D3^{BJ}) Energy = -2528.436461
SCF (C_6H_6) Energy = -2528.148396
SCF (BS2) Energy = -2530.649901

Mg	3.05551	0.80059	0.99458
N	1.14778	1.02793	0.47818
N	-2.79662	-1.18628	0.03637
C	0.08829	0.14386	0.42529
C	0.43232	-1.30472	0.85162
C	-0.42931	-1.72016	2.08294
H	-0.21276	-1.03235	2.93167
H	-1.49593	-1.59843	1.80829
C	1.92118	-1.50596	1.26362
H	2.59547	-1.27383	0.39658
C	0.15910	-2.29208	-0.32369
H	-0.90087	-2.17702	-0.62559
H	0.79588	-2.01296	-1.19373
C	-0.14736	-3.18371	2.49075
H	-0.79574	-3.44616	3.35527
C	1.33445	-3.33934	2.89117
H	1.57189	-2.68697	3.76156

H	1.54972	-4.38412	3.20616
C	2.22221	-2.96676	1.68448
H	3.29597	-3.04211	1.96479
C	1.91921	-3.90807	0.49913
H	2.14040	-4.95882	0.78919
H	2.58061	-3.66803	-0.36360
C	0.43697	-3.75181	0.10060
H	0.20950	-4.42348	-0.75600
C	-0.46352	-4.12046	1.30210
H	-0.29413	-5.18049	1.59541
H	-1.53368	-4.02840	1.01876
C	-1.16918	0.60799	0.00744
H	-1.12190	1.67925	-0.23384
C	-2.51183	0.06810	-0.19339
C	-3.52963	1.15776	-0.70359
C	-3.62788	2.32477	0.33210
H	-3.99587	1.92214	1.30199
H	-2.62334	2.75465	0.52712
C	-4.96973	0.62309	-0.92879
H	-5.36971	0.19418	0.01102
H	-4.95902	-0.20670	-1.66259
C	-3.03995	1.74937	-2.06540
H	-2.01262	2.15700	-1.96301
H	-2.98475	0.93288	-2.81936
C	-4.57012	3.44226	-0.17282
H	-4.60076	4.25541	0.58639
C	-5.98797	2.87169	-0.38512
H	-6.68051	3.67318	-0.72725
H	-6.39378	2.48518	0.57628
C	-5.92038	1.73719	-1.42914
H	-6.93239	1.29846	-1.57186
C	-5.40338	2.29917	-2.77023
H	-6.08716	3.09199	-3.14827
H	-5.38432	1.49676	-3.54102
C	-3.98477	2.86908	-2.56071
H	-3.59325	3.26891	-3.52270
C	-4.03278	4.00202	-1.50999
H	-3.01731	4.43310	-1.36454
H	-4.68546	4.82909	-1.86844
C	0.98860	2.41013	0.12190
C	1.25805	2.83180	-1.21797
C	1.61473	1.80926	-2.29948
H	1.22171	0.83011	-1.95463
C	0.96729	2.10827	-3.66504
H	-0.13224	2.21856	-3.57497
H	1.16870	1.28014	-4.37632
H	1.36722	3.03628	-4.12611
C	3.14736	1.67628	-2.44845
H	3.60227	2.63026	-2.78992
H	3.41524	0.88673	-3.18200
H	3.65446	1.41561	-1.48987
C	1.23514	4.21129	-1.51022
H	1.44853	4.55107	-2.53609
C	0.94581	5.15997	-0.51855
H	0.93416	6.23311	-0.76719
C	0.67047	4.73824	0.79047
H	0.44278	5.48996	1.56282
C	0.67983	3.37091	1.13548
C	0.41640	2.92671	2.57631
C	-0.73633	3.69215	3.25361
H	-0.49249	4.76373	3.41488
H	-0.95470	3.25589	4.25077
H	-1.66480	3.64058	2.64986
C	1.70230	3.02612	3.42809
H	2.55258	2.44686	2.99766
H	1.53604	2.64214	4.45679
H	2.05177	4.07745	3.50700
C	-3.94030	-1.97788	-0.05381
C	-4.77159	-2.18270	1.10137
C	-4.51162	-1.44222	2.41658
H	-3.68700	-0.72186	2.23154
C	-5.73358	-0.63195	2.89951
H	-6.07784	0.08994	2.13035
H	-5.48881	-0.05831	3.81907
H	-6.59307	-1.29263	3.14212
C	-4.03877	-2.41149	3.52212
H	-4.83113	-3.14807	3.77554
H	-3.78124	-1.86187	4.45322
H	-3.14508	-2.98500	3.20258
C	-5.83679	-3.09998	1.02656
H	-6.47704	-3.24555	1.91293
C	-6.10588	-3.83126	-0.13956
H	-6.94719	-4.54104	-0.17533
C	-5.27839	-3.64678	-1.25682
H	-5.47726	-4.22462	-2.17512
C	-4.19827	-2.74417	-1.24280
C	-3.31231	-2.61650	-2.48555
H	-2.58204	-1.80375	-2.28720
C	-4.10673	-2.22598	-3.75016
H	-4.83655	-3.01365	-4.03488
H	-3.42510	-2.07985	-4.61538
H	-4.67600	-1.28489	-3.60343
C	-2.50678	-3.91034	-2.73525
H	-1.90904	-4.19294	-1.84502
H	-1.81126	-3.78970	-3.59376
H	-3.18002	-4.76364	-2.96605
C	7.44798	-2.85835	-2.25937
H	7.65711	-3.84264	-1.78738
H	6.56781	-2.98879	-2.92324
C	8.66778	-2.33838	-3.03021
H	8.41357	-1.37153	-3.52242
H	8.91239	-3.04009	-3.86067
C	9.91500	-2.14221	-2.15316
H	10.16942	-3.10911	-1.66087
H	9.66908	-1.44069	-1.32340
C	11.13466	-1.62131	-2.92335
H	10.92578	-0.63681	-3.39473
H	12.01543	-1.49226	-2.26032
H	11.42929	-2.31922	-3.73655
H	2.16679	-0.85812	2.14687
H	0.12150	1.85759	2.53159
C	7.13478	2.39842	1.62768
C	7.87039	1.28836	2.07890
C	5.76818	2.25925	1.31909
H	8.94026	1.39306	2.32245
H	5.22620	3.15633	0.96822
C	7.23138	0.04392	2.21829
C	5.08953	1.01761	1.44771
H	7.80021	-0.83174	2.57291
H	7.15511	-2.15883	-1.44713
C	5.86380	-0.08201	1.90596
H	5.39935	-1.07787	2.03010
H	7.62868	3.37811	1.51603

L

SCF (BP86) Energy = -2369.768812
Enthalpy 0K = -2368.689594
Enthalpy 298K = -2368.632992

Free Energy 298K = -2368.782828
Lowest Frequency = 9.2566 cm^{-1}
Second Frequency = 13.1972 cm^{-1}
SCF (BP86-D3^{BJ}) Energy = -2370.093261
SCF (C_6H_6) Energy = -2369.815498
SCF (BS2) Energy = -2372.143672

Mg	3.93683	-0.27767	-0.00001
N	2.06627	0.39908	-0.00011
N	-2.28746	-0.90689	0.00006
C	0.84449	-0.24420	-0.00003
C	0.92747	-1.79068	-0.00003
C	0.23171	-2.36628	1.27096
H	0.74552	-1.97730	2.17928
H	-0.81251	-1.99570	1.28415
C	2.38318	-2.34573	-0.00004
H	2.92353	-2.00909	-0.92463
C	0.23169	-2.36630	-1.27099
H	-0.81253	-1.99571	-1.28417
H	0.74547	-1.97734	-2.17932
C	0.24889	-3.91146	1.26256
H	-0.27408	-4.28071	2.17186
C	1.70592	-4.41883	1.26372
H	2.23659	-4.07292	2.17929
H	1.73569	-5.53062	1.27743
C	2.42122	-3.89497	-0.00003
H	3.48347	-4.22488	-0.00004
C	1.70588	-4.41885	-1.26375
H	1.73565	-5.53064	-1.27744
H	2.23653	-4.07297	-2.17934
C	0.24886	-3.91148	-1.26256
H	-0.27413	-4.28074	-2.17184
C	-0.47826	-4.42868	0.00001
H	-0.49947	-5.54131	0.00002
H	-1.53395	-4.08299	0.00002
C	-0.33164	0.52267	0.00007
H	-0.08971	1.59484	0.00009
C	-1.77471	0.29500	0.00008
C	-2.59718	1.63946	0.00009
C	-2.24168	2.48292	1.26742
H	-2.50868	1.90298	2.17883
H	-1.14796	2.66568	1.31605
C	-4.13675	1.44036	0.00009
H	-4.44570	0.84966	0.88483
H	-4.44571	0.84966	-0.88464
C	-2.24169	2.48291	-1.26725
H	-1.14796	2.66567	-1.31587
H	-2.50868	1.90297	-2.17866
C	-2.98416	3.83966	1.26255
H	-2.69321	4.41074	2.17235
C	-4.50831	3.59934	1.26103
H	-5.05345	4.56964	1.28011
H	-4.80988	3.04320	2.17649
C	-4.88687	2.79413	0.00009
H	-5.98028	2.59039	0.00009
C	-4.50832	3.59933	-1.26086
H	-5.05346	4.56963	-1.27994
H	-4.80988	3.04318	-2.17632
C	-2.98417	3.83966	-1.26239
H	-2.69322	4.41073	-2.17219
C	-2.58566	4.63811	0.00008
H	-1.49016	4.83318	0.00008
H	-3.08938	5.63049	0.00009
C	2.15866	1.83235	-0.00016
C	2.26569	2.53466	-1.24155
C	2.17937	1.78318	-2.57212
H	1.65104	0.82905	-2.36593
C	1.37067	2.53190	-3.64865
H	0.35912	2.79833	-3.28116
H	1.24814	1.89580	-4.55014
H	1.87224	3.46689	-3.97724
C	3.58930	1.43640	-3.10204
H	4.17216	2.35527	-3.32474
H	3.53392	0.83189	-4.03199
H	4.19564	0.85313	-2.36990
C	2.50070	3.92492	-1.21368
H	2.59365	4.47749	-2.16208
C	2.61899	4.61832	-0.00026
H	2.80370	5.70435	-0.00031
C	2.50087	3.92499	1.21321
H	2.59397	4.47760	2.16157
C	2.26584	2.53473	1.24119
C	2.17965	1.78336	2.57183
C	1.37090	2.53213	3.64829
H	1.87236	3.46723	3.97675
H	1.24845	1.89614	4.54986
H	0.35931	2.79840	3.28078
C	3.58962	1.43674	3.10172
H	4.19594	0.85341	2.36962
H	3.53431	0.83235	4.03176
H	4.17244	2.35567	3.32429
C	-3.57675	-1.43651	0.00004
C	-4.21055	-1.79837	1.23890
C	-3.55850	-1.49444	2.59099
H	-2.64092	-0.90260	2.38865
C	-4.46059	-0.64753	3.51400
H	-4.76818	0.30198	3.02912
H	-3.93170	-0.39214	4.45720
H	-5.38753	-1.19250	3.79304
C	-3.12289	-2.79113	3.30798
H	-4.00092	-3.42193	3.56438
H	-2.58488	-2.56434	4.25374
H	-2.45324	-3.40173	2.66890
C	-5.45008	-2.46487	1.20674
H	-5.93613	-2.73126	2.16043
C	-6.08226	-2.79827	0.00003
H	-7.05374	-3.31702	0.00003
C	-5.45008	-2.46484	-1.20667
H	-5.93613	-2.73121	-2.16037
C	-4.21055	-1.79834	-1.23882
C	-3.55850	-1.49437	-2.59090
H	-2.64092	-0.90254	-2.38854
C	-4.46059	-0.64742	-3.51388
H	-5.38754	-1.19237	-3.79293
H	-3.93170	-0.39201	-4.45708
H	-4.76817	0.30207	-3.02898
C	-3.12291	-2.79104	-3.30793
H	-2.45327	-3.40167	-2.66887
H	-2.58490	-2.56422	-4.25368
H	-4.00095	-3.42183	-3.56436
H	2.92354	-2.00909	0.92453
H	1.65137	0.82917	2.36577
C	8.31046	0.39417	0.00061
C	8.85648	-0.90151	0.00021
C	6.91354	0.56945	0.00057
H	9.94956	-1.04333	0.00023
H	6.52503	1.60419	0.00089
C	7.99844	-2.01499	-0.00022

C	6.01617	-0.53224	0.00013
H	8.41811	-3.03482	-0.00055
C	6.60299	-1.82608	-0.00026
H	5.96375	-2.72844	-0.00063
H	8.97685	1.27288	0.00094

M^{Ad}

SCF (BP86) Energy = -2370.998113
Enthalpy 0K = -2369.901249
Enthalpy 298K = -2369.843228
Free Energy 298K = -2370.002271
Lowest Frequency = 2.6582 cm^{-1}
Second Frequency = 3.9123 cm^{-1}
SCF (BP86-D3^{BJ}) Energy = -2371.317703
SCF (C_6H_6) Energy = -2371.043091
SCF (BS2) Energy = -2373.375682

N	0.41260	-1.38022	0.02417
C	-0.92597	-1.54013	0.01165
C	1.44877	-2.36475	-0.07716
C	1.88505	-2.79983	-1.36923
C	2.99807	-3.65888	-1.45481
H	3.33665	-4.00341	-2.44497
C	3.68709	-4.08196	-0.30877
H	4.55291	-4.75690	-0.39845
C	3.27186	-3.62659	0.94911
H	3.82308	-3.94670	1.84835
C	2.16586	-2.76375	1.09272
C	1.20221	-2.30897	-2.64919
H	0.20865	-1.90534	-2.36260
C	2.00383	-1.14792	-3.27895
H	2.16186	-0.31207	-2.56353
H	1.48300	-0.73798	-4.17023
H	3.01261	-1.48725	-3.59619
C	0.96095	-3.42541	-3.68439
H	1.91145	-3.82624	-4.09573
H	0.37485	-3.03657	-4.54353
H	0.39980	-4.27719	-3.24709
C	1.78156	-2.26623	2.48932
H	0.85858	-1.65683	2.37703
C	1.45273	-3.41883	3.46176
H	0.64510	-4.07162	3.07078
H	1.12129	-3.01958	4.44367
H	2.33953	-4.06086	3.64898
C	2.88150	-1.35407	3.07722
H	3.82291	-1.91916	3.24537
H	2.56550	-0.93143	4.05444
H	3.12629	-0.51187	2.39555
H	2.96454	0.94350	0.11905
Mg	1.30940	0.47653	0.07494
N	-0.43805	1.57156	0.05312
C	-1.65529	0.99547	-0.01146
C	-1.81134	-0.42222	-0.01583
H	-2.86134	-0.72462	-0.04842
C	-0.09179	2.95459	0.19835
C	0.36030	3.69700	-0.93598
C	0.82853	5.01289	-0.74282
H	1.16994	5.59429	-1.61482
C	0.87304	5.59505	0.53033
H	1.24203	6.62486	0.65892
C	0.45766	4.84833	1.64229
H	0.51037	5.30092	2.64540
C	-0.02060	3.53028	1.50687
C	0.37057	3.09849	-2.34568
H	-0.10063	2.09395	-2.27798
C	1.81591	2.90912	-2.85828
H	2.42772	2.30656	-2.15358
H	1.82329	2.40325	-3.84694
H	2.32888	3.88711	-2.97819
C	-0.45903	3.92600	-3.35024
H	-0.03047	4.94013	-3.49704
H	-0.47880	3.42957	-4.34347
H	-1.50861	4.05324	-3.01345
C	-0.39905	2.72668	2.75440
H	-0.99799	1.85418	2.41955
C	-1.26363	3.51760	3.75615
H	-0.70671	4.36379	4.21124
H	-2.17229	3.93512	3.27484
H	-1.59096	2.86000	4.58878
C	0.86741	2.17477	3.44636
H	0.60338	1.53277	4.31344
H	1.49262	1.57063	2.75383
H	1.51153	3.00128	3.81450
C	7.00011	2.51686	0.12327
C	6.33405	1.28527	-0.01694
C	8.40362	2.57541	0.04419
H	5.23327	1.23696	0.04418
H	8.92291	3.54144	0.15441
C	7.07815	0.11124	-0.23667
C	9.14523	1.40028	-0.17564
H	6.55626	-0.85316	-0.34601
H	10.24477	1.44534	-0.23772
C	8.48169	0.16765	-0.31605
H	9.06241	-0.75334	-0.48822
H	6.41877	3.43720	0.29555
C	-3.02383	1.76758	-0.12799
C	-2.93881	3.32065	-0.11586
H	-2.48149	3.67564	0.82790
H	-2.27983	3.67554	-0.93183
C	-3.97835	1.36127	1.04415
H	-4.09175	0.25874	1.09336
H	-3.52330	1.67265	2.01040
C	-3.70293	1.36439	-1.48180
H	-3.04639	1.67730	-2.32378
H	-3.79736	0.26125	-1.55289
C	-4.33453	3.97393	-0.27205
H	-4.20338	5.07816	-0.25668
C	-5.24500	3.54211	0.89580
H	-6.24711	4.01716	0.80327
H	-4.81611	3.88266	1.86444
C	-5.37513	2.00513	0.88260
H	-6.01929	1.67349	1.72700
C	-6.00051	1.55152	-0.45629
H	-7.02074	1.98133	-0.56699
H	-6.11683	0.44462	-0.47031
C	-5.10071	2.00756	-1.62750
H	-5.54608	1.67675	-2.59197
C	-4.96936	3.54421	-1.61033
H	-4.33972	3.88474	-2.46227
H	-5.96686	4.02098	-1.73749
C	-1.67910	-2.92315	0.06019
C	-2.55565	-2.96500	1.35895
H	-1.89017	-2.89425	2.24778
H	-3.22693	-2.08272	1.40074
C	-0.78080	-4.19275	0.08714
H	-0.14455	-4.23498	-0.81778
H	-0.08854	-4.15522	0.95069
C	-2.62471	-3.06789	-1.17874

H	-3.30349	-2.19377	-1.25745
H	-2.01191	-3.07332	-2.10723
C	-3.40351	-4.25528	1.42820
H	-4.01898	-4.22736	2.35471
C	-2.47468	-5.48630	1.45278
H	-3.07196	-6.42247	1.52567
H	-1.81808	-5.45346	2.35057
C	-1.62212	-5.49066	0.16759
H	-0.92318	-6.35553	0.18419
C	-2.54337	-5.59052	-1.06559
H	-3.14287	-6.52731	-1.02772
H	-1.93621	-5.63490	-1.99702
C	-3.47179	-4.35889	-1.09381
H	-4.13650	-4.40737	-1.98473
C	-4.32886	-4.32937	0.19225
H	-5.01668	-3.45439	0.17515
H	-4.96889	-5.23780	0.24665

TS (M^{Ad}–N^{Ad})

SCF (BP86) Energy = -2370.946386
Enthalpy 0K = -2369.852302
Enthalpy 298K = -2369.795840
Free Energy 298K = -2369.942476
Lowest Frequency = -1089.9204 cm^{-1}
Second Frequency = 6.9050 cm^{-1}
SCF (BP86-D3^{BJ}) Energy = -2371.287181
SCF (C_6H_6) Energy = -2370.984264
SCF (BS2) Energy = -2373.323422

N	1.57286	0.36423	0.25119
C	1.33105	-0.95706	0.12569
C	2.81231	1.07490	0.13119
C	3.29516	1.45367	-1.16367
C	4.45809	2.24460	-1.25072
H	4.83705	2.53201	-2.24492
C	5.14063	2.67635	-0.10538
H	6.05221	3.28791	-0.19696
C	4.63983	2.33540	1.15747
H	5.16547	2.68908	2.05903
C	3.47623	1.55173	1.30822
C	2.57017	1.06426	-2.45630
H	1.77472	0.33774	-2.18942
C	1.88347	2.29475	-3.08832
H	1.19052	2.79115	-2.37923
H	1.30602	2.00500	-3.99152
H	2.63331	3.05400	-3.39703
C	3.49497	0.38302	-3.48731
H	4.27627	1.07770	-3.86220
H	2.90999	0.04400	-4.36820
H	4.01226	-0.50189	-3.06307
C	2.98330	1.23577	2.72599
H	1.97928	0.76776	2.63181
C	3.89957	0.22692	3.45419
H	3.98227	-0.73448	2.90863
H	3.50664	0.00533	4.46905
H	4.92718	0.63245	3.57346
C	2.82237	2.50890	3.58336
H	3.80260	2.98538	3.79858
H	2.35568	2.25876	4.55912
H	2.17599	3.25900	3.08662
H	-0.00480	2.46687	2.37421
Mg	-0.00243	1.62994	0.64151
N	-1.57135	0.35705	0.24916
C	-1.32349	-0.96295	0.12275
C	0.00508	-1.48711	0.12467
H	0.00764	-2.57796	0.06821
C	-2.81627	1.06101	0.14294
C	-3.30152	1.46264	-1.14401
C	-4.47401	2.24119	-1.21546
H	-4.85580	2.54447	-2.20394
C	-5.16185	2.64169	-0.06217
H	-6.08064	3.24408	-0.14177
C	-4.65629	2.28263	1.19383
H	-5.18504	2.61364	2.10221
C	-3.48433	1.50886	1.32925
C	-2.57304	1.11028	-2.44552
H	-1.74335	0.41897	-2.18894
C	-1.95041	2.37177	-3.08321
H	-1.27646	2.90118	-2.37981
H	-1.36734	2.10858	-3.99083
H	-2.73761	3.09399	-3.38778
C	-3.47902	0.39171	-3.46832
H	-4.29947	1.05067	-3.82371
H	-2.89229	0.08903	-4.36125
H	-3.94563	-0.52097	-3.04451
C	-2.98663	1.17404	2.74116
H	-1.98360	0.70606	2.63747
C	-3.90187	0.15829	3.46105
H	-4.92747	0.56522	3.59208
H	-3.99108	-0.79552	2.90334
H	-3.50376	-0.07794	4.47056
C	-2.82157	2.43695	3.61302
H	-2.35790	2.17398	4.58690
H	-2.17039	3.18906	3.12573
H	-3.80024	2.91524	3.83133
C	-1.22283	4.56833	-0.00985
C	-0.00626	3.90776	0.30834
C	-1.23726	5.80737	-0.67601
H	-0.00186	3.17753	1.57503
H	-2.19570	6.29629	-0.91761
C	1.20195	4.56780	-0.04155
C	-0.02344	6.42656	-1.02843
H	2.17364	4.10682	0.21413
H	-0.03000	7.40040	-1.54517
C	1.19895	5.80687	-0.70786
H	2.15087	6.29550	-0.97460
H	-2.18735	4.10752	0.27225
C	-2.41874	-2.09250	-0.00695
C	-3.90032	-1.61935	-0.04631
H	-4.14177	-1.02788	0.85698
H	-4.06697	-0.94636	-0.90824
C	-2.28807	-3.05903	1.22070
H	-1.25040	-3.44212	1.30523
H	-2.48632	-2.48936	2.15506
C	-2.17989	-2.92124	-1.31353
H	-2.30113	-2.25489	-2.19553
H	-1.13798	-3.29959	-1.35080
C	-4.88371	-2.81218	-0.14814
H	-5.91856	-2.40546	-0.16981
C	-4.71503	-3.74000	1.07220
H	-5.42768	-4.59276	1.01285
H	-4.95008	-3.18995	2.01034
C	-3.26151	-4.25457	1.10695
H	-3.11562	-4.91622	1.98953
C	-2.96002	-5.04225	-0.18829
H	-3.63237	-5.92543	-0.26479
H	-1.91852	-5.43434	-0.16521
C	-3.15476	-4.11753	-1.41156

H	-2.93294	-4.68105	-2.34496
C	-4.60911	-3.60482	-1.44243
H	-4.76897	-2.95664	-2.33261
H	-5.31927	-4.45677	-1.53430
C	2.43248	-2.08159	0.00471
C	2.30215	-3.04064	1.23841
H	2.49368	-2.46345	2.16959
H	1.26606	-3.42837	1.32136
C	3.91165	-1.60069	-0.03203
H	4.07824	-0.93374	-0.89867
H	4.14595	-1.00073	0.86766
C	2.20359	-2.92011	-1.29727
H	1.16392	-3.30441	-1.33671
H	2.32550	-2.25914	-2.18326
C	3.28232	-4.23169	1.13666
H	3.13622	-4.88828	2.02298
C	4.73314	-3.70940	1.10470
H	5.45073	-4.55855	1.05407
H	4.96104	-3.15179	2.04015
C	4.90192	-2.78880	-0.12108
H	5.93465	-2.37663	-0.14115
C	4.63703	-3.59148	-1.41121
H	5.35204	-4.44025	-1.49442
H	4.79721	-2.94834	-2.30498
C	3.18529	-4.11172	-1.38311
H	2.97056	-4.68264	-2.31366
C	2.99037	-5.02941	-0.15459
H	1.95088	-5.42688	-0.13318
H	3.66769	-5.90949	-0.22254

N^{Ad}

SCF (BP86) Energy = -2370.982223
Enthalpy 0K = -2369.891469
Enthalpy 298K = -2369.830735
Free Energy 298K = -2369.998308
Lowest Frequency = 0.8186 cm^{-1}
Second Frequency = 3.8652 cm^{-1}
SCF (BP86-D3^{BJ}) Energy = -2371.318038
SCF (C_6H_6) Energy = -2371.021211
SCF (BS2) Energy = -2373.359637

N	1.53376	0.33322	-0.03502
C	1.31735	-0.99725	-0.05314
C	2.77261	1.05383	-0.06594
C	3.33031	1.44638	-1.32411
C	4.46772	2.27814	-1.33159
H	4.90576	2.58091	-2.29633
C	5.05162	2.73179	-0.14019
H	5.94140	3.38036	-0.16892
C	4.48371	2.36318	1.08659
H	4.93386	2.73234	2.02248
C	3.34454	1.53504	1.15315
C	2.69852	1.01778	-2.65185
H	1.96710	0.21384	-2.42502
C	1.91671	2.18839	-3.28846
H	1.15836	2.61121	-2.59516
H	1.39429	1.86316	-4.21306
H	2.59856	3.02270	-3.55803
C	3.72027	0.44019	-3.65237
H	4.45045	1.20639	-3.98874
H	3.20354	0.06263	-4.55981
H	4.29636	-0.40115	-3.21438
C	2.74337	1.19867	2.52124
H	1.91248	0.48180	2.34744
C	3.75442	0.51124	3.46333
H	4.18169	-0.40825	3.01259
H	3.26544	0.22549	4.41860
H	4.60160	1.18384	3.71549
C	2.14700	2.45675	3.19096
H	2.93970	3.19879	3.42525
H	1.64013	2.19517	4.14390
H	1.41204	2.96753	2.53366
H	0.03013	-2.84614	8.12030
Mg	0.00099	1.71821	-0.00934
N	-1.53416	0.33583	-0.03121
C	-1.31999	-0.99482	-0.05899
C	-0.00178	-1.53847	-0.06522
H	-0.00266	-2.63124	-0.08445
C	-2.77185	1.05692	0.02398
C	-3.34209	1.58109	-1.17805
C	-4.47947	2.40897	-1.08345
H	-4.92825	2.81134	-2.00623
C	-5.04734	2.73593	0.15510
H	-5.93571	3.38509	0.20585
C	-4.46542	2.23966	1.33038
H	-4.90368	2.50936	2.30476
C	-3.32971	1.40633	1.29473
C	-2.74125	1.29012	-2.55668
H	-1.91213	0.56572	-2.40715
C	-2.14175	2.56866	-3.18339
H	-1.40475	3.05450	-2.50974
H	-1.63637	2.33839	-4.14517
H	-2.93247	3.32057	-3.39146
C	-3.75374	0.63744	-3.52168
H	-4.59907	1.32035	-3.75134
H	-3.26513	0.38275	-4.48589
H	-4.18358	-0.29558	-3.10230
C	-2.69964	0.92987	2.60692
H	-1.97353	0.12925	2.35277
C	-3.72413	0.32598	3.58890
H	-4.44854	1.08539	3.95207
H	-4.30656	-0.49615	3.12367
H	-3.20884	-0.08544	4.48231
C	-1.90991	2.07365	3.28144
H	-1.38954	1.71472	4.19464
H	-1.14869	2.51425	2.60249
H	-2.58618	2.90320	3.57834
C	-1.19521	4.59509	0.15169
C	0.00389	3.84037	0.02598
C	-1.20042	6.00375	0.17748
H	-0.03257	-2.27090	8.62440
H	-2.15379	6.55017	0.27721
C	1.20551	4.59496	-0.07397
C	0.00872	6.71388	0.07528
H	2.17681	4.07687	-0.17187
H	0.01056	7.81645	0.09424
C	1.21546	6.00364	-0.05138
H	2.17066	6.54997	-0.13245
H	-2.16829	4.07723	0.23157
C	-2.44170	-2.10080	-0.10477
C	-3.91147	-1.59162	-0.08674
H	-4.10316	-1.00012	0.82924
H	-4.09353	-0.90959	-0.93938
C	-2.28665	-3.06224	1.12186
H	-1.25642	-3.47078	1.17060
H	-2.43820	-2.48337	2.05981
C	-2.27251	-2.94064	-1.41659
H	-2.41323	-2.27325	-2.29554

H	-1.24022	-3.34035	-1.49054
C	-4.92623	-2.75996	-0.15101
H	-5.95130	-2.32889	-0.13617
C	-4.73392	-3.68621	1.06727
H	-5.46782	-4.52231	1.03936
H	-4.92073	-3.12583	2.01027
C	-3.29238	-4.23468	1.05122
H	-3.12995	-4.89590	1.93121
C	-3.05901	-5.03573	-0.25003
H	-3.75460	-5.90288	-0.29559
H	-2.02708	-5.45274	-0.26427
C	-3.27790	-4.11320	-1.47094
H	-3.10522	-4.68695	-2.40848
C	-4.71897	-3.56377	-1.45087
H	-4.89433	-2.91456	-2.33742
H	-5.45357	-4.39742	-1.51310
C	2.43715	-2.10592	-0.04836
C	2.26625	-2.99679	1.22941
H	2.40680	-2.36470	2.13408
H	1.23363	-3.39837	1.28705
C	3.90753	-1.59826	-0.04485
H	4.10100	-0.97134	-0.93648
H	4.08915	-0.95054	0.83430
C	2.28198	-3.01878	-1.31177
H	1.25127	-3.42402	-1.37669
H	2.43446	-2.40391	-2.22635
C	3.27054	-4.17155	1.23823
H	3.09654	-4.78151	2.15240
C	4.71226	-3.62341	1.24093
H	5.44580	-4.45978	1.27056
H	4.88767	-3.00998	2.15259
C	4.92115	-2.76895	-0.02601
H	5.94661	-2.33860	-0.02318
C	4.72874	-3.64626	-1.27998
H	5.46183	-4.48353	-1.28471
H	4.91668	-3.04935	-2.20008
C	3.28669	-4.19362	-1.28649
H	3.12447	-4.81959	-2.19194
C	3.05173	-5.04523	-0.01805
H	2.01943	-5.46157	-0.02094
H	3.74656	-5.91410	-0.00616

H_2

SCF (BP86) Energy = -1.172397
Enthalpy 0K = -1.162653
Enthalpy 298K = -1.159349
Free Energy 298K = -1.174202
Frequency = 4276.9563 cm^{-1}
SCF (BP86-D3^{BJ}) Energy = -1.172508
SCF (C_6H_6) Energy = -1.171993
SCF (BS2) Energy = -1.177713

H	0.00000	0.00000	0.38368
H	0.00000	0.00000	-0.38368

M^{Me}

SCF (BP86) Energy = -1671.134906
Enthalpy 0K = -1670.416566
Enthalpy 298K = -1670.370826
Free Energy 298K = -1670.506609
Lowest Frequency = 2.6918 cm^{-1}
Second Frequency = 3.3221 cm^{-1}
SCF (BP86-D3^{BJ}) Energy = -1671.323075
SCF (C_6H_6) Energy = -1671.170531
SCF (BS2) Energy = -1672.787509

N	-1.65705	-1.42260	0.00022
C	-2.81514	-3.60530	0.00075
H	-2.58736	-4.68722	0.00090
H	-3.44245	-3.37277	-0.88482
H	-3.44223	-3.37246	0.88639
C	-1.54937	-2.76625	0.00046
C	-2.95422	-0.80337	0.00022
C	-3.57291	-0.45878	1.23981
C	-4.80705	0.22246	1.21152
H	-5.29328	0.49716	2.16138
C	-5.42574	0.56079	0.00020
H	-6.39015	1.09318	0.00019
C	-4.80735	0.22189	-1.21111
H	-5.29382	0.49613	-2.16098
C	-3.57322	-0.45937	-1.23940
C	-2.89974	-0.76445	2.58022
H	-2.08952	-1.49756	2.38245
C	-2.23835	0.50771	3.15736
H	-1.51972	0.96168	2.44126
H	-1.69124	0.28272	4.09751
H	-2.99986	1.28394	3.38437
C	-3.85491	-1.40525	3.60682
H	-4.65939	-0.70657	3.92015
H	-3.30076	-1.69520	4.52428
H	-4.34241	-2.31619	3.20143
C	-2.90037	-0.76566	-2.57983
H	-2.09016	-1.49875	-2.38193
C	-3.85582	-1.40680	-3.60596
H	-4.34328	-2.31755	-3.20010
H	-3.30187	-1.69715	-4.52342
H	-4.66032	-0.70821	-3.91939
C	-2.23903	0.50622	-3.15763
H	-3.00054	1.28238	-3.38485
H	-1.69208	0.28079	-4.09778
H	-1.52025	0.96048	-2.44186
H	0.13145	1.54755	-0.00029
Mg	-0.02358	-0.16806	-0.00008
N	1.36387	-1.68977	0.00006
C	2.12149	-4.04166	0.00034
H	2.77973	-3.92237	0.88594
H	1.70754	-5.06688	0.00048
H	2.77964	-3.92256	-0.88535
C	1.02246	-2.99369	0.00028
C	-0.31558	-3.46937	0.00045
H	-0.41235	-4.56377	0.00062
C	2.74928	-1.30675	-0.00006
C	3.41879	-1.07503	1.23940
C	4.75283	-0.61951	1.21098
H	5.27942	-0.43390	2.16094
C	5.42113	-0.39439	-0.00032
H	6.46363	-0.03840	-0.00042
C	4.75274	-0.61995	-1.21150
H	5.27927	-0.43468	-2.16156
C	3.41871	-1.07548	-1.23966
C	2.70391	-1.25904	2.58044
H	1.77208	-1.82924	2.38159
C	2.28977	0.10955	3.16726
H	1.66477	0.69217	2.45656
H	1.71334	-0.01696	4.10832
H	3.18166	0.73098	3.39601
C	3.52786	-2.07129	3.59956
H	4.44939	-1.53552	3.91142

H	2.93348	-2.25827	4.51862
H	3.83627	-3.05472	3.18776
C	2.70371	-1.25991	-2.58059
H	1.77191	-1.83006	-2.38149
C	3.52759	-2.07244	-3.59953
H	3.83608	-3.05574	-3.18745
H	2.93311	-2.25974	-4.51847
H	4.44906	-1.53674	-3.91167
C	2.28951	0.10850	-3.16779
H	3.18138	0.72987	-3.39680
H	1.71300	-0.01830	-4.10875
H	1.66457	0.69134	-2.45722
C	0.75011	7.75011	-0.00062
C	-0.53256	7.17223	-0.00191
C	1.89078	6.92665	0.00080
H	-1.42668	7.81685	-0.00302
H	2.89604	7.37878	0.00182
C	-0.67326	5.77238	-0.00179
C	1.74778	5.52703	0.00091
H	-1.67784	5.31910	-0.00281
H	2.64095	4.88138	0.00201
C	0.46599	4.94643	-0.00039
H	0.35489	3.84859	-0.00030
H	0.86123	8.84668	-0.00071

TS (M^{Me}–N^{Me})

SCF (BP86) Energy = -1671.086565
Enthalpy 0K = -1670.371769
Enthalpy 298K = -1670.327272
Free Energy 298K = -1670.451115
Lowest Frequency = -1097.9034 cm^{-1}
Second Frequency = 14.0116 cm^{-1}
SCF (BP86-D3^{BJ}) Energy = -1671.291489
SCF (C_6H_6) Energy = -1671.116691
SCF (BS2) Energy = -1672.739019

N	-1.53796	-0.83692	0.66763
C	-2.46626	-2.07801	2.59335
H	-3.25076	-2.53661	1.95820
H	-2.95140	-1.22681	3.11519
H	-2.14301	-2.81151	3.35499
C	-1.29550	-1.59479	1.75465
C	-2.87548	-0.42011	0.35030
C	-3.42015	0.74820	0.96947
C	-4.70703	1.17764	0.58611
H	-5.13511	2.07837	1.05547
C	-5.45145	0.48719	-0.38036
H	-6.45645	0.83905	-0.66293
C	-4.90516	-0.65135	-0.98719
H	-5.49074	-1.19142	-1.74886
C	-3.62080	-1.12473	-0.64514
C	-2.63622	1.56246	2.00342
H	-1.72025	0.98875	2.25495
C	-2.18083	2.91633	1.41520
H	-1.57585	2.78202	0.49505
H	-1.56791	3.47995	2.15035
H	-3.05267	3.55098	1.14771
C	-3.42445	1.77361	3.31340
H	-4.32608	2.40279	3.15563
H	-2.79390	2.28812	4.06893
H	-3.76416	0.81252	3.75273
C	-3.07812	-2.38057	-1.33363
H	-2.02186	-2.50407	-1.01190
C	-3.84583	-3.64716	-0.89368
H	-3.81834	-3.78721	0.20657
H	-3.40720	-4.55448	-1.36044
H	-4.91373	-3.59662	-1.19639
C	-3.07713	-2.24995	-2.87071
H	-4.10711	-2.16468	-3.27825
H	-2.60937	-3.14353	-3.33478
H	-2.50191	-1.36199	-3.20225
H	-0.00016	-1.08776	-2.35980
Mg	0.00001	-0.31262	-0.58512
N	1.53816	-0.83707	0.66739
C	2.46657	-2.07830	2.59304
H	2.95074	-1.22737	3.11622
H	2.14348	-2.81295	3.35365
H	3.25174	-2.53555	1.95774
C	1.29580	-1.59495	1.75442
C	0.00016	-1.97004	2.20342
H	0.00020	-2.61852	3.09026
C	2.87565	-0.42024	0.34995
C	3.42063	0.74767	0.96957
C	4.70754	1.17703	0.58622
H	5.13587	2.07746	1.05592
C	5.45167	0.48689	-0.38069
H	6.45671	0.83866	-0.66323
C	4.90504	-0.65122	-0.98803
H	5.49038	-1.19102	-1.75006
C	3.62062	-1.12448	-0.64603
C	2.63698	1.56167	2.00394
H	1.72092	0.98805	2.25535
C	2.18179	2.91585	1.41627
H	1.57658	2.78198	0.49622
H	1.56915	3.47936	2.15174
H	3.05372	3.55038	1.14882
C	3.42539	1.77219	3.31391
H	4.32712	2.40127	3.15627
H	2.79502	2.28653	4.06970
H	3.76499	0.81088	3.75284
C	3.07750	-2.37983	-1.33506
H	2.02101	-2.50276	-1.01384
C	3.84420	-3.64699	-0.89499
H	3.81596	-3.78730	0.20520
H	3.40532	-4.55393	-1.36226
H	4.91231	-3.59697	-1.19706
C	3.07724	-2.24891	-2.87211
H	4.10745	-2.16439	-3.27925
H	2.60896	-3.14203	-3.33653
H	2.50288	-1.36042	-3.20372
C	1.21193	2.11356	-2.34177
C	-0.00024	1.42173	-2.08193
C	1.21807	3.44228	-2.80440
H	-0.00015	-0.01607	-2.37223
H	2.17276	3.96070	-2.99374
C	-1.21260	2.11315	-2.34192
C	-0.00063	4.10839	-3.03312
H	-2.18069	1.60145	-2.18709
H	-0.00078	5.14731	-3.40167
C	-1.21914	3.44186	-2.80457
H	-2.17397	3.95995	-2.99404
H	2.18018	1.60220	-2.18684

N^{Me}

SCF (BP86) Energy = -1671.119059
Enthalpy 0K = -1670.405950
Enthalpy 298K = -1670.358285
Free Energy 298K = -1670.491399

Lowest Frequency = 17.5927 cm^{-1}
Second Frequency = 19.4249 cm^{-1}
SCF (BP86-D3^{BJ}) Energy = -1671.320903
SCF (C_6H_6) Energy = -1671.149736
SCF (BS2) Energy = -1672.771482

N	-1.51227	-0.99614	0.40301
C	-2.47992	-3.11297	1.22957
H	-3.12992	-3.26368	0.34281
H	-3.11847	-2.62609	1.99569
H	-2.16119	-4.10141	1.60868
C	-1.29062	-2.23487	0.88317
C	-2.85434	-0.51453	0.22315
C	-3.49211	0.21063	1.27527
C	-4.76912	0.75953	1.03806
H	-5.26969	1.32516	1.84054
C	-5.41276	0.60371	-0.19736
H	-6.40995	1.04185	-0.36214
C	-4.77808	-0.11112	-1.22259
H	-5.28579	-0.22874	-2.19358
C	-3.50130	-0.68072	-1.03912
C	-2.80730	0.43621	2.62595
H	-1.91234	-0.22027	2.65489
C	-2.31366	1.89452	2.75802
H	-1.63835	2.18093	1.92376
H	-1.76391	2.04288	3.71174
H	-3.16543	2.60749	2.74459
C	-3.70055	0.04478	3.82107
H	-4.59388	0.69964	3.90280
H	-3.13838	0.13849	4.77408
H	-4.06081	-1.00193	3.74024
C	-2.82598	-1.42147	-2.19645
H	-1.93551	-1.93876	-1.78140
C	-3.73111	-2.50132	-2.82394
H	-4.09892	-3.22211	-2.06431
H	-3.17517	-3.07433	-3.59562
H	-4.61974	-2.05920	-3.32239
C	-2.32339	-0.42881	-3.26881
H	-3.17011	0.12061	-3.73274
H	-1.78089	-0.96063	-4.07915
H	-1.63885	0.33401	-2.84012
H	0.00068	-5.49427	-1.12082
Mg	0.00006	0.30526	-0.10324
N	1.51252	-0.99595	0.40303
C	2.48044	-3.11267	1.22958
H	3.11910	-2.62559	1.99546
H	2.16183	-4.10105	1.60893
H	3.13028	-3.26355	0.34272
C	1.29103	-2.23472	0.88318
C	0.00024	-2.78953	1.09465
H	0.00030	-3.81115	1.49893
C	2.85453	-0.51419	0.22312
C	3.49218	0.21124	1.27513
C	4.76912	0.76027	1.03785
H	5.26960	1.32611	1.84024
C	5.41282	0.60432	-0.19752
H	6.40995	1.04256	-0.36235
C	4.77826	-0.11078	-1.22264
H	5.28601	-0.22851	-2.19360
C	3.50155	-0.68051	-1.03910
C	2.80735	0.43691	2.62578
H	1.91236	-0.21953	2.65472
C	2.31378	1.89526	2.75779
H	1.63848	2.18165	1.92351
H	1.76401	2.04367	3.71149
H	3.16558	2.60819	2.74435
C	3.70056	0.04549	3.82092
H	4.59394	0.70027	3.90260
H	3.13840	0.13929	4.77393
H	4.06072	-1.00126	3.74015
C	2.82627	-1.42147	-2.19633
H	1.93614	-1.93918	-1.78112
C	3.73170	-2.50085	-2.82420
H	4.10009	-3.22152	-2.06474
H	3.17575	-3.07407	-3.59572
H	4.61994	-2.05830	-3.32295
C	2.32298	-0.42890	-3.26845
H	3.16935	0.12094	-3.73252
H	1.78050	-0.96084	-4.07871
H	1.63822	0.33359	-2.83950
C	1.20527	2.98617	-1.13404
C	-0.00026	2.28140	-0.86341
C	1.21301	4.30147	-1.63889
H	0.00346	-6.16887	-1.48725
H	2.17088	4.81264	-1.83518
C	-1.20615	2.98569	-1.13372
C	-0.00093	4.96377	-1.89298
H	-2.18275	2.50151	-0.94801
H	-0.00119	5.99317	-2.28808
C	-1.21454	4.30099	-1.63856
H	-2.17267	4.81177	-1.83459
H	2.18211	2.50237	-0.94860

A′ Ad

SCF (BP86) Energy = -2528.107929
Enthalpy 0K = -2526.901049
Enthalpy 298K = -2526.837719
Free Energy 298K = -2527.005305
Lowest Frequency = 3.7550 cm^{-1}
Second Frequency = 5.5177 cm^{-1}
SCF (BP86-D3^{BJ}) Energy = -2528.453509
SCF (C_6H_6) Energy = -2528.154214
SCF (BS2) Energy = -2530.656849

Mg	-0.21975	-0.72338	1.34270
N	1.05550	-1.44586	-0.15842
N	-0.31355	1.21707	0.52014
C	2.13649	-0.61510	-0.02783
C	3.61128	-1.16351	0.04569
C	3.76587	-2.64740	-0.38669
H	3.05599	-3.27979	0.18641
H	3.48329	-2.77241	-1.45264
C	4.02562	-1.08965	1.55221
H	3.94847	-0.04343	1.91140
H	3.31942	-1.69594	2.16164
C	4.64327	-0.32916	-0.76108
H	4.38617	-0.36253	-1.85099
H	4.59898	0.73895	-0.46729
C	5.21221	-3.16371	-0.16720
H	5.25157	-4.22765	-0.50660
C	5.58254	-3.07610	1.32638
H	6.60601	-3.46240	1.49683
H	4.88744	-3.70994	1.92910
C	5.47292	-1.60340	1.77969
H	5.71111	-1.52167	2.86116
C	6.45307	-0.74444	0.95229
H	7.50239	-1.07981	1.11336
H	6.40181	0.31601	1.28172

C	6.08959	-0.84022	-0.54436
H	6.77850	-0.21093	-1.15061
C	6.18373	-2.31327	-0.99819
H	5.95112	-2.40491	-2.08202
H	7.23133	-2.68732	-0.86609
C	2.02326	0.76753	0.27513
H	2.98964	1.27784	0.37833
C	0.90119	1.67808	0.22666
C	1.24961	3.10342	-0.31932
C	1.97031	2.91737	-1.70454
H	2.84195	2.24843	-1.59657
H	1.26476	2.40153	-2.40219
C	2.24842	3.83971	0.64178
H	1.77229	4.00638	1.61451
H	3.14877	3.21307	0.82373
C	0.03500	4.03974	-0.55325
H	-0.68157	3.55129	-1.23705
H	-0.49956	4.23012	0.38555
C	2.41048	4.27086	-2.29540
H	2.91840	4.08036	-3.27206
C	3.39810	4.95322	-1.32102
H	4.30314	4.31932	-1.18091
H	3.75494	5.91950	-1.75014
C	2.69184	5.19379	0.03035
H	3.39783	5.68680	0.73929
C	1.45410	6.09816	-0.18133
H	0.95373	6.29636	0.78889
H	1.78091	7.08480	-0.59483
C	0.49138	5.39648	-1.15970
H	-0.41547	6.02662	-1.30283
C	1.18135	5.17321	-2.51794
H	1.49215	6.14702	-2.95517
H	0.47786	4.70131	-3.24107
C	0.91656	-2.50190	-1.09818
C	1.11756	-2.30488	-2.50105
C	1.58256	-0.98545	-3.12747
H	1.86094	-0.28756	-2.31536
C	2.82699	-1.15581	-4.03190
H	3.68147	-1.60684	-3.48614
H	3.14831	-0.17258	-4.44150
H	2.60706	-1.82405	-4.90711
C	0.44698	-0.31957	-3.92730
H	0.12331	-0.95165	-4.77973
H	0.77456	0.66301	-4.34788
H	-0.44937	-0.13162	-3.29646
C	0.81556	-3.36612	-3.39401
H	0.96409	-3.20485	-4.47897
C	0.34330	-4.60133	-2.95131
H	0.11531	-5.40889	-3.66755
C	0.15810	-4.79264	-1.57217
H	-0.20664	-5.77145	-1.20617
C	0.44177	-3.78331	-0.64203
C	0.22881	-4.09786	0.83976
H	0.63893	-3.22799	1.41471
C	1.02077	-5.33563	1.30454
H	0.66132	-6.26049	0.82053
H	0.92522	-5.47336	2.40111
H	2.09804	-5.24115	1.06813
C	-1.26629	-4.24677	1.17835
H	-1.84625	-3.34118	0.89566
H	-1.40784	-4.41514	2.26580
H	-1.72344	-5.10395	0.63982
C	-1.56237	1.90091	0.69817
C	-2.63267	1.62034	-0.21536
C	-2.41508	0.67426	-1.38857
H	-1.31049	0.62108	-1.57116
C	-3.06809	1.15611	-2.70145
H	-4.17604	1.15951	-2.62905
H	-2.79933	0.48511	-3.54302
H	-2.73472	2.18433	-2.96786
C	-2.88623	-0.75356	-1.04049
H	-2.42073	-1.12795	-0.10016
H	-2.61485	-1.47709	-1.84086
H	-3.98514	-0.78644	-0.88906
C	-3.90545	2.17847	0.01382
H	-4.72135	1.96524	-0.69047
C	-4.14639	2.98309	1.13036
H	-5.14693	3.40418	1.29703
C	-3.10760	3.23818	2.04248
H	-3.30463	3.85657	2.92247
C	-1.81232	2.71539	1.85193
C	-0.74867	2.98842	2.93469
H	0.22684	2.65451	2.53228
C	-0.62830	4.48283	3.30340
H	-0.46587	5.12545	2.41199
H	0.21829	4.63094	3.99694
H	-1.54723	4.84894	3.81607
C	-1.03867	2.14925	4.19613
H	-2.00130	2.43227	4.65685
H	-0.23175	2.31589	4.95600
H	-1.06759	1.06603	3.96936
C	-0.33921	-1.46893	3.34506
H	0.23903	-2.42507	3.35668
H	0.26371	-0.77987	3.96482
C	-1.70645	-1.71025	4.05678
H	-2.30717	-0.76373	4.08362
H	-2.32269	-2.41720	3.46028
C	-1.59934	-2.25499	5.50145
H	-0.99180	-1.54446	6.10528
H	-1.00870	-3.20552	5.48620
C	-2.94863	-2.48874	6.17375
H	-3.54434	-1.54747	6.23617
H	-2.83208	-2.88393	7.21266
H	-3.57138	-3.22372	5.61293
C	-7.43508	-2.12315	-0.88301
C	-8.03394	-1.02650	-0.23396
C	-7.80318	-2.42730	-2.22173
H	-7.74927	-0.78518	0.81611
H	-7.33377	-3.29003	-2.72200
C	-9.00131	-0.23458	-0.92380
C	-8.77032	-1.63514	-2.91144
H	-9.46276	0.62695	-0.42306
H	-9.06615	-1.87773	-3.96111
C	-9.36948	-0.53893	-2.26234
H	-10.11609	0.09072	-2.82171
H	-6.68179	-2.75440	-0.33300

O^Me

SCF (BP86) Energy = -3424.451593
Enthalpy 0K = -3422.890006
Enthalpy 298K = -3422.791371
Free Energy 298K = -3423.062985
Lowest Frequency = 1.0385 cm^{-1}
Second Frequency = 1.5237 cm^{-1}
SCF (BP86-D3^{BJ}) Energy = -3424.858949
SCF (C_6H_6) Energy = -3424.516226
SCF (BS2) Energy = -3427.852773

N	-6.07663	1.41080	-1.23493
C	-6.60851	2.84074	-3.18003
H	-6.78527	3.75870	-2.58167
H	-7.27584	2.85931	-4.06147
H	-5.55617	2.90225	-3.52622
C	-6.82996	1.59771	-2.33555
C	-5.08634	2.38522	-0.86764
C	-5.44044	3.45415	0.01121
C	-4.43351	4.34767	0.42960
H	-4.69486	5.17420	1.11017
C	-3.10672	4.20647	-0.00082
H	-2.33395	4.91522	0.33619
C	-2.77035	3.15496	-0.86377
H	-1.72594	3.04577	-1.19865
C	-3.73826	2.23253	-1.31264
C	-6.87003	3.61971	0.53397
H	-7.52302	2.96137	-0.07656
C	-6.97707	3.13977	1.99837
H	-6.64113	2.08706	2.11252
H	-8.02326	3.20661	2.36603
H	-6.34148	3.75696	2.66856
C	-7.40308	5.05914	0.38534
H	-6.84661	5.77691	1.02466
H	-8.47026	5.11219	0.68785
H	-7.32810	5.41848	-0.66206
C	-3.31575	1.08934	-2.23943
H	-4.23884	0.54836	-2.53696
C	-2.64497	1.59810	-3.53301
H	-3.29385	2.31332	-4.08004
H	-2.42092	0.75179	-4.21638
H	-1.68454	2.11452	-3.32223
C	-2.40395	0.08152	-1.50534
H	-1.44990	0.55609	-1.19158
H	-2.15036	-0.77555	-2.16506
H	-2.88968	-0.31885	-0.59155
Mg	-6.24087	-0.25361	-0.01051
N	-7.86259	-1.03636	-1.04878
C	-9.38139	-1.24948	-2.99815
H	-9.85043	-0.59083	-3.75309
H	-8.92971	-2.11201	-3.53394
H	-10.16854	-1.66993	-2.34205
C	-8.31450	-0.51058	-2.20496
C	-7.83735	0.69910	-2.77504
H	-8.33448	0.99082	-3.71055
C	-8.43777	-2.24747	-0.53700
C	-9.42994	-2.16592	0.49046
C	-9.95920	-3.36459	1.01125
H	-10.72948	-3.31876	1.79648
C	-9.52059	-4.61578	0.55513
H	-9.94832	-5.53949	0.97629
C	-8.52461	-4.68382	-0.42781
H	-8.16847	-5.66959	-0.76813
C	-7.96454	-3.51712	-0.98786
C	-9.92468	-0.81222	1.01150
H	-9.09148	-0.08857	0.86065
C	-10.26481	-0.82266	2.51397
H	-9.43990	-1.24516	3.12250
H	-10.45794	0.21076	2.87008
H	-11.18139	-1.41227	2.72861
C	-11.12605	-0.28068	0.19728
H	-11.98258	-0.98614	0.25545
H	-11.46627	0.69947	0.59408
H	-10.87479	-0.13865	-0.87193
C	-6.83506	-3.63823	-2.01492
H	-6.69120	-2.63645	-2.47140
C	-7.15929	-4.62279	-3.15693
H	-8.11826	-4.37377	-3.65732
H	-6.35827	-4.60397	-3.92566
H	-7.23642	-5.66918	-2.79269
C	-5.50683	-4.01773	-1.32176
H	-5.57939	-5.01616	-0.84021
H	-4.67246	-4.05392	-2.05432
H	-5.23647	-3.28882	-0.52930
C	-4.90959	-0.95978	1.50875
H	-4.26720	-0.09638	1.80920
H	-4.20259	-1.65522	0.99325
C	-5.44588	-1.65398	2.77618
H	-6.03531	-2.56037	2.50175
H	-4.60749	-2.03622	3.41151
C	-6.32194	-0.75412	3.66089
H	-7.18701	-0.38560	3.05809
H	-5.74448	0.15929	3.93385
C	-6.83910	-1.43402	4.93508
H	-5.99971	-1.77226	5.58019
H	-7.47147	-0.75294	5.54291
H	-7.44820	-2.33257	4.69555
N	8.17101	-0.91547	0.18566
C	10.25940	-1.81528	1.15993
H	10.59398	-2.46338	1.99094
H	10.70082	-0.80603	1.29240
H	10.68184	-2.21080	0.21345
C	8.74376	-1.73454	1.09093
C	8.98942	-0.18540	-0.74239
C	9.41297	-0.80427	-1.96006
C	10.13100	-0.03253	-2.89613
H	10.45701	-0.49855	-3.84033
C	10.43778	1.31367	-2.65400
H	10.99896	1.89810	-3.40033
C	10.02607	1.90878	-1.45445
H	10.27252	2.96607	-1.26322
C	9.30379	1.18410	-0.48361
C	9.09063	-2.26676	-2.28408
H	8.64982	-2.72091	-1.37255
C	8.03342	-2.37353	-3.40415
H	7.10178	-1.83128	-3.14004
H	7.76716	-3.43404	-3.59934
H	8.40773	-1.93836	-4.35516
C	10.34898	-3.08404	-2.64634
H	10.81092	-2.73225	-3.59309
H	10.09163	-4.15538	-2.78469
H	11.12531	-3.02125	-1.85560
C	8.89091	1.88052	0.81610
H	8.33188	1.13672	1.42361
C	10.11678	2.31755	1.64773
H	10.78930	1.46352	1.87073
H	9.79791	2.76006	2.61522
H	10.71790	3.08353	1.11296
C	7.94891	3.07529	0.55596
H	8.45598	3.87346	-0.02679
H	7.61256	3.52640	1.51355
H	7.04796	2.76663	-0.01263
C	4.79706	0.86780	-0.74943
H	3.75893	0.47780	-0.61243
H	4.85103	1.73817	-0.04956
C	4.95291	1.38744	-2.19242
H	5.96659	1.82958	-2.33948
H	4.24030	2.22588	-2.39702
C	4.72883	0.31925	-3.27307

H	5.45242	-0.51517	-3.11042
H	3.72063	-0.13418	-3.13138
C	4.86324	0.83801	-4.71024
H	4.12770	1.64504	-4.91758
H	4.69535	0.03529	-5.45901
H	5.87451	1.26072	-4.89408
Mg	6.12114	-0.58935	0.09139
N	5.68514	-2.03090	1.51855
C	6.19698	-3.73351	3.23618
H	5.57193	-4.52536	2.77335
H	7.06703	-4.20965	3.72486
H	5.56246	-3.26279	4.01514
C	6.62713	-2.71346	2.19622
C	8.02458	-2.54680	2.00565
H	8.65330	-3.15961	2.66616
C	4.29317	-2.27532	1.77919
C	3.59048	-3.24546	1.00123
C	2.20527	-3.40484	1.20860
H	1.65222	-4.14705	0.61053
C	1.51619	-2.63731	2.15794
H	0.43314	-2.77555	2.30359
C	2.21562	-1.69102	2.91868
H	1.67056	-1.08804	3.66323
C	3.60154	-1.49046	2.75095
C	4.30001	-4.07562	-0.07187
H	5.39221	-3.95498	0.08682
C	3.97926	-3.53518	-1.48305
H	4.24096	-2.45992	-1.58192
H	4.53702	-4.09601	-2.26299
H	2.89528	-3.62586	-1.70848
C	3.98677	-5.58232	0.02619
H	2.91981	-5.80054	-0.19174
H	4.59303	-6.15420	-0.70767
H	4.20933	-5.98271	1.03709
C	4.31536	-0.42929	3.59298
H	5.39989	-0.50291	3.36629
C	4.14545	-0.66815	5.10835
H	4.48299	-1.68252	5.40661
H	4.73672	0.07075	5.68958
H	3.08566	-0.56444	5.42424
C	3.85727	0.99411	3.20574
H	2.77777	1.14153	3.42281
H	4.42223	1.76090	3.77753
H	4.00666	1.19095	2.12405
C	1.08249	6.01598	1.75377
C	0.60888	7.30906	2.04257
C	1.90613	5.80208	0.63321
H	-0.03510	7.47651	2.92100
H	2.27785	4.78978	0.40740
C	0.95887	8.38836	1.21093
C	2.25606	6.88173	-0.19791
H	0.58818	9.40116	1.43713
H	2.90135	6.71399	-1.07519
C	1.78246	8.17449	0.09078
H	2.05673	9.01992	-0.56065
H	0.80976	5.17068	2.40589

TS(O^{Me}-P^{Me})

SCF (BP86) Energy = -3424.363851
Enthalpy 0K = -3422.804377
Enthalpy 298K = -3422.709423
Free Energy 298K = -3422.938545
Lowest Frequency = -1063.2950 cm^{-1}
Second Frequency = 11.7746 cm^{-1}
SCF (BP86-D3^{BJ}) Energy = -3424.857678
SCF (C_6H_6) Energy = -3424.414464
SCF (BS2) Energy = -3427.757379

N	-3.89866	1.61058	0.47856
C	-6.05892	2.55382	1.27108
H	-5.67049	3.13007	2.13528
H	-7.08138	2.21122	1.51452
H	-6.11310	3.26786	0.42551
C	-5.14341	1.37917	0.95719
C	-3.59279	2.95936	0.06882
C	-3.12232	3.93570	0.99950
C	-2.91474	5.25621	0.54609
H	-2.56349	6.01818	1.26070
C	-3.14607	5.62168	-0.78461
H	-2.98570	6.66101	-1.11246
C	-3.56892	4.64884	-1.70058
H	-3.73392	4.93423	-2.75100
C	-3.79398	3.31476	-1.30413
C	-2.82402	3.61804	2.46845
H	-3.11361	2.56000	2.64698
C	-1.31573	3.75355	2.77035
H	-0.70400	3.08510	2.13230
H	-1.09964	3.50171	3.82877
H	-0.96289	4.79118	2.59203
C	-3.62736	4.50112	3.44942
H	-3.32038	5.56630	3.38210
H	-3.45358	4.17463	4.49622
H	-4.71949	4.46139	3.25948
C	-4.28689	2.29406	-2.33489
H	-3.99111	1.28955	-1.95880
C	-5.82804	2.29480	-2.45439
H	-6.32160	2.02187	-1.50125
H	-6.16135	1.56531	-3.22272
H	-6.19832	3.29766	-2.75759
C	-3.64853	2.48058	-3.72500
H	-4.01175	3.40072	-4.23014
H	-3.91270	1.62822	-4.38501
H	-2.54299	2.53561	-3.66801
Mg	-2.57863	-0.02196	0.46457
N	-4.05227	-1.52616	0.37102
C	-6.35543	-2.27926	0.97786
H	-6.38971	-2.62454	2.03309
H	-7.35524	-1.86849	0.73887
H	-6.16782	-3.16595	0.34492
C	-5.28231	-1.20927	0.82158
C	-5.70717	0.09823	1.18224
H	-6.72551	0.12990	1.59420
C	-3.80021	-2.86449	-0.09837
C	-3.52220	-3.93612	0.80603
C	-3.28685	-5.22227	0.27412
H	-3.07903	-6.05586	0.96435
C	-3.30980	-5.46494	-1.10373
H	-3.12397	-6.47819	-1.49362
C	-3.56869	-4.40550	-1.98447
H	-3.58868	-4.59872	-3.06787
C	-3.81869	-3.10181	-1.51014
C	-3.47236	-3.76156	2.32725
H	-3.70205	-2.69823	2.55124
C	-2.06589	-4.06470	2.88716
H	-1.28763	-3.43922	2.40968
H	-2.03037	-3.87714	3.98050
H	-1.78911	-5.12761	2.72171
C	-4.51747	-4.63965	3.05263

H	-4.28371	-5.71965	2.93981
H	-4.52424	-4.41717	4.14054
H	-5.54359	-4.48289	2.66483
C	-4.17854	-1.98849	-2.49697
H	-3.87584	-1.02588	-2.02674
C	-5.70660	-1.91777	-2.71957
H	-6.25648	-1.73119	-1.77643
H	-5.96387	-1.10110	-3.42681
H	-6.08296	-2.87074	-3.14922
C	-3.44756	-2.09840	-3.84672
H	-3.79908	-2.96769	-4.44194
H	-3.63848	-1.19340	-4.45967
H	-2.35104	-2.19549	-3.71605
C	-0.85133	-0.25250	2.06605
H	-0.93343	0.81380	1.78065
H	-0.78457	-1.02754	1.28185
C	-1.16137	-0.67973	3.49410
H	-2.14177	-1.20357	3.59570
H	-0.39831	-1.43287	3.80891
C	-1.12890	0.47567	4.50755
H	-1.91075	1.21965	4.23318
H	-0.15533	1.00652	4.42056
C	-1.34332	0.02019	5.95580
H	-0.55185	-0.69081	6.27551
H	-1.32603	0.87615	6.66236
H	-2.32070	-0.49475	6.08005
N	3.29149	1.51275	-1.12019
C	4.27176	2.39778	-3.22820
H	3.44032	3.06823	-3.53035
H	4.75978	2.01661	-4.14449
H	4.99416	3.03190	-2.67677
C	3.75336	1.25846	-2.35949
C	3.52450	2.81921	-0.56659
C	2.62774	3.89915	-0.82603
C	2.88857	5.16255	-0.25453
H	2.19582	5.99611	-0.45522
C	4.00847	5.37867	0.55549
H	4.20073	6.37327	0.98893
C	4.88235	4.31342	0.81416
H	5.75911	4.48574	1.45621
C	4.66669	3.02919	0.27319
C	1.36621	3.72223	-1.66967
H	1.41868	2.71742	-2.13843
C	0.12052	3.74807	-0.76476
H	0.20052	2.98875	0.03910
H	-0.80923	3.54899	-1.33701
H	0.00278	4.73583	-0.27337
C	1.23544	4.76419	-2.79998
H	1.09537	5.79068	-2.39967
H	0.35335	4.53933	-3.43596
H	2.13177	4.78402	-3.45416
C	5.66067	1.90207	0.56910
H	5.06824	0.96140	0.60411
C	6.71064	1.74463	-0.55524
H	6.25271	1.46250	-1.52277
H	7.44374	0.95217	-0.29342
H	7.27631	2.69007	-0.70215
C	6.37658	2.06200	1.92305
H	7.09763	2.90751	1.91593
H	6.95729	1.14676	2.15658
H	5.65903	2.22474	2.75139
C	-0.21899	0.08320	-1.03725
H	-0.62920	-0.91748	-0.74219
H	-0.60037	0.89199	-0.36145
C	-0.53287	0.40919	-2.51273
H	-0.21782	1.45723	-2.71935
H	-1.62742	0.39357	-2.74313
C	0.16504	-0.50452	-3.53249
H	1.26203	-0.48235	-3.34577
H	-0.15034	-1.55913	-3.35705
C	-0.10809	-0.12356	-4.99305
H	-1.19275	-0.15988	-5.23216
H	0.41101	-0.80454	-5.69971
H	0.24167	0.90877	-5.21047
Mg	1.99398	-0.01580	-0.30640
N	3.26295	-1.56485	-1.11441
C	4.20195	-2.48405	-3.21970
H	3.42885	-3.27692	-3.28163
H	4.45622	-2.14893	-4.24253
H	5.09793	-2.96586	-2.77887
C	3.72212	-1.32512	-2.35792
C	3.81180	-0.03592	-2.94949
H	4.14231	-0.04085	-3.99741
C	3.51035	-2.85798	-0.54051
C	2.50246	-3.86930	-0.57078
C	2.74997	-5.11065	0.05150
H	1.96835	-5.88791	0.02858
C	3.96962	-5.38021	0.68412
H	4.14760	-6.35662	1.16250
C	4.96451	-4.39380	0.69221
H	5.92983	-4.60694	1.18015
C	4.76636	-3.13393	0.09059
C	1.15913	-3.64767	-1.26281
H	1.18877	-2.63933	-1.72536
C	0.00866	-3.66020	-0.24063
H	0.17846	-2.90792	0.55780
H	-0.96696	-3.44494	-0.72396
H	-0.08273	-4.64861	0.25592
C	0.89752	-4.66909	-2.38976
H	0.81946	-5.70436	-1.99479
H	-0.05742	-4.44169	-2.90893
H	1.70817	-4.66290	-3.14762
C	5.91473	-2.12140	0.12485
H	5.54473	-1.18486	-0.34041
C	7.13448	-2.60575	-0.69124
H	6.87211	-2.83129	-1.74499
H	7.92977	-1.83045	-0.70340
H	7.57449	-3.52745	-0.25337
C	6.34242	-1.78959	1.56892
H	6.72483	-2.68871	2.09814
H	7.16076	-1.03915	1.56951
H	5.49875	-1.38172	2.16000
C	2.48041	-1.25381	2.66600
C	3.33948	-1.22310	3.77932
C	1.96597	-0.06553	2.06461
H	3.70795	-2.16522	4.21793
H	0.62719	-0.13328	1.87536
C	3.72288	0.01004	4.33834
C	2.38828	1.16346	2.65780
H	4.38698	0.03908	5.21753
H	2.01175	2.12117	2.25349
C	3.24719	1.20740	3.76846
H	3.54008	2.17828	4.20182
H	2.18613	-2.23956	2.26210

P^{Me}

SCF (BP86) Energy = -3424.465657

Enthalpy 0K = -3422.903779

Enthalpy 298K = -3422.804517
Free Energy 298K = -3423.076294
Lowest Frequency = 1.3003 cm^{-1}
Second Frequency = 1.7596 cm^{-1}
SCF (BP86-D3^{BJ}) Energy = -3424.873847
SCF (C_6H_6) Energy = -3424.529739
SCF (BS2) Energy = -3427.866071

N	-6.17666	0.96952	1.14707
C	-7.31706	0.10073	3.16003
H	-6.52019	0.42519	3.86097
H	-7.76841	-0.83326	3.54272
H	-8.08462	0.90227	3.18084
C	-6.76320	-0.07810	1.75701
C	-6.08274	2.23443	1.82107
C	-4.92667	2.53815	2.60154
C	-4.81851	3.82144	3.17598
H	-3.92877	4.06921	3.77709
C	-5.81705	4.78829	2.99597
H	-5.71255	5.78514	3.45313
C	-6.94896	4.47935	2.22873
H	-7.73027	5.24330	2.08730
C	-7.10673	3.21167	1.63250
C	-3.79483	1.52357	2.78383
H	-4.17890	0.53733	2.44835
C	-2.59195	1.87860	1.88100
H	-2.88823	1.95651	0.81338
H	-1.79220	1.11217	1.96178
H	-2.15399	2.85820	2.16860
C	-3.35529	1.36759	4.25358
H	-2.88903	2.29491	4.64897
H	-2.60181	0.55782	4.34949
H	-4.21118	1.11633	4.91408
C	-8.33109	2.92148	0.76058
H	-8.38756	1.82040	0.62923
C	-9.65722	3.37425	1.40280
H	-9.78904	2.94457	2.41756
H	-10.51906	3.05170	0.78142
H	-9.72007	4.47918	1.49636
C	-8.15680	3.54138	-0.64422
H	-8.09405	4.64886	-0.58535
H	-9.01082	3.28238	-1.30553
H	-7.22304	3.19201	-1.13580
Mg	-5.36904	0.88105	-0.75849
N	-5.91322	-1.09776	-1.04954
C	-6.84213	-3.29823	-0.41363
H	-5.92049	-3.85329	-0.68549
H	-7.31917	-3.80047	0.44810
H	-7.51672	-3.38088	-1.29084
C	-6.52511	-1.84422	-0.10912
C	-6.90552	-1.36422	1.17183
H	-7.40077	-2.10739	1.81178
C	-5.59445	-1.67184	-2.32768
C	-4.29674	-2.22397	-2.55253
C	-3.97724	-2.69581	-3.84250
H	-2.97714	-3.12070	-4.02741
C	-4.90293	-2.63494	-4.89282
H	-4.63253	-3.00808	-5.89343
C	-6.17538	-2.09450	-4.66100
H	-6.90068	-2.04619	-5.48953
C	-6.54692	-1.60844	-3.39080
C	-3.24500	-2.29300	-1.44202
H	-3.74251	-1.98486	-0.49816
C	-2.09175	-1.29903	-1.70314
H	-2.46609	-0.25988	-1.81549
H	-1.35896	-1.31303	-0.86875
H	-1.54489	-1.55474	-2.63566
C	-2.70413	-3.72235	-1.22854
H	-2.14581	-4.08854	-2.11621
H	-2.00510	-3.75023	-0.36632
H	-3.52161	-4.44569	-1.02770
C	-7.93755	-0.99889	-3.19171
H	-8.07020	-0.82739	-2.10304
C	-9.07160	-1.93893	-3.65052
H	-9.01089	-2.93016	-3.15490
H	-10.06304	-1.49969	-3.41104
H	-9.04651	-2.11315	-4.74718
C	-8.04525	0.37466	-3.88978
H	-7.93238	0.27511	-4.99044
H	-9.03209	0.84498	-3.69258
H	-7.25561	1.07436	-3.54489
C	-4.38026	-4.46486	4.55040
H	-4.56605	-3.97901	5.53255
H	-4.83281	-3.82013	3.76852
C	-4.94949	-5.88859	4.51498
H	-6.05599	-5.85239	4.64331
H	-4.78148	-6.33314	3.50701
C	-4.35114	-6.82108	5.58070
H	-4.51886	-6.37681	6.58880
H	-3.24486	-6.85764	5.45245
C	-4.92016	-8.24479	5.54507
H	-4.73396	-8.73120	4.56330
H	-4.46617	-8.88889	6.32673
H	-6.01936	-8.24633	5.70932
N	6.42232	2.04383	0.01336
C	7.68079	3.96000	-0.90669
H	6.77533	4.55532	-1.14653
H	8.46056	4.18130	-1.65861
H	8.02803	4.32021	0.08395
C	7.35403	2.47797	-0.85762
C	5.74910	2.97099	0.88053
C	4.51787	3.56230	0.46295
C	3.82562	4.38989	1.37068
H	2.87234	4.84854	1.06220
C	4.32332	4.63844	2.65752
H	3.76592	5.28752	3.35147
C	5.53359	4.05441	3.05607
H	5.92011	4.24998	4.06948
C	6.26643	3.21874	2.18821
C	3.92190	3.28196	-0.91923
H	4.71252	2.79181	-1.52546
C	2.73965	2.29192	-0.81820
H	3.03275	1.34472	-0.31665
H	2.34502	2.03974	-1.82531
H	1.90516	2.72589	-0.22742
C	3.50442	4.56615	-1.66433
H	2.66264	5.08489	-1.15866
H	3.16642	4.32516	-2.69411
H	4.34381	5.28826	-1.74161
C	7.56668	2.57289	2.67444
H	8.04884	2.10027	1.79292
C	8.56481	3.60132	3.24530
H	8.78389	4.40998	2.51731
H	9.52533	3.10872	3.50583
H	8.18121	4.08081	4.17081
C	7.27936	1.45251	3.69897
H	6.80039	1.86070	4.61442
H	8.21866	0.94583	4.00692

H	6.59198	0.68241	3.28869
C	-4.28229	2.35323	-1.85953
H	-3.19797	2.08408	-1.80165
H	-4.35189	3.31034	-1.28701
C	-4.63692	2.62260	-3.33630
H	-5.70276	2.94482	-3.42393
H	-4.57328	1.67937	-3.92891
C	-3.76102	3.68726	-4.02775
H	-3.82480	4.63640	-3.44699
H	-2.69446	3.37058	-3.96034
C	-4.13143	3.94989	-5.49353
H	-4.04243	3.02599	-6.10550
H	-3.47806	4.71952	-5.95654
H	-5.18087	4.30463	-5.58891
Mg	5.87572	0.06737	0.18510
N	7.17387	-0.59196	-1.26980
C	8.91337	-0.35850	-3.00813
H	8.32743	-0.88550	-3.78957
H	9.53113	0.42036	-3.49203
H	9.58552	-1.12243	-2.56500
C	7.99535	0.23031	-1.95165
C	8.06622	1.63422	-1.75083
H	8.79096	2.15348	-2.39284
C	7.16906	-2.00305	-1.54055
C	6.24922	-2.53648	-2.49389
C	6.19476	-3.93457	-2.66947
H	5.48597	-4.35860	-3.39912
C	7.02067	-4.79540	-1.93356
H	6.96145	-5.88488	-2.08537
C	7.92098	-4.26095	-1.00135
H	8.56624	-4.94121	-0.42230
C	8.01636	-2.87072	-0.78618
C	5.30402	-1.63376	-3.29127
H	5.63100	-0.58582	-3.12467
C	3.85458	-1.75264	-2.76917
H	3.78602	-1.52951	-1.68310
H	3.18022	-1.05295	-3.30710
H	3.46026	-2.78096	-2.91465
C	5.36802	-1.89681	-4.80991
H	4.99710	-2.91073	-5.07075
H	4.73660	-1.16640	-5.35837
H	6.40386	-1.81057	-5.19913
C	8.98422	-2.32897	0.26930
H	9.03516	-1.22801	0.13507
C	10.41673	-2.87660	0.10507
H	10.81324	-2.69240	-0.91512
H	11.10385	-2.39144	0.82998
H	10.46654	-3.97063	0.29004
C	8.45024	-2.59445	1.69488
H	8.38105	-3.68422	1.89891
H	9.12121	-2.14904	2.45991
H	7.43321	-2.17238	1.84216
C	4.29237	-2.34680	1.35595
C	3.37238	-3.02453	2.18024
C	4.48433	-0.93910	1.42538
H	3.25477	-4.11825	2.09456
H	-3.28107	-4.46437	4.38697
C	2.60416	-2.30684	3.11386
C	3.68798	-0.24984	2.38139
H	1.88374	-2.83193	3.76284
H	3.78214	0.84629	2.49200
C	2.76433	-0.91346	3.21302
H	2.16724	-0.34083	3.94307
H	4.87658	-2.94962	0.63625

Q

SCF (BP86) Energy = -4824.090123
Enthalpy 0K = -4821.772183
Enthalpy 298K = -4821.651084
Free Energy 298K = -4821.936729
Lowest Frequency = 5.4835 cm^{-1}
Second Frequency = 6.6773 cm^{-1}
SCF (BP86-$D3^{BJ}$) Energy = -4824.829646
SCF (C_6H_6) Energy = -4824.169009
SCF (BS2) Energy = -4828.936207

N	-4.78705	-1.49214	0.83426
C	-5.82535	-1.23017	0.00870
C	-4.73944	-2.51619	1.82368
C	-3.76787	-3.56881	1.74467
C	-3.68557	-4.51898	2.78200
H	-2.94538	-5.33269	2.70249
C	-4.52238	-4.46444	3.90307
H	-4.44340	-5.21911	4.70176
C	-5.45655	-3.42491	3.99244
H	-6.10791	-3.36309	4.87981
C	-5.58431	-2.44765	2.98488
C	-2.82007	-3.72496	0.55339
H	-3.03369	-2.89463	-0.15232
C	-1.34669	-3.60075	0.99110
H	-1.15627	-2.63025	1.49120
H	-0.67237	-3.67661	0.11064
H	-1.06894	-4.41074	1.70006
C	-3.03035	-5.05264	-0.20750
H	-2.80106	-5.93079	0.43388
H	-2.36346	-5.10443	-1.09480
H	-4.07332	-5.16812	-0.56600
C	-6.59223	-1.31698	3.21347
H	-6.65505	-0.71937	2.28145
C	-8.01122	-1.82927	3.54060
H	-8.38024	-2.54830	2.78094
H	-8.73052	-0.98405	3.58763
H	-8.04657	-2.34273	4.52518
C	-6.09967	-0.37722	4.33405
H	-6.05535	-0.90817	5.30930
H	-6.78037	0.49264	4.45368
H	-5.08234	0.00305	4.11734
Mg	-3.08556	-0.14508	0.65964
N	-4.37032	1.61583	0.23187
C	-5.57346	1.38779	-0.31613
C	-6.06330	0.05731	-0.54519
H	-6.93667	0.05655	-1.20239
C	-3.77400	2.88438	0.51343
C	-2.97896	3.55383	-0.47840
C	-2.39434	4.79892	-0.16593
H	-1.81367	5.32331	-0.94293
C	-2.53762	5.38852	1.09602
H	-2.08546	6.36972	1.31318
C	-3.25165	4.69809	2.08284
H	-3.34627	5.14070	3.08785
C	-3.85998	3.45206	1.82848
C	-2.72134	2.97904	-1.87582
H	-3.14172	1.95149	-1.89237
C	-1.20867	2.87994	-2.16604
H	-0.67127	2.36540	-1.34628
H	-1.02436	2.31390	-3.10288
H	-0.75370	3.88614	-2.29079
C	-3.38485	3.79280	-3.00887

H	-2.98998	4.83137	-3.04043
H	-3.17681	3.32668	-3.99569
H	-4.48378	3.85995	-2.89615
C	-4.60573	2.77113	2.97944
H	-4.88443	1.75428	2.63191
C	-5.90377	3.51548	3.36277
H	-6.61178	3.58879	2.51331
H	-6.42529	2.99093	4.19132
H	-5.68667	4.54979	3.70691
C	-3.70762	2.62050	4.22539
H	-3.44880	3.60834	4.66350
H	-4.23095	2.04229	5.01534
H	-2.76350	2.09554	3.98189
C	-1.55236	-0.49991	-1.02981
H	-0.96464	-1.33255	-0.57900
H	-1.00837	0.44874	-0.81564
C	-1.78889	-0.71159	-2.51597
H	-2.35656	0.14731	-2.94527
H	-0.82038	-0.72309	-3.10122
C	-2.53171	-2.00738	-2.88362
H	-3.51436	-2.01630	-2.36212
H	-1.96823	-2.87841	-2.47645
C	-2.74429	-2.19565	-4.39126
H	-1.77690	-2.21716	-4.93926
H	-3.27886	-3.14171	-4.62106
H	-3.34289	-1.36322	-4.82072
N	5.00352	-1.48672	-0.76853
C	6.07559	-1.35602	0.04165
C	4.39348	-2.66716	-1.31141
C	3.35245	-3.30592	-0.56466
C	2.59828	-4.31552	-1.19591
H	1.79385	-4.81717	-0.63754
C	2.85946	-4.70390	-2.51957
H	2.25383	-5.49259	-2.99287
C	3.90682	-4.09749	-3.22534
H	4.12657	-4.42667	-4.25409
C	4.69233	-3.07881	-2.64601
C	3.08900	-2.92768	0.89911
H	4.06907	-2.63578	1.33495
C	2.15808	-1.70500	1.04540
H	2.64096	-0.75997	0.70069
H	1.90023	-1.51881	2.10806
H	1.20743	-1.82935	0.48854
C	2.55320	-4.10100	1.74218
H	1.51283	-4.37081	1.46803
H	2.53969	-3.82295	2.81595
H	3.18373	-5.00719	1.63002
C	5.85157	-2.47124	-3.44367
H	6.38392	-1.77051	-2.76572
C	6.86759	-3.54098	-3.90096
H	7.25339	-4.13894	-3.05035
H	7.73592	-3.06337	-4.40179
H	6.41608	-4.24887	-4.62765
C	5.36086	-1.65877	-4.65913
H	4.83353	-2.30773	-5.39032
H	6.21460	-1.18441	-5.18739
H	4.65142	-0.85715	-4.36967
C	-1.67973	0.00609	2.36537
H	-1.51614	1.10139	2.53011
H	-0.71317	-0.36011	1.93449
C	-1.92250	-0.68701	3.71327
H	-2.09829	-1.77858	3.56417
H	-2.87046	-0.31824	4.17087
C	-0.79858	-0.52448	4.76206
H	0.15465	-0.90639	4.32458
H	-0.62478	0.56288	4.93971
C	-1.07180	-1.22989	6.09804
H	-1.99402	-0.83697	6.57845
H	-0.23853	-1.09934	6.82205
H	-1.22331	-2.32166	5.95362
Mg	3.77875	0.09427	-1.19119
N	4.91767	1.56084	-0.32446
C	6.02175	1.25744	0.39640
C	6.48186	-0.08334	0.53525
H	7.36803	-0.15164	1.17057
C	4.38288	2.85331	-0.65817
C	3.25652	3.36110	0.06010
C	2.61693	4.52198	-0.42591
H	1.74251	4.91791	0.11485
C	3.06974	5.17998	-1.57707
H	2.55278	6.08231	-1.93965
C	4.19578	4.68968	-2.25627
H	4.56175	5.22361	-3.14748
C	4.87214	3.53395	-1.81847
C	2.73143	2.70053	1.33908
H	3.40842	1.85058	1.57424
C	1.30670	2.13930	1.15619
H	1.24163	1.41518	0.31793
H	0.95315	1.61156	2.06547
H	0.57440	2.94504	0.94090
C	2.76462	3.66334	2.54705
H	2.06901	4.51659	2.40432
H	2.44892	3.13357	3.46962
H	3.77749	4.08101	2.72156
C	6.10607	3.03208	-2.57695
H	6.67116	2.37343	-1.88488
C	7.05830	4.16682	-3.00496
H	7.33552	4.81623	-2.14915
H	7.99315	3.74547	-3.43027
H	6.61082	4.81568	-3.78701
C	5.71736	2.17189	-3.79605
H	5.14208	2.76352	-4.53859
H	6.61799	1.76629	-4.30302
H	5.08066	1.30697	-3.51204
C	1.91045	1.45331	-2.80719
C	2.14779	1.59170	-4.18012
C	1.69603	0.15291	-2.23442
H	2.32126	2.59422	-4.60044
H	1.17408	0.06054	-1.25258
C	2.13648	0.45882	-5.02603
C	1.63255	-0.97687	-3.11622
H	2.30537	0.57714	-6.10761
H	1.40795	-1.97334	-2.70537
C	1.86891	-0.81674	-4.48857
H	1.82559	-1.69512	-5.15097
H	1.86908	2.34946	-2.16750
C	-6.63977	2.48104	-0.75522
C	-6.25965	3.97189	-0.51536
H	-6.03540	4.14338	0.55432
H	-5.33704	4.23404	-1.06404
C	-7.96488	2.22973	0.04471
H	-8.29708	1.18111	-0.08820
H	-7.76200	2.35301	1.13105
C	-6.96729	2.33807	-2.28087
H	-6.05008	2.53606	-2.87585
H	-7.27209	1.29946	-2.52296
C	-7.39032	4.93686	-0.95250
H	-7.05049	5.97776	-0.75595

C	-8.67215	4.64769	-0.14544
H	-8.48872	4.81229	0.93953
H	-9.48601	5.34636	-0.44410
C	-9.09547	3.18653	-0.39798
H	-10.01129	2.95116	0.18898
C	-9.37880	2.98470	-1.90376
H	-9.70655	1.93837	-2.09527
H	-10.21180	3.64668	-2.23057
C	-8.10021	3.29854	-2.71327
H	-8.29879	3.14772	-3.79804
C	-7.67665	4.75953	-2.45761
H	-8.47846	5.45862	-2.78620
H	-6.77068	5.00756	-3.05412
C	-6.85025	-2.31698	-0.52769
C	-6.70818	-3.73709	0.08684
H	-5.66914	-4.09583	-0.03685
H	-6.89705	-3.70996	1.17728
C	-6.59560	-2.46701	-2.06749
H	-6.68699	-1.47971	-2.56626
H	-5.54777	-2.80226	-2.22869
C	-8.33921	-1.88642	-0.33002
H	-8.55570	-1.78903	0.75487
H	-8.52098	-0.88665	-0.77492
C	-7.67964	-4.75152	-0.56469
H	-7.52331	-5.74198	-0.08279
C	-7.39746	-4.85907	-2.07721
H	-6.36138	-5.22677	-2.24977
H	-8.08428	-5.59728	-2.54945
C	-7.57924	-3.46716	-2.71666
H	-7.36525	-3.52148	-3.80762
C	-9.03003	-2.98340	-2.49528
H	-9.17876	-1.98816	-2.97124
H	-9.74673	-3.68209	-2.98236
C	-9.31568	-2.89615	-0.97906
H	-10.35748	-2.54019	-0.81551
C	-9.13406	-4.28901	-0.34104
H	-9.84805	-5.01668	-0.78861
H	-9.35939	-4.24513	0.74776
C	7.00729	-2.53273	0.51501
C	7.07052	-2.56583	2.08014
H	7.36700	-1.57590	2.48312
H	6.05496	-2.77551	2.48347
C	8.45371	-2.27863	-0.03198
H	8.42886	-2.28367	-1.14424
H	8.80862	-1.27107	0.26854
C	6.59486	-3.96077	0.05684
H	5.58250	-4.20983	0.43080
H	6.54196	-4.01073	-1.04698
C	8.07141	-3.63229	2.58130
H	8.09109	-3.60347	3.69305
C	9.47966	-3.31517	2.02834
H	9.81707	-2.31657	2.38575
H	10.21636	-4.05614	2.40972
C	9.44625	-3.34572	0.48347
H	10.45740	-3.11264	0.08241
C	9.00135	-4.74265	0.00459
H	8.99486	-4.78329	-1.10739
H	9.72102	-5.51805	0.34892
C	7.59178	-5.03479	0.55963
H	7.24367	-6.02625	0.19593
C	7.62948	-5.02961	2.10116
H	8.33123	-5.80784	2.47478
H	6.62600	-5.27778	2.51234
C	6.89843	2.29180	1.19954
C	8.39538	2.19448	0.74888
H	8.76003	1.14910	0.81659
H	8.47418	2.48427	-0.32251
C	6.81433	1.92793	2.72285
H	5.75962	2.02610	3.06216
H	7.09700	0.86725	2.88405
C	6.49362	3.78861	1.07498
H	6.55056	4.11841	0.01975
H	5.44292	3.93052	1.39091
C	9.30426	3.10030	1.61157
H	10.35442	2.98129	1.26431
C	9.19602	2.67597	3.09393
H	9.52795	1.62069	3.21677
H	9.87283	3.29814	3.72006
C	7.73463	2.83477	3.57110
H	7.65262	2.52439	4.63610
C	7.29780	4.30586	3.41853
H	6.25368	4.43570	3.77980
H	7.93776	4.96771	4.04315
C	7.40359	4.70374	1.93215
H	7.05985	5.75286	1.79941
C	8.86644	4.57158	1.46225
H	9.52803	5.23581	2.06129
H	8.96190	4.89501	0.40182

TS(Q-R)
SCF (BP86) Energy = -4824.082836
Enthalpy 0K = -4821.766206
Enthalpy 298K = -4821.646160
Free Energy 298K = -4821.927702
Lowest Frequency = -535.4764 cm^{-1}
Second Frequency = 6.5824 cm^{-1}
SCF (BP86-D3^{BJ}) Energy = -4824.832988
SCF (C_6H_6) Energy = -4824.156106
SCF (BS2) Energy = -4828.925928

N	-4.58566	-1.55715	0.65736
C	-5.57240	-1.18316	-0.19416
C	-4.61098	-2.70405	1.51087
C	-3.64781	-3.75600	1.36262
C	-3.64591	-4.83026	2.27462
H	-2.91023	-5.64100	2.14272
C	-4.55807	-4.90120	3.33412
H	-4.54266	-5.75216	4.03346
C	-5.48445	-3.86344	3.49302
H	-6.19423	-3.90171	4.33565
C	-5.53069	-2.76293	2.61309
C	-2.61894	-3.77764	0.23096
H	-2.79287	-2.87198	-0.38780
C	-1.18106	-3.70593	0.78375
H	-1.03576	-2.80879	1.41817
H	-0.44124	-3.67687	-0.04494
H	-0.94914	-4.59773	1.40530
C	-2.76199	-5.00644	-0.69390
H	-2.56403	-5.95283	-0.14646
H	-2.03596	-4.94857	-1.53254
H	-3.77712	-5.08445	-1.13302
C	-6.53804	-1.65034	2.92111
H	-6.51683	-0.92393	2.08361
C	-7.98517	-2.17205	3.05288
H	-8.29601	-2.76377	2.16778
H	-8.69699	-1.32680	3.16522
H	-8.10781	-2.82337	3.94439
C	-6.13223	-0.89319	4.20384

H	-6.16322	-1.56262	5.09002	H	2.69740	-0.84903	1.08697
H	-6.82170	-0.04453	4.39835	H	1.82202	-1.57121	2.42971
H	-5.10091	-0.49451	4.12731	H	1.27704	-1.91949	0.76556
Mg	-2.95245	-0.19101	0.88980	C	2.56302	-4.11004	2.29876
N	-4.15664	1.60334	0.51193	H	1.53482	-4.41844	2.01759
C	-5.34334	1.45261	-0.09816	H	2.51237	-3.73570	3.34168
C	-5.79956	0.17038	-0.55680	H	3.20627	-5.01412	2.29076
H	-6.63936	0.26779	-1.25013	C	5.83466	-2.70563	-3.00399
C	-3.53993	2.82885	0.92565	H	6.29488	-1.90018	-2.39371
C	-2.86624	3.67237	-0.02409	C	6.95413	-3.69848	-3.38575
C	-2.20843	4.83228	0.43614	H	7.39659	-4.18940	-2.49482
H	-1.71124	5.48728	-0.29826	H	7.77060	-3.17373	-3.92527
C	-2.18165	5.18082	1.79142	H	6.58021	-4.50064	-4.05658
H	-1.67413	6.10006	2.12507	C	5.25627	-2.04716	-4.27255
C	-2.80600	4.33633	2.71730	H	4.78539	-2.80148	-4.93801
H	-2.77770	4.59840	3.78633	H	6.05630	-1.53999	-4.85201
C	-3.47428	3.15985	2.32112	H	4.47646	-1.29573	-4.03616
C	-2.81213	3.38894	-1.53118	C	-1.56107	-0.30407	2.56045
H	-3.41086	2.47452	-1.72574	H	-1.31160	0.74726	2.85213
C	-1.36621	3.11888	-1.99555	H	-0.60535	-0.69489	2.12931
H	-0.88976	2.31035	-1.41193	C	-1.89712	-1.11305	3.82558
H	-1.34375	2.82516	-3.06544	H	-2.14959	-2.16637	3.55724
H	-0.73722	4.02722	-1.88533	H	-2.82847	-0.71989	4.29707
C	-3.39010	4.53639	-2.39090	C	-0.79756	-1.14594	4.90915
H	-2.77015	5.45435	-2.30701	H	0.13470	-1.56215	4.45895
H	-3.40205	4.24665	-3.46301	H	-0.54153	-0.09960	5.19757
H	-4.42382	4.80961	-2.10394	C	-1.16821	-1.95246	6.16148
C	-4.14564	2.30934	3.40476	H	-2.06966	-1.53231	6.65819
H	-4.27937	1.28993	2.98269	H	-0.34962	-1.96257	6.91294
C	-5.54446	2.84794	3.77979	H	-1.40245	-3.00871	5.90624
H	-6.24983	2.81172	2.92692	Mg	3.40668	-0.14296	-1.03927
H	-5.98533	2.24929	4.60413	N	4.54650	1.51907	-0.55648
H	-5.48228	3.90286	4.12378	C	5.67798	1.42046	0.17783
C	-3.29127	2.18039	4.68187	C	6.23190	0.15919	0.53669
H	-3.24328	3.13642	5.24602	H	7.13086	0.25991	1.14885
H	-3.73796	1.42879	5.36526	C	3.94131	2.70978	-1.08692
H	-2.25563	1.86136	4.45281	C	2.87905	3.33886	-0.36462
C	-1.14210	-0.33996	-0.91251	C	2.18861	4.40619	-0.97183
H	-0.93947	-1.31843	-0.45217	H	1.37304	4.89924	-0.42019
H	-0.89543	0.55606	-0.31974	C	2.52517	4.85777	-2.25670
C	-1.70065	-0.23164	-2.30990	H	1.97216	5.69367	-2.71348
H	-2.58547	0.44744	-2.35582	C	3.57601	4.24308	-2.95039
H	-0.95363	0.25892	-2.99768	H	3.84674	4.60888	-3.95437
C	-2.09175	-1.57995	-2.93875	C	4.30197	3.17043	-2.39143
H	-2.92015	-2.02840	-2.34561	C	2.49152	2.87759	1.04354
H	-1.23630	-2.28572	-2.84737	H	3.33720	2.26971	1.43000
C	-2.51035	-1.46993	-4.40907	C	1.24554	1.97018	1.00745
H	-1.67958	-1.07822	-5.03464	H	1.36044	1.12465	0.29446
H	-2.80977	-2.45380	-4.82771	H	1.01986	1.53668	2.00333
H	-3.37265	-0.77958	-4.53241	H	0.35084	2.54052	0.68174
N	4.83369	-1.53739	-0.46774	C	2.27121	4.04186	2.03060
C	5.92245	-1.20103	0.25301	H	1.36876	4.63494	1.77413
C	4.37844	-2.83494	-0.86780	H	2.11447	3.64841	3.05641
C	3.41854	-3.51056	-0.04893	H	3.13840	4.73403	2.05781
C	2.80673	-4.67405	-0.55761	C	5.45864	2.55716	-3.18891
H	2.06571	-5.20826	0.05491	H	5.92646	1.77901	-2.54951
C	3.13178	-5.17591	-1.82827	C	6.54807	3.59771	-3.52989
H	2.63532	-6.08345	-2.20638	H	6.93280	4.11005	-2.62474
C	4.10609	-4.52967	-2.59983	H	7.40817	3.10990	-4.03542
H	4.37984	-4.94475	-3.58349	H	6.16244	4.38103	-4.21638
C	4.74880	-3.36021	-2.14316	C	4.96745	1.86687	-4.47808
C	3.11086	-3.01046	1.36986	H	4.50716	2.59870	-5.17579
H	4.08096	-2.67768	1.80161	H	5.81212	1.38282	-5.01211
C	2.17505	-1.78134	1.39939	H	4.20299	1.09159	-4.26993

C	1.41407	0.74347	-3.08793
C	1.51008	0.60237	-4.48301
C	1.48745	-0.38690	-2.18914
H	1.45654	1.49825	-5.12256
H	0.63293	-0.35790	-1.31543
C	1.66273	-0.66911	-5.06459
C	1.61998	-1.67451	-2.83845
H	1.72996	-0.77888	-6.15830
H	1.63403	-2.58821	-2.21918
C	1.70671	-1.80814	-4.22647
H	1.81474	-2.81173	-4.66858
H	1.28422	1.75559	-2.66766
C	-6.43037	2.58083	-0.35180
C	-6.10578	4.00573	0.18536
H	-5.89610	3.96840	1.27133
H	-5.18992	4.40296	-0.28579
C	-7.75866	2.13659	0.35781
H	-8.05336	1.12500	0.01659
H	-7.57463	2.05080	1.45136
C	-6.73050	2.72720	-1.88175
H	-5.81468	3.06969	-2.40826
H	-6.99543	1.74558	-2.32616
C	-7.26568	5.00039	-0.07424
H	-6.96494	5.99291	0.32800
C	-8.54763	4.51891	0.63386
H	-8.38559	4.47093	1.73347
H	-9.38025	5.23744	0.46151
C	-8.91577	3.12447	0.08902
H	-9.82986	2.74748	0.59957
C	-9.17017	3.21090	-1.43285
H	-9.45854	2.21205	-1.83034
H	-10.02077	3.89715	-1.64261
C	-7.89208	3.71718	-2.13862
H	-8.06995	3.77773	-3.23551
C	-7.52616	5.11221	-1.59093
H	-8.34959	5.83407	-1.79094
H	-6.62441	5.50574	-2.11027
C	-6.53130	-2.18708	-0.95825
C	-6.42013	-3.67694	-0.53248
H	-5.36883	-4.01041	-0.61134
H	-6.70496	-3.79945	0.53060
C	-6.13948	-2.11793	-2.47503
H	-6.20964	-1.07052	-2.83647
H	-5.07466	-2.41698	-2.58790
C	-8.03950	-1.79570	-0.83819
H	-8.35273	-1.84998	0.22613
H	-8.20169	-0.74604	-1.15817
C	-7.31186	-4.59666	-1.40176
H	-7.18111	-5.64324	-1.04824
C	-6.89240	-4.49182	-2.88257
H	-5.83847	-4.82607	-3.00834
H	-7.51975	-5.16177	-3.51250
C	-7.04220	-3.02638	-3.34039
H	-6.73078	-2.92895	-4.40433
C	-8.51545	-2.58650	-3.18727
H	-8.64001	-1.53626	-3.53448
H	-9.17301	-3.21585	-3.82771
C	-8.93632	-2.71105	-1.70541
H	-9.99419	-2.38562	-1.58929
C	-8.78837	-4.17733	-1.25005
H	-9.44633	-4.83981	-1.85616
H	-9.11097	-4.28532	-0.19061
C	6.97220	-2.21099	0.85671
C	7.05497	-2.00945	2.40880
H	7.25822	-0.94771	2.65654
H	6.06771	-2.25108	2.86207
C	8.38285	-1.91068	0.24516
H	8.34767	-2.08095	-0.85355
H	8.64622	-0.84228	0.38736
C	6.69344	-3.72367	0.62101
H	5.71239	-4.00810	1.04864
H	6.63740	-3.94163	-0.46203
C	8.15770	-2.89268	3.03631
H	8.18660	-2.69810	4.13122
C	9.52385	-2.53351	2.40863
H	9.76910	-1.46619	2.60733
H	10.33112	-3.13909	2.87673
C	9.47674	-2.79531	0.88616
H	10.45737	-2.53281	0.43096
C	9.16067	-4.28280	0.62826
H	9.14599	-4.48806	-0.46525
H	9.95377	-4.92950	1.06512
C	7.79063	-4.61615	1.25359
H	7.53281	-5.67805	1.04731
C	7.84407	-4.38006	2.77647
H	8.61964	-5.02761	3.24252
H	6.87249	-4.65501	3.24366
C	6.51351	2.63144	0.75199
C	7.98538	2.55423	0.22196
H	8.42366	1.55688	0.43115
H	7.98072	2.66693	-0.88443
C	6.54392	2.52075	2.31658
H	5.50636	2.61130	2.70756
H	6.90919	1.52188	2.63118
C	5.99405	4.06157	0.42512
H	5.95233	4.21980	-0.66921
H	4.96021	4.18862	0.79780
C	8.87631	3.64218	0.86436
H	9.91059	3.53254	0.46929
C	8.88479	3.46090	2.39937
H	9.29623	2.46150	2.66570
H	9.55160	4.21651	2.87053
C	7.44600	3.60791	2.94434
H	7.44836	3.47108	4.04839
C	6.89973	5.00584	2.59058
H	5.87222	5.13075	2.99841
H	7.52855	5.79776	3.05494
C	6.88823	5.15839	1.05617
H	6.46374	6.14863	0.78069
C	8.32663	5.03970	0.51282
H	8.97327	5.83261	0.95027
H	8.33654	5.19007	-0.58967

R

SCF (BP86) Energy = -4824.191580
Enthalpy 0K = -4821.872547
Enthalpy 298K = -4821.748778
Free Energy 298K = -4822.067176
Lowest Frequency = 1.0769 cm^{-1}
Second Frequency = 1.7318 cm^{-1}
SCF (BP86-D3^{BJ}) Energy = -4824.869000
SCF (C_6H_6) Energy = -4824.272531
SCF (BS2) Energy = -4829.041183

N	7.27017	-0.06631	1.76987
C	7.90370	0.97567	1.19524
C	6.84933	-0.23687	3.12795
C	5.47105	-0.07443	3.47119

C	5.05383	-0.40470	4.77729	C	0.10553	6.83560	-1.11455
H	3.99385	-0.27462	5.05067	H	0.91543	7.47669	-0.69561
C	5.95387	-0.89305	5.73289	H	0.00647	7.14122	-2.18166
H	5.60639	-1.14638	6.74690	C	-1.21010	7.14266	-0.38096
C	7.30178	-1.06275	5.38403	H	-1.11064	6.83872	0.68661
H	8.00687	-1.45296	6.13506	H	-2.01777	6.49999	-0.79942
C	7.77385	-0.74718	4.09521	C	-1.63018	8.61565	-0.45863
C	4.43820	0.44604	2.46670	H	-1.77598	8.94027	-1.51155
H	4.98351	0.67423	1.52550	H	-2.58249	8.80099	0.08083
C	3.37132	-0.62330	2.14569	H	-0.85991	9.28196	-0.01338
H	3.83178	-1.56863	1.79183	N	-8.12337	1.15443	-0.09786
H	2.67363	-0.26190	1.36084	C	-8.88006	0.23011	0.52741
H	2.76557	-0.86894	3.04389	C	-8.32466	2.56944	-0.20977
C	3.76532	1.75364	2.93734	C	-7.66217	3.45326	0.69876
H	3.17505	1.59650	3.86508	C	-7.75613	4.84314	0.48234
H	3.06825	2.13584	2.16193	H	-7.25315	5.53041	1.18193
H	4.50885	2.54988	3.14690	C	-8.46932	5.36936	-0.60322
C	9.24540	-0.98281	3.74095	H	-8.52986	6.45883	-0.75352
H	9.47105	-0.37234	2.84167	C	-9.09384	4.49490	-1.50369
C	10.22203	-0.54242	4.84933	H	-9.64211	4.90882	-2.36536
H	10.04927	0.51022	5.15548	C	-9.03351	3.09699	-1.33522
H	11.27111	-0.62666	4.49560	C	-6.83426	2.93248	1.87743
H	10.13562	-1.17453	5.75831	H	-6.98379	1.83269	1.92358
C	9.49090	-2.45873	3.36001	C	-5.32794	3.19201	1.65265
H	9.27629	-3.13257	4.21608	H	-4.97398	2.77406	0.68644
H	10.54400	-2.62235	3.04739	H	-4.72208	2.74366	2.46837
H	8.83799	-2.78674	2.52281	H	-5.10942	4.28082	1.63017
Mg	6.83377	-1.83009	0.76202	C	-7.28882	3.51567	3.23195
N	7.71648	-1.24311	-1.02593	H	-7.12794	4.61353	3.28133
C	8.31139	-0.04725	-1.19991	H	-6.71166	3.06152	4.06493
C	8.35747	0.92191	-0.15477	H	-8.36555	3.32744	3.42294
H	8.85837	1.84323	-0.46283	C	-9.69199	2.18356	-2.37341
C	7.49618	-2.29572	-1.97192	H	-9.68368	1.15358	-1.95851
C	6.31214	-2.28598	-2.77621	C	-11.16239	2.55301	-2.65805
C	6.03211	-3.40538	-3.58429	H	-11.77055	2.57195	-1.73003
H	5.12457	-3.40384	-4.20906	H	-11.62100	1.81551	-3.35004
C	6.88032	-4.52178	-3.61000	H	-11.25309	3.55060	-3.13764
H	6.64231	-5.38615	-4.24987	C	-8.87389	2.16322	-3.68382
C	8.02752	-4.53039	-2.80621	H	-8.86426	3.16527	-4.16299
H	8.68969	-5.41162	-2.81993	H	-9.30504	1.44326	-4.41137
C	8.35487	-3.43852	-1.97633	H	-7.81278	1.88450	-3.50945
C	5.33385	-1.10769	-2.74413	C	5.79079	-3.63588	1.23152
H	5.86674	-0.24750	-2.28748	H	6.13159	-4.41634	0.50875
C	4.12183	-1.42565	-1.84087	H	4.71693	-3.48142	0.95922
H	4.43260	-1.70389	-0.81118	C	5.85653	-4.19920	2.66472
H	3.43830	-0.55341	-1.76633	H	5.52607	-3.42758	3.39967
H	3.54131	-2.28361	-2.24153	H	6.91453	-4.41970	2.94377
C	4.86373	-0.66501	-4.14425	C	5.02488	-5.47737	2.89562
H	4.24043	-1.43952	-4.63918	H	3.96150	-5.26420	2.63787
H	4.24312	0.25282	-4.07200	H	5.35344	-6.25708	2.17000
H	5.71976	-0.44529	-4.81548	C	5.10923	-6.03064	4.32452
C	9.60829	-3.51578	-1.09895	H	6.15527	-6.29201	4.59628
H	9.70688	-2.53727	-0.58166	H	4.49426	-6.94653	4.45403
C	10.89577	-3.73288	-1.92252	H	4.75651	-5.28390	5.06871
H	11.03117	-2.94578	-2.69270	Mg	-6.39689	0.67585	-1.12628
H	11.78918	-3.71980	-1.26287	N	-6.48458	-1.35550	-0.76223
H	10.88584	-4.71267	-2.44565	C	-7.47408	-1.92678	-0.04672
C	9.46911	-4.60801	-0.01563	C	-8.53296	-1.15262	0.51258
H	9.39391	-5.61813	-0.47174	H	-9.25024	-1.76676	1.06277
H	10.34988	-4.61032	0.66078	C	-5.33904	-1.96230	-1.37516
H	8.55814	-4.46120	0.60122	C	-4.10652	-2.03728	-0.65236
C	0.52242	5.36148	-1.03960	C	-2.95616	-2.50286	-1.32031
H	0.66636	5.03529	0.01313	H	-2.00454	-2.56924	-0.76861
H	1.47502	5.17610	-1.57871	C	-2.99489	-2.88030	-2.66994

H	-2.08414	-3.24306	-3.17204	H	8.26003	1.74892	3.99719
C	-4.20078	-2.77979	-3.37689	C	7.43063	3.47923	1.08425
H	-4.22743	-3.06318	-4.44166	H	7.70900	3.44016	0.01102
C	-5.38161	-2.31962	-2.75934	H	6.33717	3.27949	1.13099
C	-4.00099	-1.59383	0.80985	C	9.72023	2.68502	1.84628
H	-5.03552	-1.44019	1.18314	H	10.26833	1.91998	2.43970
C	-3.26091	-0.24221	0.92078	H	10.10973	2.61392	0.81000
H	-3.72838	0.54308	0.28918	C	8.03951	3.91182	3.93270
H	-3.24914	0.12047	1.97049	H	7.69707	3.94105	4.99041
H	-2.20716	-0.33642	0.58247	C	7.28282	4.97585	3.11161
C	-3.33716	-2.65118	1.71601	H	6.18505	4.80823	3.18212
H	-2.27293	-2.81828	1.44630	H	7.47920	5.99260	3.51929
H	-3.35720	-2.32155	2.77620	C	7.74047	4.88711	1.64133
H	-3.85473	-3.63090	1.65525	H	7.19550	5.63997	1.02965
C	-6.66120	-2.19103	-3.59132	C	9.26114	5.15062	1.55579
H	-7.48124	-1.91915	-2.89303	H	9.59896	5.10686	0.49612
C	-7.06218	-3.51276	-4.27968	H	9.49397	6.17464	1.92333
H	-7.17458	-4.34156	-3.55052	C	10.01594	4.09824	2.39979
H	-8.02909	-3.39631	-4.81334	H	11.11118	4.28346	2.33521
H	-6.30833	-3.82945	-5.03134	C	9.55540	4.18980	3.86911
C	-6.53439	-1.05488	-4.63054	H	9.78361	5.19636	4.28536
H	-5.74807	-1.28561	-5.38041	H	10.10856	3.45151	4.49132
H	-7.48968	-0.91000	-5.17822	C	-10.19863	0.52420	1.33915
H	-6.25400	-0.08956	-4.15849	C	-10.00769	0.04638	2.81894
C	-3.83991	1.31262	-2.79569	H	-9.68812	-1.01558	2.84658
C	-2.92083	2.09293	-3.52472	H	-9.18576	0.63225	3.28688
C	-5.01013	1.86642	-2.20650	C	-11.38817	-0.27704	0.70971
H	-2.02617	1.62226	-3.96684	H	-11.55528	0.07768	-0.33143
H	-0.24991	4.69963	-1.48629	H	-11.13612	-1.35484	0.63530
C	-3.14489	3.47103	-3.69212	C	-10.64979	2.01137	1.40592
C	-5.19956	3.26343	-2.39544	H	-9.85335	2.63623	1.85408
H	-2.43190	4.08572	-4.26638	H	-10.81841	2.40895	0.38667
H	-6.08576	3.76402	-1.96486	C	-11.30942	0.20588	3.63722
C	-4.29018	4.05626	-3.12325	H	-11.12732	-0.16334	4.67089
H	-4.47797	5.13614	-3.24971	C	-12.43528	-0.62487	2.98007
H	-3.62692	0.23317	-2.69173	H	-12.15708	-1.70249	2.96310
C	9.03653	0.43143	-2.51519	H	-13.36939	-0.54551	3.57925
C	9.02102	-0.56422	-3.71015	C	-12.68124	-0.11519	1.54163
H	9.47729	-1.52842	-3.41356	H	-13.48802	-0.71543	1.06541
H	7.97980	-0.79357	-4.00802	C	-13.09013	1.37139	1.58439
C	10.54192	0.71306	-2.18388	H	-13.29113	1.74374	0.55523
H	10.62506	1.42412	-1.33633	H	-14.03293	1.49843	2.16185
H	11.02219	-0.23237	-1.84709	C	-11.94889	2.18208	2.23203
C	8.38718	1.75958	-3.03033	H	-12.21404	3.26210	2.24011
H	7.32566	1.56585	-3.30115	C	-11.72016	1.69198	3.67643
H	8.36956	2.52406	-2.22663	H	-12.64436	1.82391	4.28216
C	9.78035	-0.00185	-4.93754	H	-10.92540	2.29675	4.16713
H	9.73253	-0.75901	-5.75073	C	-7.60973	-3.46657	0.26060
C	11.25245	0.26939	-4.56561	C	-8.96189	-3.99071	-0.33212
H	11.75386	-0.67677	-4.26287	H	-9.81109	-3.38027	0.03814
H	11.80959	0.66063	-5.44599	H	-8.94614	-3.86675	-1.43762
C	11.29298	1.28675	-3.40702	C	-7.62893	-3.69006	1.81070
H	12.34820	1.48602	-3.11565	H	-6.65933	-3.35226	2.23905
C	10.61955	2.60480	-3.85255	H	-8.41386	-3.06600	2.28516
H	10.65944	3.35330	-3.02967	C	-6.48871	-4.38355	-0.30690
H	11.17146	3.04546	-4.71229	H	-6.42865	-4.28259	-1.40766
C	9.15158	2.32644	-4.24914	H	-5.50151	-4.07406	0.08681
H	8.66370	3.27518	-4.56504	C	-9.20350	-5.47280	0.03504
C	9.11463	1.30794	-5.40689	H	-10.18063	-5.79040	-0.39198
H	9.64230	1.71422	-6.29853	C	-9.23556	-5.62285	1.57317
H	8.06294	1.11602	-5.71547	H	-10.06192	-5.01210	2.00111
C	8.18834	2.36493	1.88471	H	-9.44298	-6.68013	1.85060
C	7.74322	2.49988	3.36902	C	-7.87953	-5.17413	2.16445
H	6.66026	2.29200	3.46622	H	-7.90286	-5.27649	3.27212

C	-6.74886	-6.04629	1.58158
H	-5.76975	-5.74687	2.01723
H	-6.90576	-7.11515	1.84866
C	-6.72732	-5.87196	0.04931
H	-5.89521	-6.46886	-0.38431
C	-8.07025	-6.34373	-0.54477
H	-8.24446	-7.41677	-0.30670
H	-8.05141	-6.25988	-1.65421

7 References

1. A. Causero, G. Ballmann, J. Pahl, H. Zijlstra, C. Farber and S. Harder, *Organometallics*, 2016, **35**, 3350-3360.
2. R. E. Cowley, K. P. Chiang and P. L. Holland, in *Inorganic Syntheses*, ed. T. Rauchfuss, Wiley, 2010, vol. 35, ch. 1. Complexes of Bulky Β-Diketiminate Ligands, pp. 13-19.
3. S. K. Nune, D. B. Lao, M. E. Bowden, H. T. Schaef, R. S. Vemuri, R. K. Motkuri and B. P. McGrail, *Inorg. Chim. Acta*, 2019, **489**, 150-154.
4. H. Hao, H. W. Roesky, Y. Ding, C. Cui, M. Schormann, H.-G. Schmidt, M. Noltemeyer and B. Žemva, *J. Fluorine Chem.*, 2002, **115**, 143-147.
5. S. P. Green, C. Jones and A. Stasch, *Angew. Chem. Int. Ed.*, 2008, **47**, 9079-9083.
6. A. P. Dove, V. C. Gibson, P. Hormnirun, E. L. Marshall, J. A. Segal, A. J. P. White and D. J. Williams, *Dalton Trans.*, 2003, 3088-3097.
7. L. Falivene, Z. Cao, A. Petta, L. Serra, A. Poater, R. Oliva, V. Scarano and L. Cavallo, *Nature Chem.*, 2019, **11**, 872-879.
8. S. Burnett, C. Bourne, A. M. Z. Slawin, T. van Mourik and A. Stasch, *Angew. Chem. Int. Ed.*, 2022, **61**, e202204472.
9. V. Balasanthiran, M. H. Chisholm, K. Choojun, C. B. Durr and P. M. Wambua, *Polyhedron*, 2016, **103**, 235-240.
10. B. Rösch, T. X. Gentner, J. Eyselein, A. Friedrich, J. Langer and S. Harder, *Chem. Commun.*, 2020, **56**, 11402-11405.
11. C. Jones, S. J. Bonyhady, S. Nembenna and A. Stasch, *Eur. J. Inorg. Chem.*, 2012, 2596-2601.
12. O. V. Dolomanov, L. J. Bourhis, R. J. Gildea, J. A. K. Howard and H. Puschmann, *J. Appl. Cryst.*, 2009, **42**, 339-341.
13. G. M. Sheldrick, *Acta Cryst. A*, 2015, **71**, 3-8.
14. G. M. Sheldrick, *Acta Cryst. C*, 2015, **71**, 3-8.
15. M. J. Frisch, G. W. Trucks, H. B. Schlegel, G. E. Scuseria, M. A. Robb, J. R. Cheeseman, G. Scalmani, V. Barone, G. A. Petersson, H. Nakatsuji, X. Li, M. Caricato, A. V. Marenich, J. Bloino, B. G. Janesko, R. Gomperts, B. Mennucci, H. P. Hratchian, J. V. Ortiz, A. F. Izmaylov, J. L. Sonnenberg, Williams, F. Ding, F. Lipparini, F. Egidi, J. Goings, B. Peng, A. Petrone, T. Henderson, D. Ranasinghe, V. G. Zakrzewski, J. Gao, N. Rega, G. Zheng, W. Liang, M. Hada, M. Ehara, K. Toyota, R. Fukuda, J. Hasegawa, M. Ishida, T. Nakajima, Y. Honda, O. Kitao, H. Nakai, T. Vreven, K. Throssell, J. A. Montgomery Jr., J. E. Peralta, F. Ogliaro, M. J. Bearpark, J. J. Heyd, E. N. Brothers, K. N. Kudin, V. N. Staroverov, T. A. Keith, R. Kobayashi, J. Normand, K. Raghavachari, A. P. Rendell, J. C. Burant, S. S. Iyengar, J. Tomasi, M. Cossi, J. M. Millam, M. Klene, C. Adamo, R. Cammi, J. W. Ochterski, R. L. Martin, K. Morokuma, O. Farkas, J. B. Foresman, D. J. Fox, Wallingford, CT, 201
16. A. D. Becke, *Phys. Rev. A* 1988, **38**, 3098-3100; b) J. P. Perdew, *Phys. Rev. B* 1986, **33**, 8822-882.
17. F. Weigend; R. Ahlrichs, *Phys. Chem. Chem. Phys*. 2005, **7**, 3297-330
18. S. Grimme, S. Ehrlich, L. Goerigk, *J. Comp. Chem*. 2011, **32**, 1456-146
19. J. Tomasi, B. Mennucci, R. Cammi, *Chem. Rev*. 2005, **105**, 2999-309
20. R. Paton. Ball & Stick style for Pymol. Zenodo. 2022. https://doi.org/10.5281/zenodo.7072564
